# Comprehensive Annotation and Functional Exploration of MicroRNAs in Lettuce

**DOI:** 10.3389/fpls.2021.781836

**Published:** 2021-12-24

**Authors:** Yang Deng, Yajuan Qin, Pan Yang, Jianjun Du, Zheng Kuang, Yongxin Zhao, Ying Wang, Dayong Li, Jianhua Wei, Xinyu Guo, Lei Li, Xiaozeng Yang

**Affiliations:** ^1^Beijing Academy of Agriculture and Forestry Sciences, Beijing, China; ^2^Beijing Key Laboratory of Agricultural Genetic Resources and Biotechnology, Beijing Agro-Biotechnology Research Center, Beijing, China; ^3^College of Life Sciences, Fujian Agriculture and Forestry University, Fuzhou, China; ^4^State Key Laboratory of Protein and Plant Gene Research, Peking-Tsinghua Center for Life Sciences, School of Advanced Agricultural Sciences and School of Life Sciences, Peking University, Beijing, China; ^5^Beijing Key Lab of Digital Plant, Beijing Research Center for Information Technology in Agriculture, Beijing, China; ^6^Beijing Key Laboratory of Vegetable Germplasm Improvement, Beijing Vegetable Research Center, Beijing, China

**Keywords:** lettuce, microRNA (miRNA), sRNA-Seq, expression correlation, regulatory network, miR408

## Abstract

MicroRNA (miRNA) is an important endogenous post-transcriptional regulator, while lettuce (*Lactuca sativa*) is a leafy vegetable of global economic significance. However, there are few studies on miRNAs in lettuce, and research on miRNA regulatory network in lettuce is absent. In this study, through deep sequencing of small RNAs in different tissues, together with a reference genome, 157 high-confidence miRNA loci in lettuce were comprehensively identified, and their expression patterns were determined. Using a combination of computational prediction and high-throughput experimental verification, a set of reliable lettuce miRNA targets were obtained. Furthermore, through RNA-Seq, the expression profiles of these targets and a comprehensive view of the negative regulatory relationship between miRNAs and their targets was acquired based on a correlation analysis. To further understand miRNA functions, a miRNA regulatory network was constructed, with miRNAs at the core and combining transcription factors and miRNA target genes. This regulatory network, mainly composed of feed forward loop motifs, greatly increases understanding of the potential functions of miRNAs, and many unknown potential regulatory links were discovered. Finally, considering its specific expression pattern, *Lsa-MIR408* as a hub gene was employed to illustrate the function of the regulatory network, and genetic experiments revealed its ability to increase the fresh weight and achene size of lettuce. In short, this work lays a solid foundation for the study of miRNA functions and regulatory networks in lettuce.

## Introduction

Lettuce (*Lactuca sativa*), of the Asteraceae family, is a leafy vegetable with important economic value. According to the Food and Agriculture Organization^1^, the global production of lettuce, together with chicory, exceeded 29 million tons in 2019, ranking third of all leafy vegetables. In the United States alone, lettuce production in 2019 exceeded 3.6 million tons, with a market value of over 1.5 billion USD. The worldwide popularity of lettuce is due to its diverse cultivars, taste, and high nutritional value, being rich in vitamins, folates, and minerals (Tamura et al., 2018). With the availability of a reference genome (Reyes-Chin-Wo et al., 2017) and numerous transcriptomes (Zhang L. et al., 2017), lettuce has become a model for studying Asteraceae and related plants. Lettuce has numerous cultivars, including butterhead, crisphead, looseleaf, romaine, stem, and many mixed types, which are highly varied in morphology, and provide good material for studying the morphological development of plants (Seki et al., 2020; Yu et al., 2020). The same is true for lettuce in terms of leaf colors (Su et al., 2020), metabolites (Zhang et al., 2020), heat responses (Chen et al., 2017, 2018), viral resistance (Inderbitzin et al., 2019), etc. Lettuce is also an important example of crop domestication and for adaptation research (Zhang L. et al., 2017; Wei et al., 2021). Recently, lettuce has been used as a novel platform for biopharmaceutical production due to its numerous advantages, including low processing cost, consistent and scalable production, and the excellent biosafety profile of transgenic plants (Daniell et al., 2019; Zhang et al., 2019).

MicroRNA (miRNA) is an important post-transcriptional regulatory factor, which has received considerable research interest (Fromm et al., 2020). In plants, major mature miRNAs only have 20–24 nucleotides (nts), which can find target mRNAs through nearly perfect sequence complementation, leading to degradation or translation block (Voinnet, 2009). MiRNA genes share many common features with protein-coding genes such as defined promoters and transcription start sites as well as the exon-intron gene structure (Voinnet, 2009). In general, RNA polymerase II (Pol II) is responsible for transcriptional activation of miRNA genes in plants (Lee et al., 2004). The initially transcribed miRNA, called pri-miRNA, has a stem-loop structure (Bartel, 2009). In plants, pri-miRNA is first cut into pre-miRNA by Dicer-like, and then further processed into a duplex composed of mature miRNA and miRNA*. Only the mature miRNA guides the miRNA-induced silencing complex to regulate its mRNA targets, and other parts are degraded (Bartel, 2009; Voinnet, 2009). Studies have found that plant miRNAs have very diverse functions in development, responses to environmental challenges, etc. (Chandra et al., 2017). For example, miR156 can regulate the transition from juvenile to adult developmental stages (Wang et al., 2009; Wu et al., 2009), miR319/159 is related to leaf morphology and reproduction (Yang et al., 2013; Cheng et al., 2020), and miR399 is associated with the phosphate-starvation response (Fujii et al., 2005). It has recently been discovered that miRNAs in plants can also be used as carriers for information exchange between different species (Shahid et al., 2018; Tsikou et al., 2018).

In the last decade, high-throughput sequencing has become the most powerful method to identify miRNAs. The combination of small RNA sequencing (sRNA-Seq) and subsequent bioinformatic analysis has uncovered a large number of new miRNAs (Guo et al., 2020). The method of miRNA target gene exploration is constantly improving, which further improved the prediction accuracy of miRNA targets as well (Zhao et al., 2021). The high-throughput target gene verification method, parallel analysis of RNA ends sequencing [PARE-Seq (German et al., 2009)] or degradome sequencing enables identification of miRNA target genes on a genomic scale. In addition, combined with RNA-Seq data, the correlation of expression patterns between miRNAs and potential targets provides not only a reference for target gene prediction, but also the potential for the discovery of new regulatory miRNA-target pairs. Motif scanning, together with methods such as Chromatin Immunoprecipitation Sequencing (Johnson et al., 2007) or DNA Affinity Purification Sequencing (Bartlett et al., 2017), has been widely employed as a high-throughput means to detect the regulation between transcriptional factors (TFs) and their targets, which could be used to discover the upstream regulation from TFs to miRNAs. With all these technical advantages, a whole genome scaled picture of miRNA-based regulatory networks in a specific species can be achieved. For example, a multiple layer regulation network was recently constructed in Arabidopsis (Gao et al., 2021). However, despite being an important economic crop, case studies of miRNAs in lettuce are scarce (Huo et al., 2016), and the systematic identification and annotation of miRNAs and their regulatory networks are absent.

In this study, by sequencing sRNAs in four different tissues and using a pipeline centered by miRDeep-P2 (Kuang et al., 2019), 157 high-confidence miRNAs were identified in lettuce. The detailed characteristics of these miRNAs were annotated, including sequences, structure, conservation, clusters, selection after duplication, and expression patterns in different tissues. The targets of these miRNAs were predicted by three different methods, and Gene Ontology [GO (Mi et al., 2019)] and Kyoto Encyclopedia of Genes and Genomes [KEGG (Ogata et al., 1999)] enrichment analyses indicated that these target genes are widely involved in signal transduction, protein binding, and various enzyme activities, partially reflecting the diversification of miRNA functions and the role of a specific group of miRNA targets, TFs. Furthermore, a correlation analysis on miRNA and target expression patterns was performed, and the negative regulatory relationship between miRNAs and target genes is highlighted, although this correlation is affected by numerous factors. In addition, a regulatory network involving miRNAs, TFs, and targets, was constructed, and the connection between different feed forward loops (FFLs) helped in defining a number of new regulatory links. Finally, based on its specific expression pattern and potential function, miR408 was used as an example to verify this exploration of miRNAs and miRNA regulatory network. Genetic experiments not only confirmed the solidity of the data, but also found that *Lsa-MIR408* can promote the vegetative growth of lettuce and increase achene size. This work systematically identified and annotated miRNAs in lettuce, and conducted preliminary explorations of the functions of these miRNAs by means of target genes, expression correlation, regulatory networks, etc., and lays a solid foundation for further research on lettuce miRNAs.

## Materials and Methods

### Plant Materials

Lettuce (*Lactuca sativa L.*) achenes were purchased from the vegetable institute of Beijing Academy of Agriculture and Forestry Sciences, Beijing. Achenes were first sown on a wet filter paper in a petri dish and moved directly to the soil in plastic pots after germination. The seedlings were cultured in a greenhouse (16 h/8 h day/night cycle, light intensity 200 μmol m^–2^ s^–1^, relative humidity 30–50%). The root, stem, and leaf were collected from 4-week-old plants. The flowers were collected at full bloomed capitula (19-week-old plants). After collection, the samples of each tissue were quickly frozen in liquid nitrogen and stored at −80^°^C for the extraction of total RNA, respectively.

### Small RNA and mRNA Libraries Construction and Sequencing

The RNA from roots, stems, leaves, and flowers of lettuce was, respectively, isolated using the OminiPlant RNA Kit (Cwbio, China), following the manufacturer’s protocol. The integrity and quality of RNAs were validated by an Agilent 2100 Bioanalyzer. Small RNA cDNA libraries were prepared using the Small RNA Sample Prep Kit (Illumina, United States), and mRNA cDNA libraries were prepared using the TruSeq Stranded RNA LT Kit (Illumina, United States), based on the manufacturer’s protocols. Briefly, sRNAs were isolated from 20 μg of total RNA by 15% polyacrylamide gel electrophoresis and ligated two adaptors, including a 5′-RNA and a 3′-RNA adaptor. Then, the samples were converted and amplified to cDNA by RT-PCR (mRNA cDNA library preparation is similar). Lastly, the validated cDNA libraries were sequenced by Illumina Hiseq2500. Small RNA and mRNA cDNA libraries included two biological replicates, and 16 libraries were produced.

### Identification of Conserved, Asteraceae-Specific, and Lettuce-Specific MicroRNAs

After sRNA library sequencing, clean reads were obtained by filtering low-quality sequences, including junk reads, adaptor sequences, polyA tags and reads <18 bp and >30 bp. The extracted clean reads (19–25 nt in length) were used for miRNA prediction. Reads matching plant non-coding RNAs, including tRNA, rRNA snRNA, and snoRNA sequences in the Rfam database (version 13.0; Kalvari et al., 2018), with no more than one mismatch were further filtered. Next, the remaining sequences were mapped to the lettuce genome (Reyes-Chin-Wo et al., 2017), and candidate miRNAs were detected via miRDeep-P2 software (version 1.1.4; Kuang et al., 2019). The adjacent sequences of mapped sRNAs were extracted as candidate miRNA precursor sequences (details in the miRDeep-P2 manual). The secondary structures of all candidates were predicted by RNAfold (version 2.1.2; Tav et al., 2016). The newly updated plant miRNA criteria (Axtell and Meyers, 2018) were employed to identify miRNAs.

To annotate the conservation of miRNAs in lettuce, all the predicted mature miRNA sequences with ± 1 nt adjacent nucleotide were aligned with all plant miRNAs in PmiREN1.0 (Guo et al., 2020), with no more than two mismatches, using Bowtie (version 1.2.2; Langmead, 2010) software. Those with no matched miRNAs in PmiREN1.0 were assigned as lettuce-specific miRNAs, while those which only hit miRNAs in other Asteraceae species were considered Asteraceae-specific miRNAs, and others were annotated as conserved.

### Synteny Analysis of Identified MicroRNAs

The synteny analysis of miRNAs was carried out using JCVI (MCscanX python version 1.1.18; Wang et al., 2012). Firstly, the transformed Browser Extensible Data (BED) file and protein sequences were set as input files, and the collinearity blocks across the whole lettuce genome were obtained. Subsequently, the genomic locations of all miRNAs were reflected to these collinearity blocks via BEDTools (version 2.26.0; Quinlan and Hall, 2010; subfunction: intersect), and members from the same miRNA family that followed similar gene orders in these collinearity blocks were considered as syntenic miRNAs. The result plot was generated with Circos (version 0.69.9; Krzywinski et al., 2009).

### Targets Prediction of Identified MicroRNAs

Two plant miRNA target prediction toolkits, psRNATarget (Dai et al., 2018) and RNAhybrid (Kruger and Rehmsmeier, 2006), were used to predict miRNA targets. The identified lettuce miRNA sequences and mRNA transcript sequences were uploaded to psRNATarget webserver^2^ and the latest default parameters (2017 release) were used. Targets with an *E*-value ≤ 3.0 were kept as possible miRNA targets. Meanwhile, RNAhybrid (version 2.1.2) was used to predict energetically plausible miRNA-mRNA duplexes with plant-specific constraints where “−*d*” parameter was set as “8,12.” A strict cut-off value for minimum free energy/minimum duplex energy of 0.75 was used (Alves et al., 2009).

Degradome-seq (PARE-seq) data downloaded from GEO datasets (SRP078275) were also used to predict miRNA targets. CleaveLand4.0 (Addo-Quaye et al., 2009) software was used to identify putative miRNA cleavage sites with default settings. The reads were aligned to lettuce transcript sequences to generated density files. The predicted miRNA mature sequences were aligned to transcript sequences to identify potential miRNA target sites. The density distribution of reads and miRNA-mRNA alignment were used together to classify miRNA target candidates. All potential miRNA targets were assigned into one of five categories, and to reduce false positives, only results from categories 0, 1, and 2 were retained.

### MicroRNA and mRNA Expression Analysis

For miRNA expression, expression of mature and star miRNAs was normalized by RPM. For each sRNA-seq dataset, reads mapped to pri-miRNAs (no mismatches allowed) and localized in genomic positions of mature miRNAs (no more than 2-nt shift allowed) were considered to correspond to mature miRNAs. The total numbers of these reads were counted to calculate the RPM for the mature miRNAs.

For mRNA expression, all raw mRNA reads were checked by Fastqc (version 0.11.9; Brown et al., 2017) and low-quality reads and adapters were removed by Cutadapt (version 3.2; Kechin et al., 2017). Clean reads were aligned to the lettuce genome using HISTAT2 (version 2.1.0; Kim et al., 2019). StringTie (version 2.1.5; Pertea et al., 2015) was used to assemble the transcript and quantify each mRNA-seq dataset. The expression values of transcripts were normalized by fragments per kilobase per million fragments (FPKM) and the results of StringTie were extracted.

### Correlation Analysis Between the Expression of MicroRNAs and Predicted Targets

The Pearson correlation coefficient between the expression matrix of miRNAs and targets was calculated using the R function “cor.test().” Firstly, according to the biological replicates of four tissues, the raw expression matrix of miRNAs and targets were averaged and transposed by the R function “*t*()” to obtain a matrix with miRNAs and targets as factors. Then, the “for loop” of R language, was used to calculate the correlation coefficient between each miRNA and target, and a Student’s *t*-test was used to test significance. The results were displayed by the R package (Pheatmap), where blue bars showed positive correlation, red bars showed negative correlation, and a *P*-value of <0.05 is presented as “*” (Figure 3A).

**FIGURE 1:**
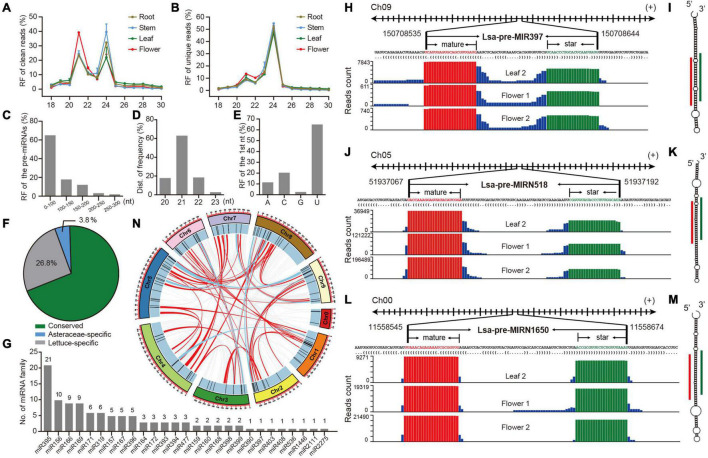
Identification and annotation of miRNAs in lettuce. **(A,B)** Relative frequency (RF) in length of clean and unique reads, respectively, from sRNA libraries in different tissues. **(C)** RF of pre-miRNAs in length. **(D)** RF of mature miRNAs in length. **(E)** RF of the first nucleotide (nt) of mature miRNAs. **(F)** The proportion of conserved, Asteraceae-specific and lettuce-specific miRNAs. **(G)** Member numbers of conserved miRNA families. **(H,J,L)** Examples of conserved, Asteraceae-specific, and lettuce-specific miRNA candidates, miR397, miRN518, and miRN1650, respectively. For each miRNA, presented information includes pri-miRNA excerpt, pre-miRNA, secondary structure in dot-bracket notation, and read abundance along the precursor. Letters with red and green colors indicate mature and star miRNAs, respectively, while the histogram shows the copy number of small reads from sRNA libraries. **(I,K,M)** Respective secondary structures of pre-miR397, pre-miRN518, and pre-miRN1650, predicted by RNAfold. Red lines indicate positions of mature miRNAs, while green lines show positions of miRNA stars. **(N)** Synteny analysis of lettuce miRNAs across all pseudo-chromosomes. Red lines indicate miRNAs are located in synteny blocks while light blue lines indicate there are paired miRNAs in synteny blocks.

**FIGURE 2:**
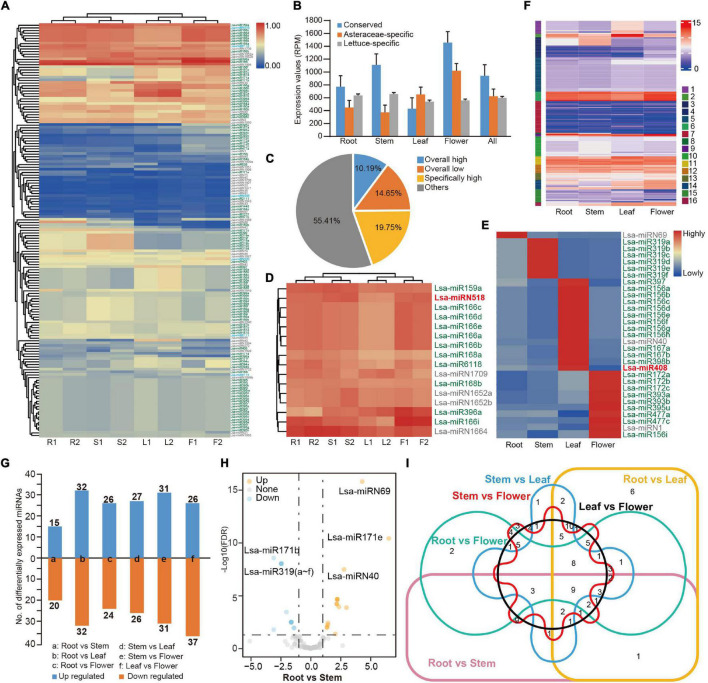
Expression profile of miRNAs in lettuce. **(A)** Heat map of expression profile of 157 miRNAs in four tissues. miRNA IDs with green, blue, and gray colors represent conserved, Asteraceae-specific, and lettuce-specific miRNAs, respectively. R1 and R2 indicate two samples from root tissue while S, L, and F represent samples from stem, leaf and flower, respectively. **(B)** Average expression values of three types of miRNAs. Values were normalized by reads per million (RPM). **(C)** The percentage of miRNAs based on expression pattern. Overall high, low, and specifically high, indicate miRNAs with high or low expression in all tissues, or with high expression in specific tissues, respectively. **(D)** Highly expressed miRNAs (house-keeping miRNAs) in all tissues. **(E)** Tissue-specific highly expressed miRNAs. **(F)** Expression pattern categories by k-means cluster analysis (*k* = 16). **(G)** Differentially expressed miRNAs in a paired-tissue comparison. **(H)** Volcano plot displaying differentially expressed miRNAs between root and stem. **(I)** Venn diagram showing the differentially expressed miRNAs in six paired-tissue comparisons.

**FIGURE 3:**
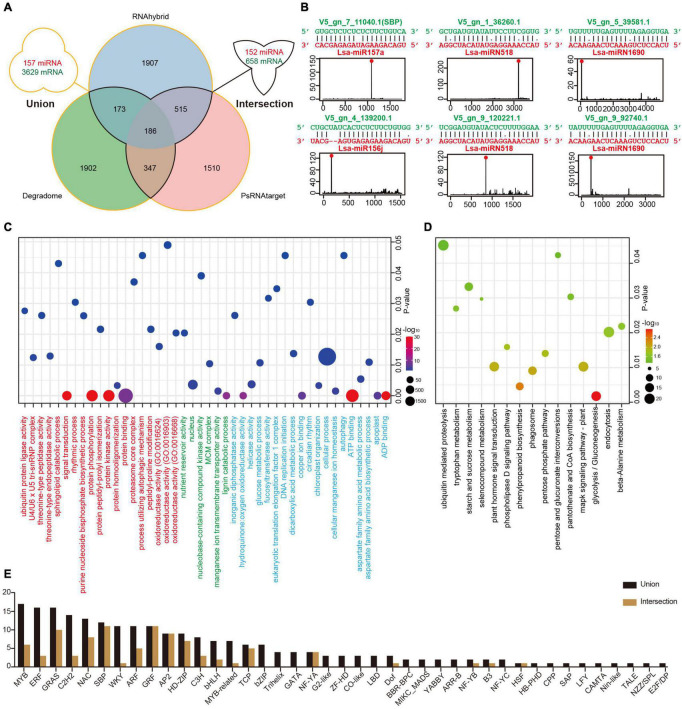
Target identification of miRNAs in lettuce. **(A)** Potential miRNA targets identified via three methods. The “intersection” indicates targets detected by at least two methods, while the “union” shows all targets predicted by any of the three methods. **(B)** Examples of high confidence miRNA-target pairs. Six pairs represent conserved (Lsa-miR156j, Lsa-miR157a, and their targets), Asteraceae-specific (two circuses of Lsa-miRN518), and lettuce-specific (two circuses of Lsa-miRN1690) examples, respectively. **(C)** Gene Ontology (GO) enrichment analysis of miRNA targets. Terms under red, green, and blue present the enrichment analyses in three ontologies, biological process (BP), cellular component (CC), and molecular function (MF), respectively. **(D)** Kyoto Encyclopedia of Genes and Genomes (KEGG) enrichment analysis of miRNA targets. **(E)** List of transcriptional factor families regulated by miRNAs. The “intersection” and “union” are the same as described in **(A)**.

### Network Construction

For the miRNA network, TFs of lettuce were annotated by searching all mRNA transcripts of lettuce against TF datasets from PlantTFDB^3^ (Jin et al., 2017), and the default filtered result was kept. Upstream 2000 nts were extracted as putative promoter sequences for each pre-miRNAs and miRNA target transcripts, and then submitted into PlantRegMap webserver^4^ (Tian et al., 2020) to scan TF binding sites. FFL motifs were selected by an in-house perl script based on these interactions between TFs-miRNAs, miRNAs-targets and TF-miRNA targets. Then, a genome-wide miRNA network was constructed in terms of all FFL motifs and Cytoscape (version 3.7.1; Shannon et al., 2003) was employed to display the network structure (Figure 5B).

**FIGURE 4:**
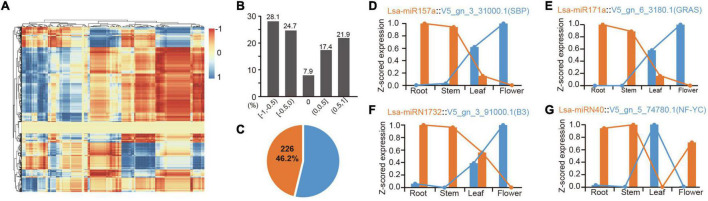
Expression correlation of miRNAs and their targets. **(A)** The heatmap of expression correlation of all miRNAs and their targets. Each dot indicates a value of expression correlation of a miRNA, and one of its specific target. Color range shows the variation from totally positive (1) to full negative (−1) correlation. **(B)** Distribution of miRNA-target expression correlation in divided sections. The five respective sections from −1 to 1, are −1 ≤ *R* < −0.5 ([−1, −0.5]), −0.5 ≤ *R* < 0 ([−0.5, 0]), *R* = 0 [0], 0 < *R* ≤ 0.5 ([0, 0.5]), and 0.5 < *R* ≤ 1 ([0.5, 1]), where R indicates the correlation values between miRNA and target expression. **(C)** Percentage of high confidence negative correlation: over 46% of these correlations are high confidence (*R* < −0.9, *P* < 0.05). **(D,E)** Two representative examples of negative correlation of conserved miRNAs. **(F,G)** Two representative examples of negative correlation of lettuce-specific miRNAs.

**FIGURE 5:**
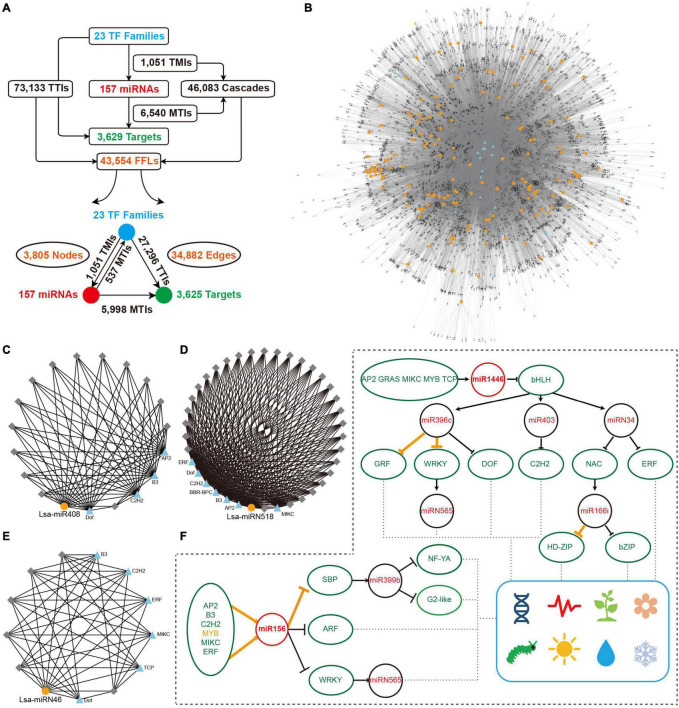
Construction of miRNA regulatory network. **(A)** Overall interactions and feed forward loops (FFLs) in an miRNA regulatory network. **(B)** Entire regulatory network based on all FFLs. Blue triangles and gray diamonds represent transcriptional factors (TFs) and targets, respectively, while yellow dots indicate miRNAs. **(C–E)** Examples of a local FFL network hubbed by three miRNAs: Lsa-miR408, Lsa-miRN518; and Lsa-miRN46. Blue triangles and yellow dots represent TFs and miRNAs, respectively, while gray diamonds indicate targets that could be regulated by these TFs and miRNAs. **(F)** A particle of miRNA and TF regulatory network. Orange lines show the regulations have been validated in other species, while black lines indicate new regulatory relationships expanded by this regulatory network. These expanded networks might be related to development, circadian clock, flowering, responses to insect and environmental challenges, etc. TMI: TF and miRNA interaction; MTI: miRNA and target interaction; and TTI: TF and target interaction.

### Construction of Plant Expression Vector and Lettuce Transformation

To construct the miR408 overexpression vector, genomic DNA of lettuce containing pre-miR408 was PCR amplified using the primers MIR408-F/R (Supplementary Table 22). This fragment was then cloned into pBI121 vector after *Xba*I and *Sac*I double digestion, to create the MIR408-overexpressing vector 35S:MIR408. The vector containing the overexpression cassette for MIR408 was then introduced into the *Agrobacterium tumefaciens* strain EHA105 by the freeze-thaw method. Cotyledon explants of lettuce plants were transformed with Agrobacterium EHA105 containing the 35S:MIR408 construct, following the reported protocol.

### Quantitative RT-PCR

Total RNA from lettuce was reverse transcribed using the Maxima First Strand cDNA Synthesis Kit (Thermofisher, United States). The resultant cDNA was analyzed by SYBR premix Ex Taq (ABMgood, United States) with the CFX96 Touch System (Bio-Rad, United States). The PCR protocol was 95^°^C for 30 s, 40 cycles of 95^°^C for 1 min, and 60^°^C for 10 s. 18S ribosomal RNA was used as the internal control gene. Total small RNA was extracted by the miRcute miRNA Isolation Kit (TianGen, China), following the manufacturer’s instructions. Reverse miRNA transcription was conducted using the miRcute Plus miRNA First-Strand cDNA SynthesisKit (TianGen, China). The resultant cDNA was analyzed using the miRcute miRNA qPCR Detection Kit, SYBR Green (TianGen, China), with the CFX96 Touch System (Bio-Rad, United States). PCR was performed under the following conditions: 95^°^C for 15 min, 34 cycles of 94^°^C for 30 s, and 60^°^C for 34 s. U6 snRNA was used as the reference gene. PCR reactions were carried out with three biological replicates and each biological replicate was performed with four technical repeats. The results were calculated using the 2^–ΔΔCt^ method. All primers used for qRT-PCR analysis are listed in Supplementary Table 22.

## Results

### Comprehensive Identification and Annotation of MicroRNAs in Lettuce

To comprehensively identify miRNAs in lettuce, sRNA libraries from different tissues, including leaf, stem, root and flower, were prepared in duplicate (details in section “Materials and Methods”) and observed with well consistency (Supplementary Figure 1A). These eight libraries produced 240 million raw reads and 235 million clean reads (Supplementary Table 1). Length distribution of these sRNA libraries showed two peaks at 21 and 24 nt in both clean and unique sRNAs, potentially reflecting the abundance of miRNAs and small interference RNAs (siRNAs), respectively (Figures 1A,B and Supplementary Tables 2, 3). The large decrease at 21 nt from clean to unique read distributions further supports this speculation (Figures 1A,B) because, in general, miRNAs prefer to accumulate many identical reads, while siRNAs possess much greater sequence diversity (Carthew and Sontheimer, 2009; Axtell and Meyers, 2018). Compared to other tissues, abundance at the 21-nt peak is higher than that at 24-nt in the flower (Figure 1A), suggesting higher miRNA activities in flower tissue.

MiRDeep-P2 (Kuang et al., 2019) was employed to identify miRNAs when only 19–25 nt reads were selected as inputs. Filtered with strict plant miRNA criteria (Axtell and Meyers, 2018), 157 miRNA loci, belonging to 67 families, were annotated (Supplementary Table 4). Several features of the miRNA loci were scanned, and most of their precursors, accounting for 80%, were less than 150 nts (Figure 1C), and the length of mature miRNAs were predominantly 21 nts (Figure 1D), while the first nucleic acid of mature miRNAs is frequently U (Figure 1E). All of these observations meet plant miRNA characteristics, as indicated by previous research (Axtell and Meyers, 2018), suggesting that the identification and annotation is reliable. A conservation search against all miRNA entries in PmiREN1.0 (Guo et al., 2020), found that 109, including 27 families, are conserved since their counterparts could be found in other non-Asteraceae species, while only six are considered Asteraceae-specific, because counterparts were only detected in Asteraceae species. Forty-two items were annotated as lettuce specific, considering no conserved ones were detected (Supplementary Table 4 and Figure 1F). Among the conserved families, miR395 has the most abundant members, while the other 18 families have multiple members (Figure 1G). The annotation for each miRNA includes all relevant information, including genomic location, sequences (Supplementary Table 4), secondary structure, and reads signature along precursors. Figures 1H–M, show conserved, Asteraceae-specific and lettuce-specific examples, respectively. Standard stem-loop structure and reads signature, such as the accumulation at mature and star miRNAs, strongly support them being highly confident candidates.

The miRNA clusters were further scanned, and nine clusters were obtained when a cluster region length not exceeding 10 kb was defined (Supplementary Table 5). There were four miR395 clusters, containing 13 miR395 members (61.2% of 21 miR395s), indicating why miR395 is the most abundant miRNA family, and its family expansion may be due to segmental duplication after a tandem duplication, considering all four clusters are located at the same chromosome (Supplementary Table 5 and Figure 1G). Since there exists a whole genome triplication (WGT) event during the evolution of the lettuce genome (Reyes-Chin-Wo et al., 2017), the WGT impact on miRNA selection was further investigated. Interestingly, even though there are clearly numerous synteny blocks (Supplementary Tables 6, 7), only six were kept in two-paired synteny blocks after the WGT event (Supplementary Table 7); most duplicated miRNA members were lost during the genome evolution (Figure 1N).

### Expression Patterns of Lettuce MicroRNAs in Different Tissues

The replicated tissue samples enabled a systematic examination of the expression patterns of lettuce miRNAs. Normalized by reads per million (RPM), the complete expression profile of 157 miRNAs was obtained (Figure 2A, Supplementary Table 8, and Supplementary Figure 1B). In general, conserved miRNAs had higher average expression (Figure 2B). MiRNAs varied in their expression patterns, and some were consistently highly expressed in different tissues, while some varied significantly between different tissues (Figure 2A). When the low (<10 RPM) and high expression (>500 RPM) was defined, approximately 16 miRNAs were consistently highly expressed in different tissues, such as house-keeping genes, which account for 10% of all miRNAs (Figures 2C,D), while 31 miRNAs were highly expressed in a specific tissue (Figures 2C,E). Over half of the miRNAs were expressed at an ordinary level, and approximately 14% were had constantly low expression (Figure 2C). To further explore the expression pattern of miRNAs in different tissues, a *k*-means cluster analysis was performed. When *k* = 16, the expression patterns of all miRNAs could be separated well (Figure 2F, Supplementary Figure 2, and Supplementary Table 8).

Differently expressed miRNAs were compared among the sampled tissues. In general, 35 to 64 miRNAs were up or down-regulated between a pair of tissues (Figures 2G,H, Supplementary Figure 3A, and Supplementary Table 9), but less than 10 of them were differently expressed in multiple tissues (Figure 2I). Analysis of a specific miRNA or miRNA family identified that, as in other species, miR166 is the house-keeping miRNA, highly expressed in all examined tissues (Figure 2D). As expected, considering their roles in the transition between juvenile and adult, miR156 was highly expressed in juvenile leaf tissue, while miR172 was highly expressed in flower tissue (Wang et al., 2009; Wu et al., 2009; Figure 2E). However, Lsa-miR156i, an miR156 member, was highly expressed in flower tissue (Figure 2E). Meanwhile, a number of lettuce-specific miRNAs were consistently highly expressed in all tissues, including Lsa-miRN1709, Lsa-miRN1652a/b, and Lsa-miRN1664 (Figure 2D), and some of them, including Lsa-miRN1, Lsa-miRN40, Lsa-miRN69 (Figure 2E), were highly expressed in a specific tissue. This was less common, as species-specific miRNAs, in general, had low expression (Yang et al., 2021).

### Integrative Target Identification of Lettuce MicroRNAs

To elucidate the function of miRNAs in lettuce, a combination of computational methods and high-throughput experiments were employed to explore potential miRNA targets. Two computational tools, psRNATarget and RNAhybrid with strict selection criteria (details in Materials and Methods), were first used to predict miRNA targets, with 157 miRNAs and all annotated transcripts of lettuce as inputs. The CleaveLand4.0 (Addo-Quaye et al., 2009) with PARE-Seq data was employed to further detect miRNA targets, where only categories 0–2 were kept for high confidence. As shown in Figure 3A, the “union” includes 157 miRNAs and 3,629 transcripts, comprised of 6,540 miRNA-target regulatory pairs when the result from these three methods was united, while the “intersection” possesses 152 miRNAs and 658 transcripts if only the intersected result was kept by at least two methods (Supplementary Table 10 and Supplementary Data 1).

Among the regulatory pairs between these miRNAs and target genes, almost all conserved regulatory relationships that have been studied or reported in model plants, such as Arabidopsis, rice, and maize, were detected, indicating that the predictions are reliable (Supplementary Table 10). A number of new regulatory relationships were also discovered. For example, the target genes of conserved miR172 and miR396 can be TFs, ERF and WRKY, respectively, and these predictions are well supported by the PARE-Seq data (Supplementary Data 1). In addition, the regulatory relationships between many miRNAs specific to Asteraceae or lettuce and their target genes were discovered. For example, TFs, Dof, and ERF, could potentially be regulated by Lsa-miRN1655 and Lsa-miRN69, which is strongly supported by the evidence (Supplementary Table 10 and Supplementary Data 1). Figure 3B shows six examples: miR157a and miR156j are representatives of conserved miRNAs, miRN518 is Asteraceae-specific, while miRN28 is lettuce-specific. All of the regulatory pairs are well supported by PARE-Seq results.

To understand the overall situation of miRNA target genes at the genome-wide level, GO and KEGG enrichment analyses were performed on all miRNA target genes. In GO, biological process (BP) analysis showed the largest enrichment groups were protein binding, protein kinase activity, protein phosphorylation, and signal transduction (Figure 3C and Supplementary Table 11). In the cellular component analysis, the largest enriched group was nucleus, indicating that a large part of the gene regulated by miRNAs functions in the nucleus. In molecular function analysis, the most abundant genes were related to cellular process, ATP and ADP binding, and various enzyme activities. KEGG enrichment analysis identified signaling pathway or transduction as the main enriched pathways (Figure 3D and Supplementary Table 12). These are all closely related to miRNAs as regulatory factors and its a large class of target genes, TFs, such as the transmission and transduction of various signals, and functions in the nucleus. The TFs that miRNAs can regulate were investigated further, and 41/23 families with different constrictions were identified (Figure 3E).

### Expression Correlation Between MicroRNA-Target Regulatory Pairs

Identifying expression patterns of miRNAs and targets in different tissues provides strong evidence for determining whether there is a real regulatory relationship between them. High-throughput sequencing enables the identification of expression patterns between miRNAs and target genes in batches. To further confirm the regulation between miRNAs and target genes, the mRNAs were sequenced from four tissues, as before. Principal component analysis showed consistency between mRNA and miRNA samples, indicating the reliability of these data (Supplementary Figures 1, 4). The expression patterns of all potential miRNA targets were obtained from these four tissues. To understand these miRNA-target regulatory pairs, a matrix analysis of the expression patterns of all miRNAs and their targets was pioneered (Figure 4A and Supplementary Table 13). Considering one miRNA can regulate multiple targets, the same target can inversely accept regulations from different miRNAs, and differential spatiotemporal expression exists between miRNAs and targets; thereby a complex matrix was formed (Figure 4A). Interestingly, although these miRNAs and their targets potentially only have a negative regulatory relationship in a specific tissue, the entire matrix showed good support for the negative regulation of miRNAs and target genes (Figures 4A,B). Data analysis based on “intersection” datasets showed that >46% of the miRNA-target pairs support a strict negative regulatory relationship (*R* < = 0.9, *P* < 0.05, Figure 4C).

There are numerous classic cases, such as the expression pattern of miR156 and miR172 in these tissues, which are well supported for their role in developmental transition (Wang et al., 2009; Wu et al., 2009; Supplementary Table 13). MiR157a is a good example, where Lsa-miR157a and its target V5_gn_3_31000.1, a gene harboring SBP (Squamosa-promoter binding protein) domain, have a reverse expression pattern in the four tissues (Figure 4D). Lsa-miR171a is another representative conserved miRNA, whose target, V5_gn_6_3180.1, a GRAS transcriptional factor, has opposite expression patterns (Figure 4E). A number of novel regulatory relationships were also revealed, including the relationship between the Asteraceae- and lettuce-specific miRNAs (Supplementary Table 13). Figures 4F,G lists the two representative lettuce-specific examples that have a strong negative correlation.

### Regulatory Network Exploration of Lettuce MicroRNAs

The regulatory elements of miRNAs and target genes do not exist independently, and further constitute a regulatory network. Taking into account the particularity of TFs, that is, they are important miRNA targets that can, in return, regulate miRNA expression, a regulatory network was explored, with TFs, miRNAs, and targets as basic elements. First, miRNA targets from 23 TF families were collected (Figure 3E), then the promoter sequences of miRNAs and target genes were extracted, and by a search of known binding motifs of these 23 TFs, a preliminary regulatory relationship of TFs on miRNA and miRNA target genes was constructed. At the same time, using the predicted regulatory relationship between miRNAs and targets, the regulatory relationship between miRNA and target genes was determined (Figure 5A). In detail, 1,051 interactions were found between TFs and miRNAs, named TMIs, and 73,133 interactions identified between TFs and miRNA targets, so called TTIs. Together with the 6,540 interactions predicted between miRNAs and targets (MTIs), 46,083 cascades binding TMIs and MTIs were achieved, and 43,554 FFL motifs were constructed (Figure 5A). Based on these FFLs and the links crossing them, a complex regulatory network with TFs, miRNAs, and targets was constructed (Figure 5B).

The regulatory network was constructed to further understand the regulation between miRNAs, as the core, and a combination of TFs and target genes. In this network, any element centered on a specific miRNA can independently become a module. For example, miR408 accepts regulations from four TFs, and a dozen targets could be regulated by both miR408 and these four TFs (Figure 5C). As described previously, Lsa-miRN518 is an Asteraceae-specific miRNA (Figure 2D), and its 30 targets can also be regulated by several TFs (Figure 5D). Figure 5E shows a lettuce-specific miRNA, Lsa-miRN46, which has a relatively simple FFL module. When the function of each gene was added into these modules, their functions could be further determined. Using Figure 5F as an example where all non-TFs and non-miRNA nodes were filtered out, the regulatory network uncovered numerous new links between miRNAs and TFs. In this two particles of the large network, regulations between MYB to miR156, miR156 to SBP, and miR396 to GRF and WRKY, and miR166 to HD-ZIP, have been well established by previous studies (Wu et al., 2009; Zhu et al., 2011; Liebsch and Palatnik, 2020). However, this data supports that more TFs could regulate miR156, and the targets of miR156 could further regulate the modules of miR399 and miRN565. Moreover, more modules are involved in the regulation network of miR396 and miR166, forming a seven-layer network system. Based on the known functions of these miRNAs and TFs, these two network particles, could potentially be involved in development, circadian clock, flowering, response to insect damage and environmental challenges, etc.

### Functional Study of Lsa-MIR408

**FIGURE 6:**
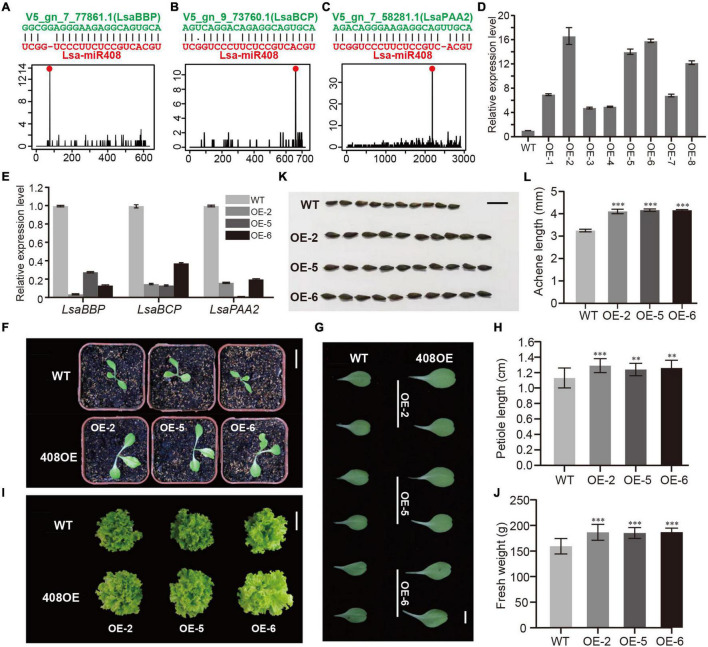
Functional study of Lsa-miR408. **(A–C)** Sequence alignments and degradome profiles of three target genes of Lsa-miR408. The top of each panel shows miRNA-target alignment, while the bottom diagram represents the reads count of degradome data across the target transcript, where the red dot indicates miRNA cleavage site. **(D)** Relative expression levels of miR408 in transgenic lines and wild-type (WT) plants through qRT-PCR analysis. **(E)** Relative expression levels of three target transcripts in three transgenic lines and WT plants by qRT-PCR analysis. **(F)** 12-day seedlings of WT and three transgenic lines, bar = 5 cm. **(G)** Comparison of 12-day cotyledons between WT and three transgenic lines, bar = 1 cm. **(H)** Quantitative measurement of petiole length of 12-day WT and 408OE plants, bar = 1 cm. **(I)** Size comparison of 50-day WT and 408OE plants (top view), bar = 15 cm. **(J)** Quantitative measurement of fresh weight of 50-day WT and miR408-OE plants. **(K)** Comparison of achene morphology and achene size between WT and 408OE plants, bar = 50 mm. **(L)** Quantitative measurement of achene length of WT and 408OE lines. Among these panels, OE/408OE indicates the transgenic lines in which *Lsa-MIR408* was overexpressed. Error bars represent standard deviations (SDs), the number of plants and achenes for quantitative measurement are 20 and 30, respectively, *P* < 0.01 (^**^), and *P* < 0.001 (^***^) by Student’s *t*-test.

The above expression pattern analysis indicated that Lsa-miR408 is highly expressed in leaf, while Lsa-miR408 as a hub gene is in the miRNA regulatory network (Figure 5E and Supplementary Table 20). In addition, previous (Zhao et al., 2016; Zhang J. P. et al., 2017; Pan et al., 2018) research shows that miR408 is highly conserved in the plant kingdom, and has multiple roles in different life activities, especially in promoting growth which is critical to leafy vegetables. Thus, to further verify the function of the miRNA regulatory network, miR408 was chosen as an example, and a series of experiments were carried out. Supplementary Table 4 shows that only one miR408 locus was identified, namely Lsa-MIR408, which has a standard stem-loop and a comparable secondary structure to model species, such as Arabidopsis thaliana (Supplementary Figure 6A). Target gene prediction, together with the PARE-Seq experiment, identified that the top three targets of Lsa-miR408 are copper related genes: basic blue protein (LsaBBP), blue copper protein (LsaBCP), and copper-transporting ATPase PAA2 (LsaPAA2; Figures 6A–C, Supplementary Figure 6B, and Supplementary Table 21). Furthermore, two of the top three targets of Ath-miR408 are shared with Arabidopsis (Supplementary Figures 6C–E), suggesting that *Lsa-MIR408* might have similar functions as its companion in Arabidopsis. To further explore its function, an Lsa-miR408 overexpression construct was conducted and introduced into lettuce by *A. tumefaciens*-mediated plant transformation (see section “Materials and Methods”). Compared with wild type plants (WTs), eight independent transgenic lines showed higher accumulation of mature miR408, and were designated as OE/408OE (Figure 6D). Based on the expression level of miR408, three transgenic lines, OE-2, OE-5, and OE-6, were used in subsequent experiments. To test whether the higher expression level of miR408 is functional, the expression level of three target genes was examined. Quantitative reverse transcription PCR showed that expression of three targets was significantly suppressed in the three independent transgenic lines (Figure 6E), indicating that constitutive expression of miR408 is functional.

Compared to WTs, 408OE plants showed increased growth vigor, exhibiting larger leaves and longer petiole at the seedling stage (Figures 6F–J). The petiole of left side blade of 15-day seedlings was used as representatives to quantitatively detect differences in petiole length. Petiole length of 408OE plants increased by 20% (Figures 6G,H). At the harvest stage, transgenic plants showed a significant improvement in vegetative development and size (Figure 6I). Thus, 50-day 408OE plants were weighed, and were significantly heavier (a 15% increase) than the respective control (Figure 6J). Achenes in the transgenic line were also significantly larger than those of WTs (Figures 6K,L), and the achenes of 408OE plants were 20% longer than the controls (Figure 6L). All of these phenotypic changes are comparable to these in Arabidopsis (Jiang et al., 2021) and rice (Zhang J. P. et al., 2017), suggesting the similar molecular mechanism of miR408 exists in lettuce.

## Discussion

The systematic identification and annotation of miRNAs is the first step in studying their functions in a specific species. In this study, based on the lettuce reference genome (Reyes-Chin-Wo et al., 2017), miRNAs in lettuce were systematically annotated by sRNA sequencing and the plant miRNA identification pipeline (Kuang et al., 2019) developed by our laboratory. For the 157 high confidence miRNAs, detailed basic information is provided, such as sequences, locations, structures, miRNA clusters, etc., as well as an analysis of the overall characteristics of these miRNAs, such as the length distribution of precursors, the base preference of the first nt of mature miRNA, conservation between species, and the first attempt to scan their selection after the WGT event. Furthermore, the expression pattern of these miRNAs in four selected tissues was established through sRNA sequencing. The combination of these analyses provides a strong foundation for understanding and mining miRNA functions in lettuce.

The function of a miRNA is mainly reflected in its targets, and accurate prediction of miRNA targets is critical for exploring miRNA functions. Through the psRNATarget (Dai et al., 2018) and RNAhybrid (Kruger and Rehmsmeier, 2006) pipelines, and high-throughput experimental verification, PARE-Seq, all potential target genes of miRNAs in lettuce were systematically identified. GO and KEGG analyses identified the diversity of these miRNA target genes, and also explained the significance of miRNA functions, being involved in almost all biological activities (Mi et al., 2019). According to the analysis of TFs, many conservative regulations were also detected in lettuce, such as miR156-SPB, miR172-AP2, etc., confirming reliability of these data. In addition, the expression patterns between miRNAs and their targets in different tissues were correlated: although the expression correlation among them is highly complicated, a general negative expression correlation was observed between miRNAs and their target genes.

In the past few years, plant miRNA research has continued to expand, and new functions of well-studied miRNAs are being continuously discovered. For example, miR156 research has moved beyond its role in development transition, and new functions in seed dormancy have been discovered (Miao et al., 2019), while the miR168-AOG1 module has a newly discovered role in plant immunity (Wang H. et al., 2021). At the same time, the study of the miRNA regulation network is becoming increasingly popular because it can promote the discovery of new miRNA functions or regulatory mechanisms. To this end, the miRNA regulatory network in lettuce was constructed, which centered on miRNAs and combined upstream TFs and downstream target genes. Since TFs are also an important class of miRNA target genes, the FFL motifs composed of miRNAs, miRNA targets, and TFs are powerful way to understand the miRNA regulatory network. The regulatory network constructed in this study, not only verified the conserved regulatory relationships found in other species, but discovered numerous novel regulatory modules and potential new connections between different modules. For example, a particle of this network displayed in Figure 5F could be involved in many biological activities, as the regulatory network centered on miR156, miR396, and miR166 has been expanded.

To further confirm the function of this regulation network, miR408 was selected as an example, given its role in promoting vegetative growth in model species (Zhao et al., 2016; Zhang J. P. et al., 2017; Pan et al., 2018). As one of the most commercially important leafy vegetables, accelerating vegetative growth and increasing the fresh weight are of great significance to the lettuce industry. Thus, via genetic experiments, the function of miR408 was verified. Compared with WT lettuce, the fresh weight of the over-expressed *Lsa-MIR408* lines was significantly increased. More interestingly, the plants overexpressing *Lsa-MIR408* can bear larger achenes, which has important potential for lettuce breeding and industrial application. Lettuce achenes in general are very small; therefore, increasing achene size is beneficial to mechanized achene screening and planting, as well as potentially increasing the yield of oil-rich achene lettuce, as described in watermelon (Wang Y. et al., 2021). The example of miR408 demonstrates the significance of constructing the miRNA regulatory network for understanding miRNA functions.

In brief, as an important economic crop and a representative Asteraceae species, research on the variation in morphology, color selection, and domesticated trait evolution of lettuce is very limited. Here, miRNAs in lettuce were systematically identified and annotated, and a comprehensive miRNA regulatory network was constructed. This research lays the foundation and provides resources for studying the functions of miRNAs in lettuce, and also provides new ideas for lettuce research and breeding.

## Data Availability Statement

The sequencing data for this study can be found in the NCBI Sequence Read Archive (SRA) under accession number PRJNA748591. All annotated miRNA loci were deposited into PmiREN database (https://www.pmiren.com/).

## Author Contributions

XY, LL, and XG initiated and designed the research. YD, ZK, and JD analyzed the data. YQ, PY, YZ, and YW performed the experiments. XY, YD, and PY wrote the manuscript. XY, LL, DL, and JW revised the manuscript. All authors contributed to the article and approved the submitted version.

## Conflict of Interest

The authors declare that the research was conducted in the absence of any commercial or financial relationships that could be construed as a potential conflict of interest.

## Publisher’s Note

All claims expressed in this article are solely those of the authors and do not necessarily represent those of their affiliated organizations, or those of the publisher, the editors and the reviewers. Any product that may be evaluated in this article, or claim that may be made by its manufacturer, is not guaranteed or endorsed by the publisher.

## Footnotes

1
http://www.fao.org/

2
https://www.zhaolab.org/psRNATarget/

3
http://planttfdb.gao-lab.org/prediction.php

4
http://plantregmap.gao-lab.org/

## Supplementary Material

Supplementary Table 1 Reads summary in sRNA libraries.

Supplementary Table 2 Length distribution of clean reads in sRNA libraries.

Supplementary Table 3 Length distribution of unique reads in sRNA libraries.

Supplementary Table 4 Summary of 157 miRNAs in lettuce.

Supplementary Table 5 List of miRNA clusters within 10 kb.

Supplementary Table 6 List of single miRNAs in synteny blocks.

Supplementary Table 7 List of paired miRNAs in synteny blocks.

Supplementary Table 8 Expression profile of 157 miRNAs in lettuce.

Supplementary Table 9 Differentially expressed miRNAs among tissues.

Supplementary Table 10 Detailed annotation information and expression profile of 6,540 miRNA-target pairs.

Supplementary Table 11 The result of gene ontology (GO) enrichment analysis of 3,629 candidate targets.

Supplementary Table 12 The result of Kyoto Encyclopedia of Genes and Genomes (KEGG) enrichment analysis of 3,629 candidate targets.

Supplementary Table 13 Correlation analysis of expression profile between 157 miRNAs and 3,629 targets.

Supplementary Table 14 Detailed information of 46,083 Cascade motifs based on “union” datasets.

Supplementary Table 15 Detailed information of 8,859 Cascade motifs based on “intersection” datasets.

Supplementary Table 16 Detailed information of 43,554 FFLs based on “union” datasets.

Supplementary Table 17 Detailed information of 8,424 FFLs based on “intersection” datasets.

Supplementary Table 18 Regulatory network statistics of 157 miRNAs as hub nodes based on “union” datasets.

Supplementary Table 19 Regulatory network statistics of 152 miRNAs as hub nodes based on “intersection” datasets.

Supplementary Table 20 The information between Lsa-miR408 and its target.

Supplementary Table 21 The result of gene ontology (GO) enrichment analysis of Lsa-miR408 targets.

Supplementary Table 22 Primers used in this study.

Supplementary Data 1 Results of categories 0–2 from PARE-Seq analysis (including three subfiles:1_1, 1_2, 1_3).
