# Supplementary figures and images for: Comprehensive Annotation and Functional Exploration of MicroRNAs in Lettuce (part 2 of 6)
Source: Front Plant Sci. 2021 Dec 24;12:781836. doi: 10.3389/fpls.2021.781836 (PMC8739914; doi:10.3389/fpls.2021.781836)

**T=Lsat\_1\_v5\_gn\_9\_26661.1\_Q=Lsa-miR164a\_S=364**

category=2\_p=0.760751033750871

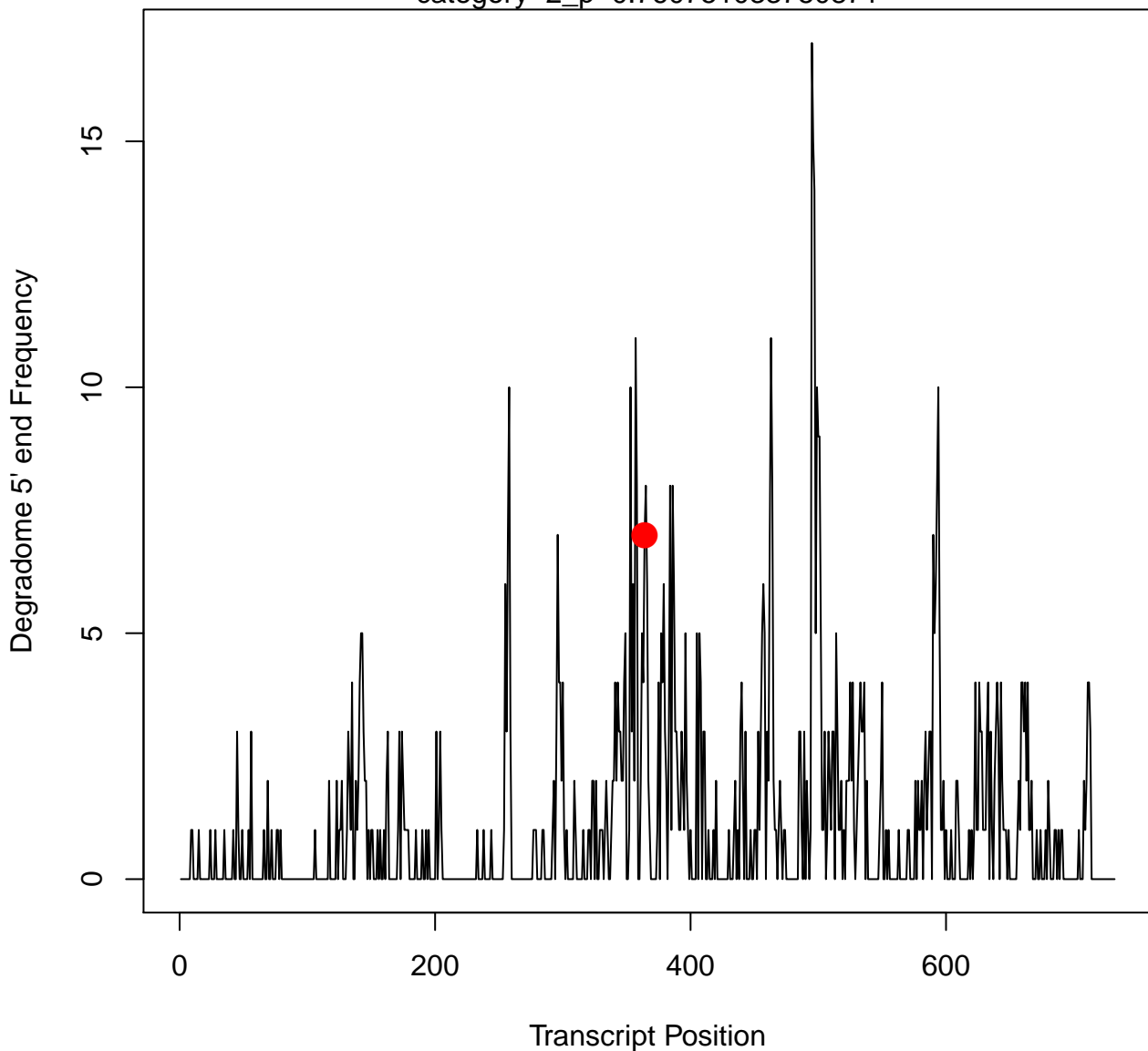

Supplement: Supplementary file 2 [file Data_Sheet_8.ZIP › GSM2230747.plot/Lsa-miR164a_Lsat_1_v5_gn_9_26661.1_364_TPlot.pdf]

**T=Lsat\_1\_v5\_gn\_8\_121820.1\_Q=Lsa-miR164b\_S=665**

category=2\_p=0.272276026438281

Degradome 5' end Frequency

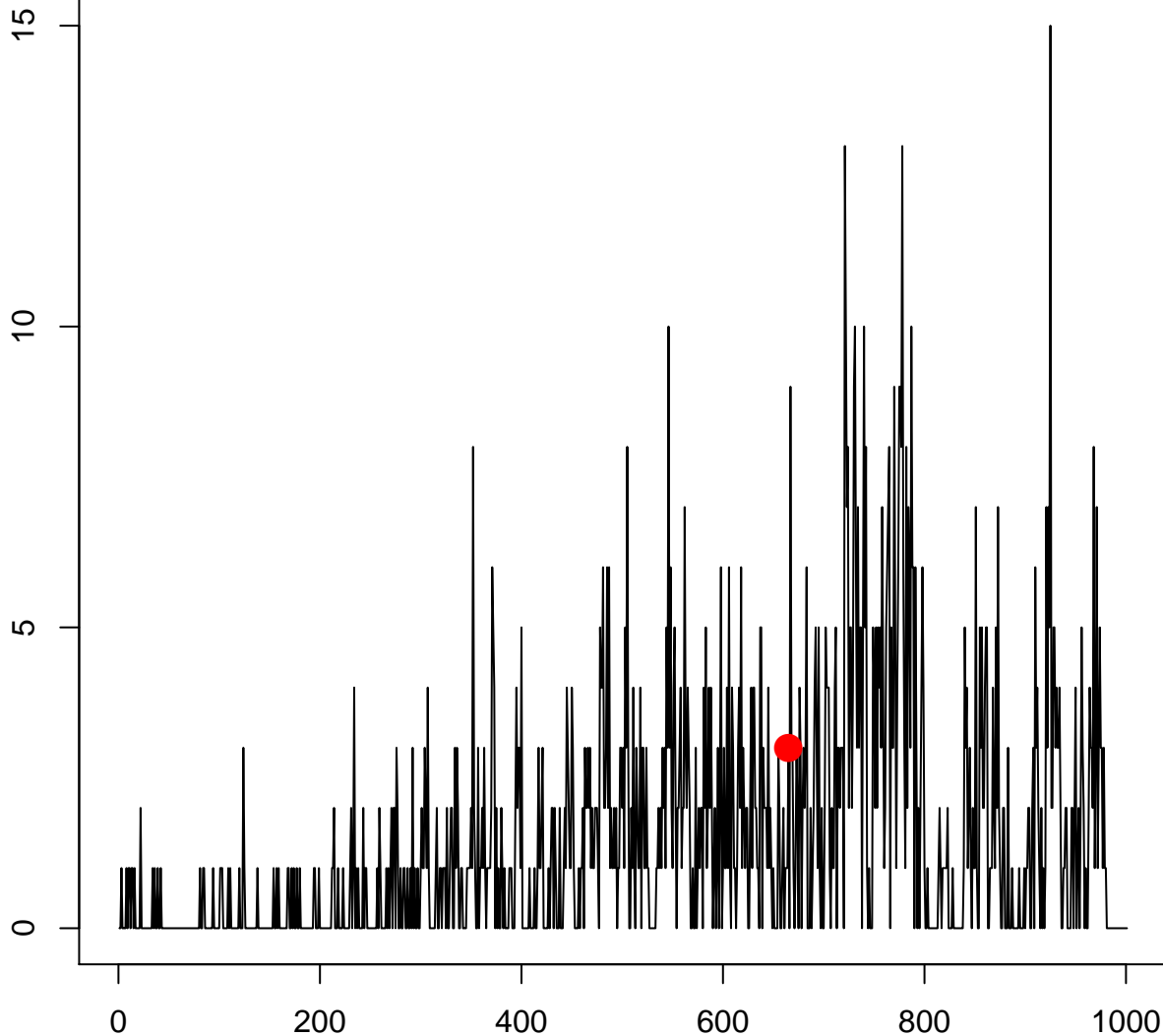

Transcript Position

Supplement: Supplementary file 2 [file Data_Sheet_8.ZIP › GSM2230747.plot/Lsa-miR164b_Lsat_1_v5_gn_8_121820.1_665_TPlot.pdf]

**T=Lsat\_1\_v5\_gn\_0\_45640.1\_Q=Lsa-miR164c\_S=643**

category=0\_p=0.00257945440126084

Degradome 5' end Frequency

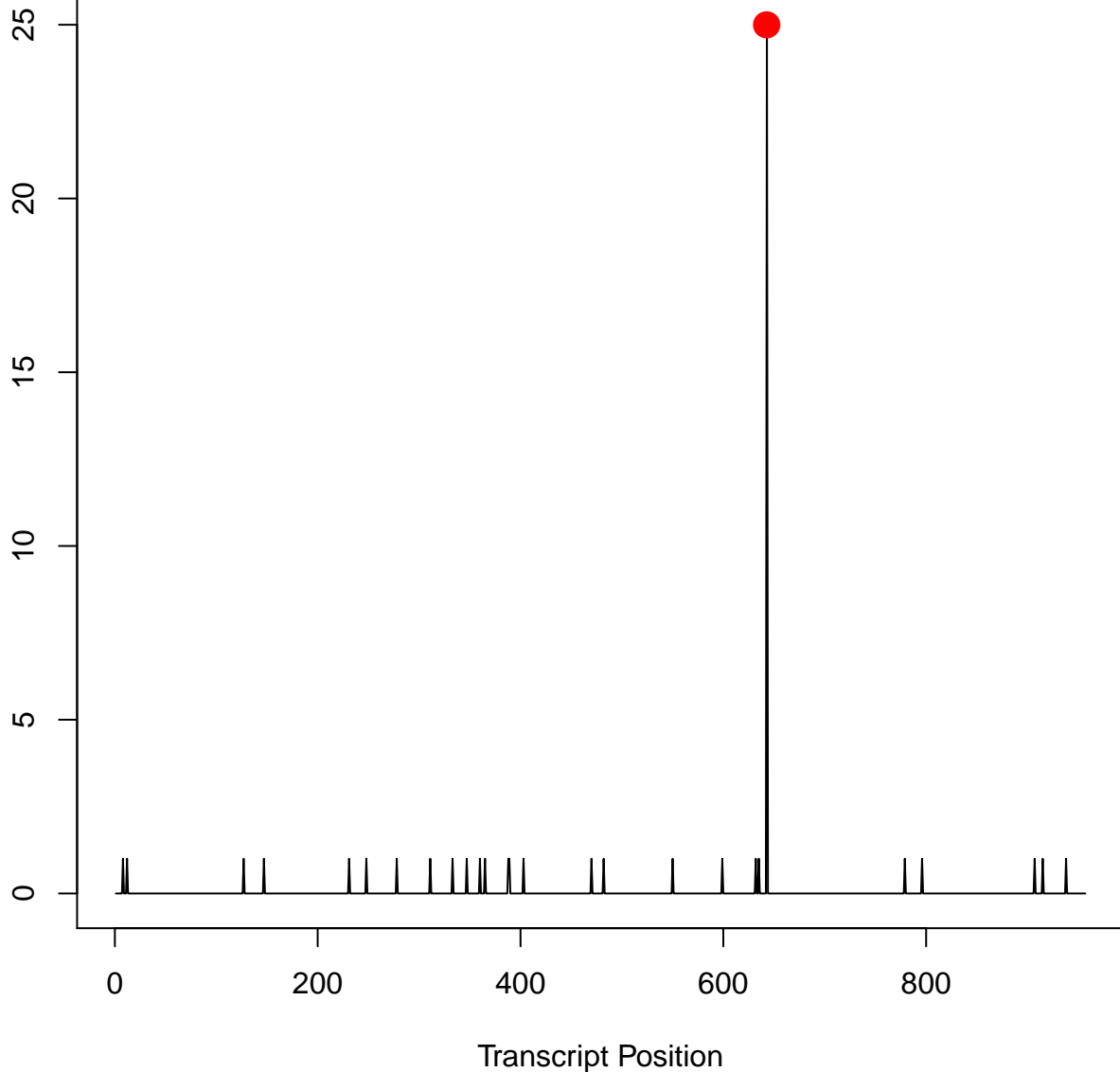

Supplement: Supplementary file 2 [file Data_Sheet_8.ZIP › GSM2230747.plot/Lsa-miR164c_Lsat_1_v5_gn_0_45640.1_643_TPlot.pdf]

**T=Lsat\_1\_v5\_gn\_4\_169300.1\_Q=Lsa-miR164c\_S=643**

category=0\_p=0.00221136867856231

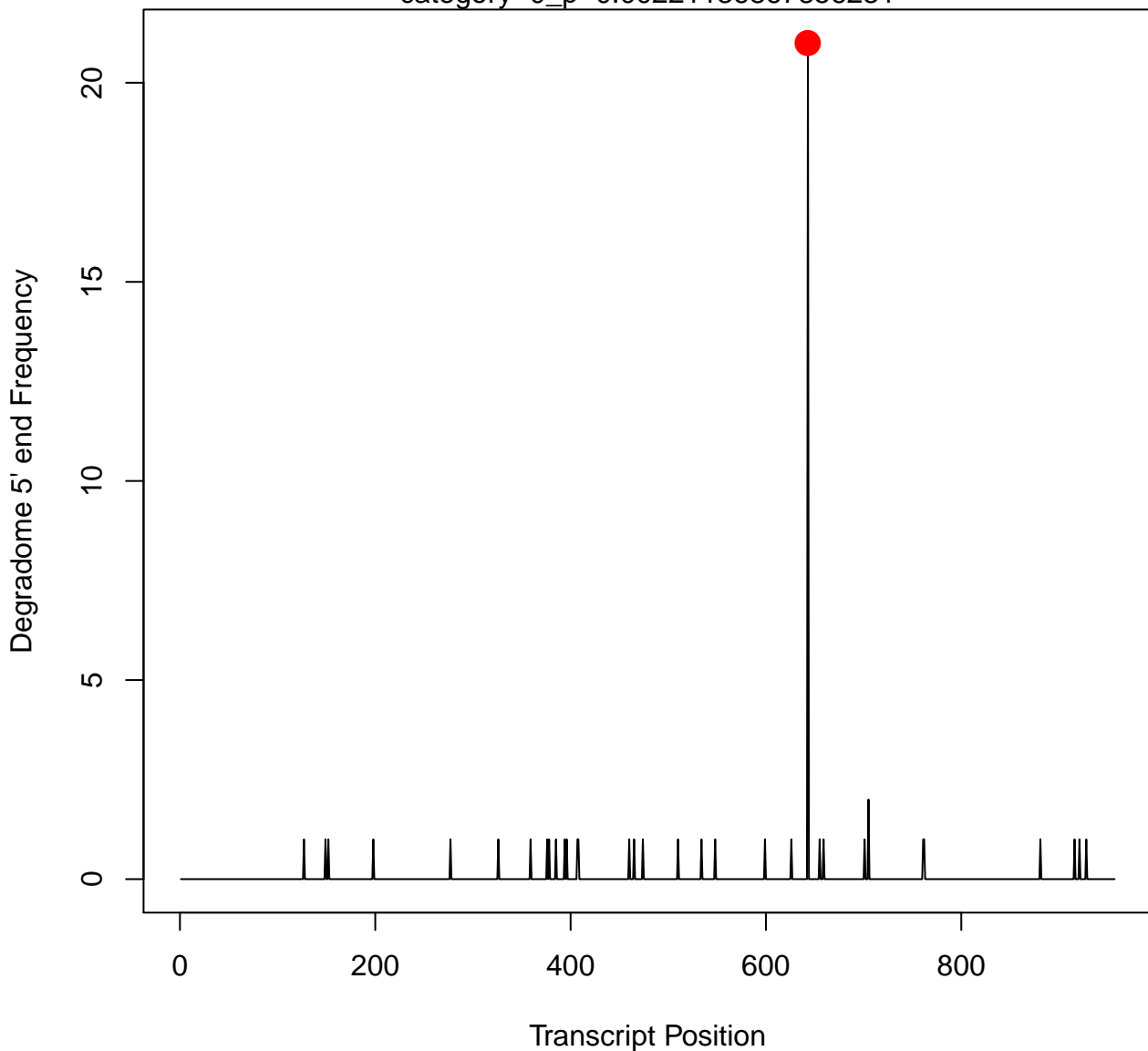

Supplement: Supplementary file 2 [file Data_Sheet_8.ZIP › GSM2230747.plot/Lsa-miR164c_Lsat_1_v5_gn_4_169300.1_643_TPlot.pdf]

**T=Lsat\_1\_v5\_gn\_4\_62541.1\_Q=Lsa-miR164c\_S=396**

category=2\_p=0.562363663605761

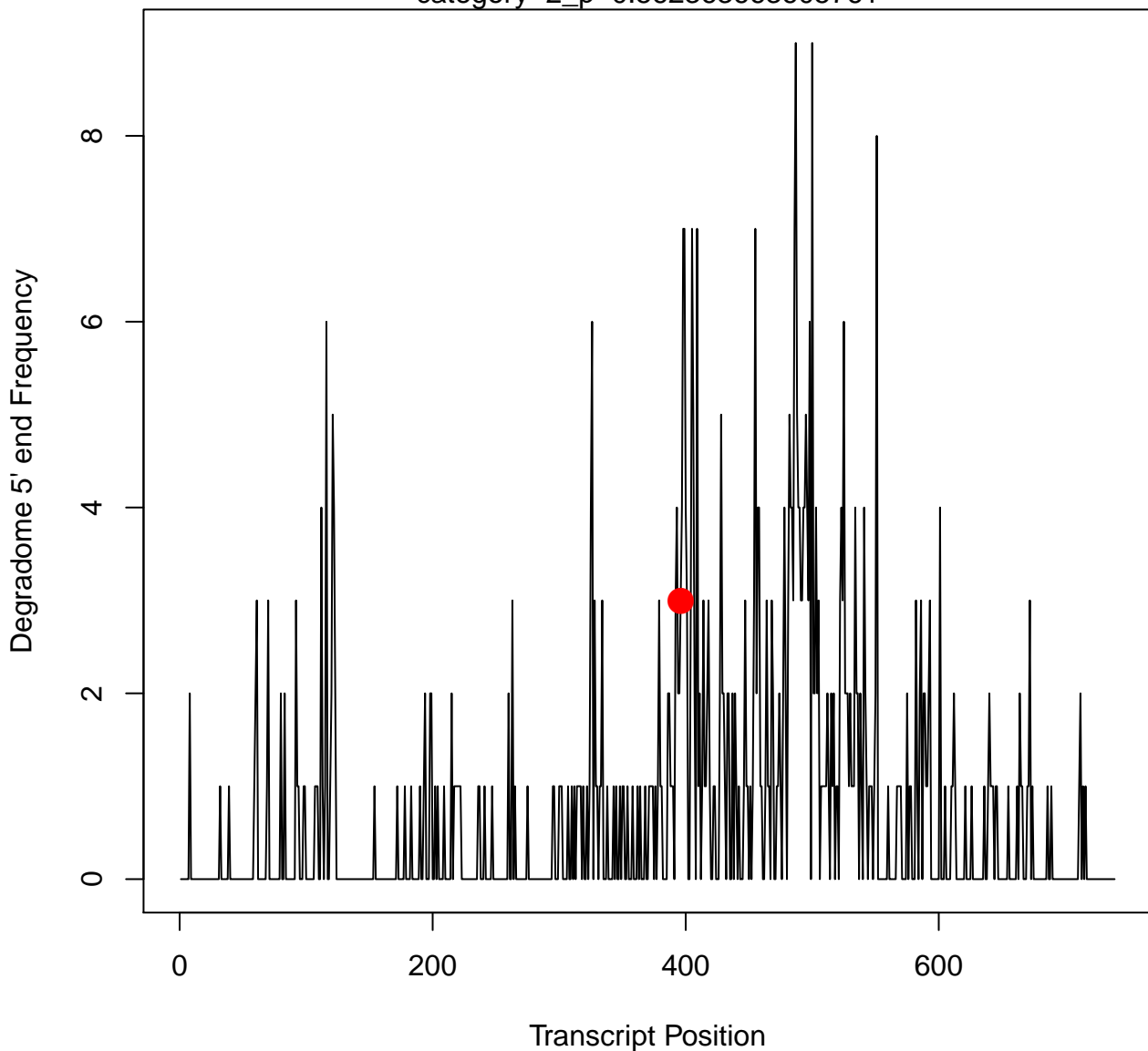

Supplement: Supplementary file 2 [file Data_Sheet_8.ZIP › GSM2230747.plot/Lsa-miR164c_Lsat_1_v5_gn_4_62541.1_396_TPlot.pdf]

**T=Lsat\_1\_v5\_gn\_5\_20601.1\_Q=Lsa-miR164c\_S=655**

category=0\_p=0.00110629628501624

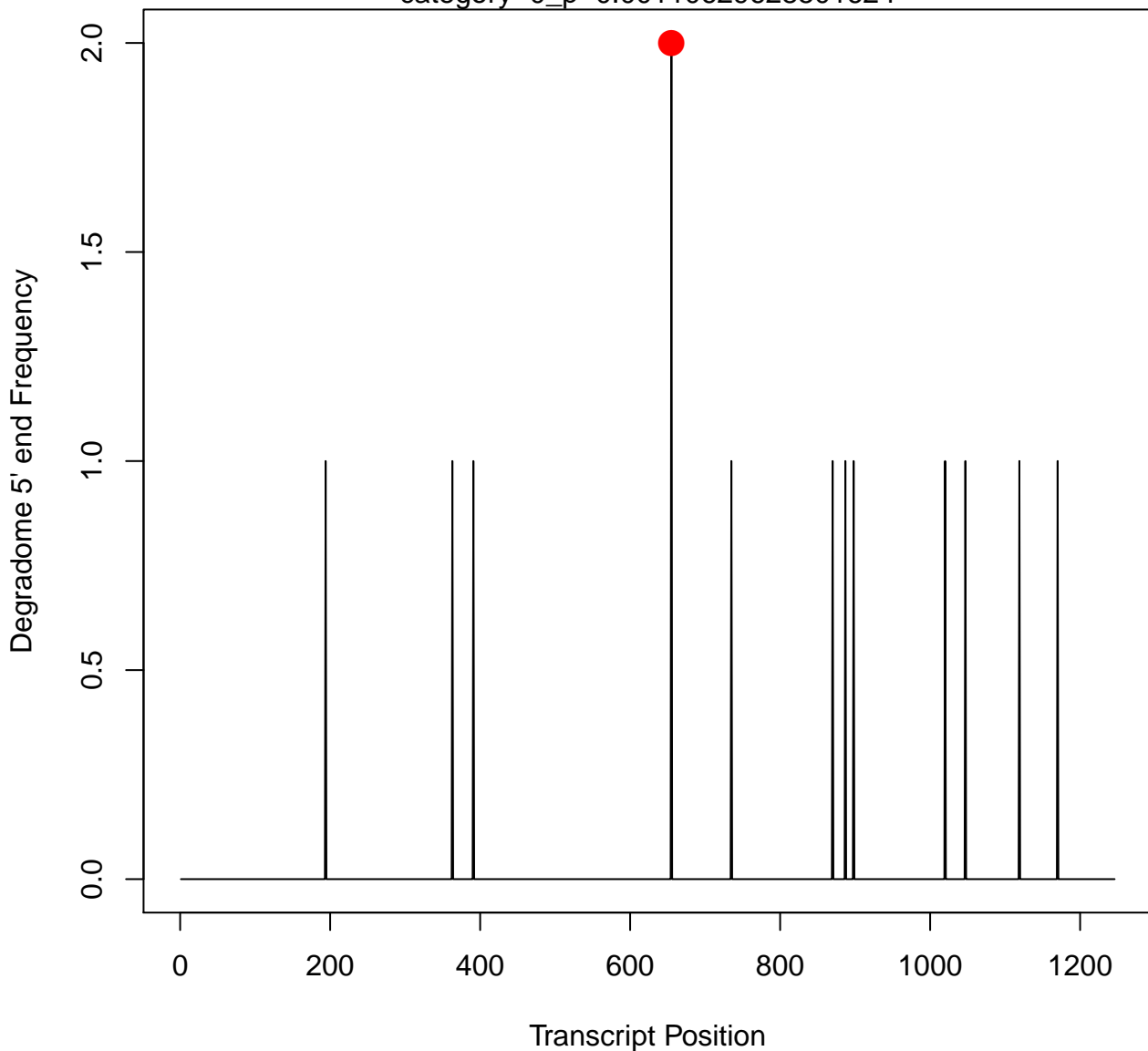

Supplement: Supplementary file 2 [file Data_Sheet_8.ZIP › GSM2230747.plot/Lsa-miR164c_Lsat_1_v5_gn_5_20601.1_655_TPlot.pdf]

**T=Lsat\_1\_v5\_gn\_5\_981.1\_Q=Lsa-miR164c\_S=601**

category=0\_p=0.00147478967057835

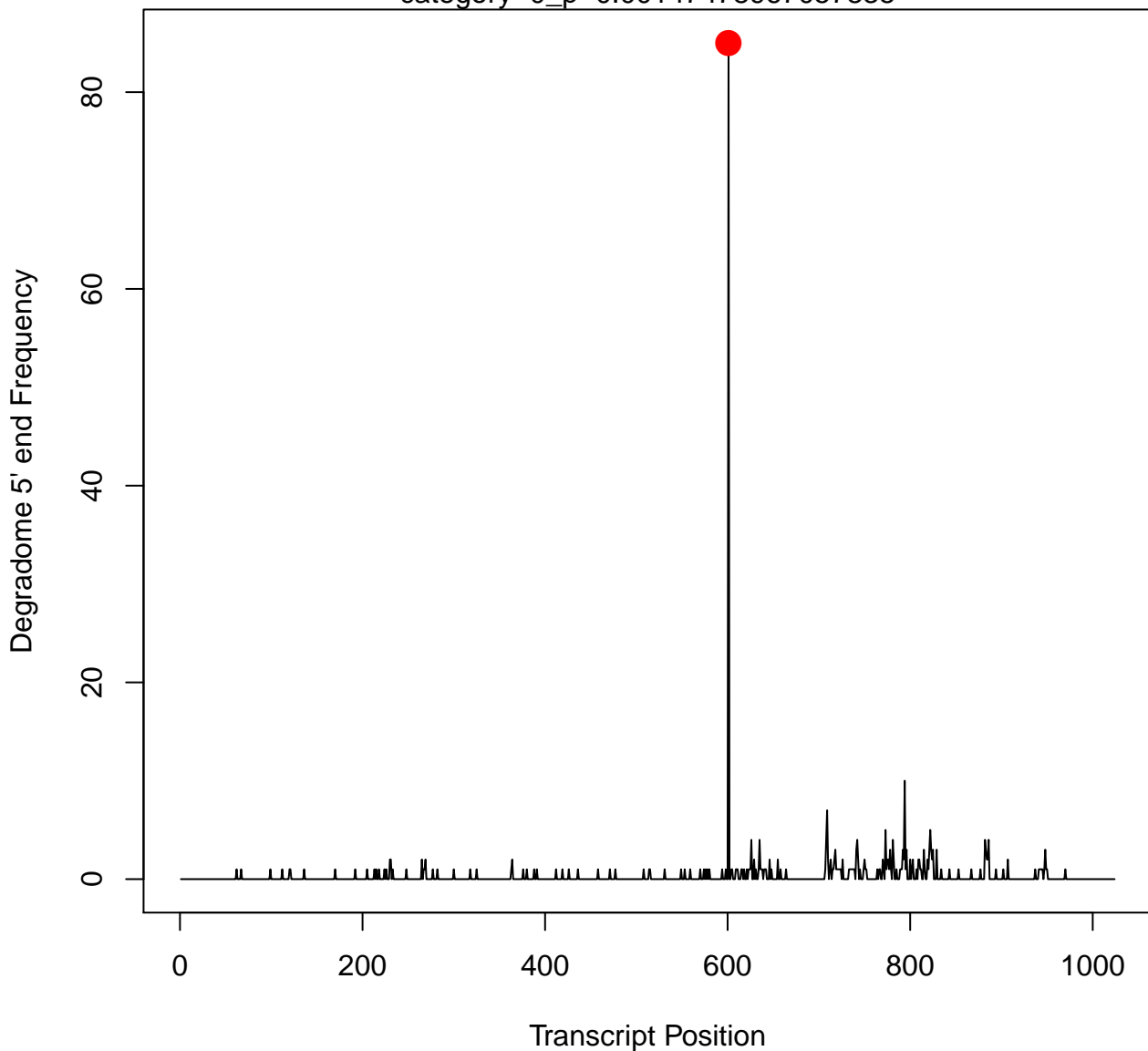

Supplement: Supplementary file 2 [file Data_Sheet_8.ZIP › GSM2230747.plot/Lsa-miR164c_Lsat_1_v5_gn_5_981.1_601_TPlot.pdf]

**T=Lsat\_1\_v5\_gn\_6\_32921.1\_Q=Lsa-miR164c\_S=810**

category=2\_p=0.975733330133494

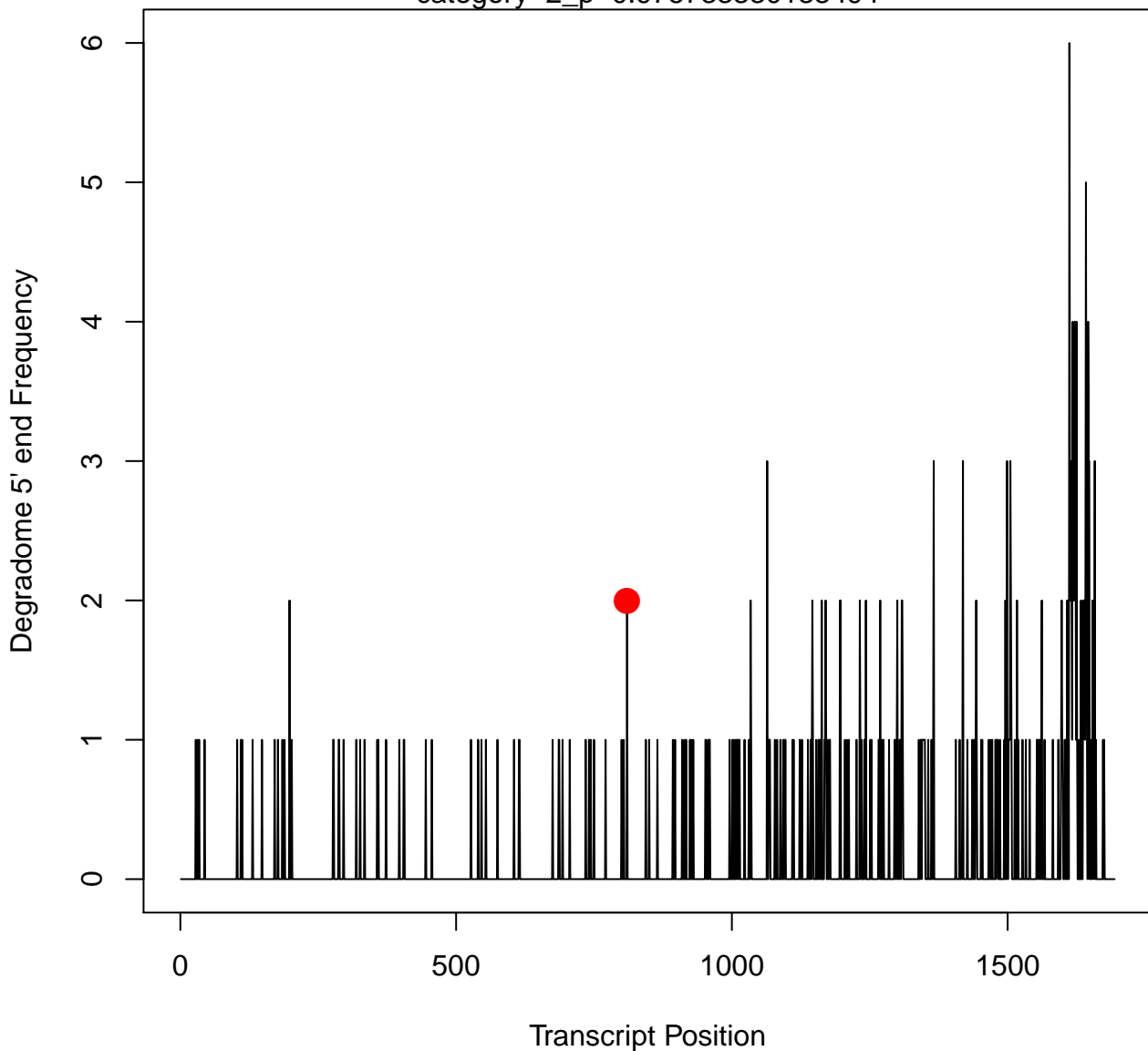

Supplement: Supplementary file 2 [file Data_Sheet_8.ZIP › GSM2230747.plot/Lsa-miR164c_Lsat_1_v5_gn_6_32921.1_810_TPlot.pdf]

**T=Lsat\_1\_v5\_gn\_7\_104460.1\_Q=Lsa-miR164c\_S=727**

category=0\_p=0.000368901499921082

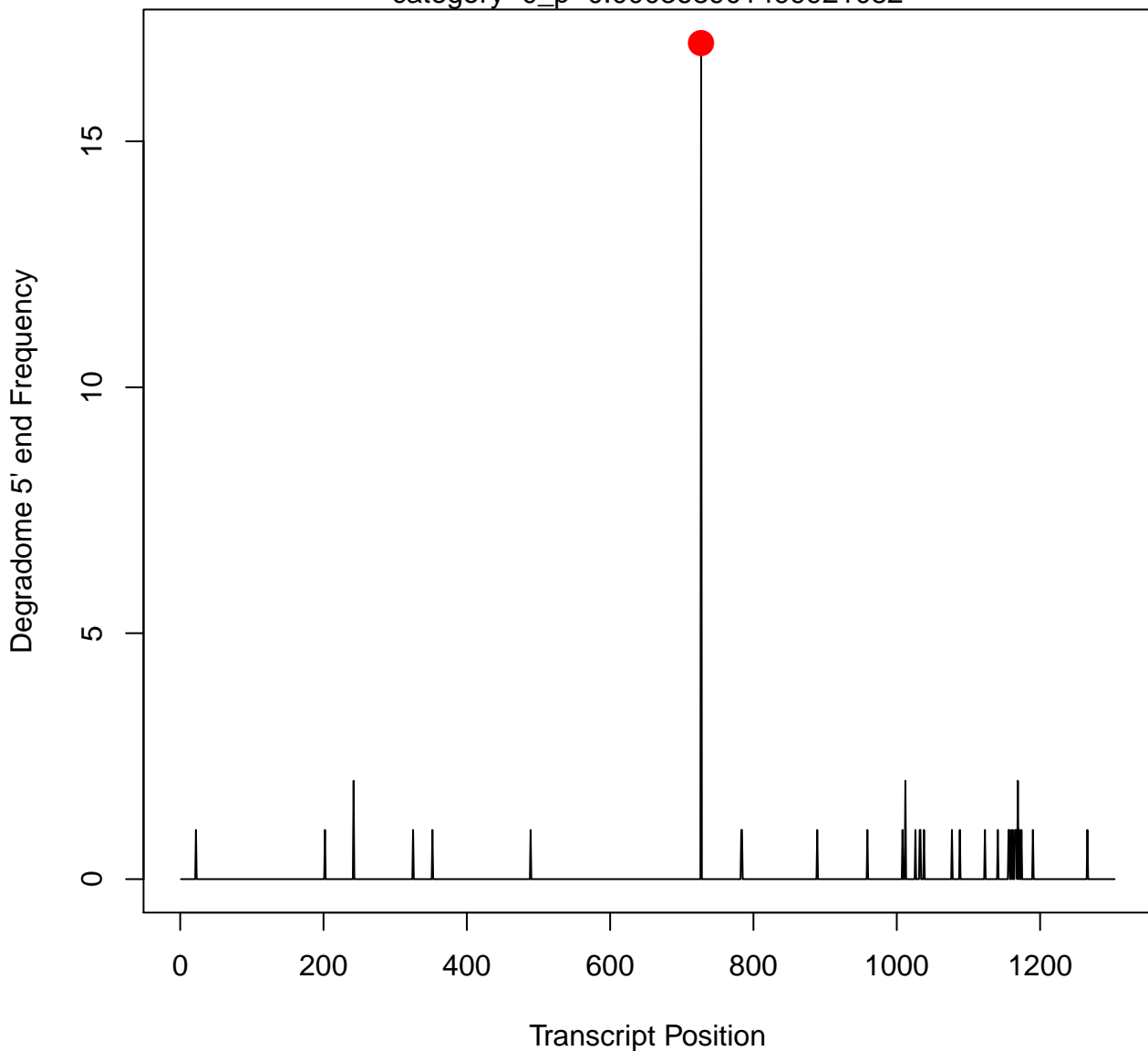

Supplement: Supplementary file 2 [file Data_Sheet_8.ZIP › GSM2230747.plot/Lsa-miR164c_Lsat_1_v5_gn_7_104460.1_727_TPlot.pdf]

**T=Lsat\_1\_v5\_gn\_9\_26661.1\_Q=Lsa-miR164c\_S=363**

category=2\_p=0.856121215077028

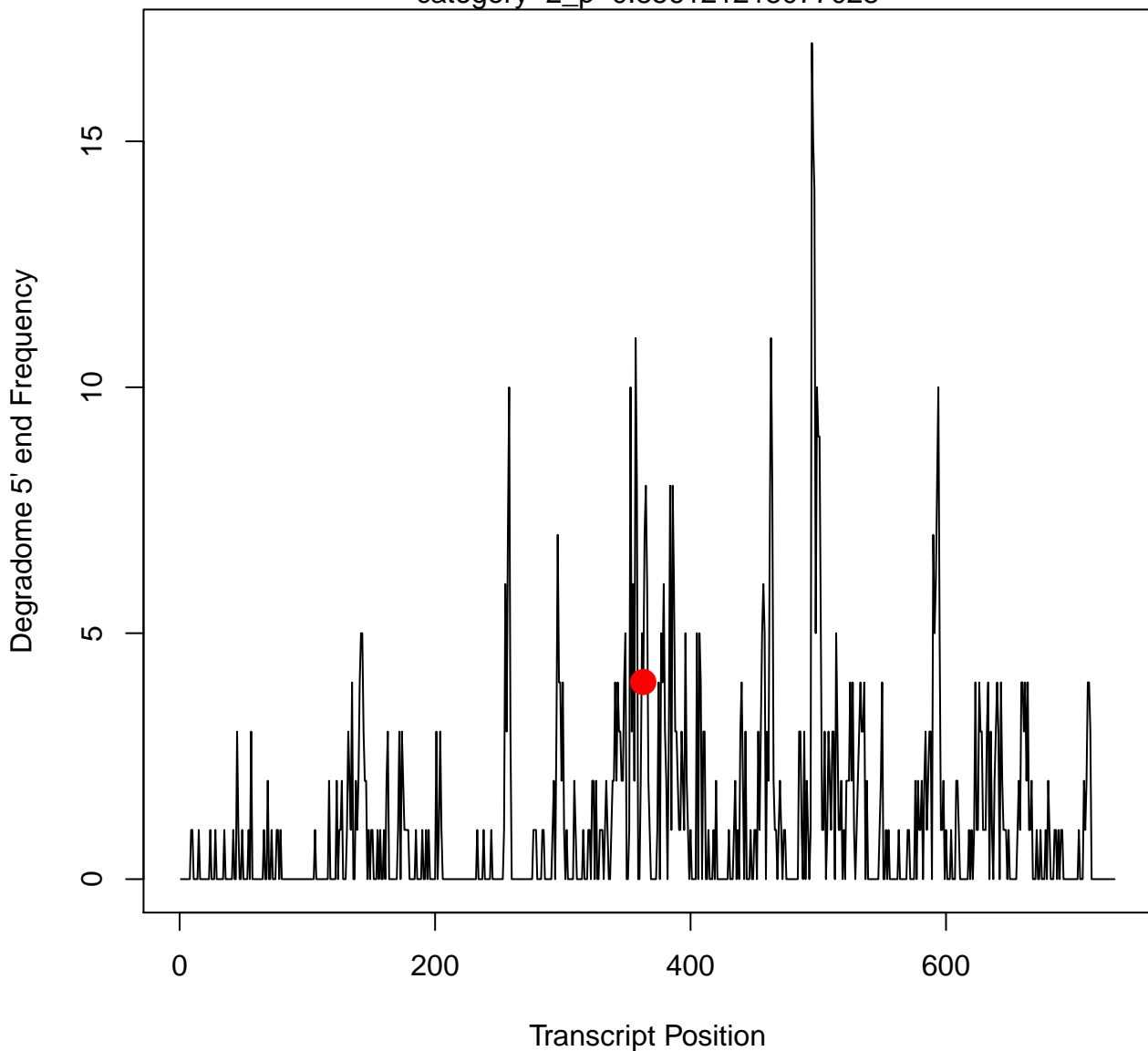

Supplement: Supplementary file 2 [file Data_Sheet_8.ZIP › GSM2230747.plot/Lsa-miR164c_Lsat_1_v5_gn_9_26661.1_363_TPlot.pdf]

**T=Lsat\_1\_v5\_gn\_4\_100080.1\_Q=Lsa-miR166a\_S=1067**

category=0\_p=0.000737666911525325

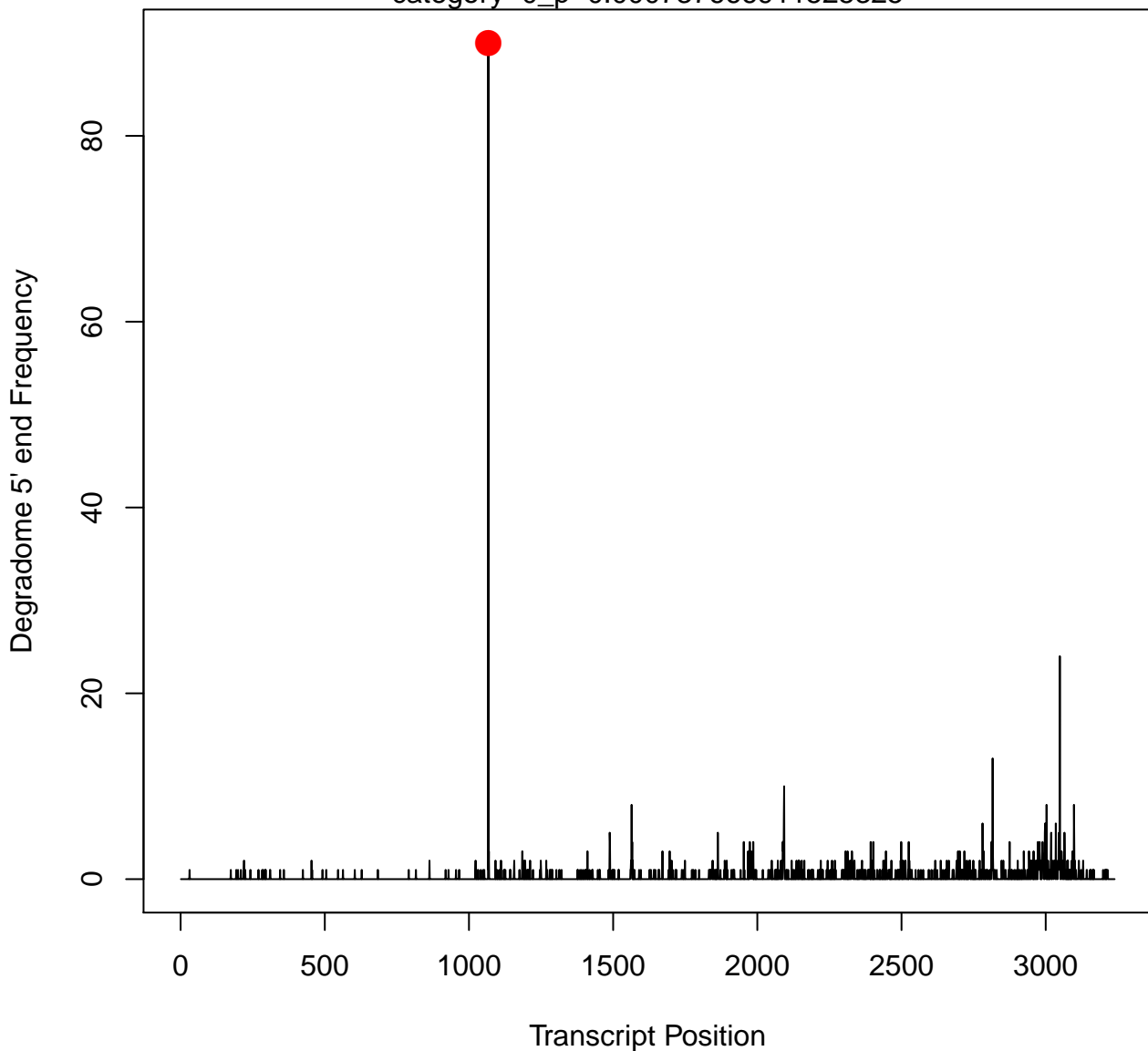

Supplement: Supplementary file 2 [file Data_Sheet_8.ZIP › GSM2230747.plot/Lsa-miR166a_Lsat_1_v5_gn_4_100080.1_1067_TPlot.pdf]

**T=Lsat\_1\_v5\_gn\_5\_134441.1\_Q=Lsa-miR166b\_S=568**

category=0\_p=0.000737666911525325

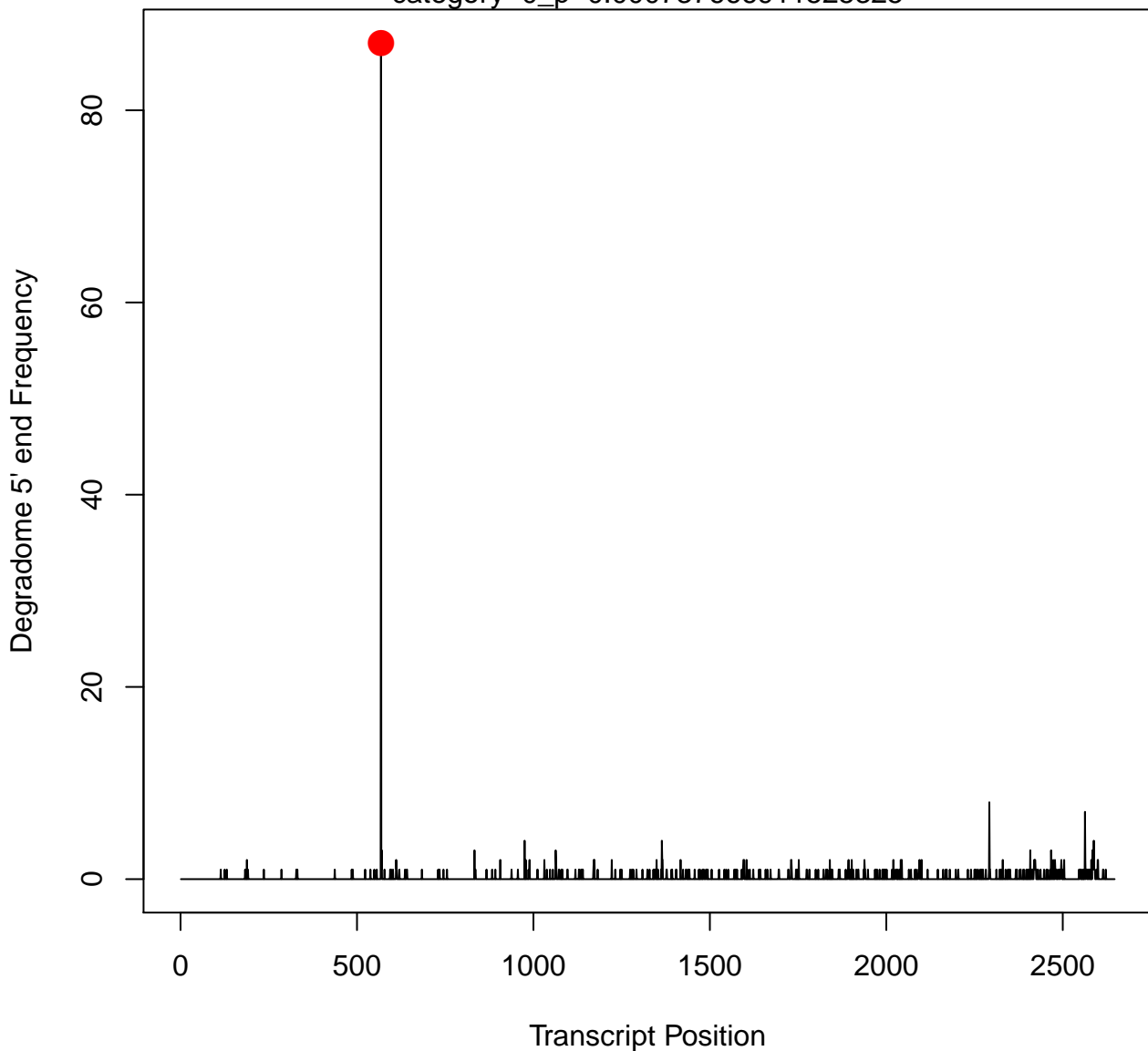

Supplement: Supplementary file 2 [file Data_Sheet_8.ZIP › GSM2230747.plot/Lsa-miR166b_Lsat_1_v5_gn_5_134441.1_568_TPlot.pdf]

**T=Lsat\_1\_v5\_gn\_6\_45641.1\_Q=Lsa-miR166b\_S=1309**

category=0\_p=0.00184314711837774

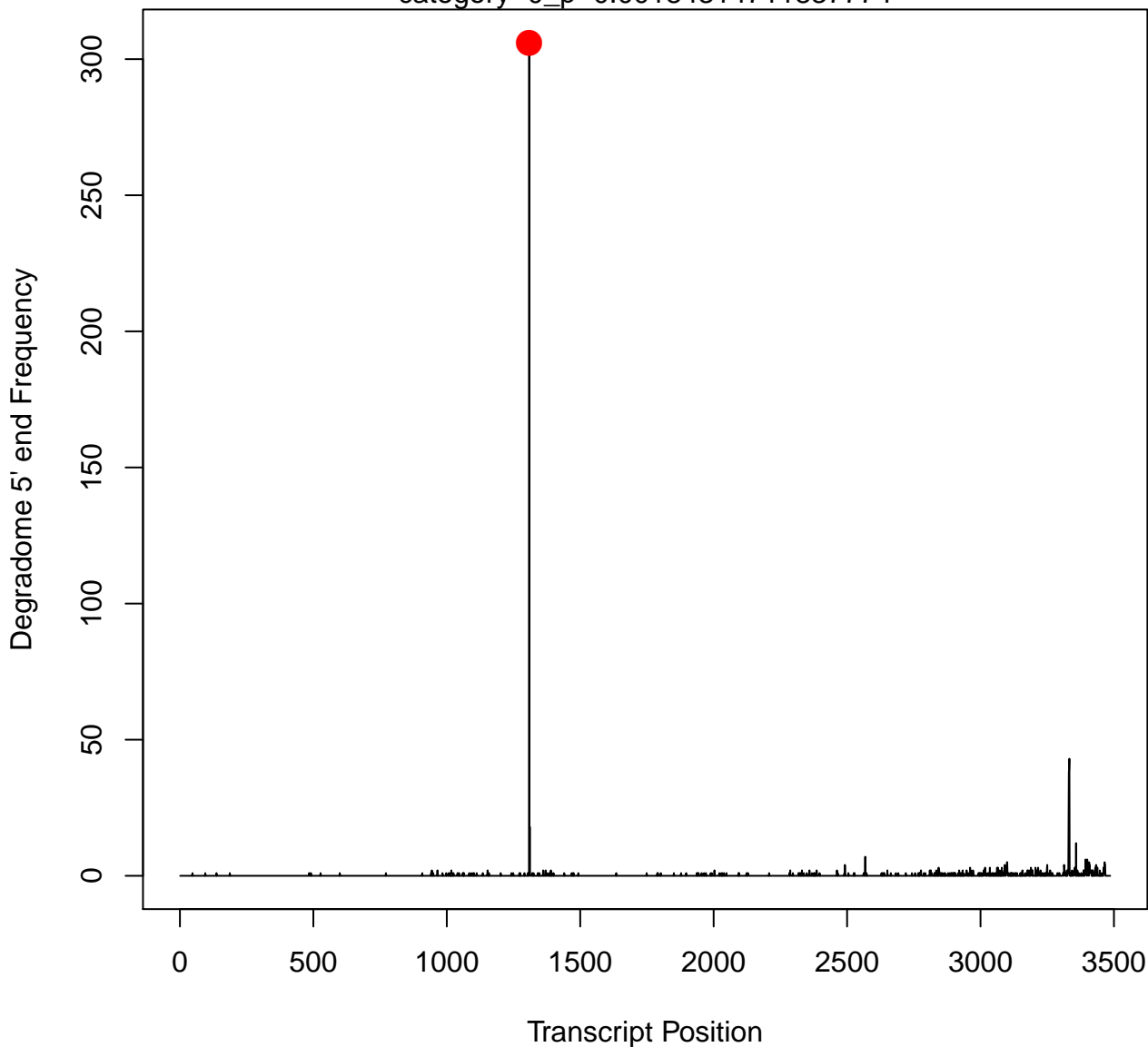

Supplement: Supplementary file 2 [file Data_Sheet_8.ZIP › GSM2230747.plot/Lsa-miR166b_Lsat_1_v5_gn_6_45641.1_1309_TPlot.pdf]

**T=Lsat\_1\_v5\_gn\_5\_32500.1\_Q=Lsa-miR166d\_S=565**

category=0\_p=0.00147478967057835

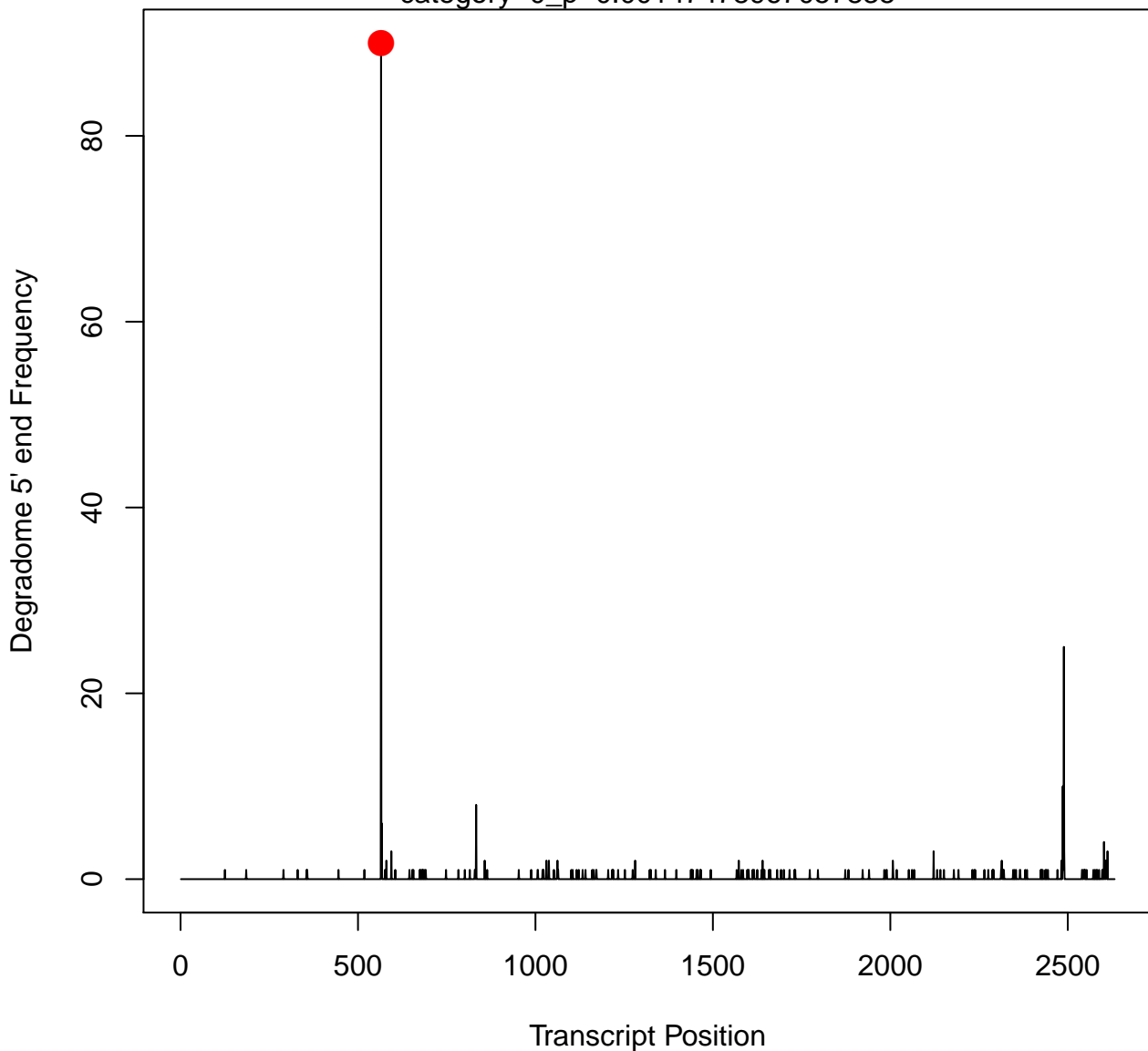

Supplement: Supplementary file 2 [file Data_Sheet_8.ZIP › GSM2230747.plot/Lsa-miR166d_Lsat_1_v5_gn_5_32500.1_565_TPlot.pdf]

**T=Lsat\_1\_v5\_gn\_4\_113220.1\_Q=Lsa-miR166e\_S=830**

category=2\_p=0.898571474531527

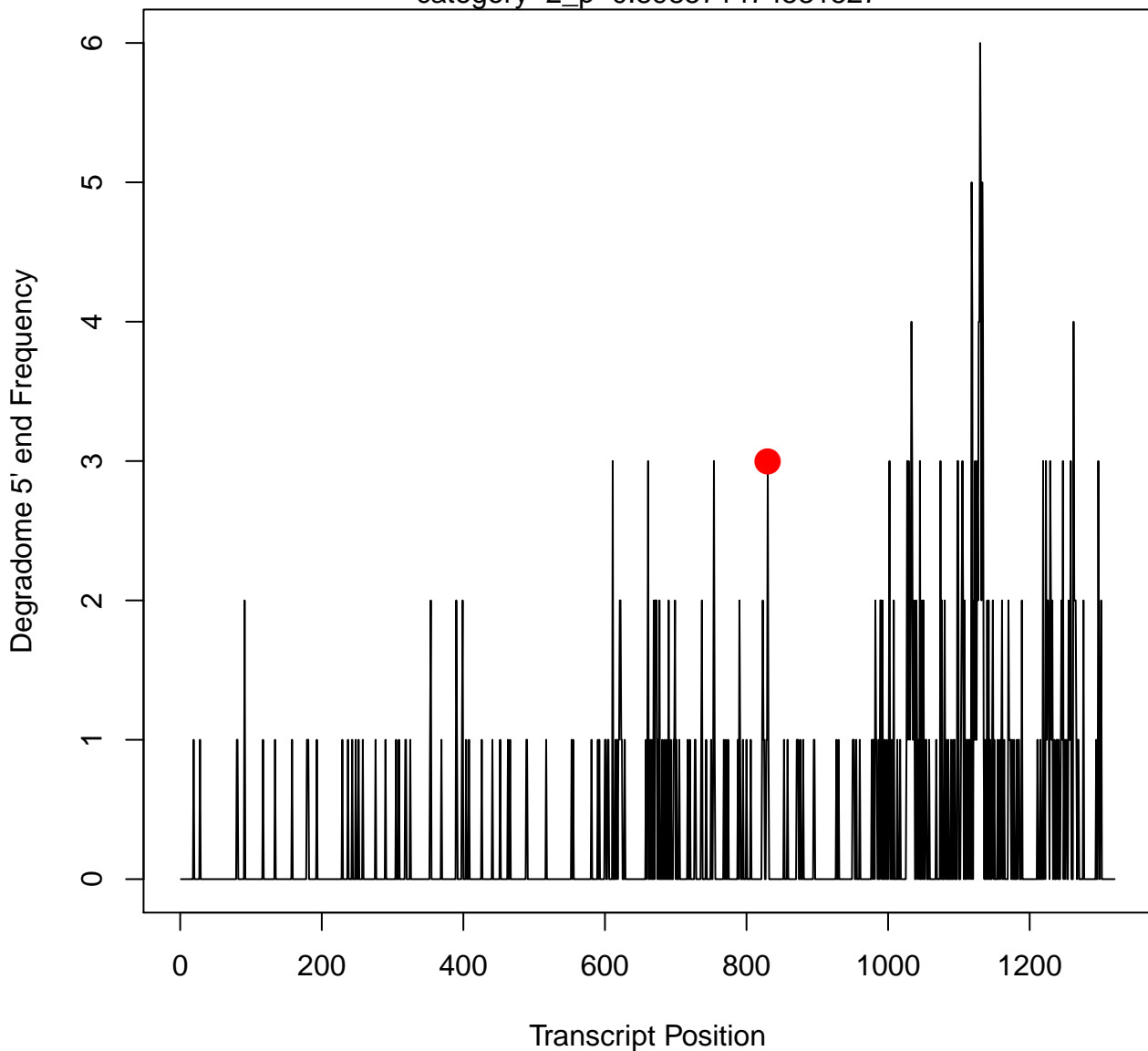

Supplement: Supplementary file 2 [file Data_Sheet_8.ZIP › GSM2230747.plot/Lsa-miR166e_Lsat_1_v5_gn_4_113220.1_830_TPlot.pdf]

**T=Lsat\_1\_v5\_gn\_2\_100140.1\_Q=Lsa-miR166f\_S=1400**

category=0\_p=0.00147478967057835

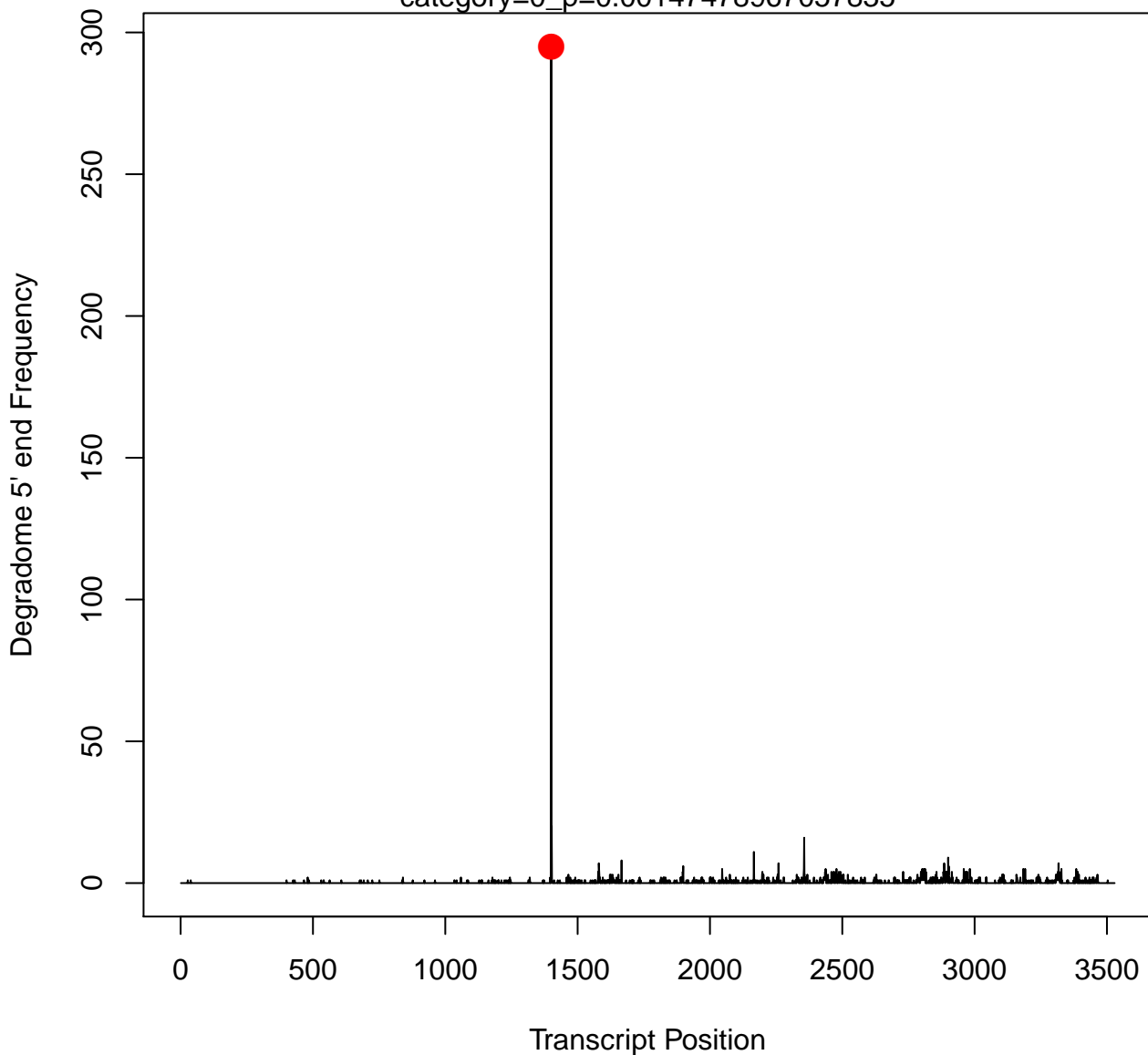

Supplement: Supplementary file 2 [file Data_Sheet_8.ZIP › GSM2230747.plot/Lsa-miR166f_Lsat_1_v5_gn_2_100140.1_1400_TPlot.pdf]

**T=Lsat\_1\_v5\_gn\_3\_10861.1\_Q=Lsa-miR166g\_S=514**

category=2\_p=0.98831756022218

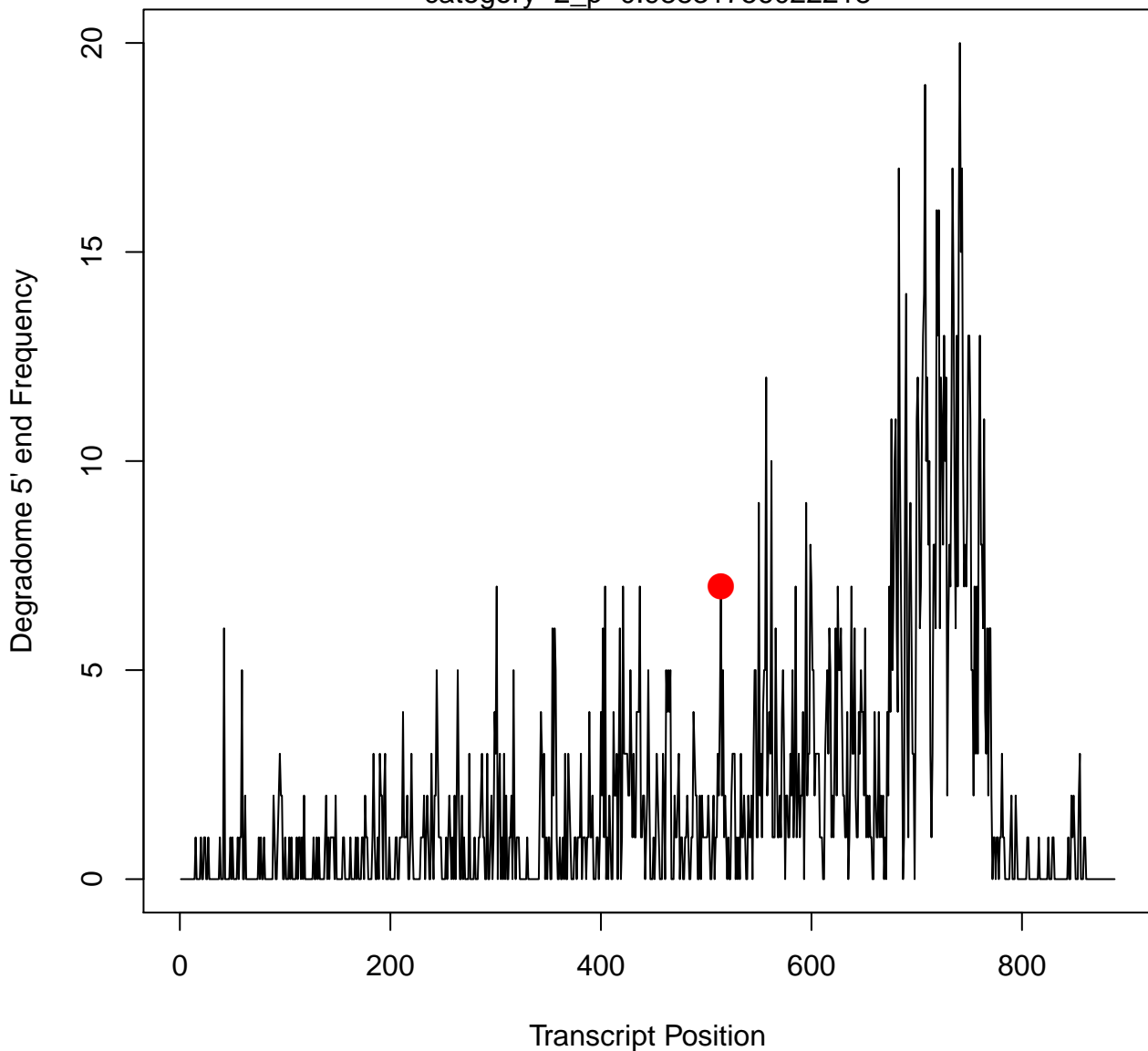

Supplement: Supplementary file 2 [file Data_Sheet_8.ZIP › GSM2230747.plot/Lsa-miR166g_Lsat_1_v5_gn_3_10861.1_514_TPlot.pdf]

**T=Lsat\_1\_v5\_gn\_7\_105501.1\_Q=Lsa-miR166g\_S=1457**

category=2\_p=0.626666712311993

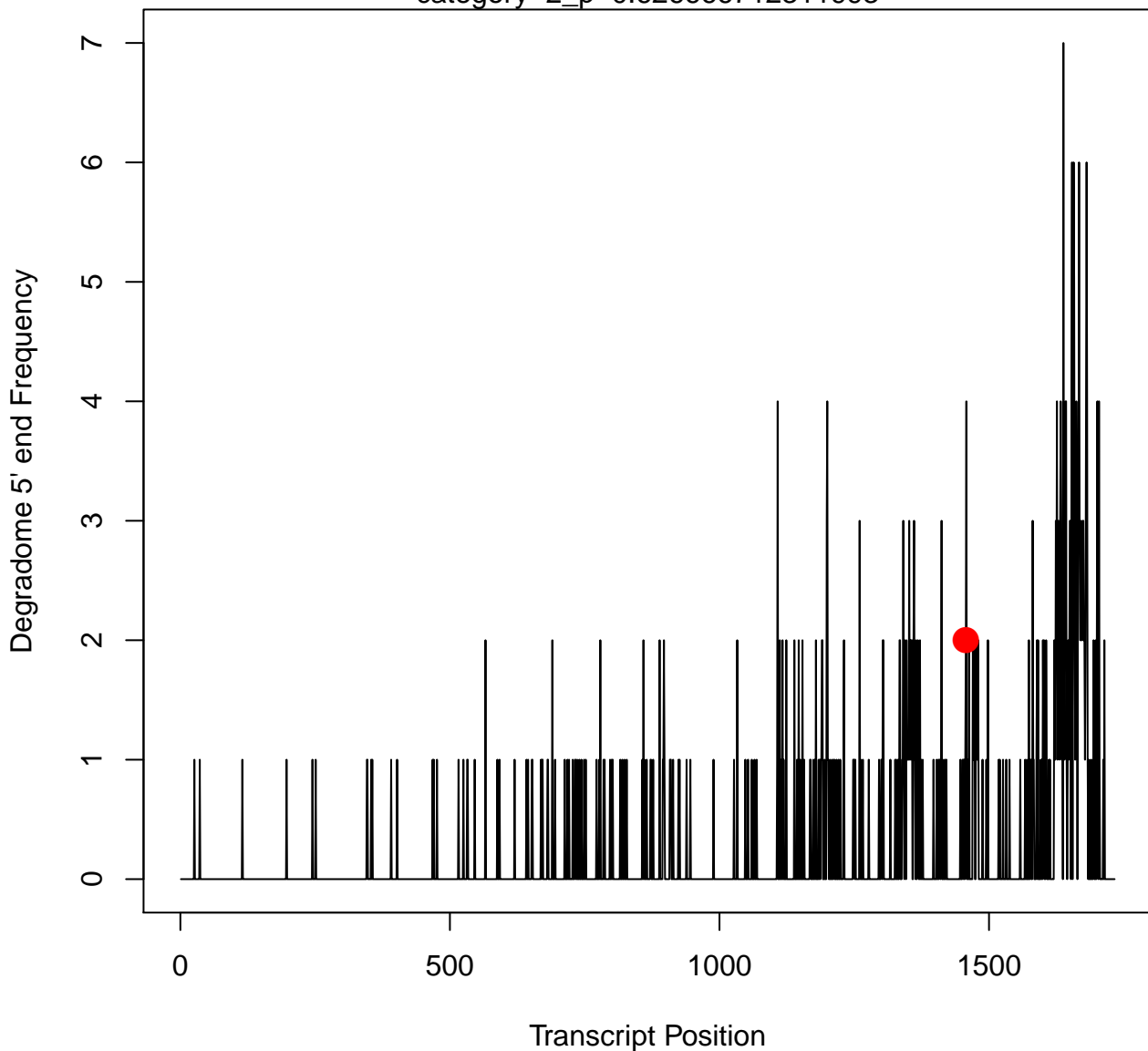

Supplement: Supplementary file 2 [file Data_Sheet_8.ZIP › GSM2230747.plot/Lsa-miR166g_Lsat_1_v5_gn_7_105501.1_1457_TPlot.pdf]

**T=Lsat\_1\_v5\_gn\_9\_89121.1\_Q=Lsa-miR166g\_S=1993**

category=2\_p=0.974140695184308

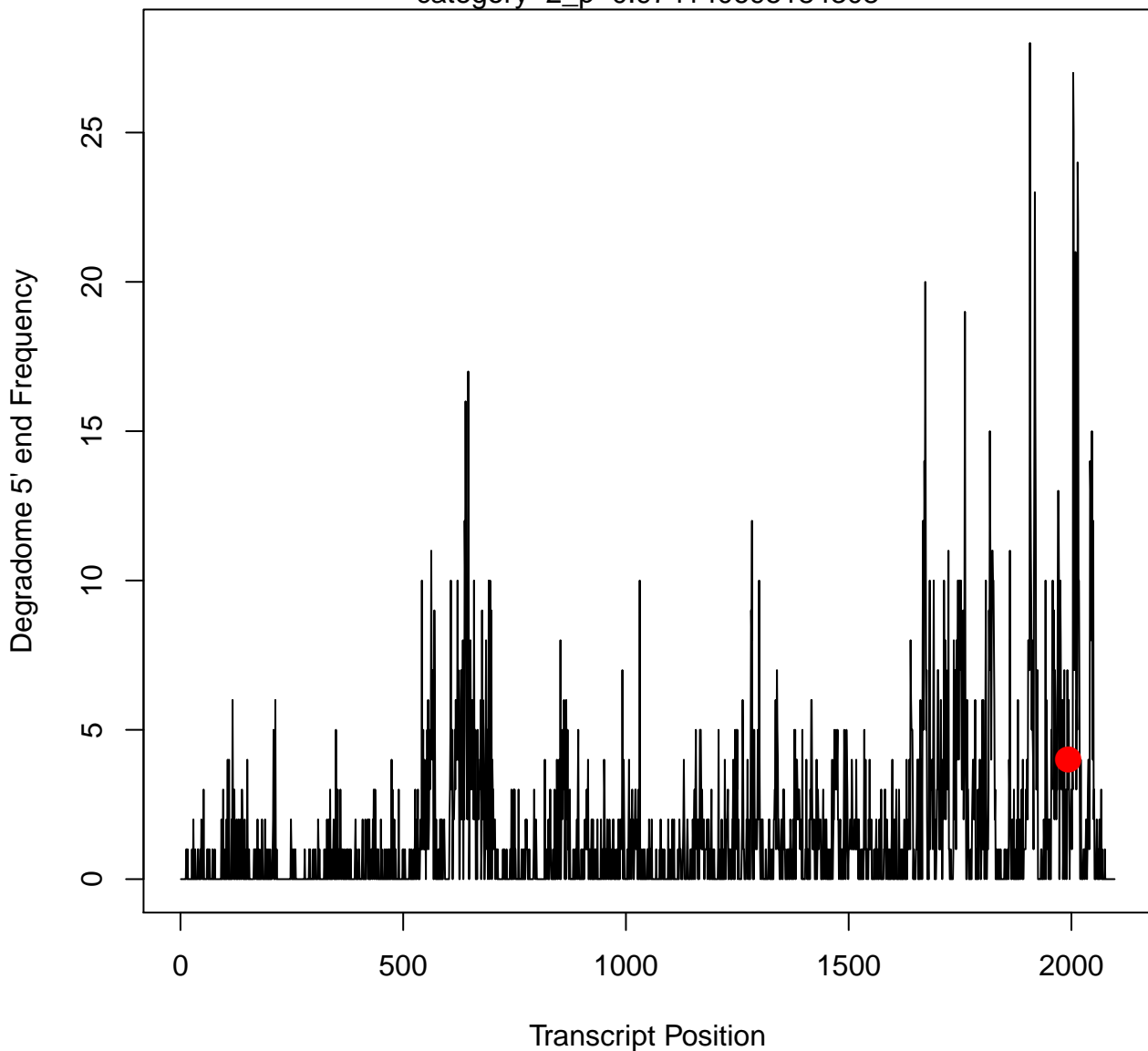

Supplement: Supplementary file 2 [file Data_Sheet_8.ZIP › GSM2230747.plot/Lsa-miR166g_Lsat_1_v5_gn_9_89121.1_1993_TPlot.pdf]

**T=Lsat\_1\_v5\_gn\_1\_37081.1\_Q=Lsa-miR166h\_S=1030**

category=0\_p=0.000368901499921082

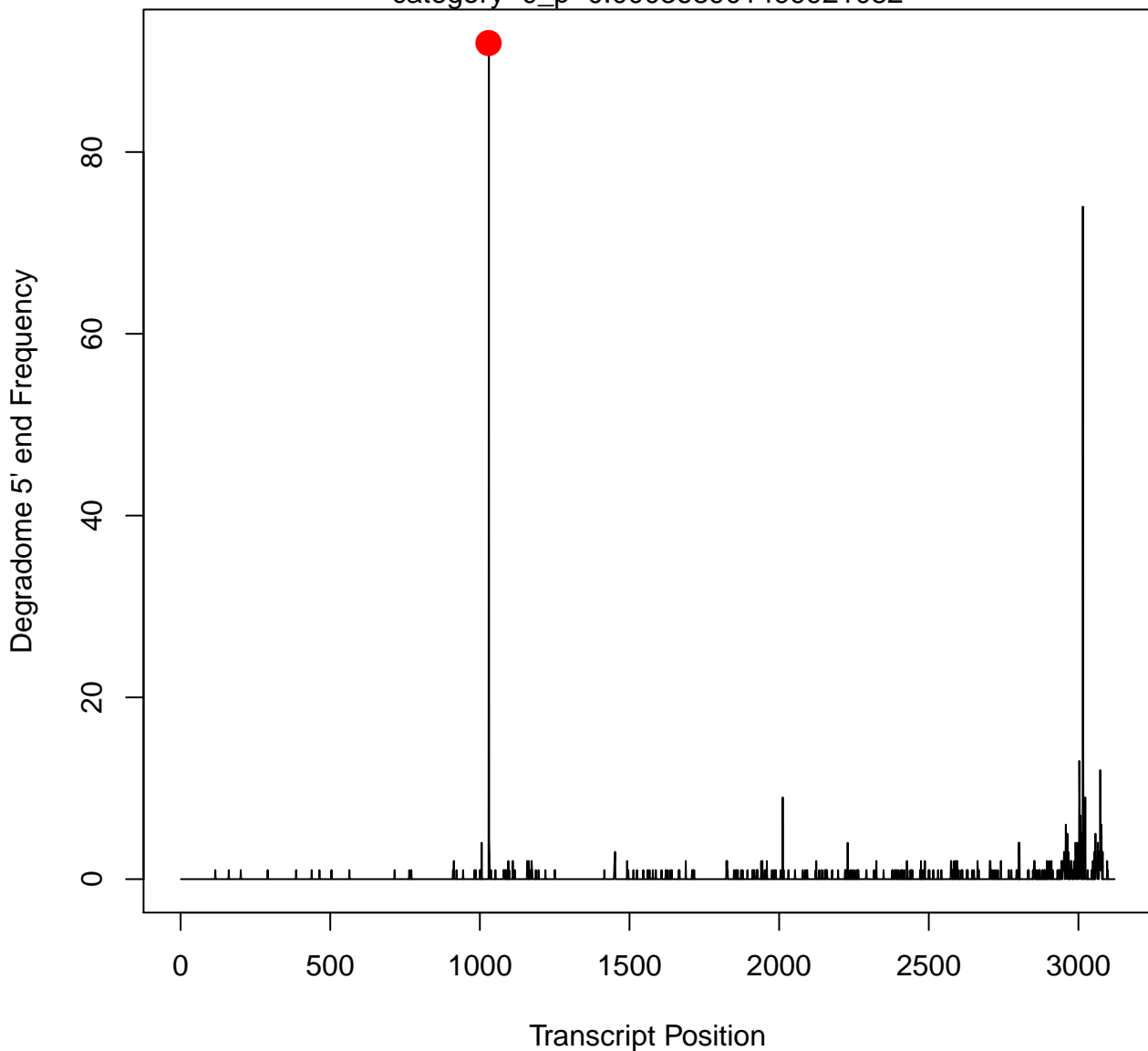

Supplement: Supplementary file 2 [file Data_Sheet_8.ZIP › GSM2230747.plot/Lsa-miR166h_Lsat_1_v5_gn_1_37081.1_1030_TPlot.pdf]

**T=Lsat\_1\_v5\_gn\_2\_103140.1\_Q=Lsa-miR166h\_S=617**

category=2\_p=0.98755083524573

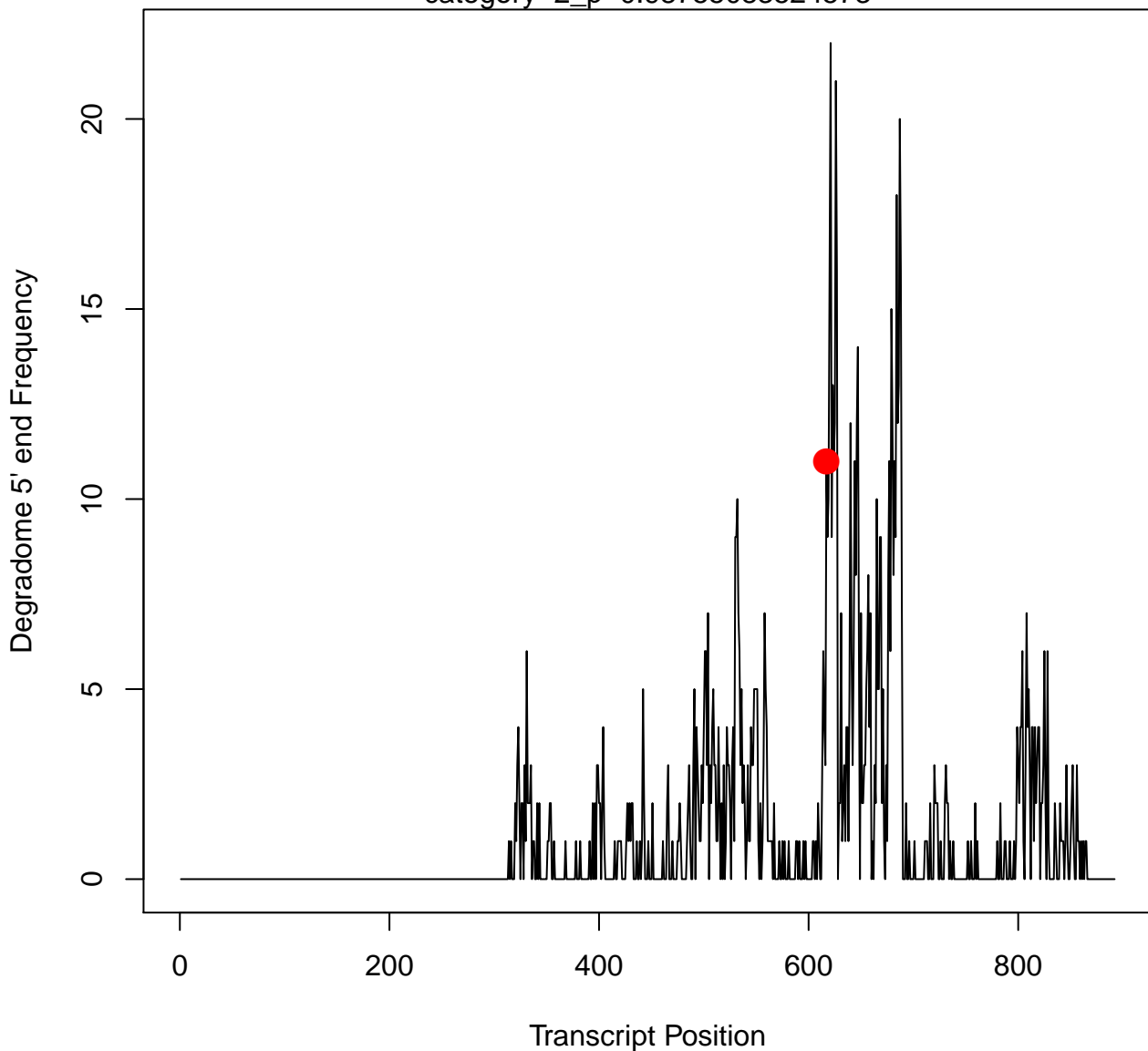

Supplement: Supplementary file 2 [file Data_Sheet_8.ZIP › GSM2230747.plot/Lsa-miR166h_Lsat_1_v5_gn_2_103140.1_617_TPlot.pdf]

**T=Lsat\_1\_v5\_gn\_3\_115940.1\_Q=Lsa-miR166h\_S=1058**

category=2\_p=0.224515100030538

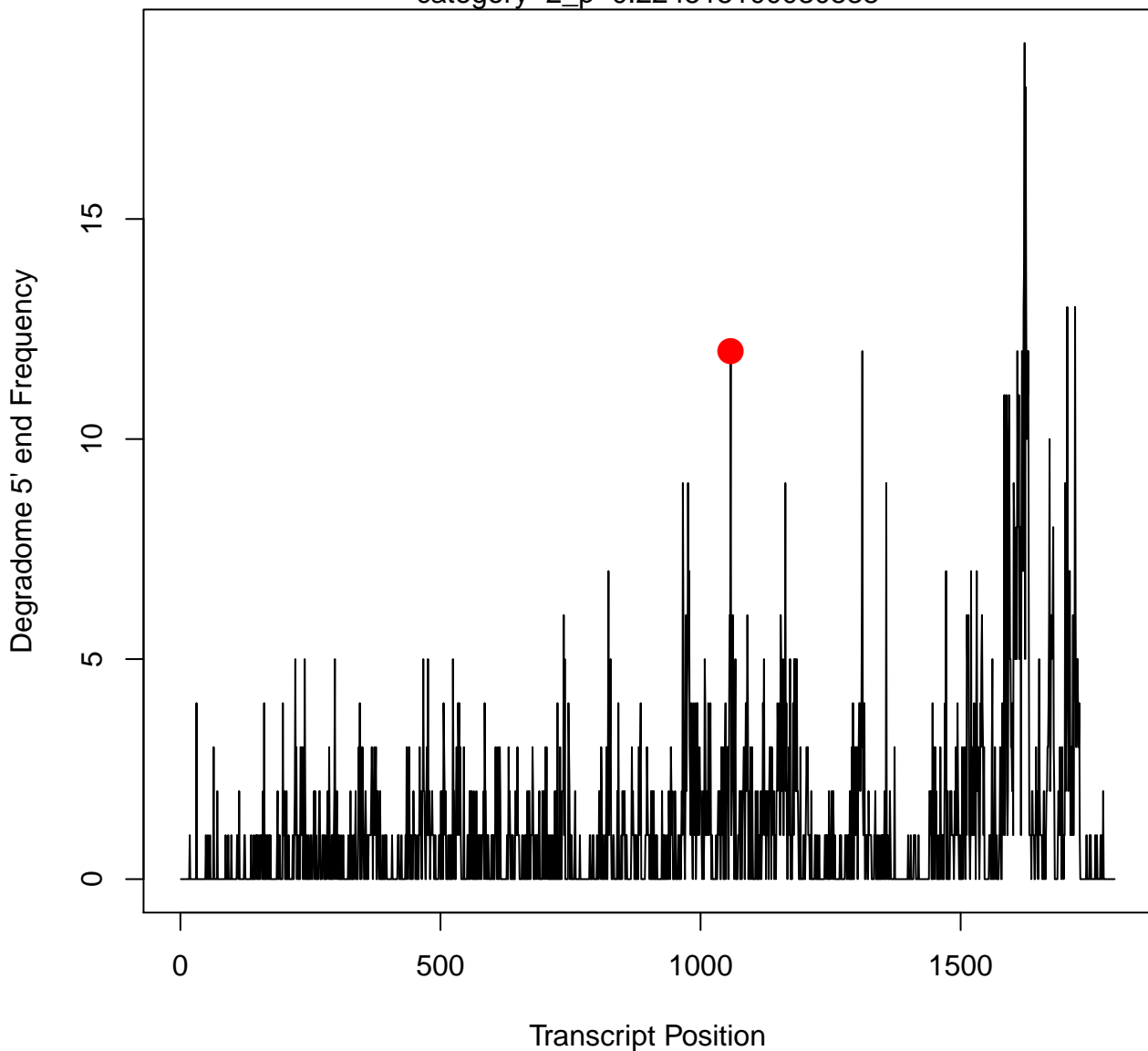

Supplement: Supplementary file 2 [file Data_Sheet_8.ZIP › GSM2230747.plot/Lsa-miR166h_Lsat_1_v5_gn_3_115940.1_1058_TPlot.pdf]

**T=Lsat\_1\_v5\_gn\_4\_39300.1\_Q=Lsa-miR166h\_S=873**

category=2\_p=0.317095430591475

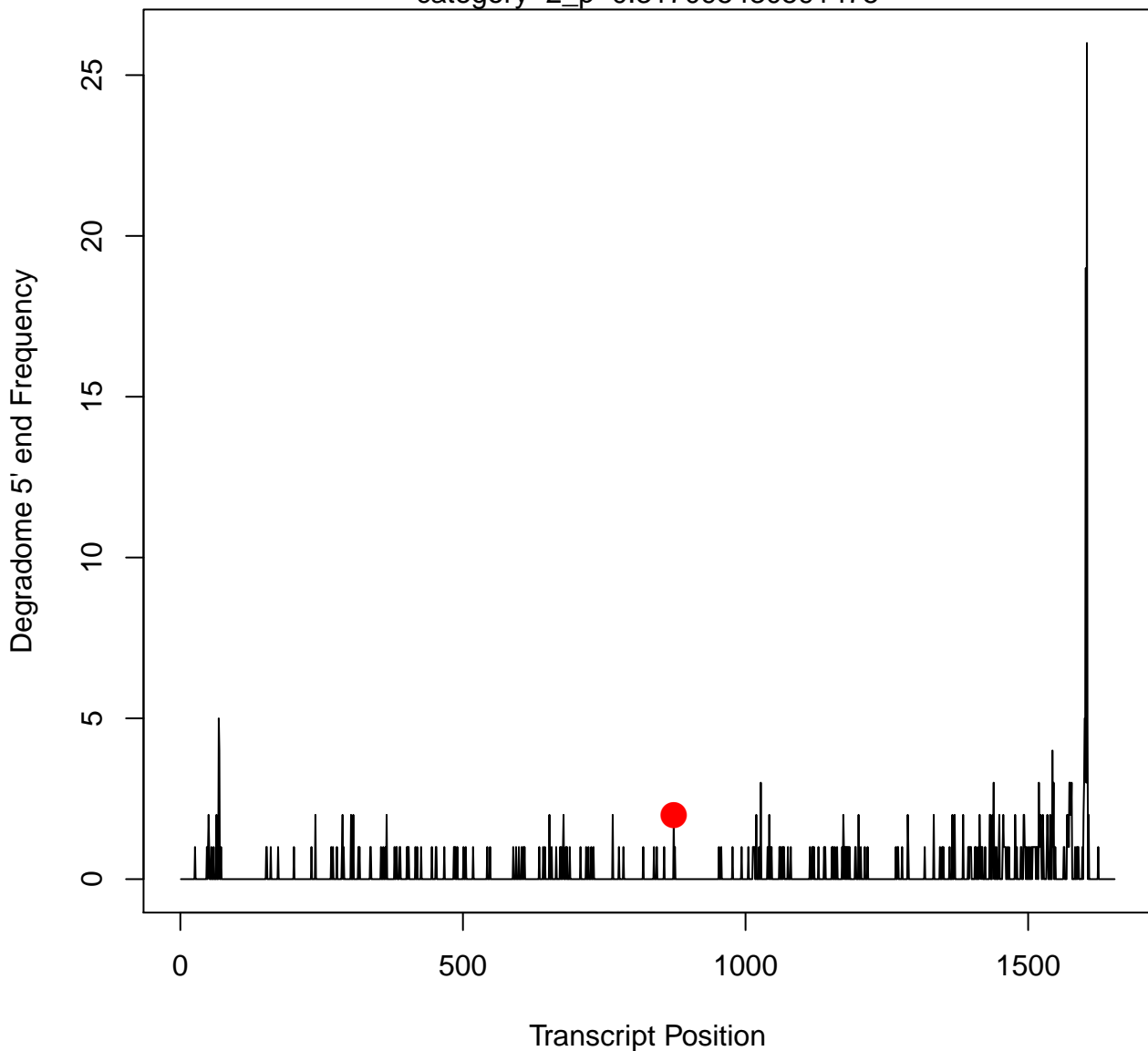

Supplement: Supplementary file 2 [file Data_Sheet_8.ZIP › GSM2230747.plot/Lsa-miR166h_Lsat_1_v5_gn_4_39300.1_873_TPlot.pdf]

**T=Lsat\_1\_v5\_gn\_5\_184280.1\_Q=Lsa-miR166h\_S=912**

category=2\_p=0.248775031802797

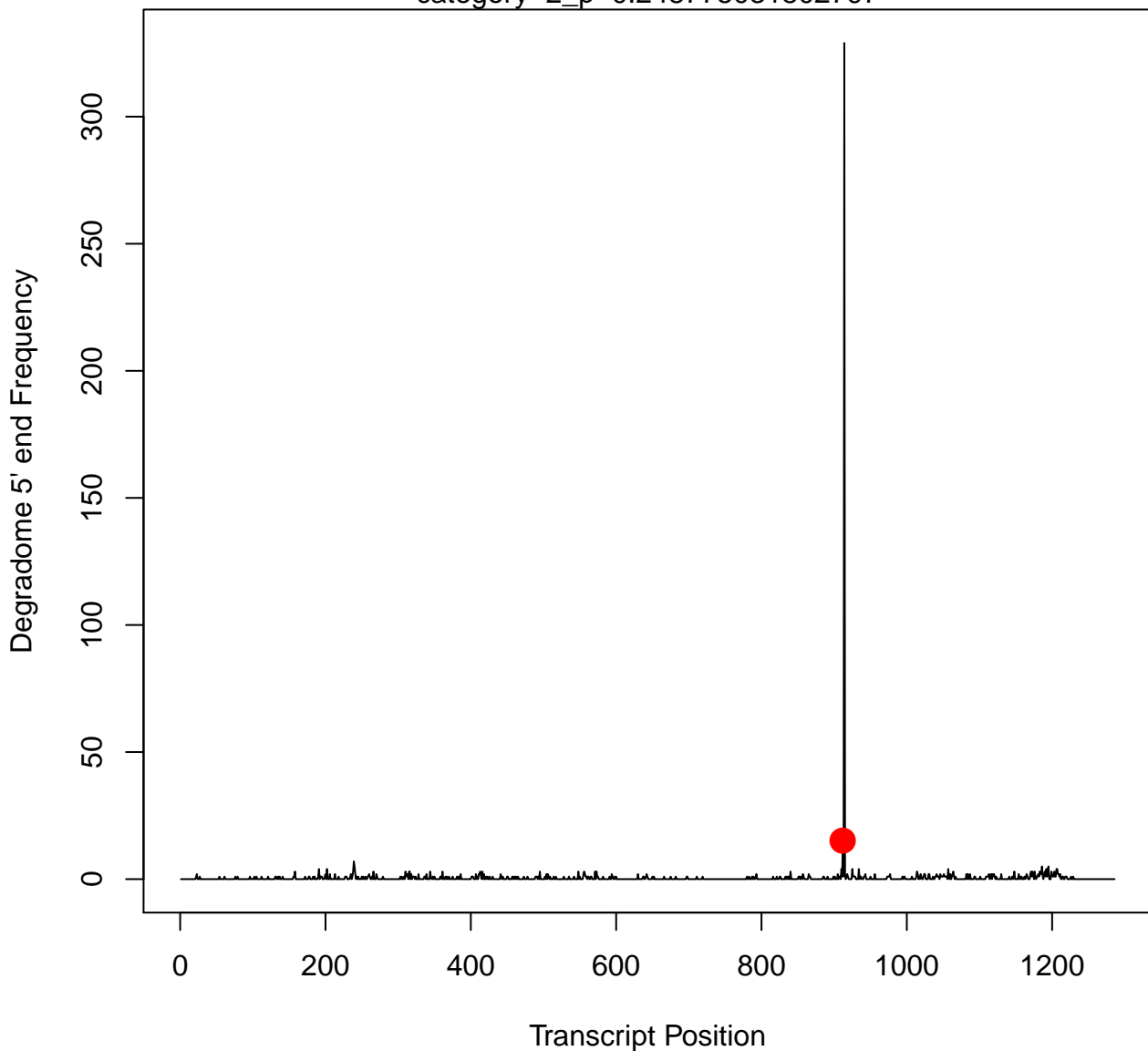

Supplement: Supplementary file 2 [file Data_Sheet_8.ZIP › GSM2230747.plot/Lsa-miR166h_Lsat_1_v5_gn_5_184280.1_912_TPlot.pdf]

**T=Lsat\_1\_v5\_gn\_5\_44541.1\_Q=Lsa-miR166h\_S=515**

category=2\_p=0.94796564871546

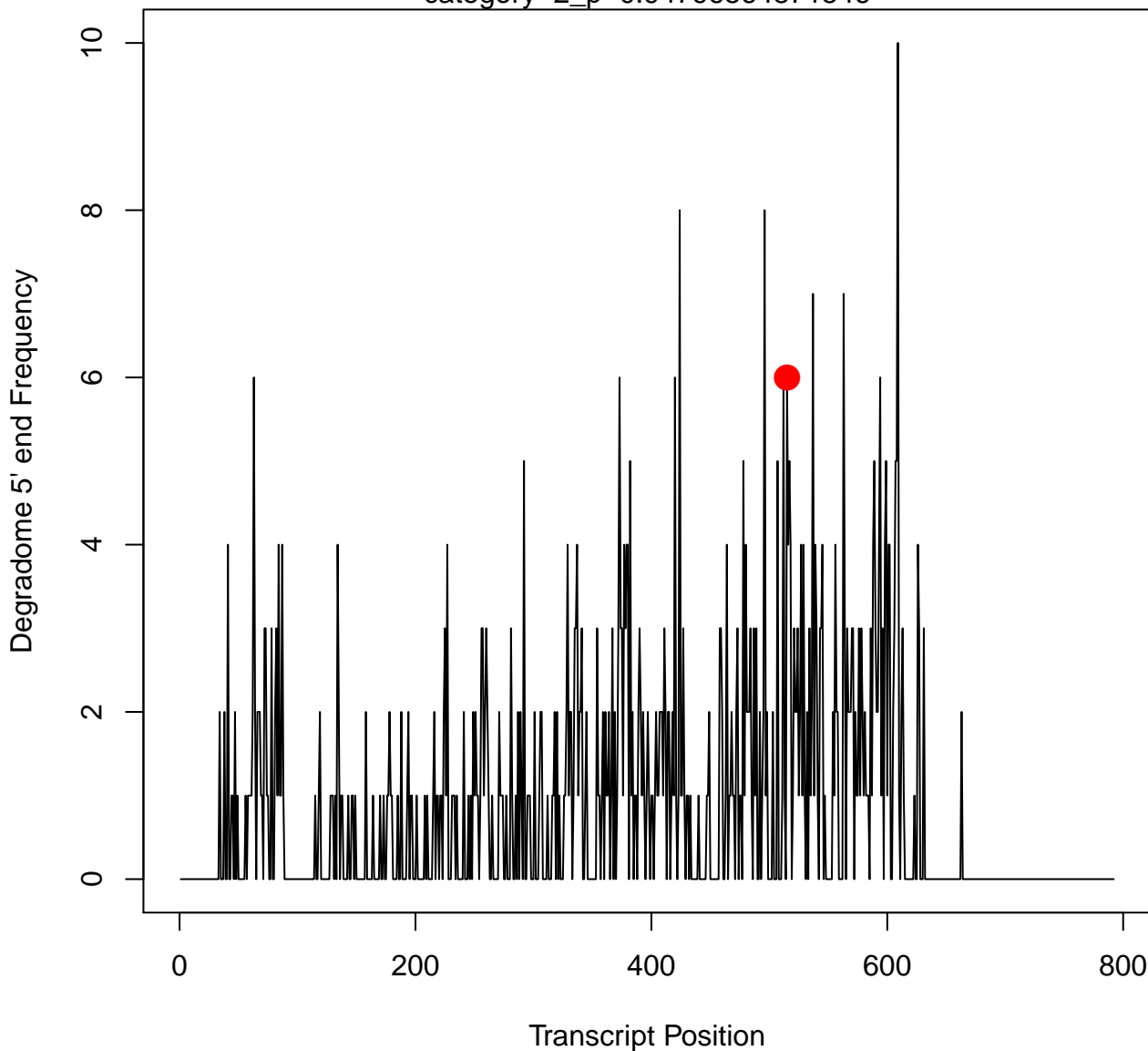

Supplement: Supplementary file 2 [file Data_Sheet_8.ZIP › GSM2230747.plot/Lsa-miR166h_Lsat_1_v5_gn_5_44541.1_515_TPlot.pdf]

**T=Lsat\_1\_v5\_gn\_5\_84680.1\_Q=Lsa-miR166h\_S=2194**

category=2\_p=0.981181563945981

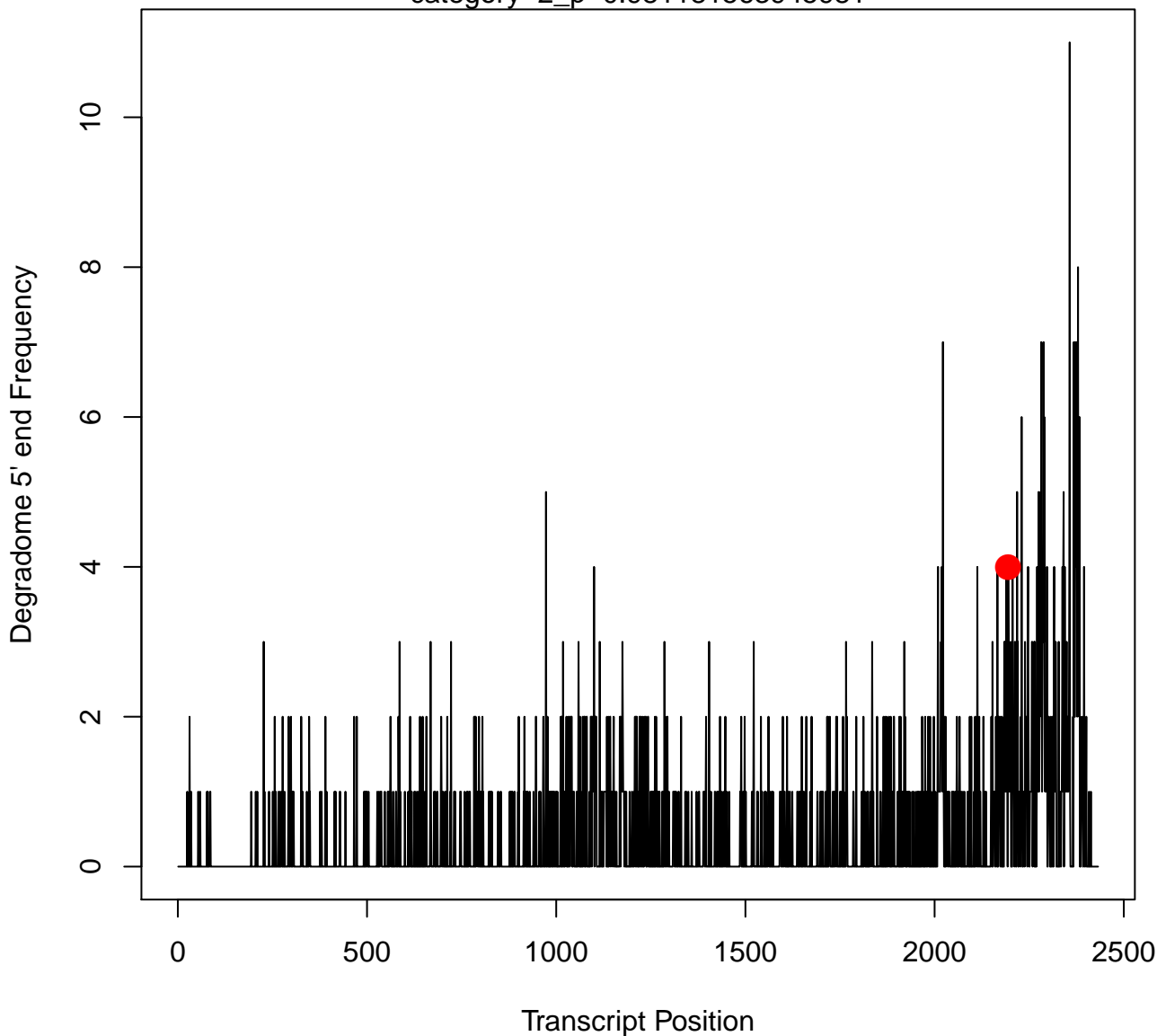

Supplement: Supplementary file 2 [file Data_Sheet_8.ZIP › GSM2230747.plot/Lsa-miR166h_Lsat_1_v5_gn_5_84680.1_2194_TPlot.pdf]

**T=Lsat\_1\_v5\_gn\_6\_22720.1\_Q=Lsa-miR166h\_S=565**

category=0\_p=0.00147478967057835

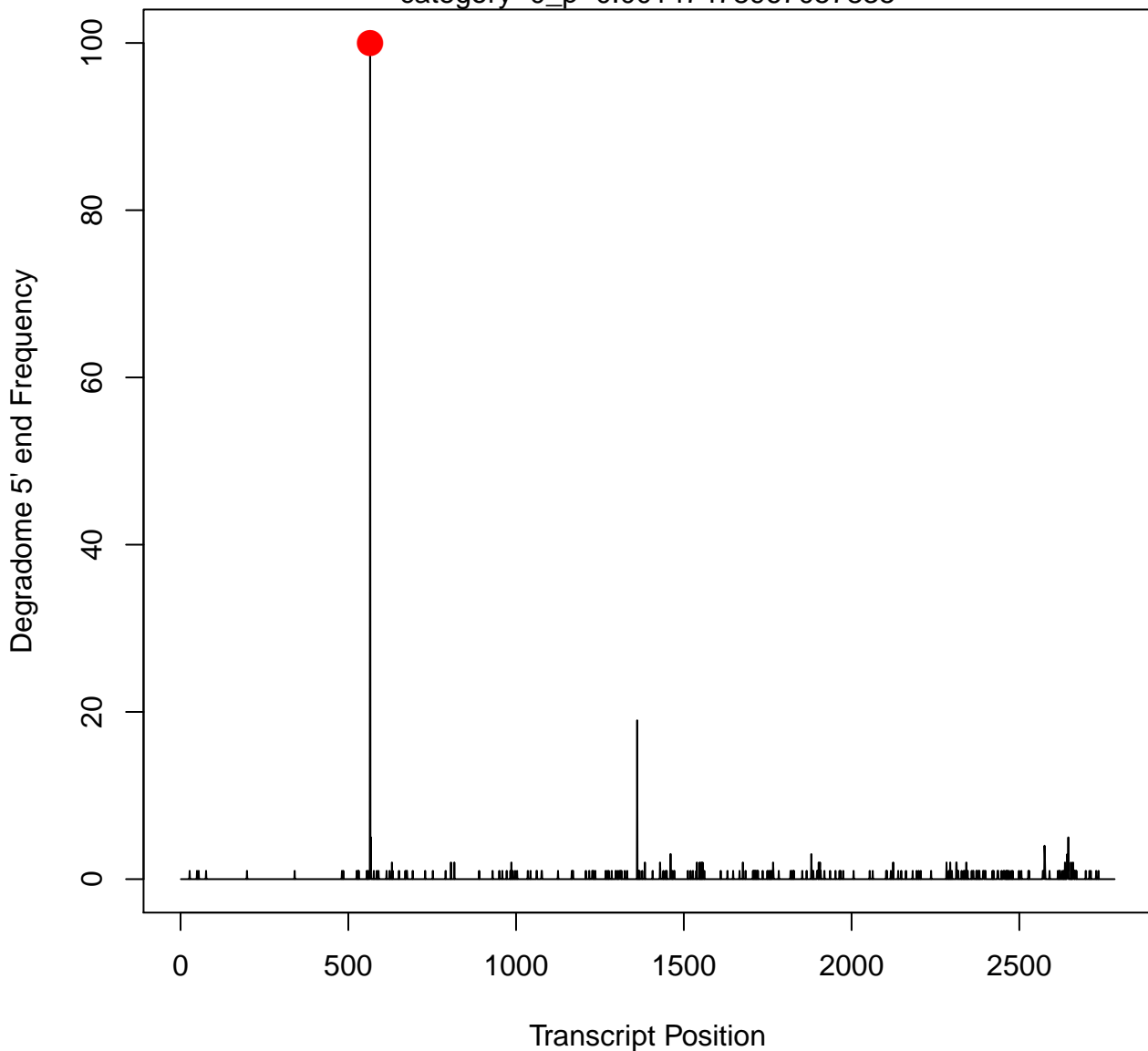

Supplement: Supplementary file 2 [file Data_Sheet_8.ZIP › GSM2230747.plot/Lsa-miR166h_Lsat_1_v5_gn_6_22720.1_565_TPlot.pdf]

**T=Lsat\_1\_v5\_gn\_8\_60221.1\_Q=Lsa-miR166h\_S=2203**

category=2\_p=0.967697200048155

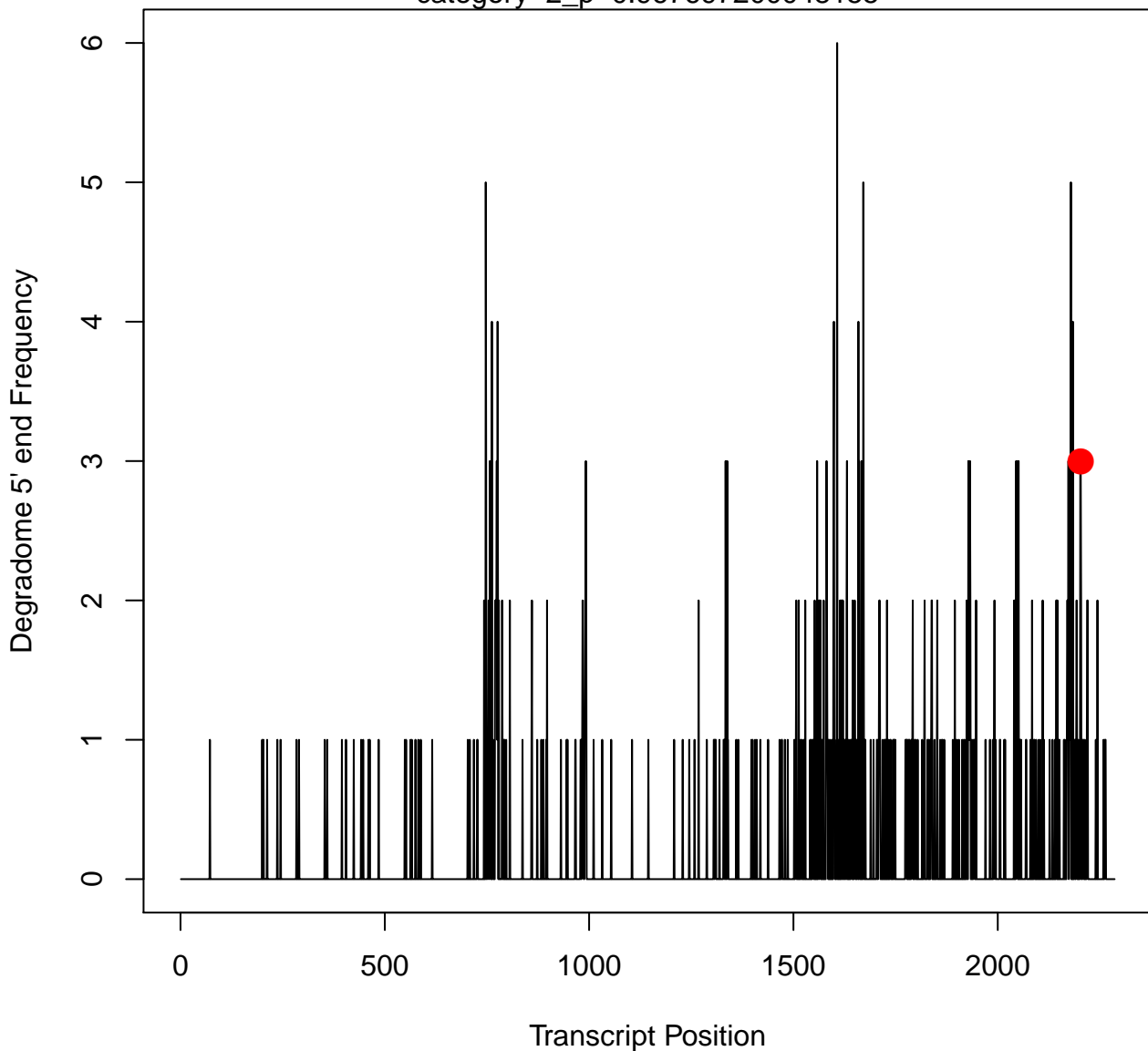

Supplement: Supplementary file 2 [file Data_Sheet_8.ZIP › GSM2230747.plot/Lsa-miR166h_Lsat_1_v5_gn_8_60221.1_2203_TPlot.pdf]

**T=Lsat\_1\_v5\_gn\_9\_89140.1\_Q=Lsa-miR166h\_S=1993**

category=2\_p=0.97155363090542

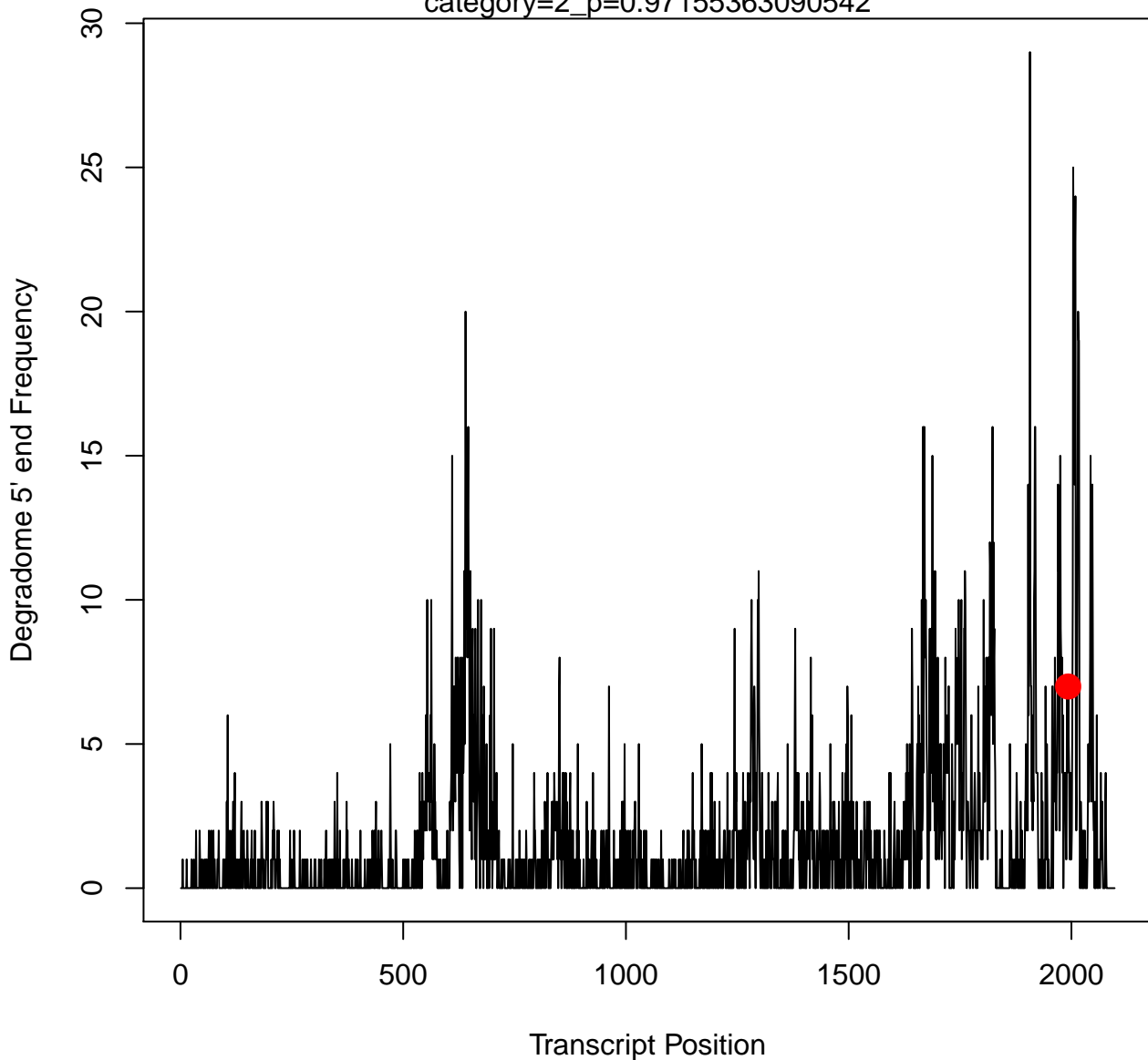

Supplement: Supplementary file 2 [file Data_Sheet_8.ZIP › GSM2230747.plot/Lsa-miR166h_Lsat_1_v5_gn_9_89140.1_1993_TPlot.pdf]

**T=Lsat\_1\_v5\_gn\_1\_37081.1\_Q=Lsa-miR166i\_S=1032**

category=2\_p=0.0312835643520768

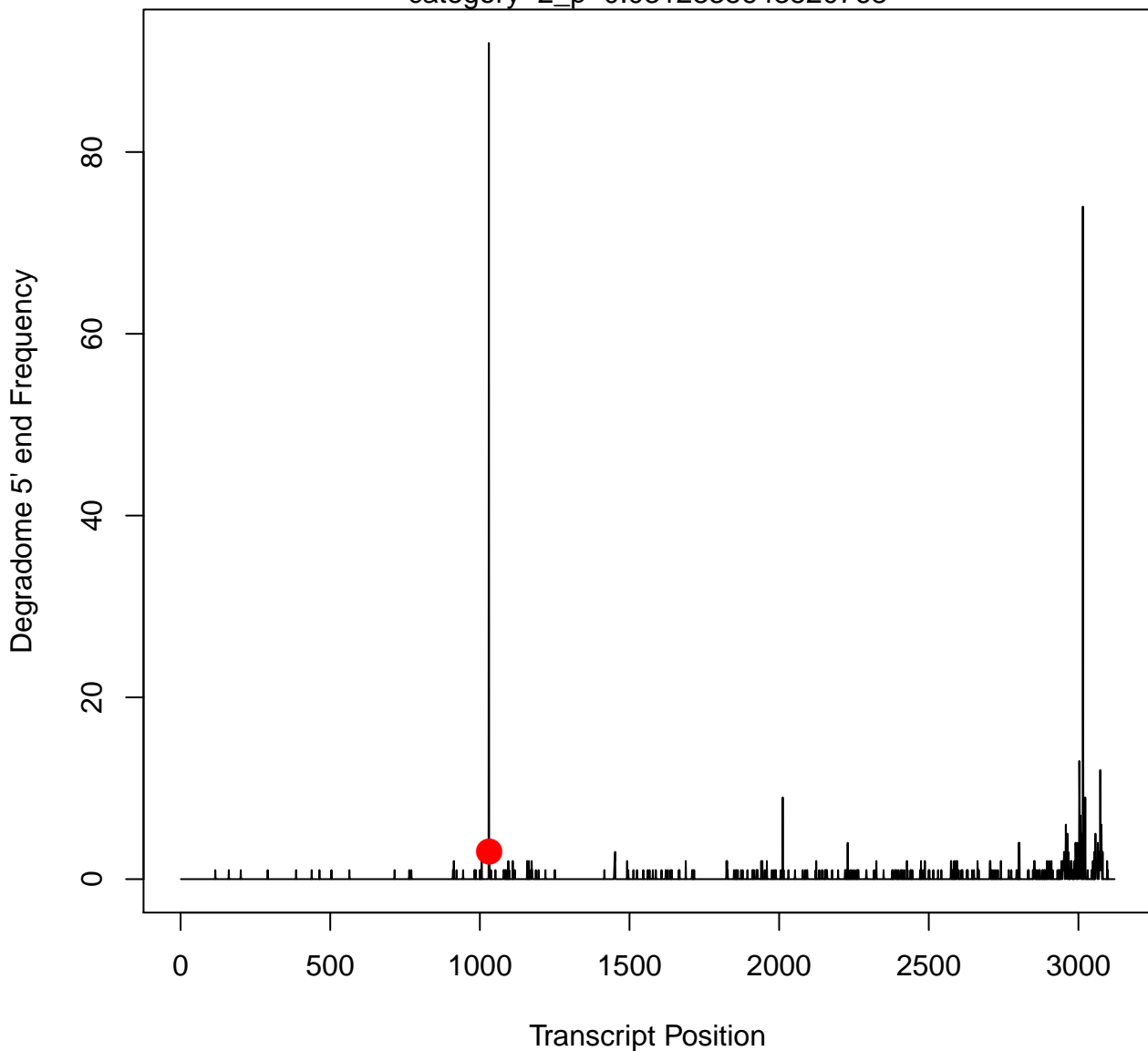

Supplement: Supplementary file 2 [file Data_Sheet_8.ZIP › GSM2230747.plot/Lsa-miR166i_Lsat_1_v5_gn_1_37081.1_1032_TPlot.pdf]

**T=Lsat\_1\_v5\_gn\_1\_75641.1\_Q=Lsa-miR166i\_S=1068**

category=2\_p=0.921343710073031

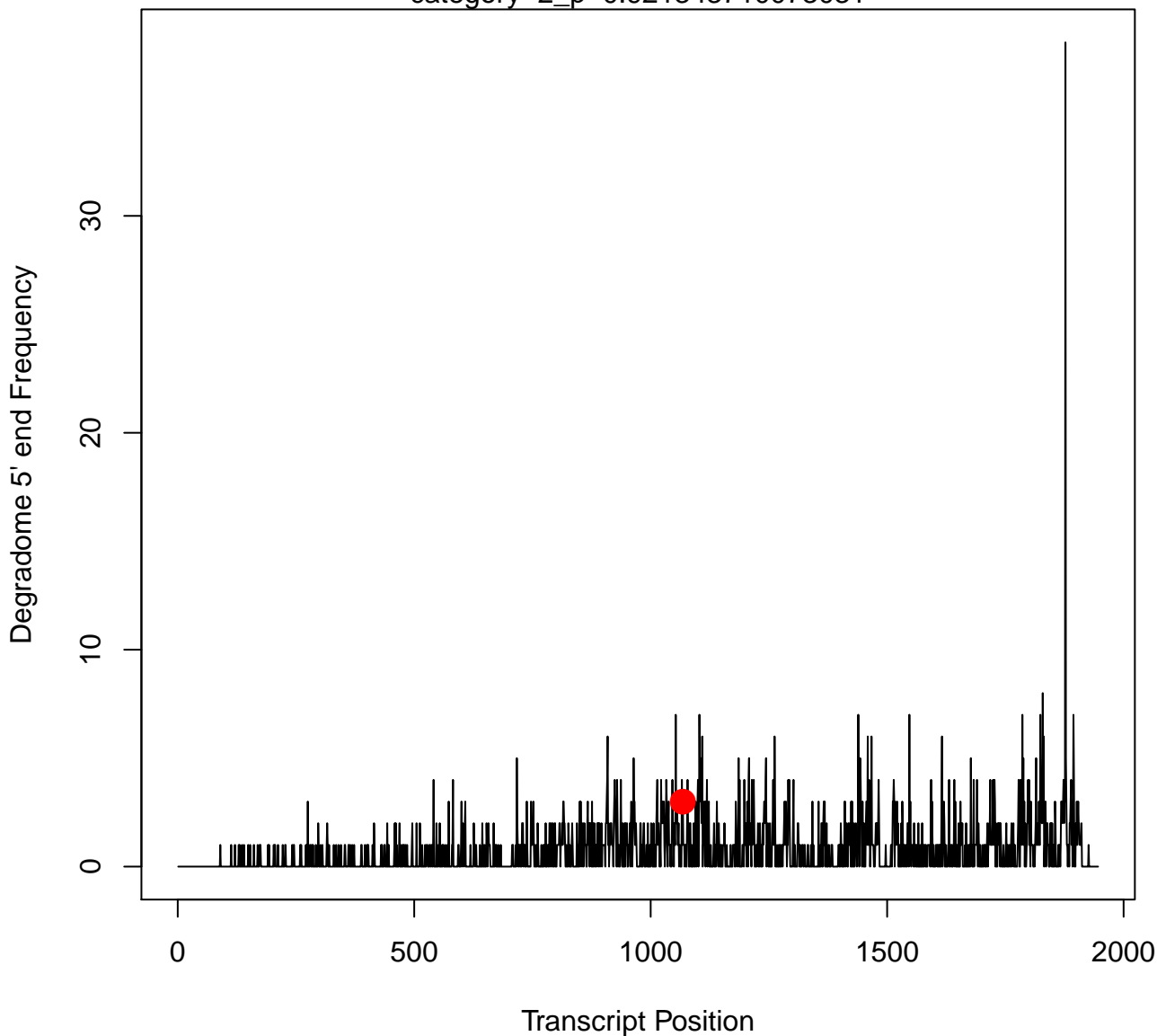

Supplement: Supplementary file 2 [file Data_Sheet_8.ZIP › GSM2230747.plot/Lsa-miR166i_Lsat_1_v5_gn_1_75641.1_1068_TPlot.pdf]

**T=Lsat\_1\_v5\_gn\_2\_100140.1\_Q=Lsa-miR166i\_S=1402**

category=2\_p=0.199471722134268

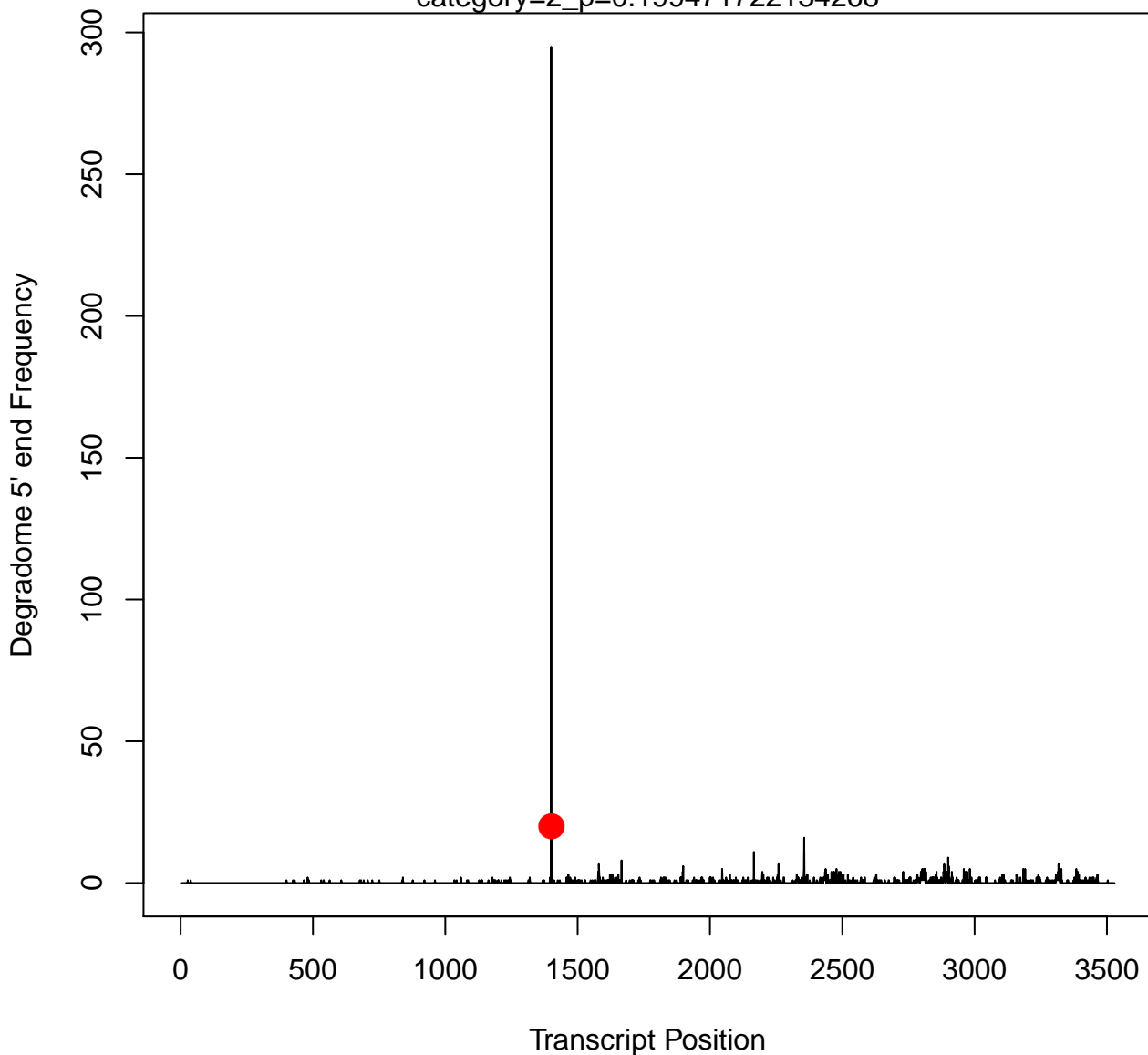

Supplement: Supplementary file 2 [file Data_Sheet_8.ZIP › GSM2230747.plot/Lsa-miR166i_Lsat_1_v5_gn_2_100140.1_1402_TPlot.pdf]

**T=Lsat\_1\_v5\_gn\_3\_10861.1\_Q=Lsa-miR166i\_S=516**

category=2\_p=0.952697929697815

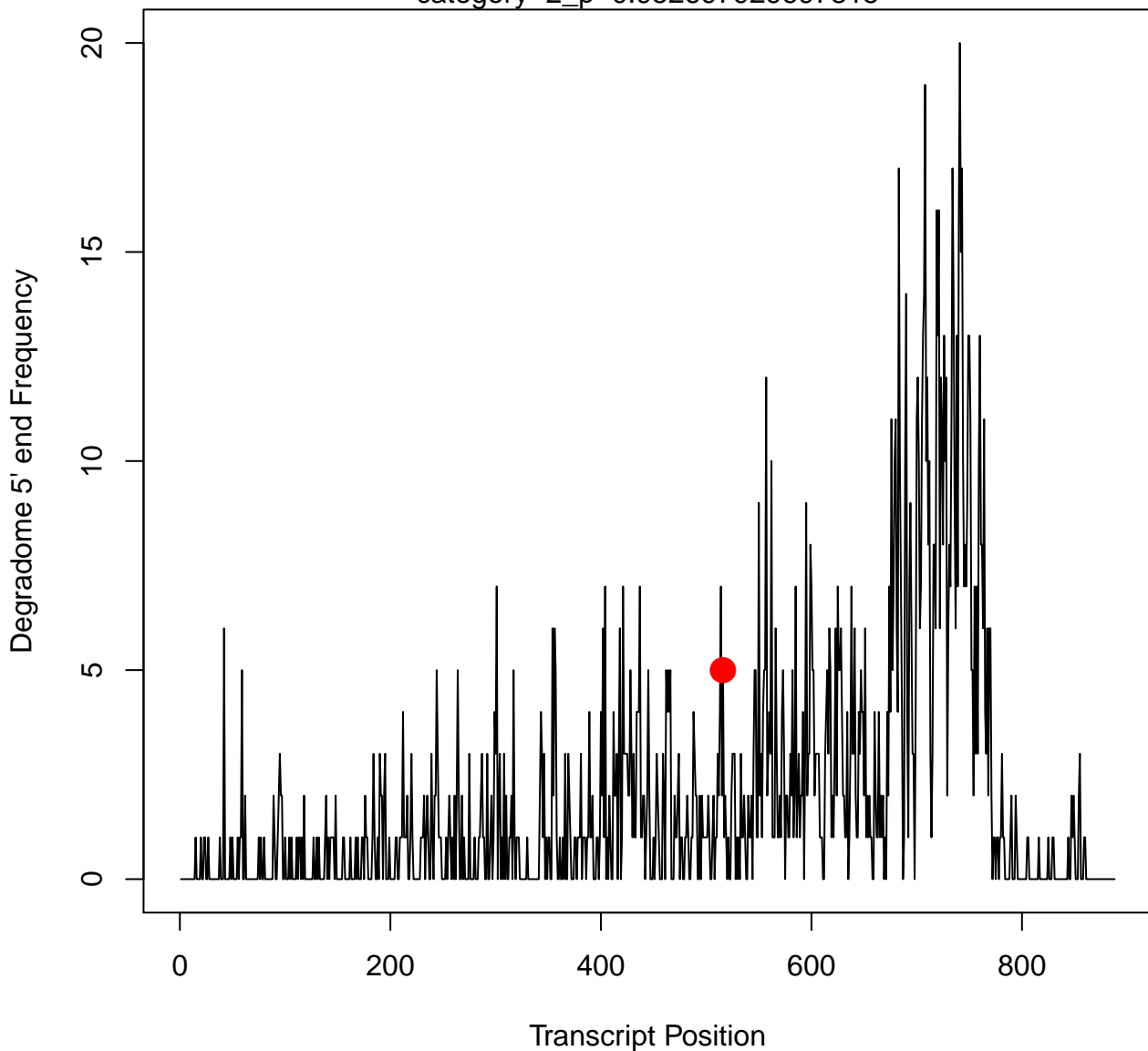

Supplement: Supplementary file 2 [file Data_Sheet_8.ZIP › GSM2230747.plot/Lsa-miR166i_Lsat_1_v5_gn_3_10861.1_516_TPlot.pdf]

**T=Lsat\_1\_v5\_gn\_4\_100080.1\_Q=Lsa-miR166i\_S=1069**

category=2\_p=0.0615884673055831

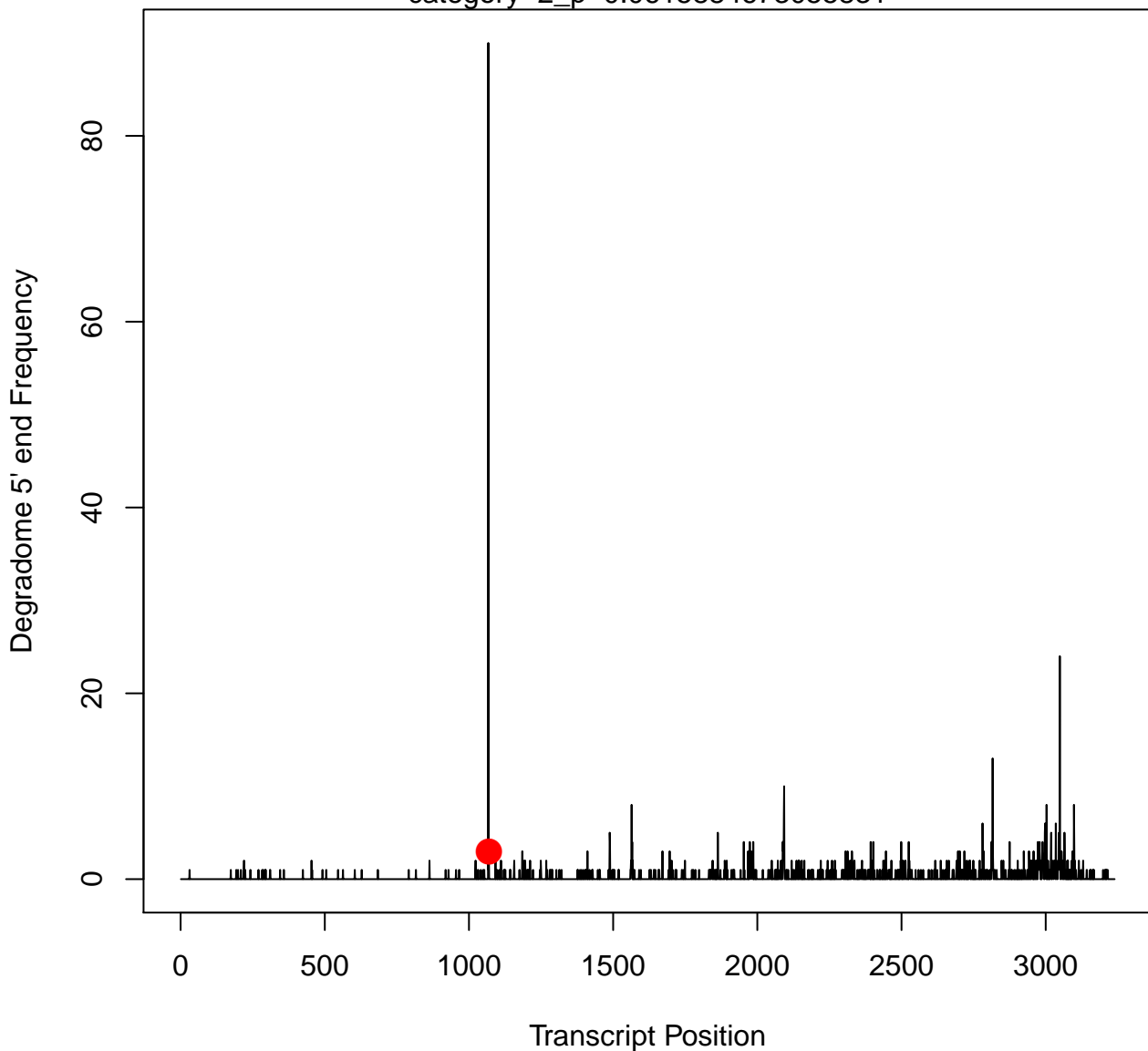

Supplement: Supplementary file 2 [file Data_Sheet_8.ZIP › GSM2230747.plot/Lsa-miR166i_Lsat_1_v5_gn_4_100080.1_1069_TPlot.pdf]

**T=Lsat\_1\_v5\_gn\_5\_120280.1\_Q=Lsa-miR166i\_S=327**

category=2\_p=0.589317014801513

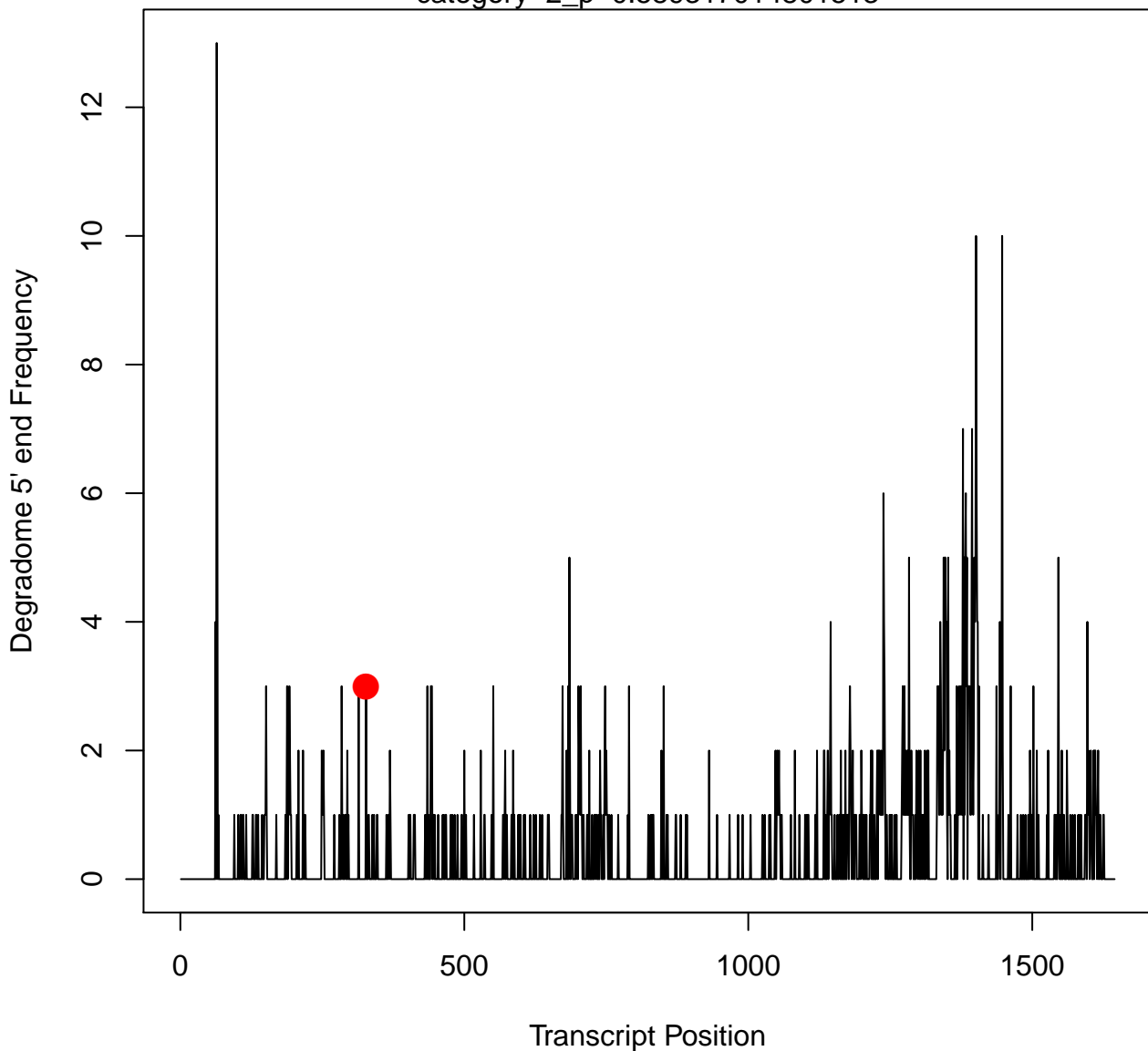

Supplement: Supplementary file 2 [file Data_Sheet_8.ZIP › GSM2230747.plot/Lsa-miR166i_Lsat_1_v5_gn_5_120280.1_327_TPlot.pdf]

**T=Lsat\_1\_v5\_gn\_5\_134441.1\_Q=Lsa-miR166i\_S=570**

category=2\_p=0.0909453248773598

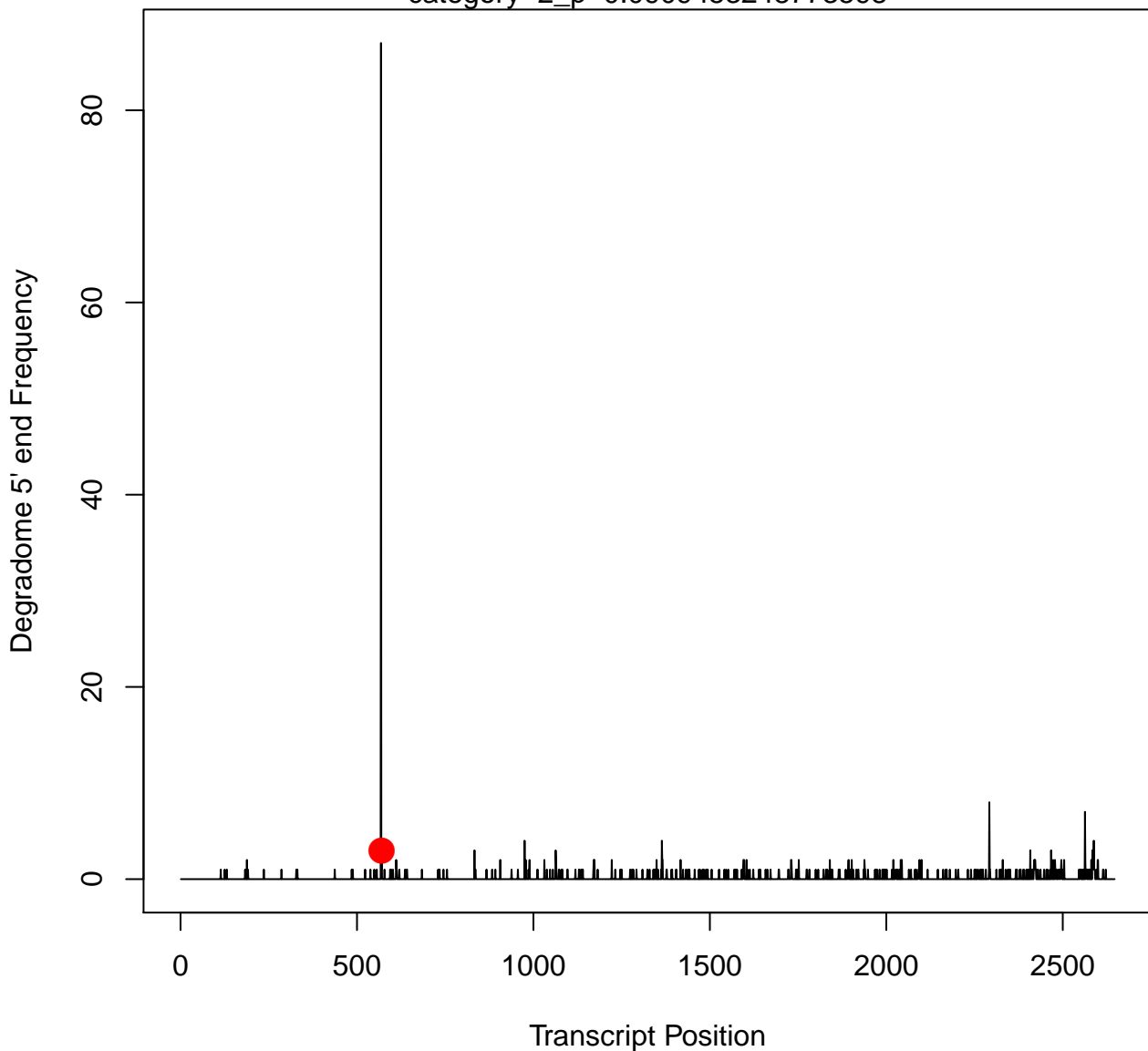

Supplement: Supplementary file 2 [file Data_Sheet_8.ZIP › GSM2230747.plot/Lsa-miR166i_Lsat_1_v5_gn_5_134441.1_570_TPlot.pdf]

**T=Lsat\_1\_v5\_gn\_5\_184280.1\_Q=Lsa-miR166i\_S=914**

category=0\_p=0.00294740433658425

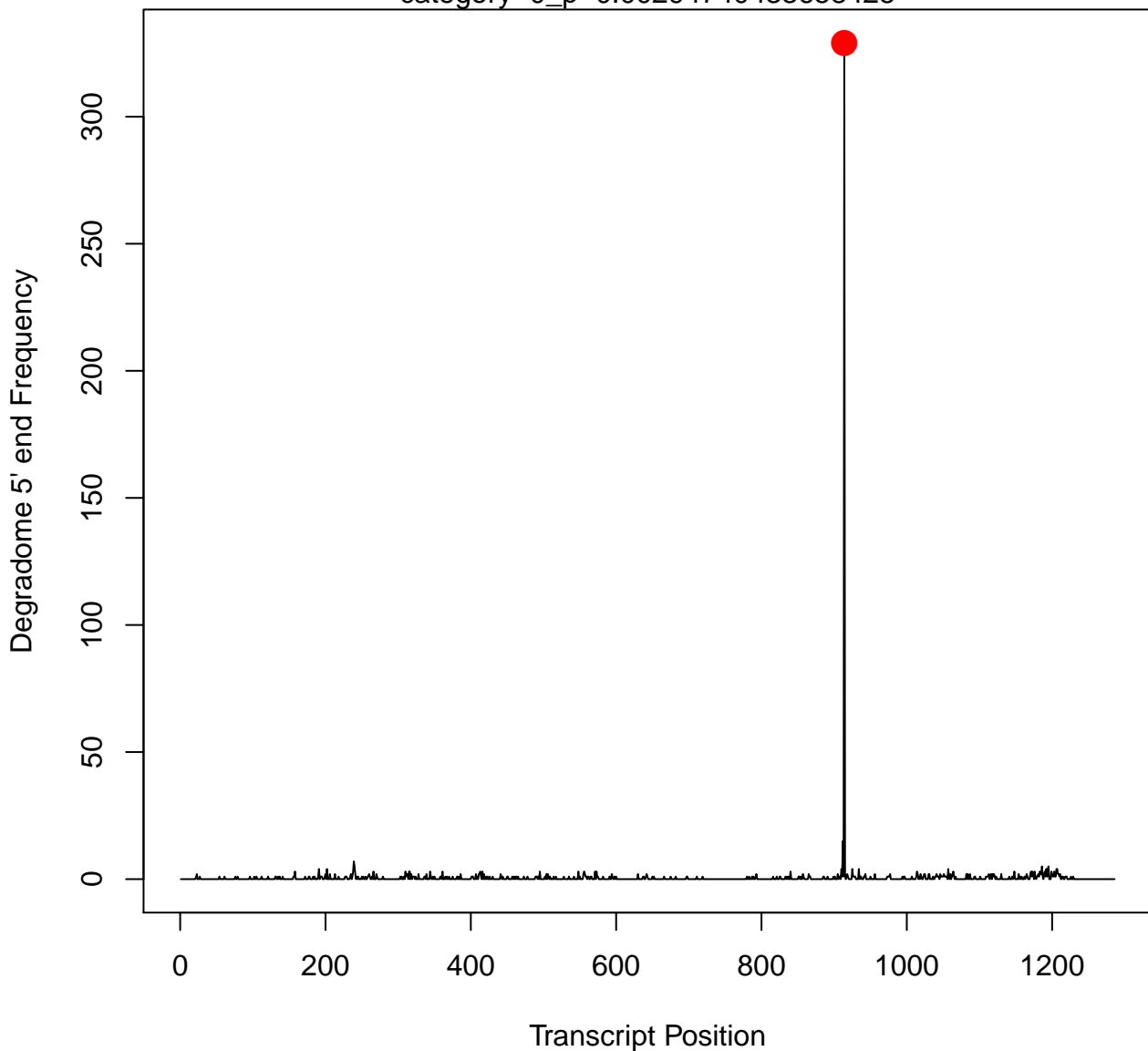

Supplement: Supplementary file 2 [file Data_Sheet_8.ZIP › GSM2230747.plot/Lsa-miR166i_Lsat_1_v5_gn_5_184280.1_914_TPlot.pdf]

T=Lsat\_1\_v5\_gn\_5\_32500.1\_Q=Lsa-miR166i\_S=567

category=2\_p=0.146932609015138

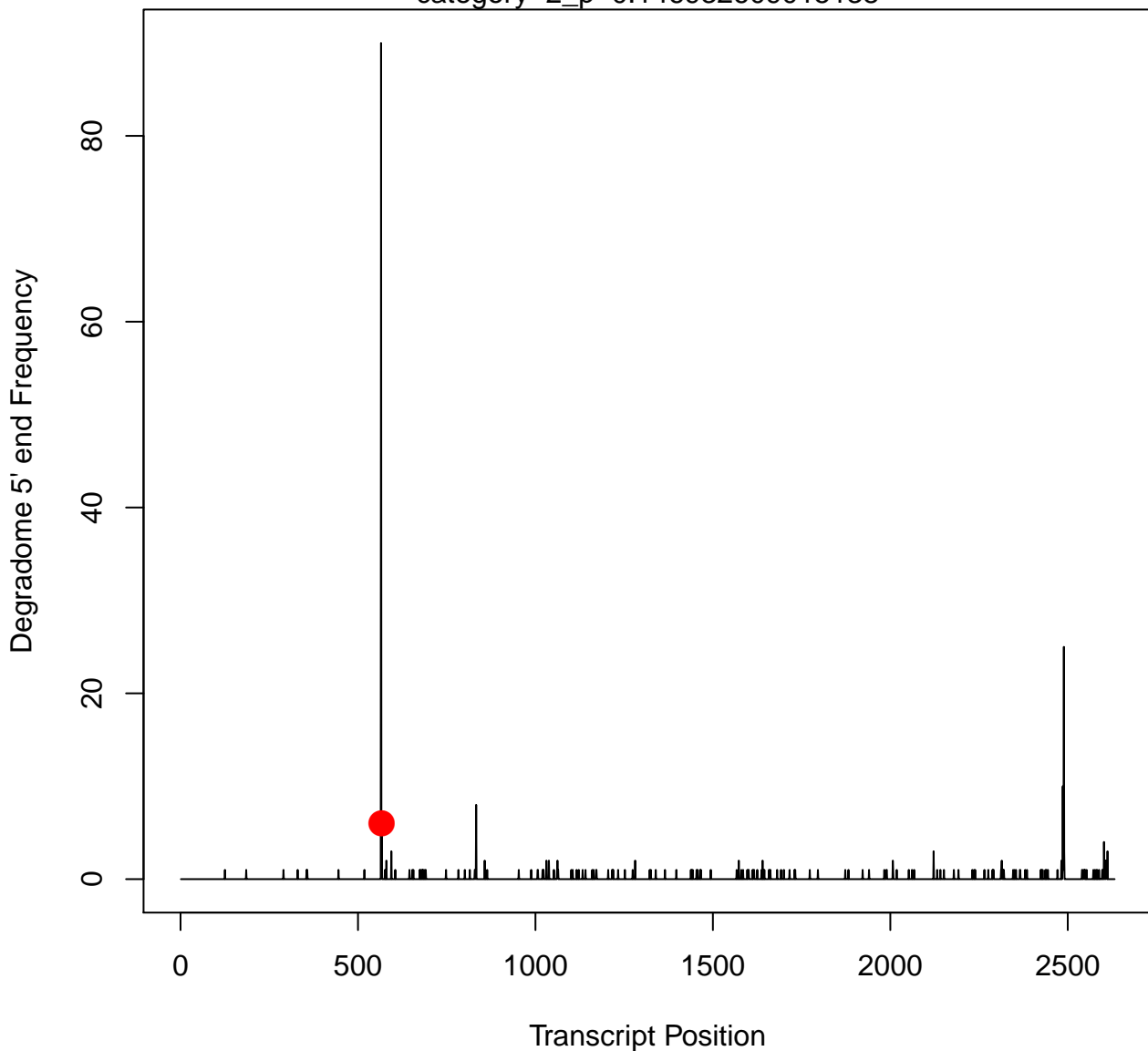

Supplement: Supplementary file 2 [file Data_Sheet_8.ZIP › GSM2230747.plot/Lsa-miR166i_Lsat_1_v5_gn_5_32500.1_567_TPlot.pdf]

**T=Lsat\_1\_v5\_gn\_5\_84680.1\_Q=Lsa-miR166i\_S=2196**

category=2\_p=0.873298010485164

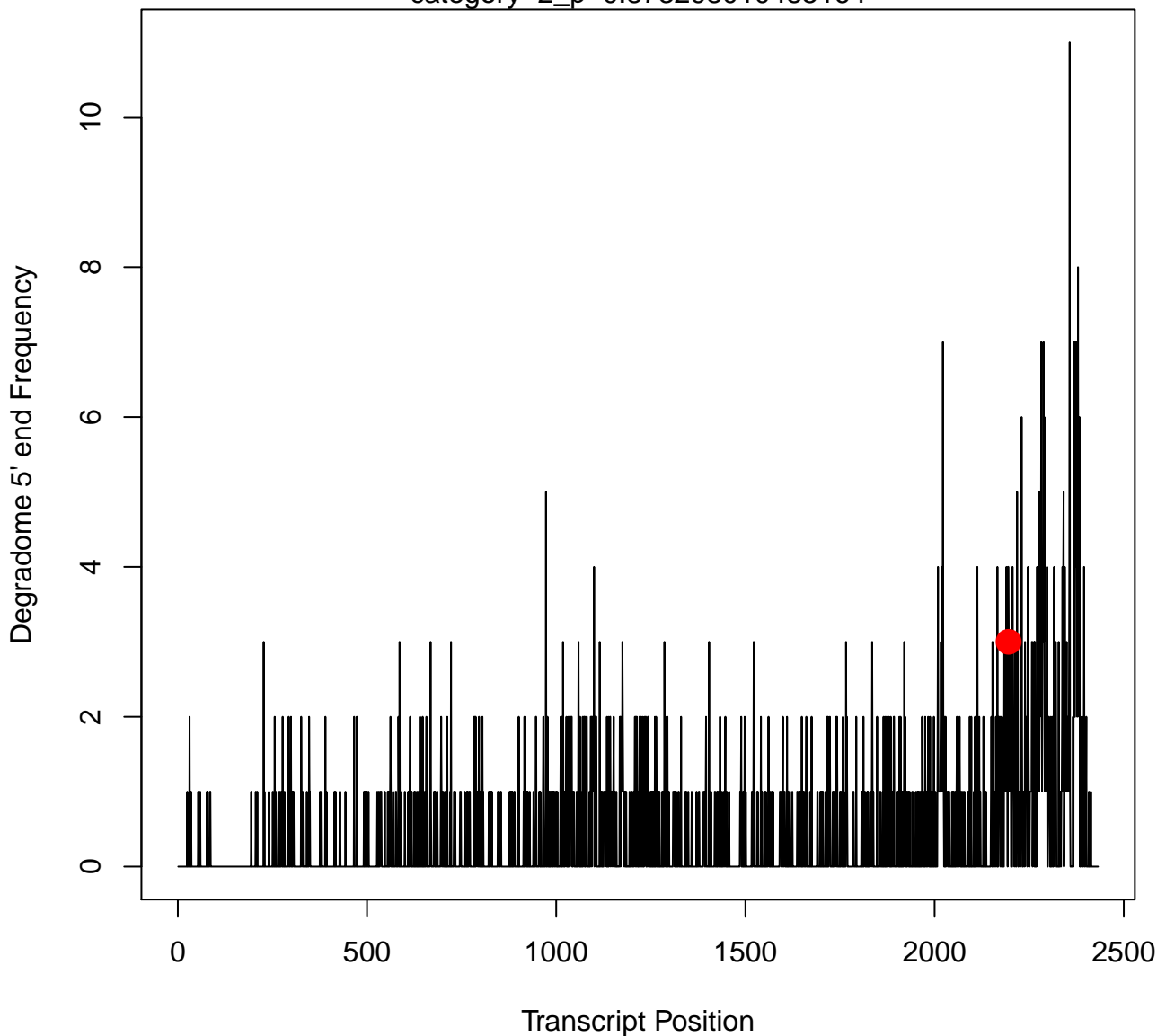

Supplement: Supplementary file 2 [file Data_Sheet_8.ZIP › GSM2230747.plot/Lsa-miR166i_Lsat_1_v5_gn_5_84680.1_2196_TPlot.pdf]

**T=Lsat\_1\_v5\_gn\_6\_22720.1\_Q=Lsa-miR166i\_S=567**

category=2\_p=0.119383795306115

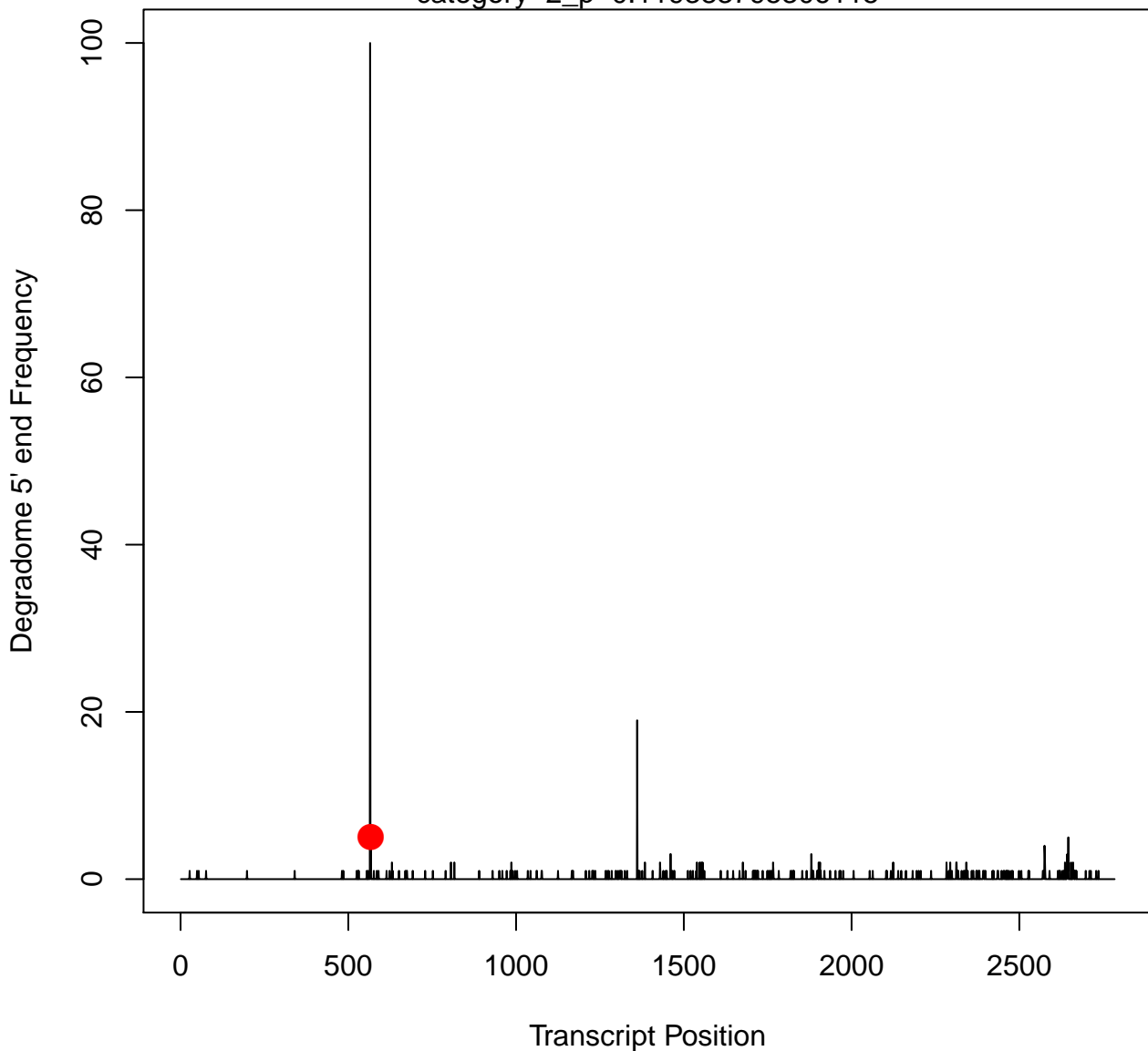

Supplement: Supplementary file 2 [file Data_Sheet_8.ZIP › GSM2230747.plot/Lsa-miR166i_Lsat_1_v5_gn_6_22720.1_567_TPlot.pdf]

**T=Lsat\_1\_v5\_gn\_6\_2981.1\_Q=Lsa-miR166i\_S=487**

category=2\_p=0.660619629448312

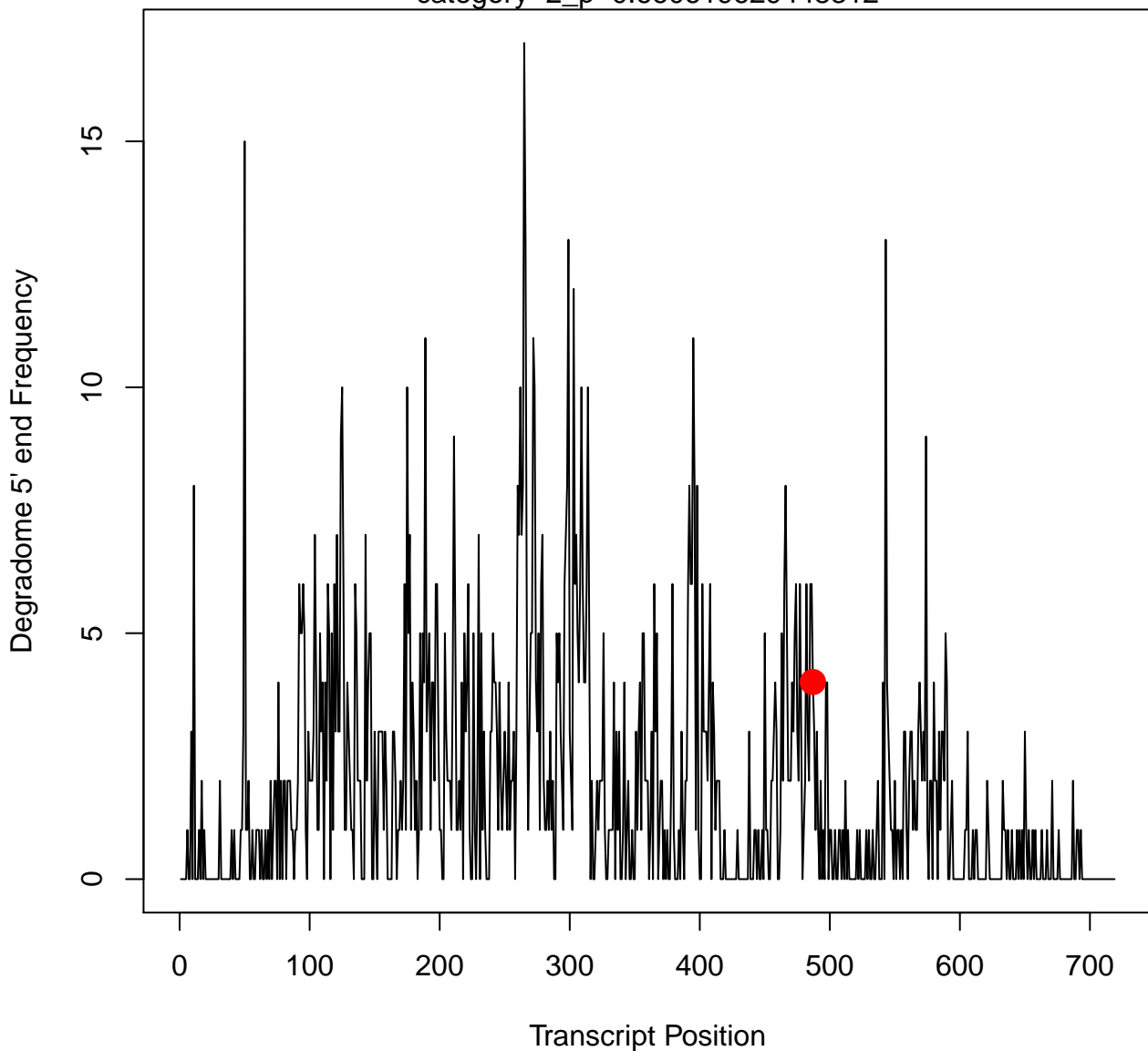

Supplement: Supplementary file 2 [file Data_Sheet_8.ZIP › GSM2230747.plot/Lsa-miR166i_Lsat_1_v5_gn_6_2981.1_487_TPlot.pdf]

**T=Lsat\_1\_v5\_gn\_6\_45641.1\_Q=Lsa-miR166i\_S=1311**

category=2\_p=0.173619597637671

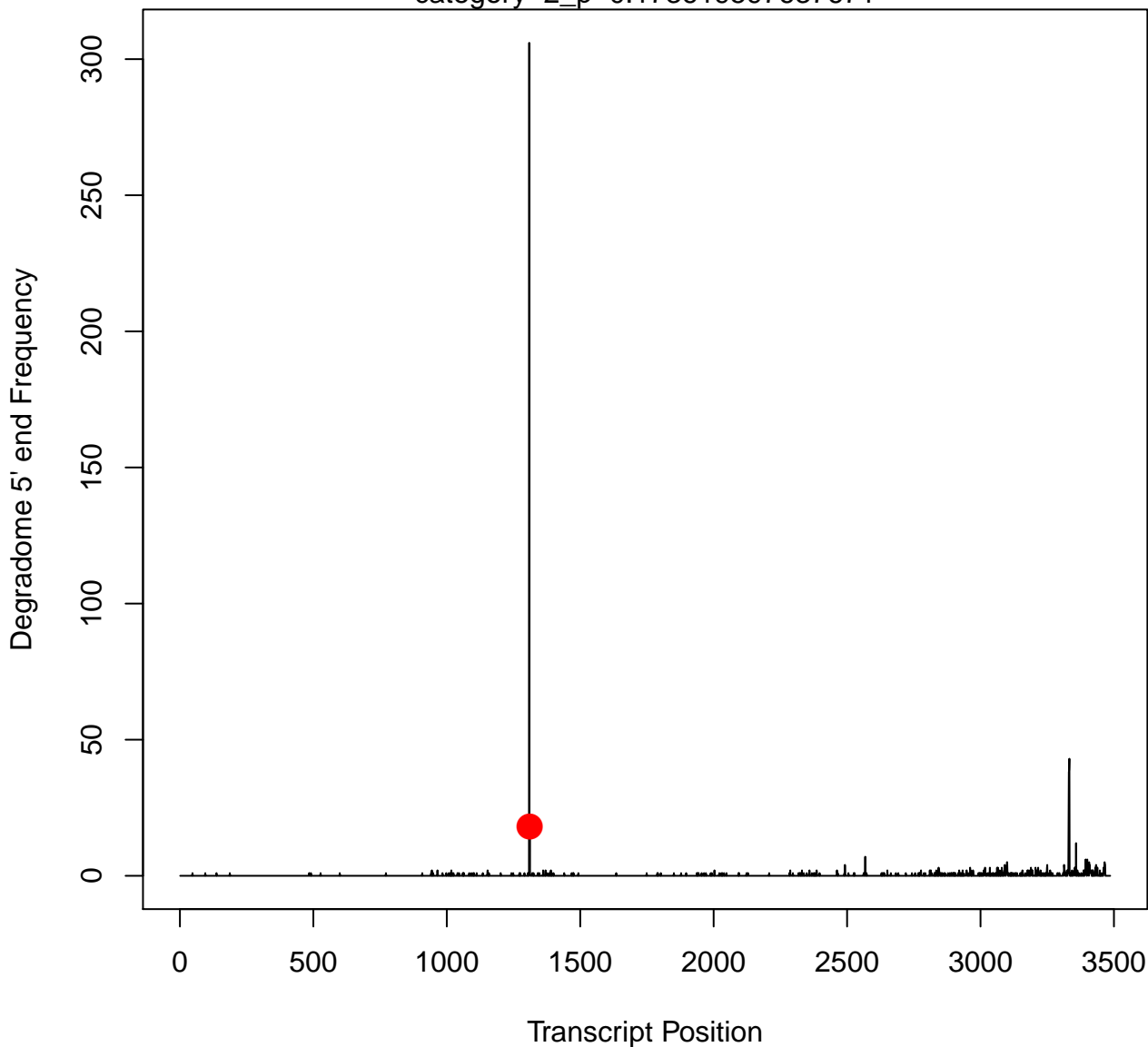

Supplement: Supplementary file 2 [file Data_Sheet_8.ZIP › GSM2230747.plot/Lsa-miR166i_Lsat_1_v5_gn_6_45641.1_1311_TPlot.pdf]

**T=Lsat\_1\_v5\_gn\_8\_99701.1\_Q=Lsa-miR166i\_S=1628**

category=2\_p=0.958345030376327

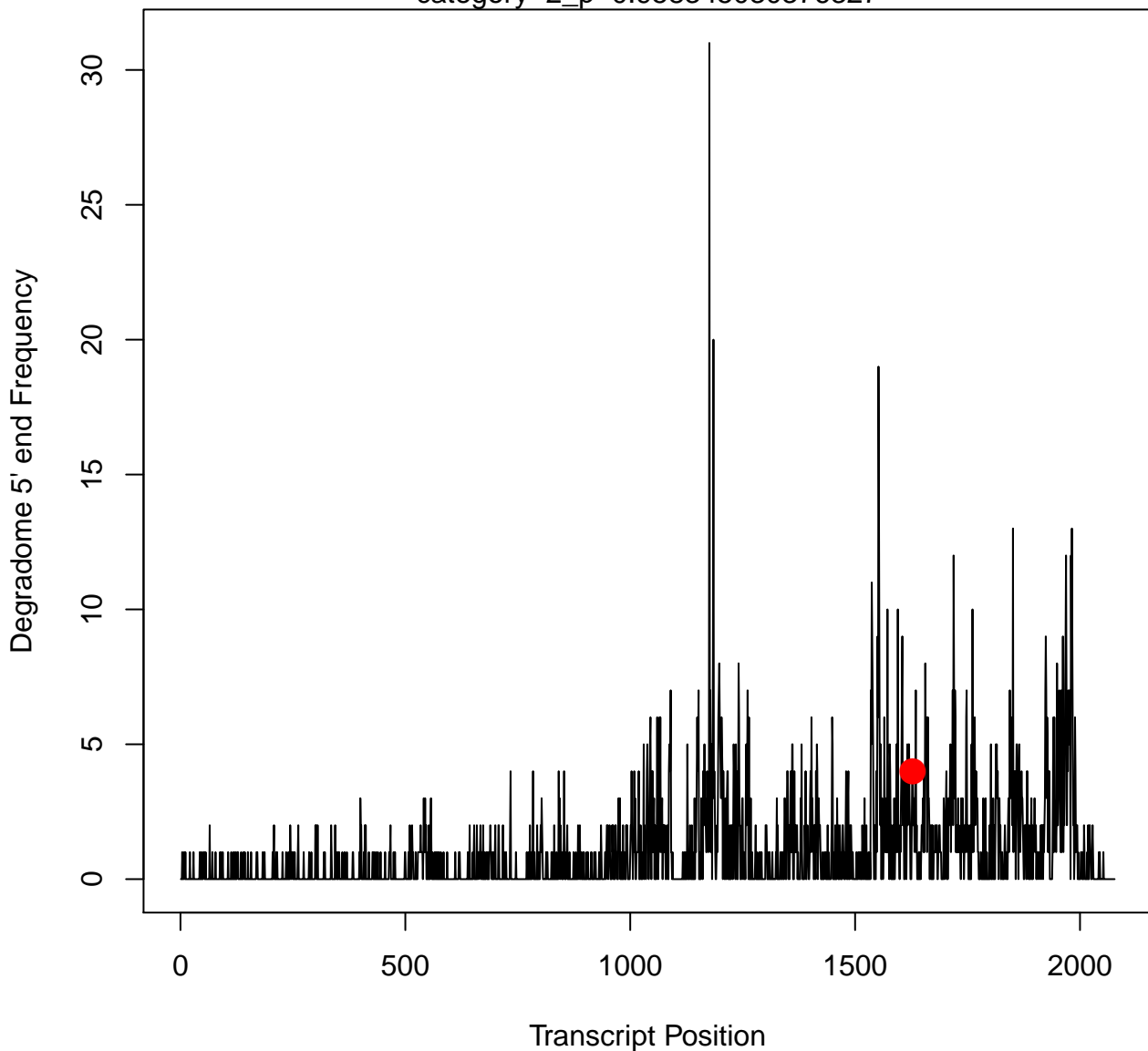

Supplement: Supplementary file 2 [file Data_Sheet_8.ZIP › GSM2230747.plot/Lsa-miR166i_Lsat_1_v5_gn_8_99701.1_1628_TPlot.pdf]

**T=Lsat\_1\_v5\_gn\_3\_72820.1\_Q=Lsa-miR167a\_S=531**

category=2\_p=0.789313483364757

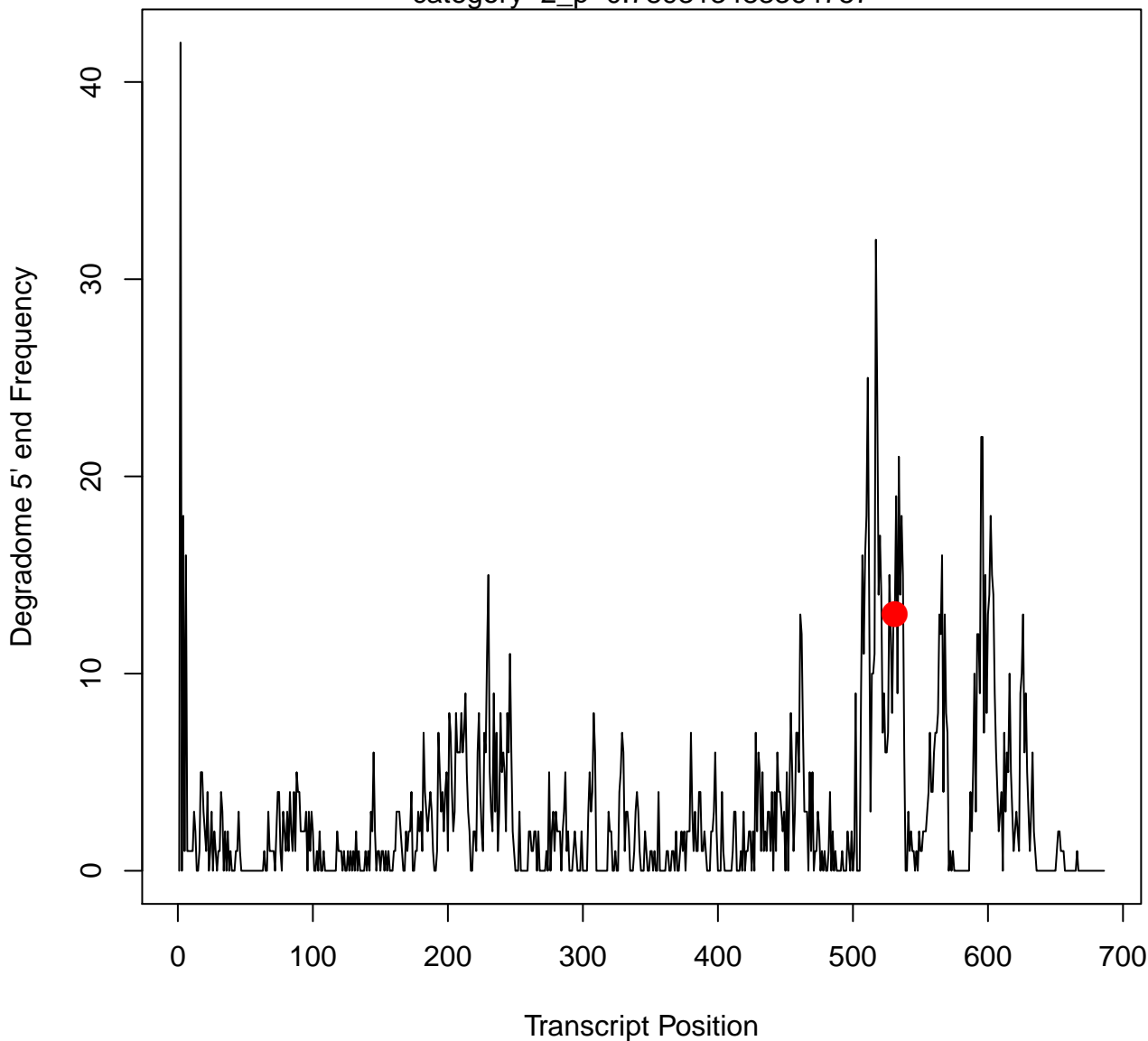

Supplement: Supplementary file 2 [file Data_Sheet_8.ZIP › GSM2230747.plot/Lsa-miR167a_Lsat_1_v5_gn_3_72820.1_531_TPlot.pdf]

**T=Lsat\_1\_v5\_gn\_4\_9441.1\_Q=Lsa-miR167a\_S=468**

category=2\_p=0.417436380683029

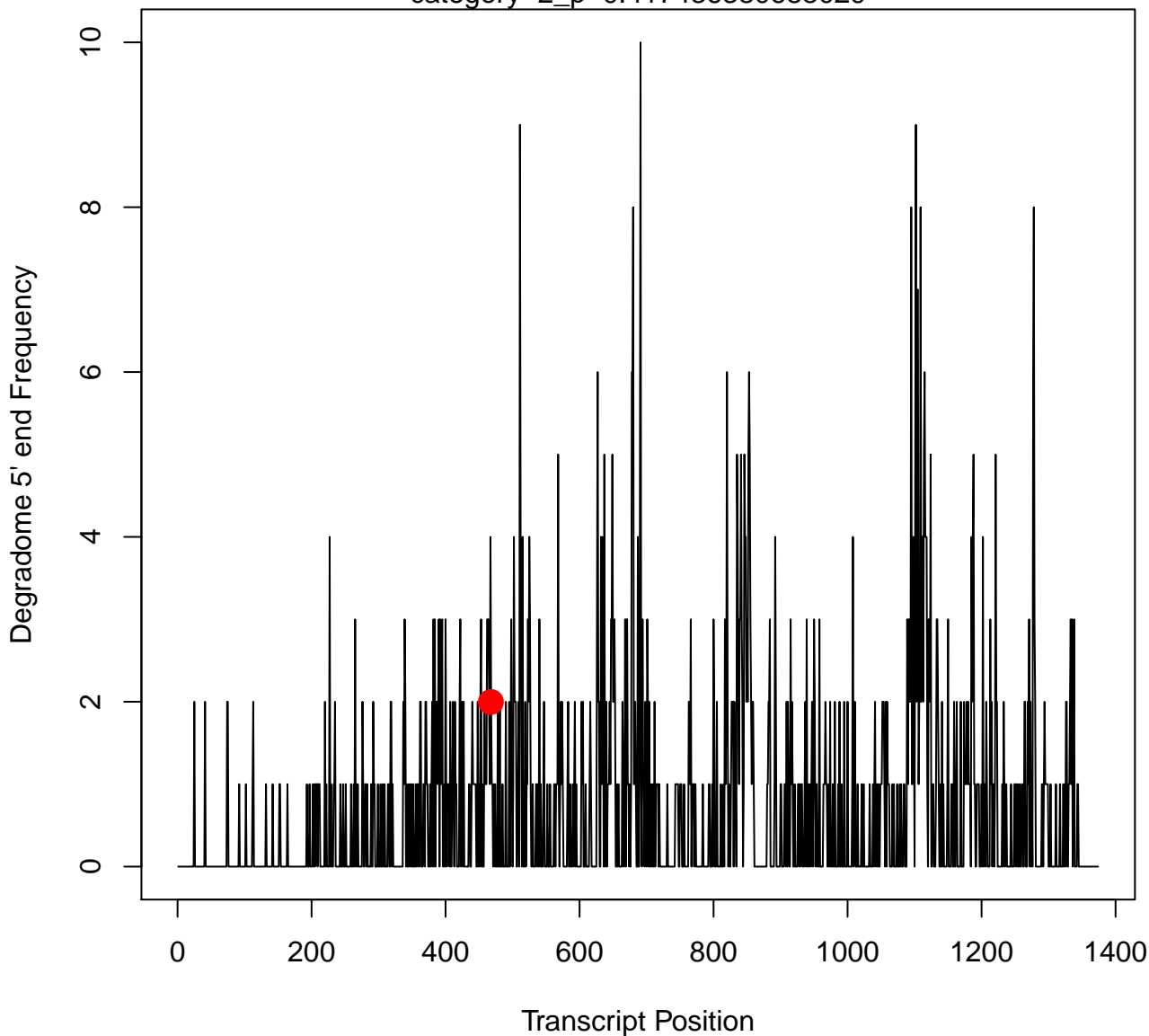

Supplement: Supplementary file 2 [file Data_Sheet_8.ZIP › GSM2230747.plot/Lsa-miR167a_Lsat_1_v5_gn_4_9441.1_468_TPlot.pdf]

**T=Lsat\_1\_v5\_gn\_2\_72540.1\_Q=Lsa-miR167d\_S=2777**

category=2\_p=0.146932609015138

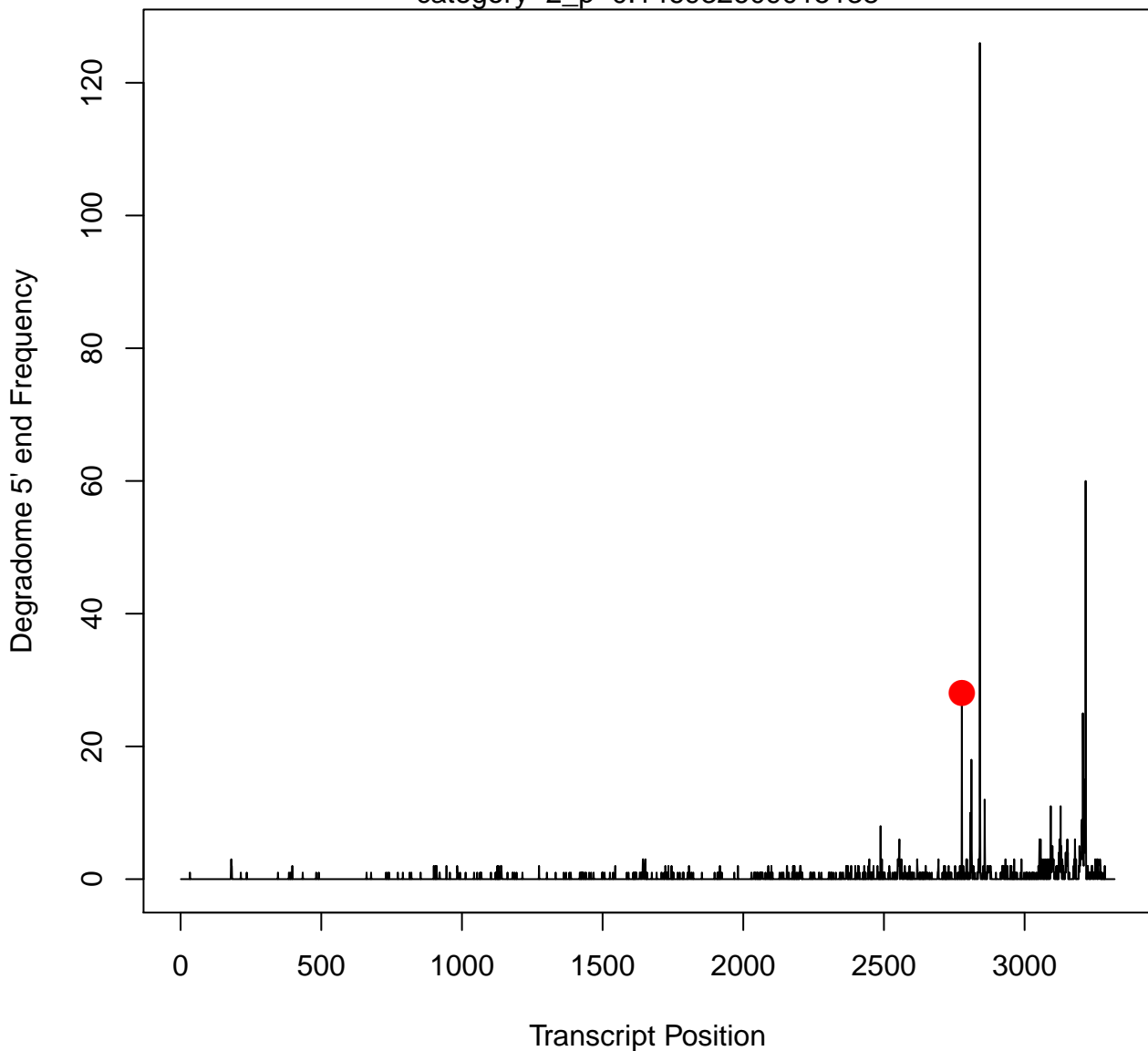

Supplement: Supplementary file 2 [file Data_Sheet_8.ZIP › GSM2230747.plot/Lsa-miR167d_Lsat_1_v5_gn_2_72540.1_2777_TPlot.pdf]

**T=Lsat\_1\_v5\_gn\_3\_121740.1\_Q=Lsa-miR167d\_S=283**

category=1\_p=0.000448555869187017

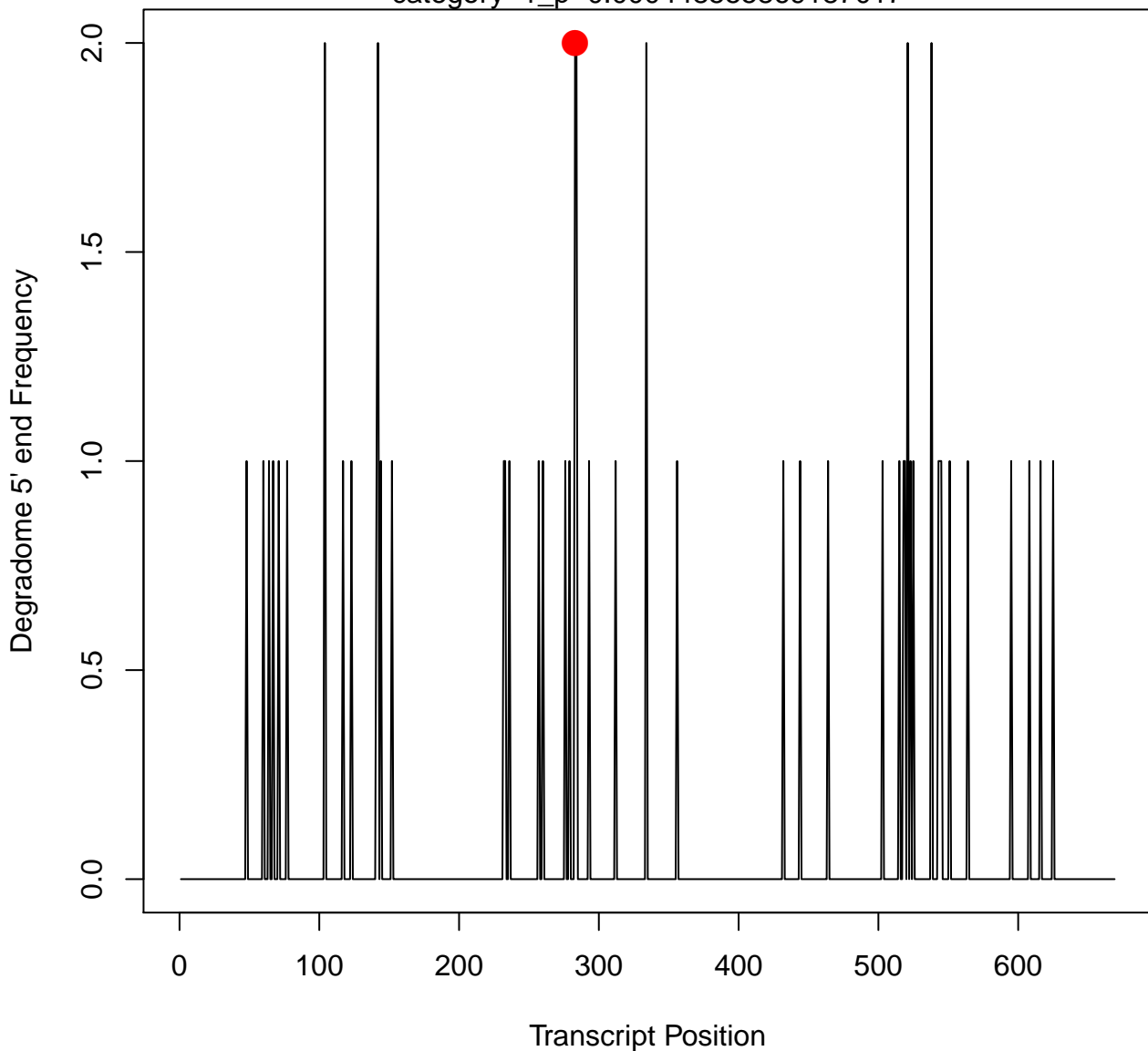

Supplement: Supplementary file 2 [file Data_Sheet_8.ZIP › GSM2230747.plot/Lsa-miR167d_Lsat_1_v5_gn_3_121740.1_283_TPlot.pdf]

**T=Lsat\_1\_v5\_gn\_7\_36161.1\_Q=Lsa-miR167d\_S=520**

category=2\_p=0.548230709948131

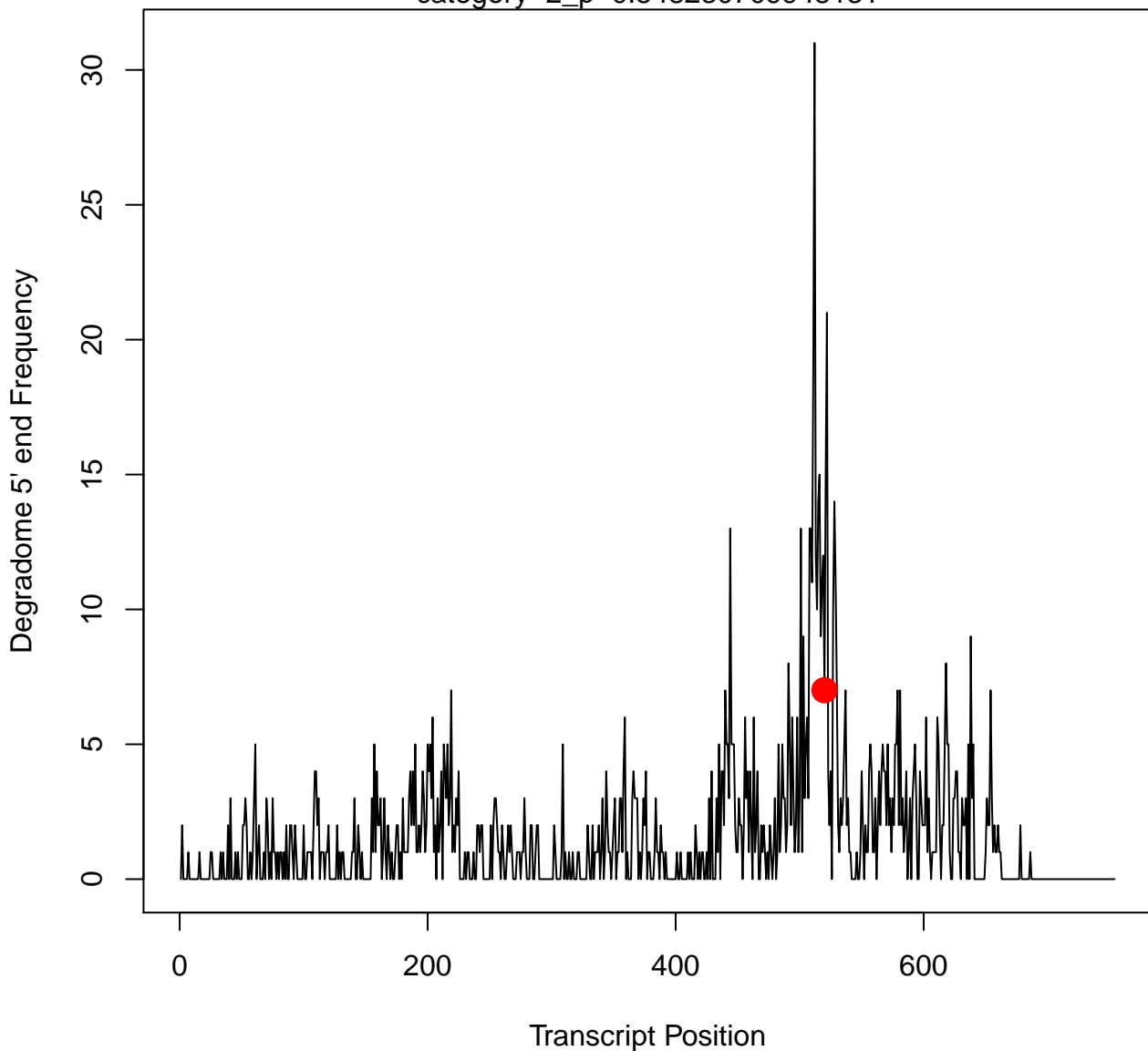

Supplement: Supplementary file 2 [file Data_Sheet_8.ZIP › GSM2230747.plot/Lsa-miR167d_Lsat_1_v5_gn_7_36161.1_520_TPlot.pdf]

**T=Lsat\_1\_v5\_gn\_3\_68721.1\_Q=Lsa-miR167e\_S=2661**

category=2\_p=0.146932609015138

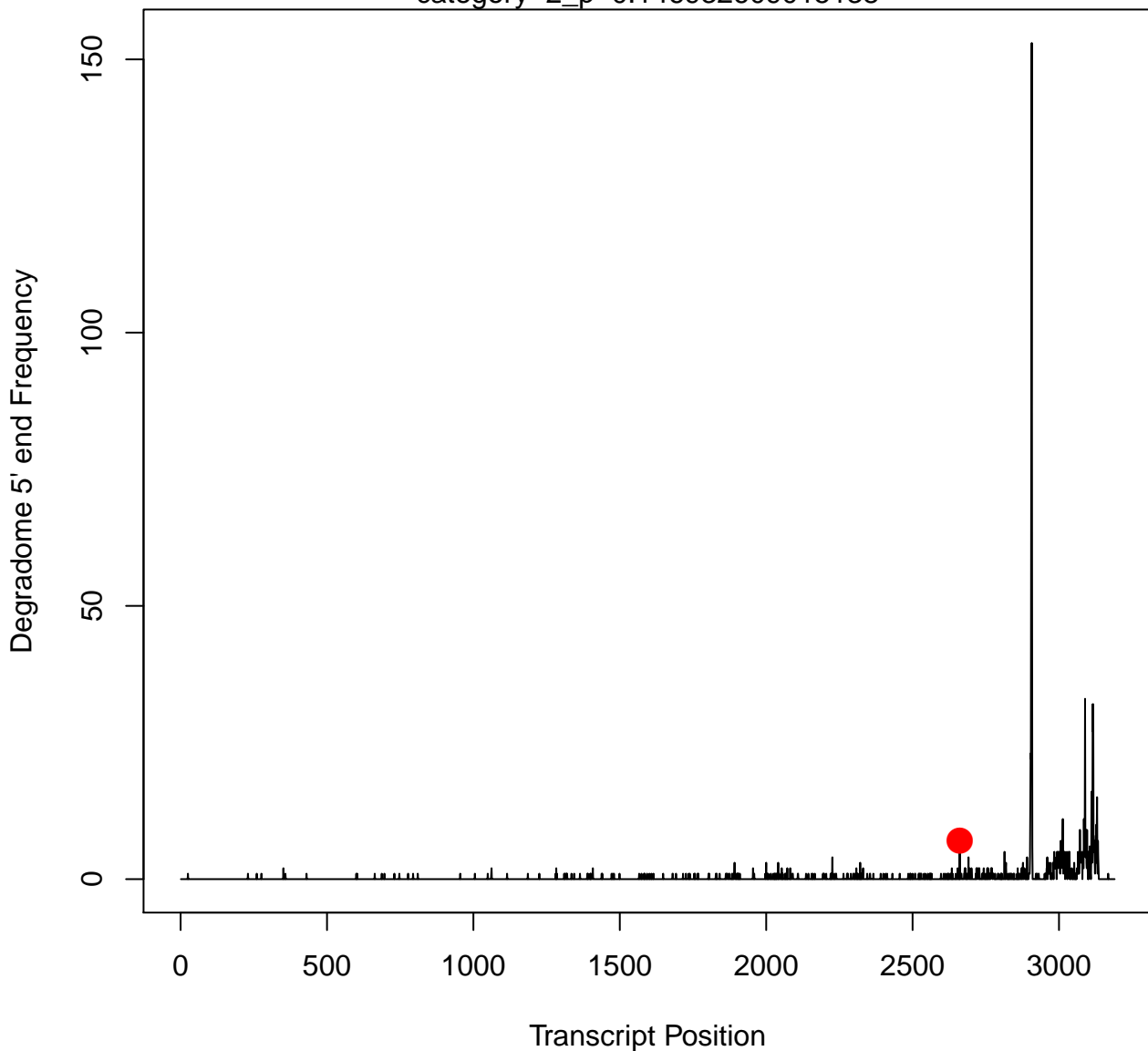

Supplement: Supplementary file 2 [file Data_Sheet_8.ZIP › GSM2230747.plot/Lsa-miR167e_Lsat_1_v5_gn_3_68721.1_2661_TPlot.pdf]

**T=Lsat\_1\_v5\_gn\_4\_159141.1\_Q=Lsa-miR167e\_S=1744**

category=2\_p=0.995638556349529

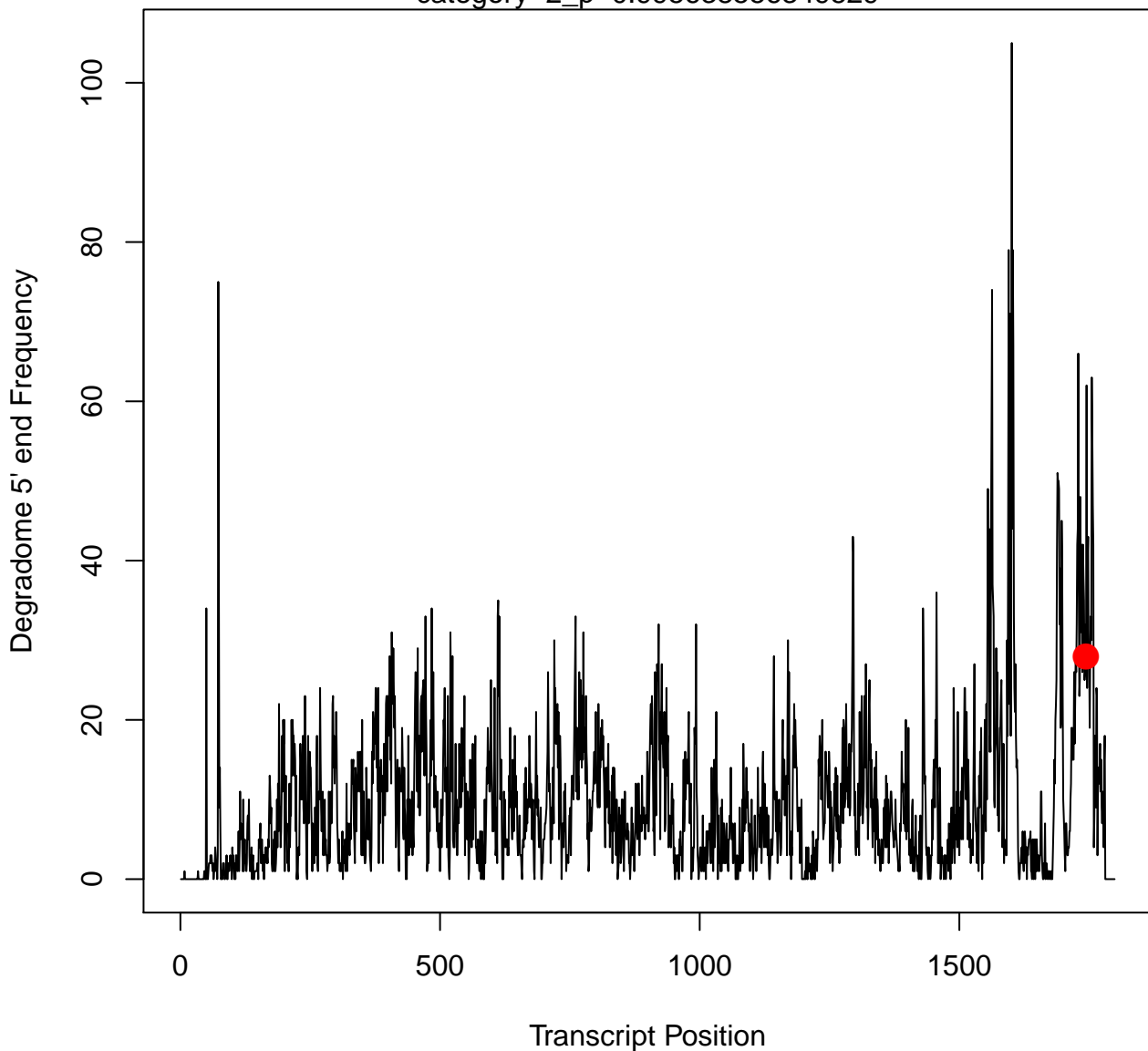

Supplement: Supplementary file 2 [file Data_Sheet_8.ZIP › GSM2230747.plot/Lsa-miR167e_Lsat_1_v5_gn_4_159141.1_1744_TPlot.pdf]

**T=Lsat\_1\_v5\_gn\_7\_27300.1\_Q=Lsa-miR167e\_S=2921**

category=0\_p=0.00147478967057835

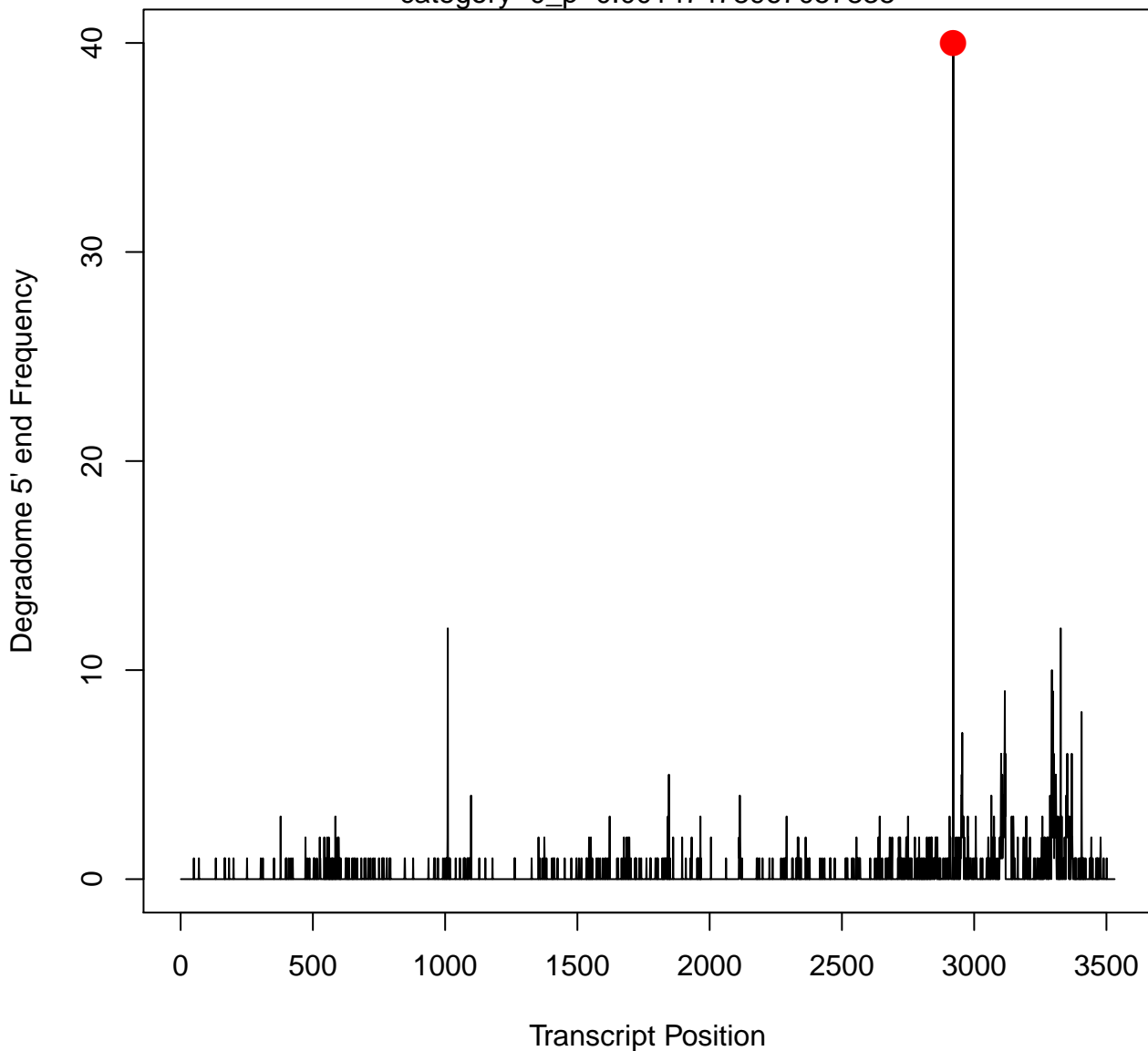

Supplement: Supplementary file 2 [file Data_Sheet_8.ZIP › GSM2230747.plot/Lsa-miR167e_Lsat_1_v5_gn_7_27300.1_2921_TPlot.pdf]

T=Lsat\_1\_v5\_gn\_1\_25461.1\_Q=Lsa-miR168a\_S=596

category=2\_p=0.944550605494866

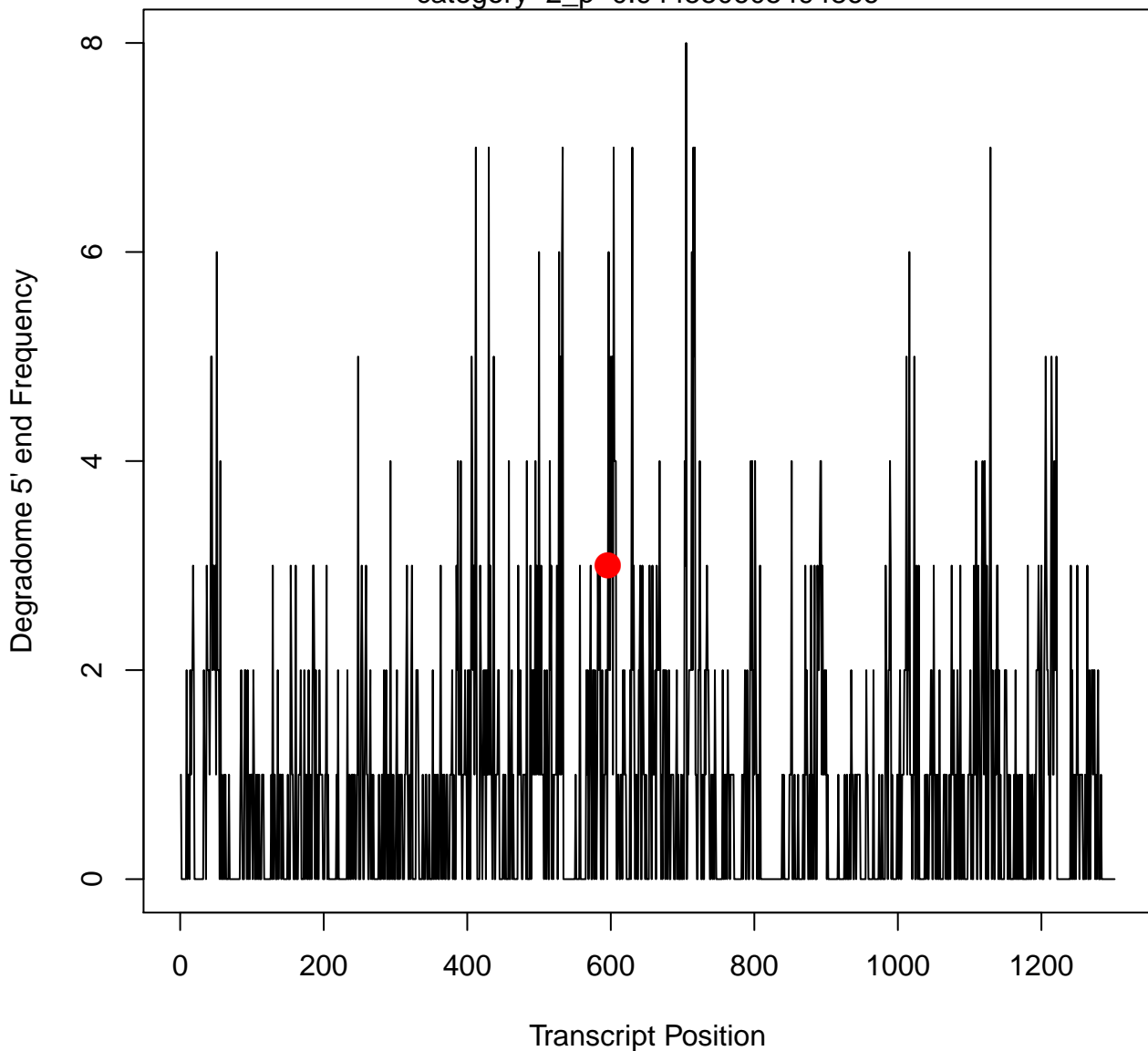

Supplement: Supplementary file 2 [file Data_Sheet_8.ZIP › GSM2230747.plot/Lsa-miR168a_Lsat_1_v5_gn_1_25461.1_596_TPlot.pdf]

**T=Lsat\_1\_v5\_gn\_1\_71221.1\_Q=Lsa-miR168a\_S=1266**

category=2\_p=0.877261700327688

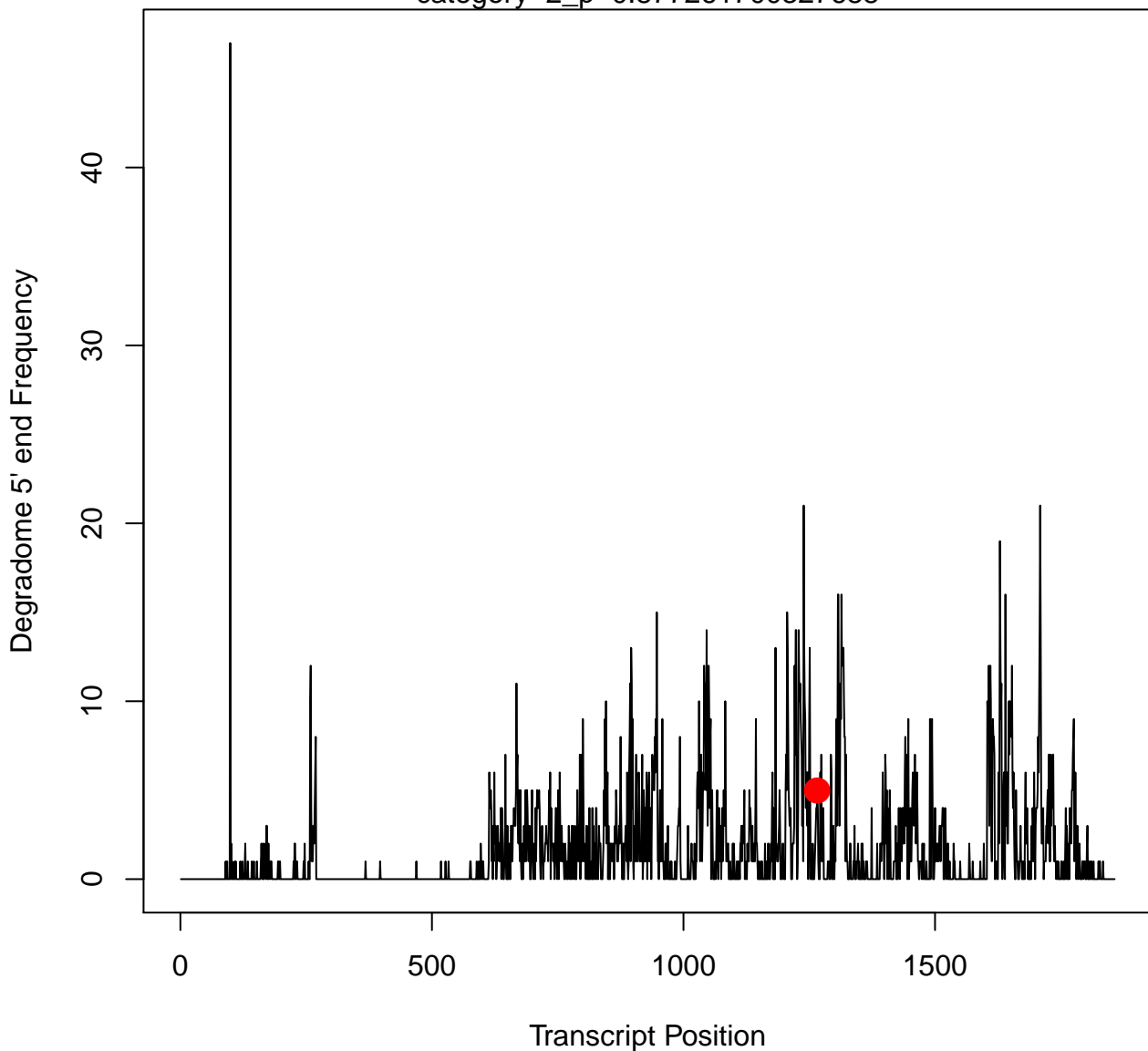

Supplement: Supplementary file 2 [file Data_Sheet_8.ZIP › GSM2230747.plot/Lsa-miR168a_Lsat_1_v5_gn_1_71221.1_1266_TPlot.pdf]

**T=Lsat\_1\_v5\_gn\_2\_37340.1\_Q=Lsa-miR168a\_S=441**

category=2\_p=0.94796564871546

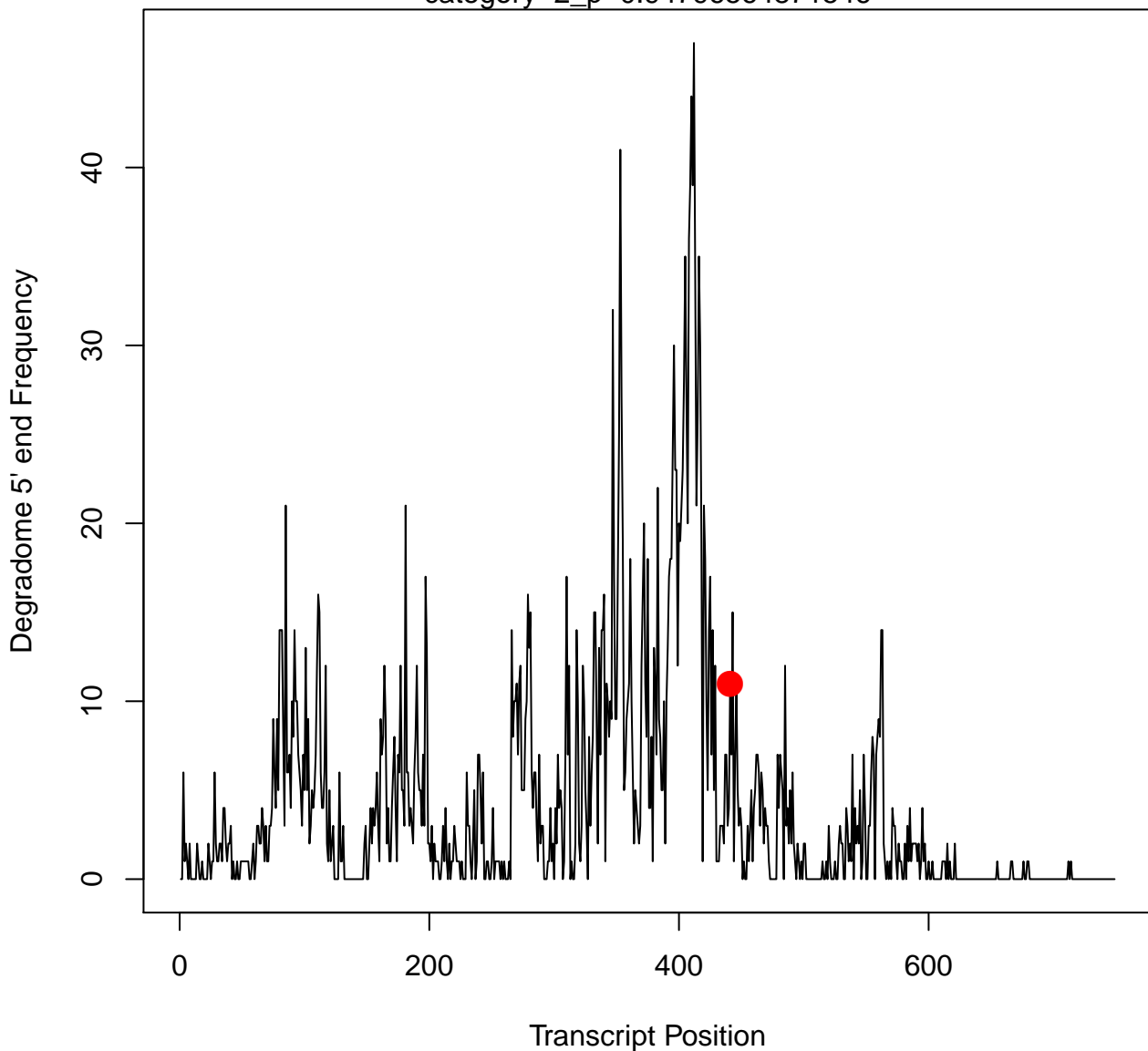

Supplement: Supplementary file 2 [file Data_Sheet_8.ZIP › GSM2230747.plot/Lsa-miR168a_Lsat_1_v5_gn_2_37340.1_441_TPlot.pdf]

**T=Lsat\_1\_v5\_gn\_3\_110841.1\_Q=Lsa-miR168a\_S=3532**

category=2\_p=0.562363663605761

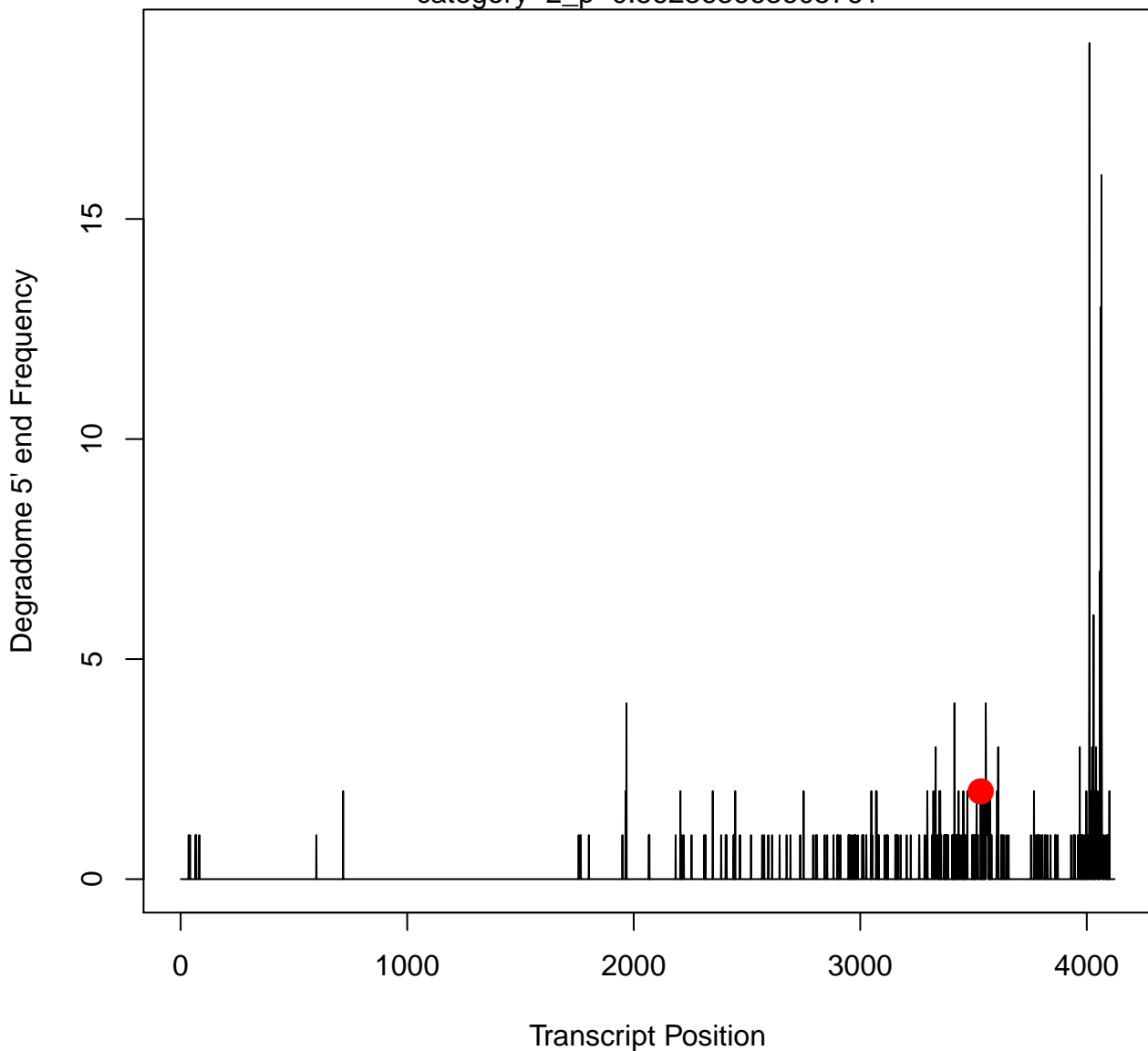

Supplement: Supplementary file 2 [file Data_Sheet_8.ZIP › GSM2230747.plot/Lsa-miR168a_Lsat_1_v5_gn_3_110841.1_3532_TPlot.pdf]

**T=Lsat\_1\_v5\_gn\_3\_35820.1\_Q=Lsa-miR168a\_S=1750**

category=2\_p=0.898571474531527

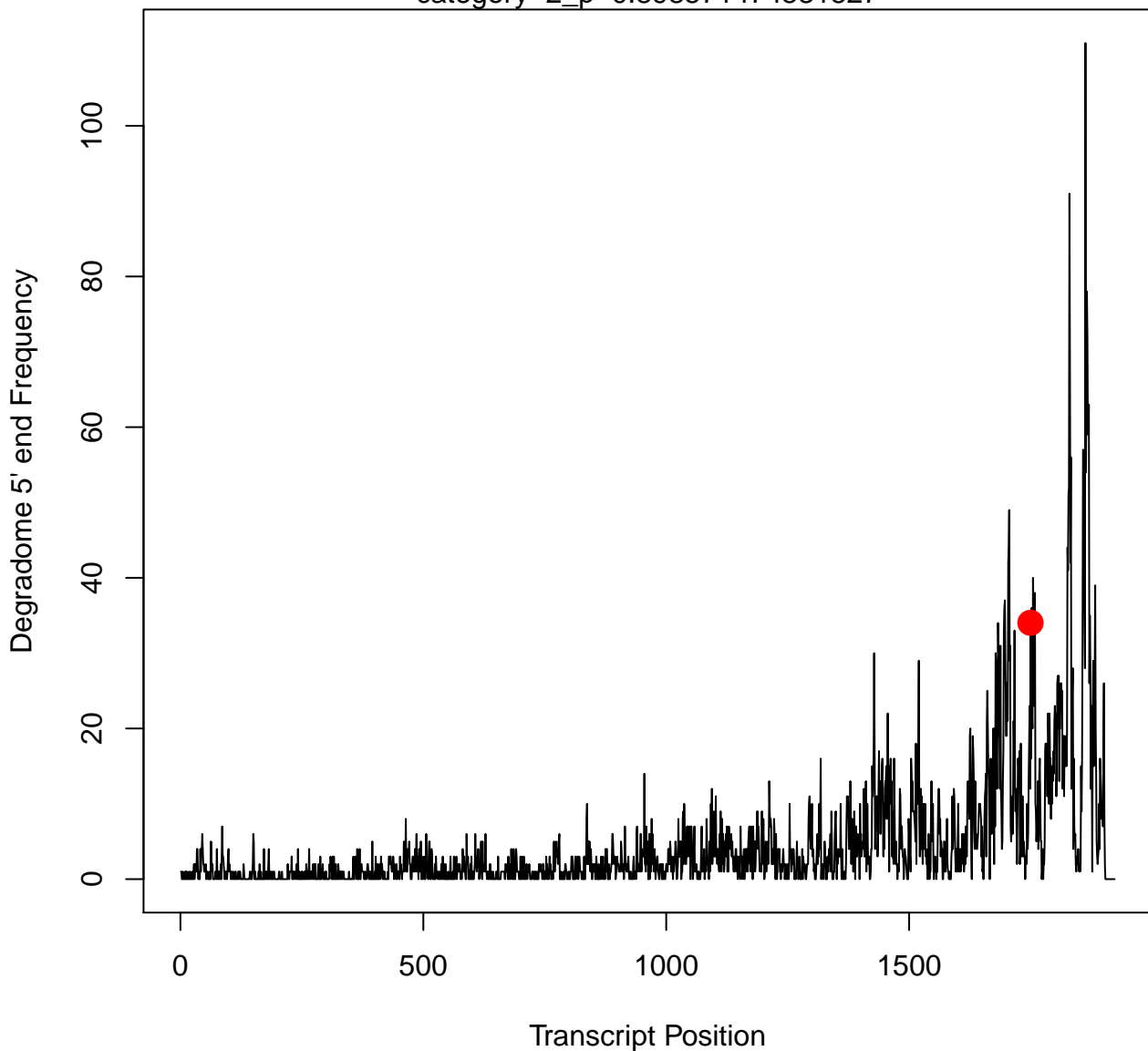

Supplement: Supplementary file 2 [file Data_Sheet_8.ZIP › GSM2230747.plot/Lsa-miR168a_Lsat_1_v5_gn_3_35820.1_1750_TPlot.pdf]

**T=Lsat\_1\_v5\_gn\_5\_103680.1\_Q=Lsa-miR168a\_S=1484**

category=2\_p=0.820270202938285

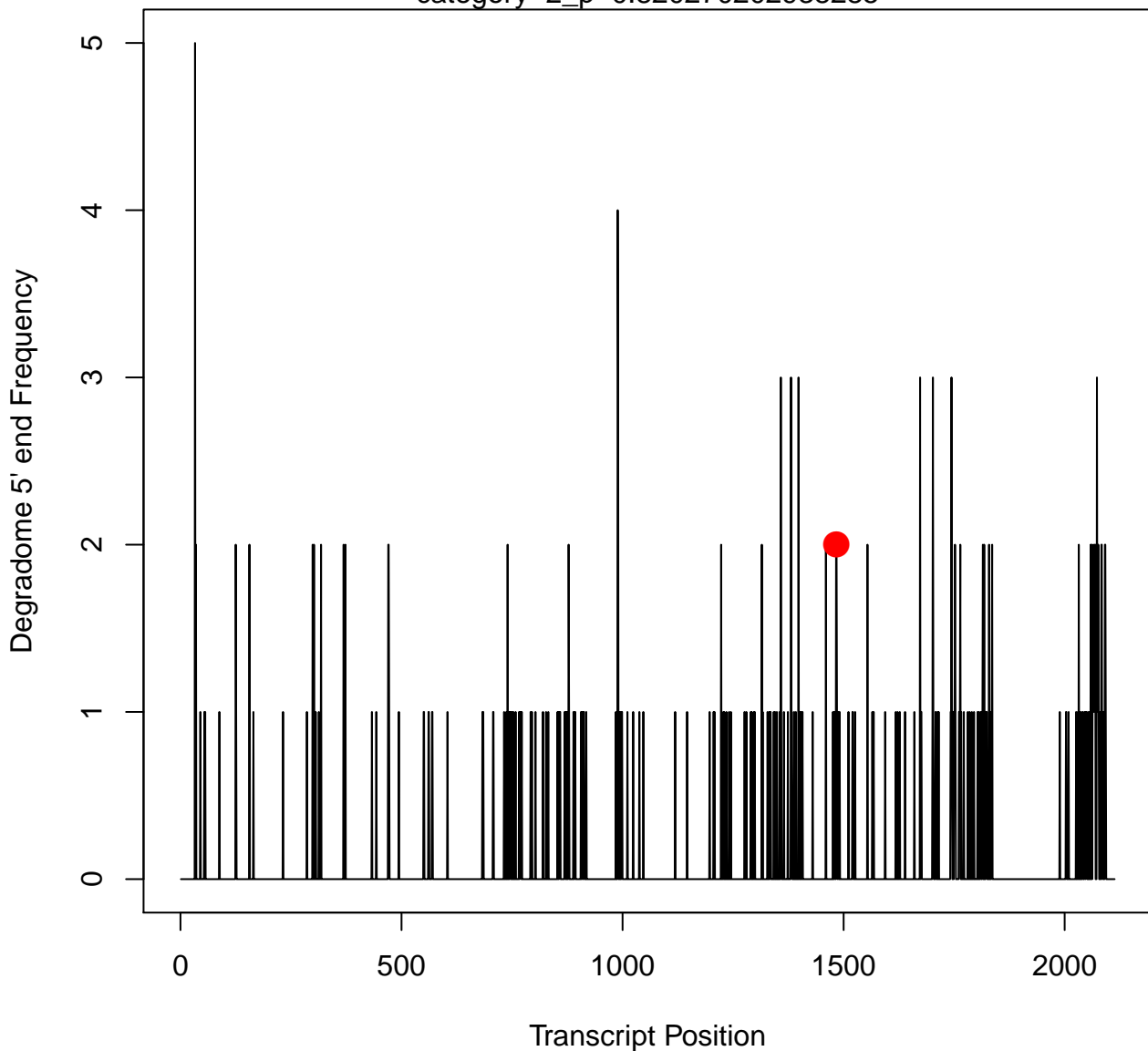

Supplement: Supplementary file 2 [file Data_Sheet_8.ZIP › GSM2230747.plot/Lsa-miR168a_Lsat_1_v5_gn_5_103680.1_1484_TPlot.pdf]

**T=Lsat\_1\_v5\_gn\_5\_56460.1\_Q=Lsa-miR168a\_S=213**

category=2\_p=0.338459119634894

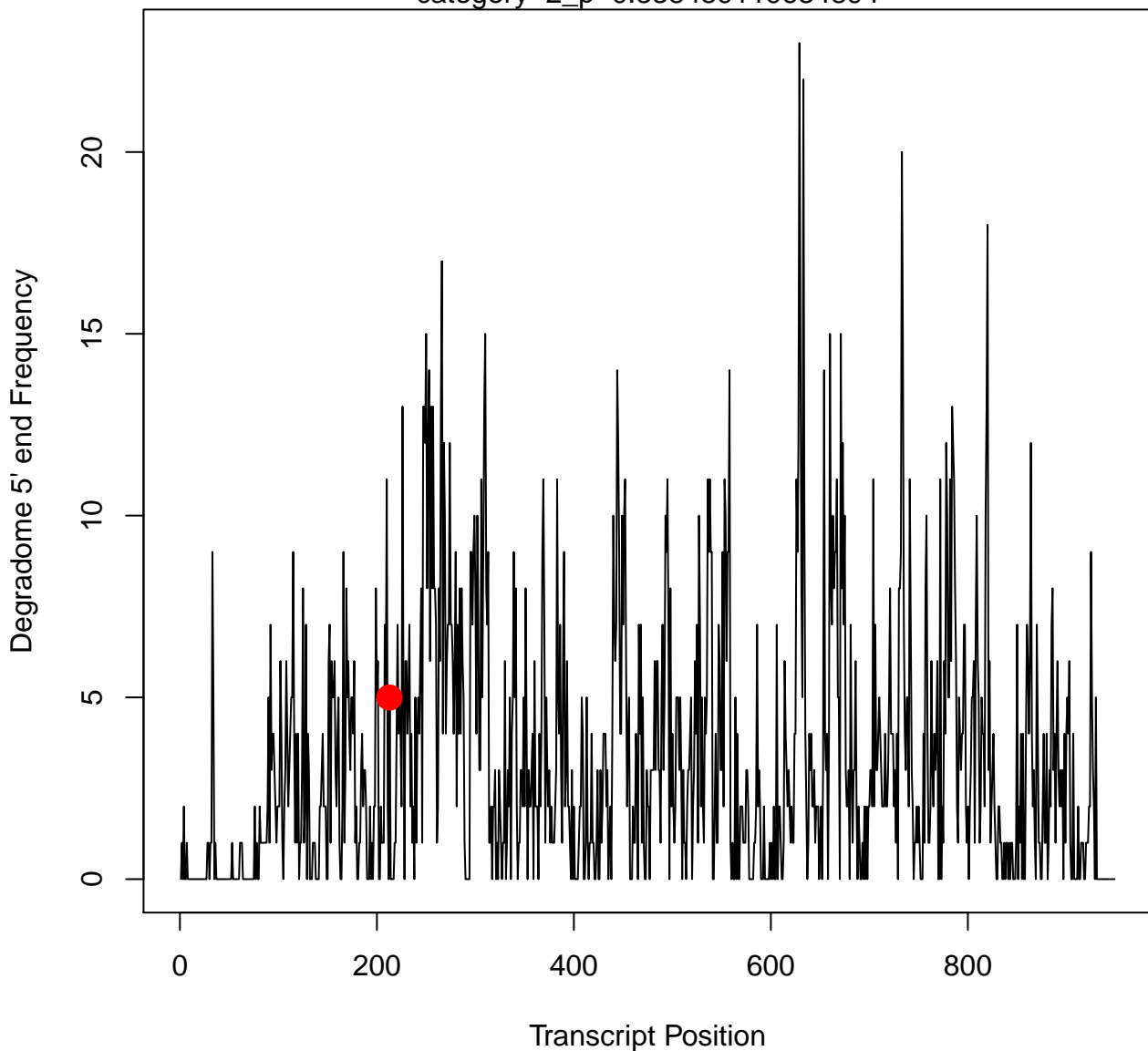

Supplement: Supplementary file 2 [file Data_Sheet_8.ZIP › GSM2230747.plot/Lsa-miR168a_Lsat_1_v5_gn_5_56460.1_213_TPlot.pdf]

**T=Lsat\_1\_v5\_gn\_8\_261.1\_Q=Lsa-miR168a\_S=1295**

category=0\_p=0.000368901499921082

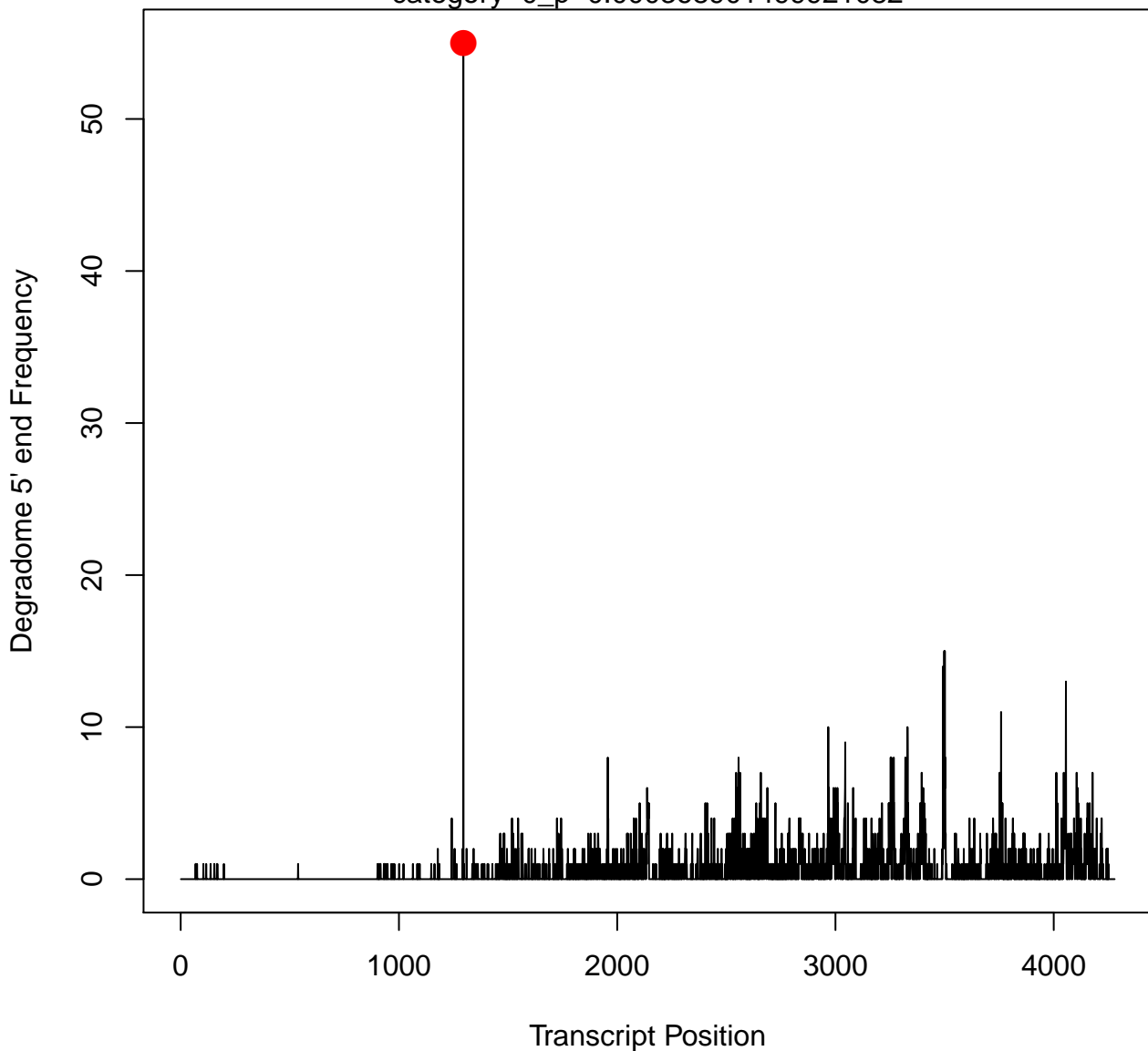

Supplement: Supplementary file 2 [file Data_Sheet_8.ZIP › GSM2230747.plot/Lsa-miR168a_Lsat_1_v5_gn_8_261.1_1295_TPlot.pdf]

**T=Lsat\_1\_v5\_gn\_9\_103440.1\_Q=Lsa-miR168a\_S=1363**

category=2\_p=0.681521546304191

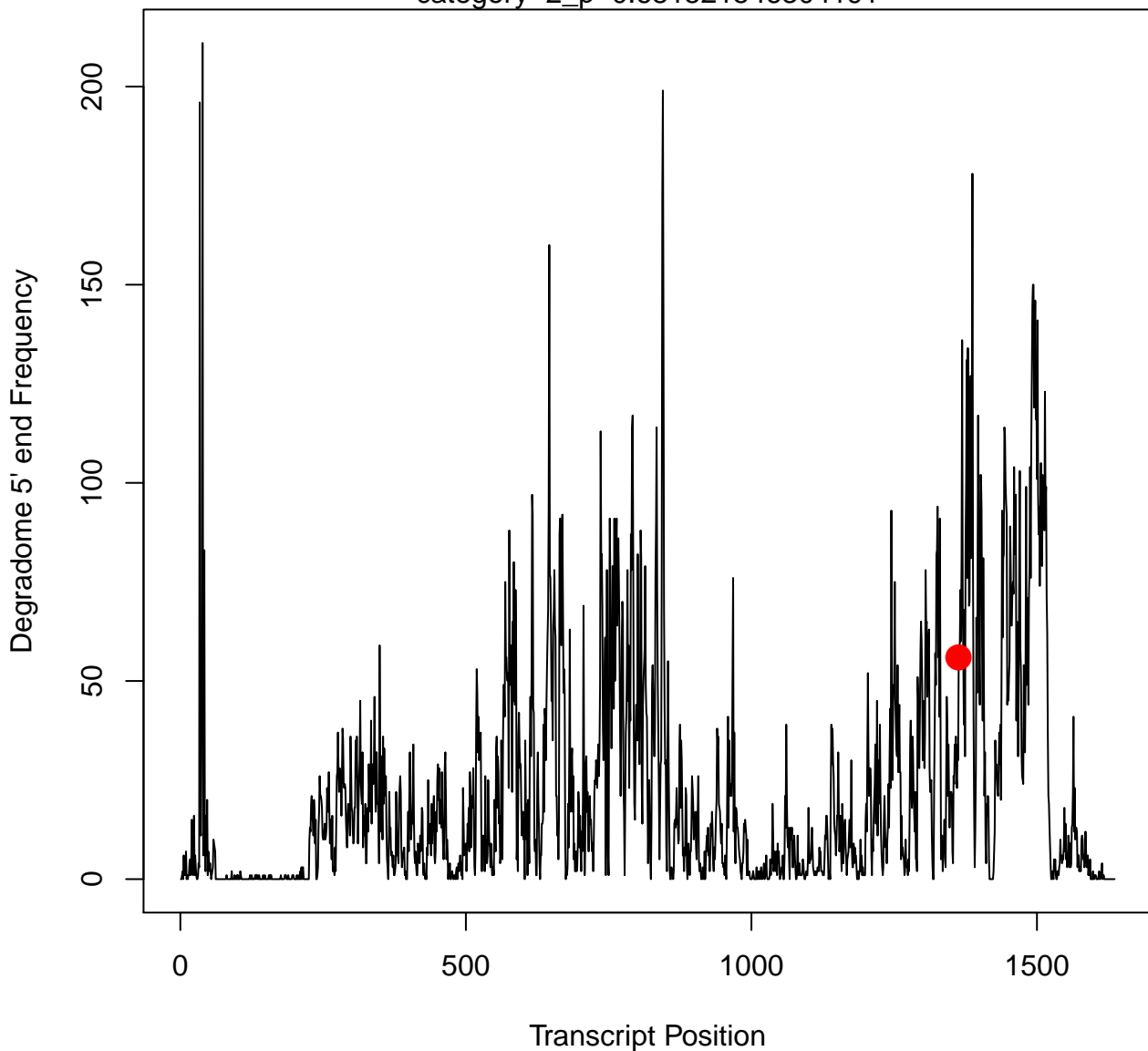

Supplement: Supplementary file 2 [file Data_Sheet_8.ZIP › GSM2230747.plot/Lsa-miR168a_Lsat_1_v5_gn_9_103440.1_1363_TPlot.pdf]

**T=Lsat\_1\_v5\_gn\_2\_19760.1\_Q=Lsa-miR168b\_S=1492**

category=0\_p=0.000368901499921082

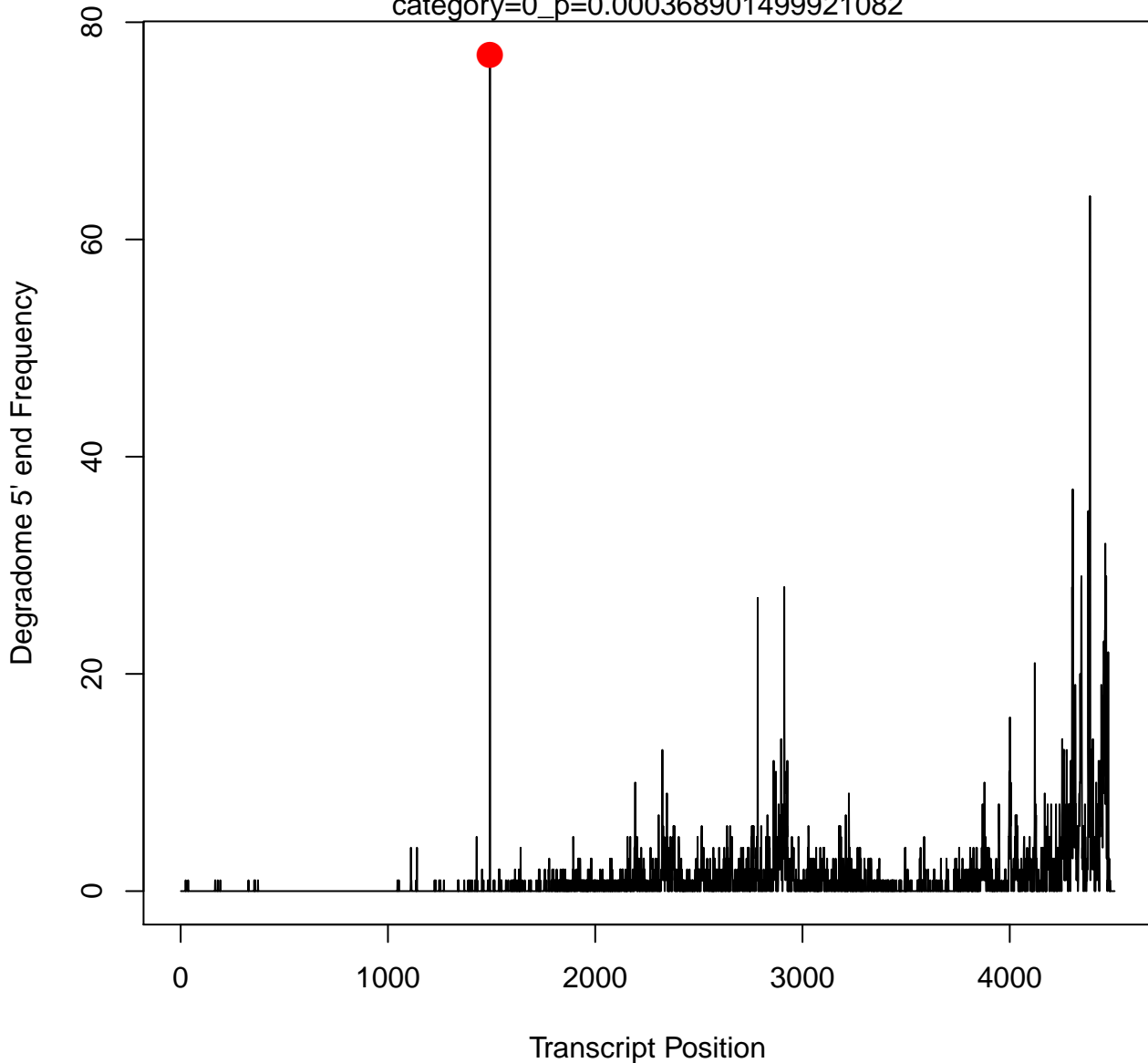

Supplement: Supplementary file 2 [file Data_Sheet_8.ZIP › GSM2230747.plot/Lsa-miR168b_Lsat_1_v5_gn_2_19760.1_1492_TPlot.pdf]

**T=Lsat\_1\_v5\_gn\_4\_173421.1\_Q=Lsa-miR168b\_S=938**

category=2\_p=0.782509608712971

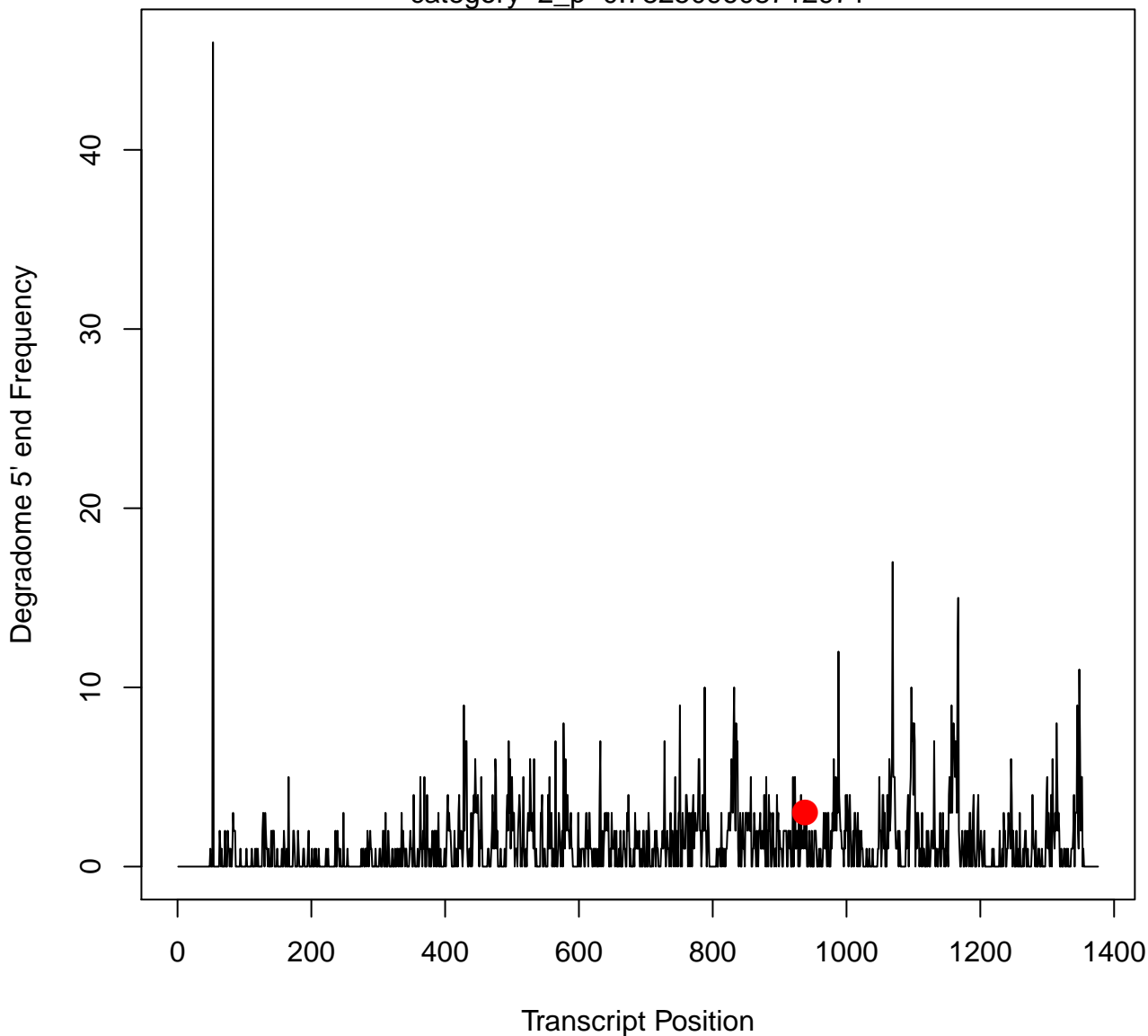

Supplement: Supplementary file 2 [file Data_Sheet_8.ZIP › GSM2230747.plot/Lsa-miR168b_Lsat_1_v5_gn_4_173421.1_938_TPlot.pdf]

**T=Lsat\_1\_v5\_gn\_8\_88620.1\_Q=Lsa-miR168b\_S=991**

category=2\_p=0.453315581104755

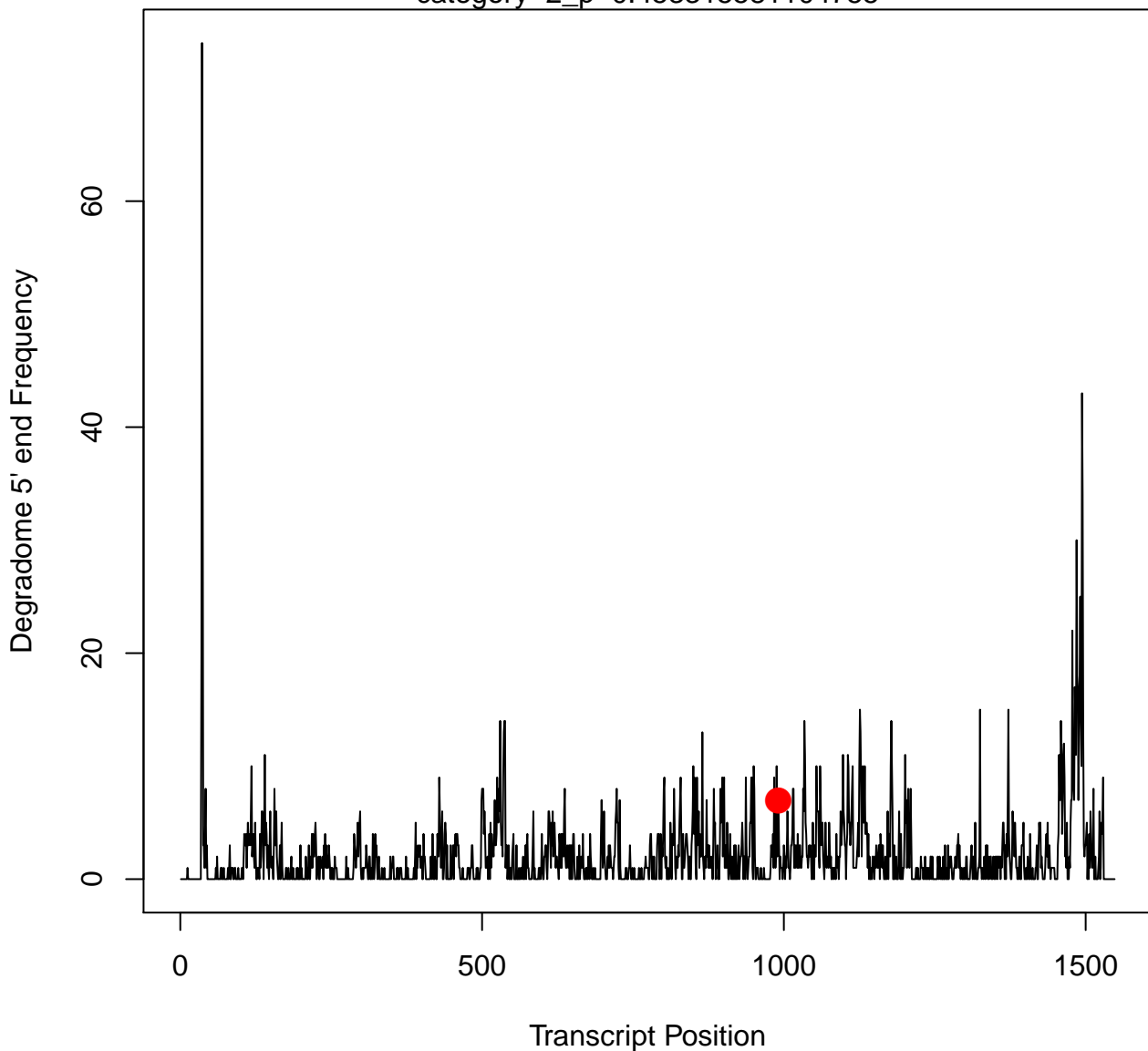

Supplement: Supplementary file 2 [file Data_Sheet_8.ZIP › GSM2230747.plot/Lsa-miR168b_Lsat_1_v5_gn_8_88620.1_991_TPlot.pdf]

**T=Lsat\_1\_v5\_gn\_1\_37160.1\_Q=Lsa-miR169a\_S=2396**

category=2\_p=0.0312835643520768

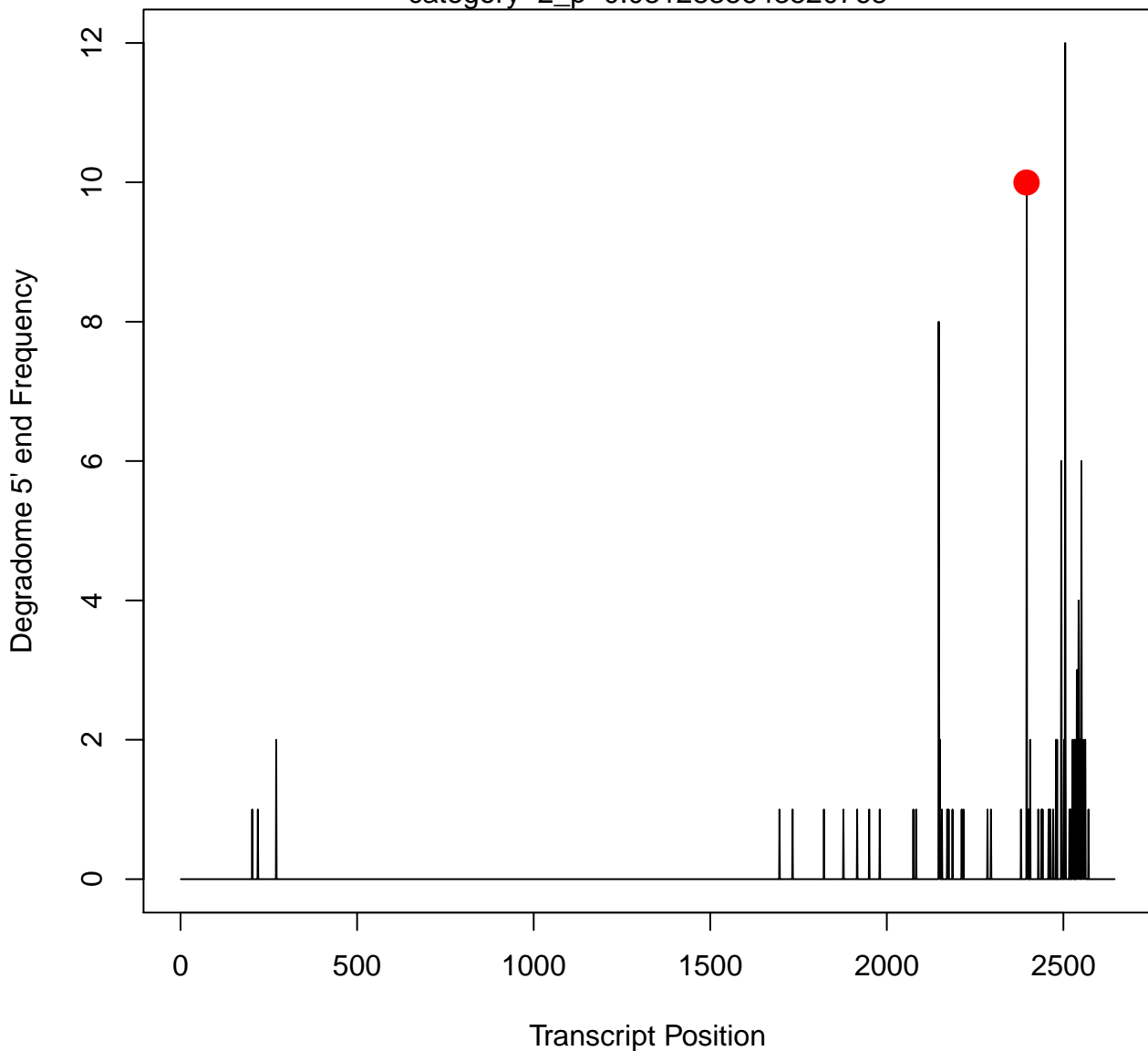

Supplement: Supplementary file 2 [file Data_Sheet_8.ZIP › GSM2230747.plot/Lsa-miR169a_Lsat_1_v5_gn_1_37160.1_2396_TPlot.pdf]

**T=Lsat\_1\_v5\_gn\_3\_90900.1\_Q=Lsa-miR169a\_S=642**

category=2\_p=0.814466039340497

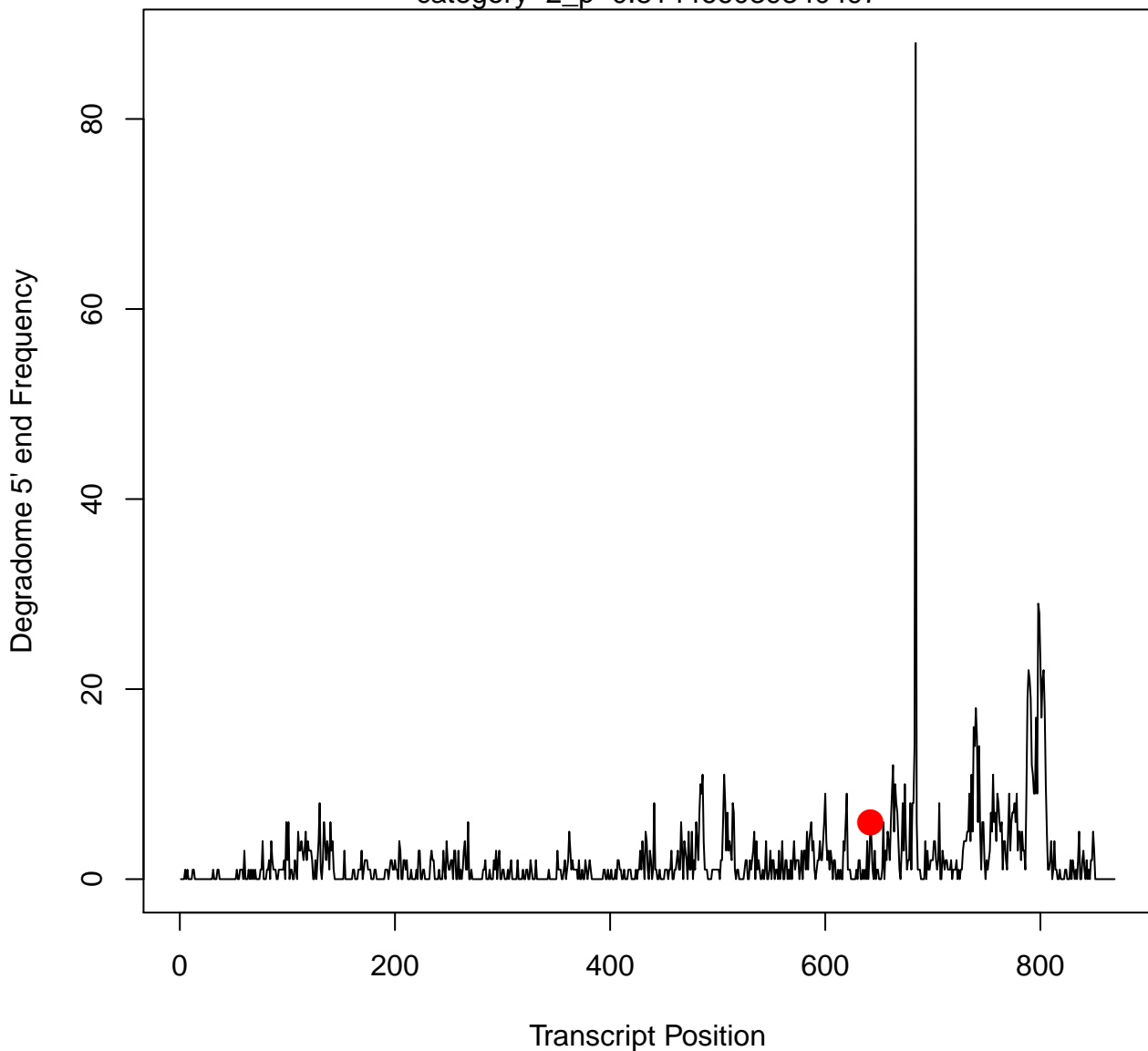

Supplement: Supplementary file 2 [file Data_Sheet_8.ZIP › GSM2230747.plot/Lsa-miR169a_Lsat_1_v5_gn_3_90900.1_642_TPlot.pdf]

**T=Lsat\_1\_v5\_gn\_4\_15920.1\_Q=Lsa-miR169a\_S=803**

category=2\_p=0.934999983481823

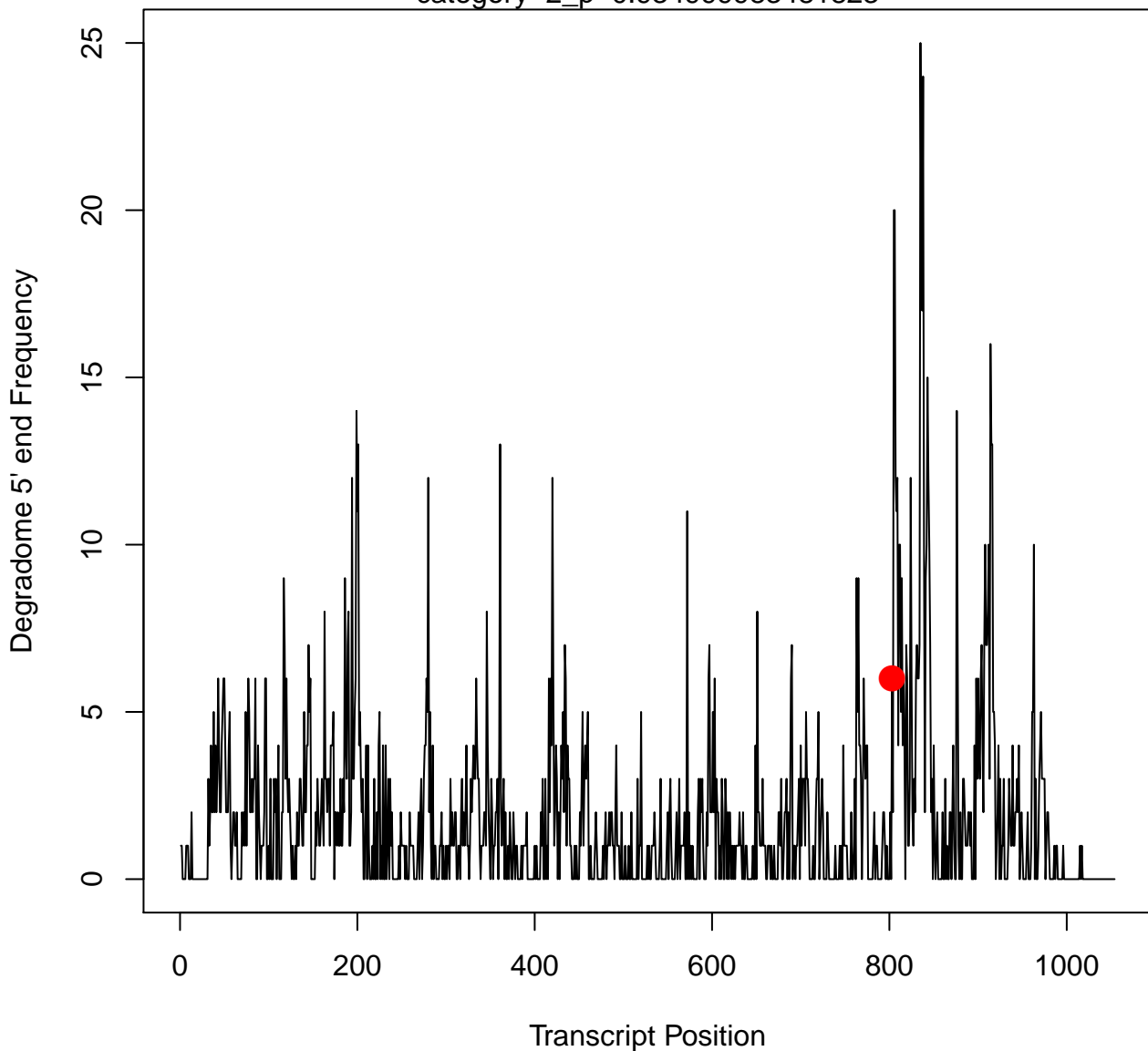

Supplement: Supplementary file 2 [file Data_Sheet_8.ZIP › GSM2230747.plot/Lsa-miR169a_Lsat_1_v5_gn_4_15920.1_803_TPlot.pdf]

**T=Lsat\_1\_v5\_gn\_5\_57660.1\_Q=Lsa-miR169a\_S=212**

category=2\_p=0.956999831848819

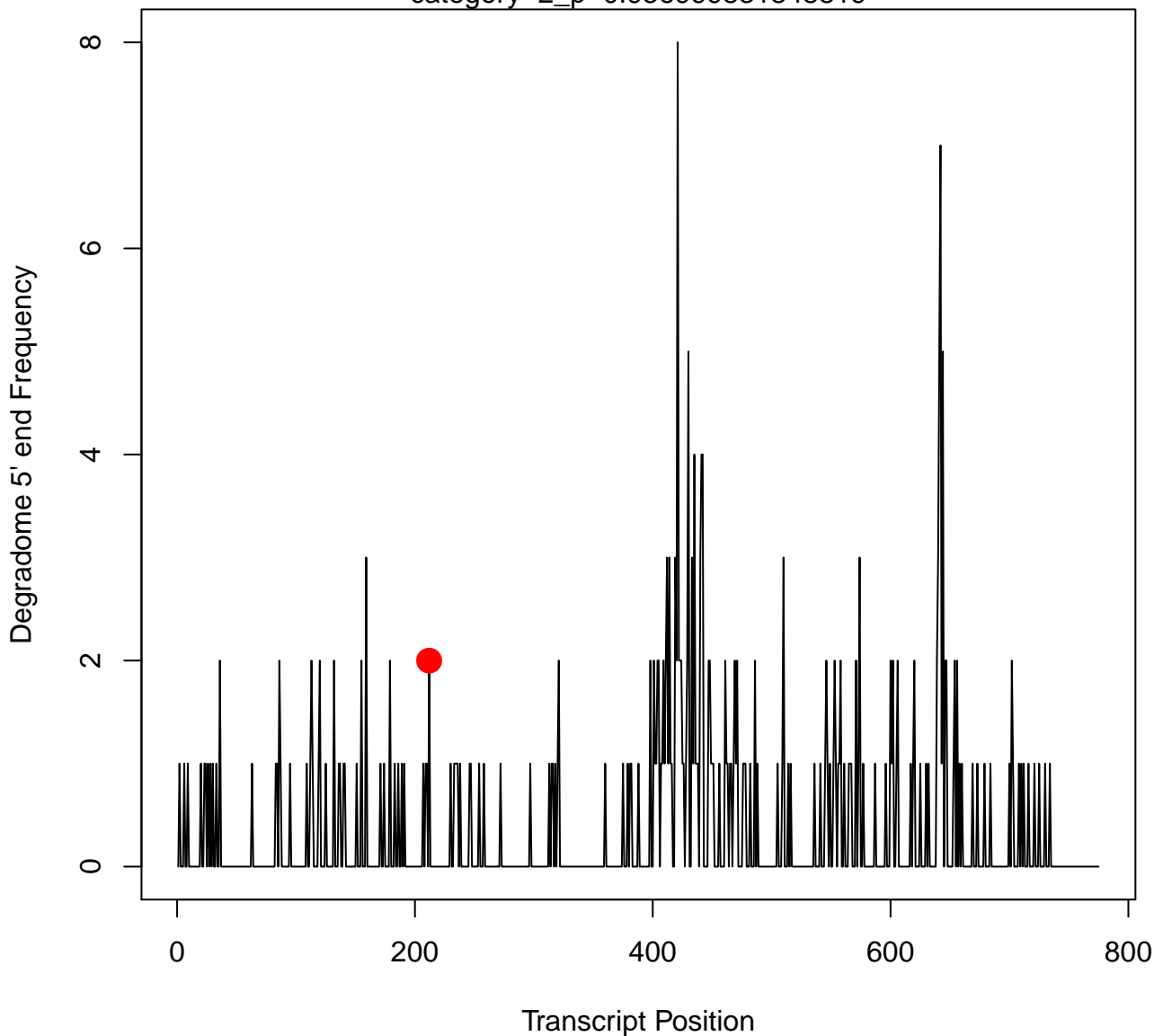

Supplement: Supplementary file 2 [file Data_Sheet_8.ZIP › GSM2230747.plot/Lsa-miR169a_Lsat_1_v5_gn_5_57660.1_212_TPlot.pdf]

**T=Lsat\_1\_v5\_gn\_6\_97140.1\_Q=Lsa-miR169a\_S=825**

category=2\_p=0.701136146137168

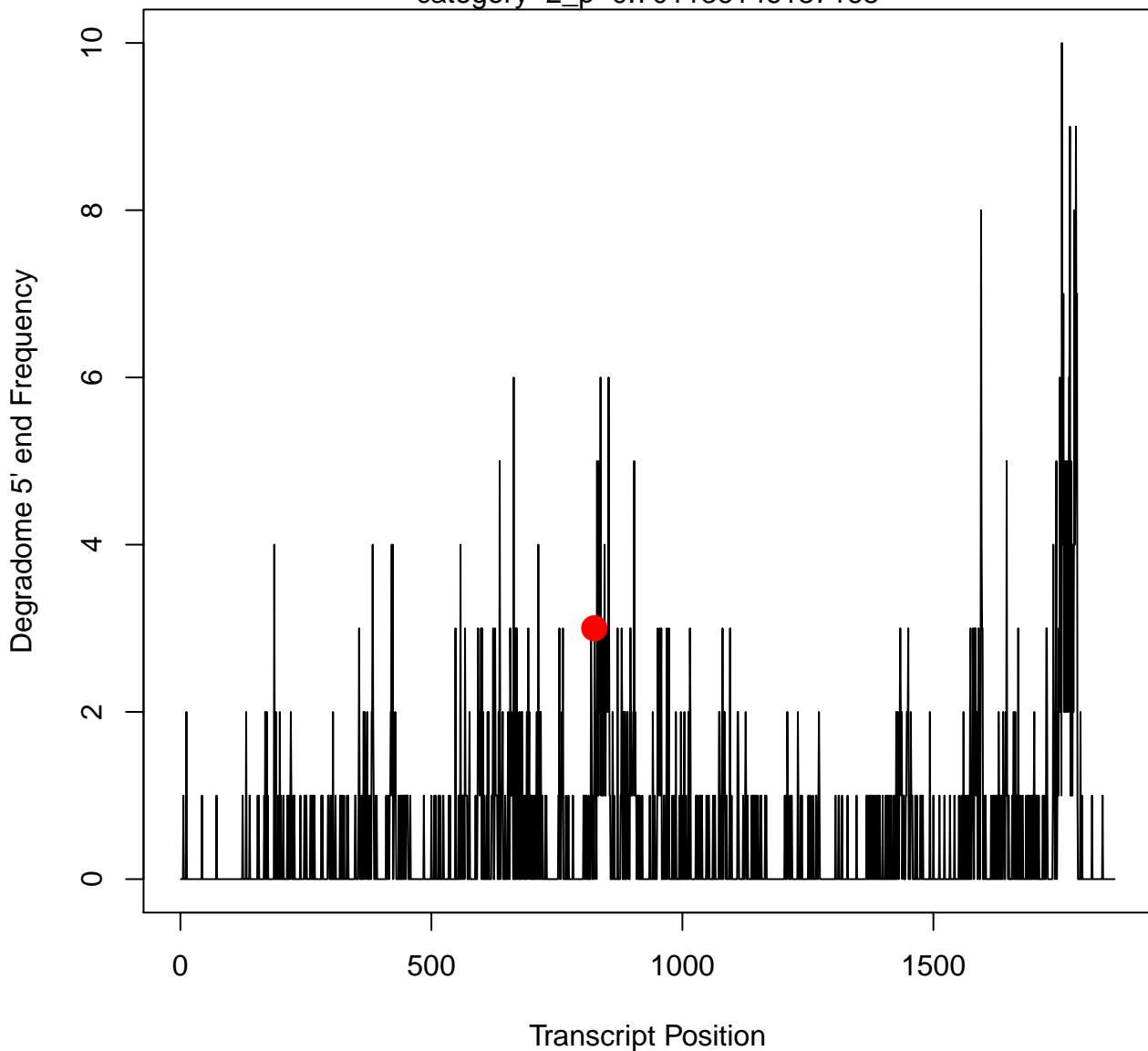

Supplement: Supplementary file 2 [file Data_Sheet_8.ZIP › GSM2230747.plot/Lsa-miR169a_Lsat_1_v5_gn_6_97140.1_825_TPlot.pdf]

**T=Lsat\_1\_v5\_gn\_7\_116120.1\_Q=Lsa-miR169b\_S=323**

category=2\_p=0.990034091578397

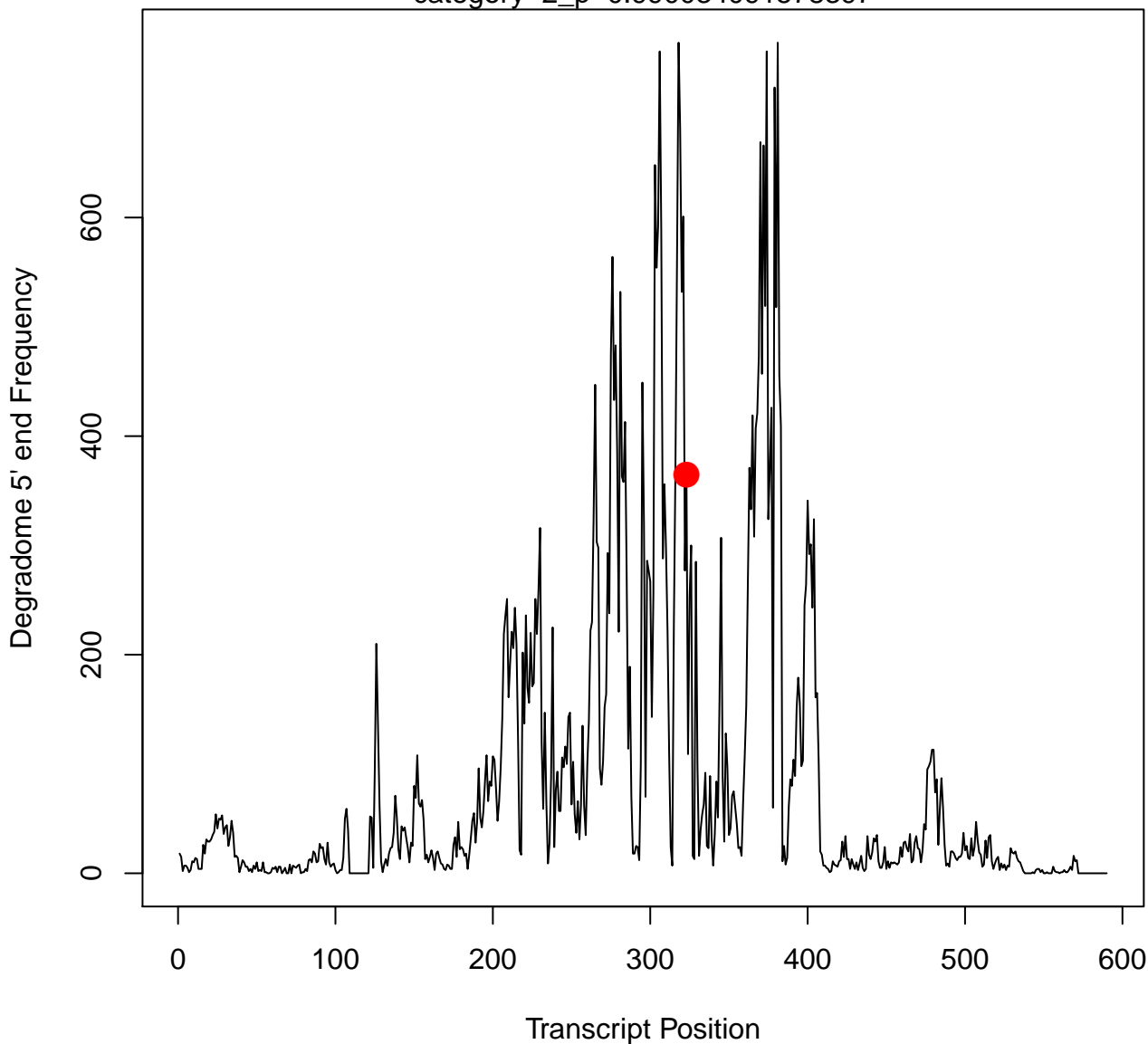

Supplement: Supplementary file 2 [file Data_Sheet_8.ZIP › GSM2230747.plot/Lsa-miR169b_Lsat_1_v5_gn_7_116120.1_323_TPlot.pdf]

**T=Lsat\_1\_v5\_gn\_8\_111520.1\_Q=Lsa-miR169d\_S=266**

category=2\_p=0.918803597180262

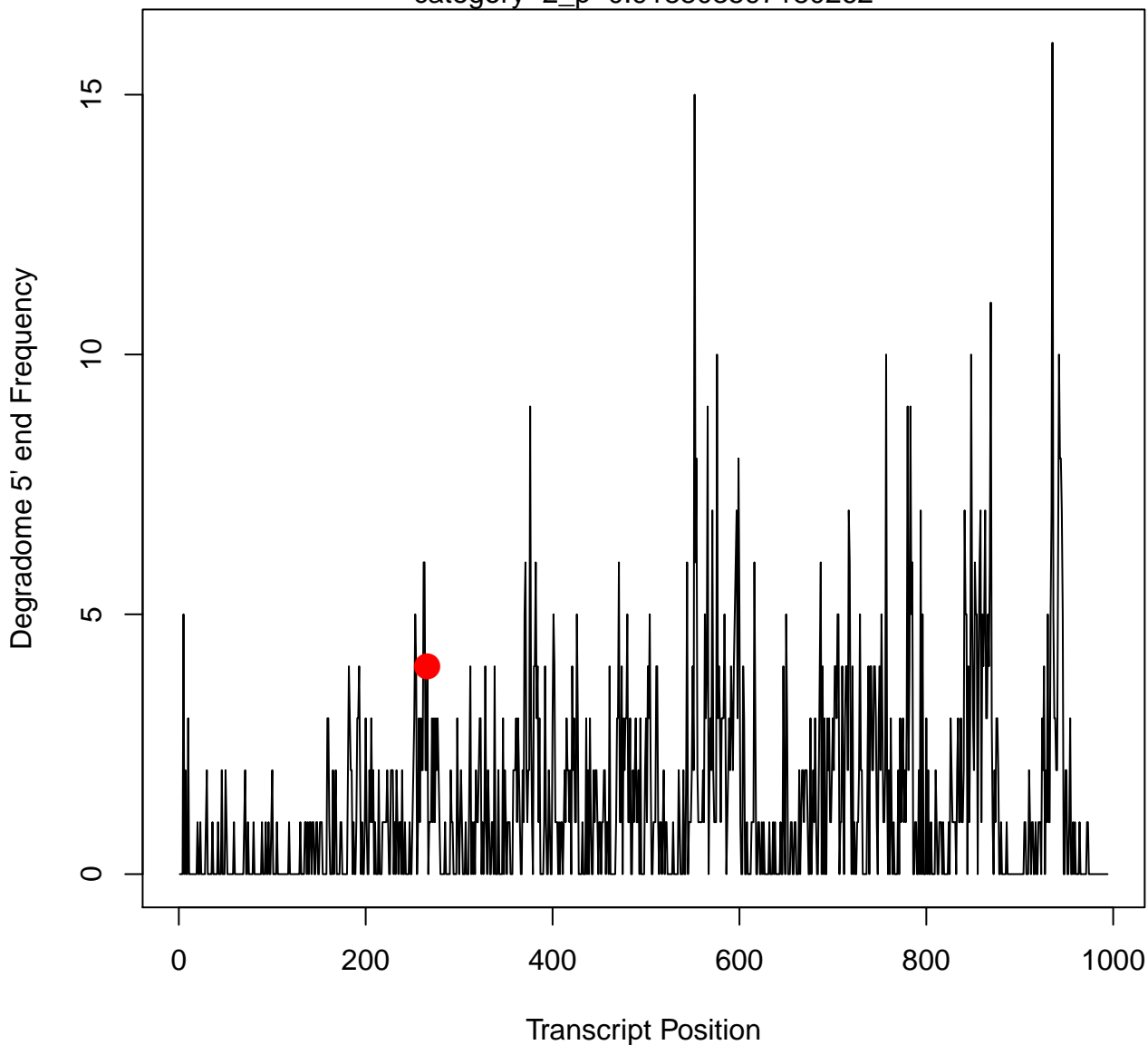

Supplement: Supplementary file 2 [file Data_Sheet_8.ZIP › GSM2230747.plot/Lsa-miR169d_Lsat_1_v5_gn_8_111520.1_266_TPlot.pdf]

T=Lsat\_1\_v5\_gn\_1\_43561.1\_Q=Lsa-miR169f\_S=171

category=2\_p=0.989712254221291

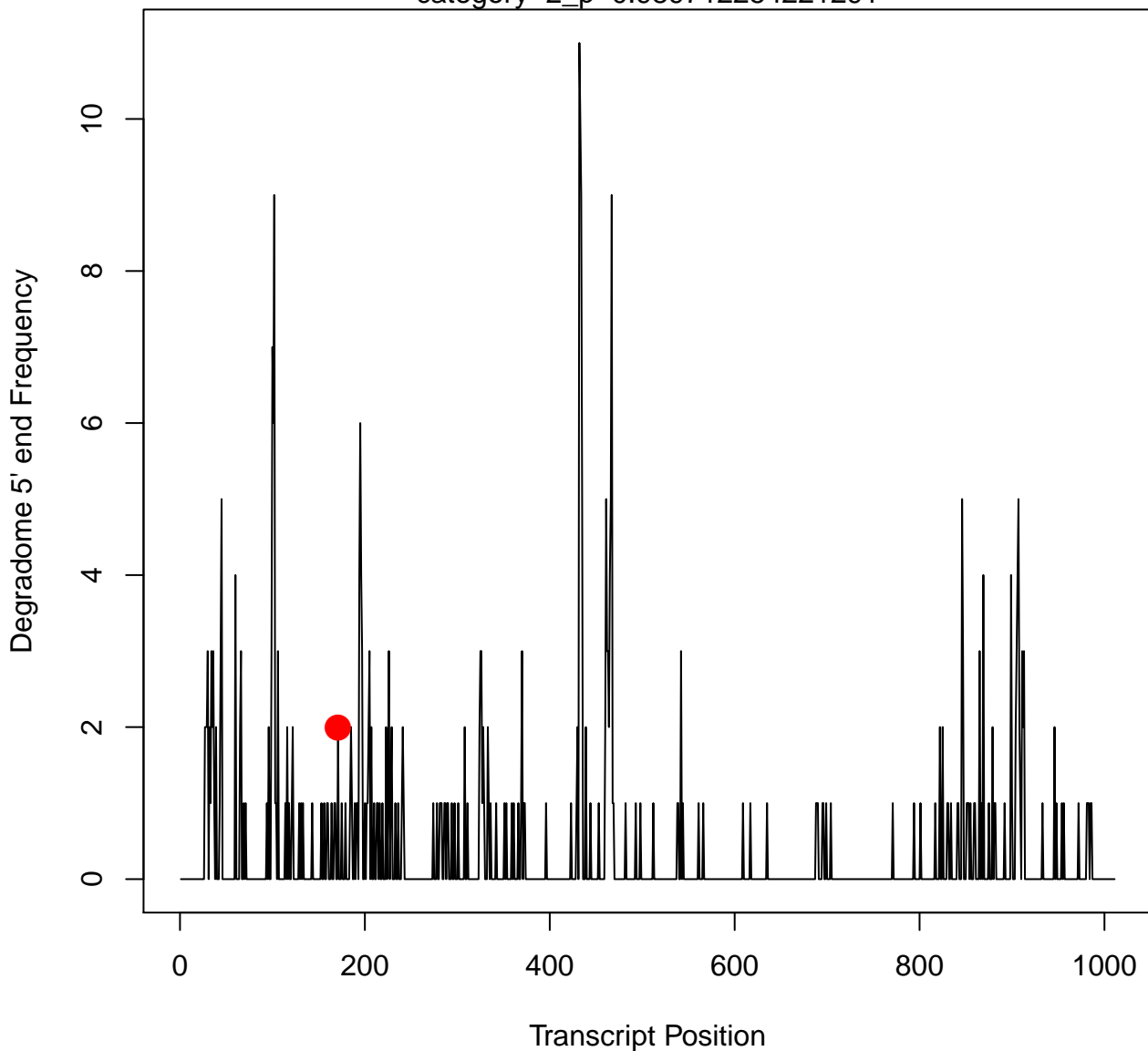

Supplement: Supplementary file 2 [file Data_Sheet_8.ZIP › GSM2230747.plot/Lsa-miR169f_Lsat_1_v5_gn_1_43561.1_171_TPlot.pdf]

**T=Lsat\_1\_v5\_gn\_6\_47121.1\_Q=Lsa-miR169i\_S=1633**

category=0\_p=0.00441784720569194

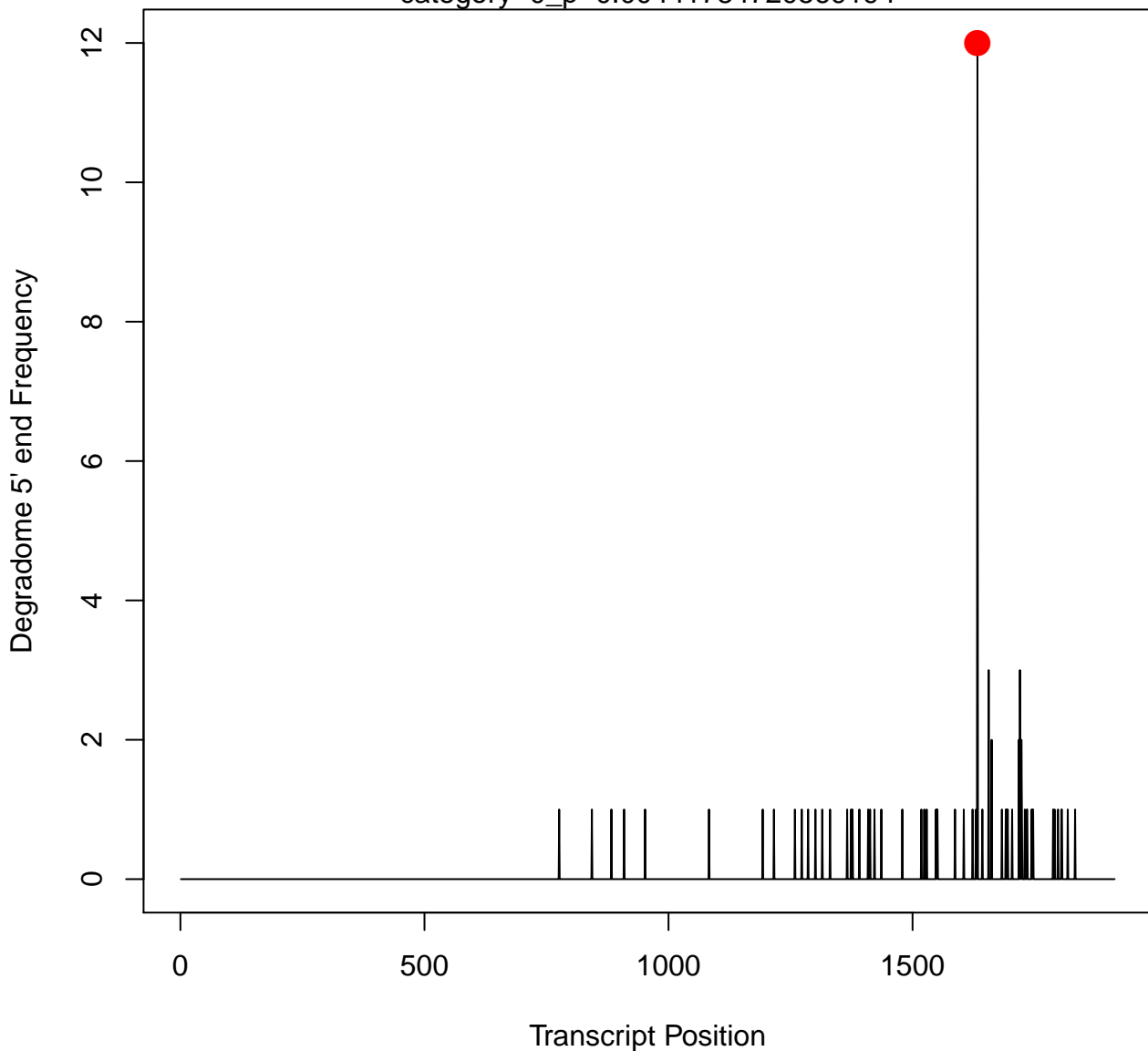

Supplement: Supplementary file 2 [file Data_Sheet_8.ZIP › GSM2230747.plot/Lsa-miR169i_Lsat_1_v5_gn_6_47121.1_1633_TPlot.pdf]

**T=Lsat\_1\_v5\_gn\_7\_34841.1\_Q=Lsa-miR169i\_S=986**

category=0\_p=0.00110629628501624

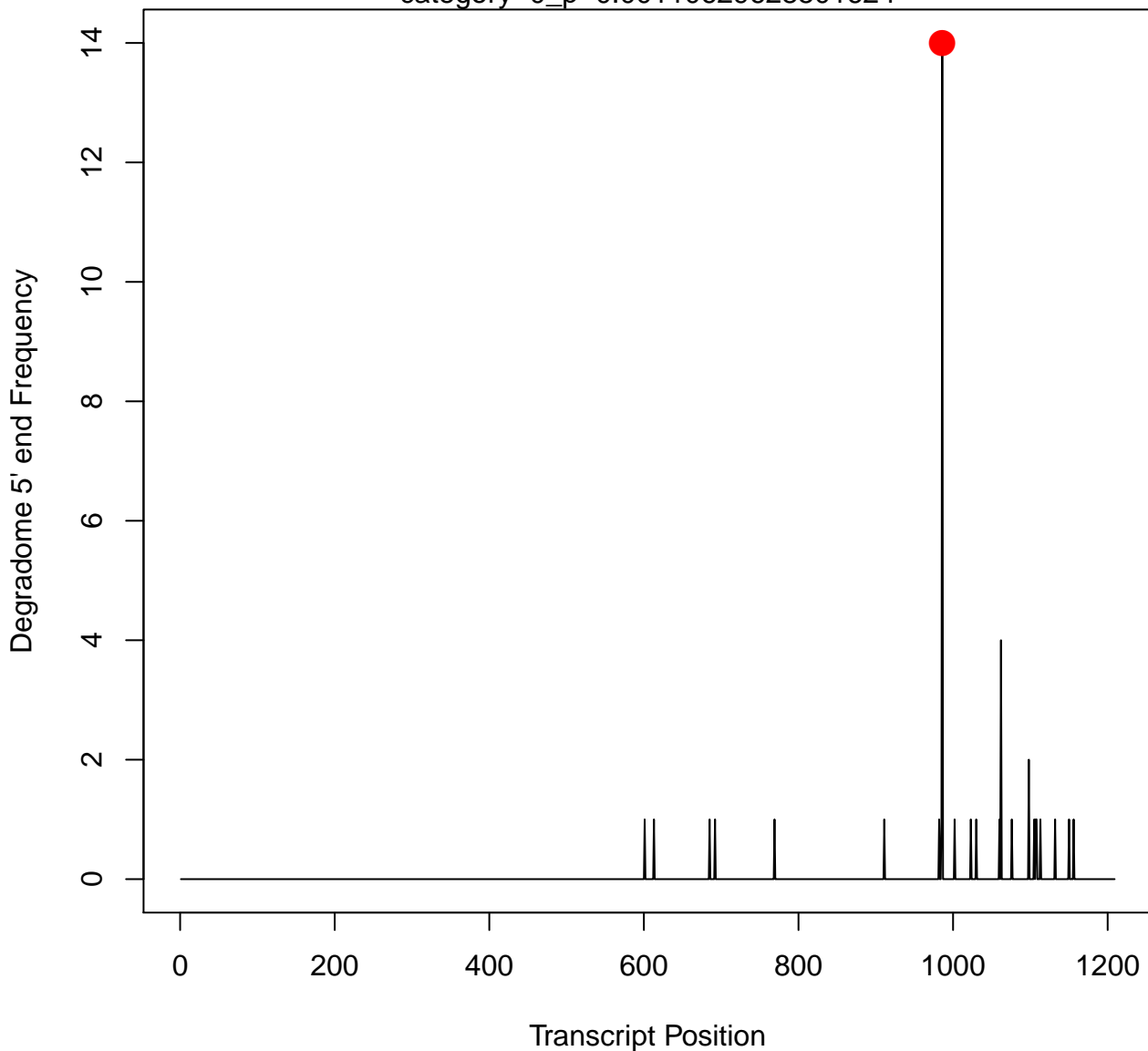

Supplement: Supplementary file 2 [file Data_Sheet_8.ZIP › GSM2230747.plot/Lsa-miR169i_Lsat_1_v5_gn_7_34841.1_986_TPlot.pdf]

**T=Lsat\_1\_v5\_gn\_1\_27961.1\_Q=Lsa-miR171a\_S=2558**

category=2\_p=0.977227877137183

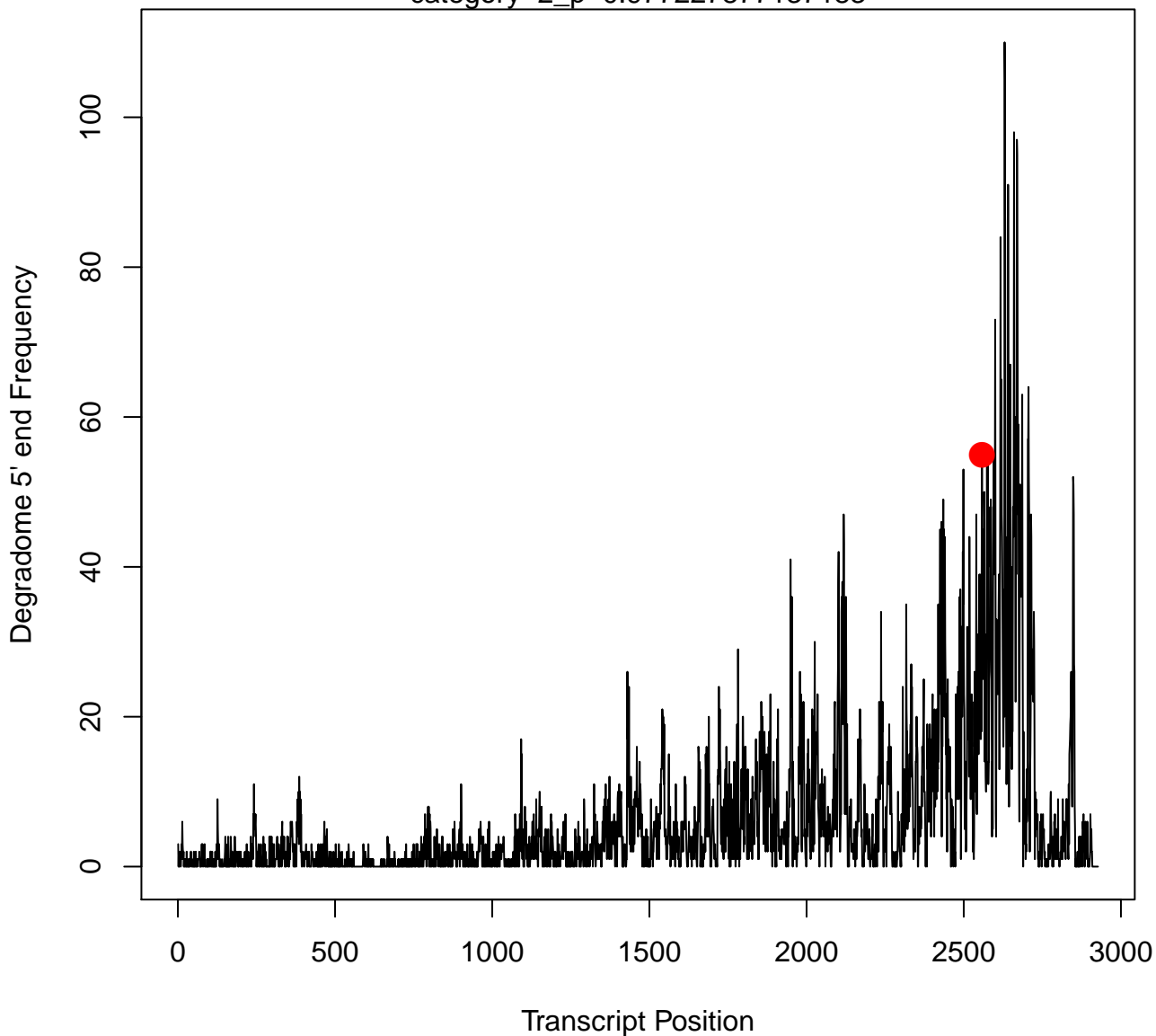

Supplement: Supplementary file 2 [file Data_Sheet_8.ZIP › GSM2230747.plot/Lsa-miR171a_Lsat_1_v5_gn_1_27961.1_2558_TPlot.pdf]

**T=Lsat\_1\_v5\_gn\_2\_104501.1\_Q=Lsa-miR171a\_S=3690**

category=2\_p=0.224515100030538

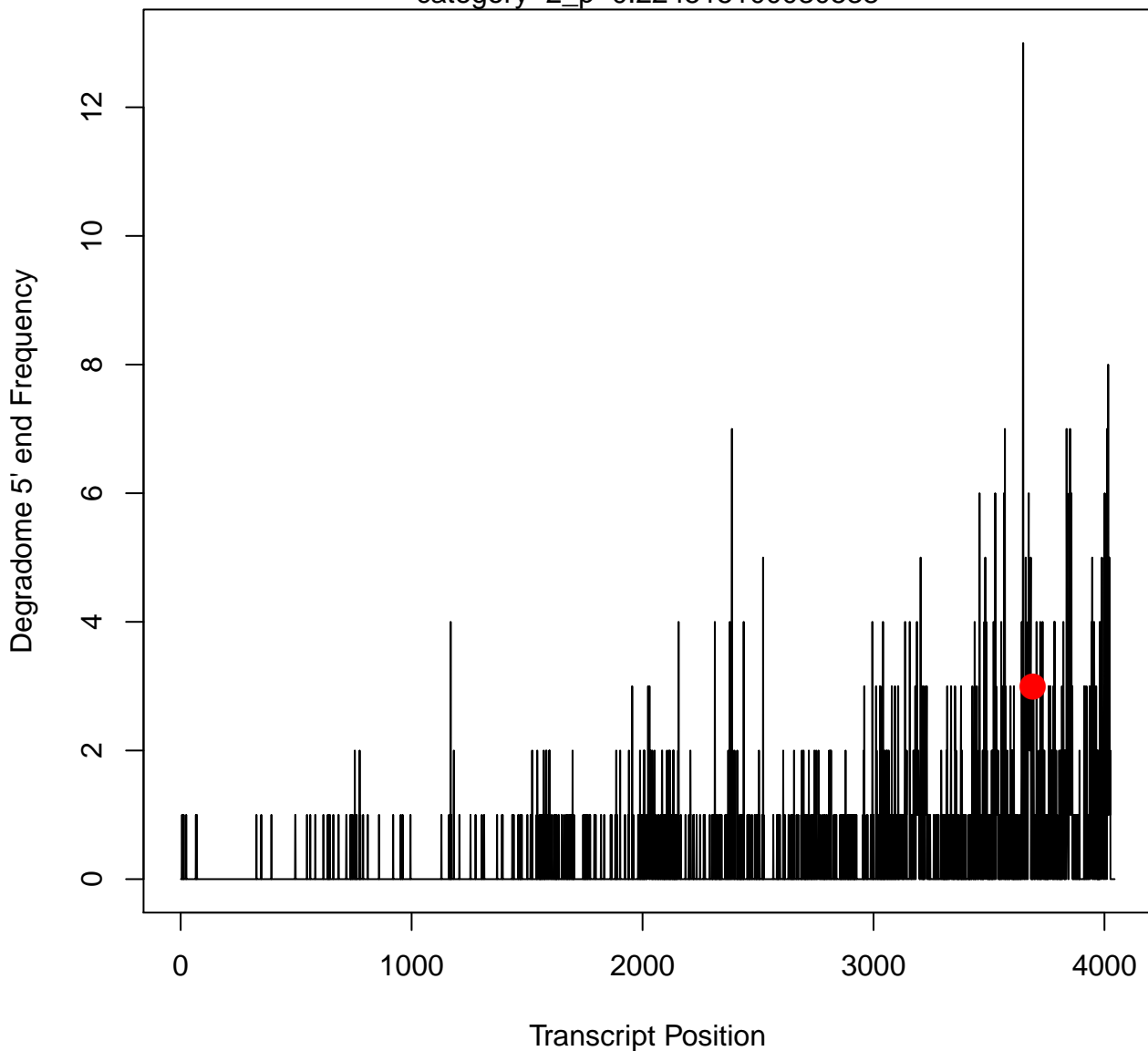

Supplement: Supplementary file 2 [file Data_Sheet_8.ZIP › GSM2230747.plot/Lsa-miR171a_Lsat_1_v5_gn_2_104501.1_3690_TPlot.pdf]

T=Lsat\_1\_v5\_gn\_3\_128721.1\_Q=Lsa-miR171a\_S=47

category=2\_p=0.0615884673055831

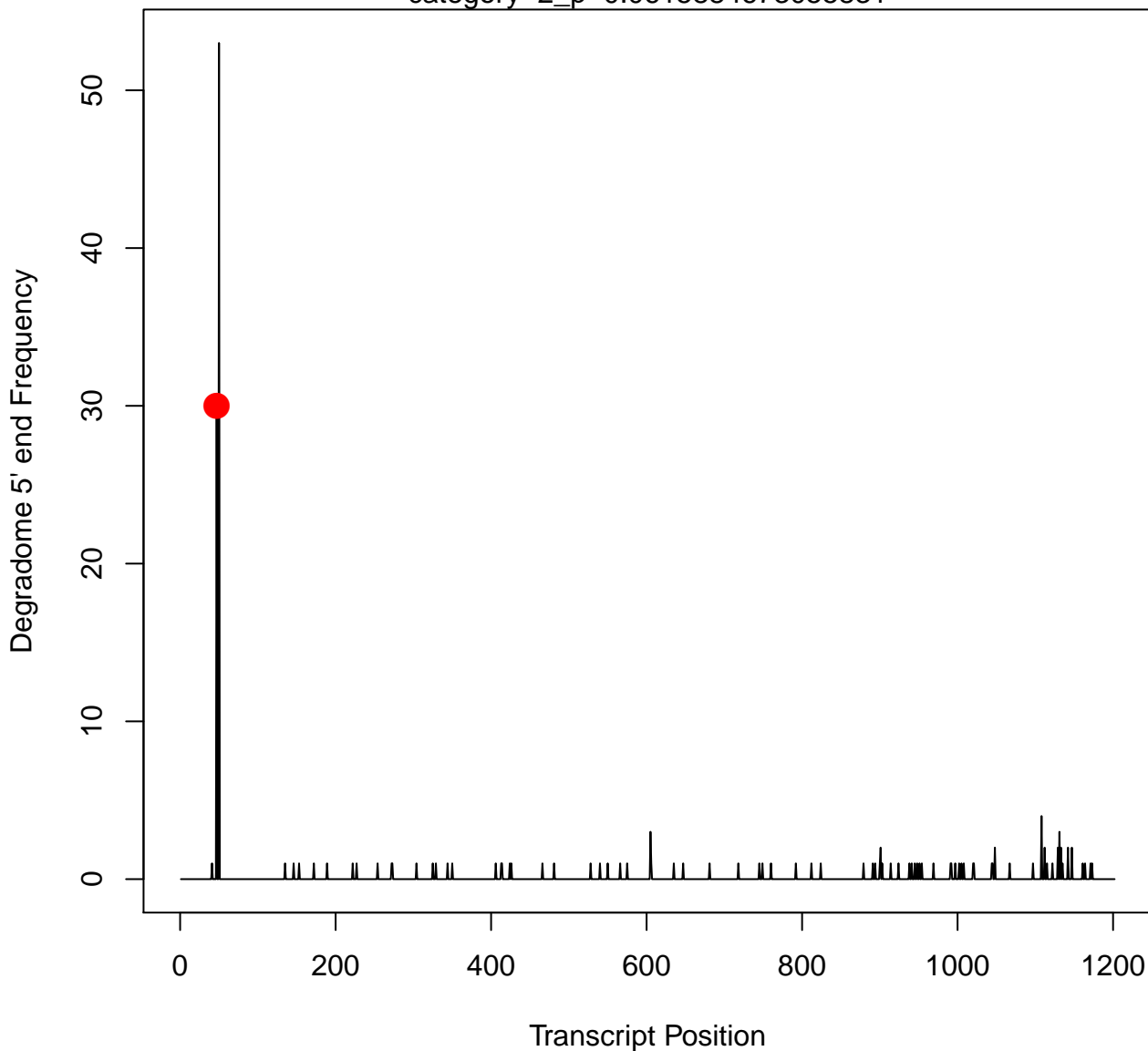

Supplement: Supplementary file 2 [file Data_Sheet_8.ZIP › GSM2230747.plot/Lsa-miR171a_Lsat_1_v5_gn_3_128721.1_47_TPlot.pdf]

**T=Lsat\_1\_v5\_gn\_3\_78621.1\_Q=Lsa-miR171a\_S=732**

category=2\_p=0.0312835643520768

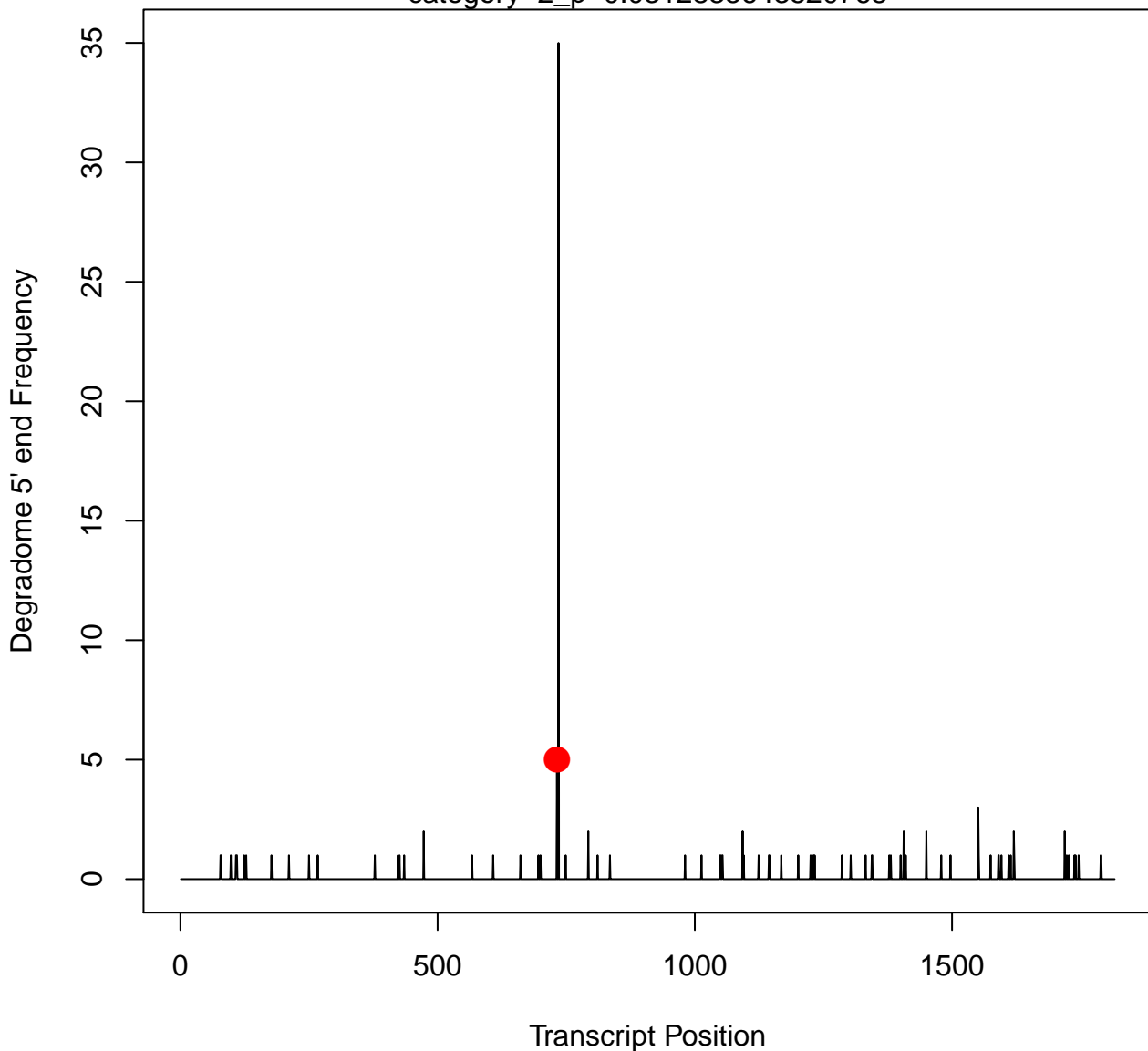

Supplement: Supplementary file 2 [file Data_Sheet_8.ZIP › GSM2230747.plot/Lsa-miR171a_Lsat_1_v5_gn_3_78621.1_732_TPlot.pdf]

**T=Lsat\_1\_v5\_gn\_5\_174381.1\_Q=Lsa-miR171a\_S=873**

category=2\_p=0.272276026438281

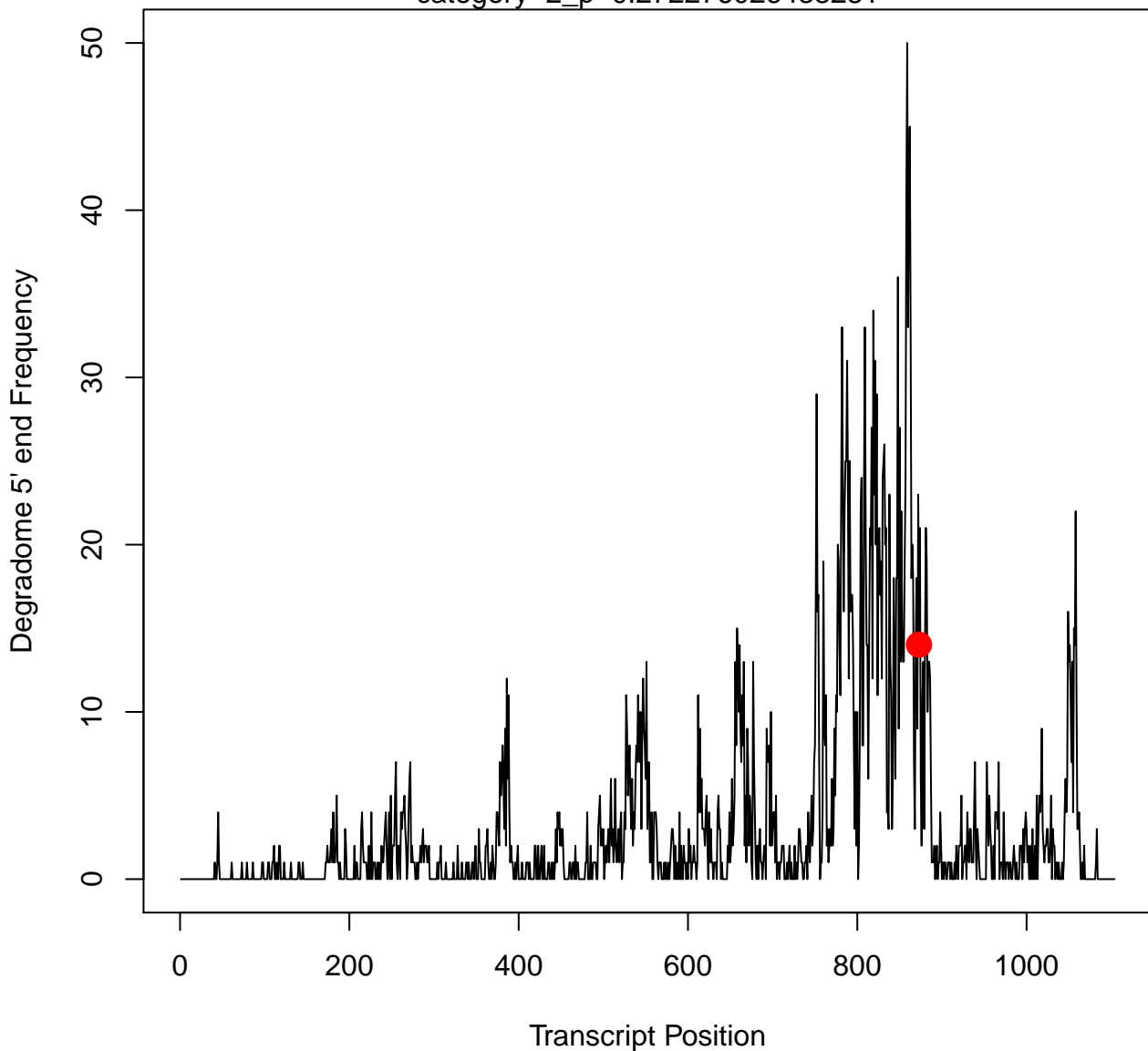

Supplement: Supplementary file 2 [file Data_Sheet_8.ZIP › GSM2230747.plot/Lsa-miR171a_Lsat_1_v5_gn_5_174381.1_873_TPlot.pdf]

**T=Lsat\_1\_v5\_gn\_7\_45260.1\_Q=Lsa-miR171a\_S=768**

category=2\_p=0.94796564871546

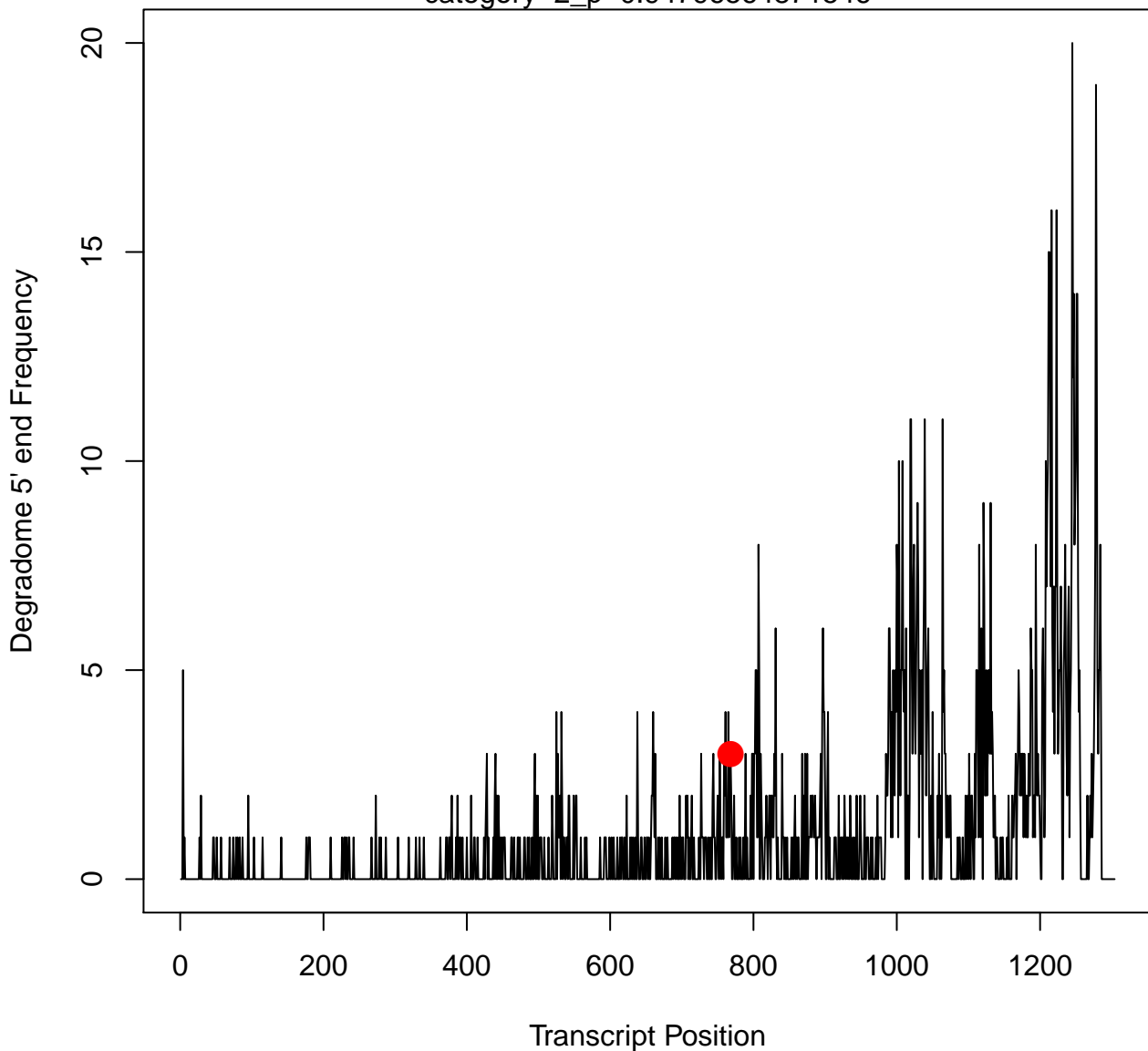

Supplement: Supplementary file 2 [file Data_Sheet_8.ZIP › GSM2230747.plot/Lsa-miR171a_Lsat_1_v5_gn_7_45260.1_768_TPlot.pdf]

**T=Lsat\_1\_v5\_gn\_1\_27961.1\_Q=Lsa-miR171b\_S=2561**

category=2\_p=0.589317014801513

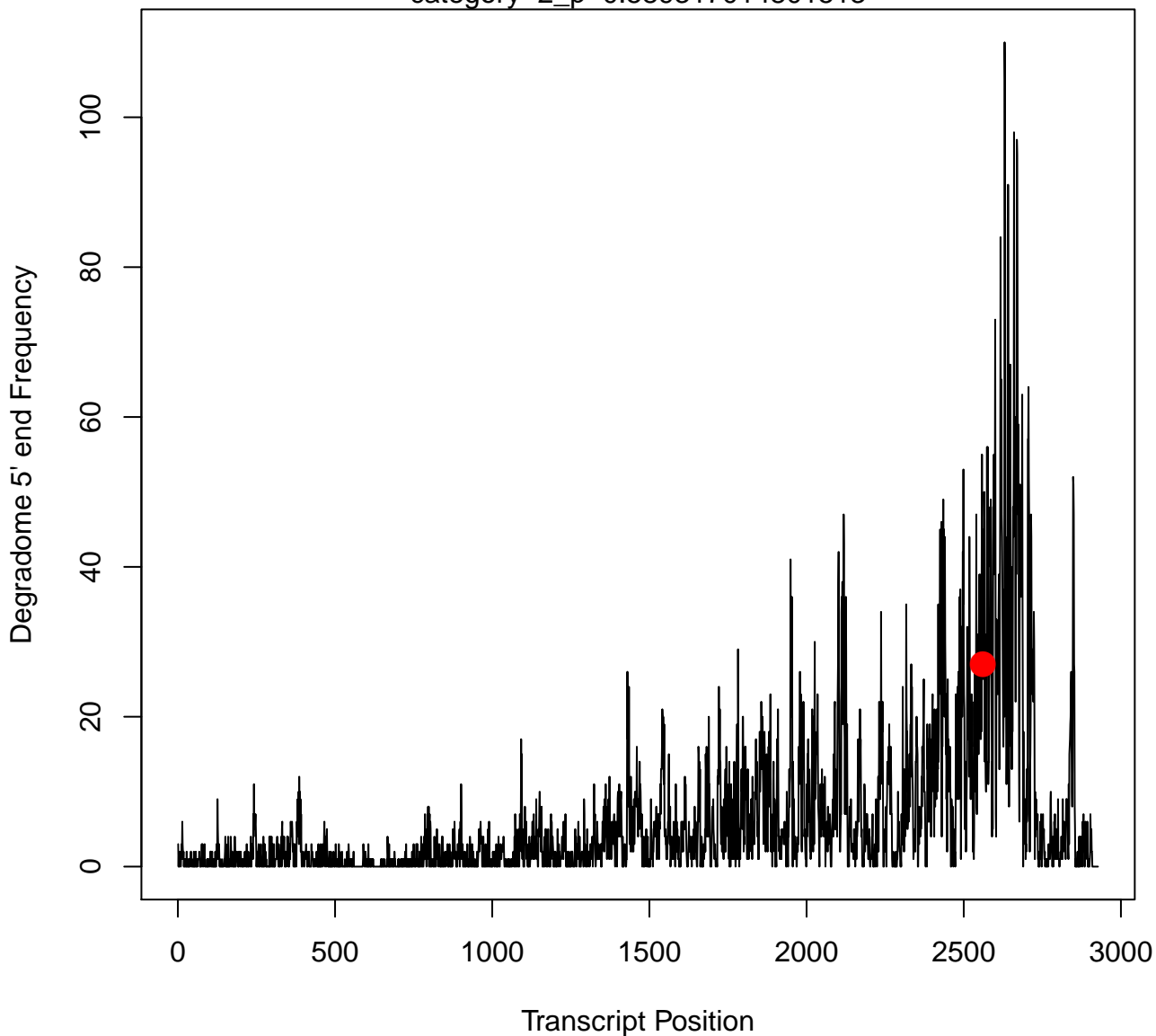

Supplement: Supplementary file 2 [file Data_Sheet_8.ZIP › GSM2230747.plot/Lsa-miR171b_Lsat_1_v5_gn_1_27961.1_2561_TPlot.pdf]

**T=Lsat\_1\_v5\_gn\_1\_4460.1\_Q=Lsa-miR171b\_S=620**

category=2\_p=0.992974451398807

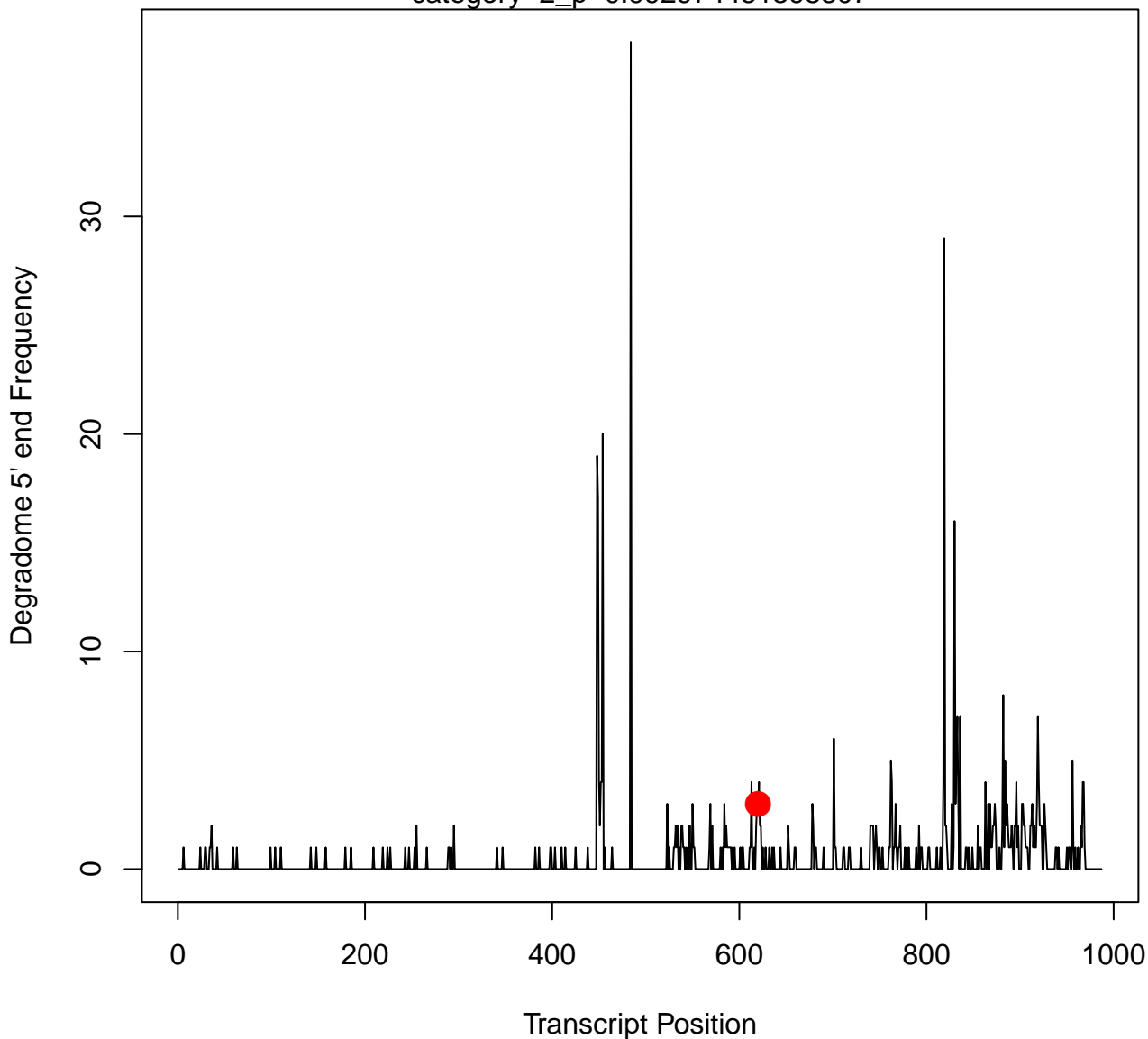

Supplement: Supplementary file 2 [file Data_Sheet_8.ZIP › GSM2230747.plot/Lsa-miR171b_Lsat_1_v5_gn_1_4460.1_620_TPlot.pdf]

**T=Lsat\_1\_v5\_gn\_2\_104501.1\_Q=Lsa-miR171b\_S=3693**

category=2\_p=0.921343710073031

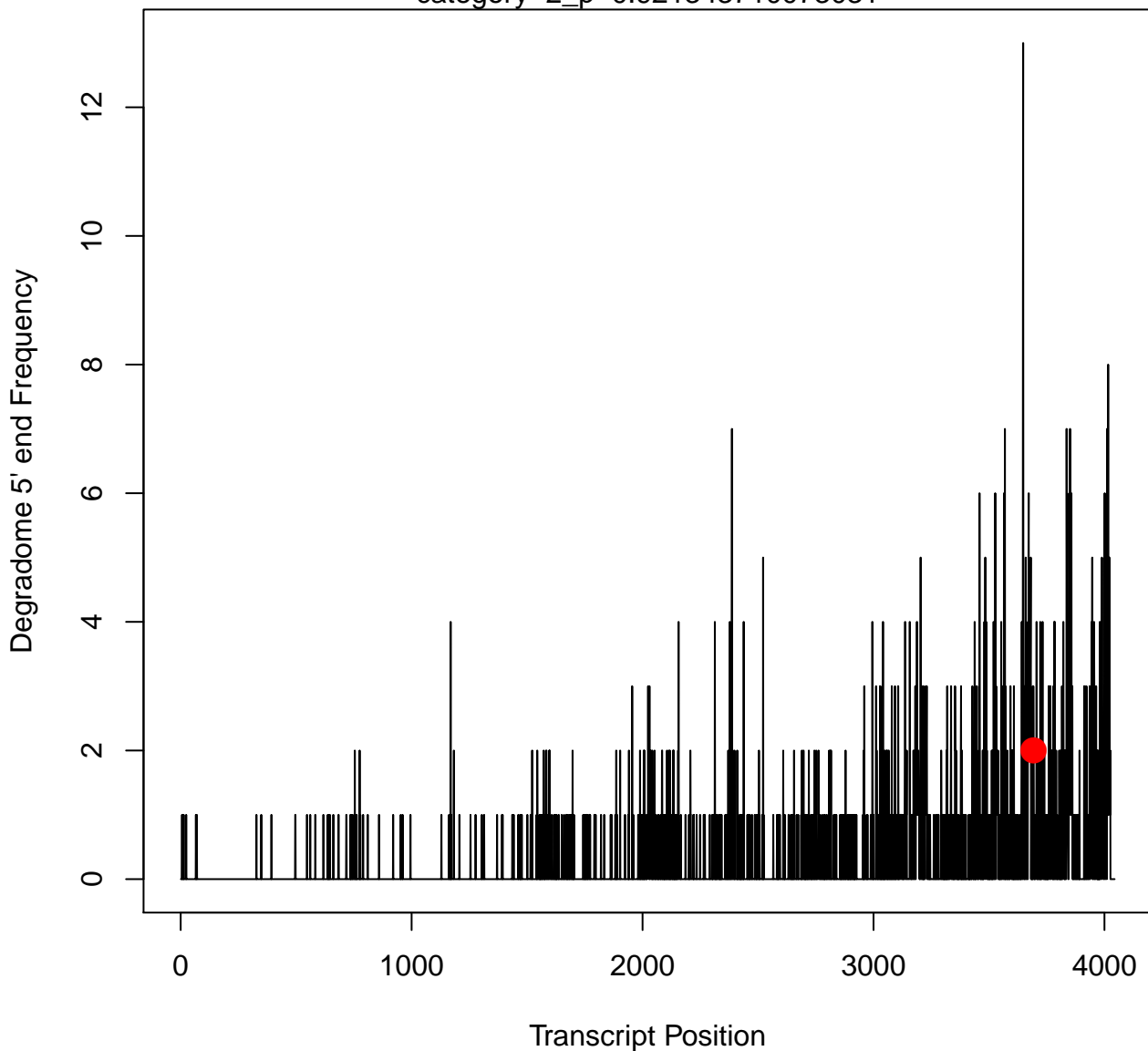

Supplement: Supplementary file 2 [file Data_Sheet_8.ZIP › GSM2230747.plot/Lsa-miR171b_Lsat_1_v5_gn_2_104501.1_3693_TPlot.pdf]

**T=Lsat\_1\_v5\_gn\_3\_128721.1\_Q=Lsa-miR171b\_S=50**

category=0\_p=0.000737666911525325

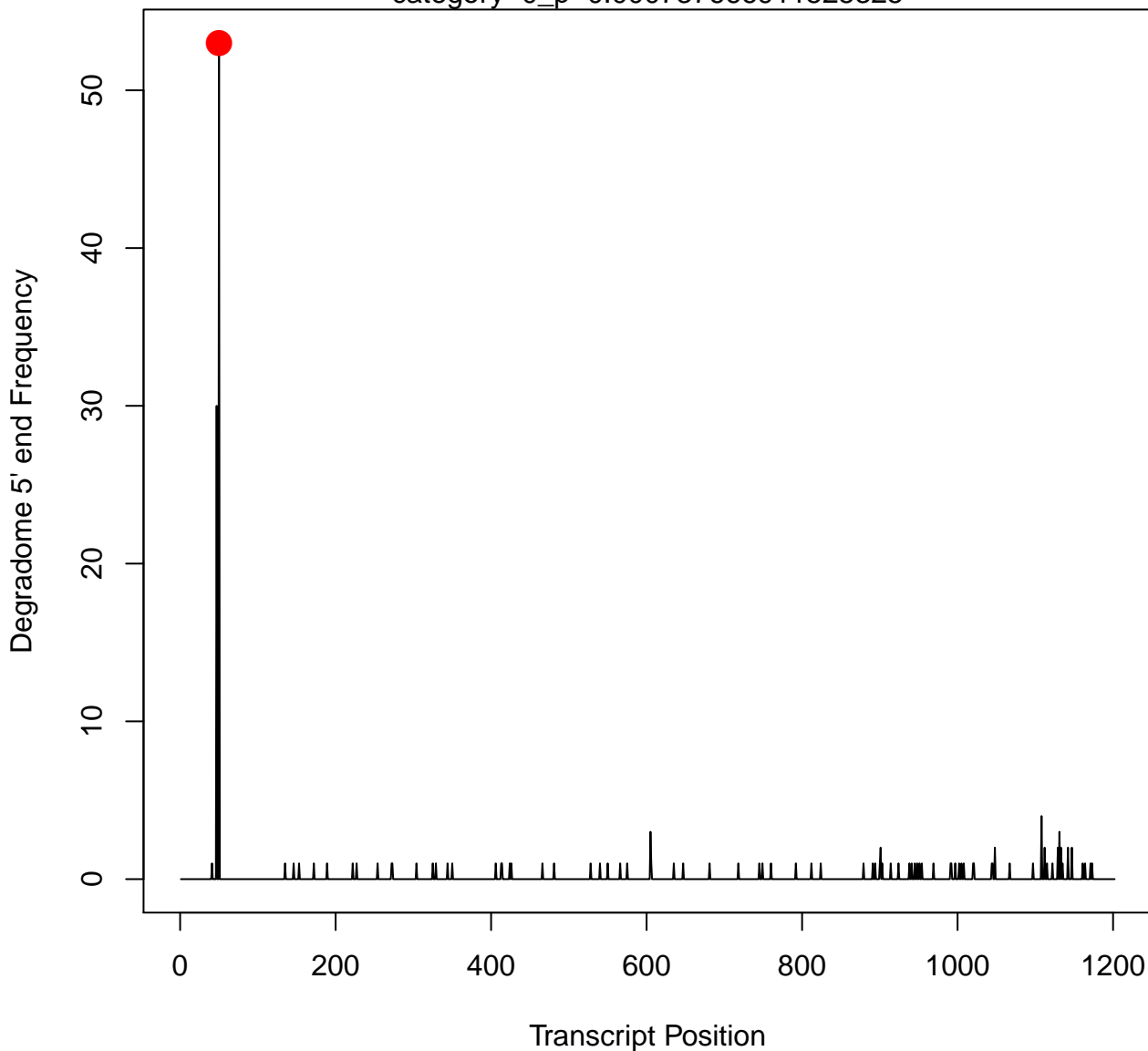

Supplement: Supplementary file 2 [file Data_Sheet_8.ZIP › GSM2230747.plot/Lsa-miR171b_Lsat_1_v5_gn_3_128721.1_50_TPlot.pdf]

**T=Lsat\_1\_v5\_gn\_3\_29621.1\_Q=Lsa-miR171b\_S=996**

category=2\_p=0.988683028578759

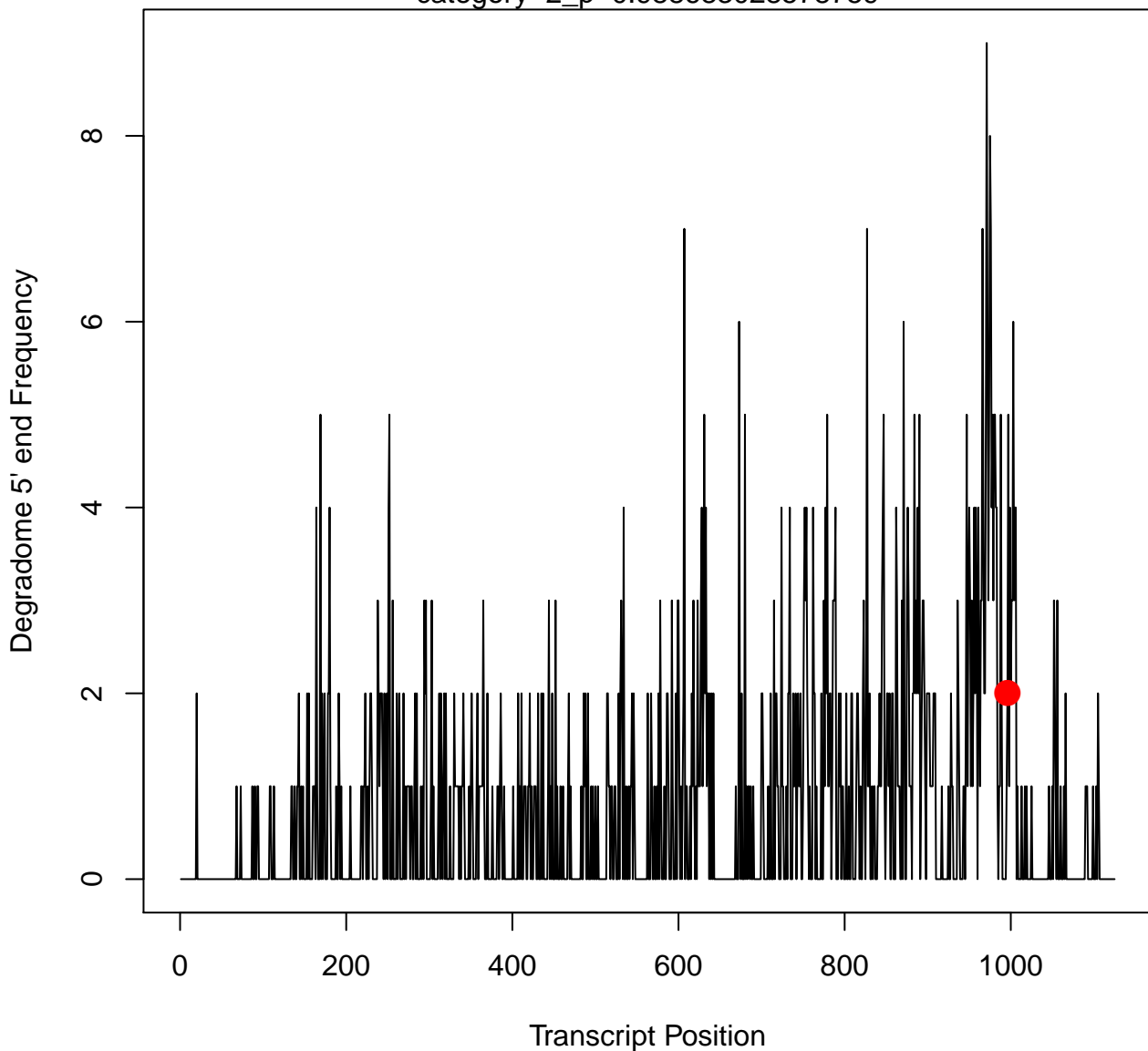

Supplement: Supplementary file 2 [file Data_Sheet_8.ZIP › GSM2230747.plot/Lsa-miR171b_Lsat_1_v5_gn_3_29621.1_996_TPlot.pdf]

**T=Lsat\_1\_v5\_gn\_3\_681.1\_Q=Lsa-miR171b\_S=716**

category=2\_p=0.0312835643520768

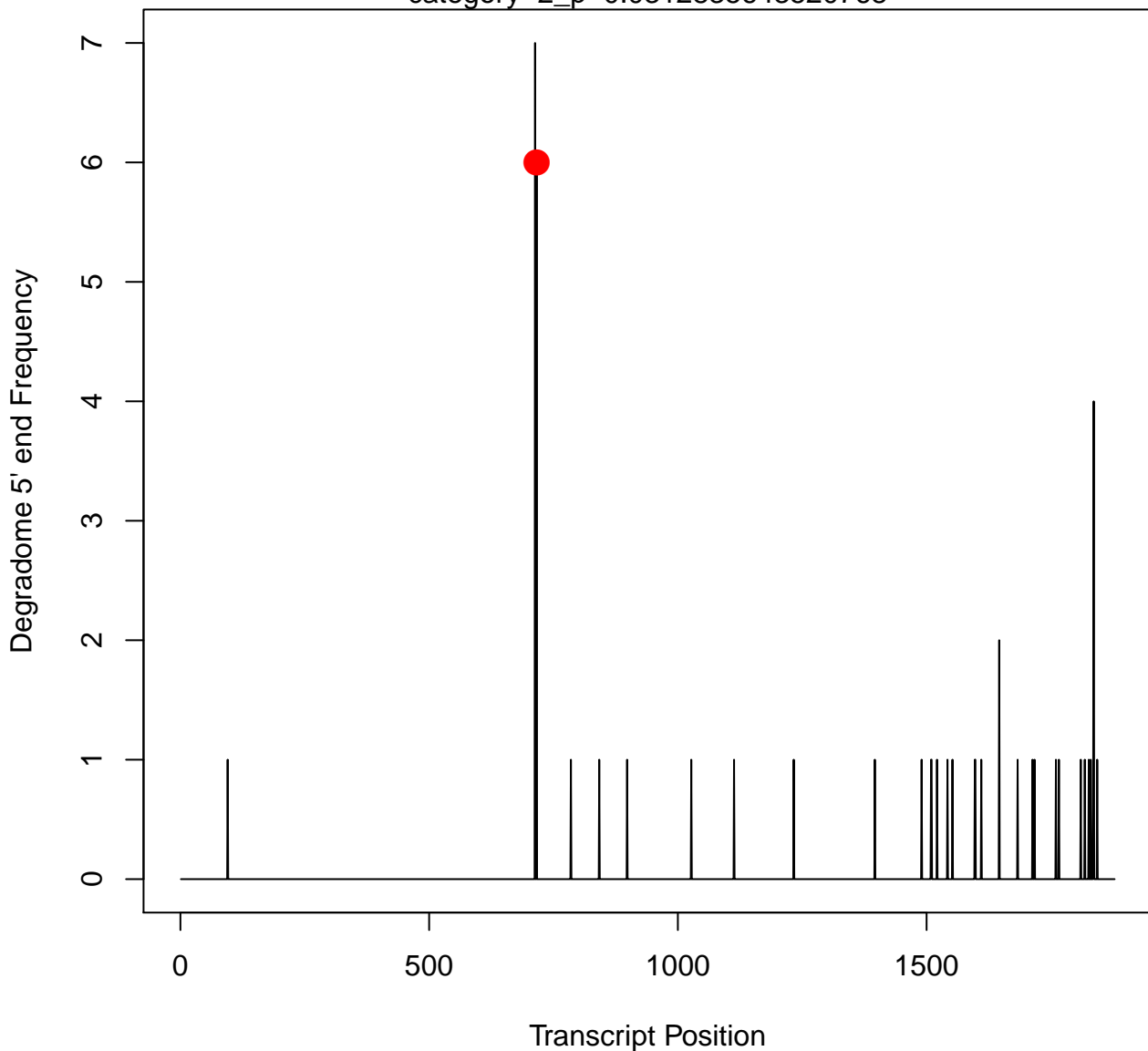

Supplement: Supplementary file 2 [file Data_Sheet_8.ZIP › GSM2230747.plot/Lsa-miR171b_Lsat_1_v5_gn_3_681.1_716_TPlot.pdf]

**T=Lsat\_1\_v5\_gn\_3\_78621.1\_Q=Lsa-miR171b\_S=735**

category=0\_p=0.00110629628501624

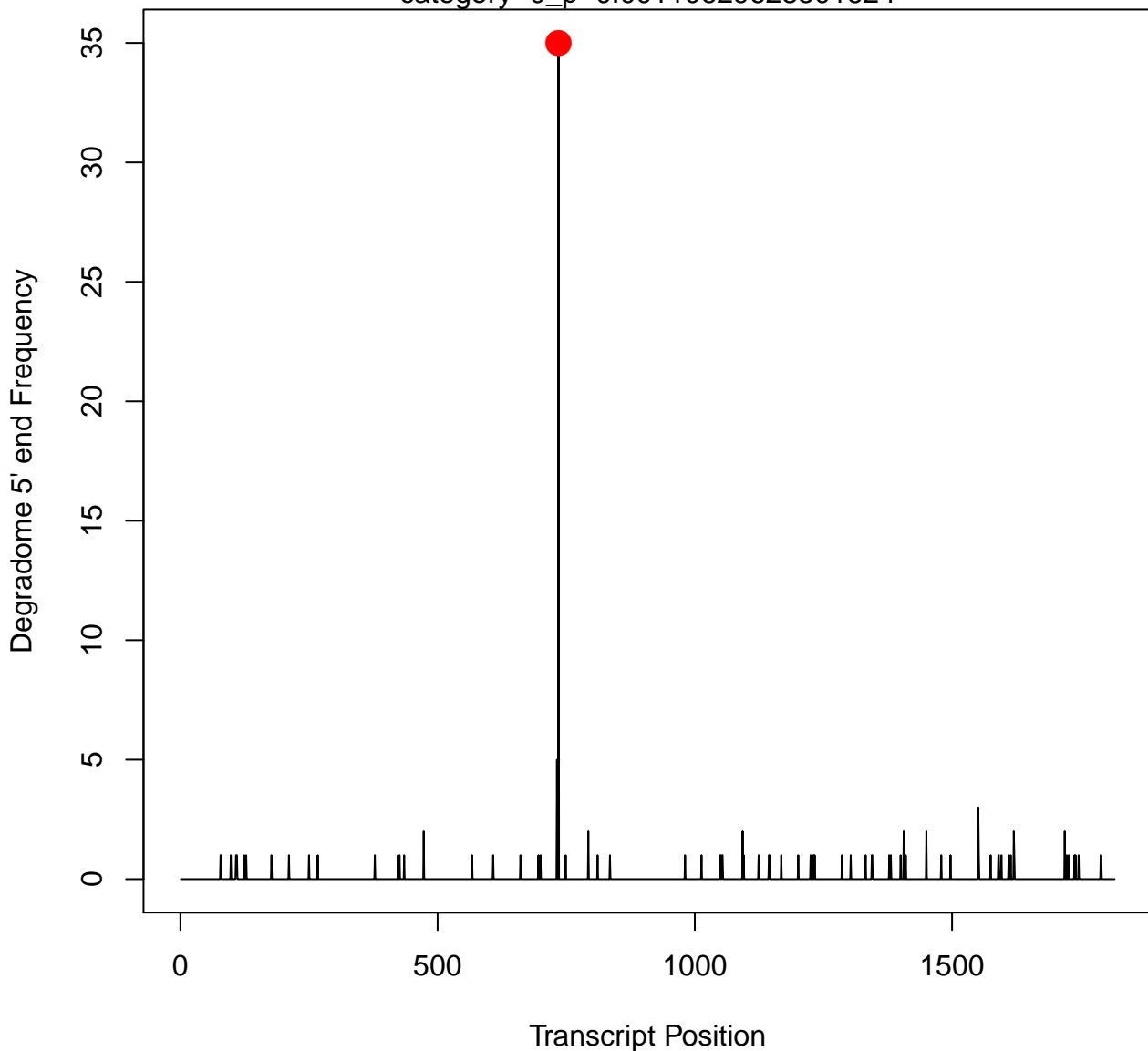

Supplement: Supplementary file 2 [file Data_Sheet_8.ZIP › GSM2230747.plot/Lsa-miR171b_Lsat_1_v5_gn_3_78621.1_735_TPlot.pdf]

**T=Lsat\_1\_v5\_gn\_4\_153400.1\_Q=Lsa-miR171b\_S=648**

category=2\_p=0.173619597637671

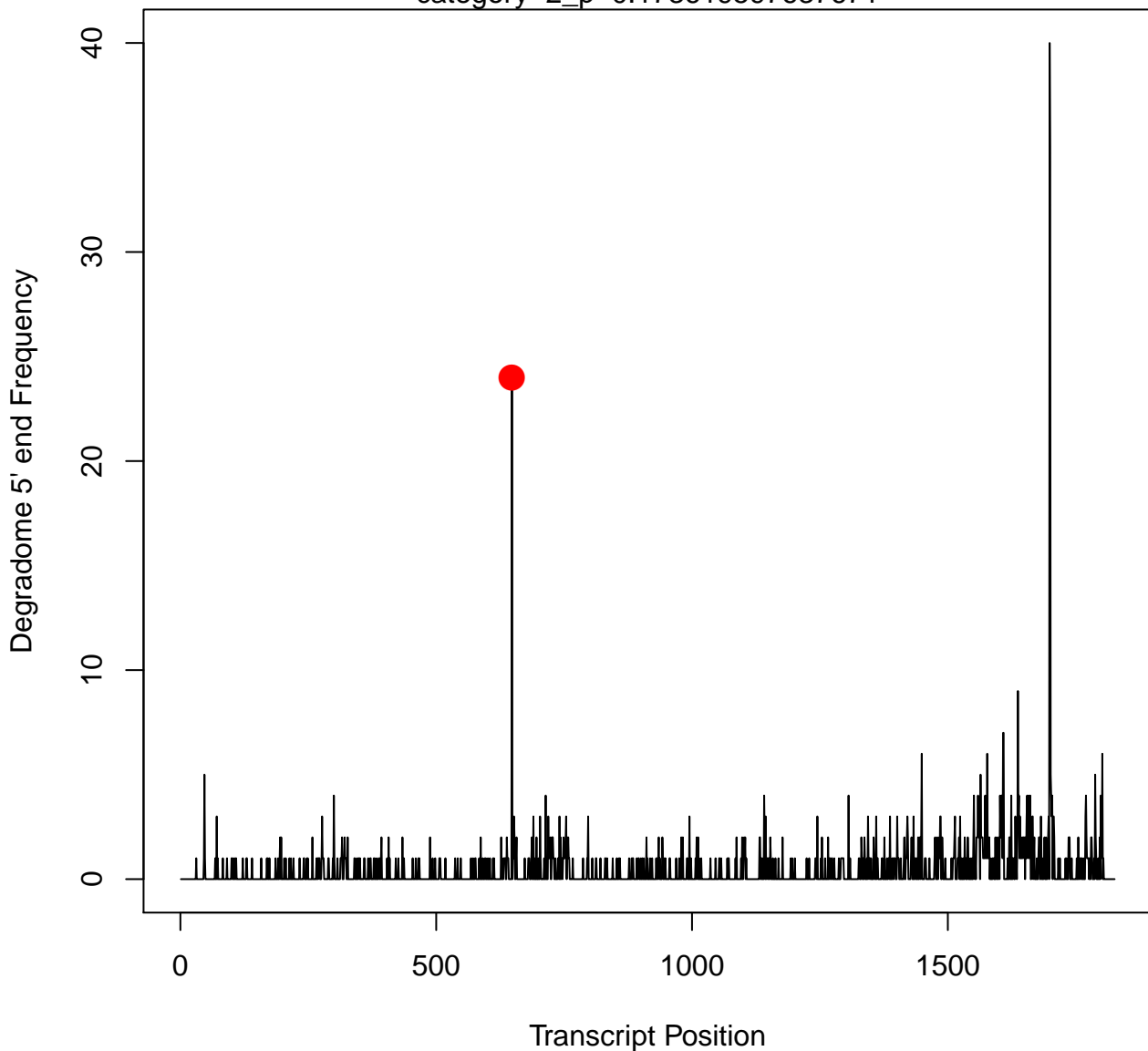

Supplement: Supplementary file 2 [file Data_Sheet_8.ZIP › GSM2230747.plot/Lsa-miR171b_Lsat_1_v5_gn_4_153400.1_648_TPlot.pdf]

**T=Lsat\_1\_v5\_gn\_6\_3180.1\_Q=Lsa-miR171b\_S=1761**

category=0\_p=0.00184314711837774

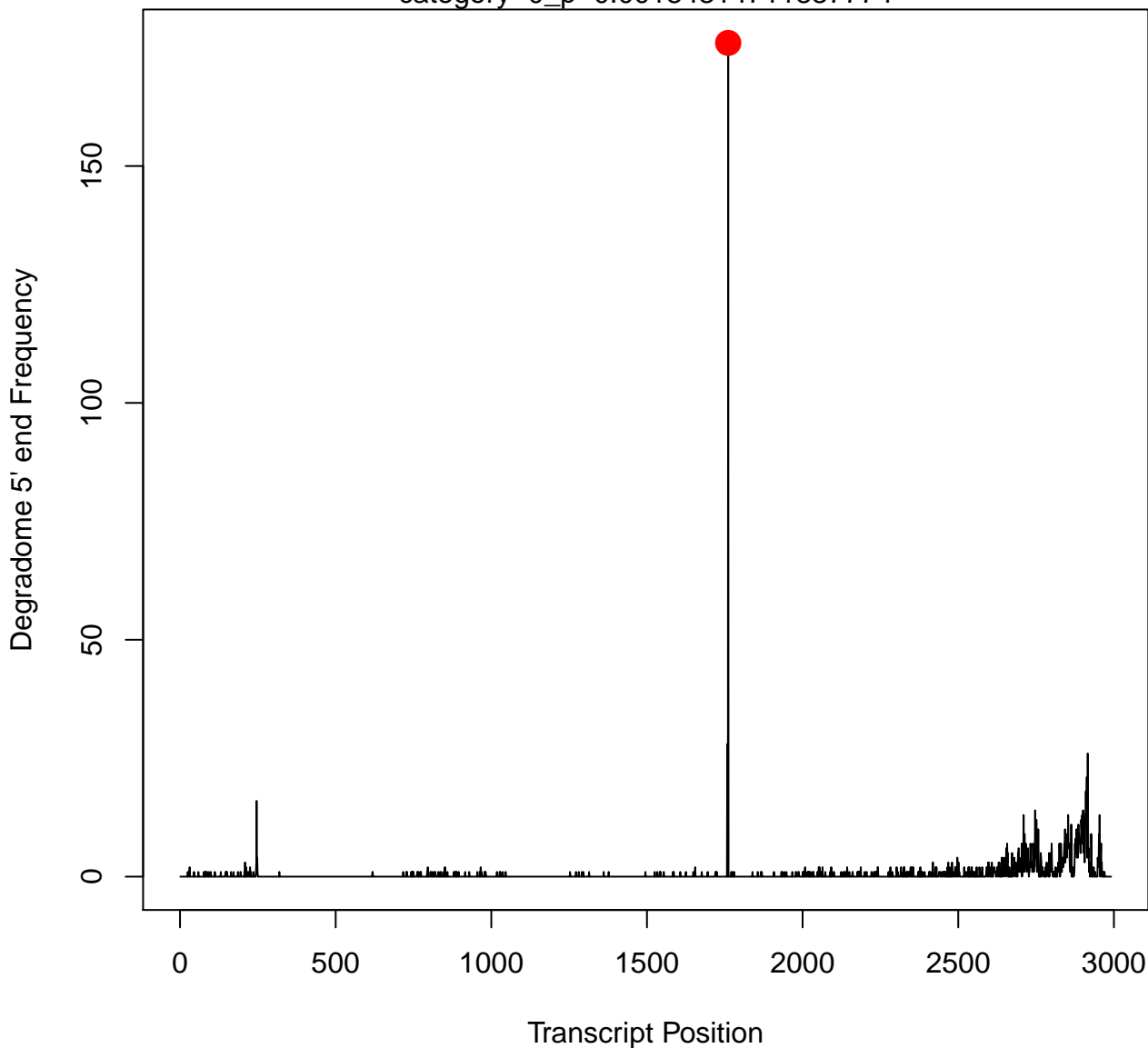

Supplement: Supplementary file 2 [file Data_Sheet_8.ZIP › GSM2230747.plot/Lsa-miR171b_Lsat_1_v5_gn_6_3180.1_1761_TPlot.pdf]

**T=Lsat\_1\_v5\_gn\_7\_9261.1\_Q=Lsa-miR171b\_S=1167**

category=0\_p=0.00147478967057835

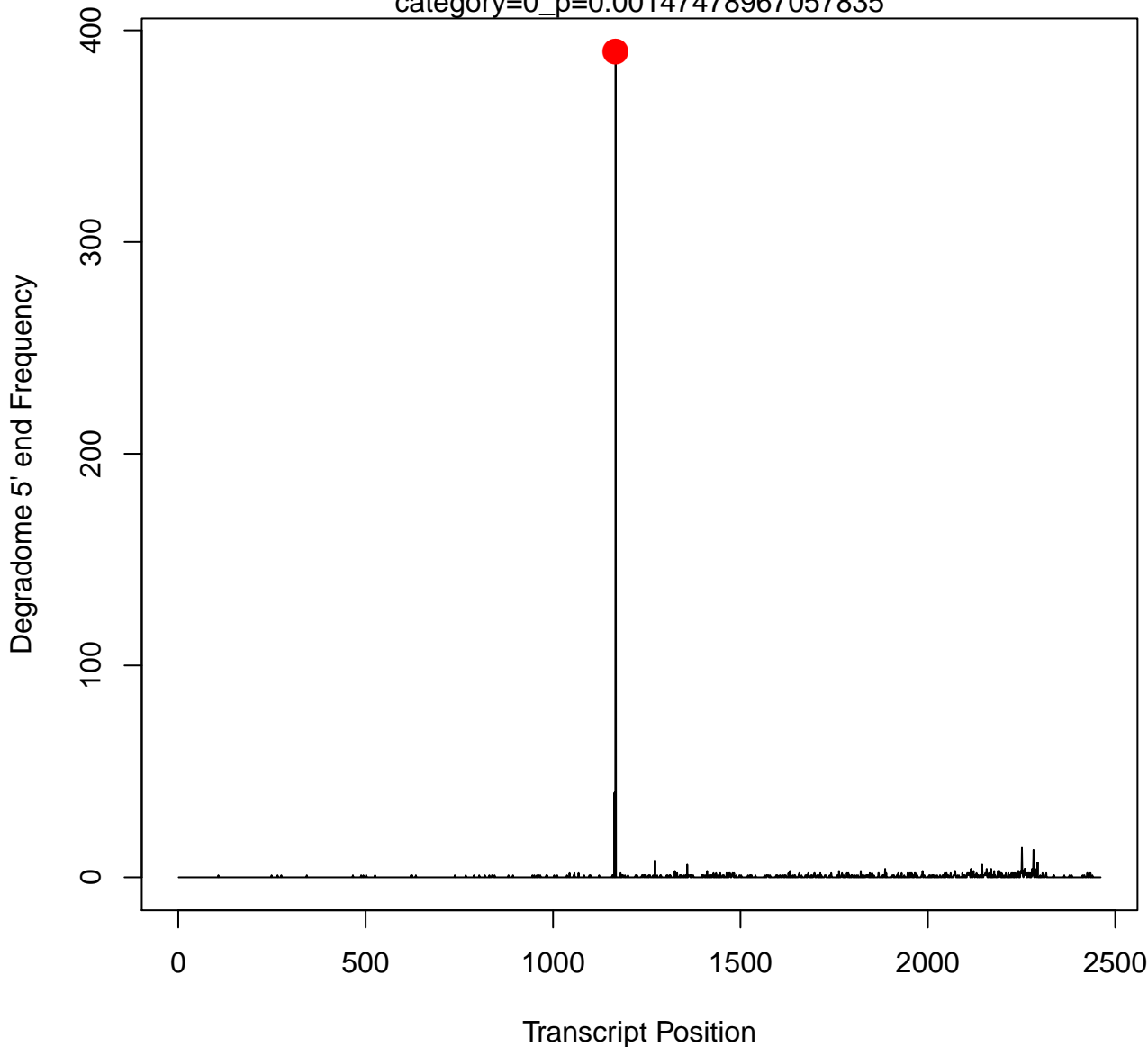

Supplement: Supplementary file 2 [file Data_Sheet_8.ZIP › GSM2230747.plot/Lsa-miR171b_Lsat_1_v5_gn_7_9261.1_1167_TPlot.pdf]

**T=Lsat\_1\_v5\_gn\_3\_45080.1\_Q=Lsa-miR171c\_S=2703**

category=2\_p=0.995497708627651

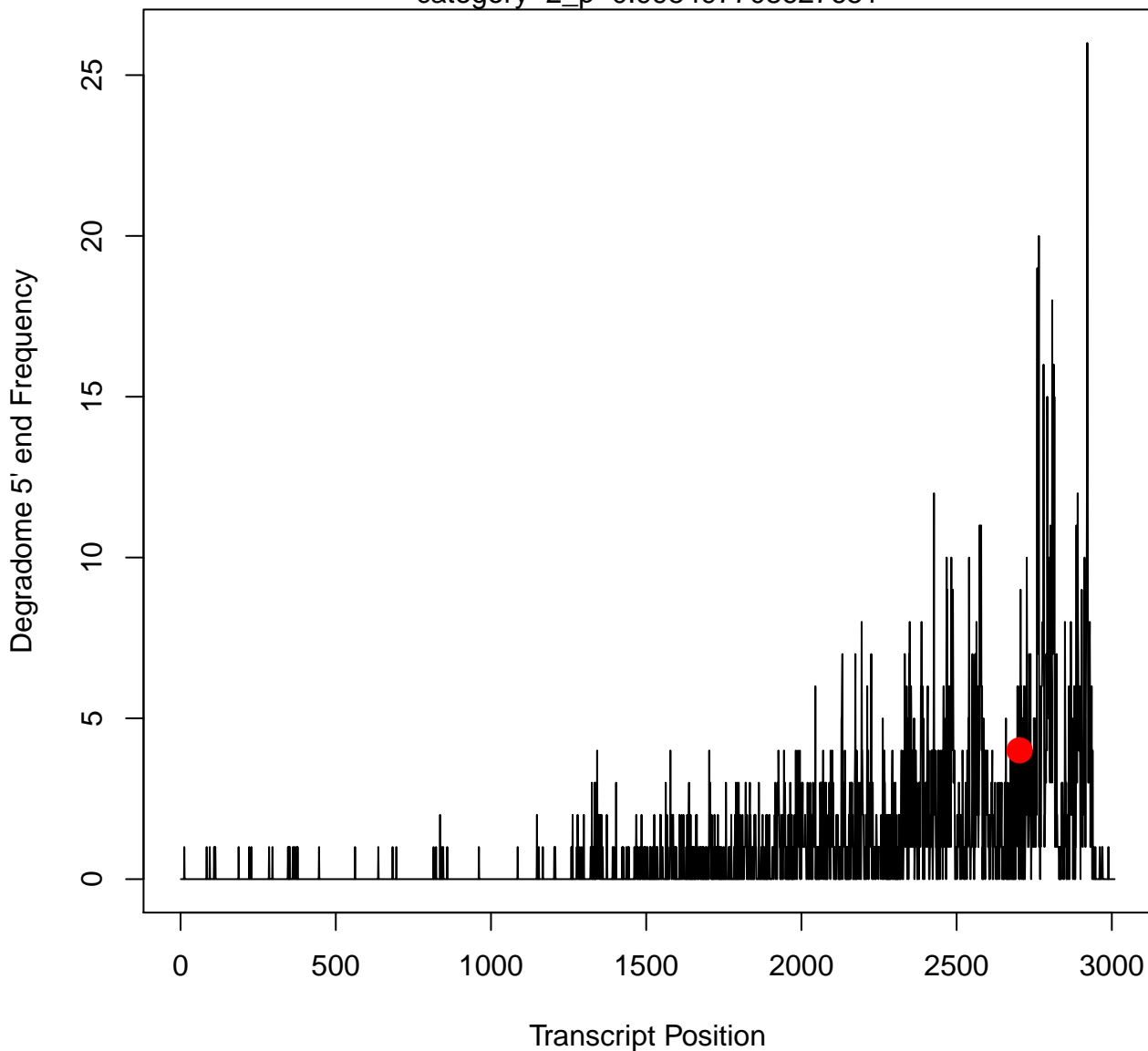

Supplement: Supplementary file 2 [file Data_Sheet_8.ZIP › GSM2230747.plot/Lsa-miR171c_Lsat_1_v5_gn_3_45080.1_2703_TPlot.pdf]

**T=Lsat\_1\_v5\_gn\_3\_63880.1\_Q=Lsa-miR171c\_S=577**

category=2\_p=0.98444881324185

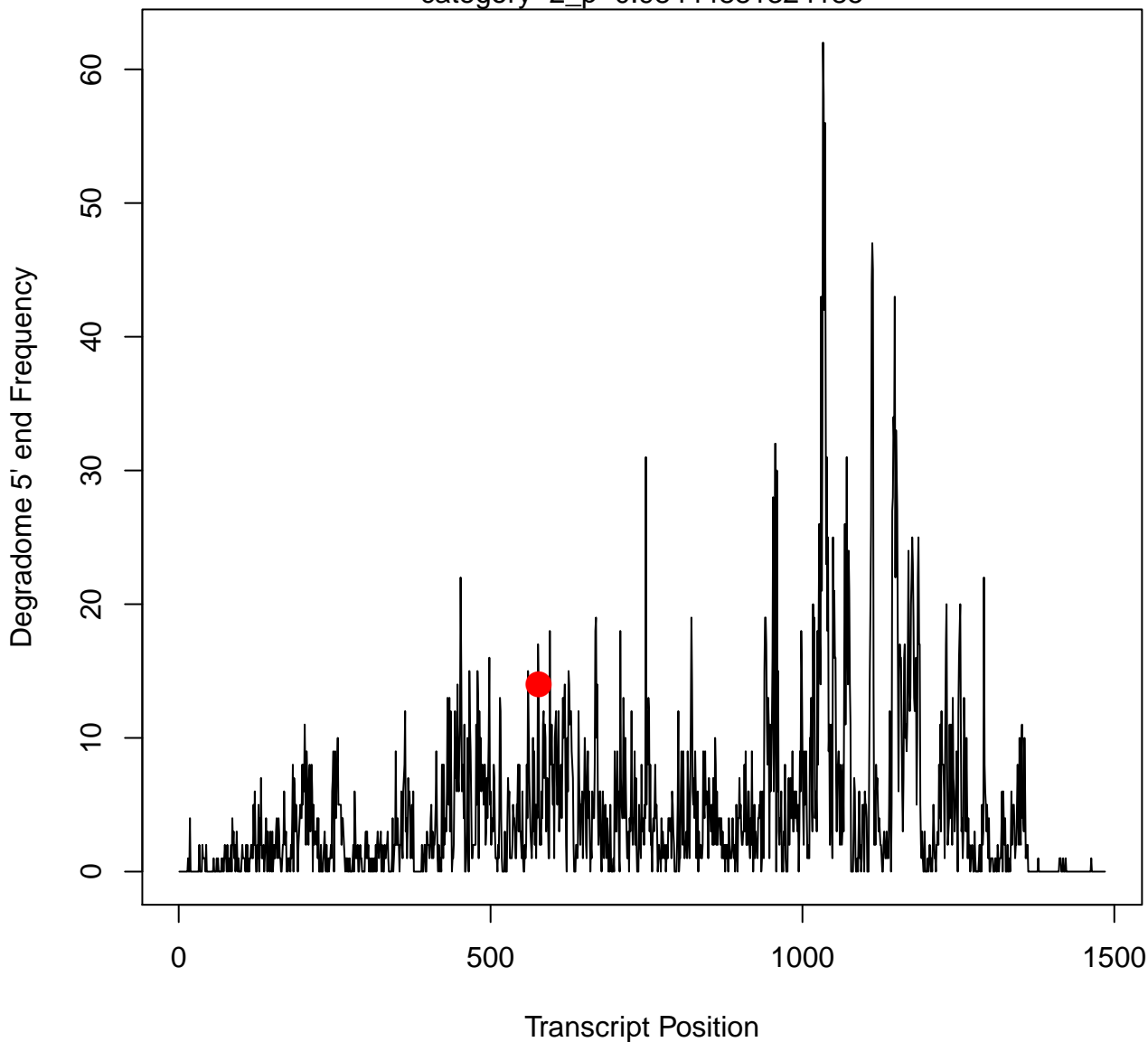

Supplement: Supplementary file 2 [file Data_Sheet_8.ZIP › GSM2230747.plot/Lsa-miR171c_Lsat_1_v5_gn_3_63880.1_577_TPlot.pdf]

**T=Lsat\_1\_v5\_gn\_3\_6981.1\_Q=Lsa-miR171c\_S=4441**

category=2\_p=0.928497131914087

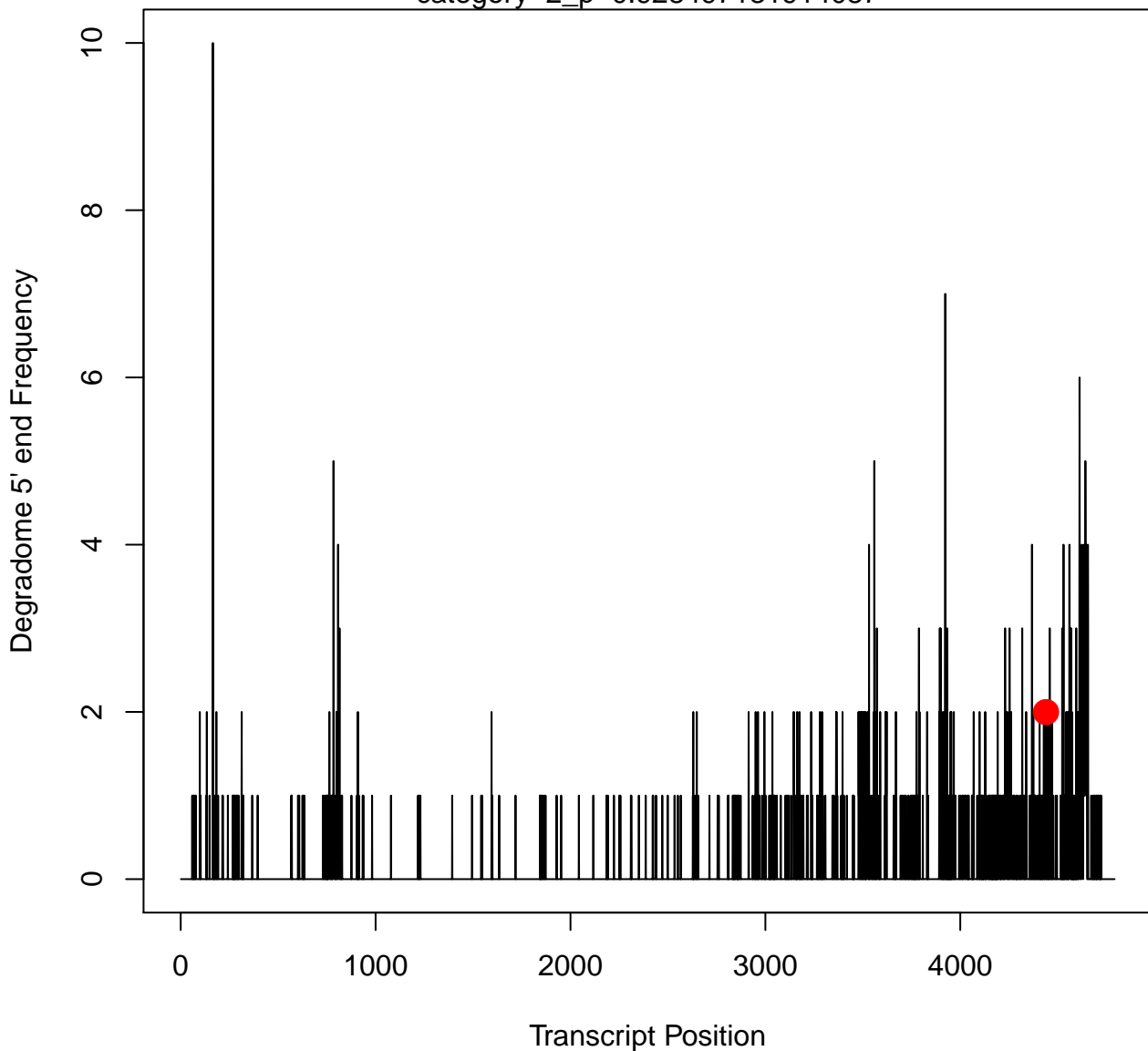

Supplement: Supplementary file 2 [file Data_Sheet_8.ZIP › GSM2230747.plot/Lsa-miR171c_Lsat_1_v5_gn_3_6981.1_4441_TPlot.pdf]

**T=Lsat\_1\_v5\_gn\_4\_149801.1\_Q=Lsa-miR171c\_S=210**

category=2\_p=0.990647876603392

Degradome 5' end Frequency

5  
4  
3  
2  
1  
0

0

200

400

600

Transcript Position

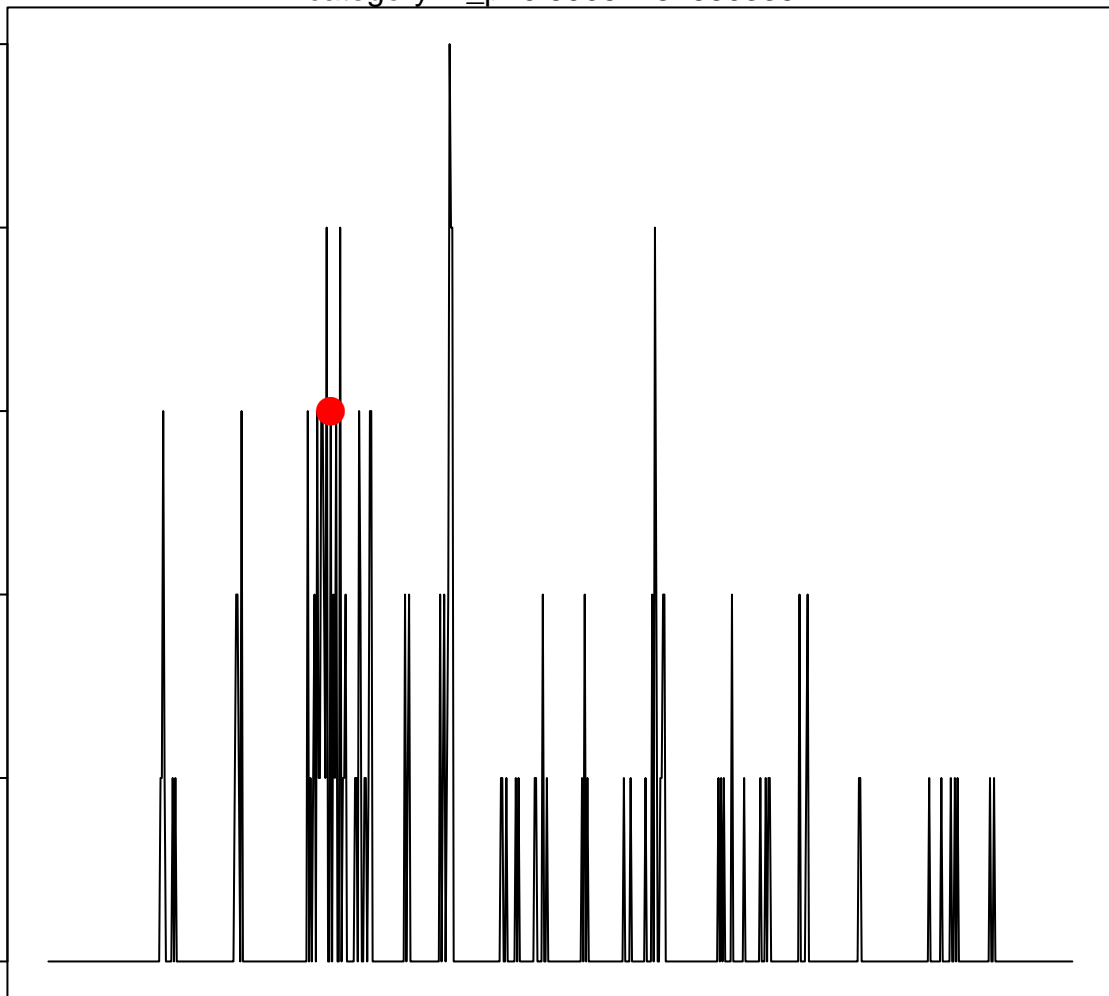

Supplement: Supplementary file 2 [file Data_Sheet_8.ZIP › GSM2230747.plot/Lsa-miR171c_Lsat_1_v5_gn_4_149801.1_210_TPlot.pdf]

**T=Lsat\_1\_v5\_gn\_4\_178061.1\_Q=Lsa-miR171c\_S=3889**

category=2\_p=0.982893012726597

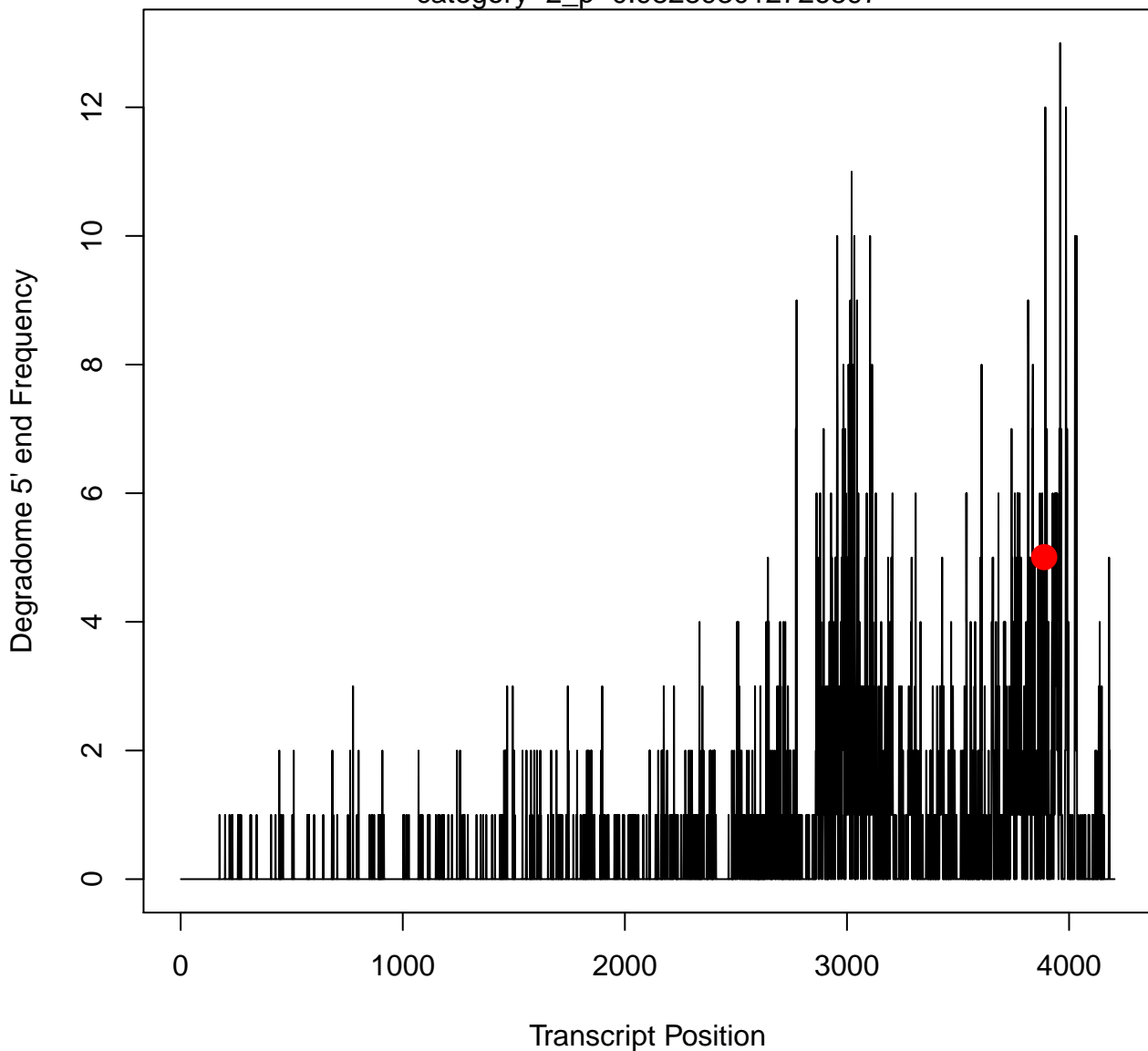

Supplement: Supplementary file 2 [file Data_Sheet_8.ZIP › GSM2230747.plot/Lsa-miR171c_Lsat_1_v5_gn_4_178061.1_3889_TPlot.pdf]

**T=Lsat\_1\_v5\_gn\_5\_53581.1\_Q=Lsa-miR171c\_S=1236**

category=2\_p=0.940911431101103

Degradome 5' end Frequency

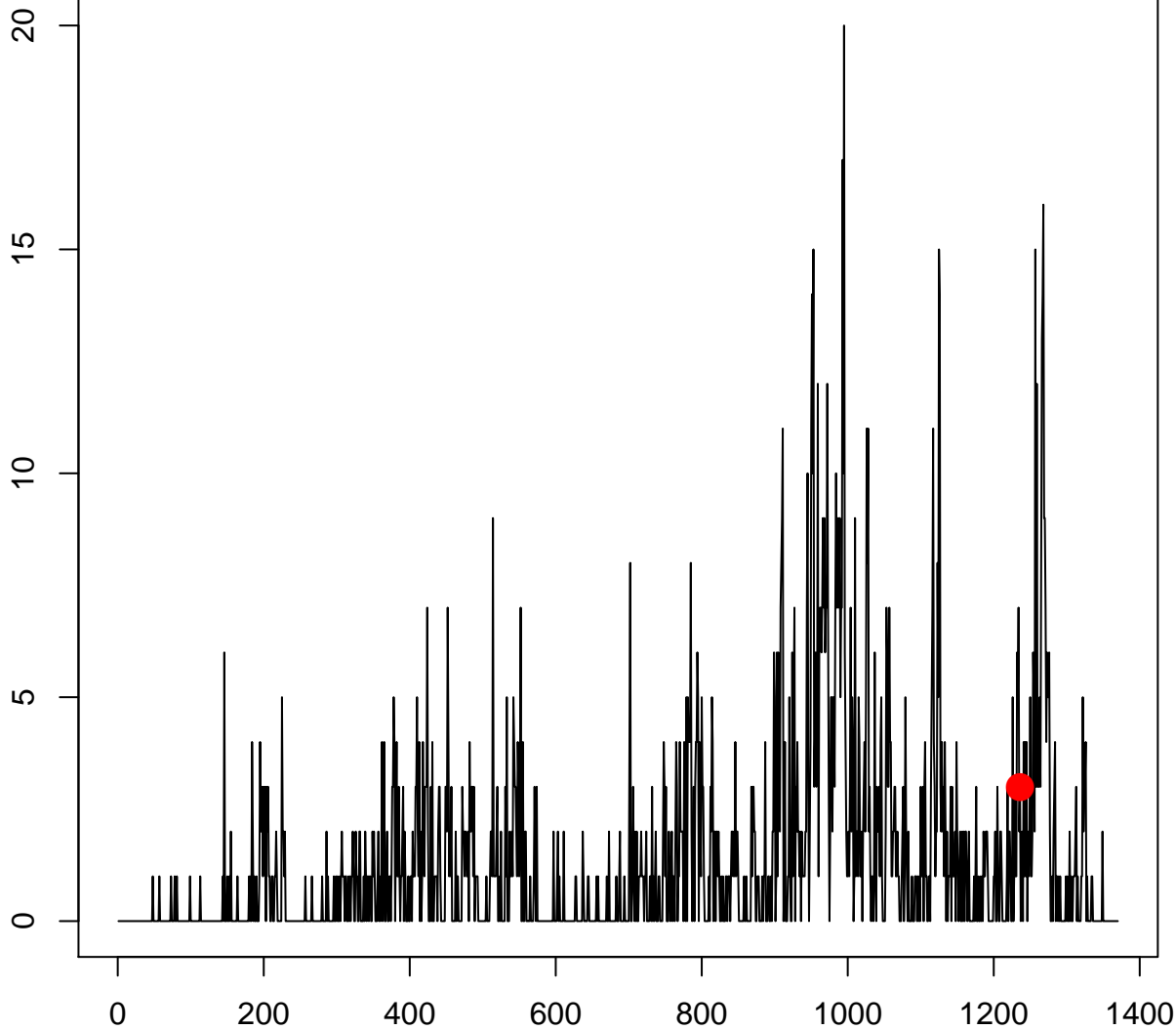

Transcript Position

Supplement: Supplementary file 2 [file Data_Sheet_8.ZIP › GSM2230747.plot/Lsa-miR171c_Lsat_1_v5_gn_5_53581.1_1236_TPlot.pdf]

**T=Lsat\_1\_v5\_gn\_6\_3180.1\_Q=Lsa-miR171c\_S=1758**

category=2\_p=0.0909453248773598

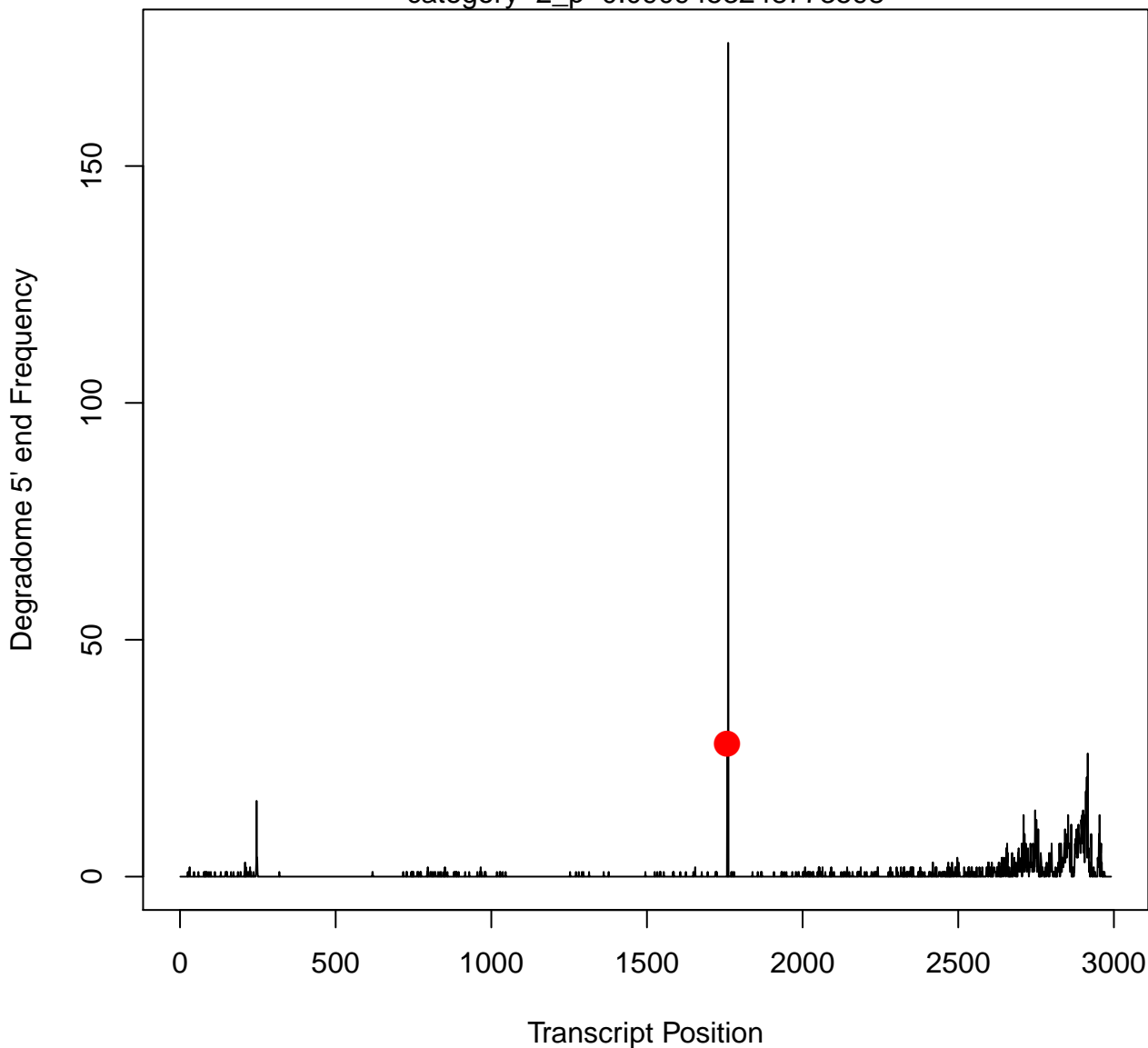

Supplement: Supplementary file 2 [file Data_Sheet_8.ZIP › GSM2230747.plot/Lsa-miR171c_Lsat_1_v5_gn_6_3180.1_1758_TPlot.pdf]

**T=Lsat\_1\_v5\_gn\_7\_110180.1\_Q=Lsa-miR171c\_S=2619**

category=2\_p=0.975733330133494

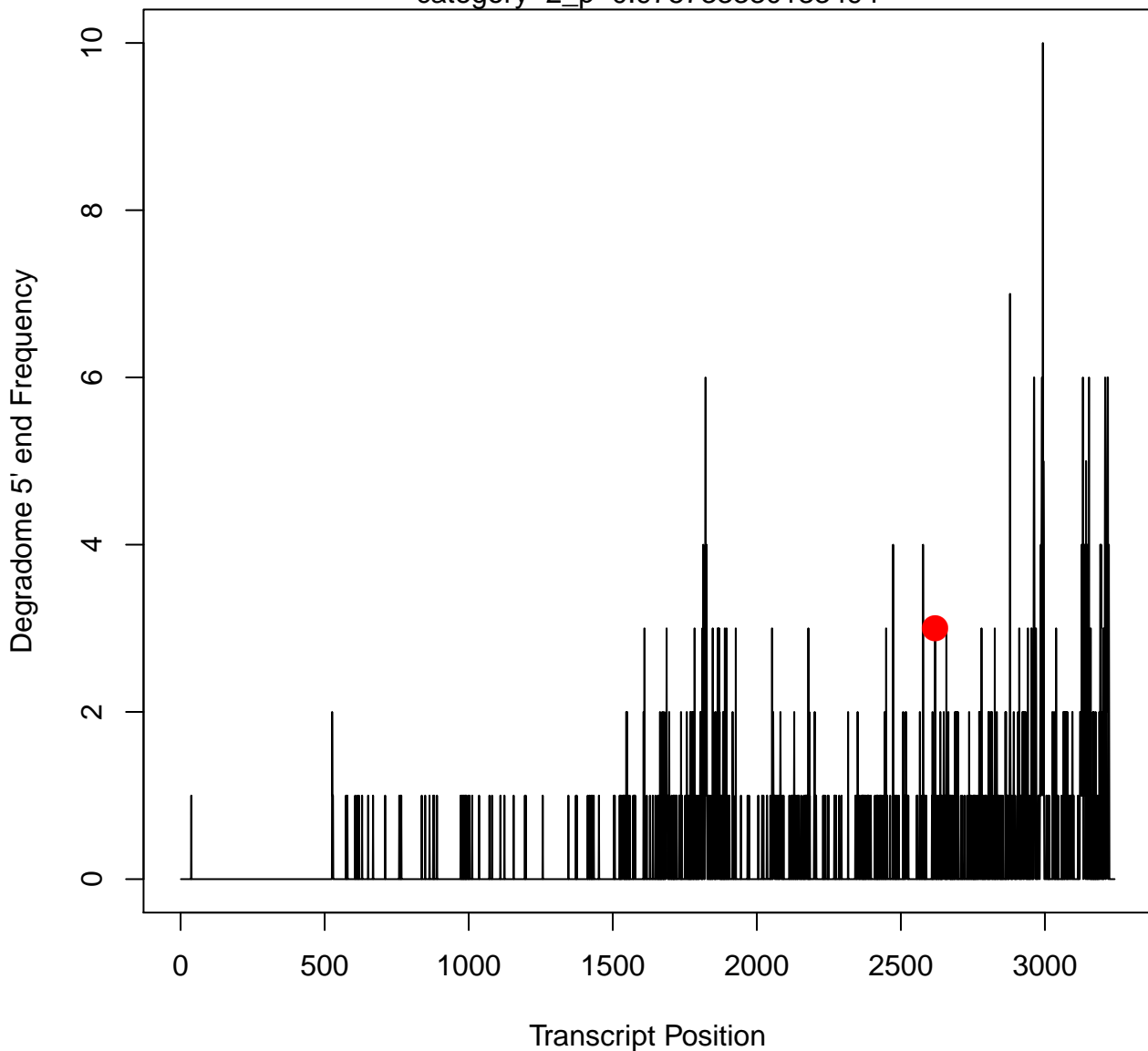

Supplement: Supplementary file 2 [file Data_Sheet_8.ZIP › GSM2230747.plot/Lsa-miR171c_Lsat_1_v5_gn_7_110180.1_2619_TPlot.pdf]

T=Lsat\_1\_v5\_gn\_7\_49541.1\_Q=Lsa-miR171c\_S=871

category=2\_p=0.398623169919353

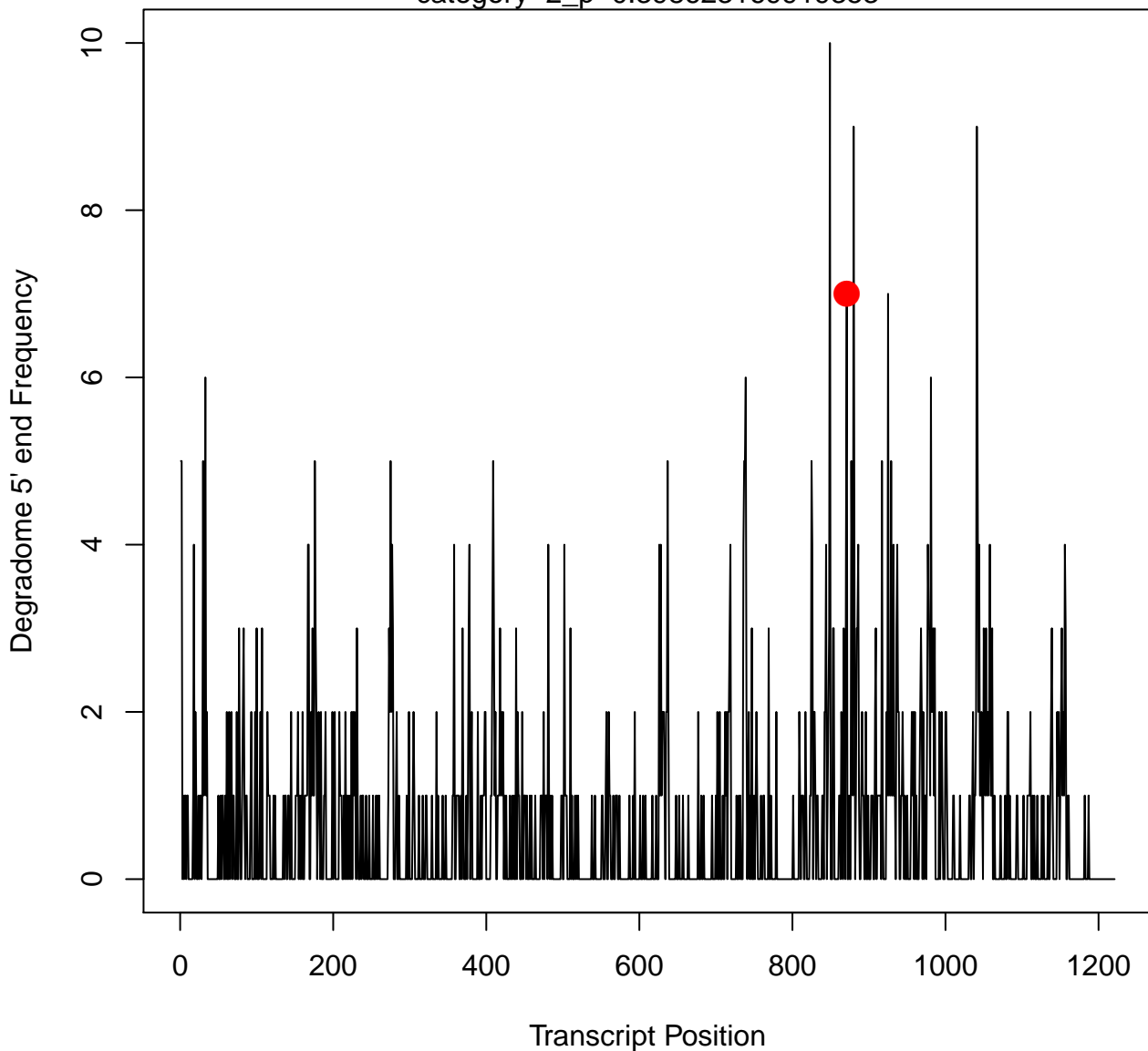

Supplement: Supplementary file 2 [file Data_Sheet_8.ZIP › GSM2230747.plot/Lsa-miR171c_Lsat_1_v5_gn_7_49541.1_871_TPlot.pdf]

**T=Lsat\_1\_v5\_gn\_7\_97240.1\_Q=Lsa-miR171c\_S=2083**

category=2\_p=0.98057384456223

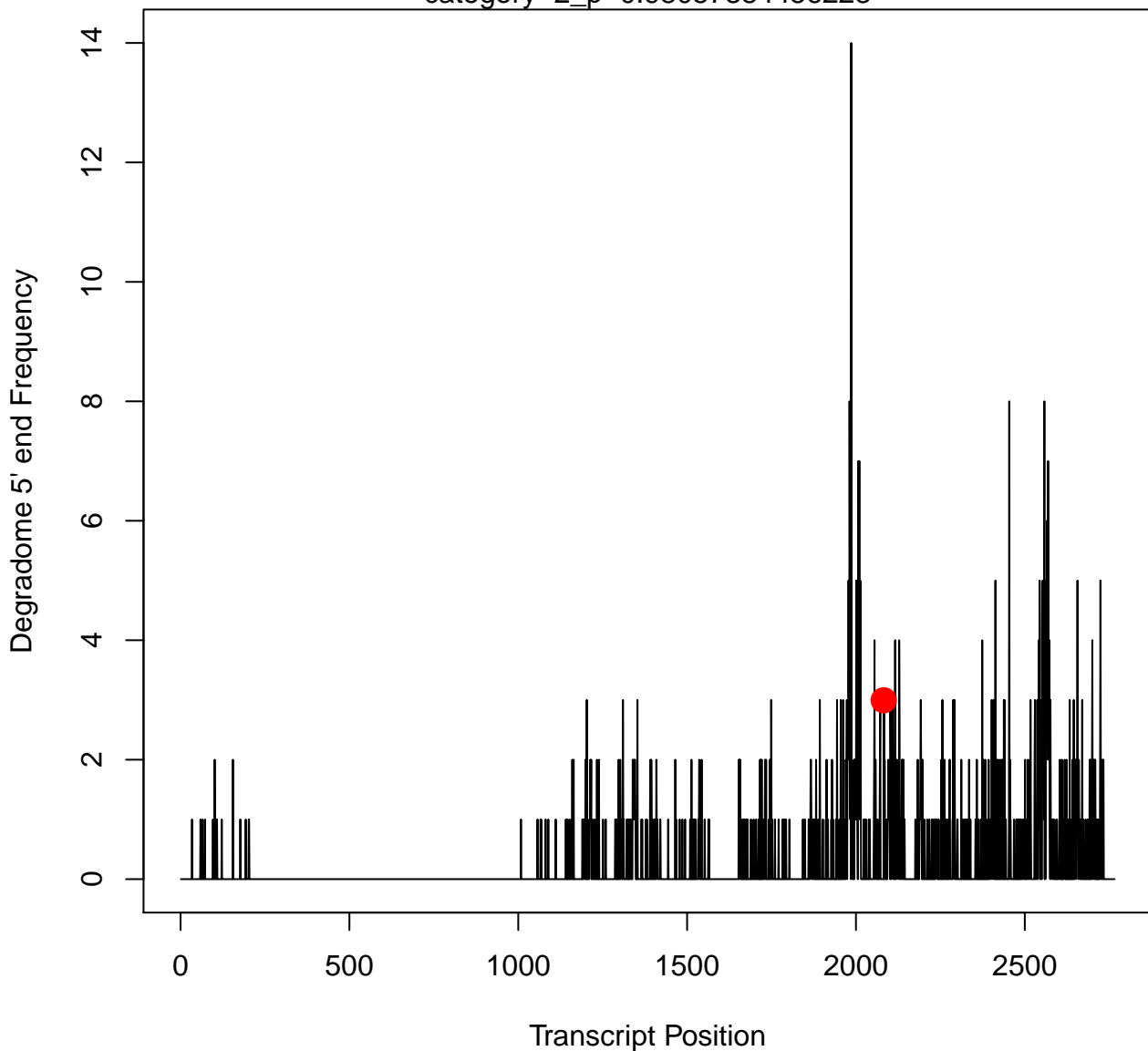

Supplement: Supplementary file 2 [file Data_Sheet_8.ZIP › GSM2230747.plot/Lsa-miR171c_Lsat_1_v5_gn_7_97240.1_2083_TPlot.pdf]

**T=Lsat\_1\_v5\_gn\_8\_8241.1\_Q=Lsa-miR171c\_S=753**

category=2\_p=0.992747569523279

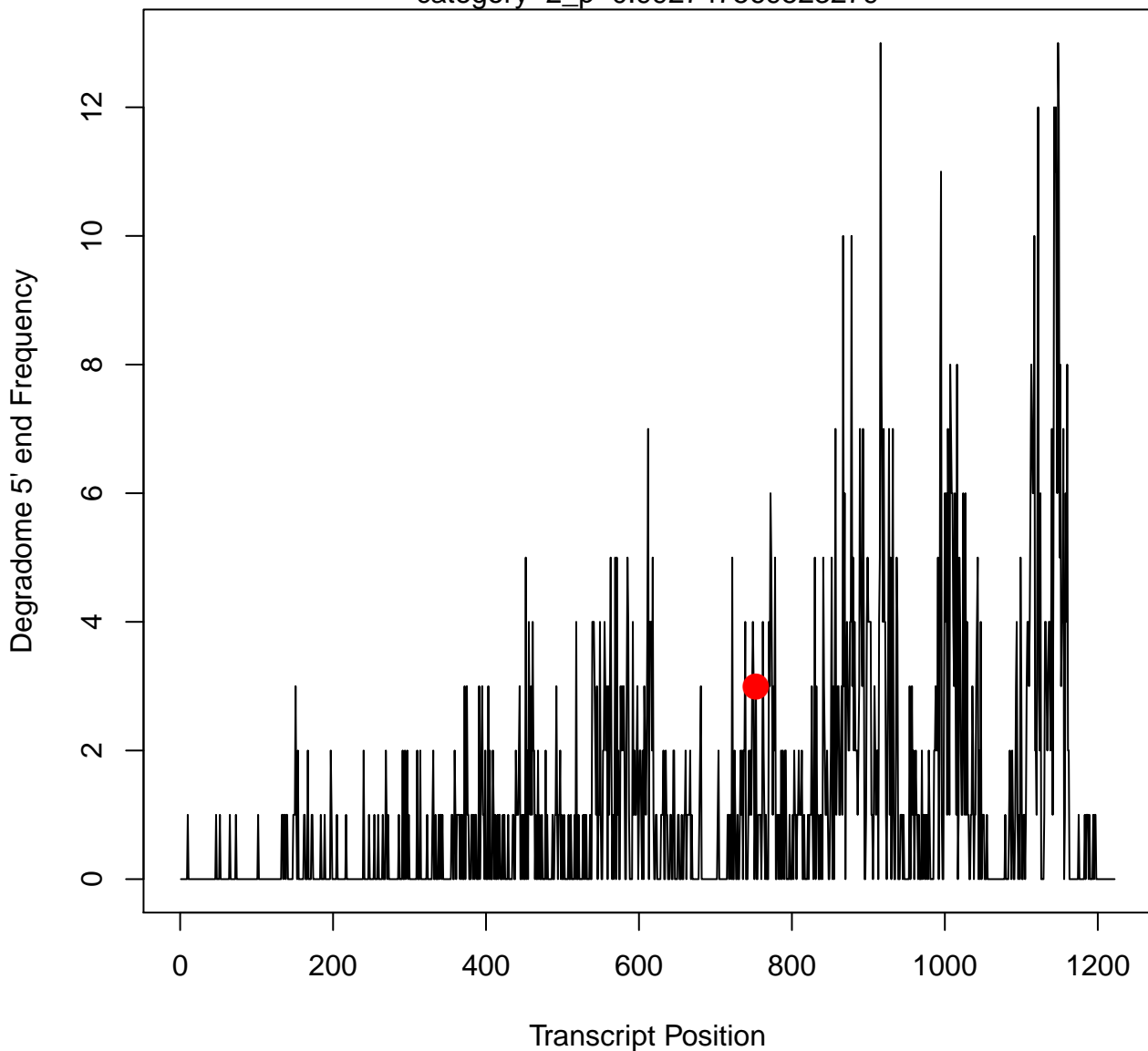

Supplement: Supplementary file 2 [file Data_Sheet_8.ZIP › GSM2230747.plot/Lsa-miR171c_Lsat_1_v5_gn_8_8241.1_753_TPlot.pdf]
