# Supplementary figures and images for: Comprehensive Annotation and Functional Exploration of MicroRNAs in Lettuce (part 1 of 6)
Source: Front Plant Sci. 2021 Dec 24;12:781836. doi: 10.3389/fpls.2021.781836 (PMC8739914; doi:10.3389/fpls.2021.781836)

T=Lsat\_1\_v5\_gn\_0\_22701.1\_Q=Lsa-miR1446\_S=951

category=2\_p=0.999909713298126

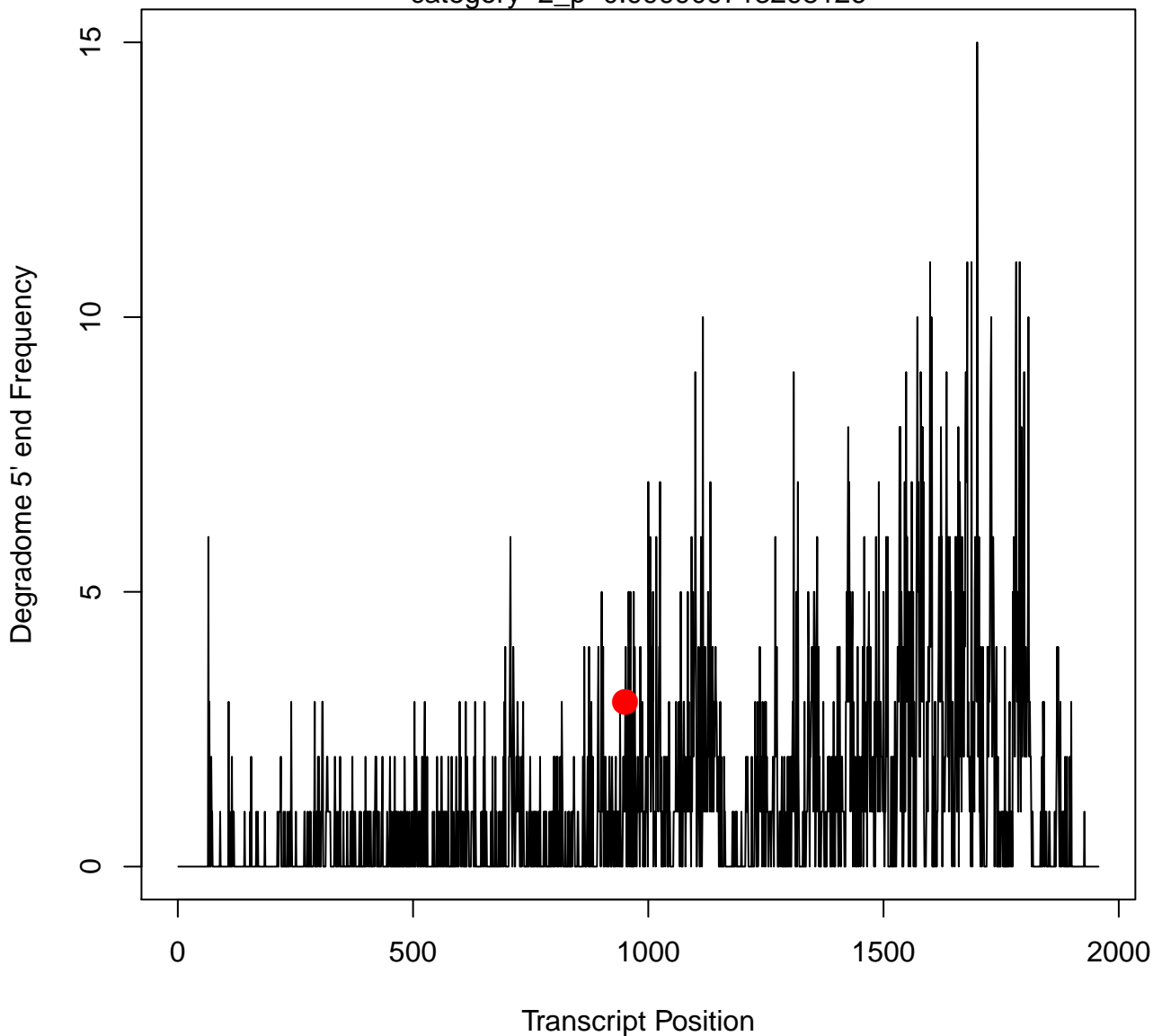

Supplement: Supplementary file 2 [file Data_Sheet_8.ZIP › GSM2230747.plot/Lsa-miR1446_Lsat_1_v5_gn_0_22701.1_951_TPlot.pdf]

**T=Lsat\_1\_v5\_gn\_2\_115240.1\_Q=Lsa-miR1446\_S=656**

category=2\_p=0.998264863505651

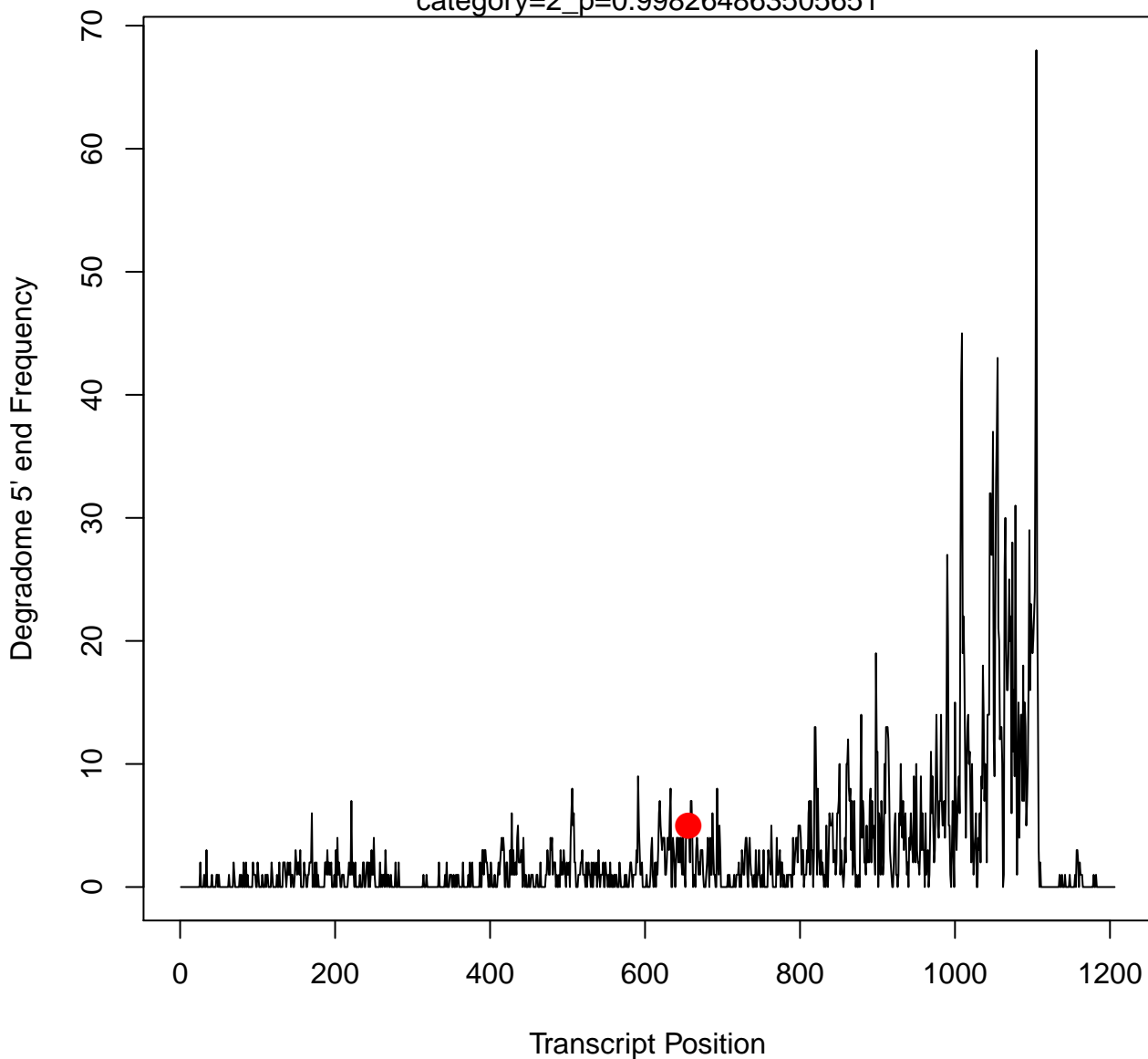

Supplement: Supplementary file 2 [file Data_Sheet_8.ZIP › GSM2230747.plot/Lsa-miR1446_Lsat_1_v5_gn_2_115240.1_656_TPlot.pdf]

**T=Lsat\_1\_v5\_gn\_2\_133880.1\_Q=Lsa-miR1446\_S=1251**

category=2\_p=0.295041826195748

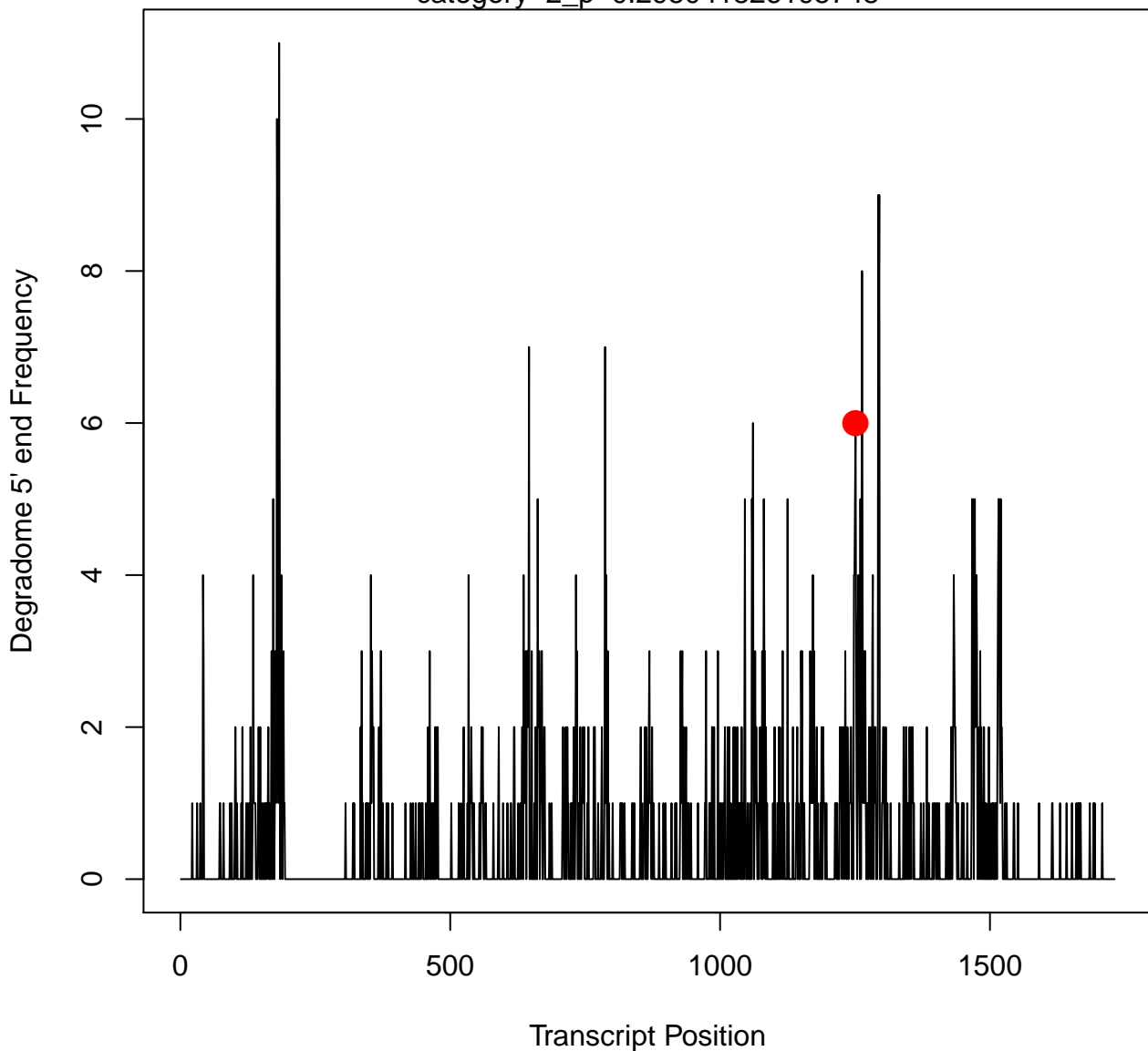

Supplement: Supplementary file 2 [file Data_Sheet_8.ZIP › GSM2230747.plot/Lsa-miR1446_Lsat_1_v5_gn_2_133880.1_1251_TPlot.pdf]

**T=Lsat\_1\_v5\_gn\_2\_66660.1\_Q=Lsa-miR1446\_S=650**

category=2\_p=0.998319144759831

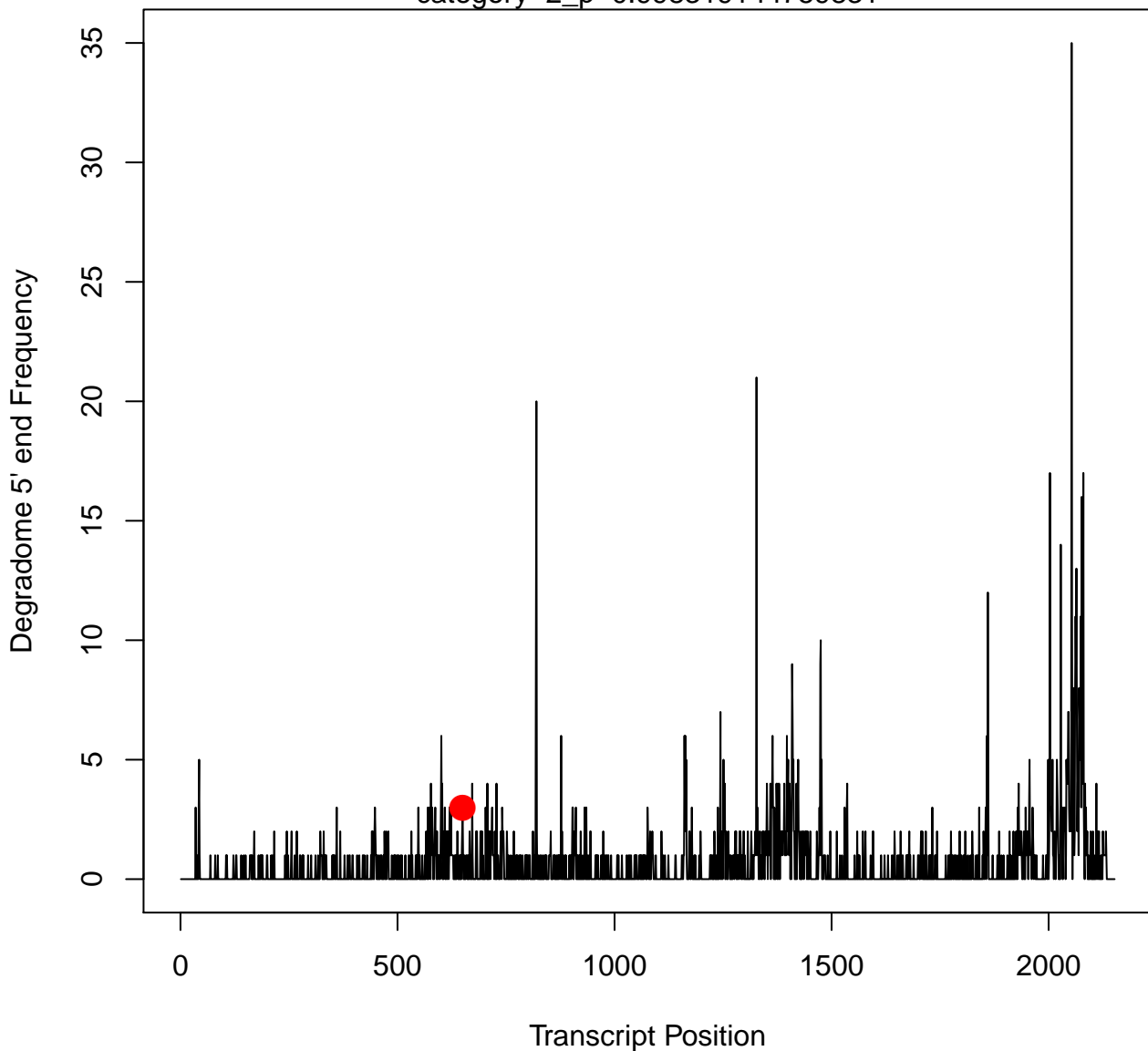

Supplement: Supplementary file 2 [file Data_Sheet_8.ZIP › GSM2230747.plot/Lsa-miR1446_Lsat_1_v5_gn_2_66660.1_650_TPlot.pdf]

T=Lsat\_1\_v5\_gn\_2\_77740.1\_Q=Lsa-miR1446\_S=759

category=1\_p=0.0680154769210826

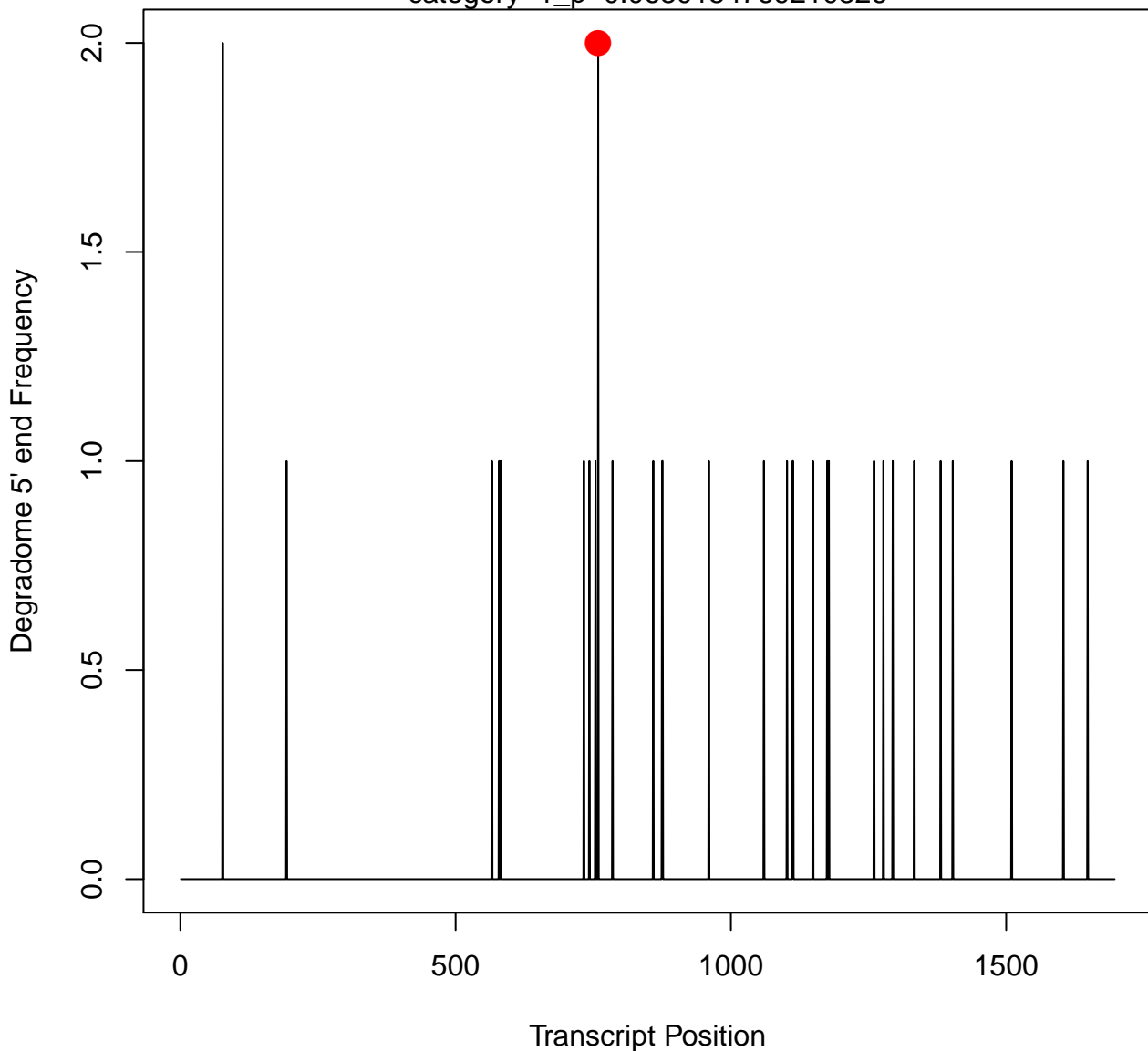

Supplement: Supplementary file 2 [file Data_Sheet_8.ZIP › GSM2230747.plot/Lsa-miR1446_Lsat_1_v5_gn_2_77740.1_759_TPlot.pdf]

T=Lsat\_1\_v5\_gn\_2\_90160.1\_Q=Lsa-miR1446\_S=492

category=2\_p=0.996826072896214

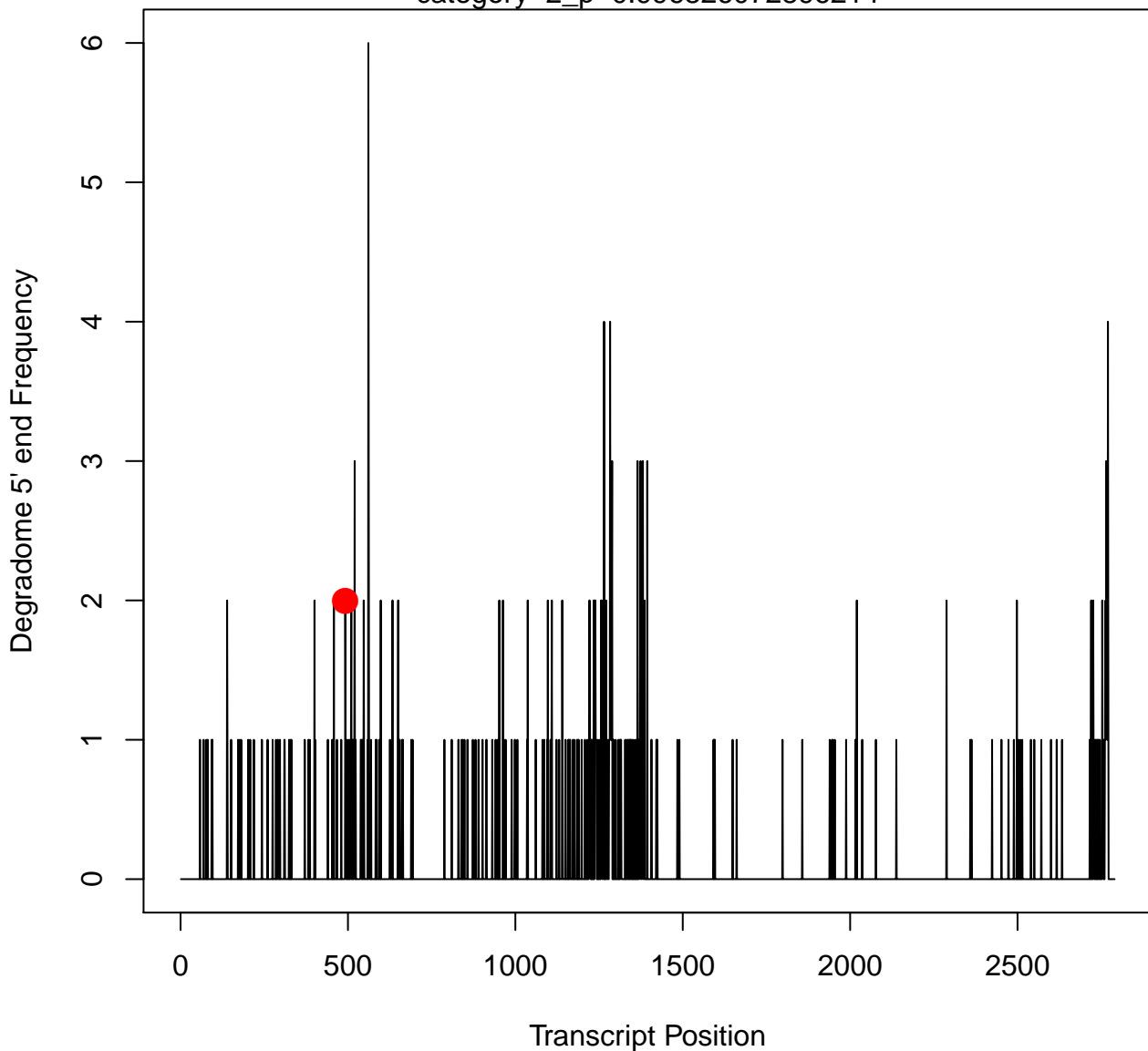

Supplement: Supplementary file 2 [file Data_Sheet_8.ZIP › GSM2230747.plot/Lsa-miR1446_Lsat_1_v5_gn_2_90160.1_492_TPlot.pdf]

**T=Lsat\_1\_v5\_gn\_2\_91621.1\_Q=Lsa-miR1446\_S=2389**

category=2\_p=0.999447396412328

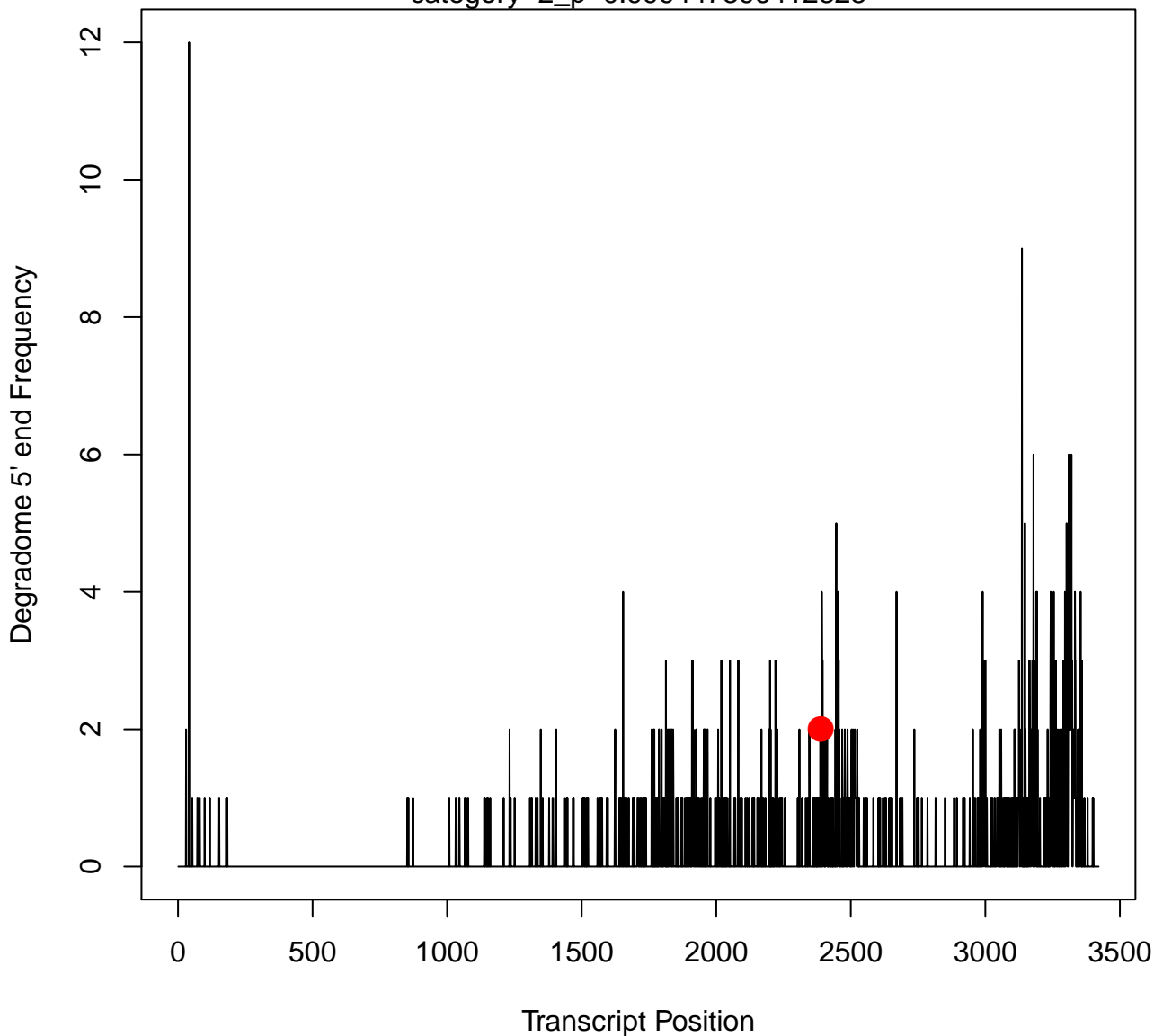

Supplement: Supplementary file 2 [file Data_Sheet_8.ZIP › GSM2230747.plot/Lsa-miR1446_Lsat_1_v5_gn_2_91621.1_2389_TPlot.pdf]

**T=Lsat\_1\_v5\_gn\_3\_102160.1\_Q=Lsa-miR1446\_S=1169**

category=2\_p=0.435661047157111

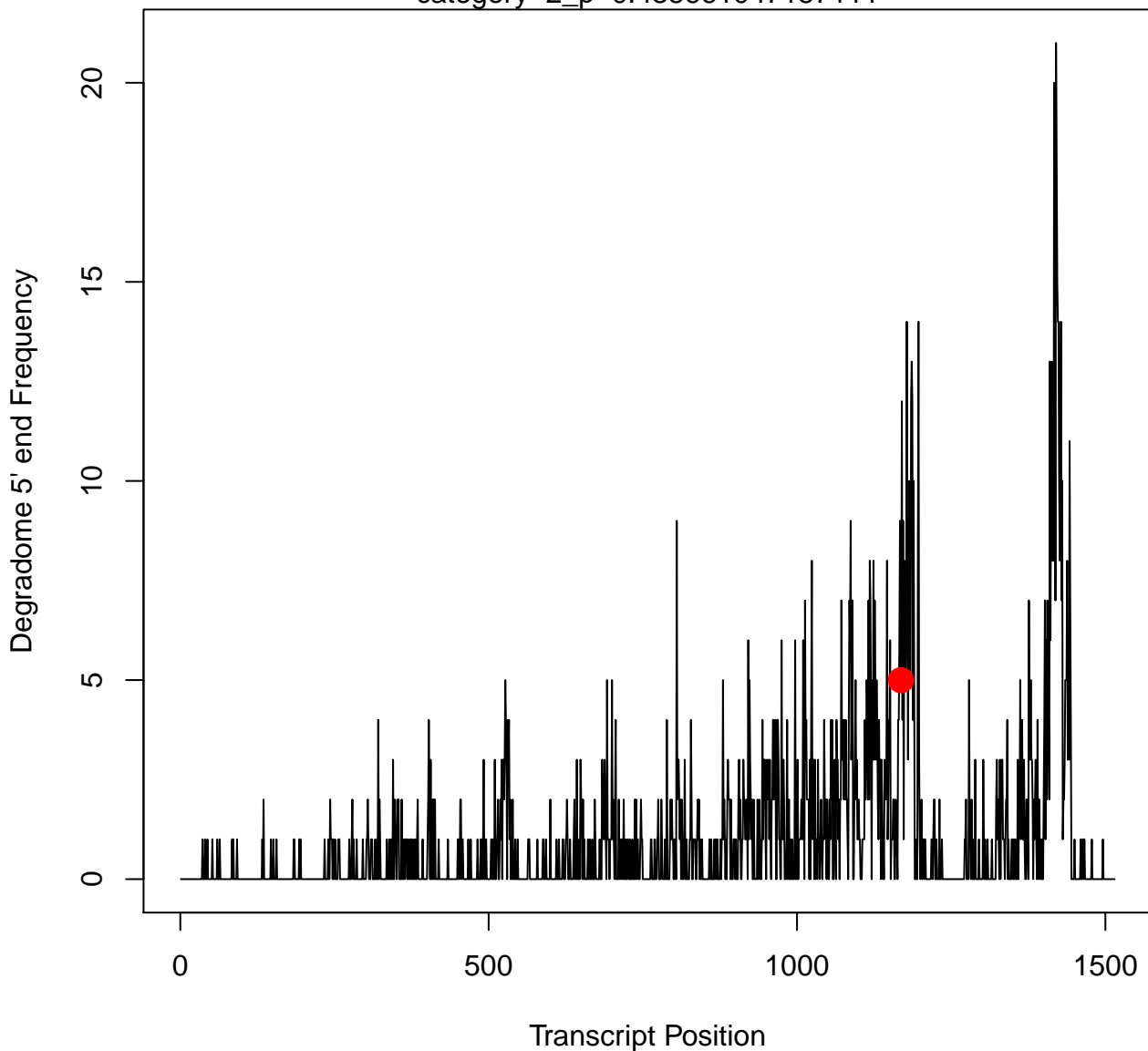

Supplement: Supplementary file 2 [file Data_Sheet_8.ZIP › GSM2230747.plot/Lsa-miR1446_Lsat_1_v5_gn_3_102160.1_1169_TPlot.pdf]

**T=Lsat\_1\_v5\_gn\_3\_32820.1\_Q=Lsa-miR1446\_S=1167**

category=2\_p=0.602164642397273

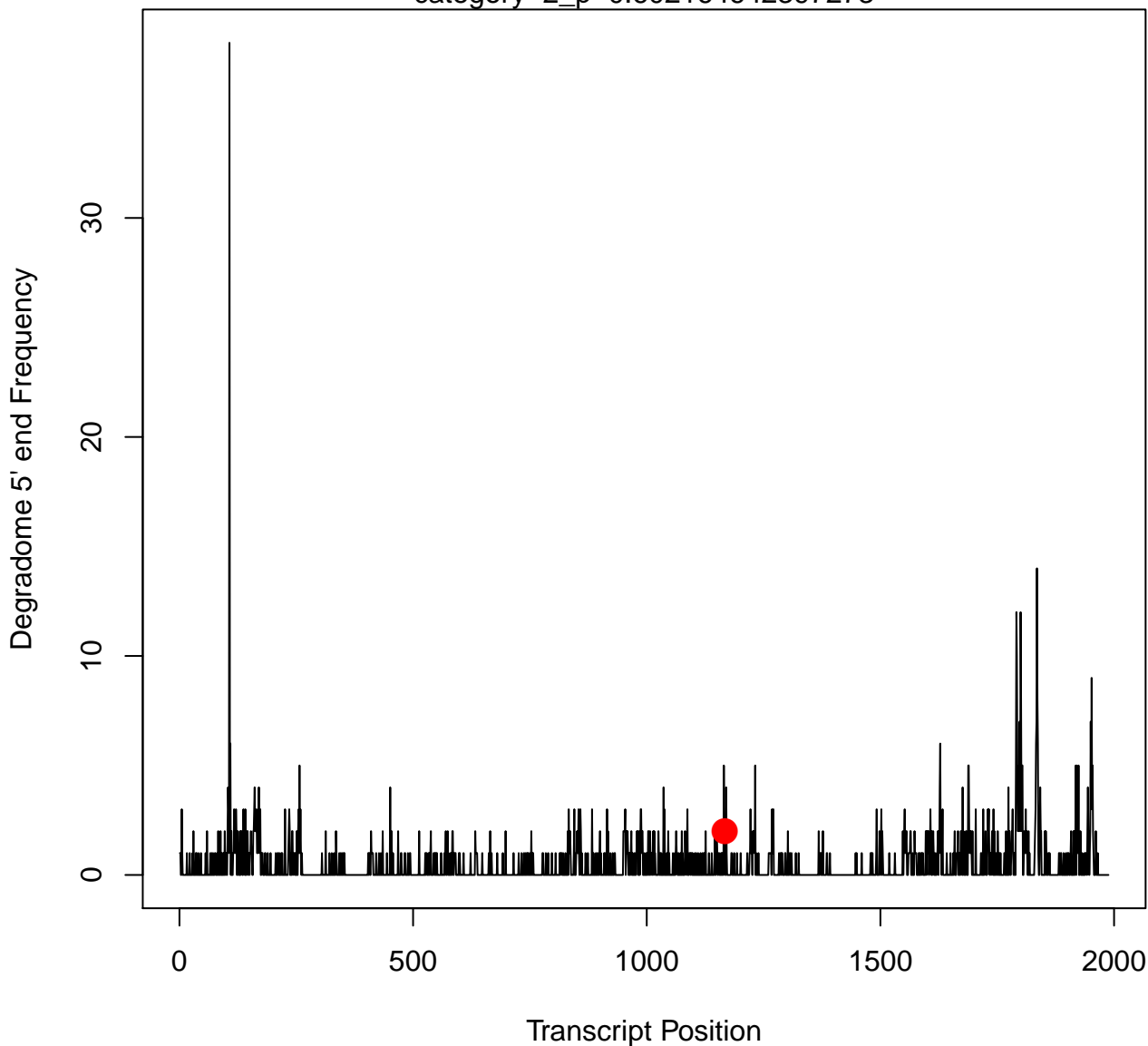

Supplement: Supplementary file 2 [file Data_Sheet_8.ZIP › GSM2230747.plot/Lsa-miR1446_Lsat_1_v5_gn_3_32820.1_1167_TPlot.pdf]

**T=Lsat\_1\_v5\_gn\_3\_53980.1\_Q=Lsa-miR1446\_S=441**

category=2\_p=0.97494966641061

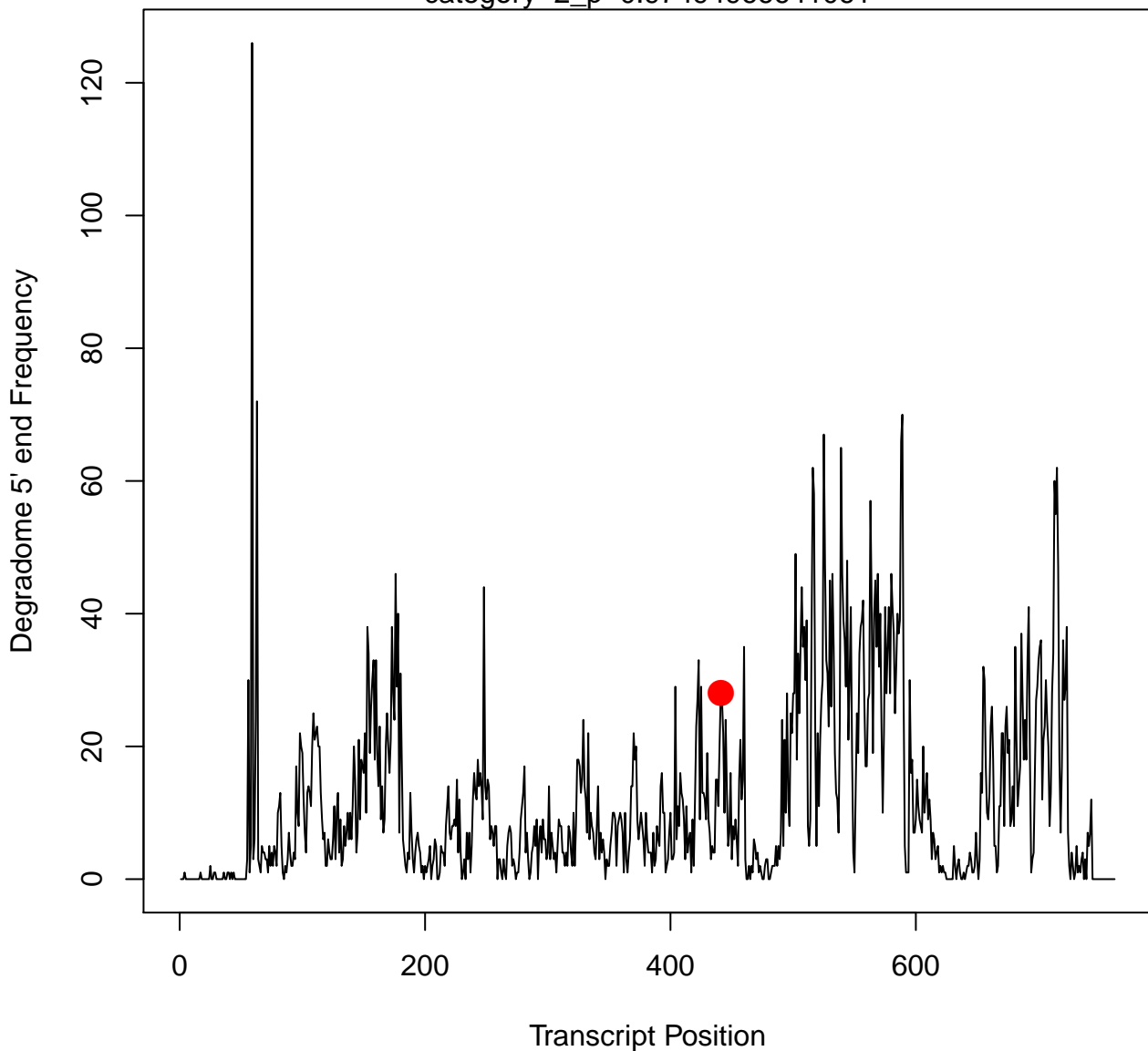

Supplement: Supplementary file 2 [file Data_Sheet_8.ZIP › GSM2230747.plot/Lsa-miR1446_Lsat_1_v5_gn_3_53980.1_441_TPlot.pdf]

**T=Lsat\_1\_v5\_gn\_3\_61481.1\_Q=Lsa-miR1446\_S=3952**

category=2\_p=0.996395788441243

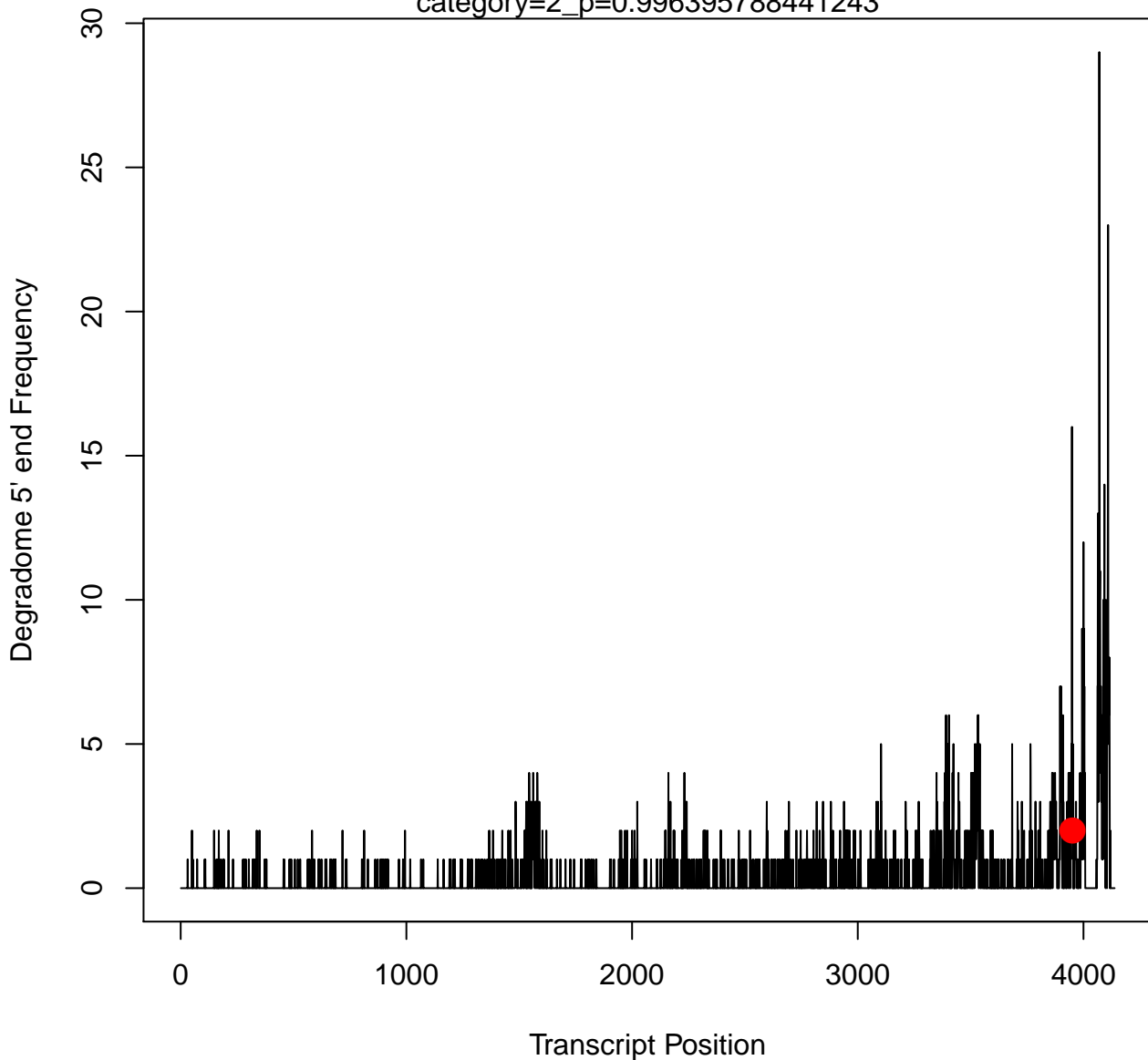

Supplement: Supplementary file 2 [file Data_Sheet_8.ZIP › GSM2230747.plot/Lsa-miR1446_Lsat_1_v5_gn_3_61481.1_3952_TPlot.pdf]

**T=Lsat\_1\_v5\_gn\_3\_84881.1\_Q=Lsa-miR1446\_S=478**

category=2\_p=0.994722232475501

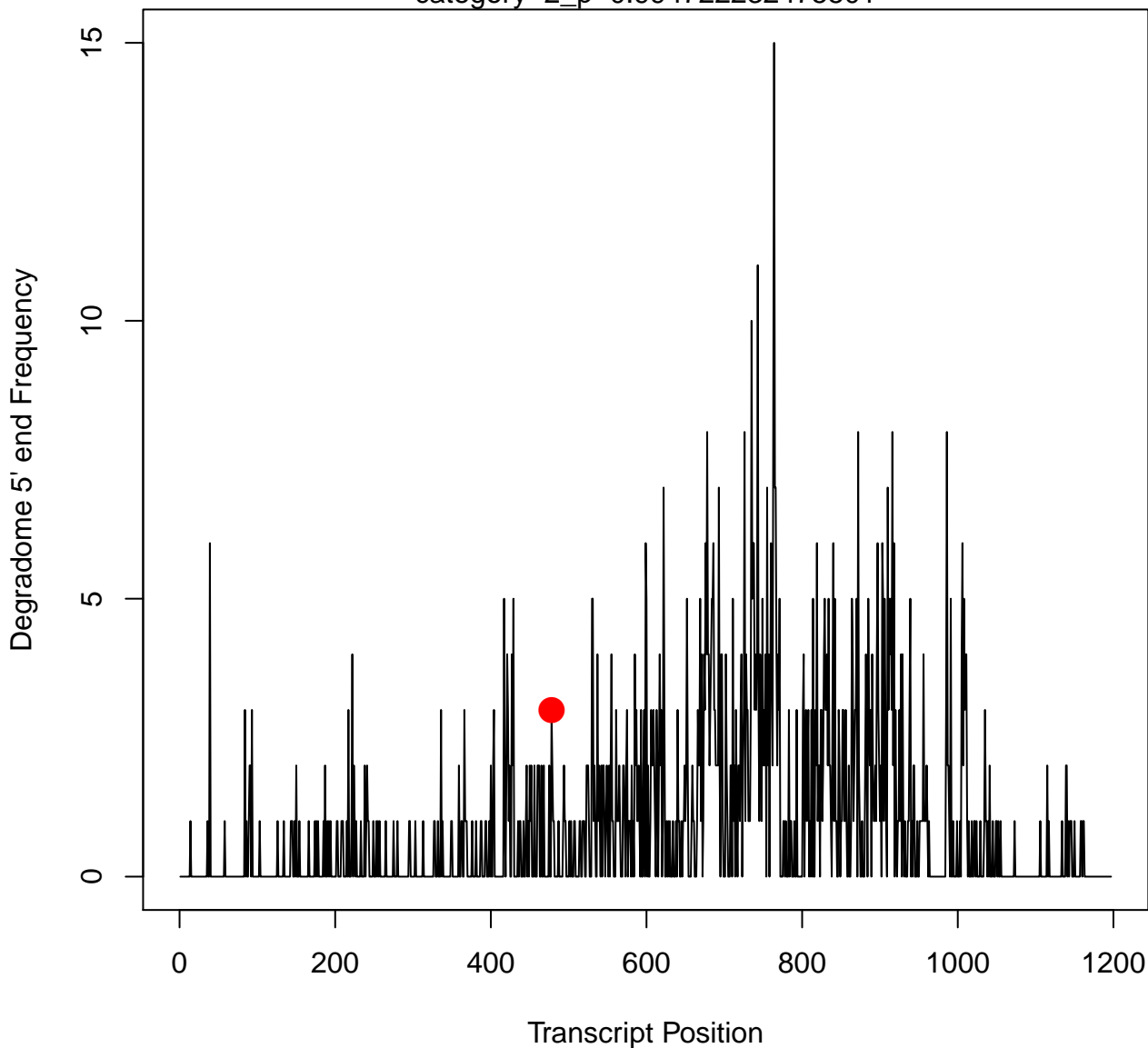

Supplement: Supplementary file 2 [file Data_Sheet_8.ZIP › GSM2230747.plot/Lsa-miR1446_Lsat_1_v5_gn_3_84881.1_478_TPlot.pdf]

**T=Lsat\_1\_v5\_gn\_4\_71321.1\_Q=Lsa-miR1446\_S=1129**

category=2\_p=0.533641349080958

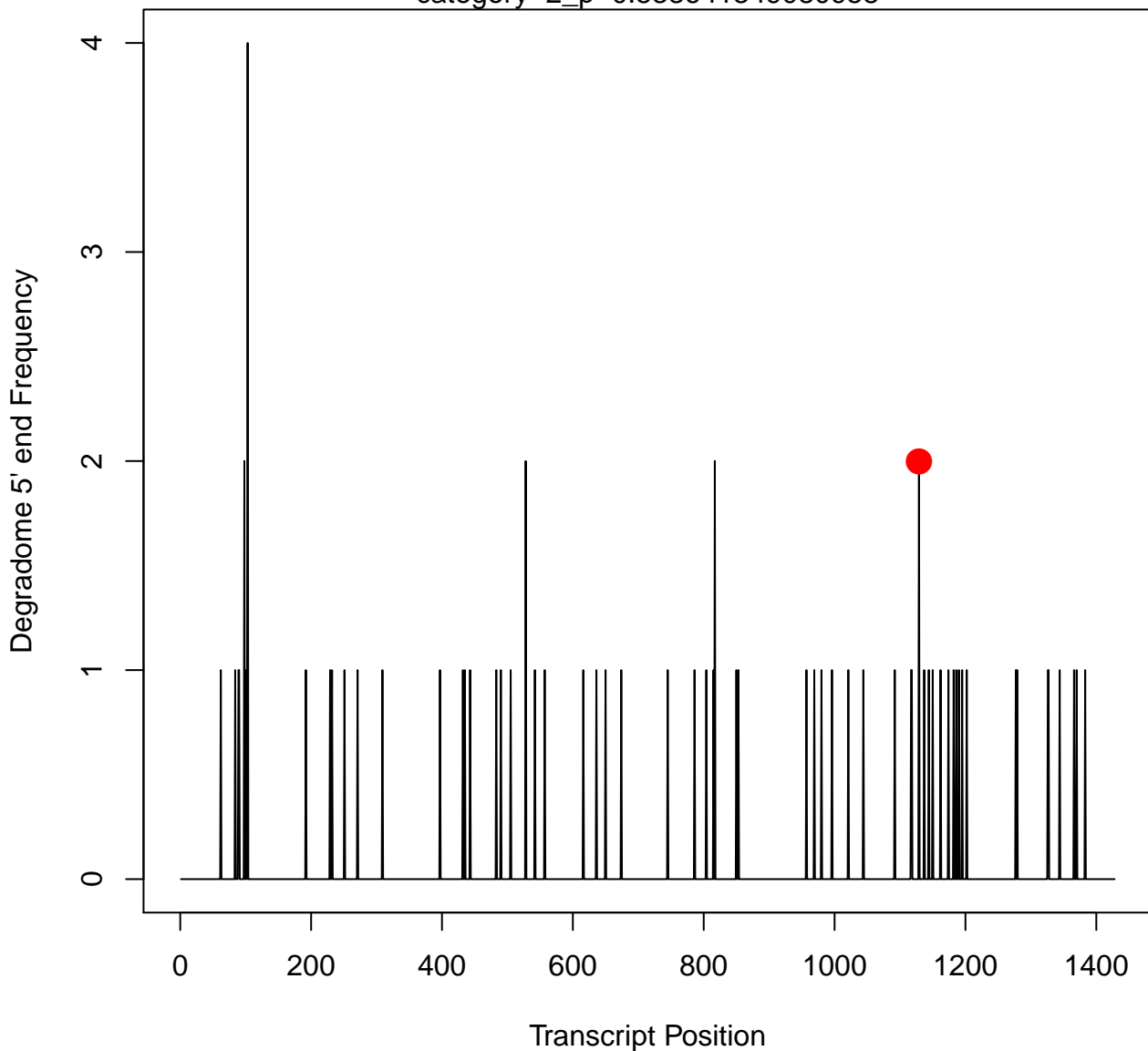

Supplement: Supplementary file 2 [file Data_Sheet_8.ZIP › GSM2230747.plot/Lsa-miR1446_Lsat_1_v5_gn_4_71321.1_1129_TPlot.pdf]

**T=Lsat\_1\_v5\_gn\_4\_73401.1\_Q=Lsa-miR1446\_S=1805**

category=2\_p=0.999991935627619

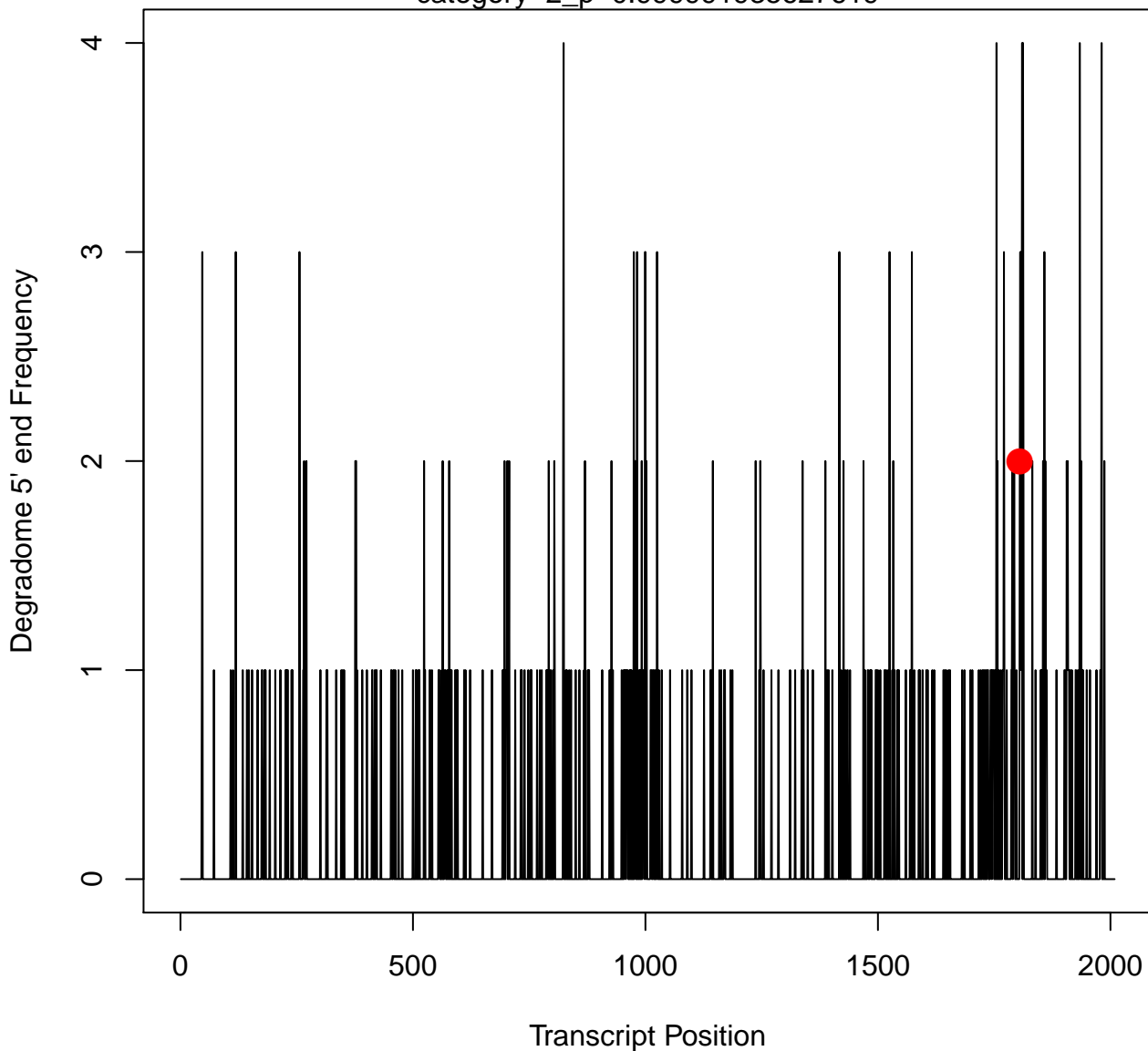

Supplement: Supplementary file 2 [file Data_Sheet_8.ZIP › GSM2230747.plot/Lsa-miR1446_Lsat_1_v5_gn_4_73401.1_1805_TPlot.pdf]

**T=Lsat\_1\_v5\_gn\_4\_76080.1\_Q=Lsa-miR1446\_S=648**

category=2\_p=0.869206317914807

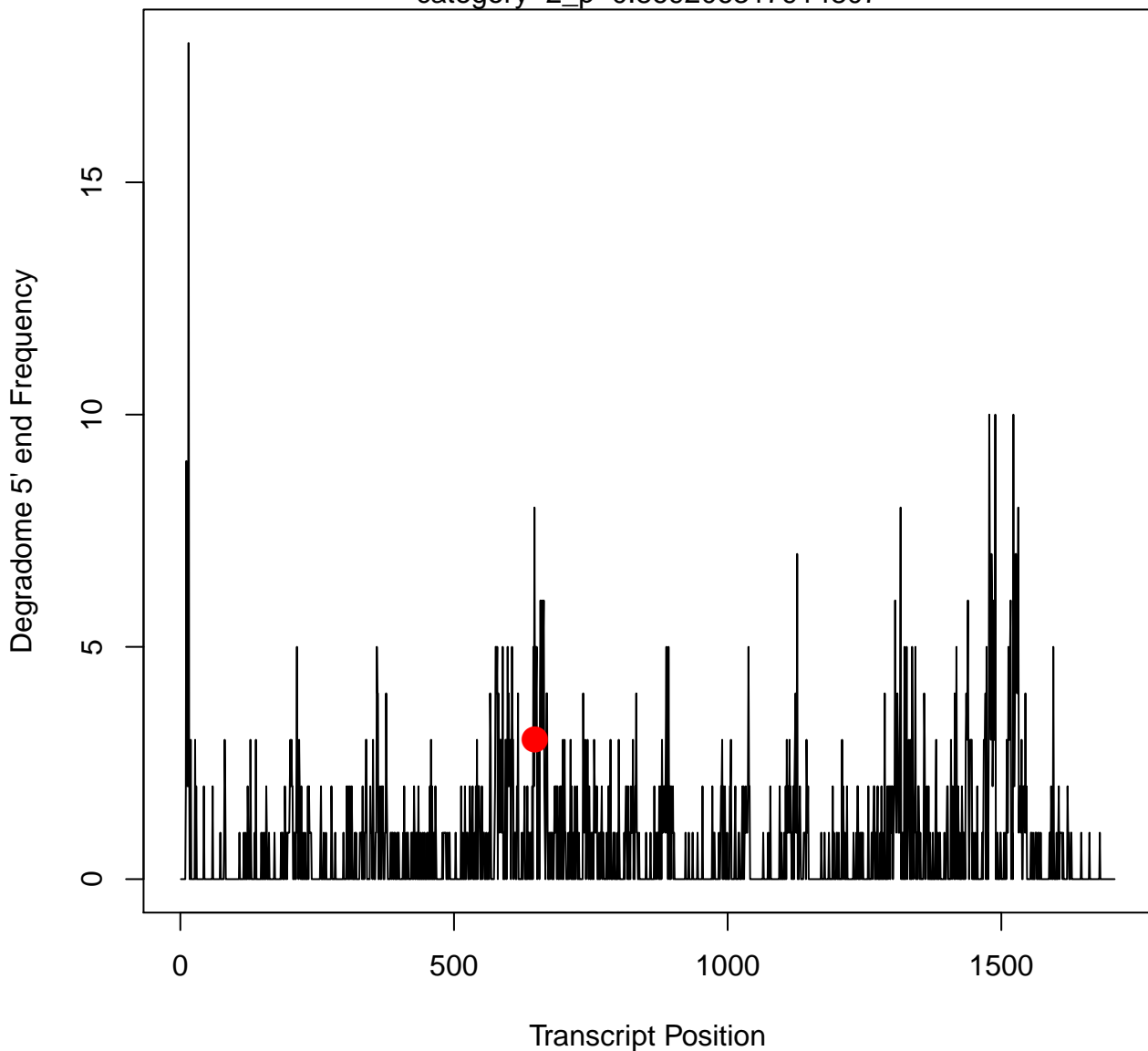

Supplement: Supplementary file 2 [file Data_Sheet_8.ZIP › GSM2230747.plot/Lsa-miR1446_Lsat_1_v5_gn_4_76080.1_648_TPlot.pdf]

**T=Lsat\_1\_v5\_gn\_5\_170600.1\_Q=Lsa-miR1446\_S=1122**

category=2\_p=0.999829513331464

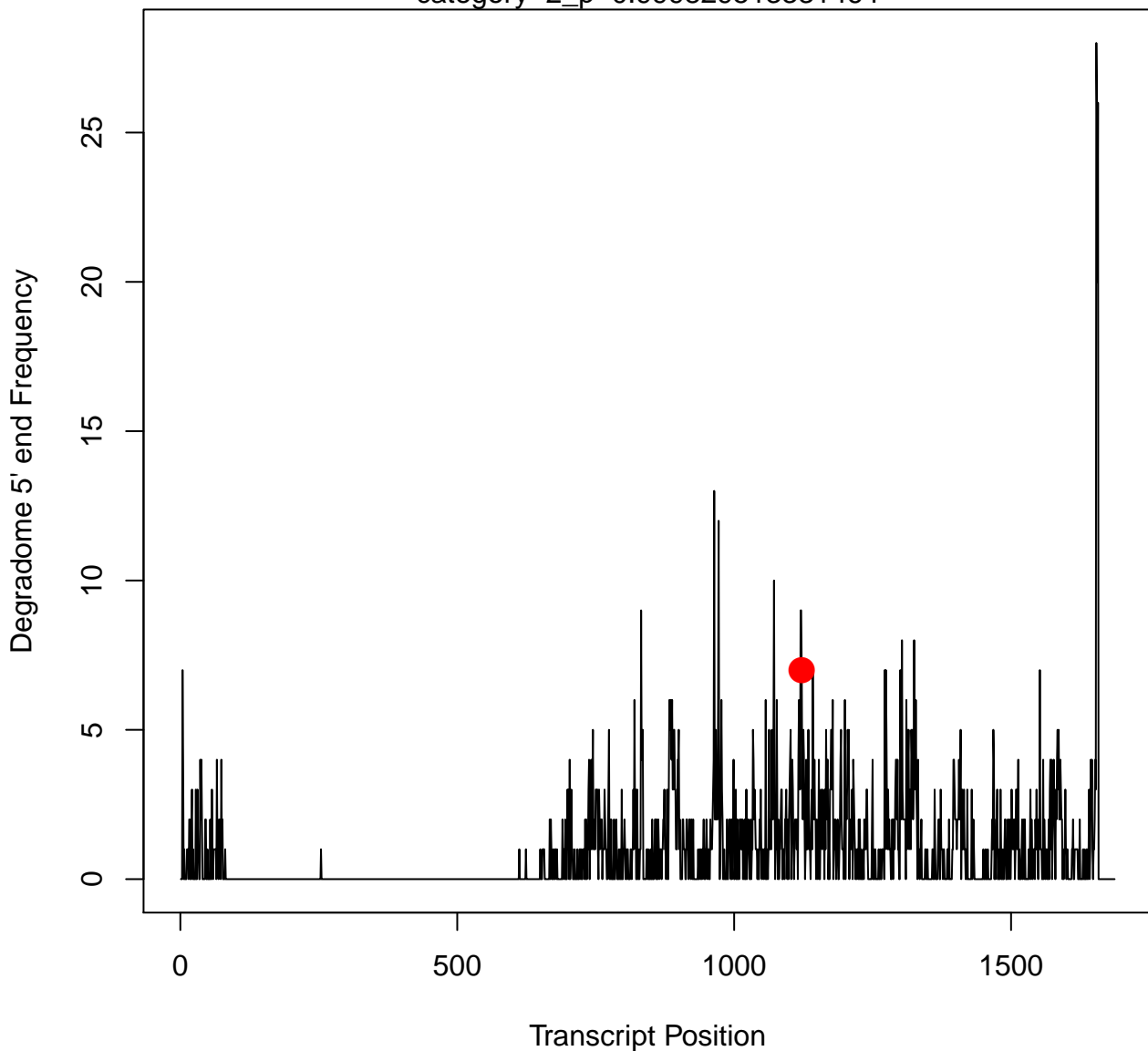

Supplement: Supplementary file 2 [file Data_Sheet_8.ZIP › GSM2230747.plot/Lsa-miR1446_Lsat_1_v5_gn_5_170600.1_1122_TPlot.pdf]

**T=Lsat\_1\_v5\_gn\_6\_47421.1\_Q=Lsa-miR1446\_S=1114**

category=2\_p=0.317095430591475

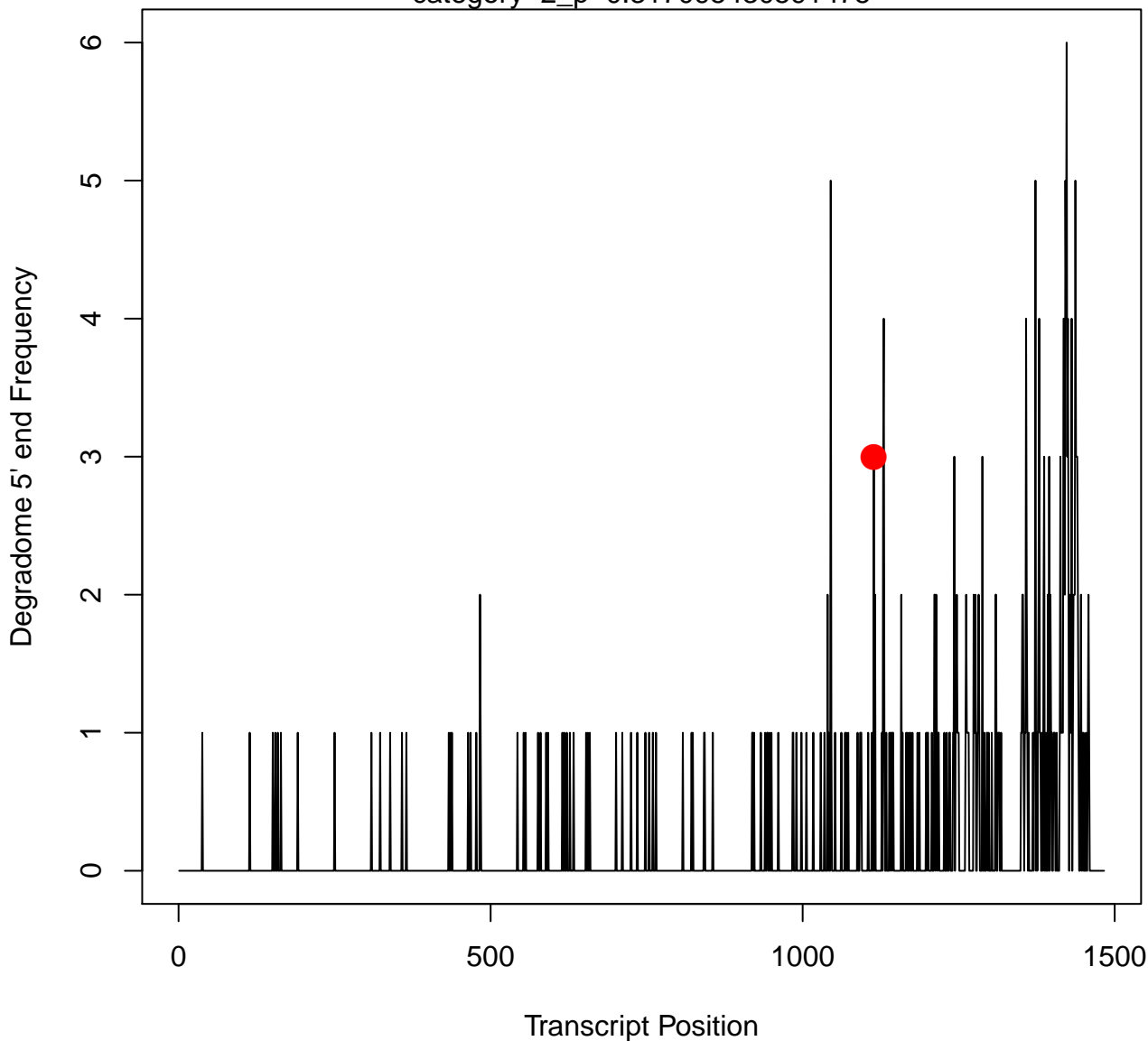

Supplement: Supplementary file 2 [file Data_Sheet_8.ZIP › GSM2230747.plot/Lsa-miR1446_Lsat_1_v5_gn_6_47421.1_1114_TPlot.pdf]

**T=Lsat\_1\_v5\_gn\_6\_8501.1\_Q=Lsa-miR1446\_S=2875**

category=2\_p=0.831339485668459

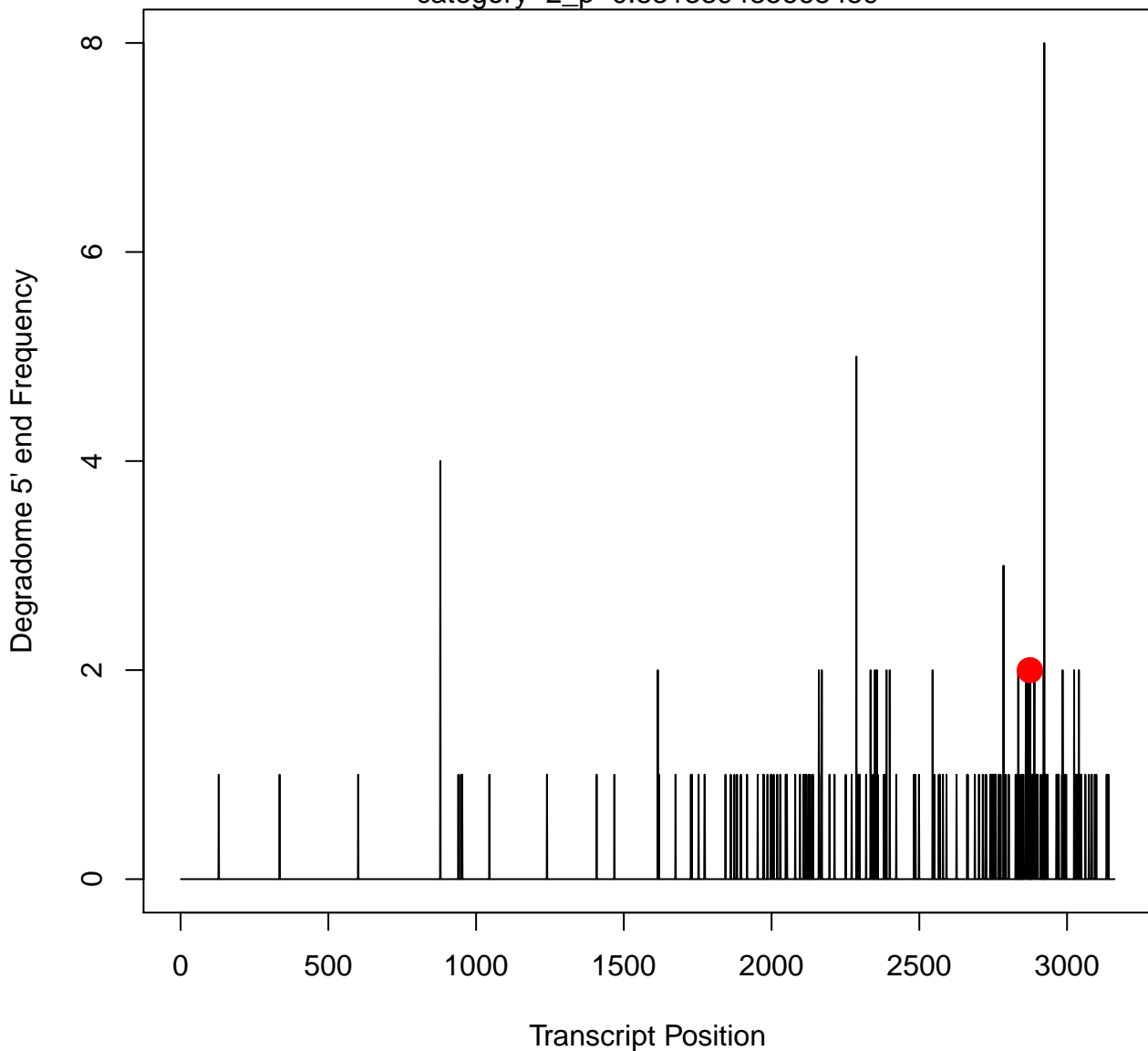

Supplement: Supplementary file 2 [file Data_Sheet_8.ZIP › GSM2230747.plot/Lsa-miR1446_Lsat_1_v5_gn_6_8501.1_2875_TPlot.pdf]

**T=Lsat\_1\_v5\_gn\_8\_65360.1\_Q=Lsa-miR1446\_S=1920**

category=2\_p=0.960910496111109

Degradome 5' end Frequency

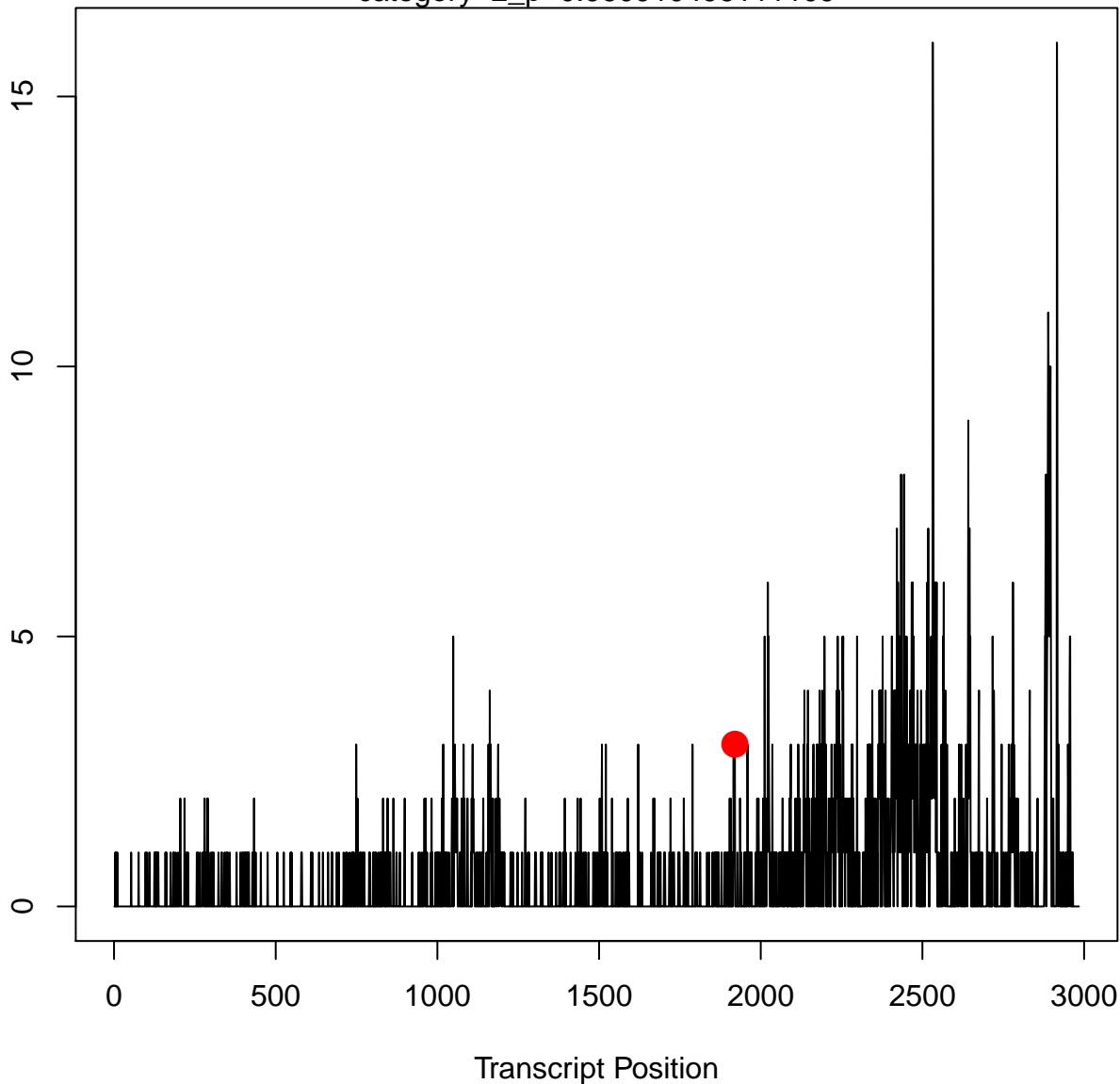

Supplement: Supplementary file 2 [file Data_Sheet_8.ZIP › GSM2230747.plot/Lsa-miR1446_Lsat_1_v5_gn_8_65360.1_1920_TPlot.pdf]

**T=Lsat\_1\_v5\_gn\_9\_121240.1\_Q=Lsa-miR1446\_S=2400**

category=2\_p=0.996035209259256

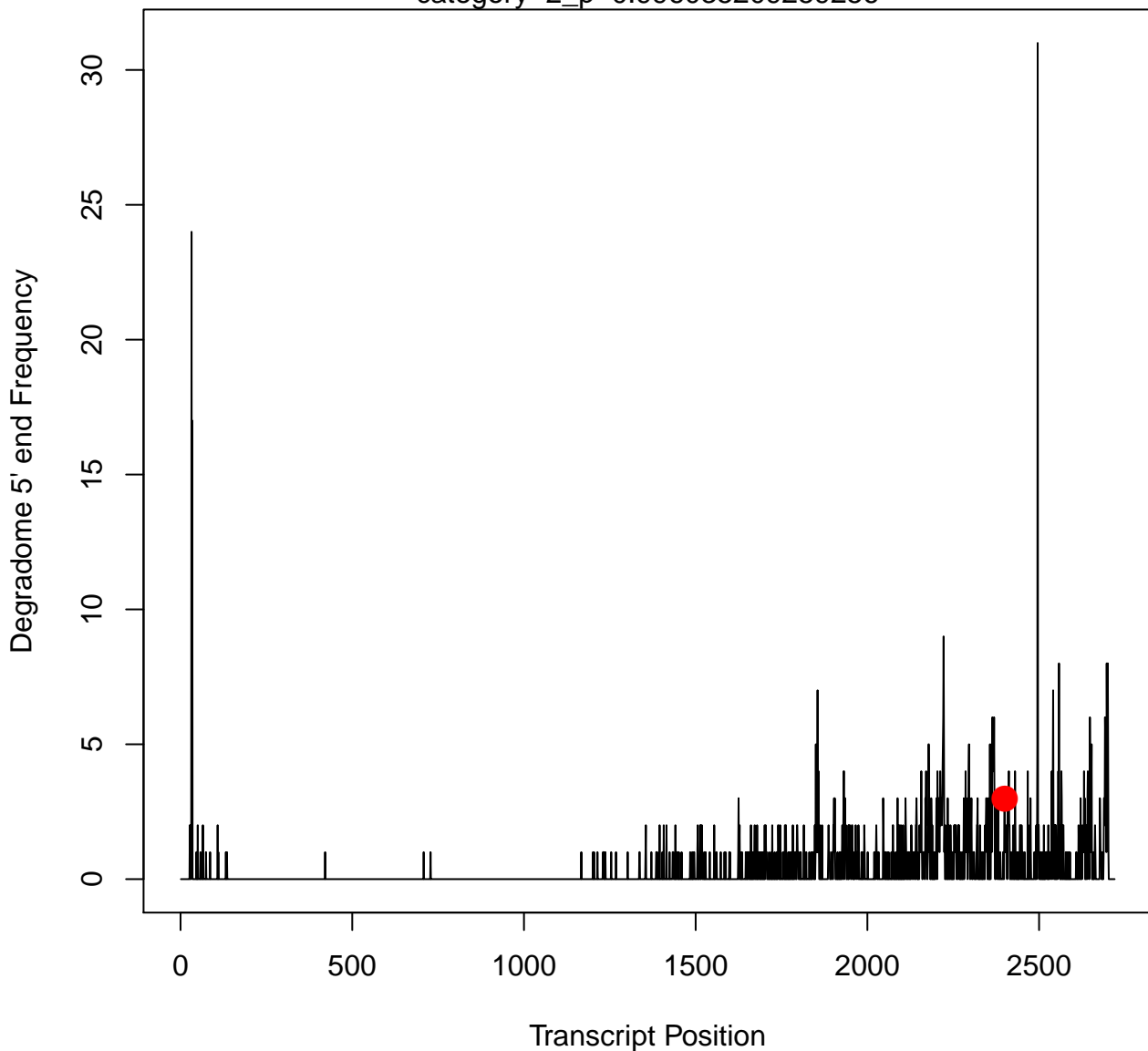

Supplement: Supplementary file 2 [file Data_Sheet_8.ZIP › GSM2230747.plot/Lsa-miR1446_Lsat_1_v5_gn_9_121240.1_2400_TPlot.pdf]

**T=Lsat\_1\_v5\_gn\_9\_23820.1\_Q=Lsa-miR1446\_S=1662**

category=2\_p=0.999854563382474

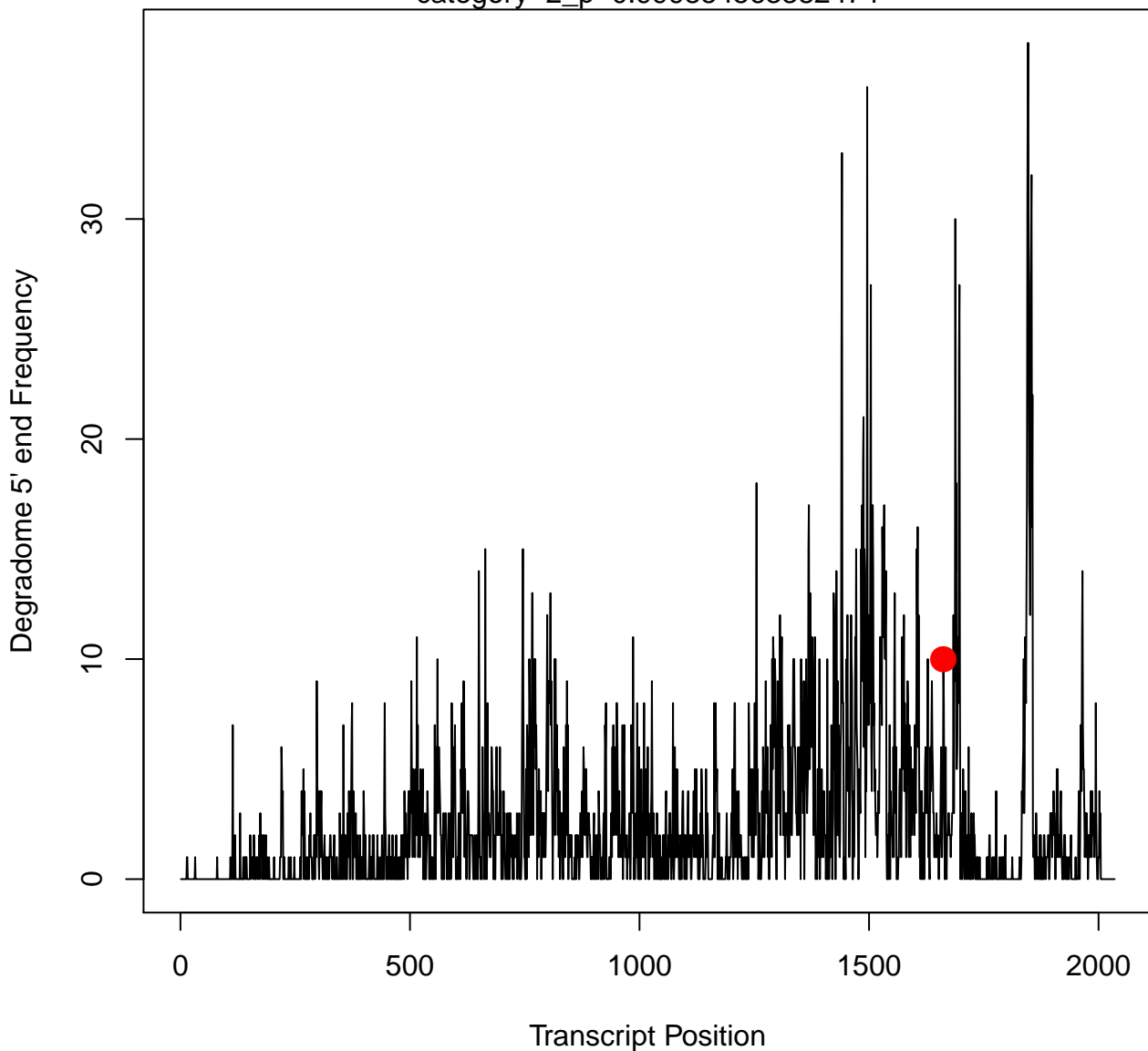

Supplement: Supplementary file 2 [file Data_Sheet_8.ZIP › GSM2230747.plot/Lsa-miR1446_Lsat_1_v5_gn_9_23820.1_1662_TPlot.pdf]

**T=Lsat\_1\_v5\_gn\_3\_31000.1\_Q=Lsa-miR156a\_S=849**

category=0\_p=0.000737666911525325

Degradome 5' end Frequency

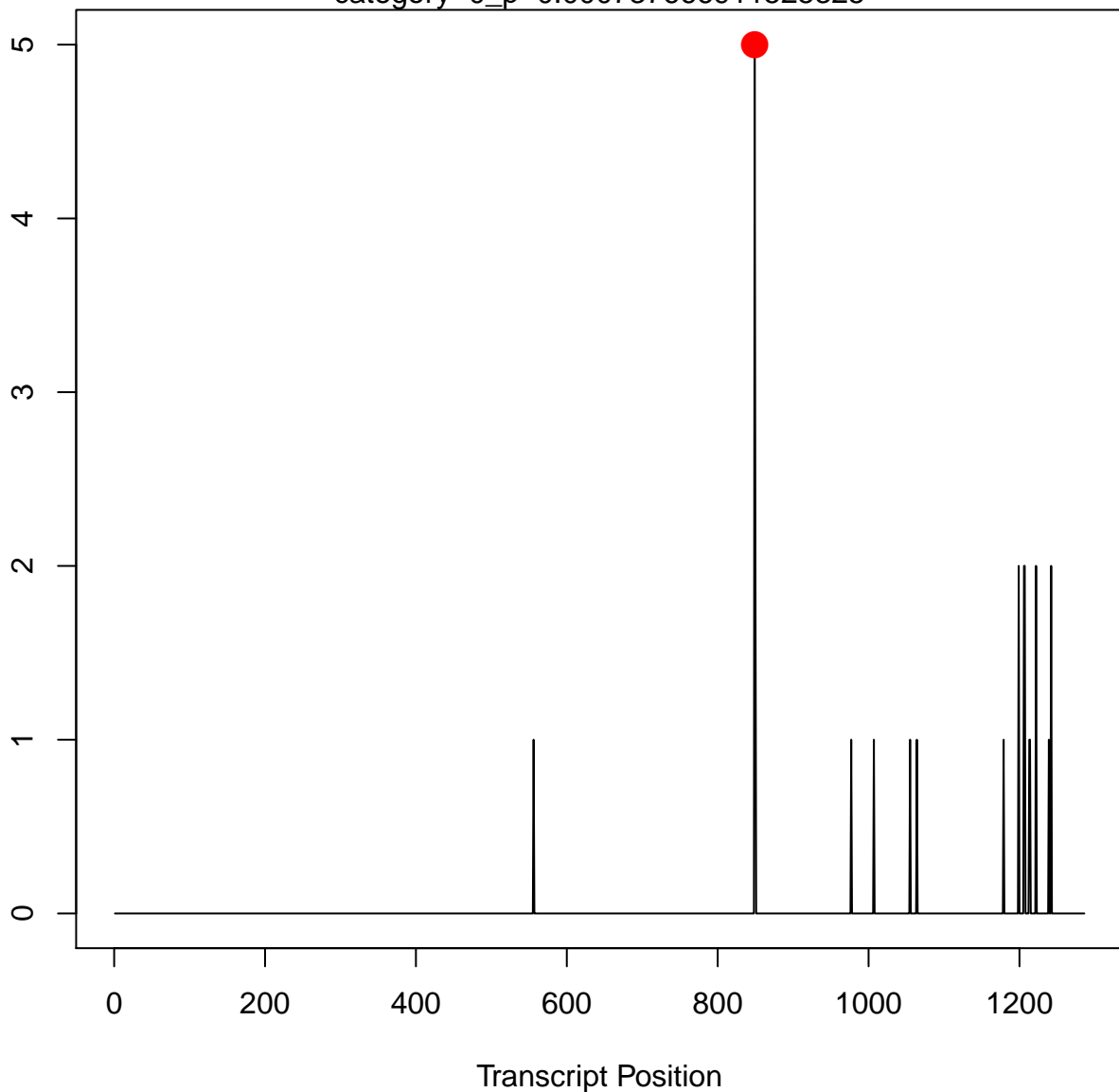

Supplement: Supplementary file 2 [file Data_Sheet_8.ZIP › GSM2230747.plot/Lsa-miR156a_Lsat_1_v5_gn_3_31000.1_849_TPlot.pdf]

**T=Lsat\_1\_v5\_gn\_3\_4041.1\_Q=Lsa-miR156a\_S=207**

category=2\_p=0.951170364658315

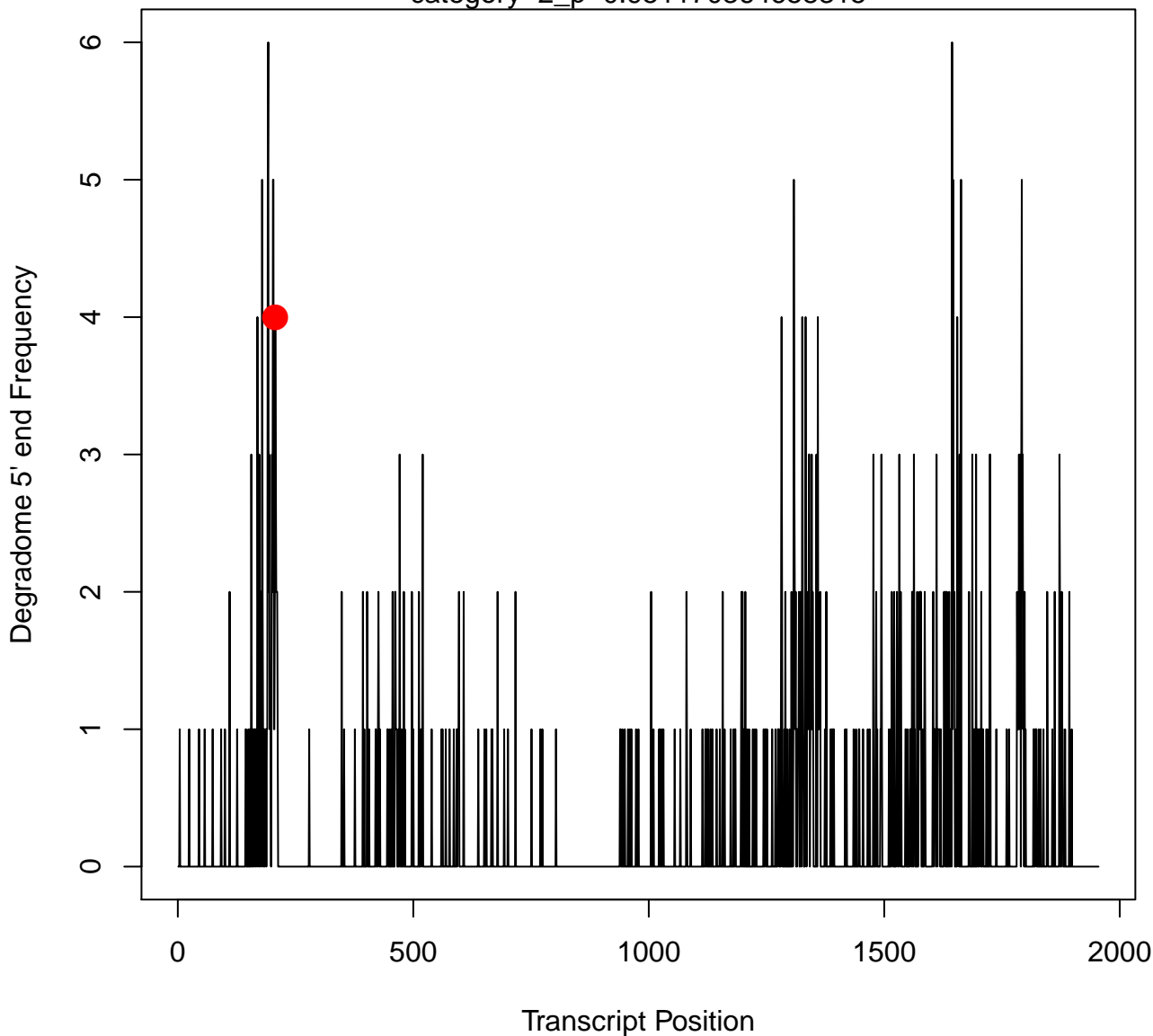

Supplement: Supplementary file 2 [file Data_Sheet_8.ZIP › GSM2230747.plot/Lsa-miR156a_Lsat_1_v5_gn_3_4041.1_207_TPlot.pdf]

**T=Lsat\_1\_v5\_gn\_4\_1141.1\_Q=Lsa-miR156d\_S=1409**

category=0\_p=0.00184314711837774

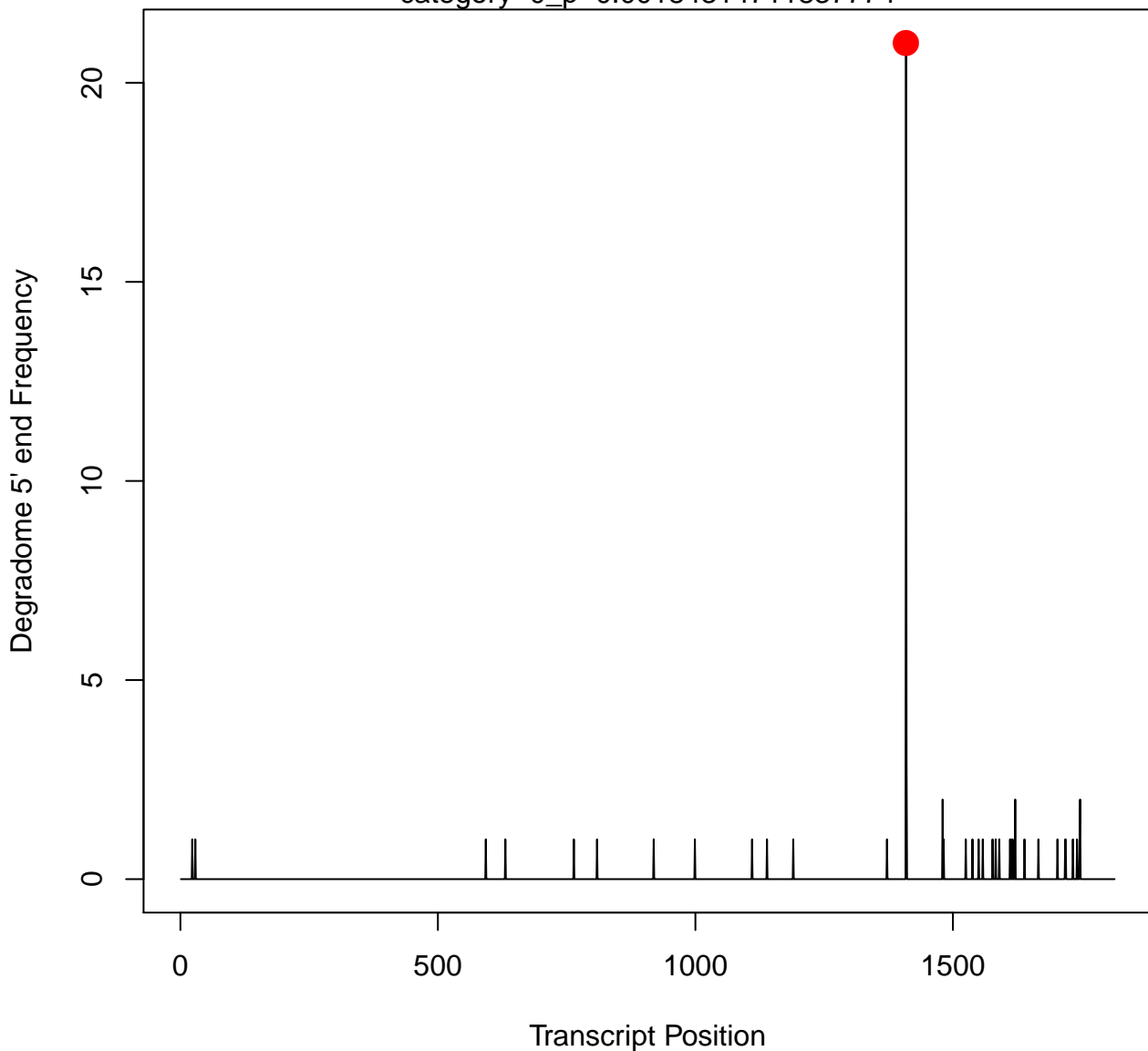

Supplement: Supplementary file 2 [file Data_Sheet_8.ZIP › GSM2230747.plot/Lsa-miR156d_Lsat_1_v5_gn_4_1141.1_1409_TPlot.pdf]

**T=Lsat\_1\_v5\_gn\_1\_50821.1\_Q=Lsa-miR156h\_S=1265**

category=2\_p=0.94796564871546

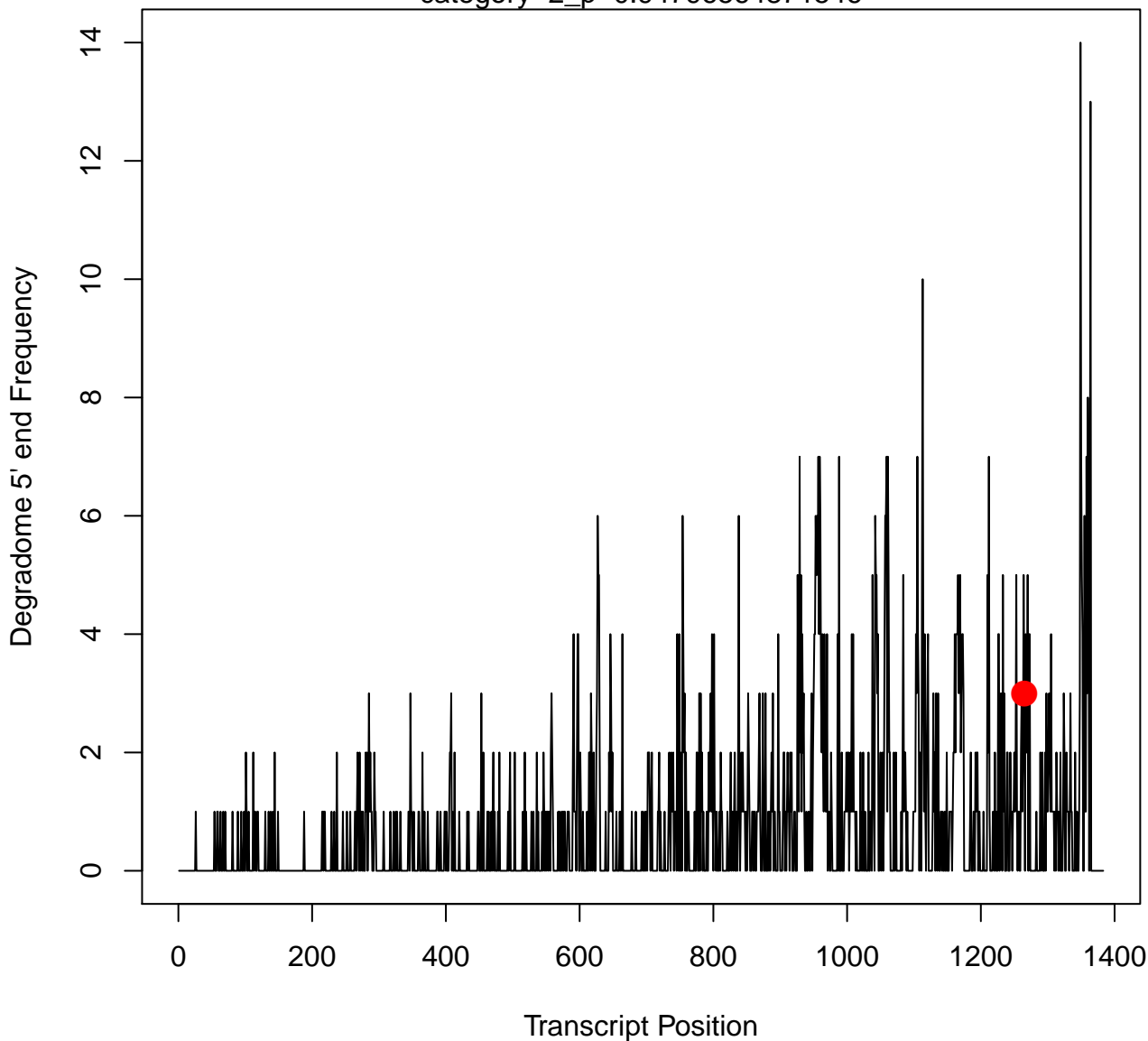

Supplement: Supplementary file 2 [file Data_Sheet_8.ZIP › GSM2230747.plot/Lsa-miR156h_Lsat_1_v5_gn_1_50821.1_1265_TPlot.pdf]

**T=Lsat\_1\_v5\_gn\_2\_88681.1\_Q=Lsa-miR156i\_S=2002**

category=2\_p=0.930733996489211

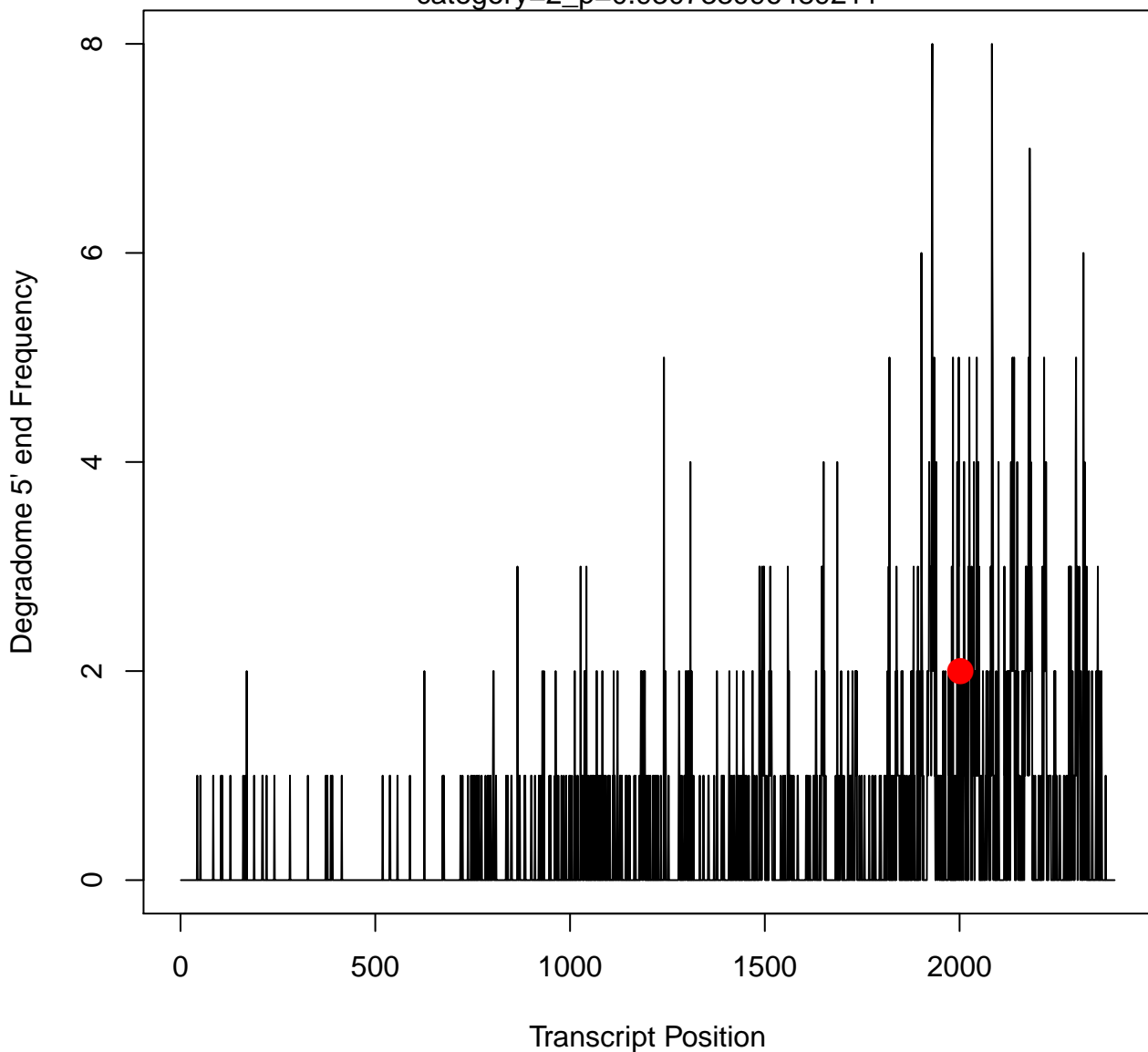

Supplement: Supplementary file 2 [file Data_Sheet_8.ZIP › GSM2230747.plot/Lsa-miR156i_Lsat_1_v5_gn_2_88681.1_2002_TPlot.pdf]

**T=Lsat\_1\_v5\_gn\_5\_12360.1\_Q=Lsa-miR156i\_S=1859**

category=0\_p=0.00110629628501624

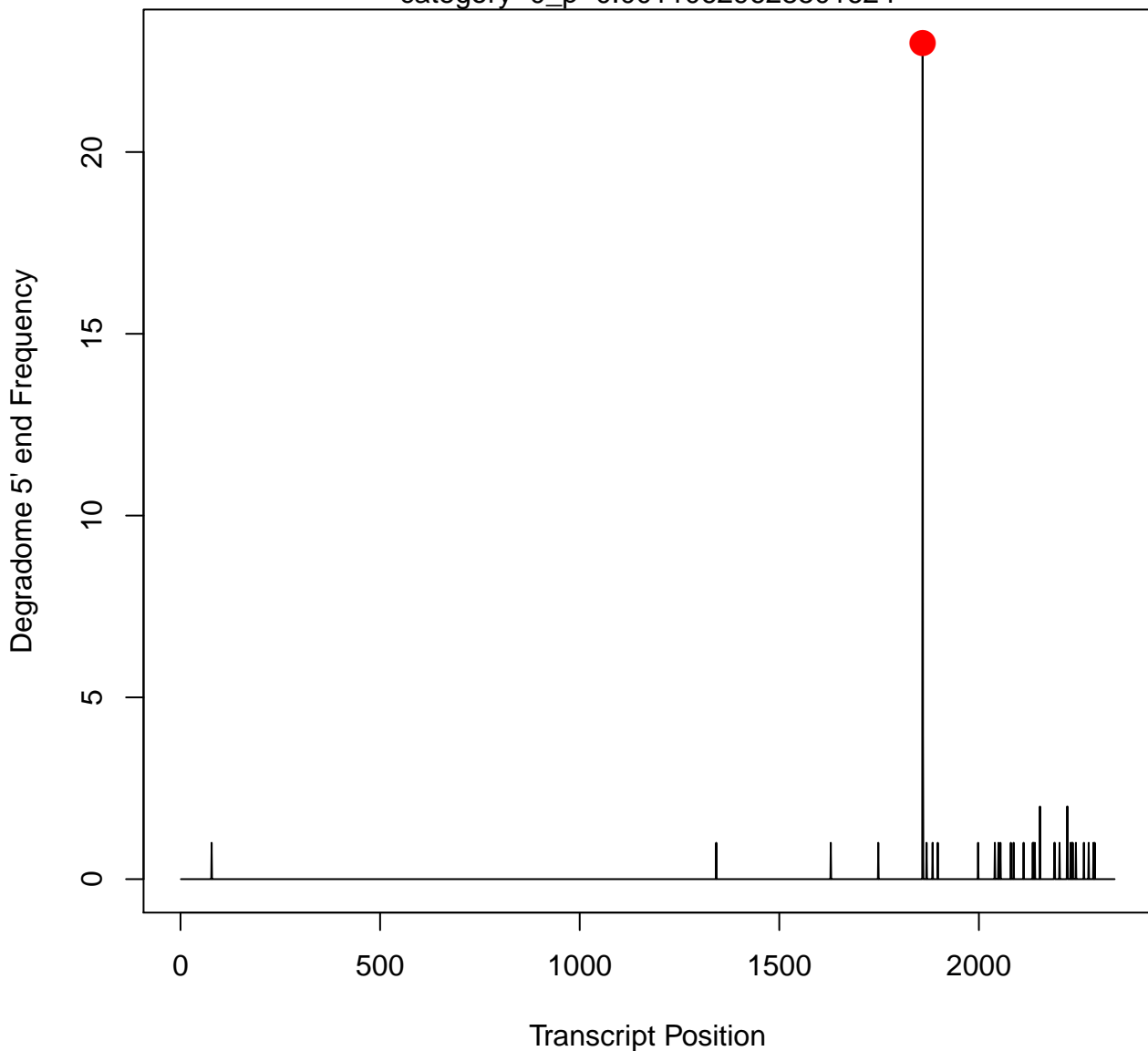

Supplement: Supplementary file 2 [file Data_Sheet_8.ZIP › GSM2230747.plot/Lsa-miR156i_Lsat_1_v5_gn_5_12360.1_1859_TPlot.pdf]

**T=Lsat\_1\_v5\_gn\_8\_134640.1\_Q=Lsa-miR156i\_S=546**

category=2\_p=0.119383795306115

Degradome 5' end Frequency

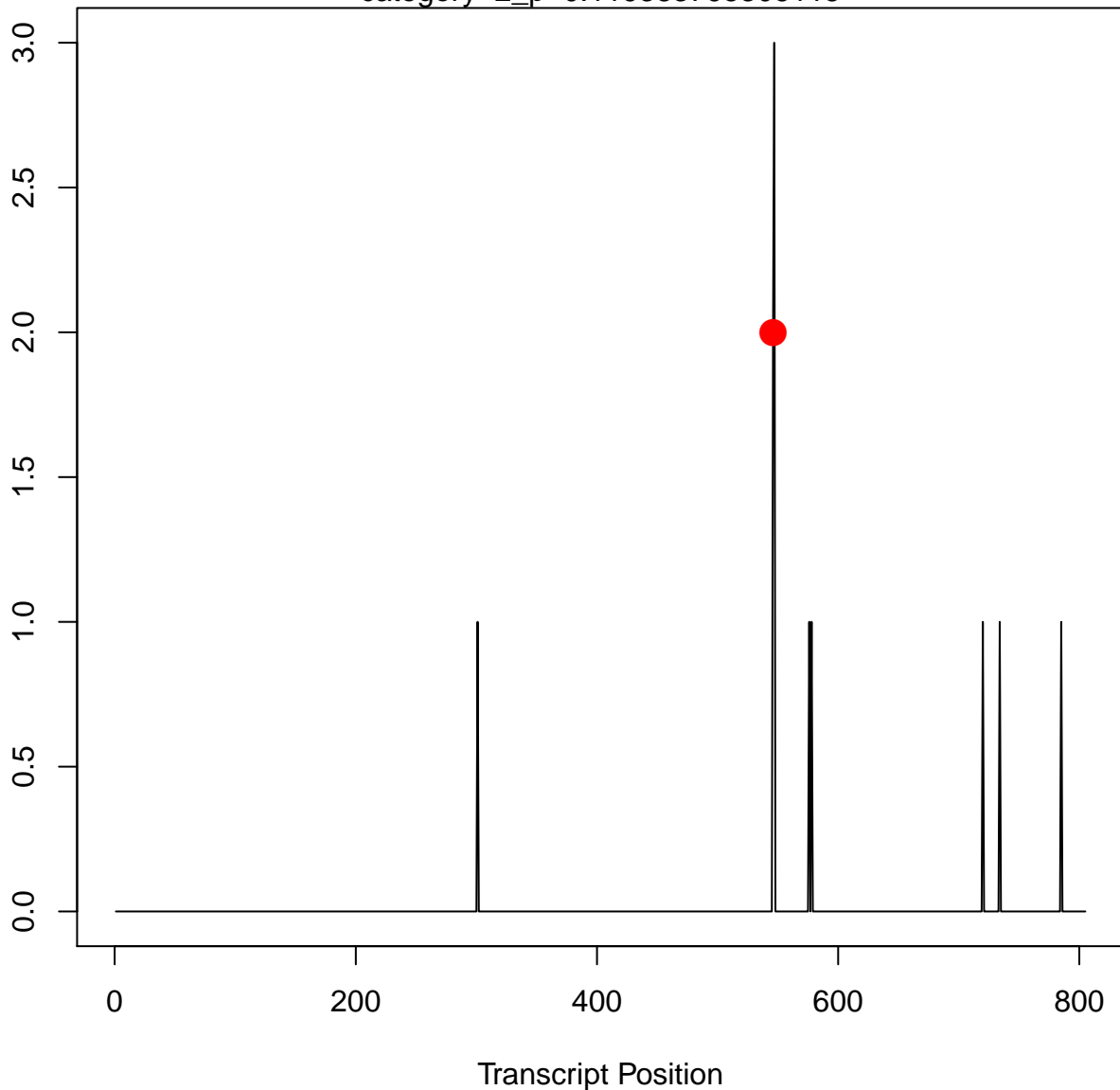

Supplement: Supplementary file 2 [file Data_Sheet_8.ZIP › GSM2230747.plot/Lsa-miR156i_Lsat_1_v5_gn_8_134640.1_546_TPlot.pdf]

**T=Lsat\_1\_v5\_gn\_9\_28021.1\_Q=Lsa-miR156i\_S=1267**

category=2\_p=0.0615884673055831

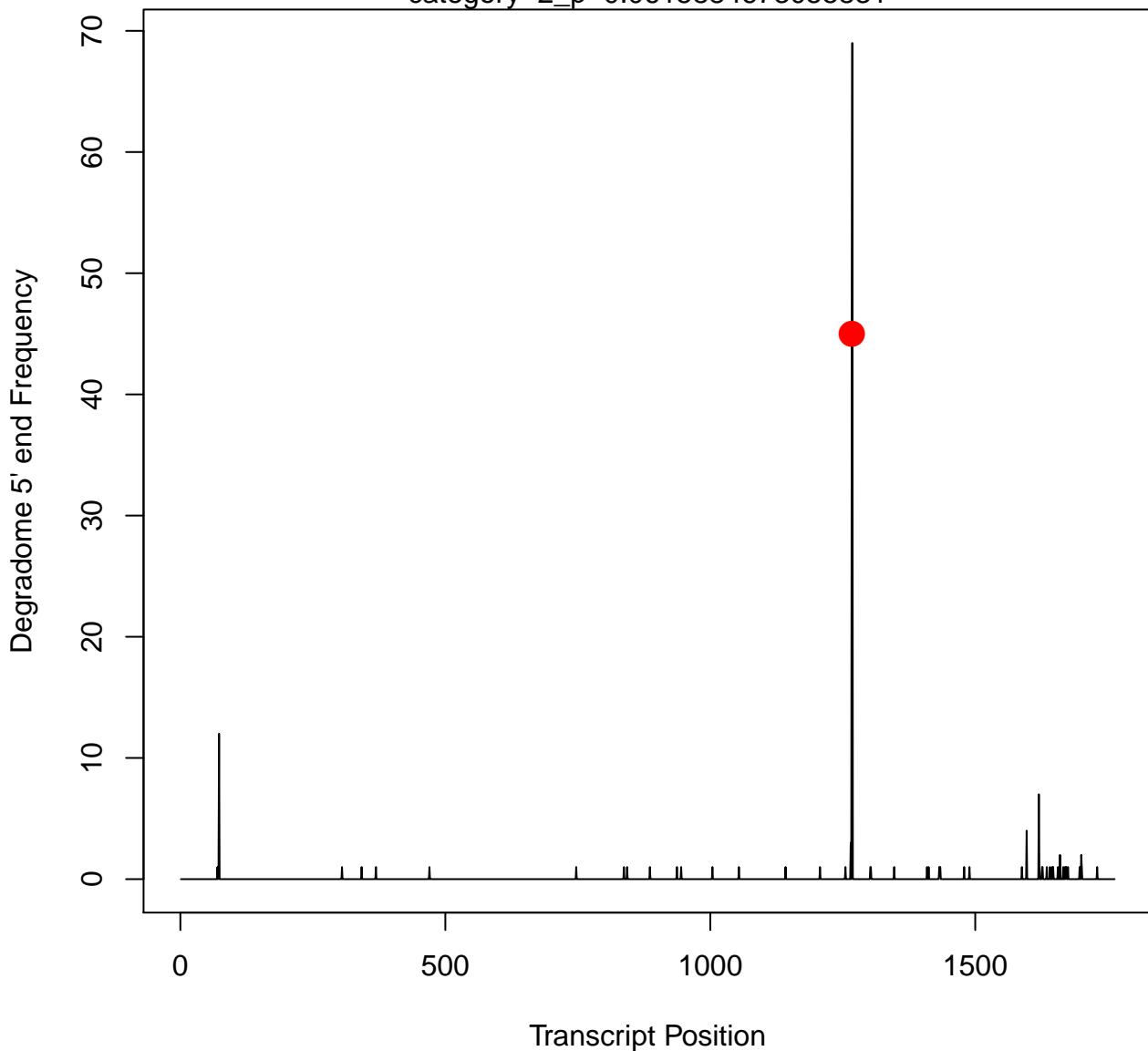

Supplement: Supplementary file 2 [file Data_Sheet_8.ZIP › GSM2230747.plot/Lsa-miR156i_Lsat_1_v5_gn_9_28021.1_1267_TPlot.pdf]

**T=Lsat\_1\_v5\_gn\_9\_65321.1\_Q=Lsa-miR156i\_S=1998**

category=2\_p=0.533641349080958

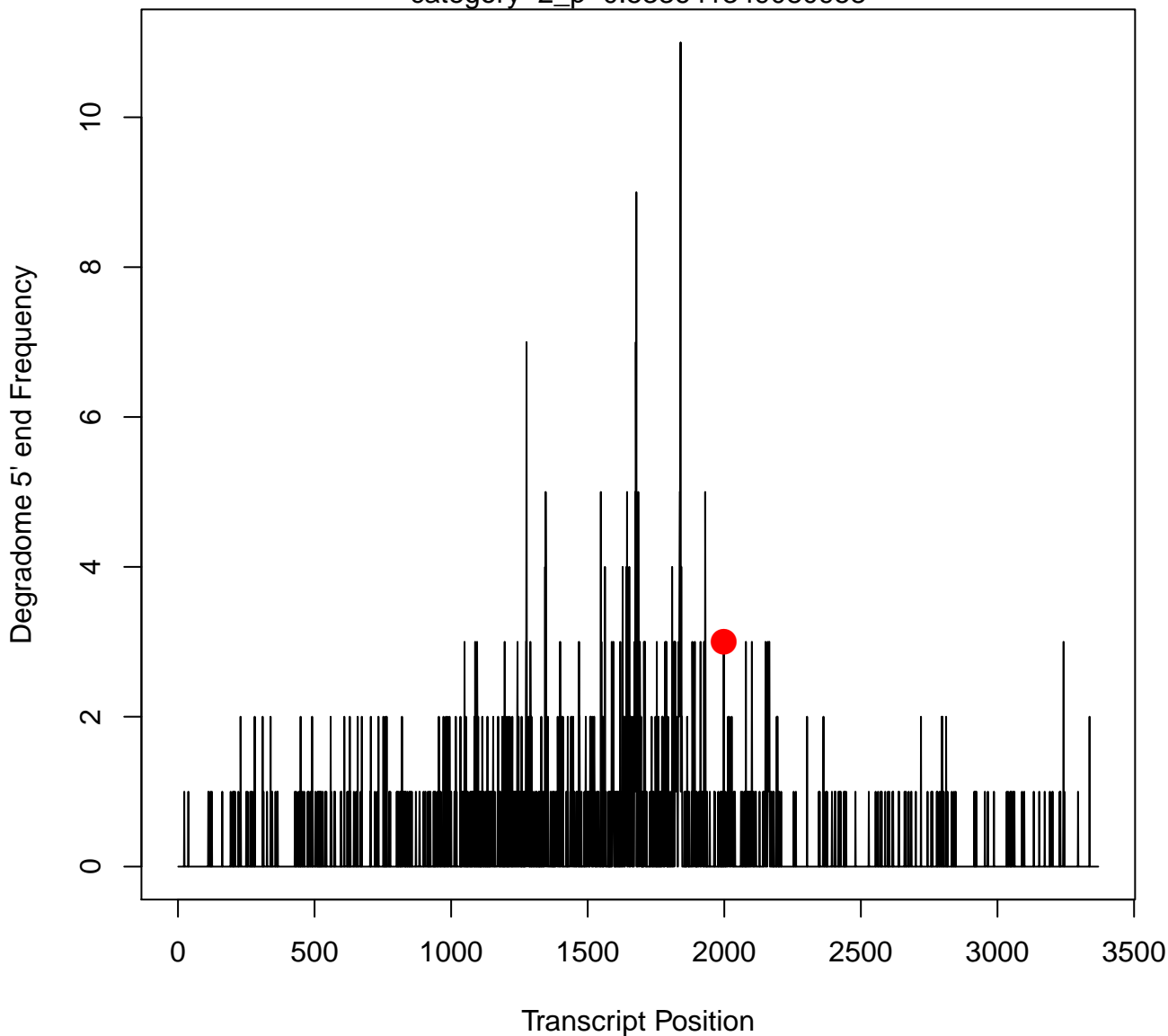

Supplement: Supplementary file 2 [file Data_Sheet_8.ZIP › GSM2230747.plot/Lsa-miR156i_Lsat_1_v5_gn_9_65321.1_1998_TPlot.pdf]

**T=Lsat\_1\_v5\_gn\_1\_126340.1\_Q=Lsa-miR156j\_S=1034**

category=2\_p=0.944550605494866

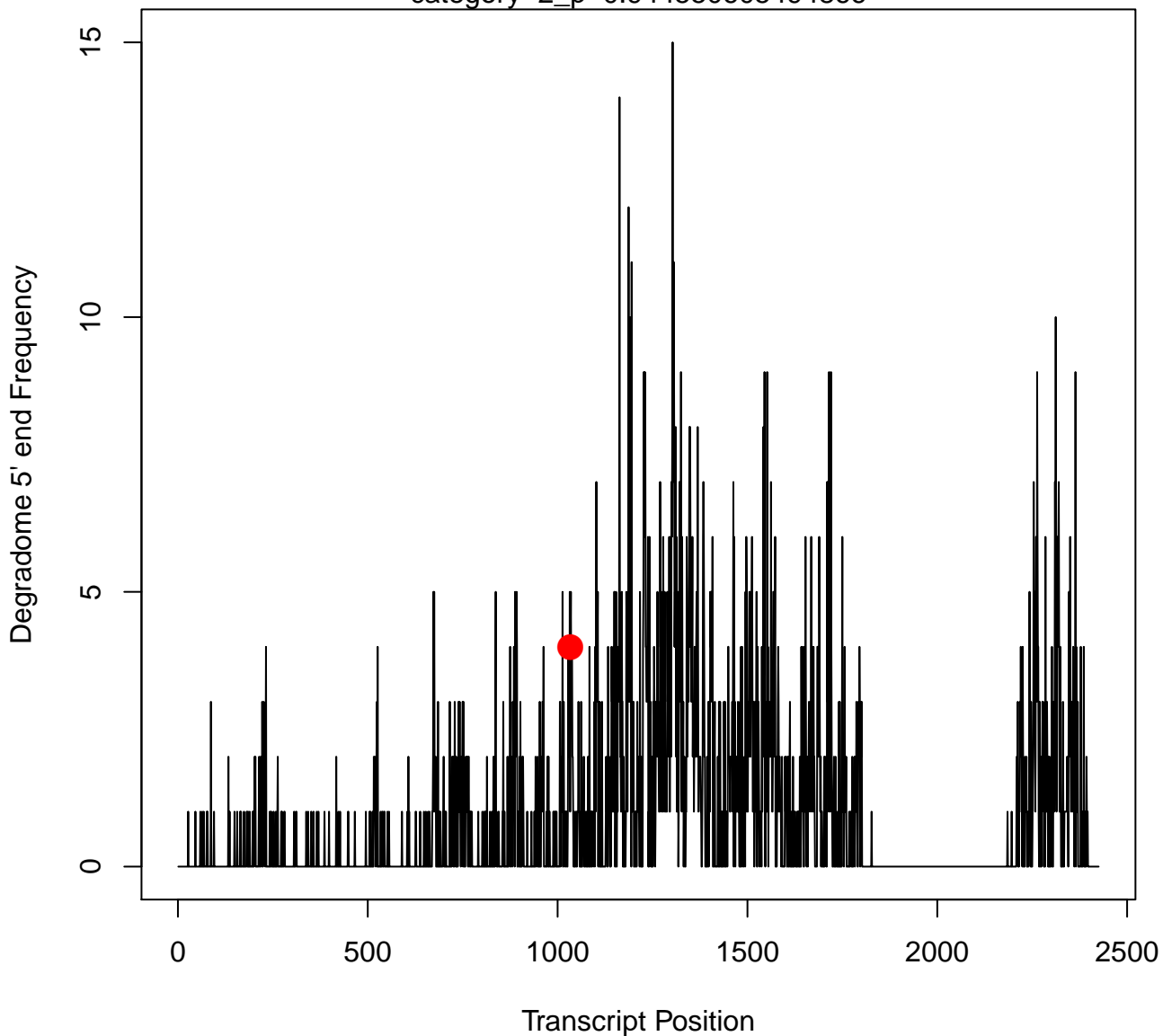

Supplement: Supplementary file 2 [file Data_Sheet_8.ZIP › GSM2230747.plot/Lsa-miR156j_Lsat_1_v5_gn_1_126340.1_1034_TPlot.pdf]

T=Lsat\_1\_v5\_gn\_1\_14560.1\_Q=Lsa-miR156j\_S=192

category=0\_p=0.000368901499921082

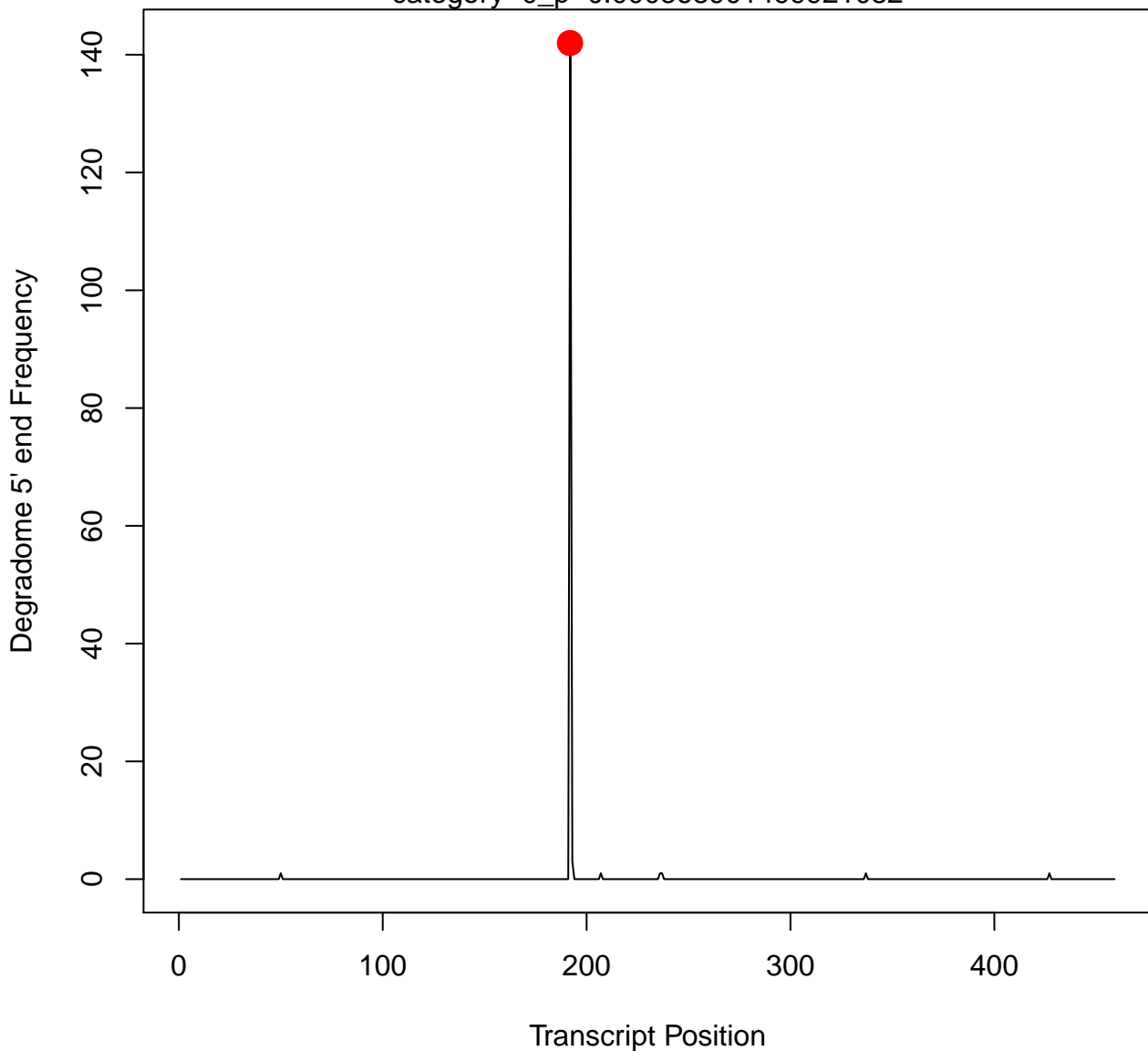

Supplement: Supplementary file 2 [file Data_Sheet_8.ZIP › GSM2230747.plot/Lsa-miR156j_Lsat_1_v5_gn_1_14560.1_192_TPlot.pdf]

T=Lsat\_1\_v5\_gn\_3\_8741.1\_Q=Lsa-miR156j\_S=164

category=2\_p=0.942759932148723

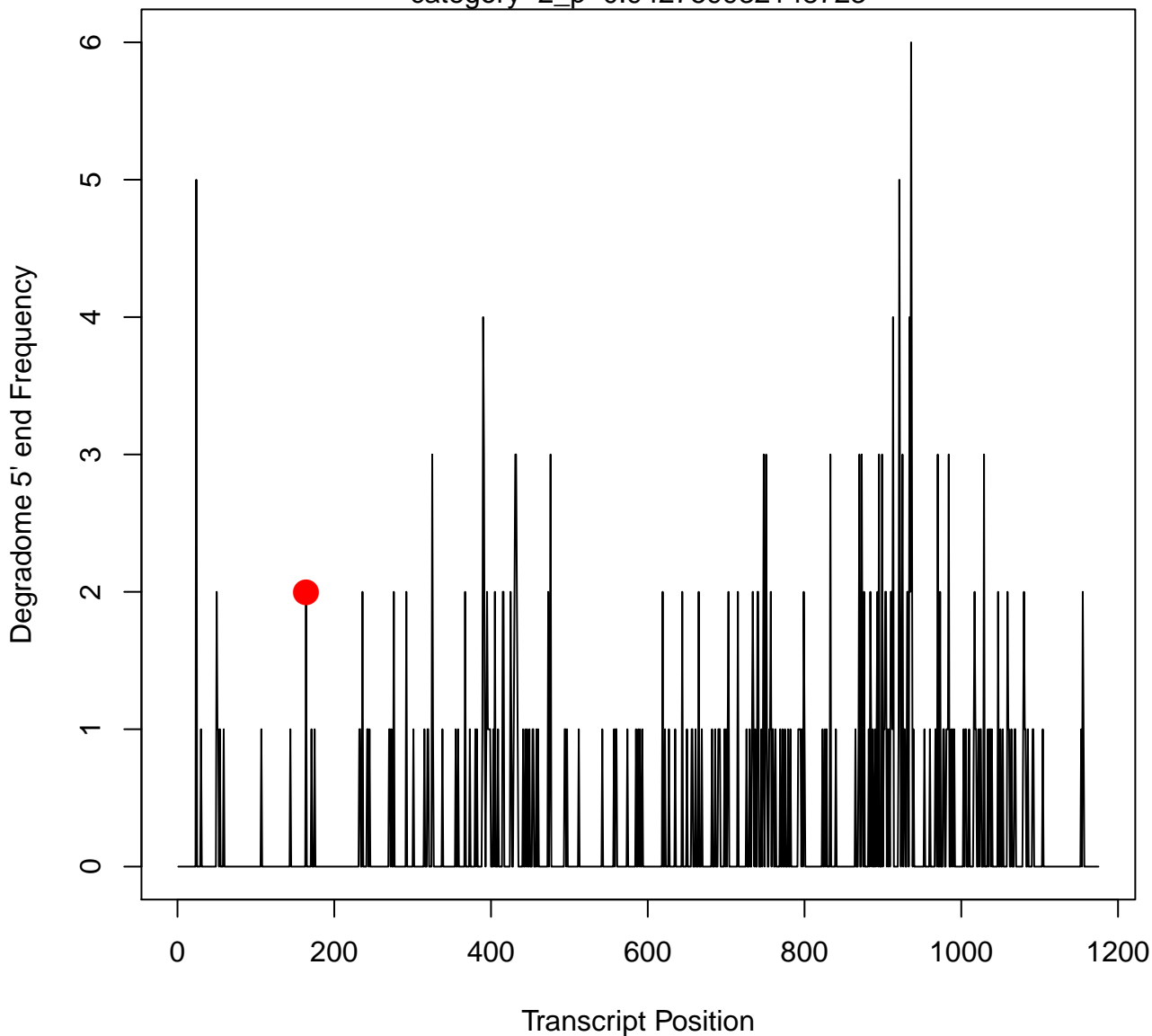

Supplement: Supplementary file 2 [file Data_Sheet_8.ZIP › GSM2230747.plot/Lsa-miR156j_Lsat_1_v5_gn_3_8741.1_164_TPlot.pdf]

**T=Lsat\_1\_v5\_gn\_4\_139200.1\_Q=Lsa-miR156j\_S=170**

category=0\_p=0.00845039290014593

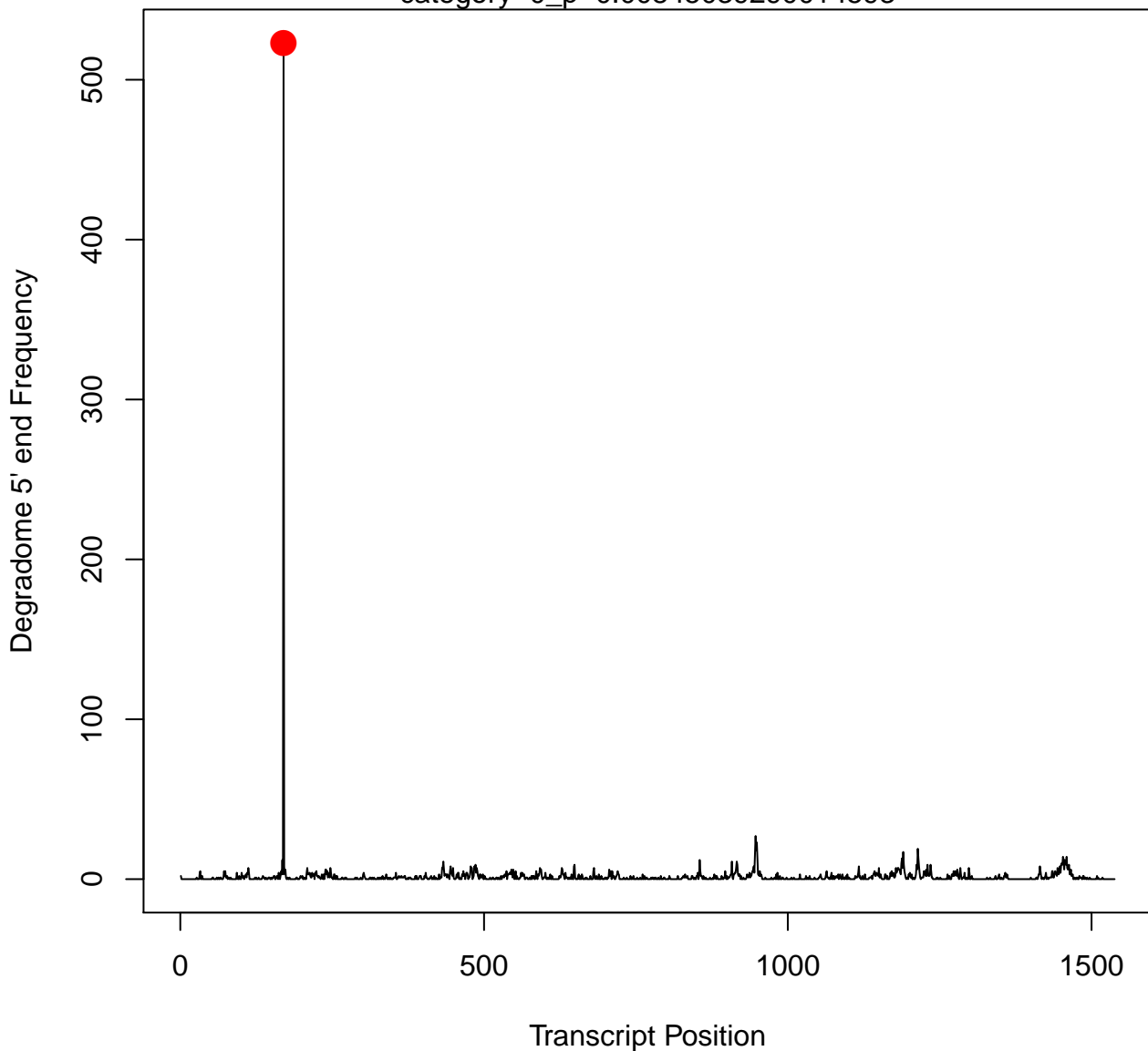

Supplement: Supplementary file 2 [file Data_Sheet_8.ZIP › GSM2230747.plot/Lsa-miR156j_Lsat_1_v5_gn_4_139200.1_170_TPlot.pdf]

**T=Lsat\_1\_v5\_gn\_4\_183040.1\_Q=Lsa-miR156j\_S=941**

category=2\_p=0.979298895249089

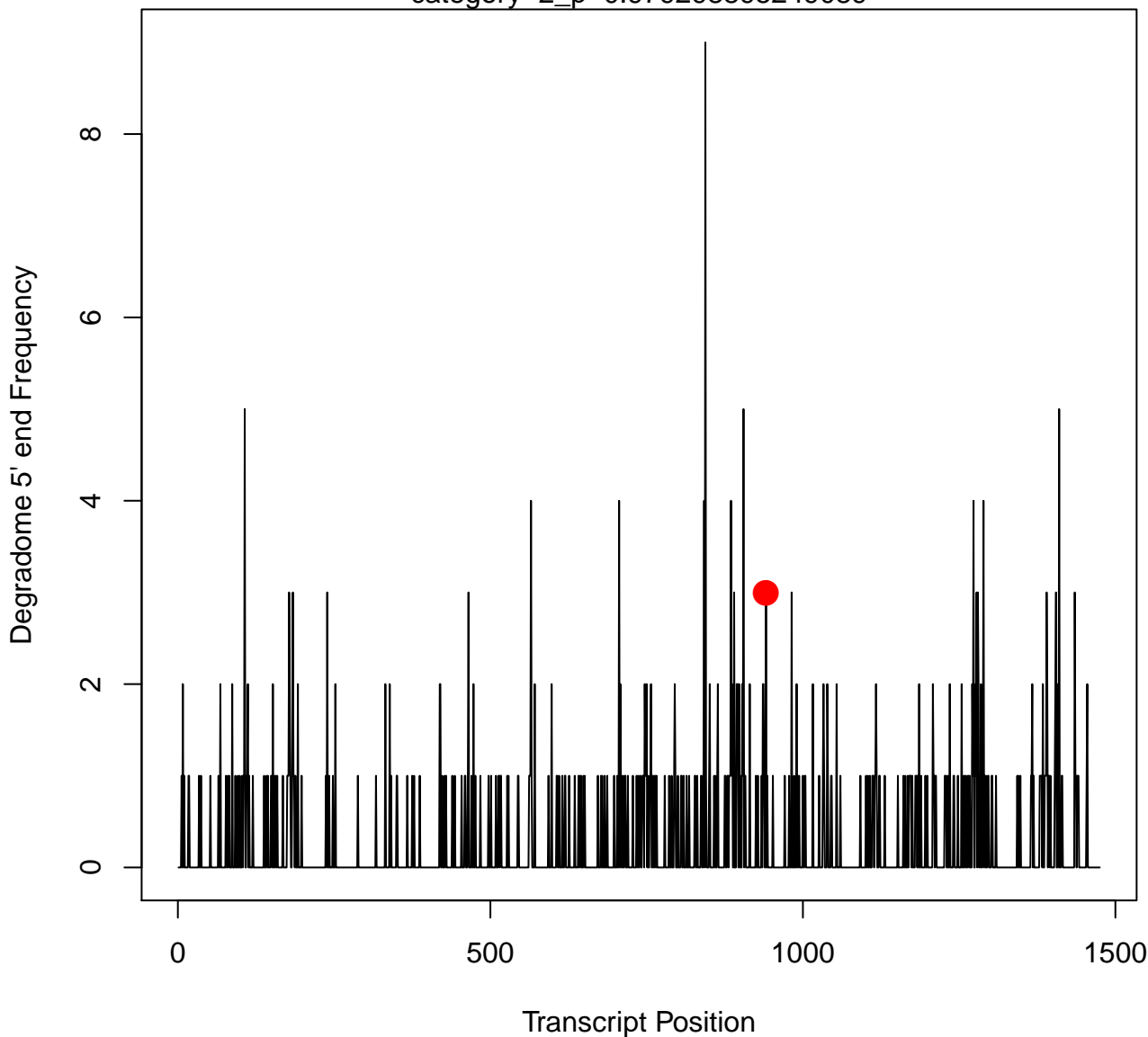

Supplement: Supplementary file 2 [file Data_Sheet_8.ZIP › GSM2230747.plot/Lsa-miR156j_Lsat_1_v5_gn_4_183040.1_941_TPlot.pdf]

**T=Lsat\_1\_v5\_gn\_4\_280.1\_Q=Lsa-miR156j\_S=1168**

category=2\_p=0.602164642397273

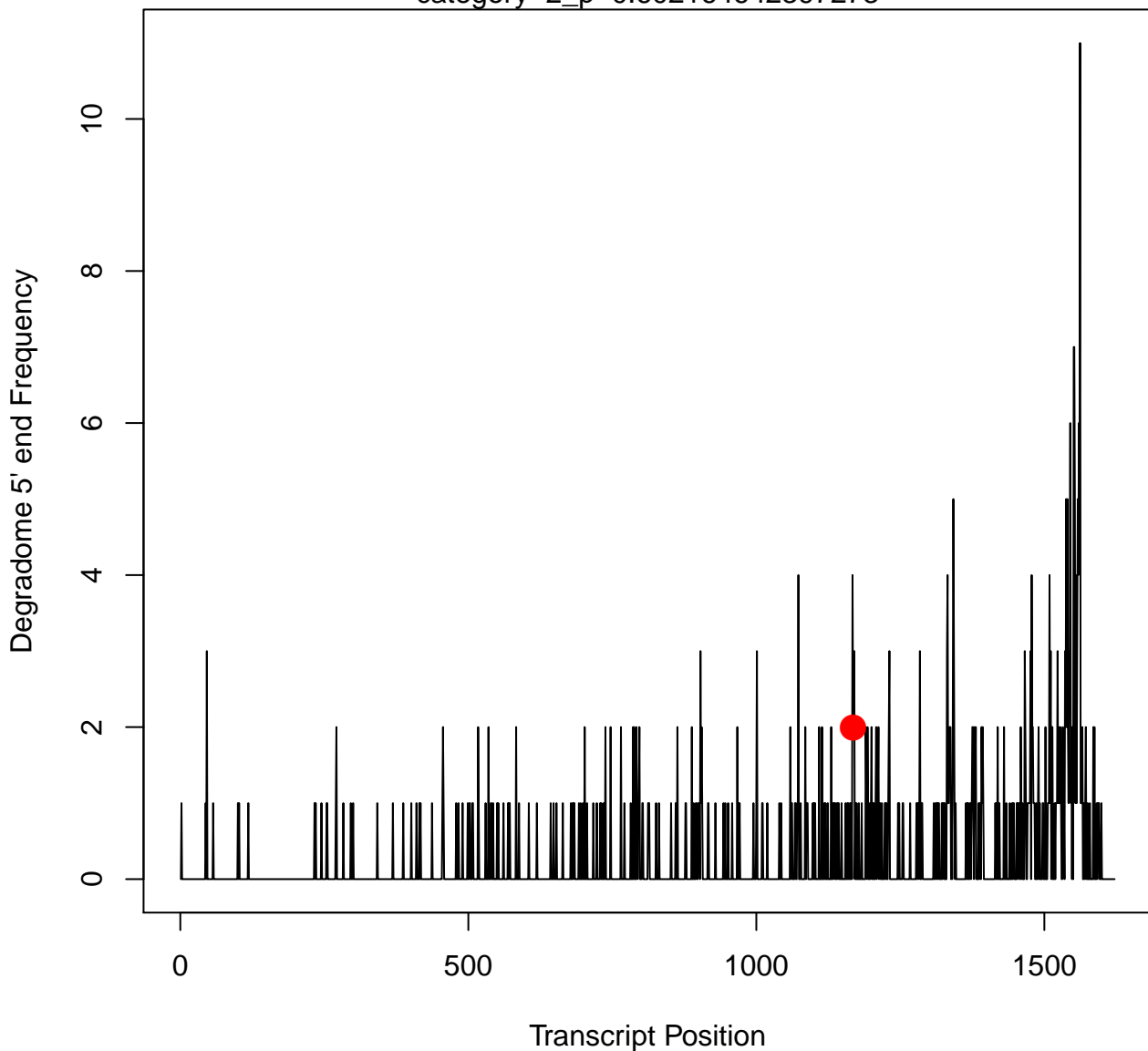

Supplement: Supplementary file 2 [file Data_Sheet_8.ZIP › GSM2230747.plot/Lsa-miR156j_Lsat_1_v5_gn_4_280.1_1168_TPlot.pdf]

**T=Lsat\_1\_v5\_gn\_4\_64540.1\_Q=Lsa-miR156j\_S=748**

category=2\_p=0.926188030413576

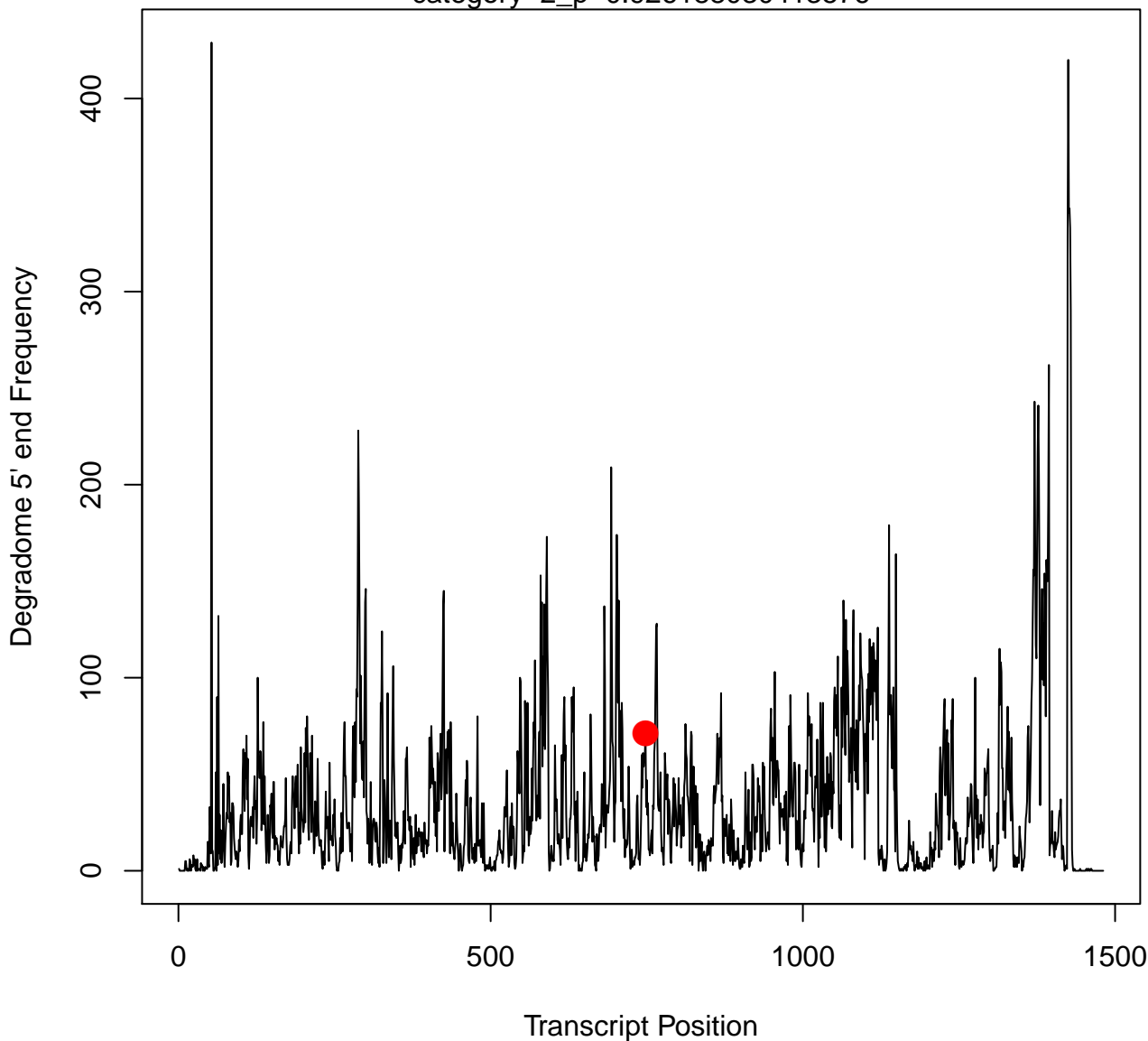

Supplement: Supplementary file 2 [file Data_Sheet_8.ZIP › GSM2230747.plot/Lsa-miR156j_Lsat_1_v5_gn_4_64540.1_748_TPlot.pdf]

**T=Lsat\_1\_v5\_gn\_4\_7021.1\_Q=Lsa-miR156j\_S=627**

category=2\_p=0.831339485668459

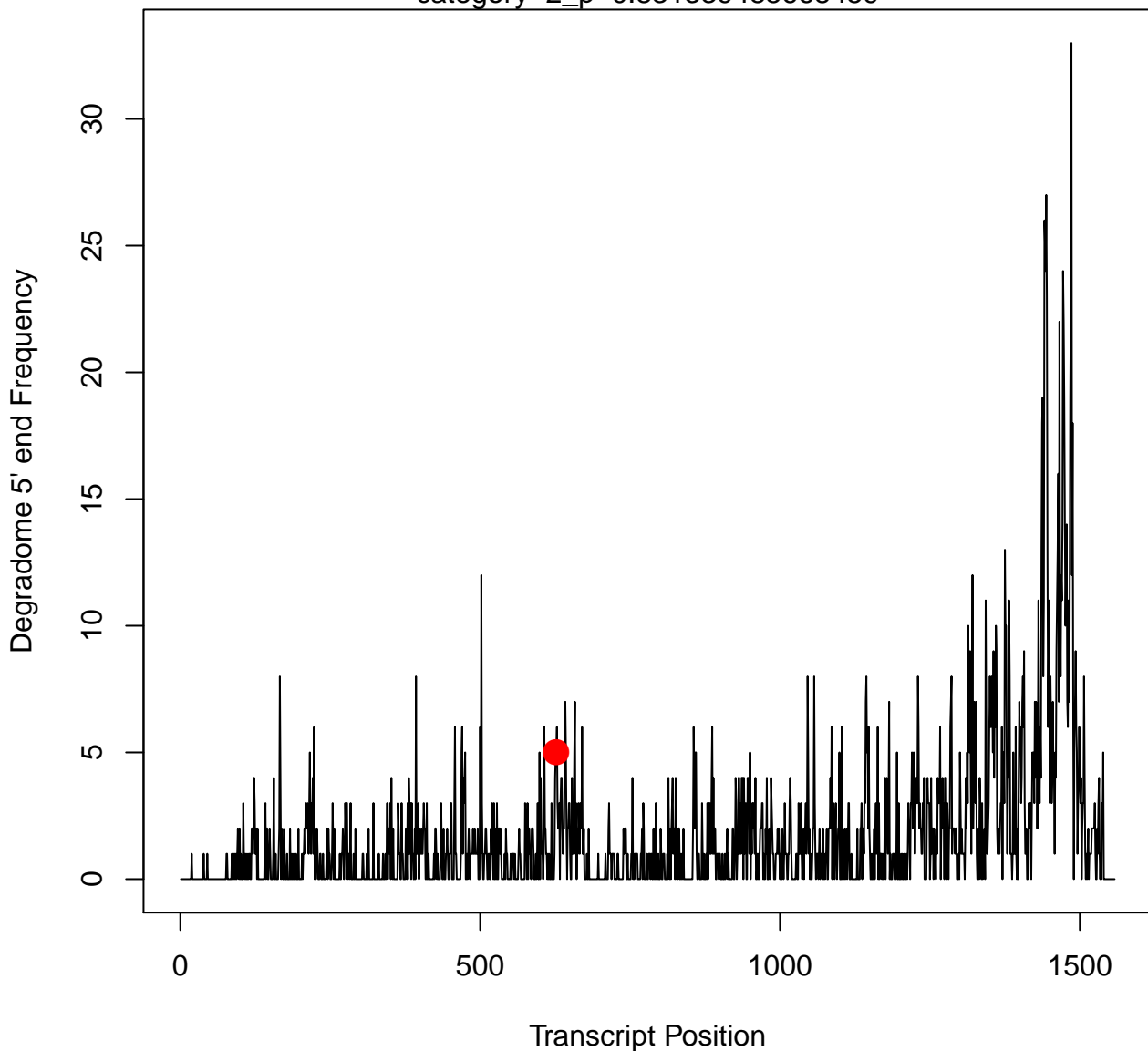

Supplement: Supplementary file 2 [file Data_Sheet_8.ZIP › GSM2230747.plot/Lsa-miR156j_Lsat_1_v5_gn_4_7021.1_627_TPlot.pdf]

T=Lsat\_1\_v5\_gn\_4\_81541.1\_Q=Lsa-miR156j\_S=850

category=0\_p=0.00368289704545566

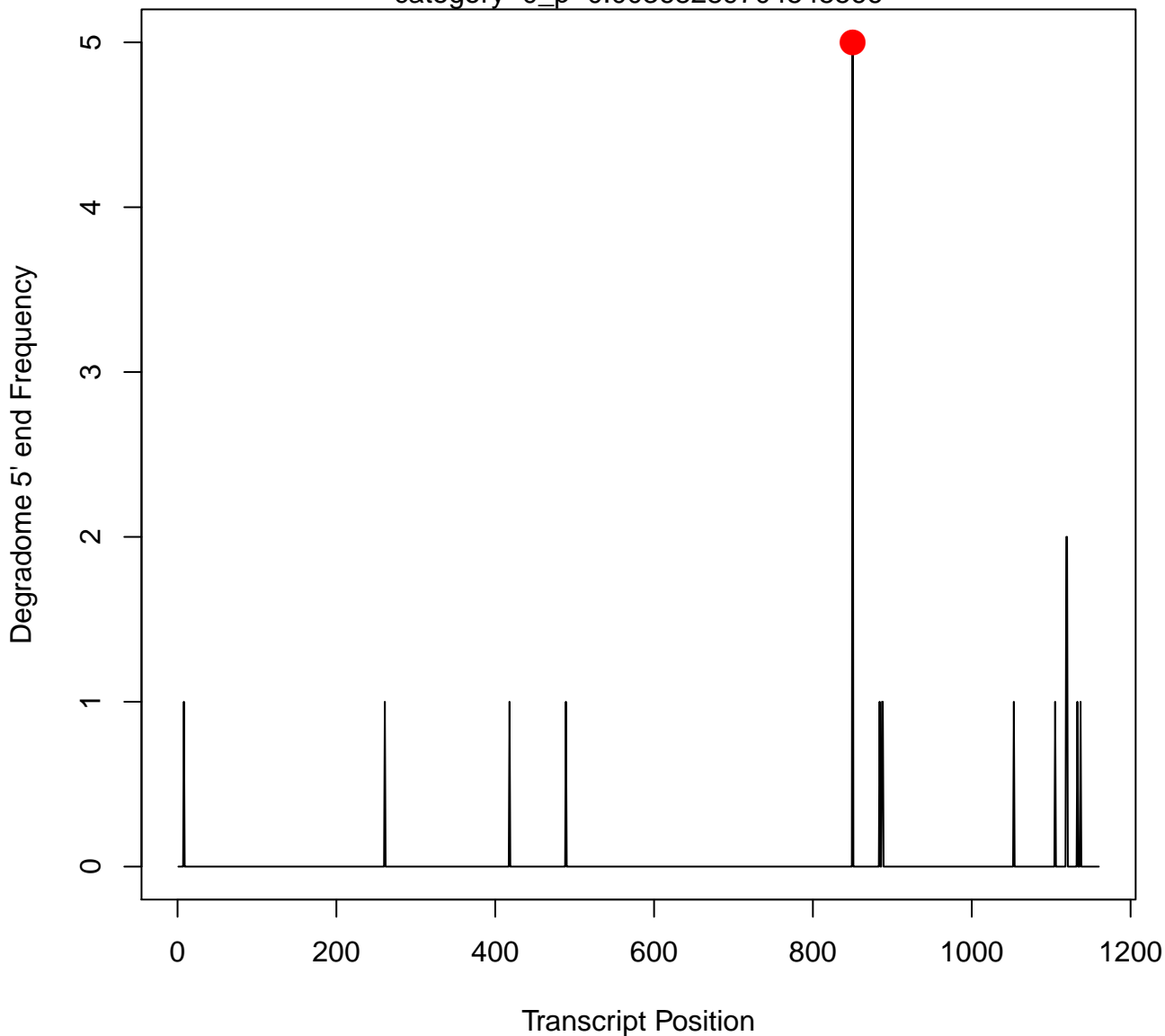

Supplement: Supplementary file 2 [file Data_Sheet_8.ZIP › GSM2230747.plot/Lsa-miR156j_Lsat_1_v5_gn_4_81541.1_850_TPlot.pdf]

**T=Lsat\_1\_v5\_gn\_5\_149040.1\_Q=Lsa-miR156j\_S=1399**

category=2\_p=0.649659737294852

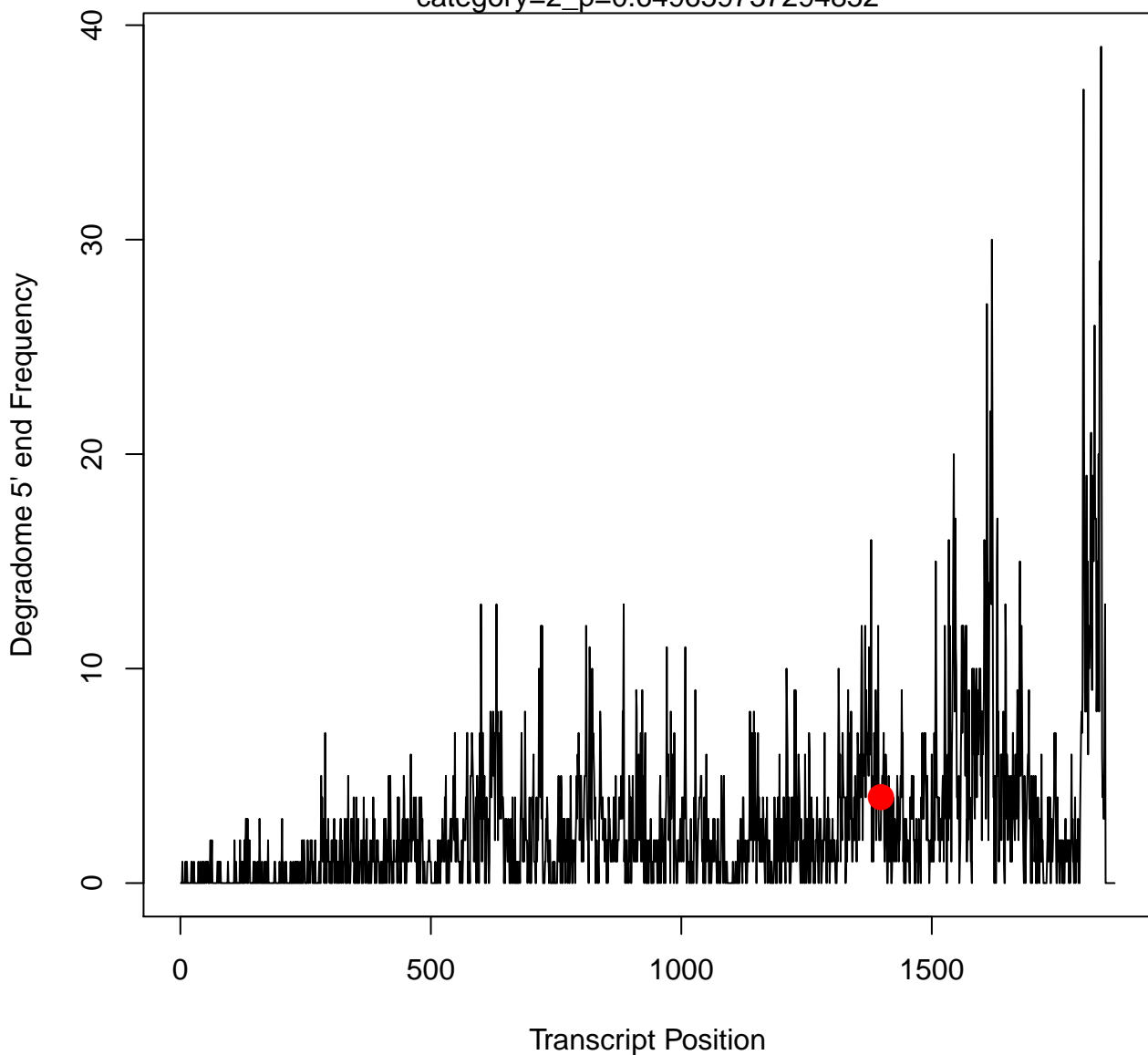

Supplement: Supplementary file 2 [file Data_Sheet_8.ZIP › GSM2230747.plot/Lsa-miR156j_Lsat_1_v5_gn_5_149040.1_1399_TPlot.pdf]

**T=Lsat\_1\_v5\_gn\_5\_42660.1\_Q=Lsa-miR156j\_S=1691**

category=2\_p=0.681521546304191

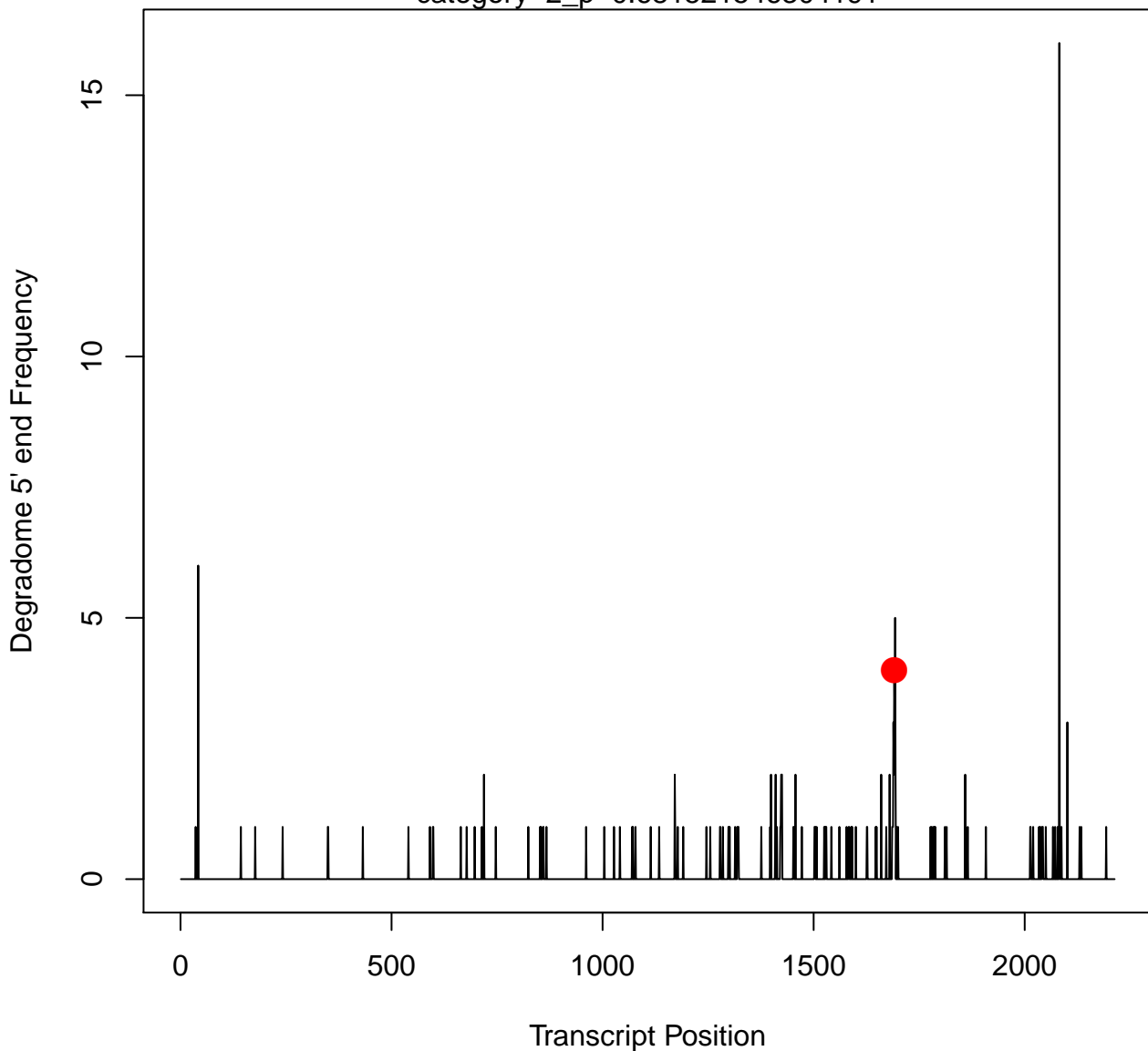

Supplement: Supplementary file 2 [file Data_Sheet_8.ZIP › GSM2230747.plot/Lsa-miR156j_Lsat_1_v5_gn_5_42660.1_1691_TPlot.pdf]

**T=Lsat\_1\_v5\_gn\_1\_128400.1\_Q=Lsa-miR157a\_S=525**

category=0\_p=0.00331521853462458

Degradome 5' end Frequency

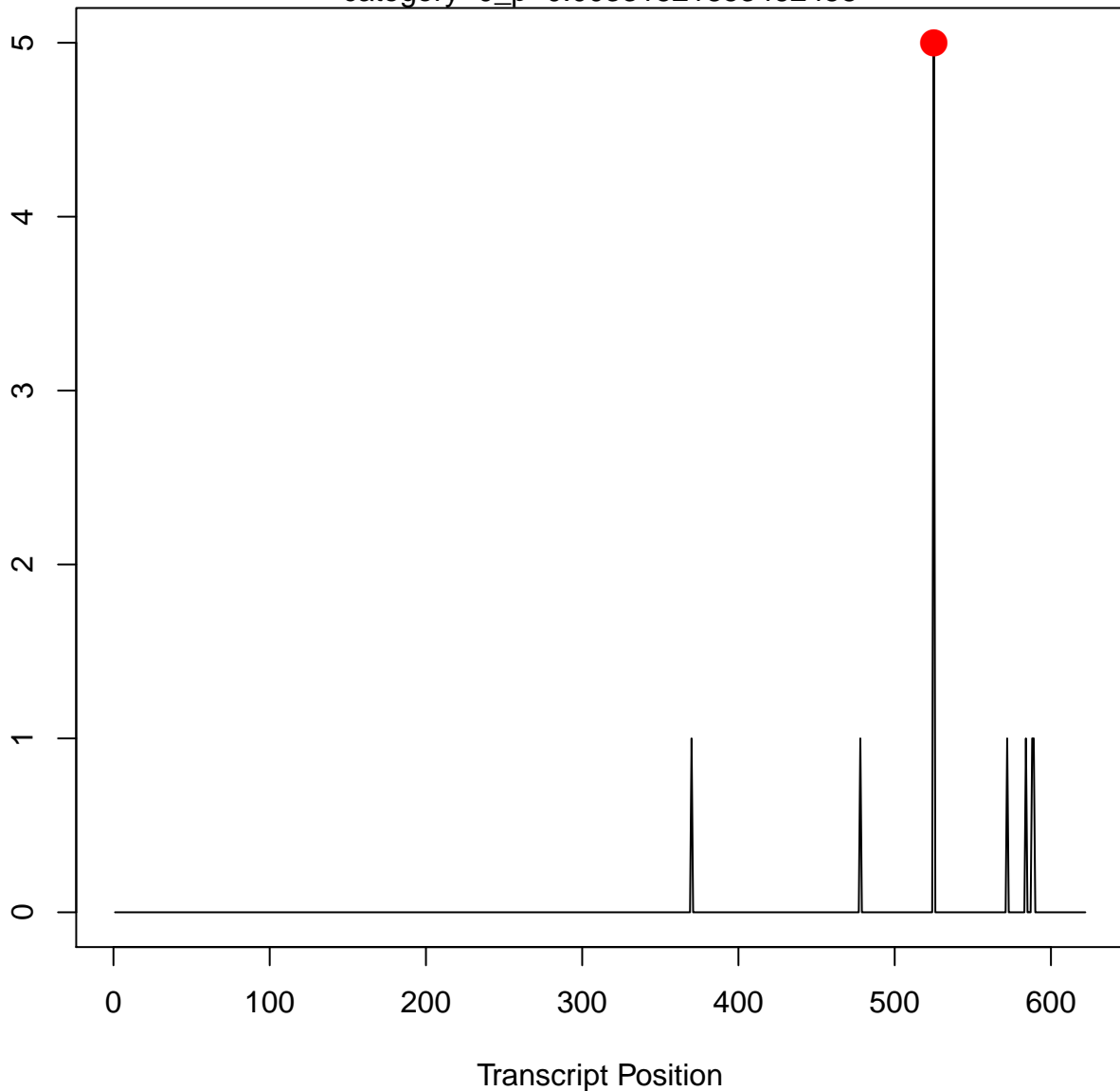

Supplement: Supplementary file 2 [file Data_Sheet_8.ZIP › GSM2230747.plot/Lsa-miR157a_Lsat_1_v5_gn_1_128400.1_525_TPlot.pdf]

**T=Lsat\_1\_v5\_gn\_1\_25881.1\_Q=Lsa-miR157a\_S=213**

category=2\_p=0.760751033750871

Degradome 5' end Frequency

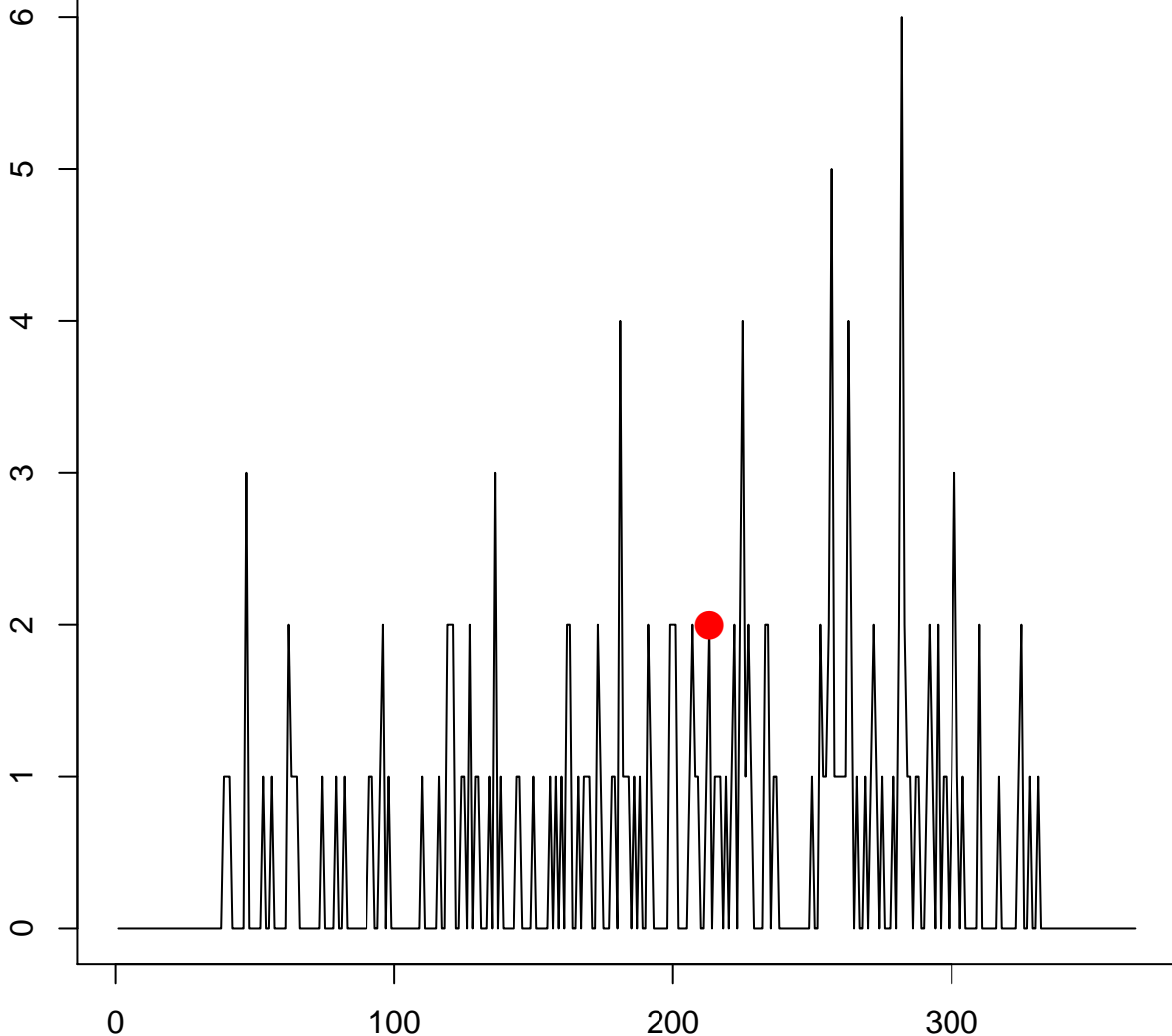

Transcript Position

Supplement: Supplementary file 2 [file Data_Sheet_8.ZIP › GSM2230747.plot/Lsa-miR157a_Lsat_1_v5_gn_1_25881.1_213_TPlot.pdf]

**T=Lsat\_1\_v5\_gn\_4\_113241.1\_Q=Lsa-miR157a\_S=869**

category=0\_p=0.00405043991913256

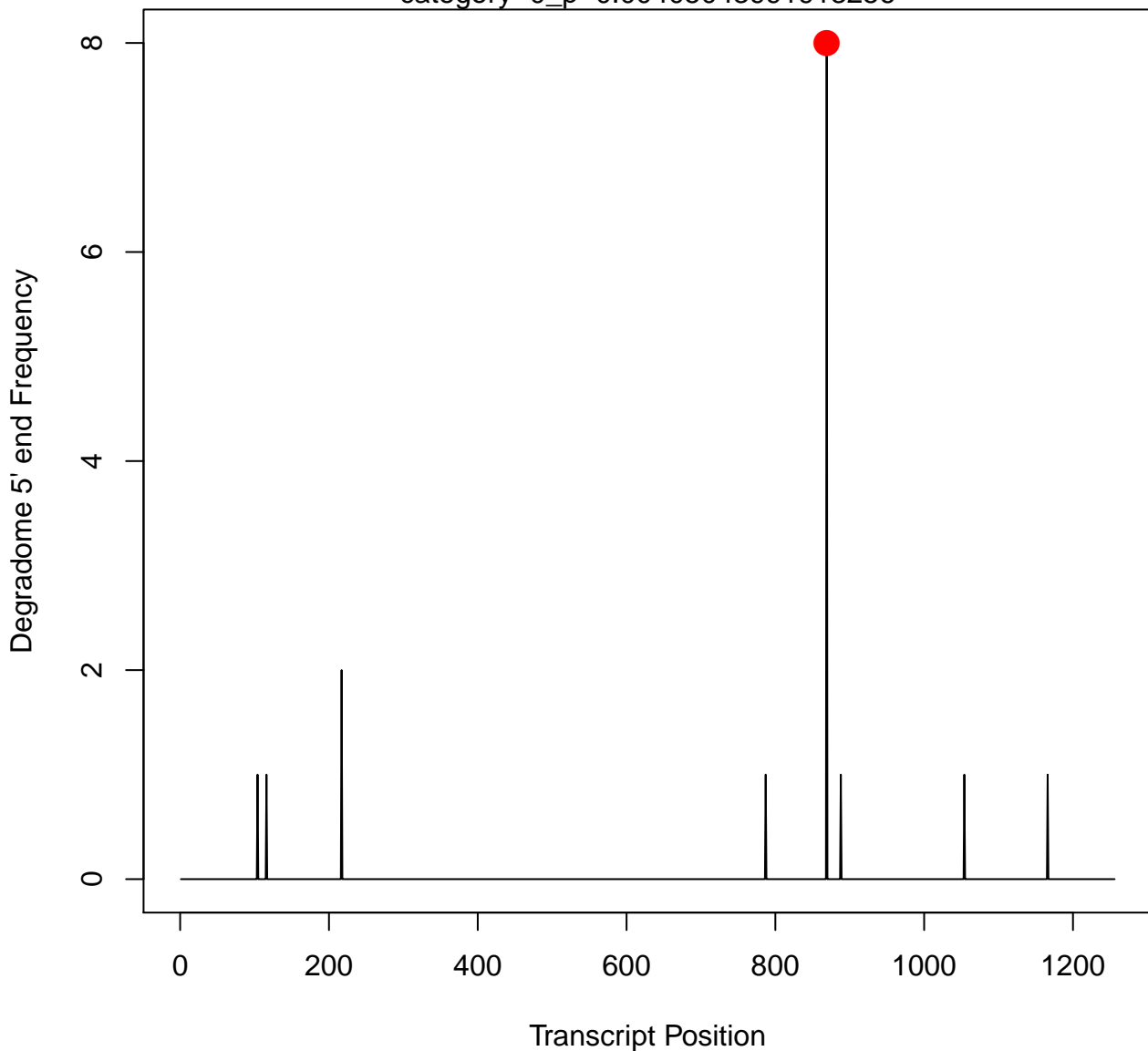

Supplement: Supplementary file 2 [file Data_Sheet_8.ZIP › GSM2230747.plot/Lsa-miR157a_Lsat_1_v5_gn_4_113241.1_869_TPlot.pdf]

**T=Lsat\_1\_v5\_gn\_4\_160760.1\_Q=Lsa-miR157a\_S=3099**

category=2\_p=0.94796564871546

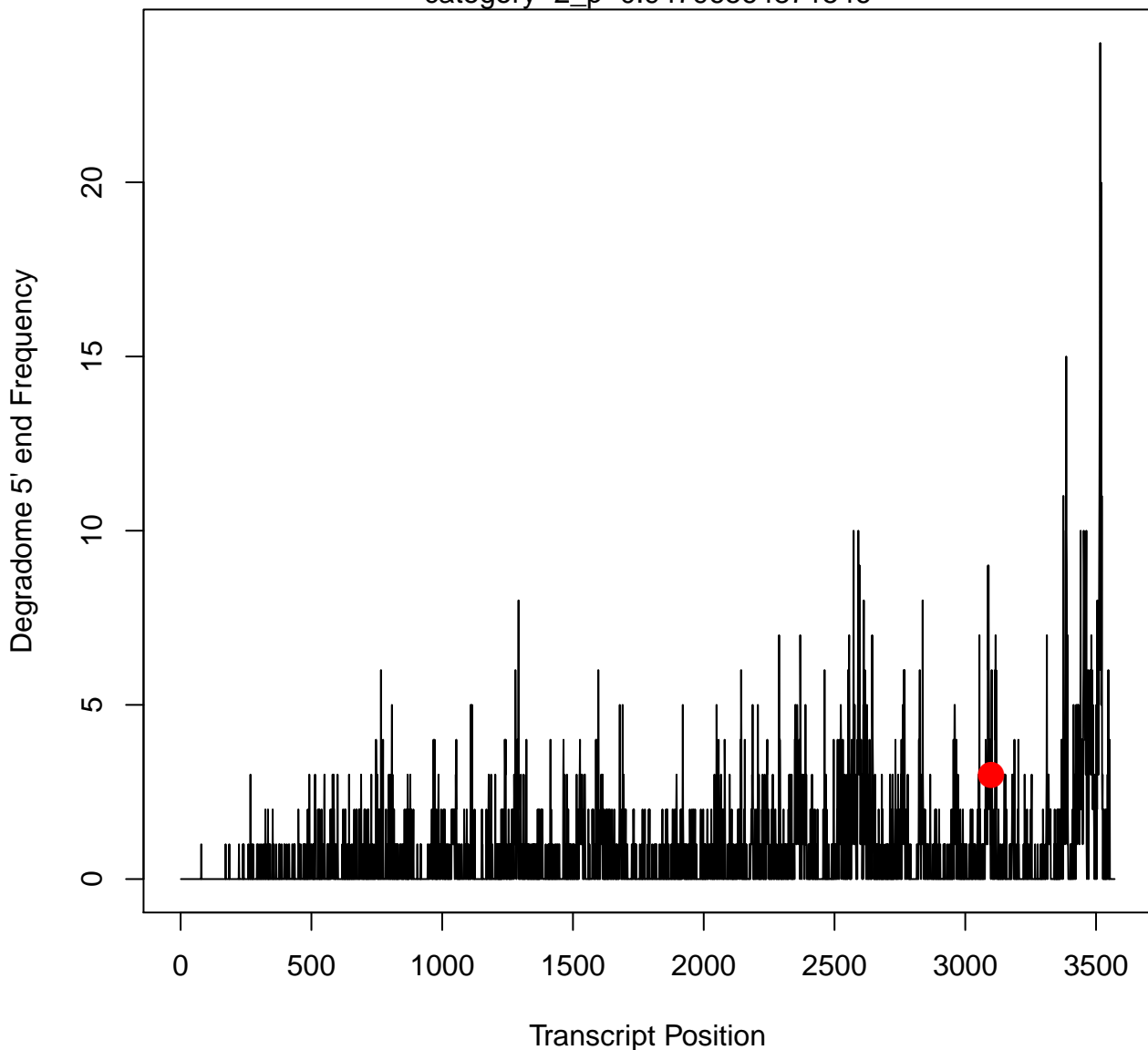

Supplement: Supplementary file 2 [file Data_Sheet_8.ZIP › GSM2230747.plot/Lsa-miR157a_Lsat_1_v5_gn_4_160760.1_3099_TPlot.pdf]

**T=Lsat\_1\_v5\_gn\_4\_421.1\_Q=Lsa-miR157a\_S=2360**

category=0\_p=0.000368901499921082

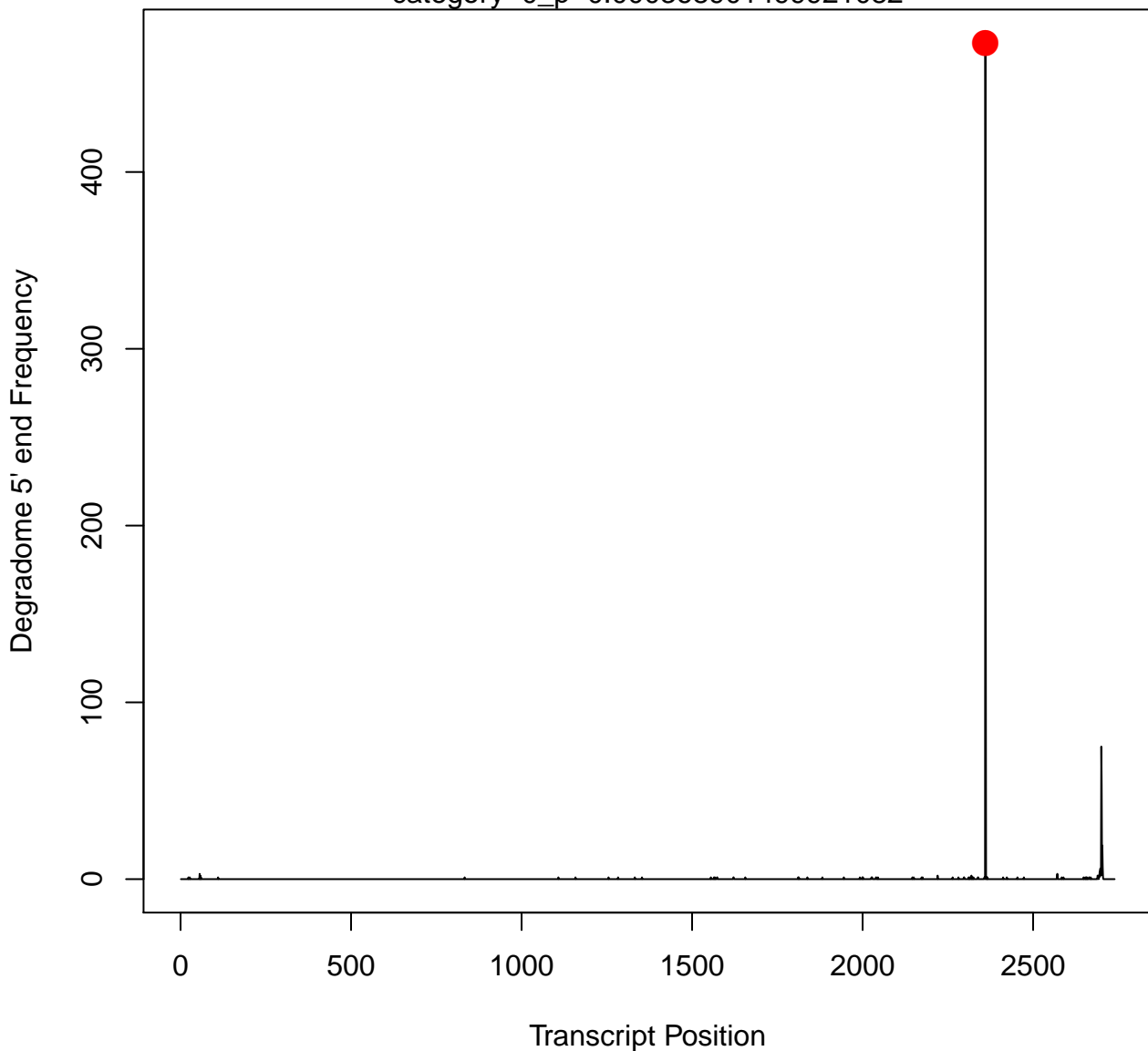

Supplement: Supplementary file 2 [file Data_Sheet_8.ZIP › GSM2230747.plot/Lsa-miR157a_Lsat_1_v5_gn_4_421.1_2360_TPlot.pdf]

**T=Lsat\_1\_v5\_gn\_5\_142161.1\_Q=Lsa-miR157a\_S=3504**

category=2\_p=0.873298010485164

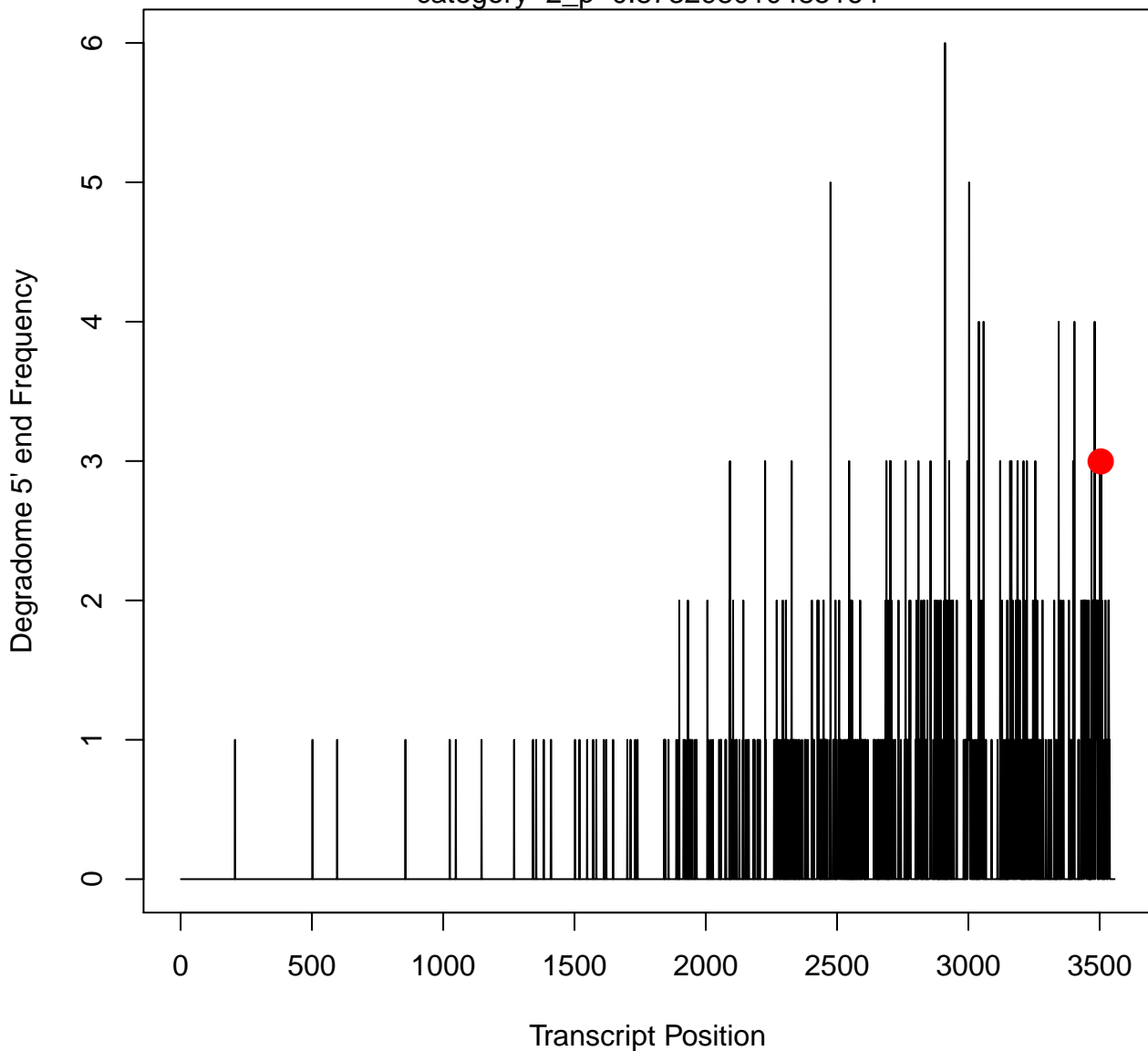

Supplement: Supplementary file 2 [file Data_Sheet_8.ZIP › GSM2230747.plot/Lsa-miR157a_Lsat_1_v5_gn_5_142161.1_3504_TPlot.pdf]

**T=Lsat\_1\_v5\_gn\_5\_71580.1\_Q=Lsa-miR157a\_S=519**

category=2\_p=0.913474632407168

Degradome 5' end Frequency

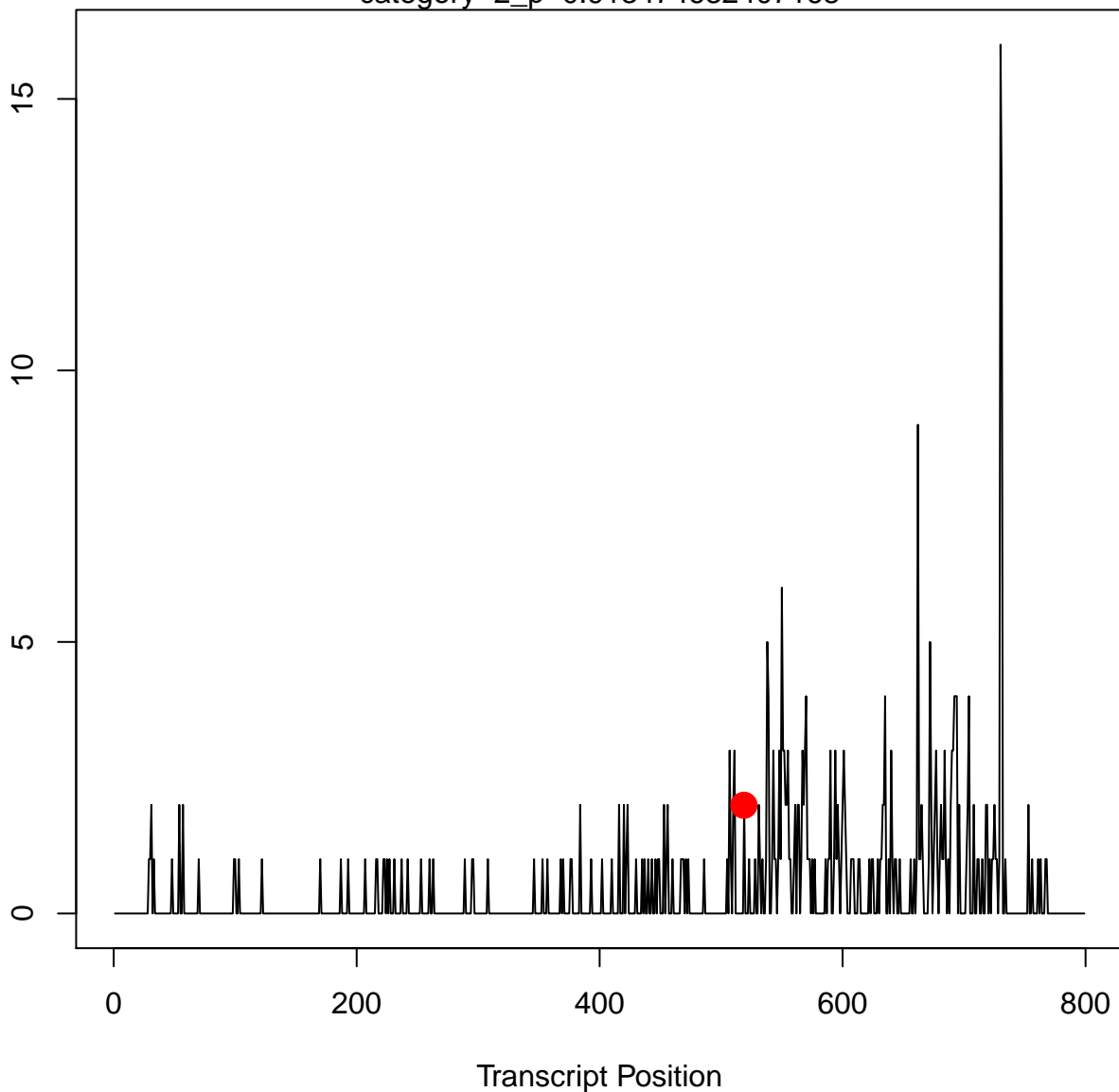

Supplement: Supplementary file 2 [file Data_Sheet_8.ZIP › GSM2230747.plot/Lsa-miR157a_Lsat_1_v5_gn_5_71580.1_519_TPlot.pdf]

**T=Lsat\_1\_v5\_gn\_7\_11040.1\_Q=Lsa-miR157a\_S=1087**

category=0\_p=0.00147478967057835

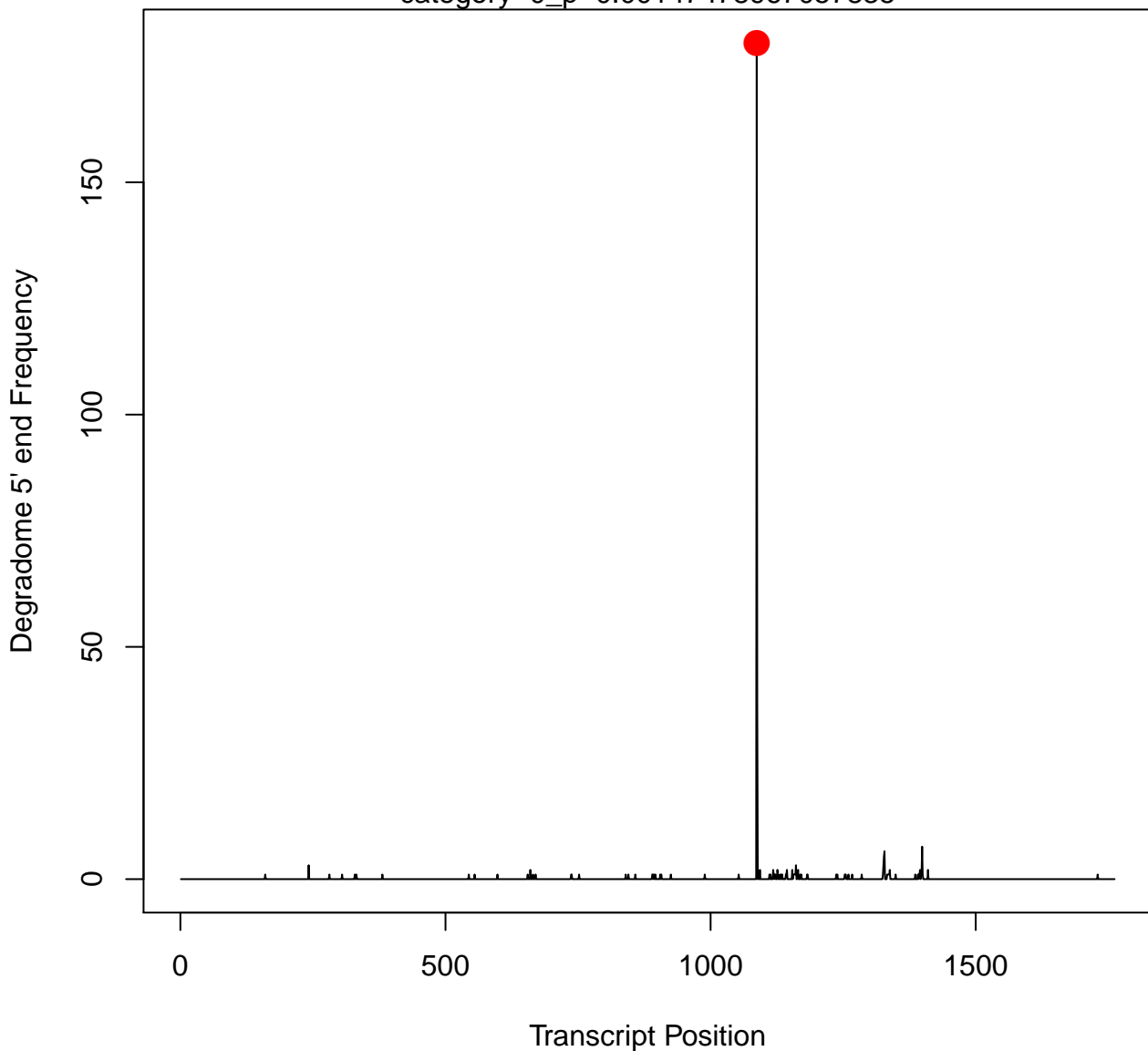

Supplement: Supplementary file 2 [file Data_Sheet_8.ZIP › GSM2230747.plot/Lsa-miR157a_Lsat_1_v5_gn_7_11040.1_1087_TPlot.pdf]

**T=Lsat\_1\_v5\_gn\_9\_40860.1\_Q=Lsa-miR157a\_S=212**

category=2\_p=0.951170364658315

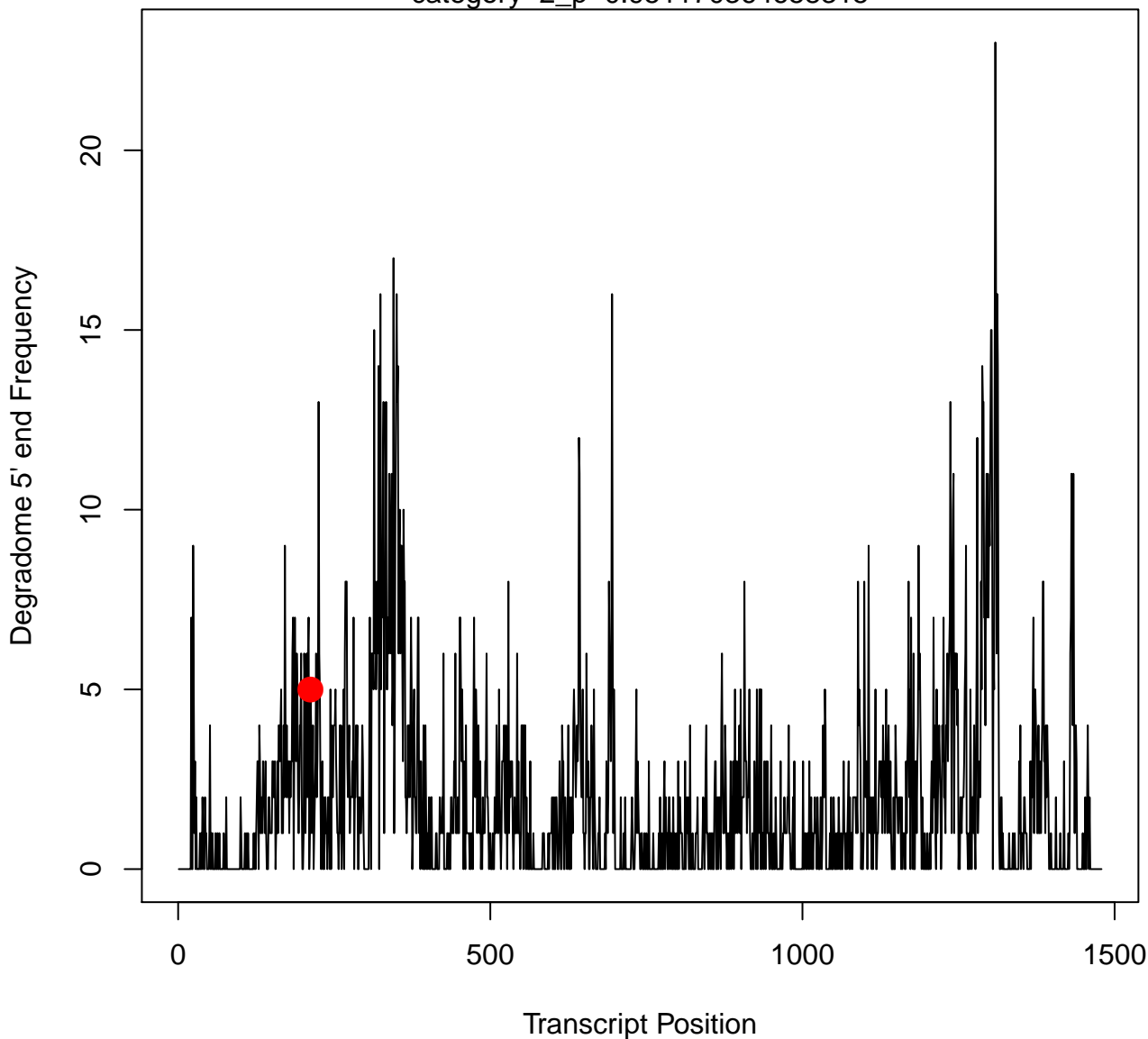

Supplement: Supplementary file 2 [file Data_Sheet_8.ZIP › GSM2230747.plot/Lsa-miR157a_Lsat_1_v5_gn_9_40860.1_212_TPlot.pdf]

**T=Lsat\_1\_v5\_gn\_9\_4540.1\_Q=Lsa-miR157a\_S=325**

category=2\_p=0.795904508566031

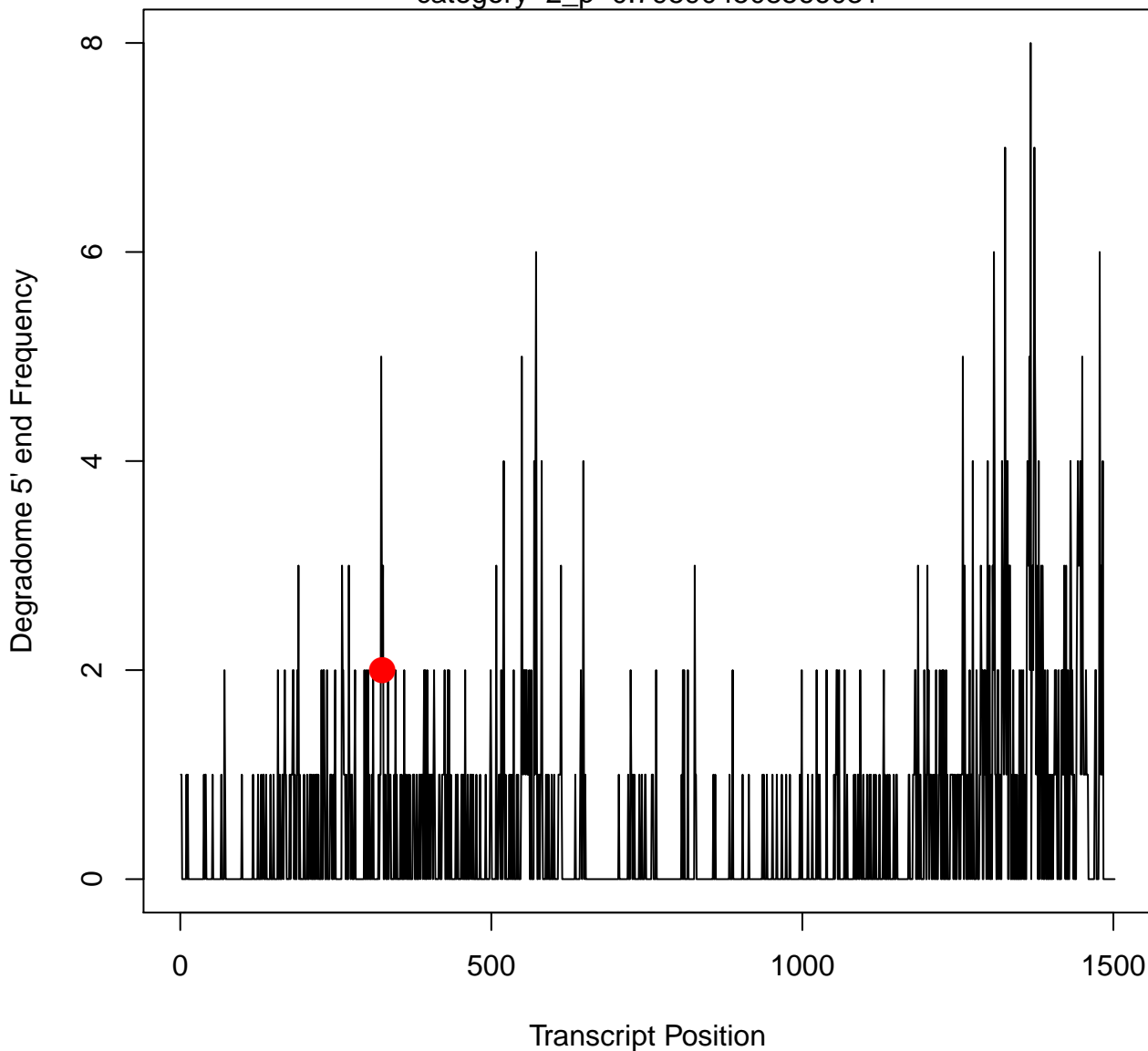

Supplement: Supplementary file 2 [file Data_Sheet_8.ZIP › GSM2230747.plot/Lsa-miR157a_Lsat_1_v5_gn_9_4540.1_325_TPlot.pdf]

**T=Lsat\_1\_v5\_gn\_2\_134361.1\_Q=Lsa-miR157b\_S=223**

category=2\_p=0.638345908242153

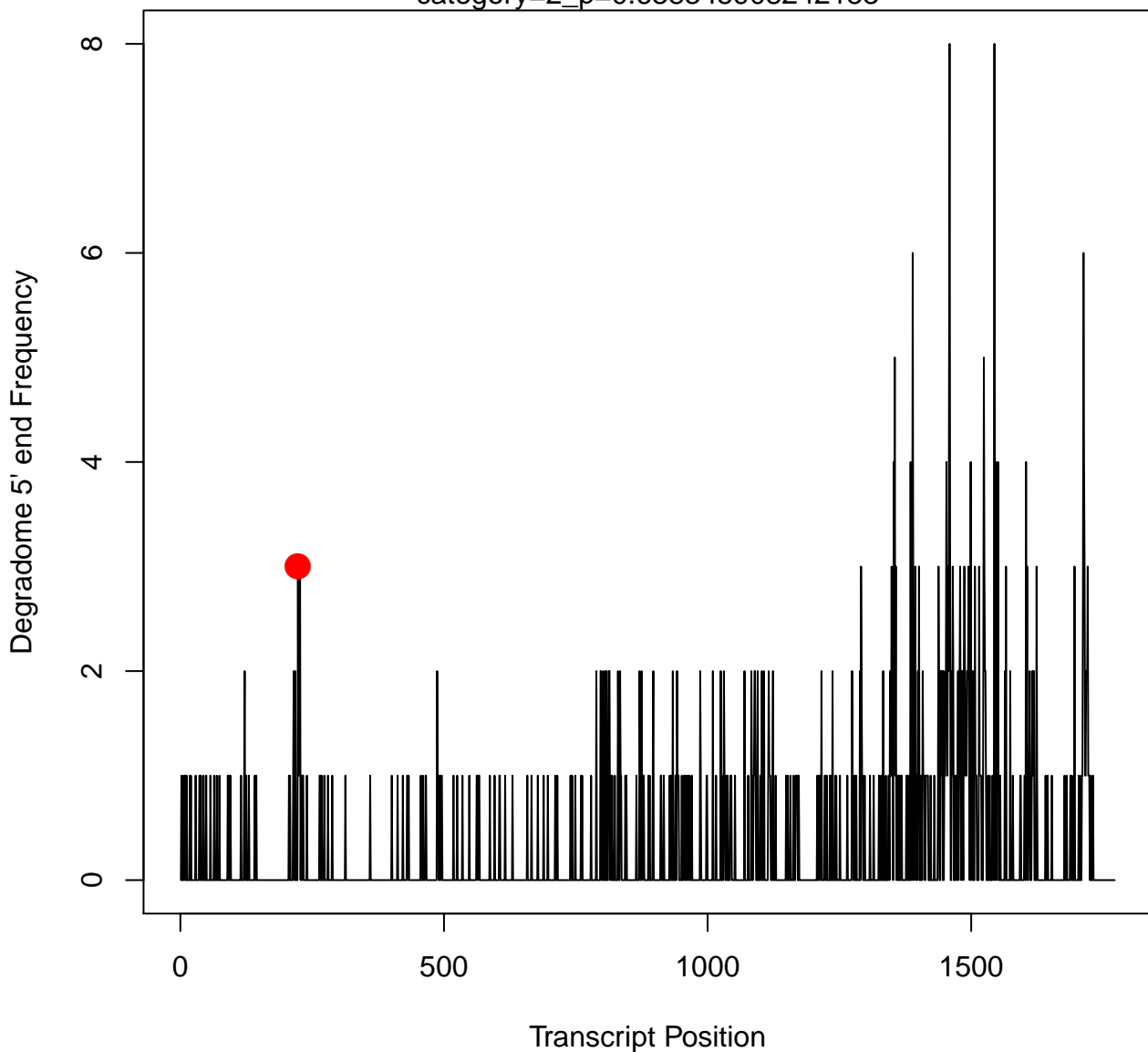

Supplement: Supplementary file 2 [file Data_Sheet_8.ZIP › GSM2230747.plot/Lsa-miR157b_Lsat_1_v5_gn_2_134361.1_223_TPlot.pdf]

T=Lsat\_1\_v5\_gn\_4\_421.1\_Q=Lsa-miR157b\_S=2361

category=2\_p=0.0312835643520768

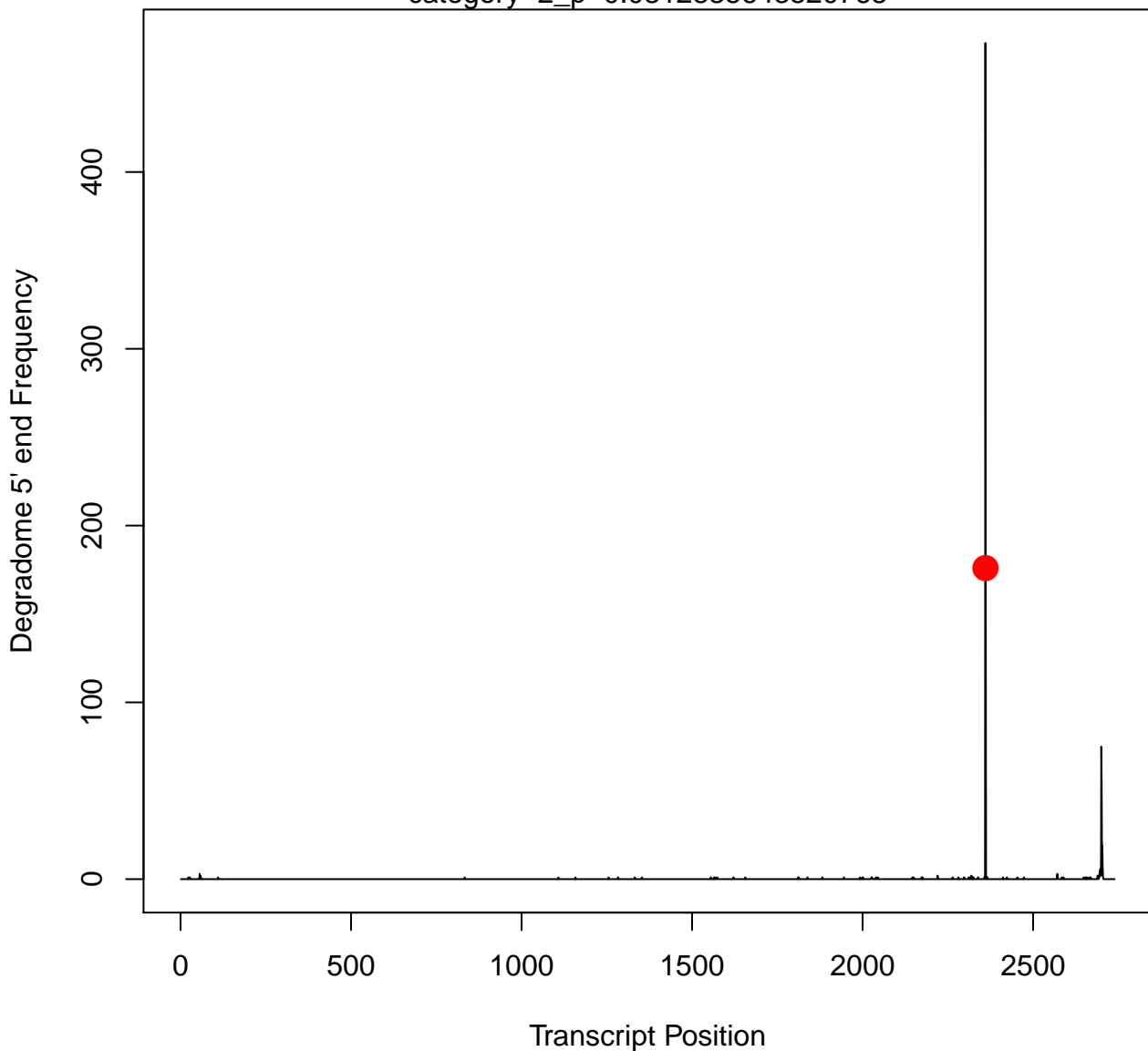

Supplement: Supplementary file 2 [file Data_Sheet_8.ZIP › GSM2230747.plot/Lsa-miR157b_Lsat_1_v5_gn_4_421.1_2361_TPlot.pdf]

**T=Lsat\_1\_v5\_gn\_5\_12360.1\_Q=Lsa-miR157b\_S=1860**

category=2\_p=0.199471722134268

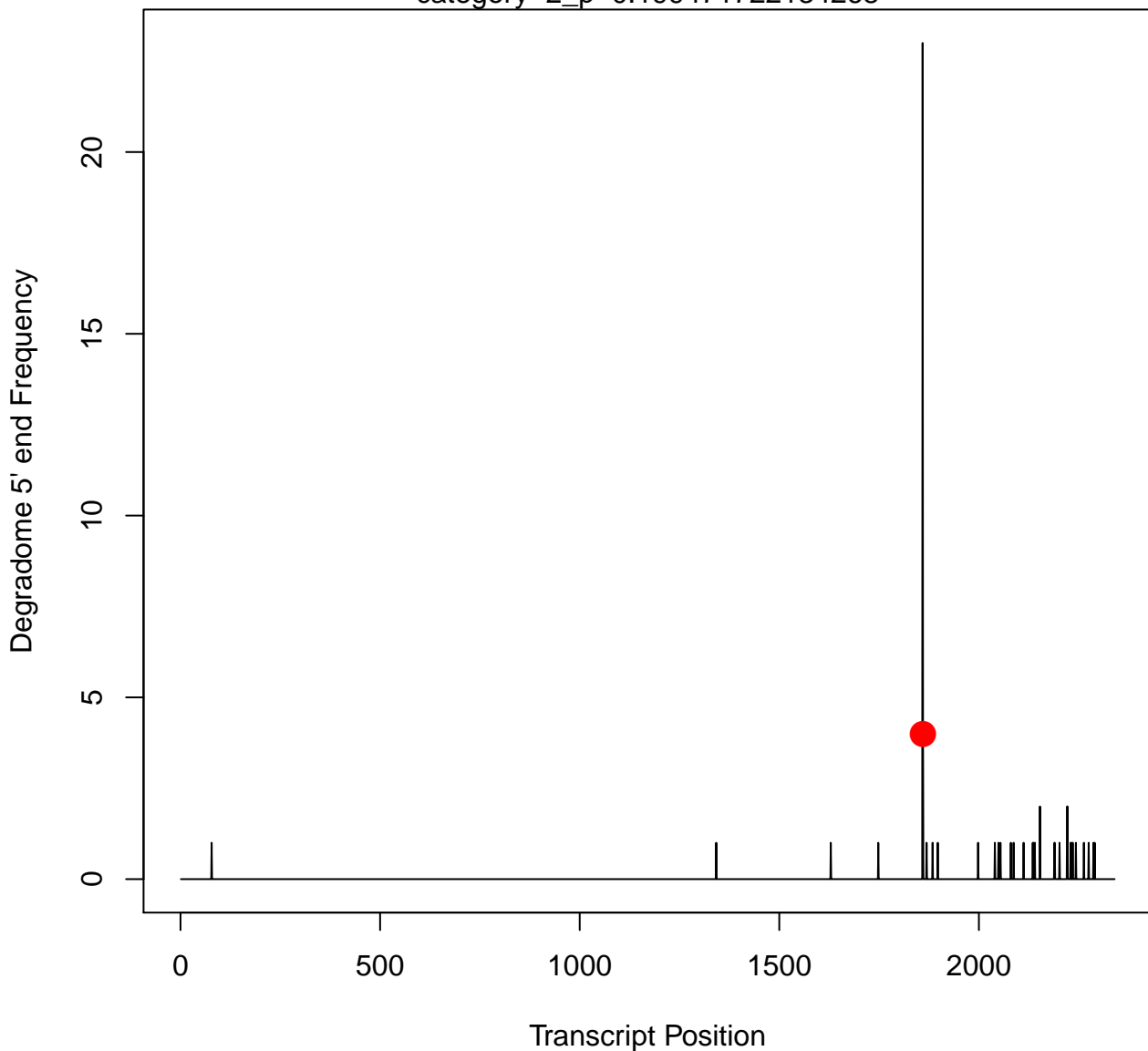

Supplement: Supplementary file 2 [file Data_Sheet_8.ZIP › GSM2230747.plot/Lsa-miR157b_Lsat_1_v5_gn_5_12360.1_1860_TPlot.pdf]

**T=Lsat\_1\_v5\_gn\_5\_142161.1\_Q=Lsa-miR157b\_S=3505**

category=2\_p=0.918803597180262

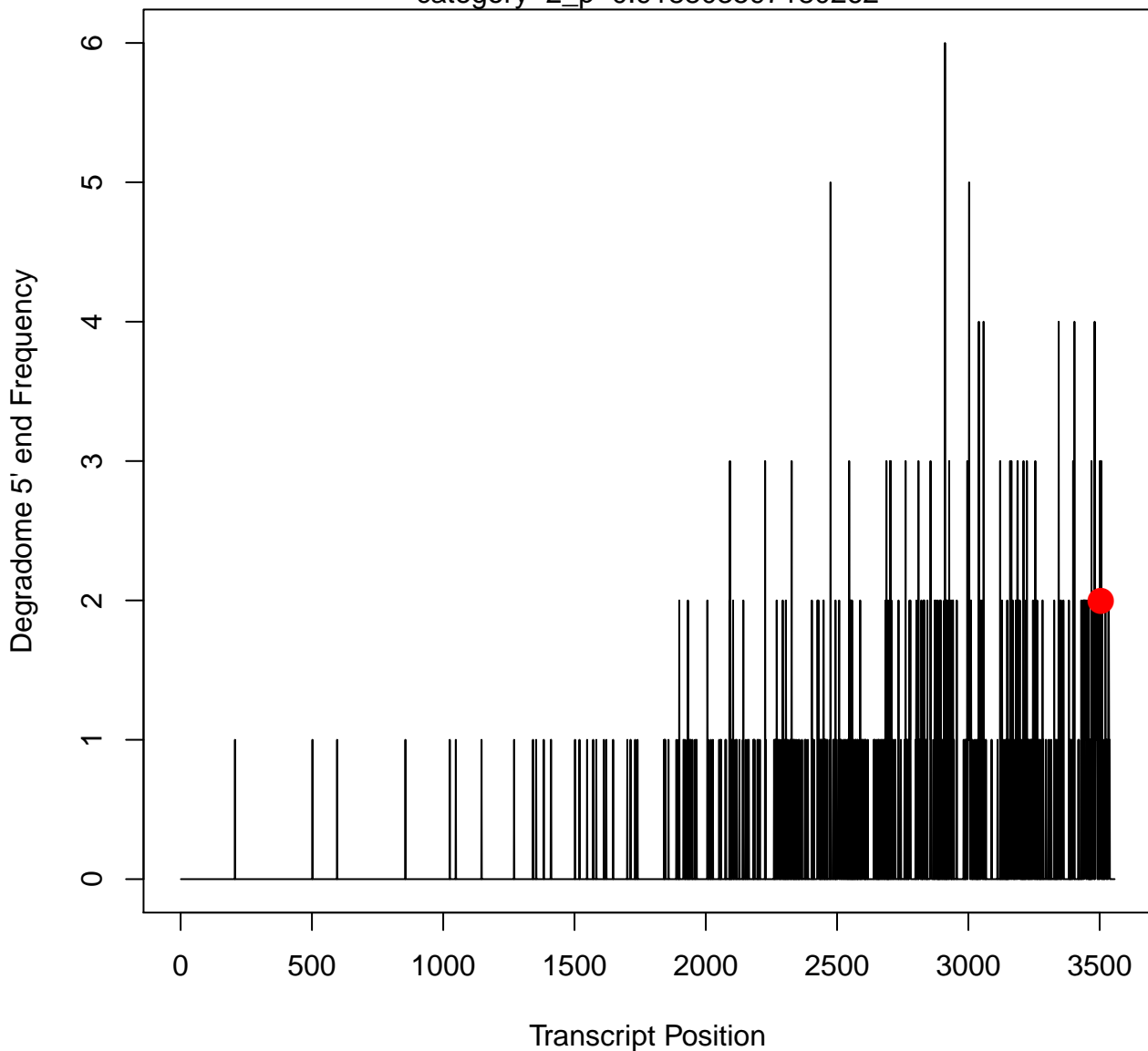

Supplement: Supplementary file 2 [file Data_Sheet_8.ZIP › GSM2230747.plot/Lsa-miR157b_Lsat_1_v5_gn_5_142161.1_3505_TPlot.pdf]

**T=Lsat\_1\_v5\_gn\_5\_94240.1\_Q=Lsa-miR157b\_S=673**

category=2\_p=0.317095430591475

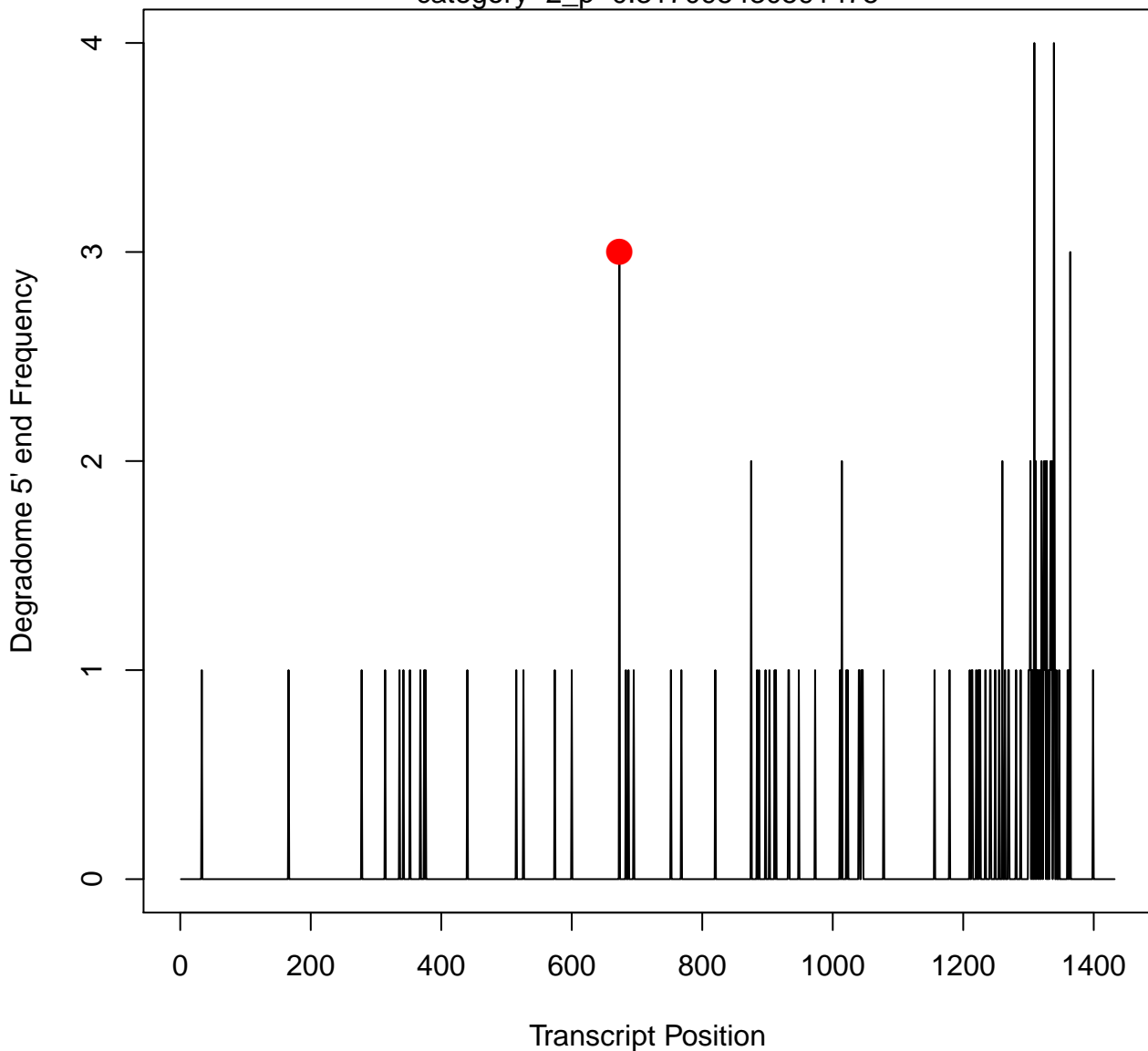

Supplement: Supplementary file 2 [file Data_Sheet_8.ZIP › GSM2230747.plot/Lsa-miR157b_Lsat_1_v5_gn_5_94240.1_673_TPlot.pdf]

**T=Lsat\_1\_v5\_gn\_7\_11040.1\_Q=Lsa-miR157b\_S=1088**

category=2\_p=0.173619597637671

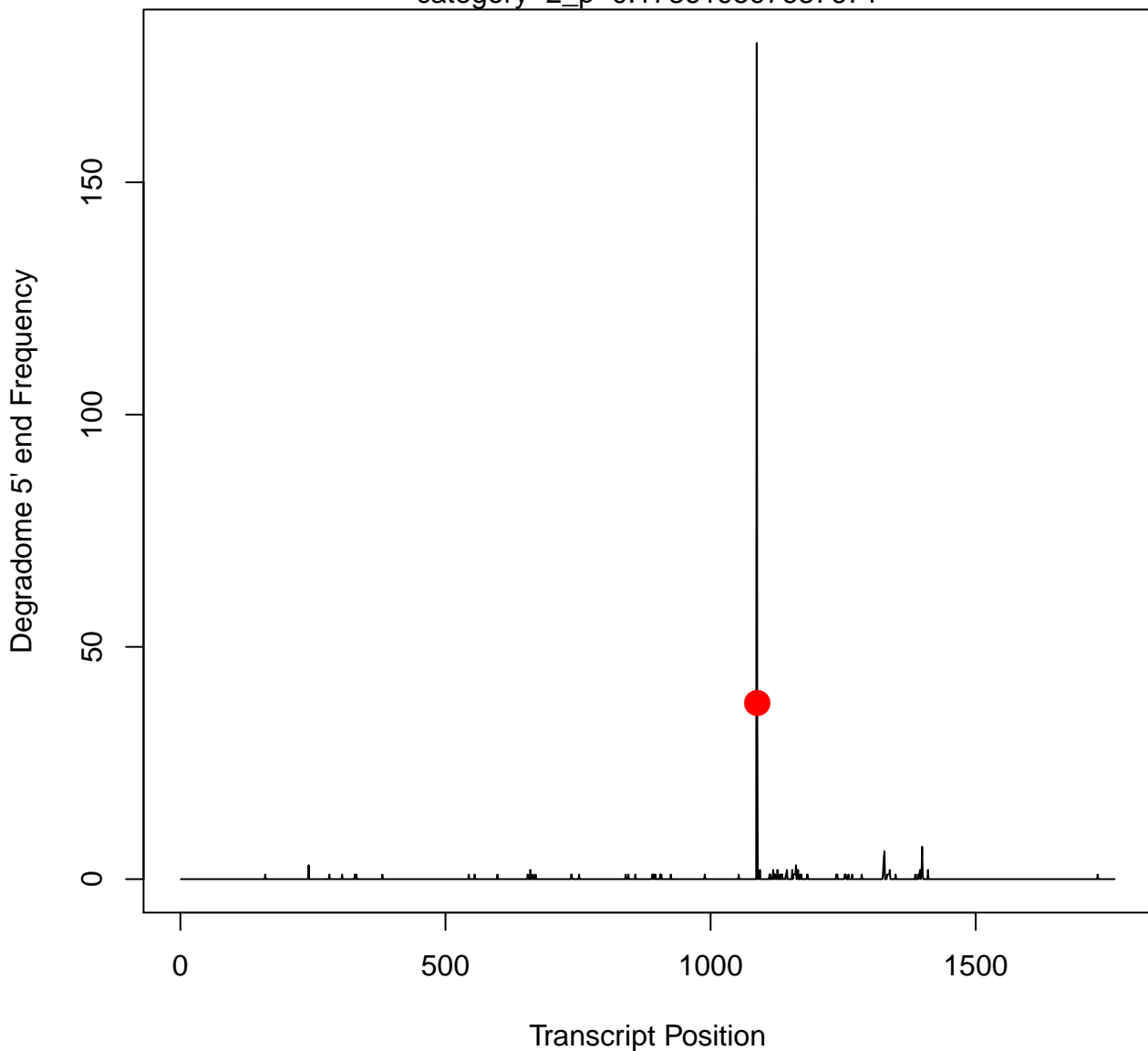

Supplement: Supplementary file 2 [file Data_Sheet_8.ZIP › GSM2230747.plot/Lsa-miR157b_Lsat_1_v5_gn_7_11040.1_1088_TPlot.pdf]

**T=Lsat\_1\_v5\_gn\_3\_31000.1\_Q=Lsa-miR157c\_S=850**

category=2\_p=0.0312835643520768

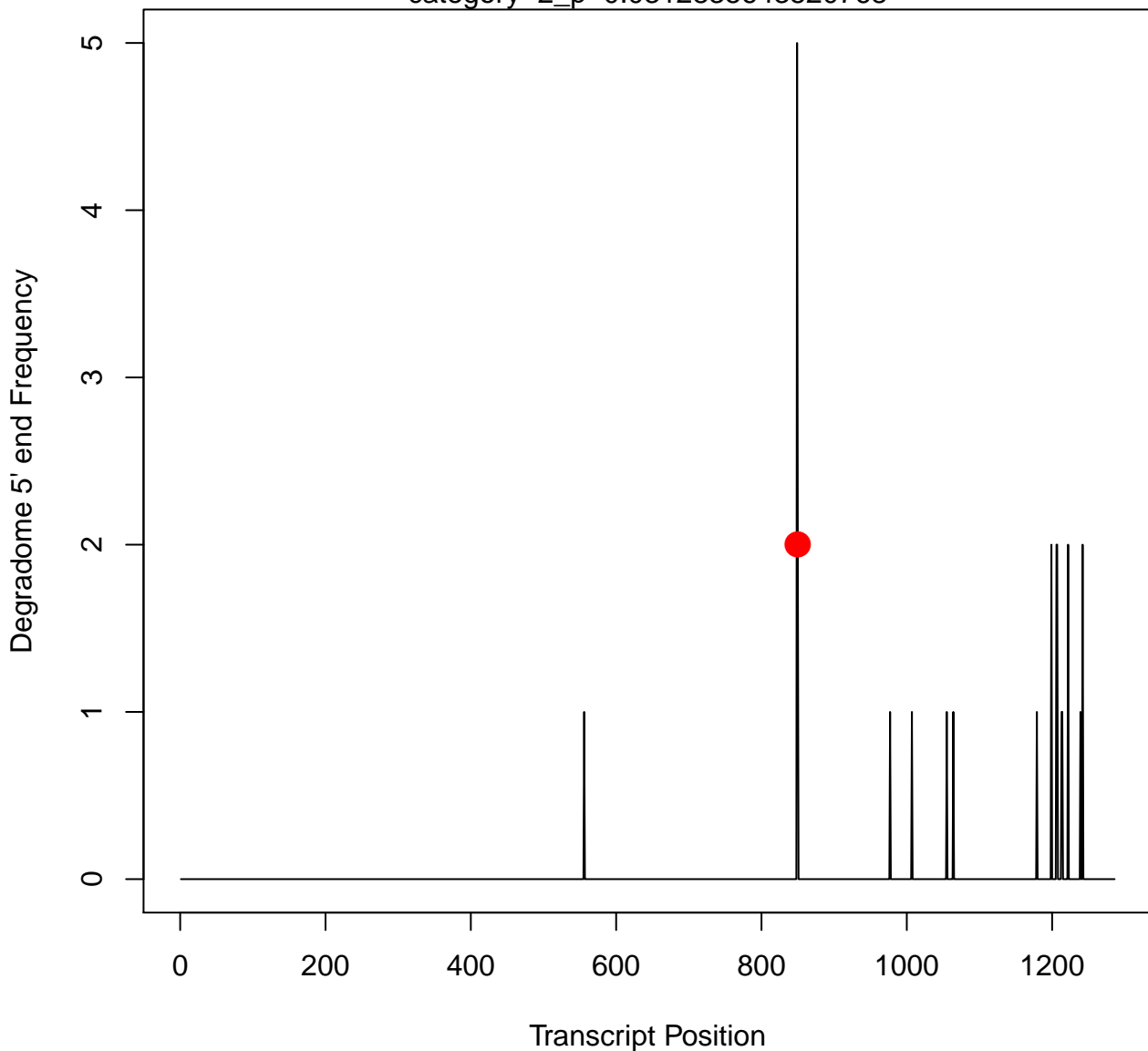

Supplement: Supplementary file 2 [file Data_Sheet_8.ZIP › GSM2230747.plot/Lsa-miR157c_Lsat_1_v5_gn_3_31000.1_850_TPlot.pdf]

**T=Lsat\_1\_v5\_gn\_9\_40860.1\_Q=Lsa-miR157c\_S=213**

category=2\_p=0.901744520335156

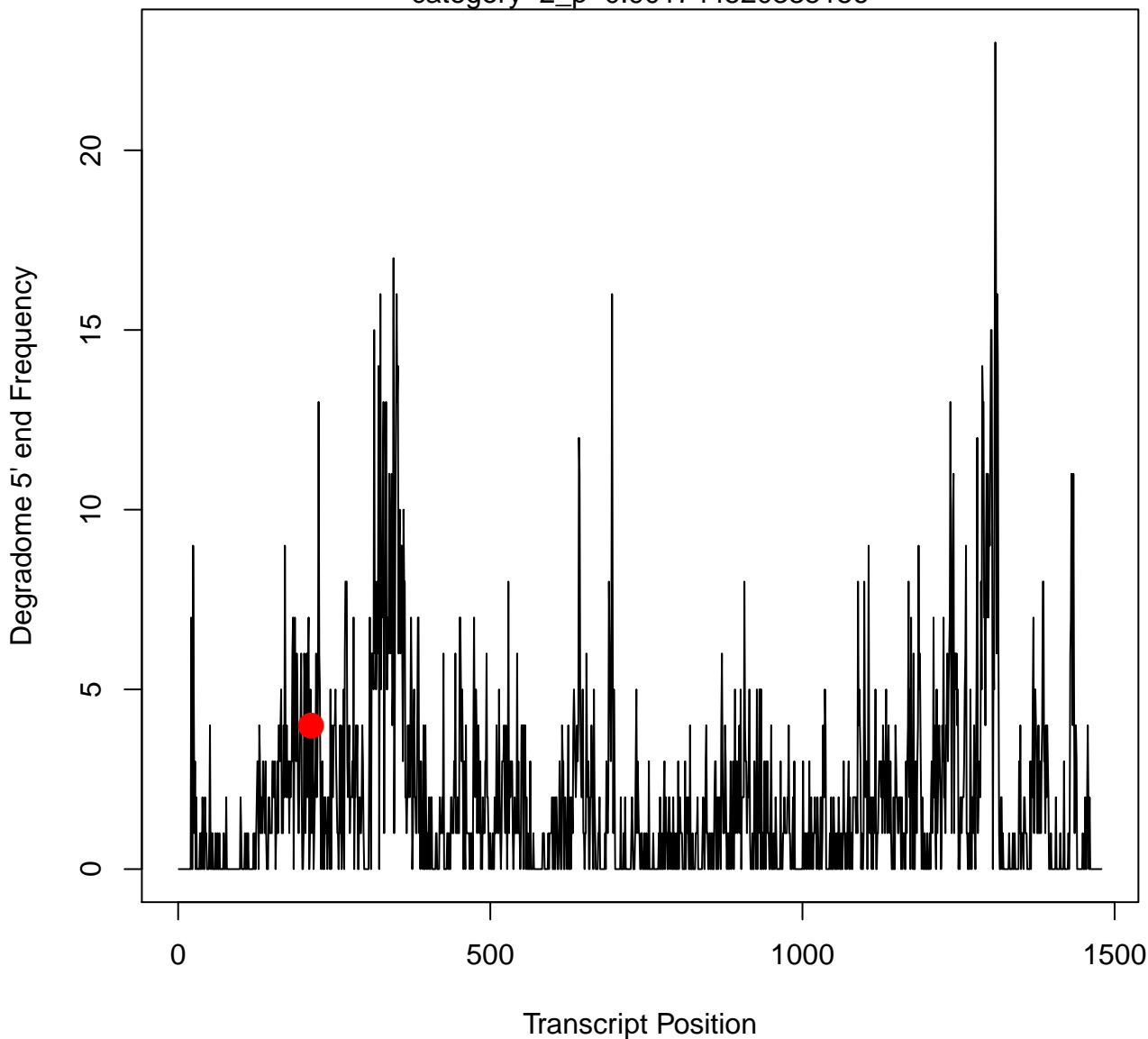

Supplement: Supplementary file 2 [file Data_Sheet_8.ZIP › GSM2230747.plot/Lsa-miR157c_Lsat_1_v5_gn_9_40860.1_213_TPlot.pdf]

**T=Lsat\_1\_v5\_gn\_4\_1141.1\_Q=Lsa-miR157e\_S=1410**

category=2\_p=0.146932609015138

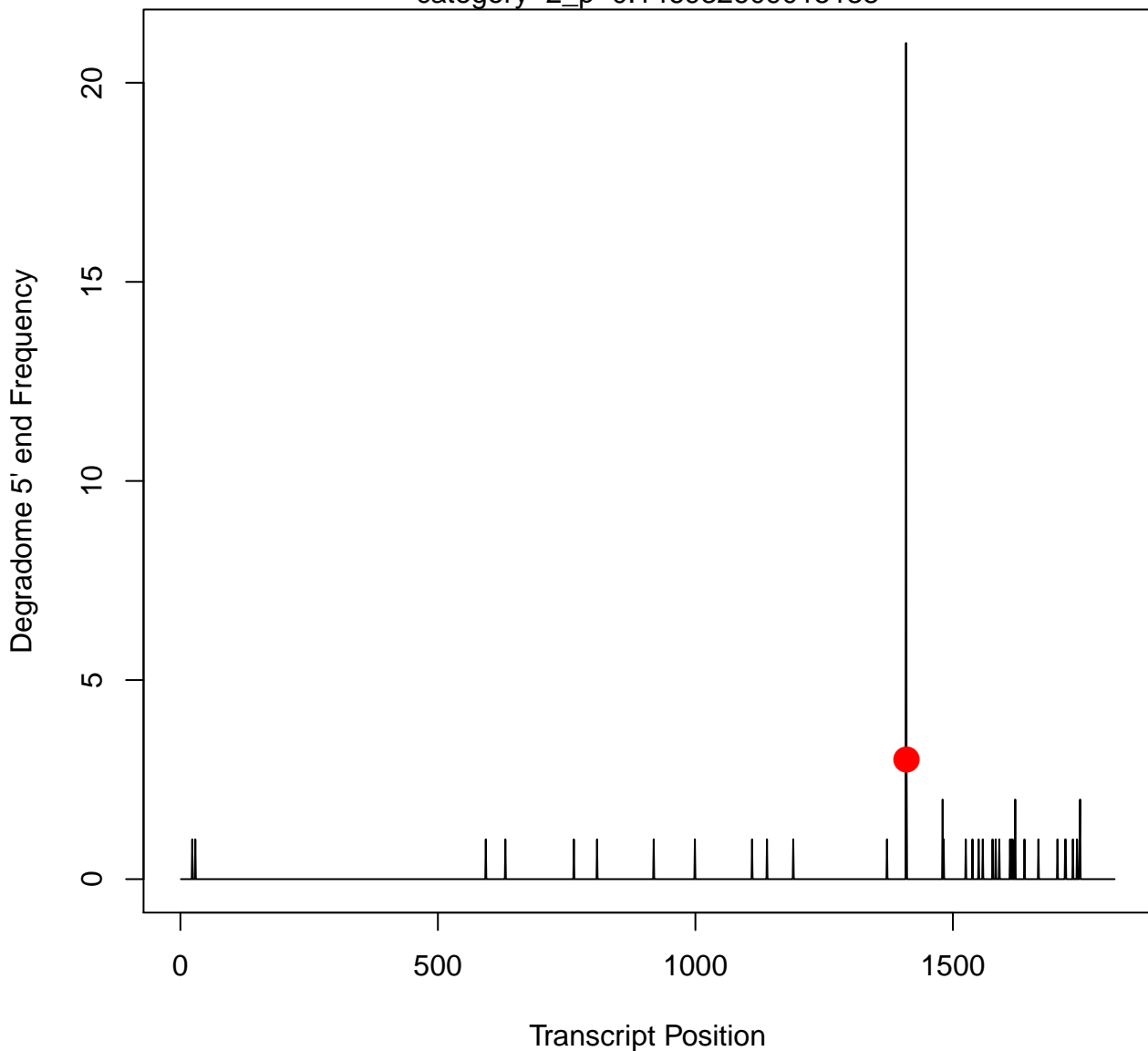

Supplement: Supplementary file 2 [file Data_Sheet_8.ZIP › GSM2230747.plot/Lsa-miR157e_Lsat_1_v5_gn_4_1141.1_1410_TPlot.pdf]

**T=Lsat\_1\_v5\_gn\_5\_104601.1\_Q=Lsa-miR157e\_S=960**

category=2\_p=0.753024768193277

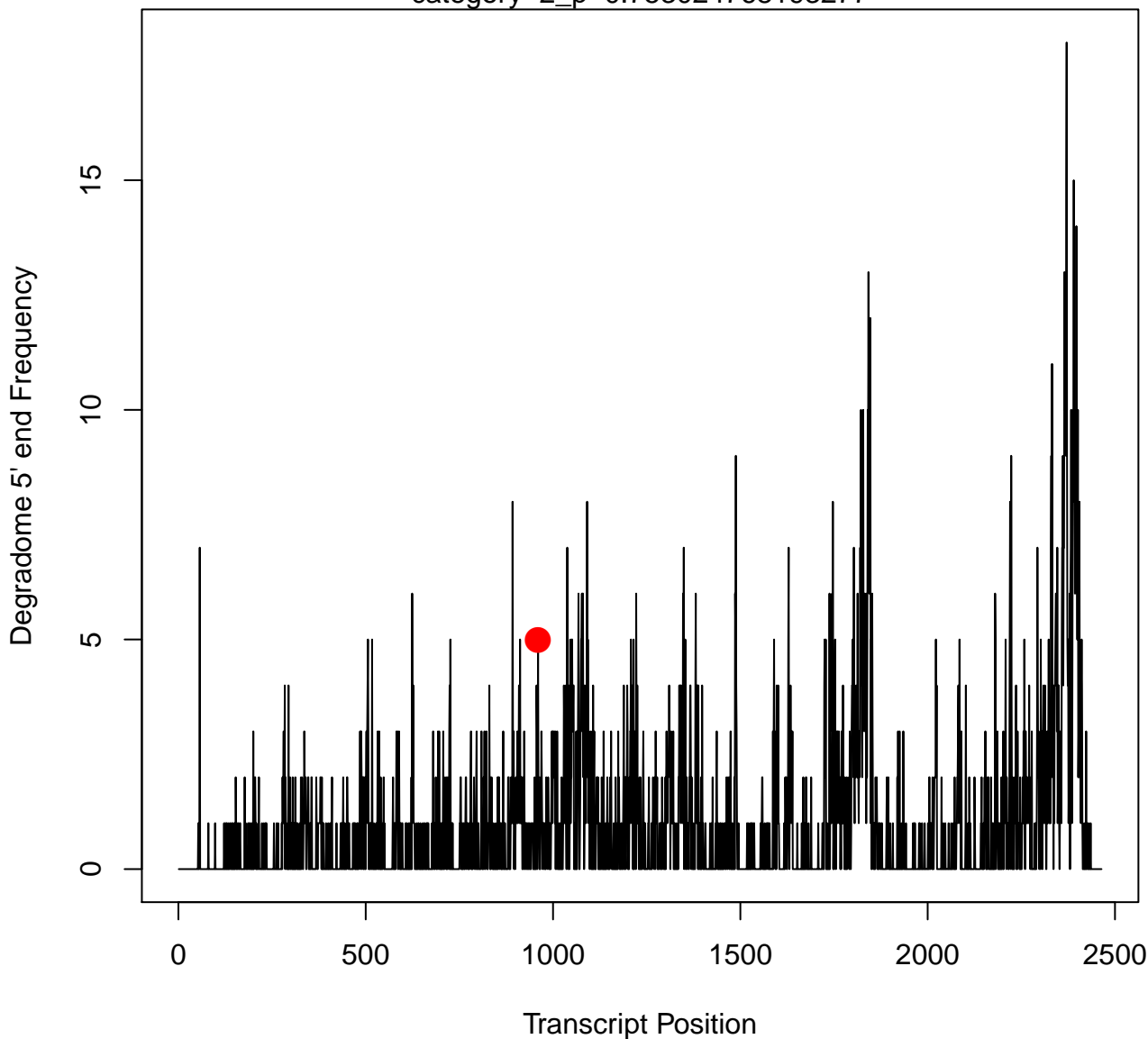

Supplement: Supplementary file 2 [file Data_Sheet_8.ZIP › GSM2230747.plot/Lsa-miR157e_Lsat_1_v5_gn_5_104601.1_960_TPlot.pdf]

**T=Lsat\_1\_v5\_gn\_8\_134640.1\_Q=Lsa-miR157e\_S=547**

category=0\_p=0.00294740433658425

Degradome 5' end Frequency

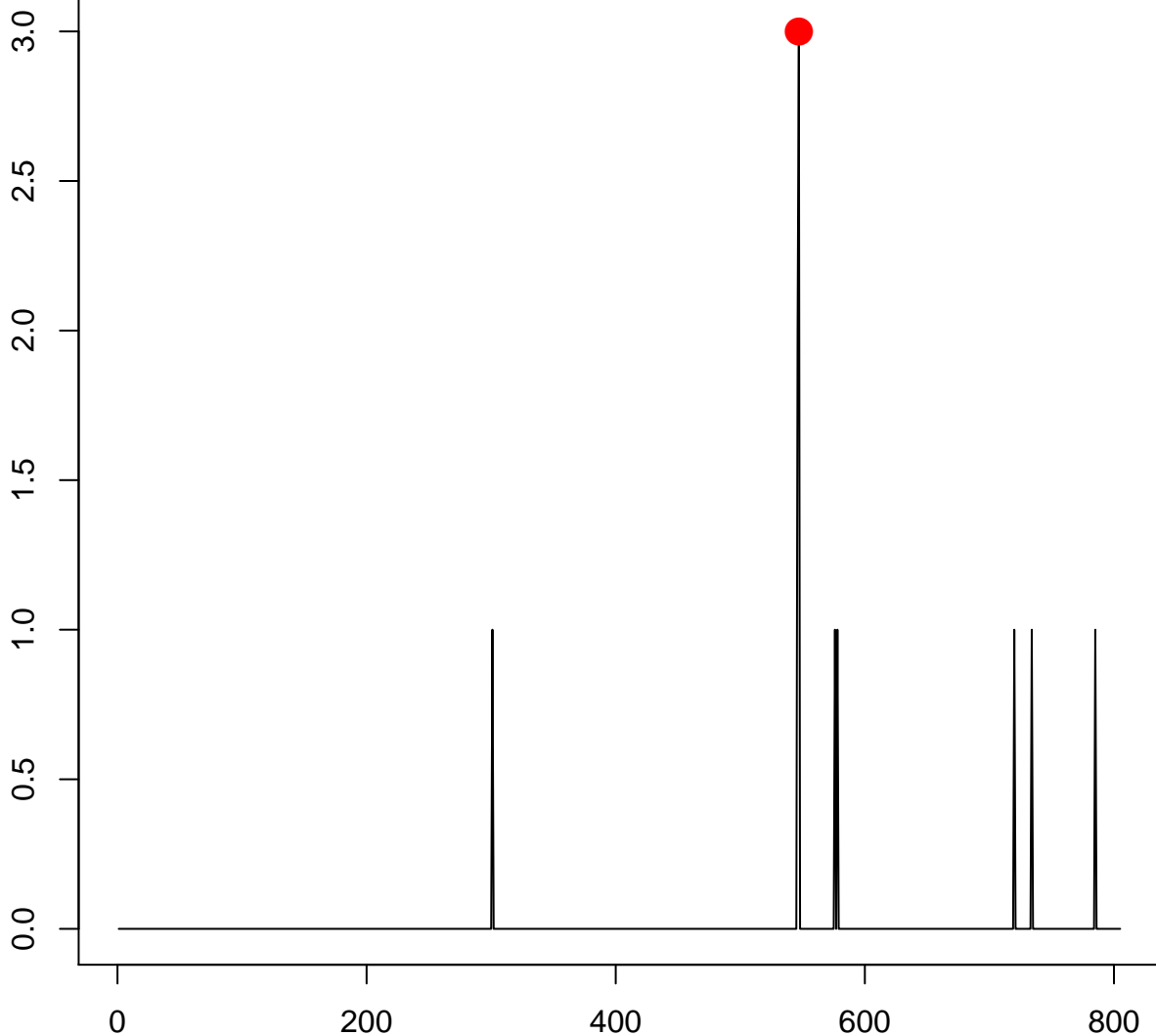

Transcript Position

Supplement: Supplementary file 2 [file Data_Sheet_8.ZIP › GSM2230747.plot/Lsa-miR157e_Lsat_1_v5_gn_8_134640.1_547_TPlot.pdf]

**T=Lsat\_1\_v5\_gn\_9\_28021.1\_Q=Lsa-miR157e\_S=1268**

category=0\_p=0.00147478967057835

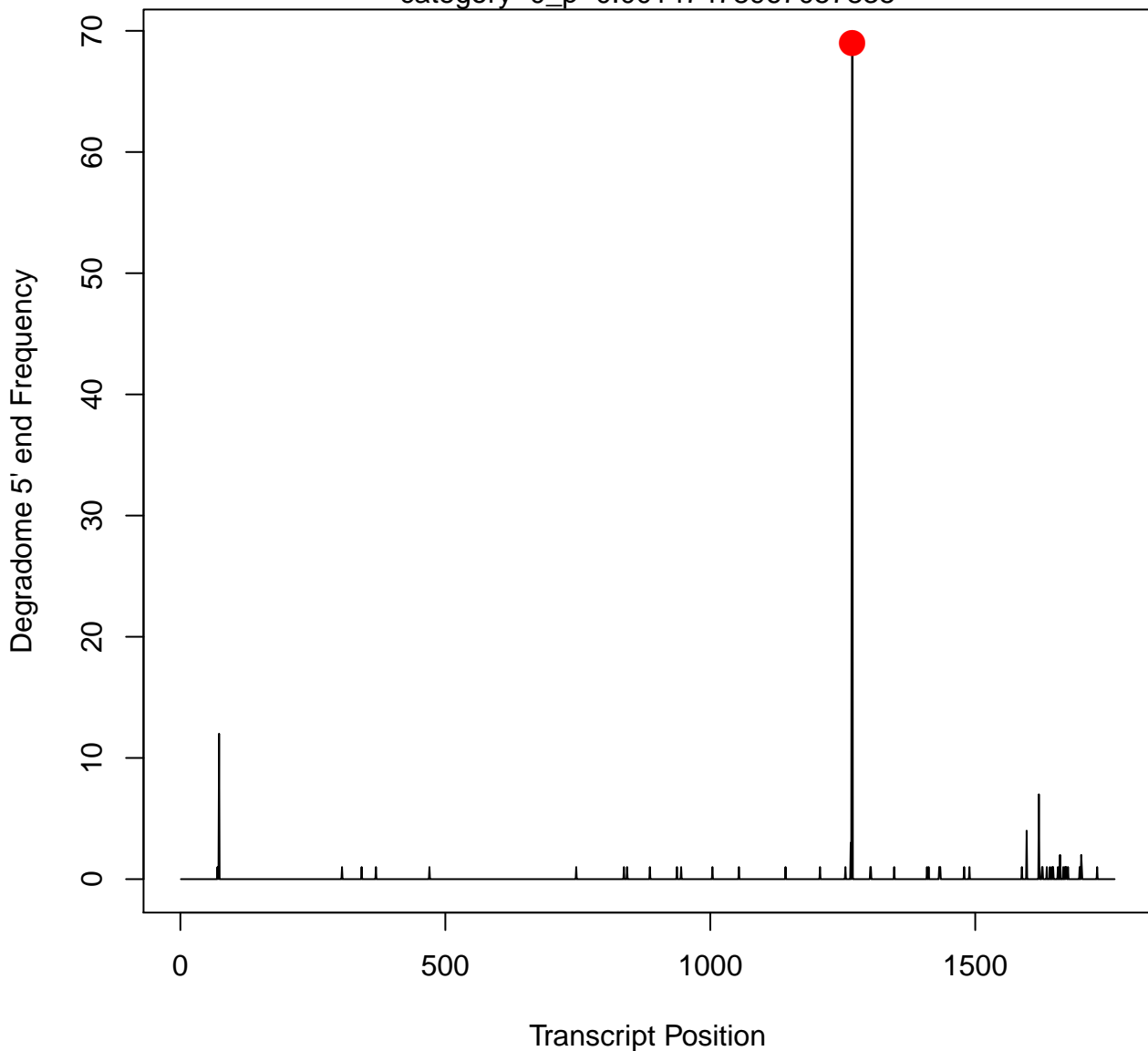

Supplement: Supplementary file 2 [file Data_Sheet_8.ZIP › GSM2230747.plot/Lsa-miR157e_Lsat_1_v5_gn_9_28021.1_1268_TPlot.pdf]

**T=Lsat\_1\_v5\_gn\_9\_4540.1\_Q=Lsa-miR157e\_S=326**

category=2\_p=0.825892791610651

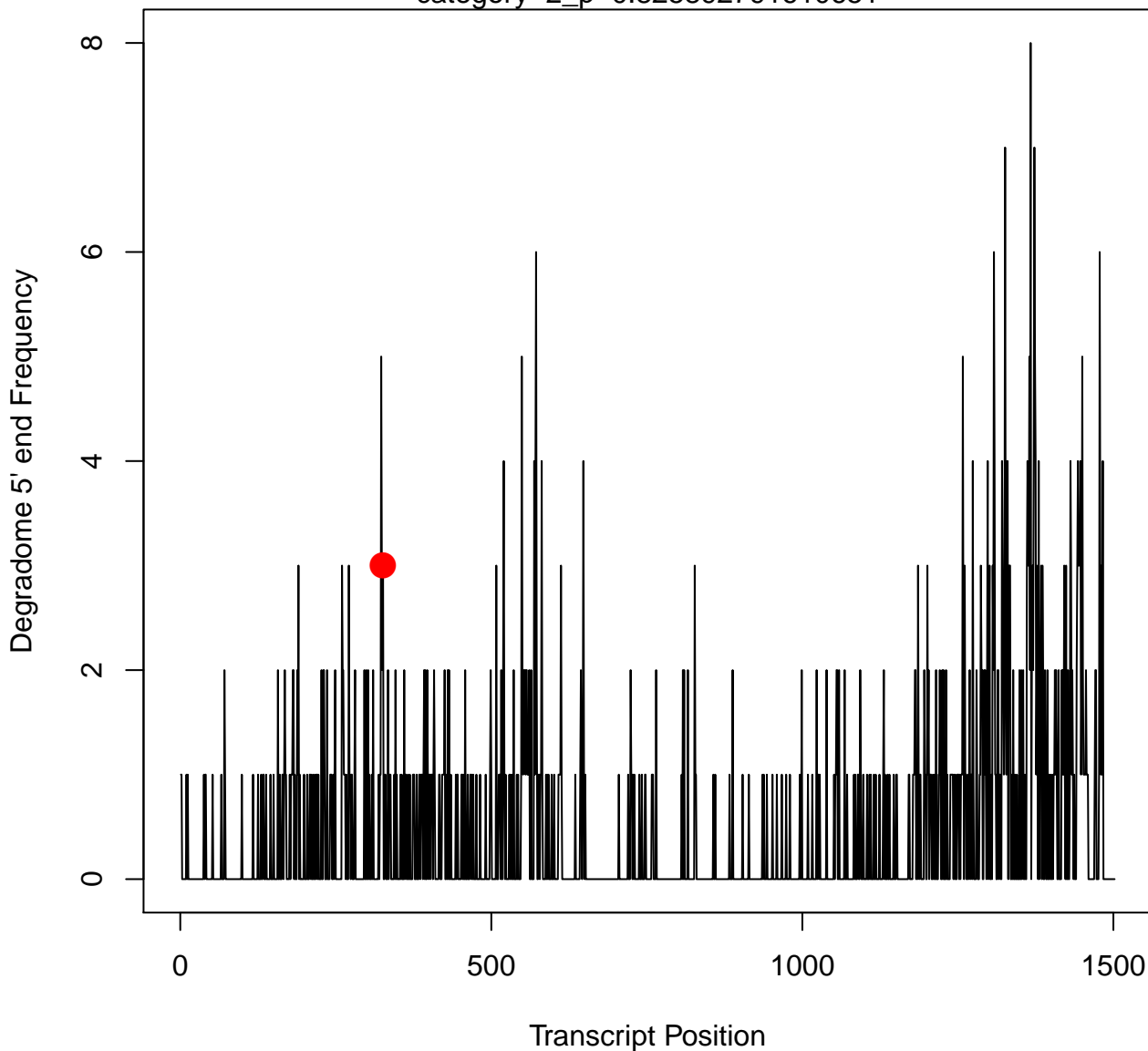

Supplement: Supplementary file 2 [file Data_Sheet_8.ZIP › GSM2230747.plot/Lsa-miR157e_Lsat_1_v5_gn_9_4540.1_326_TPlot.pdf]

**T=Lsat\_1\_v5\_gn\_2\_118860.1\_Q=Lsa-miR159a\_S=1149**

category=2\_p=0.998264863505651

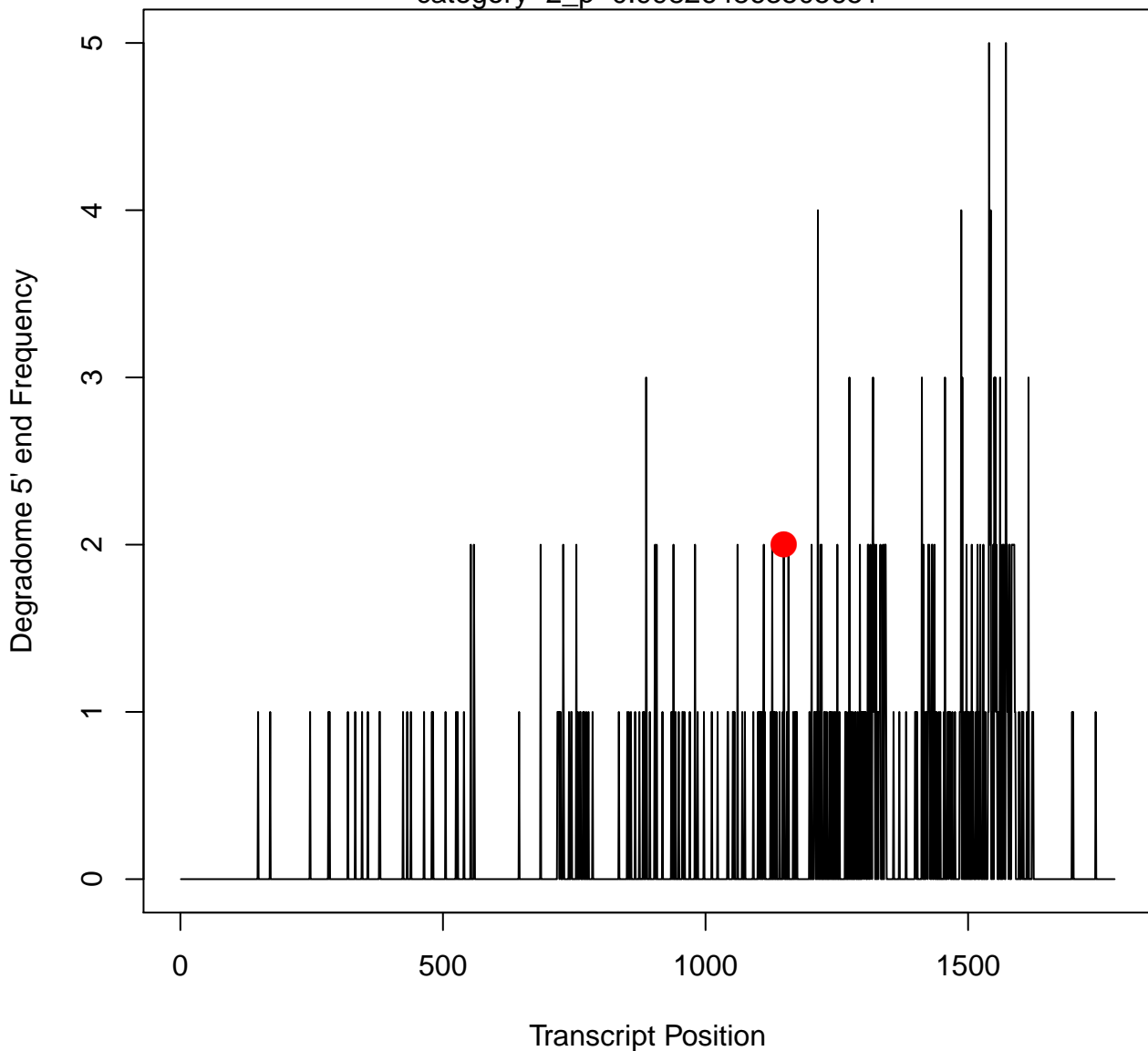

Supplement: Supplementary file 2 [file Data_Sheet_8.ZIP › GSM2230747.plot/Lsa-miR159a_Lsat_1_v5_gn_2_118860.1_1149_TPlot.pdf]

T=Lsat\_1\_v5\_gn\_2\_132241.1\_Q=Lsa-miR159a\_S=99

category=1\_p=0.0300479503252615

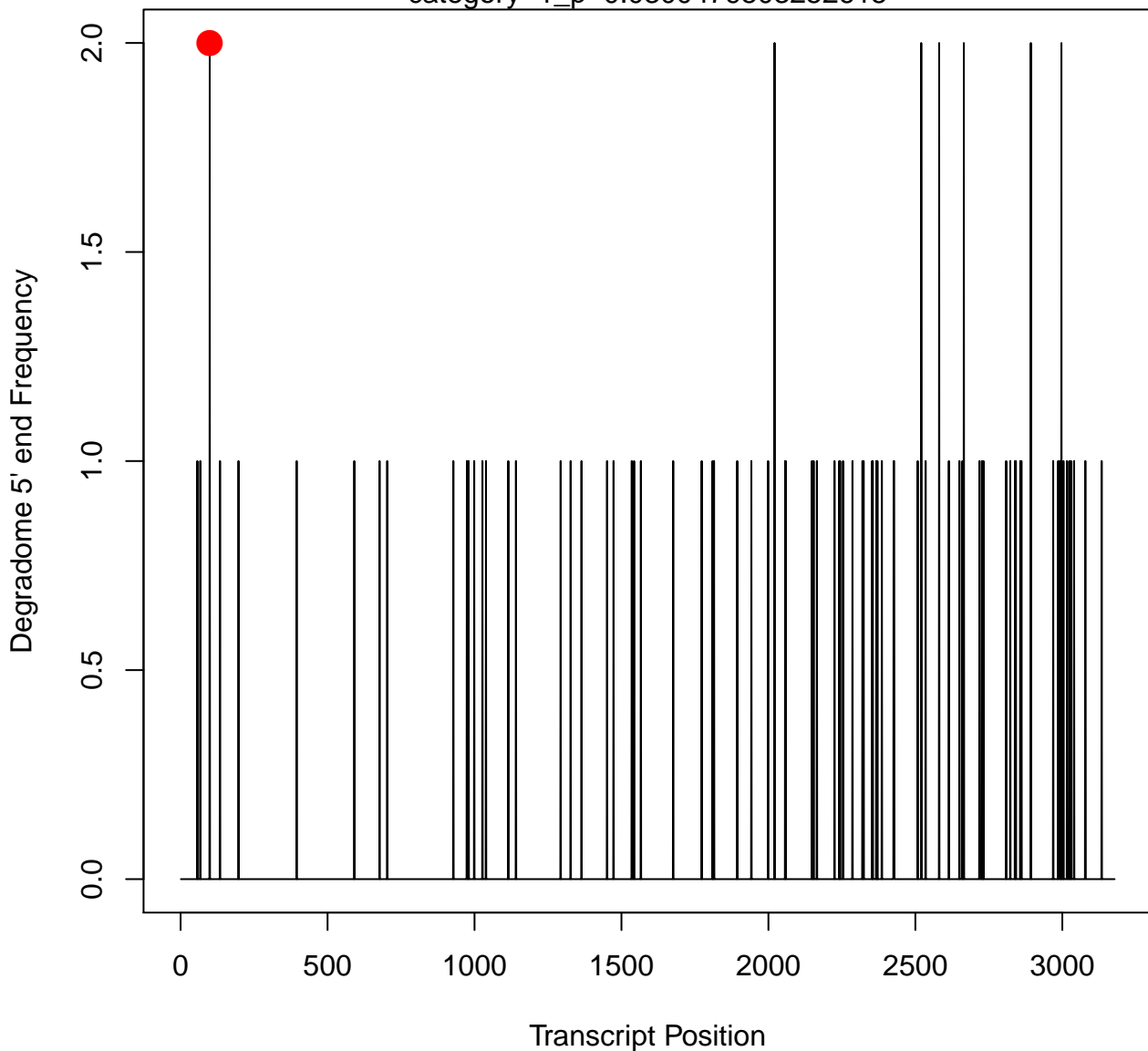

Supplement: Supplementary file 2 [file Data_Sheet_8.ZIP › GSM2230747.plot/Lsa-miR159a_Lsat_1_v5_gn_2_132241.1_99_TPlot.pdf]

**T=Lsat\_1\_v5\_gn\_2\_81541.1\_Q=Lsa-miR159a\_S=727**

category=2\_p=0.951170364658315

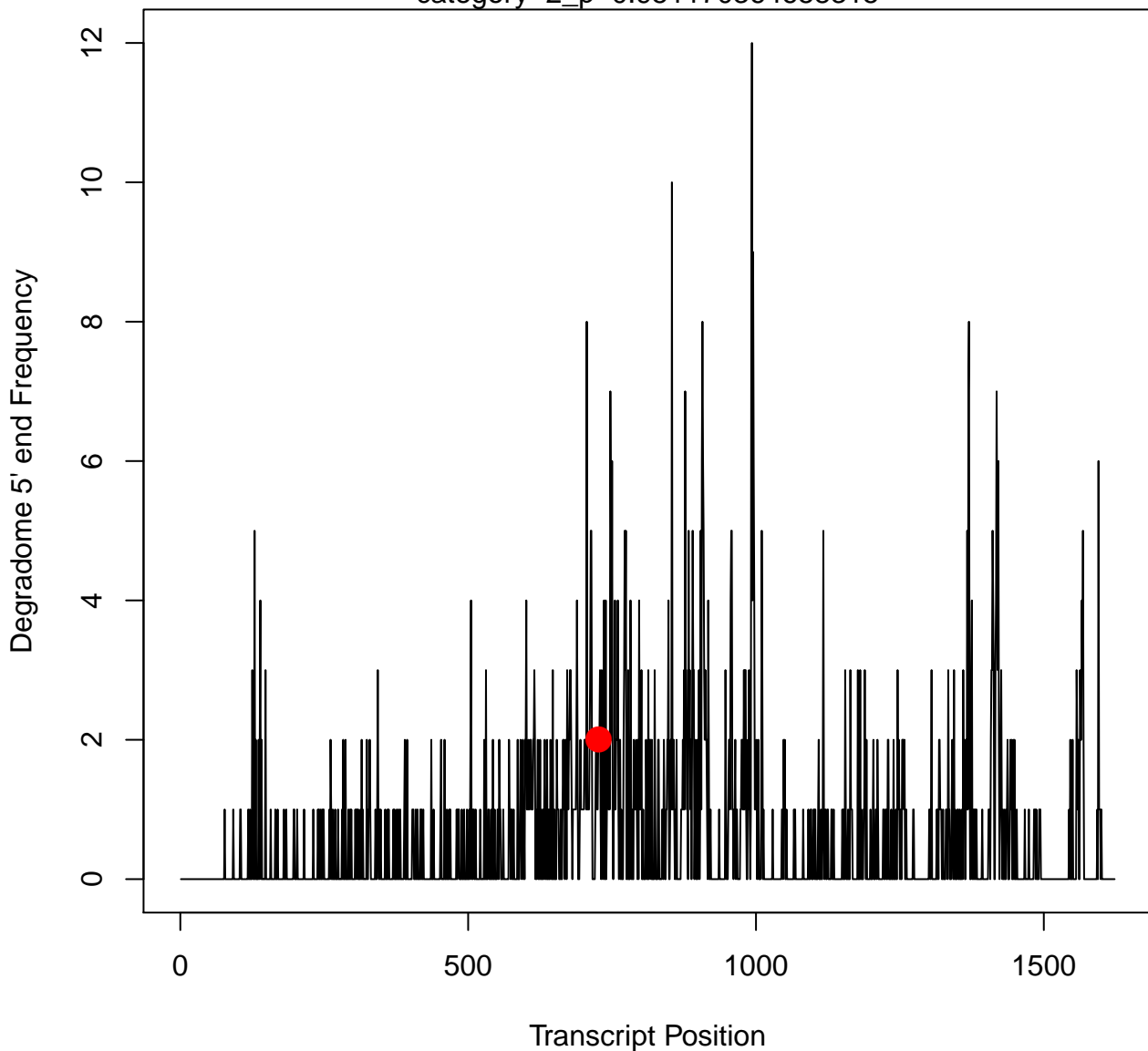

Supplement: Supplementary file 2 [file Data_Sheet_8.ZIP › GSM2230747.plot/Lsa-miR159a_Lsat_1_v5_gn_2_81541.1_727_TPlot.pdf]

**T=Lsat\_1\_v5\_gn\_3\_4041.1\_Q=Lsa-miR159a\_S=156**

category=2\_p=0.962133355121508

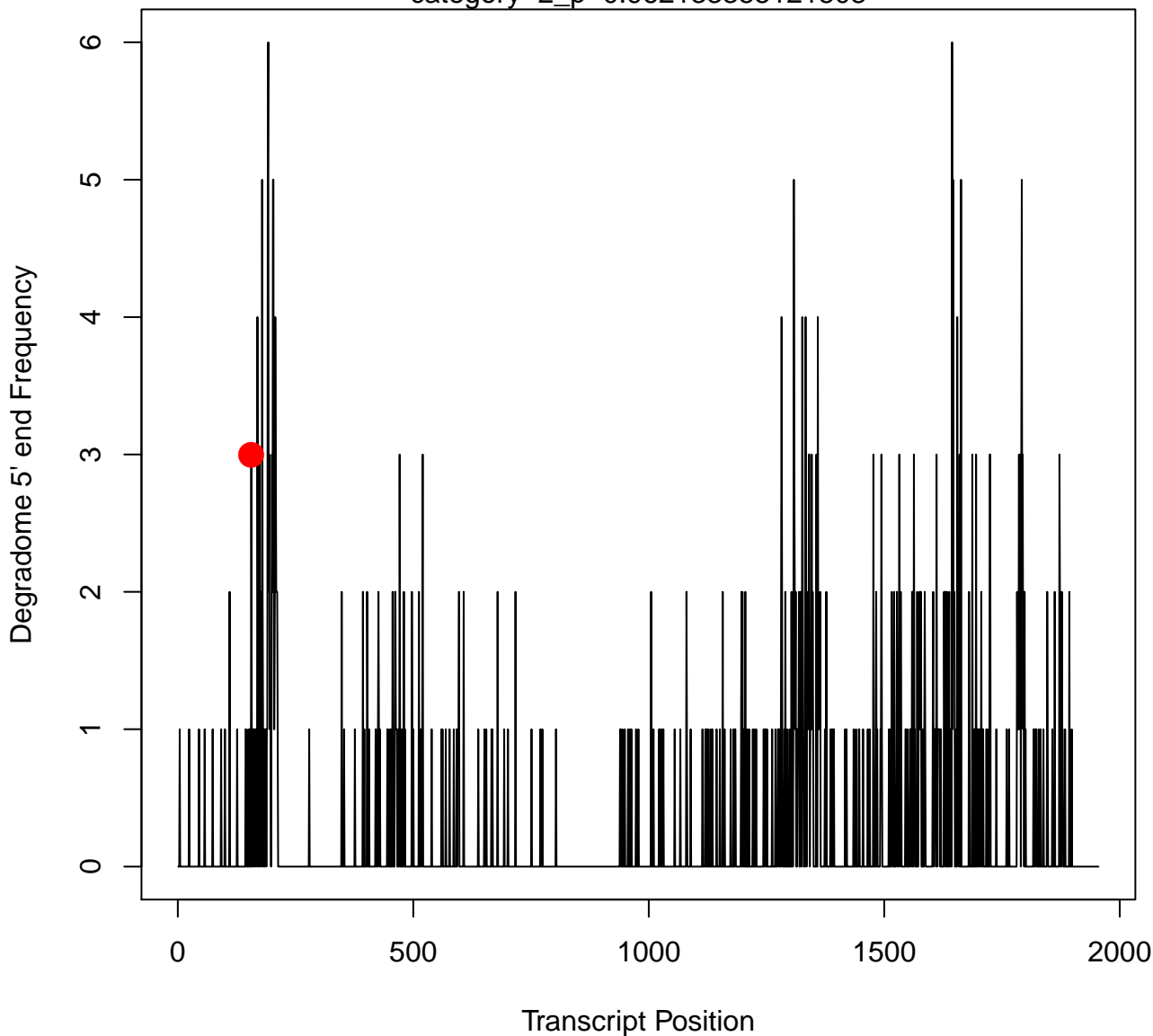

Supplement: Supplementary file 2 [file Data_Sheet_8.ZIP › GSM2230747.plot/Lsa-miR159a_Lsat_1_v5_gn_3_4041.1_156_TPlot.pdf]

T=Lsat\_1\_v5\_gn\_4\_12381.1\_Q=Lsa-miR159a\_S=621

category=2\_p=0.173619597637671

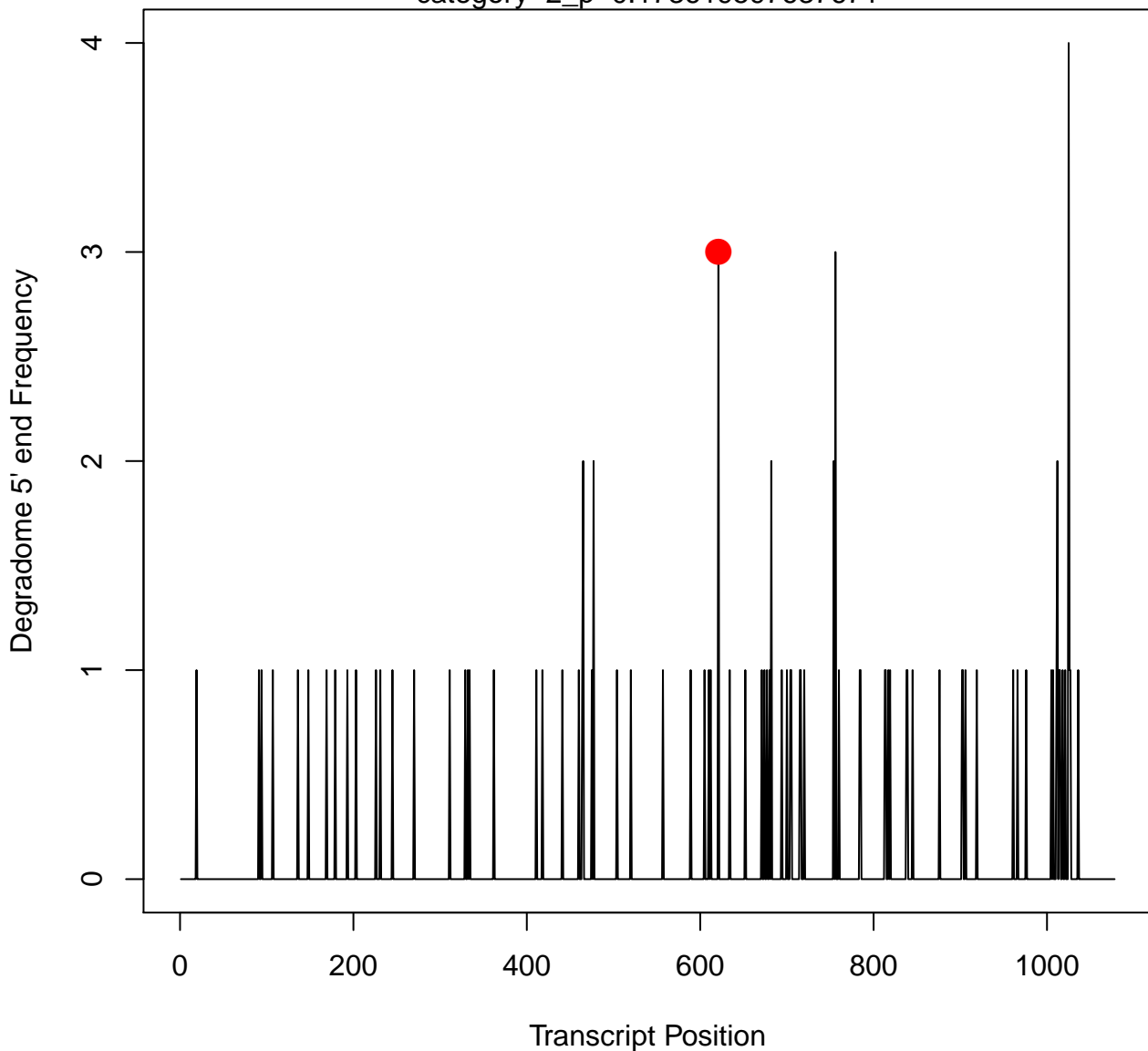

Supplement: Supplementary file 2 [file Data_Sheet_8.ZIP › GSM2230747.plot/Lsa-miR159a_Lsat_1_v5_gn_4_12381.1_621_TPlot.pdf]

**T=Lsat\_1\_v5\_gn\_4\_92901.1\_Q=Lsa-miR159a\_S=439**

category=2\_p=0.99847201068572

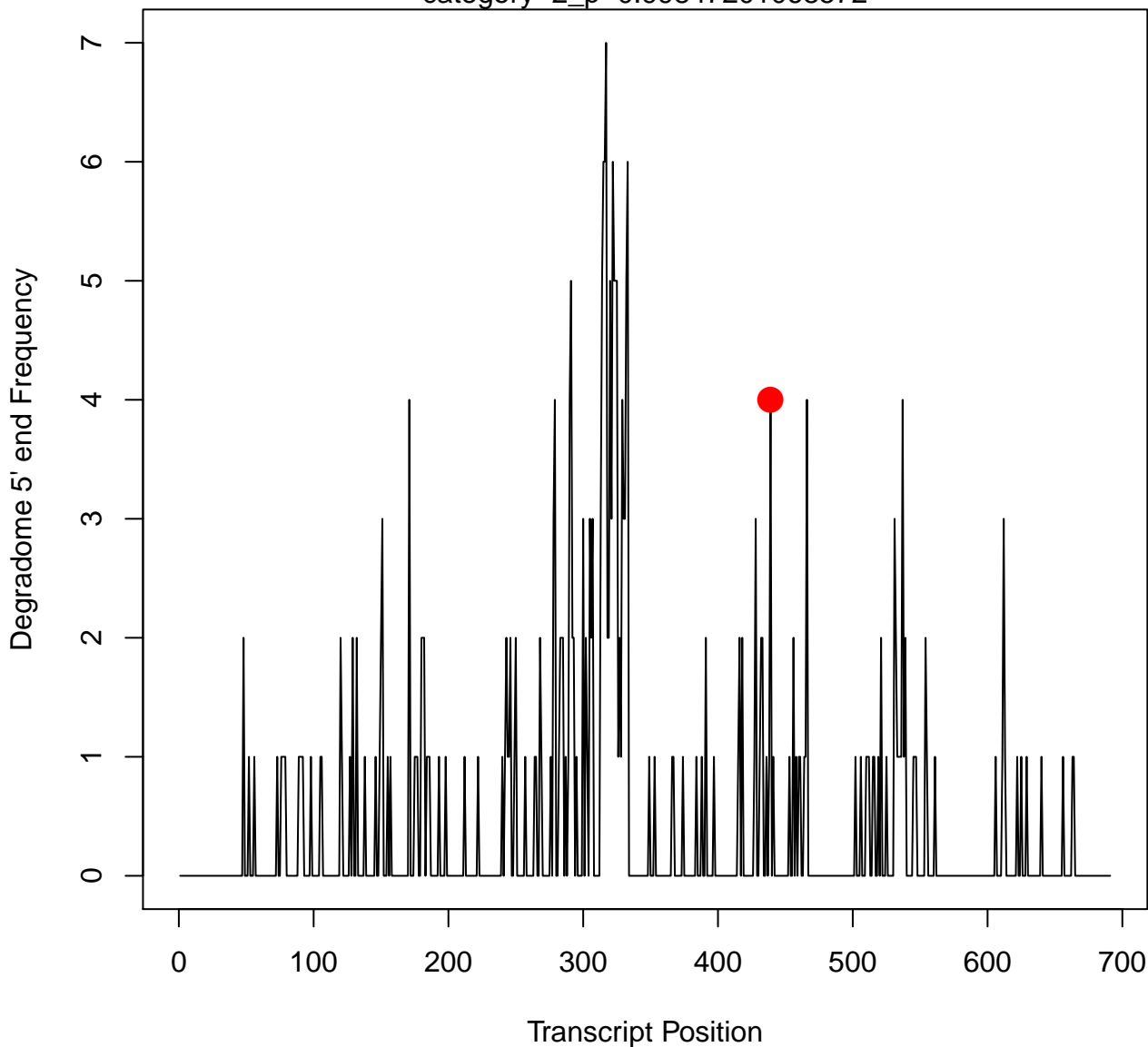

Supplement: Supplementary file 2 [file Data_Sheet_8.ZIP › GSM2230747.plot/Lsa-miR159a_Lsat_1_v5_gn_4_92901.1_439_TPlot.pdf]

**T=Lsat\_1\_v5\_gn\_5\_113161.1\_Q=Lsa-miR159a\_S=274**

category=2\_p=0.996279394644165

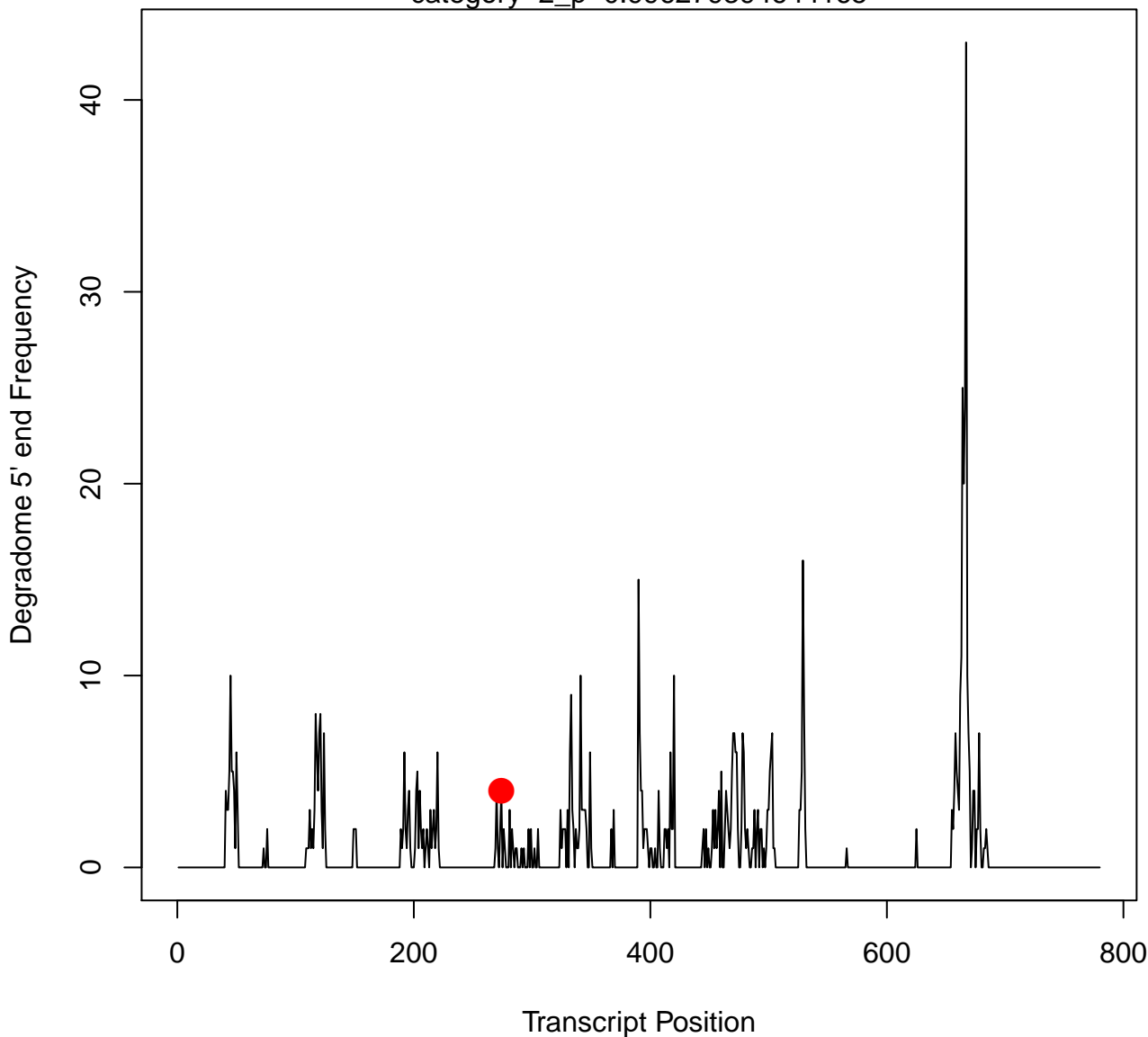

Supplement: Supplementary file 2 [file Data_Sheet_8.ZIP › GSM2230747.plot/Lsa-miR159a_Lsat_1_v5_gn_5_113161.1_274_TPlot.pdf]

**T=Lsat\_1\_v5\_gn\_5\_129201.1\_Q=Lsa-miR159a\_S=733**

category=0\_p=0.00588612148084522

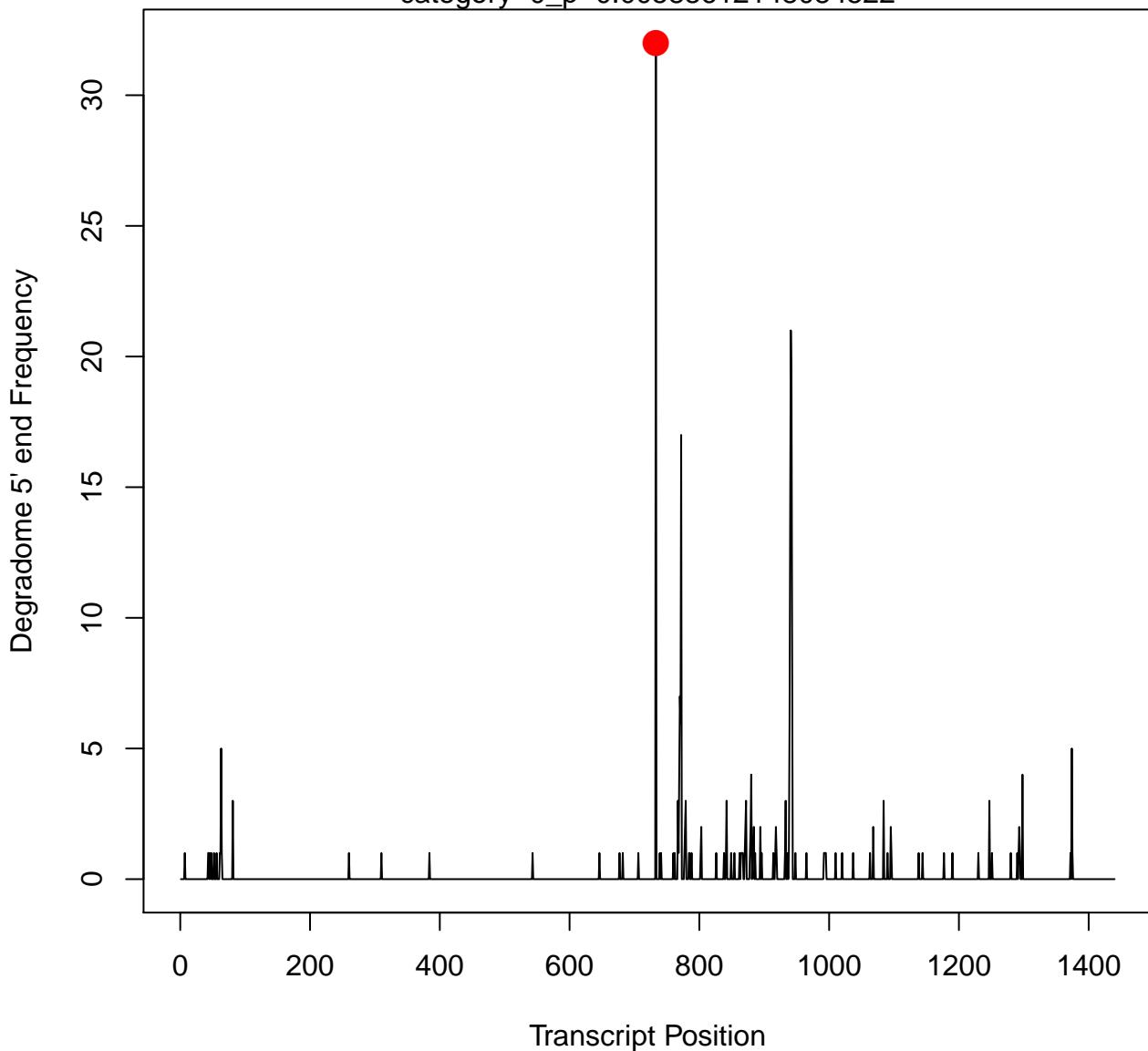

Supplement: Supplementary file 2 [file Data_Sheet_8.ZIP › GSM2230747.plot/Lsa-miR159a_Lsat_1_v5_gn_5_129201.1_733_TPlot.pdf]

**T=Lsat\_1\_v5\_gn\_5\_82881.1\_Q=Lsa-miR159a\_S=2714**

category=2\_p=0.998519811637763

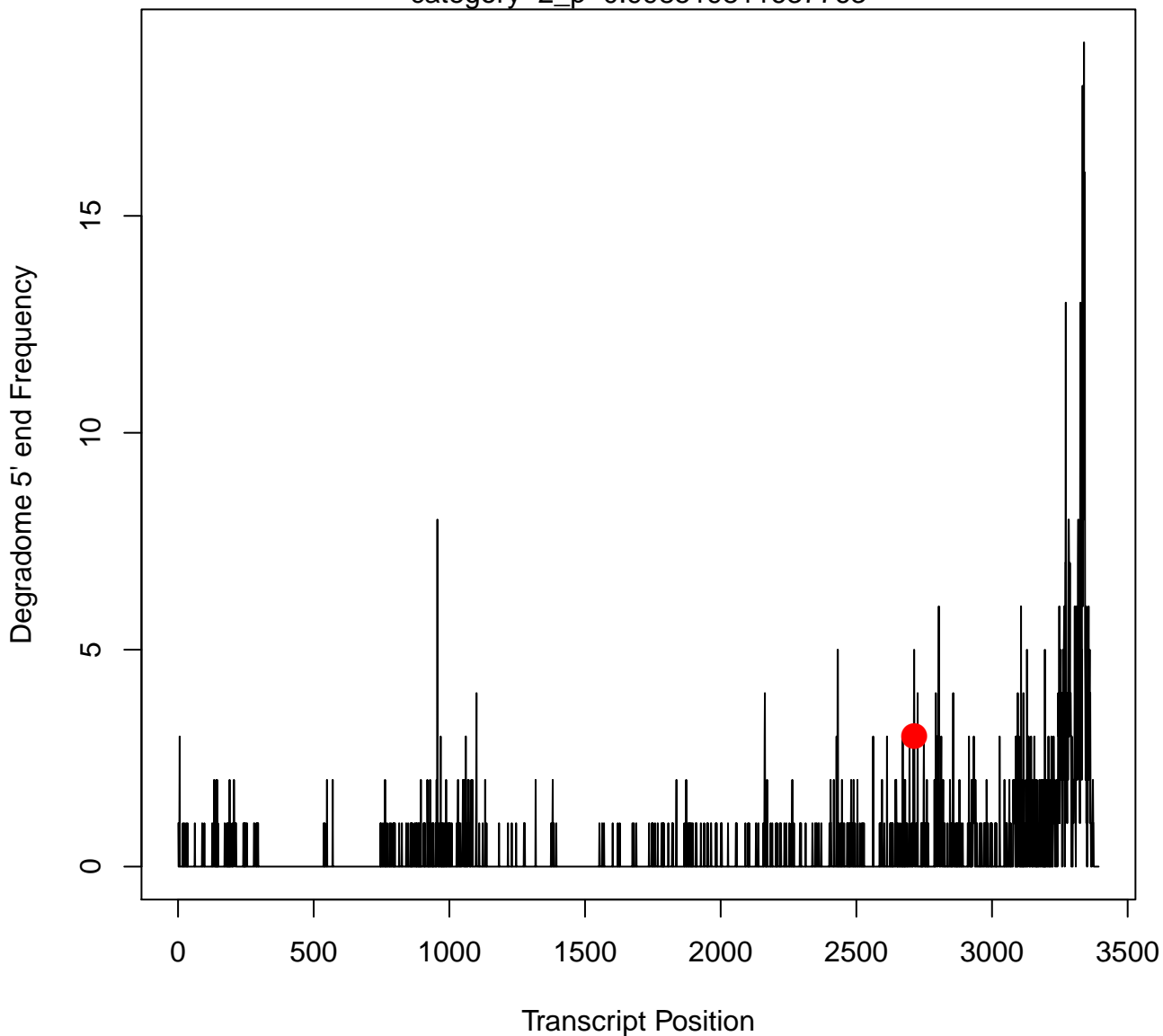

Supplement: Supplementary file 2 [file Data_Sheet_8.ZIP › GSM2230747.plot/Lsa-miR159a_Lsat_1_v5_gn_5_82881.1_2714_TPlot.pdf]

**T=Lsat\_1\_v5\_gn\_7\_103061.1\_Q=Lsa-miR159a\_S=448**

category=0\_p=0.00110629628501624

Degradome 5' end Frequency

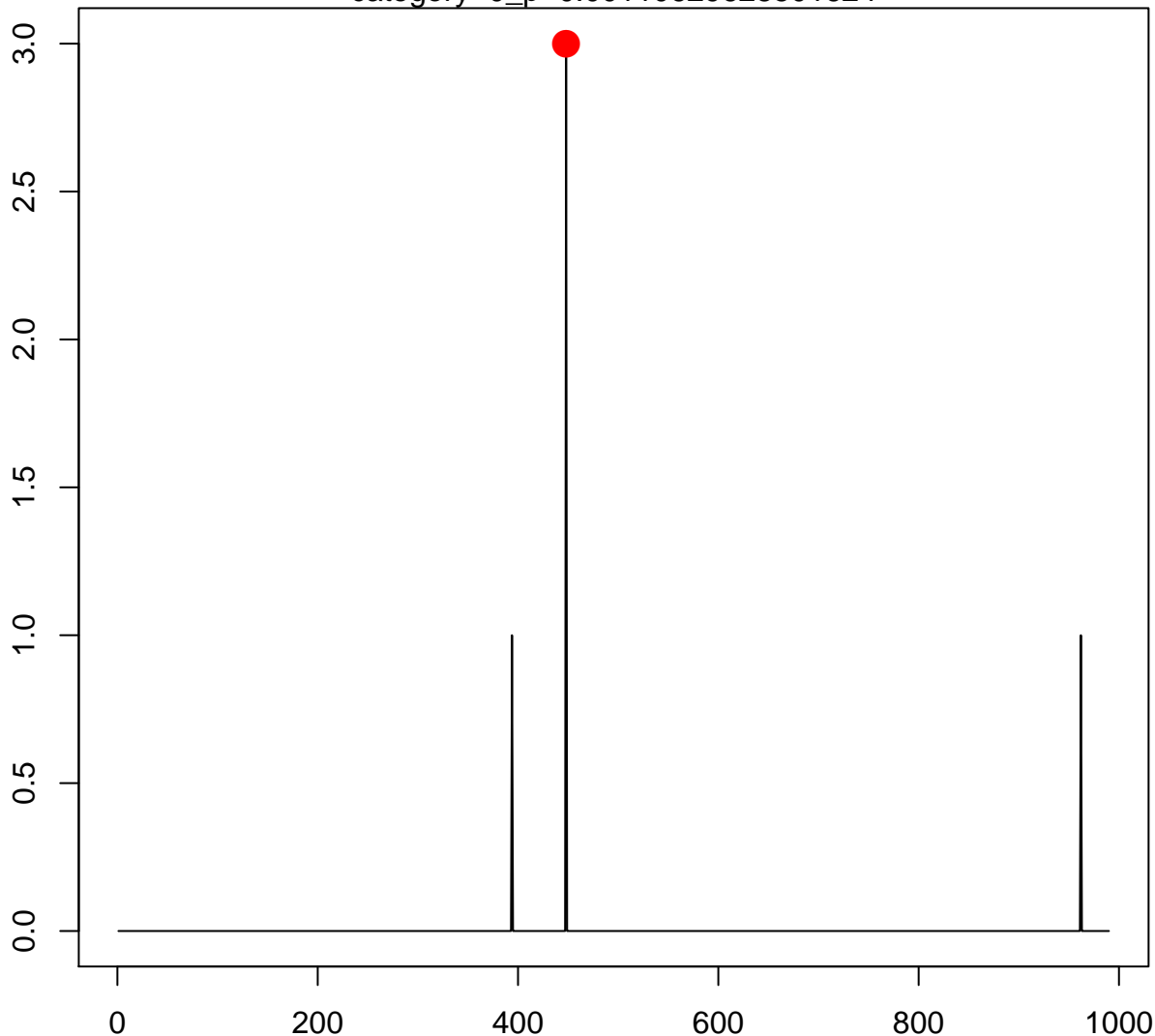

Transcript Position

Supplement: Supplementary file 2 [file Data_Sheet_8.ZIP › GSM2230747.plot/Lsa-miR159a_Lsat_1_v5_gn_7_103061.1_448_TPlot.pdf]

**T=Lsat\_1\_v5\_gn\_7\_23540.1\_Q=Lsa-miR159a\_S=697**

category=2\_p=0.990345860715831

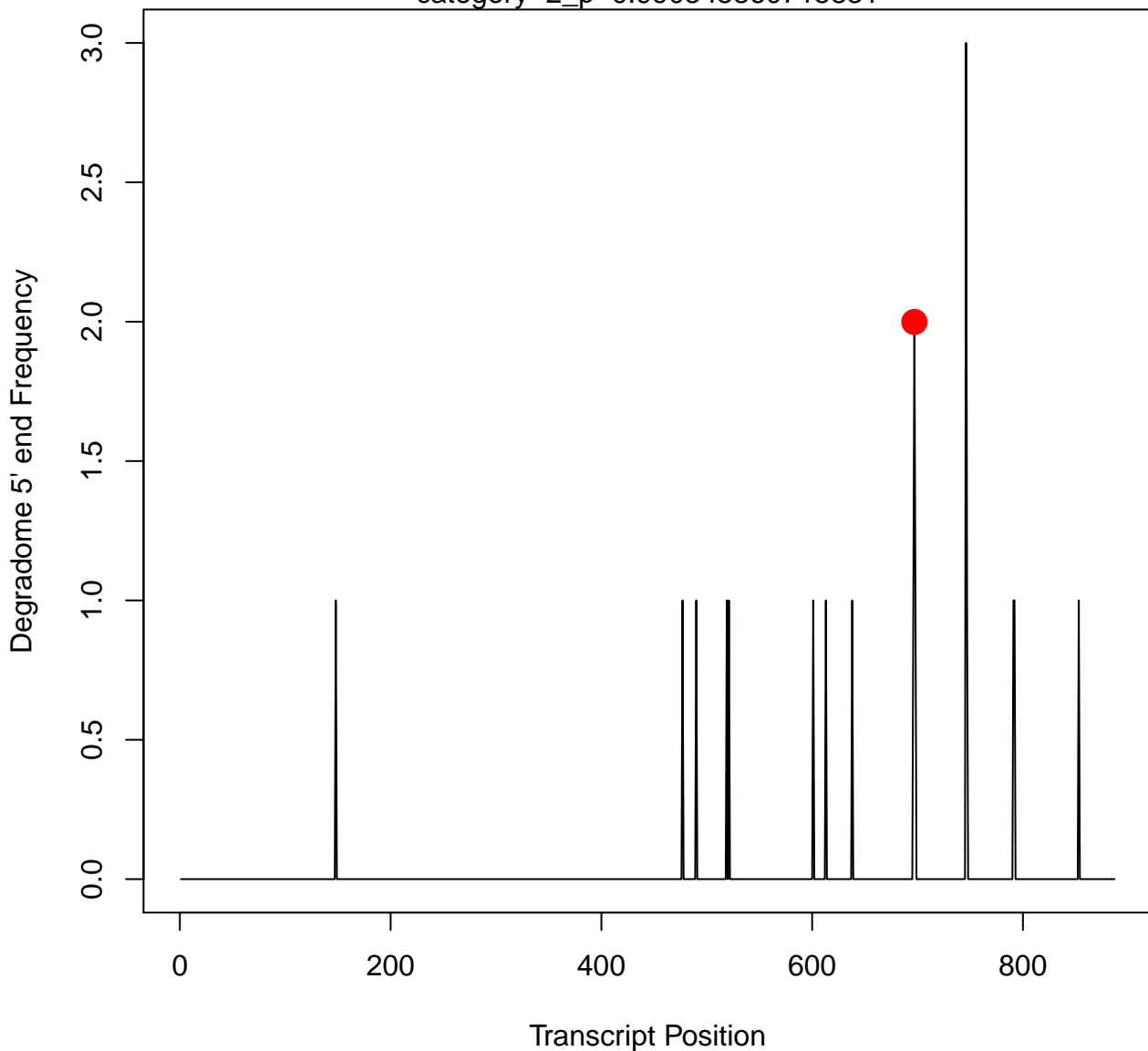

Supplement: Supplementary file 2 [file Data_Sheet_8.ZIP › GSM2230747.plot/Lsa-miR159a_Lsat_1_v5_gn_7_23540.1_697_TPlot.pdf]

**T=Lsat\_1\_v5\_gn\_8\_123241.1\_Q=Lsa-miR159a\_S=401**

category=2\_p=0.995202220757643

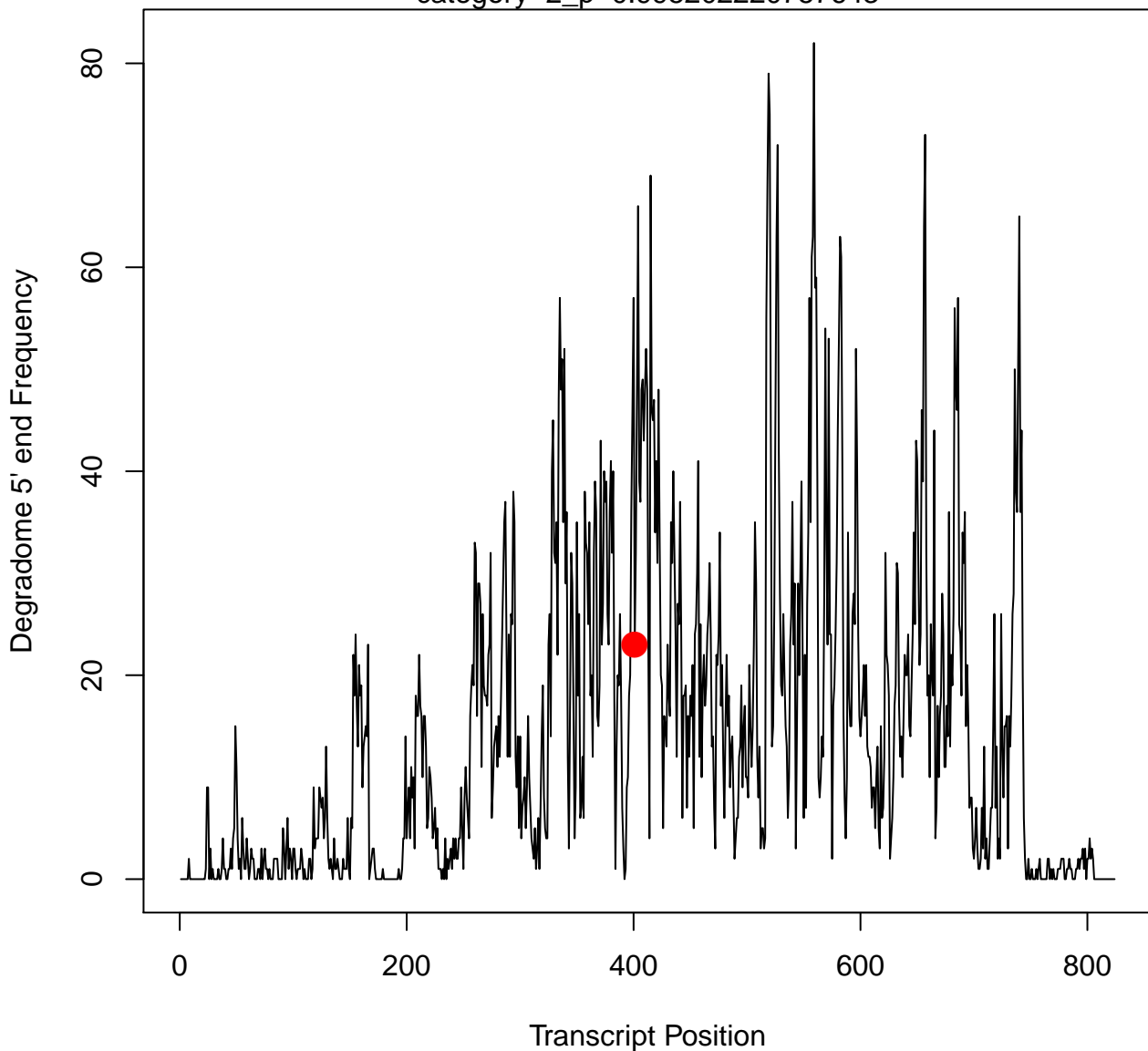

Supplement: Supplementary file 2 [file Data_Sheet_8.ZIP › GSM2230747.plot/Lsa-miR159a_Lsat_1_v5_gn_8_123241.1_401_TPlot.pdf]

**T=Lsat\_1\_v5\_gn\_8\_17320.1\_Q=Lsa-miR159a\_S=1066**

category=2\_p=0.453315581104755

Degradome 5' end Frequency

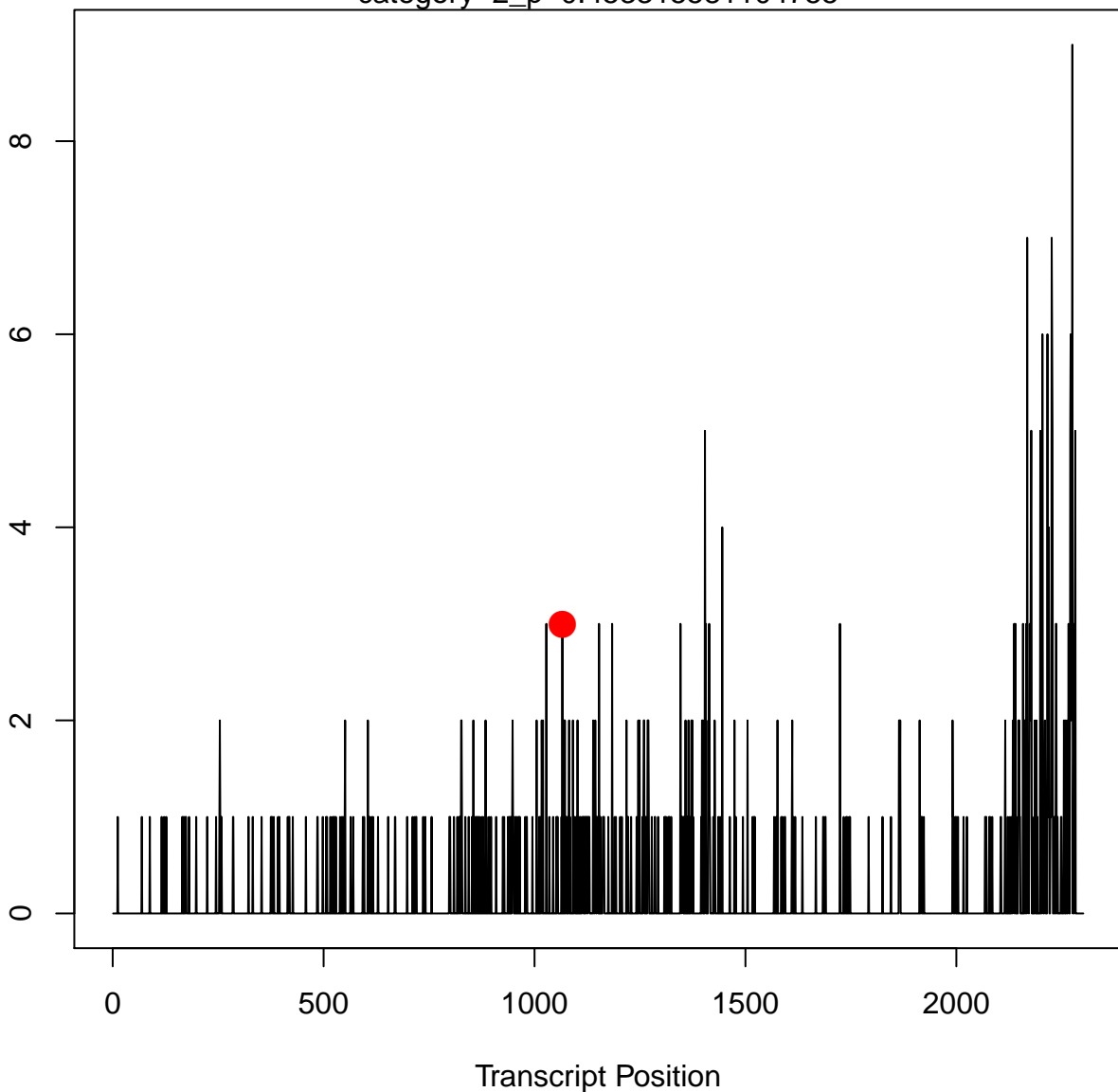

Supplement: Supplementary file 2 [file Data_Sheet_8.ZIP › GSM2230747.plot/Lsa-miR159a_Lsat_1_v5_gn_8_17320.1_1066_TPlot.pdf]

**T=Lsat\_1\_v5\_gn\_8\_31840.1\_Q=Lsa-miR159a\_S=285**

category=2\_p=0.753024768193277

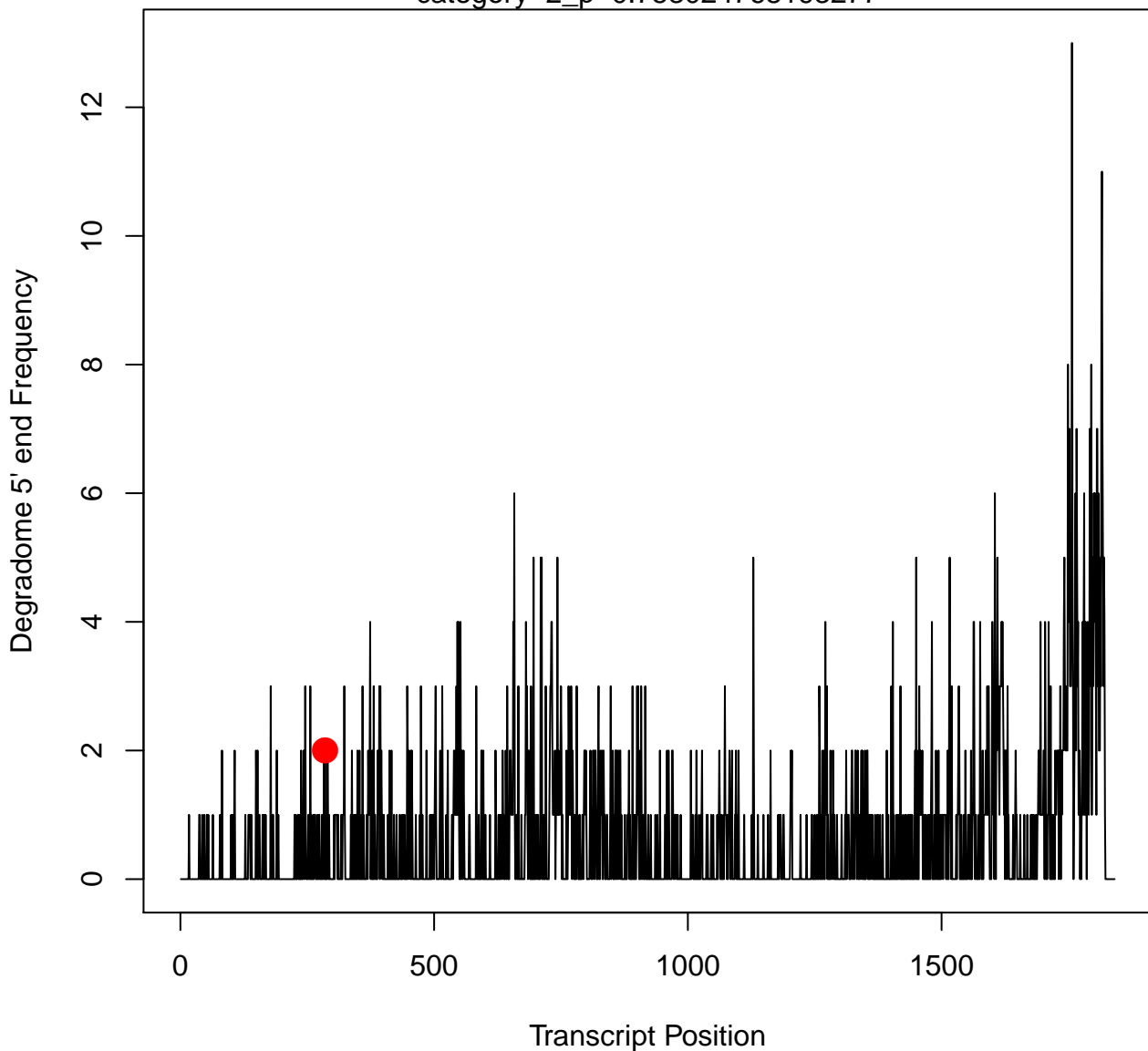

Supplement: Supplementary file 2 [file Data_Sheet_8.ZIP › GSM2230747.plot/Lsa-miR159a_Lsat_1_v5_gn_8_31840.1_285_TPlot.pdf]

**T=Lsat\_1\_v5\_gn\_8\_62341.1\_Q=Lsa-miR159a\_S=3194**

category=2\_p=0.736815647291125

Degradome 5' end Frequency

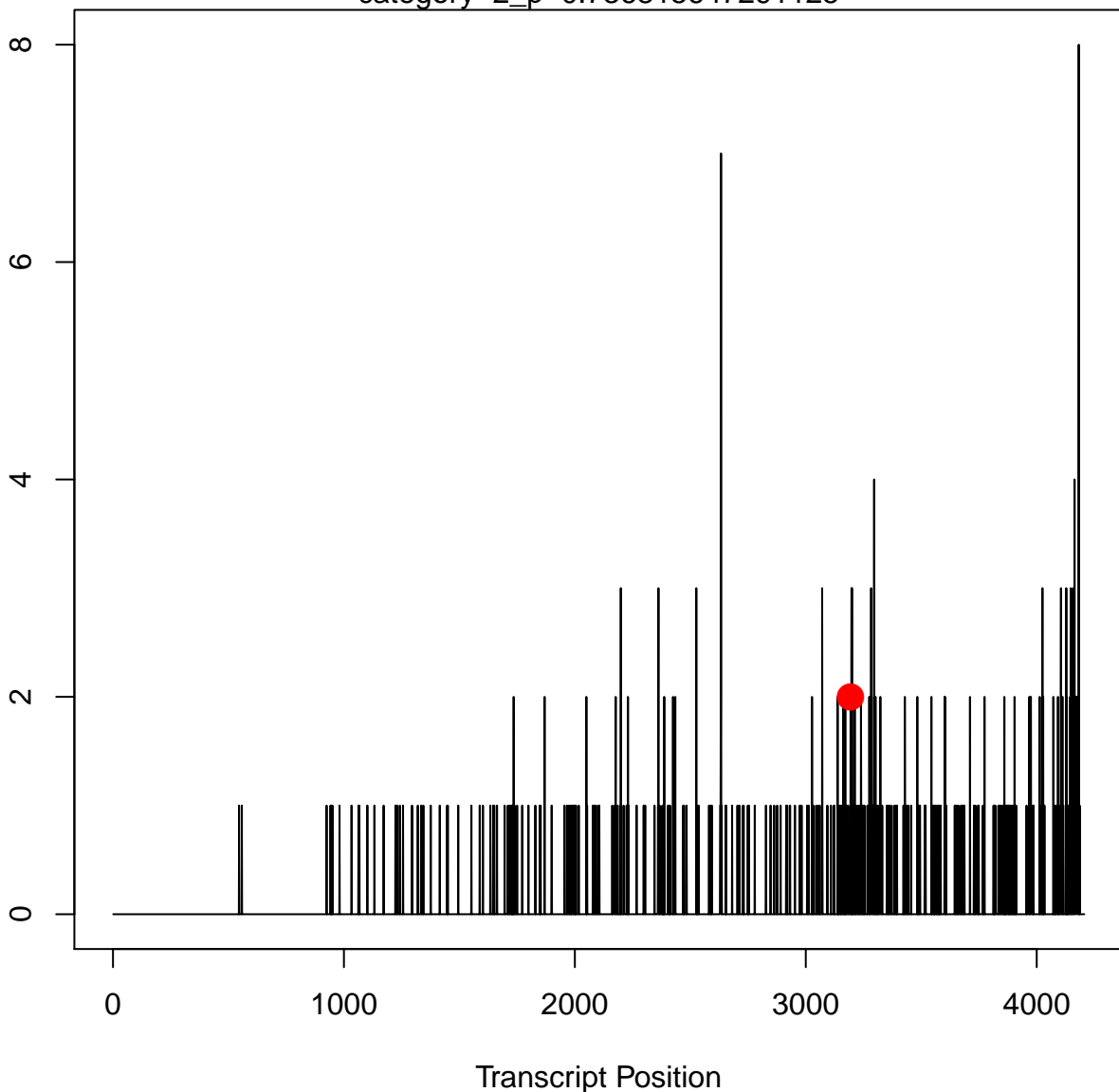

Supplement: Supplementary file 2 [file Data_Sheet_8.ZIP › GSM2230747.plot/Lsa-miR159a_Lsat_1_v5_gn_8_62341.1_3194_TPlot.pdf]

**T=Lsat\_1\_v5\_gn\_9\_68900.1\_Q=Lsa-miR159a\_S=1976**

category=2\_p=0.999955130576496

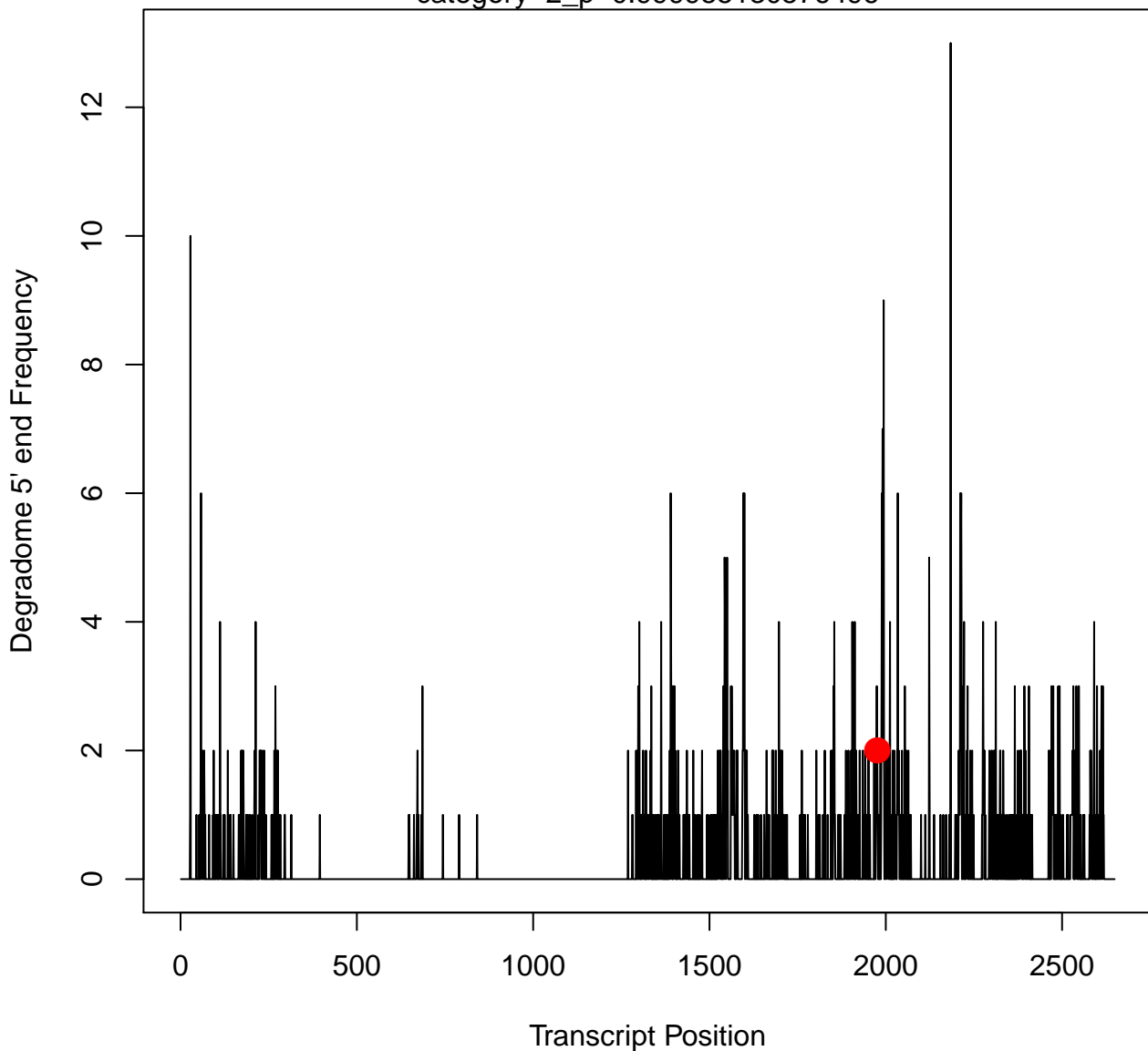

Supplement: Supplementary file 2 [file Data_Sheet_8.ZIP › GSM2230747.plot/Lsa-miR159a_Lsat_1_v5_gn_9_68900.1_1976_TPlot.pdf]

**T=Lsat\_1\_v5\_gn\_9\_9761.1\_Q=Lsa-miR159a\_S=337**

category=2\_p=0.999164672049645

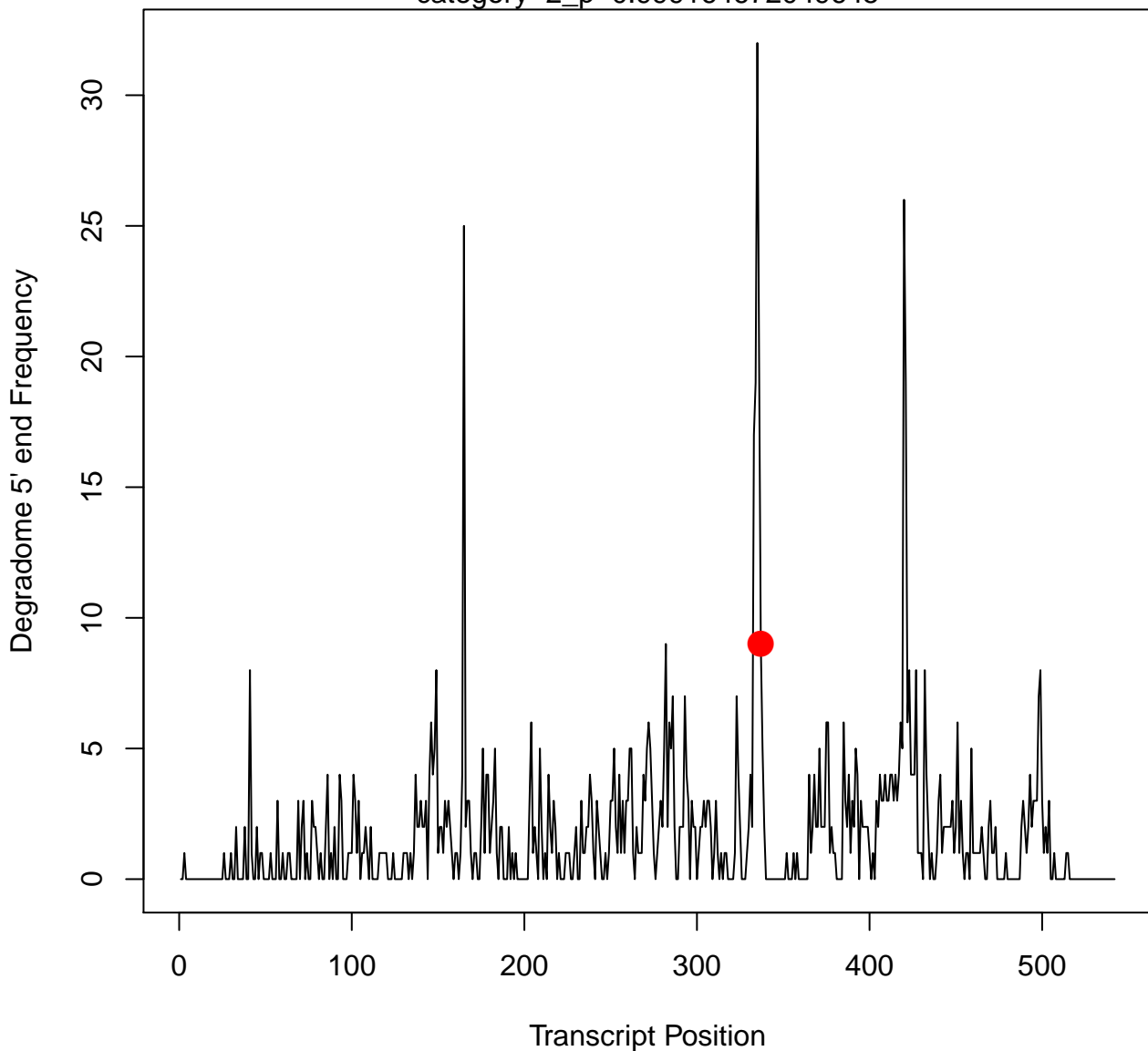

Supplement: Supplementary file 2 [file Data_Sheet_8.ZIP › GSM2230747.plot/Lsa-miR159a_Lsat_1_v5_gn_9_9761.1_337_TPlot.pdf]

**T=Lsat\_1\_v5\_gn\_0\_39281.1\_Q=Lsa-miR159b\_S=2161**

category=0\_p=0.000368901499921082

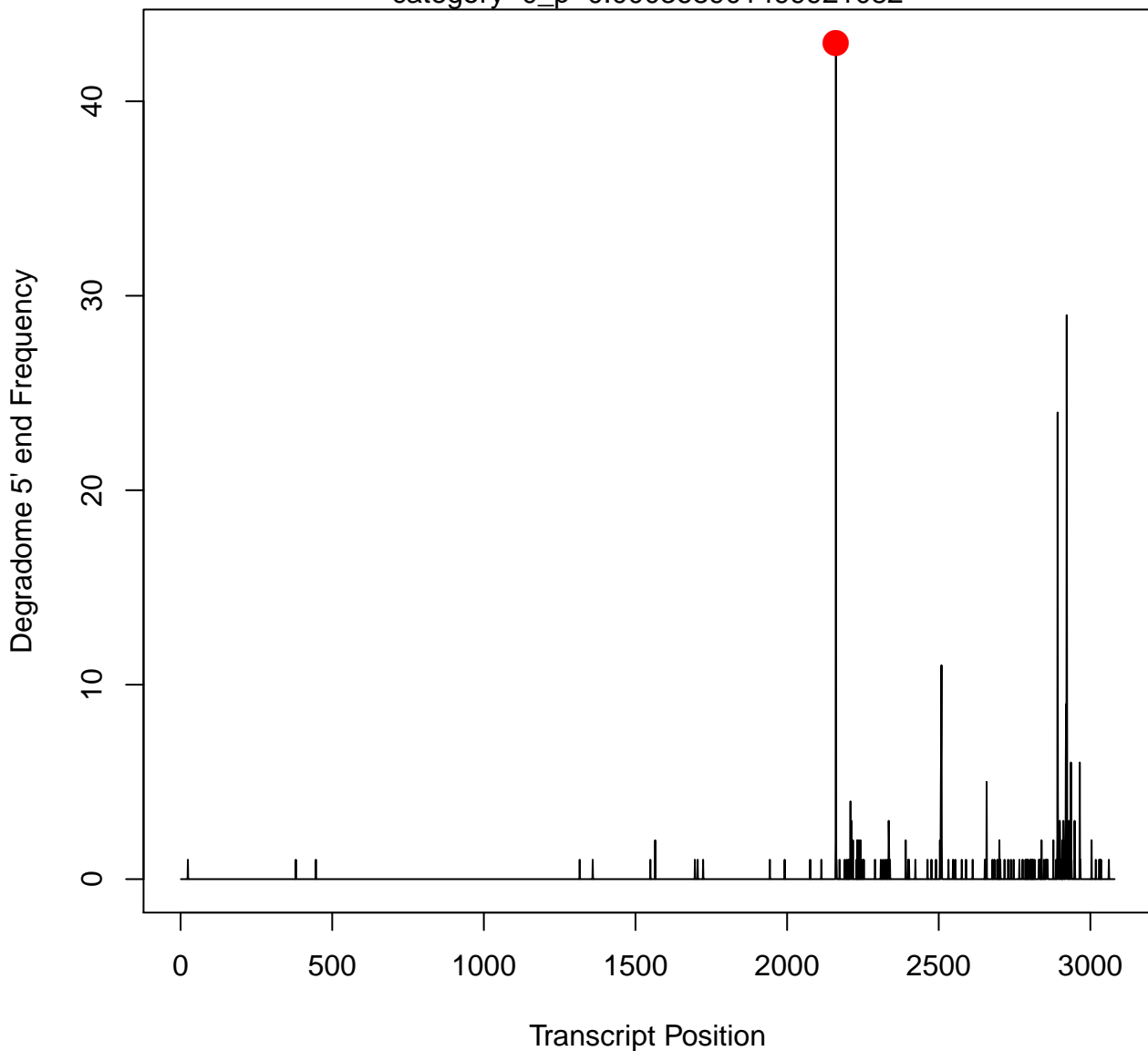

Supplement: Supplementary file 2 [file Data_Sheet_8.ZIP › GSM2230747.plot/Lsa-miR159b_Lsat_1_v5_gn_0_39281.1_2161_TPlot.pdf]

**T=Lsat\_1\_v5\_gn\_3\_54301.1\_Q=Lsa-miR159b\_S=217**

category=2\_p=0.999940271655757

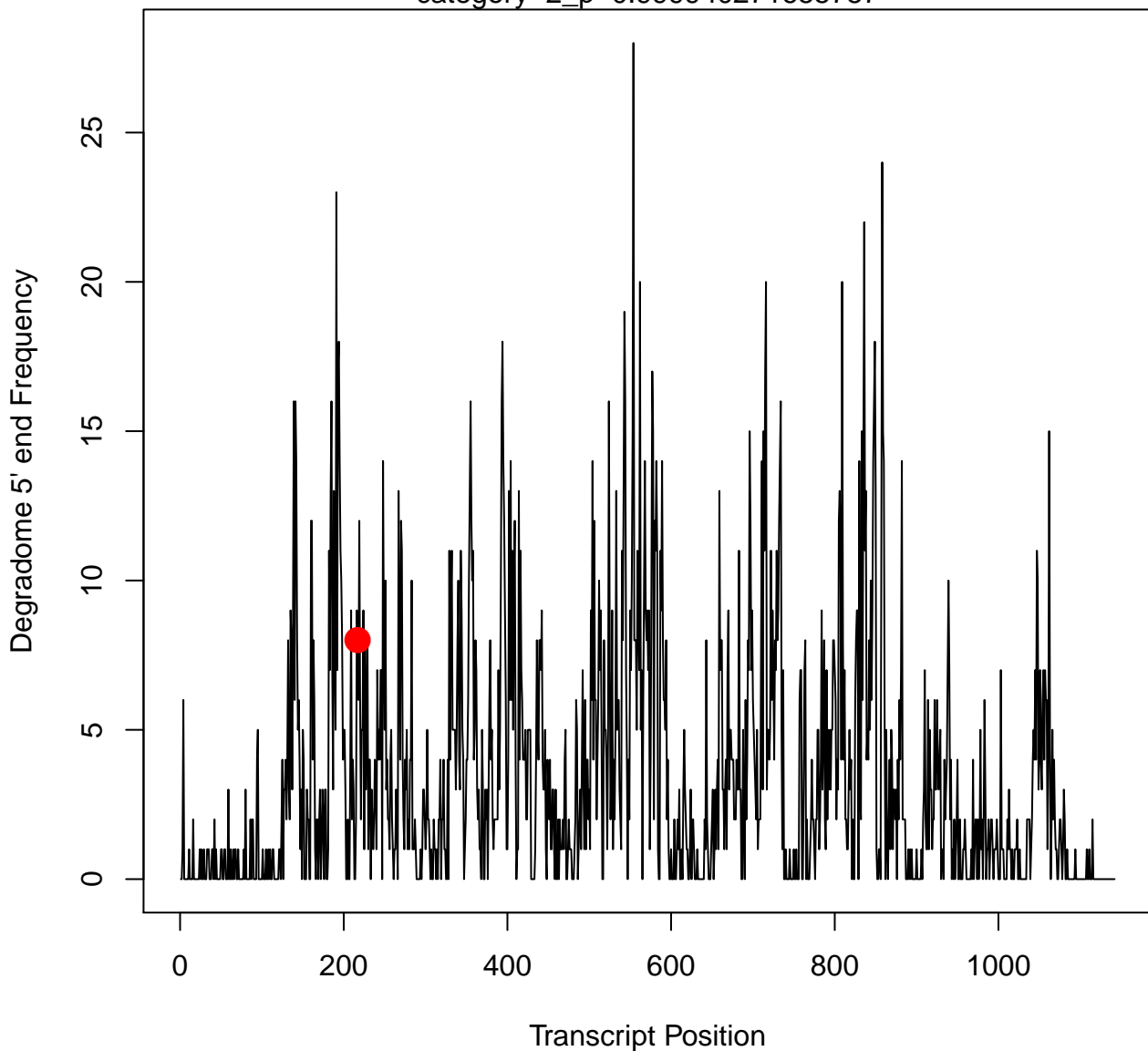

Supplement: Supplementary file 2 [file Data_Sheet_8.ZIP › GSM2230747.plot/Lsa-miR159b_Lsat_1_v5_gn_3_54301.1_217_TPlot.pdf]

**T=Lsat\_1\_v5\_gn\_3\_94000.1\_Q=Lsa-miR159b\_S=137**

category=2\_p=0.417436380683029

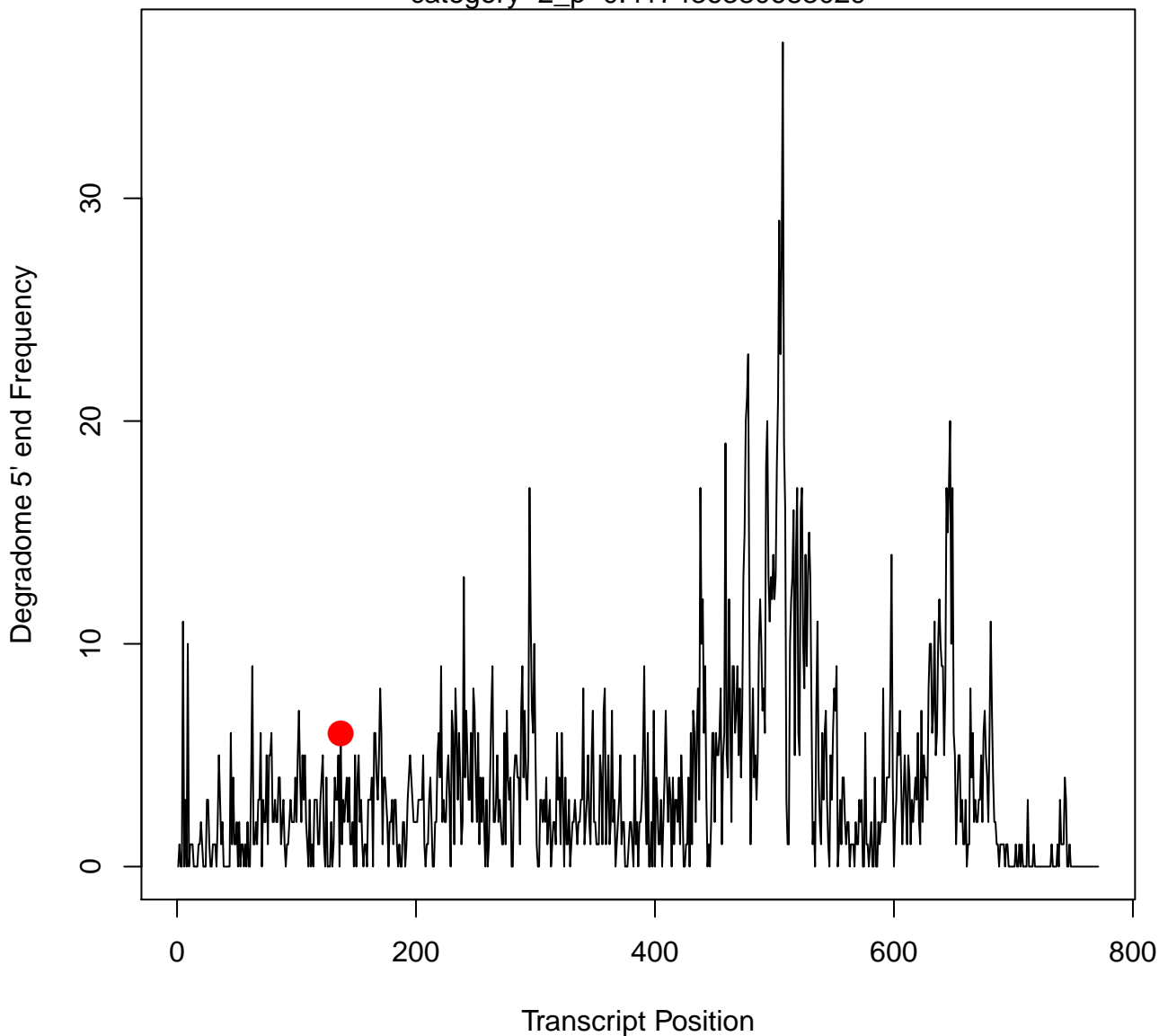

Supplement: Supplementary file 2 [file Data_Sheet_8.ZIP › GSM2230747.plot/Lsa-miR159b_Lsat_1_v5_gn_3_94000.1_137_TPlot.pdf]

**T=Lsat\_1\_v5\_gn\_5\_65281.1\_Q=Lsa-miR159b\_S=180**

category=2\_p=0.999968368933147

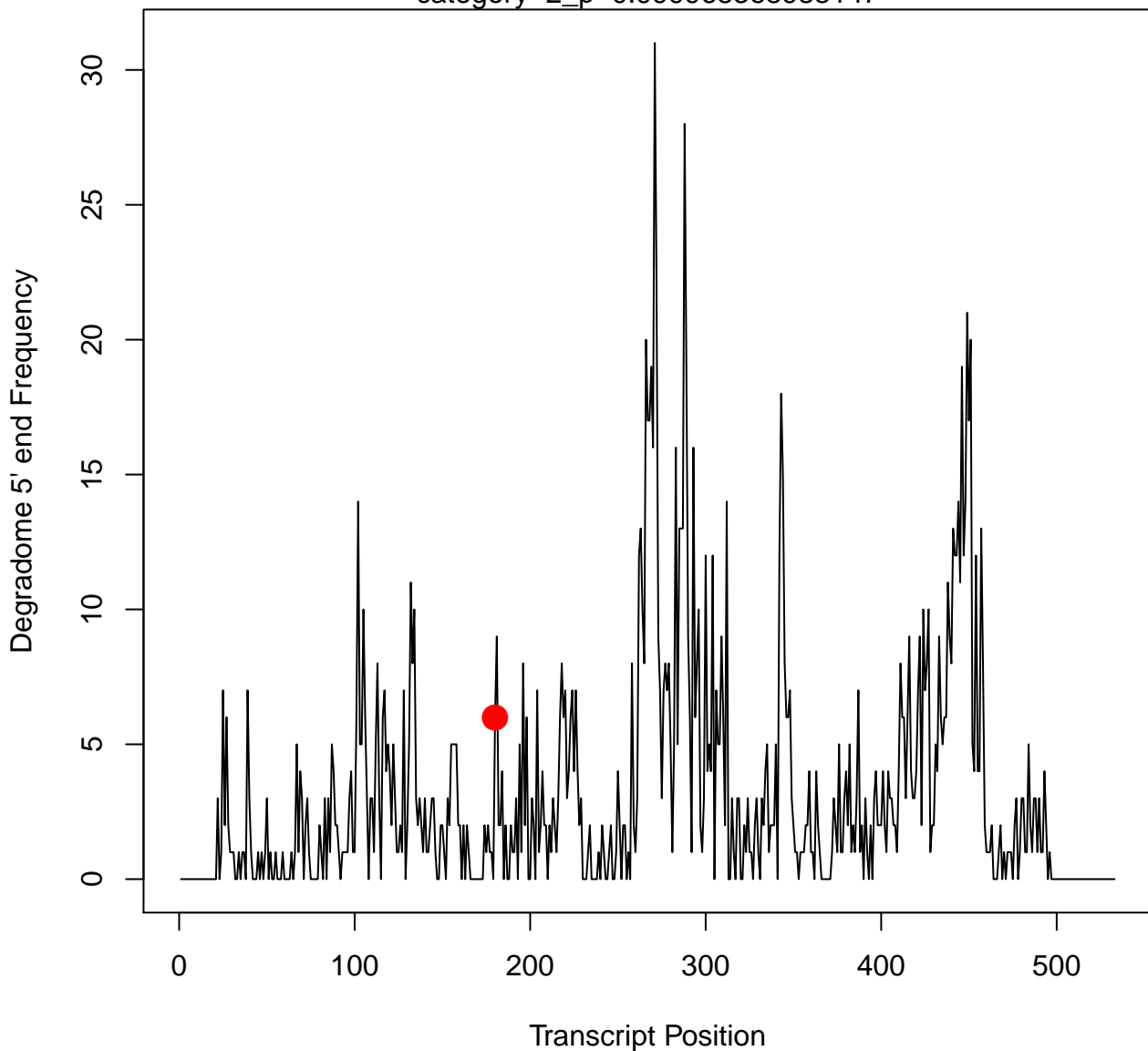

Supplement: Supplementary file 2 [file Data_Sheet_8.ZIP › GSM2230747.plot/Lsa-miR159b_Lsat_1_v5_gn_5_65281.1_180_TPlot.pdf]

**T=Lsat\_1\_v5\_gn\_5\_68180.1\_Q=Lsa-miR159b\_S=795**

category=2\_p=0.989712254221291

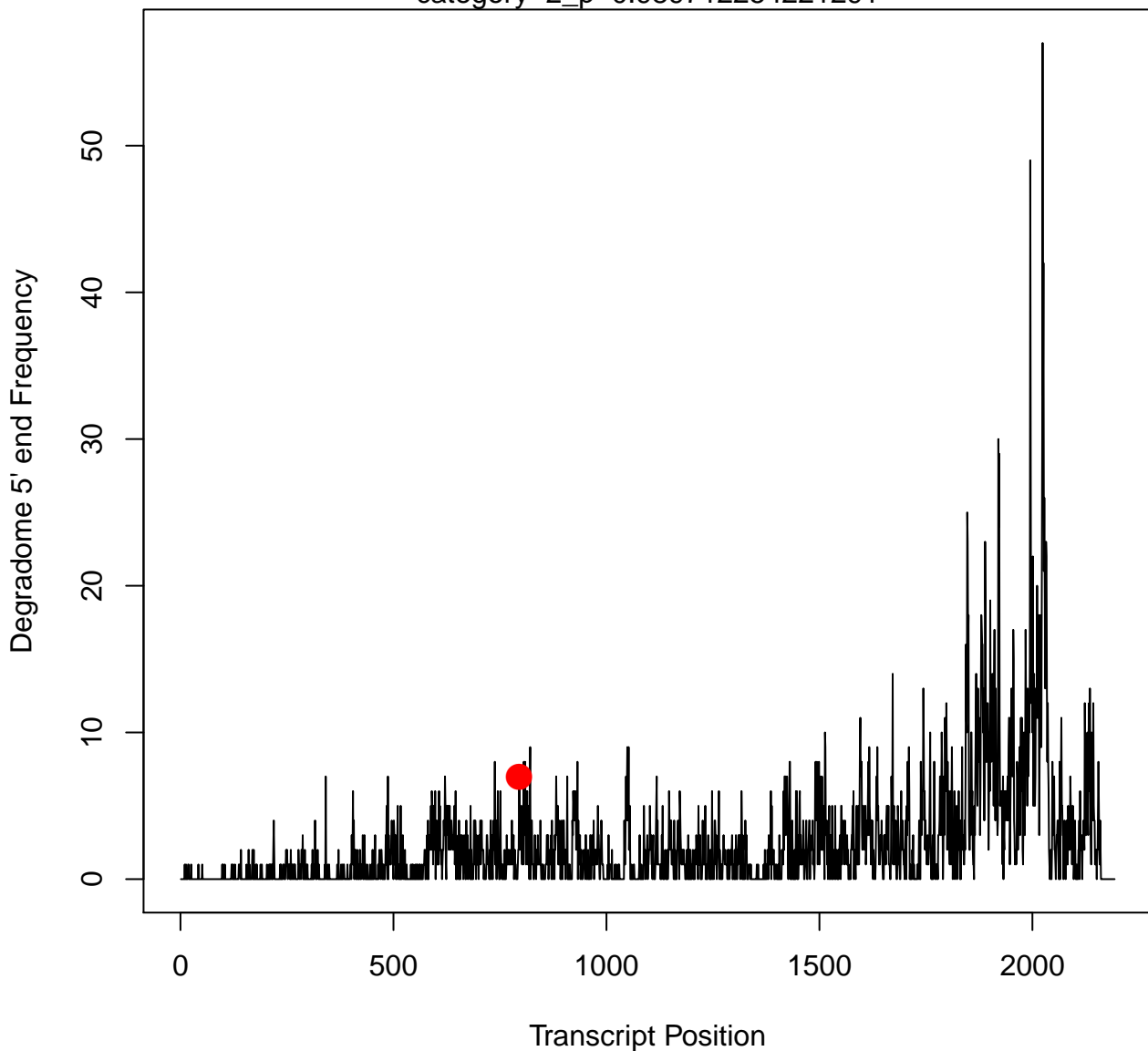

Supplement: Supplementary file 2 [file Data_Sheet_8.ZIP › GSM2230747.plot/Lsa-miR159b_Lsat_1_v5_gn_5_68180.1_795_TPlot.pdf]

**T=Lsat\_1\_v5\_gn\_5\_75800.1\_Q=Lsa-miR159b\_S=1860**

category=2\_p=0.0615884673055831

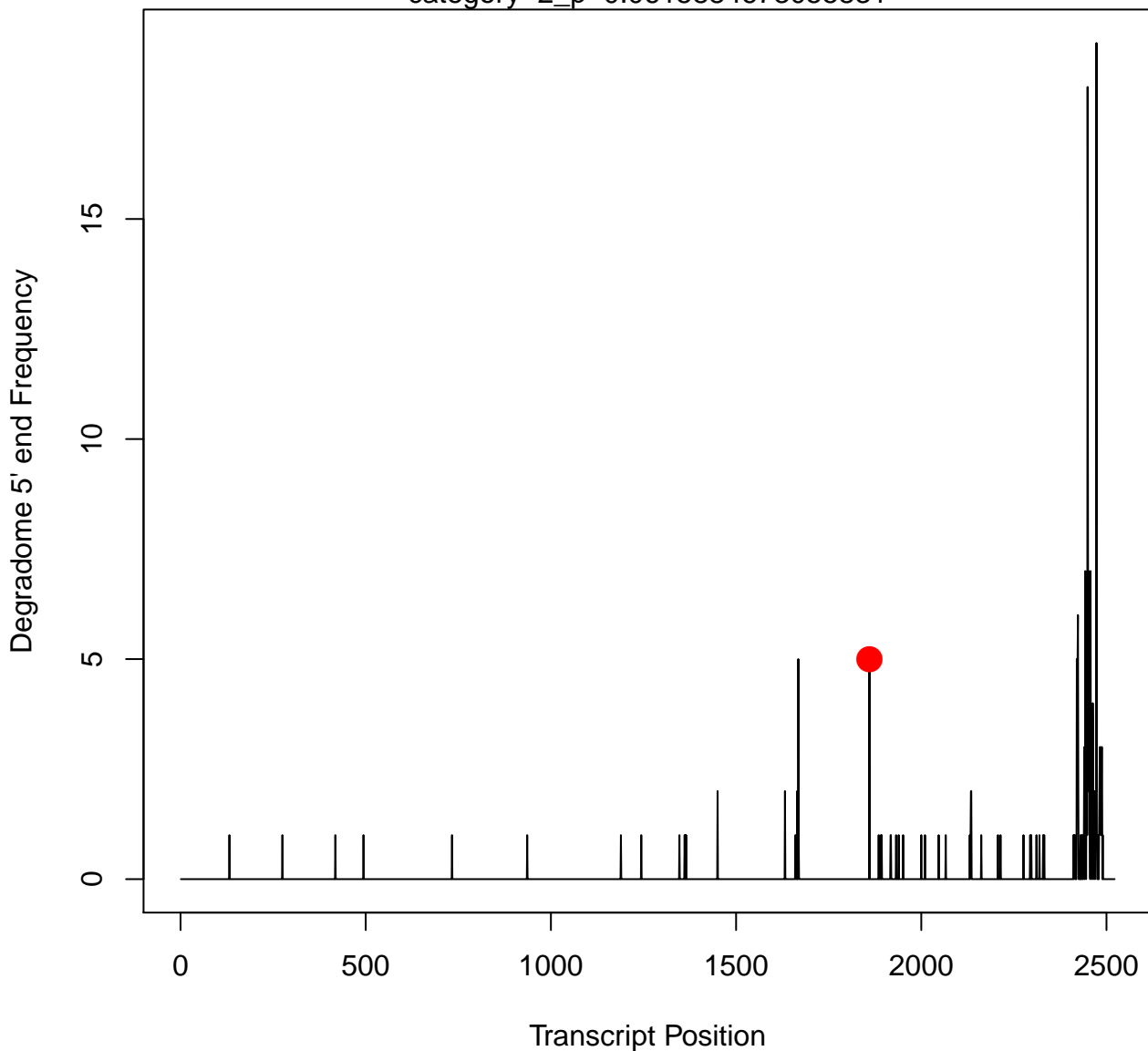

Supplement: Supplementary file 2 [file Data_Sheet_8.ZIP › GSM2230747.plot/Lsa-miR159b_Lsat_1_v5_gn_5_75800.1_1860_TPlot.pdf]

**T=Lsat\_1\_v5\_gn\_7\_29160.1\_Q=Lsa-miR159b\_S=1916**

category=2\_p=0.999927722942035

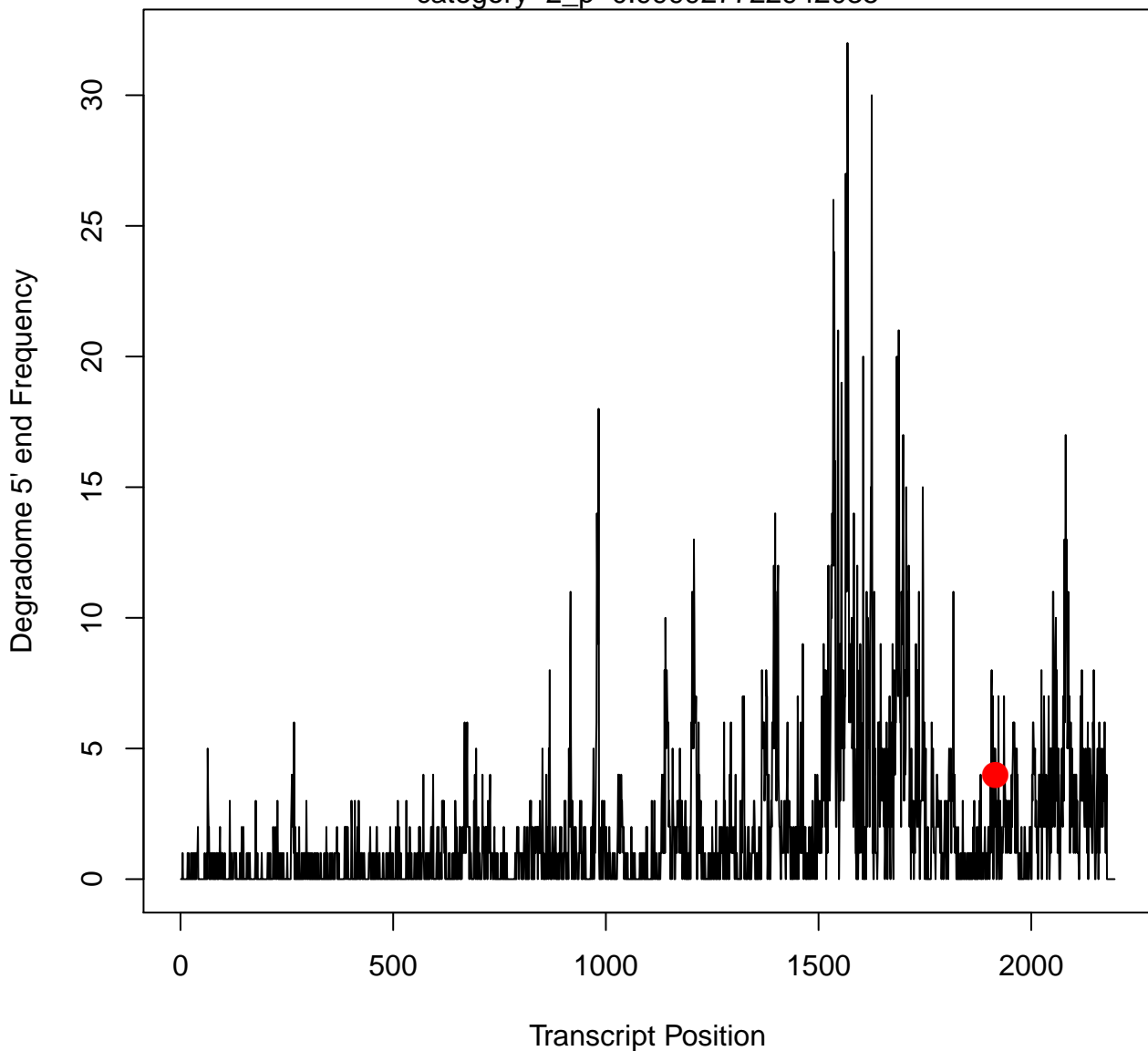

Supplement: Supplementary file 2 [file Data_Sheet_8.ZIP › GSM2230747.plot/Lsa-miR159b_Lsat_1_v5_gn_7_29160.1_1916_TPlot.pdf]

**T=Lsat\_1\_v5\_gn\_7\_35121.1\_Q=Lsa-miR159b\_S=1317**

category=2\_p=0.998956529115271

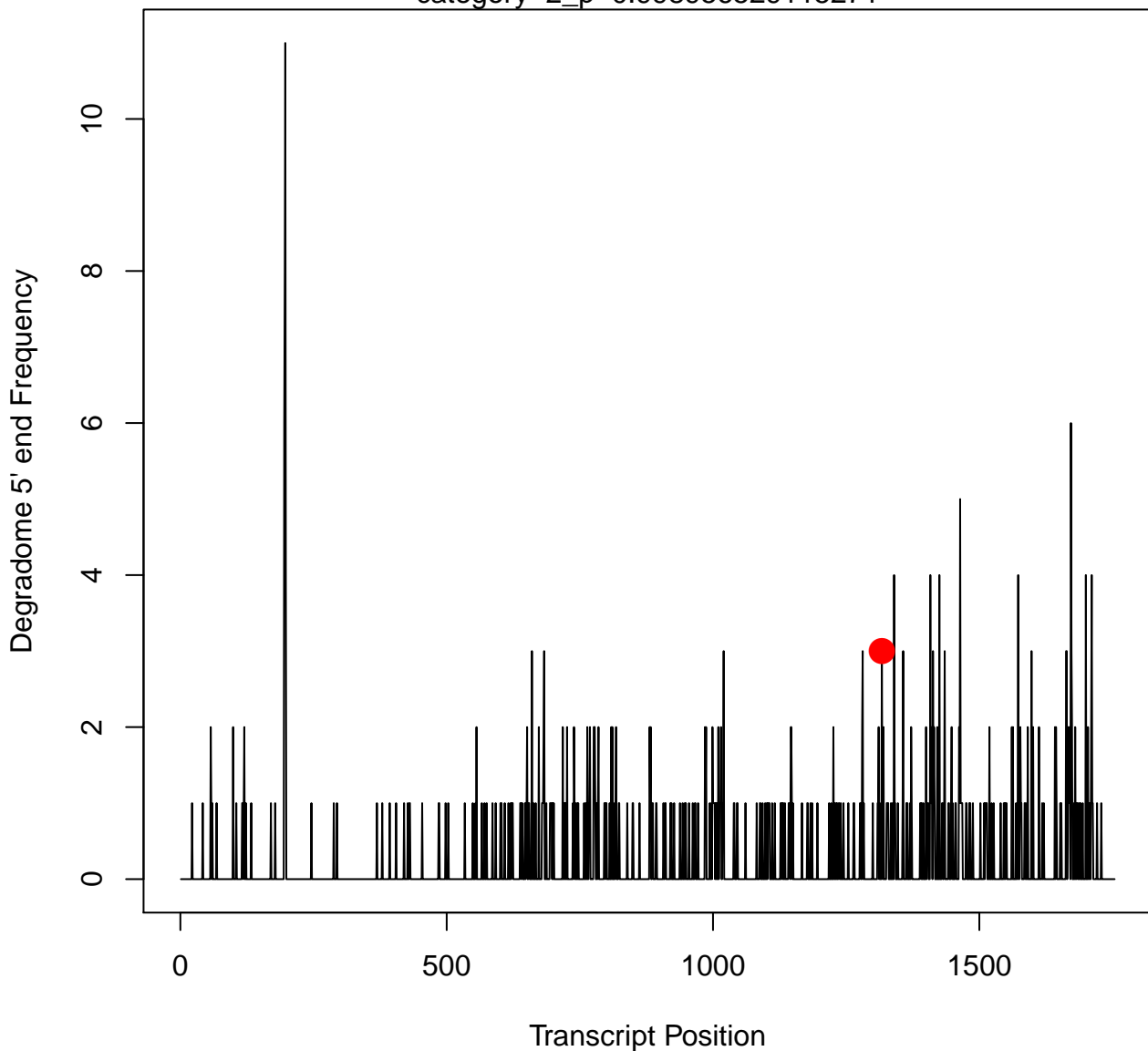

Supplement: Supplementary file 2 [file Data_Sheet_8.ZIP › GSM2230747.plot/Lsa-miR159b_Lsat_1_v5_gn_7_35121.1_1317_TPlot.pdf]

**T=Lsat\_1\_v5\_gn\_9\_121780.1\_Q=Lsa-miR159b\_S=2972**

category=2\_p=0.99939211182474

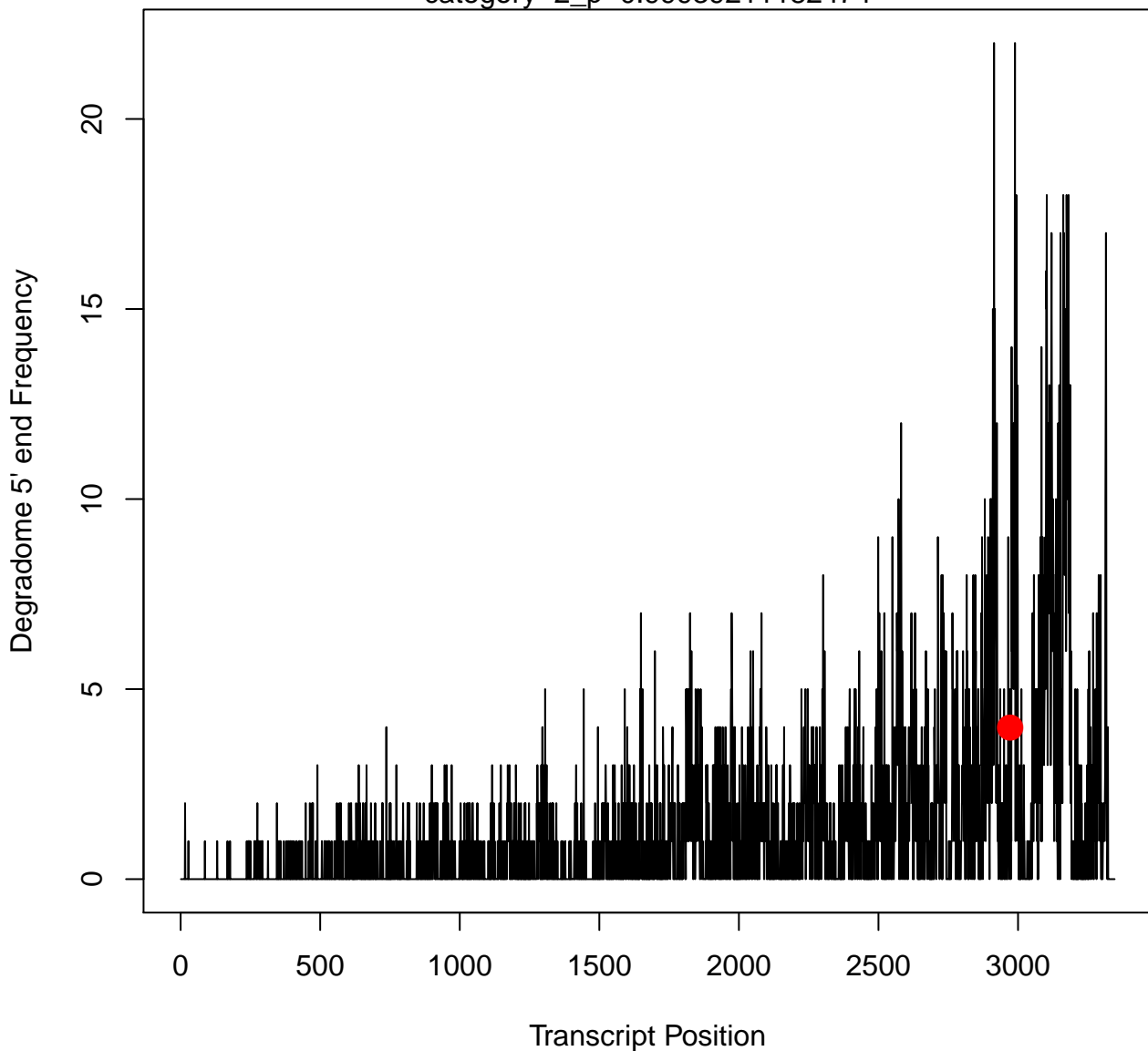

Supplement: Supplementary file 2 [file Data_Sheet_8.ZIP › GSM2230747.plot/Lsa-miR159b_Lsat_1_v5_gn_9_121780.1_2972_TPlot.pdf]

**T=Lsat\_1\_v5\_gn\_2\_100181.1\_Q=Lsa-miR160a\_S=2076**

category=2\_p=0.119383795306115

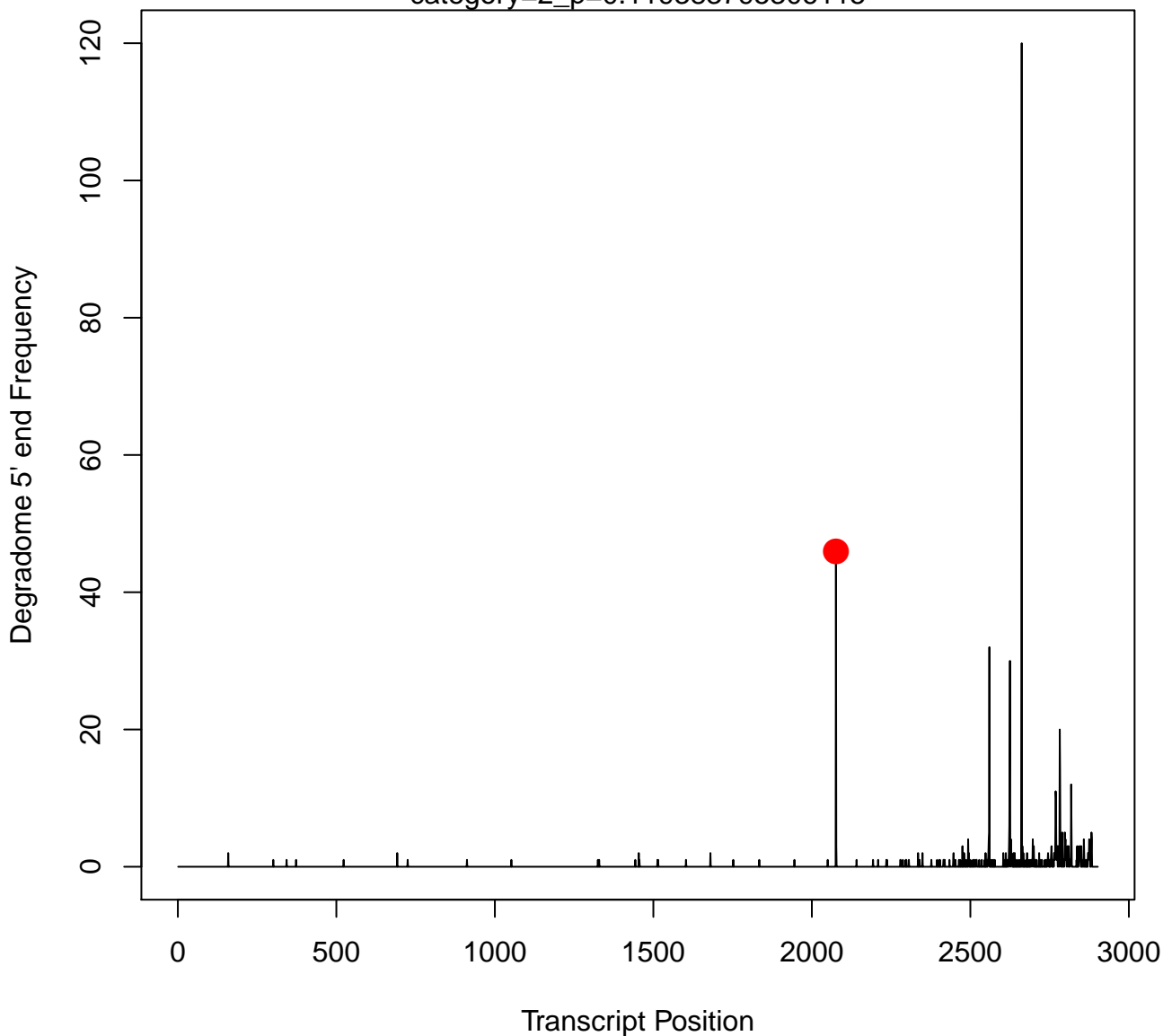

Supplement: Supplementary file 2 [file Data_Sheet_8.ZIP › GSM2230747.plot/Lsa-miR160a_Lsat_1_v5_gn_2_100181.1_2076_TPlot.pdf]

**T=Lsat\_1\_v5\_gn\_2\_112381.1\_Q=Lsa-miR160a\_S=1289**

category=2\_p=0.0909453248773598

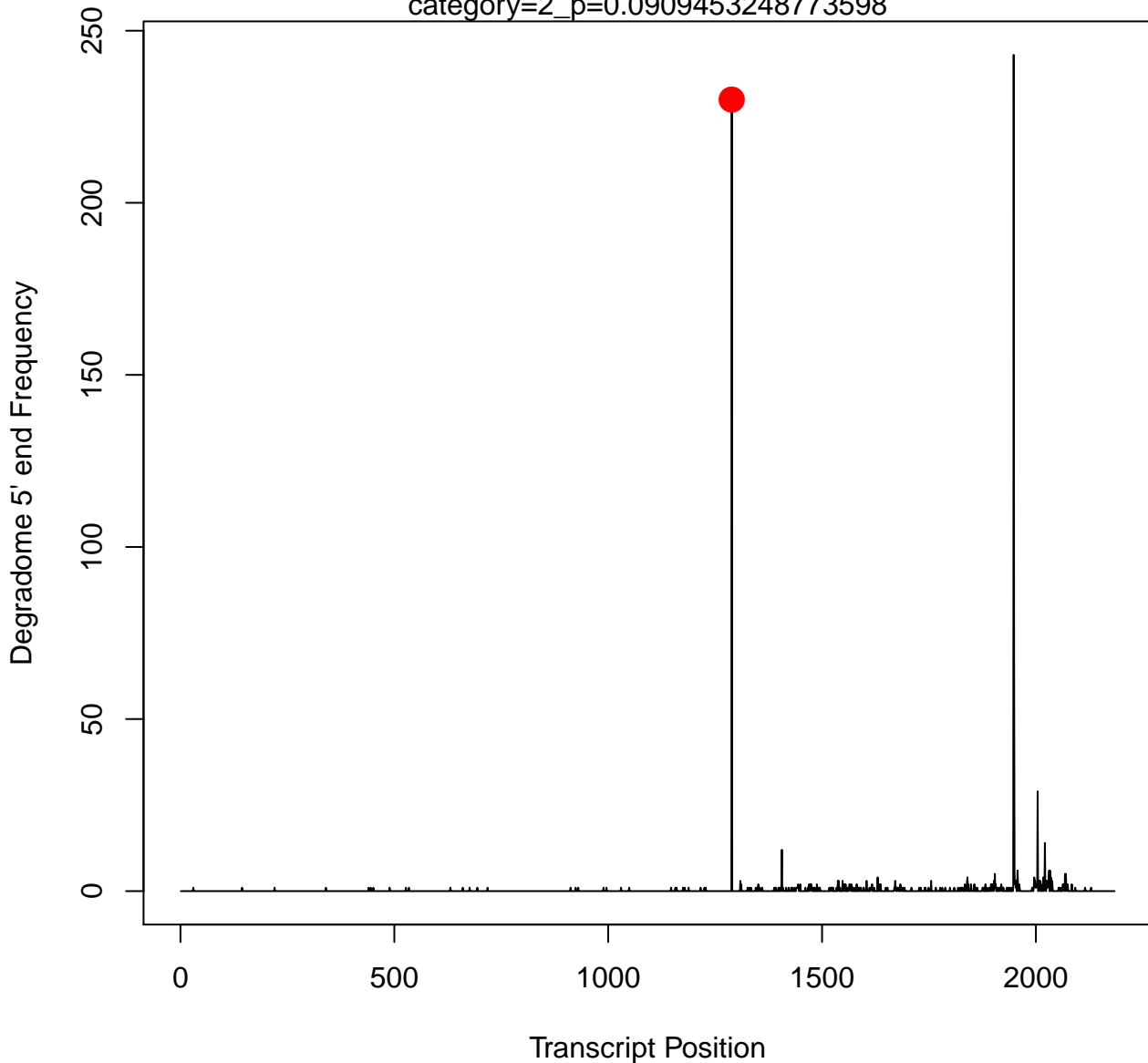

Supplement: Supplementary file 2 [file Data_Sheet_8.ZIP › GSM2230747.plot/Lsa-miR160a_Lsat_1_v5_gn_2_112381.1_1289_TPlot.pdf]

**T=Lsat\_1\_v5\_gn\_9\_69060.1\_Q=Lsa-miR160a\_S=1181**

category=0\_p=0.000737666911525325

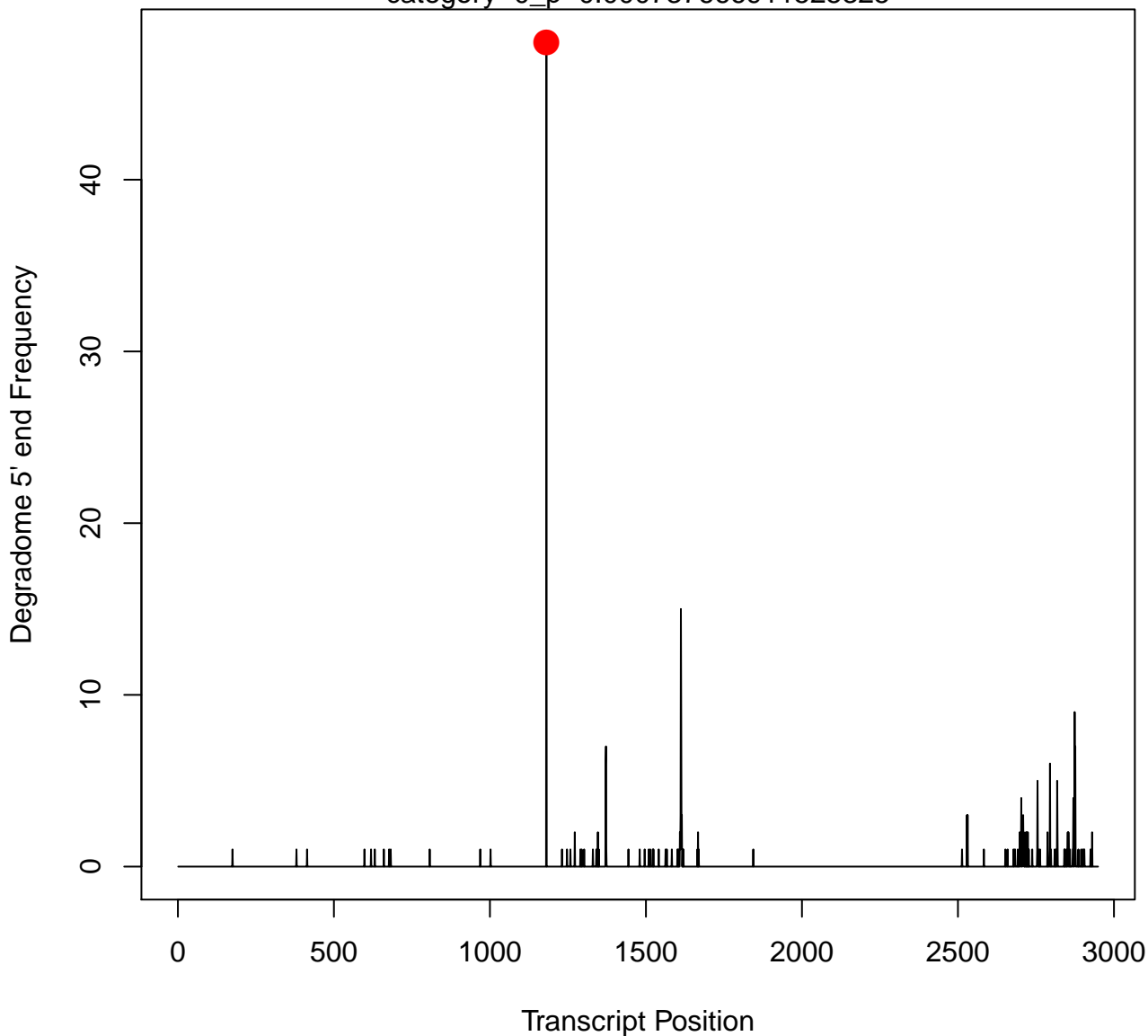

Supplement: Supplementary file 2 [file Data_Sheet_8.ZIP › GSM2230747.plot/Lsa-miR160a_Lsat_1_v5_gn_9_69060.1_1181_TPlot.pdf]

**T=Lsat\_1\_v5\_gn\_0\_37861.1\_Q=Lsa-miR160b\_S=919**

category=1\_p=0.00982189006527989

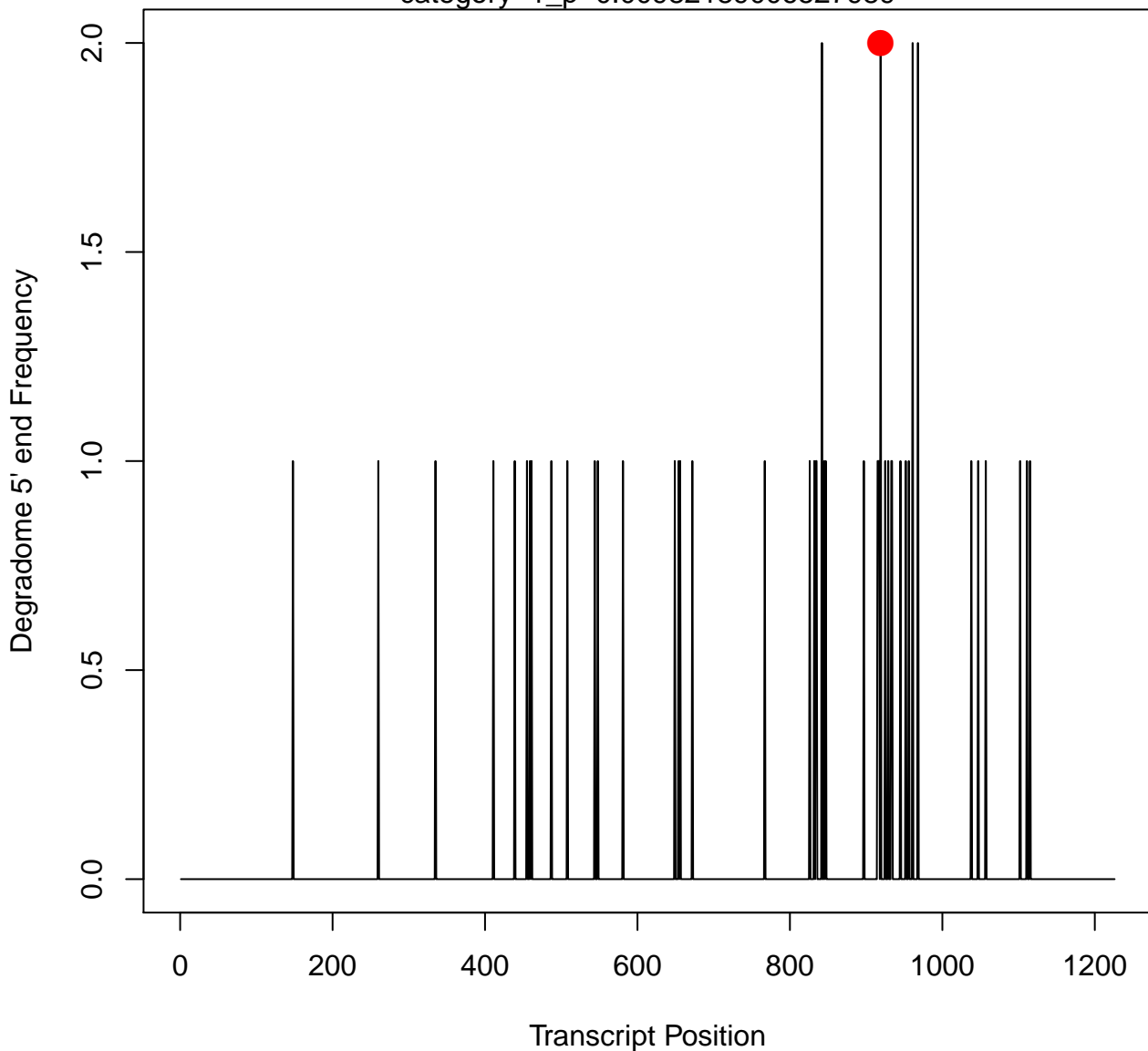

Supplement: Supplementary file 2 [file Data_Sheet_8.ZIP › GSM2230747.plot/Lsa-miR160b_Lsat_1_v5_gn_0_37861.1_919_TPlot.pdf]

**T=Lsat\_1\_v5\_gn\_5\_173401.1\_Q=Lsa-miR160b\_S=679**

category=2\_p=0.825892791610651

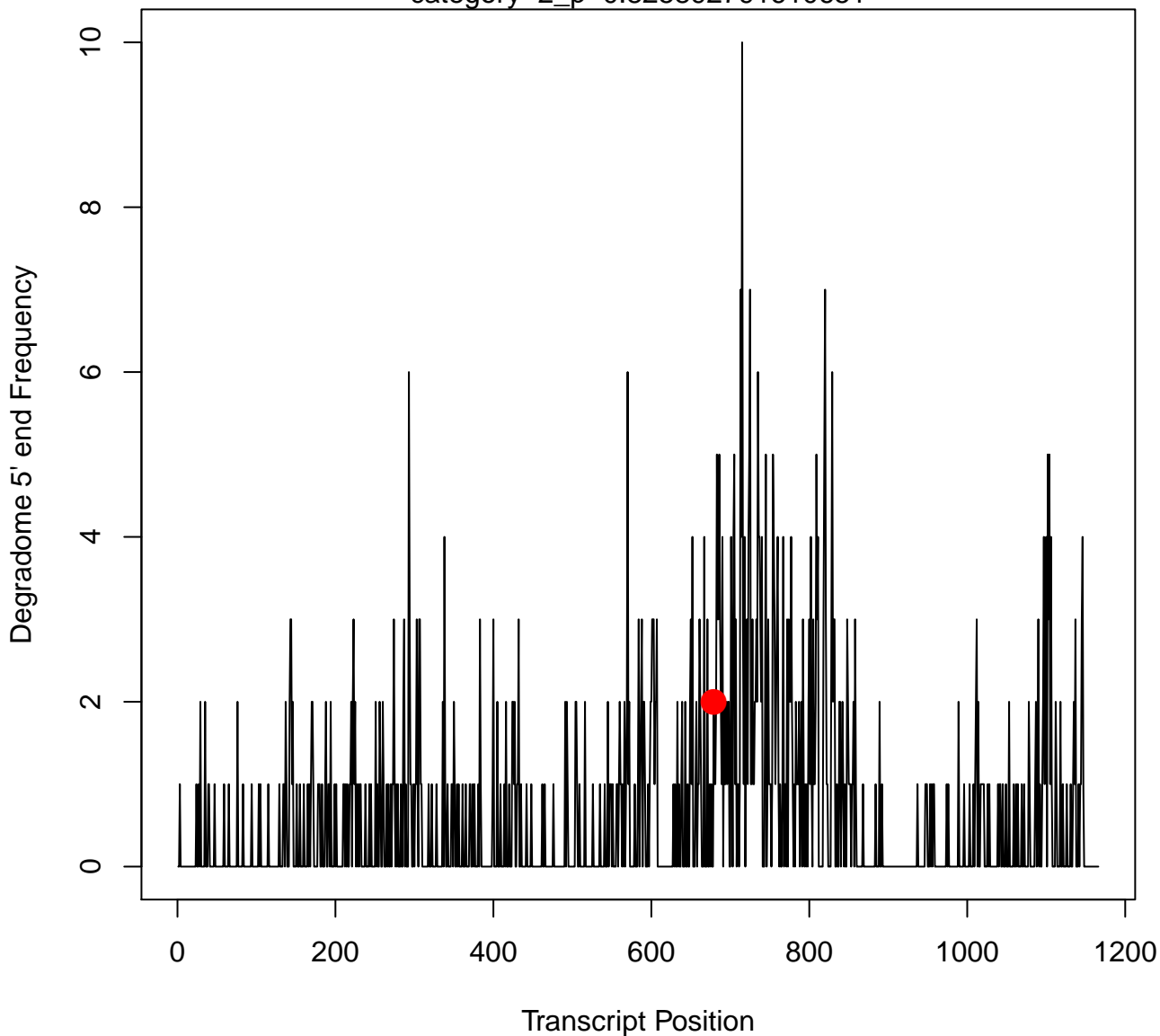

Supplement: Supplementary file 2 [file Data_Sheet_8.ZIP › GSM2230747.plot/Lsa-miR160b_Lsat_1_v5_gn_5_173401.1_679_TPlot.pdf]

**T=Lsat\_1\_v5\_gn\_6\_30741.1\_Q=Lsa-miR160b\_S=2067**

category=0\_p=0.000368901499921082

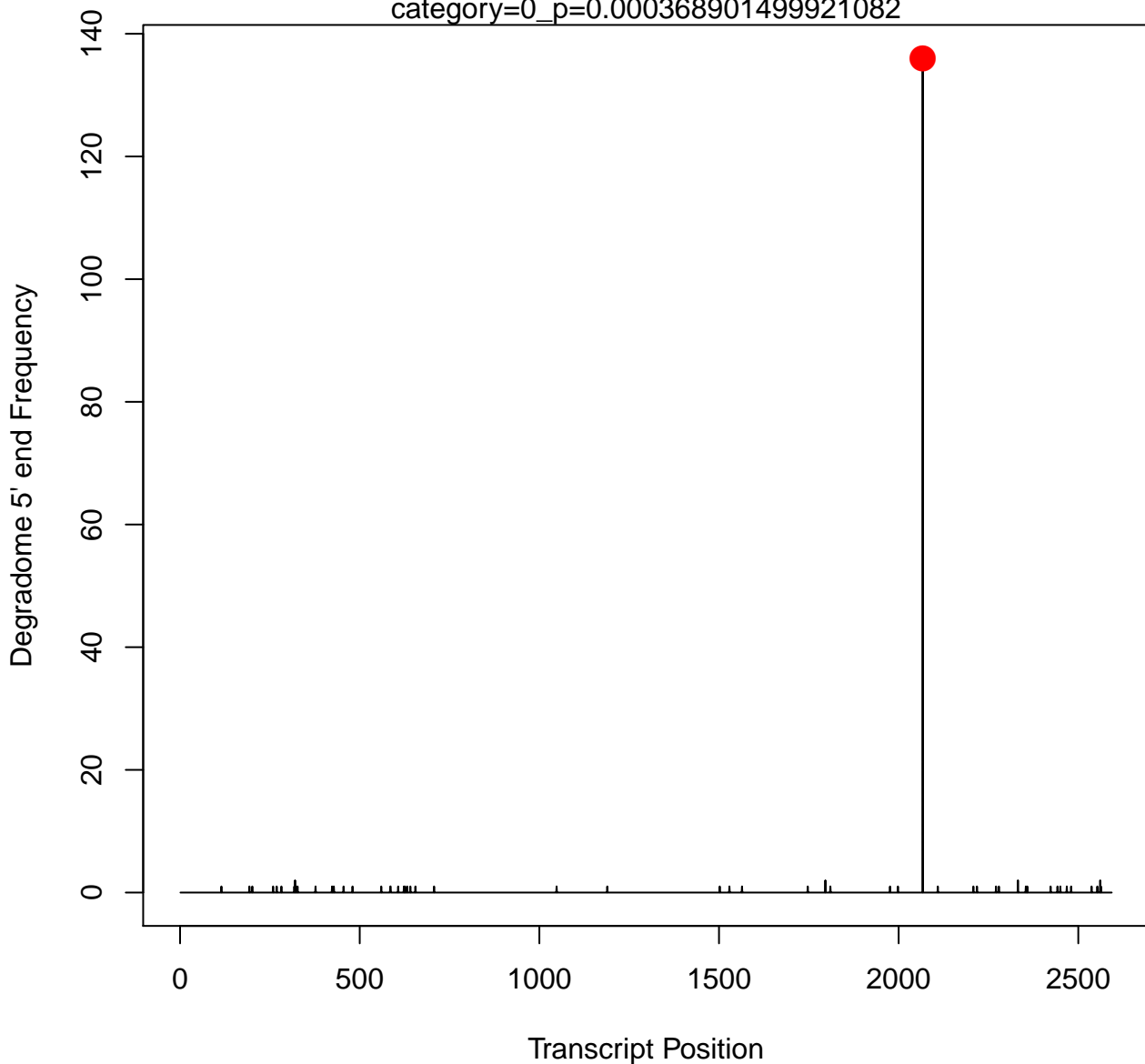

Supplement: Supplementary file 2 [file Data_Sheet_8.ZIP › GSM2230747.plot/Lsa-miR160b_Lsat_1_v5_gn_6_30741.1_2067_TPlot.pdf]

**T=Lsat\_1\_v5\_gn\_7\_80140.1\_Q=Lsa-miR160b\_S=1719**

category=2\_p=0.860622256304064

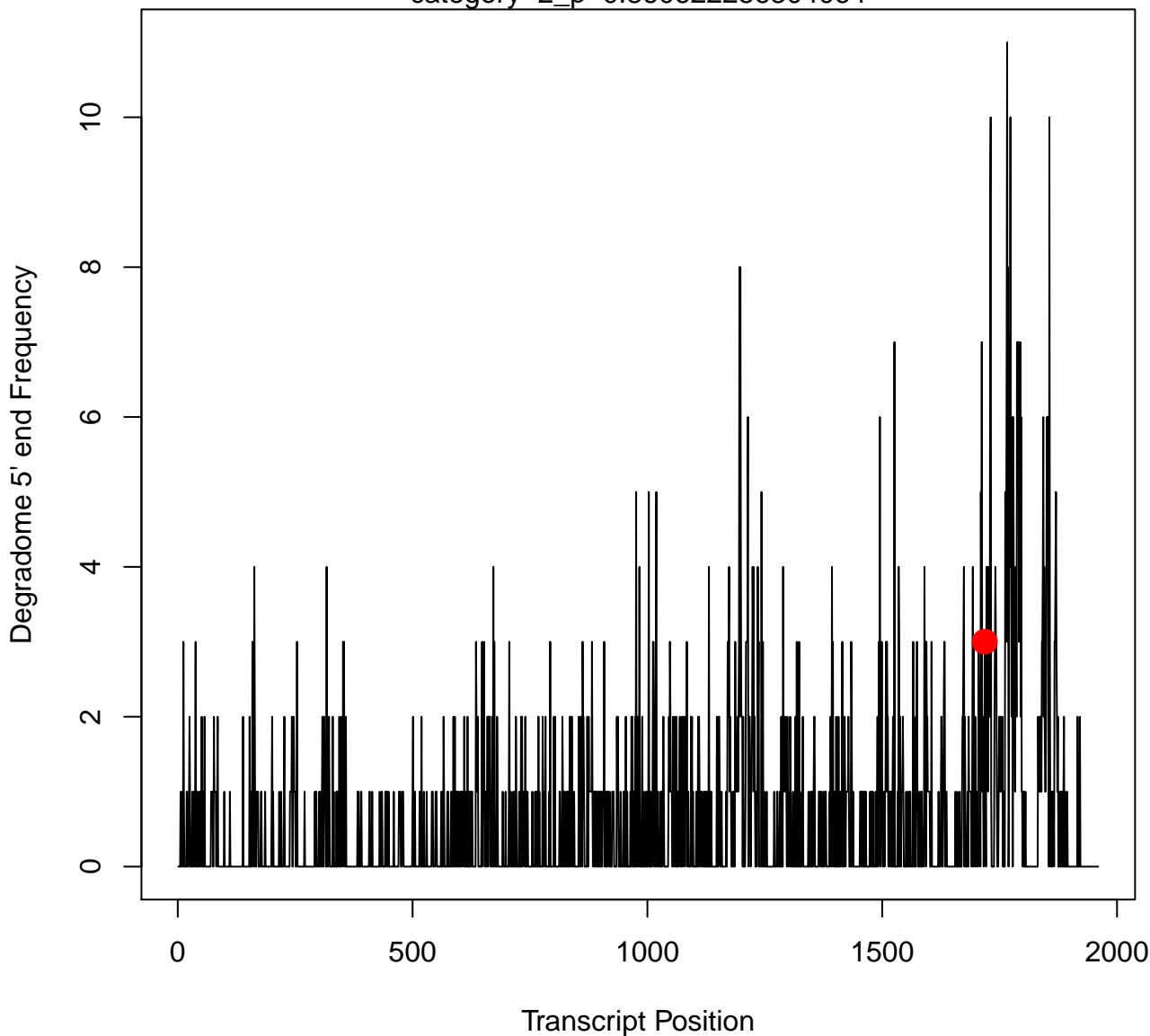

Supplement: Supplementary file 2 [file Data_Sheet_8.ZIP › GSM2230747.plot/Lsa-miR160b_Lsat_1_v5_gn_7_80140.1_1719_TPlot.pdf]

**T=Lsat\_1\_v5\_gn\_8\_20161.1\_Q=Lsa-miR160b\_S=1819**

category=2\_p=0.0909453248773598

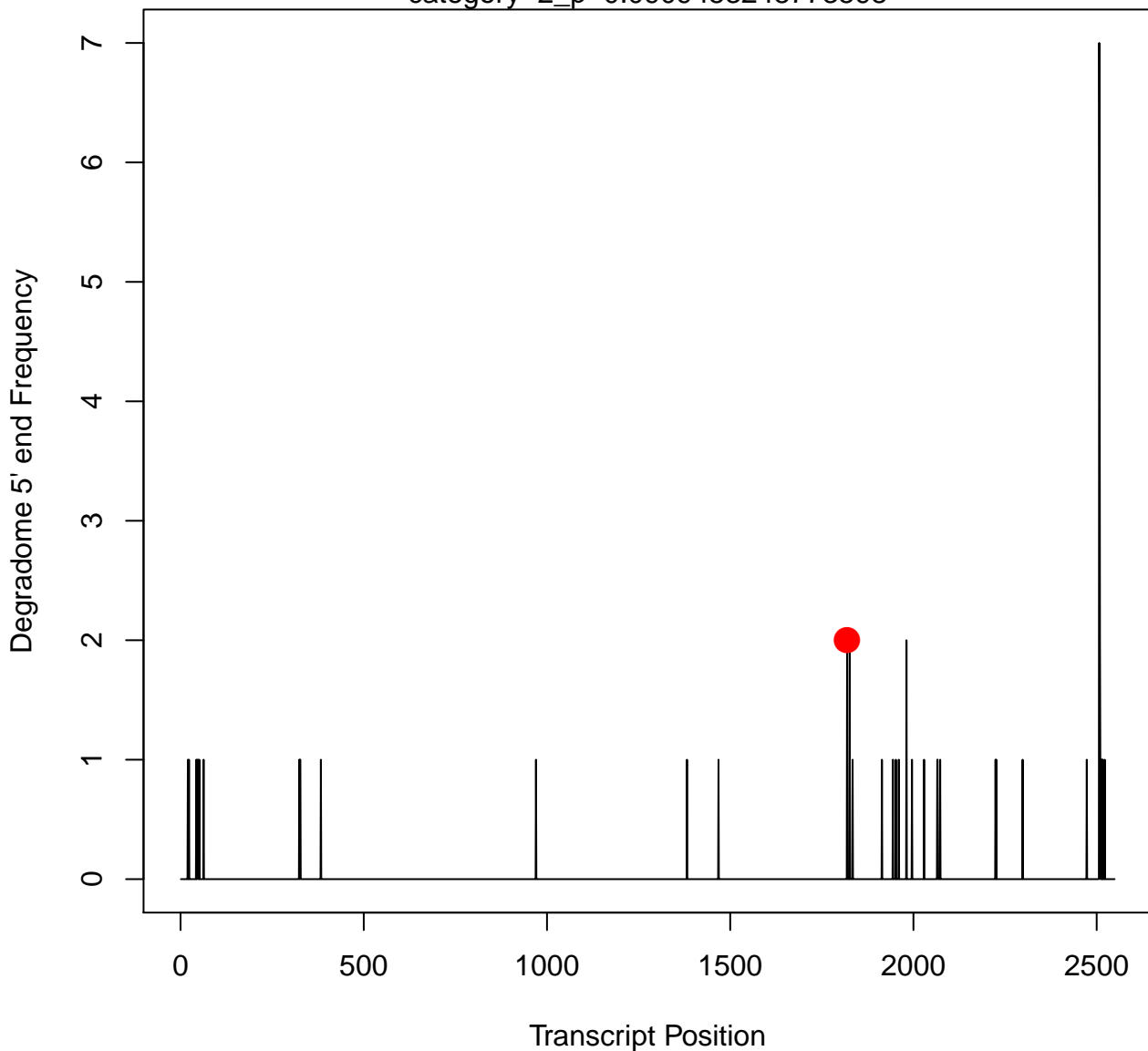

Supplement: Supplementary file 2 [file Data_Sheet_8.ZIP › GSM2230747.plot/Lsa-miR160b_Lsat_1_v5_gn_8_20161.1_1819_TPlot.pdf]

**T=Lsat\_1\_v5\_gn\_4\_109600.1\_Q=Lsa-miR164a\_S=877**

category=0\_p=0.00110629628501624

Degradome 5' end Frequency

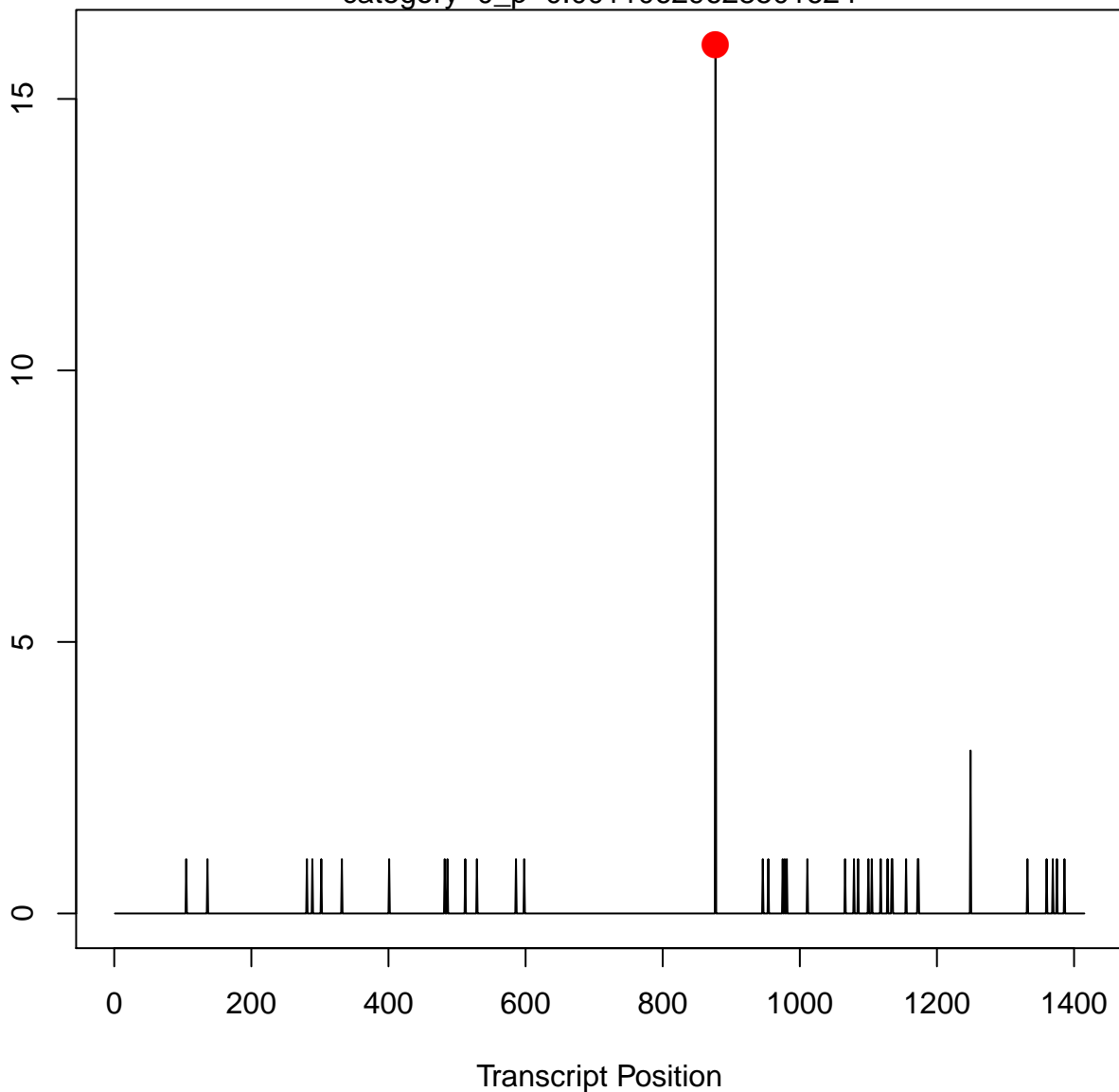

Supplement: Supplementary file 2 [file Data_Sheet_8.ZIP › GSM2230747.plot/Lsa-miR164a_Lsat_1_v5_gn_4_109600.1_877_TPlot.pdf]

**T=Lsat\_1\_v5\_gn\_5\_127660.1\_Q=Lsa-miR164a\_S=724**

category=0\_p=0.000737666911525325

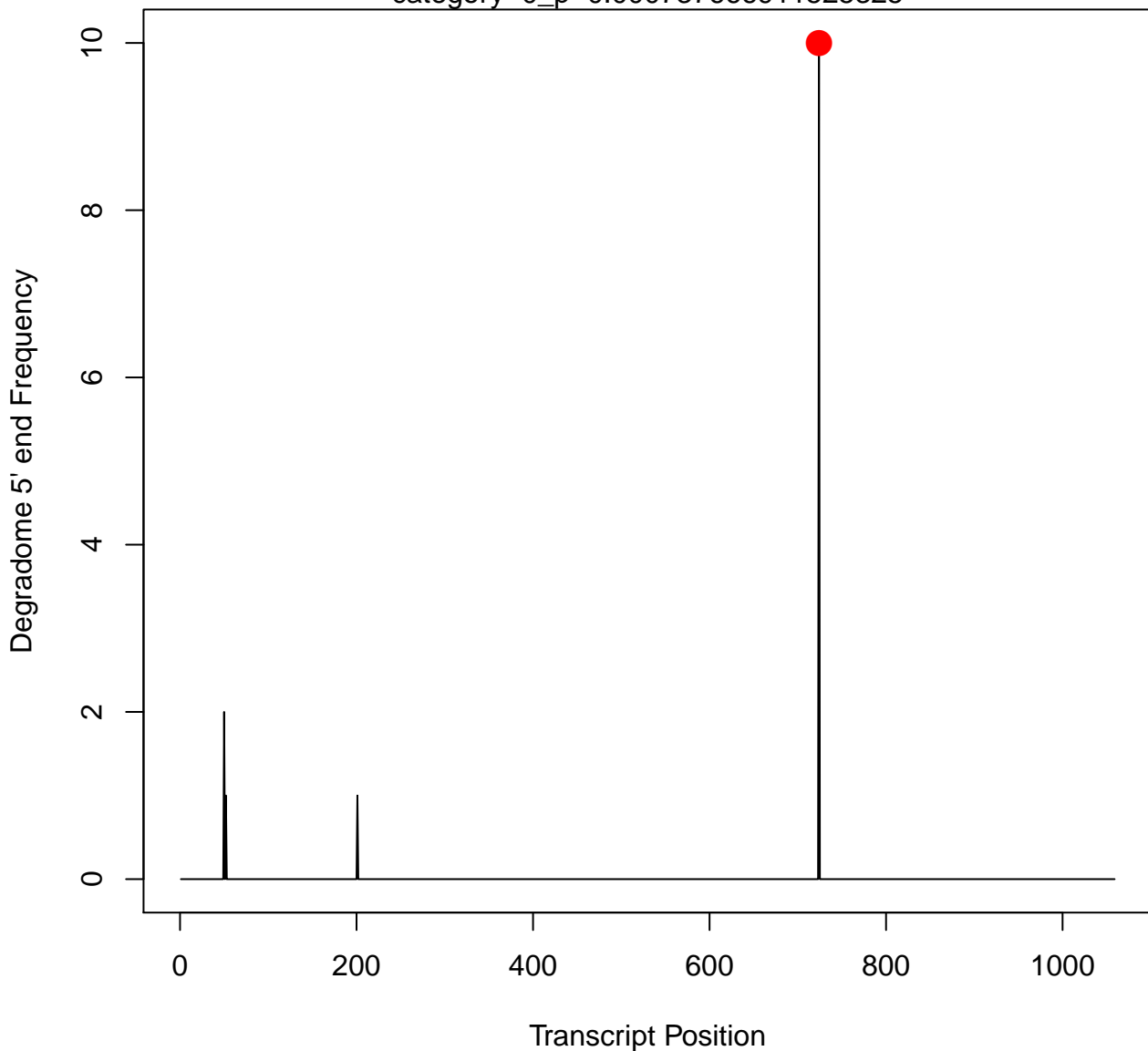

Supplement: Supplementary file 2 [file Data_Sheet_8.ZIP › GSM2230747.plot/Lsa-miR164a_Lsat_1_v5_gn_5_127660.1_724_TPlot.pdf]
