# Supplementary figures and images for: Comprehensive Annotation and Functional Exploration of MicroRNAs in Lettuce (part 4 of 6)
Source: Front Plant Sci. 2021 Dec 24;12:781836. doi: 10.3389/fpls.2021.781836 (PMC8739914; doi:10.3389/fpls.2021.781836)

**T=Lsat\_1\_v5\_gn\_5\_184280.1\_Q=Lsa-miR166g\_S=912**

category=2\_p=0.234454518831775

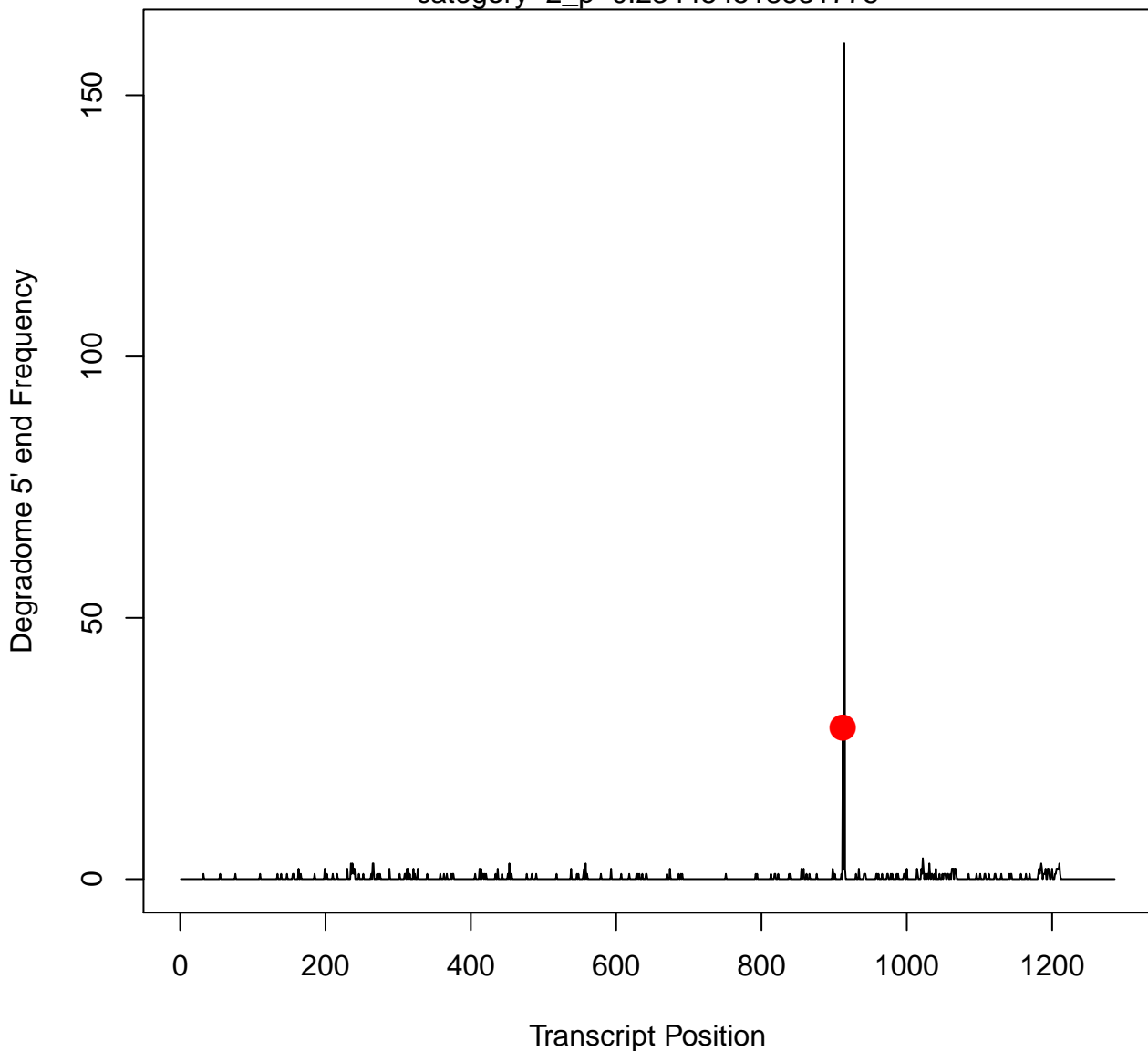

Supplement: Supplementary file 3 [file Data_Sheet_9.ZIP › GSM2230751.plot/Lsa-miR166g_Lsat_1_v5_gn_5_184280.1_912_TPlot.pdf]

**T=Lsat\_1\_v5\_gn\_5\_32500.1\_Q=Lsa-miR166g\_S=565**

category=0\_p=0.00147735698913964

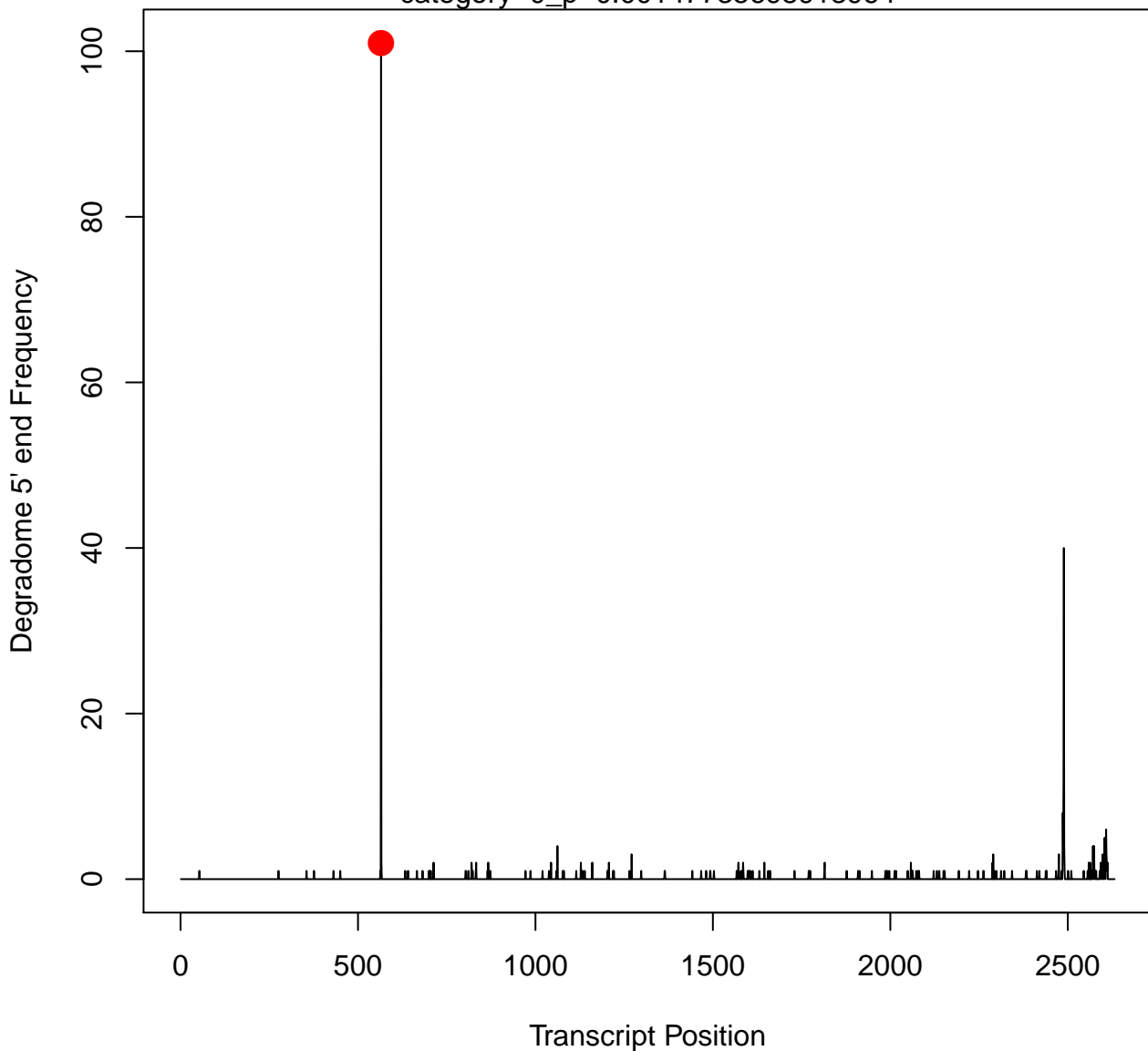

Supplement: Supplementary file 3 [file Data_Sheet_9.ZIP › GSM2230751.plot/Lsa-miR166g_Lsat_1_v5_gn_5_32500.1_565_TPlot.pdf]

**T=Lsat\_1\_v5\_gn\_5\_44541.1\_Q=Lsa-miR166g\_S=515**

category=2\_p=0.938603795024377

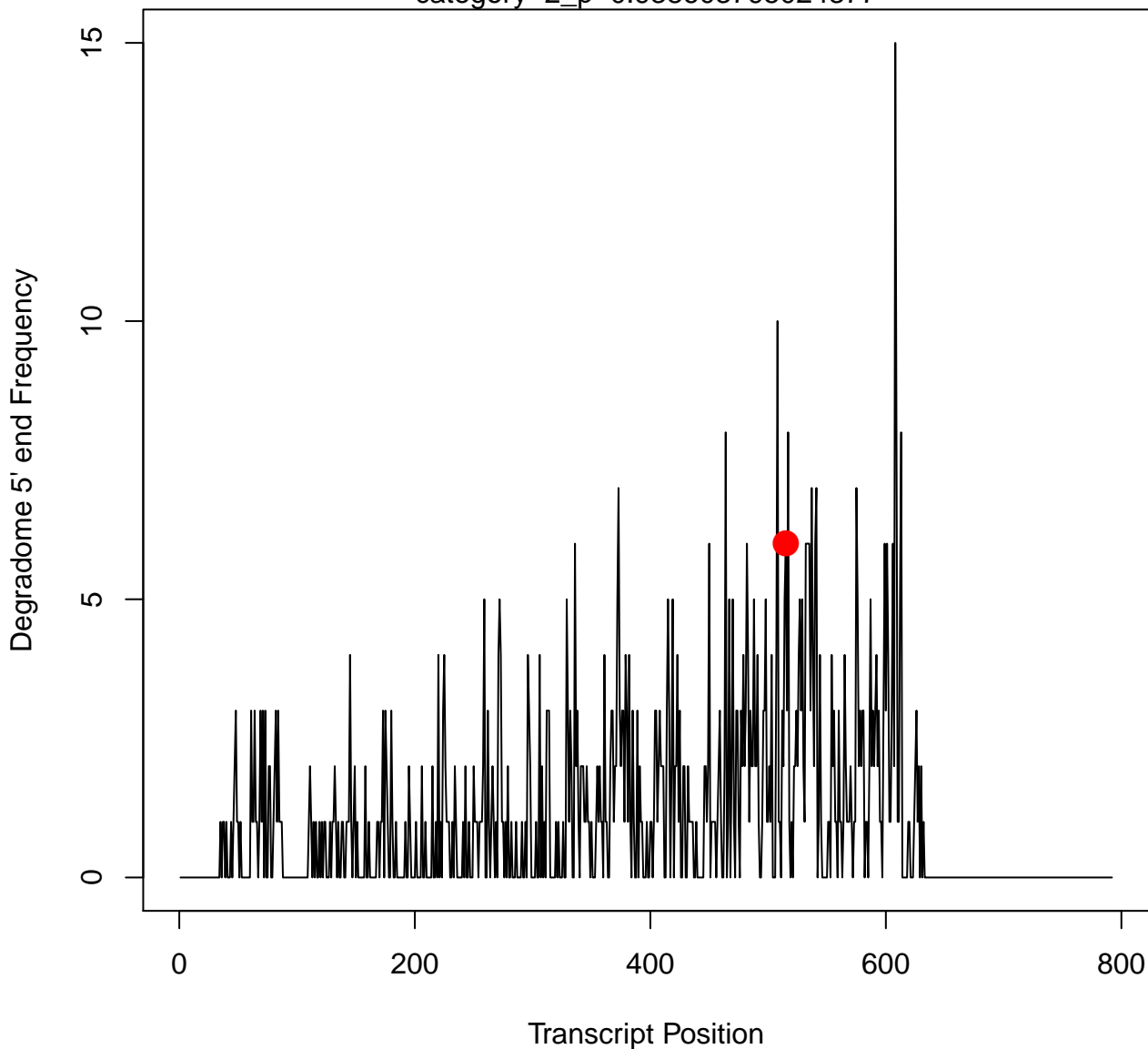

Supplement: Supplementary file 3 [file Data_Sheet_9.ZIP › GSM2230751.plot/Lsa-miR166g_Lsat_1_v5_gn_5_44541.1_515_TPlot.pdf]

**T=Lsat\_1\_v5\_gn\_7\_29400.1\_Q=Lsa-miR166g\_S=5324**

category=2\_p=0.359353690302475

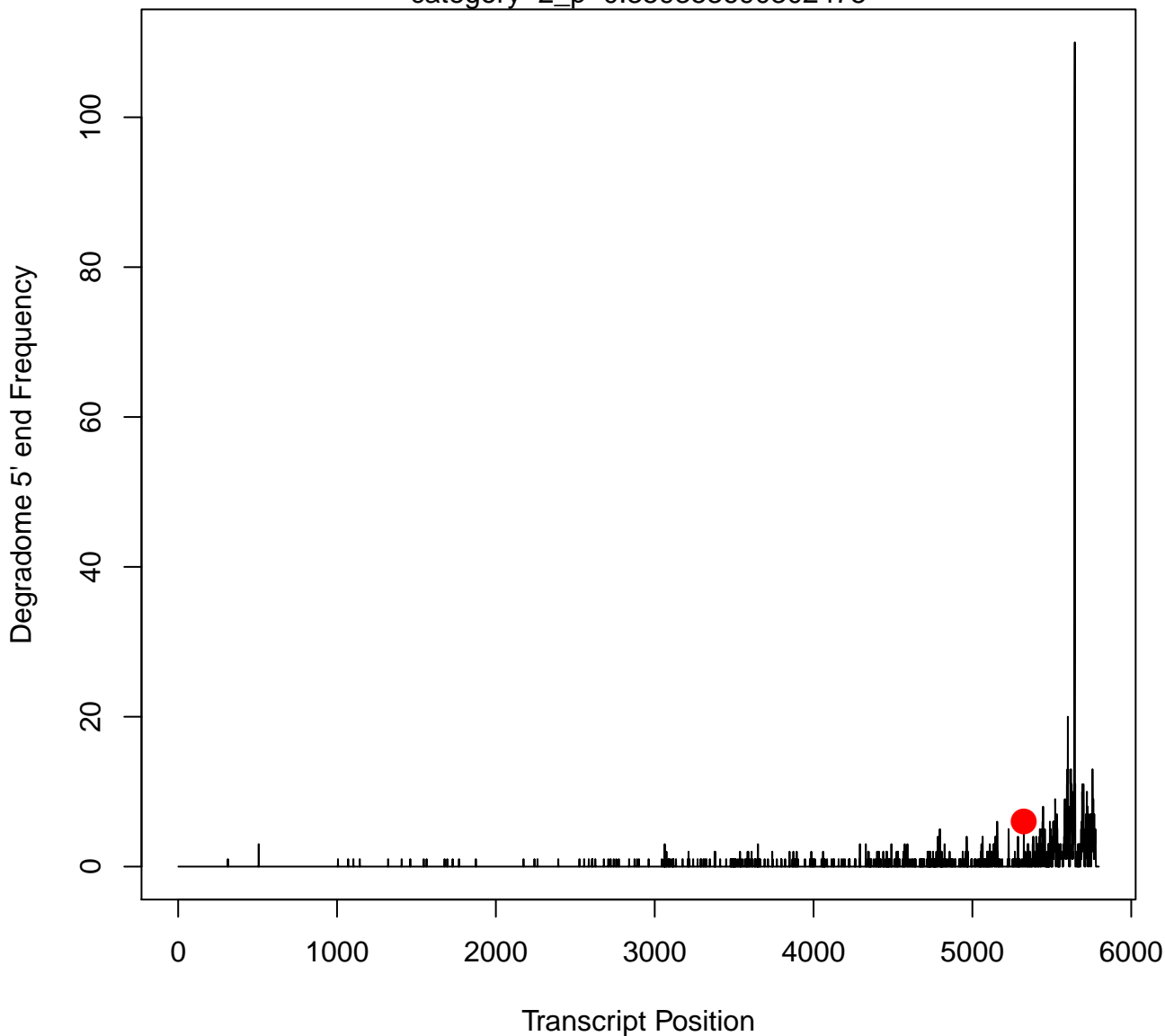

Supplement: Supplementary file 3 [file Data_Sheet_9.ZIP › GSM2230751.plot/Lsa-miR166g_Lsat_1_v5_gn_7_29400.1_5324_TPlot.pdf]

**T=Lsat\_1\_v5\_gn\_9\_89140.1\_Q=Lsa-miR166g\_S=1993**

category=2\_p=0.965070577083954

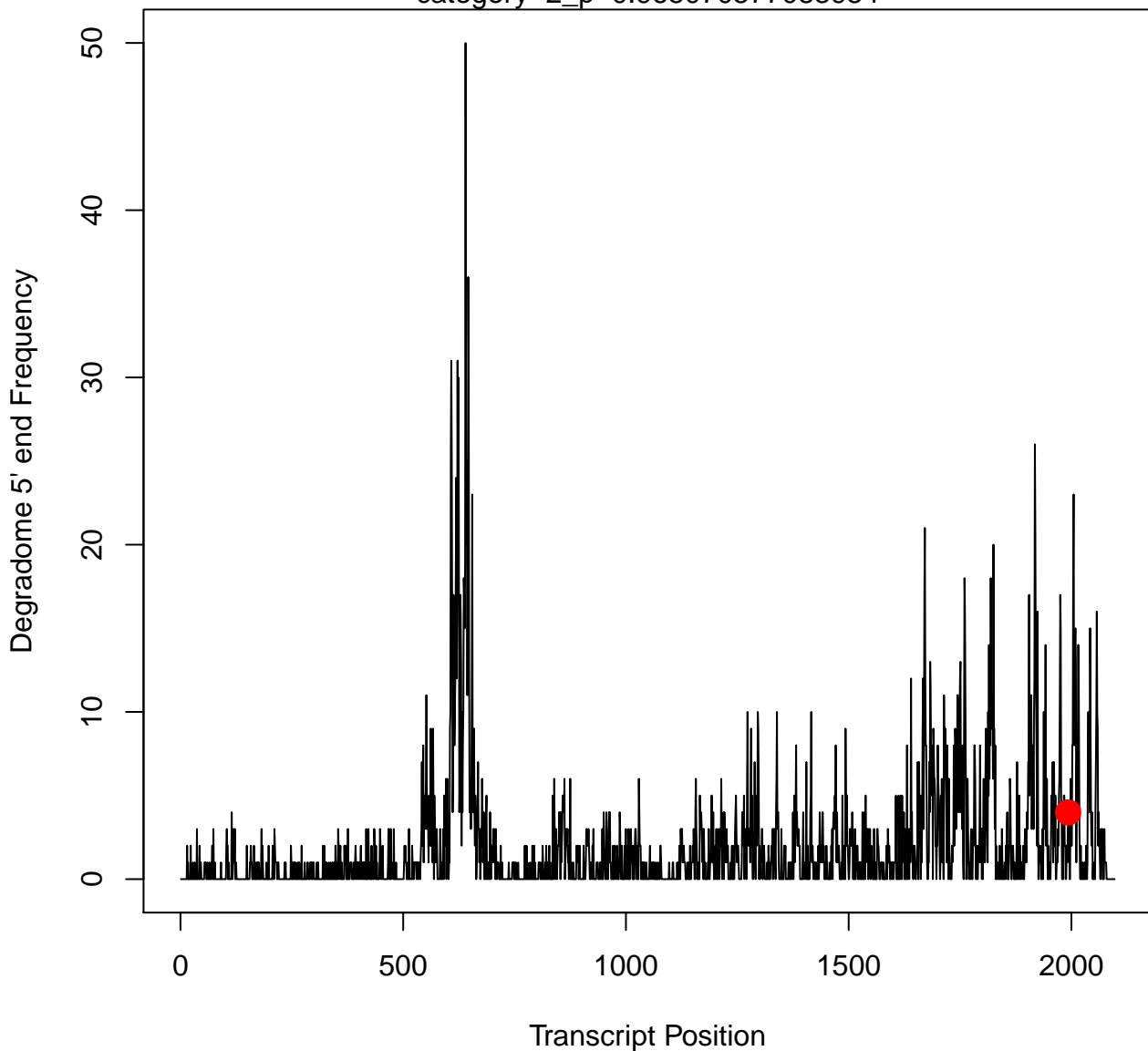

Supplement: Supplementary file 3 [file Data_Sheet_9.ZIP › GSM2230751.plot/Lsa-miR166g_Lsat_1_v5_gn_9_89140.1_1993_TPlot.pdf]

**T=Lsat\_1\_v5\_gn\_1\_37081.1\_Q=Lsa-miR166h\_S=1030**

category=0\_p=0.000369544041020964

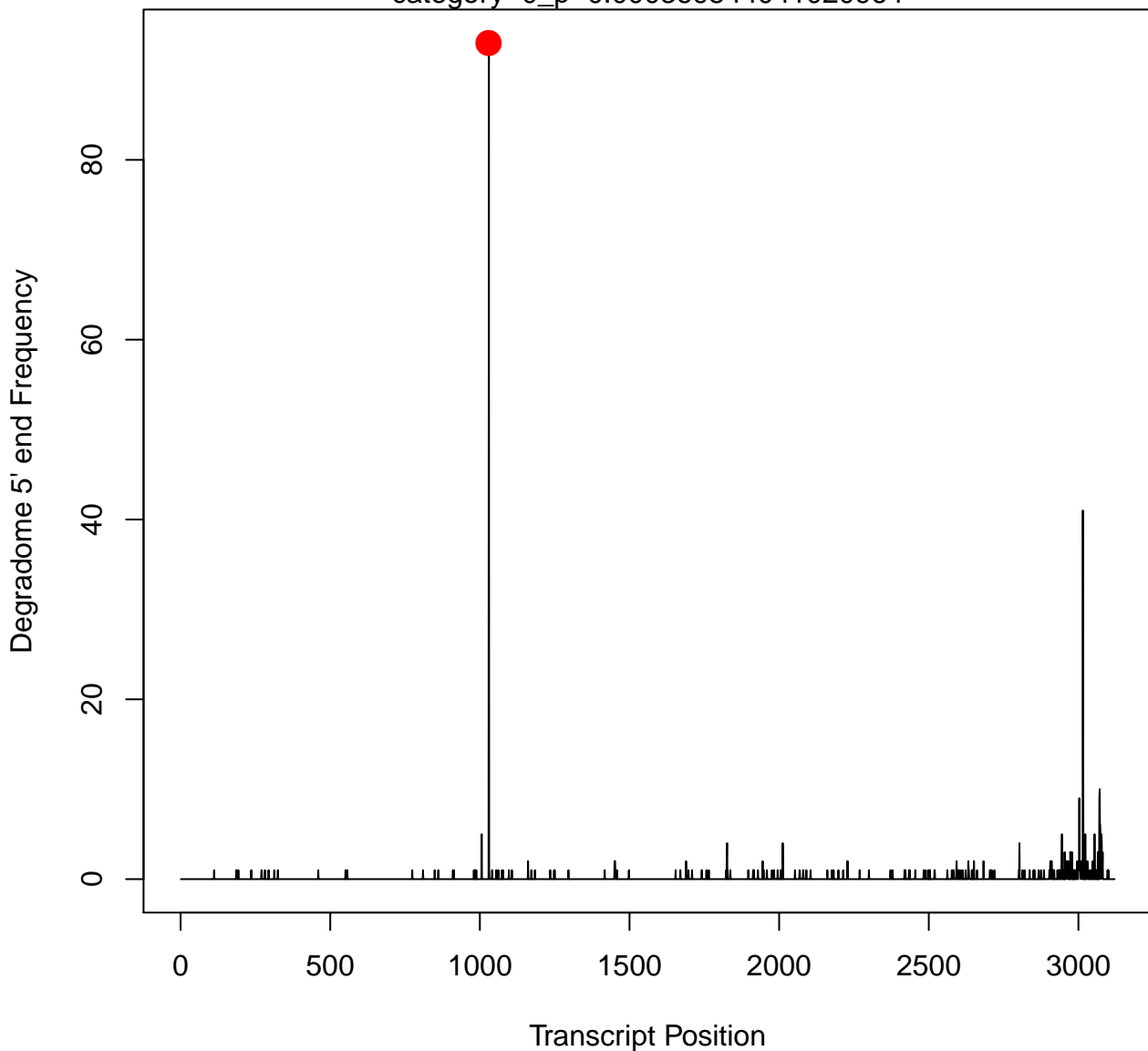

Supplement: Supplementary file 3 [file Data_Sheet_9.ZIP › GSM2230751.plot/Lsa-miR166h_Lsat_1_v5_gn_1_37081.1_1030_TPlot.pdf]

**T=Lsat\_1\_v5\_gn\_4\_100080.1\_Q=Lsa-miR166h\_S=1067**

category=0\_p=0.000738951519243747

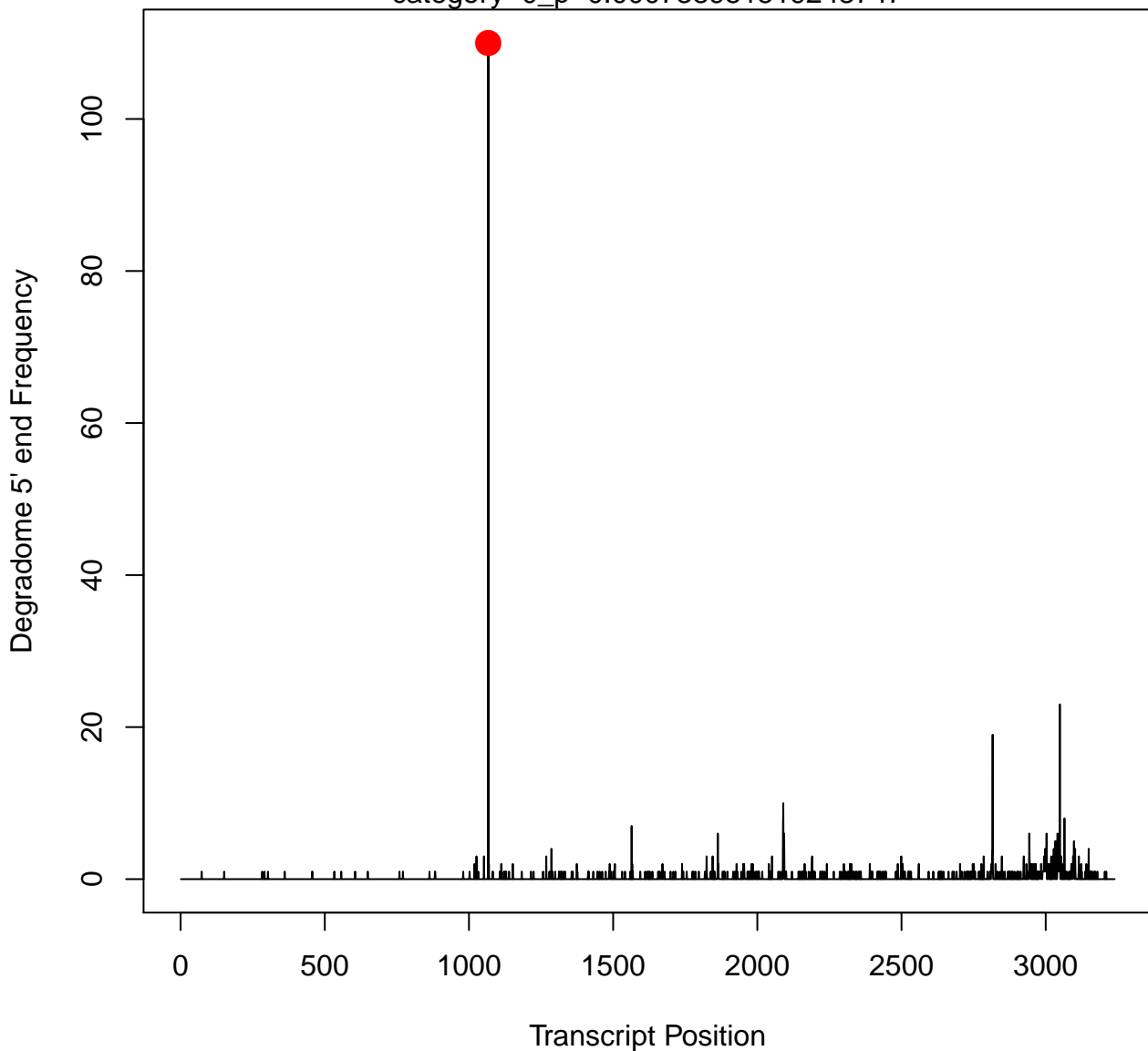

Supplement: Supplementary file 3 [file Data_Sheet_9.ZIP › GSM2230751.plot/Lsa-miR166h_Lsat_1_v5_gn_4_100080.1_1067_TPlot.pdf]

**T=Lsat\_1\_v5\_gn\_5\_84680.1\_Q=Lsa-miR166h\_S=2194**

category=2\_p=0.974042055686745

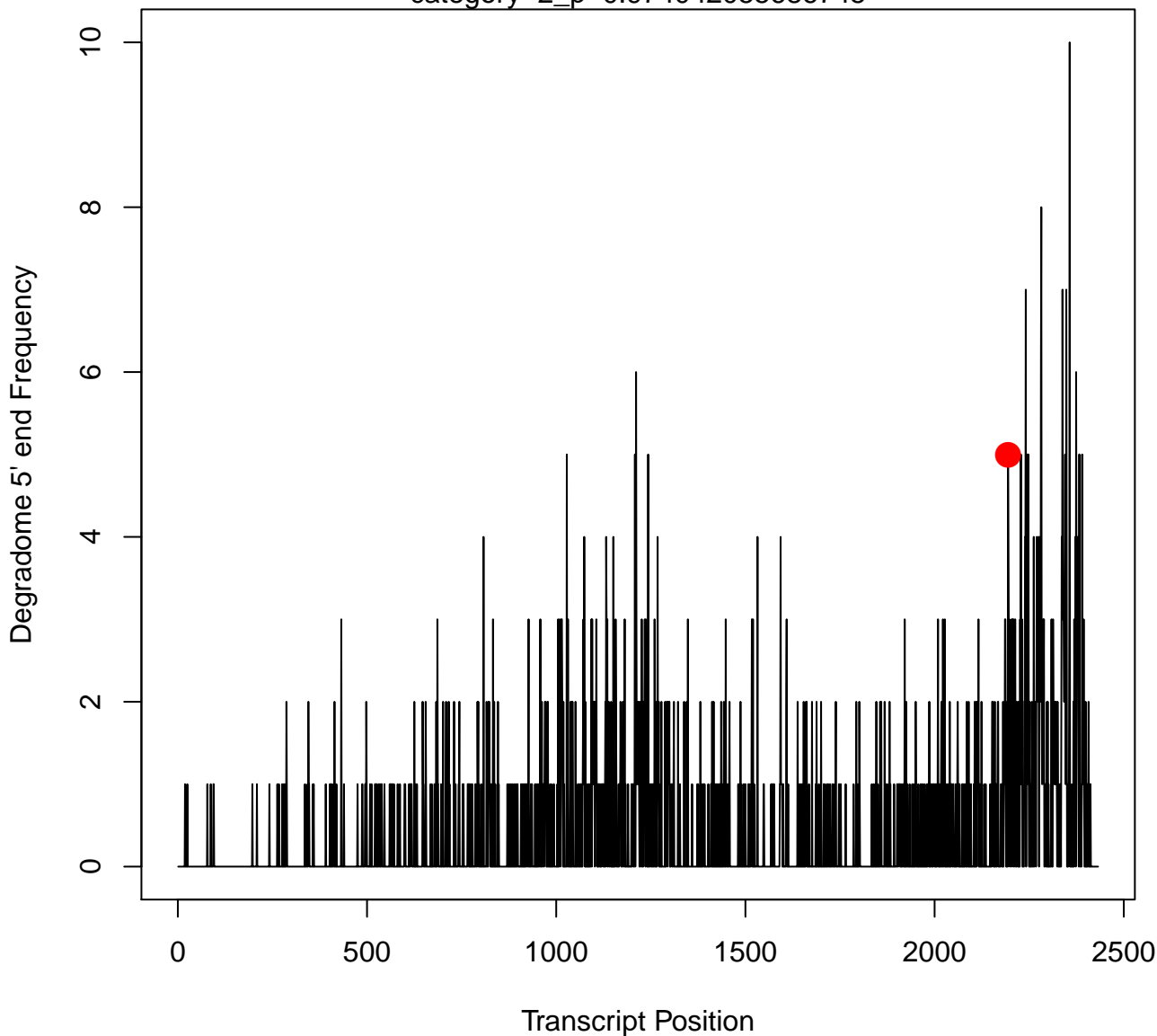

Supplement: Supplementary file 3 [file Data_Sheet_9.ZIP › GSM2230751.plot/Lsa-miR166h_Lsat_1_v5_gn_5_84680.1_2194_TPlot.pdf]

**T=Lsat\_1\_v5\_gn\_8\_60221.1\_Q=Lsa-miR166h\_S=2203**

category=2\_p=0.957003194493134

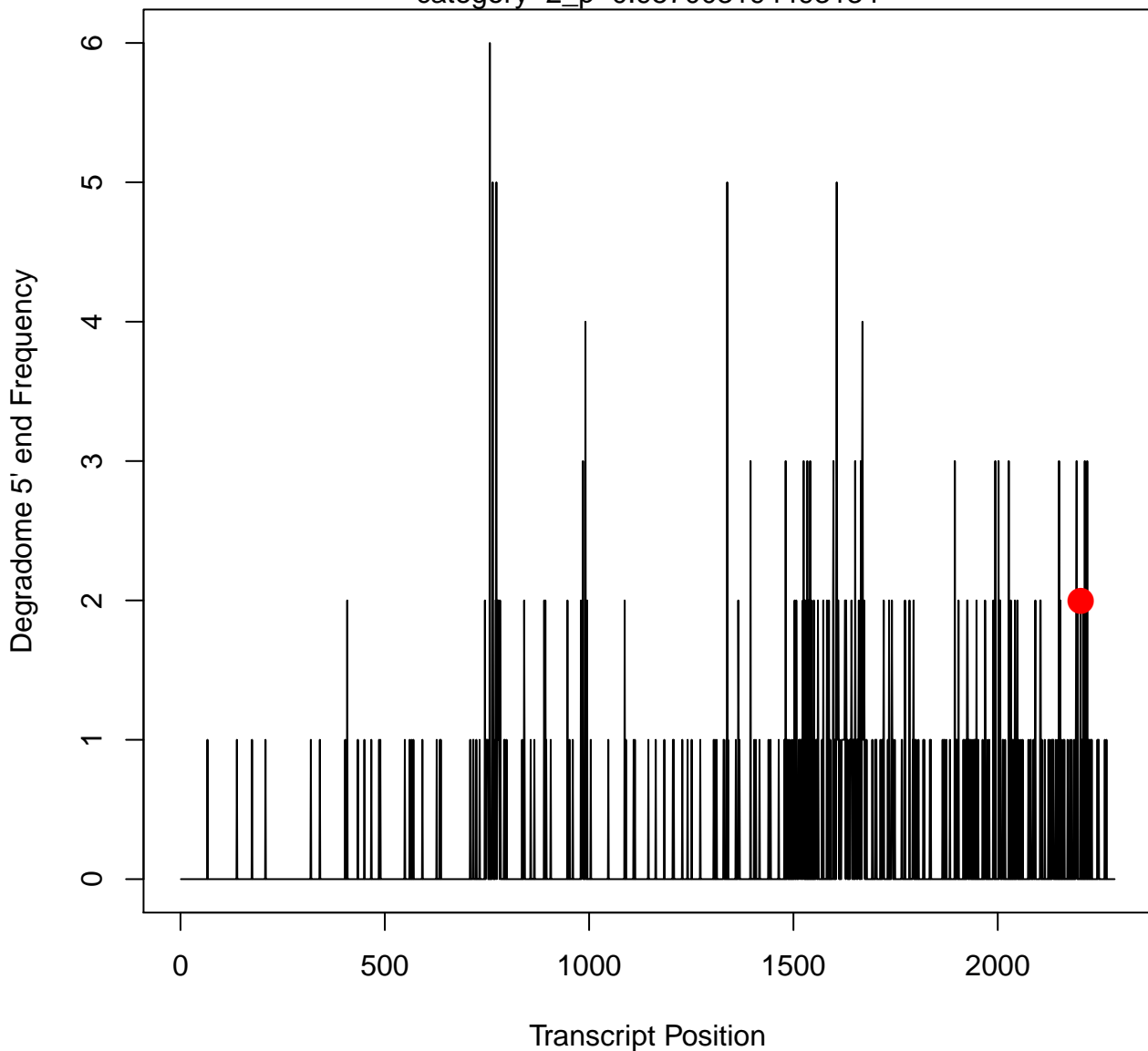

Supplement: Supplementary file 3 [file Data_Sheet_9.ZIP › GSM2230751.plot/Lsa-miR166h_Lsat_1_v5_gn_8_60221.1_2203_TPlot.pdf]

**T=Lsat\_1\_v5\_gn\_9\_89121.1\_Q=Lsa-miR166h\_S=1993**

category=2\_p=0.964018147250088

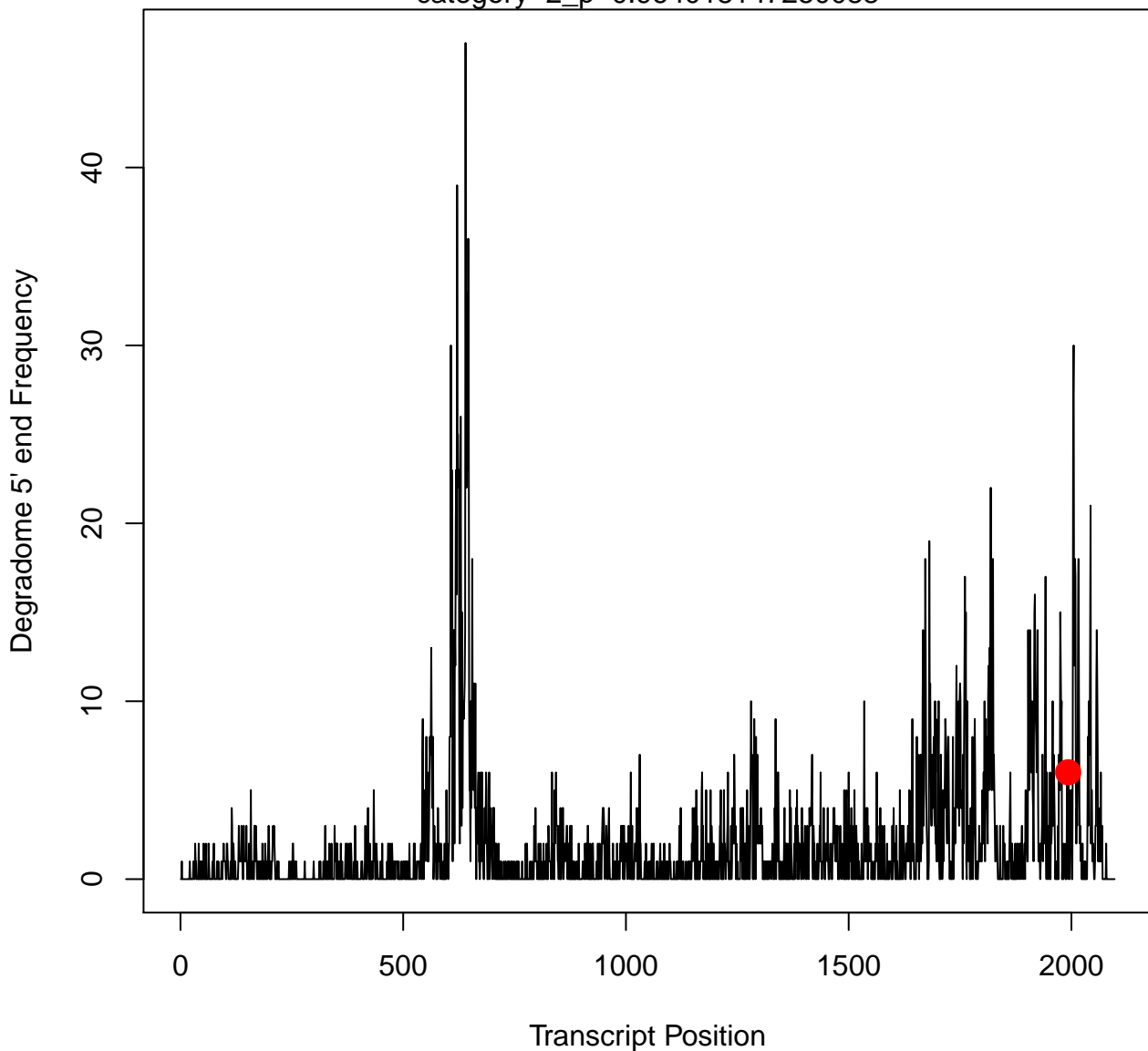

Supplement: Supplementary file 3 [file Data_Sheet_9.ZIP › GSM2230751.plot/Lsa-miR166h_Lsat_1_v5_gn_9_89121.1_1993_TPlot.pdf]

**T=Lsat\_1\_v5\_gn\_2\_100140.1\_Q=Lsa-miR166i\_S=1402**

category=2\_p=0.163150556750859

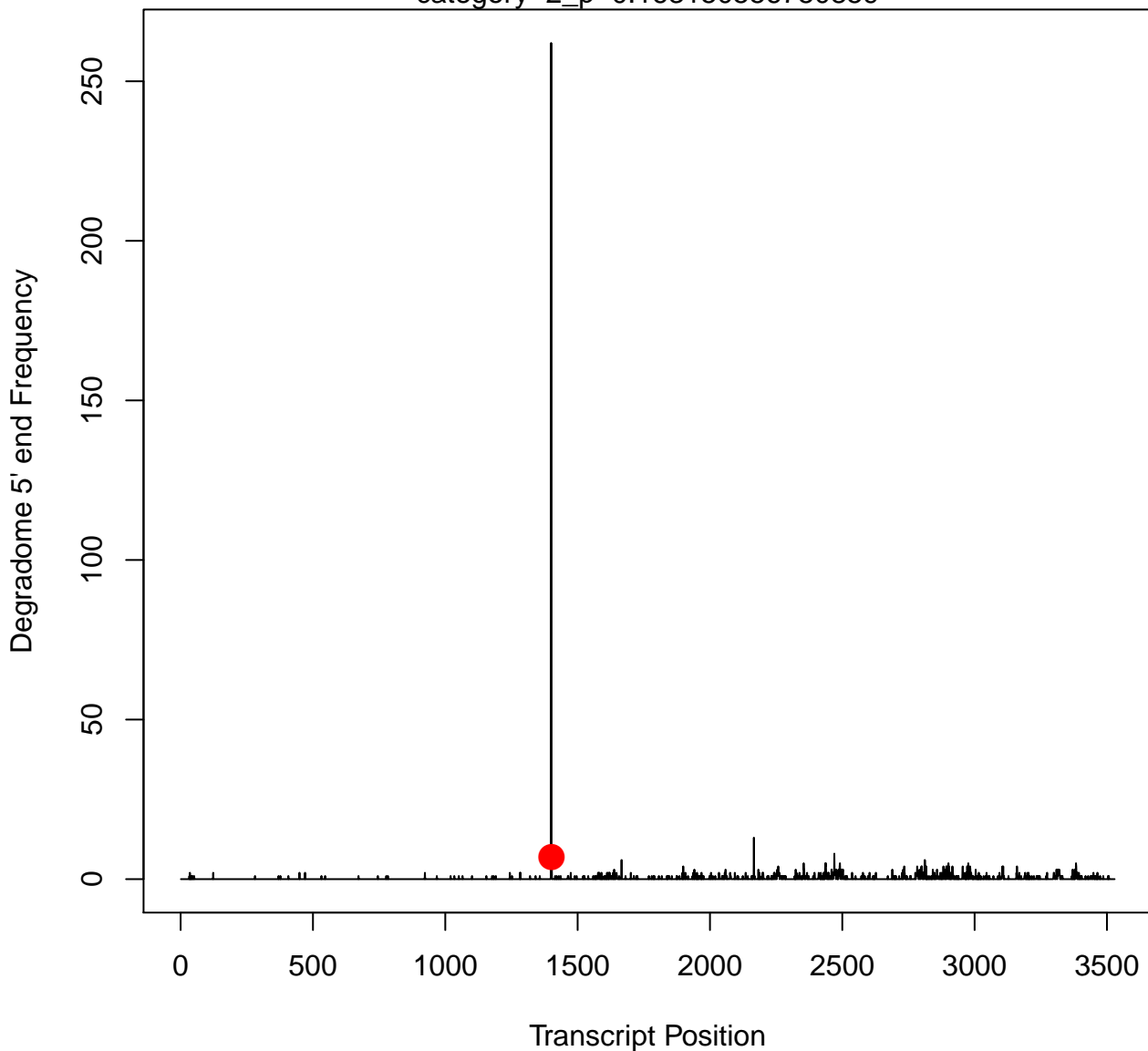

Supplement: Supplementary file 3 [file Data_Sheet_9.ZIP › GSM2230751.plot/Lsa-miR166i_Lsat_1_v5_gn_2_100140.1_1402_TPlot.pdf]

**T=Lsat\_1\_v5\_gn\_4\_100080.1\_Q=Lsa-miR166i\_S=1069**

category=2\_p=0.0852052452876977

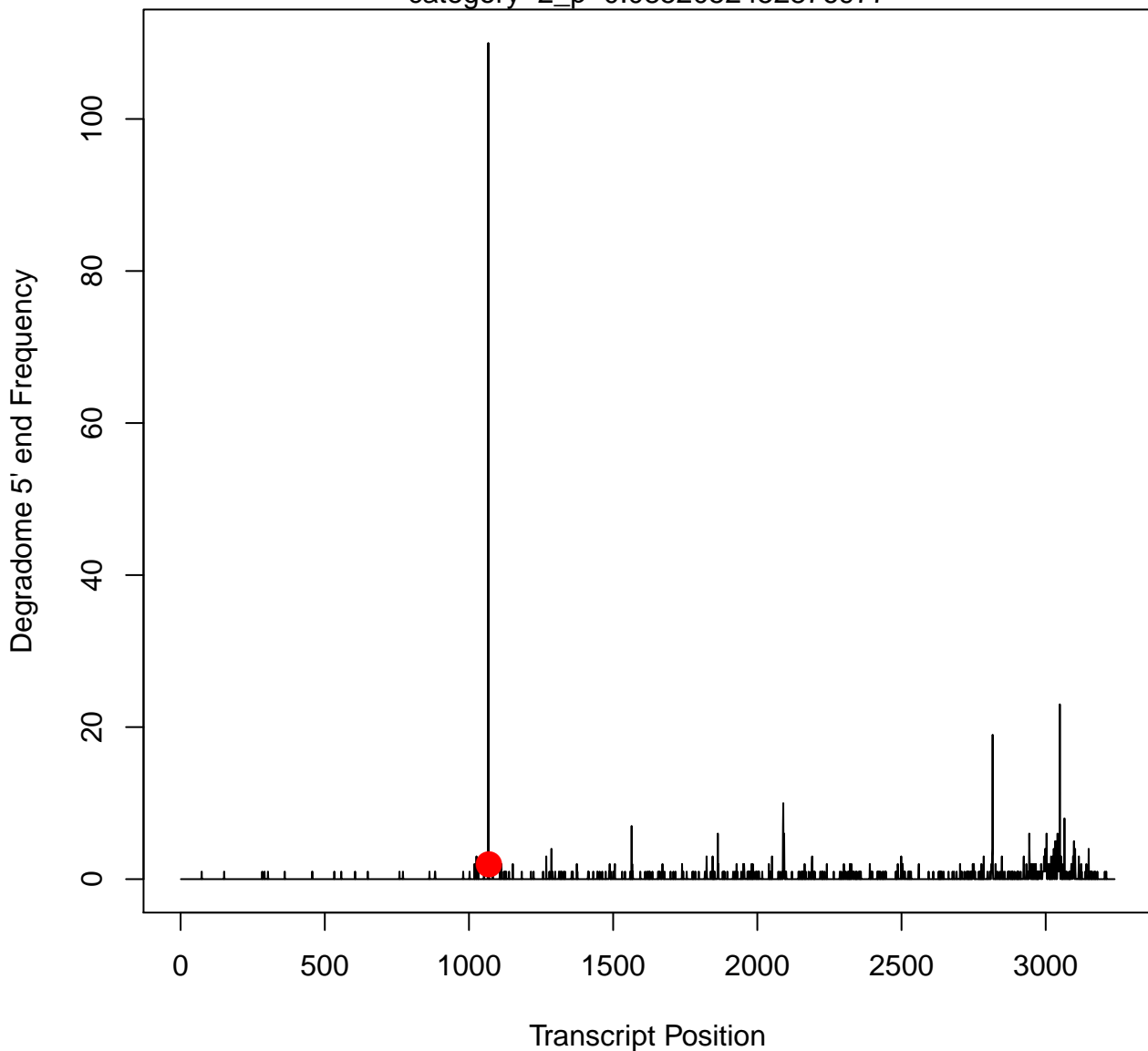

Supplement: Supplementary file 3 [file Data_Sheet_9.ZIP › GSM2230751.plot/Lsa-miR166i_Lsat_1_v5_gn_4_100080.1_1069_TPlot.pdf]

**T=Lsat\_1\_v5\_gn\_5\_134441.1\_Q=Lsa-miR166i\_S=570**

category=2\_p=0.0576423137718276

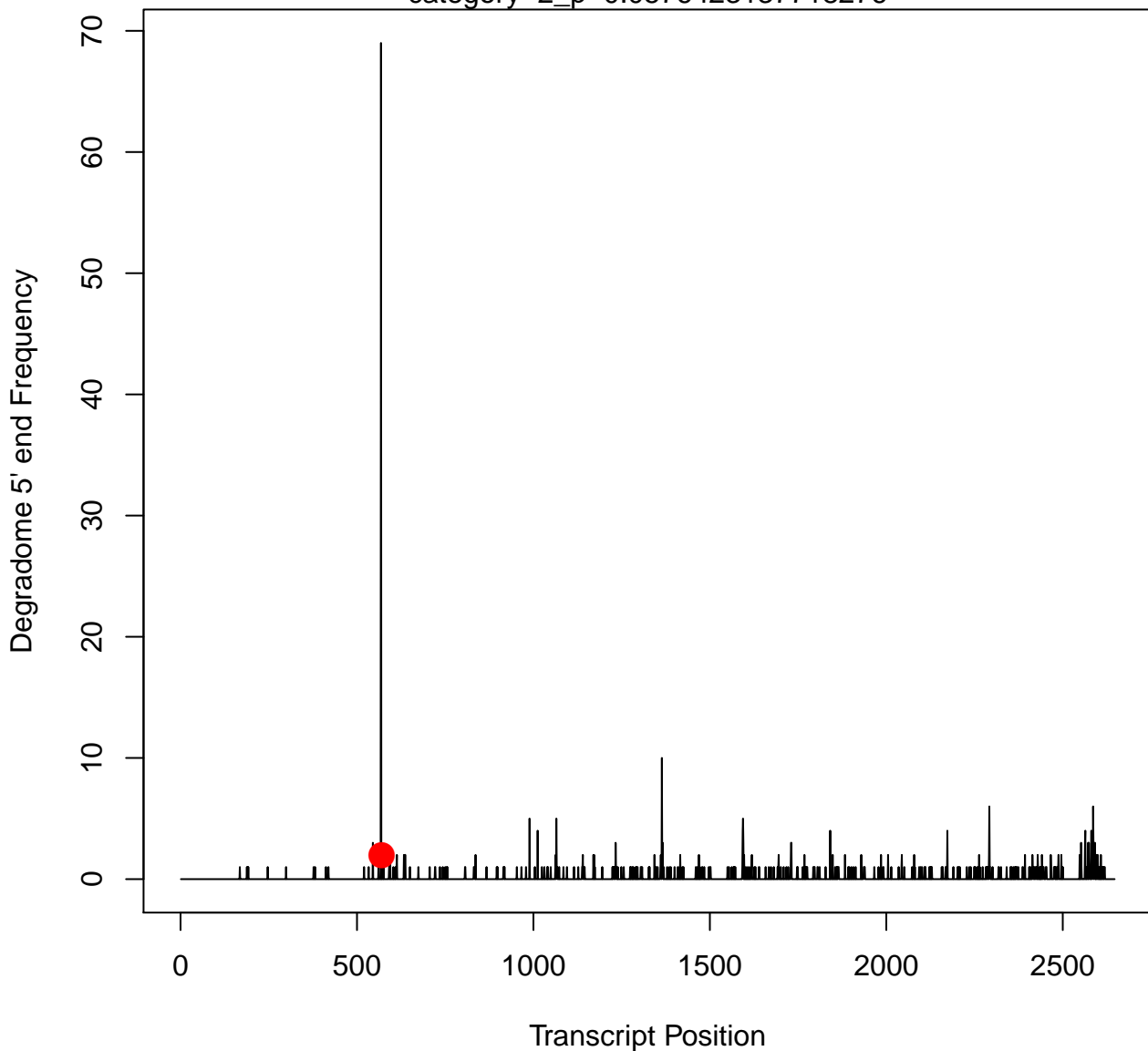

Supplement: Supplementary file 3 [file Data_Sheet_9.ZIP › GSM2230751.plot/Lsa-miR166i_Lsat_1_v5_gn_5_134441.1_570_TPlot.pdf]

**T=Lsat\_1\_v5\_gn\_5\_184280.1\_Q=Lsa-miR166i\_S=914**

category=0\_p=0.00295253139460594

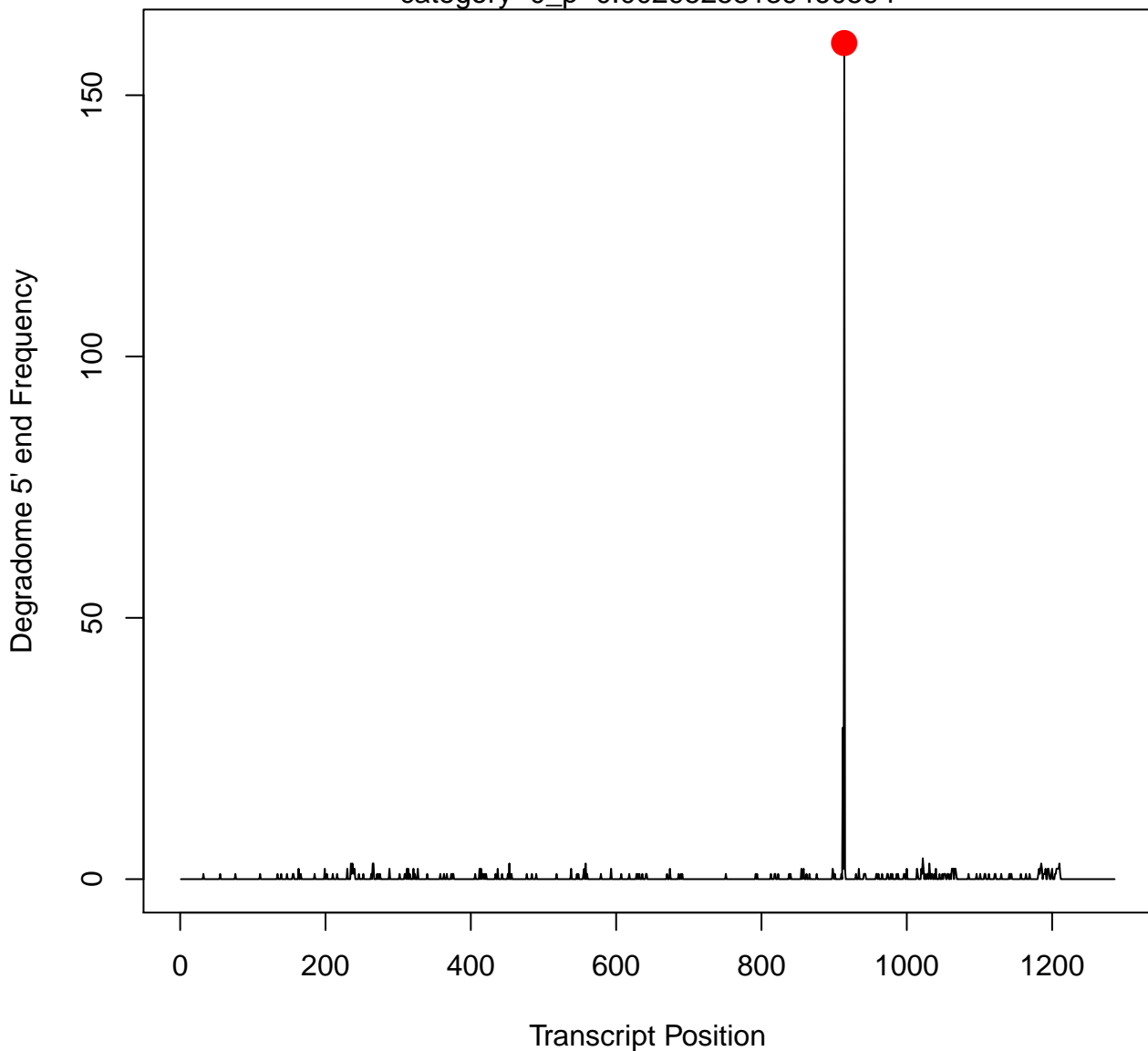

Supplement: Supplementary file 3 [file Data_Sheet_9.ZIP › GSM2230751.plot/Lsa-miR166i_Lsat_1_v5_gn_5_184280.1_914_TPlot.pdf]

**T=Lsat\_1\_v5\_gn\_5\_23401.1\_Q=Lsa-miR166i\_S=465**

category=2\_p=0.914895063331028

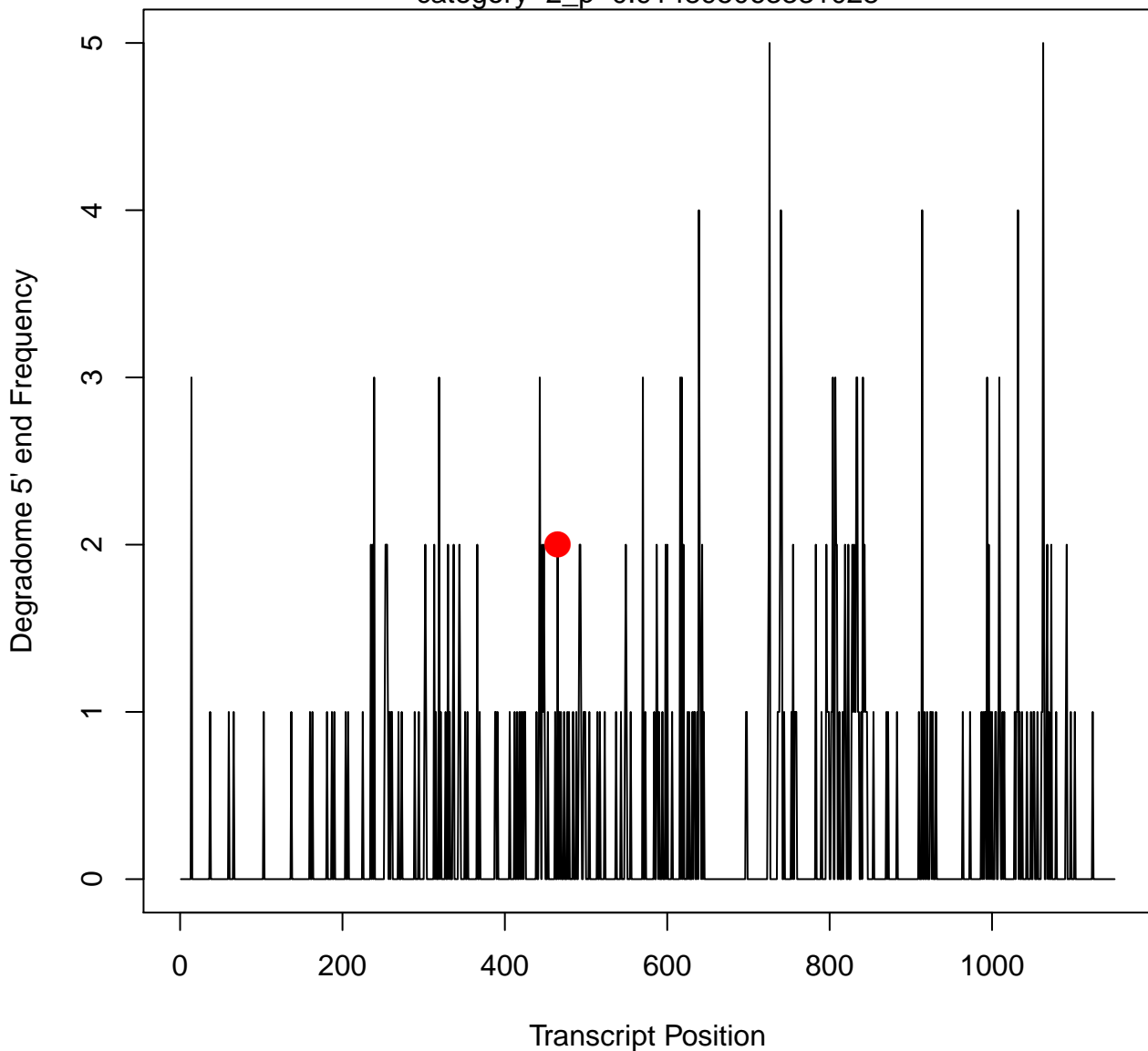

Supplement: Supplementary file 3 [file Data_Sheet_9.ZIP › GSM2230751.plot/Lsa-miR166i_Lsat_1_v5_gn_5_23401.1_465_TPlot.pdf]

**T=Lsat\_1\_v5\_gn\_5\_84680.1\_Q=Lsa-miR166i\_S=2196**

category=2\_p=0.85040920519287

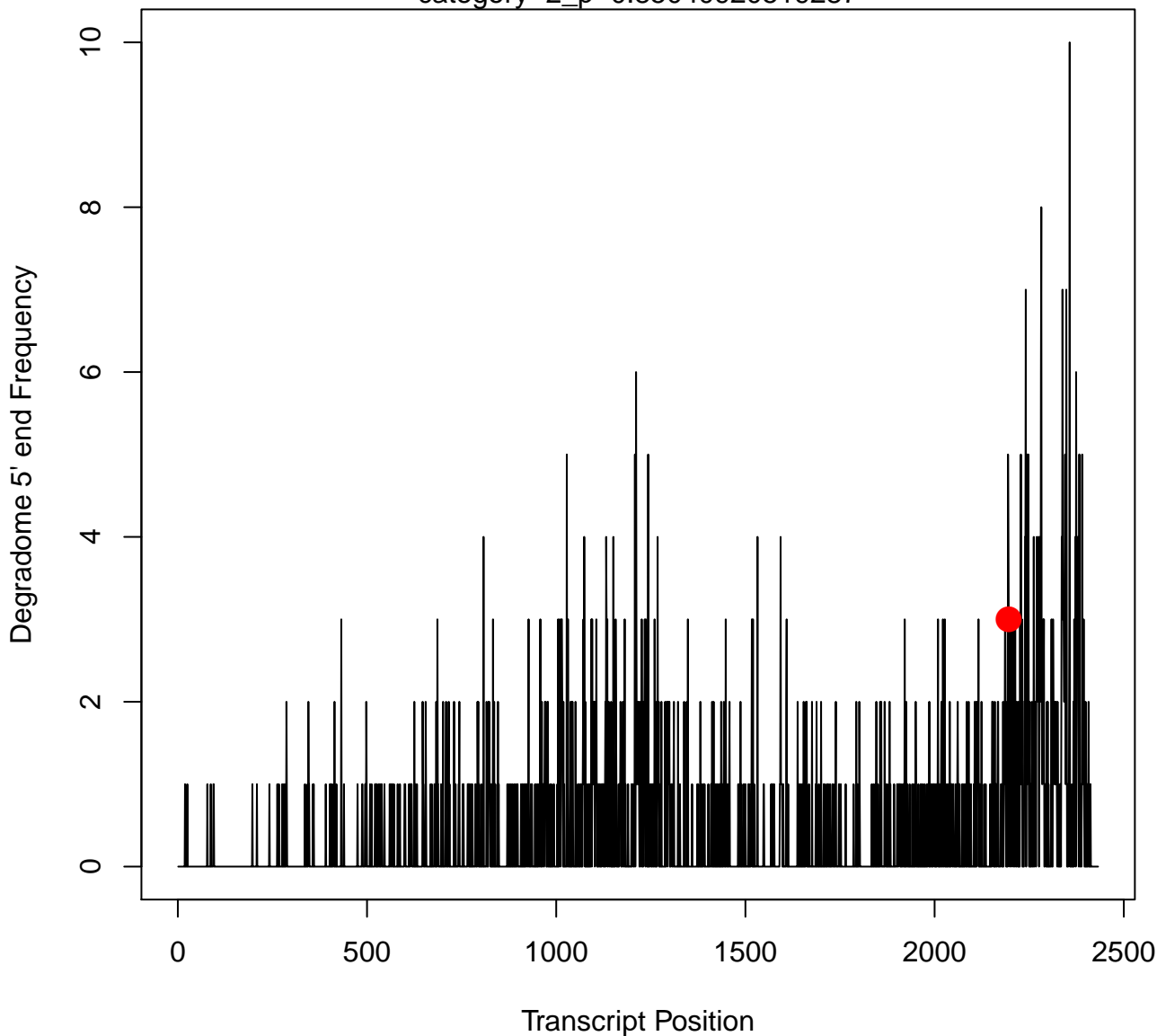

Supplement: Supplementary file 3 [file Data_Sheet_9.ZIP › GSM2230751.plot/Lsa-miR166i_Lsat_1_v5_gn_5_84680.1_2196_TPlot.pdf]

**T=Lsat\_1\_v5\_gn\_6\_2981.1\_Q=Lsa-miR166i\_S=487**

category=2\_p=0.601576866974249

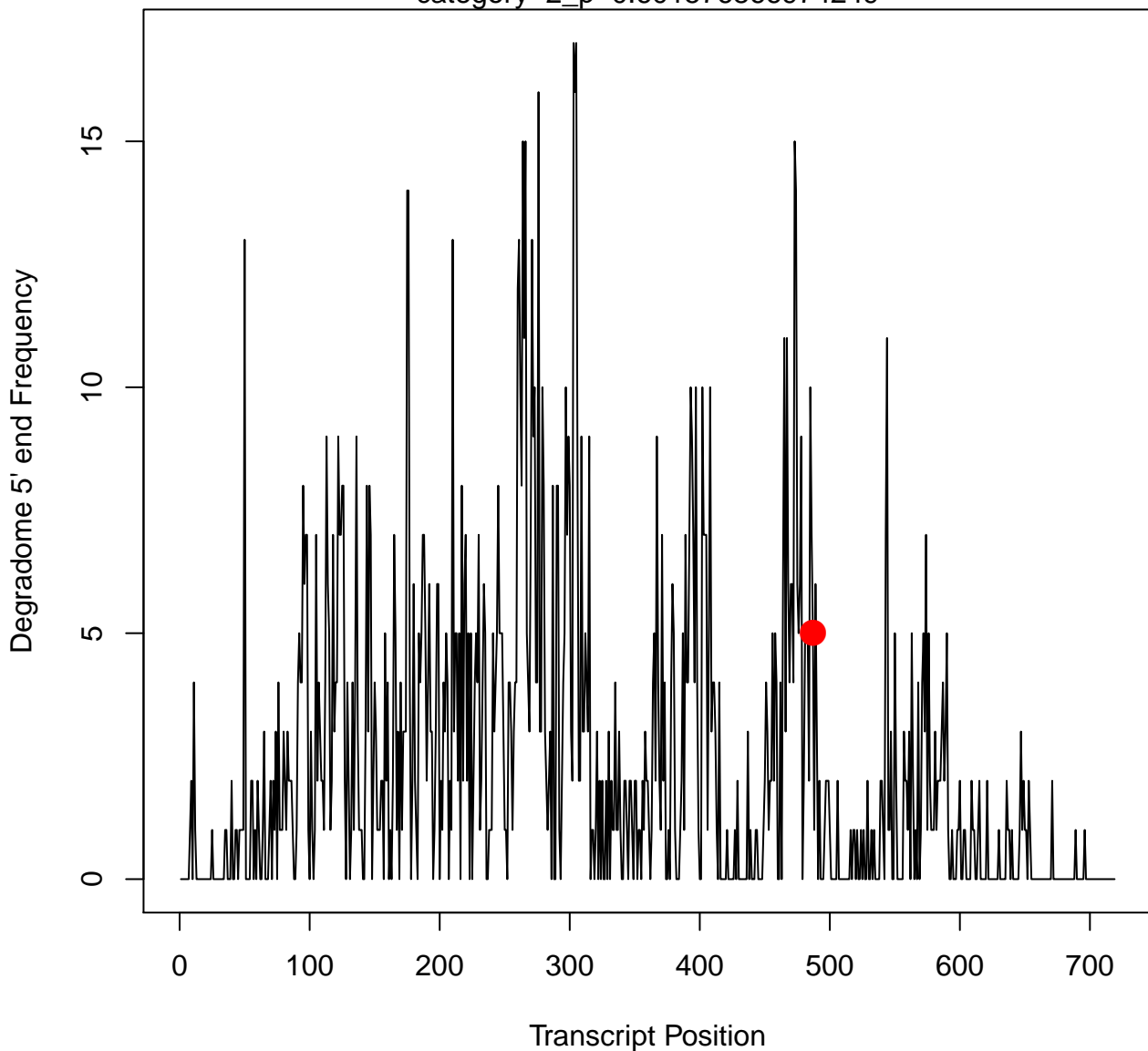

Supplement: Supplementary file 3 [file Data_Sheet_9.ZIP › GSM2230751.plot/Lsa-miR166i_Lsat_1_v5_gn_6_2981.1_487_TPlot.pdf]

**T=Lsat\_1\_v5\_gn\_6\_45641.1\_Q=Lsa-miR166i\_S=1311**

category=2\_p=0.187627487570719

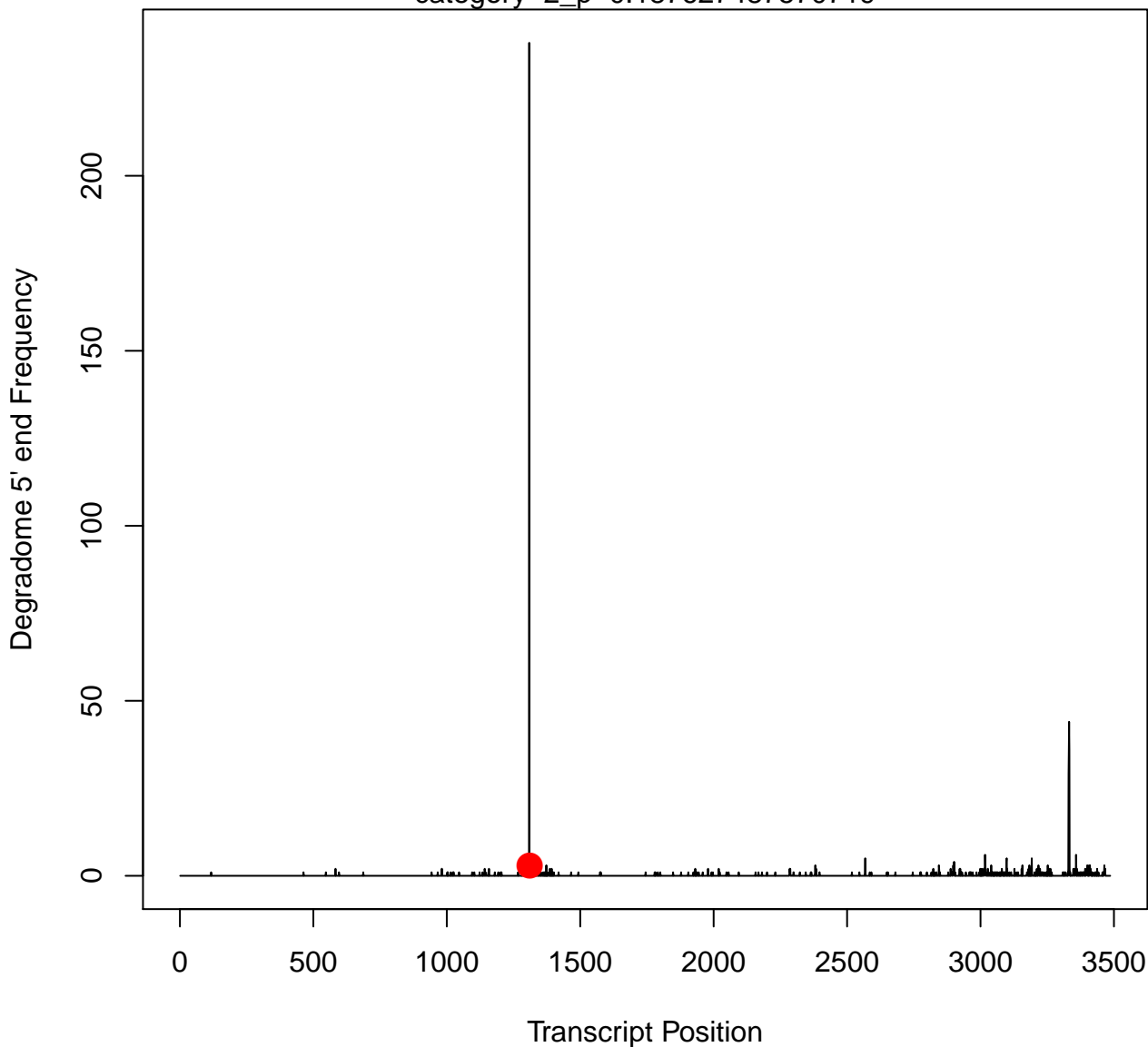

Supplement: Supplementary file 3 [file Data_Sheet_9.ZIP › GSM2230751.plot/Lsa-miR166i_Lsat_1_v5_gn_6_45641.1_1311_TPlot.pdf]

**T=Lsat\_1\_v5\_gn\_3\_72820.1\_Q=Lsa-miR167b\_S=531**

category=2\_p=0.773329799154233

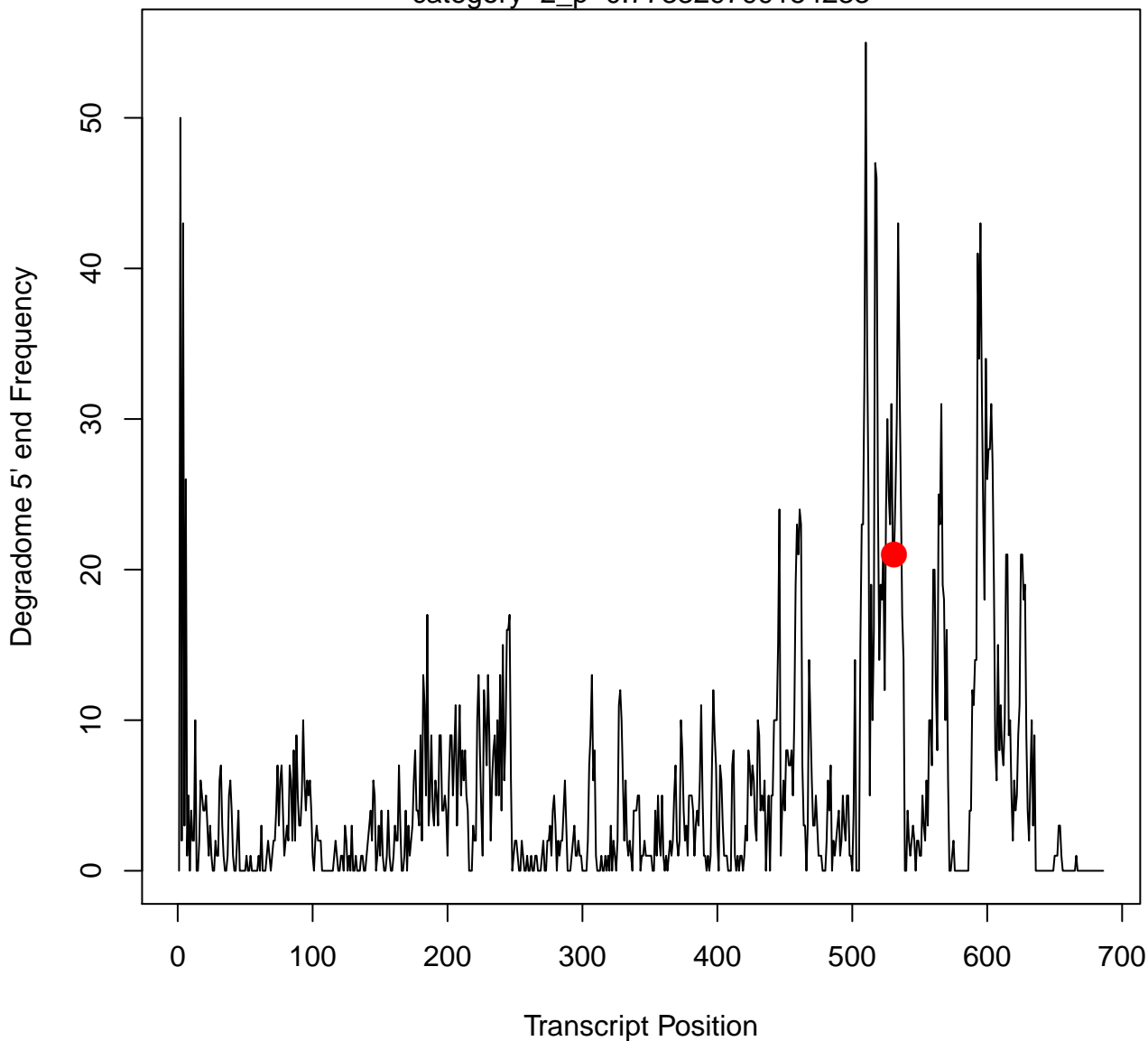

Supplement: Supplementary file 3 [file Data_Sheet_9.ZIP › GSM2230751.plot/Lsa-miR167b_Lsat_1_v5_gn_3_72820.1_531_TPlot.pdf]

**T=Lsat\_1\_v5\_gn\_7\_27300.1\_Q=Lsa-miR167c\_S=2921**

category=0\_p=0.00147735698913964

Degradome 5' end Frequency

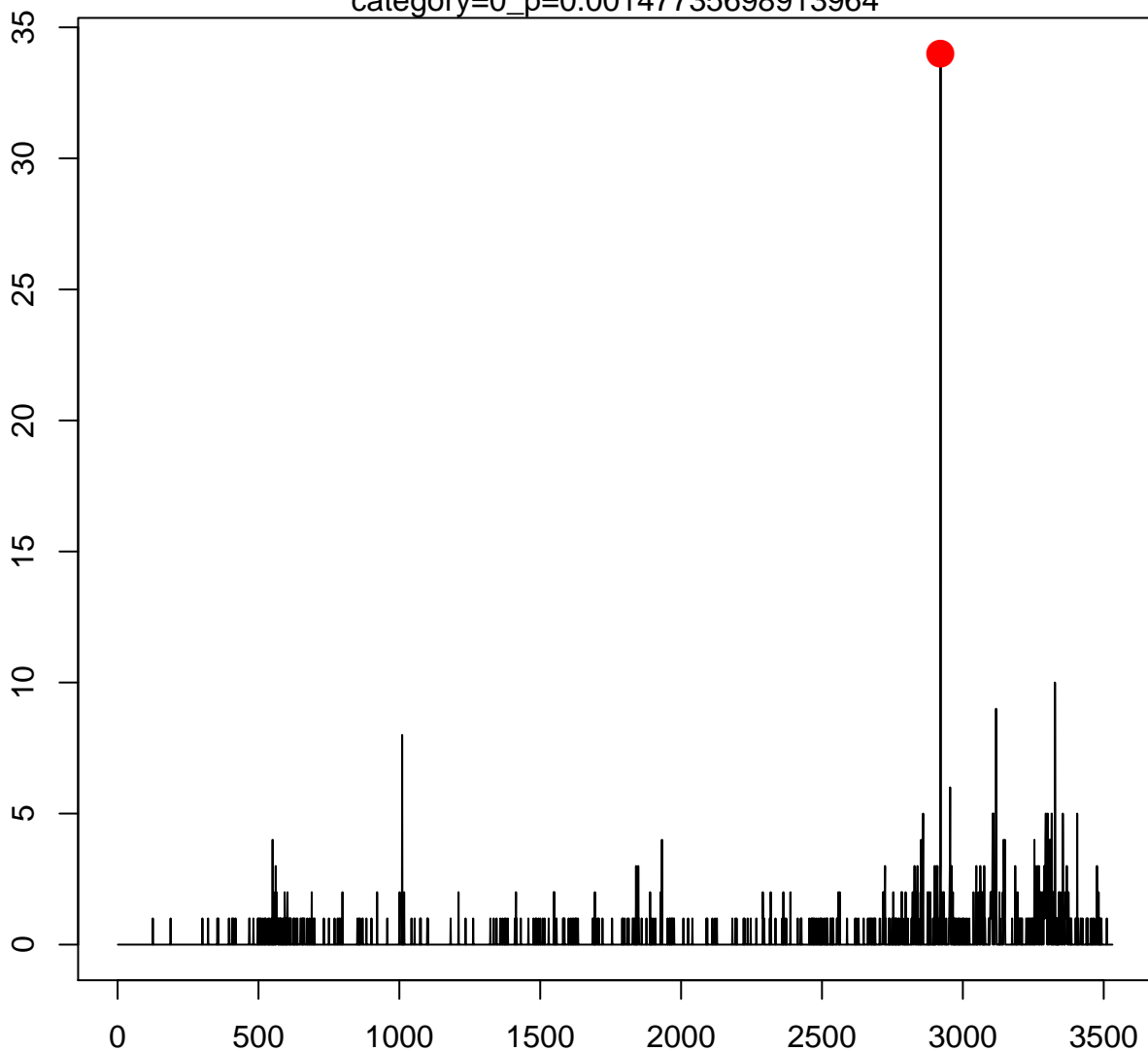

Transcript Position

Supplement: Supplementary file 3 [file Data_Sheet_9.ZIP › GSM2230751.plot/Lsa-miR167c_Lsat_1_v5_gn_7_27300.1_2921_TPlot.pdf]

**T=Lsat\_1\_v5\_gn\_3\_68721.1\_Q=Lsa-miR167d\_S=2661**

category=2\_p=0.137936131575646

Degradsome 5' end Frequency

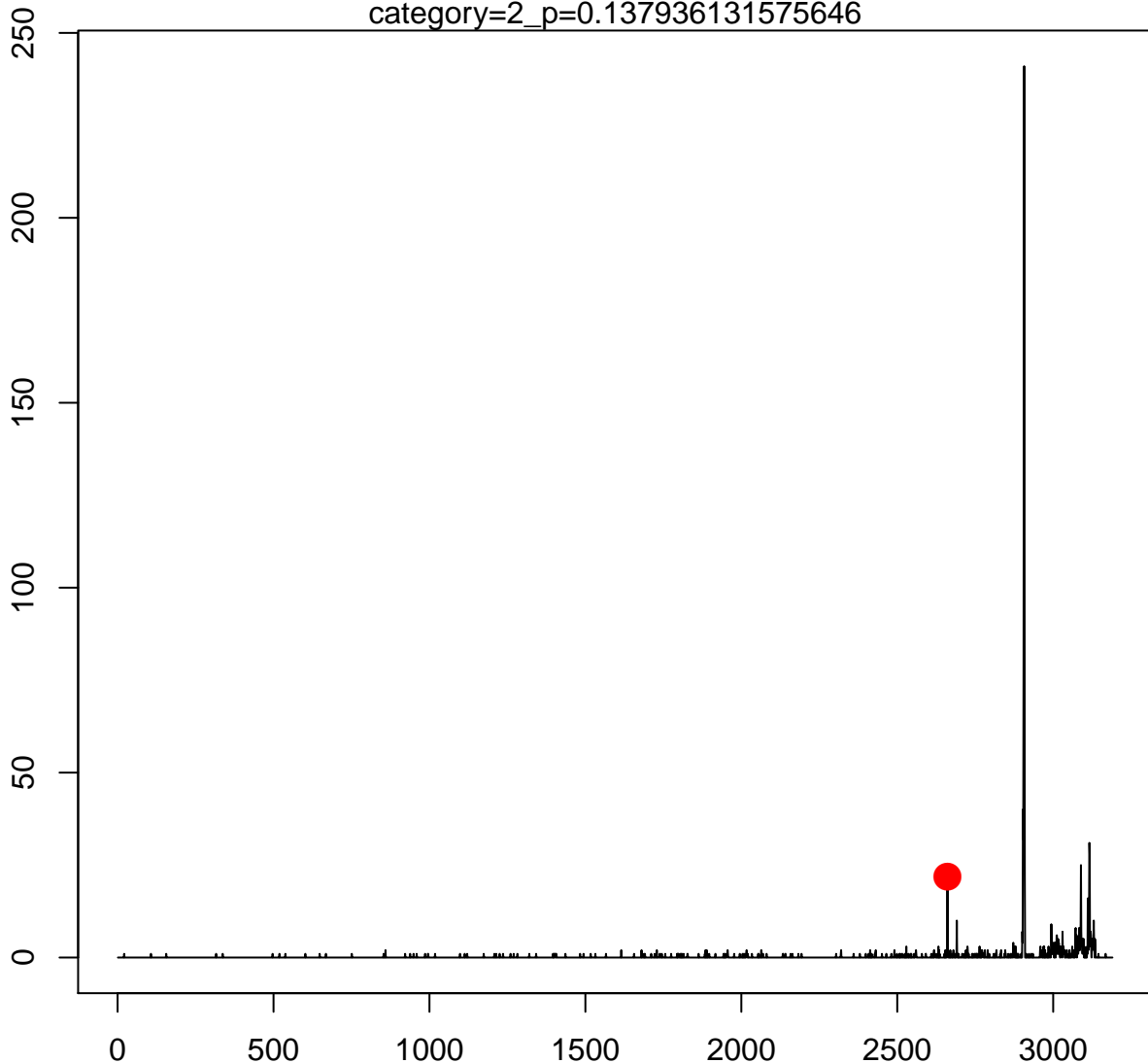

Transcript Position

Supplement: Supplementary file 3 [file Data_Sheet_9.ZIP › GSM2230751.plot/Lsa-miR167d_Lsat_1_v5_gn_3_68721.1_2661_TPlot.pdf]

**T=Lsat\_1\_v5\_gn\_7\_36161.1\_Q=Lsa-miR167d\_S=520**

category=2\_p=0.509556112583961

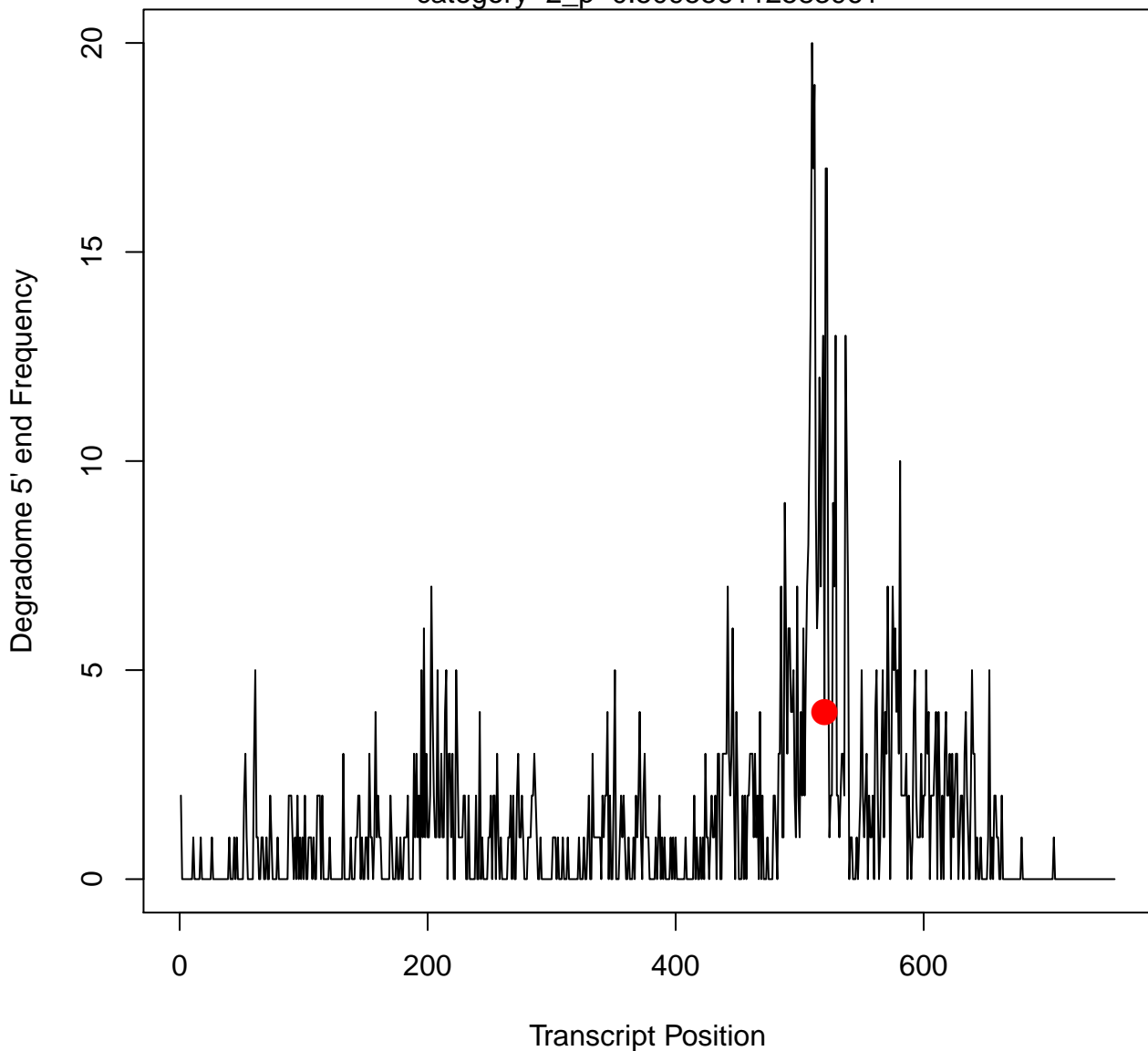

Supplement: Supplementary file 3 [file Data_Sheet_9.ZIP › GSM2230751.plot/Lsa-miR167d_Lsat_1_v5_gn_7_36161.1_520_TPlot.pdf]

**T=Lsat\_1\_v5\_gn\_2\_72540.1\_Q=Lsa-miR167e\_S=2777**

category=2\_p=0.137936131575646

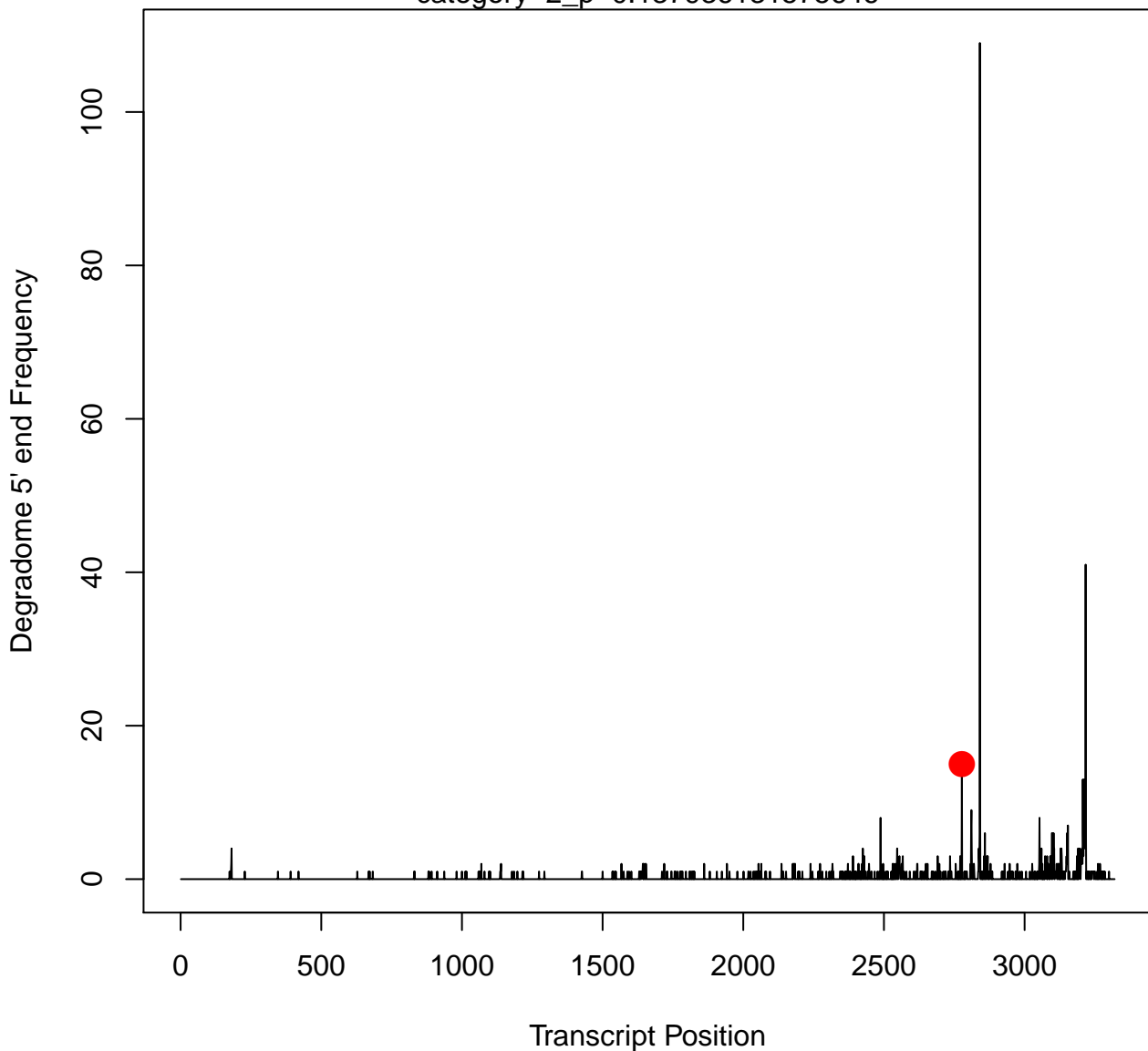

Supplement: Supplementary file 3 [file Data_Sheet_9.ZIP › GSM2230751.plot/Lsa-miR167e_Lsat_1_v5_gn_2_72540.1_2777_TPlot.pdf]

**T=Lsat\_1\_v5\_gn\_4\_159141.1\_Q=Lsa-miR167e\_S=1744**

category=2\_p=0.993938824804575

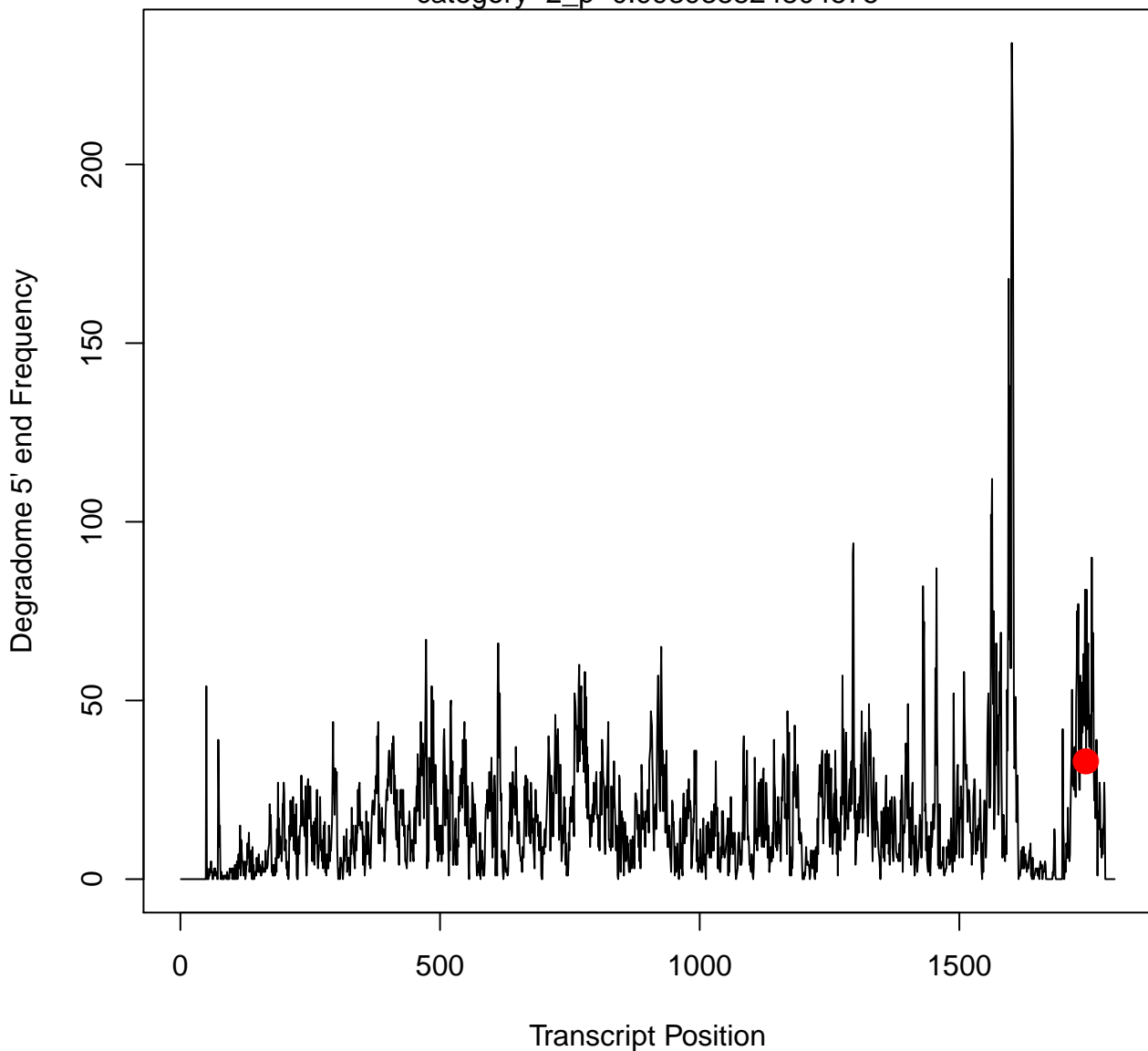

Supplement: Supplementary file 3 [file Data_Sheet_9.ZIP › GSM2230751.plot/Lsa-miR167e_Lsat_1_v5_gn_4_159141.1_1744_TPlot.pdf]

T=Lsat\_1\_v5\_gn\_1\_25461.1\_Q=Lsa-miR168a\_S=596

category=2\_p=0.936753916257189

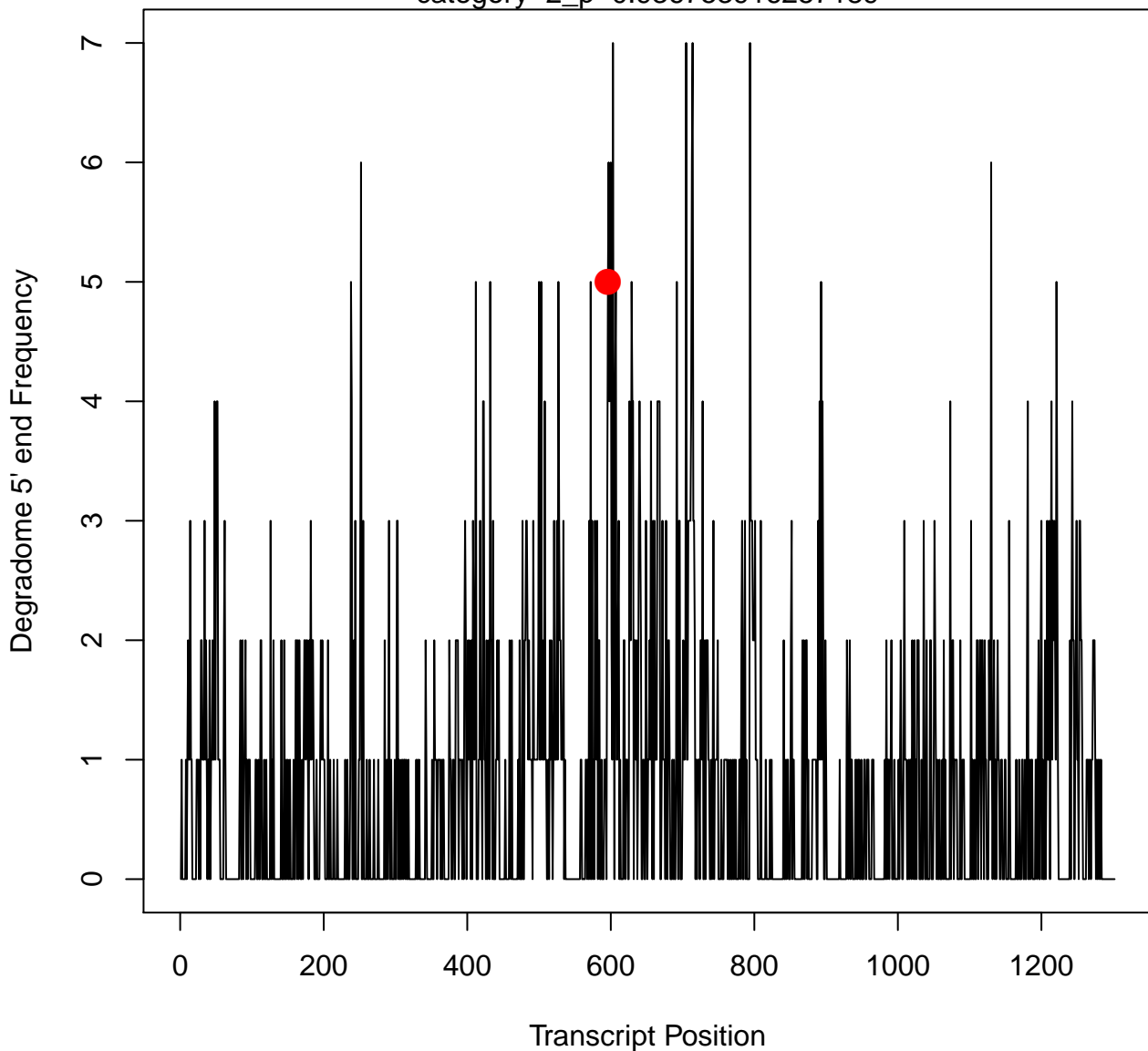

Supplement: Supplementary file 3 [file Data_Sheet_9.ZIP › GSM2230751.plot/Lsa-miR168a_Lsat_1_v5_gn_1_25461.1_596_TPlot.pdf]

**T=Lsat\_1\_v5\_gn\_1\_71221.1\_Q=Lsa-miR168a\_S=1266**

category=2\_p=0.85040920519287

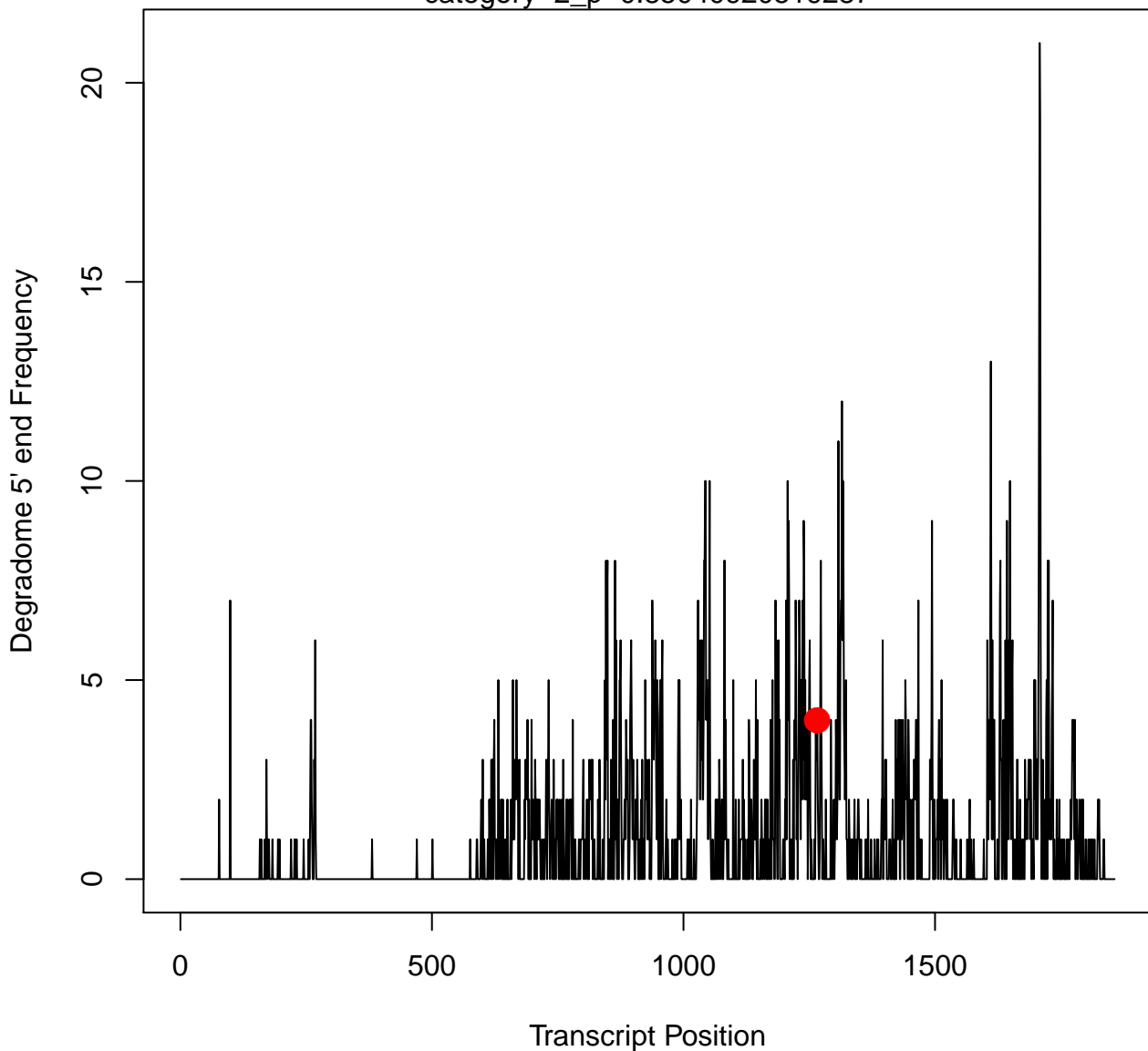

Supplement: Supplementary file 3 [file Data_Sheet_9.ZIP › GSM2230751.plot/Lsa-miR168a_Lsat_1_v5_gn_1_71221.1_1266_TPlot.pdf]

**T=Lsat\_1\_v5\_gn\_3\_2841.1\_Q=Lsa-miR168a\_S=205**

category=2\_p=0.798708246188144

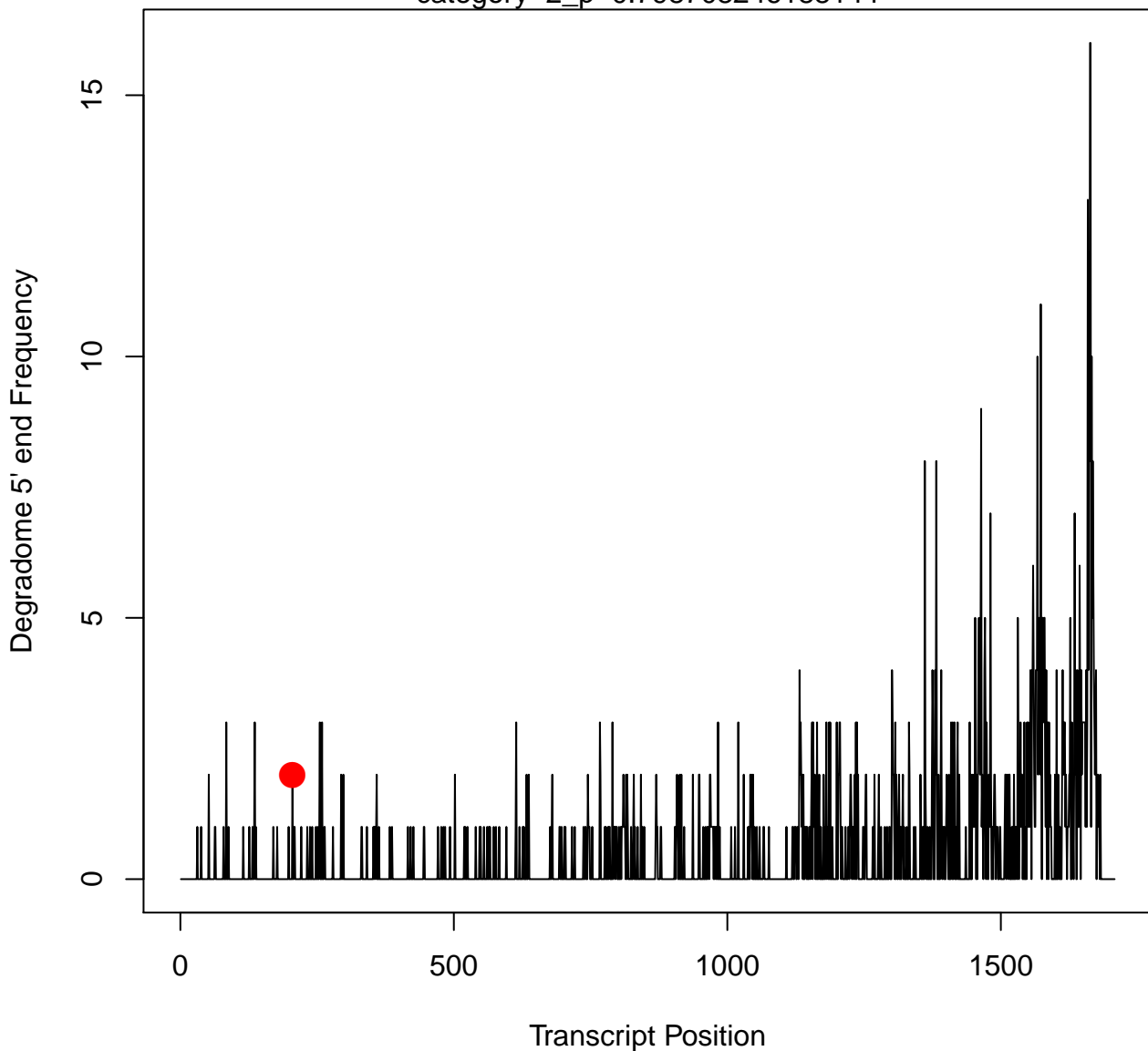

Supplement: Supplementary file 3 [file Data_Sheet_9.ZIP › GSM2230751.plot/Lsa-miR168a_Lsat_1_v5_gn_3_2841.1_205_TPlot.pdf]

**T=Lsat\_1\_v5\_gn\_3\_35820.1\_Q=Lsa-miR168a\_S=1750**

category=2\_p=0.882030978163789

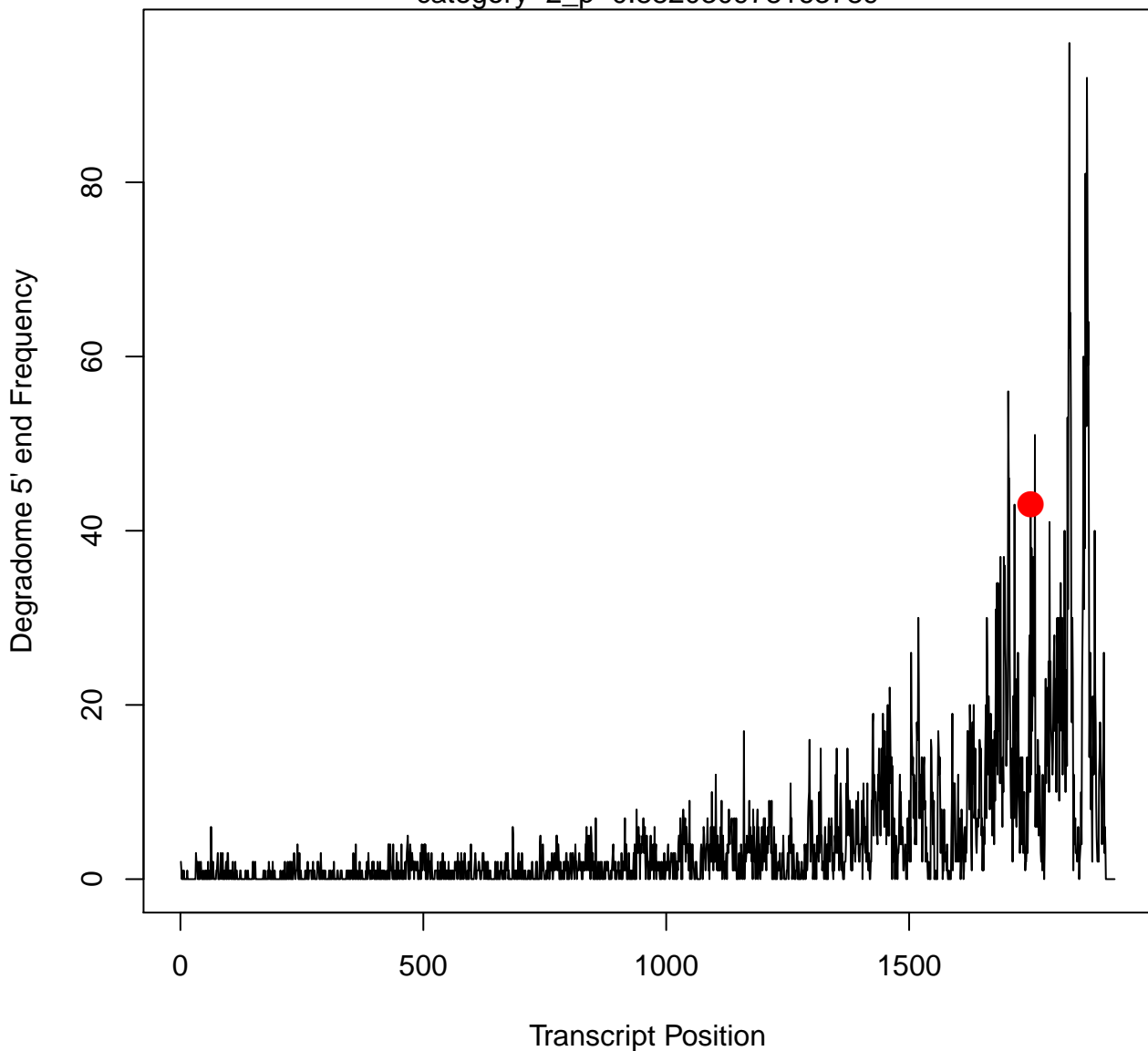

Supplement: Supplementary file 3 [file Data_Sheet_9.ZIP › GSM2230751.plot/Lsa-miR168a_Lsat_1_v5_gn_3_35820.1_1750_TPlot.pdf]

**T=Lsat\_1\_v5\_gn\_5\_103680.1\_Q=Lsa-miR168a\_S=1484**

category=2\_p=0.821245271758406

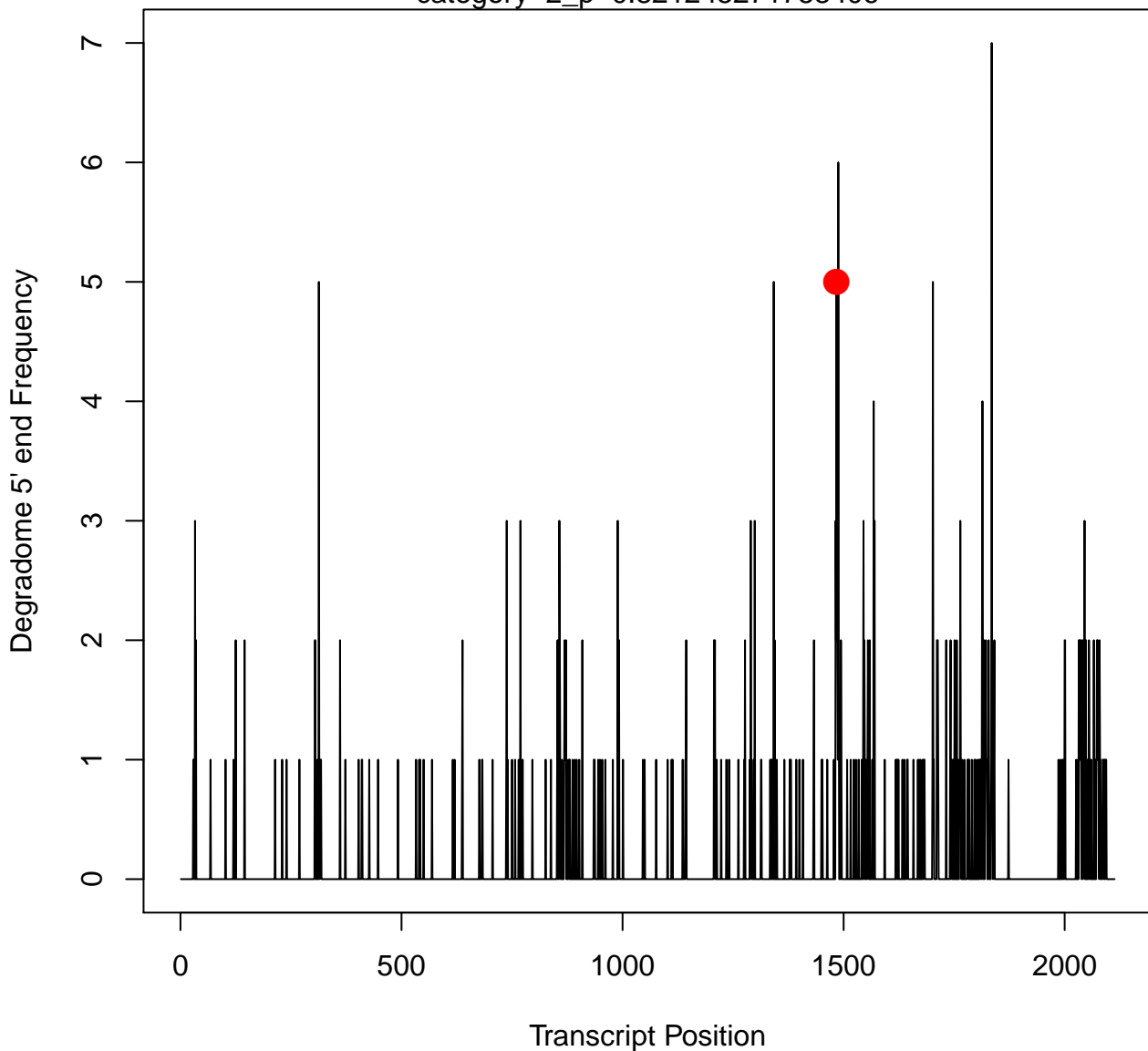

Supplement: Supplementary file 3 [file Data_Sheet_9.ZIP › GSM2230751.plot/Lsa-miR168a_Lsat_1_v5_gn_5_103680.1_1484_TPlot.pdf]

**T=Lsat\_1\_v5\_gn\_8\_261.1\_Q=Lsa-miR168a\_S=1295**

category=0\_p=0.000369544041020964

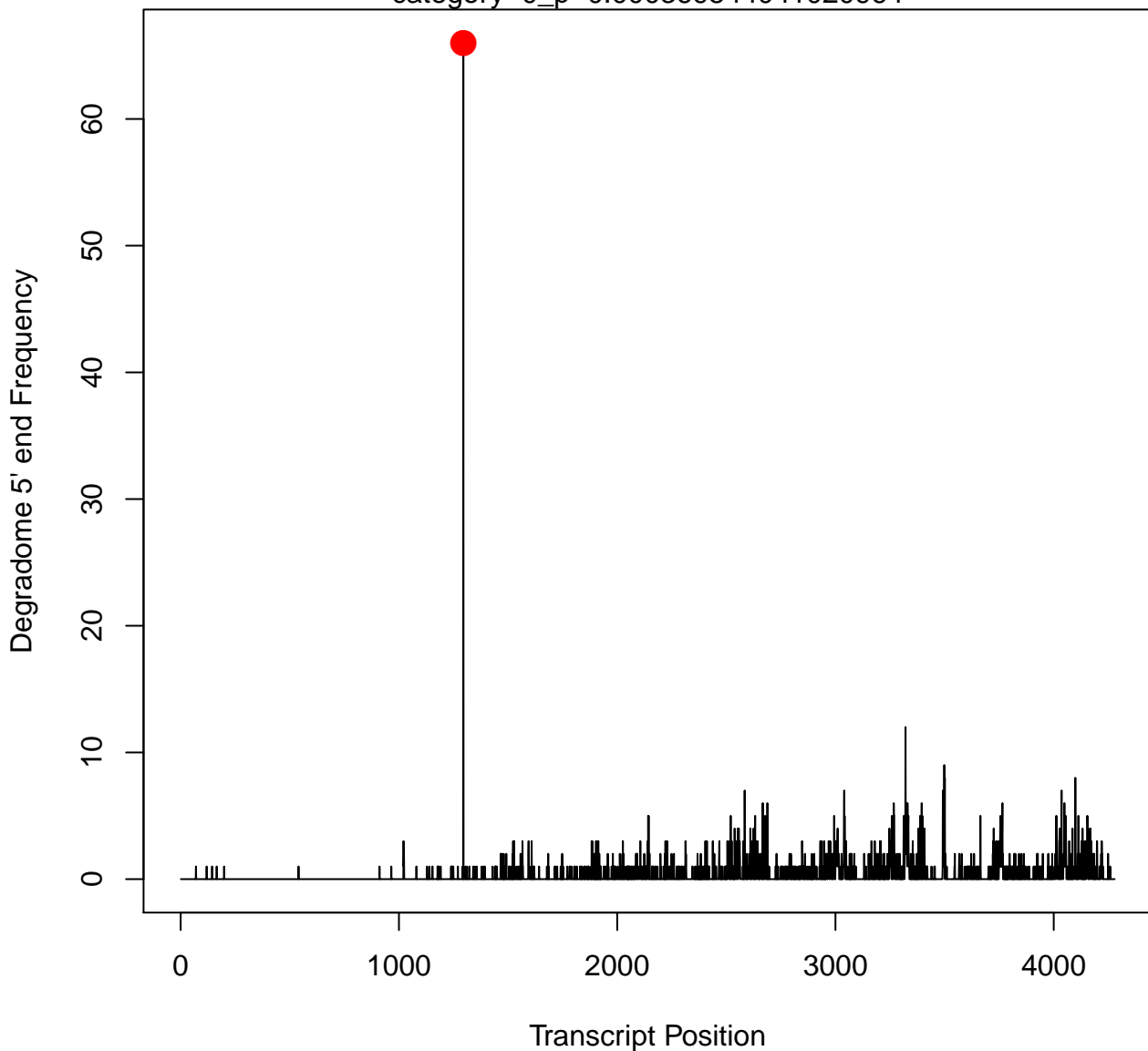

Supplement: Supplementary file 3 [file Data_Sheet_9.ZIP › GSM2230751.plot/Lsa-miR168a_Lsat_1_v5_gn_8_261.1_1295_TPlot.pdf]

**T=Lsat\_1\_v5\_gn\_8\_85500.1\_Q=Lsa-miR168a\_S=307**

category=2\_p=0.509556112583961

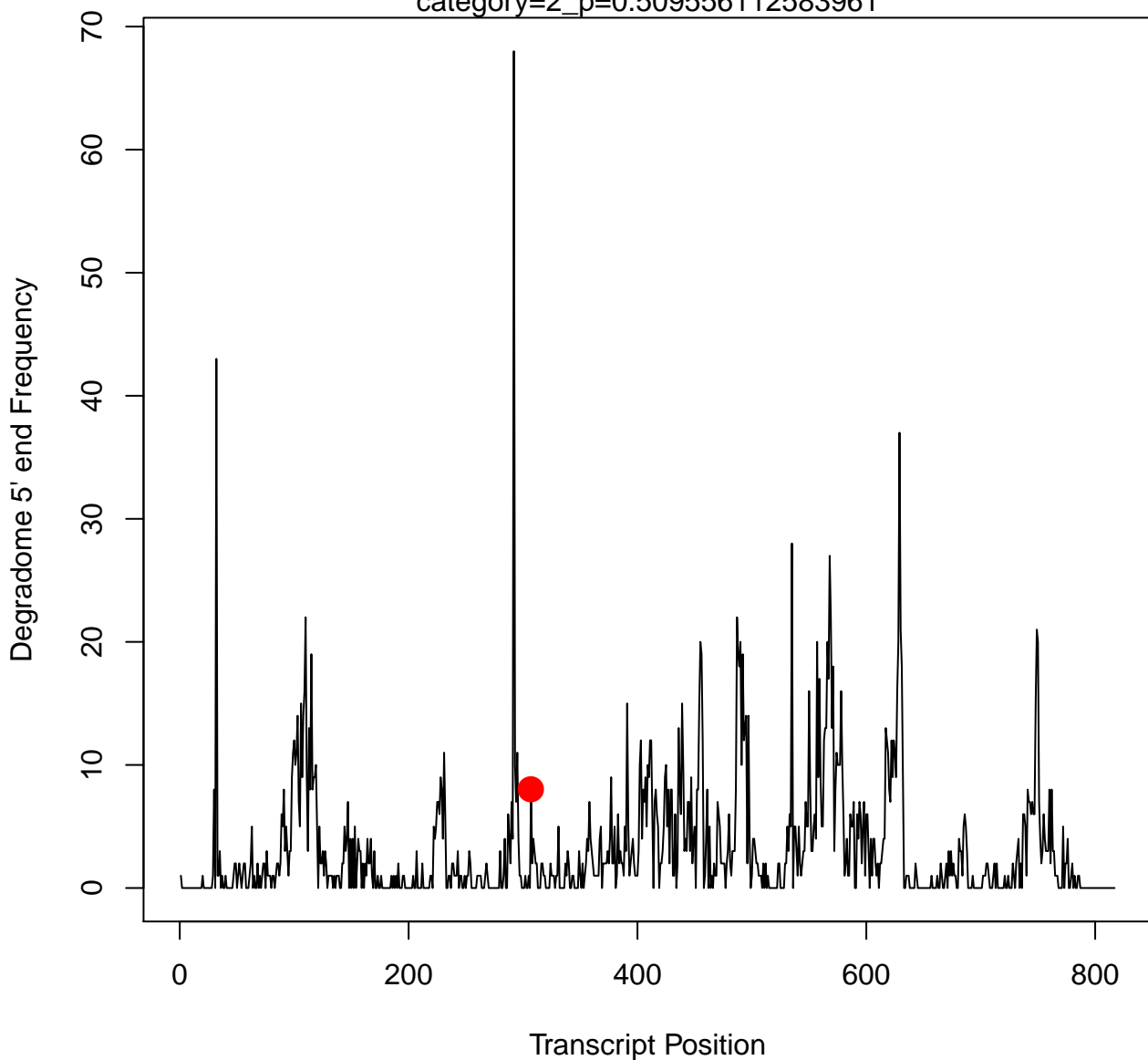

Supplement: Supplementary file 3 [file Data_Sheet_9.ZIP › GSM2230751.plot/Lsa-miR168a_Lsat_1_v5_gn_8_85500.1_307_TPlot.pdf]

**T=Lsat\_1\_v5\_gn\_9\_103440.1\_Q=Lsa-miR168a\_S=1363**

category=2\_p=0.685798933413206

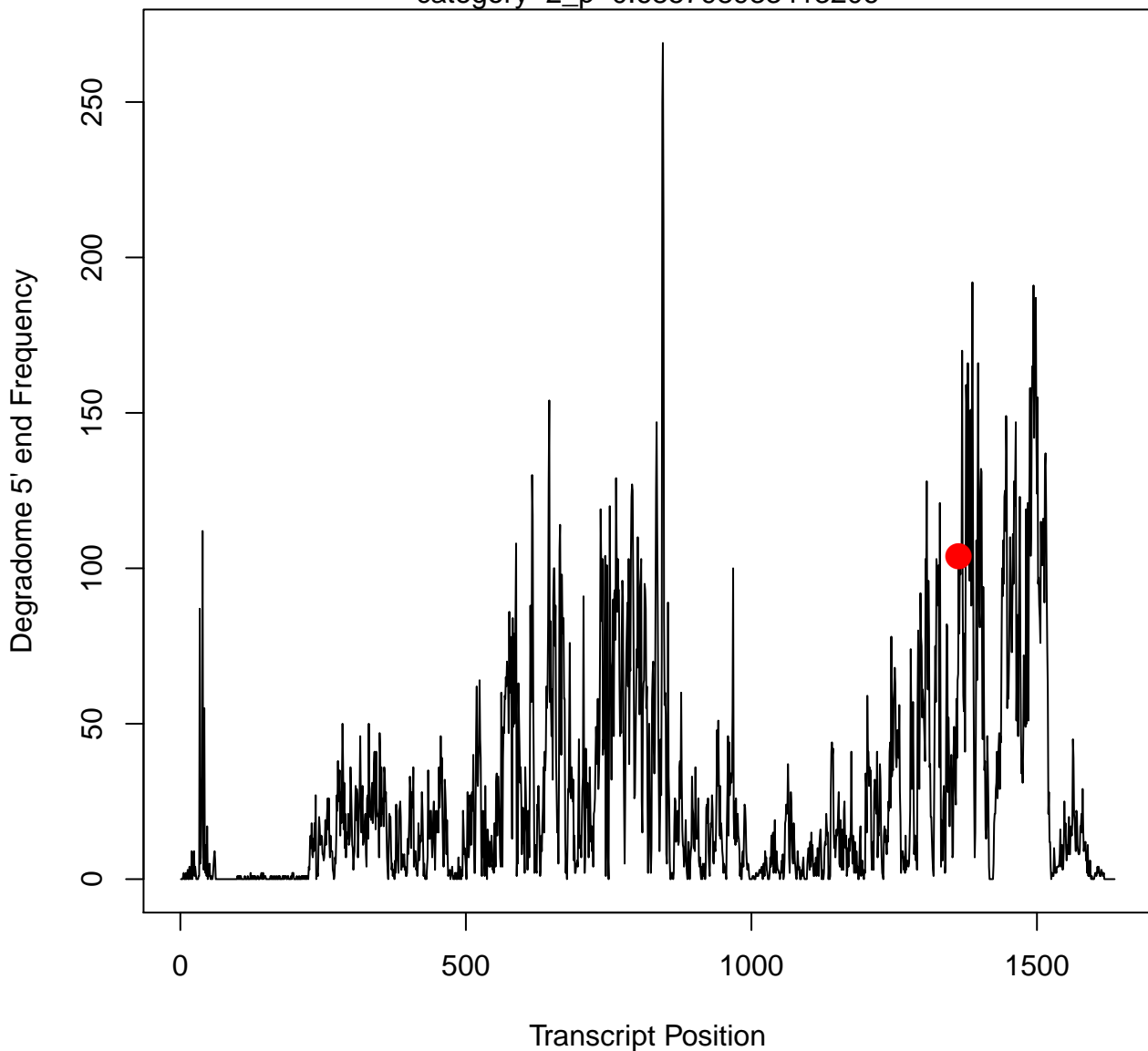

Supplement: Supplementary file 3 [file Data_Sheet_9.ZIP › GSM2230751.plot/Lsa-miR168a_Lsat_1_v5_gn_9_103440.1_1363_TPlot.pdf]

**T=Lsat\_1\_v5\_gn\_9\_56521.1\_Q=Lsa-miR168a\_S=699**

category=2\_p=0.94862062004854

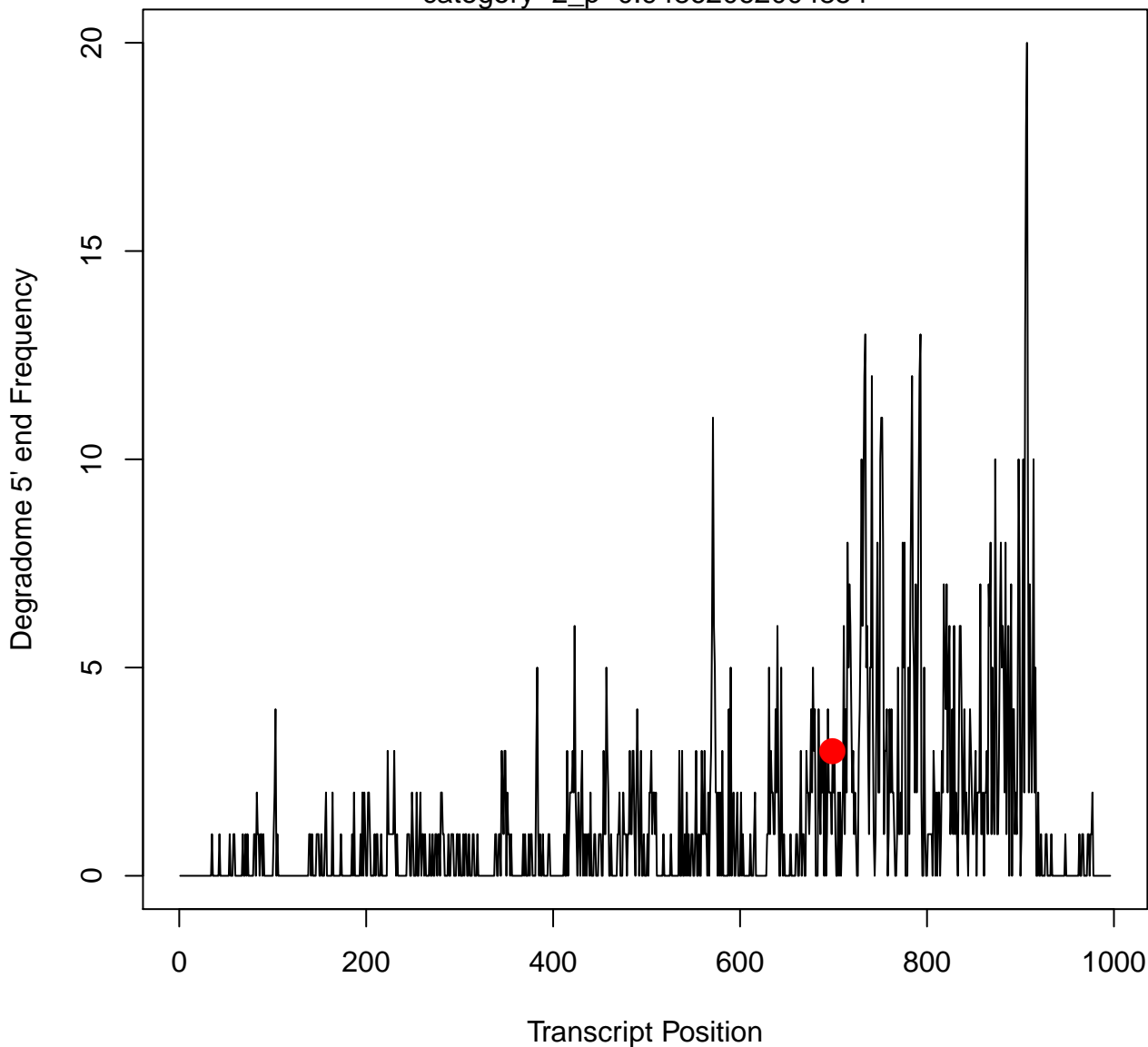

Supplement: Supplementary file 3 [file Data_Sheet_9.ZIP › GSM2230751.plot/Lsa-miR168a_Lsat_1_v5_gn_9_56521.1_699_TPlot.pdf]

**T=Lsat\_1\_v5\_gn\_2\_19760.1\_Q=Lsa-miR168b\_S=1492**

category=0\_p=0.000369544041020964

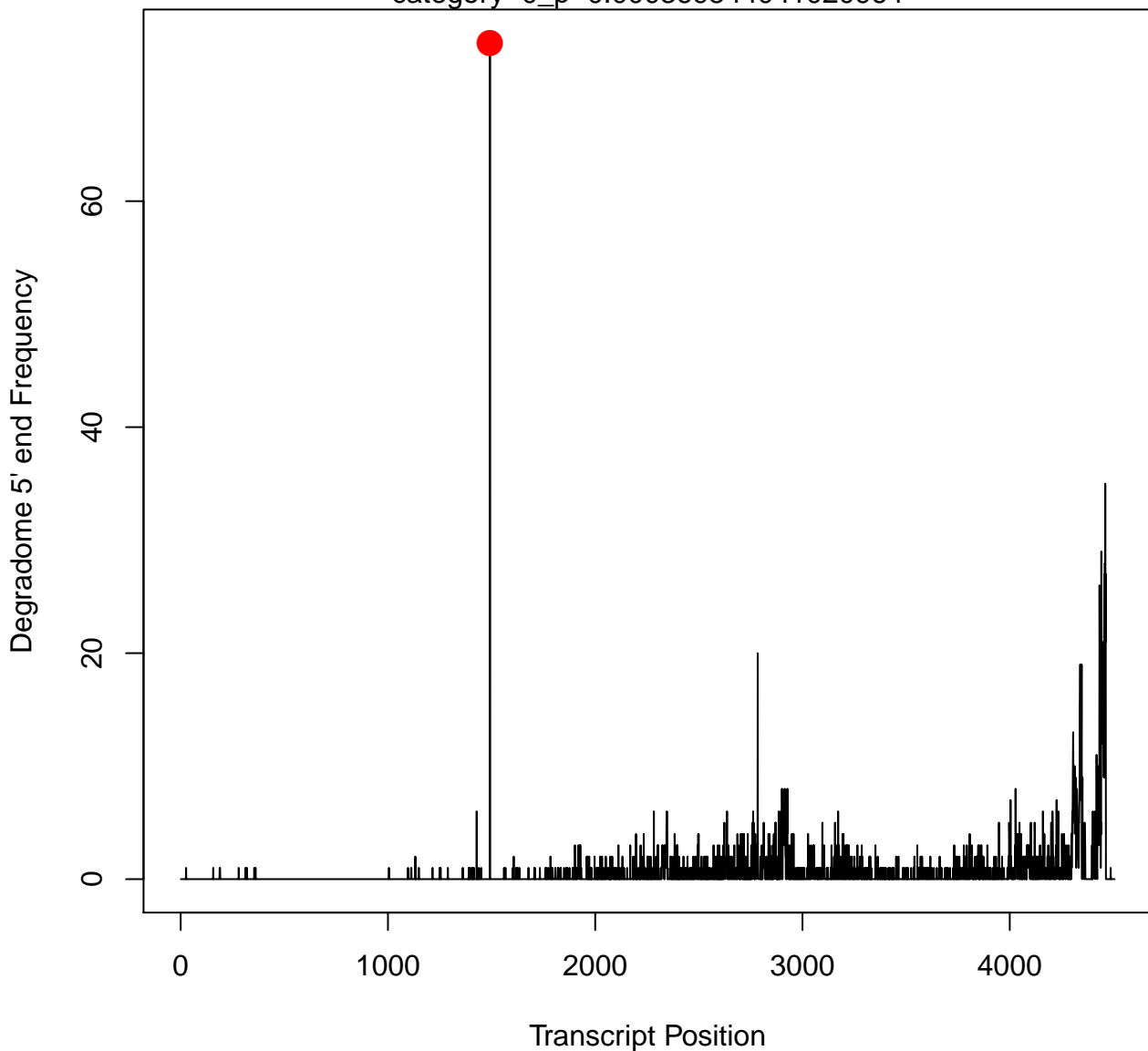

Supplement: Supplementary file 3 [file Data_Sheet_9.ZIP › GSM2230751.plot/Lsa-miR168b_Lsat_1_v5_gn_2_19760.1_1492_TPlot.pdf]

**T=Lsat\_1\_v5\_gn\_4\_131340.1\_Q=Lsa-miR168b\_S=6221**

category=2\_p=0.666579822949821

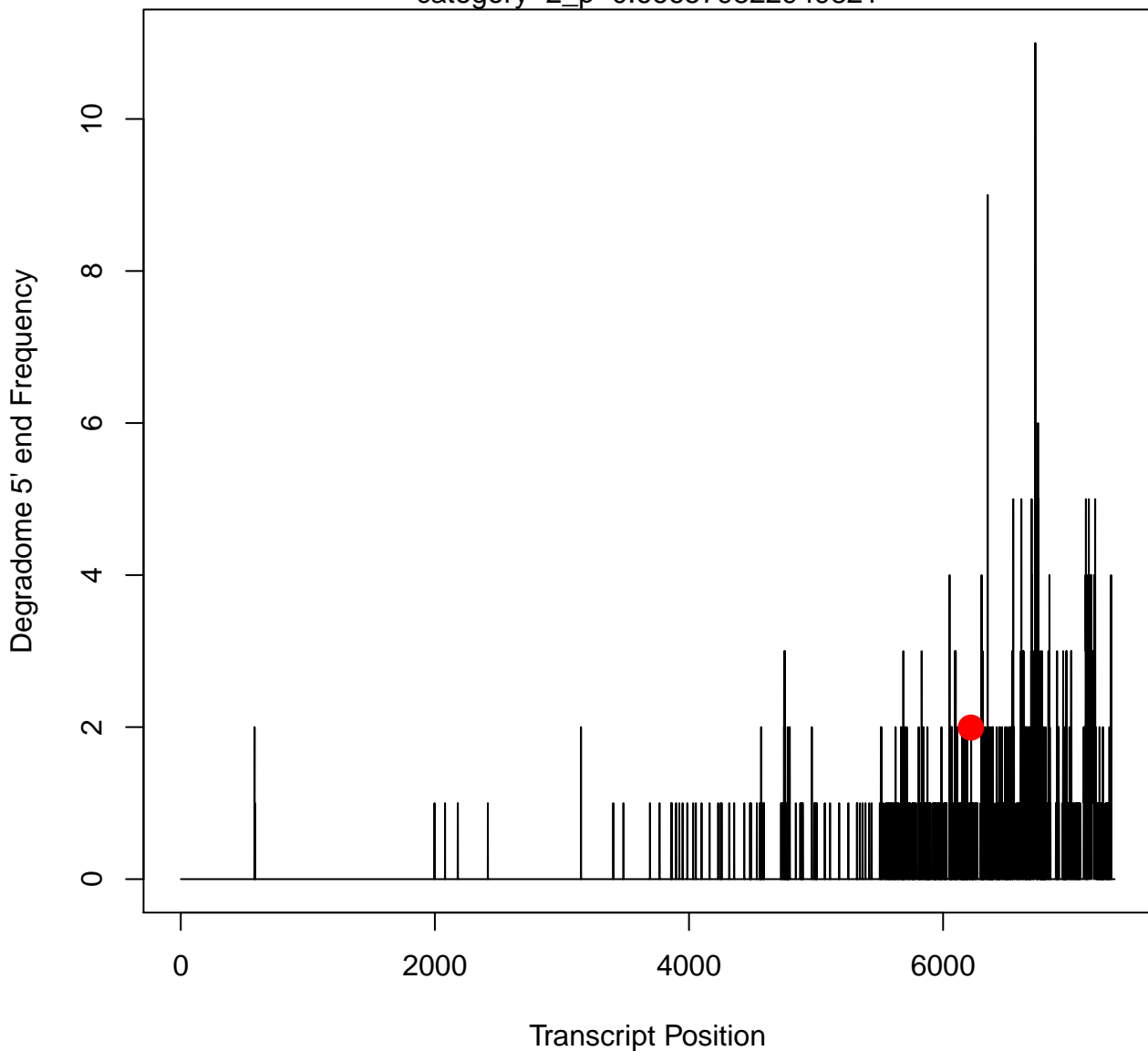

Supplement: Supplementary file 3 [file Data_Sheet_9.ZIP › GSM2230751.plot/Lsa-miR168b_Lsat_1_v5_gn_4_131340.1_6221_TPlot.pdf]

**T=Lsat\_1\_v5\_gn\_4\_173421.1\_Q=Lsa-miR168b\_S=938**

category=2\_p=0.759464793296244

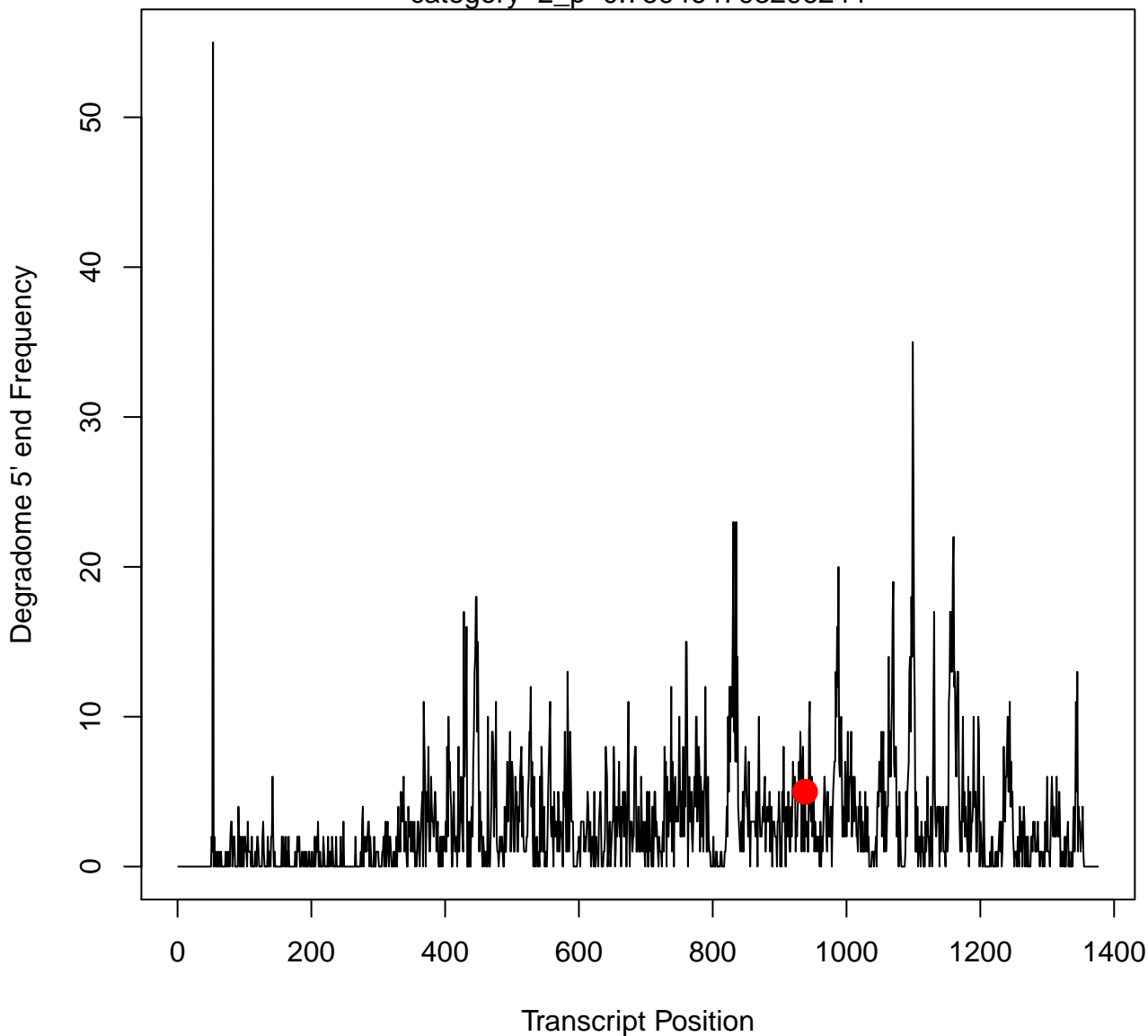

Supplement: Supplementary file 3 [file Data_Sheet_9.ZIP › GSM2230751.plot/Lsa-miR168b_Lsat_1_v5_gn_4_173421.1_938_TPlot.pdf]

**T=Lsat\_1\_v5\_gn\_8\_88620.1\_Q=Lsa-miR168b\_S=991**

category=2\_p=0.396282025902823

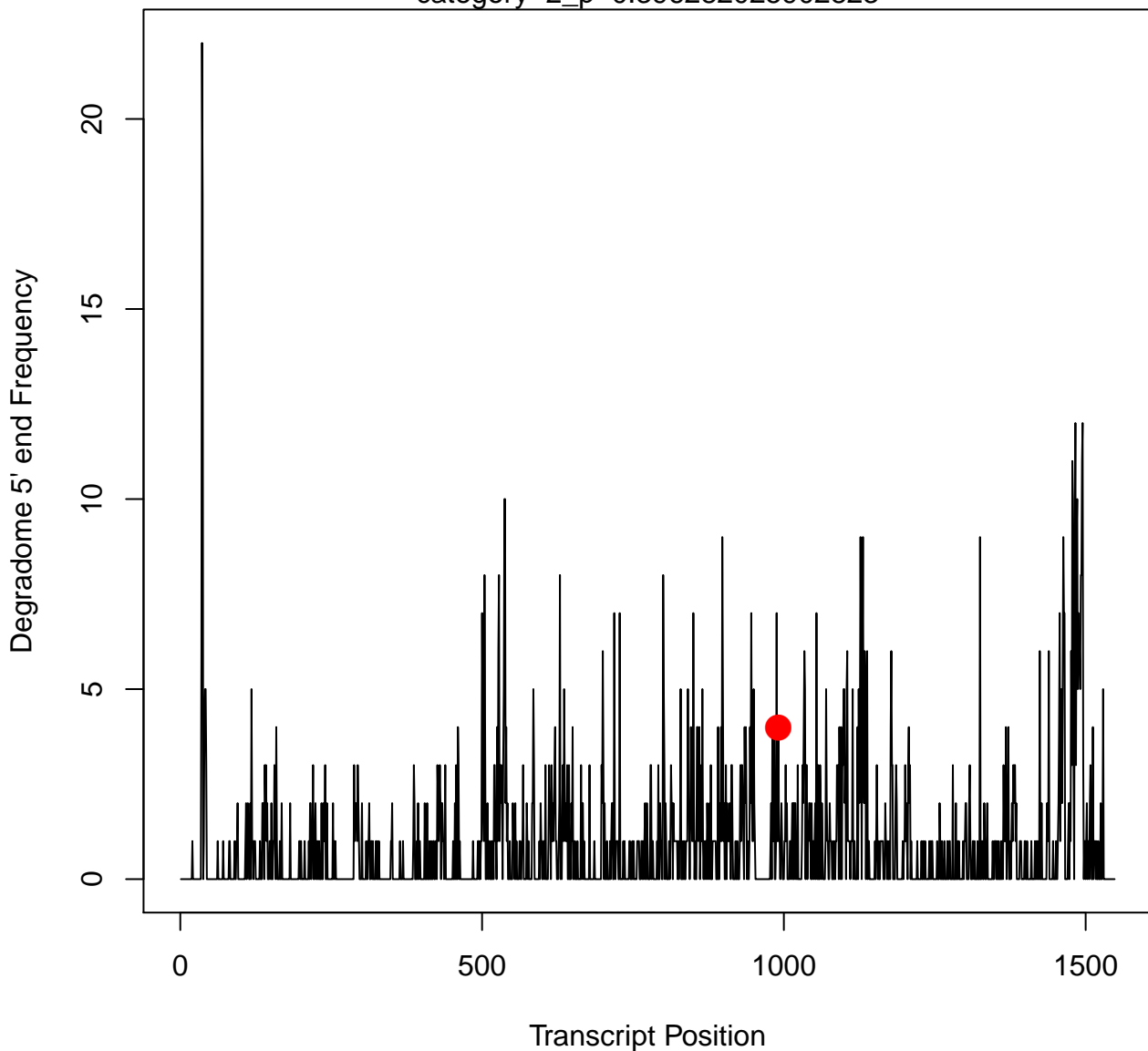

Supplement: Supplementary file 3 [file Data_Sheet_9.ZIP › GSM2230751.plot/Lsa-miR168b_Lsat_1_v5_gn_8_88620.1_991_TPlot.pdf]

**T=Lsat\_1\_v5\_gn\_7\_34841.1\_Q=Lsa-miR169a\_S=986**

category=0\_p=0.0011082224851342

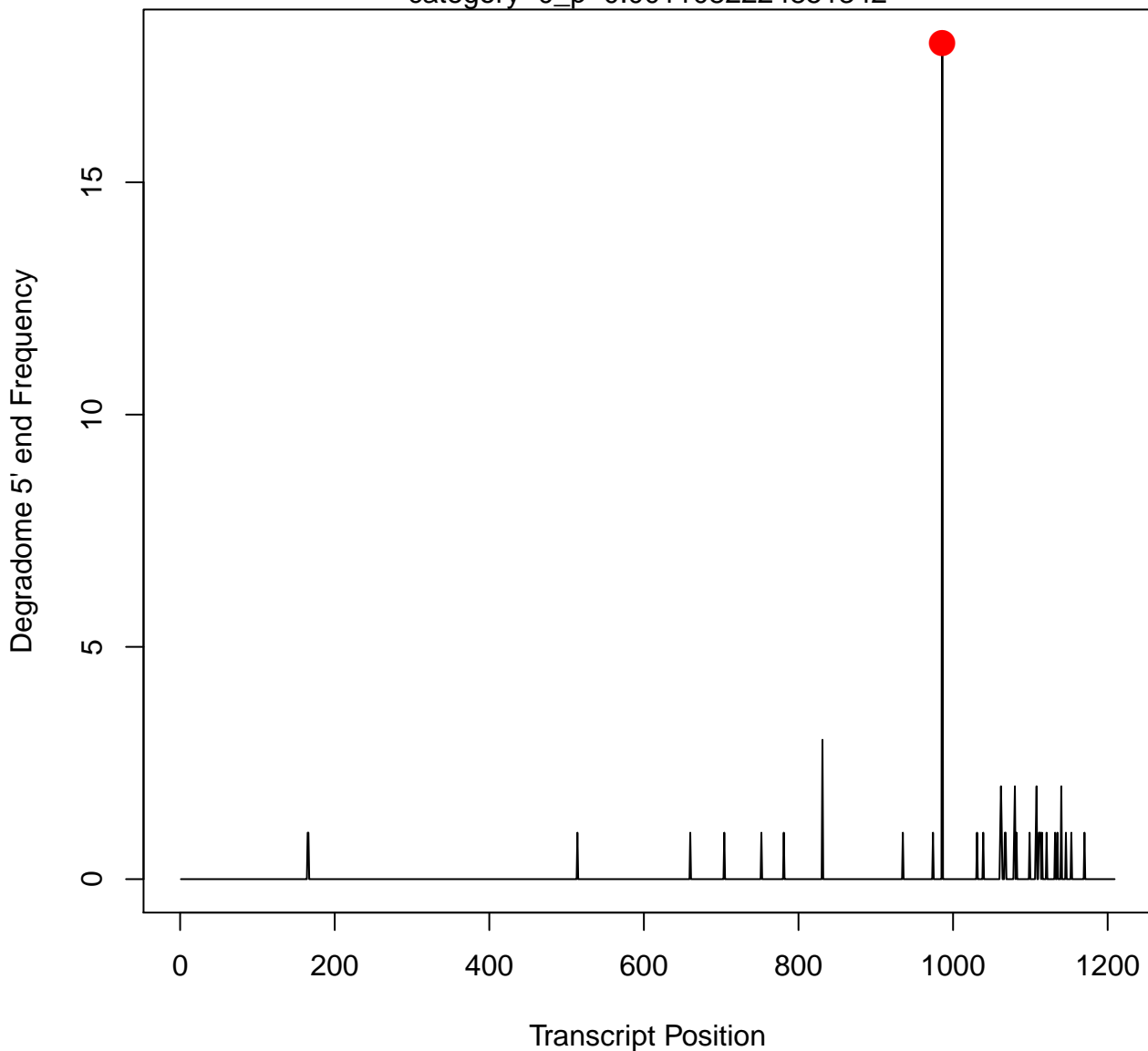

Supplement: Supplementary file 3 [file Data_Sheet_9.ZIP › GSM2230751.plot/Lsa-miR169a_Lsat_1_v5_gn_7_34841.1_986_TPlot.pdf]

**T=Lsat\_1\_v5\_gn\_3\_71621.1\_Q=Lsa-miR169e\_S=728**

category=2\_p=0.635524607752001

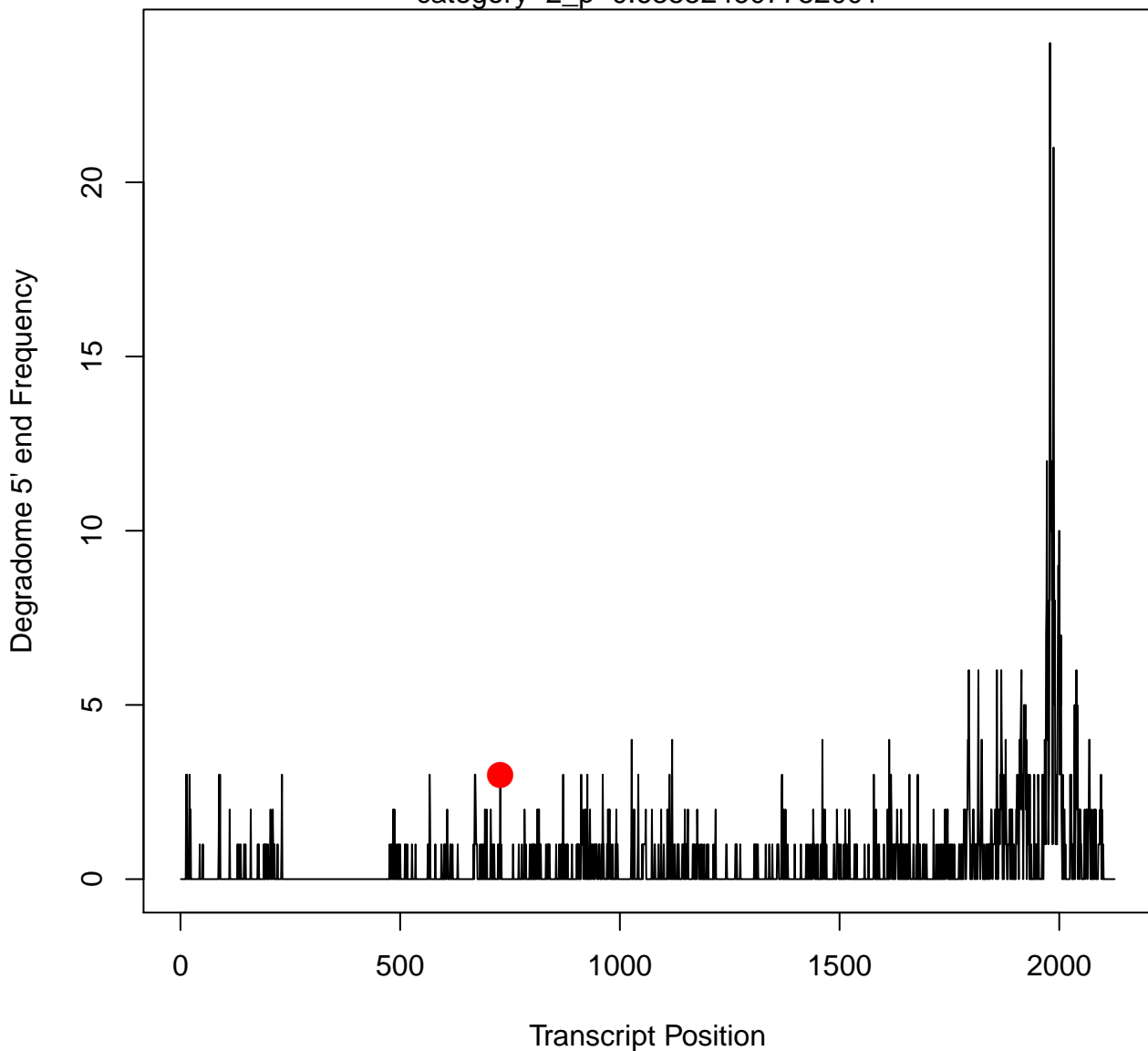

Supplement: Supplementary file 3 [file Data_Sheet_9.ZIP › GSM2230751.plot/Lsa-miR169e_Lsat_1_v5_gn_3_71621.1_728_TPlot.pdf]

**T=Lsat\_1\_v5\_gn\_4\_15920.1\_Q=Lsa-miR169e\_S=803**

category=2\_p=0.922146450335102

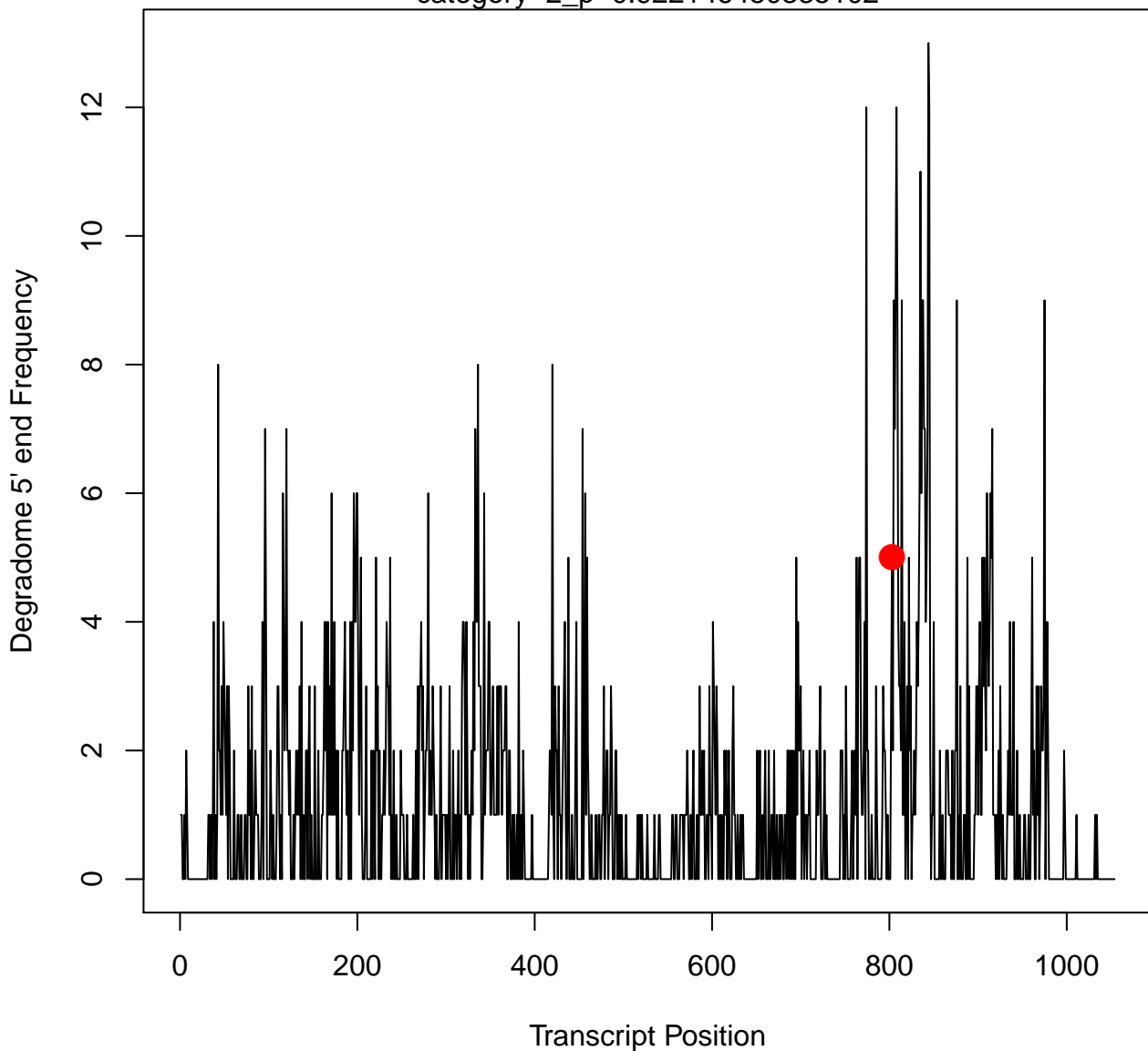

Supplement: Supplementary file 3 [file Data_Sheet_9.ZIP › GSM2230751.plot/Lsa-miR169e_Lsat_1_v5_gn_4_15920.1_803_TPlot.pdf]

**T=Lsat\_1\_v5\_gn\_3\_90900.1\_Q=Lsa-miR169g\_S=642**

category=2\_p=0.792643289216708

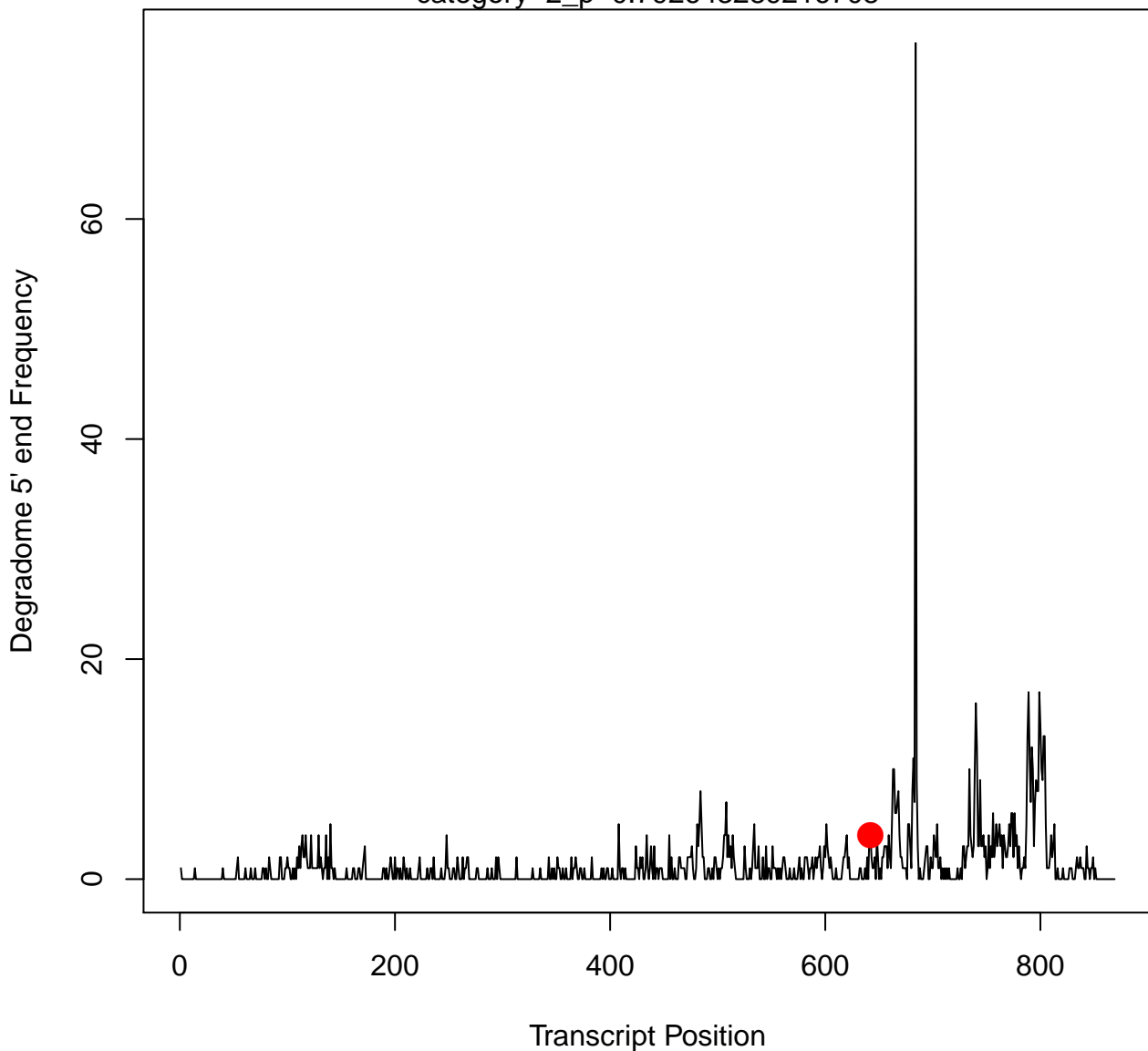

Supplement: Supplementary file 3 [file Data_Sheet_9.ZIP › GSM2230751.plot/Lsa-miR169g_Lsat_1_v5_gn_3_90900.1_642_TPlot.pdf]

**T=Lsat\_1\_v5\_gn\_5\_168921.1\_Q=Lsa-miR169g\_S=690**

category=2\_p=0.986490357849918

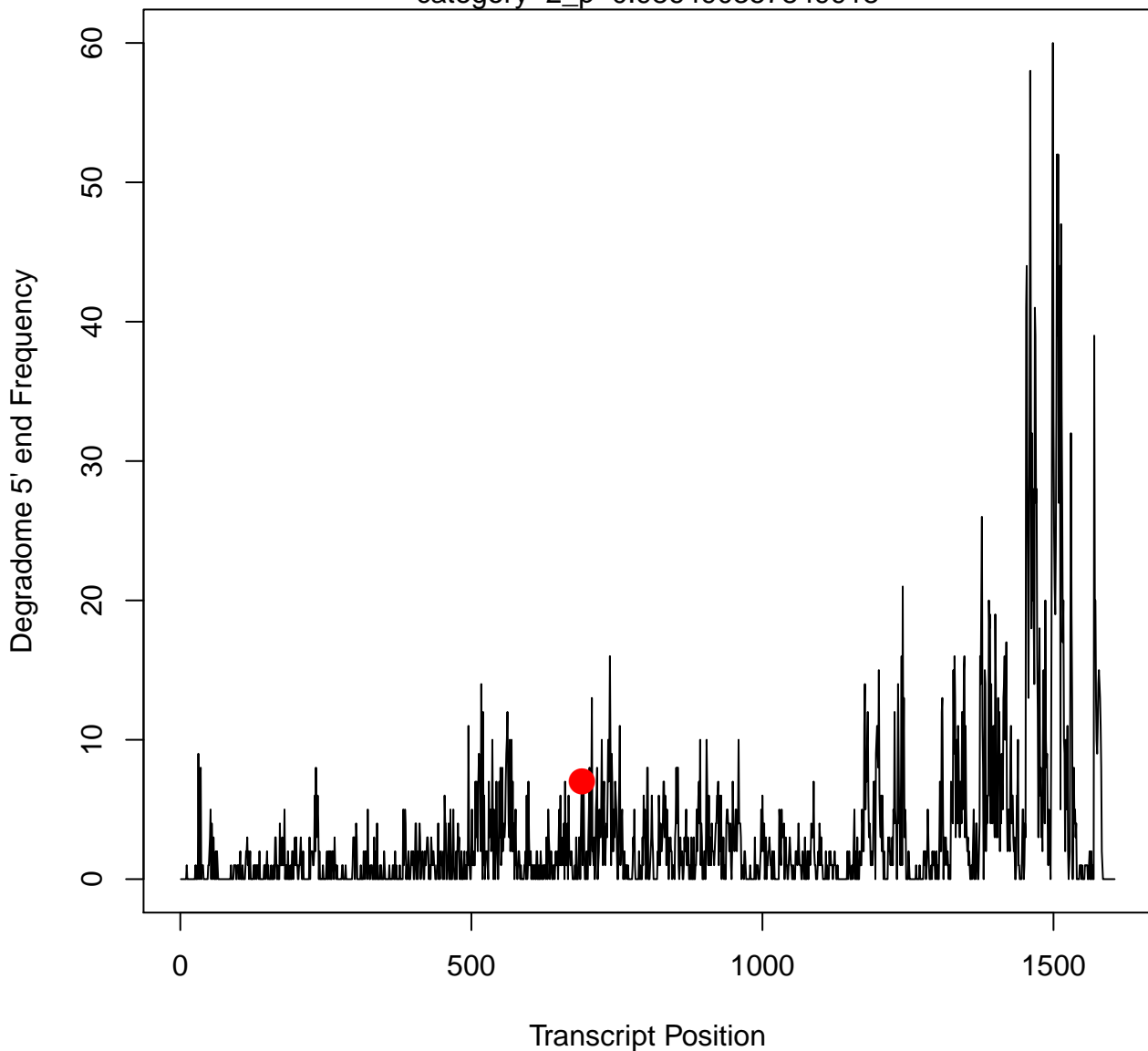

Supplement: Supplementary file 3 [file Data_Sheet_9.ZIP › GSM2230751.plot/Lsa-miR169g_Lsat_1_v5_gn_5_168921.1_690_TPlot.pdf]

**T=Lsat\_1\_v5\_gn\_7\_116120.1\_Q=Lsa-miR169h\_S=323**

category=2\_p=0.986083309887008

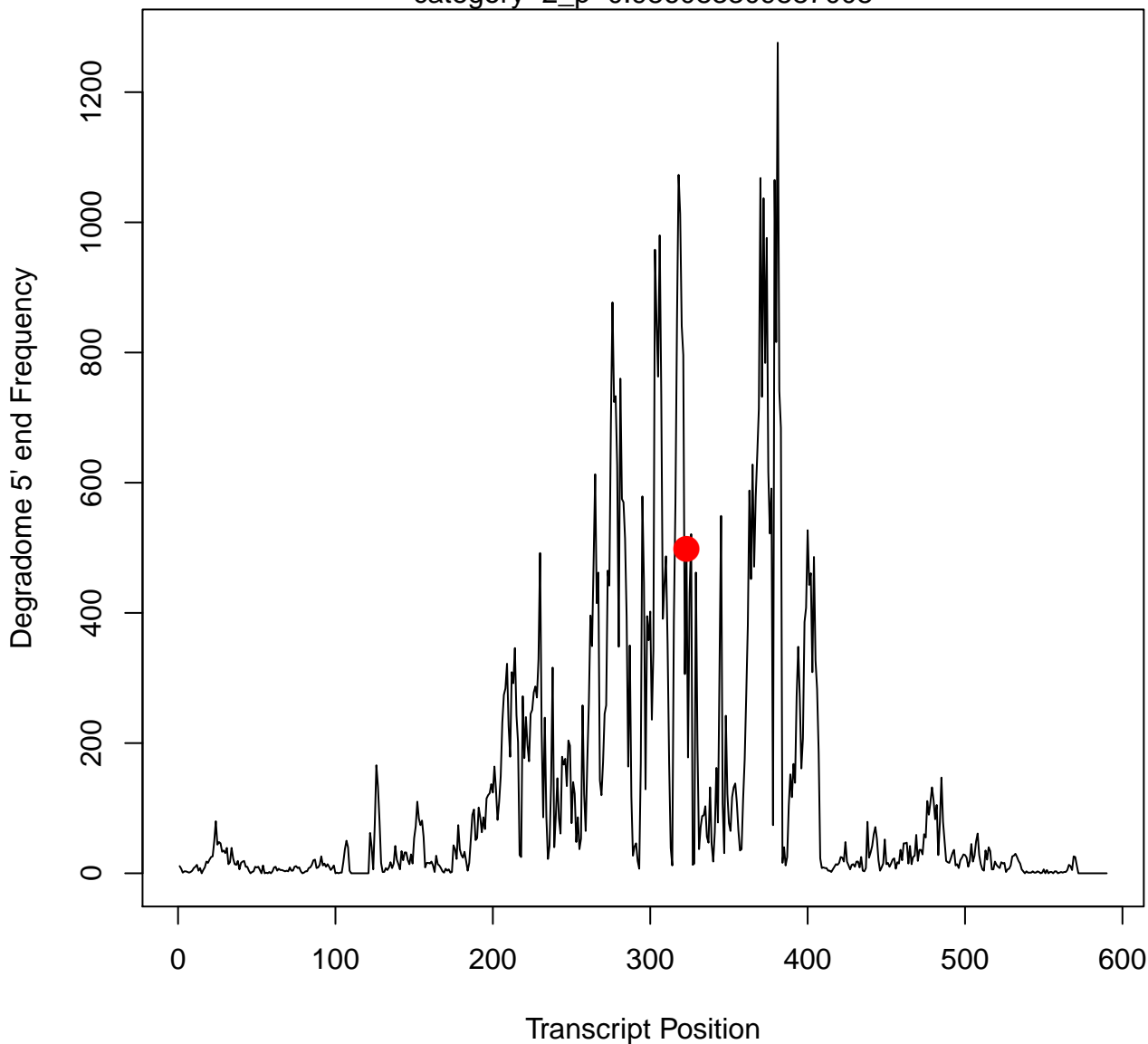

Supplement: Supplementary file 3 [file Data_Sheet_9.ZIP › GSM2230751.plot/Lsa-miR169h_Lsat_1_v5_gn_7_116120.1_323_TPlot.pdf]

**T=Lsat\_1\_v5\_gn\_8\_111520.1\_Q=Lsa-miR169h\_S=266**

category=2\_p=0.904165209342093

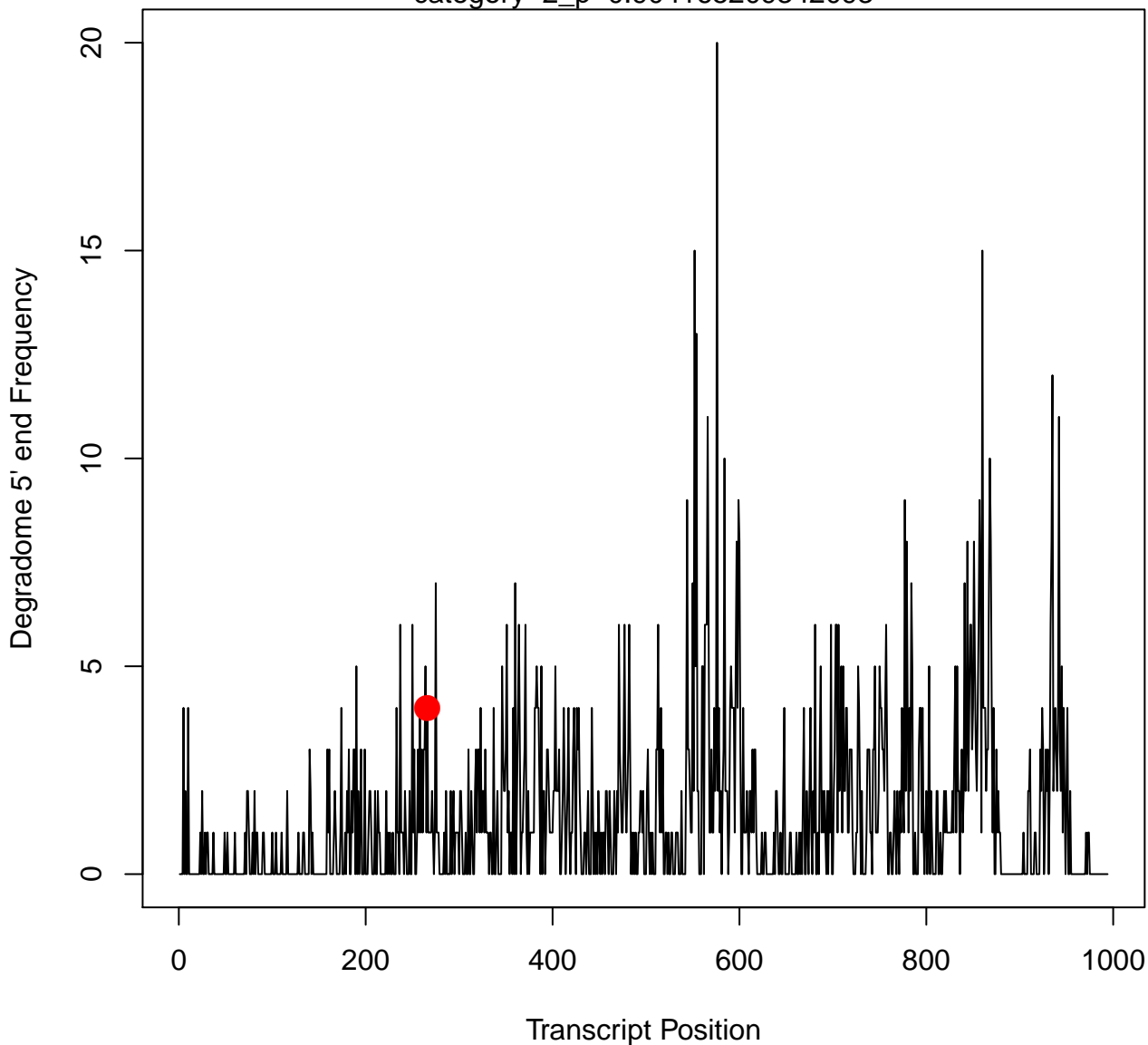

Supplement: Supplementary file 3 [file Data_Sheet_9.ZIP › GSM2230751.plot/Lsa-miR169h_Lsat_1_v5_gn_8_111520.1_266_TPlot.pdf]

**T=Lsat\_1\_v5\_gn\_1\_37160.1\_Q=Lsa-miR169i\_S=2396**

category=0\_p=0.000369544041020964

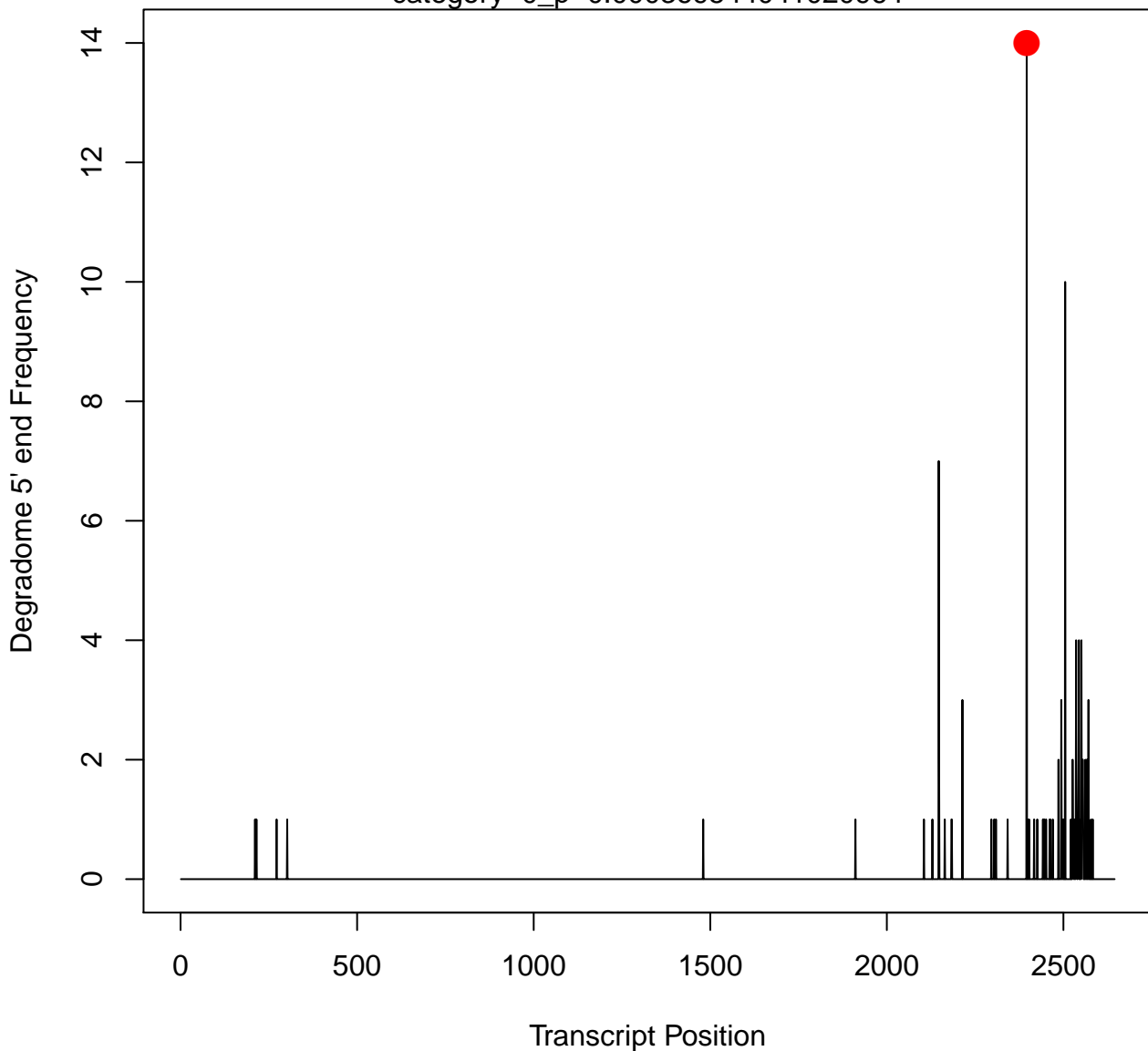

Supplement: Supplementary file 3 [file Data_Sheet_9.ZIP › GSM2230751.plot/Lsa-miR169i_Lsat_1_v5_gn_1_37160.1_2396_TPlot.pdf]

**T=Lsat\_1\_v5\_gn\_4\_9240.1\_Q=Lsa-miR169i\_S=236**

category=2\_p=0.163150556750859

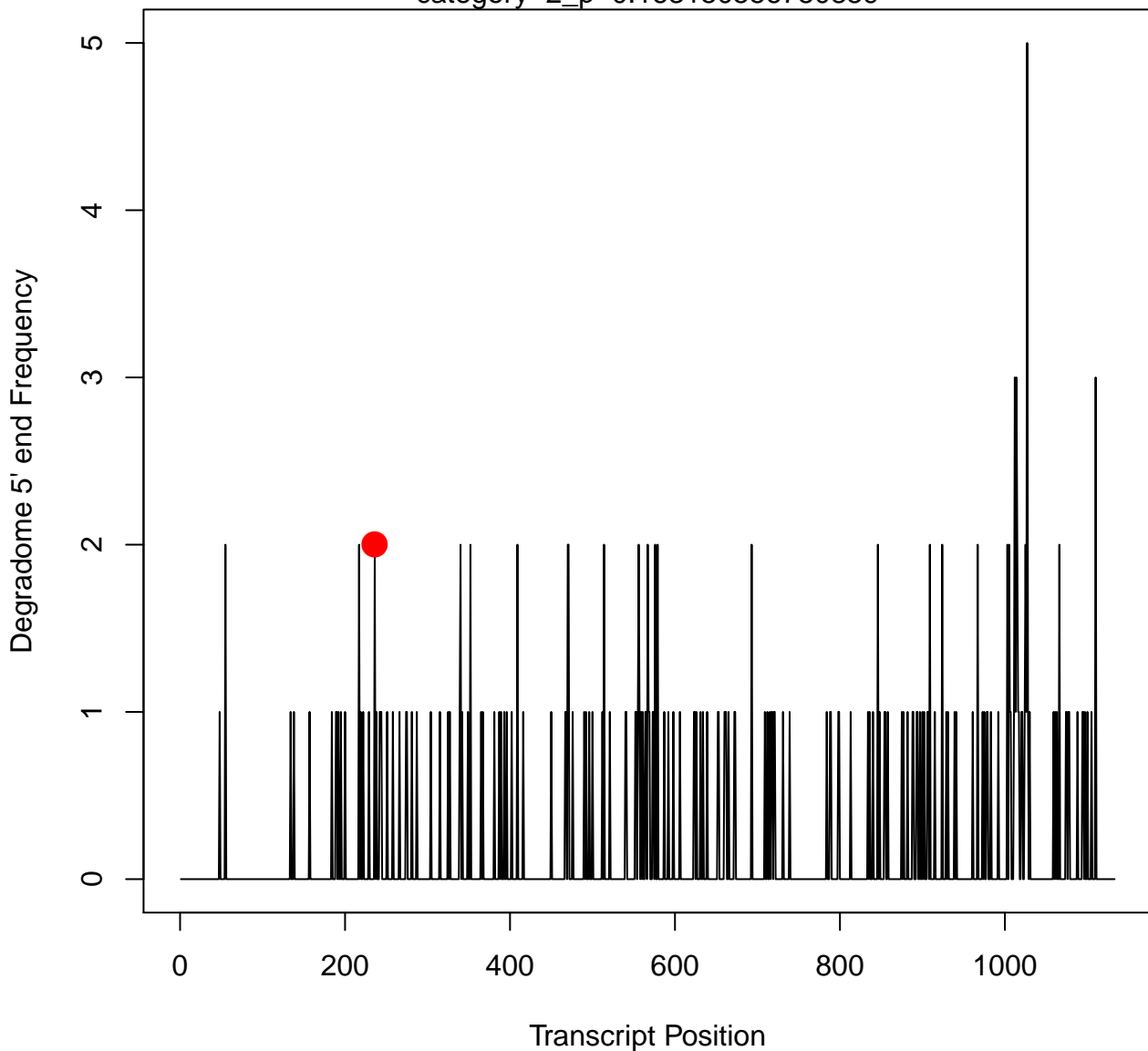

Supplement: Supplementary file 3 [file Data_Sheet_9.ZIP › GSM2230751.plot/Lsa-miR169i_Lsat_1_v5_gn_4_9240.1_236_TPlot.pdf]

**T=Lsat\_1\_v5\_gn\_6\_47121.1\_Q=Lsa-miR169i\_S=1633**

category=1\_p=0.00532089960997584

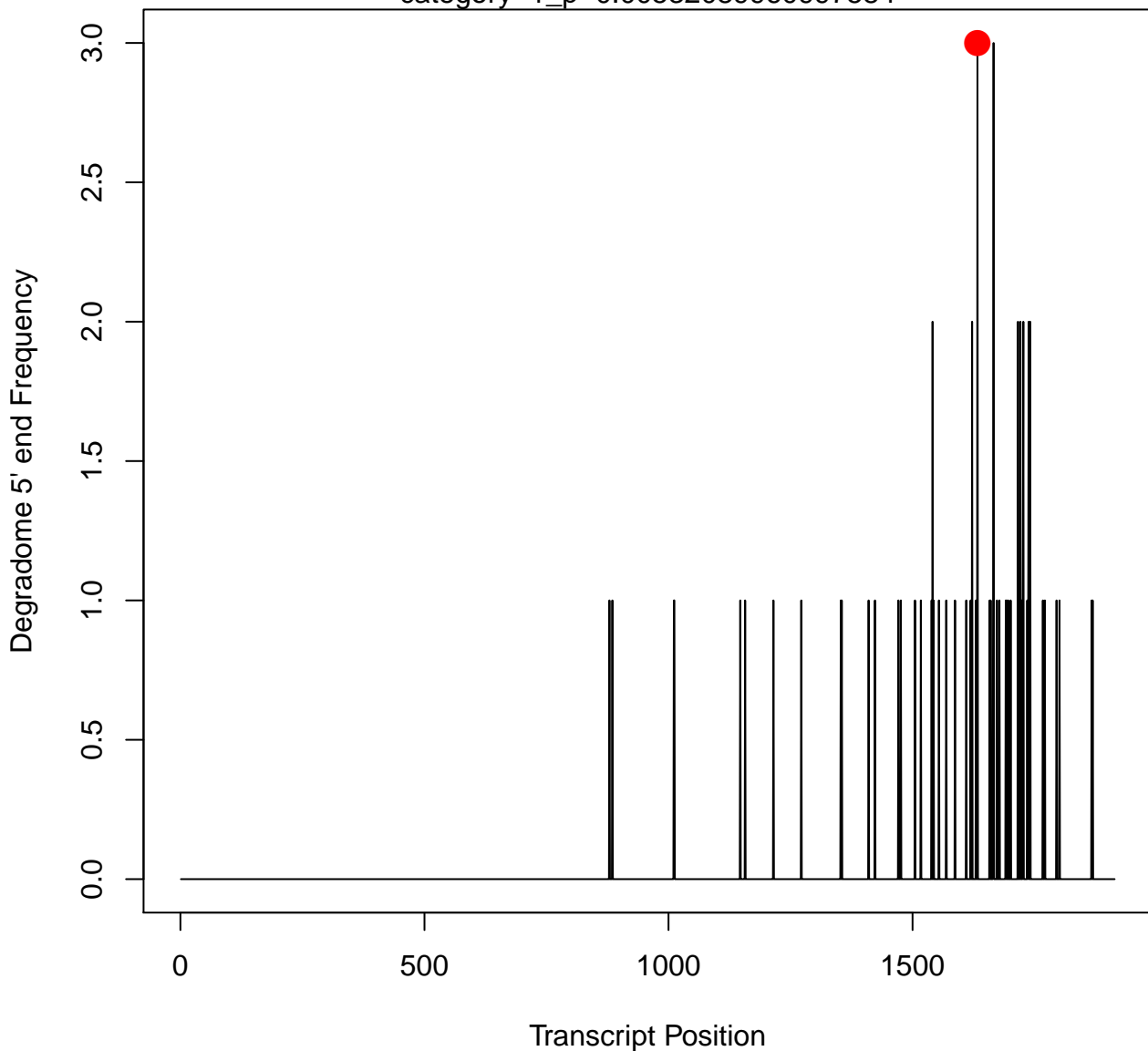

Supplement: Supplementary file 3 [file Data_Sheet_9.ZIP › GSM2230751.plot/Lsa-miR169i_Lsat_1_v5_gn_6_47121.1_1633_TPlot.pdf]

**T=Lsat\_1\_v5\_gn\_6\_97140.1\_Q=Lsa-miR169i\_S=825**

category=2\_p=0.676331998413925

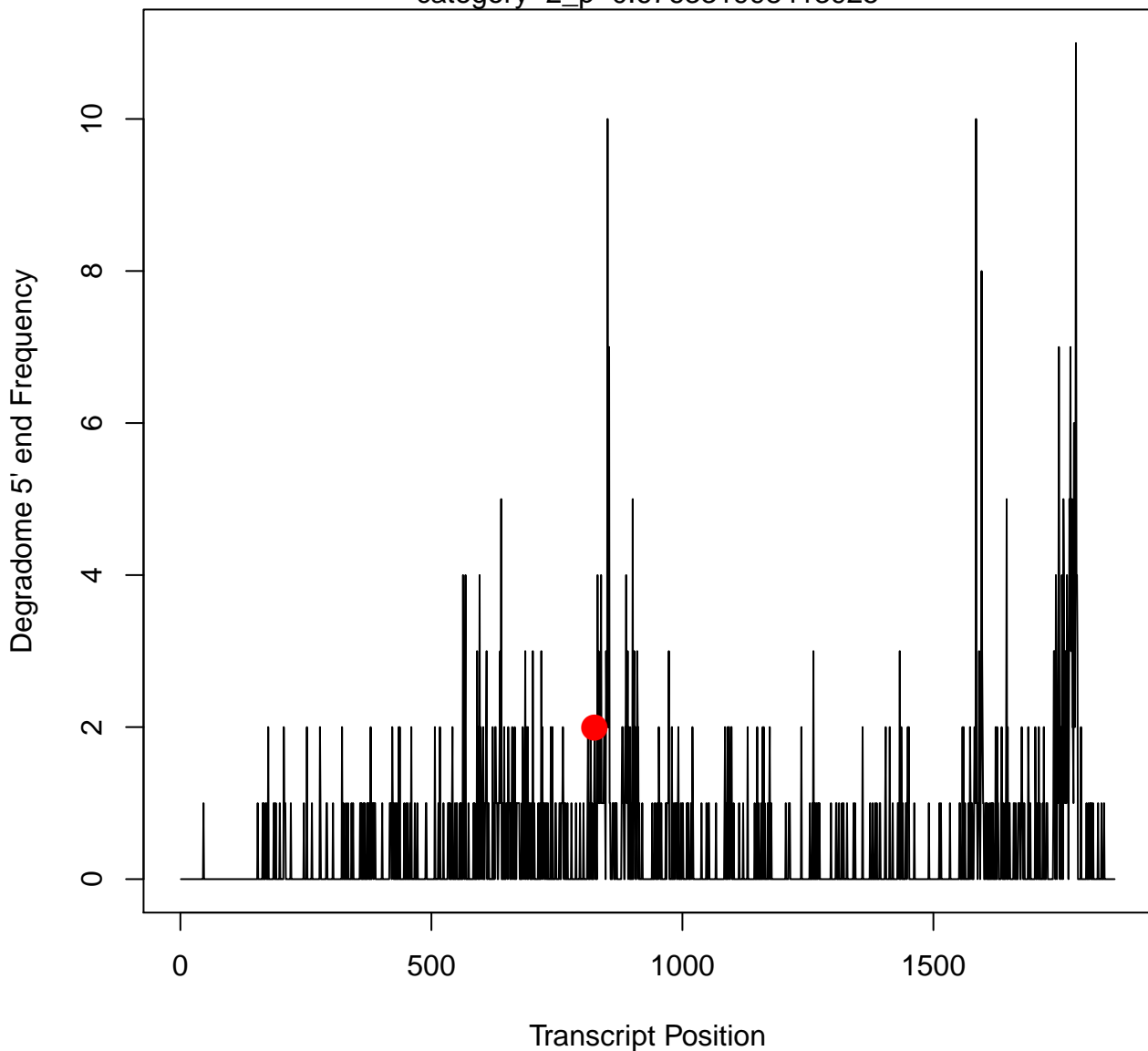

Supplement: Supplementary file 3 [file Data_Sheet_9.ZIP › GSM2230751.plot/Lsa-miR169i_Lsat_1_v5_gn_6_97140.1_825_TPlot.pdf]

**T=Lsat\_1\_v5\_gn\_1\_27961.1\_Q=Lsa-miR171a\_S=2558**

category=2\_p=0.971624296947987

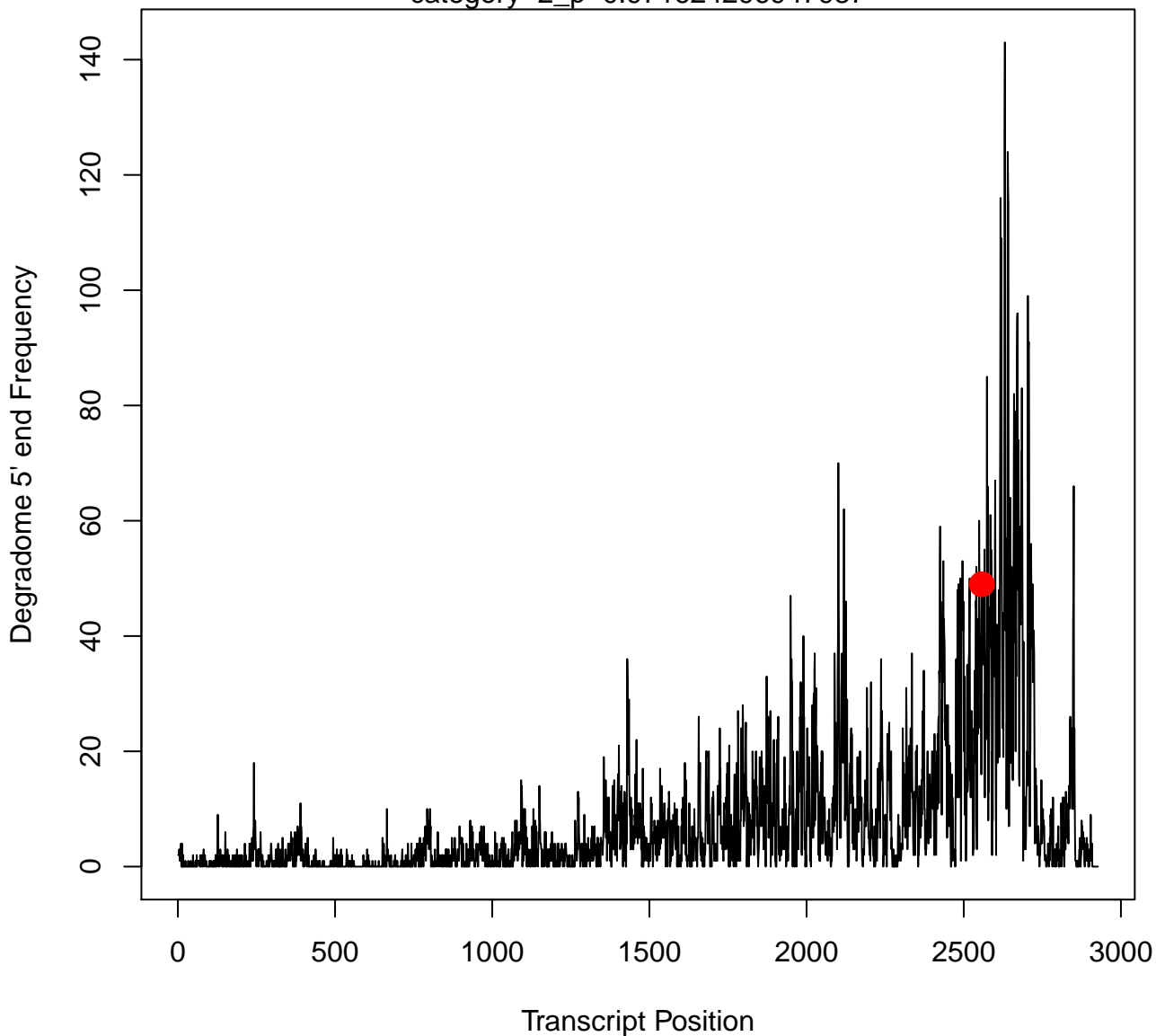

Supplement: Supplementary file 3 [file Data_Sheet_9.ZIP › GSM2230751.plot/Lsa-miR171a_Lsat_1_v5_gn_1_27961.1_2558_TPlot.pdf]

T=Lsat\_1\_v5\_gn\_3\_128721.1\_Q=Lsa-miR171a\_S=47

category=0\_p=0.000738951519243747

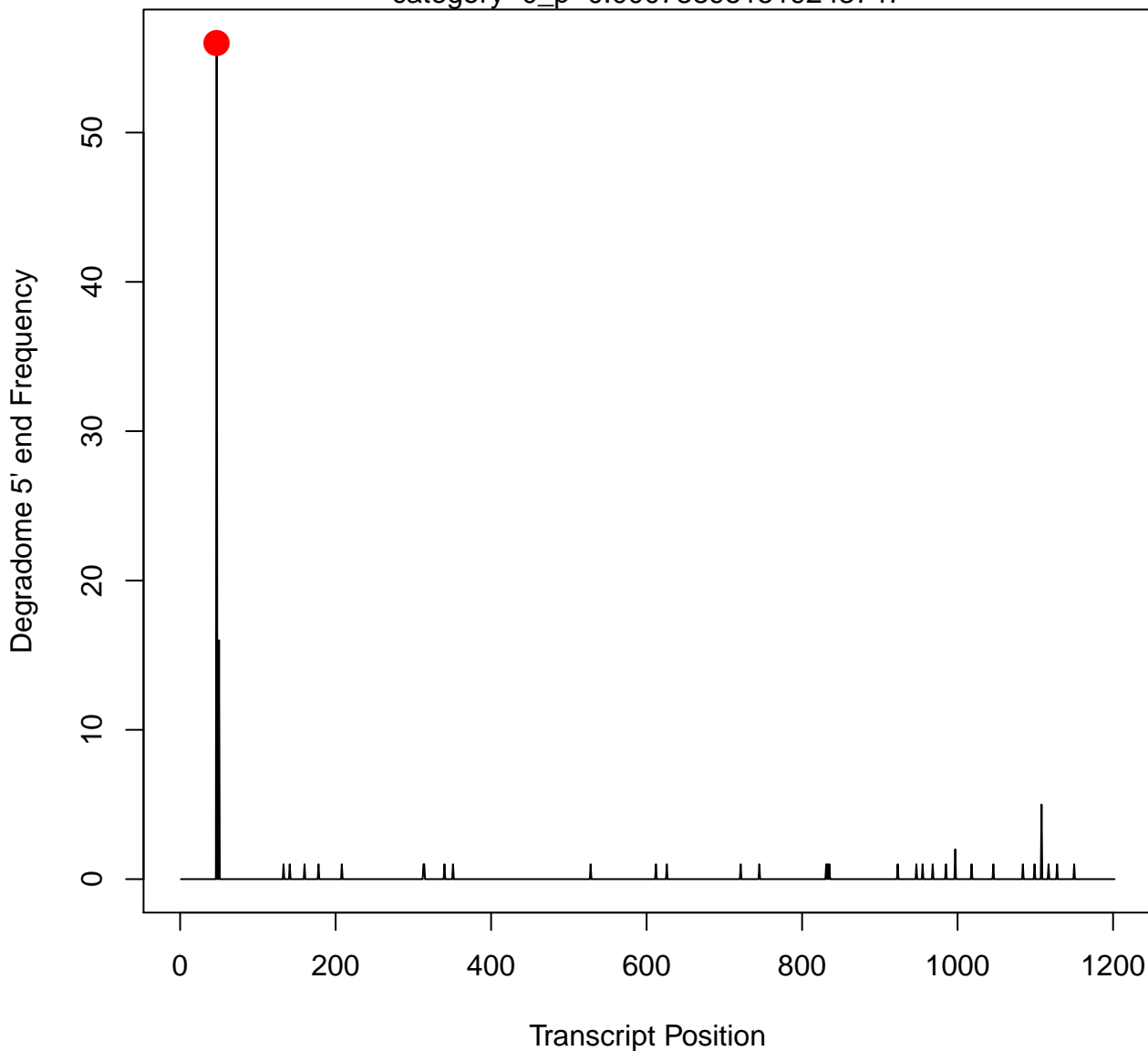

Supplement: Supplementary file 3 [file Data_Sheet_9.ZIP › GSM2230751.plot/Lsa-miR171a_Lsat_1_v5_gn_3_128721.1_47_TPlot.pdf]

**T=Lsat\_1\_v5\_gn\_4\_109141.1\_Q=Lsa-miR171a\_S=1369**

category=2\_p=0.703910209880845

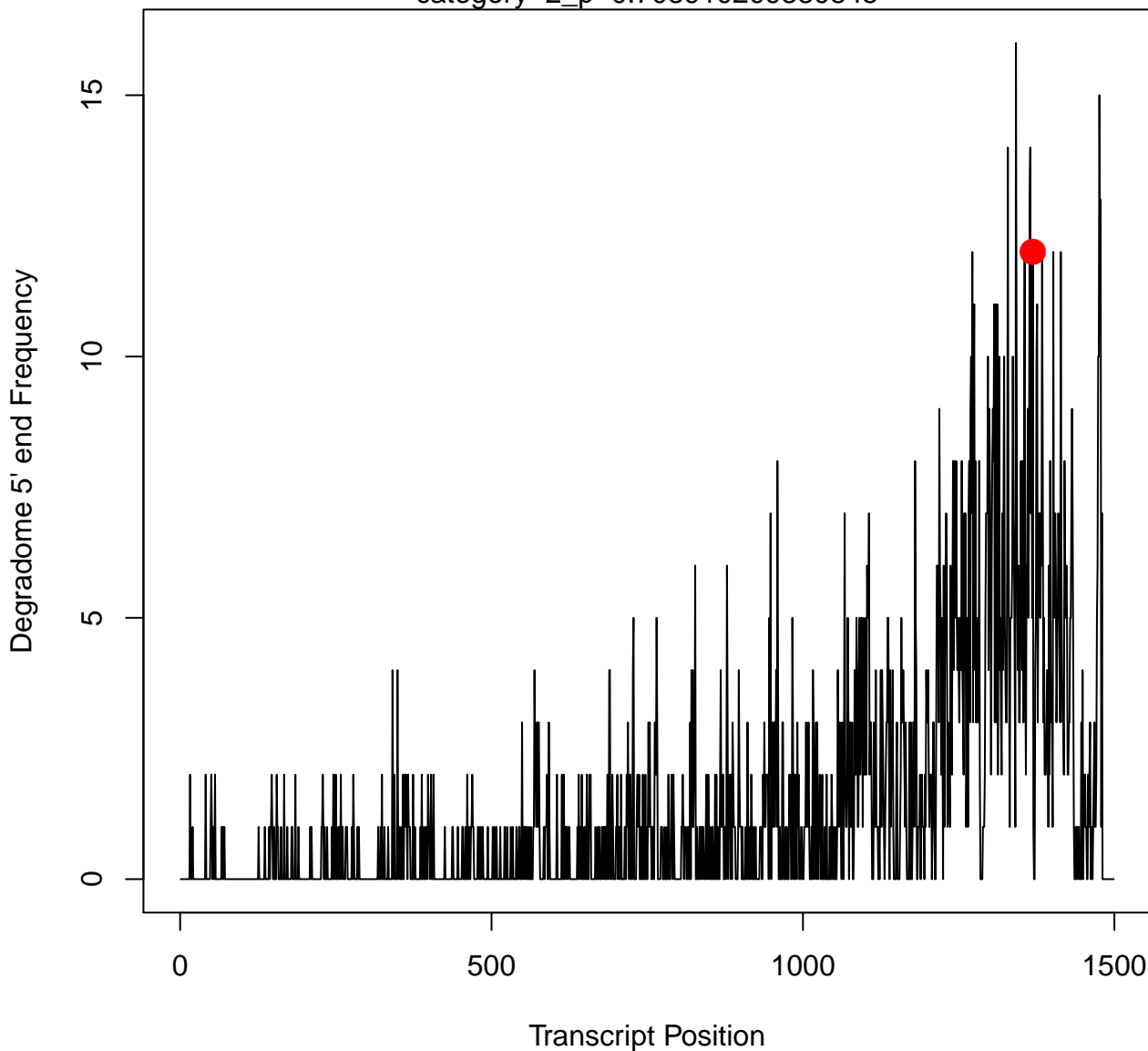

Supplement: Supplementary file 3 [file Data_Sheet_9.ZIP › GSM2230751.plot/Lsa-miR171a_Lsat_1_v5_gn_4_109141.1_1369_TPlot.pdf]

**T=Lsat\_1\_v5\_gn\_5\_174381.1\_Q=Lsa-miR171a\_S=873**

category=2\_p=0.256845886757239

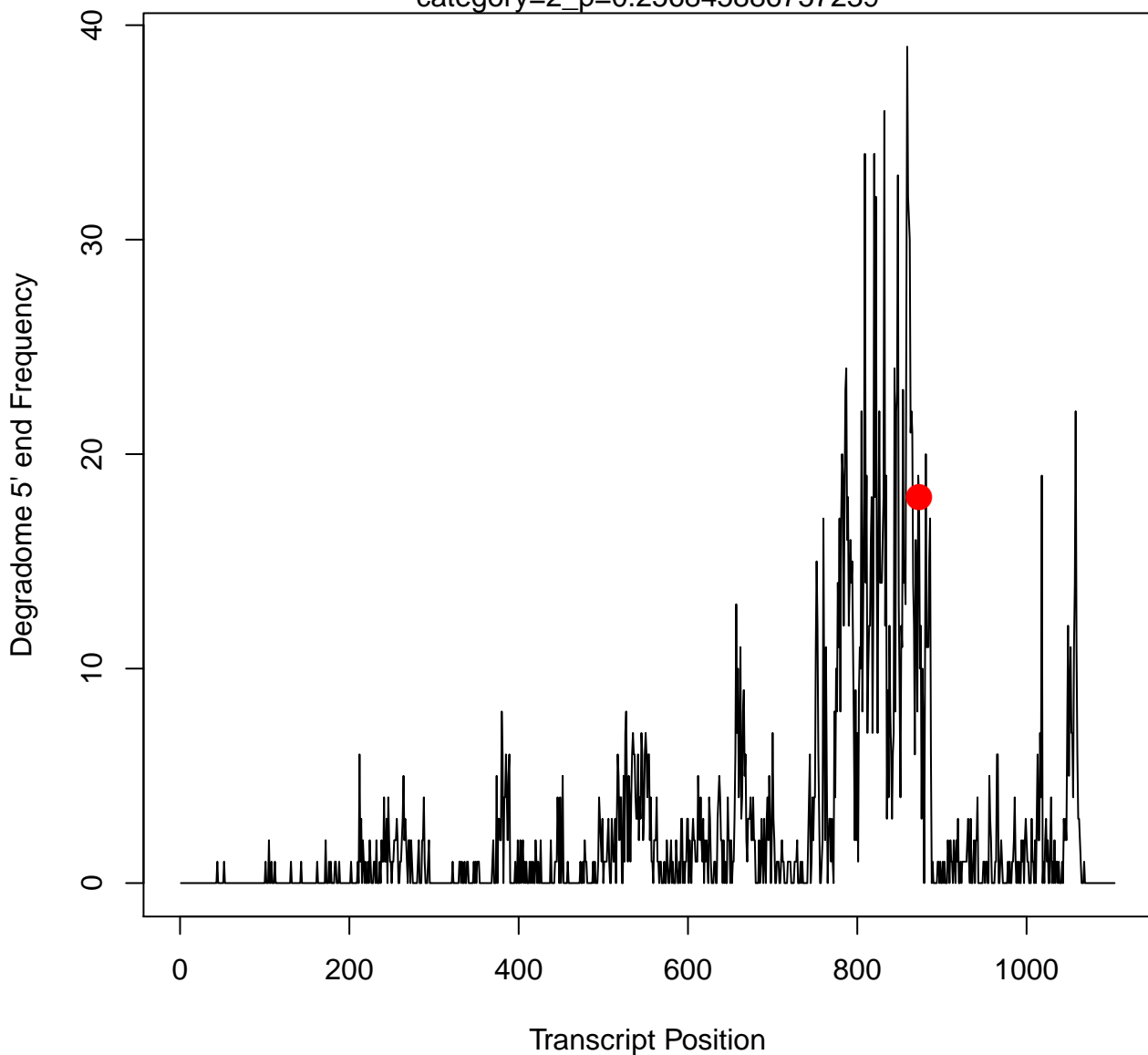

Supplement: Supplementary file 3 [file Data_Sheet_9.ZIP › GSM2230751.plot/Lsa-miR171a_Lsat_1_v5_gn_5_174381.1_873_TPlot.pdf]

**T=Lsat\_1\_v5\_gn\_7\_45260.1\_Q=Lsa-miR171a\_S=768**

category=2\_p=0.940399566861131

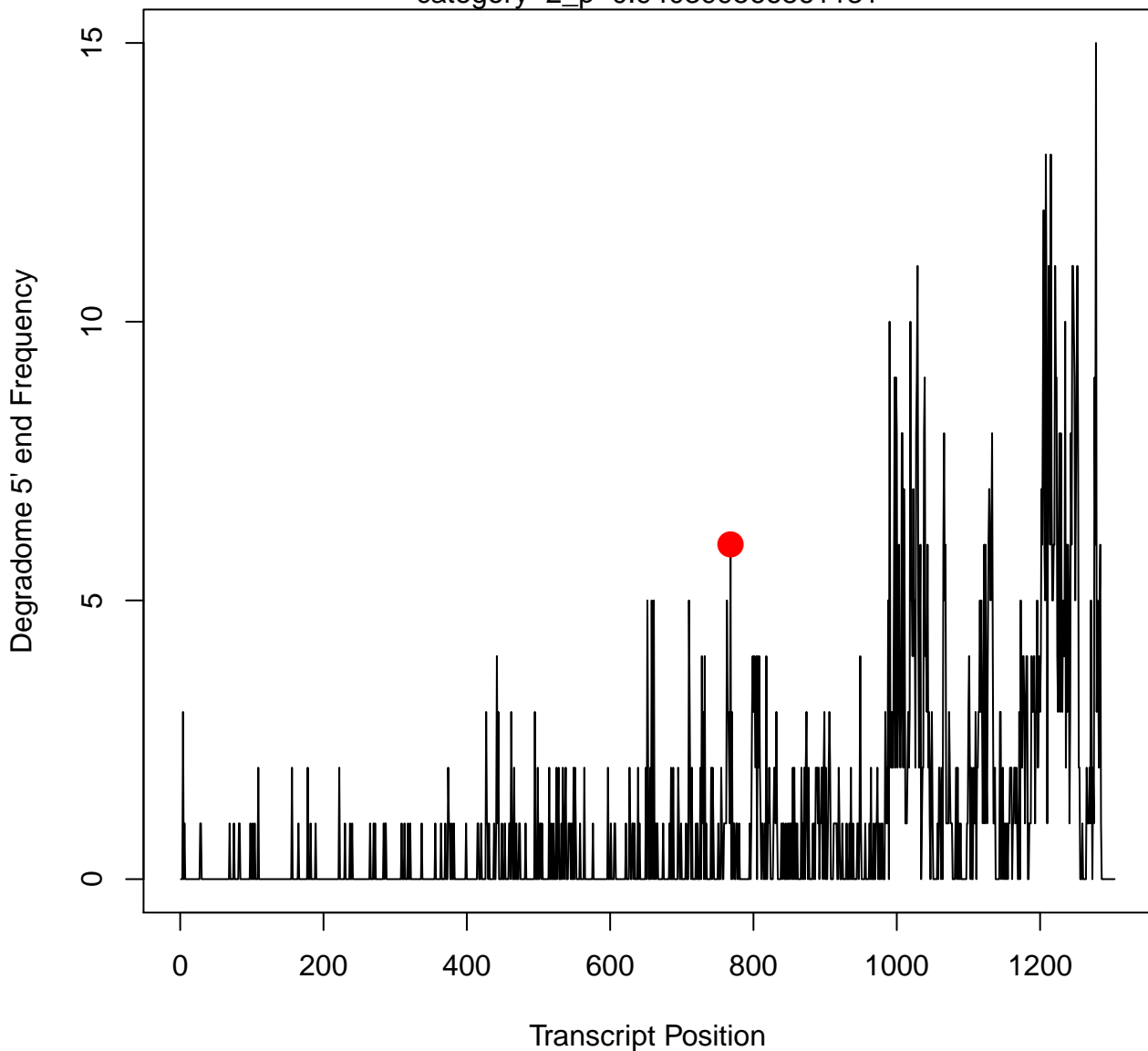

Supplement: Supplementary file 3 [file Data_Sheet_9.ZIP › GSM2230751.plot/Lsa-miR171a_Lsat_1_v5_gn_7_45260.1_768_TPlot.pdf]

**T=Lsat\_1\_v5\_gn\_9\_89160.1\_Q=Lsa-miR171a\_S=762**

category=2\_p=0.613230307796578

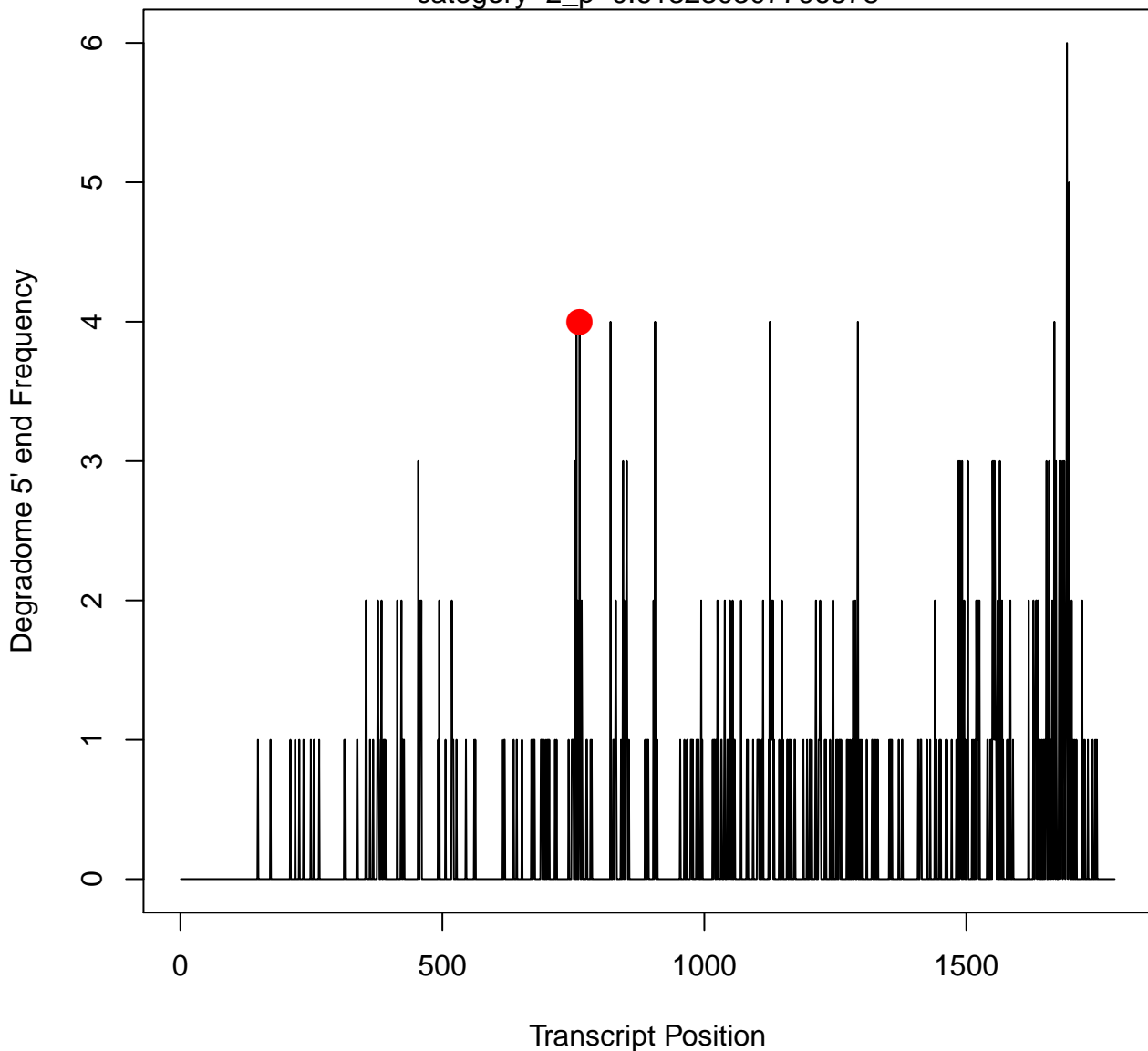

Supplement: Supplementary file 3 [file Data_Sheet_9.ZIP › GSM2230751.plot/Lsa-miR171a_Lsat_1_v5_gn_9_89160.1_762_TPlot.pdf]

**T=Lsat\_1\_v5\_gn\_1\_27961.1\_Q=Lsa-miR171b\_S=2561**

category=2\_p=0.537826433029871

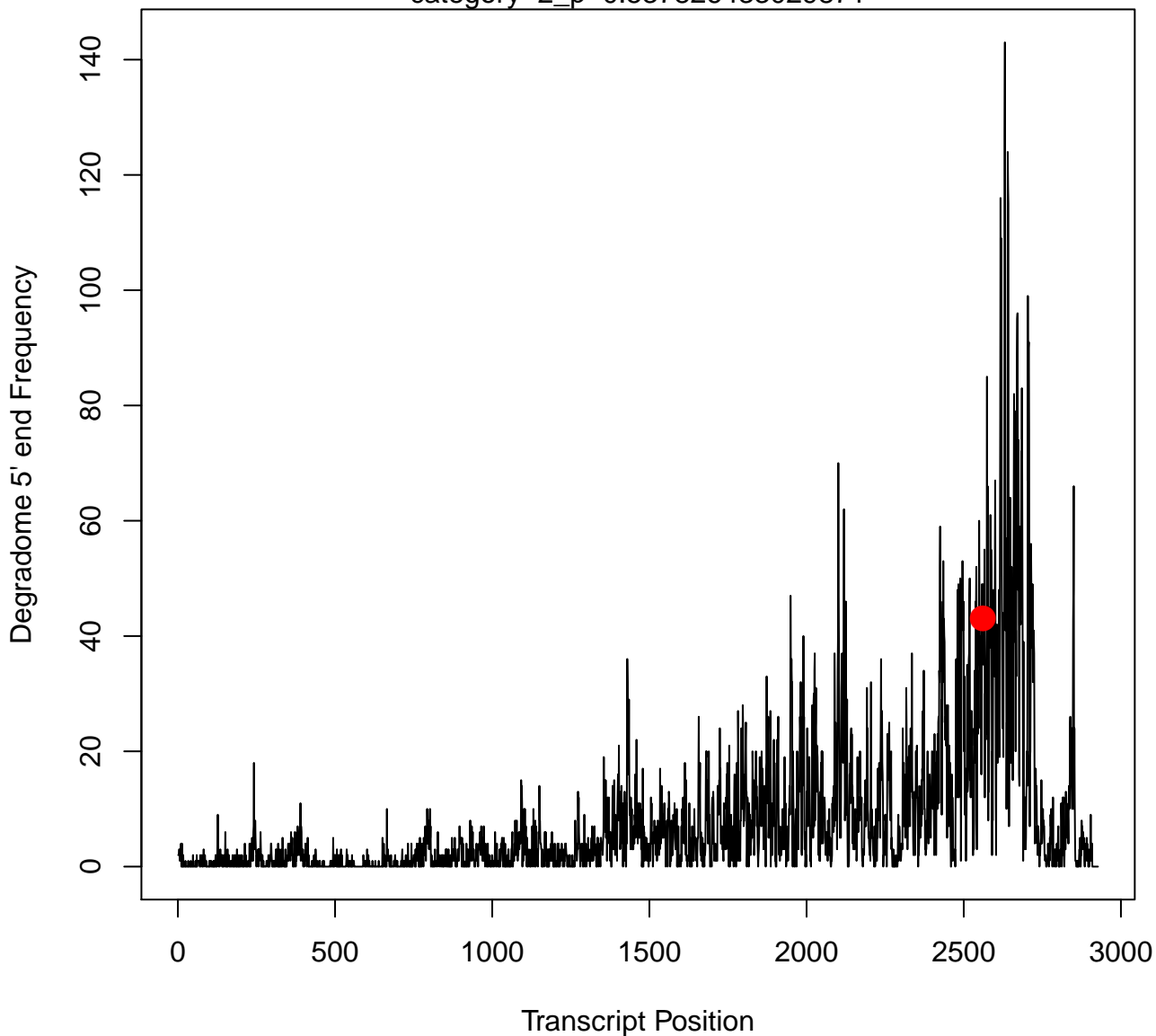

Supplement: Supplementary file 3 [file Data_Sheet_9.ZIP › GSM2230751.plot/Lsa-miR171b_Lsat_1_v5_gn_1_27961.1_2561_TPlot.pdf]

**T=Lsat\_1\_v5\_gn\_3\_128721.1\_Q=Lsa-miR171b\_S=50**

category=2\_p=0.0852052452876977

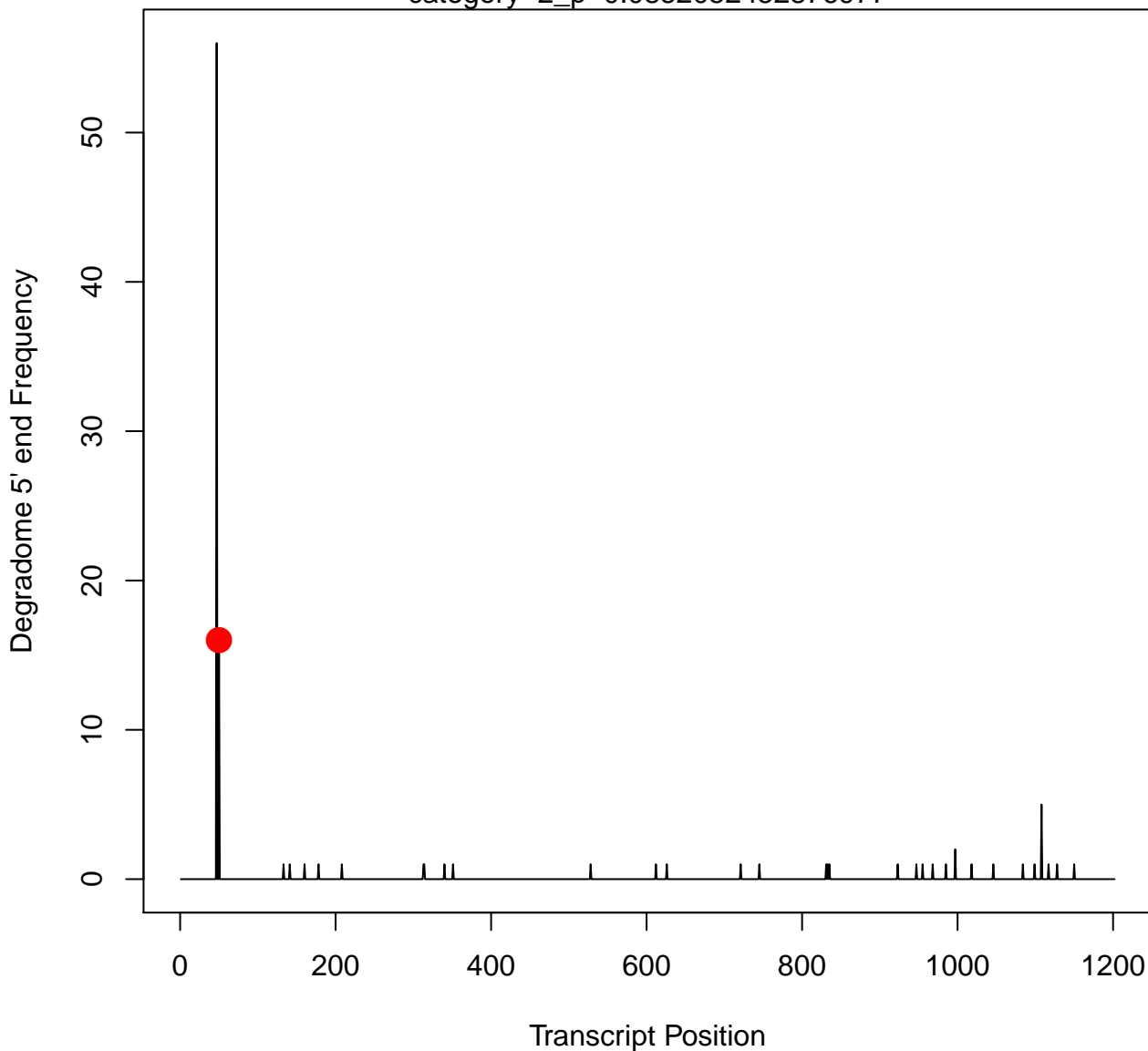

Supplement: Supplementary file 3 [file Data_Sheet_9.ZIP › GSM2230751.plot/Lsa-miR171b_Lsat_1_v5_gn_3_128721.1_50_TPlot.pdf]

**T=Lsat\_1\_v5\_gn\_3\_681.1\_Q=Lsa-miR171b\_S=716**

category=0\_p=0.000738951519243747

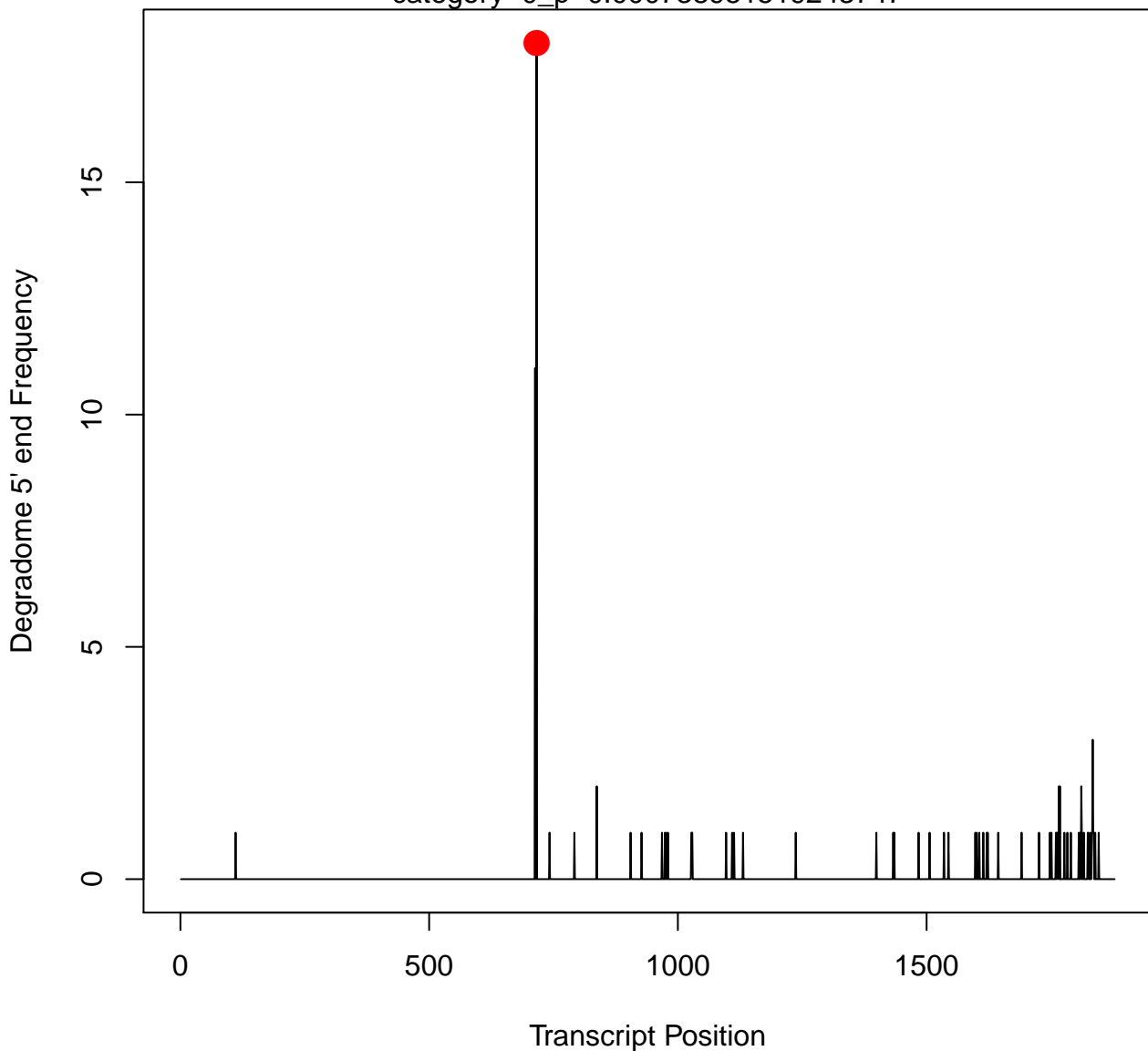

Supplement: Supplementary file 3 [file Data_Sheet_9.ZIP › GSM2230751.plot/Lsa-miR171b_Lsat_1_v5_gn_3_681.1_716_TPlot.pdf]

**T=Lsat\_1\_v5\_gn\_4\_153400.1\_Q=Lsa-miR171b\_S=648**

category=2\_p=0.163150556750859

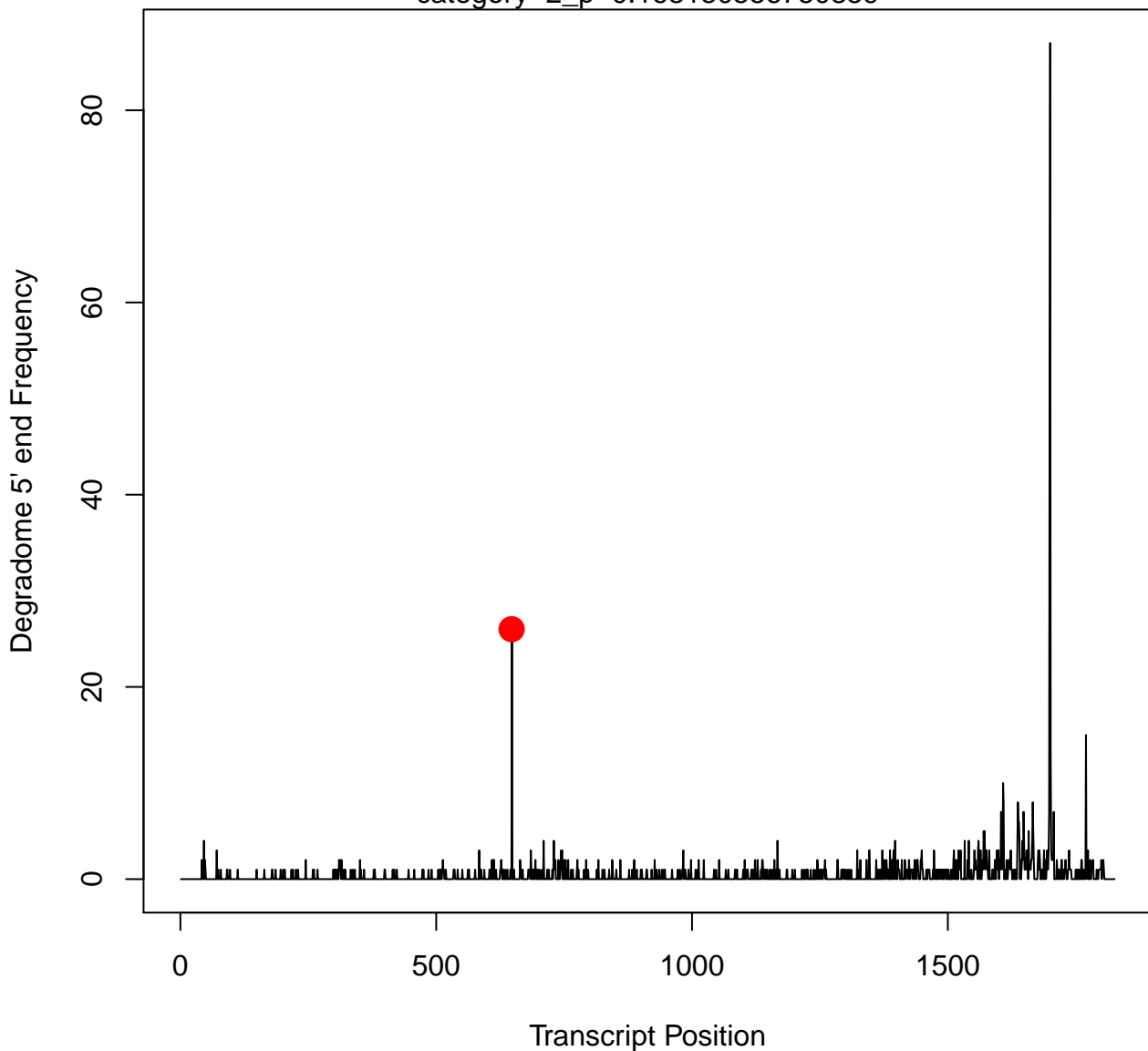

Supplement: Supplementary file 3 [file Data_Sheet_9.ZIP › GSM2230751.plot/Lsa-miR171b_Lsat_1_v5_gn_4_153400.1_648_TPlot.pdf]

**T=Lsat\_1\_v5\_gn\_5\_71000.1\_Q=Lsa-miR171b\_S=1628**

category=1\_p=0.0439105553323189

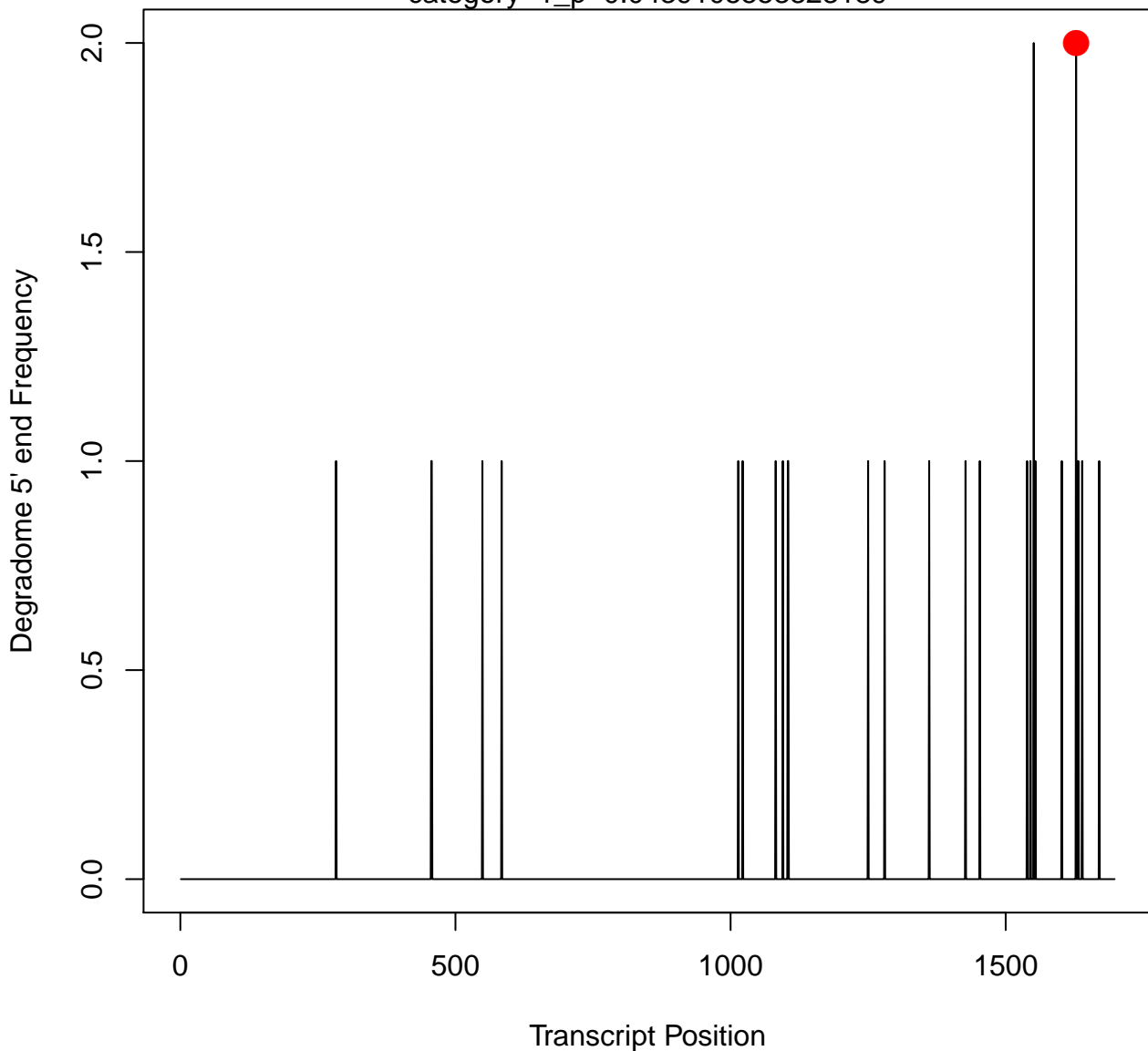

Supplement: Supplementary file 3 [file Data_Sheet_9.ZIP › GSM2230751.plot/Lsa-miR171b_Lsat_1_v5_gn_5_71000.1_1628_TPlot.pdf]

**T=Lsat\_1\_v5\_gn\_5\_71020.1\_Q=Lsa-miR171b\_S=1586**

category=1\_p=0.0460335441912159

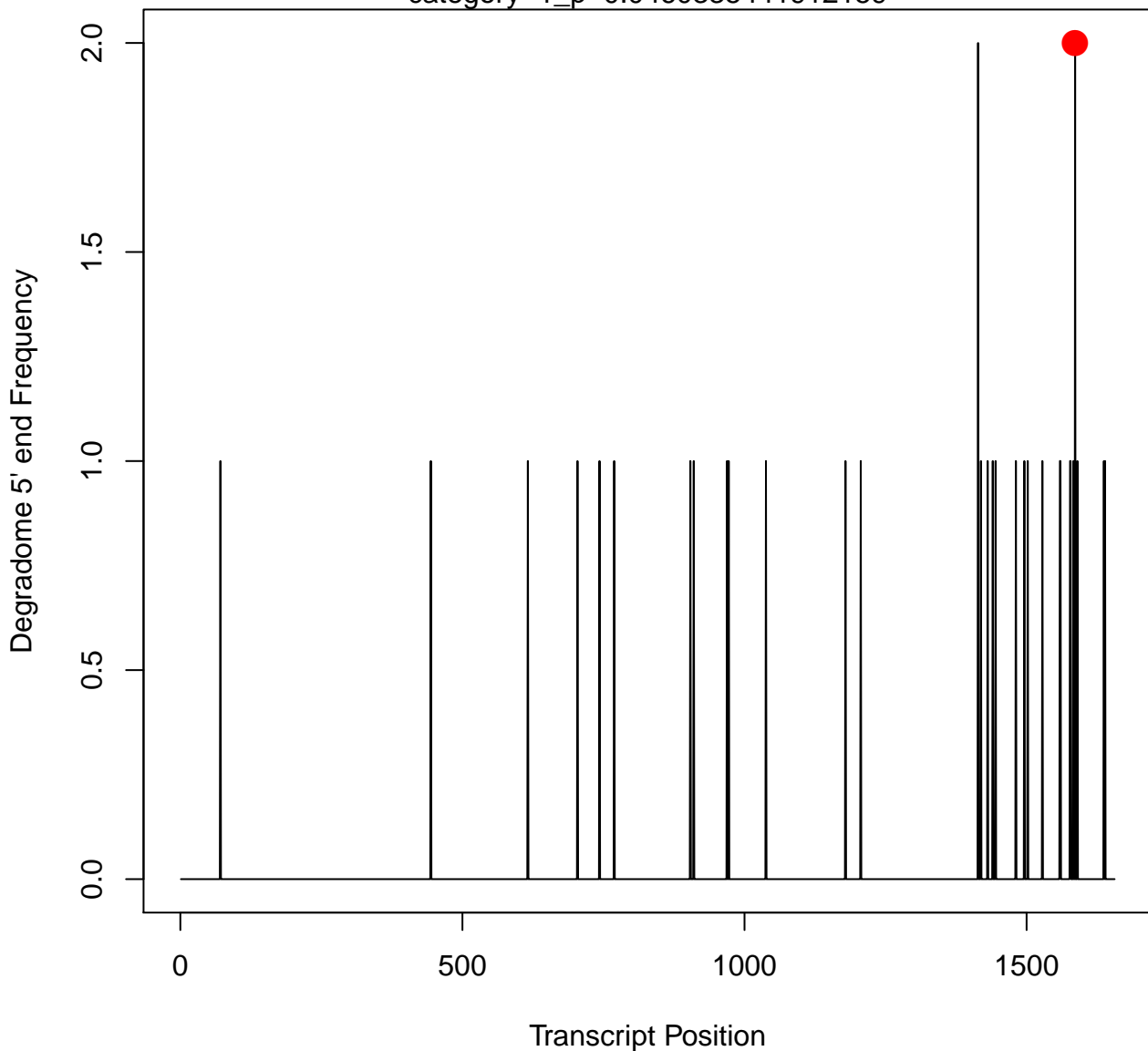

Supplement: Supplementary file 3 [file Data_Sheet_9.ZIP › GSM2230751.plot/Lsa-miR171b_Lsat_1_v5_gn_5_71020.1_1586_TPlot.pdf]

**T=Lsat\_1\_v5\_gn\_6\_3180.1\_Q=Lsa-miR171b\_S=1761**

category=0\_p=0.00147735698913964

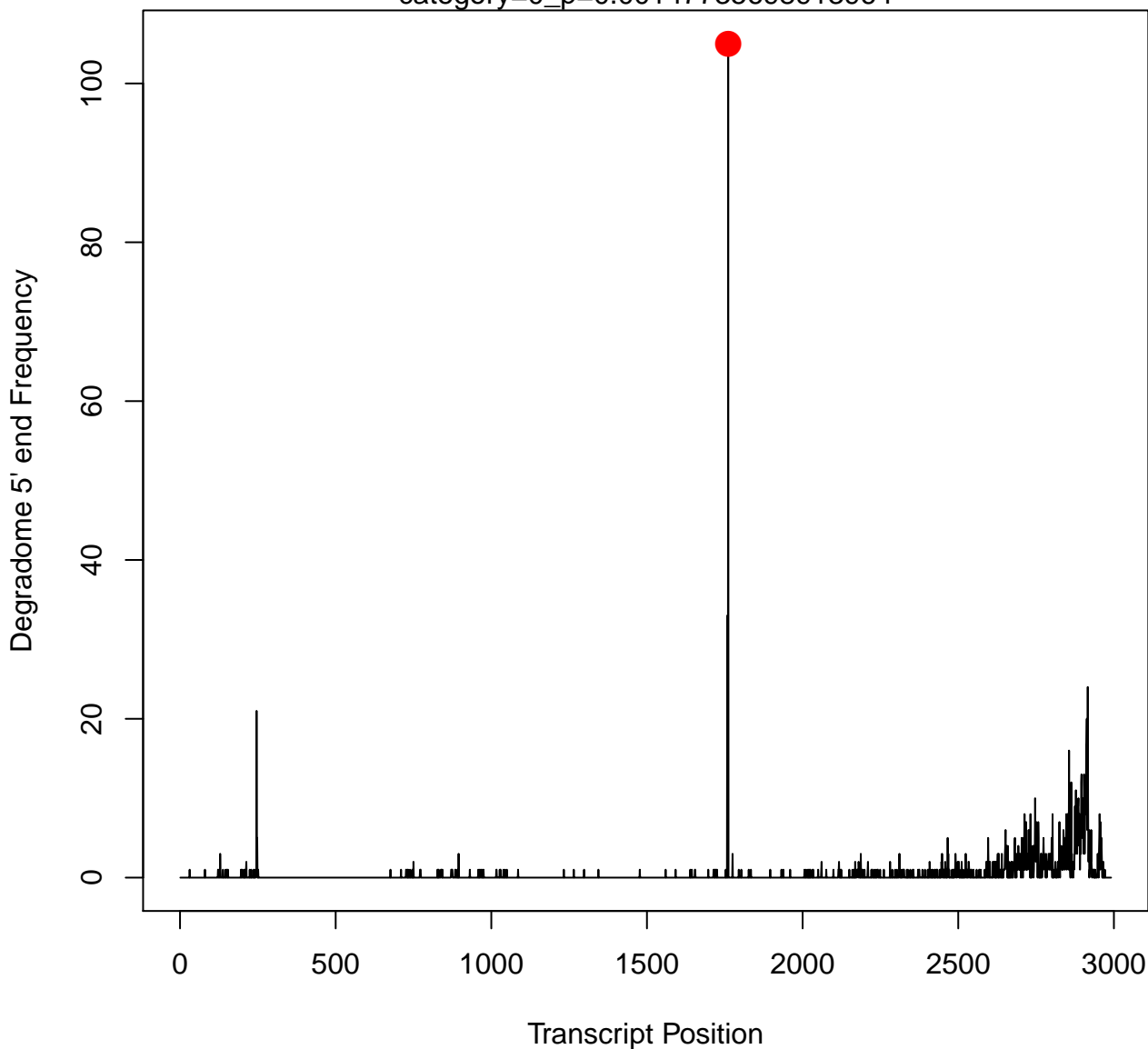

Supplement: Supplementary file 3 [file Data_Sheet_9.ZIP › GSM2230751.plot/Lsa-miR171b_Lsat_1_v5_gn_6_3180.1_1761_TPlot.pdf]

**T=Lsat\_1\_v5\_gn\_7\_9261.1\_Q=Lsa-miR171b\_S=1167**

category=0\_p=0.000369544041020964

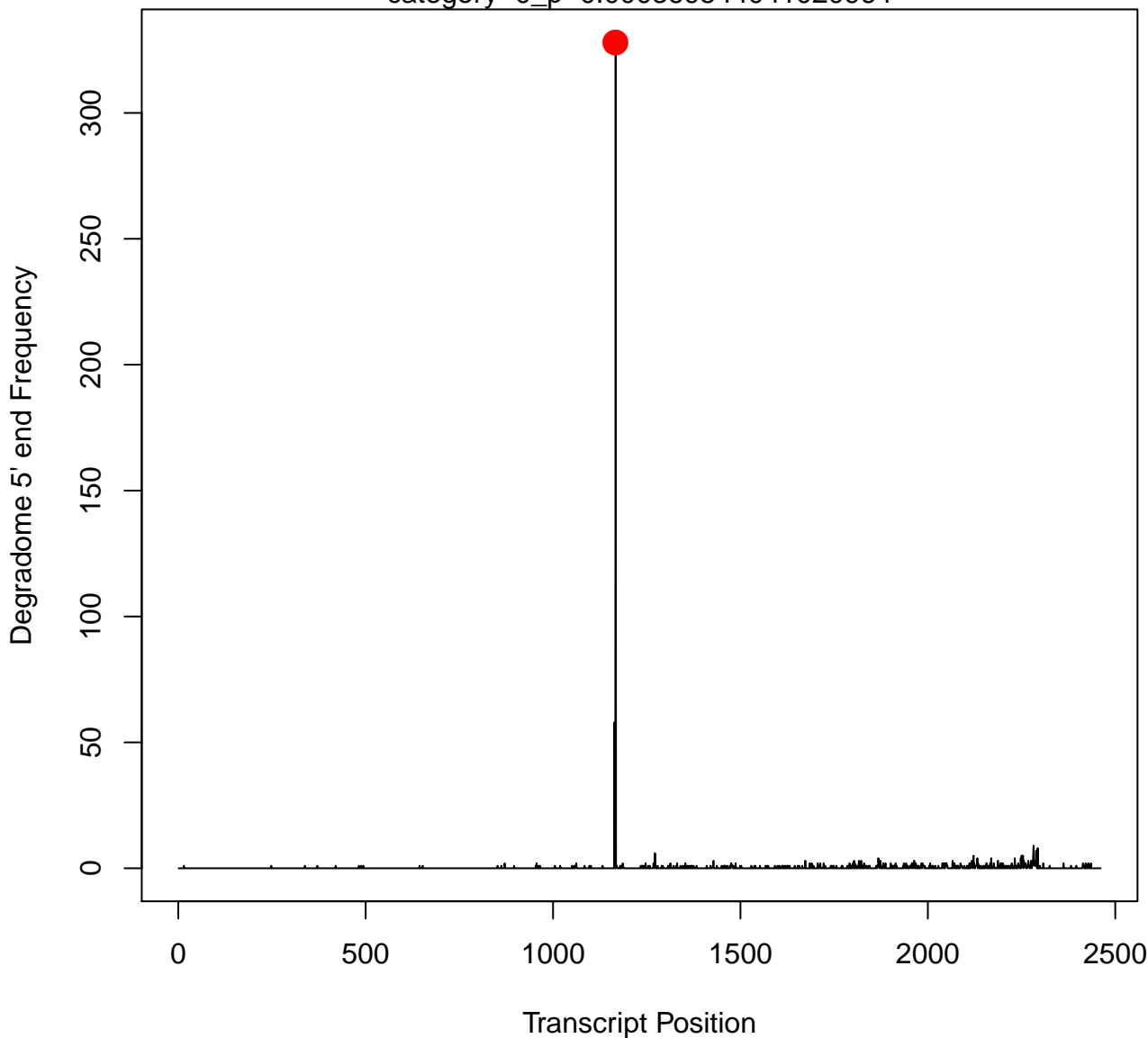

Supplement: Supplementary file 3 [file Data_Sheet_9.ZIP › GSM2230751.plot/Lsa-miR171b_Lsat_1_v5_gn_7_9261.1_1167_TPlot.pdf]

**T=Lsat\_1\_v5\_gn\_3\_63880.1\_Q=Lsa-miR171c\_S=577**

category=2\_p=0.982352920260902

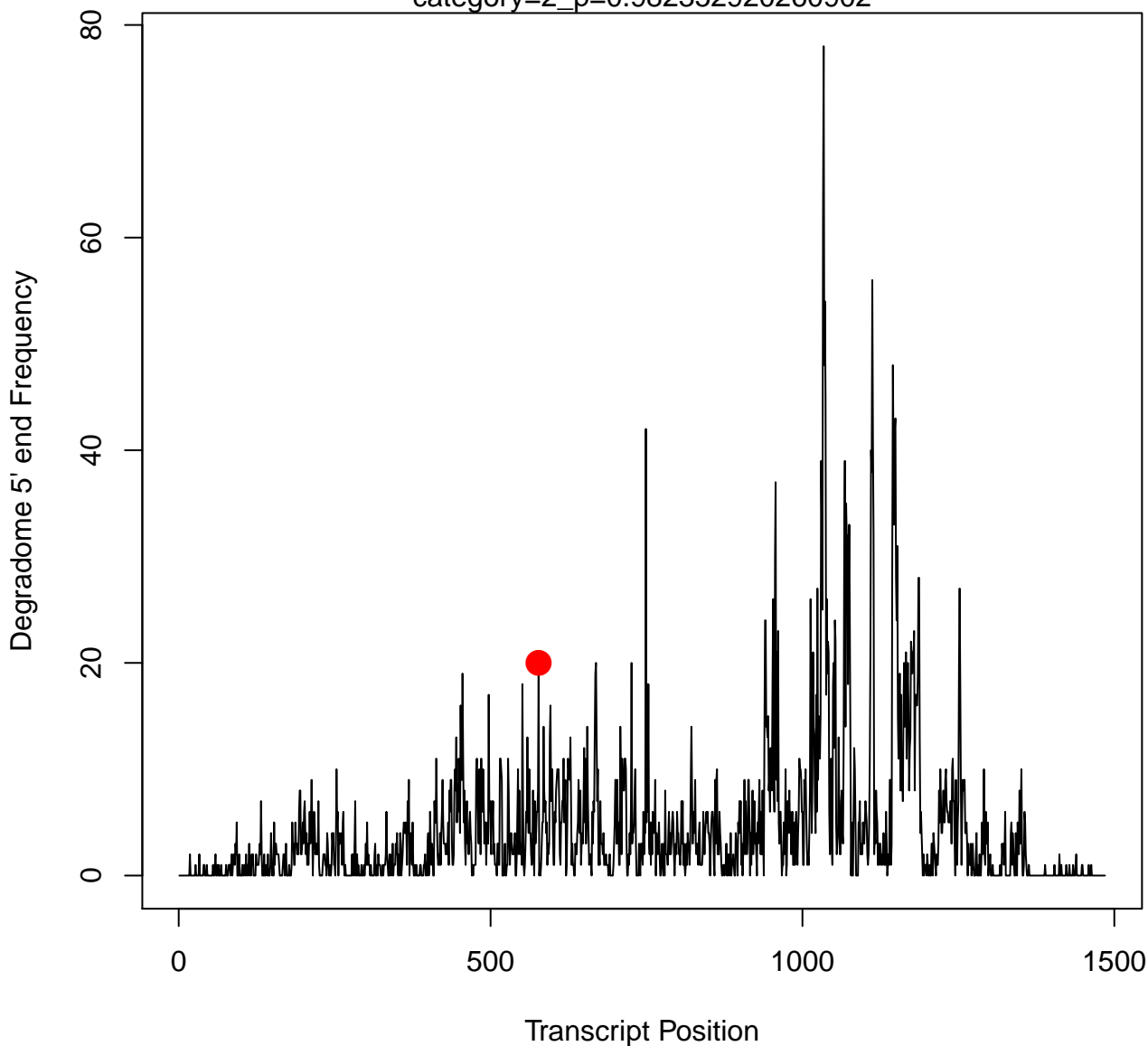

Supplement: Supplementary file 3 [file Data_Sheet_9.ZIP › GSM2230751.plot/Lsa-miR171c_Lsat_1_v5_gn_3_63880.1_577_TPlot.pdf]

**T=Lsat\_1\_v5\_gn\_3\_65860.1\_Q=Lsa-miR171c\_S=2360**

category=2\_p=0.955707692961799

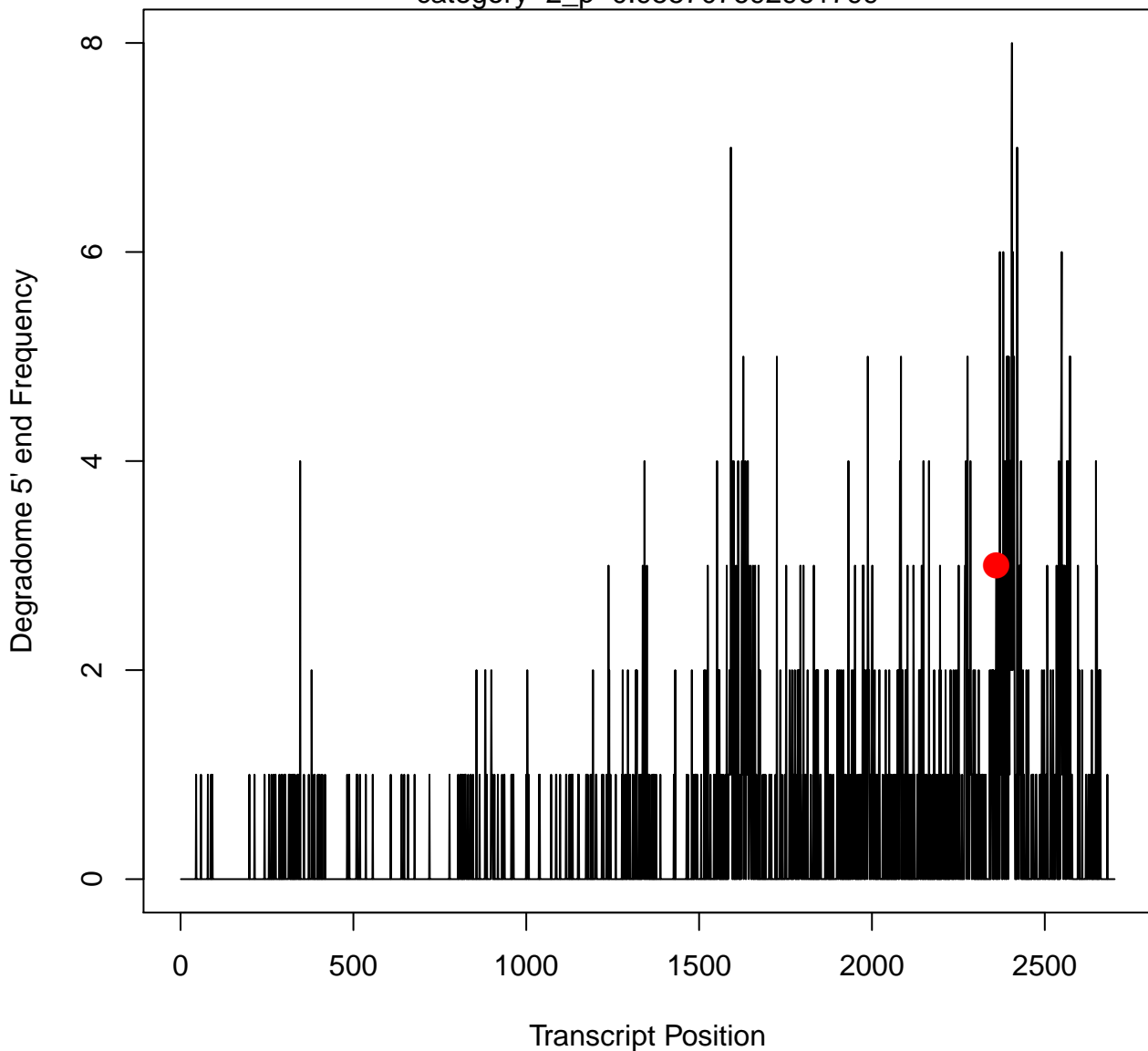

Supplement: Supplementary file 3 [file Data_Sheet_9.ZIP › GSM2230751.plot/Lsa-miR171c_Lsat_1_v5_gn_3_65860.1_2360_TPlot.pdf]

**T=Lsat\_1\_v5\_gn\_3\_681.1\_Q=Lsa-miR171c\_S=713**

category=2\_p=0.111961991206685

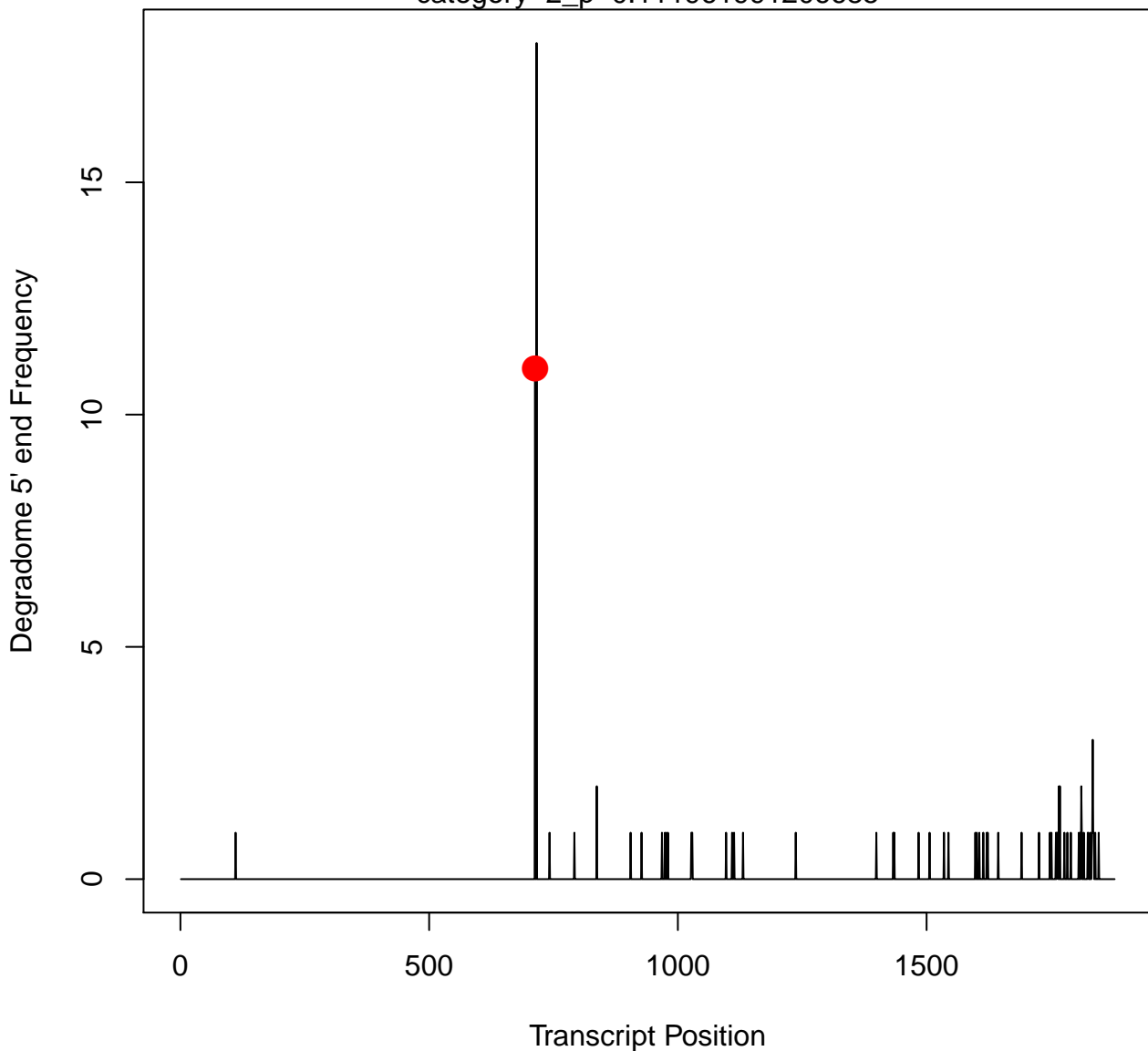

Supplement: Supplementary file 3 [file Data_Sheet_9.ZIP › GSM2230751.plot/Lsa-miR171c_Lsat_1_v5_gn_3_681.1_713_TPlot.pdf]

**T=Lsat\_1\_v5\_gn\_4\_149801.1\_Q=Lsa-miR171c\_S=210**

category=2\_p=0.987641450223065

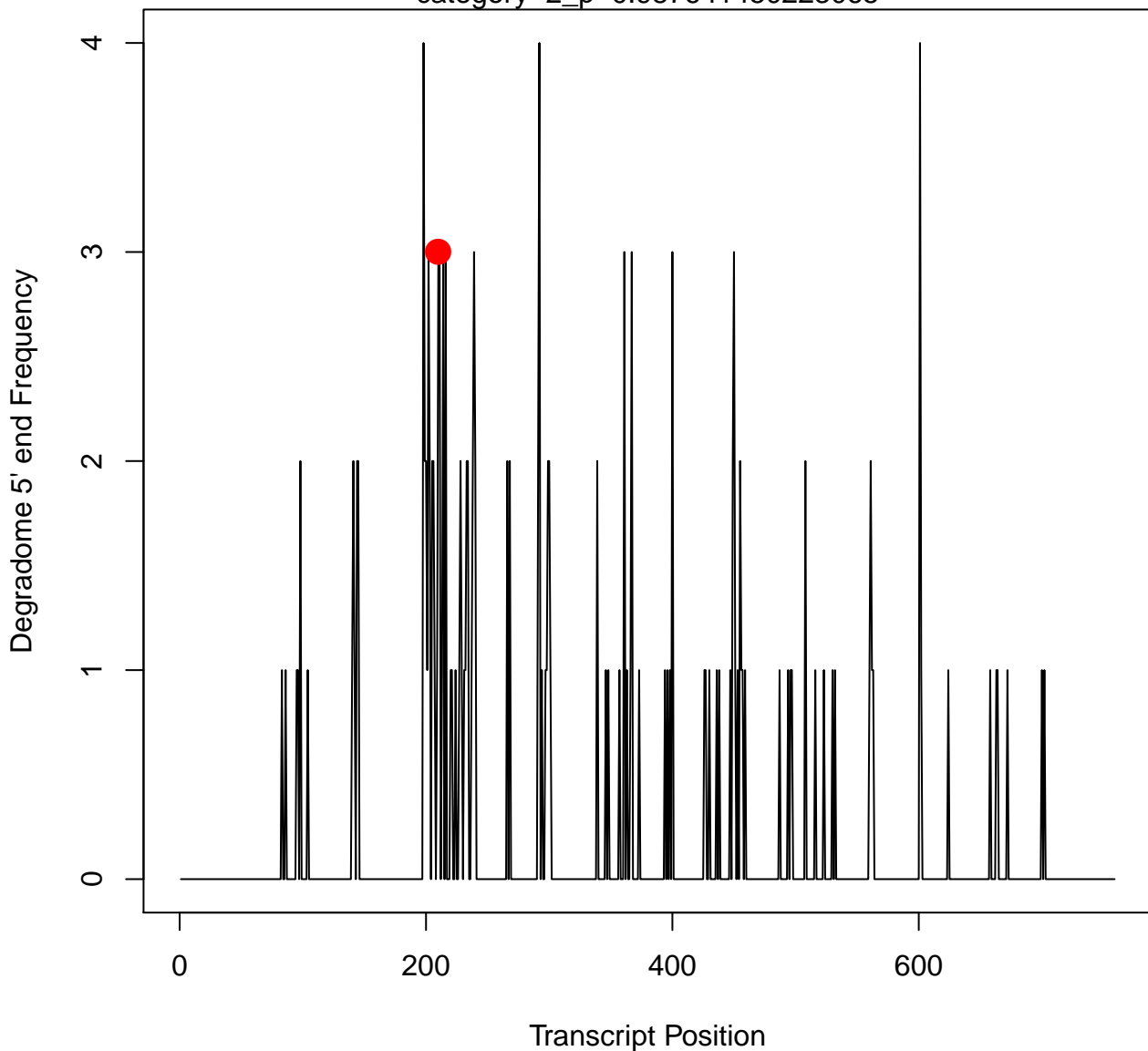

Supplement: Supplementary file 3 [file Data_Sheet_9.ZIP › GSM2230751.plot/Lsa-miR171c_Lsat_1_v5_gn_4_149801.1_210_TPlot.pdf]

**T=Lsat\_1\_v5\_gn\_4\_178061.1\_Q=Lsa-miR171c\_S=3889**

category=2\_p=0.975538331657721

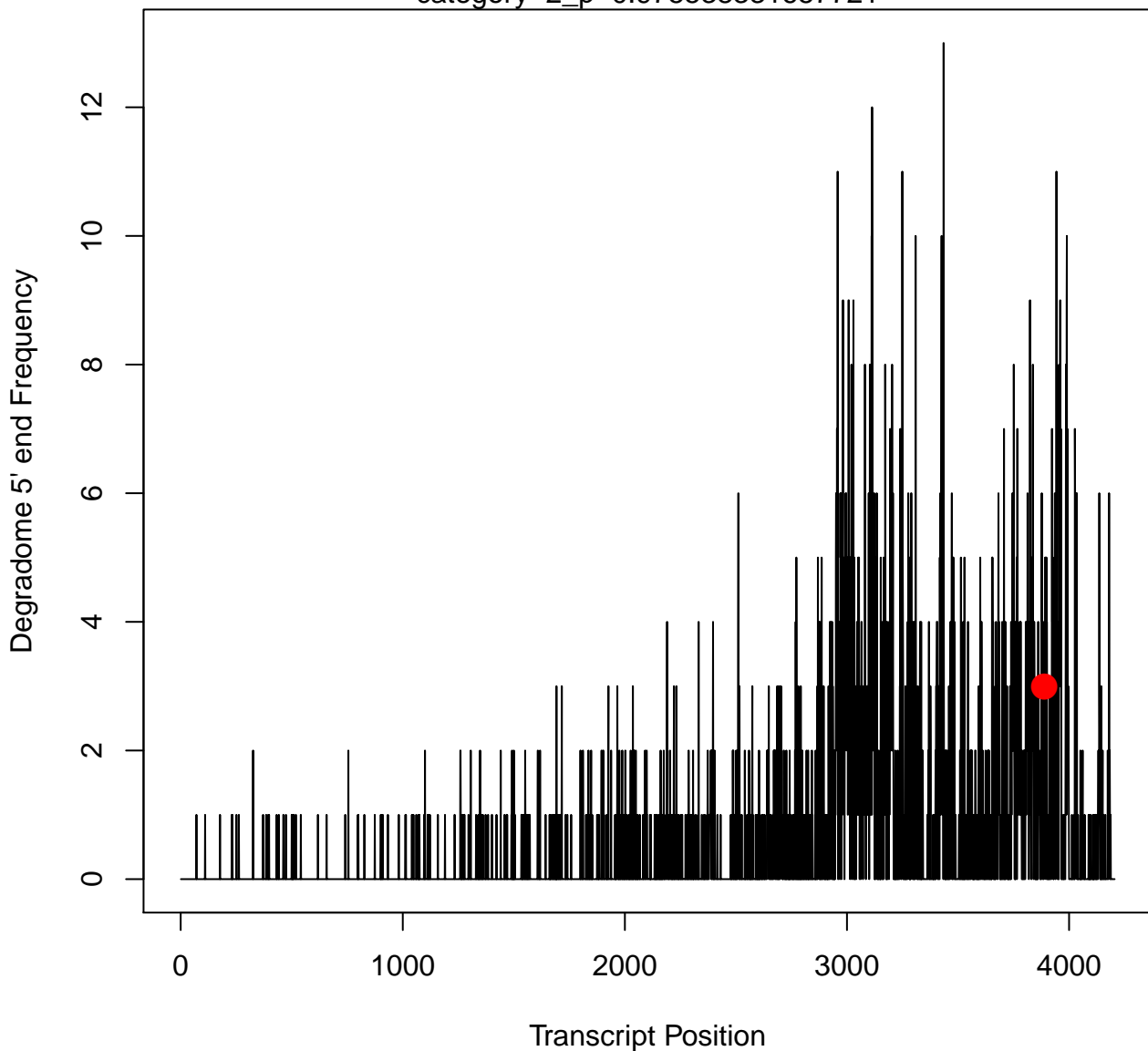

Supplement: Supplementary file 3 [file Data_Sheet_9.ZIP › GSM2230751.plot/Lsa-miR171c_Lsat_1_v5_gn_4_178061.1_3889_TPlot.pdf]

**T=Lsat\_1\_v5\_gn\_5\_124321.1\_Q=Lsa-miR171c\_S=542**

category=2\_p=0.914895063331028

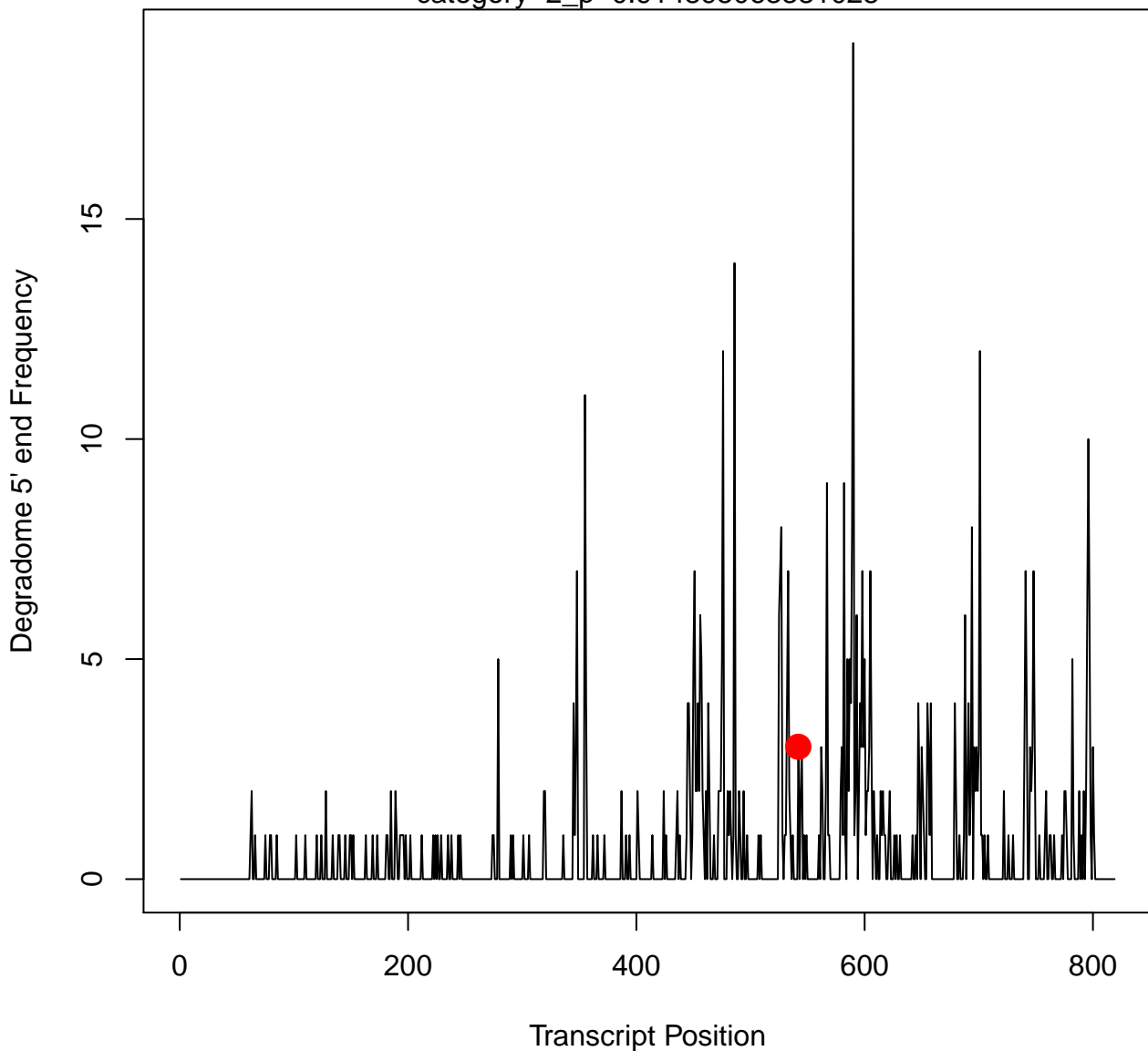

Supplement: Supplementary file 3 [file Data_Sheet_9.ZIP › GSM2230751.plot/Lsa-miR171c_Lsat_1_v5_gn_5_124321.1_542_TPlot.pdf]

**T=Lsat\_1\_v5\_gn\_5\_53581.1\_Q=Lsa-miR171c\_S=1236**

category=2\_p=0.928779981130817

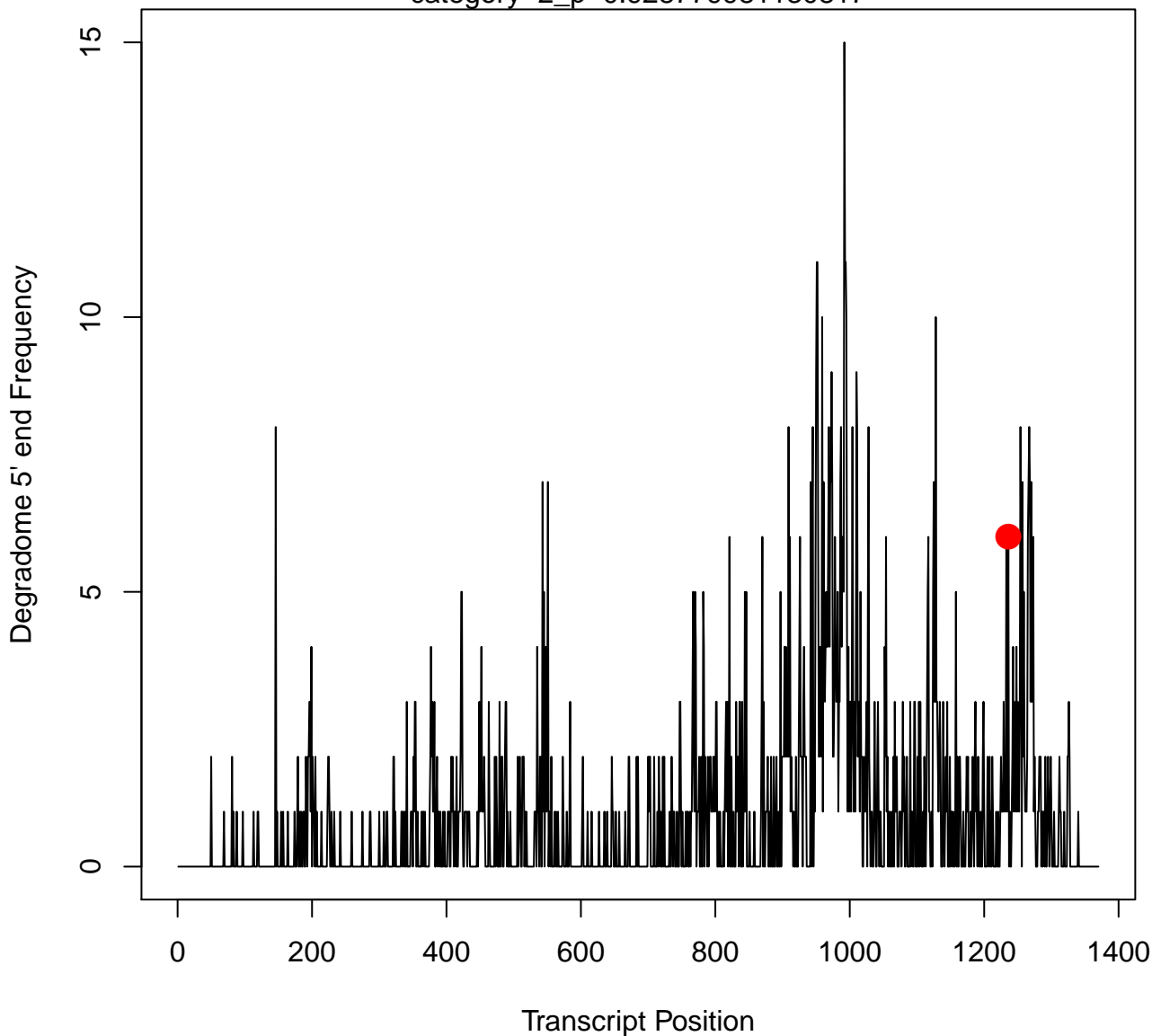

Supplement: Supplementary file 3 [file Data_Sheet_9.ZIP › GSM2230751.plot/Lsa-miR171c_Lsat_1_v5_gn_5_53581.1_1236_TPlot.pdf]

**T=Lsat\_1\_v5\_gn\_6\_3180.1\_Q=Lsa-miR171c\_S=1758**

category=2\_p=0.0852052452876977

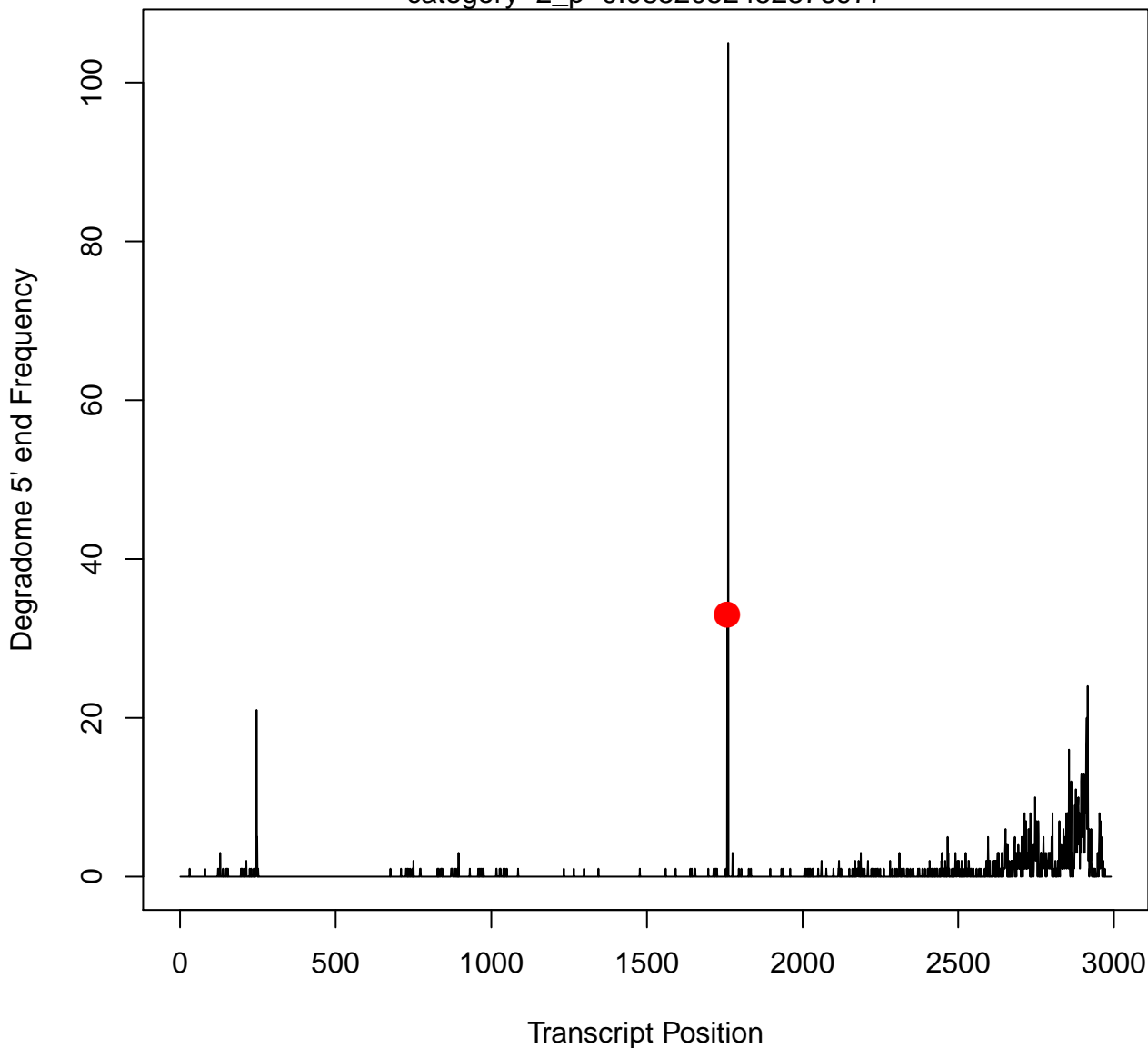

Supplement: Supplementary file 3 [file Data_Sheet_9.ZIP › GSM2230751.plot/Lsa-miR171c_Lsat_1_v5_gn_6_3180.1_1758_TPlot.pdf]

T=Lsat\_1\_v5\_gn\_7\_49541.1\_Q=Lsa-miR171c\_S=871

category=2\_p=0.396282025902823

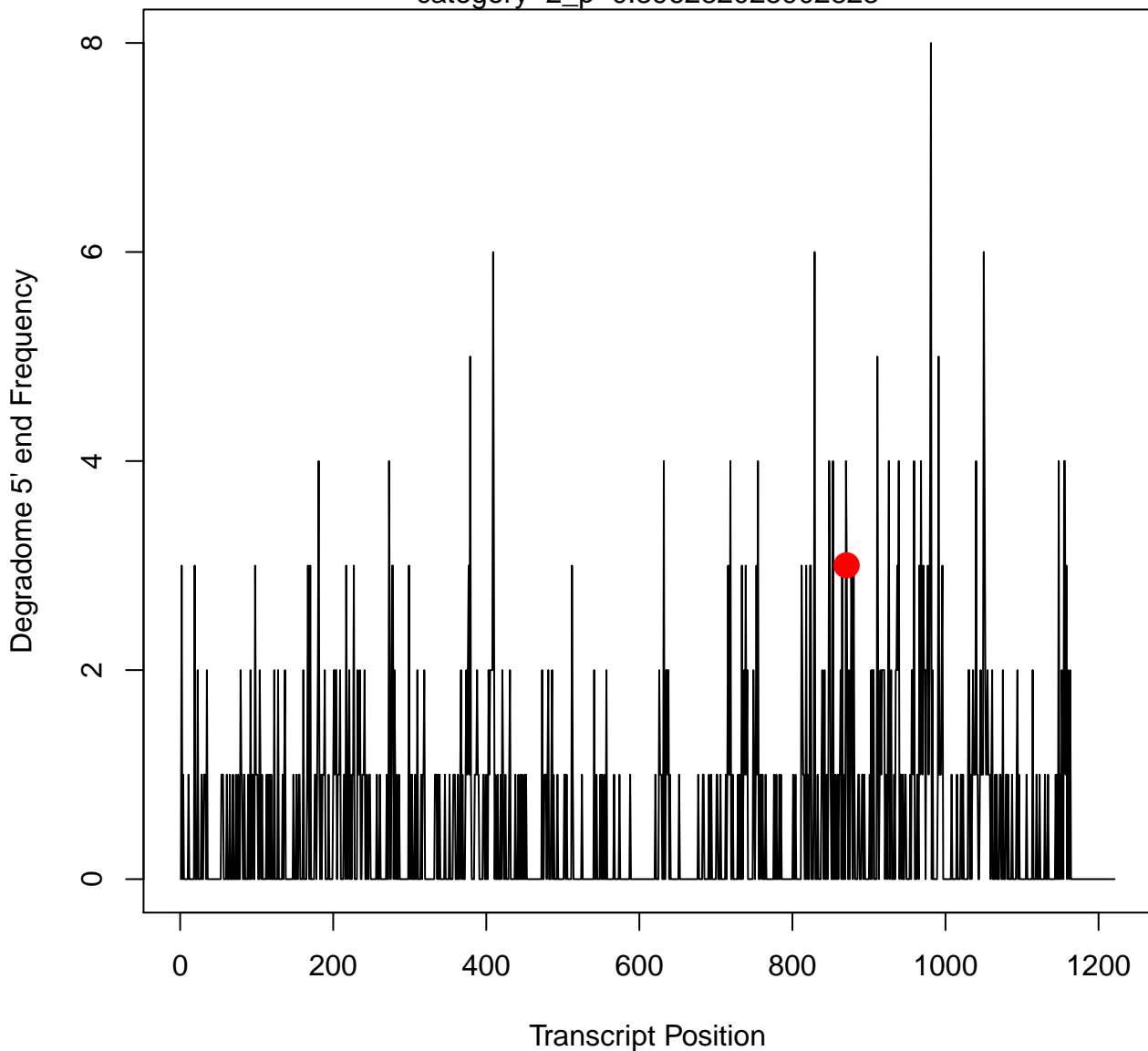

Supplement: Supplementary file 3 [file Data_Sheet_9.ZIP › GSM2230751.plot/Lsa-miR171c_Lsat_1_v5_gn_7_49541.1_871_TPlot.pdf]

**T=Lsat\_1\_v5\_gn\_7\_97240.1\_Q=Lsa-miR171c\_S=2083**

category=2\_p=0.977622594108971

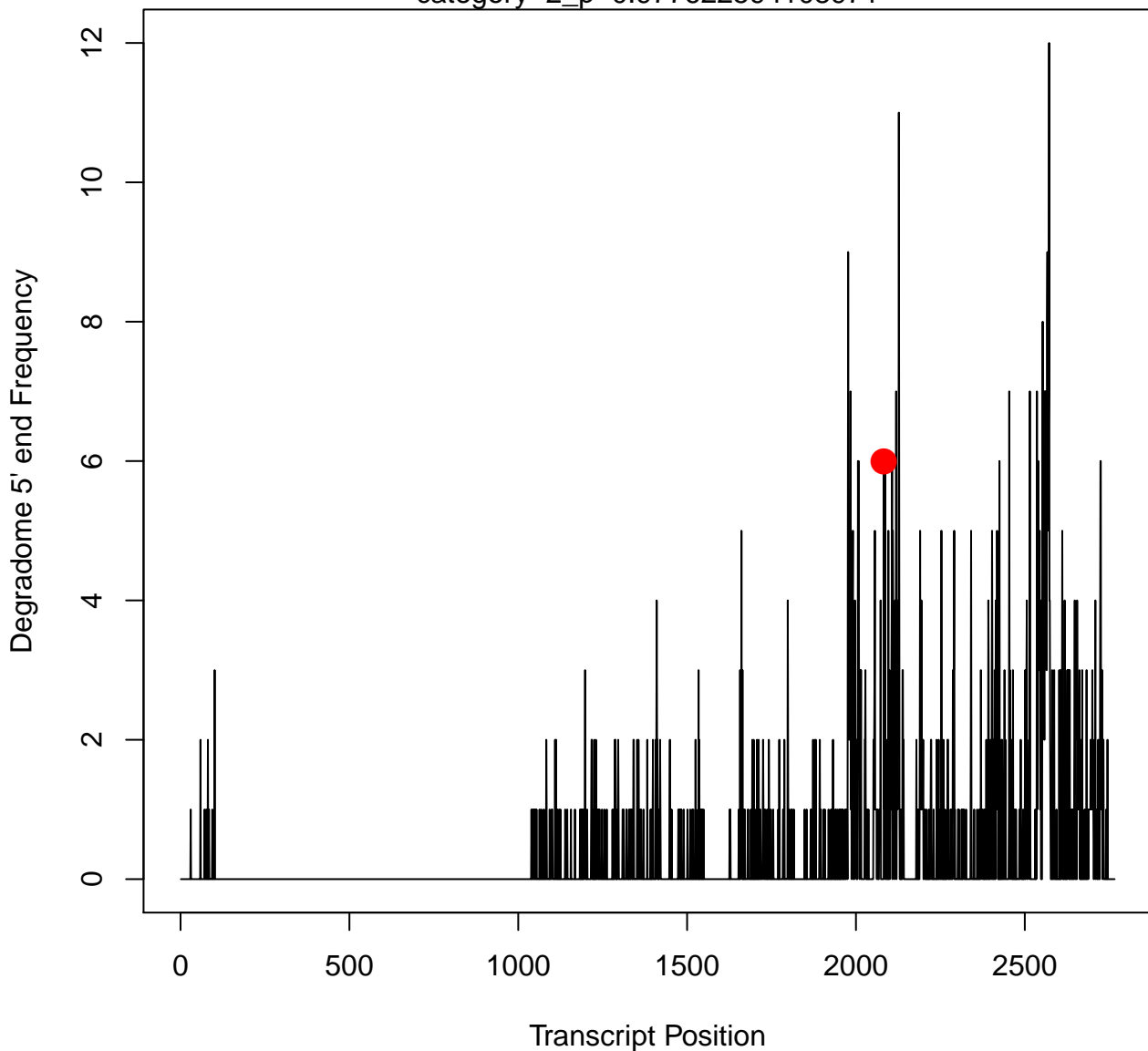

Supplement: Supplementary file 3 [file Data_Sheet_9.ZIP › GSM2230751.plot/Lsa-miR171c_Lsat_1_v5_gn_7_97240.1_2083_TPlot.pdf]

**T=Lsat\_1\_v5\_gn\_8\_12460.1\_Q=Lsa-miR171c\_S=1103**

category=2\_p=0.994455268723833

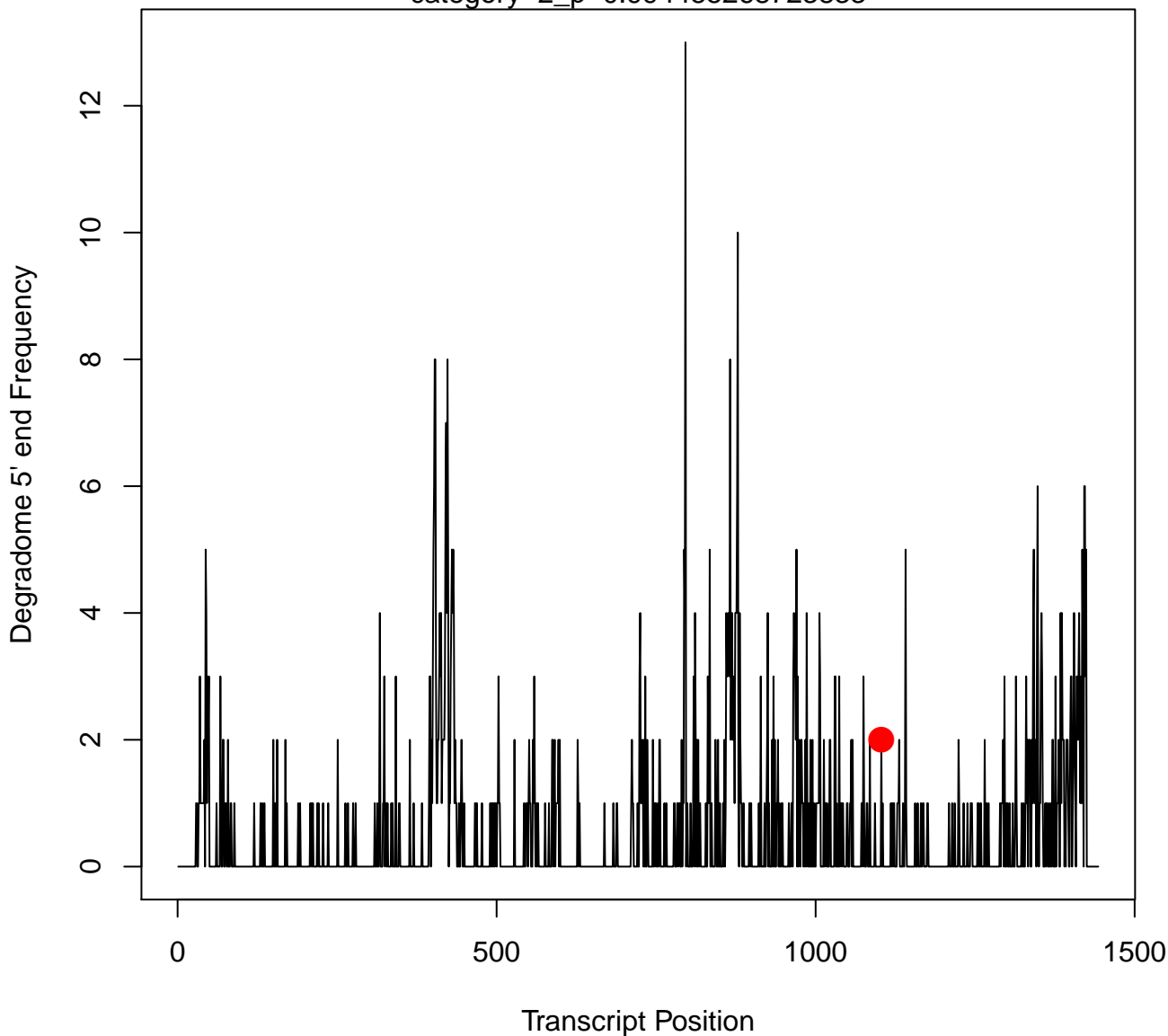

Supplement: Supplementary file 3 [file Data_Sheet_9.ZIP › GSM2230751.plot/Lsa-miR171c_Lsat_1_v5_gn_8_12460.1_1103_TPlot.pdf]

**T=Lsat\_1\_v5\_gn\_5\_181100.1\_Q=Lsa-miR171d\_S=768**

category=2\_p=0.845902007472907

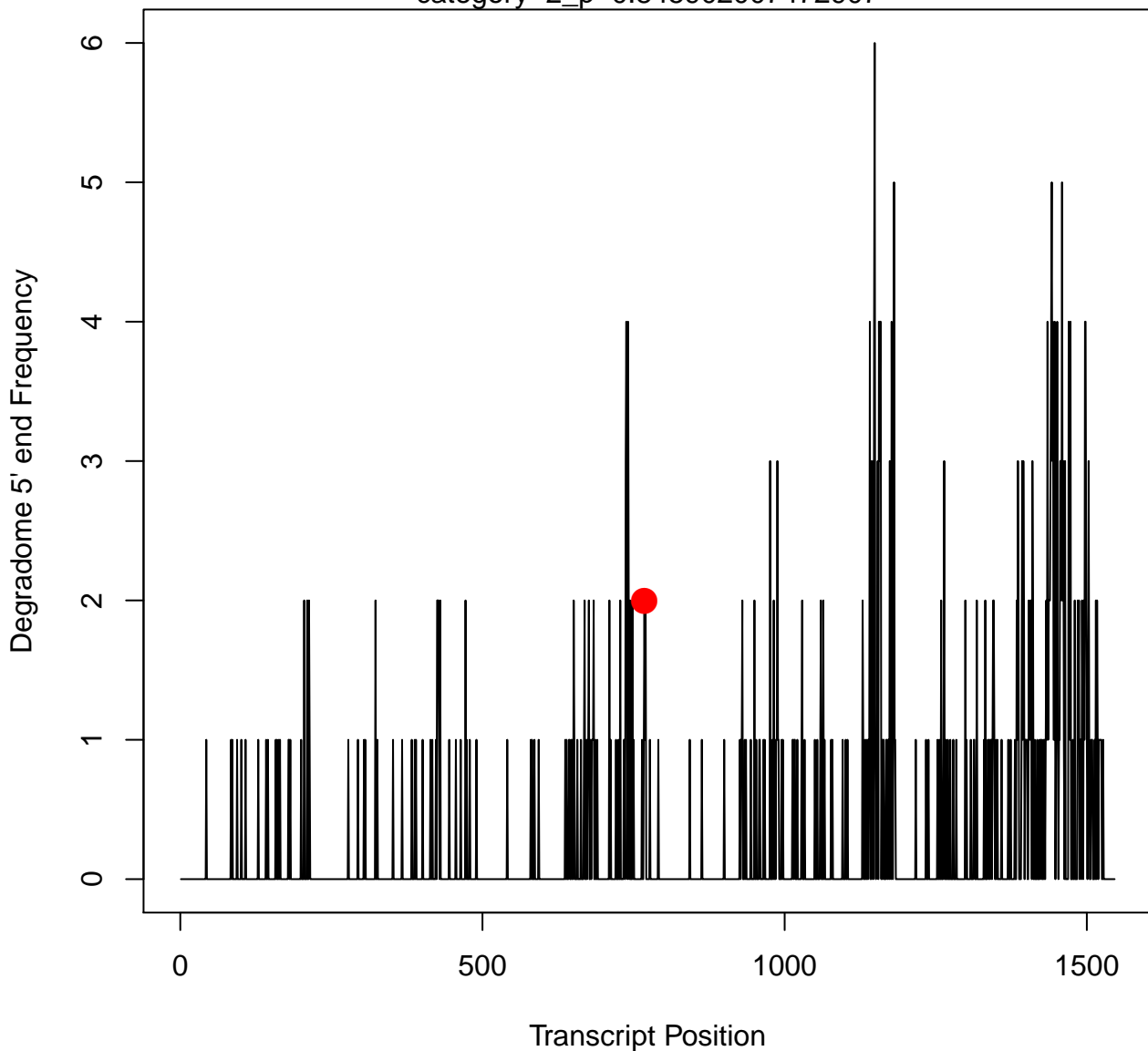

Supplement: Supplementary file 3 [file Data_Sheet_9.ZIP › GSM2230751.plot/Lsa-miR171d_Lsat_1_v5_gn_5_181100.1_768_TPlot.pdf]

**T=Lsat\_1\_v5\_gn\_7\_9261.1\_Q=Lsa-miR171d\_S=1164**

category=2\_p=0.0852052452876977

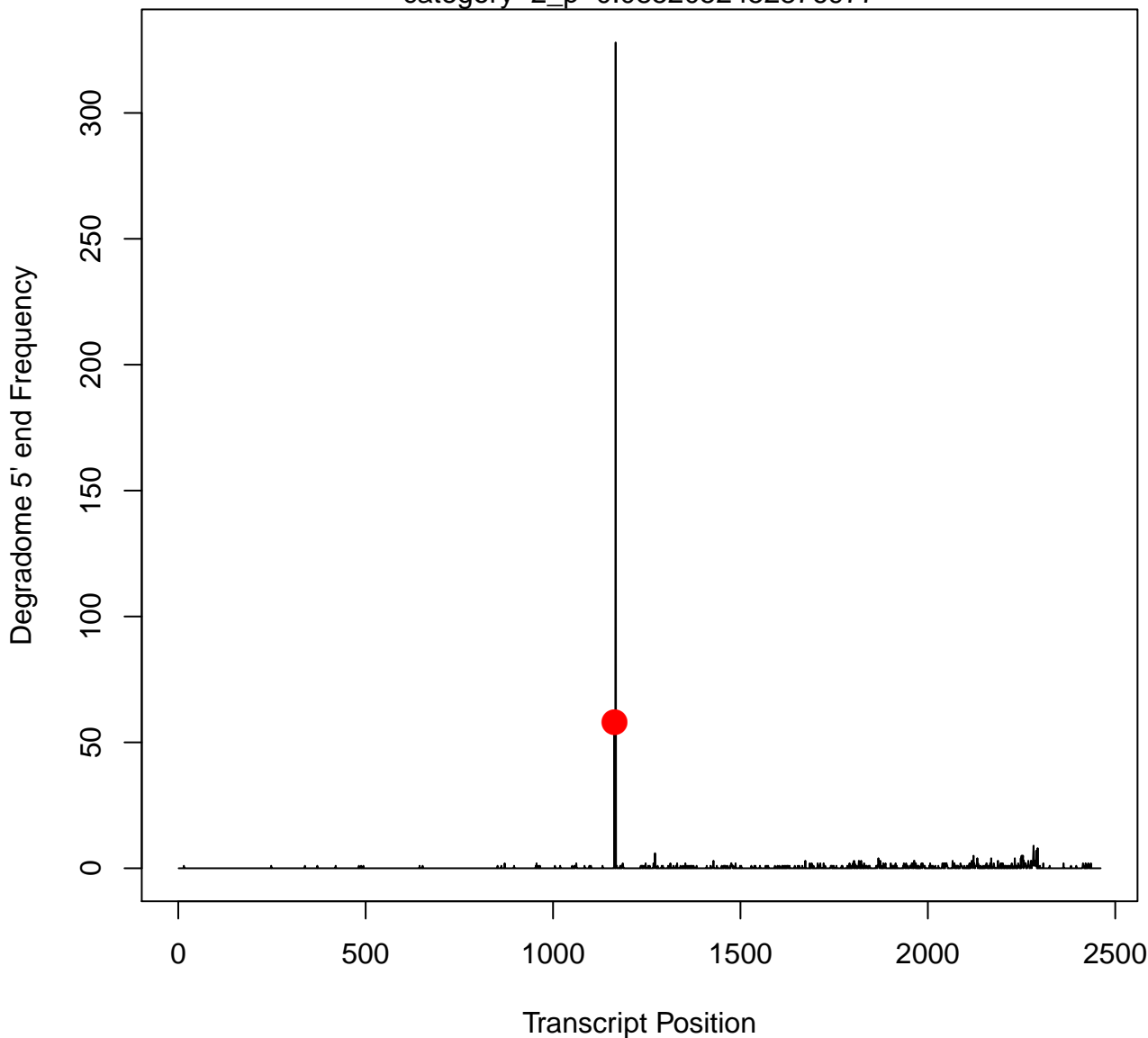

Supplement: Supplementary file 3 [file Data_Sheet_9.ZIP › GSM2230751.plot/Lsa-miR171d_Lsat_1_v5_gn_7_9261.1_1164_TPlot.pdf]

**T=Lsat\_1\_v5\_gn\_3\_55860.1\_Q=Lsa-miR171e\_S=341**

category=2\_p=0.137936131575646

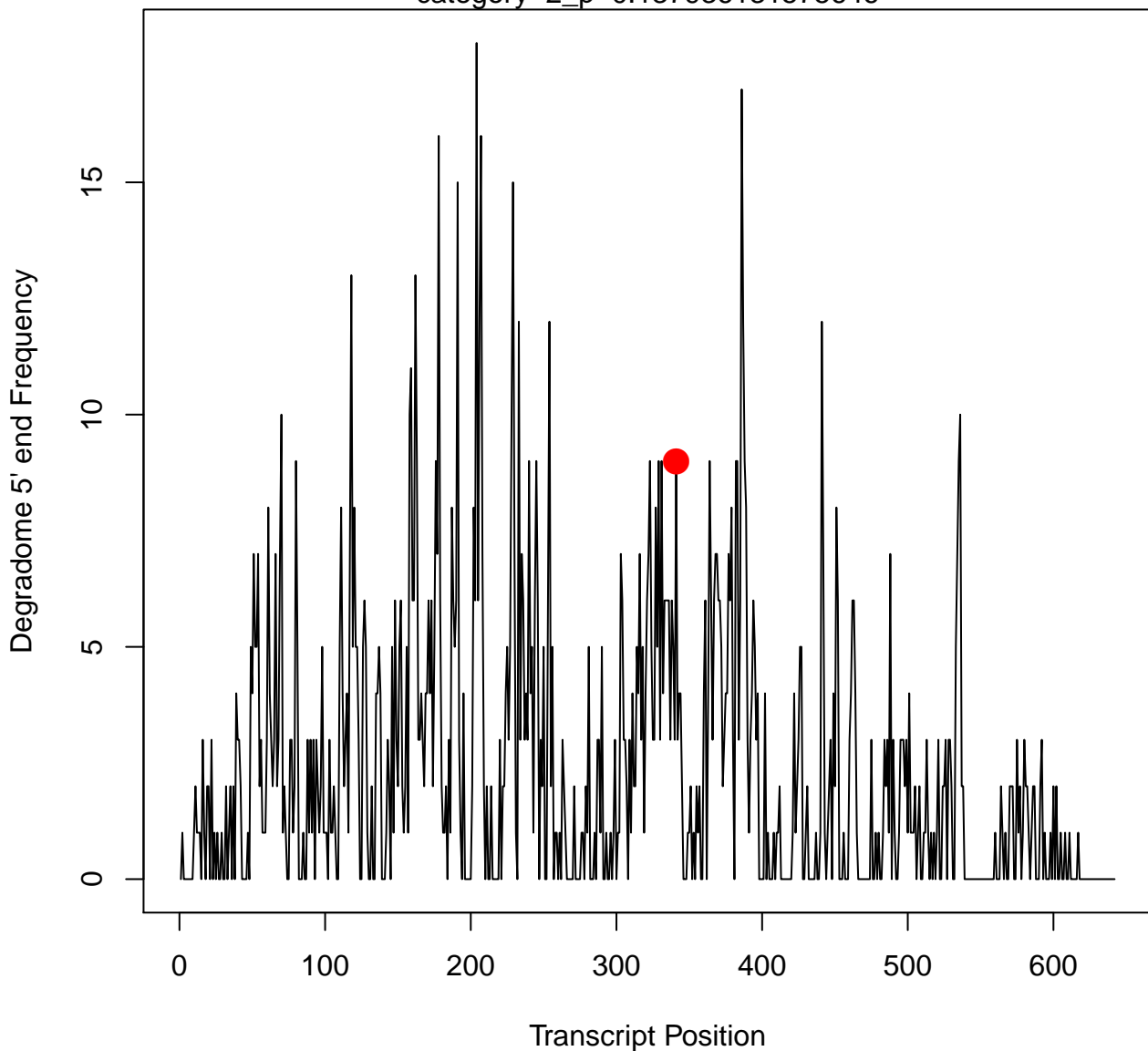

Supplement: Supplementary file 3 [file Data_Sheet_9.ZIP › GSM2230751.plot/Lsa-miR171e_Lsat_1_v5_gn_3_55860.1_341_TPlot.pdf]

**T=Lsat\_1\_v5\_gn\_5\_145661.1\_Q=Lsa-miR171e\_S=2412**

category=2\_p=0.537826433029871

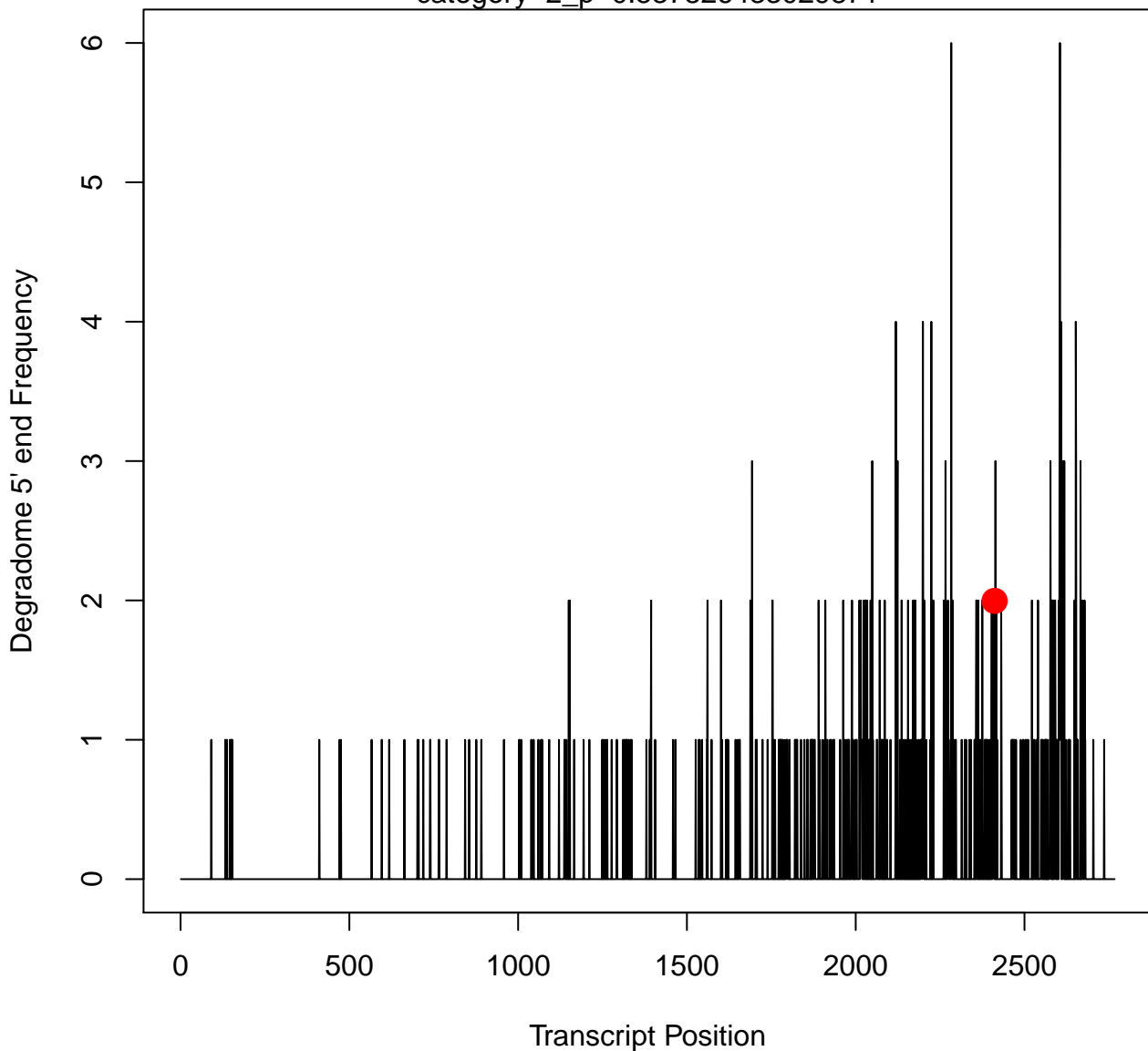

Supplement: Supplementary file 3 [file Data_Sheet_9.ZIP › GSM2230751.plot/Lsa-miR171e_Lsat_1_v5_gn_5_145661.1_2412_TPlot.pdf]

**T=Lsat\_1\_v5\_gn\_5\_167061.1\_Q=Lsa-miR171e\_S=3787**

category=2\_p=0.744751690128926

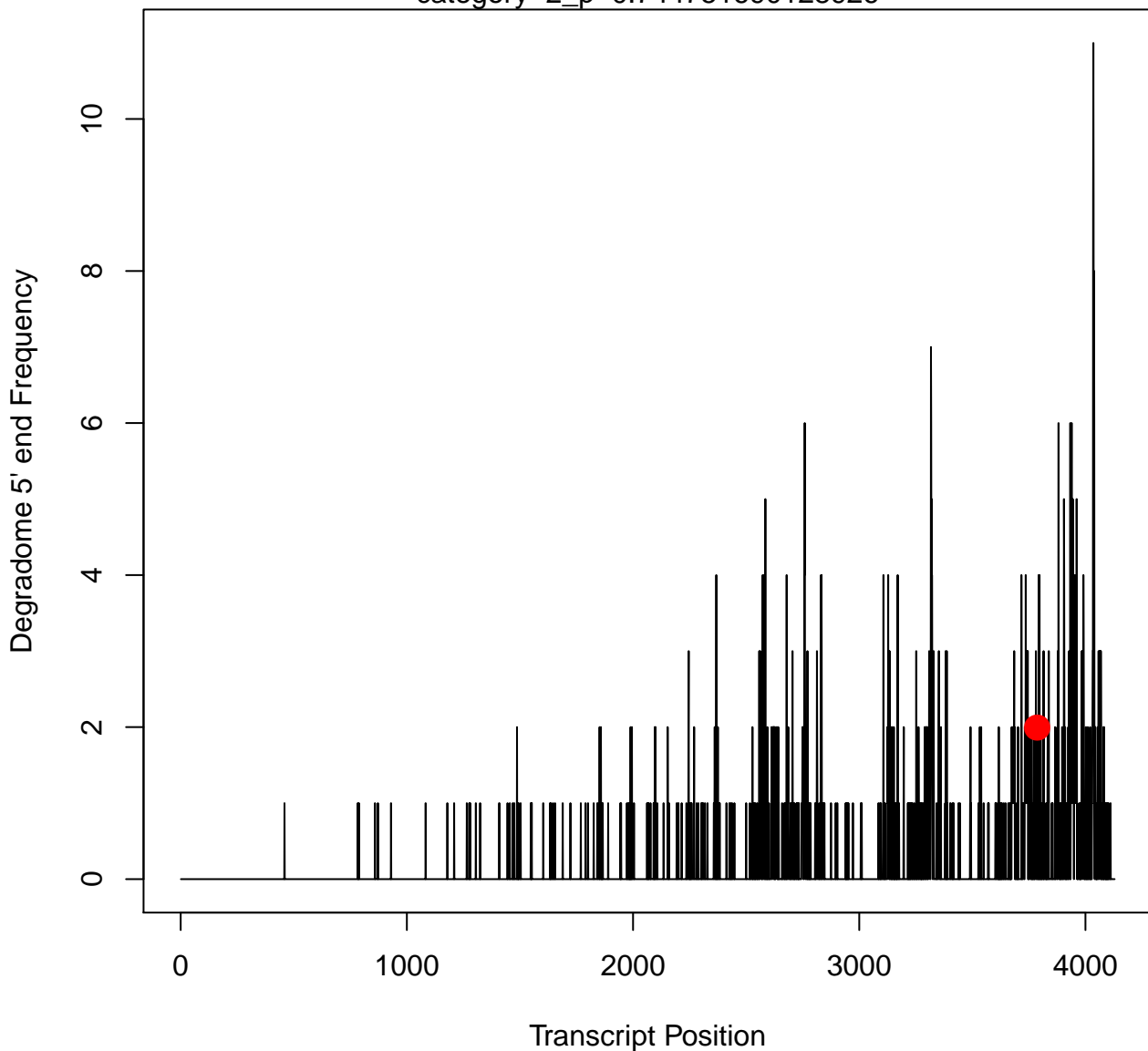

Supplement: Supplementary file 3 [file Data_Sheet_9.ZIP › GSM2230751.plot/Lsa-miR171e_Lsat_1_v5_gn_5_167061.1_3787_TPlot.pdf]

**T=Lsat\_1\_v5\_gn\_7\_31021.1\_Q=Lsa-miR171e\_S=3038**

category=2\_p=0.798708246188144

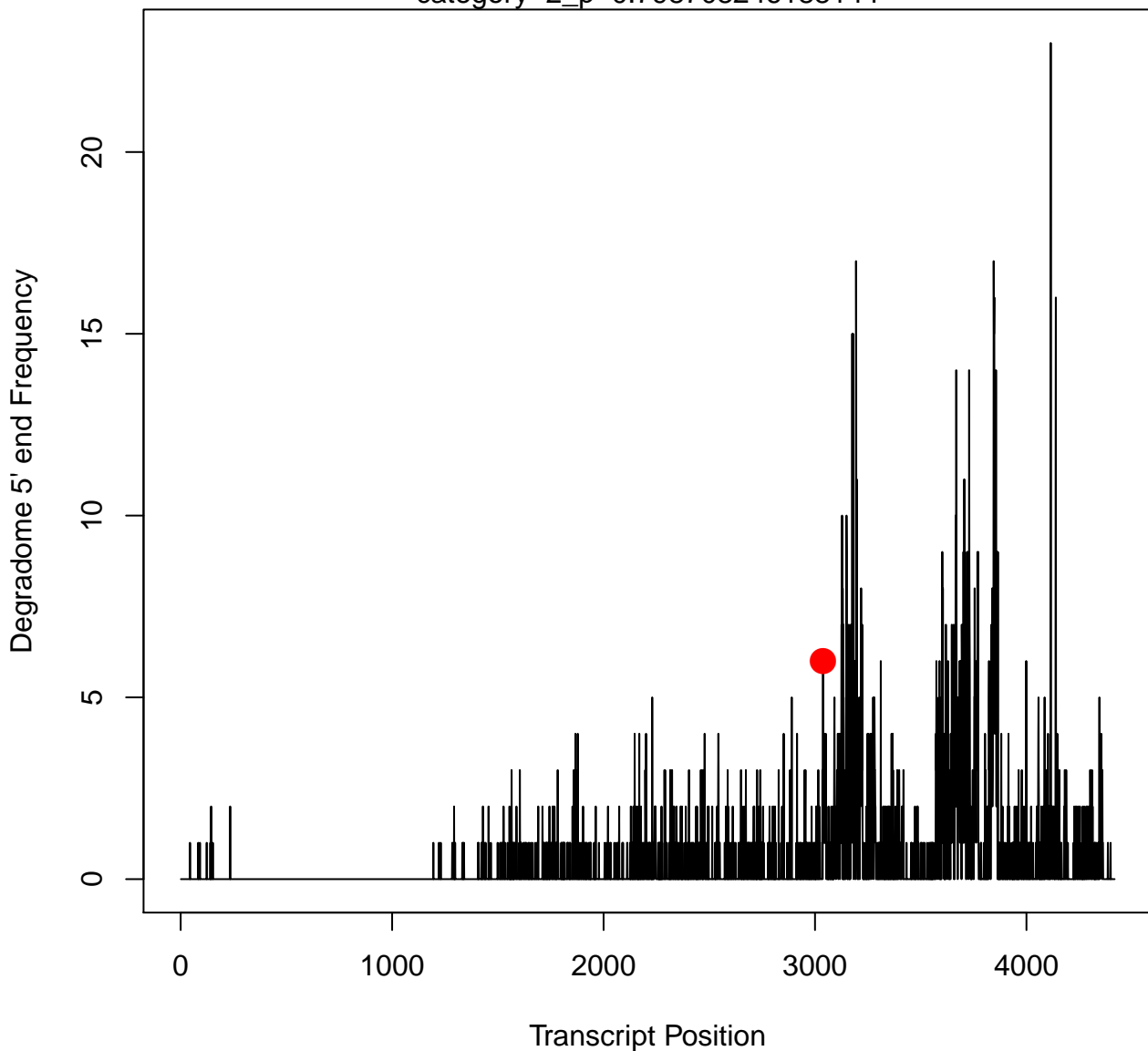

Supplement: Supplementary file 3 [file Data_Sheet_9.ZIP › GSM2230751.plot/Lsa-miR171e_Lsat_1_v5_gn_7_31021.1_3038_TPlot.pdf]

**T=Lsat\_1\_v5\_gn\_8\_3401.1\_Q=Lsa-miR171e\_S=1292**

category=2\_p=0.396282025902823

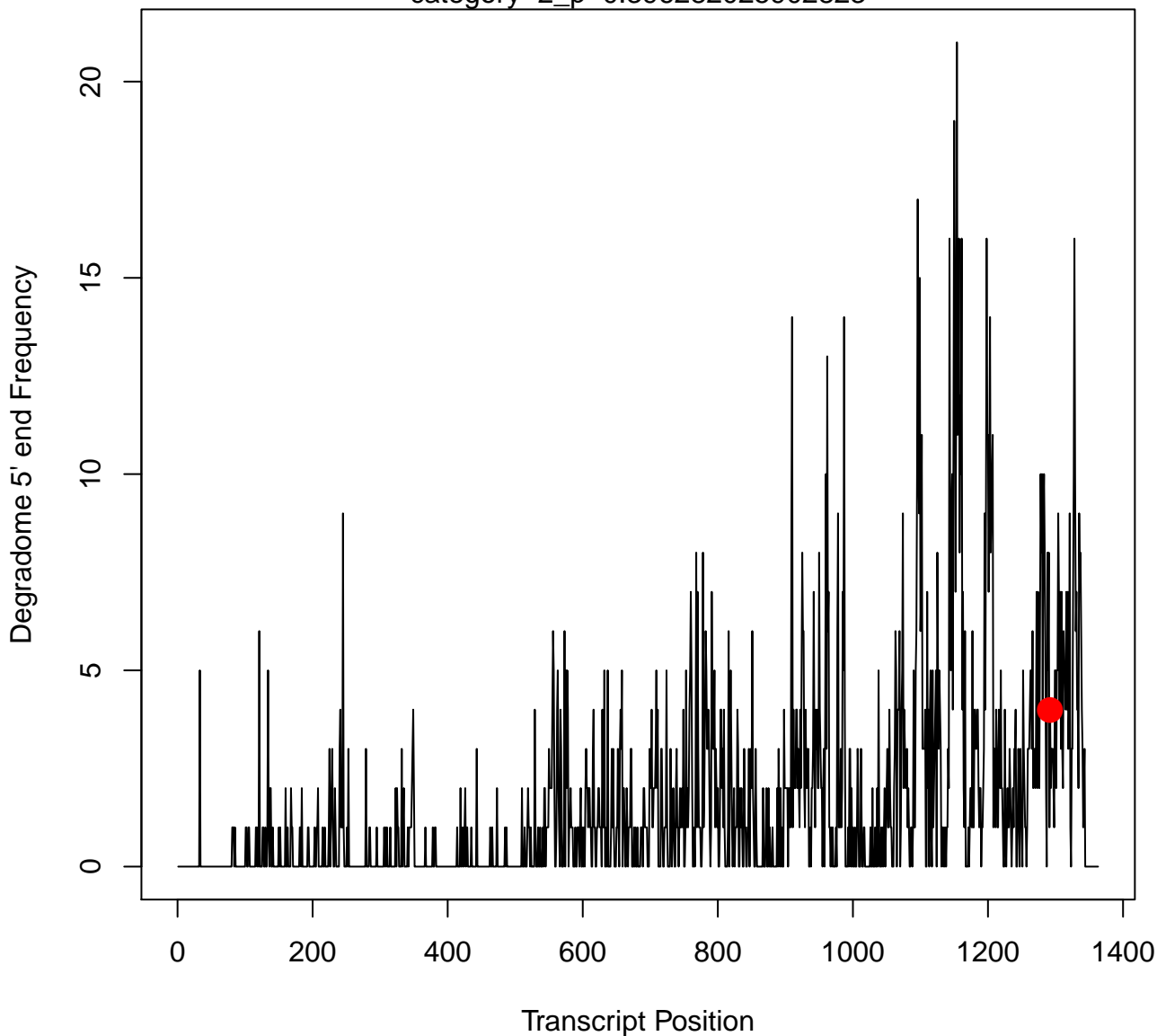

Supplement: Supplementary file 3 [file Data_Sheet_9.ZIP › GSM2230751.plot/Lsa-miR171e_Lsat_1_v5_gn_8_3401.1_1292_TPlot.pdf]

**T=Lsat\_1\_v5\_gn\_3\_36300.1\_Q=Lsa-miR171f\_S=1498**

category=2\_p=0.95437315773487

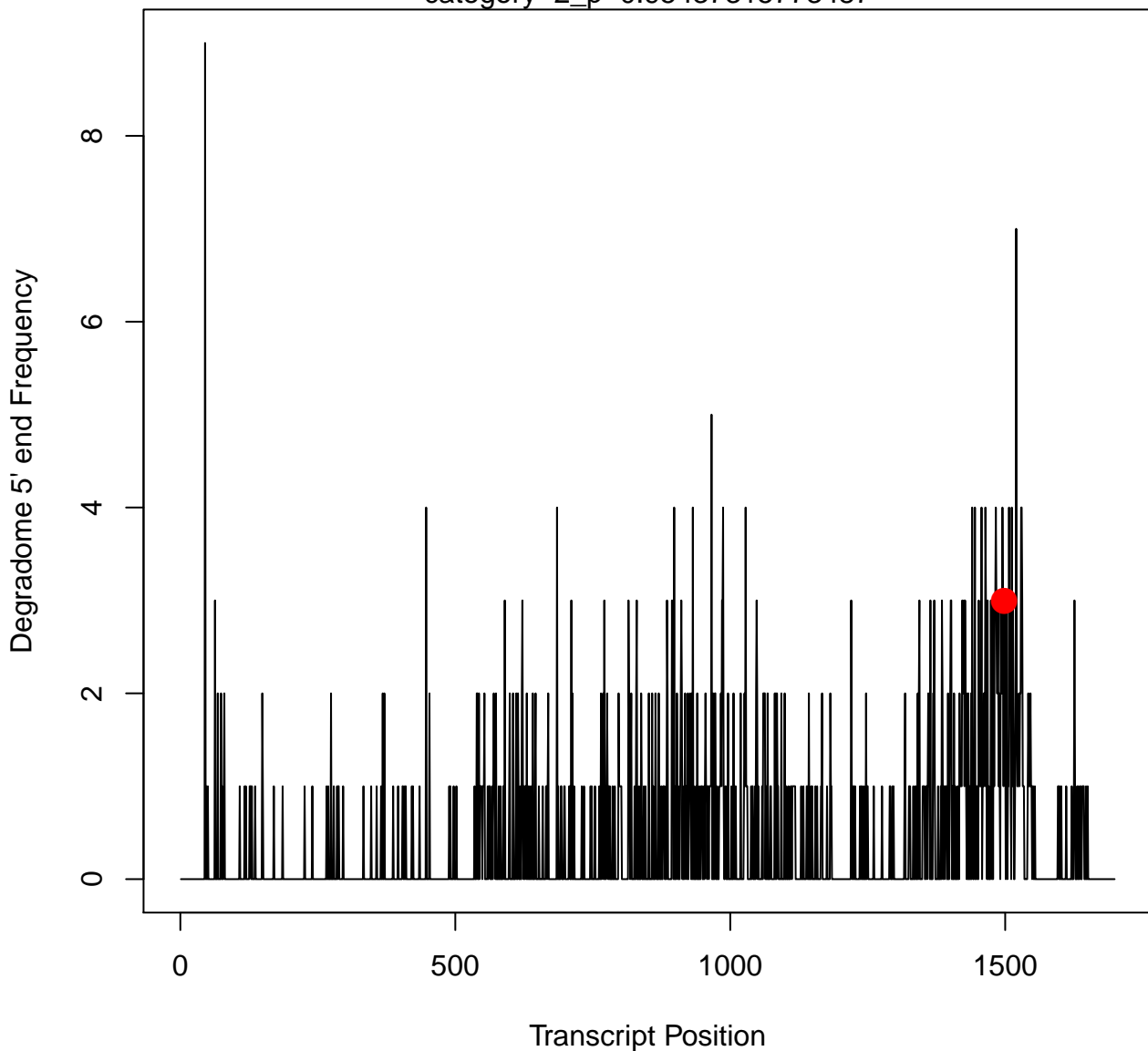

Supplement: Supplementary file 3 [file Data_Sheet_9.ZIP › GSM2230751.plot/Lsa-miR171f_Lsat_1_v5_gn_3_36300.1_1498_TPlot.pdf]

**T=Lsat\_1\_v5\_gn\_3\_78621.1\_Q=Lsa-miR171f\_S=732**

category=0\_p=0.000369544041020964

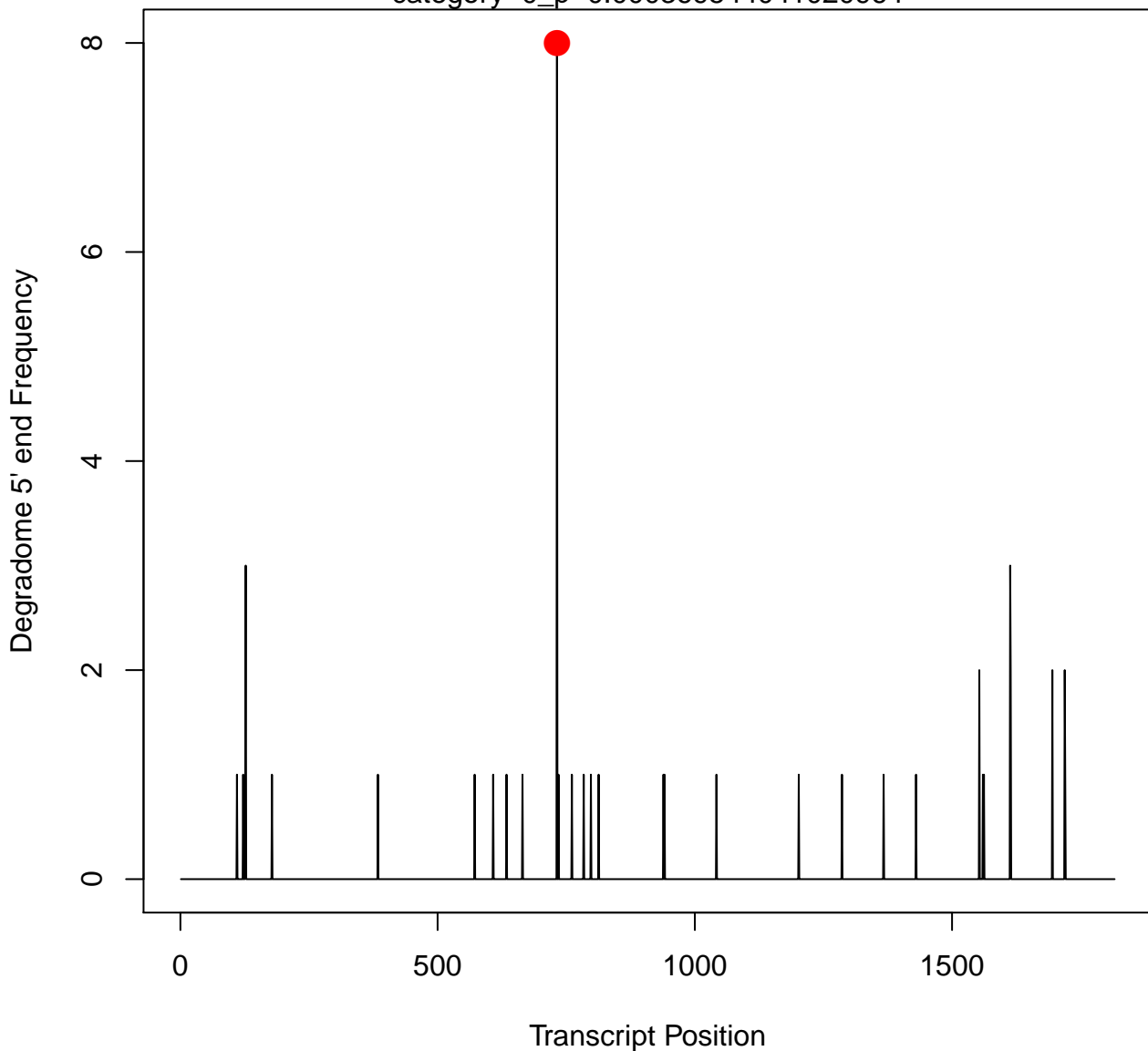

Supplement: Supplementary file 3 [file Data_Sheet_9.ZIP › GSM2230751.plot/Lsa-miR171f_Lsat_1_v5_gn_3_78621.1_732_TPlot.pdf]

**T=Lsat\_1\_v5\_gn\_8\_144901.1\_Q=Lsa-miR172a\_S=920**

category=2\_p=0.766500184980761

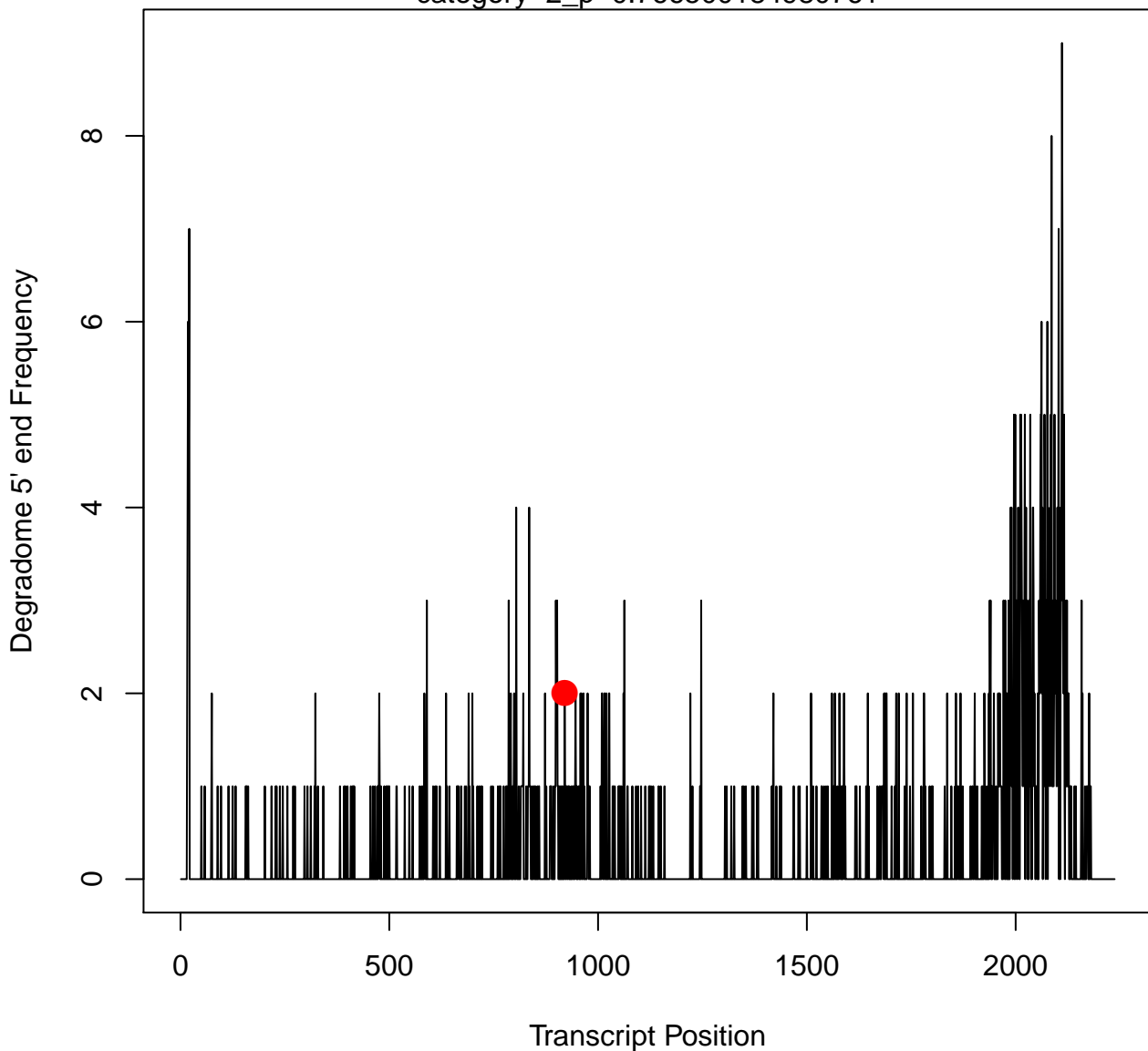

Supplement: Supplementary file 3 [file Data_Sheet_9.ZIP › GSM2230751.plot/Lsa-miR172a_Lsat_1_v5_gn_8_144901.1_920_TPlot.pdf]

**T=Lsat\_1\_v5\_gn\_9\_20420.1\_Q=Lsa-miR172a\_S=1655**

category=2\_p=0.137936131575646

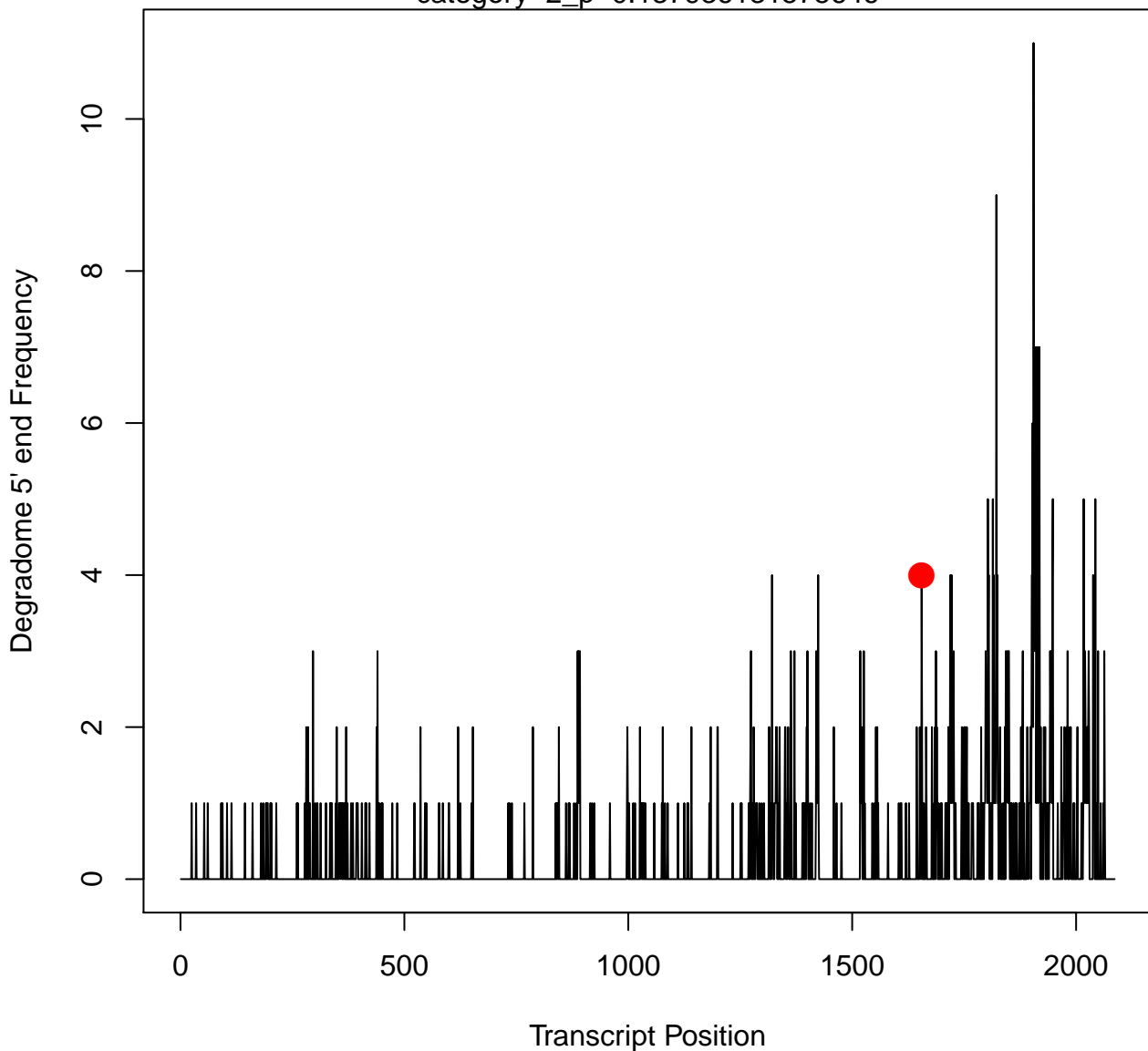

Supplement: Supplementary file 3 [file Data_Sheet_9.ZIP › GSM2230751.plot/Lsa-miR172a_Lsat_1_v5_gn_9_20420.1_1655_TPlot.pdf]

**T=Lsat\_1\_v5\_gn\_2\_109741.1\_Q=Lsa-miR172b\_S=1566**

category=2\_p=0.0852052452876977

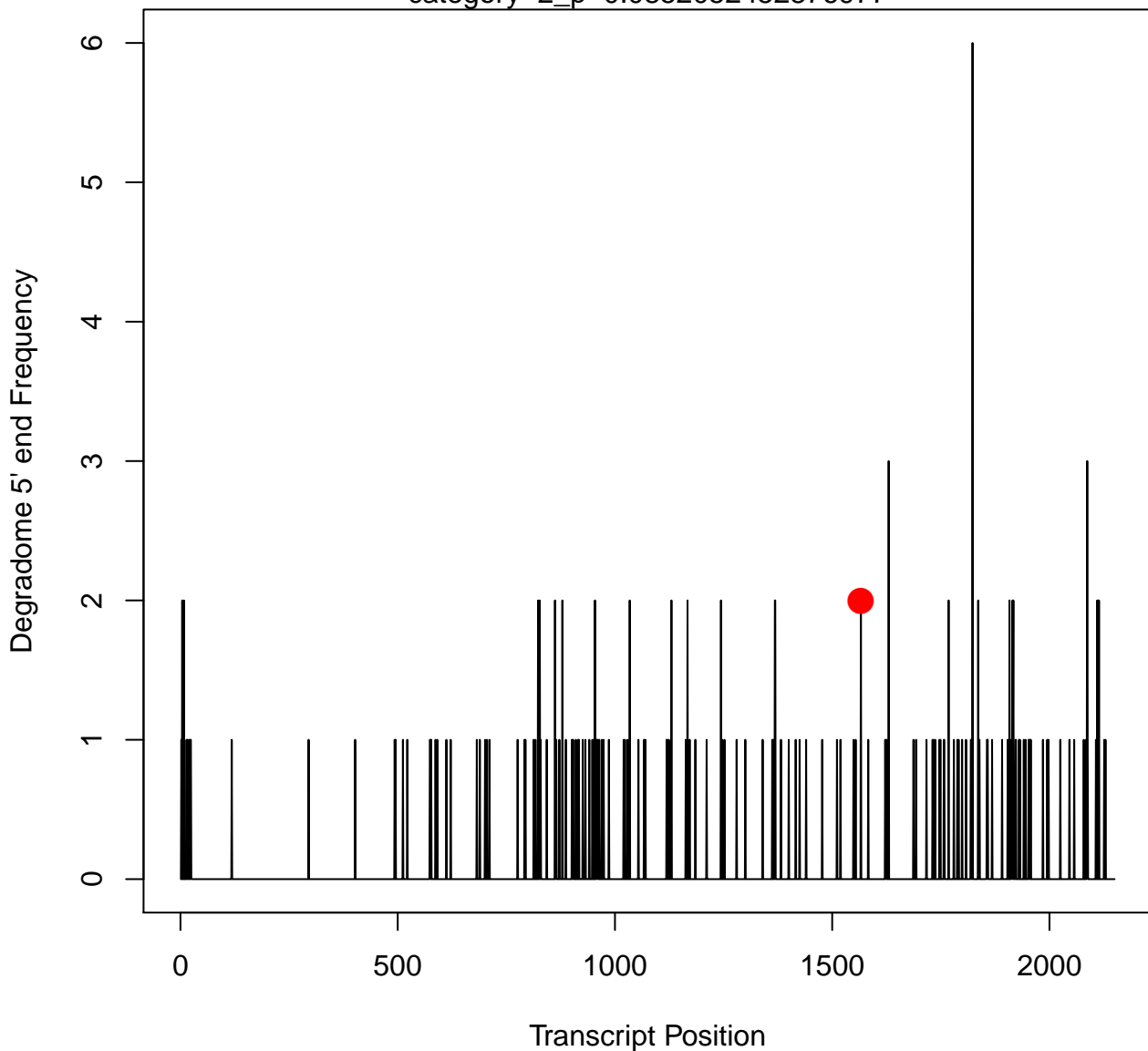

Supplement: Supplementary file 3 [file Data_Sheet_9.ZIP › GSM2230751.plot/Lsa-miR172b_Lsat_1_v5_gn_2_109741.1_1566_TPlot.pdf]

**T=Lsat\_1\_v5\_gn\_4\_105921.1\_Q=Lsa-miR172b\_S=770**

category=2\_p=0.99356807369006

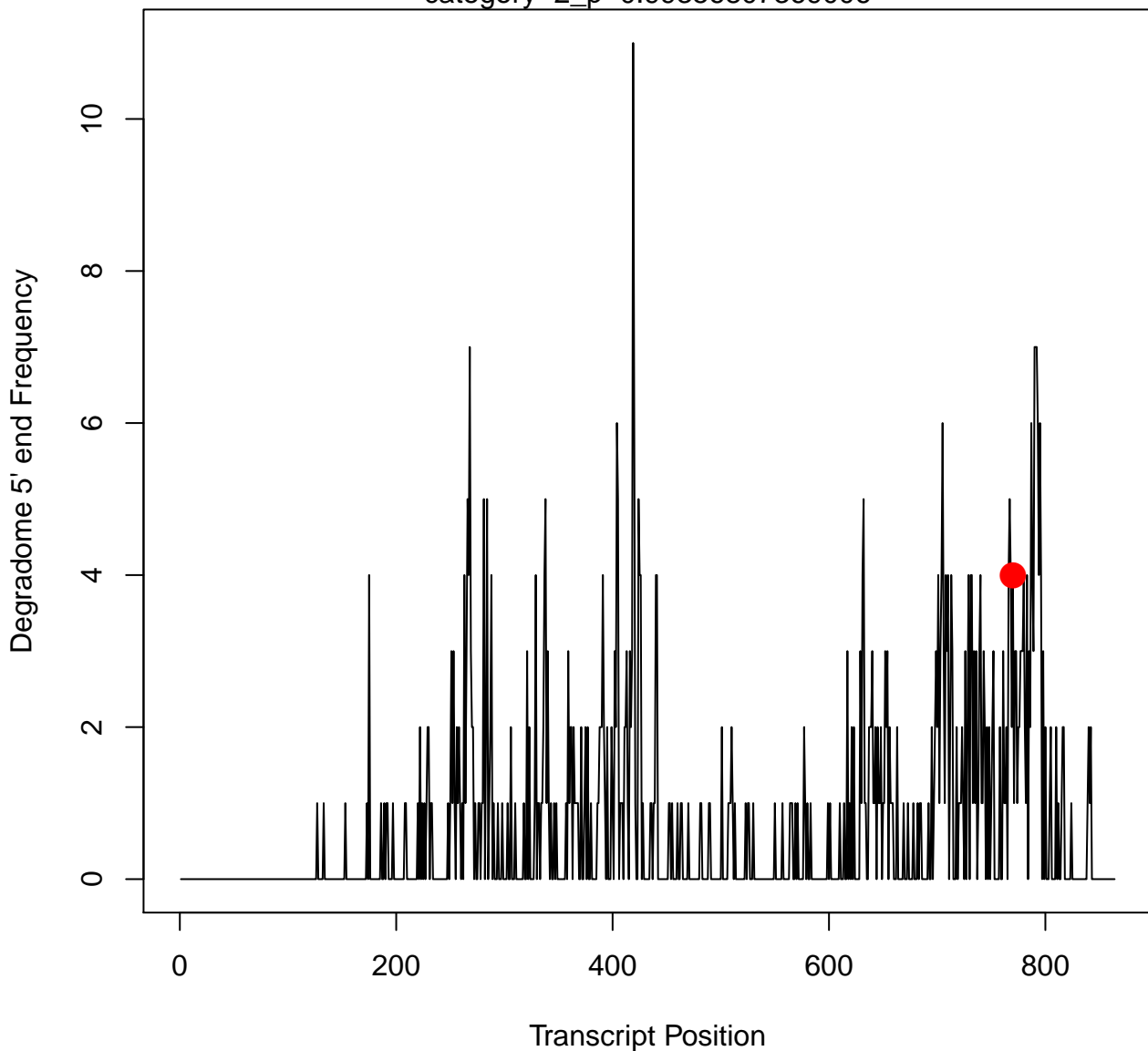

Supplement: Supplementary file 3 [file Data_Sheet_9.ZIP › GSM2230751.plot/Lsa-miR172b_Lsat_1_v5_gn_4_105921.1_770_TPlot.pdf]

**T=Lsat\_1\_v5\_gn\_5\_79461.1\_Q=Lsa-miR172b\_S=428**

category=2\_p=0.904165209342093

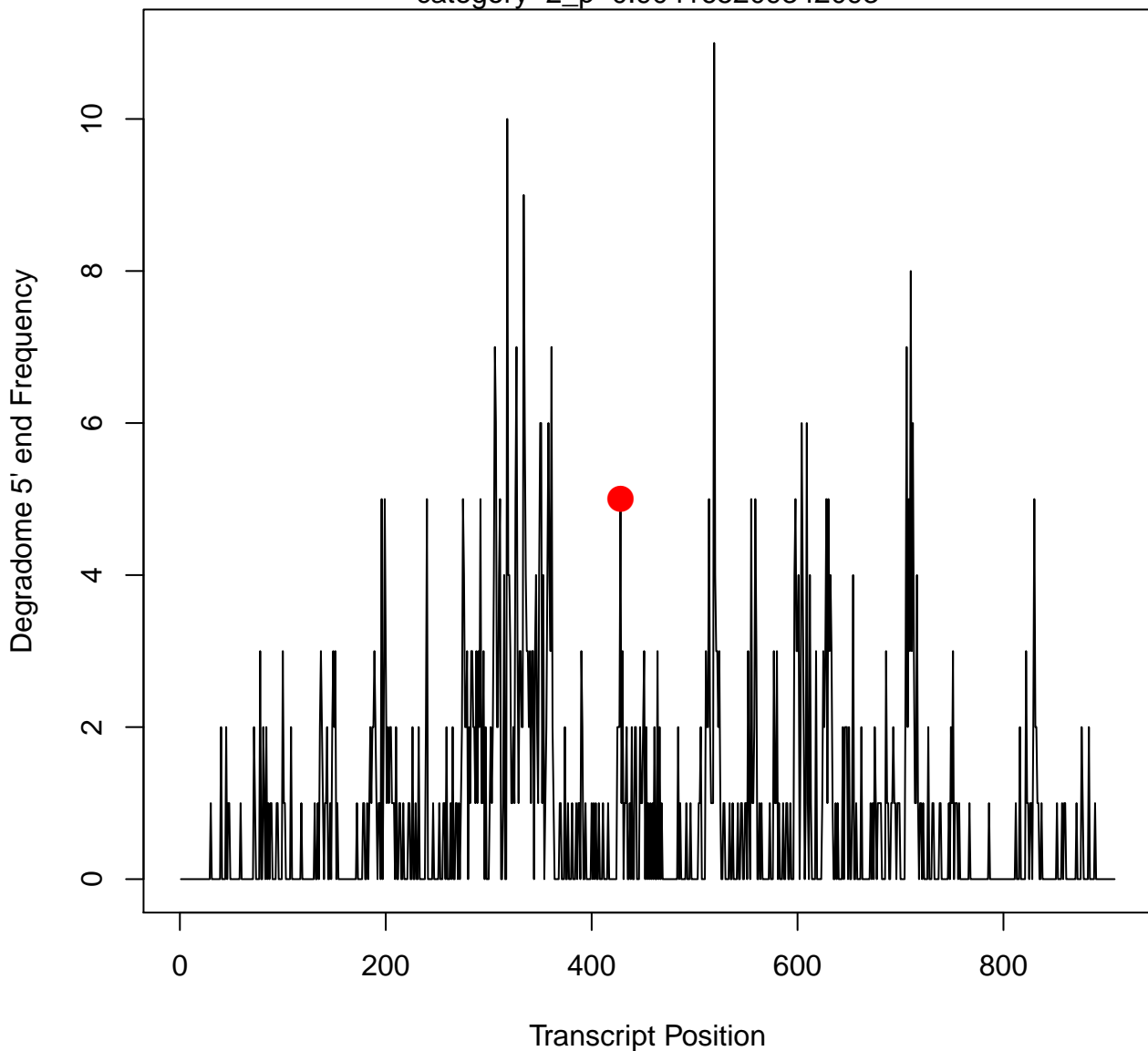

Supplement: Supplementary file 3 [file Data_Sheet_9.ZIP › GSM2230751.plot/Lsa-miR172b_Lsat_1_v5_gn_5_79461.1_428_TPlot.pdf]

**T=Lsat\_1\_v5\_gn\_8\_18040.1\_Q=Lsa-miR172b\_S=1210**

category=0\_p=0.000369544041020964

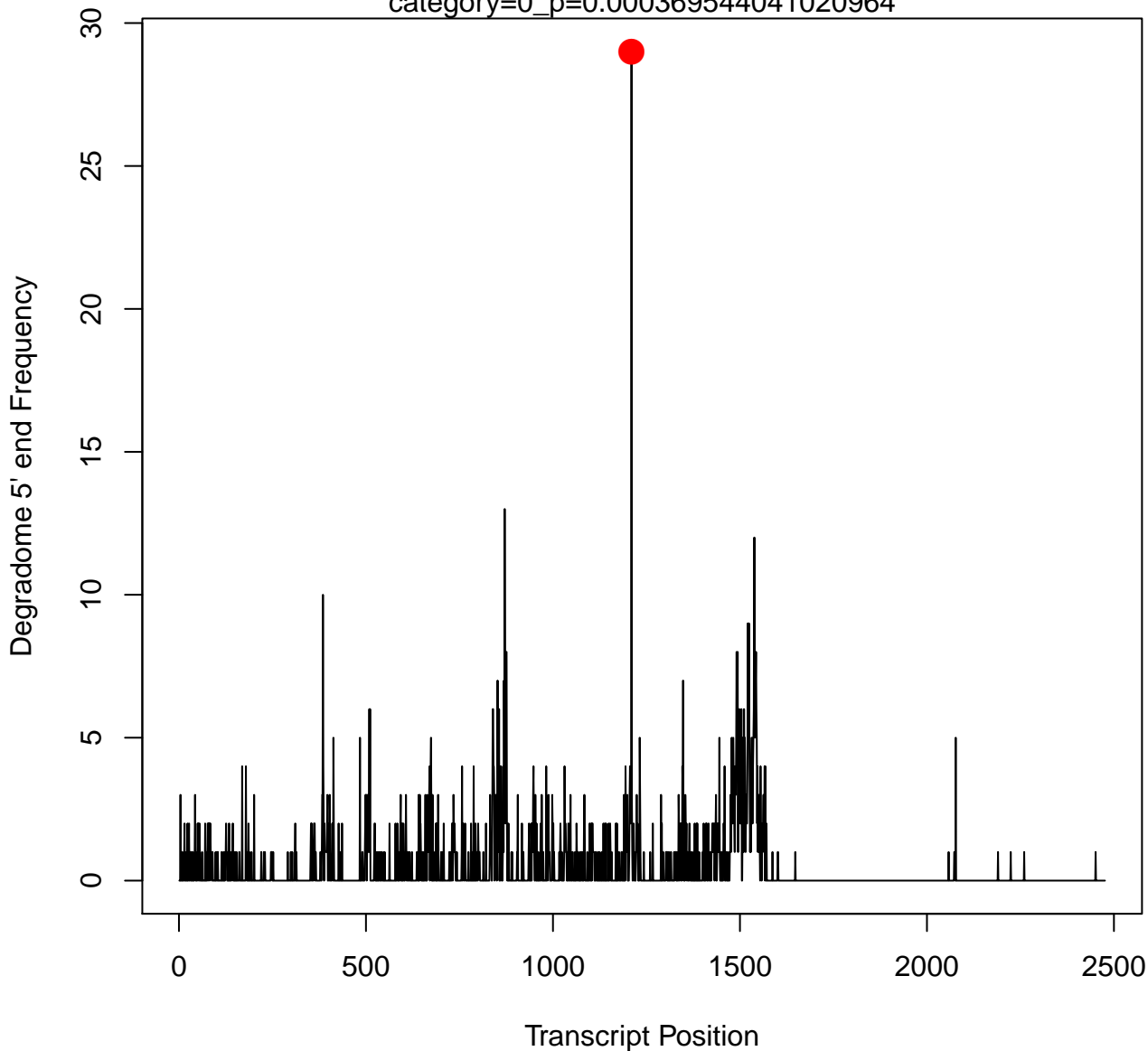

Supplement: Supplementary file 3 [file Data_Sheet_9.ZIP › GSM2230751.plot/Lsa-miR172b_Lsat_1_v5_gn_8_18040.1_1210_TPlot.pdf]

**T=Lsat\_1\_v5\_gn\_8\_72921.1\_Q=Lsa-miR172c\_S=1035**

category=0\_p=0.0879253803584356

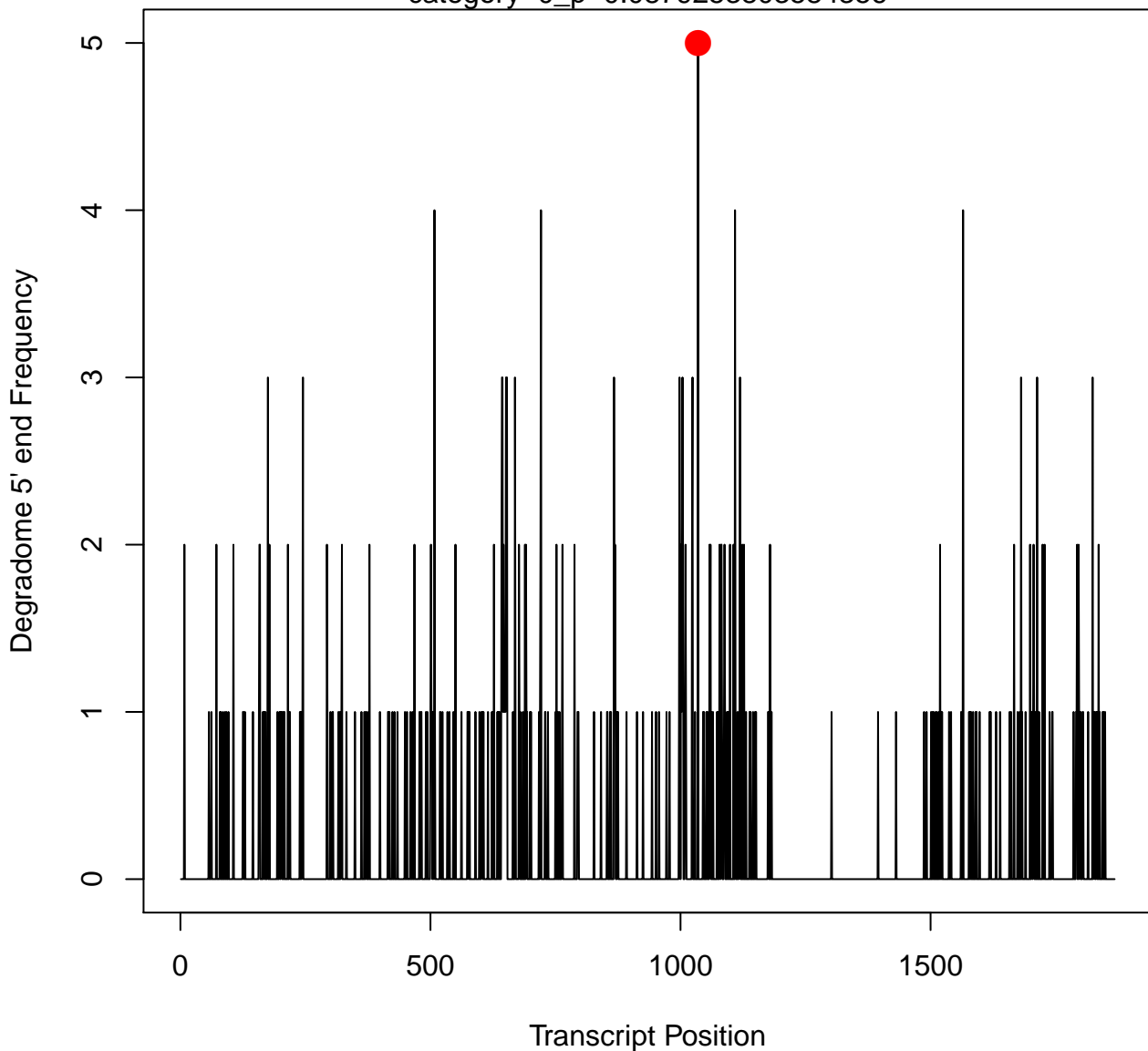

Supplement: Supplementary file 3 [file Data_Sheet_9.ZIP › GSM2230751.plot/Lsa-miR172c_Lsat_1_v5_gn_8_72921.1_1035_TPlot.pdf]

**T=Lsat\_1\_v5\_gn\_9\_64940.1\_Q=Lsa-miR172c\_S=775**

category=2\_p=0.999958630323943

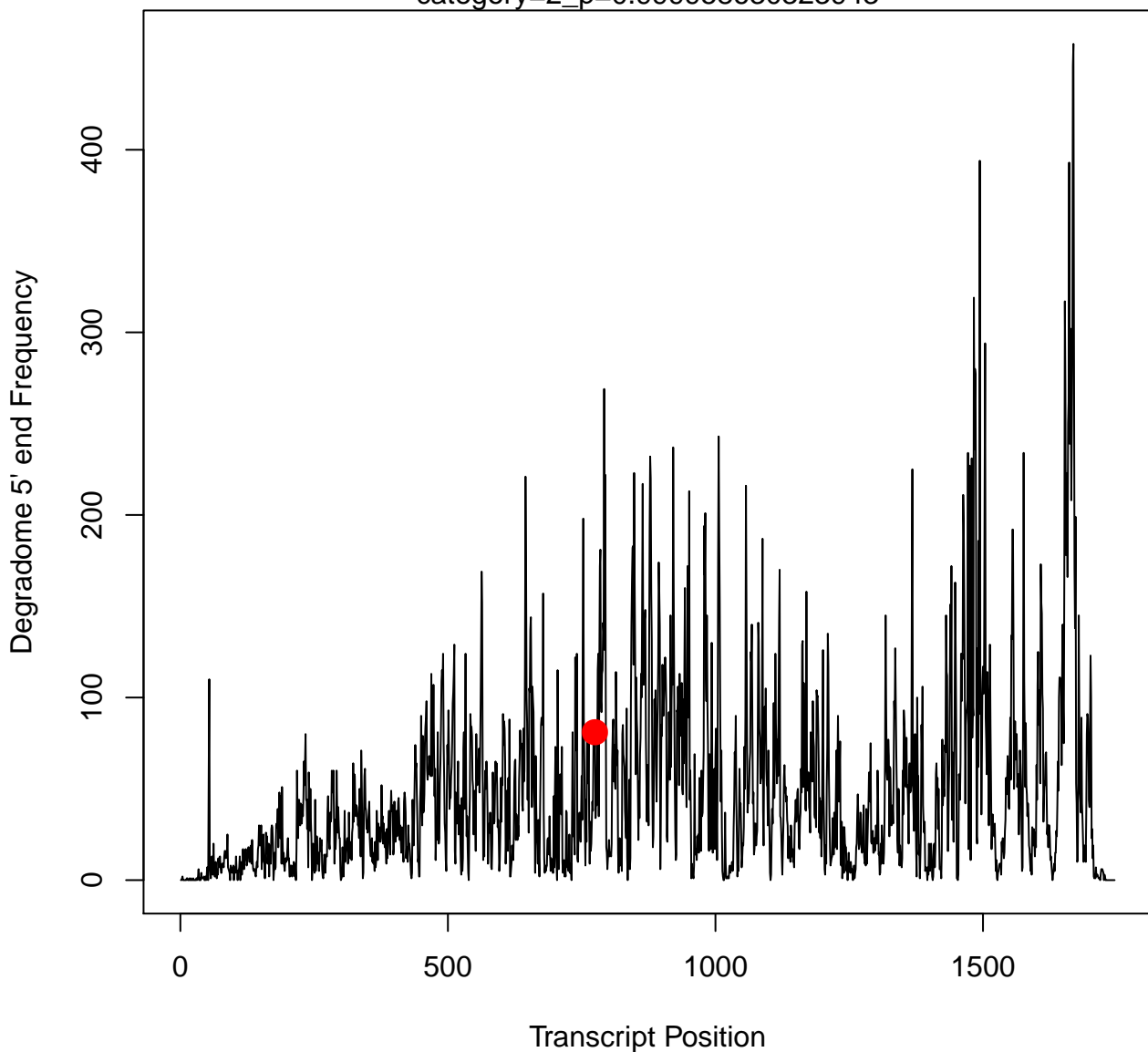

Supplement: Supplementary file 3 [file Data_Sheet_9.ZIP › GSM2230751.plot/Lsa-miR172c_Lsat_1_v5_gn_9_64940.1_775_TPlot.pdf]

**T=Lsat\_1\_v5\_gn\_0\_12040.1\_Q=Lsa-miR1871\_S=2235**

category=2\_p=0.973259938126807

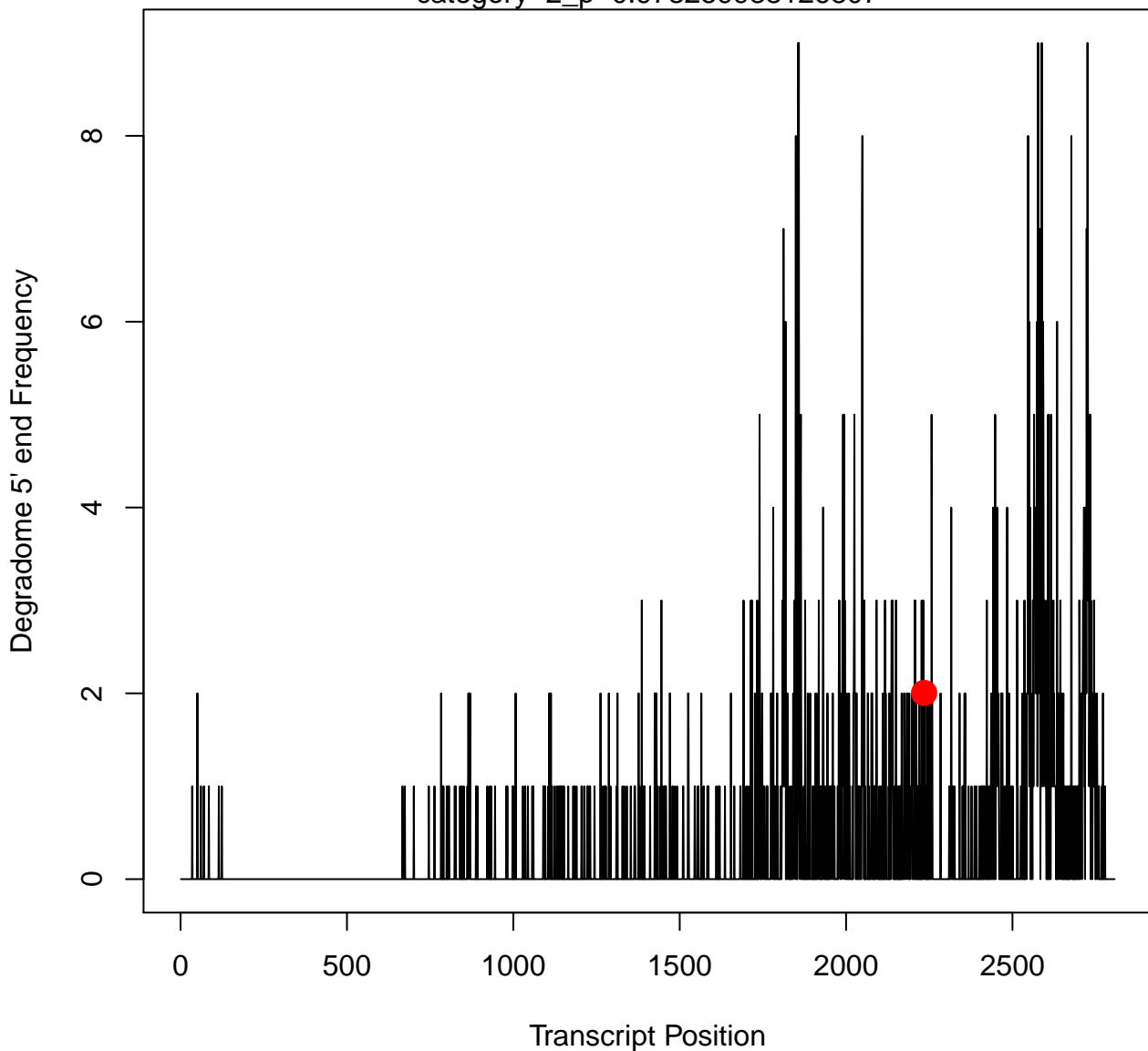

Supplement: Supplementary file 3 [file Data_Sheet_9.ZIP › GSM2230751.plot/Lsa-miR1871_Lsat_1_v5_gn_0_12040.1_2235_TPlot.pdf]

**T=Lsat\_1\_v5\_gn\_3\_121920.1\_Q=Lsa-miR1871\_S=3301**

category=2\_p=0.779959654583764

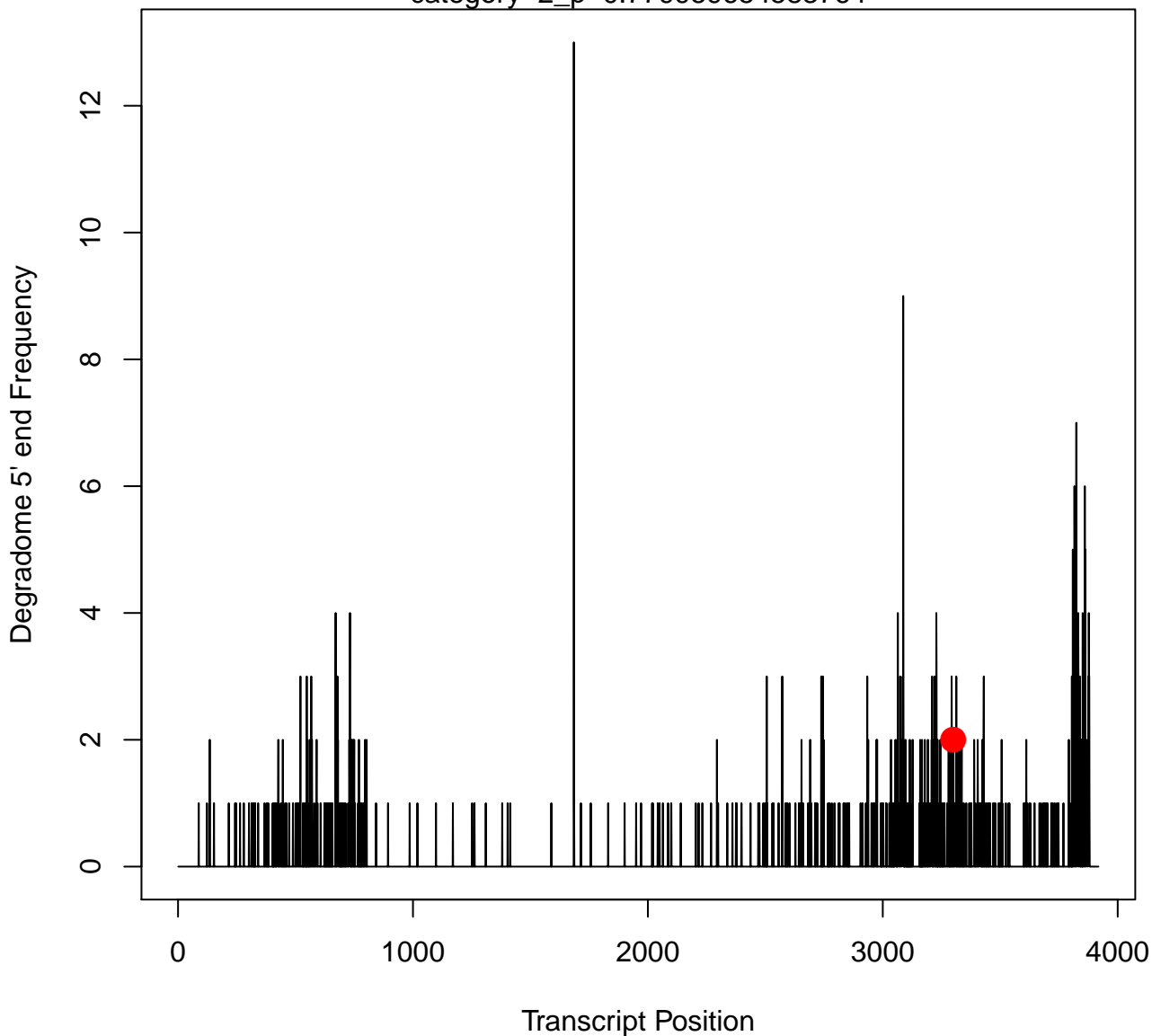

Supplement: Supplementary file 3 [file Data_Sheet_9.ZIP › GSM2230751.plot/Lsa-miR1871_Lsat_1_v5_gn_3_121920.1_3301_TPlot.pdf]

**T=Lsat\_1\_v5\_gn\_7\_9300.1\_Q=Lsa-miR1871\_S=1139**

category=2\_p=0.564467186794208

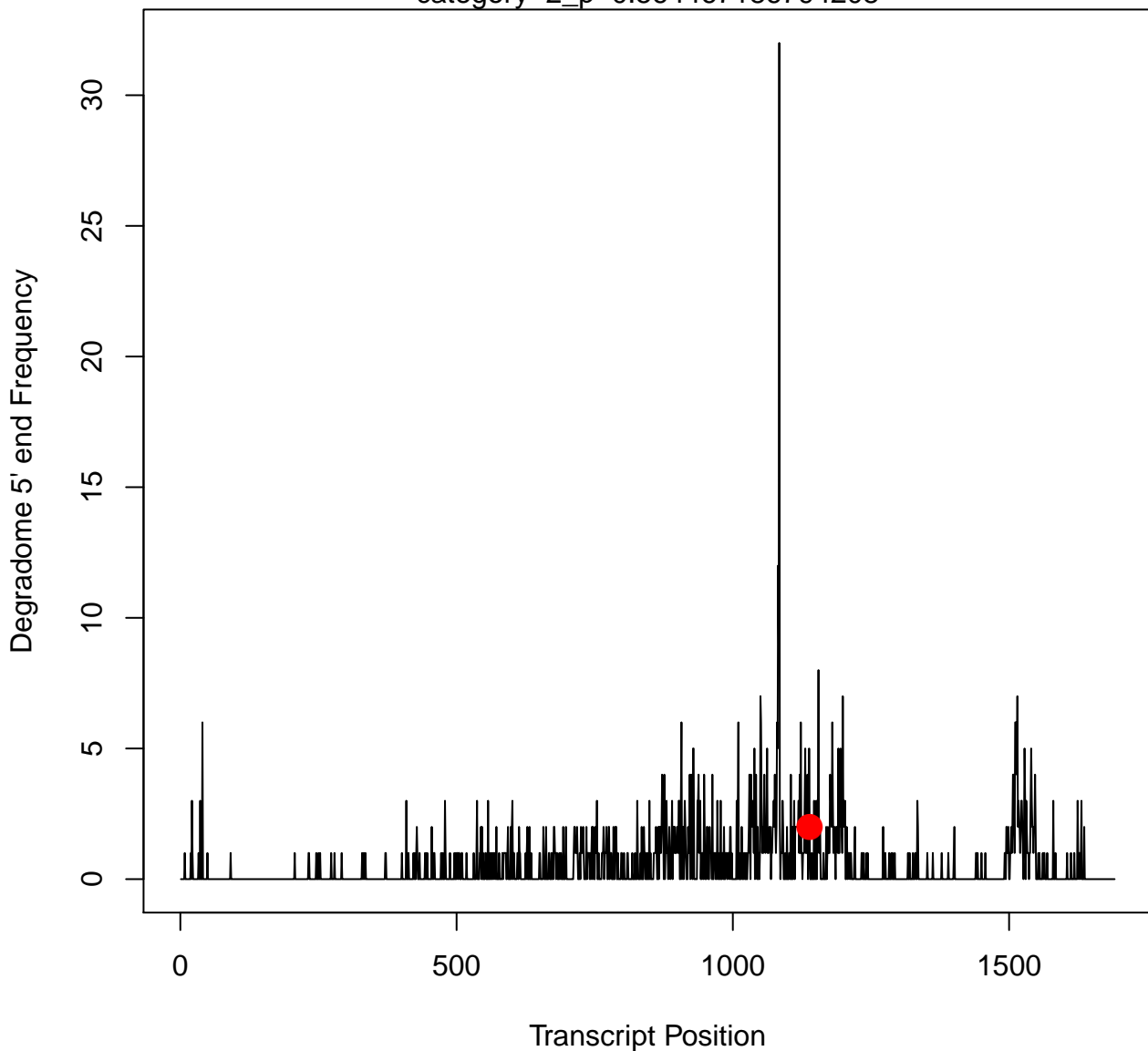

Supplement: Supplementary file 3 [file Data_Sheet_9.ZIP › GSM2230751.plot/Lsa-miR1871_Lsat_1_v5_gn_7_9300.1_1139_TPlot.pdf]

**T=Lsat\_1\_v5\_gn\_8\_12460.1\_Q=Lsa-miR1871\_S=978**

category=2\_p=0.97480129716358

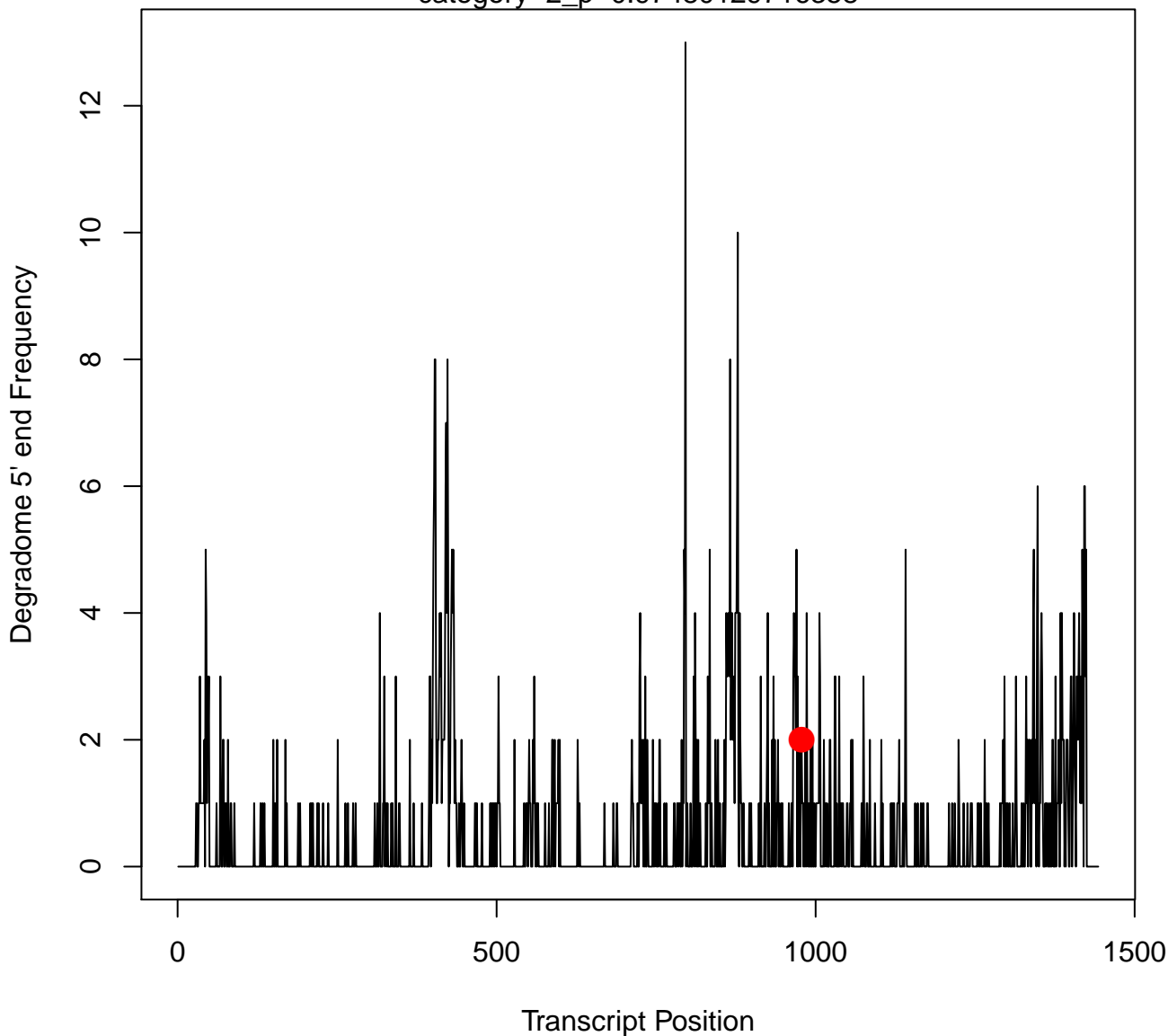

Supplement: Supplementary file 3 [file Data_Sheet_9.ZIP › GSM2230751.plot/Lsa-miR1871_Lsat_1_v5_gn_8_12460.1_978_TPlot.pdf]

**T=Lsat\_1\_v5\_gn\_8\_27820.1\_Q=Lsa-miR1871\_S=199**

category=2\_p=0.936753916257189

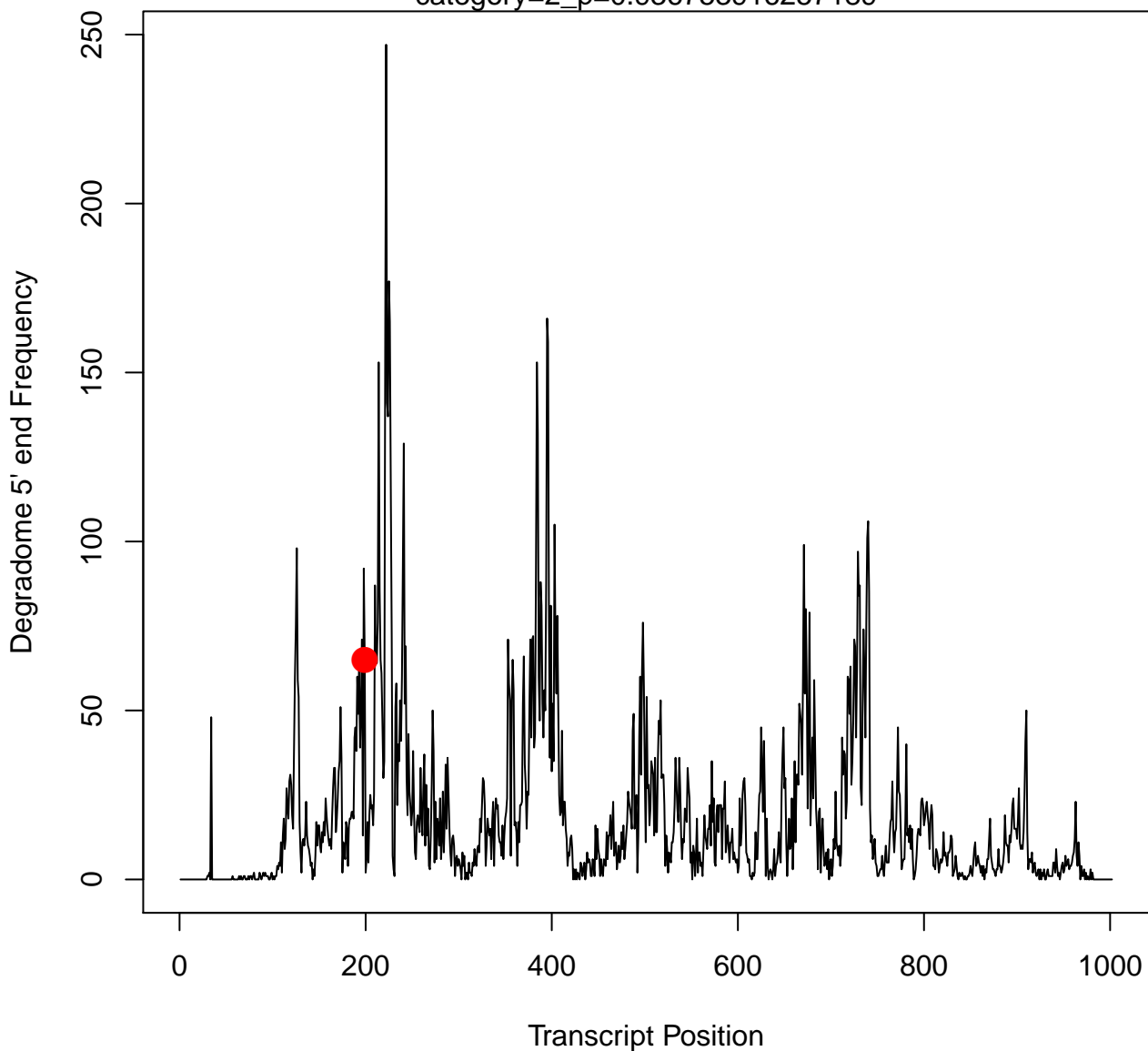

Supplement: Supplementary file 3 [file Data_Sheet_9.ZIP › GSM2230751.plot/Lsa-miR1871_Lsat_1_v5_gn_8_27820.1_199_TPlot.pdf]

**T=Lsat\_1\_v5\_gn\_8\_45621.1\_Q=Lsa-miR1871\_S=562**

category=2\_p=0.93484830030796

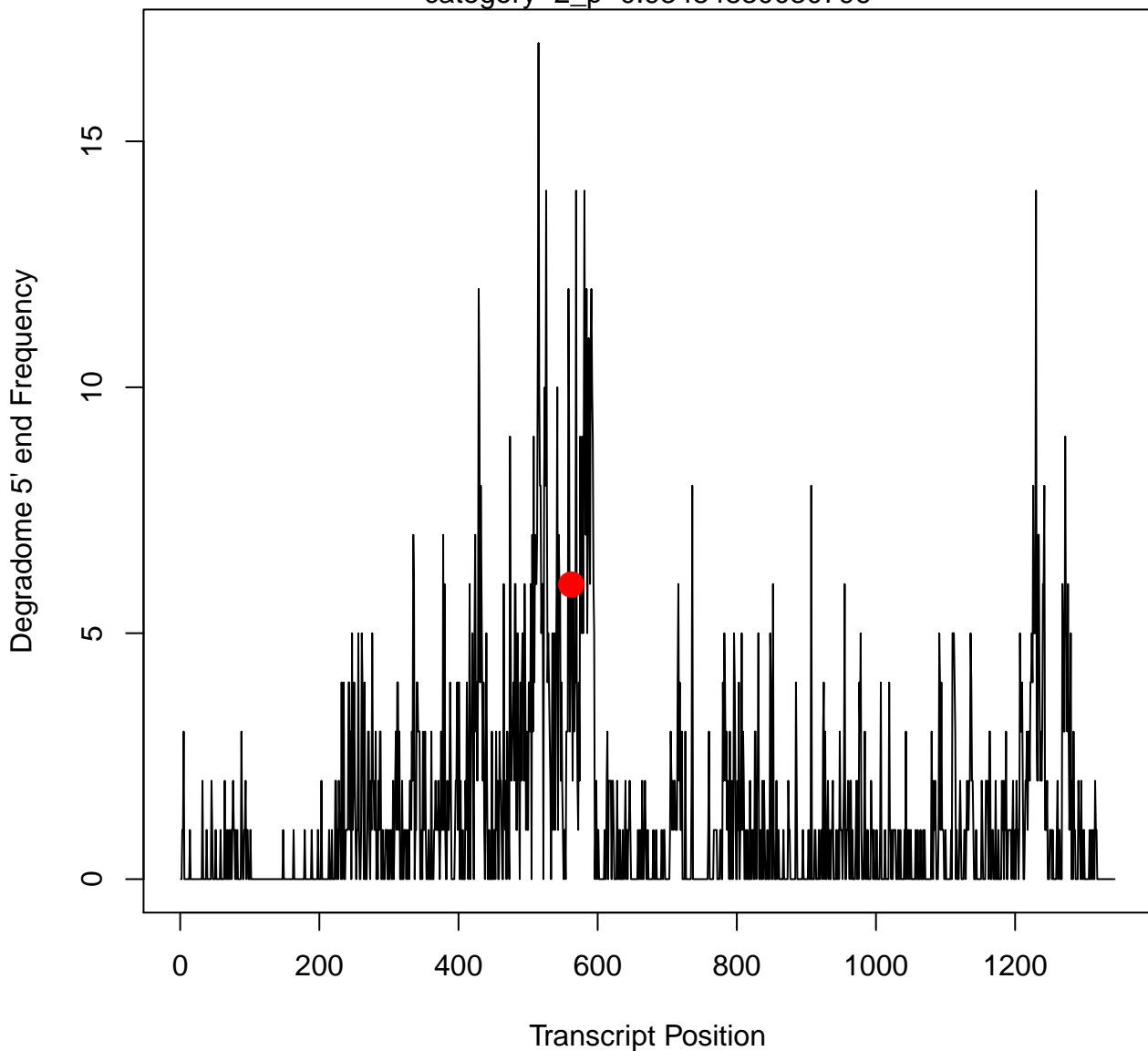

Supplement: Supplementary file 3 [file Data_Sheet_9.ZIP › GSM2230751.plot/Lsa-miR1871_Lsat_1_v5_gn_8_45621.1_562_TPlot.pdf]

**T=Lsat\_1\_v5\_gn\_1\_100400.1\_Q=Lsa-miR2111\_S=320**

category=2\_p=0.999731536567232

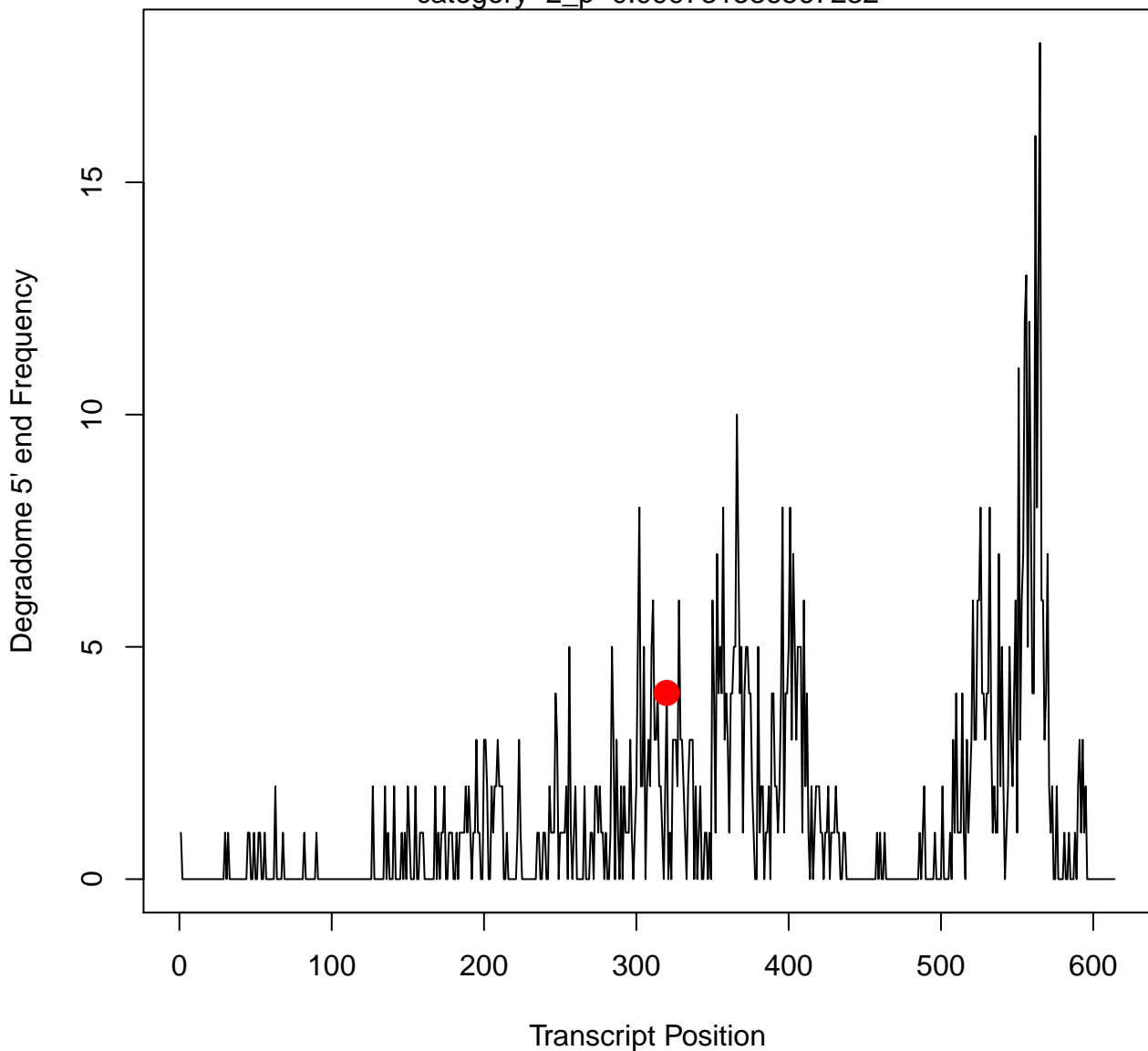

Supplement: Supplementary file 3 [file Data_Sheet_9.ZIP › GSM2230751.plot/Lsa-miR2111_Lsat_1_v5_gn_1_100400.1_320_TPlot.pdf]

T=Lsat\_1\_v5\_gn\_2\_134381.1\_Q=Lsa-miR2111\_S=217

category=2\_p=0.992968995201593

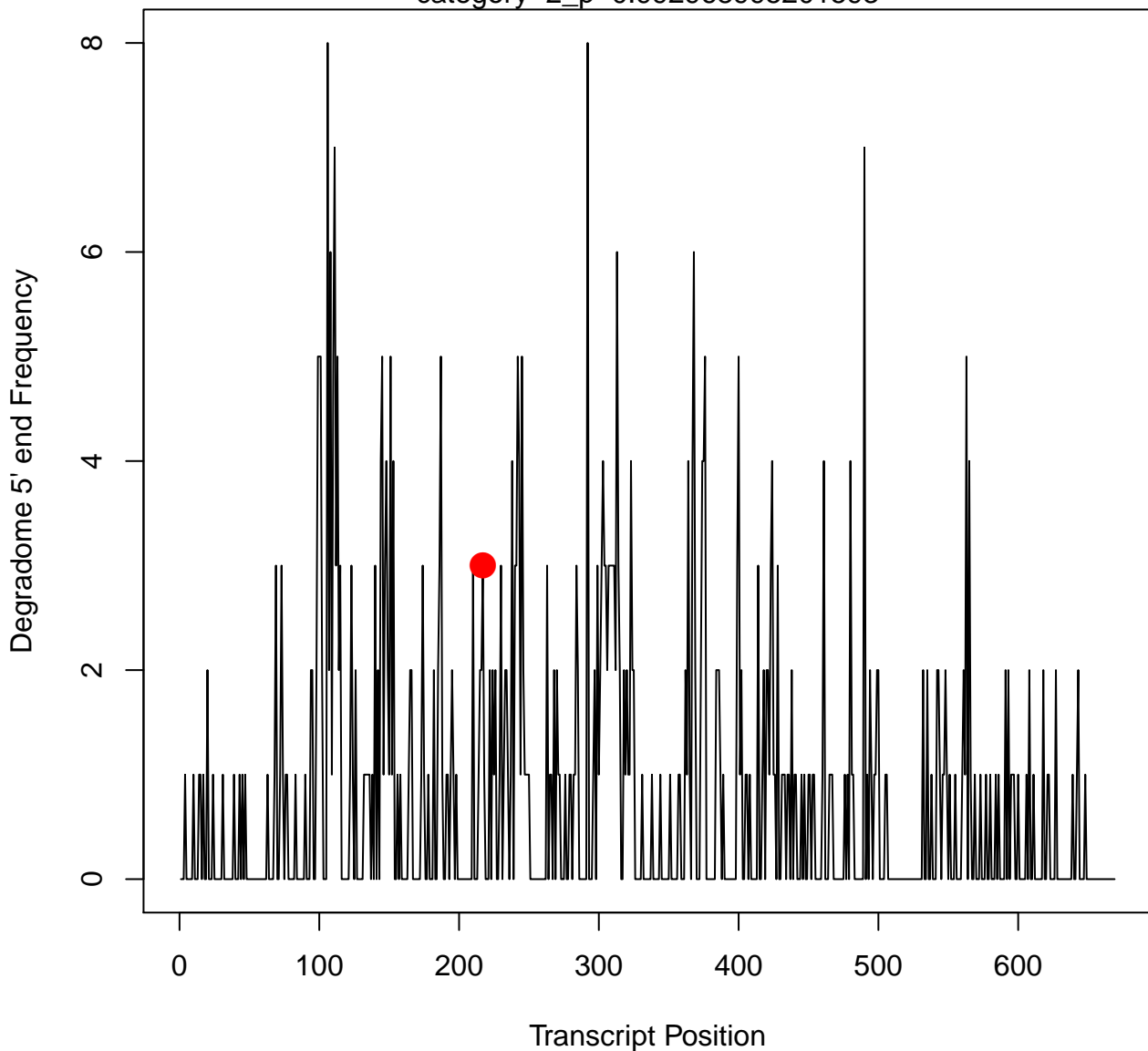

Supplement: Supplementary file 3 [file Data_Sheet_9.ZIP › GSM2230751.plot/Lsa-miR2111_Lsat_1_v5_gn_2_134381.1_217_TPlot.pdf]

**T=Lsat\_1\_v5\_gn\_3\_100.1\_Q=Lsa-miR2111\_S=4718**

category=2\_p=0.995220087506694

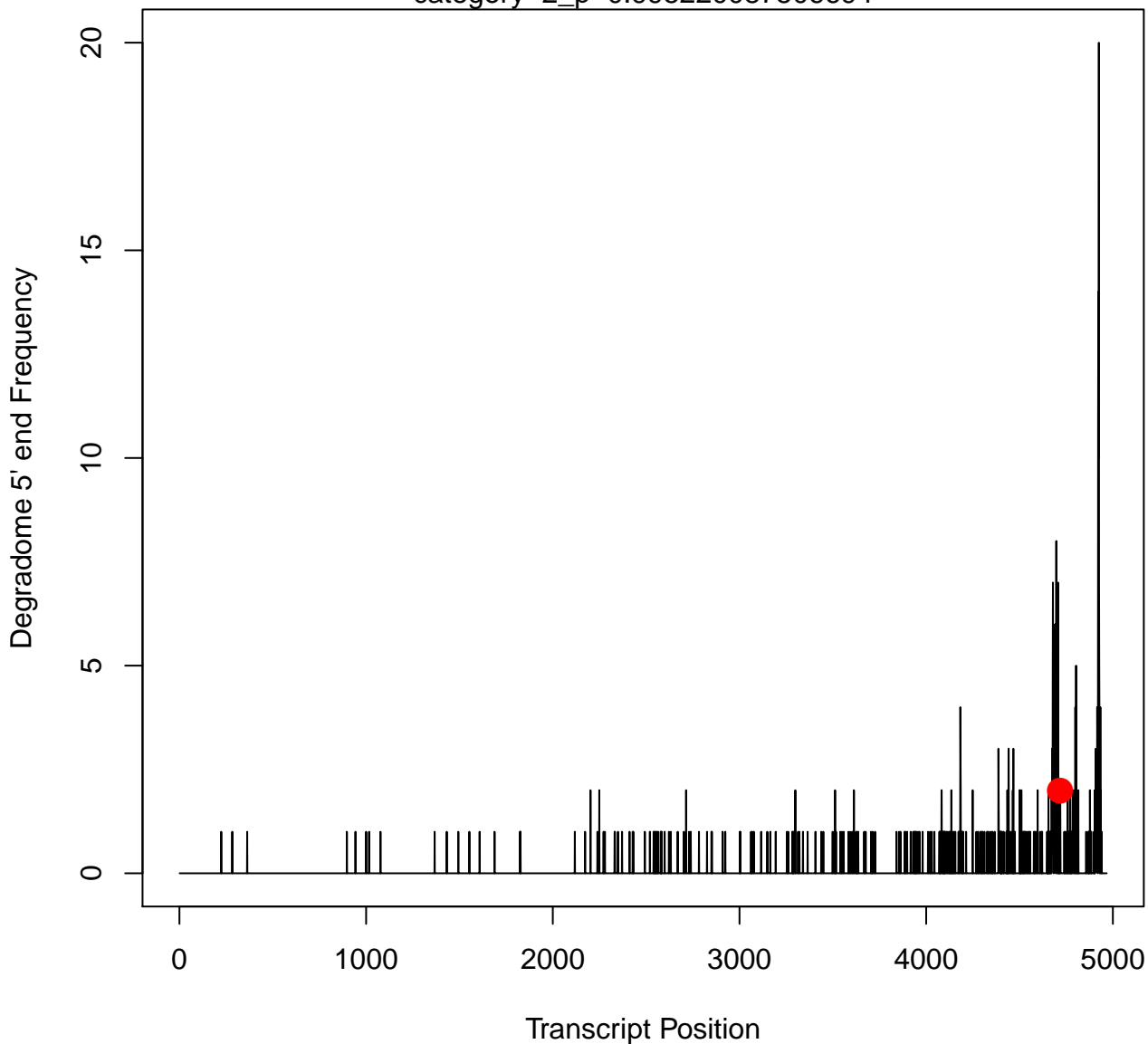

Supplement: Supplementary file 3 [file Data_Sheet_9.ZIP › GSM2230751.plot/Lsa-miR2111_Lsat_1_v5_gn_3_100.1_4718_TPlot.pdf]

**T=Lsat\_1\_v5\_gn\_4\_104321.1\_Q=Lsa-miR2111\_S=742**

category=2\_p=0.970769331879693

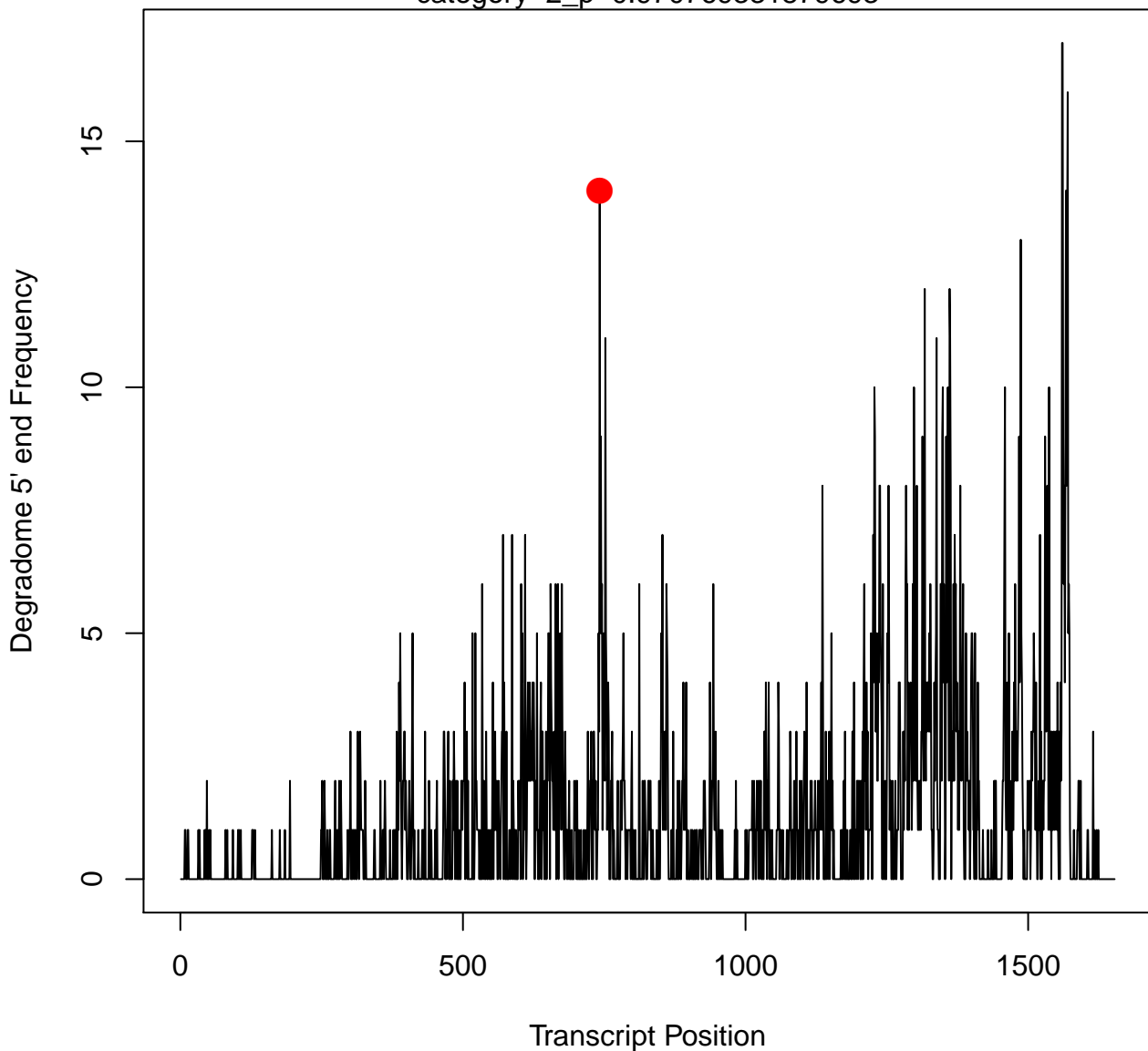

Supplement: Supplementary file 3 [file Data_Sheet_9.ZIP › GSM2230751.plot/Lsa-miR2111_Lsat_1_v5_gn_4_104321.1_742_TPlot.pdf]
