# Supplementary figures and images for: Comprehensive Annotation and Functional Exploration of MicroRNAs in Lettuce (part 3 of 6)
Source: Front Plant Sci. 2021 Dec 24;12:781836. doi: 10.3389/fpls.2021.781836 (PMC8739914; doi:10.3389/fpls.2021.781836)

**T=Lsat\_1\_v5\_gn\_2\_133880.1\_Q=Lsa-miR1446\_S=1251**

category=2\_p=0.278582331663879

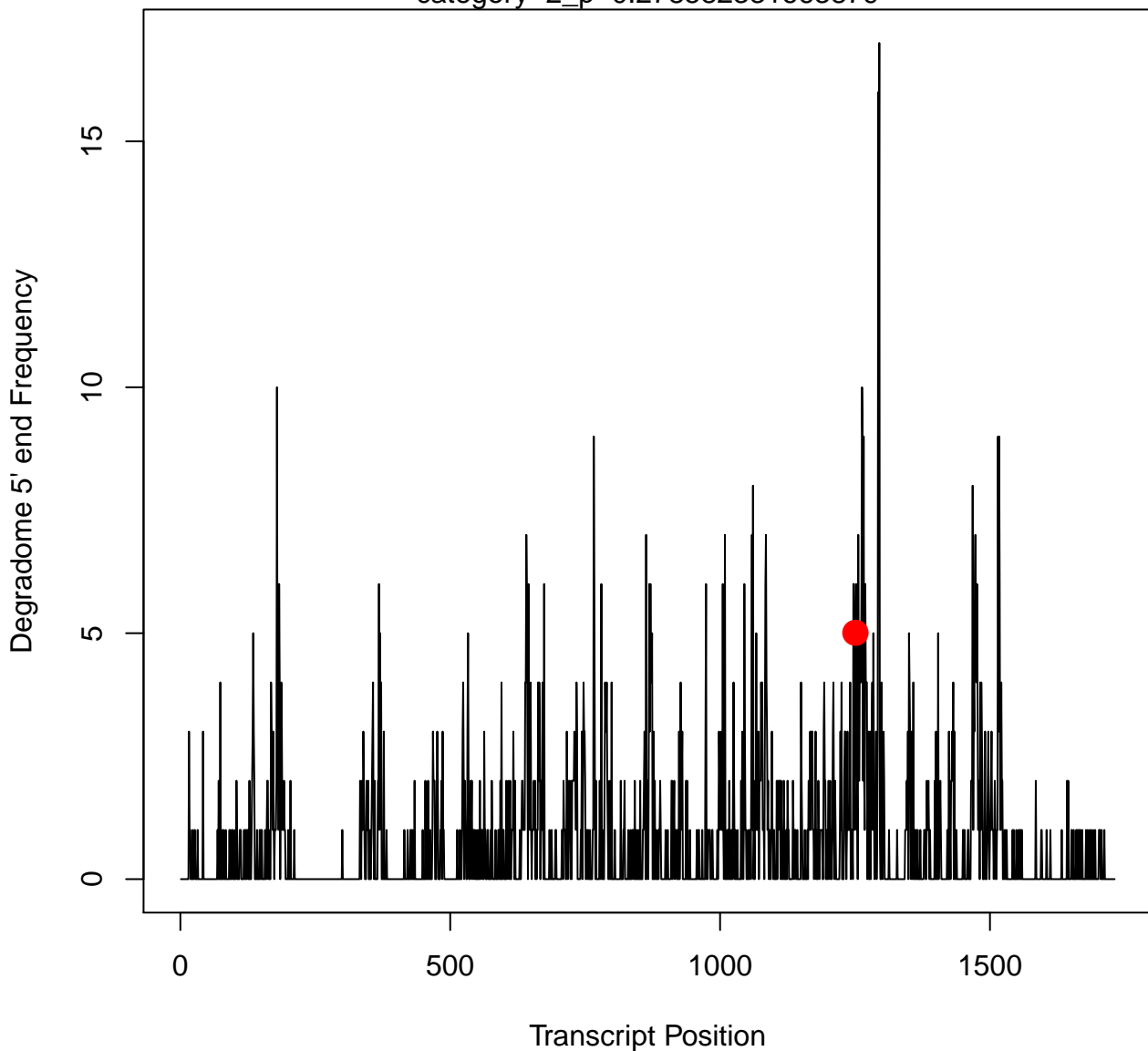

Supplement: Supplementary file 3 [file Data_Sheet_9.ZIP › GSM2230751.plot/Lsa-miR1446_Lsat_1_v5_gn_2_133880.1_1251_TPlot.pdf]

**T=Lsat\_1\_v5\_gn\_2\_91621.1\_Q=Lsa-miR1446\_S=2389**

category=2\_p=0.99906598507003

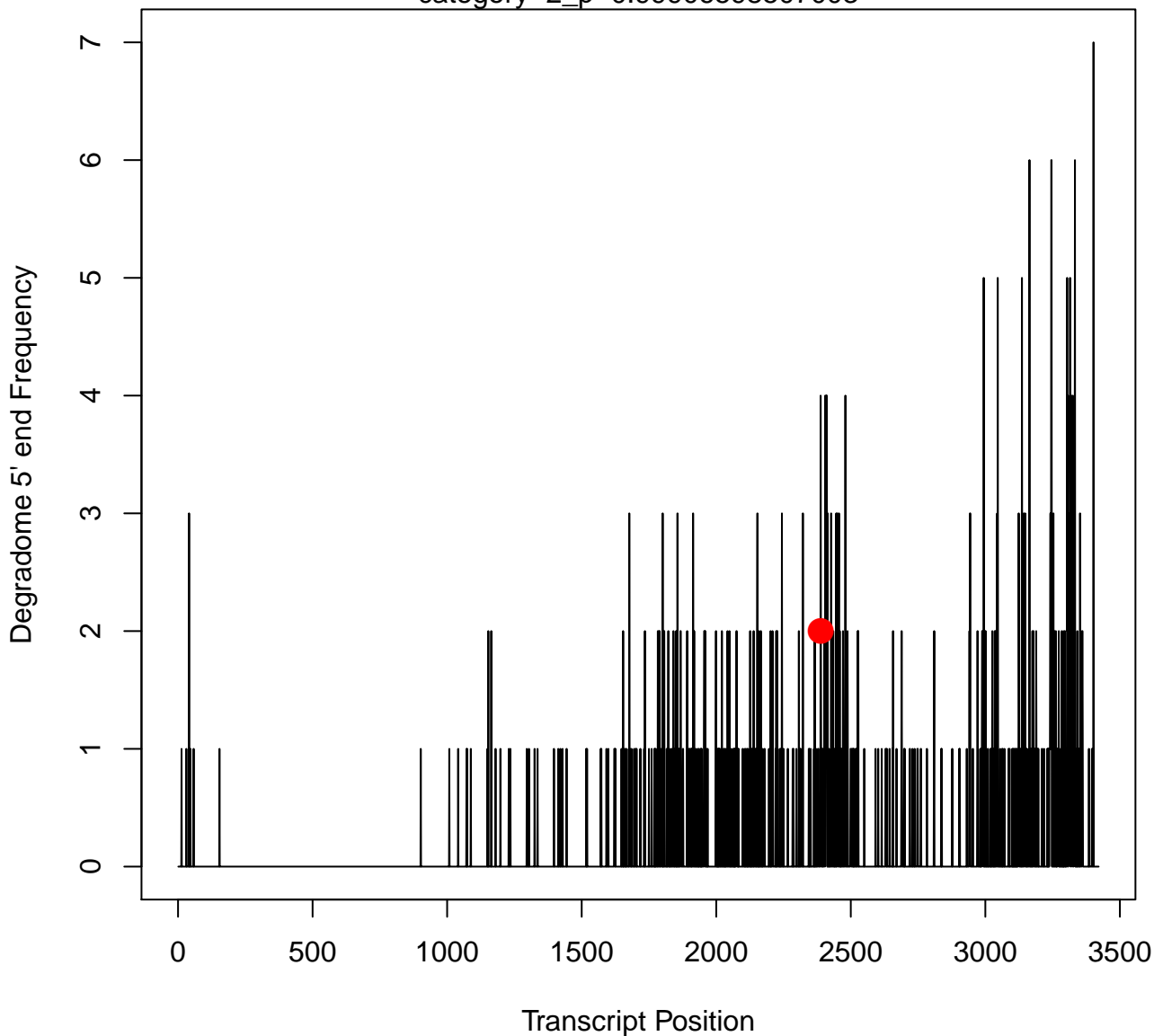

Supplement: Supplementary file 3 [file Data_Sheet_9.ZIP › GSM2230751.plot/Lsa-miR1446_Lsat_1_v5_gn_2_91621.1_2389_TPlot.pdf]

**T=Lsat\_1\_v5\_gn\_3\_102160.1\_Q=Lsa-miR1446\_S=1169**

category=2\_p=0.413940116262911

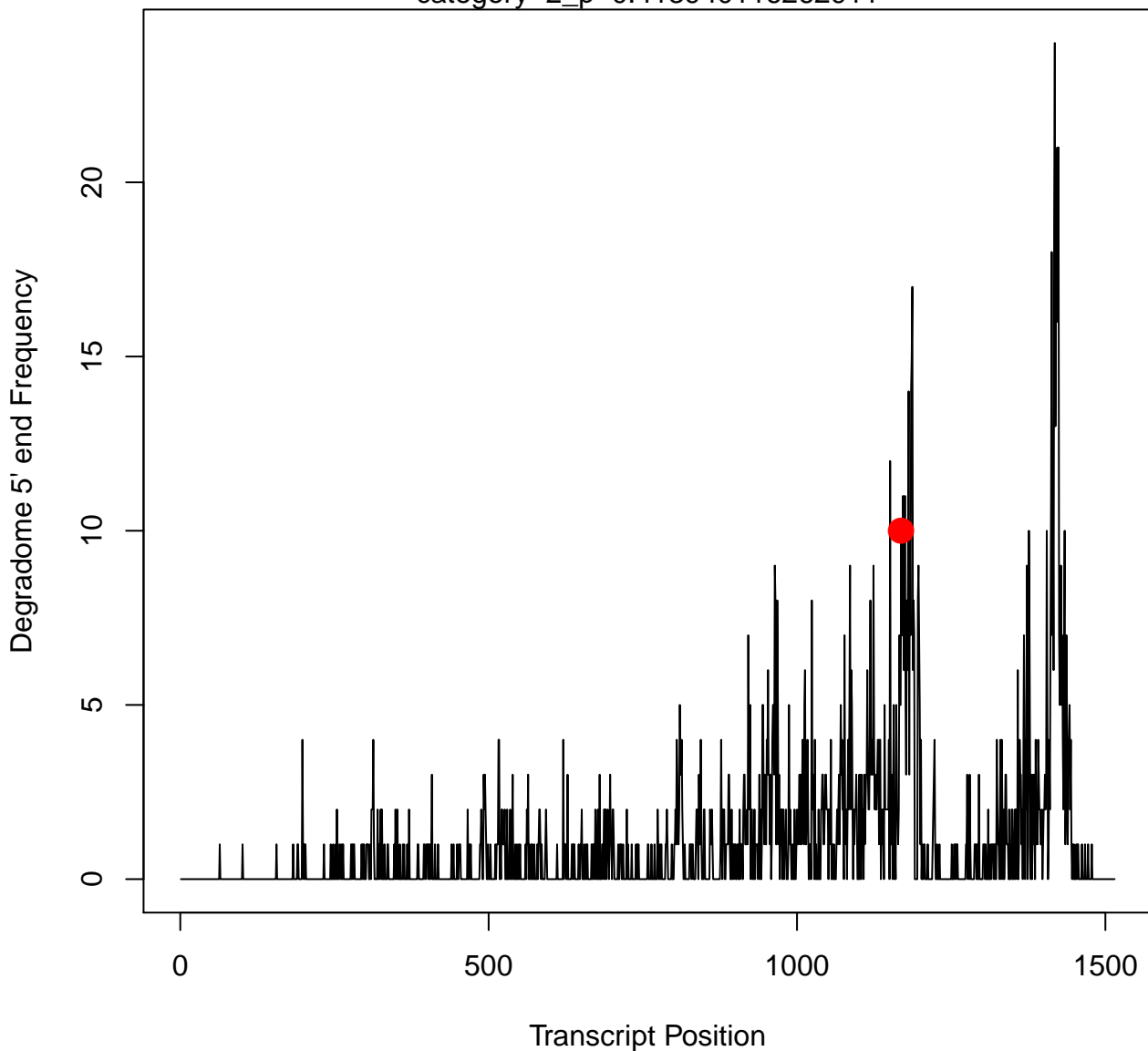

Supplement: Supplementary file 3 [file Data_Sheet_9.ZIP › GSM2230751.plot/Lsa-miR1446_Lsat_1_v5_gn_3_102160.1_1169_TPlot.pdf]

**T=Lsat\_1\_v5\_gn\_3\_32820.1\_Q=Lsa-miR1446\_S=1167**

category=2\_p=0.589572305870943

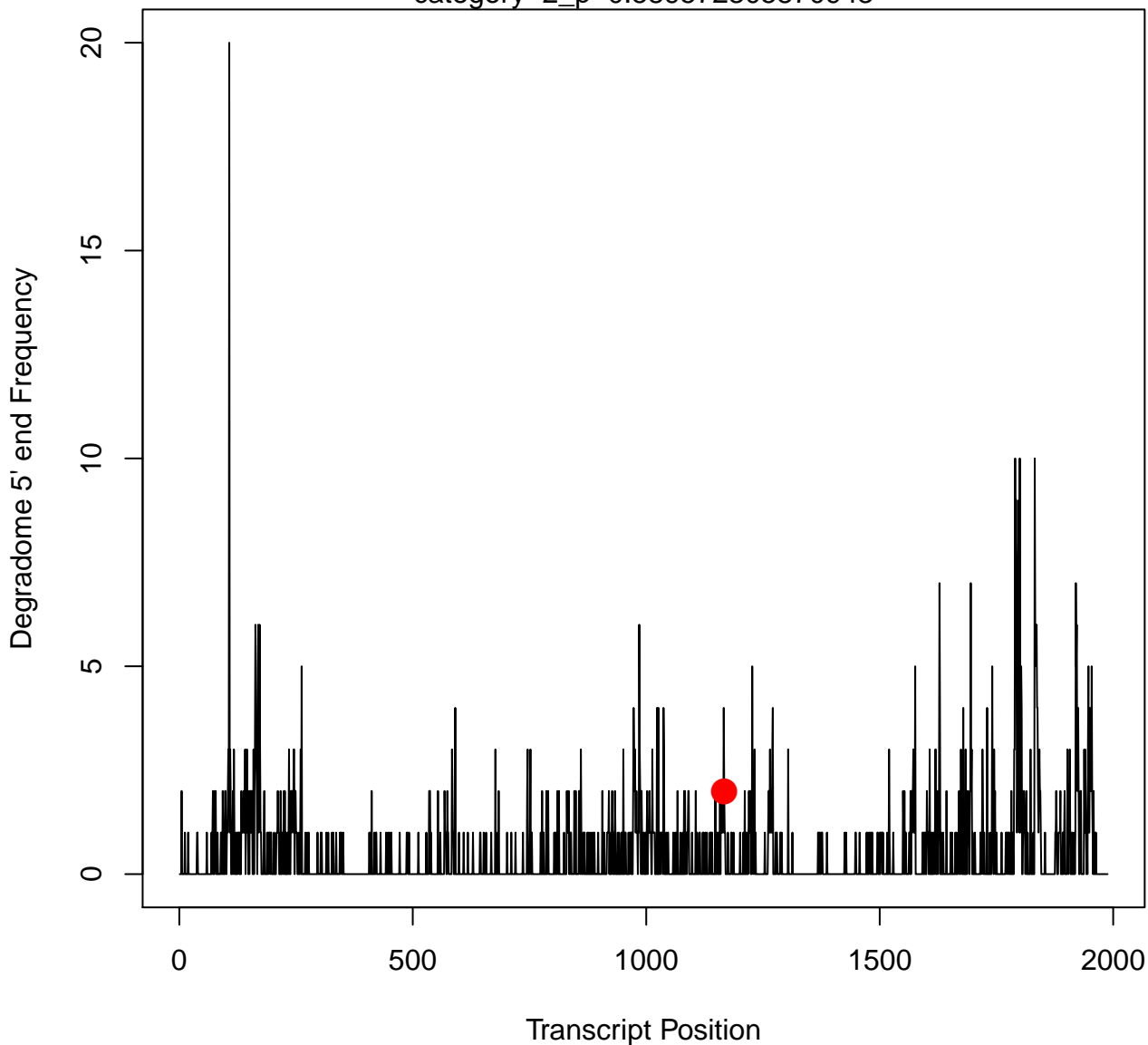

Supplement: Supplementary file 3 [file Data_Sheet_9.ZIP › GSM2230751.plot/Lsa-miR1446_Lsat_1_v5_gn_3_32820.1_1167_TPlot.pdf]

**T=Lsat\_1\_v5\_gn\_3\_48061.1\_Q=Lsa-miR1446\_S=2426**

category=2\_p=0.999995915890006

Degradome 5' end Frequency

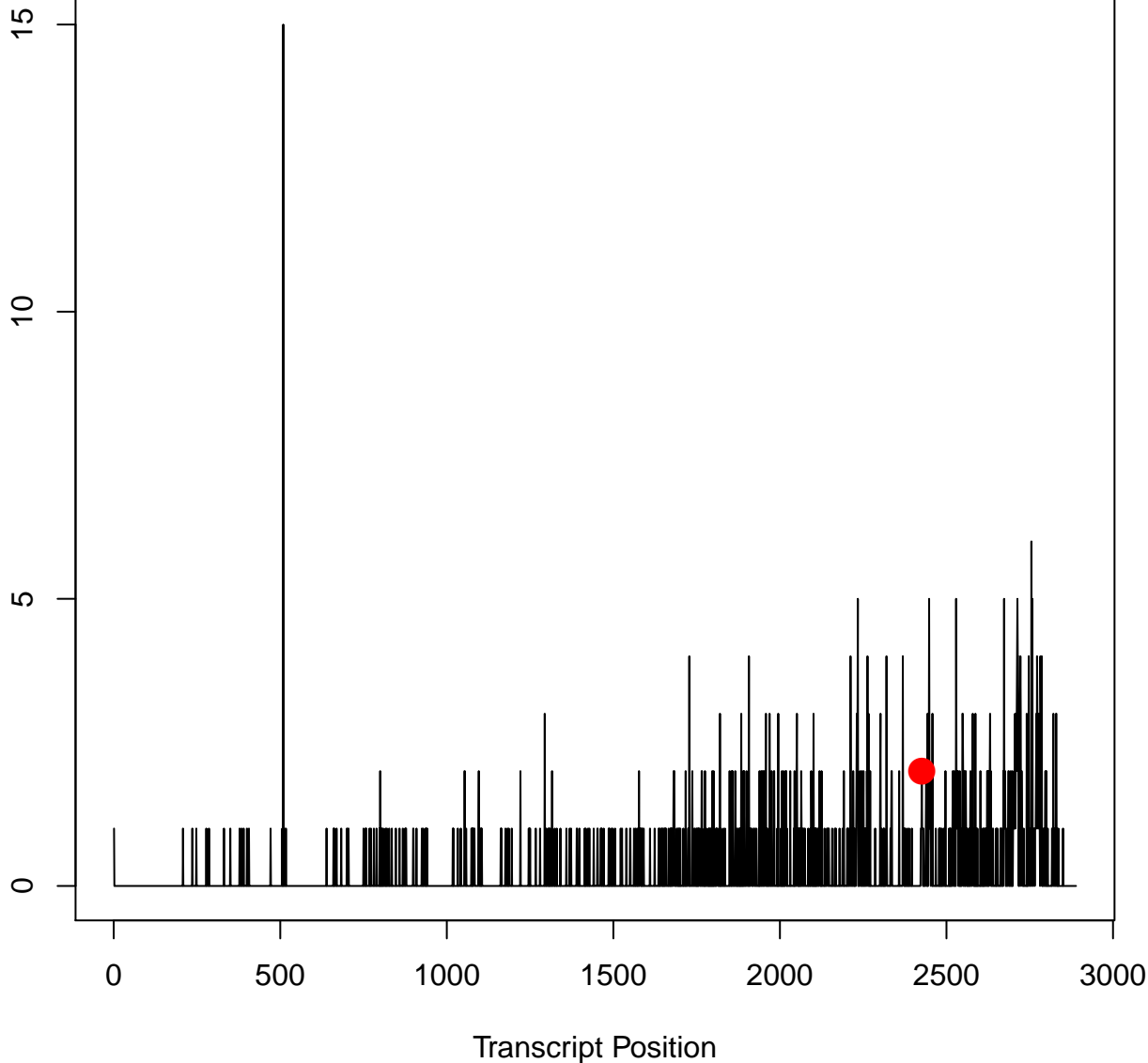

Supplement: Supplementary file 3 [file Data_Sheet_9.ZIP › GSM2230751.plot/Lsa-miR1446_Lsat_1_v5_gn_3_48061.1_2426_TPlot.pdf]

**T=Lsat\_1\_v5\_gn\_3\_53980.1\_Q=Lsa-miR1446\_S=441**

category=2\_p=0.972454255223244

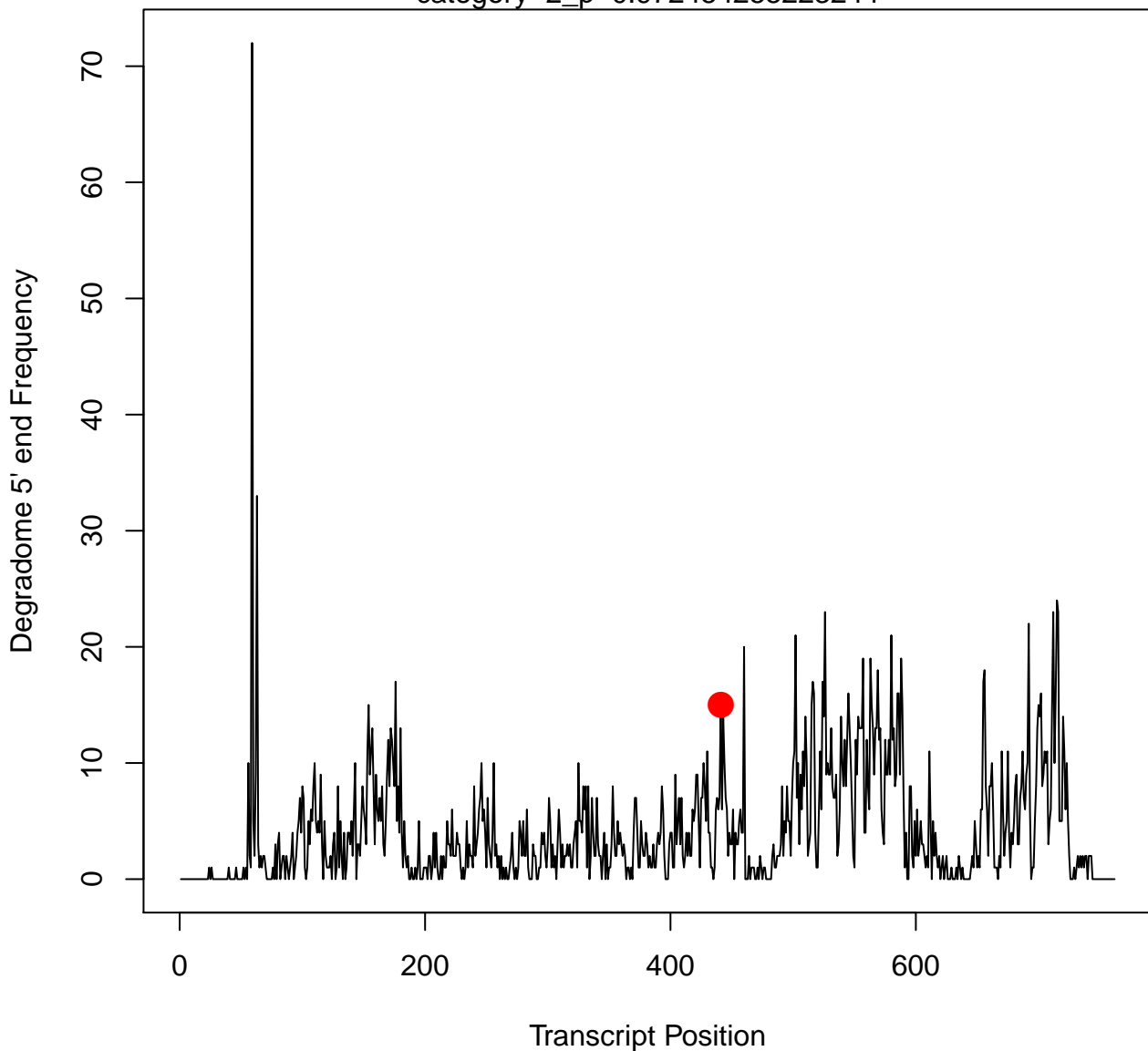

Supplement: Supplementary file 3 [file Data_Sheet_9.ZIP › GSM2230751.plot/Lsa-miR1446_Lsat_1_v5_gn_3_53980.1_441_TPlot.pdf]

**T=Lsat\_1\_v5\_gn\_3\_61481.1\_Q=Lsa-miR1446\_S=3952**

category=2\_p=0.994617446048508

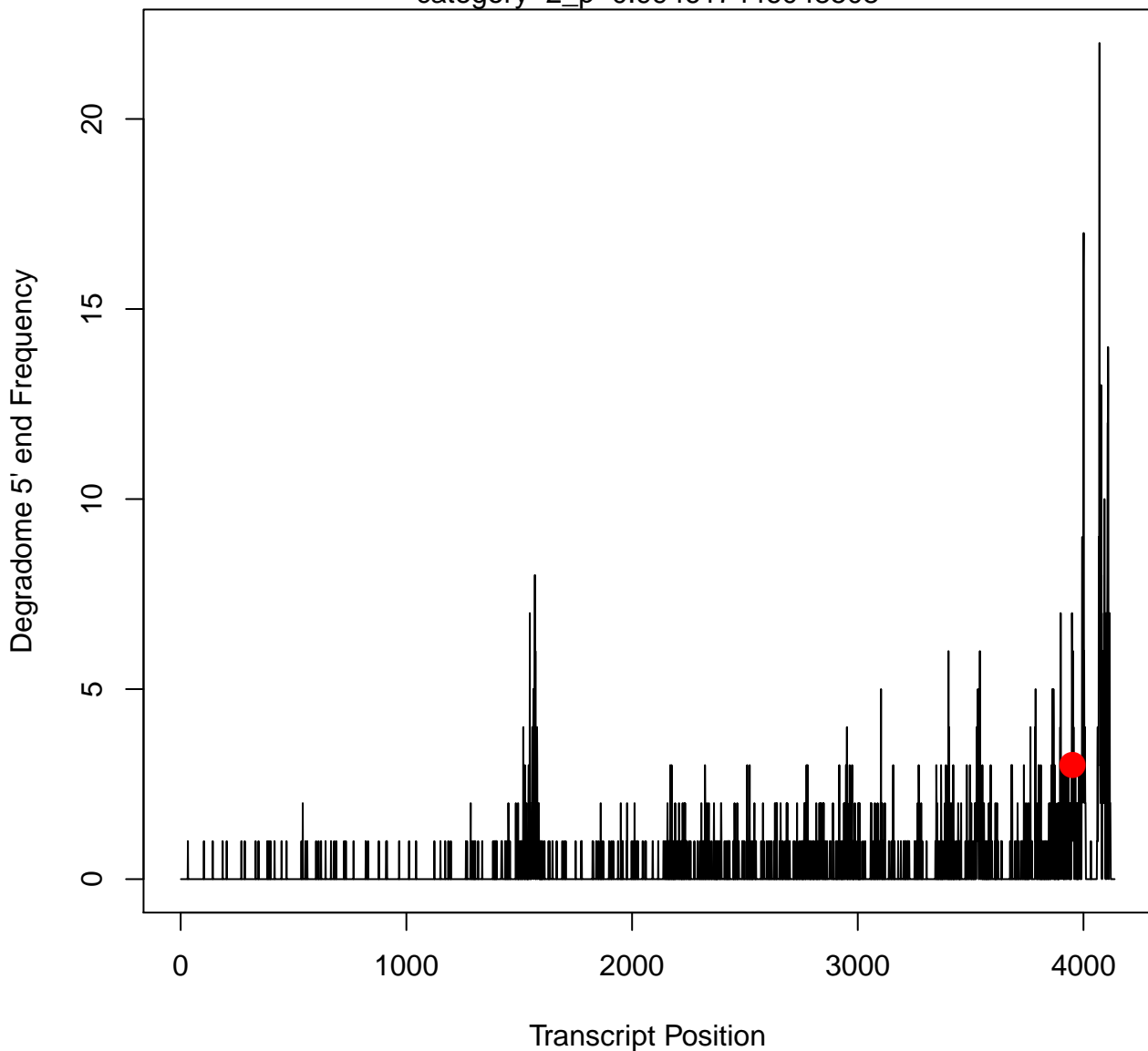

Supplement: Supplementary file 3 [file Data_Sheet_9.ZIP › GSM2230751.plot/Lsa-miR1446_Lsat_1_v5_gn_3_61481.1_3952_TPlot.pdf]

**T=Lsat\_1\_v5\_gn\_4\_170480.1\_Q=Lsa-miR1446\_S=2205**

category=2\_p=0.999931475162524

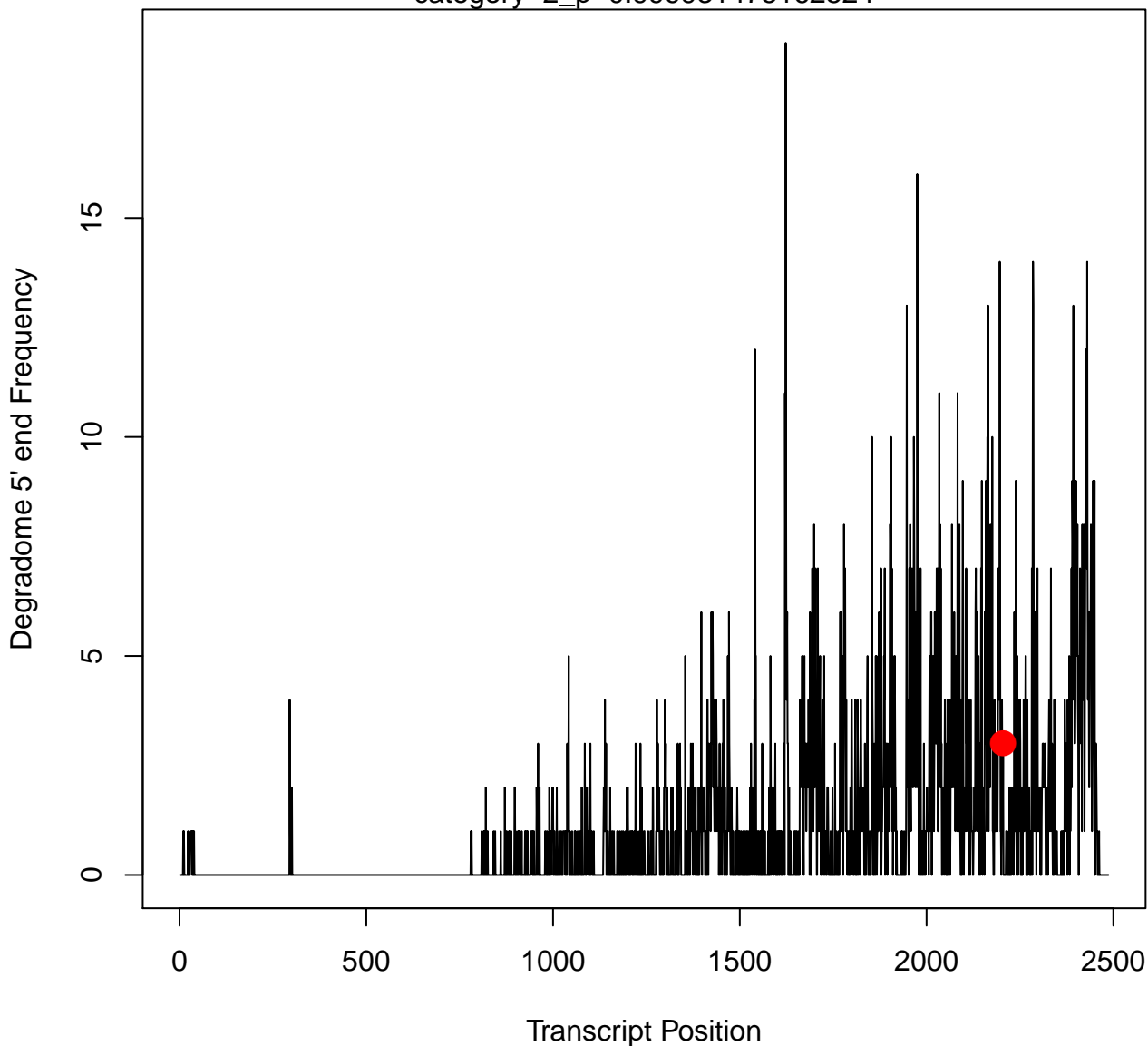

Supplement: Supplementary file 3 [file Data_Sheet_9.ZIP › GSM2230751.plot/Lsa-miR1446_Lsat_1_v5_gn_4_170480.1_2205_TPlot.pdf]

**T=Lsat\_1\_v5\_gn\_4\_76080.1\_Q=Lsa-miR1446\_S=648**

category=2\_p=0.841259007069944

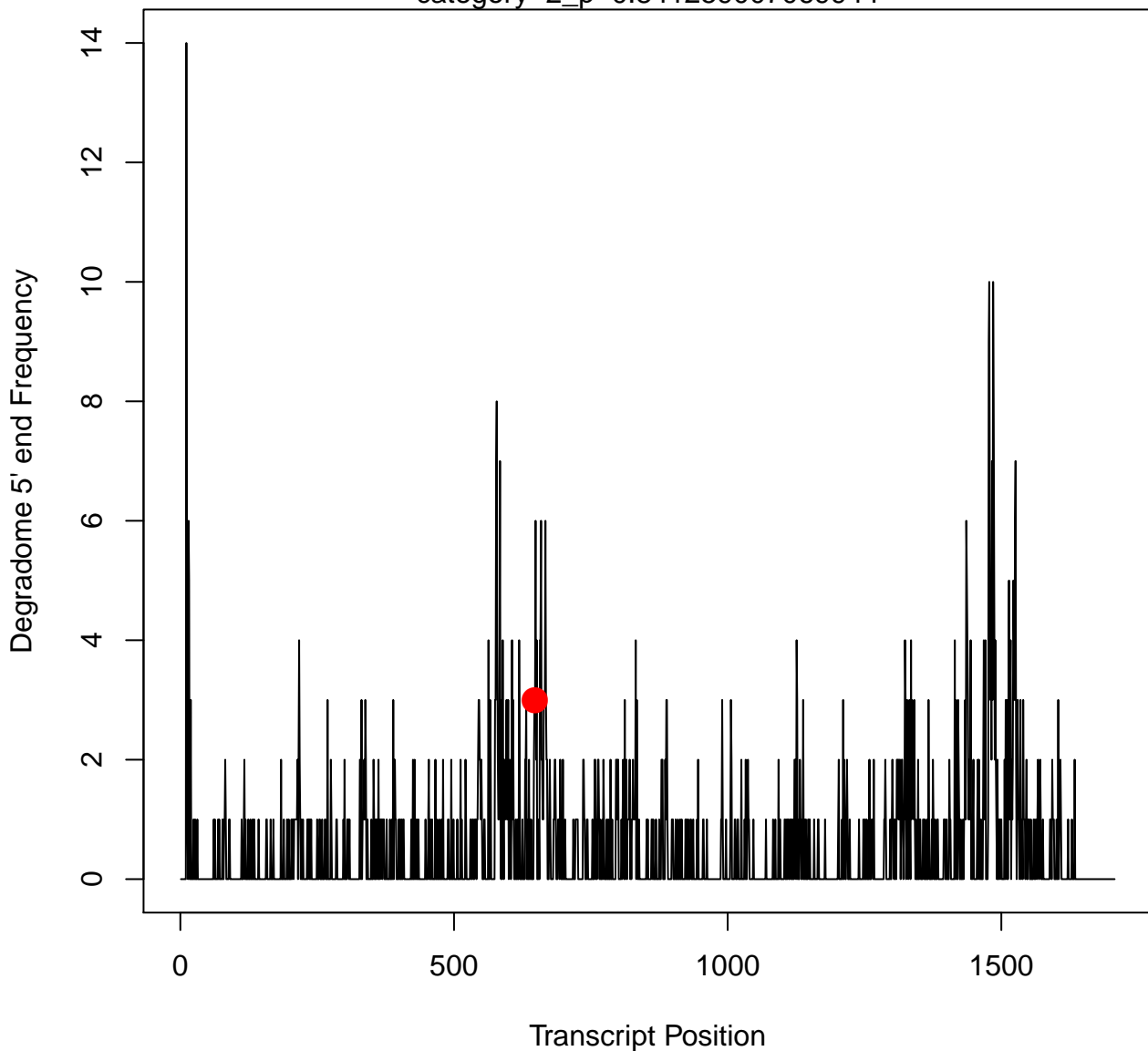

Supplement: Supplementary file 3 [file Data_Sheet_9.ZIP › GSM2230751.plot/Lsa-miR1446_Lsat_1_v5_gn_4_76080.1_648_TPlot.pdf]

**T=Lsat\_1\_v5\_gn\_5\_170600.1\_Q=Lsa-miR1446\_S=1122**

category=2\_p=0.999649317461376

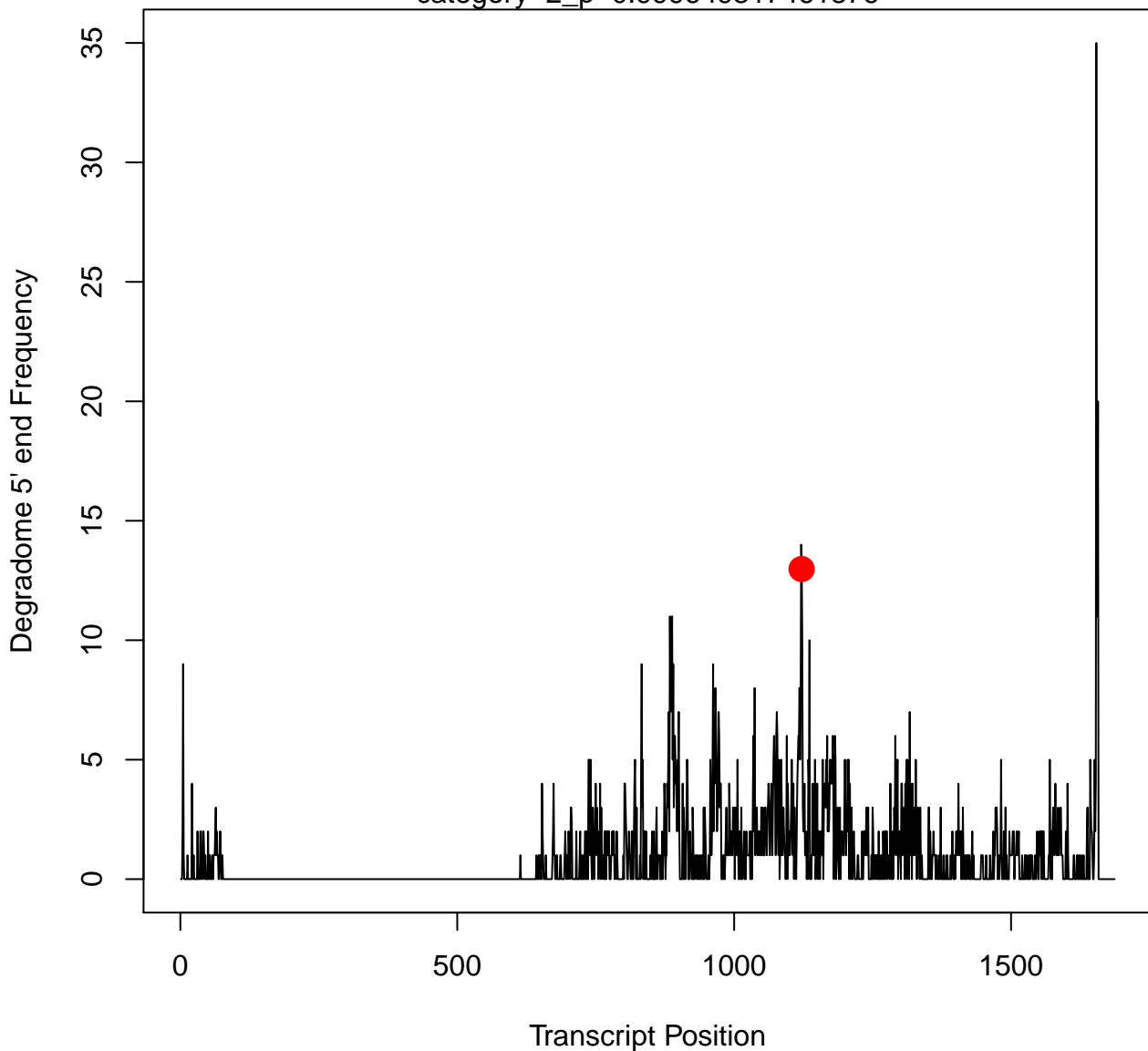

Supplement: Supplementary file 3 [file Data_Sheet_9.ZIP › GSM2230751.plot/Lsa-miR1446_Lsat_1_v5_gn_5_170600.1_1122_TPlot.pdf]

**T=Lsat\_1\_v5\_gn\_6\_110501.1\_Q=Lsa-miR1446\_S=404**

category=2\_p=0.943835073729059

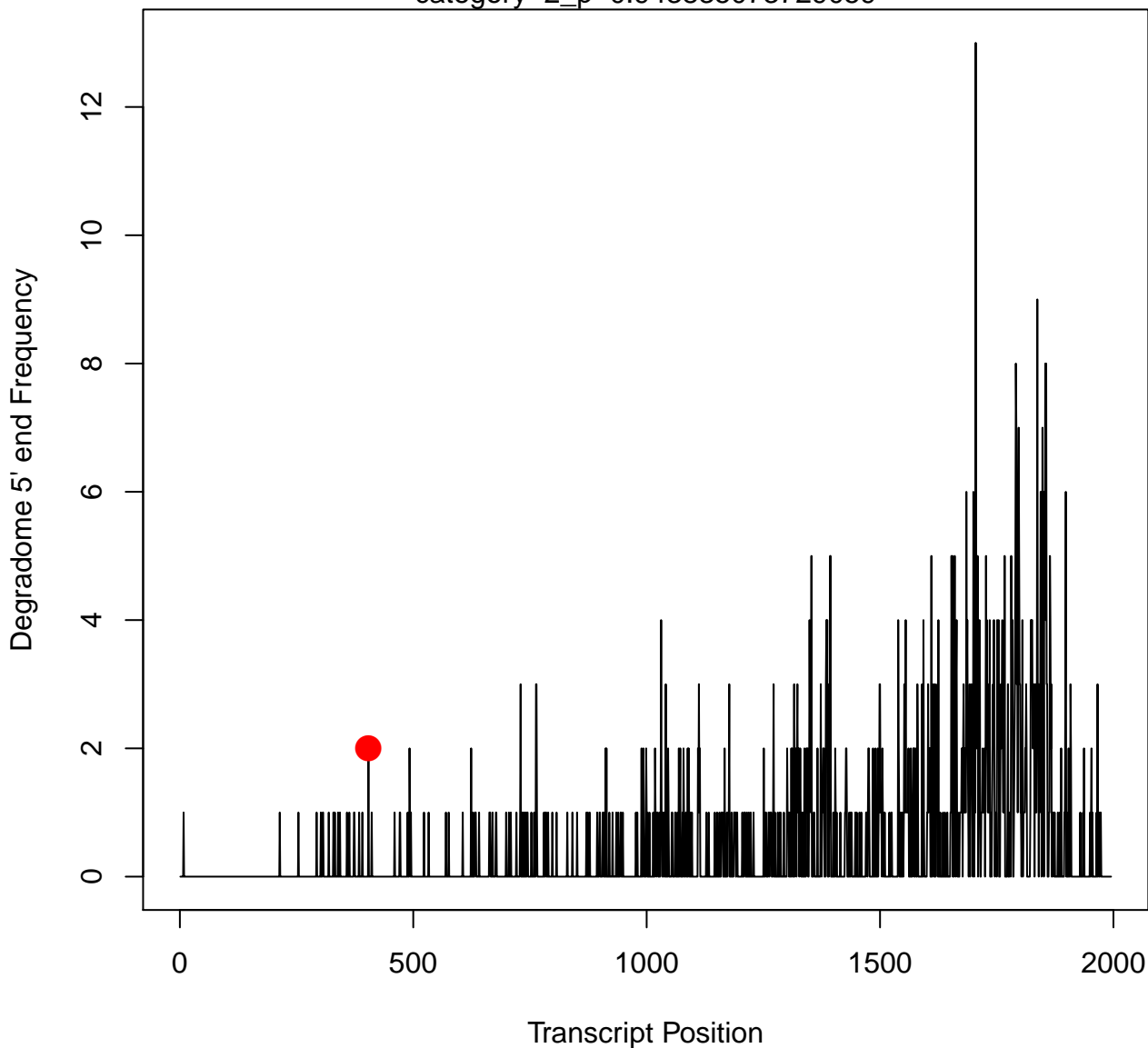

Supplement: Supplementary file 3 [file Data_Sheet_9.ZIP › GSM2230751.plot/Lsa-miR1446_Lsat_1_v5_gn_6_110501.1_404_TPlot.pdf]

**T=Lsat\_1\_v5\_gn\_6\_47421.1\_Q=Lsa-miR1446\_S=1114**

category=2\_p=0.299683009333602

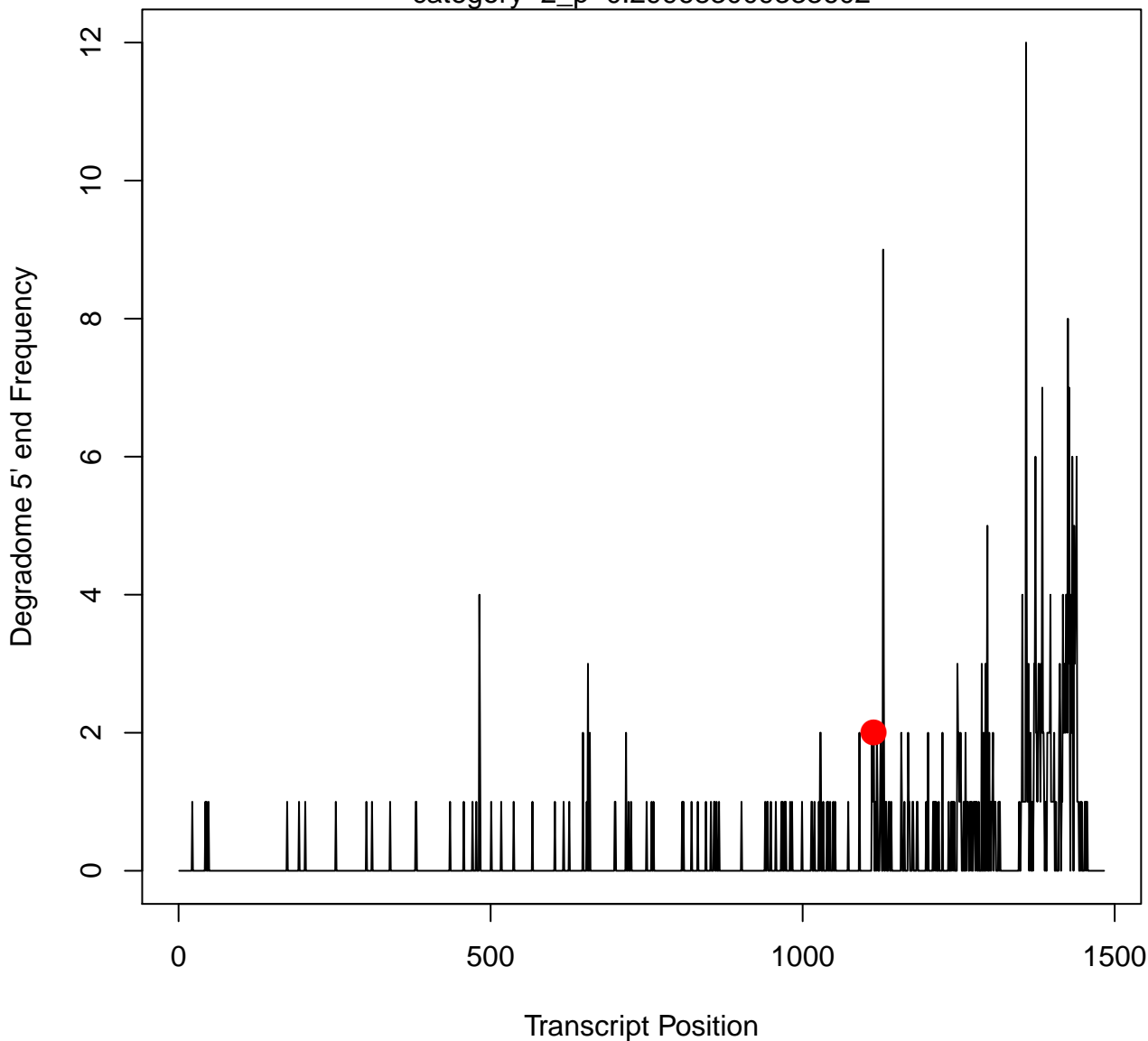

Supplement: Supplementary file 3 [file Data_Sheet_9.ZIP › GSM2230751.plot/Lsa-miR1446_Lsat_1_v5_gn_6_47421.1_1114_TPlot.pdf]

**T=Lsat\_1\_v5\_gn\_6\_8501.1\_Q=Lsa-miR1446\_S=2875**

category=2\_p=0.810311168621049

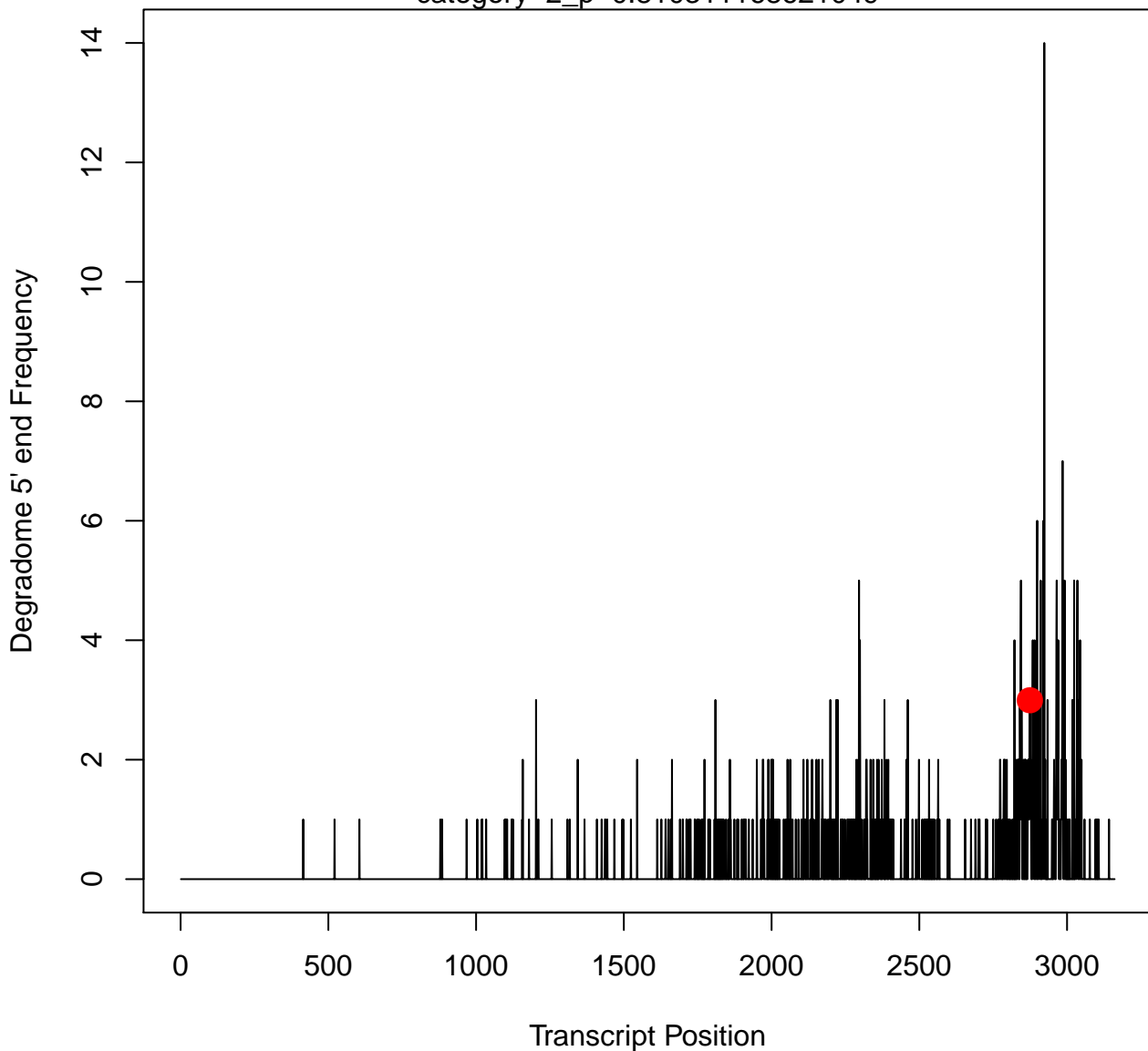

Supplement: Supplementary file 3 [file Data_Sheet_9.ZIP › GSM2230751.plot/Lsa-miR1446_Lsat_1_v5_gn_6_8501.1_2875_TPlot.pdf]

**T=Lsat\_1\_v5\_gn\_7\_29460.1\_Q=Lsa-miR1446\_S=1794**

category=2\_p=0.964018147250088

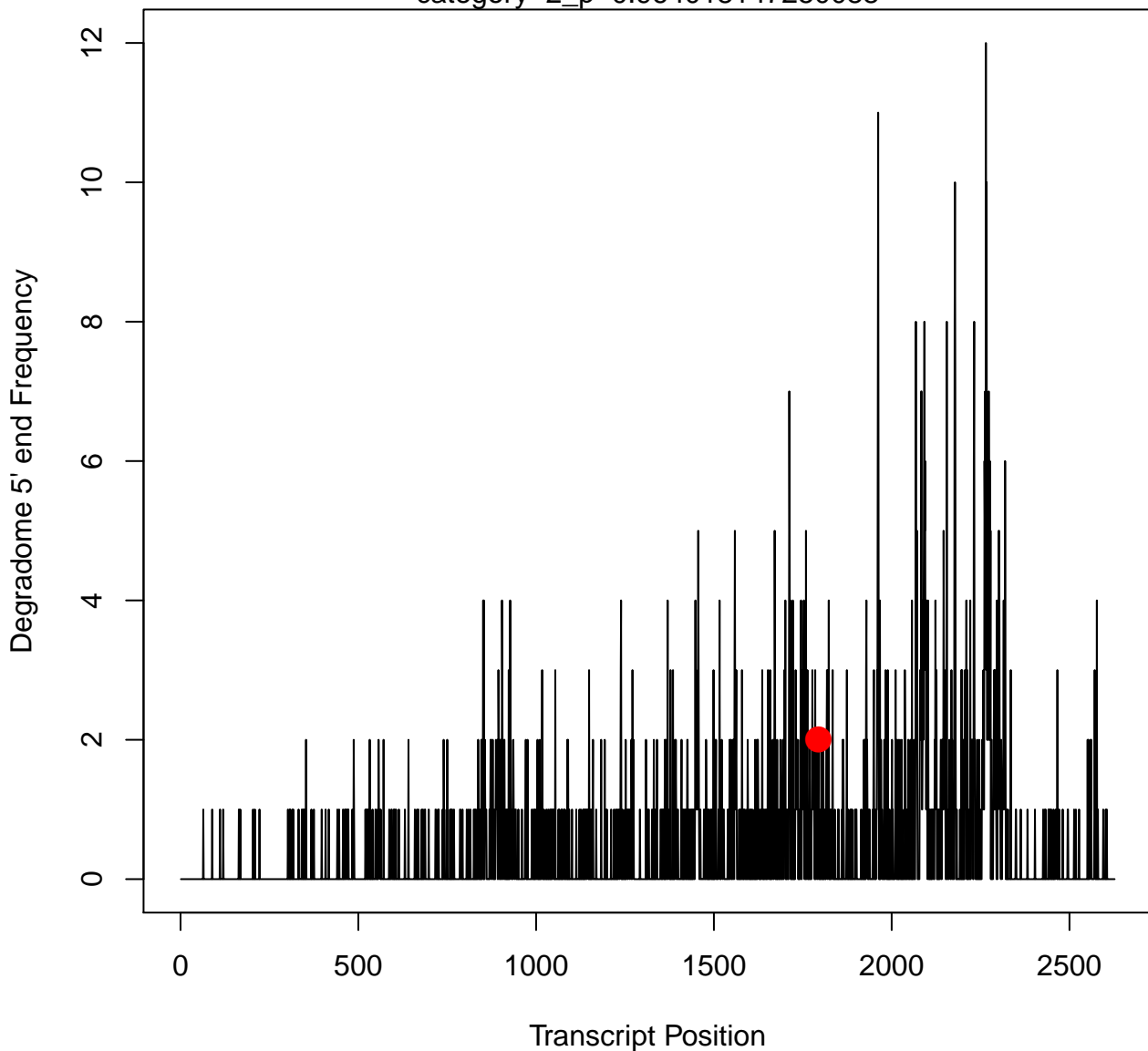

Supplement: Supplementary file 3 [file Data_Sheet_9.ZIP › GSM2230751.plot/Lsa-miR1446_Lsat_1_v5_gn_7_29460.1_1794_TPlot.pdf]

**T=Lsat\_1\_v5\_gn\_7\_71040.1\_Q=Lsa-miR1446\_S=1233**

category=2\_p=0.999991916191951

Degradome 5' end Frequency

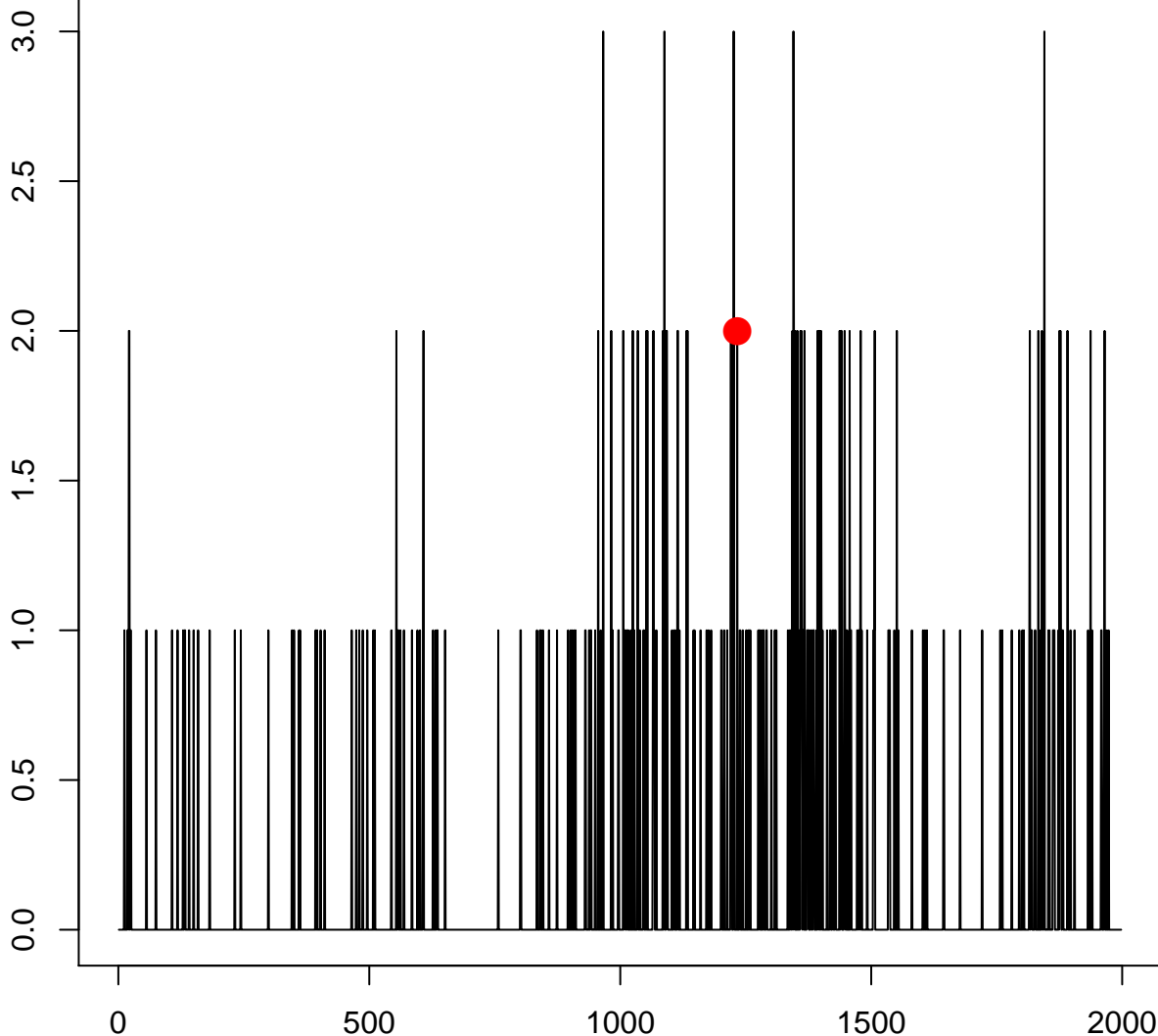

Transcript Position

Supplement: Supplementary file 3 [file Data_Sheet_9.ZIP › GSM2230751.plot/Lsa-miR1446_Lsat_1_v5_gn_7_71040.1_1233_TPlot.pdf]

**T=Lsat\_1\_v5\_gn\_7\_9300.1\_Q=Lsa-miR1446\_S=1495**

category=2\_p=0.999638751333022

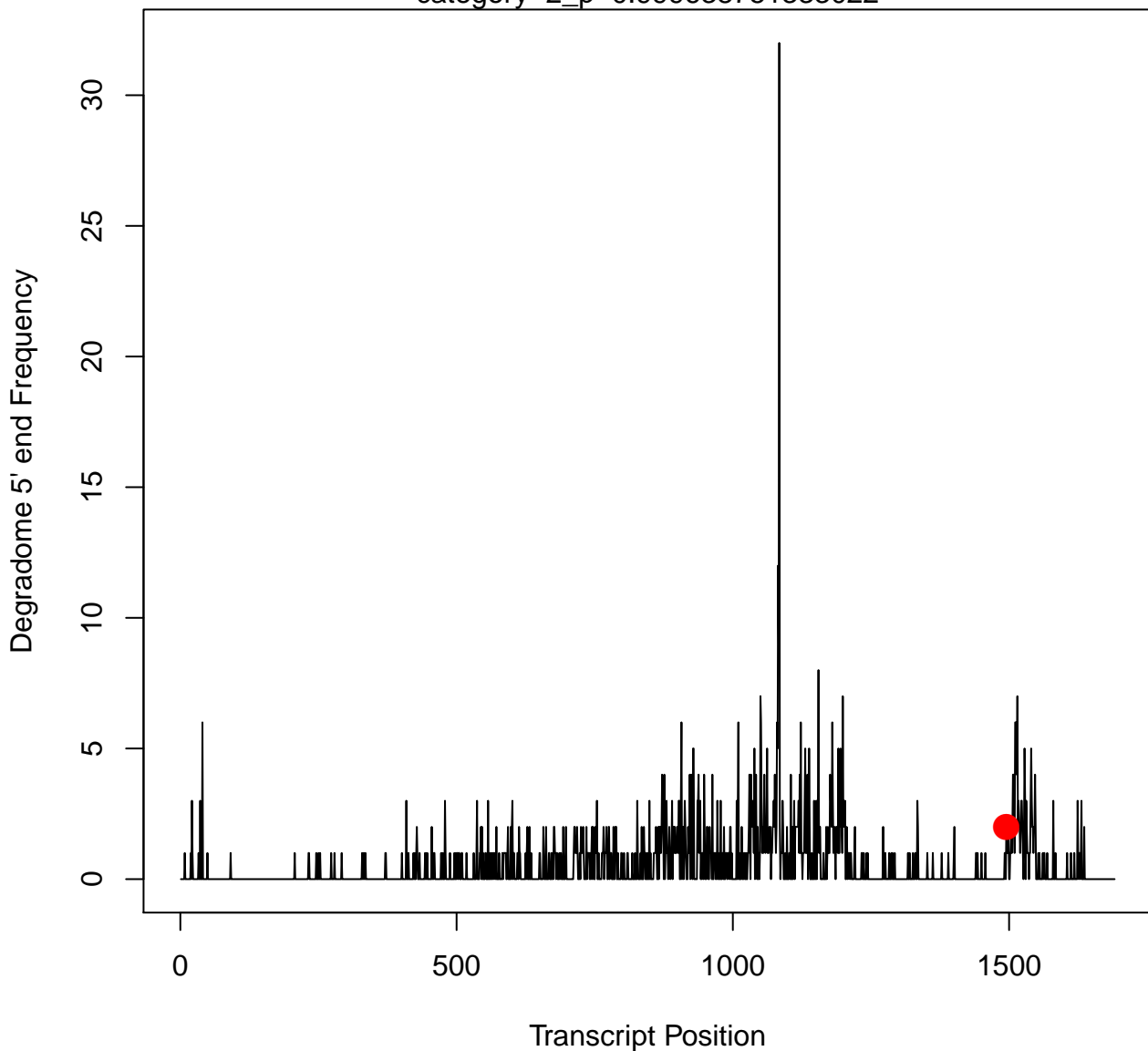

Supplement: Supplementary file 3 [file Data_Sheet_9.ZIP › GSM2230751.plot/Lsa-miR1446_Lsat_1_v5_gn_7_9300.1_1495_TPlot.pdf]

**T=Lsat\_1\_v5\_gn\_8\_167200.1\_Q=Lsa-miR1446\_S=999**

category=2\_p=0.479556547812474

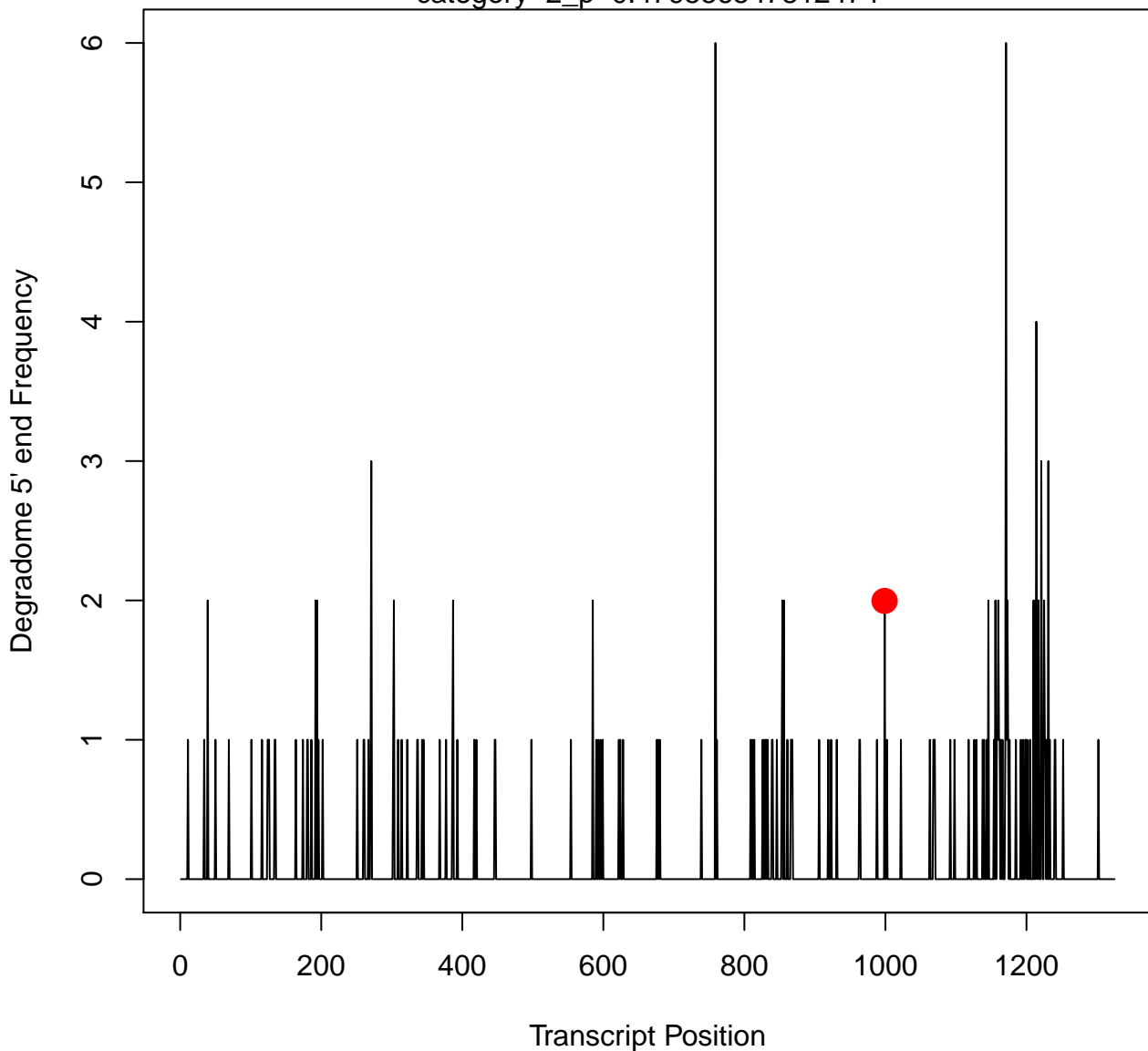

Supplement: Supplementary file 3 [file Data_Sheet_9.ZIP › GSM2230751.plot/Lsa-miR1446_Lsat_1_v5_gn_8_167200.1_999_TPlot.pdf]

**T=Lsat\_1\_v5\_gn\_8\_340.1\_Q=Lsa-miR1446\_S=2192**

category=2\_p=0.999775336525755

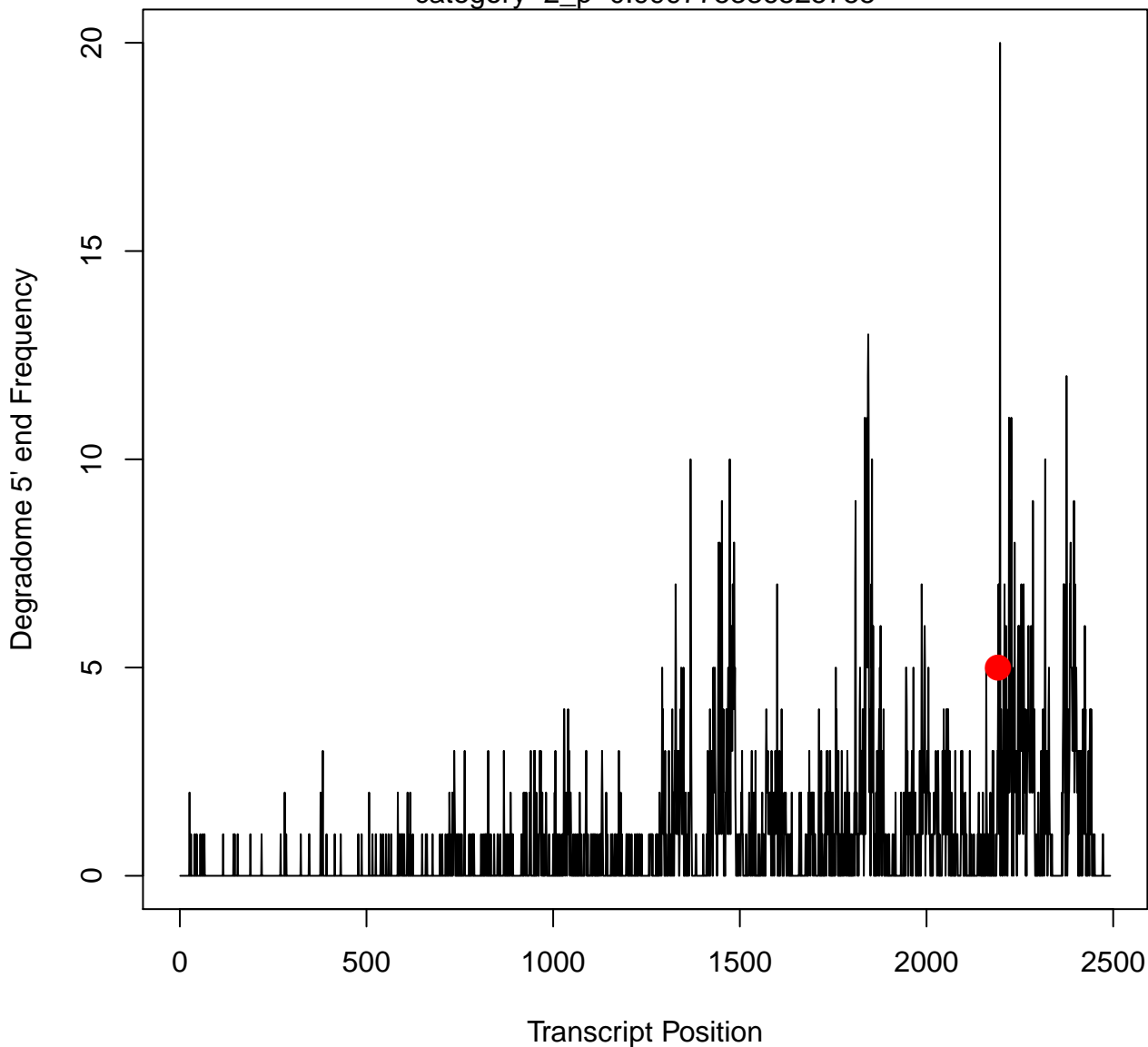

Supplement: Supplementary file 3 [file Data_Sheet_9.ZIP › GSM2230751.plot/Lsa-miR1446_Lsat_1_v5_gn_8_340.1_2192_TPlot.pdf]

**T=Lsat\_1\_v5\_gn\_8\_90101.1\_Q=Lsa-miR1446\_S=650**

category=2\_p=0.99996326215525

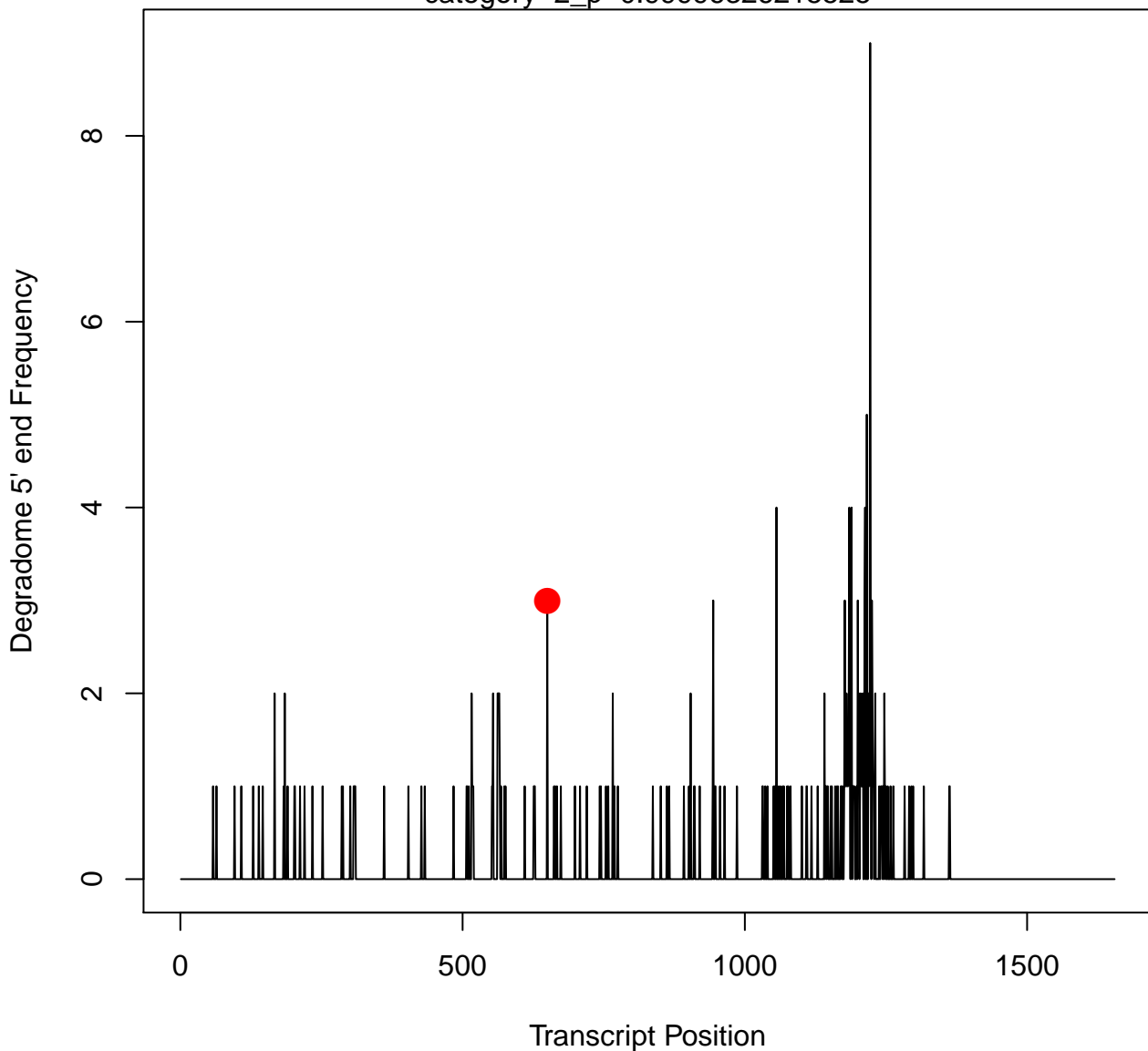

Supplement: Supplementary file 3 [file Data_Sheet_9.ZIP › GSM2230751.plot/Lsa-miR1446_Lsat_1_v5_gn_8_90101.1_650_TPlot.pdf]

**T=Lsat\_1\_v5\_gn\_9\_121240.1\_Q=Lsa-miR1446\_S=2400**

category=2\_p=0.995495612722435

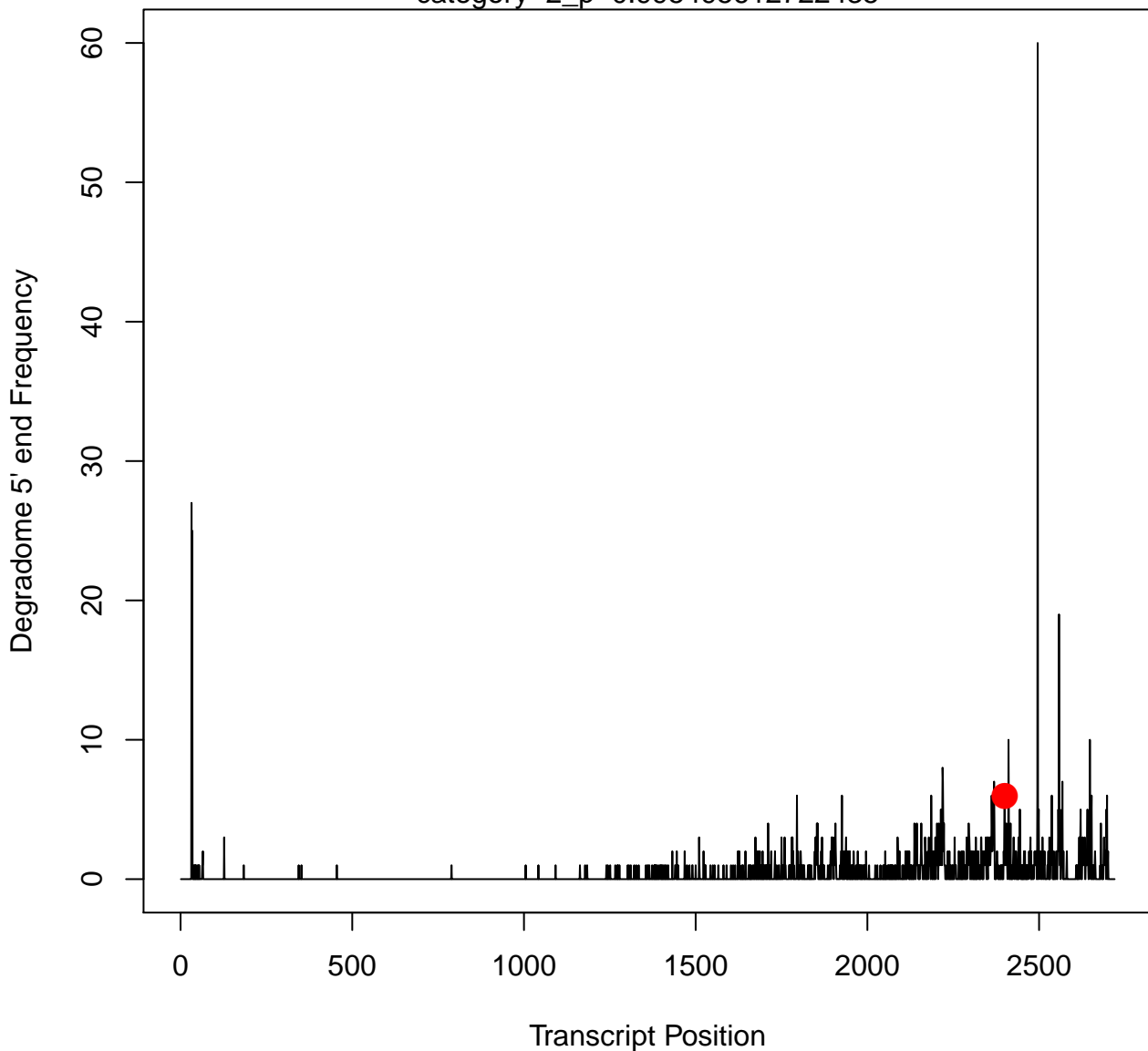

Supplement: Supplementary file 3 [file Data_Sheet_9.ZIP › GSM2230751.plot/Lsa-miR1446_Lsat_1_v5_gn_9_121240.1_2400_TPlot.pdf]

**T=Lsat\_1\_v5\_gn\_7\_11040.1\_Q=Lsa-miR156d\_S=1087**

category=0\_p=0.00184635508168884

Degradome 5' end Frequency

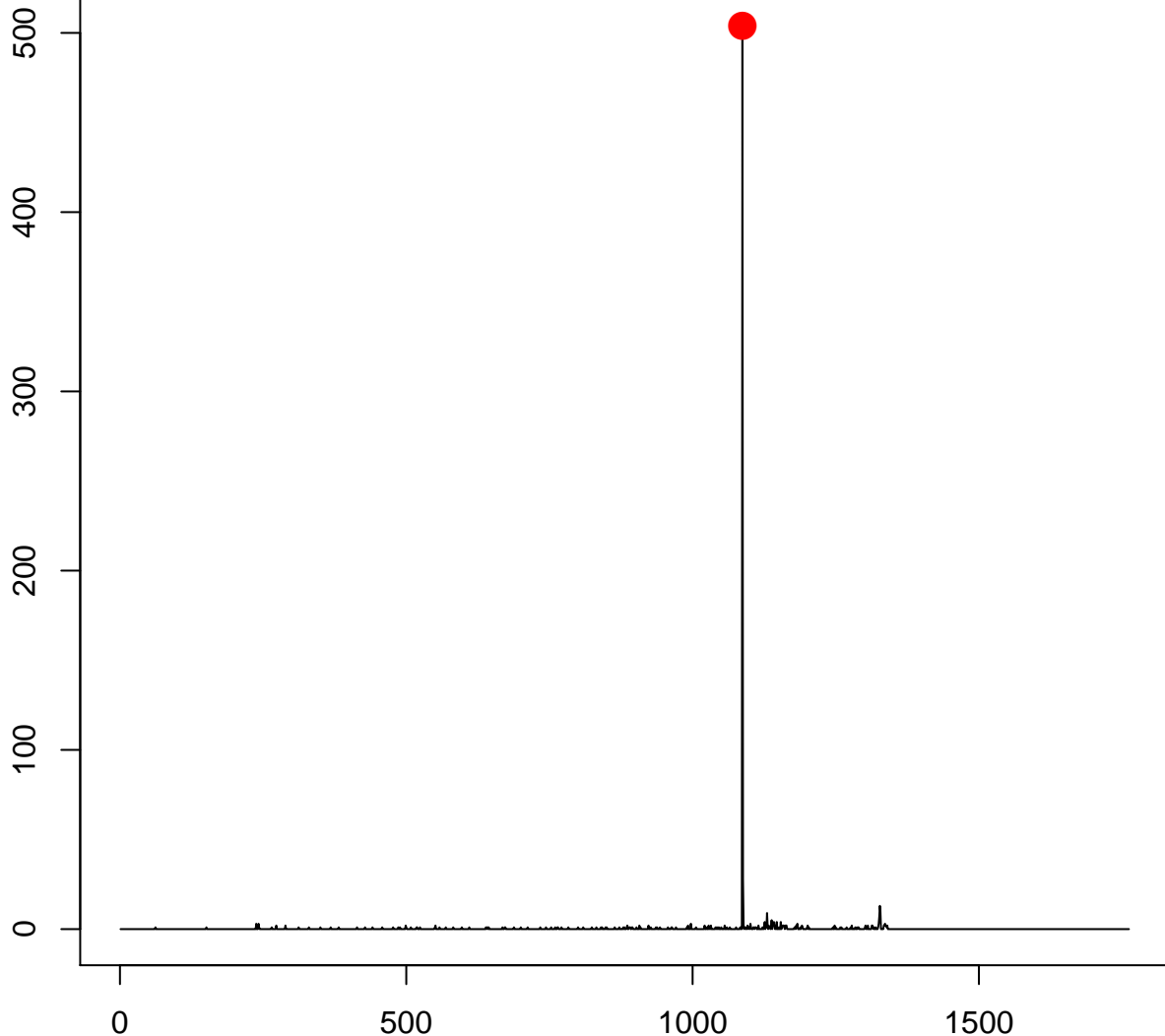

Transcript Position

Supplement: Supplementary file 3 [file Data_Sheet_9.ZIP › GSM2230751.plot/Lsa-miR156d_Lsat_1_v5_gn_7_11040.1_1087_TPlot.pdf]

**T=Lsat\_1\_v5\_gn\_5\_173741.1\_Q=Lsa-miR156f\_S=348**

category=2\_p=0.904165209342093

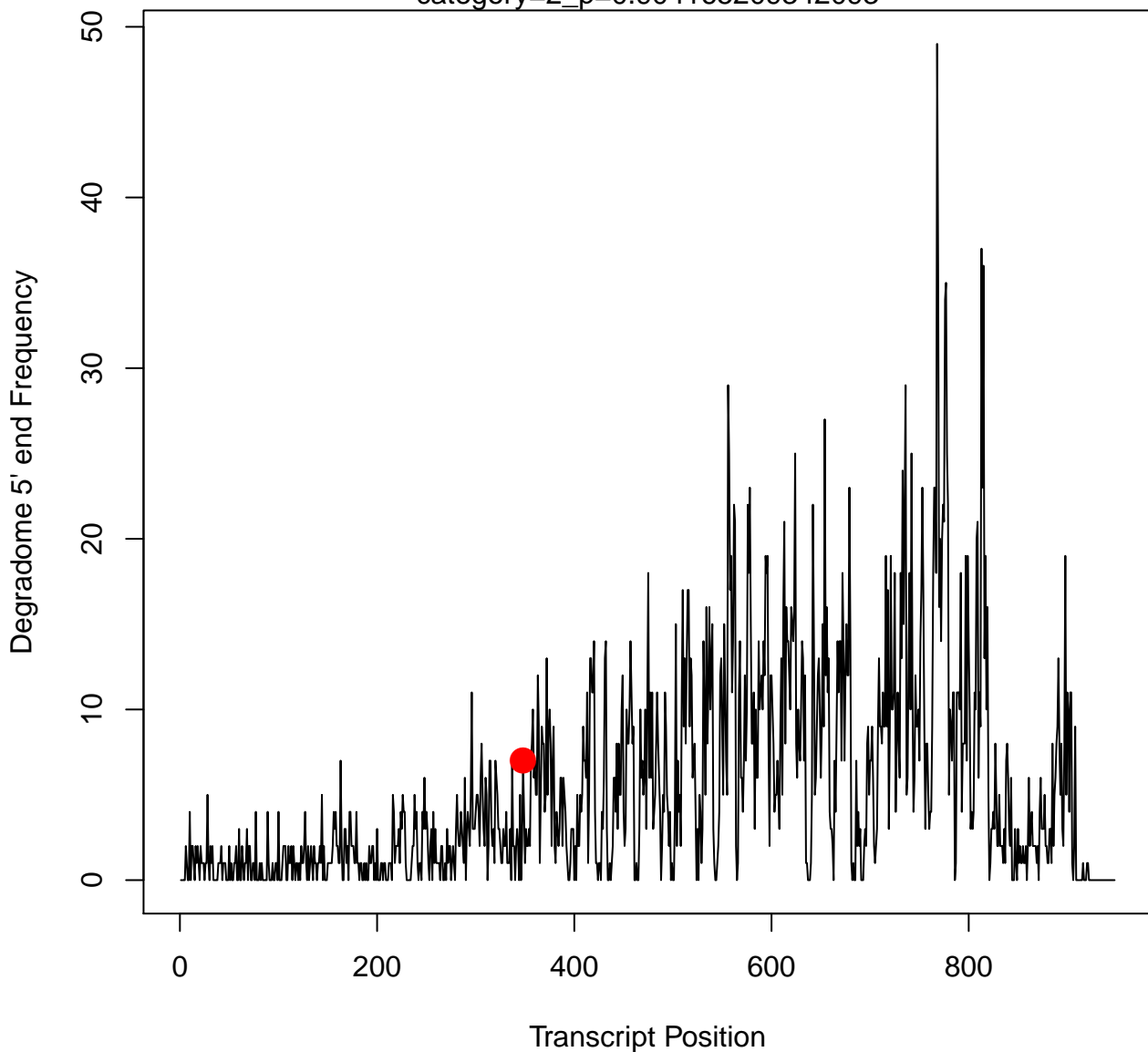

Supplement: Supplementary file 3 [file Data_Sheet_9.ZIP › GSM2230751.plot/Lsa-miR156f_Lsat_1_v5_gn_5_173741.1_348_TPlot.pdf]

**T=Lsat\_1\_v5\_gn\_1\_6040.1\_Q=Lsa-miR156h\_S=188**

category=2\_p=0.32016651526265

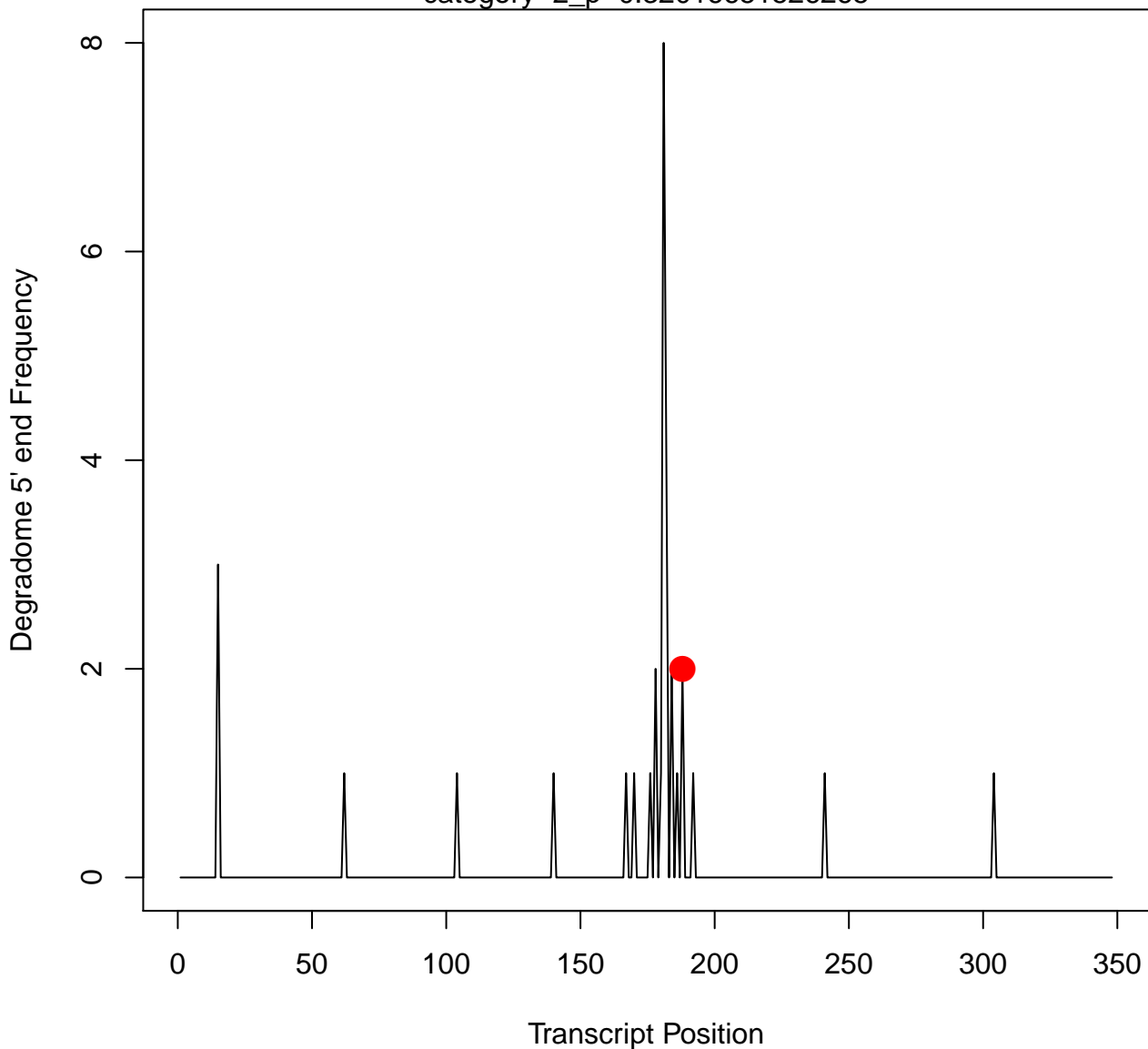

Supplement: Supplementary file 3 [file Data_Sheet_9.ZIP › GSM2230751.plot/Lsa-miR156h_Lsat_1_v5_gn_1_6040.1_188_TPlot.pdf]

**T=Lsat\_1\_v5\_gn\_2\_88681.1\_Q=Lsa-miR156i\_S=2002**

category=2\_p=0.924423581502005

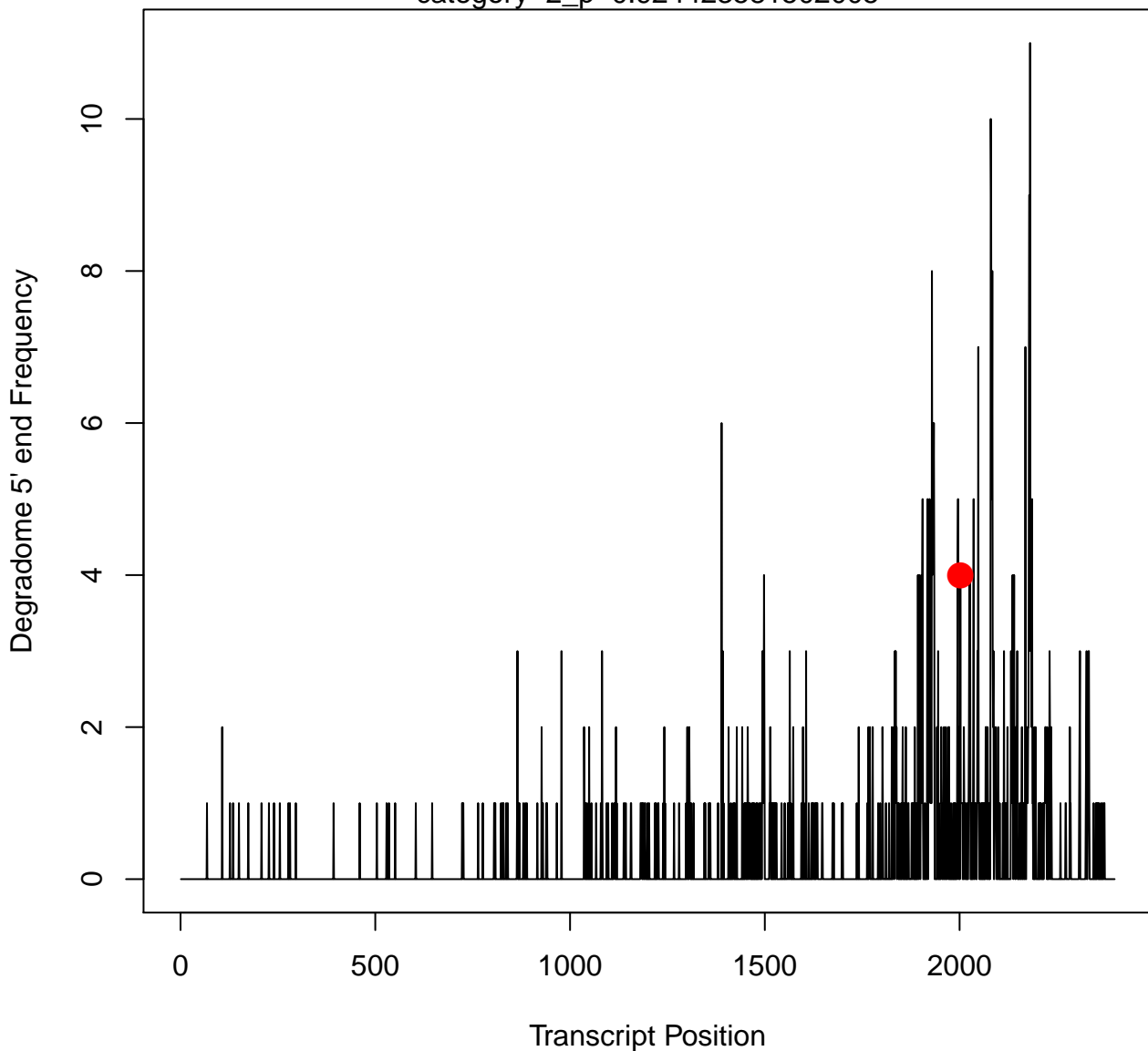

Supplement: Supplementary file 3 [file Data_Sheet_9.ZIP › GSM2230751.plot/Lsa-miR156i_Lsat_1_v5_gn_2_88681.1_2002_TPlot.pdf]

**T=Lsat\_1\_v5\_gn\_5\_12360.1\_Q=Lsa-miR156i\_S=1859**

category=0\_p=0.0011082224851342

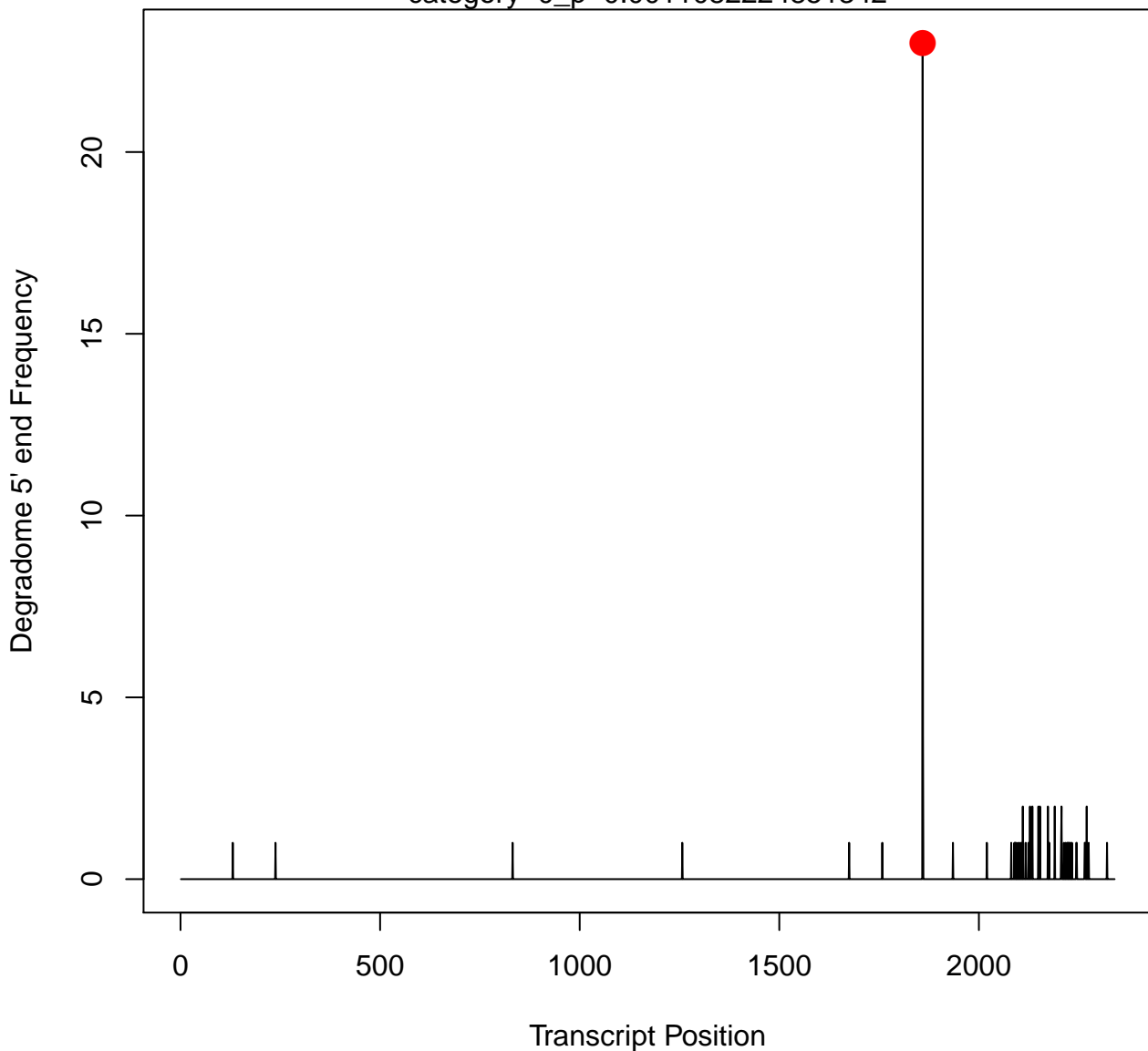

Supplement: Supplementary file 3 [file Data_Sheet_9.ZIP › GSM2230751.plot/Lsa-miR156i_Lsat_1_v5_gn_5_12360.1_1859_TPlot.pdf]

**T=Lsat\_1\_v5\_gn\_8\_161201.1\_Q=Lsa-miR156i\_S=1291**

category=2\_p=0.93484830030796

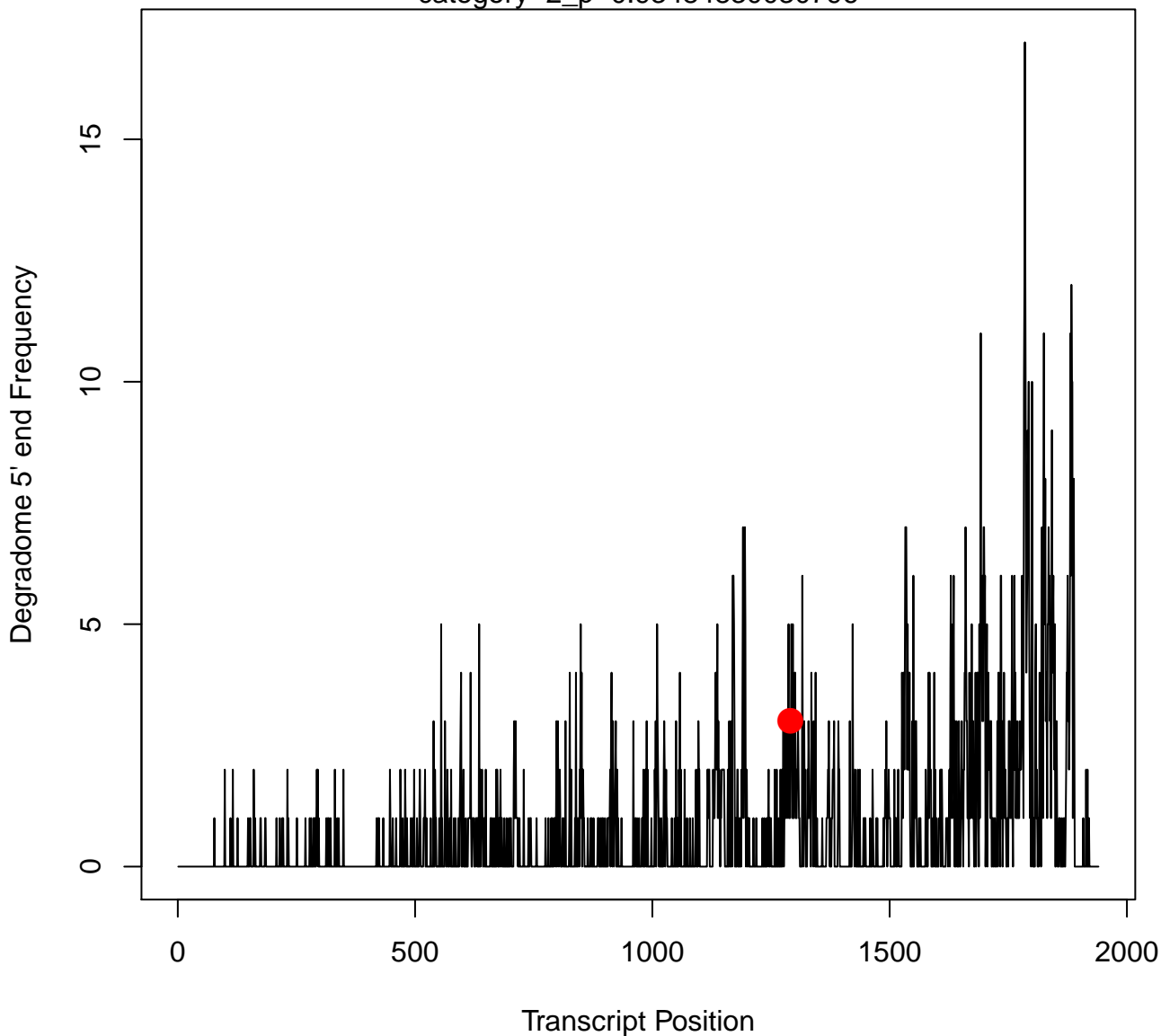

Supplement: Supplementary file 3 [file Data_Sheet_9.ZIP › GSM2230751.plot/Lsa-miR156i_Lsat_1_v5_gn_8_161201.1_1291_TPlot.pdf]

**T=Lsat\_1\_v5\_gn\_9\_28021.1\_Q=Lsa-miR156i\_S=1267**

category=0\_p=0.000738951519243747

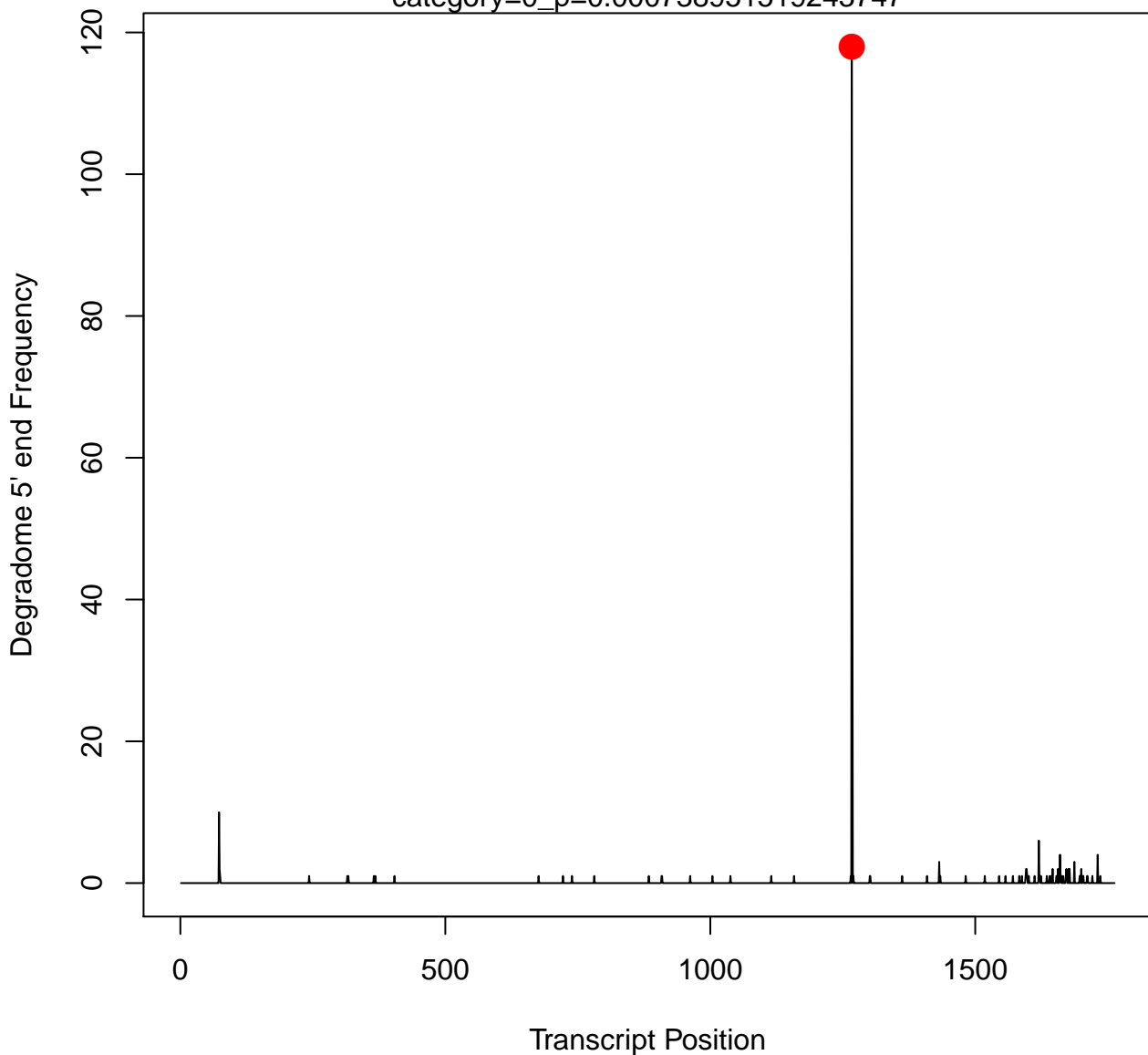

Supplement: Supplementary file 3 [file Data_Sheet_9.ZIP › GSM2230751.plot/Lsa-miR156i_Lsat_1_v5_gn_9_28021.1_1267_TPlot.pdf]

T=Lsat\_1\_v5\_gn\_0\_3541.1\_Q=Lsa-miR156j\_S=2134

category=2\_p=0.551344504311096

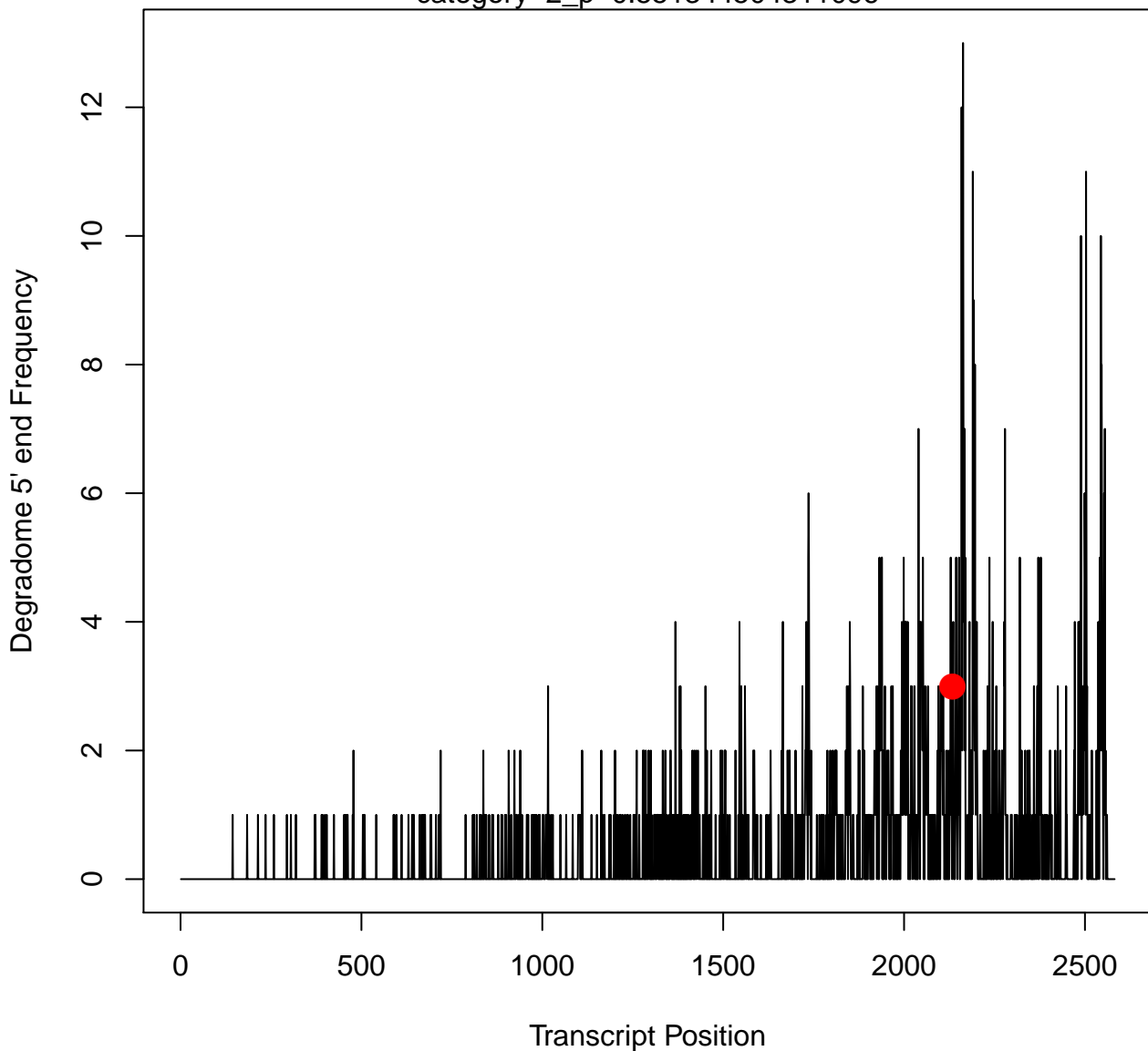

Supplement: Supplementary file 3 [file Data_Sheet_9.ZIP › GSM2230751.plot/Lsa-miR156j_Lsat_1_v5_gn_0_3541.1_2134_TPlot.pdf]

**T=Lsat\_1\_v5\_gn\_1\_126340.1\_Q=Lsa-miR156j\_S=1034**

category=2\_p=0.924423581502005

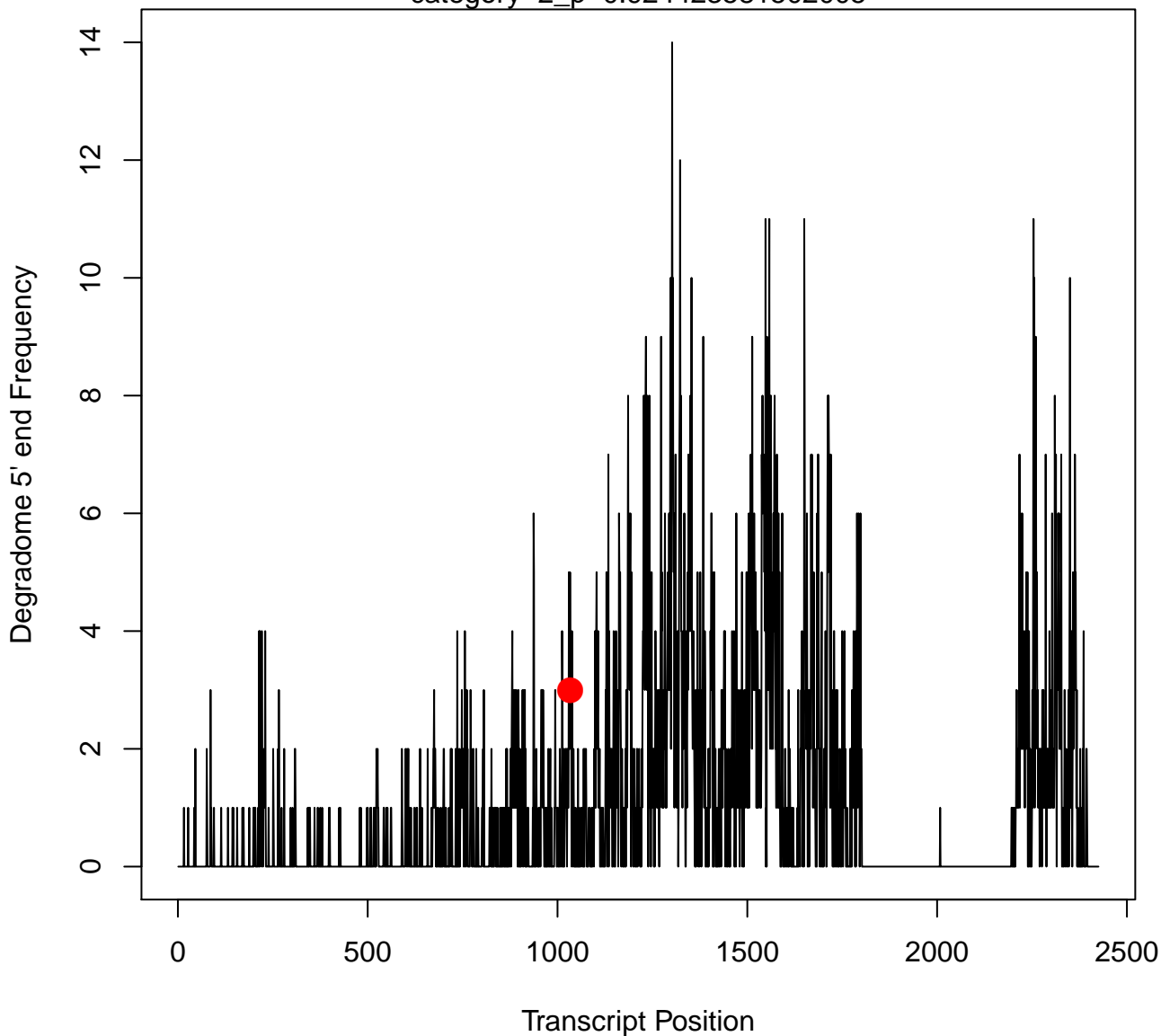

Supplement: Supplementary file 3 [file Data_Sheet_9.ZIP › GSM2230751.plot/Lsa-miR156j_Lsat_1_v5_gn_1_126340.1_1034_TPlot.pdf]

T=Lsat\_1\_v5\_gn\_1\_14560.1\_Q=Lsa-miR156j\_S=192

category=0\_p=0.000369544041020964

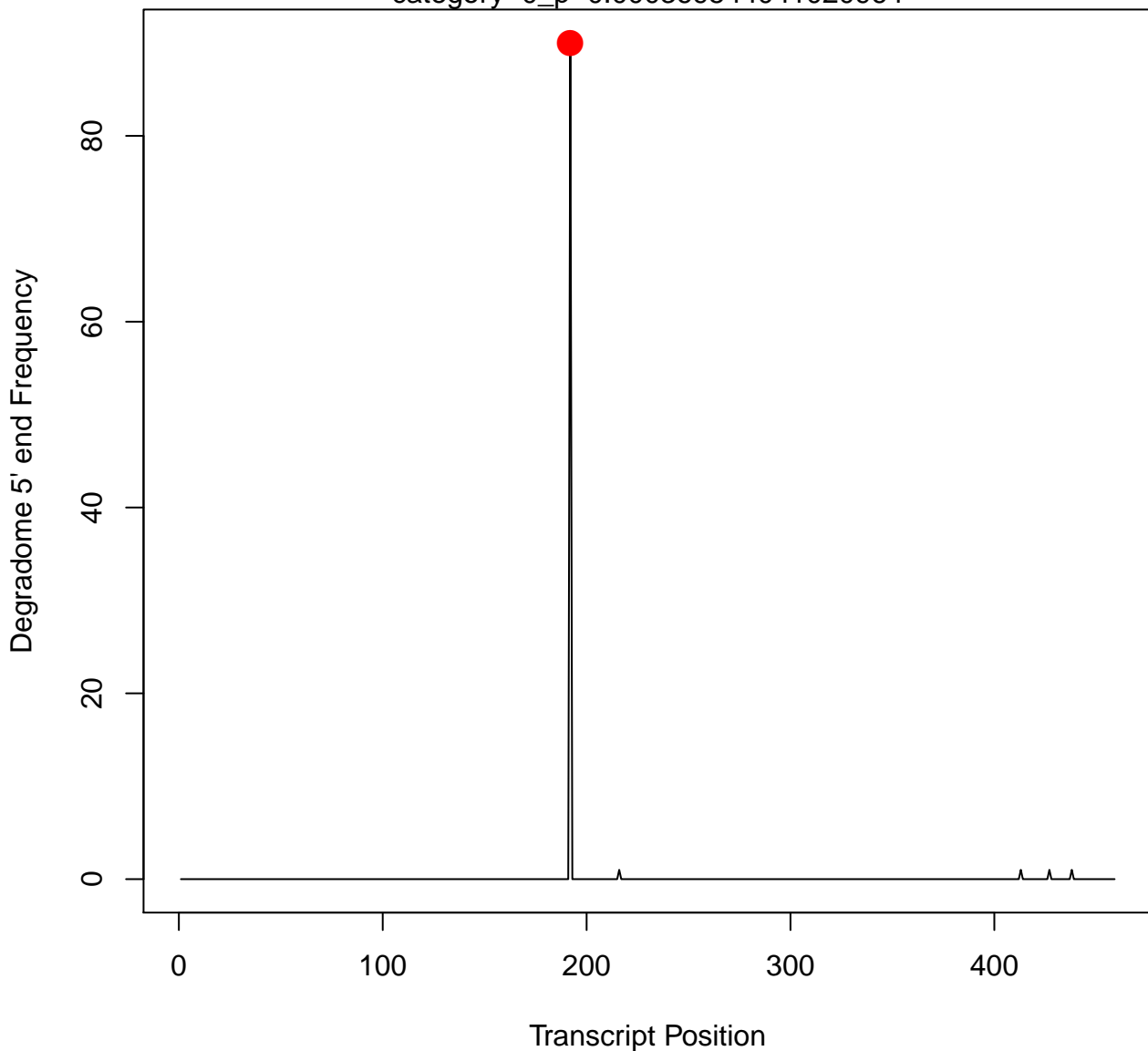

Supplement: Supplementary file 3 [file Data_Sheet_9.ZIP › GSM2230751.plot/Lsa-miR156j_Lsat_1_v5_gn_1_14560.1_192_TPlot.pdf]

T=Lsat\_1\_v5\_gn\_2\_75101.1\_Q=Lsa-miR156j\_S=699

category=2\_p=0.936753916257189

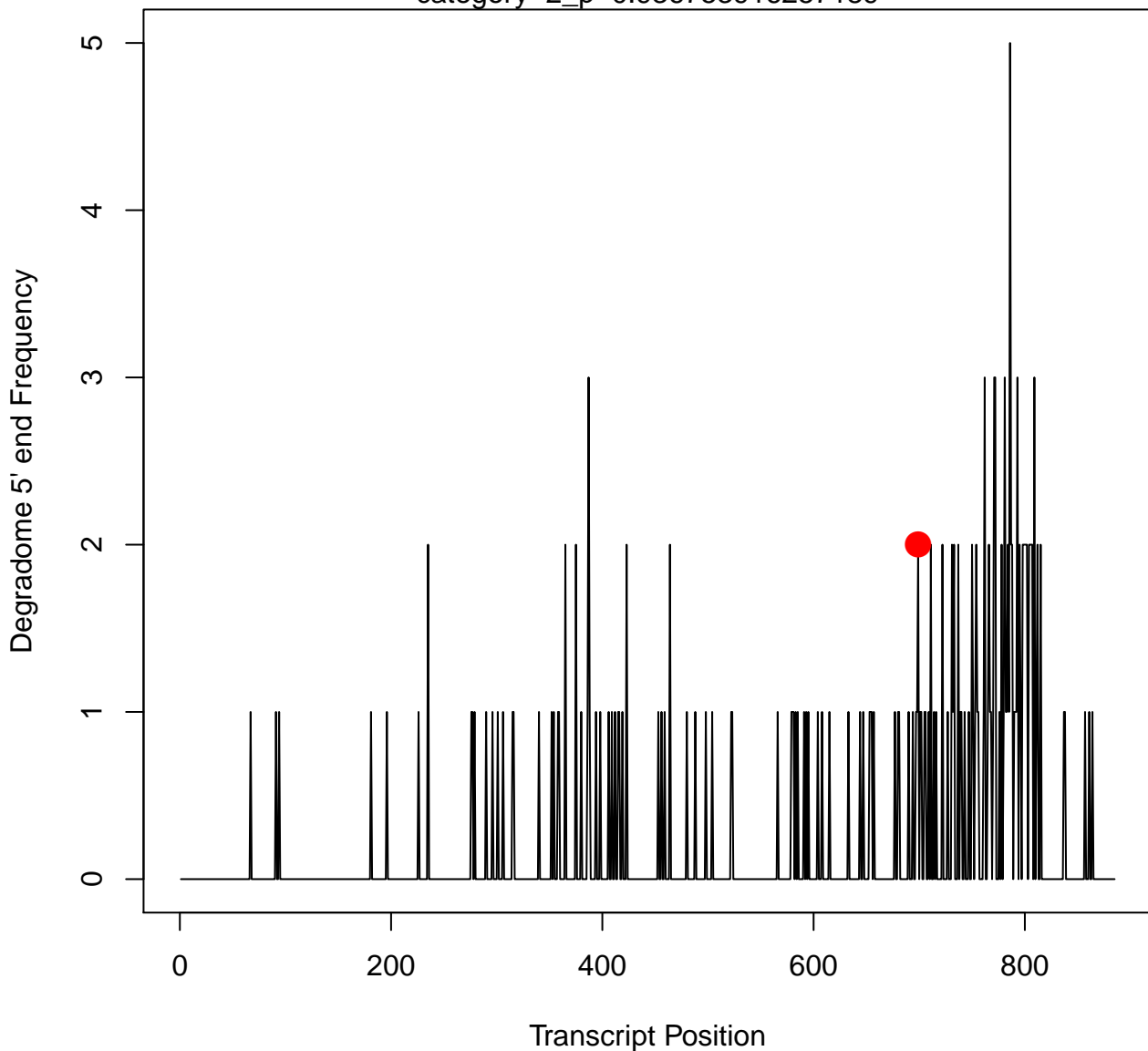

Supplement: Supplementary file 3 [file Data_Sheet_9.ZIP › GSM2230751.plot/Lsa-miR156j_Lsat_1_v5_gn_2_75101.1_699_TPlot.pdf]

**T=Lsat\_1\_v5\_gn\_4\_139200.1\_Q=Lsa-miR156j\_S=170**

category=0\_p=0.00846505176584544

Degradsome 5' end Frequency

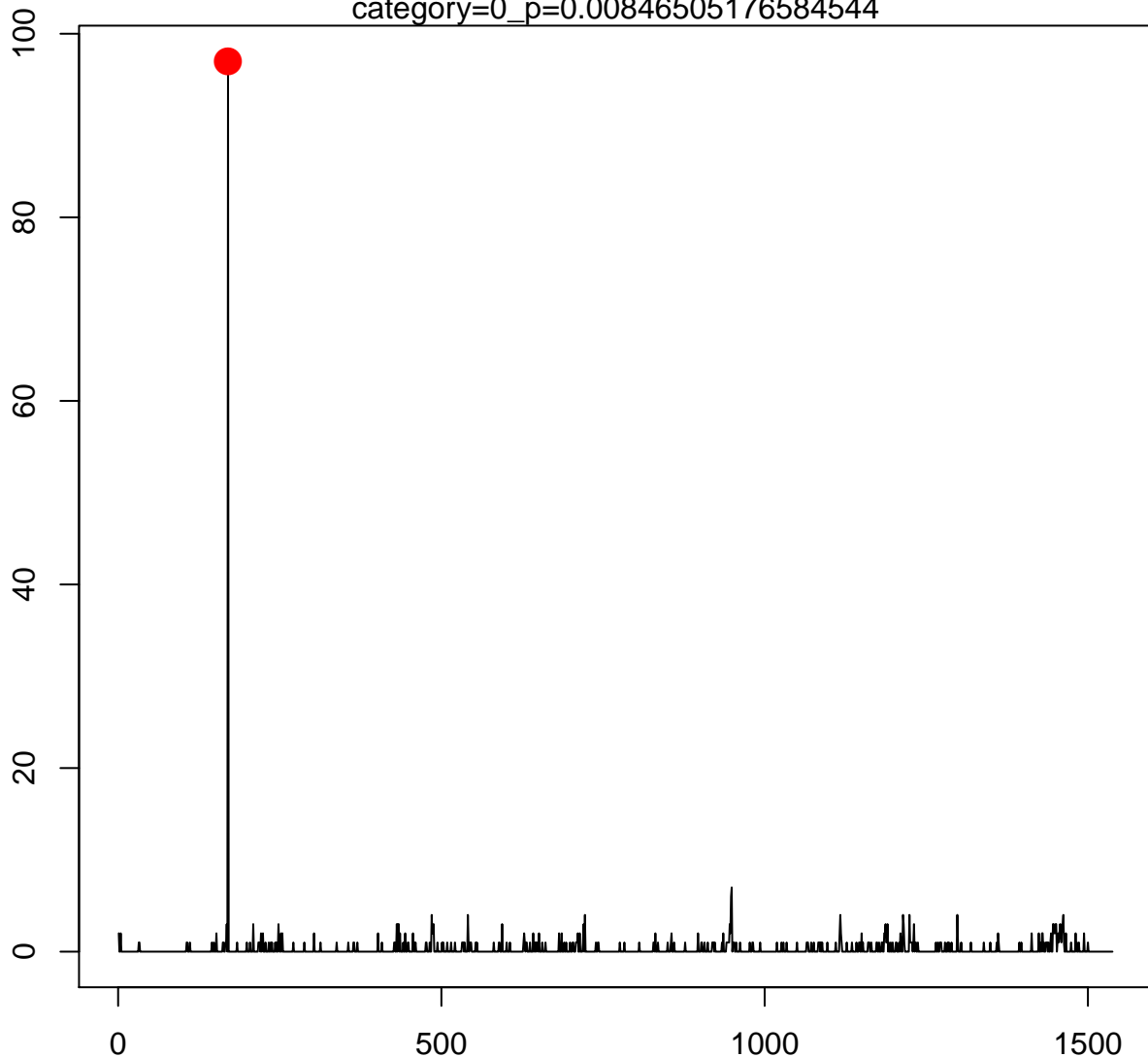

Transcript Position

Supplement: Supplementary file 3 [file Data_Sheet_9.ZIP › GSM2230751.plot/Lsa-miR156j_Lsat_1_v5_gn_4_139200.1_170_TPlot.pdf]

**T=Lsat\_1\_v5\_gn\_4\_64540.1\_Q=Lsa-miR156j\_S=748**

category=2\_p=0.906968272139102

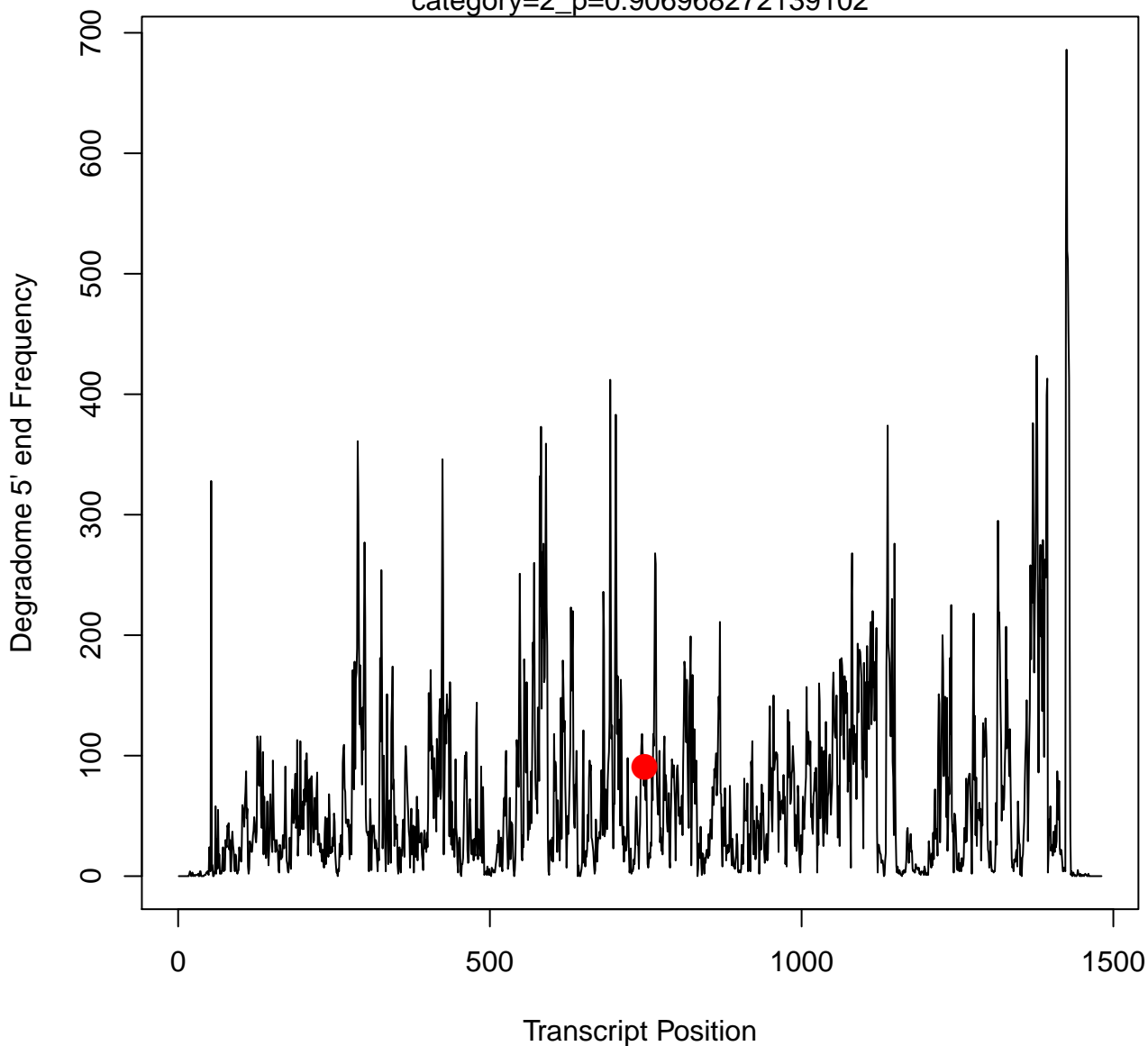

Supplement: Supplementary file 3 [file Data_Sheet_9.ZIP › GSM2230751.plot/Lsa-miR156j_Lsat_1_v5_gn_4_64540.1_748_TPlot.pdf]

**T=Lsat\_1\_v5\_gn\_4\_7021.1\_Q=Lsa-miR156j\_S=627**

category=2\_p=0.810311168621049

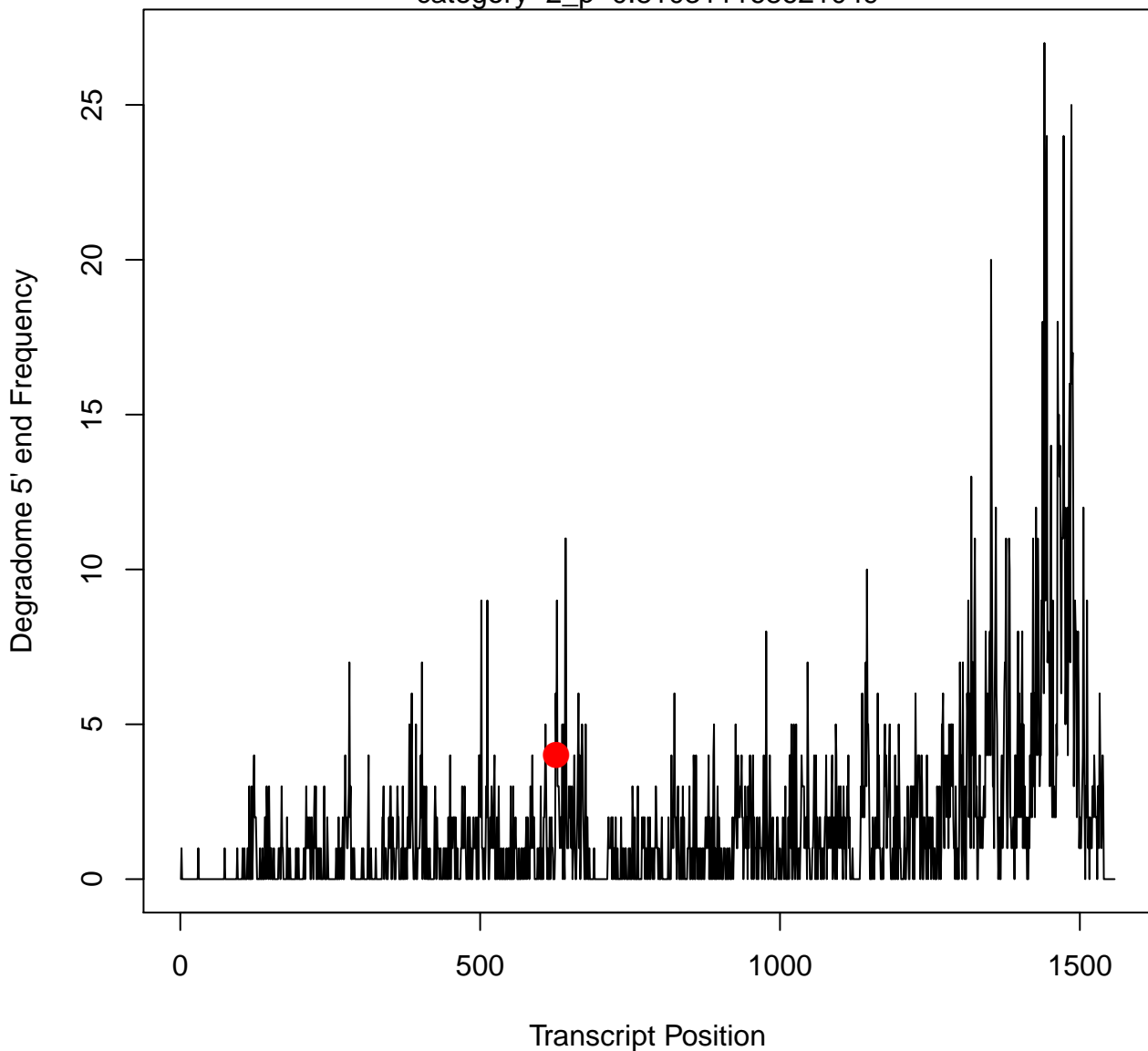

Supplement: Supplementary file 3 [file Data_Sheet_9.ZIP › GSM2230751.plot/Lsa-miR156j_Lsat_1_v5_gn_4_7021.1_627_TPlot.pdf]

**T=Lsat\_1\_v5\_gn\_4\_81541.1\_Q=Lsa-miR156j\_S=850**

category=0\_p=0.00368930113628996

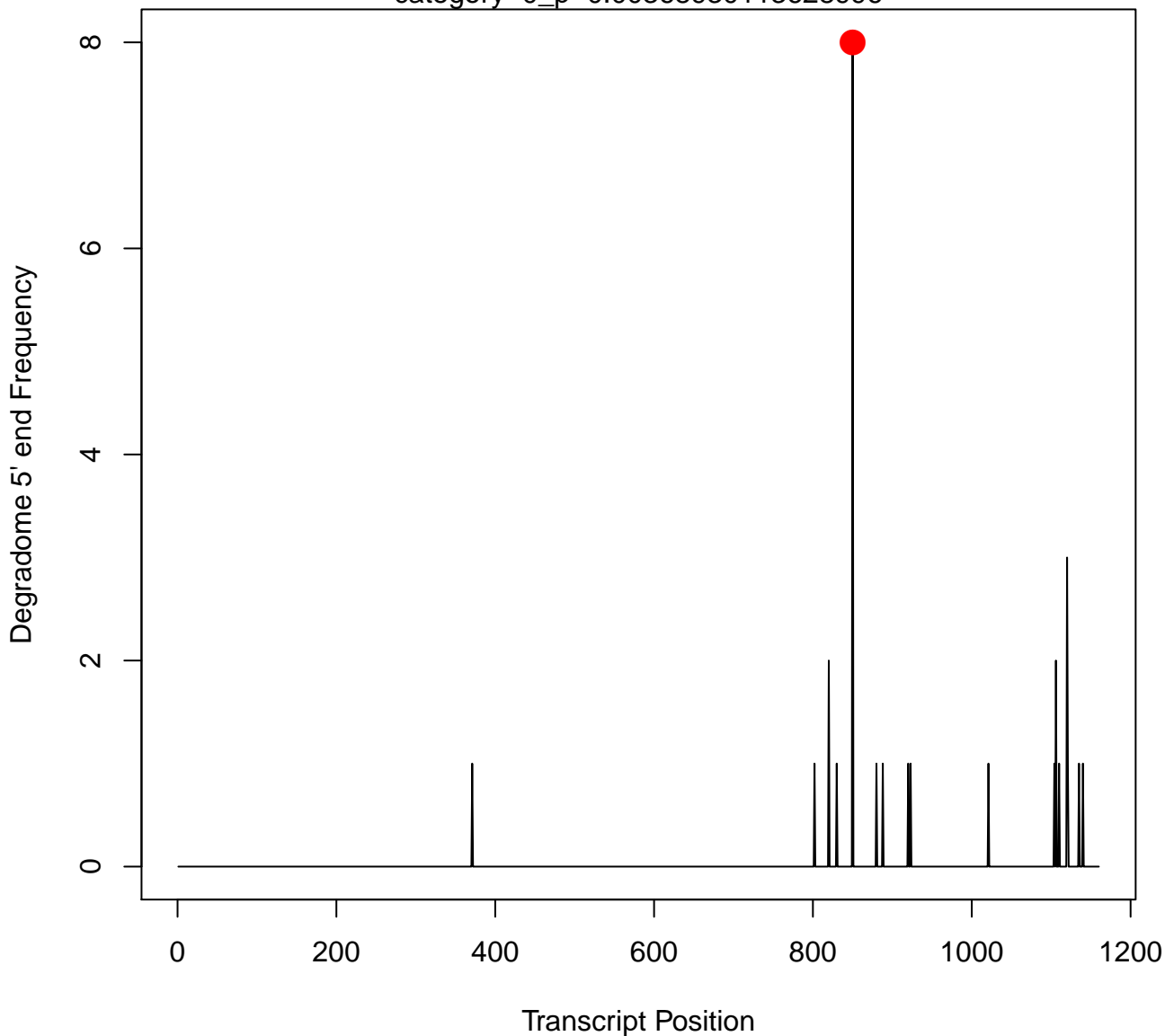

Supplement: Supplementary file 3 [file Data_Sheet_9.ZIP › GSM2230751.plot/Lsa-miR156j_Lsat_1_v5_gn_4_81541.1_850_TPlot.pdf]

**T=Lsat\_1\_v5\_gn\_5\_32220.1\_Q=Lsa-miR156j\_S=1992**

category=2\_p=0.93288526780531

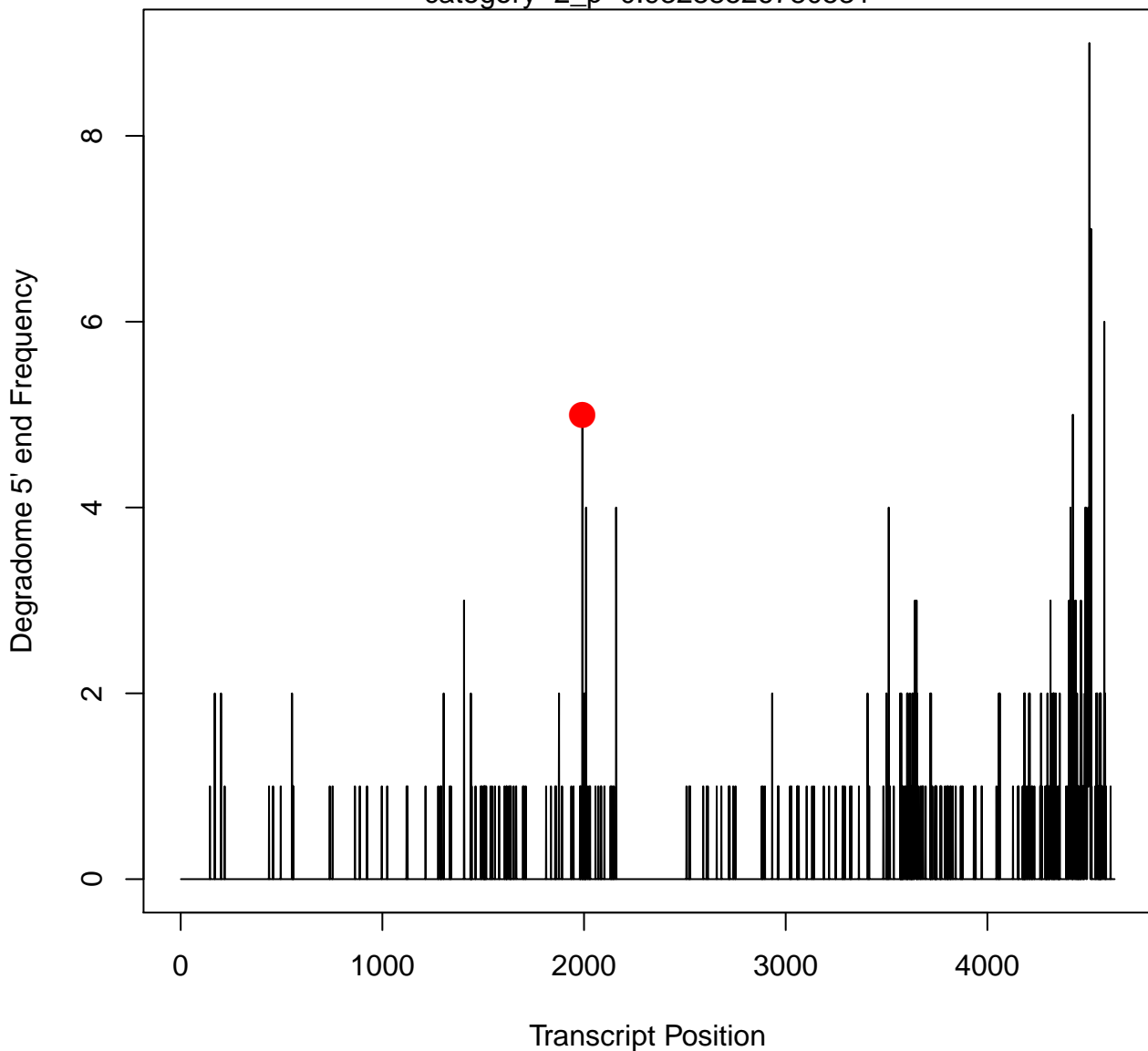

Supplement: Supplementary file 3 [file Data_Sheet_9.ZIP › GSM2230751.plot/Lsa-miR156j_Lsat_1_v5_gn_5_32220.1_1992_TPlot.pdf]

**T=Lsat\_1\_v5\_gn\_5\_42660.1\_Q=Lsa-miR156j\_S=1691**

category=2\_p=0.646185114290618

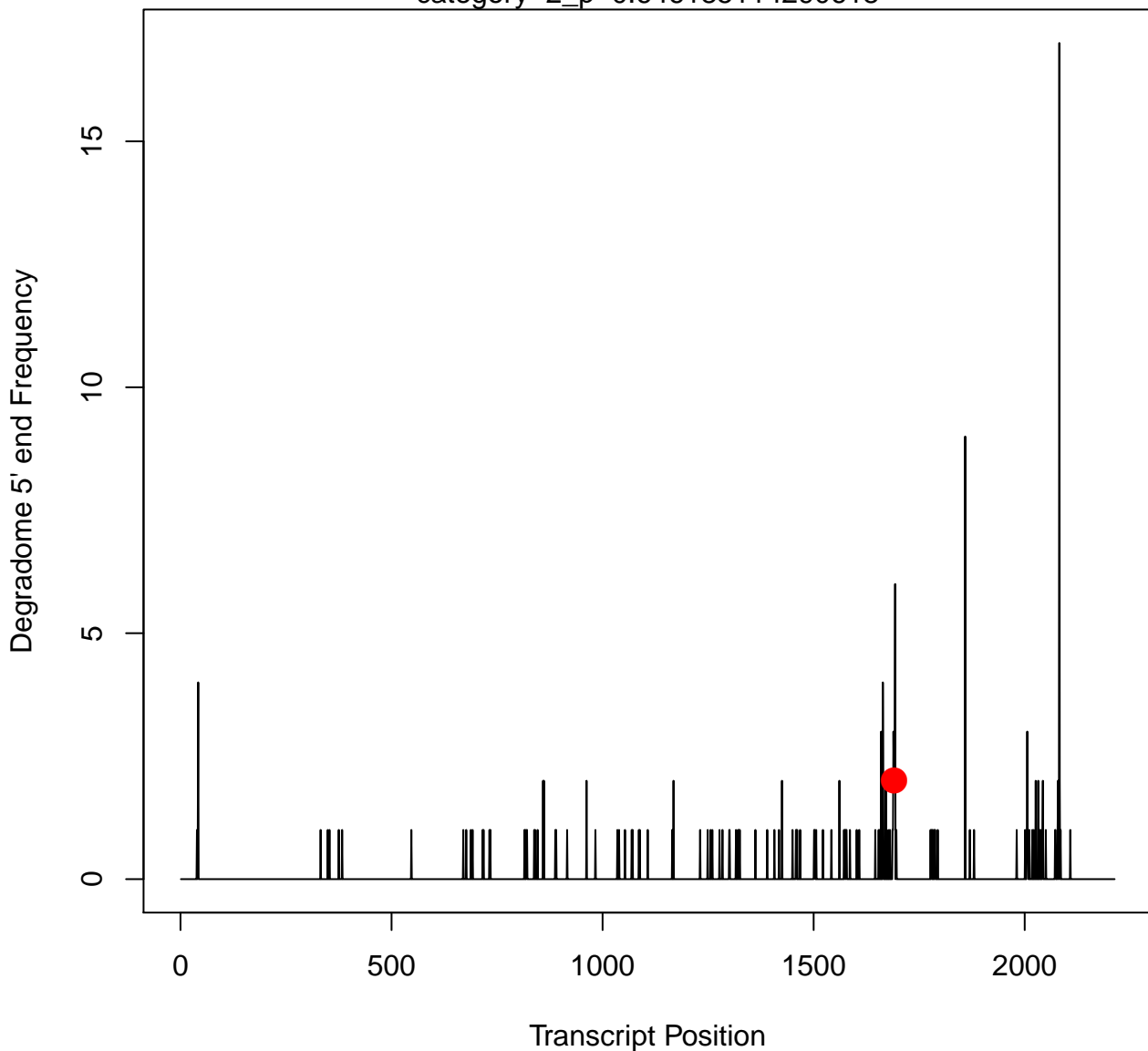

Supplement: Supplementary file 3 [file Data_Sheet_9.ZIP › GSM2230751.plot/Lsa-miR156j_Lsat_1_v5_gn_5_42660.1_1691_TPlot.pdf]

T=Lsat\_1\_v5\_gn\_5\_71601.1\_Q=Lsa-miR156j\_S=276

category=2\_p=0.685798933413206

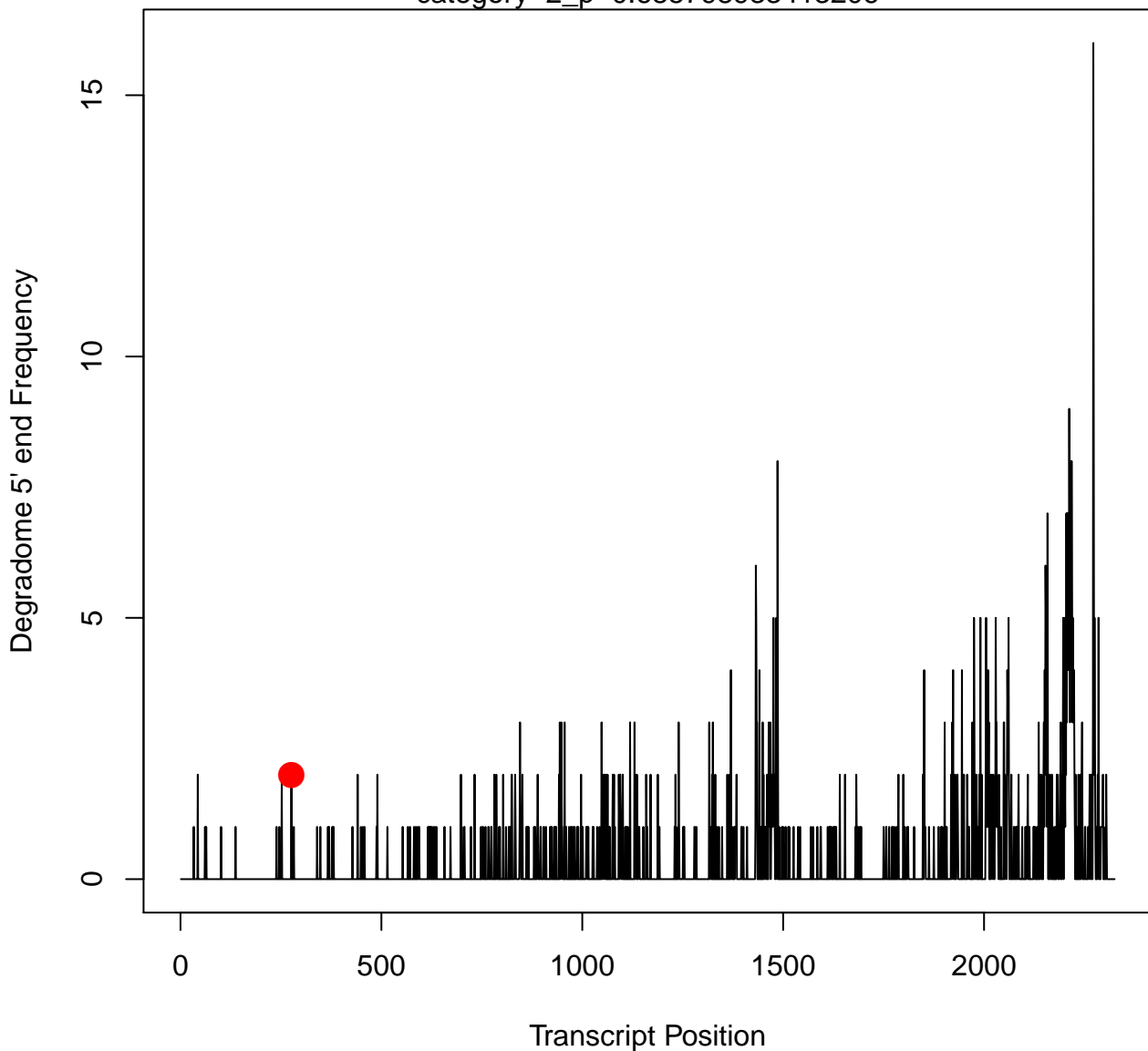

Supplement: Supplementary file 3 [file Data_Sheet_9.ZIP › GSM2230751.plot/Lsa-miR156j_Lsat_1_v5_gn_5_71601.1_276_TPlot.pdf]

**T=Lsat\_1\_v5\_gn\_6\_46020.1\_Q=Lsa-miR156j\_S=53**

category=2\_p=0.993374278586314

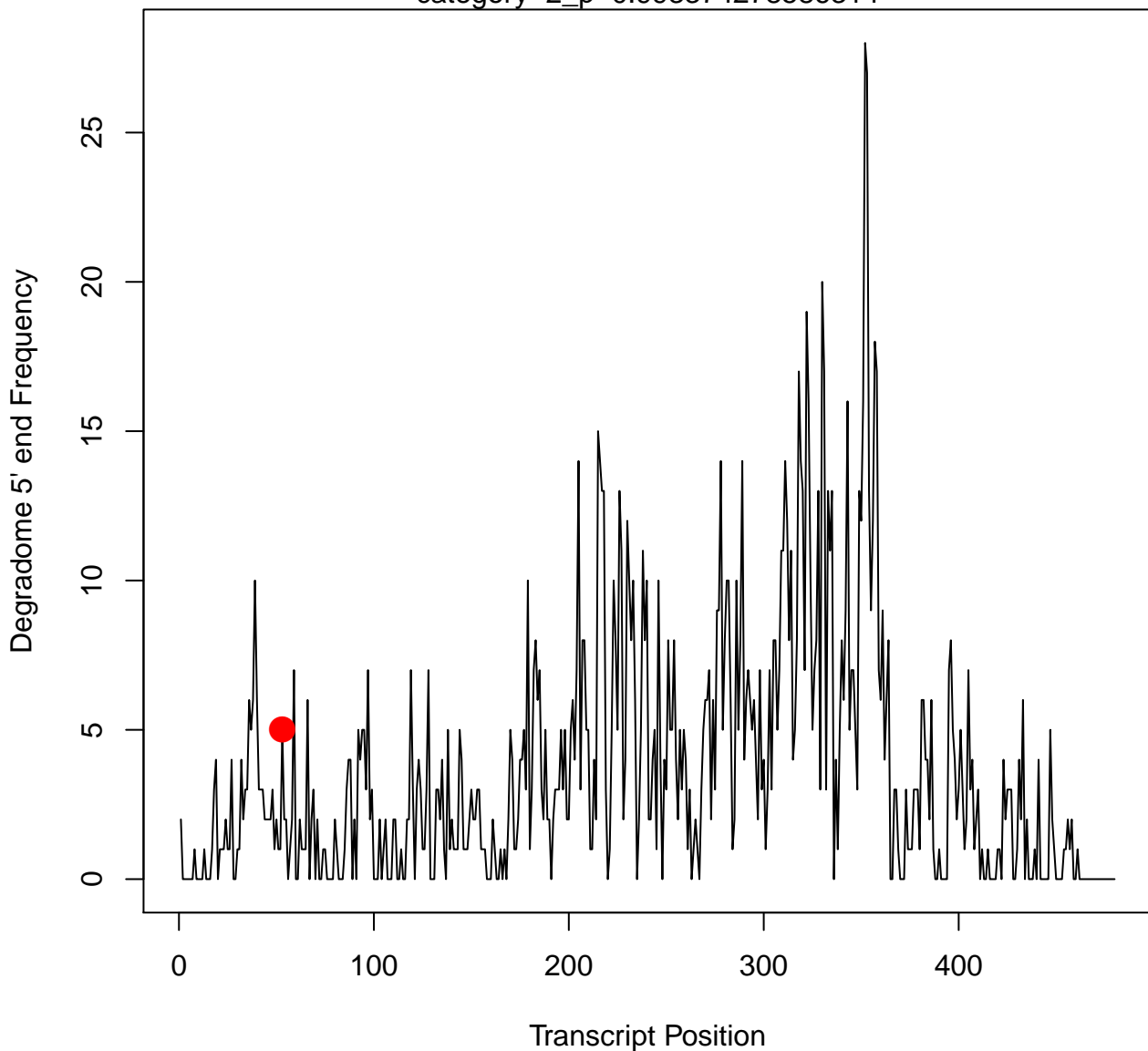

Supplement: Supplementary file 3 [file Data_Sheet_9.ZIP › GSM2230751.plot/Lsa-miR156j_Lsat_1_v5_gn_6_46020.1_53_TPlot.pdf]

**T=Lsat\_1\_v5\_gn\_8\_69380.1\_Q=Lsa-miR156j\_S=2571**

category=2\_p=0.99634075959037

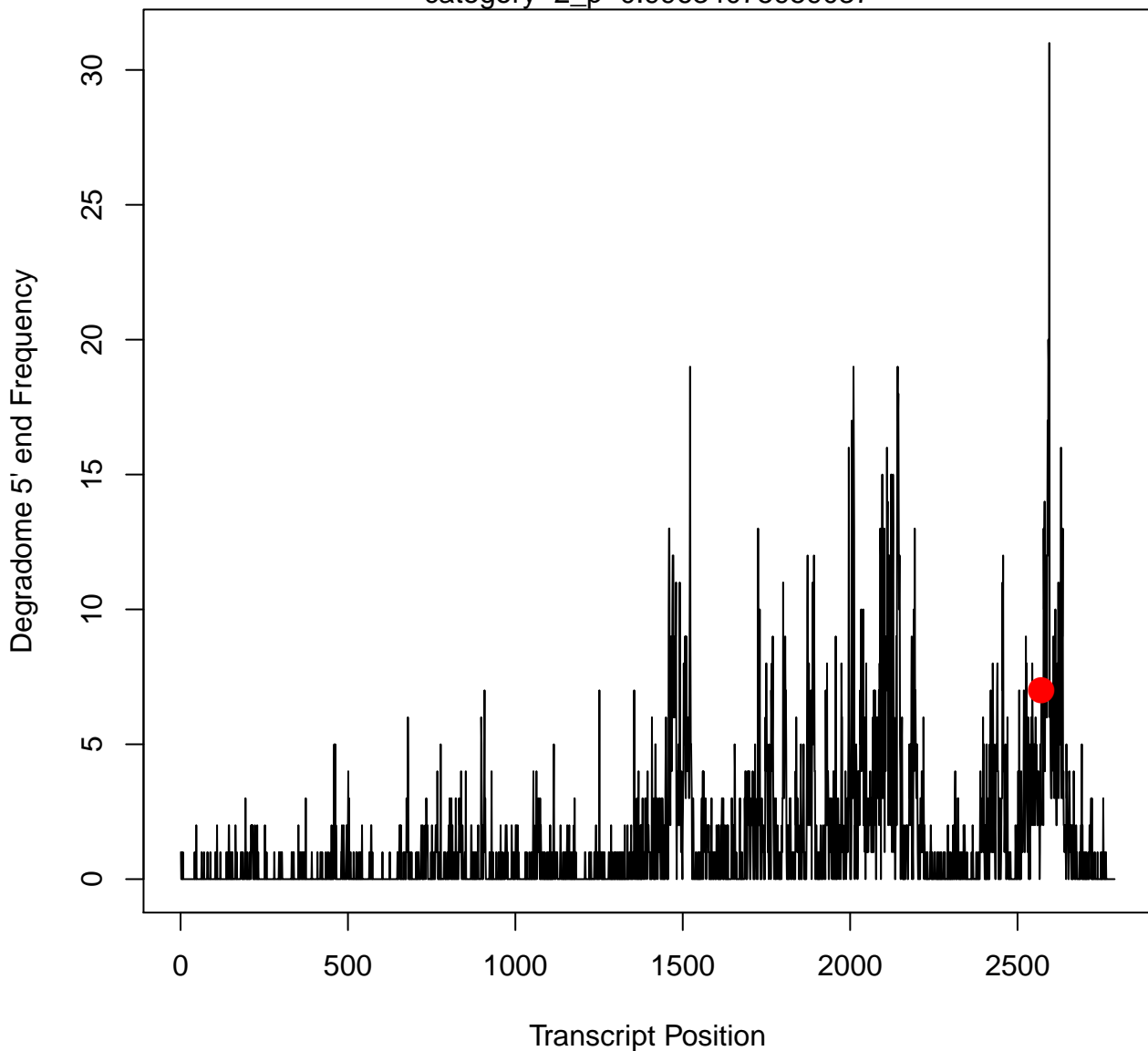

Supplement: Supplementary file 3 [file Data_Sheet_9.ZIP › GSM2230751.plot/Lsa-miR156j_Lsat_1_v5_gn_8_69380.1_2571_TPlot.pdf]

**T=Lsat\_1\_v5\_gn\_3\_31000.1\_Q=Lsa-miR157a\_S=849**

category=2\_p=0.0292489061411405

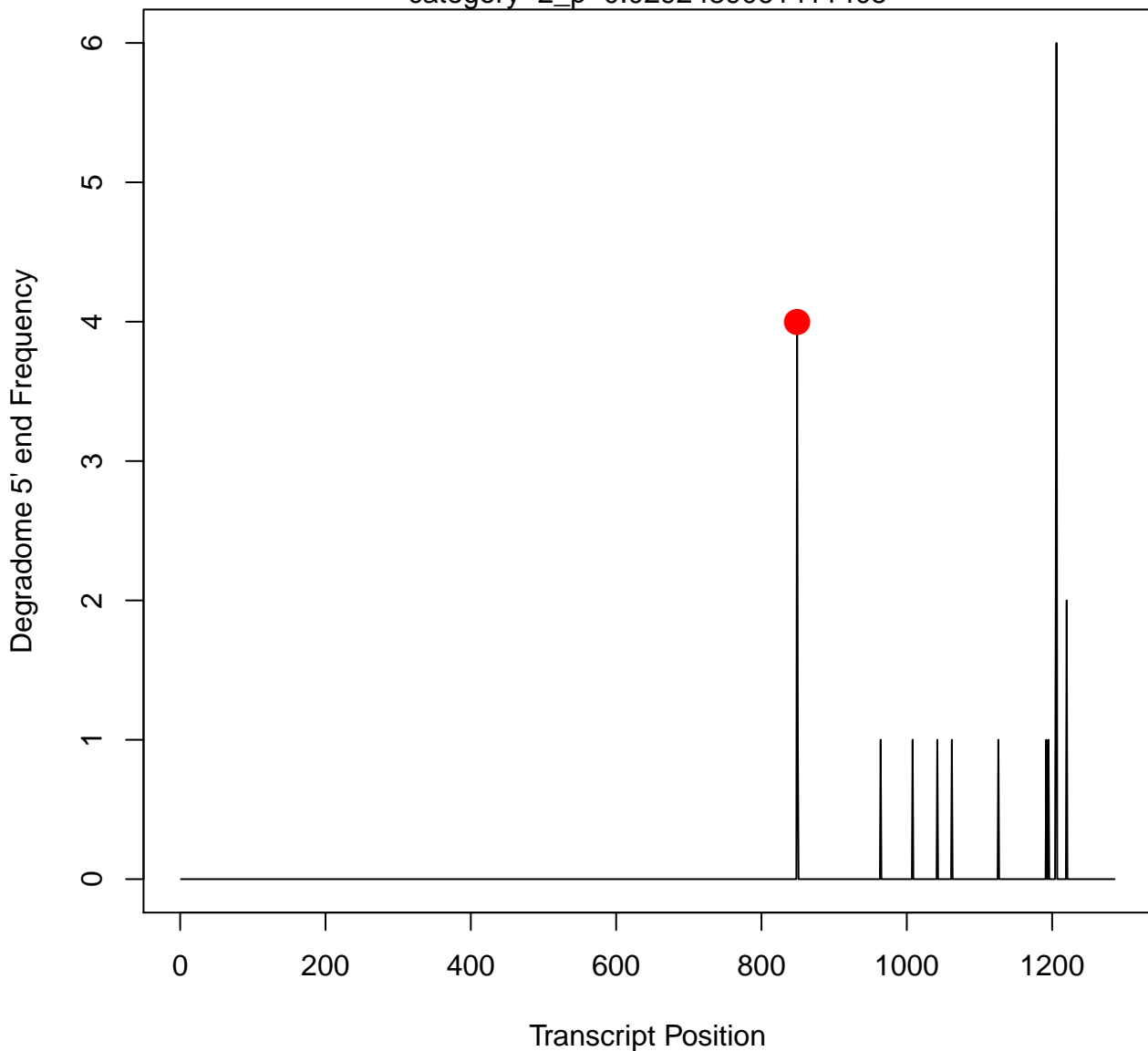

Supplement: Supplementary file 3 [file Data_Sheet_9.ZIP › GSM2230751.plot/Lsa-miR157a_Lsat_1_v5_gn_3_31000.1_849_TPlot.pdf]

**T=Lsat\_1\_v5\_gn\_4\_113241.1\_Q=Lsa-miR157a\_S=869**

category=0\_p=0.00405748181806054

Degradome 5' end Frequency

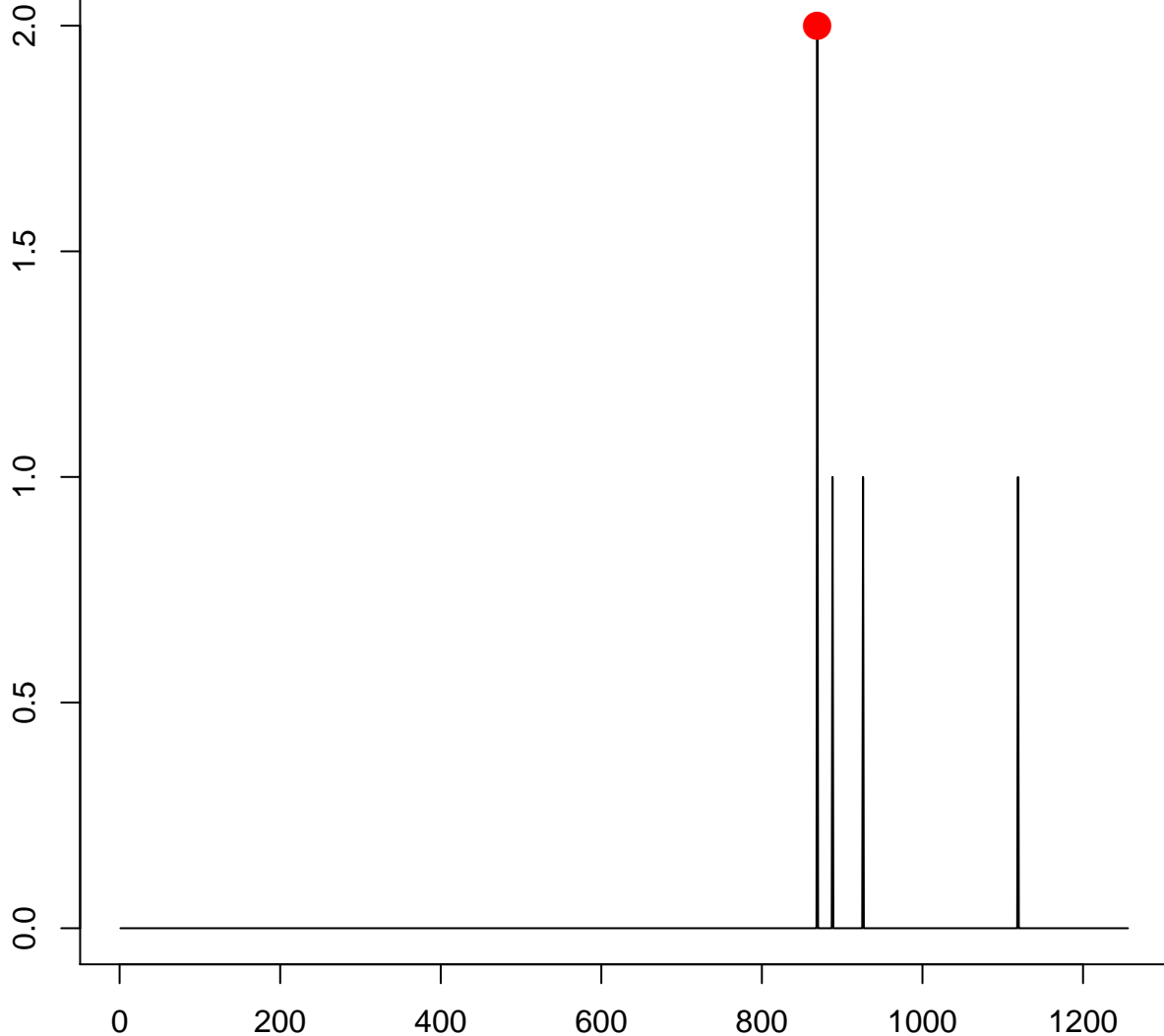

Transcript Position

Supplement: Supplementary file 3 [file Data_Sheet_9.ZIP › GSM2230751.plot/Lsa-miR157a_Lsat_1_v5_gn_4_113241.1_869_TPlot.pdf]

**T=Lsat\_1\_v5\_gn\_4\_1141.1\_Q=Lsa-miR157a\_S=1409**

category=0\_p=0.00147735698913964

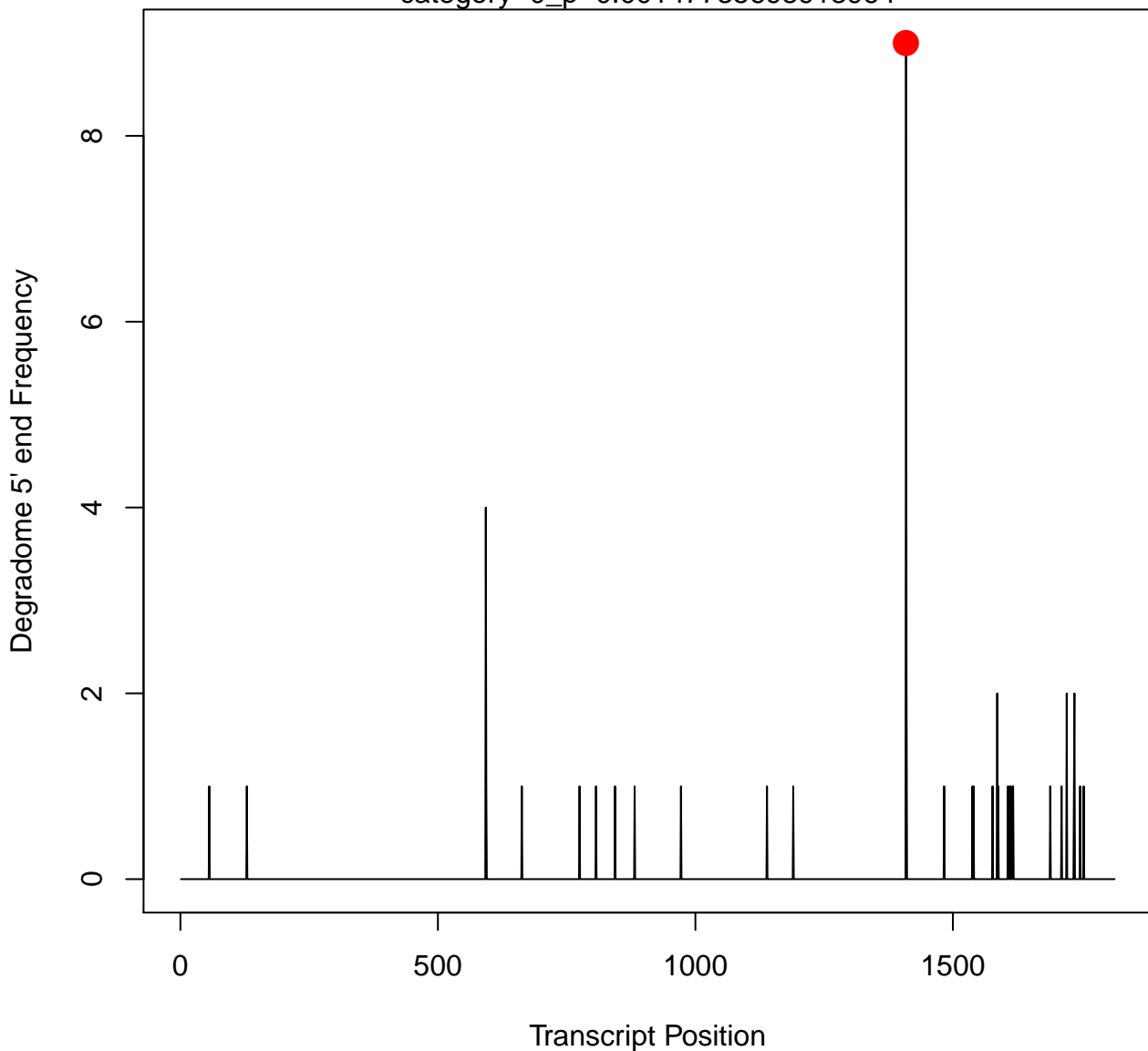

Supplement: Supplementary file 3 [file Data_Sheet_9.ZIP › GSM2230751.plot/Lsa-miR157a_Lsat_1_v5_gn_4_1141.1_1409_TPlot.pdf]

**T=Lsat\_1\_v5\_gn\_4\_33541.1\_Q=Lsa-miR157a\_S=451**

category=2\_p=0.666579822949821

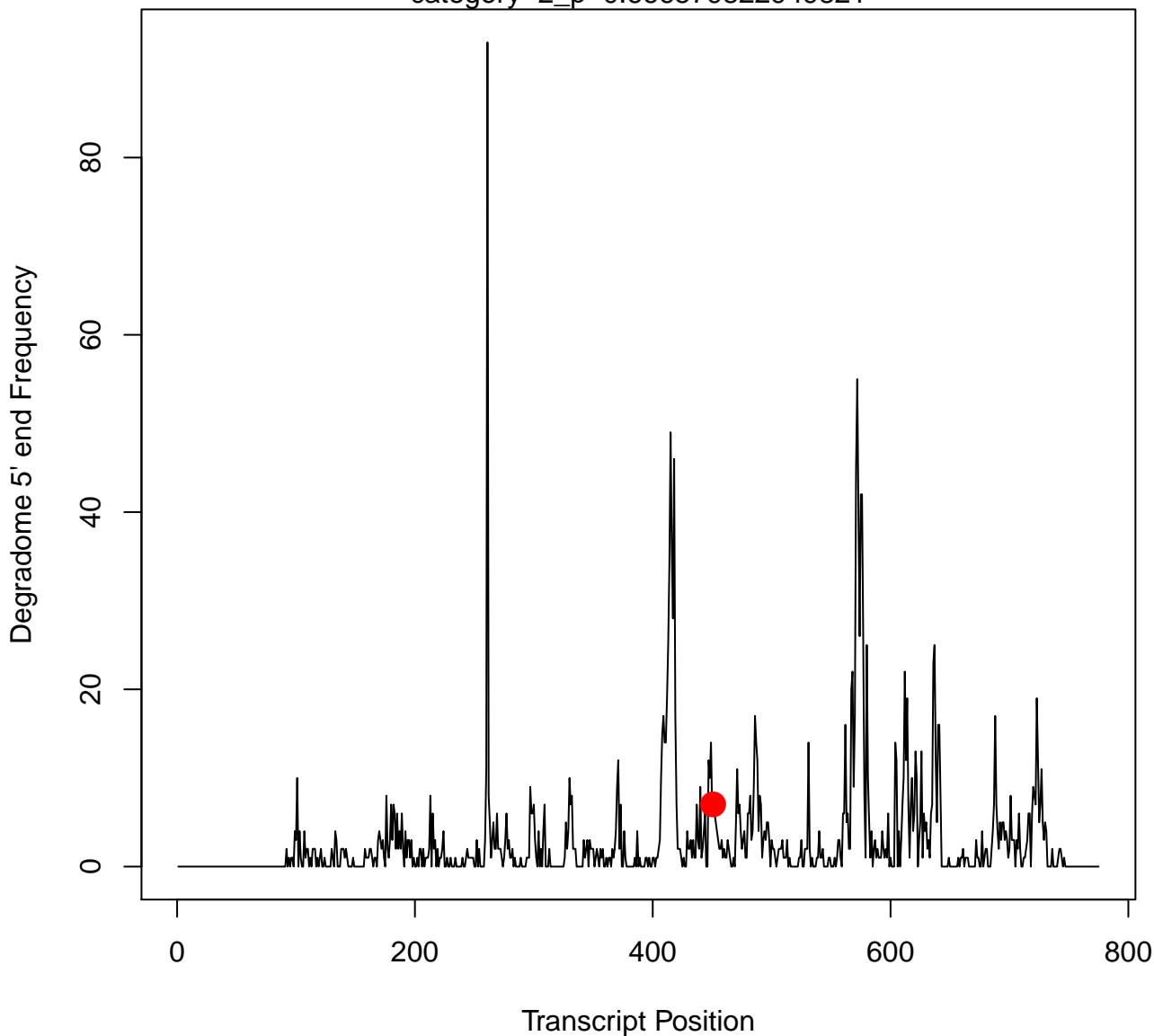

Supplement: Supplementary file 3 [file Data_Sheet_9.ZIP › GSM2230751.plot/Lsa-miR157a_Lsat_1_v5_gn_4_33541.1_451_TPlot.pdf]

**T=Lsat\_1\_v5\_gn\_4\_421.1\_Q=Lsa-miR157a\_S=2360**

category=0\_p=0.000738951519243747

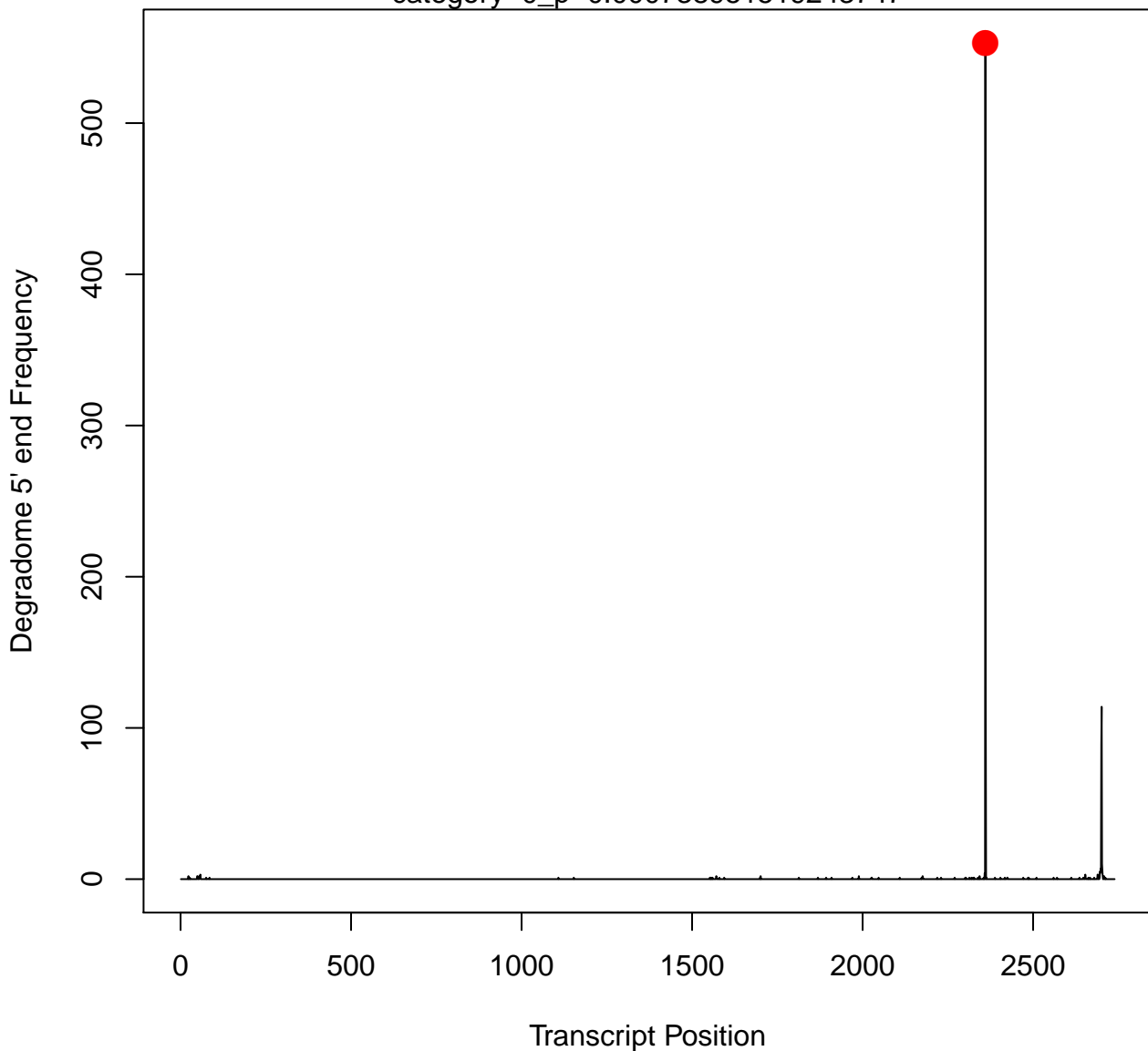

Supplement: Supplementary file 3 [file Data_Sheet_9.ZIP › GSM2230751.plot/Lsa-miR157a_Lsat_1_v5_gn_4_421.1_2360_TPlot.pdf]

**T=Lsat\_1\_v5\_gn\_5\_142161.1\_Q=Lsa-miR157a\_S=3504**

category=2\_p=0.85903196472452

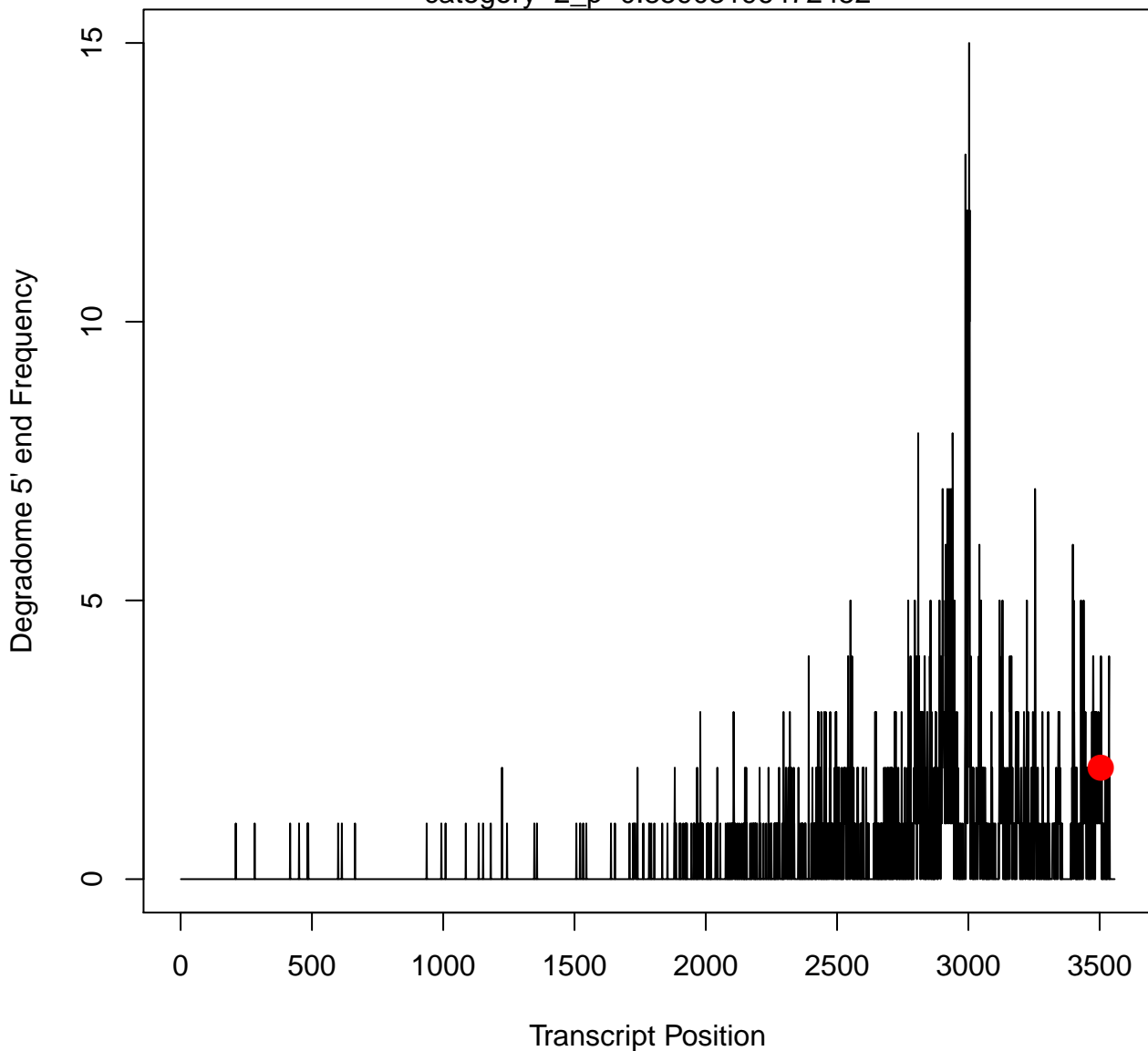

Supplement: Supplementary file 3 [file Data_Sheet_9.ZIP › GSM2230751.plot/Lsa-miR157a_Lsat_1_v5_gn_5_142161.1_3504_TPlot.pdf]

**T=Lsat\_1\_v5\_gn\_9\_4540.1\_Q=Lsa-miR157a\_S=325**

category=2\_p=0.759464793296244

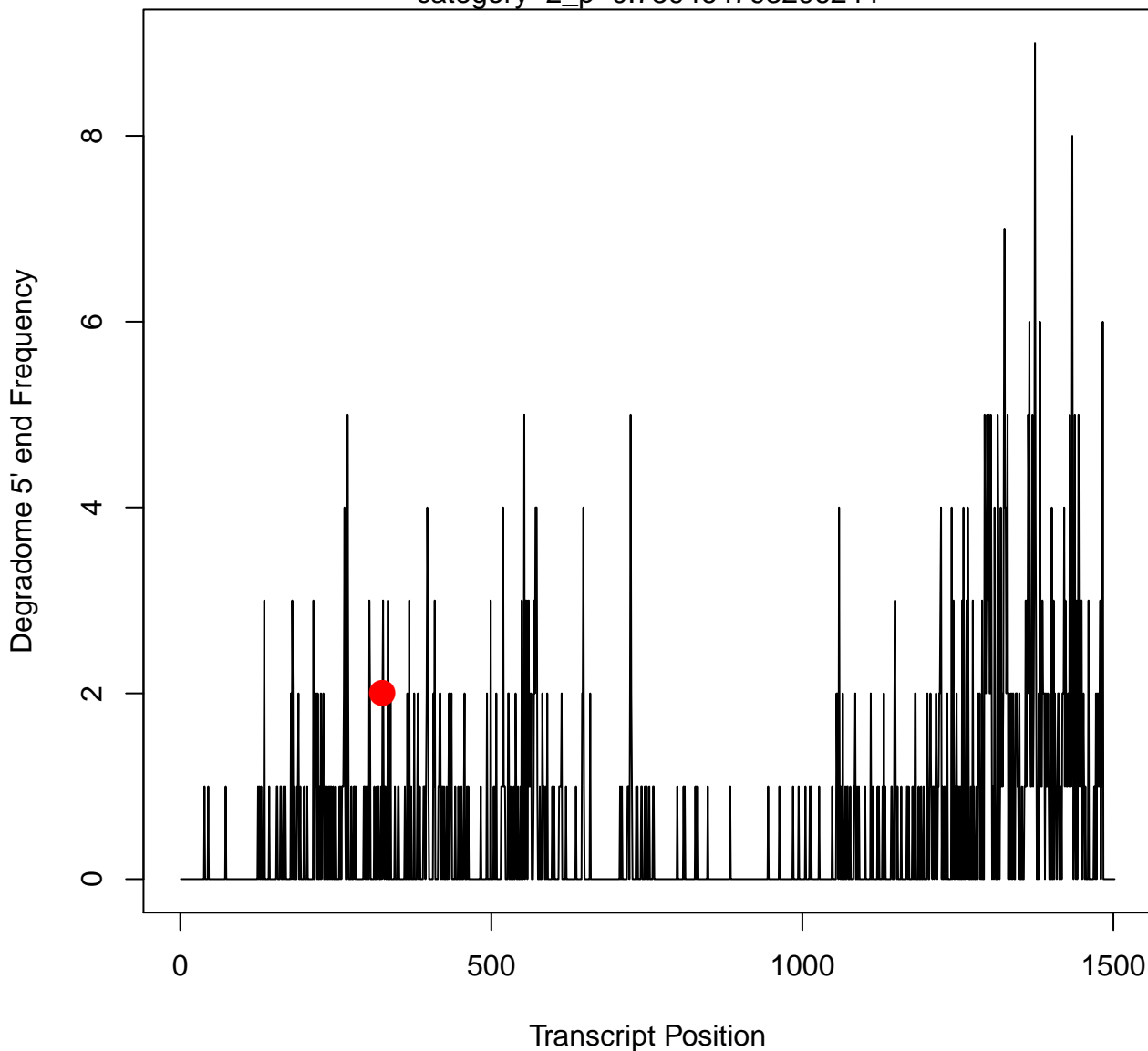

Supplement: Supplementary file 3 [file Data_Sheet_9.ZIP › GSM2230751.plot/Lsa-miR157a_Lsat_1_v5_gn_9_4540.1_325_TPlot.pdf]

**T=Lsat\_1\_v5\_gn\_4\_33541.1\_Q=Lsa-miR157b\_S=452**

category=2\_p=0.666579822949821

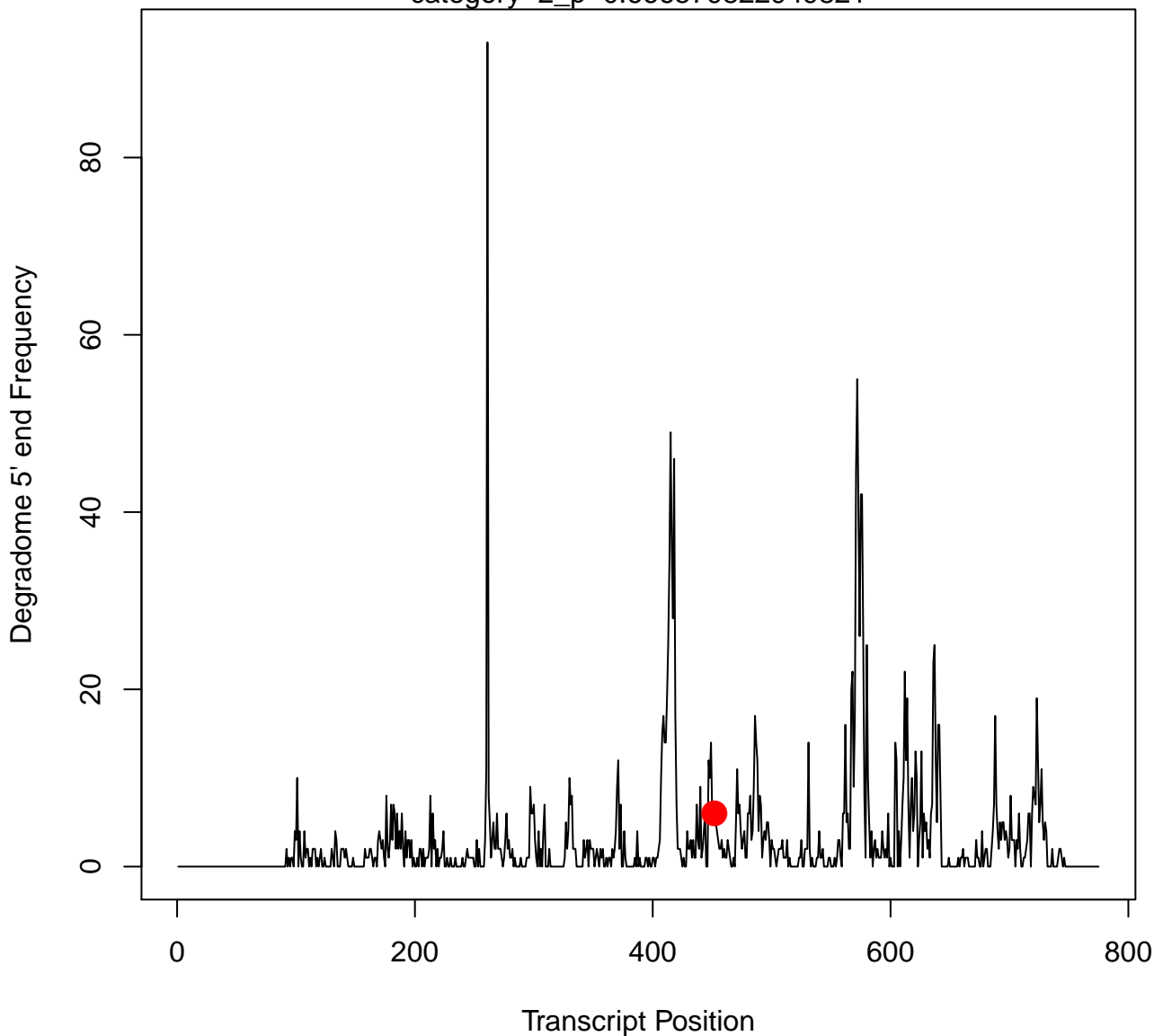

Supplement: Supplementary file 3 [file Data_Sheet_9.ZIP › GSM2230751.plot/Lsa-miR157b_Lsat_1_v5_gn_4_33541.1_452_TPlot.pdf]

**T=Lsat\_1\_v5\_gn\_7\_11040.1\_Q=Lsa-miR157b\_S=1088**

category=2\_p=0.137936131575646

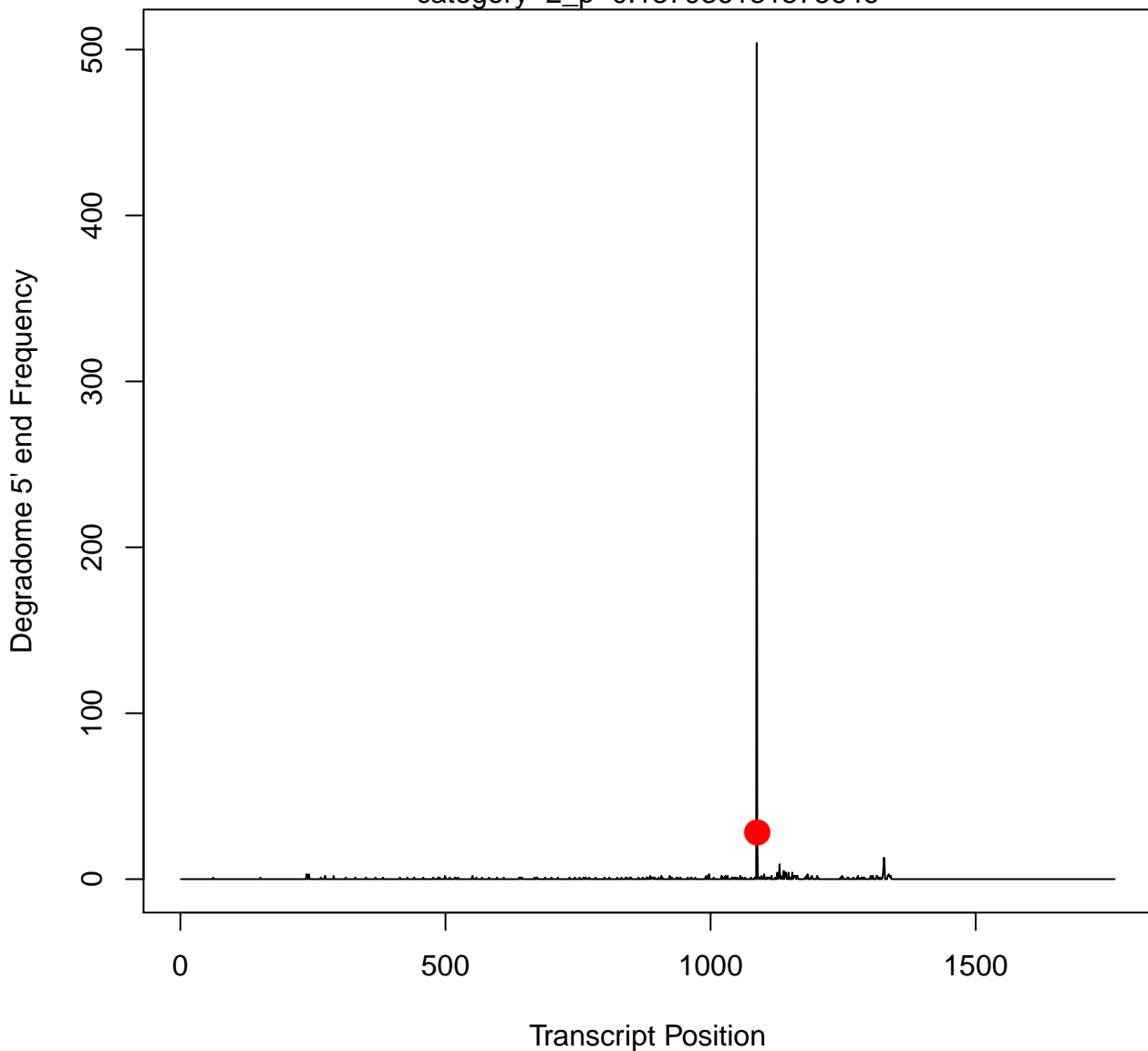

Supplement: Supplementary file 3 [file Data_Sheet_9.ZIP › GSM2230751.plot/Lsa-miR157b_Lsat_1_v5_gn_7_11040.1_1088_TPlot.pdf]

**T=Lsat\_1\_v5\_gn\_9\_28021.1\_Q=Lsa-miR157b\_S=1268**

category=2\_p=0.111961991206685

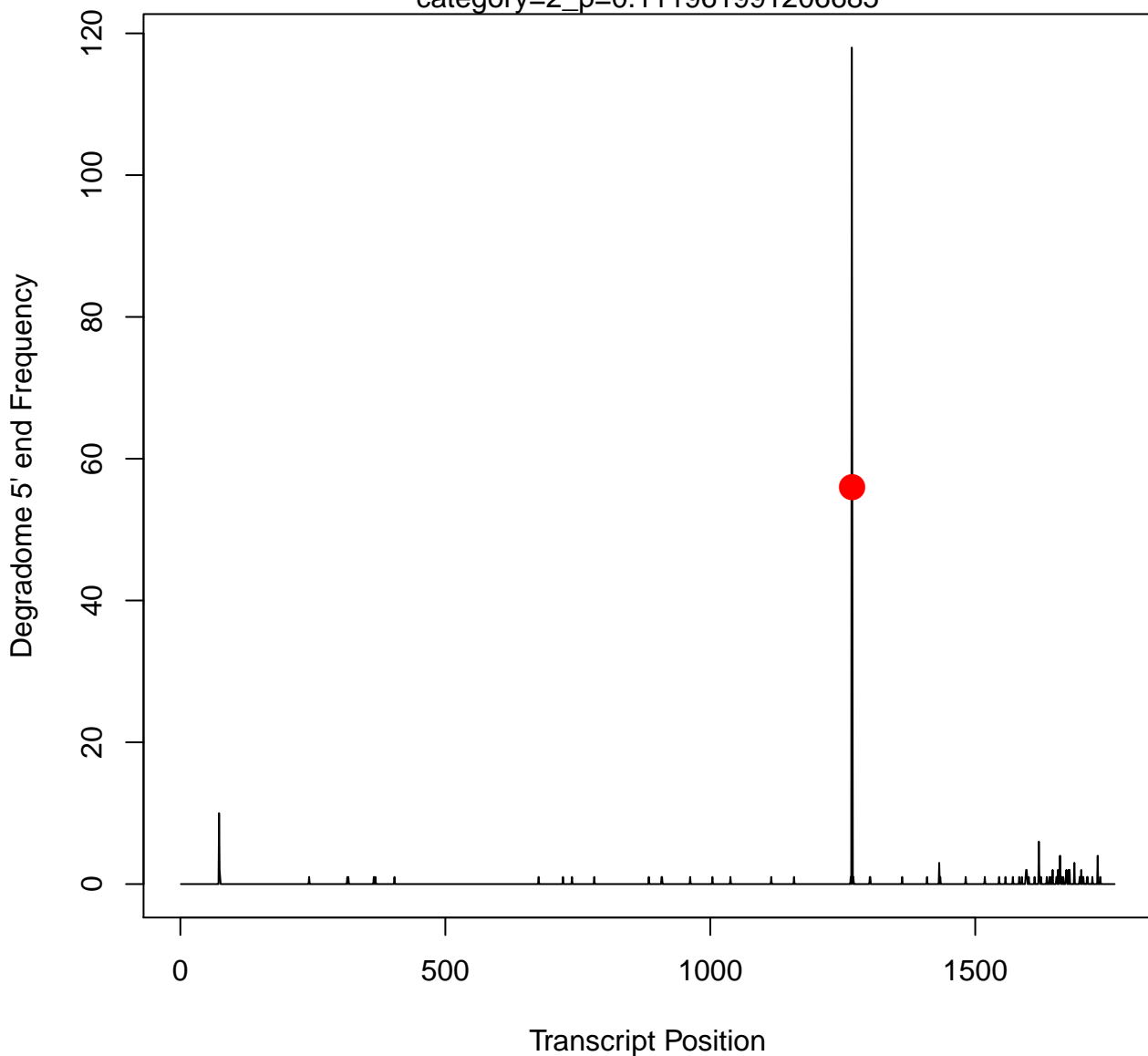

Supplement: Supplementary file 3 [file Data_Sheet_9.ZIP › GSM2230751.plot/Lsa-miR157b_Lsat_1_v5_gn_9_28021.1_1268_TPlot.pdf]

**T=Lsat\_1\_v5\_gn\_4\_1141.1\_Q=Lsa-miR157d\_S=1410**

category=2\_p=0.137936131575646

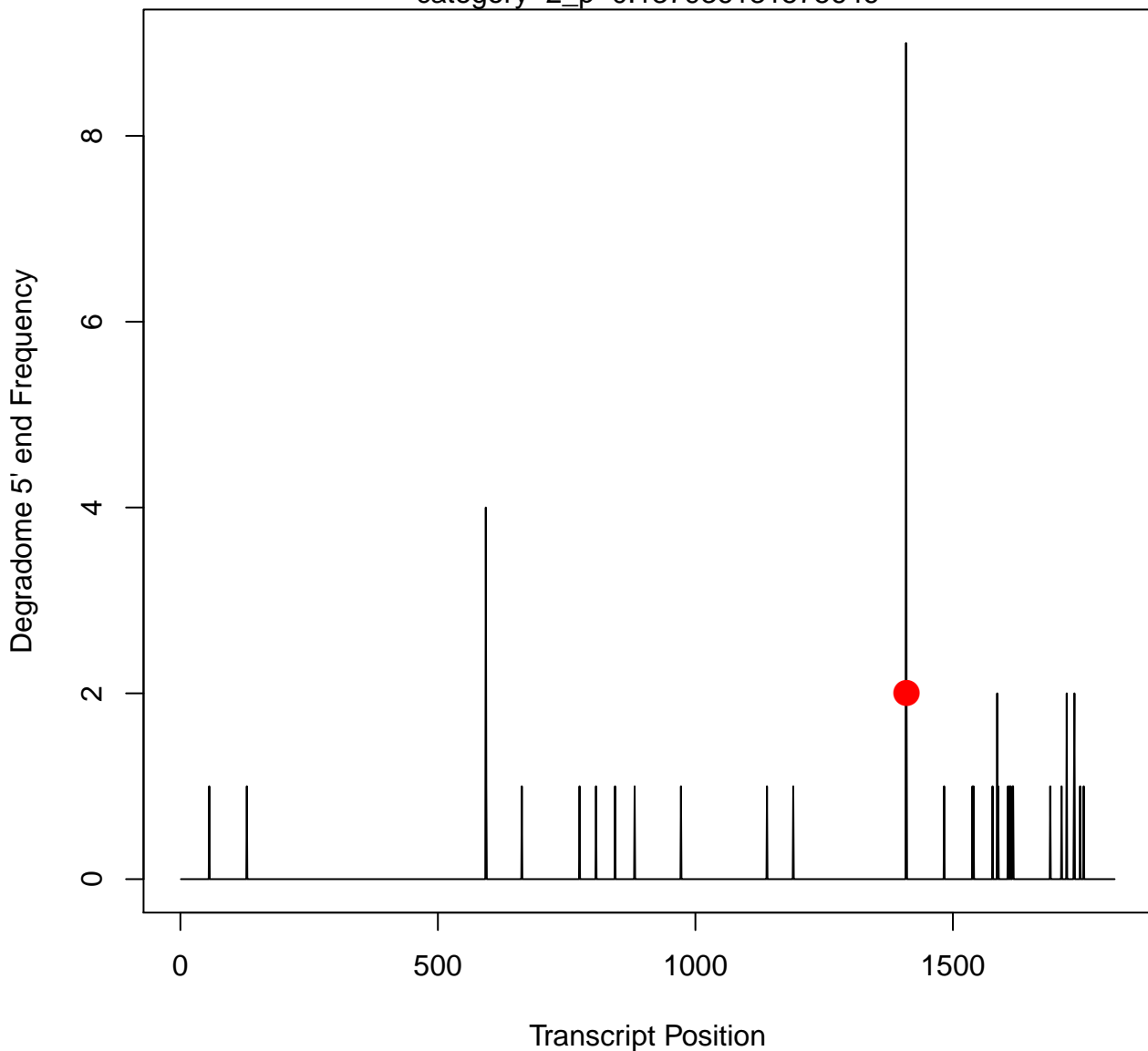

Supplement: Supplementary file 3 [file Data_Sheet_9.ZIP › GSM2230751.plot/Lsa-miR157d_Lsat_1_v5_gn_4_1141.1_1410_TPlot.pdf]

**T=Lsat\_1\_v5\_gn\_4\_421.1\_Q=Lsa-miR157d\_S=2361**

category=2\_p=0.0292489061411405

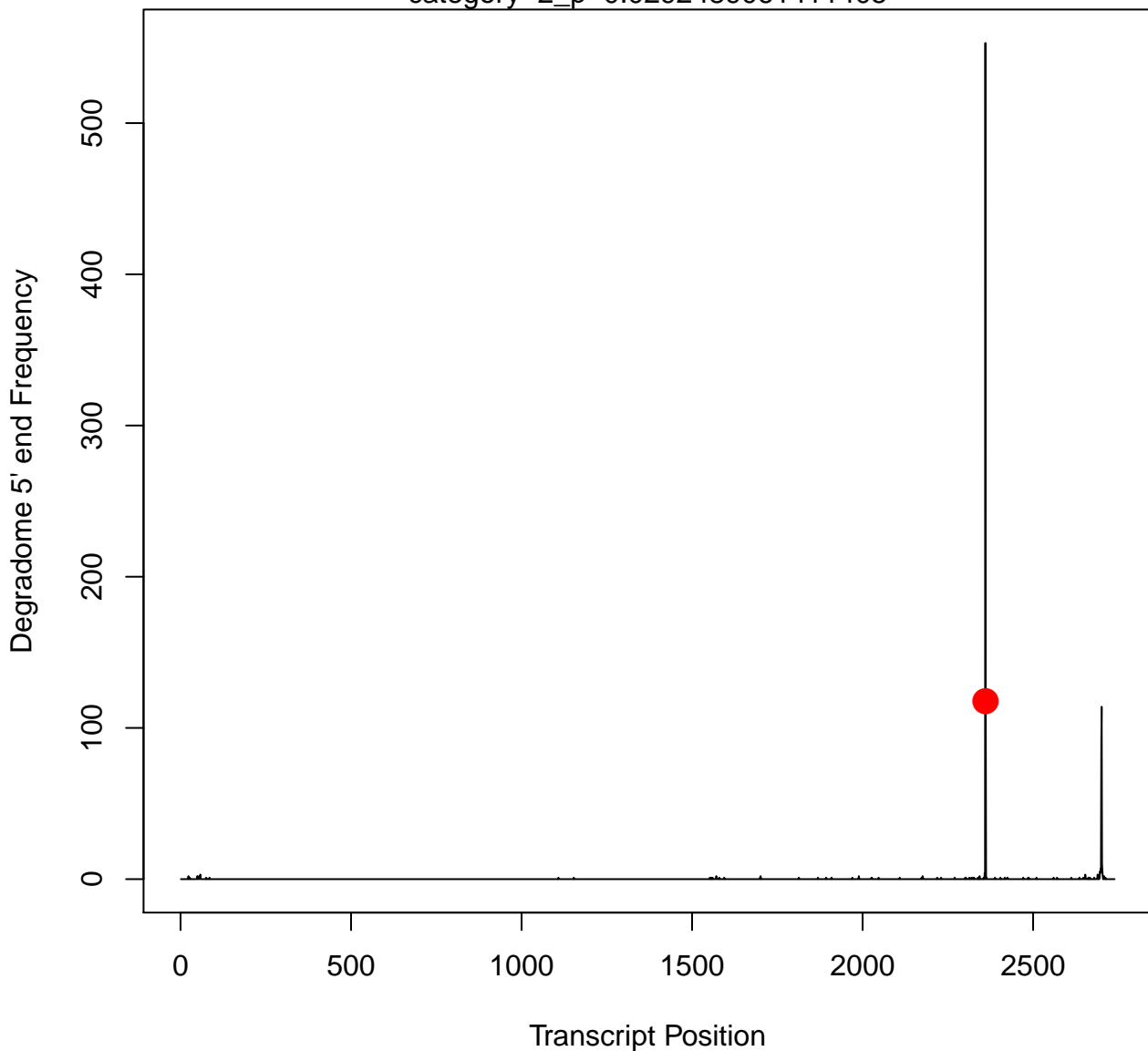

Supplement: Supplementary file 3 [file Data_Sheet_9.ZIP › GSM2230751.plot/Lsa-miR157d_Lsat_1_v5_gn_4_421.1_2361_TPlot.pdf]

**T=Lsat\_1\_v5\_gn\_5\_142161.1\_Q=Lsa-miR157d\_S=3505**

category=2\_p=0.885481443011039

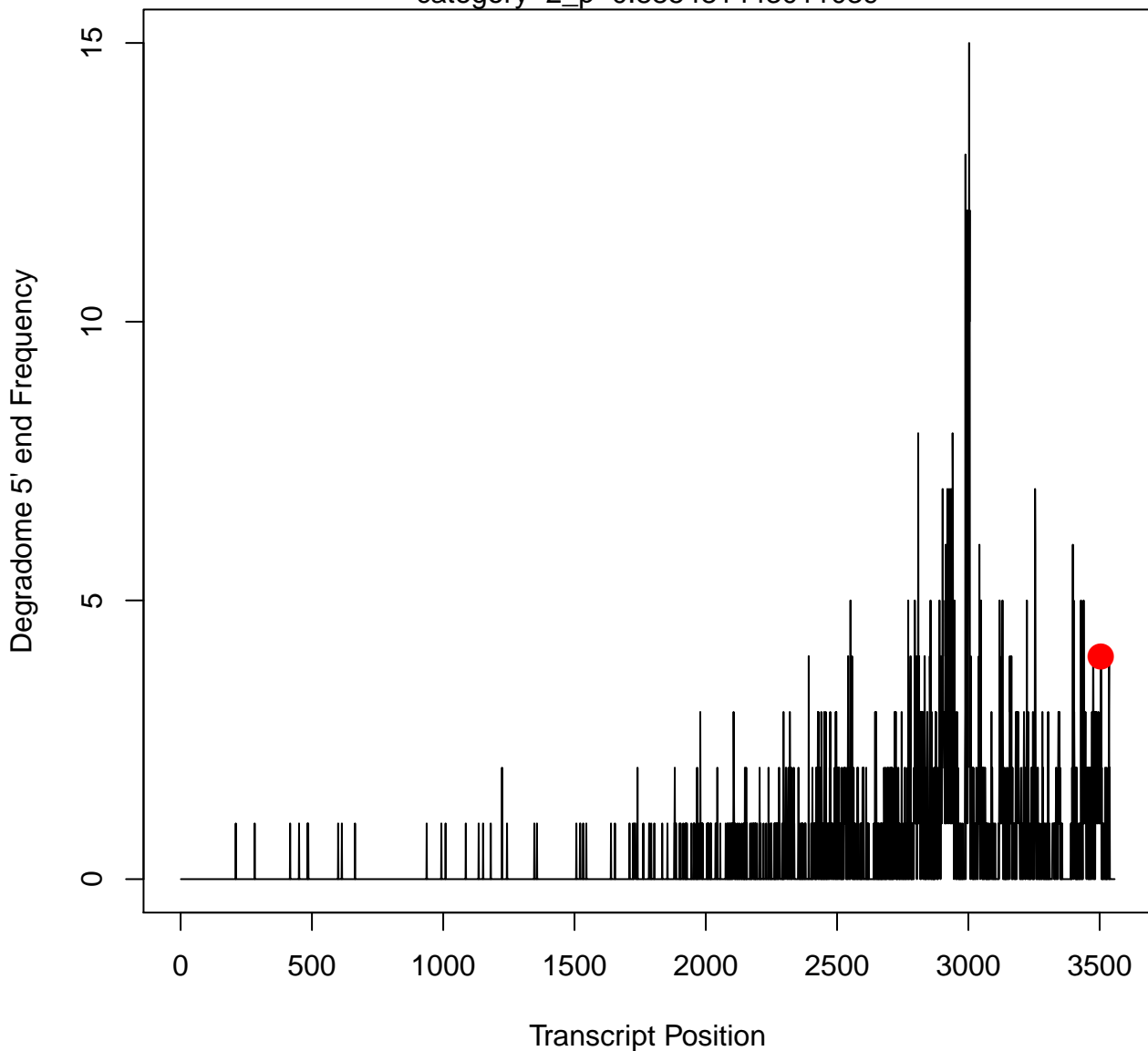

Supplement: Supplementary file 3 [file Data_Sheet_9.ZIP › GSM2230751.plot/Lsa-miR157d_Lsat_1_v5_gn_5_142161.1_3505_TPlot.pdf]

**T=Lsat\_1\_v5\_gn\_2\_134361.1\_Q=Lsa-miR157e\_S=223**

category=0\_p=0.0117579238066935

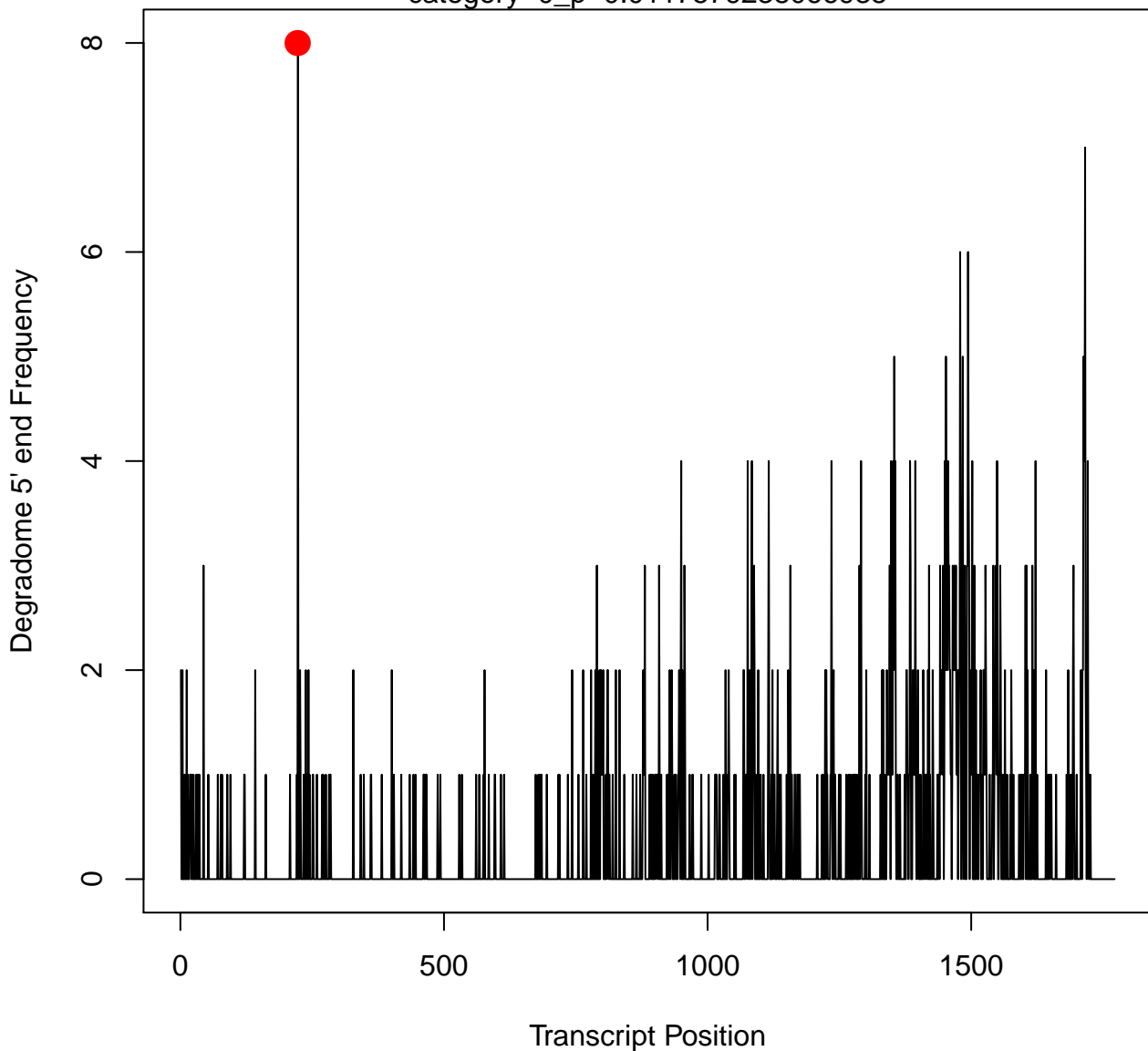

Supplement: Supplementary file 3 [file Data_Sheet_9.ZIP › GSM2230751.plot/Lsa-miR157e_Lsat_1_v5_gn_2_134361.1_223_TPlot.pdf]

**T=Lsat\_1\_v5\_gn\_5\_12360.1\_Q=Lsa-miR157e\_S=1860**

category=2\_p=0.187627487570719

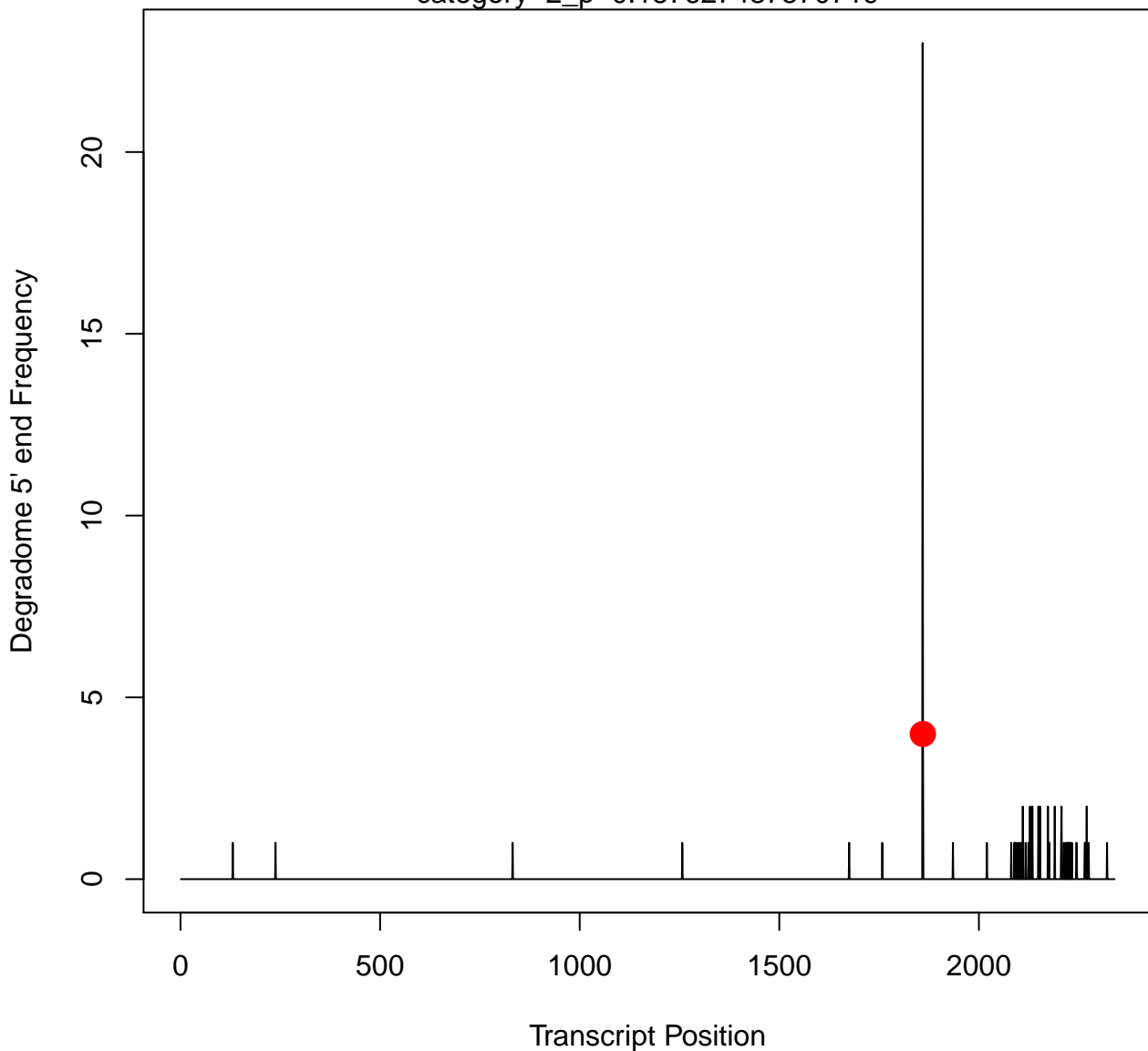

Supplement: Supplementary file 3 [file Data_Sheet_9.ZIP › GSM2230751.plot/Lsa-miR157e_Lsat_1_v5_gn_5_12360.1_1860_TPlot.pdf]

**T=Lsat\_1\_v5\_gn\_8\_134640.1\_Q=Lsa-miR157e\_S=547**

category=0\_p=0.00295253139460594

Degradome 5' end Frequency

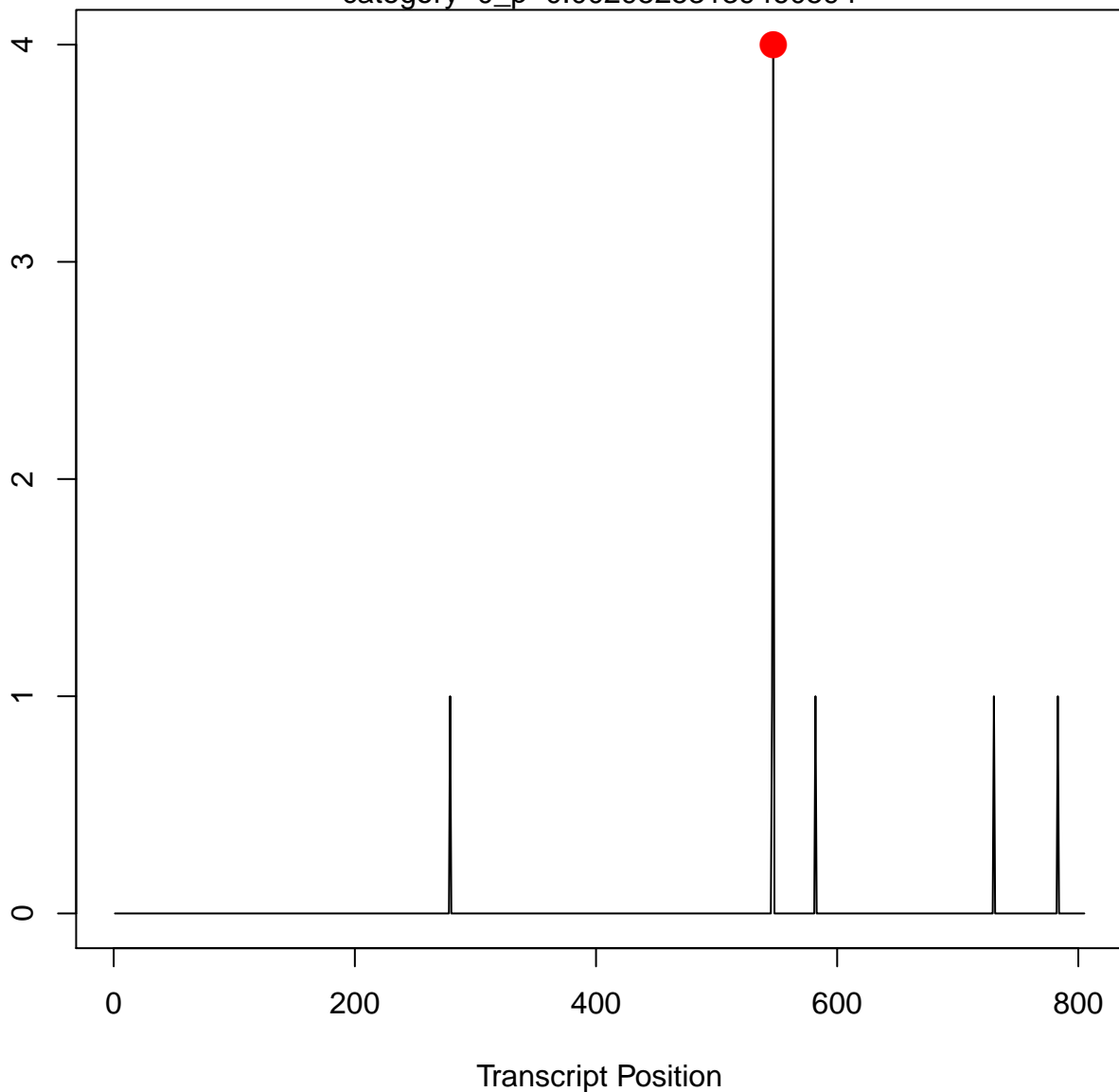

Supplement: Supplementary file 3 [file Data_Sheet_9.ZIP › GSM2230751.plot/Lsa-miR157e_Lsat_1_v5_gn_8_134640.1_547_TPlot.pdf]

**T=Lsat\_1\_v5\_gn\_9\_4540.1\_Q=Lsa-miR157e\_S=326**

category=2\_p=0.804595809802373

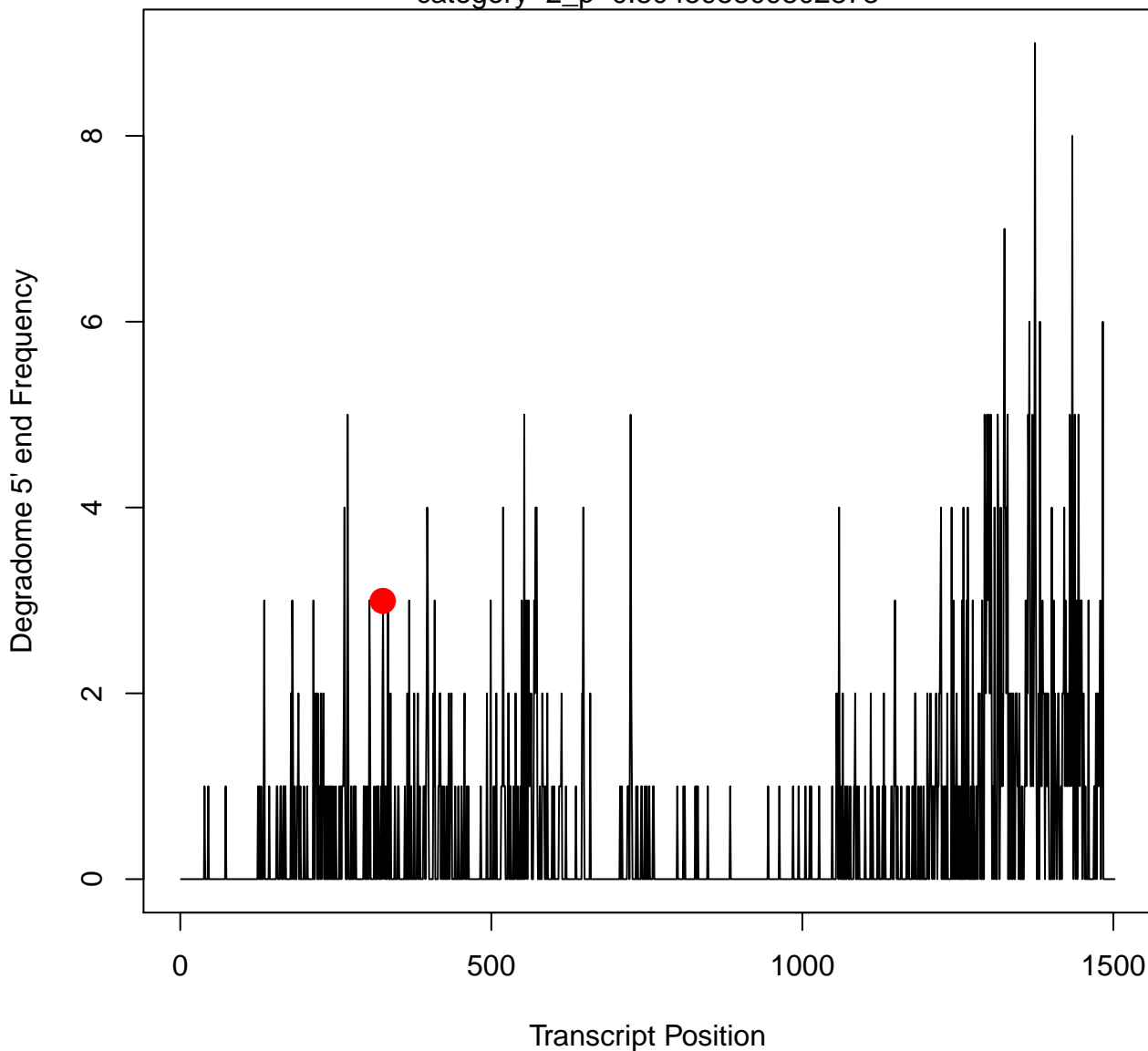

Supplement: Supplementary file 3 [file Data_Sheet_9.ZIP › GSM2230751.plot/Lsa-miR157e_Lsat_1_v5_gn_9_4540.1_326_TPlot.pdf]

T=Lsat\_1\_v5\_gn\_4\_12381.1\_Q=Lsa-miR159a\_S=621

category=2\_p=0.163150556750859

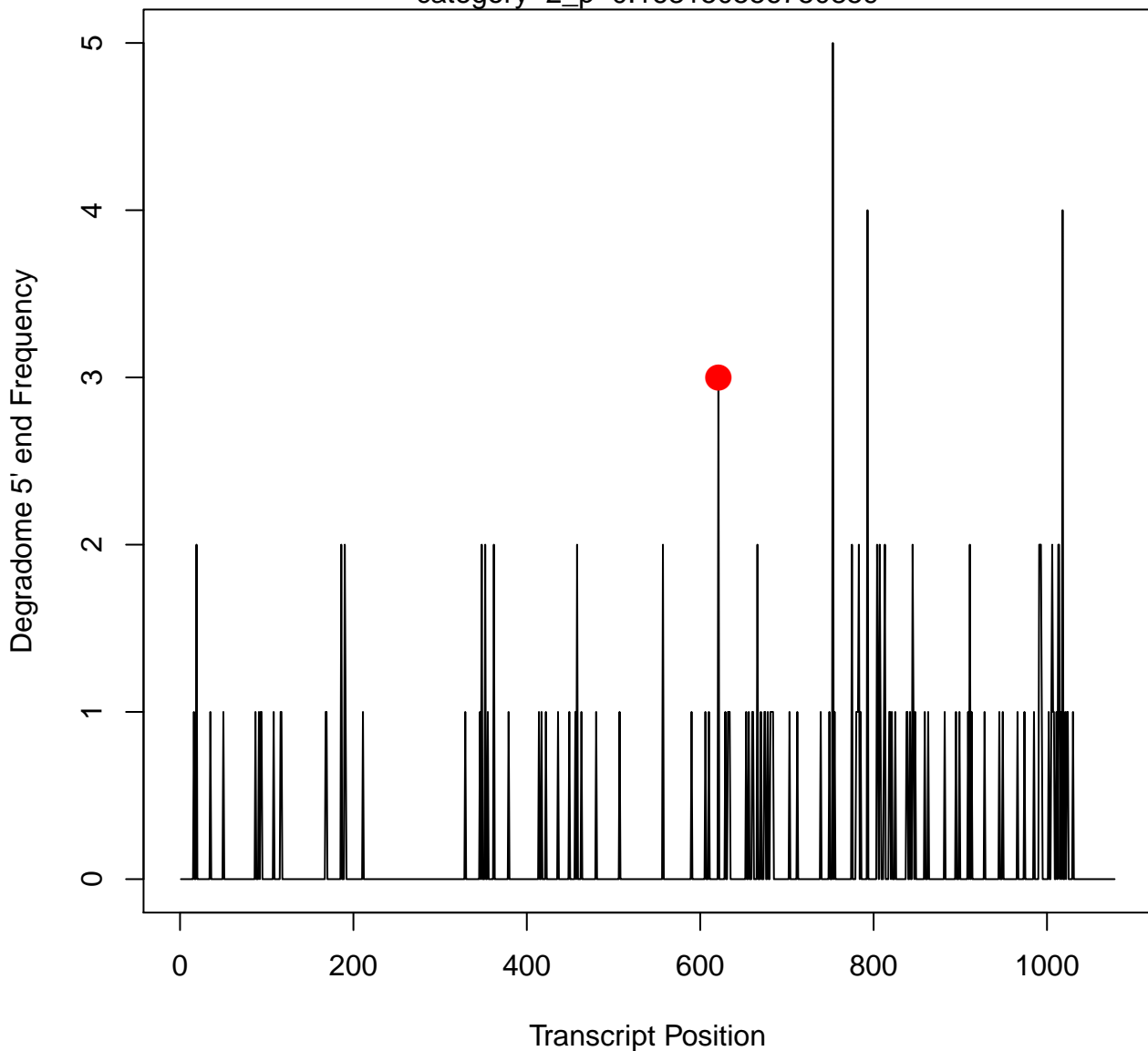

Supplement: Supplementary file 3 [file Data_Sheet_9.ZIP › GSM2230751.plot/Lsa-miR159a_Lsat_1_v5_gn_4_12381.1_621_TPlot.pdf]

**T=Lsat\_1\_v5\_gn\_5\_129201.1\_Q=Lsa-miR159a\_S=733**

category=0\_p=0.00589634534757577

Degradsome 5' end Frequency

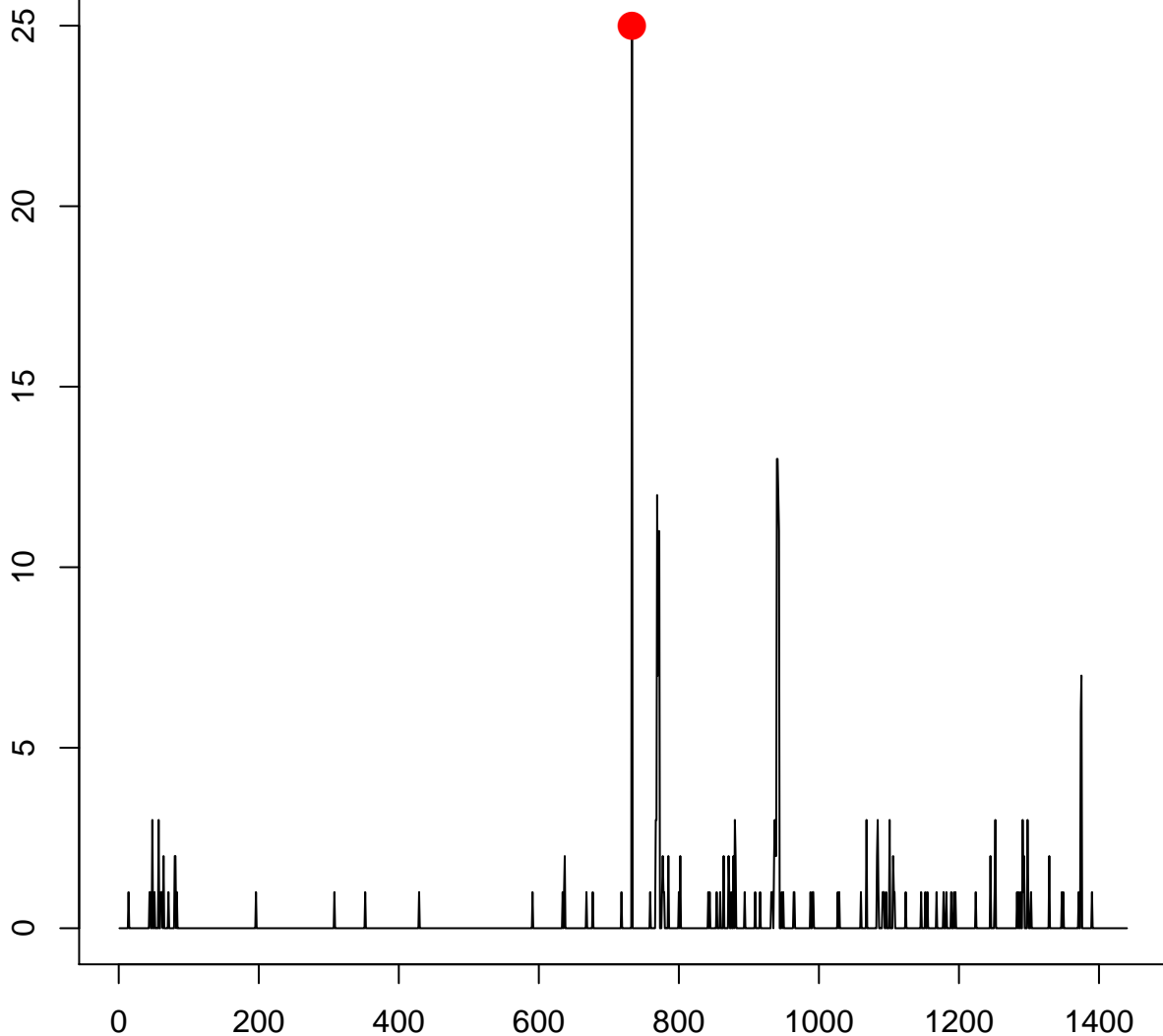

Transcript Position

Supplement: Supplementary file 3 [file Data_Sheet_9.ZIP › GSM2230751.plot/Lsa-miR159a_Lsat_1_v5_gn_5_129201.1_733_TPlot.pdf]

**T=Lsat\_1\_v5\_gn\_5\_169160.1\_Q=Lsa-miR159a\_S=490**

category=2\_p=0.971624296947987

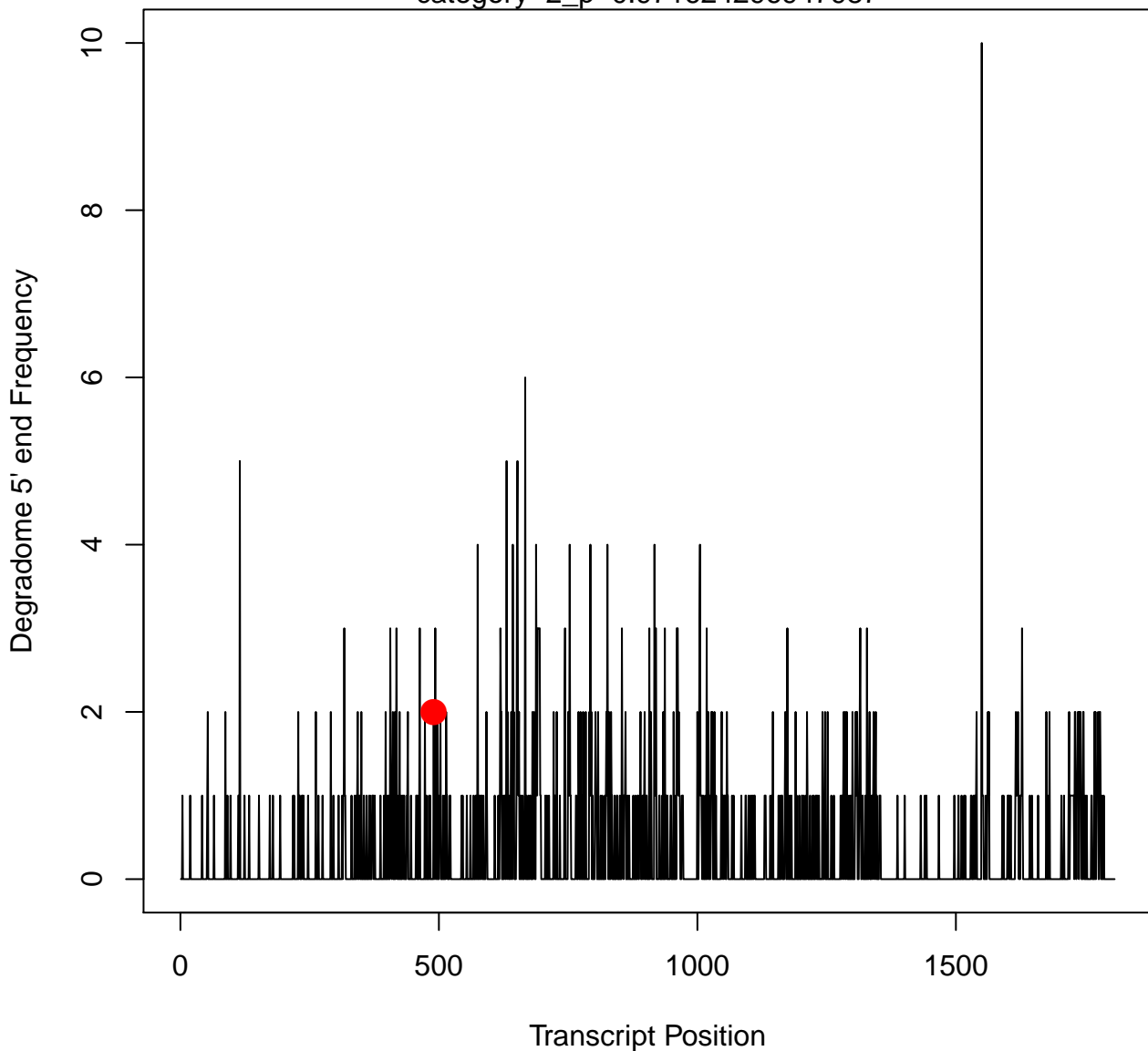

Supplement: Supplementary file 3 [file Data_Sheet_9.ZIP › GSM2230751.plot/Lsa-miR159a_Lsat_1_v5_gn_5_169160.1_490_TPlot.pdf]

**T=Lsat\_1\_v5\_gn\_5\_16921.1\_Q=Lsa-miR159a\_S=1514**

category=2\_p=0.999119823851691

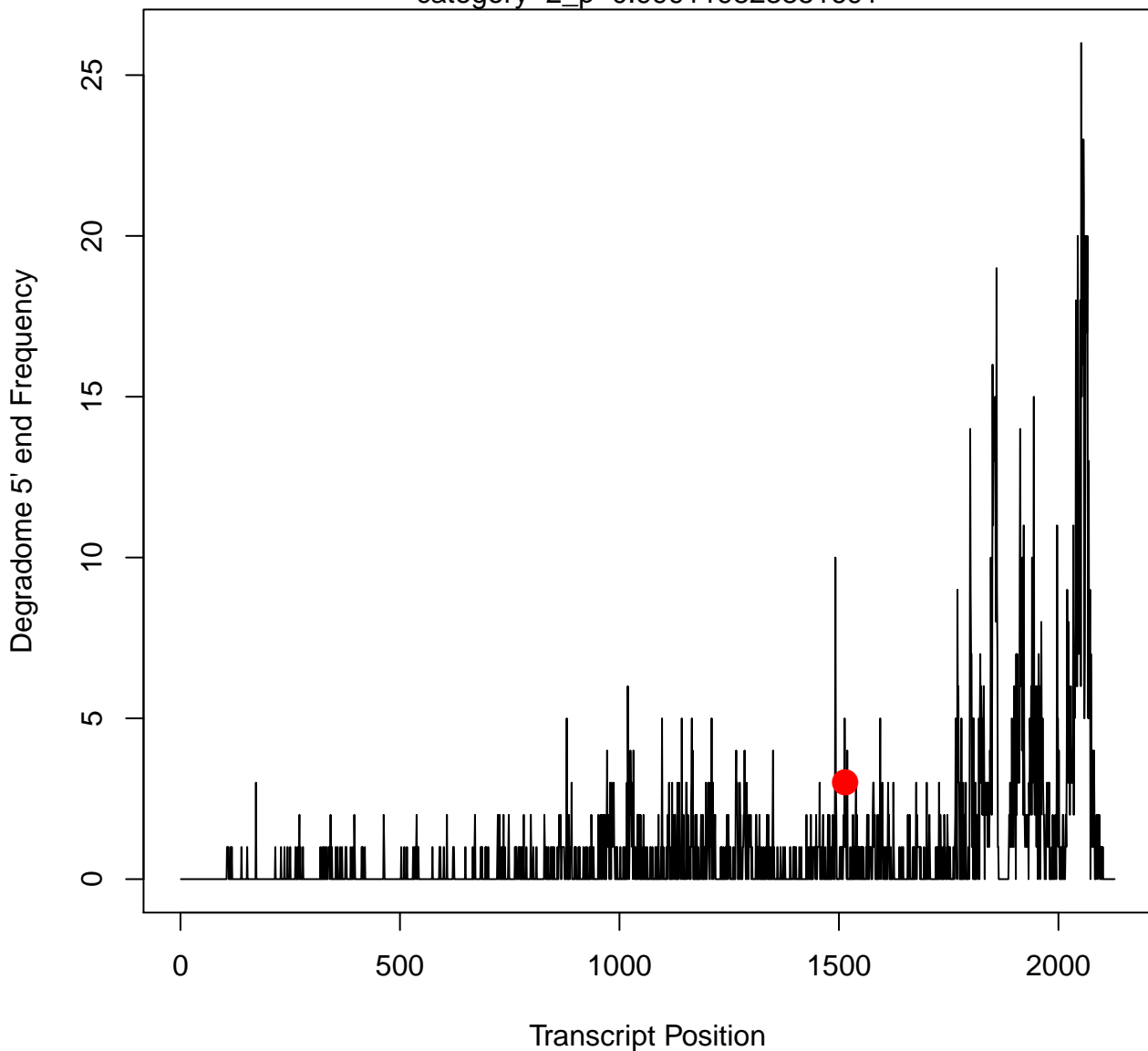

Supplement: Supplementary file 3 [file Data_Sheet_9.ZIP › GSM2230751.plot/Lsa-miR159a_Lsat_1_v5_gn_5_16921.1_1514_TPlot.pdf]

**T=Lsat\_1\_v5\_gn\_8\_123241.1\_Q=Lsa-miR159a\_S=401**

category=2\_p=0.99356807369006

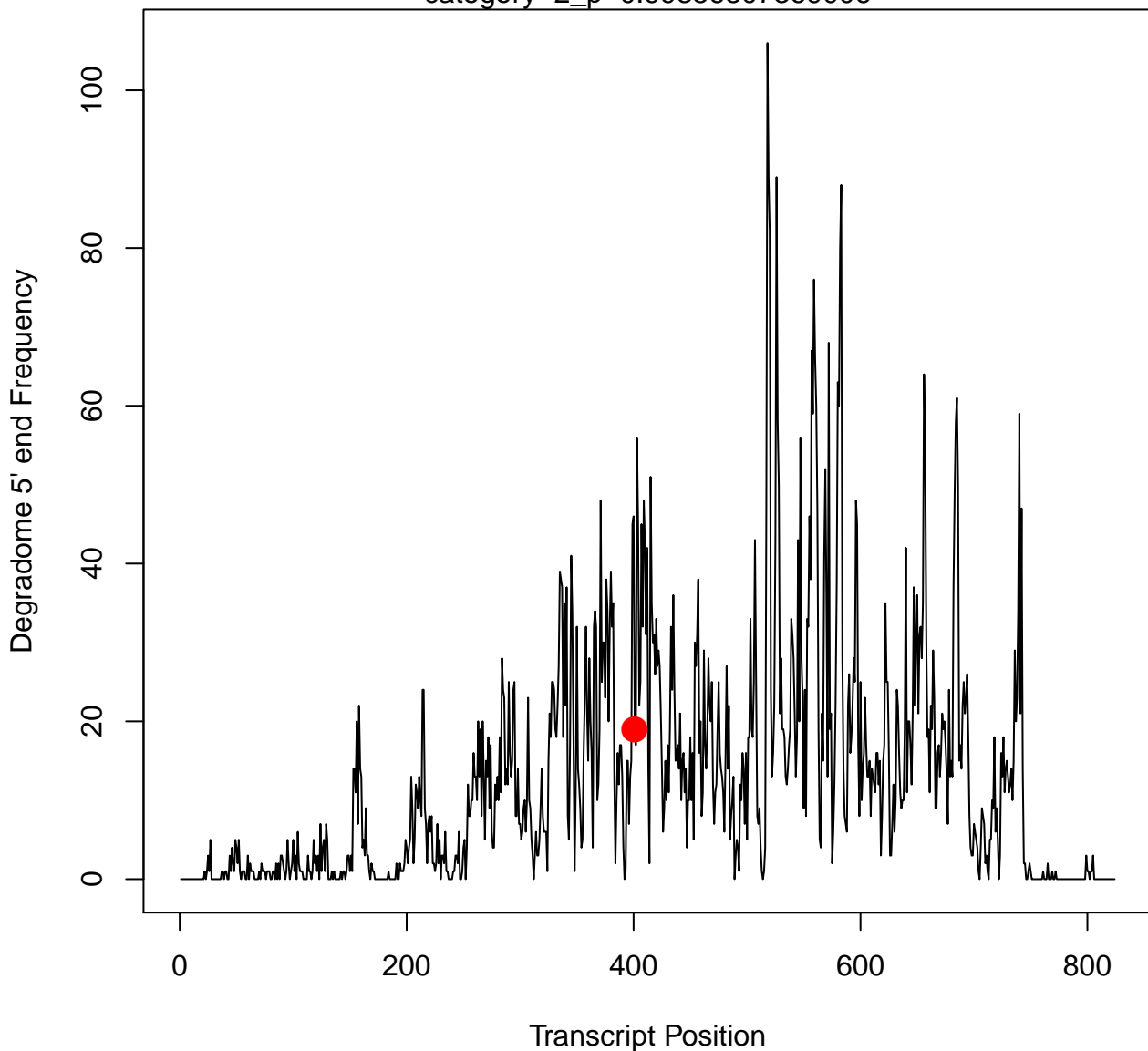

Supplement: Supplementary file 3 [file Data_Sheet_9.ZIP › GSM2230751.plot/Lsa-miR159a_Lsat_1_v5_gn_8_123241.1_401_TPlot.pdf]

**T=Lsat\_1\_v5\_gn\_9\_9761.1\_Q=Lsa-miR159a\_S=337**

category=2\_p=0.998205347908511

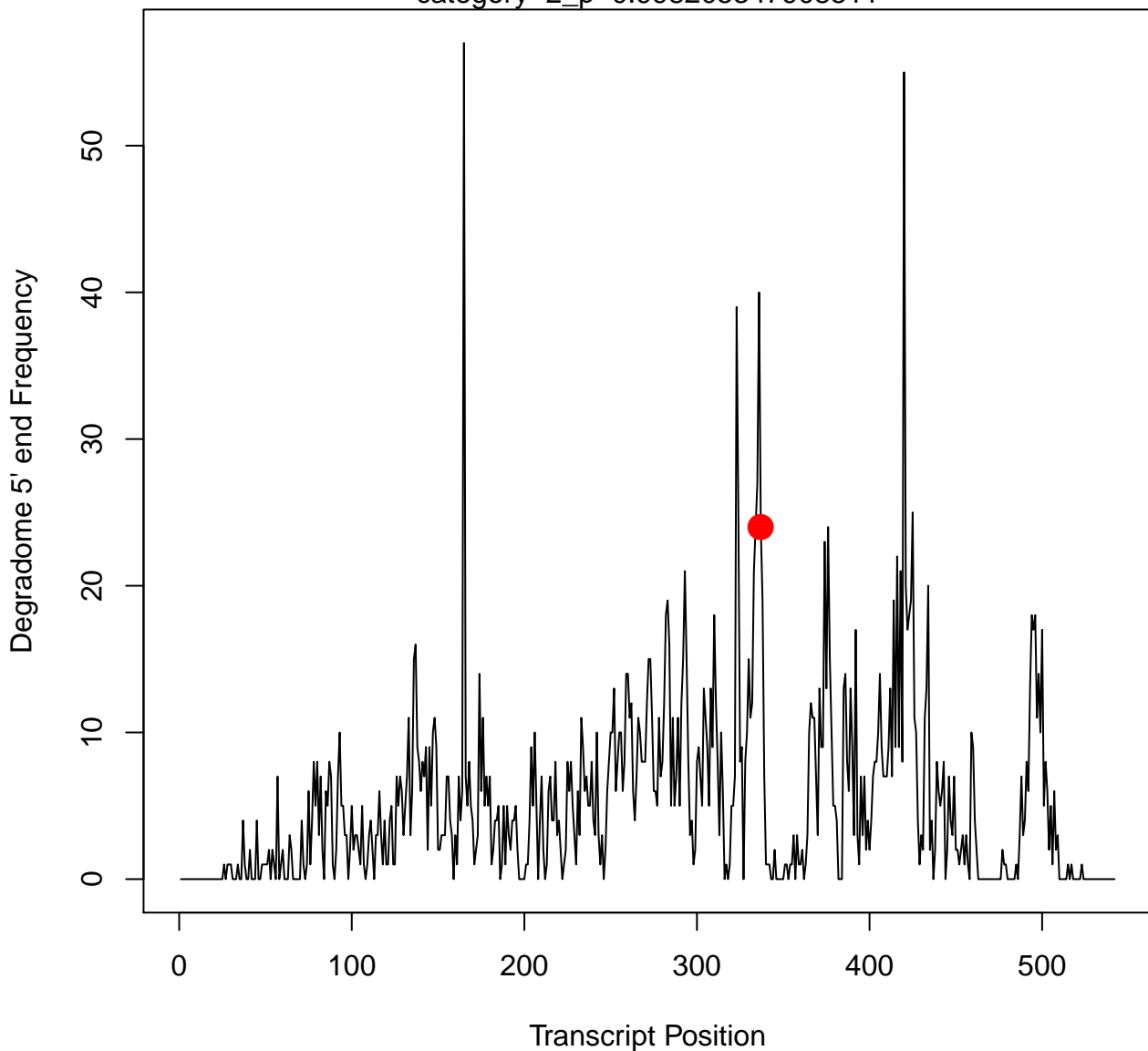

Supplement: Supplementary file 3 [file Data_Sheet_9.ZIP › GSM2230751.plot/Lsa-miR159a_Lsat_1_v5_gn_9_9761.1_337_TPlot.pdf]

**T=Lsat\_1\_v5\_gn\_0\_39281.1\_Q=Lsa-miR159b\_S=2161**

category=0\_p=0.000369544041020964

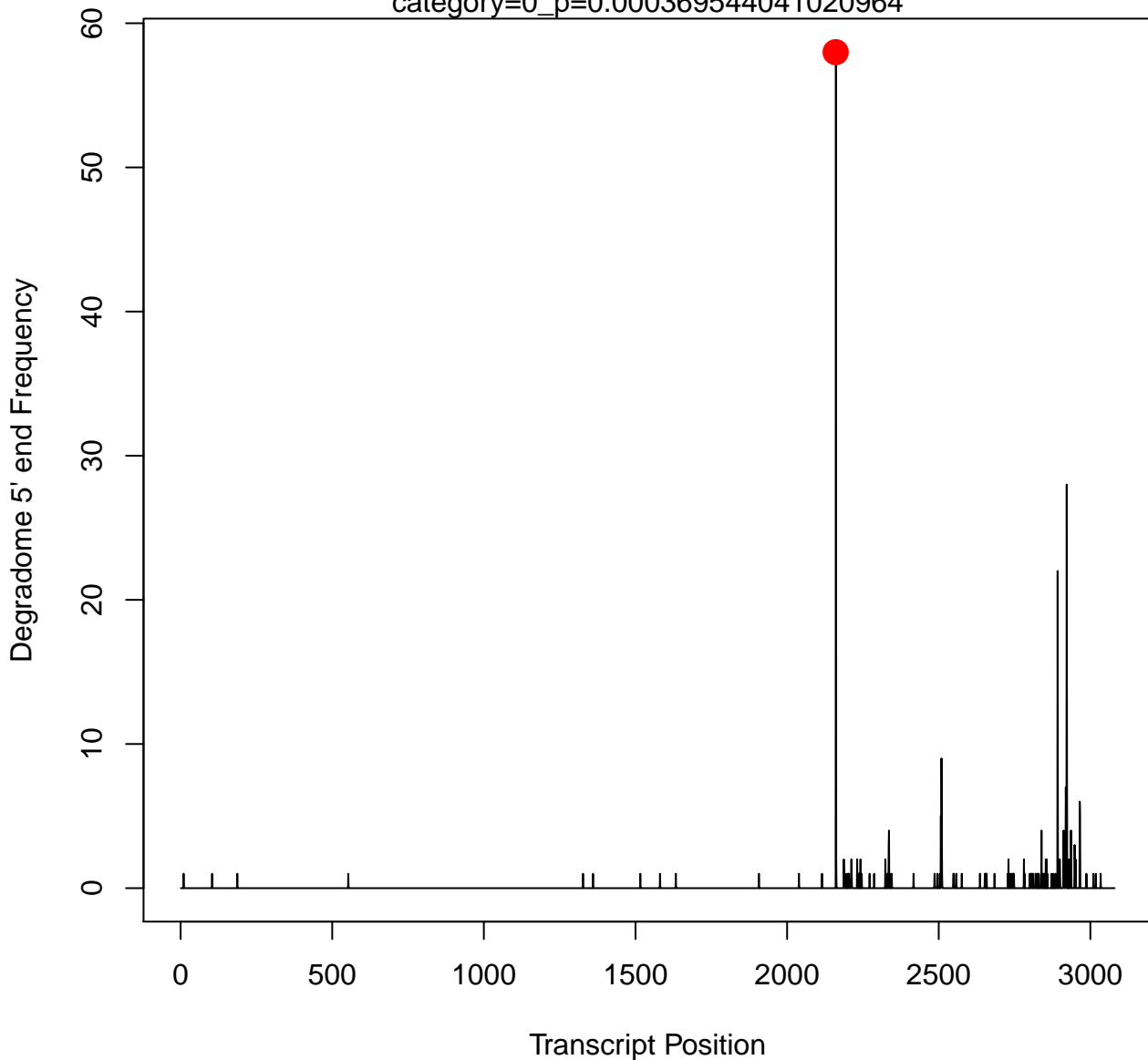

Supplement: Supplementary file 3 [file Data_Sheet_9.ZIP › GSM2230751.plot/Lsa-miR159b_Lsat_1_v5_gn_0_39281.1_2161_TPlot.pdf]

**T=Lsat\_1\_v5\_gn\_1\_64661.1\_Q=Lsa-miR159b\_S=652**

category=2\_p=0.995220087506694

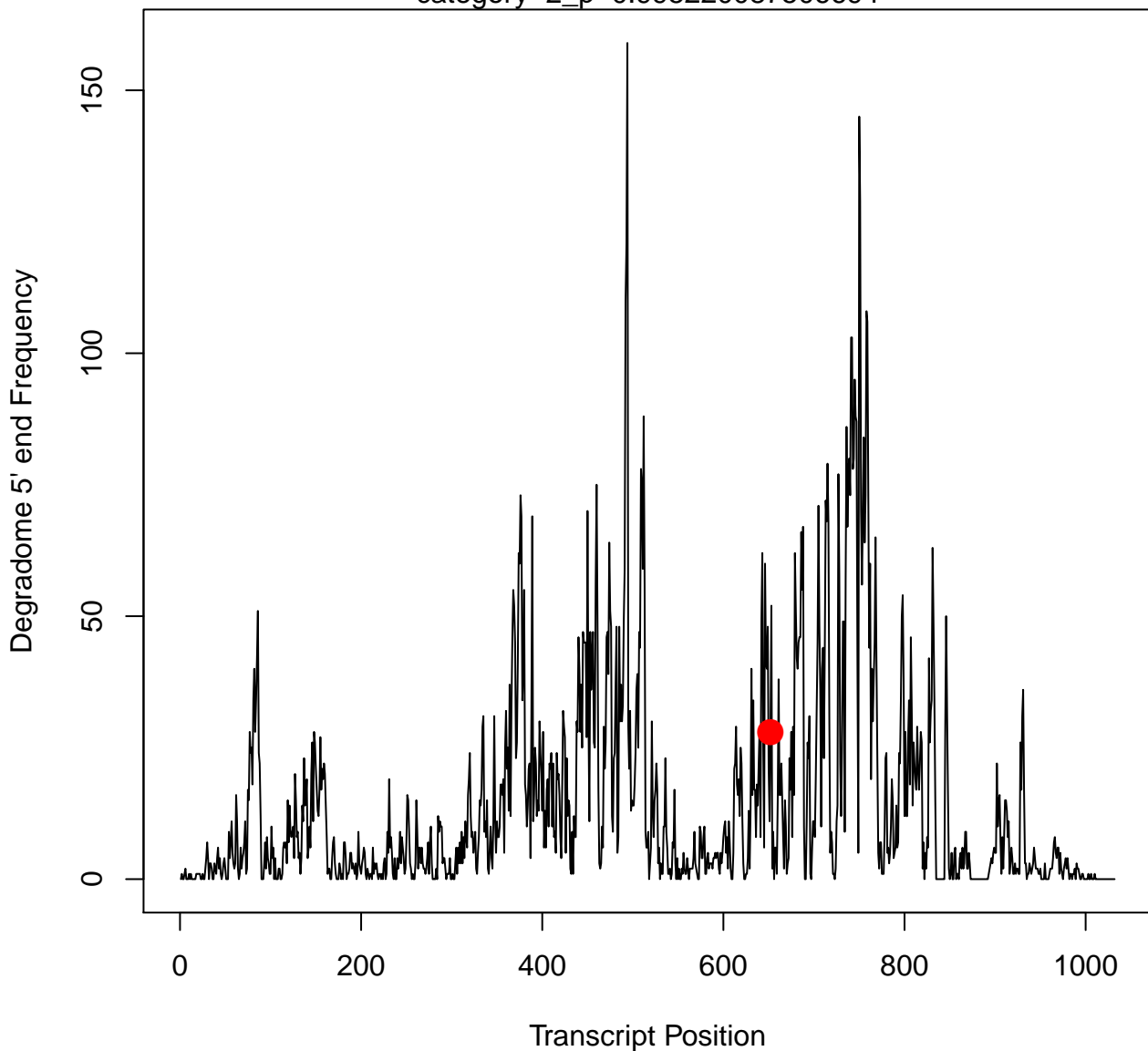

Supplement: Supplementary file 3 [file Data_Sheet_9.ZIP › GSM2230751.plot/Lsa-miR159b_Lsat_1_v5_gn_1_64661.1_652_TPlot.pdf]

**T=Lsat\_1\_v5\_gn\_3\_90141.1\_Q=Lsa-miR159b\_S=540**

category=2\_p=0.951582246389104

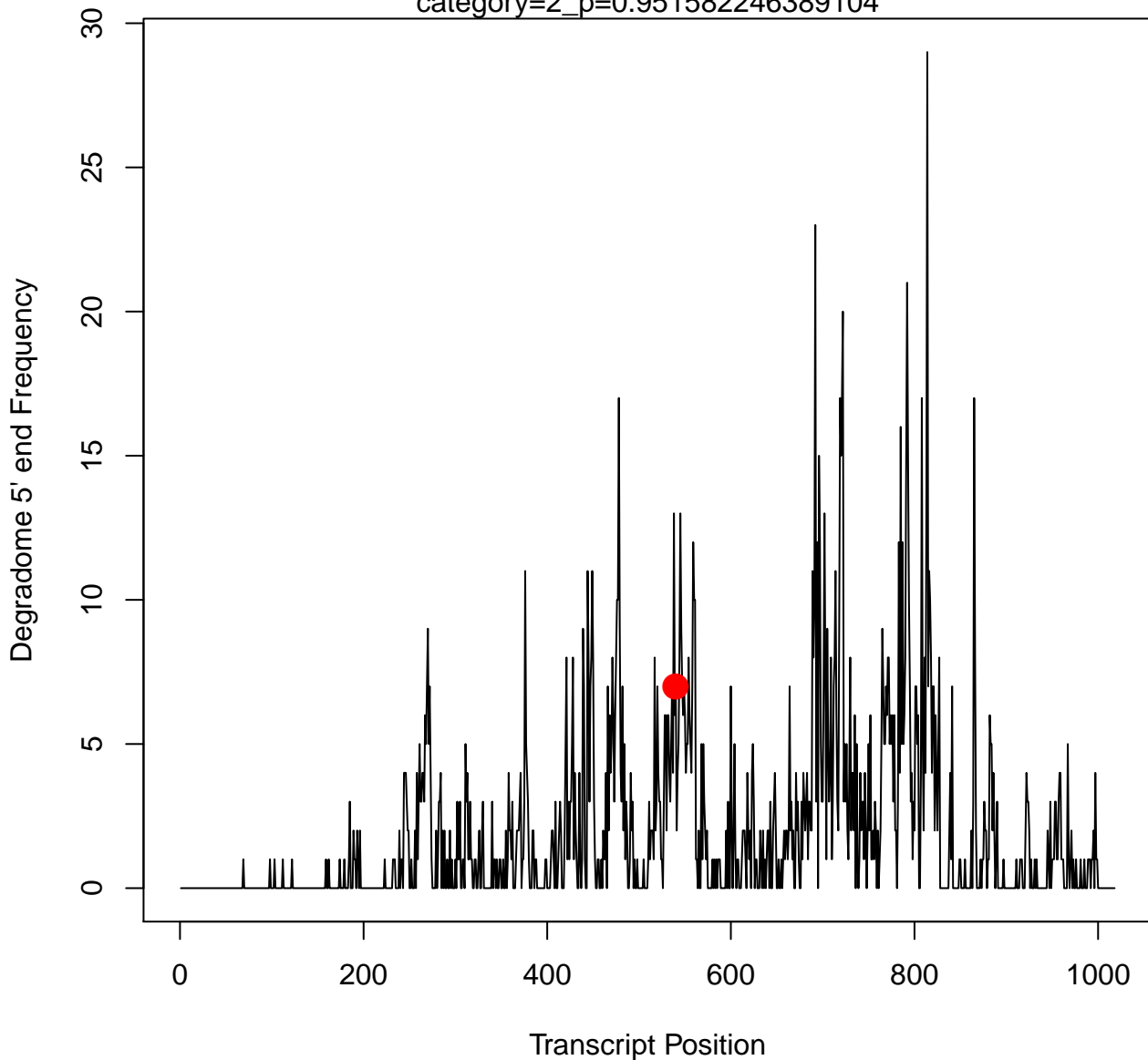

Supplement: Supplementary file 3 [file Data_Sheet_9.ZIP › GSM2230751.plot/Lsa-miR159b_Lsat_1_v5_gn_3_90141.1_540_TPlot.pdf]

**T=Lsat\_1\_v5\_gn\_3\_94000.1\_Q=Lsa-miR159b\_S=137**

category=2\_p=0.396282025902823

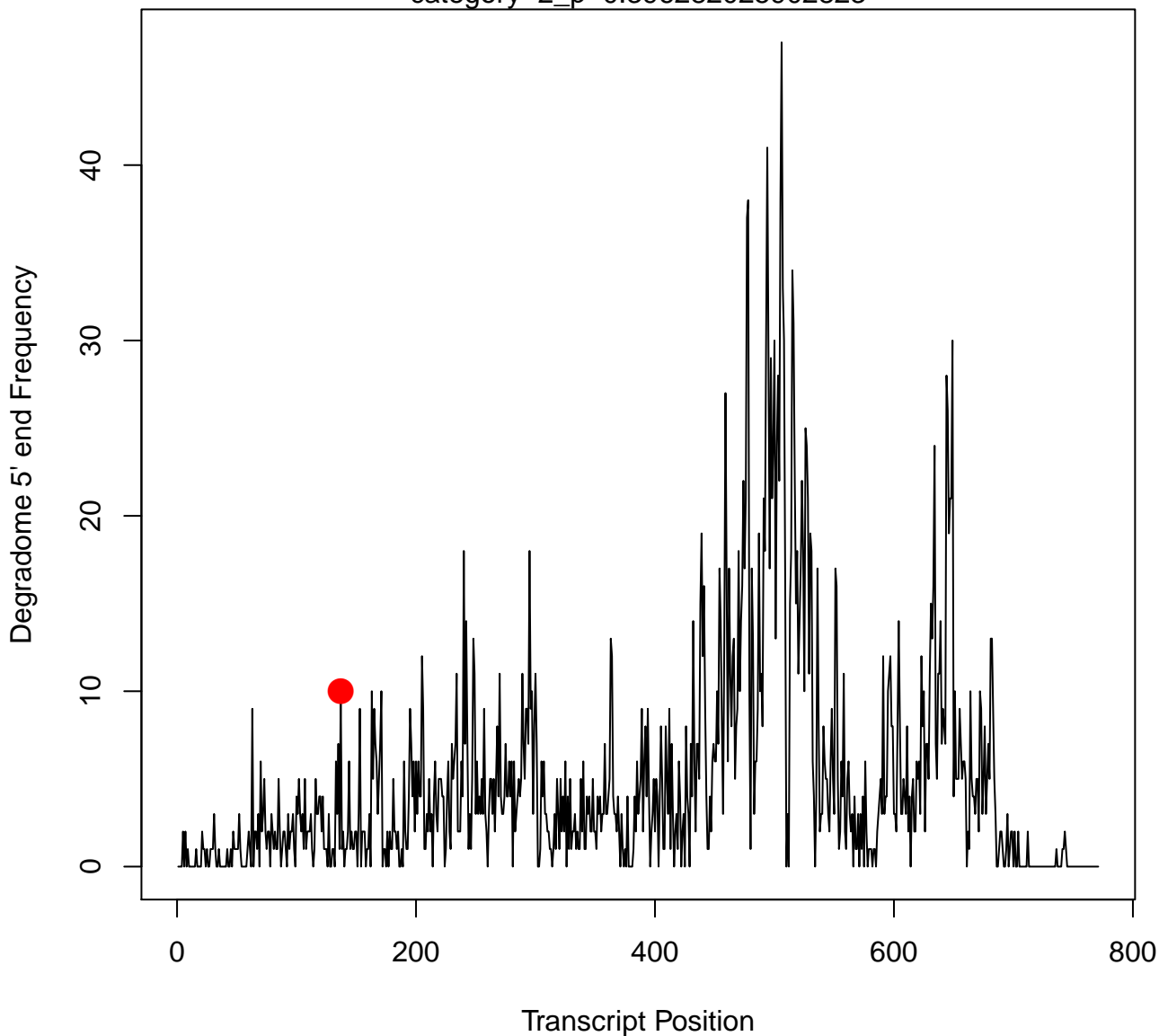

Supplement: Supplementary file 3 [file Data_Sheet_9.ZIP › GSM2230751.plot/Lsa-miR159b_Lsat_1_v5_gn_3_94000.1_137_TPlot.pdf]

**T=Lsat\_1\_v5\_gn\_5\_179060.1\_Q=Lsa-miR159b\_S=714**

category=2\_p=0.163150556750859

Degradsome 5' end Frequency

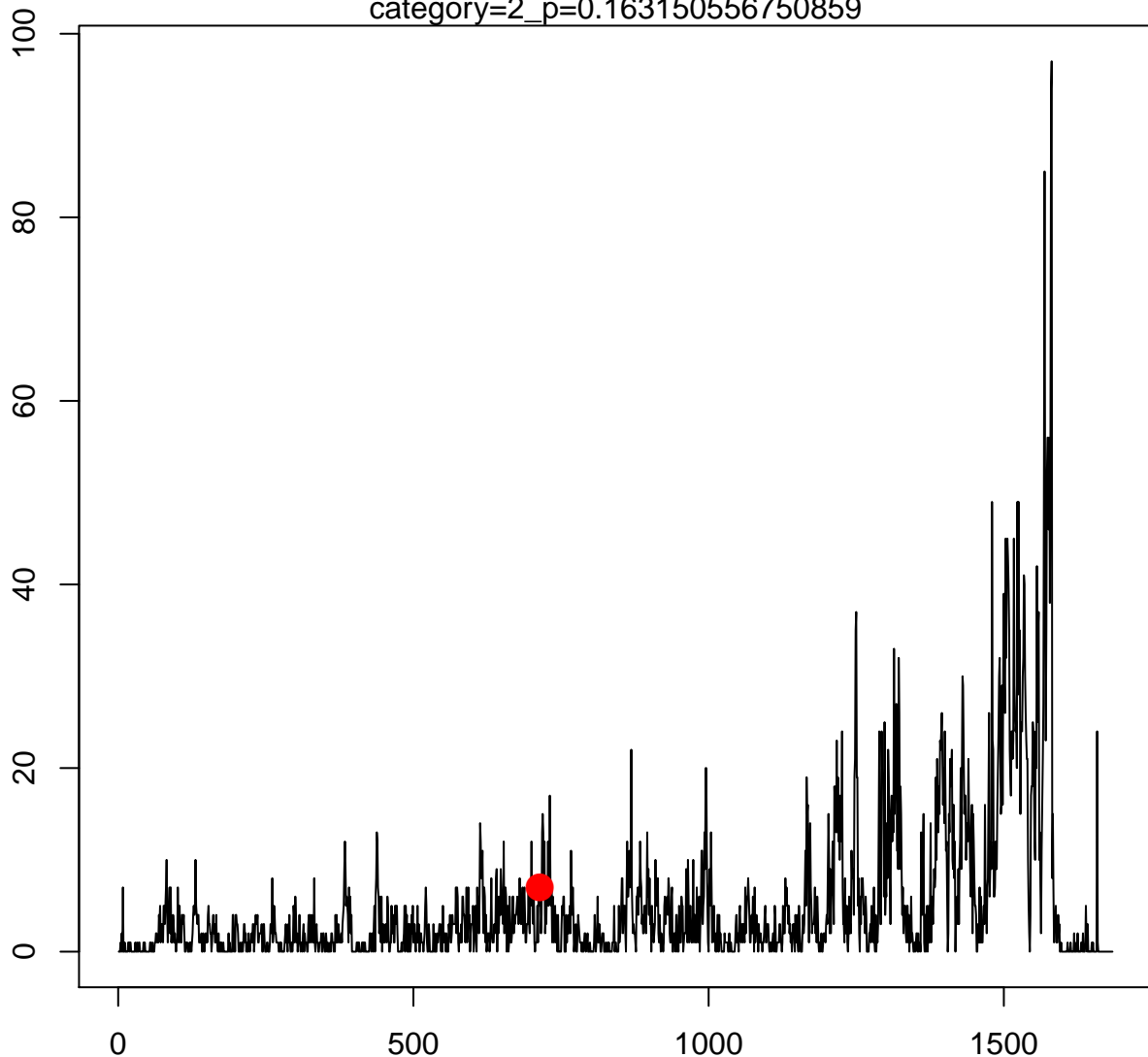

Transcript Position

Supplement: Supplementary file 3 [file Data_Sheet_9.ZIP › GSM2230751.plot/Lsa-miR159b_Lsat_1_v5_gn_5_179060.1_714_TPlot.pdf]

**T=Lsat\_1\_v5\_gn\_5\_75800.1\_Q=Lsa-miR159b\_S=1860**

category=2\_p=0.0576423137718276

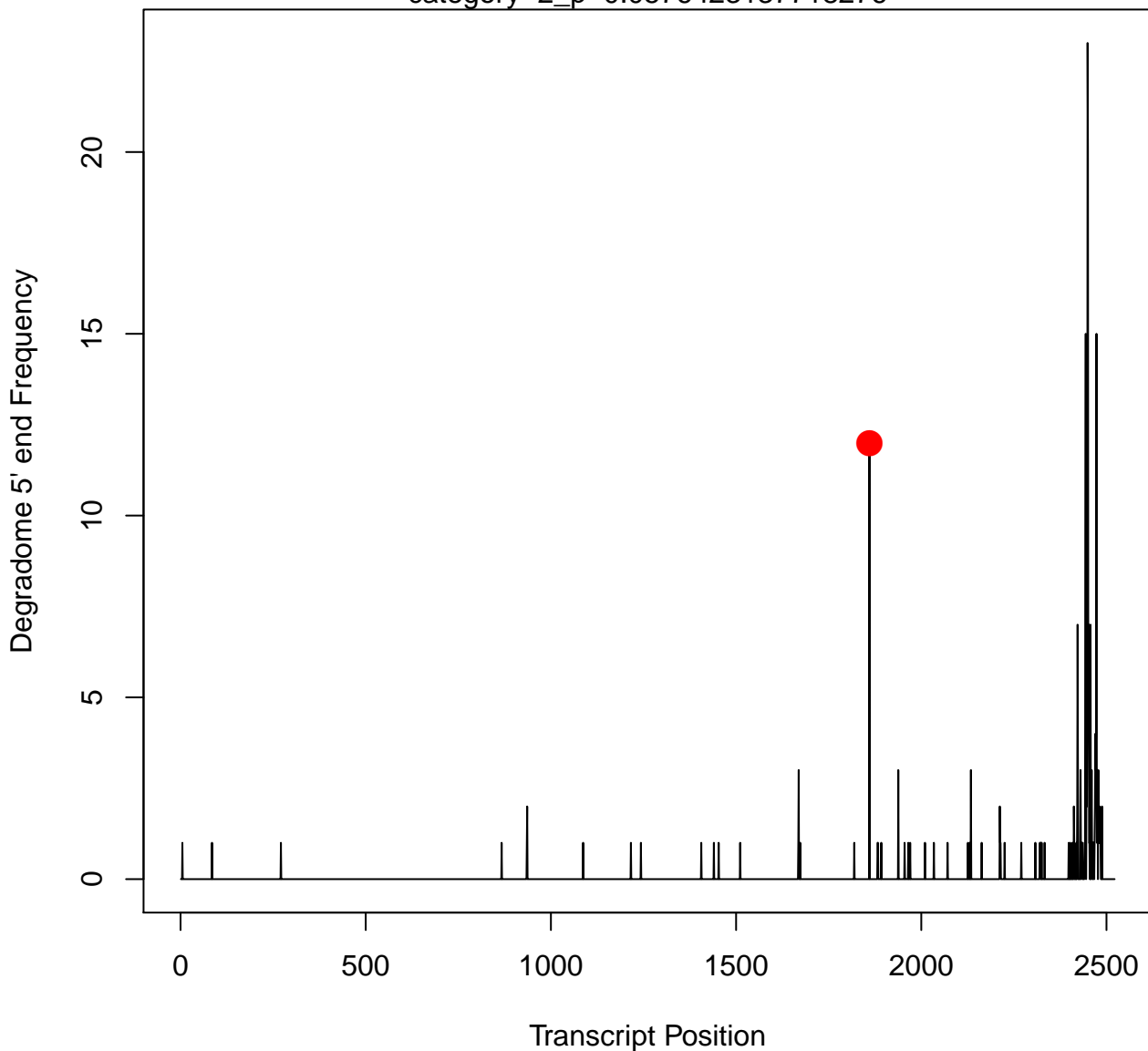

Supplement: Supplementary file 3 [file Data_Sheet_9.ZIP › GSM2230751.plot/Lsa-miR159b_Lsat_1_v5_gn_5_75800.1_1860_TPlot.pdf]

**T=Lsat\_1\_v5\_gn\_8\_18560.1\_Q=Lsa-miR159b\_S=254**

category=2\_p=0.766500184980761

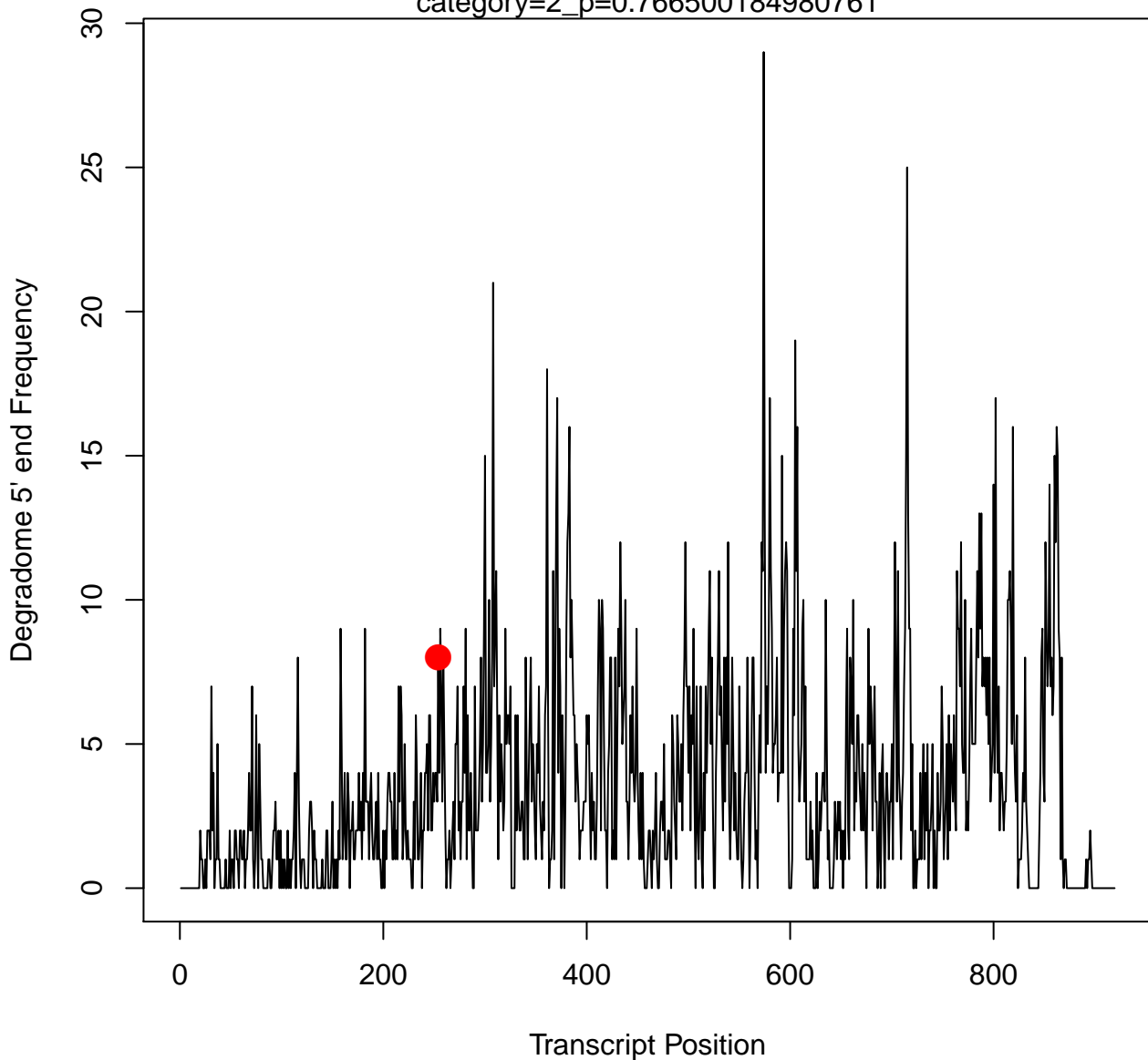

Supplement: Supplementary file 3 [file Data_Sheet_9.ZIP › GSM2230751.plot/Lsa-miR159b_Lsat_1_v5_gn_8_18560.1_254_TPlot.pdf]

**T=Lsat\_1\_v5\_gn\_2\_100181.1\_Q=Lsa-miR160a\_S=2076**

category=2\_p=0.0852052452876977

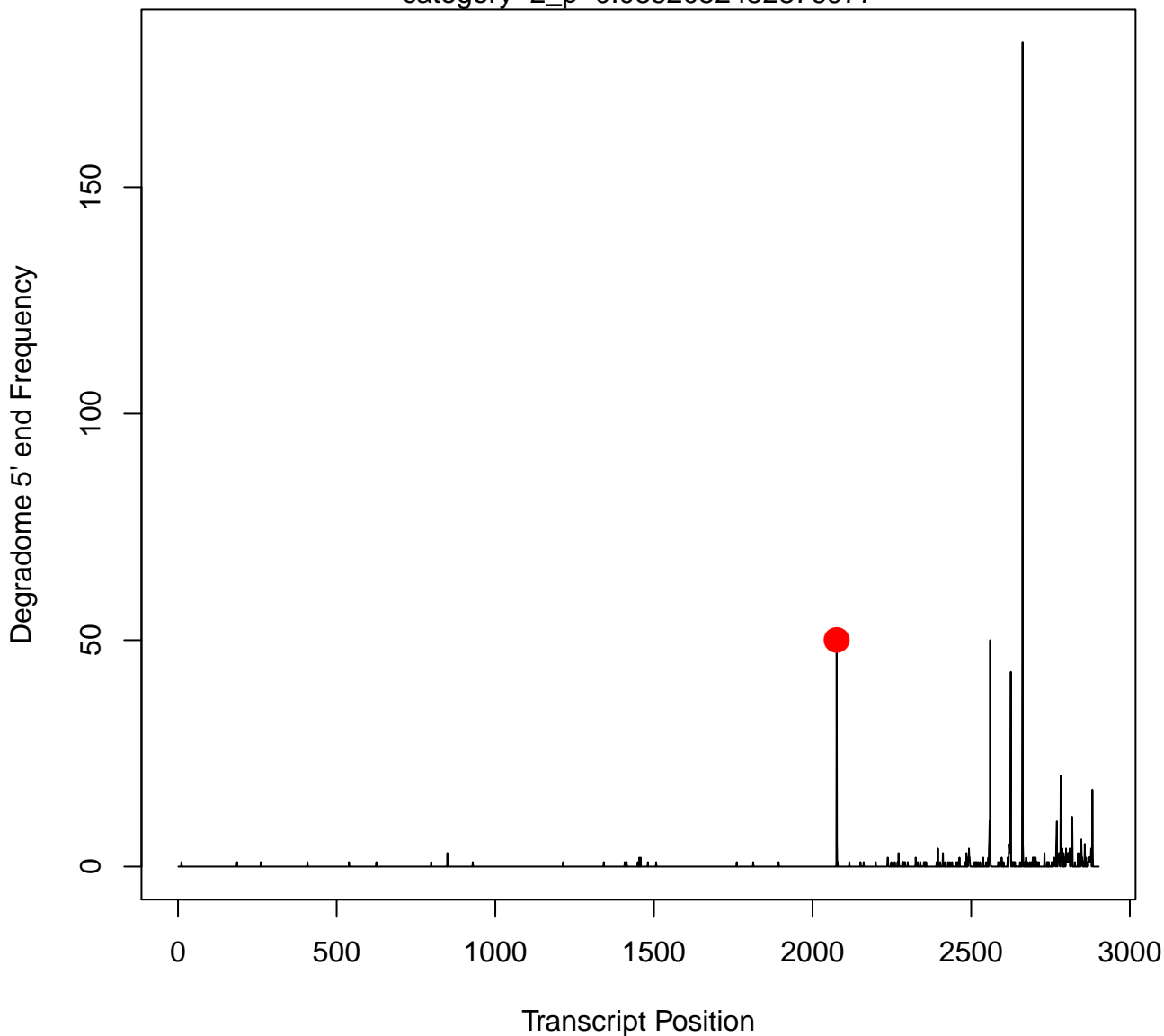

Supplement: Supplementary file 3 [file Data_Sheet_9.ZIP › GSM2230751.plot/Lsa-miR160a_Lsat_1_v5_gn_2_100181.1_2076_TPlot.pdf]

**T=Lsat\_1\_v5\_gn\_5\_78820.1\_Q=Lsa-miR160a\_S=2693**

category=2\_p=0.940399566861131

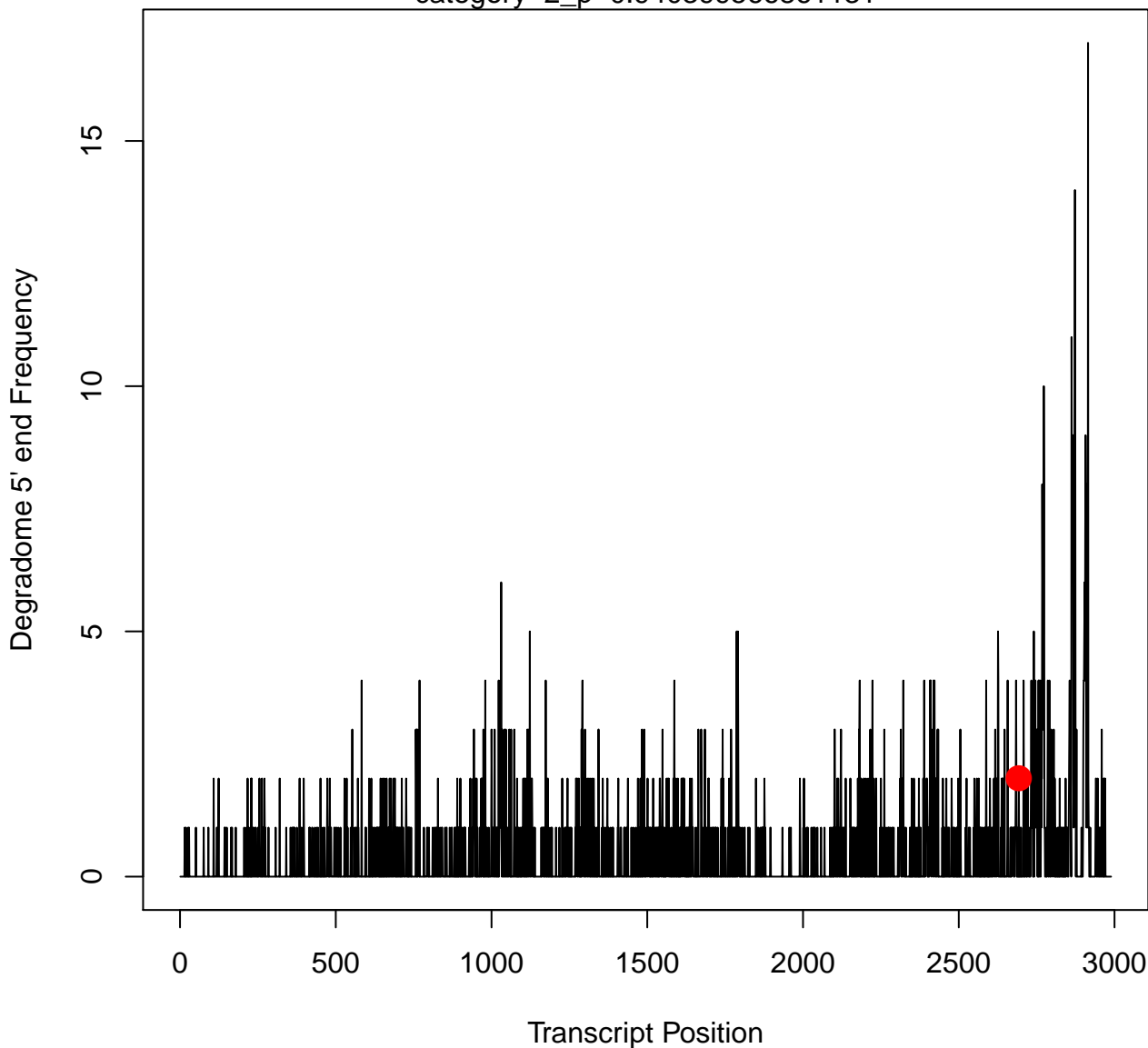

Supplement: Supplementary file 3 [file Data_Sheet_9.ZIP › GSM2230751.plot/Lsa-miR160a_Lsat_1_v5_gn_5_78820.1_2693_TPlot.pdf]

**T=Lsat\_1\_v5\_gn\_2\_112381.1\_Q=Lsa-miR160b\_S=1289**

category=0\_p=0.0011082224851342

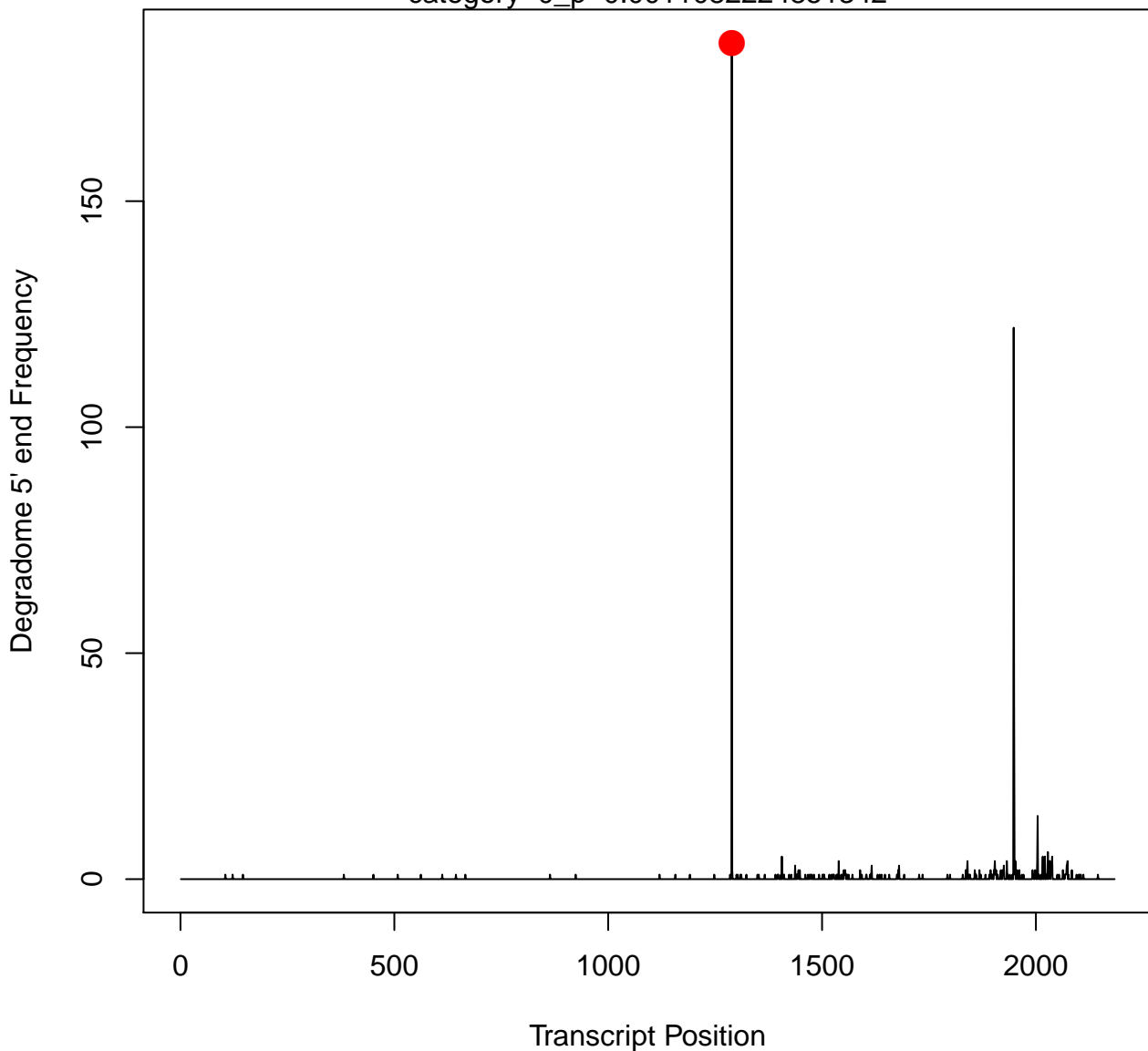

Supplement: Supplementary file 3 [file Data_Sheet_9.ZIP › GSM2230751.plot/Lsa-miR160b_Lsat_1_v5_gn_2_112381.1_1289_TPlot.pdf]

**T=Lsat\_1\_v5\_gn\_6\_30741.1\_Q=Lsa-miR160b\_S=2067**

category=0\_p=0.000369544041020964

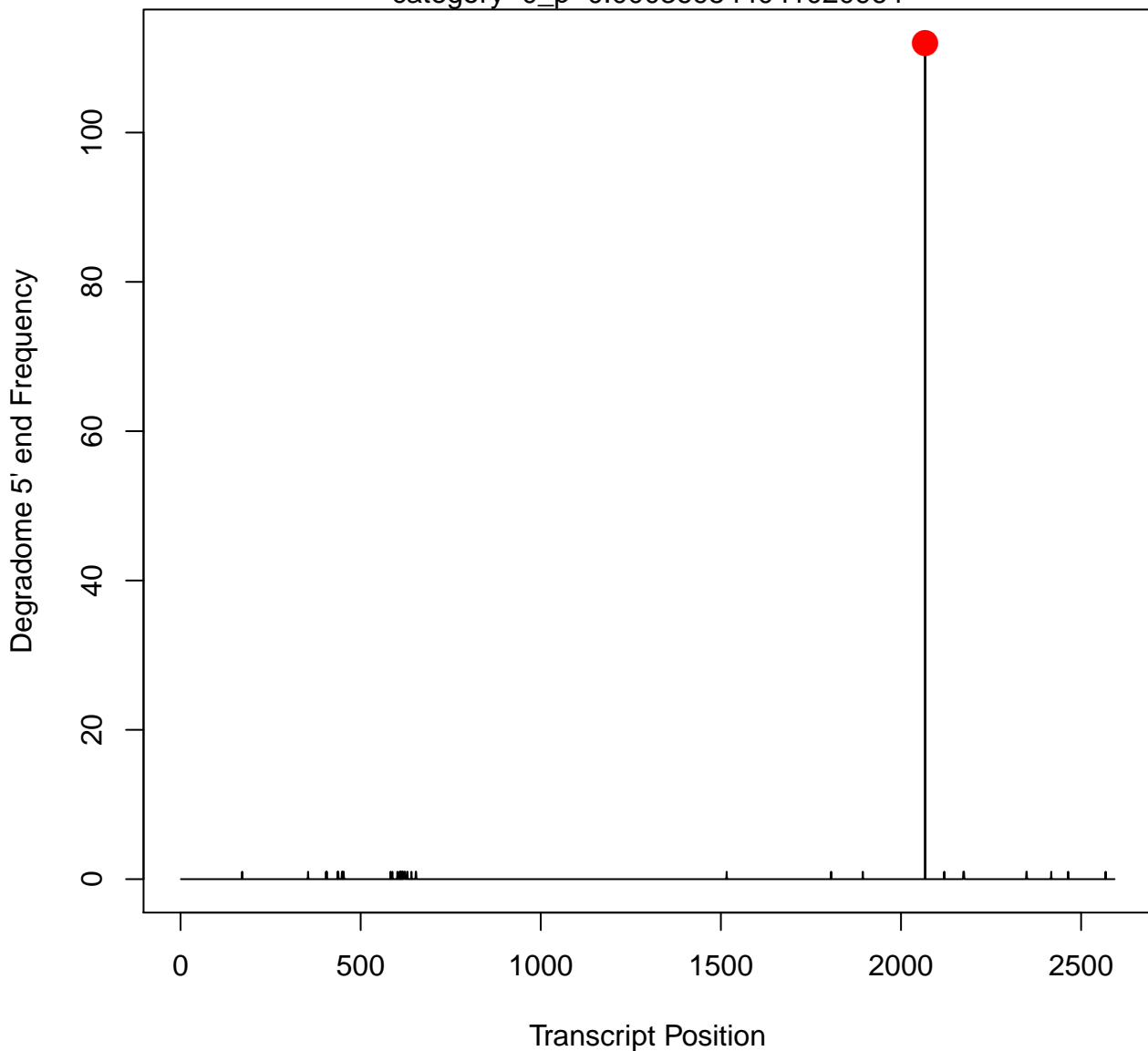

Supplement: Supplementary file 3 [file Data_Sheet_9.ZIP › GSM2230751.plot/Lsa-miR160b_Lsat_1_v5_gn_6_30741.1_2067_TPlot.pdf]

**T=Lsat\_1\_v5\_gn\_7\_80140.1\_Q=Lsa-miR160b\_S=1719**

category=2\_p=0.836476112224566

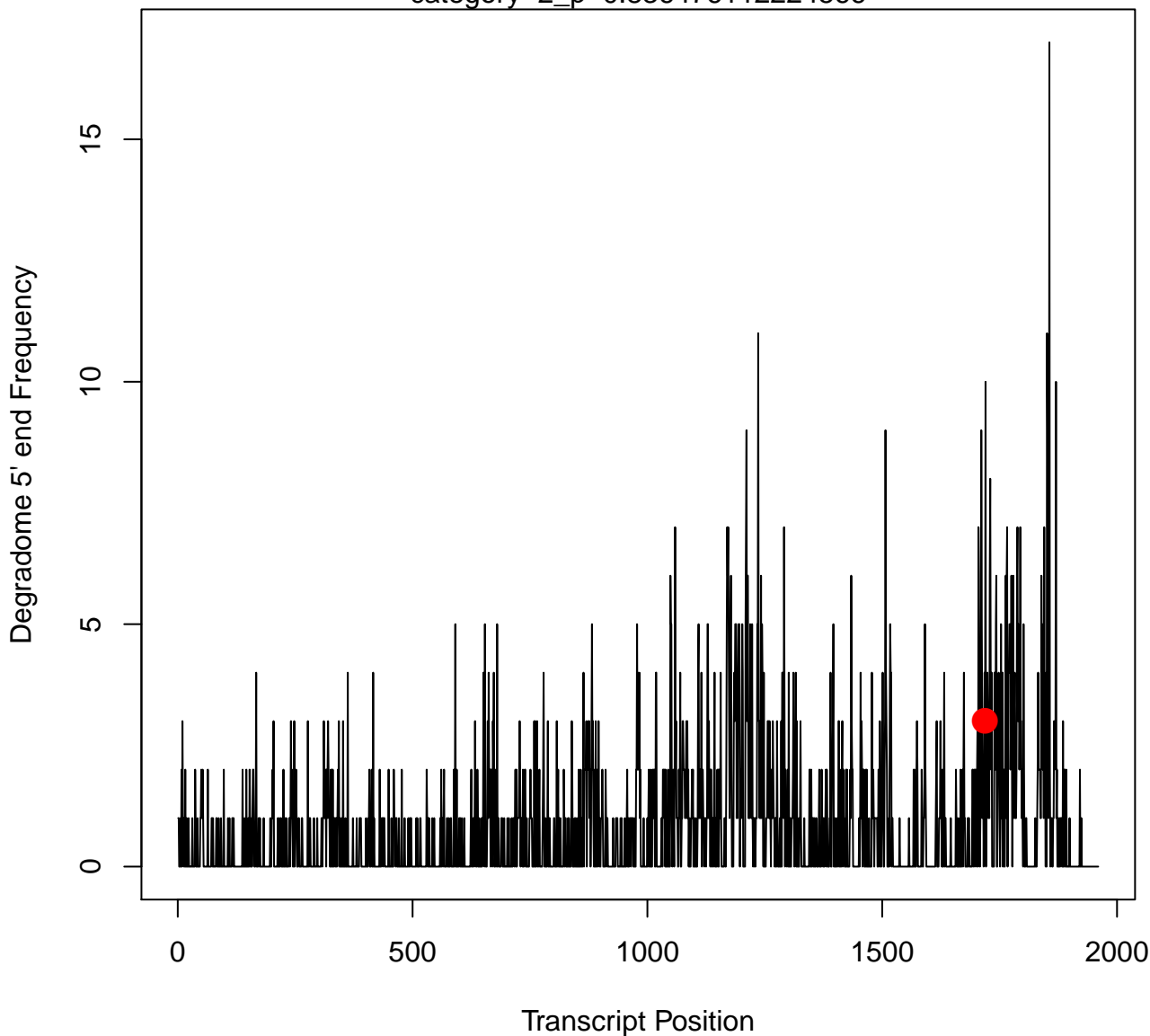

Supplement: Supplementary file 3 [file Data_Sheet_9.ZIP › GSM2230751.plot/Lsa-miR160b_Lsat_1_v5_gn_7_80140.1_1719_TPlot.pdf]

**T=Lsat\_1\_v5\_gn\_8\_20161.1\_Q=Lsa-miR160b\_S=1819**

category=0\_p=0.00147735698913964

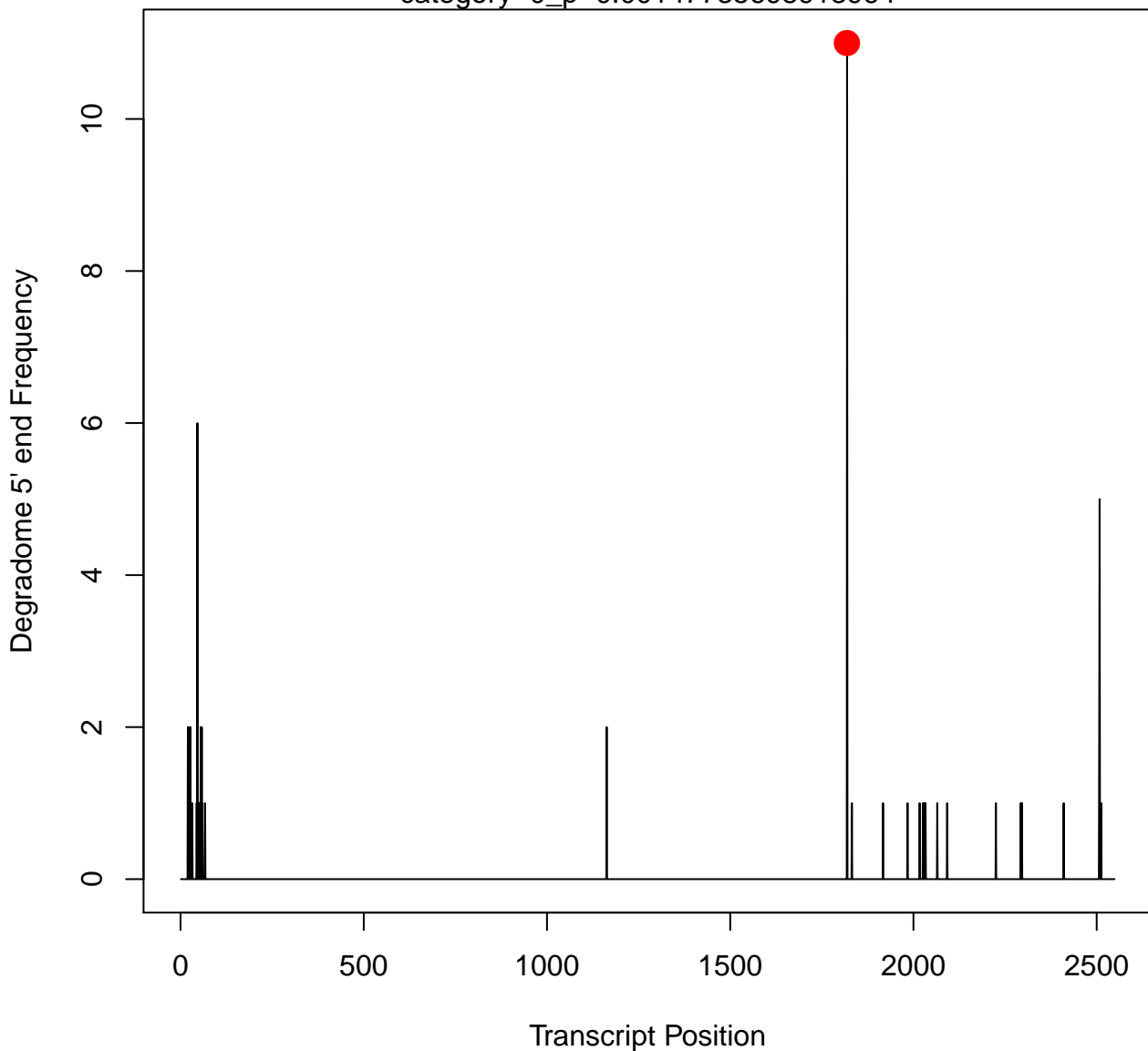

Supplement: Supplementary file 3 [file Data_Sheet_9.ZIP › GSM2230751.plot/Lsa-miR160b_Lsat_1_v5_gn_8_20161.1_1819_TPlot.pdf]

**T=Lsat\_1\_v5\_gn\_9\_69060.1\_Q=Lsa-miR160b\_S=1181**

category=0\_p=0.000738951519243747

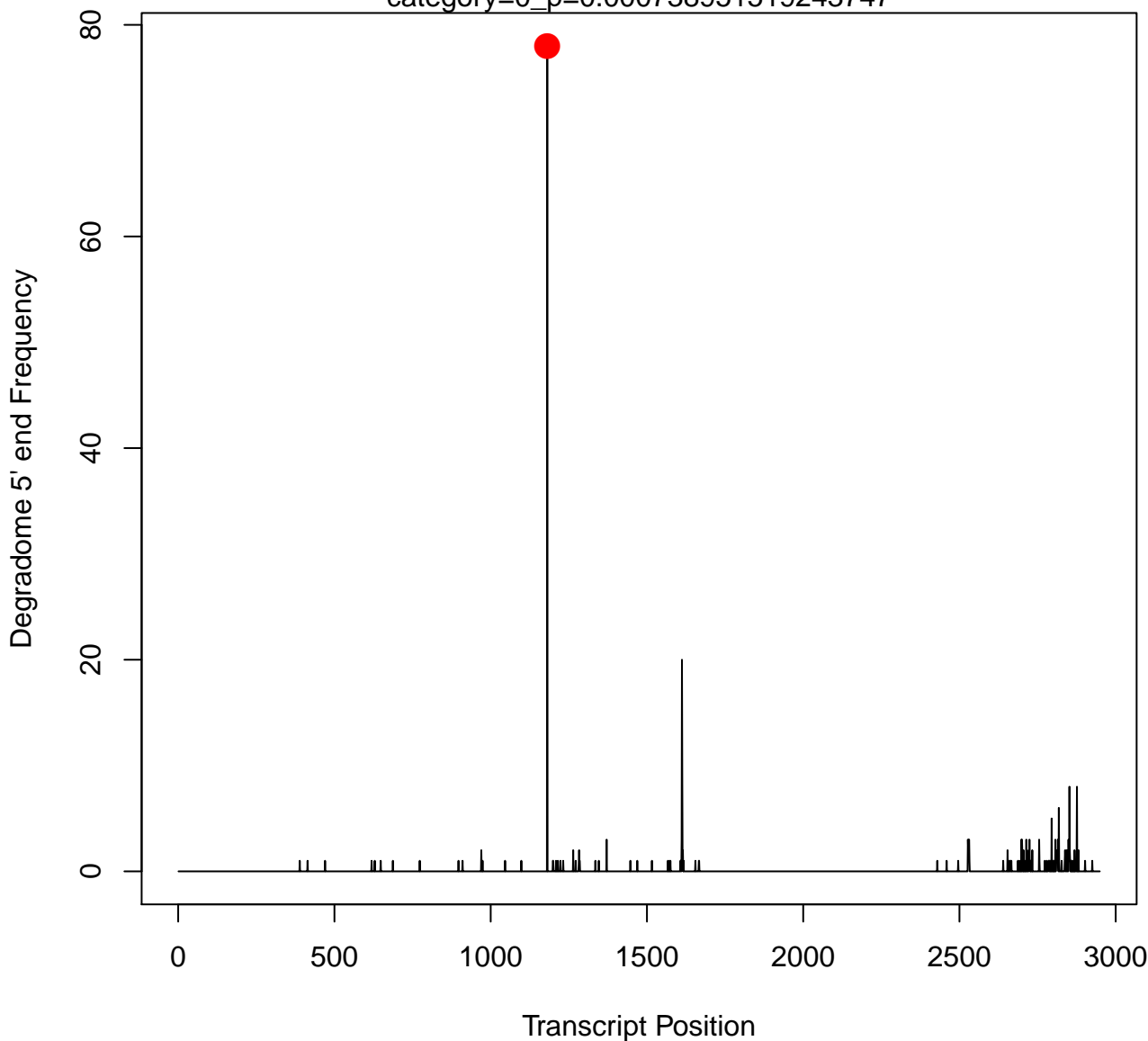

Supplement: Supplementary file 3 [file Data_Sheet_9.ZIP › GSM2230751.plot/Lsa-miR160b_Lsat_1_v5_gn_9_69060.1_1181_TPlot.pdf]

**T=Lsat\_1\_v5\_gn\_1\_110320.1\_Q=Lsa-miR164a\_S=3524**

category=2\_p=0.234454518831775

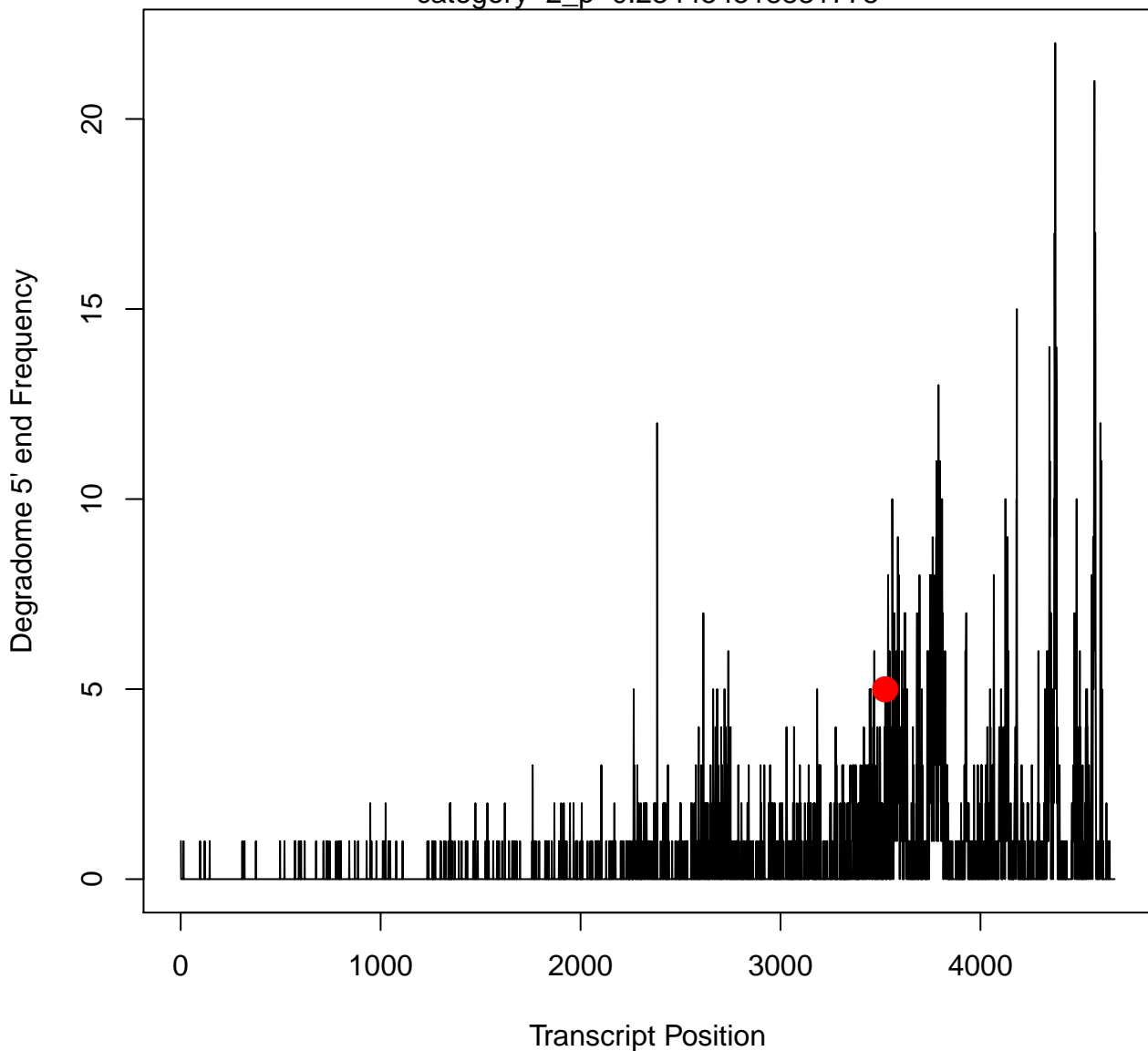

Supplement: Supplementary file 3 [file Data_Sheet_9.ZIP › GSM2230751.plot/Lsa-miR164a_Lsat_1_v5_gn_1_110320.1_3524_TPlot.pdf]

**T=Lsat\_1\_v5\_gn\_4\_109600.1\_Q=Lsa-miR164a\_S=877**

category=0\_p=0.0011082224851342

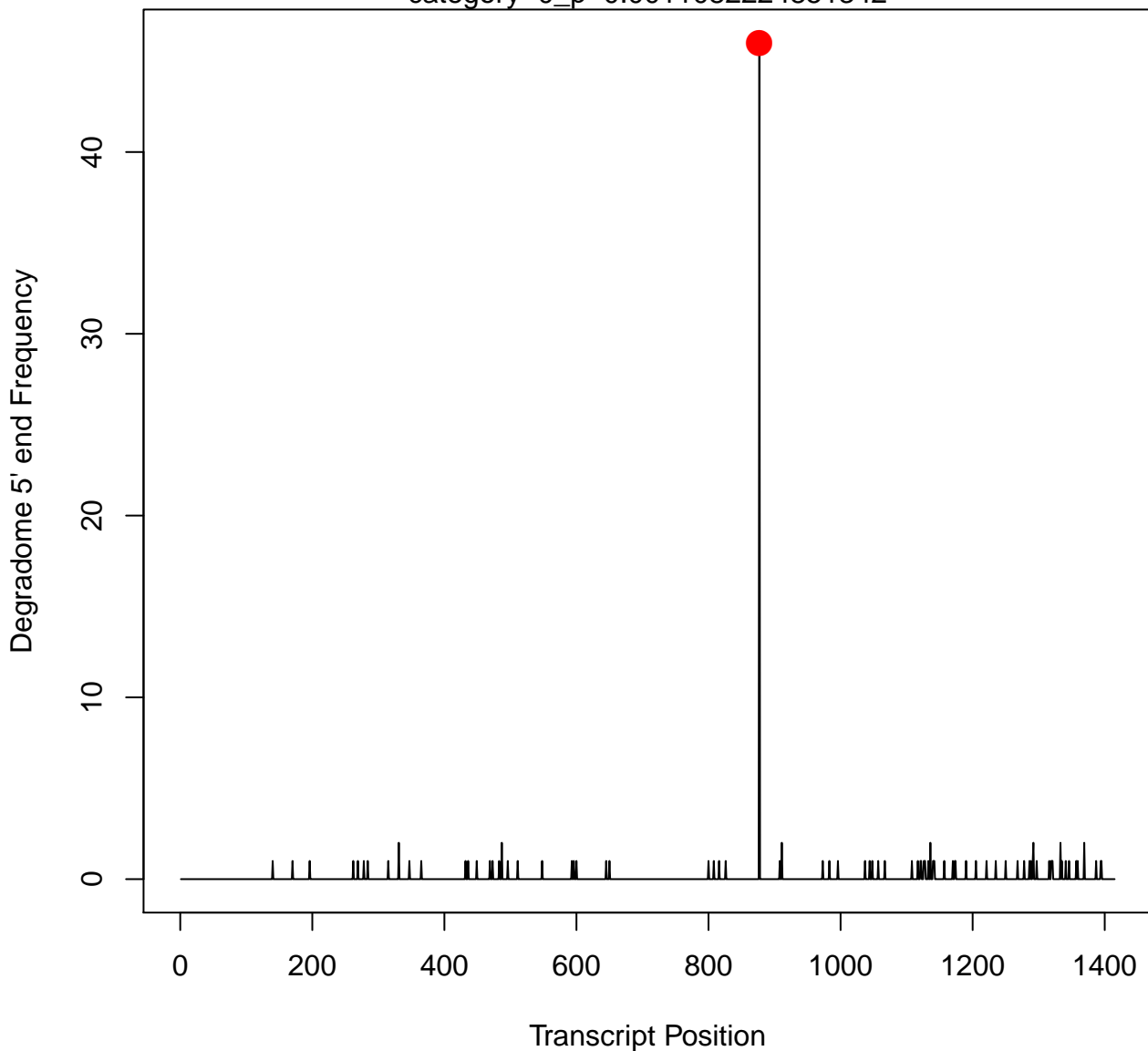

Supplement: Supplementary file 3 [file Data_Sheet_9.ZIP › GSM2230751.plot/Lsa-miR164a_Lsat_1_v5_gn_4_109600.1_877_TPlot.pdf]

**T=Lsat\_1\_v5\_gn\_4\_110501.1\_Q=Lsa-miR164a\_S=406**

category=2\_p=0.624542898222074

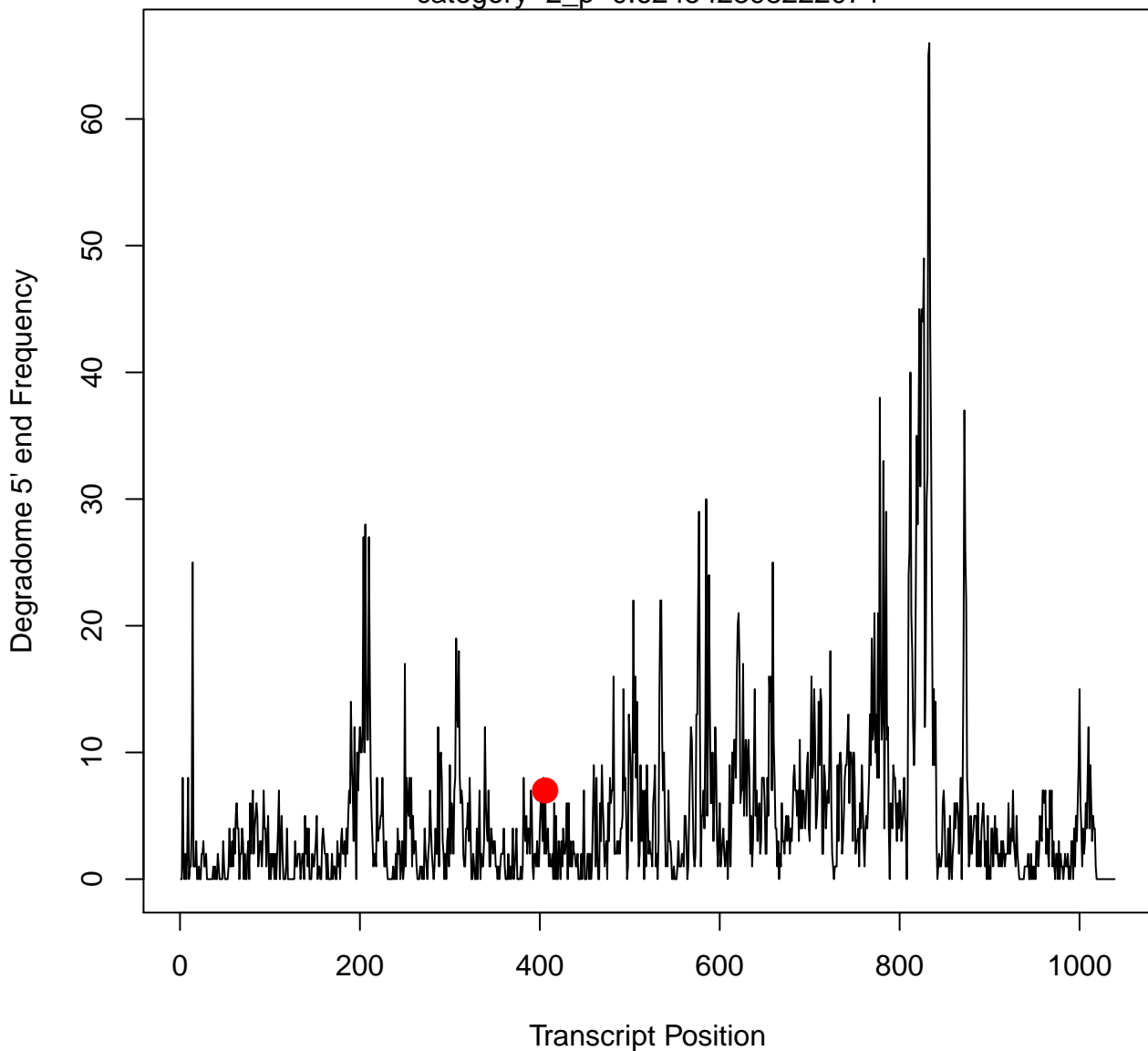

Supplement: Supplementary file 3 [file Data_Sheet_9.ZIP › GSM2230751.plot/Lsa-miR164a_Lsat_1_v5_gn_4_110501.1_406_TPlot.pdf]

T=Lsat\_1\_v5\_gn\_5\_127660.1\_Q=Lsa-miR164a\_S=724

category=0\_p=0.000738951519243747

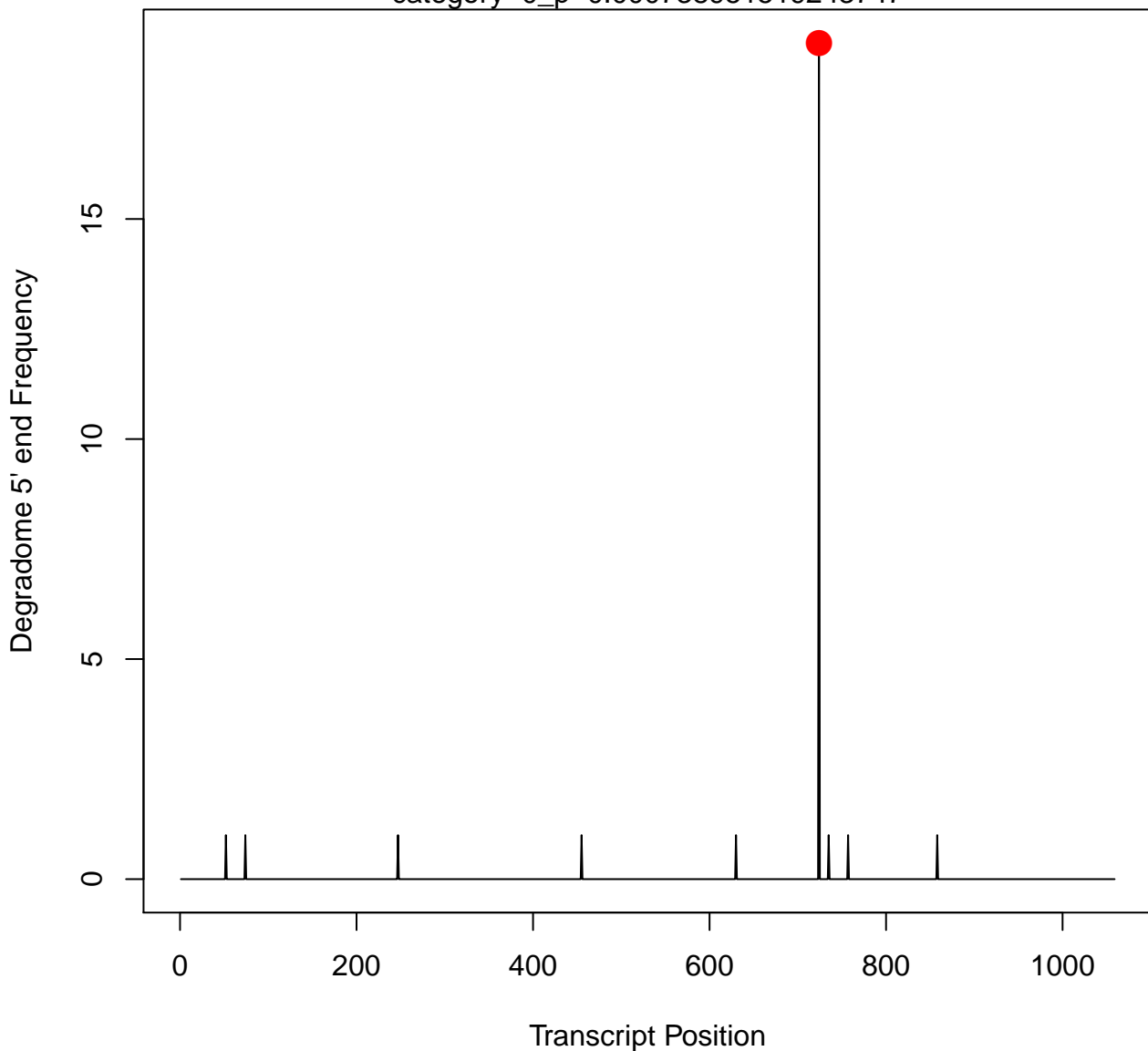

Supplement: Supplementary file 3 [file Data_Sheet_9.ZIP › GSM2230751.plot/Lsa-miR164a_Lsat_1_v5_gn_5_127660.1_724_TPlot.pdf]

**T=Lsat\_1\_v5\_gn\_5\_981.1\_Q=Lsa-miR164a\_S=601**

category=0\_p=0.00147735698913964

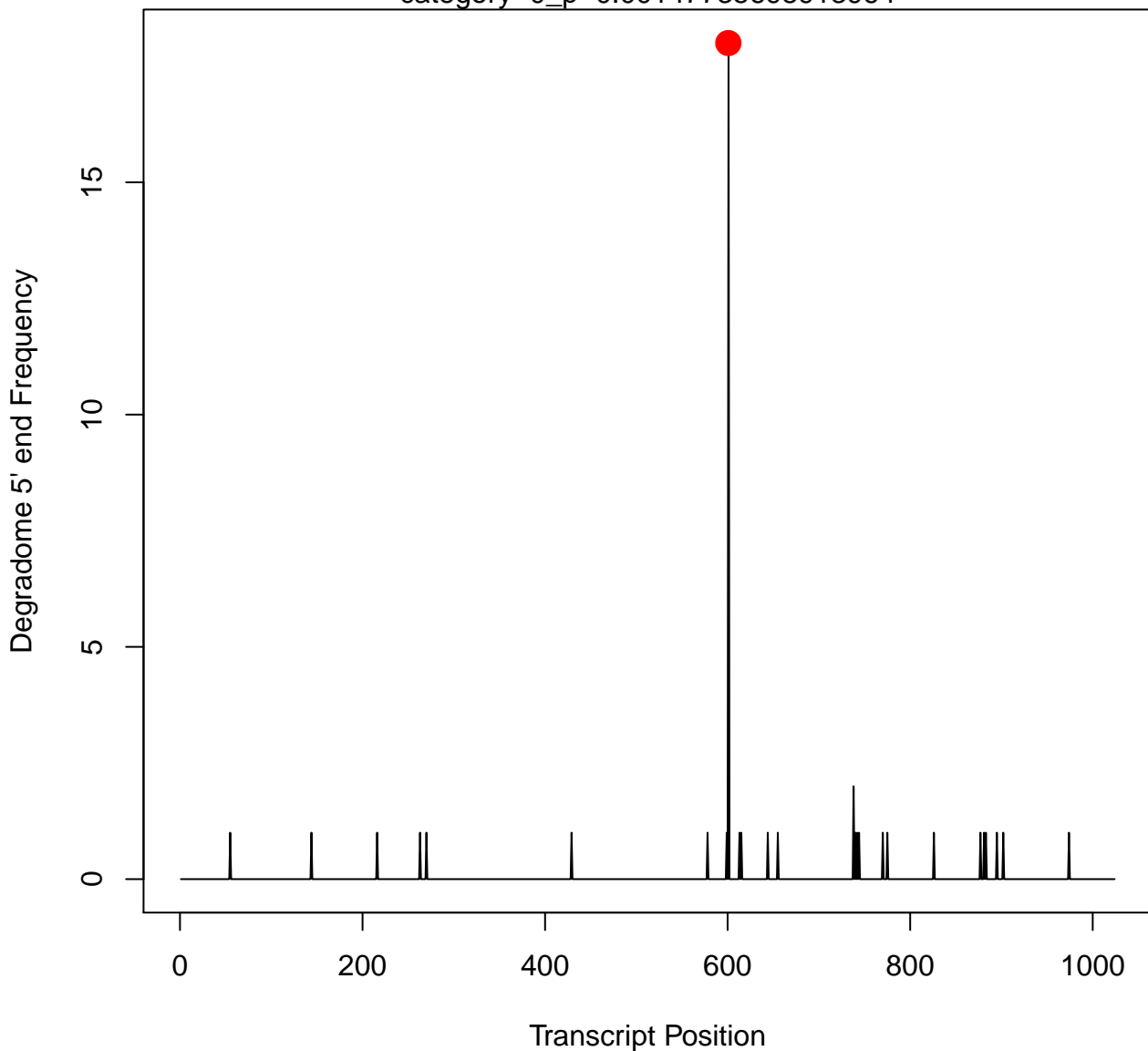

Supplement: Supplementary file 3 [file Data_Sheet_9.ZIP › GSM2230751.plot/Lsa-miR164a_Lsat_1_v5_gn_5_981.1_601_TPlot.pdf]

**T=Lsat\_1\_v5\_gn\_9\_26661.1\_Q=Lsa-miR164a\_S=364**

category=2\_p=0.737061012358555

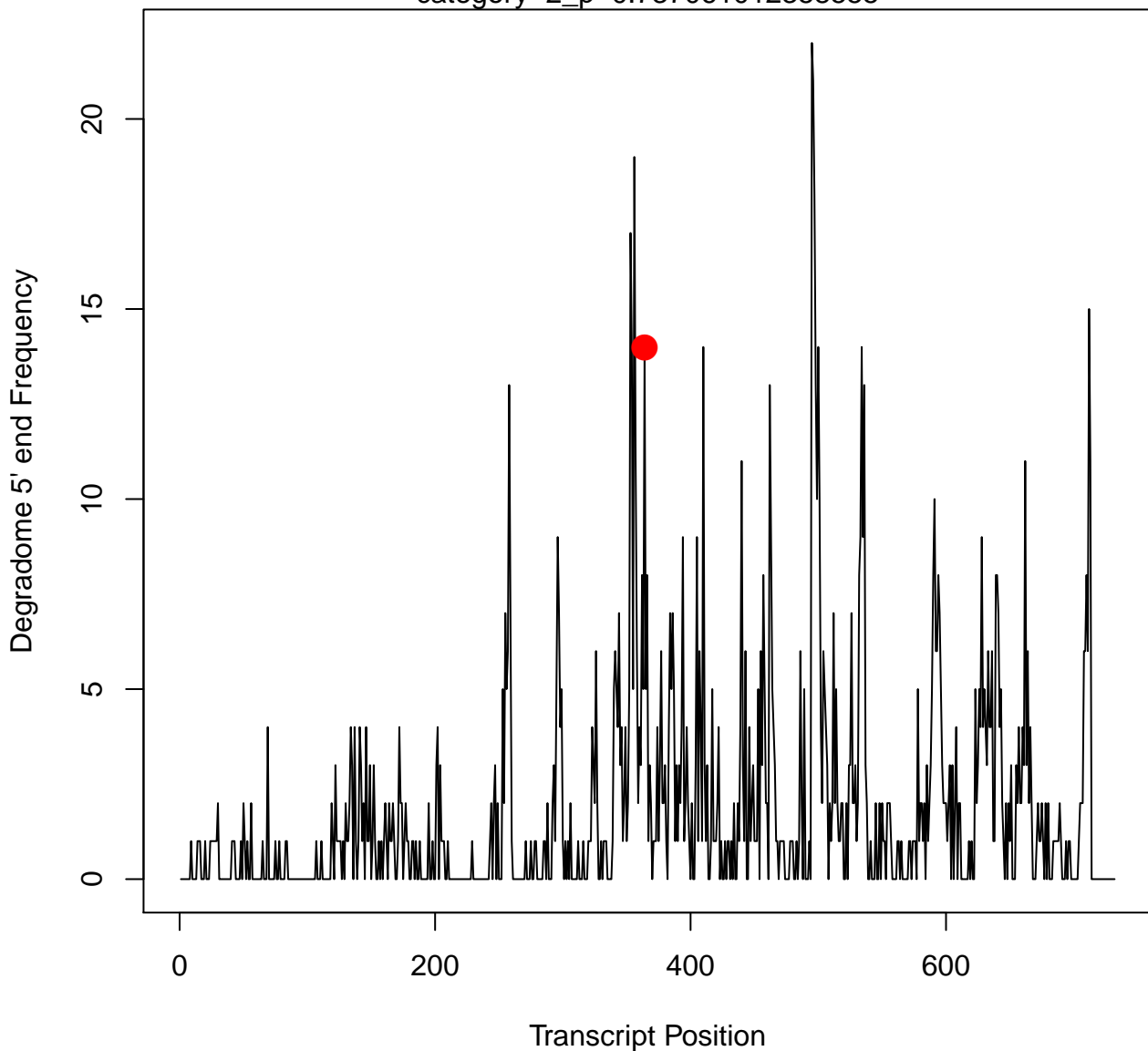

Supplement: Supplementary file 3 [file Data_Sheet_9.ZIP › GSM2230751.plot/Lsa-miR164a_Lsat_1_v5_gn_9_26661.1_364_TPlot.pdf]

**T=Lsat\_1\_v5\_gn\_4\_73580.1\_Q=Lsa-miR164b\_S=963**

category=2\_p=0.810311168621049

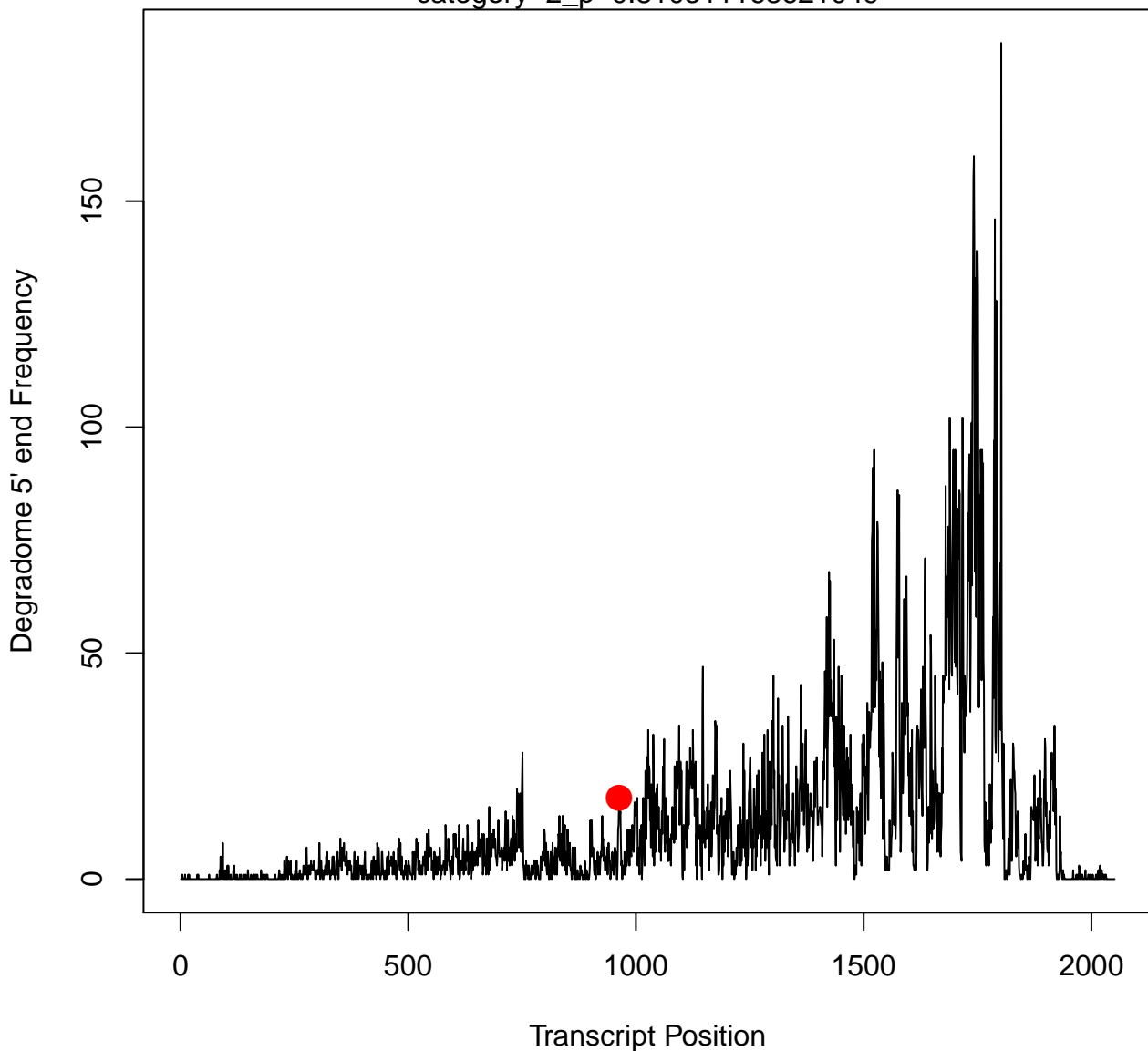

Supplement: Supplementary file 3 [file Data_Sheet_9.ZIP › GSM2230751.plot/Lsa-miR164b_Lsat_1_v5_gn_4_73580.1_963_TPlot.pdf]

**T=Lsat\_1\_v5\_gn\_6\_90981.1\_Q=Lsa-miR164b\_S=1203**

category=2\_p=0.340050901049337

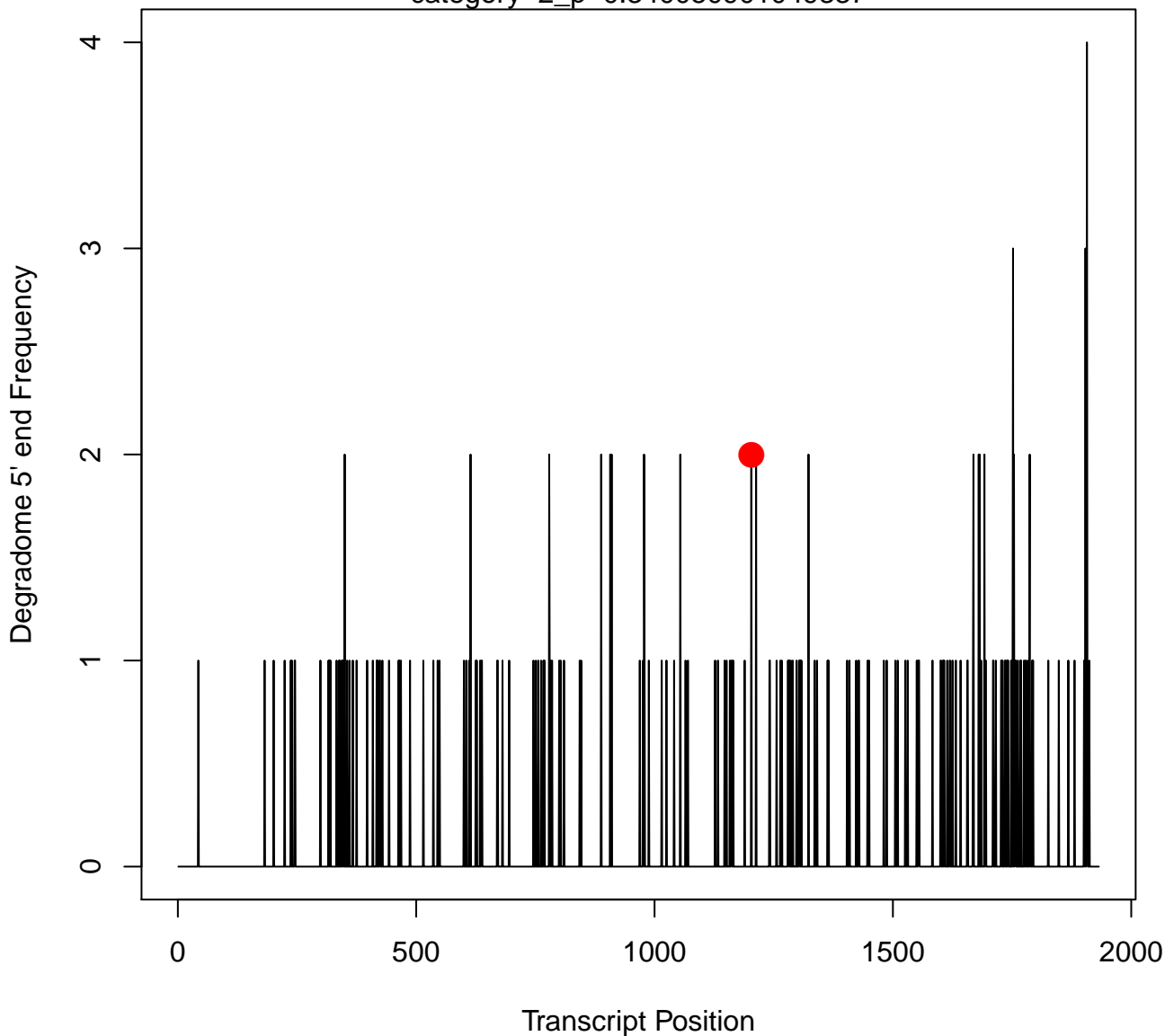

Supplement: Supplementary file 3 [file Data_Sheet_9.ZIP › GSM2230751.plot/Lsa-miR164b_Lsat_1_v5_gn_6_90981.1_1203_TPlot.pdf]

**T=Lsat\_1\_v5\_gn\_0\_45640.1\_Q=Lsa-miR164c\_S=643**

category=0\_p=0.0025839422340399

Degradome 5' end Frequency

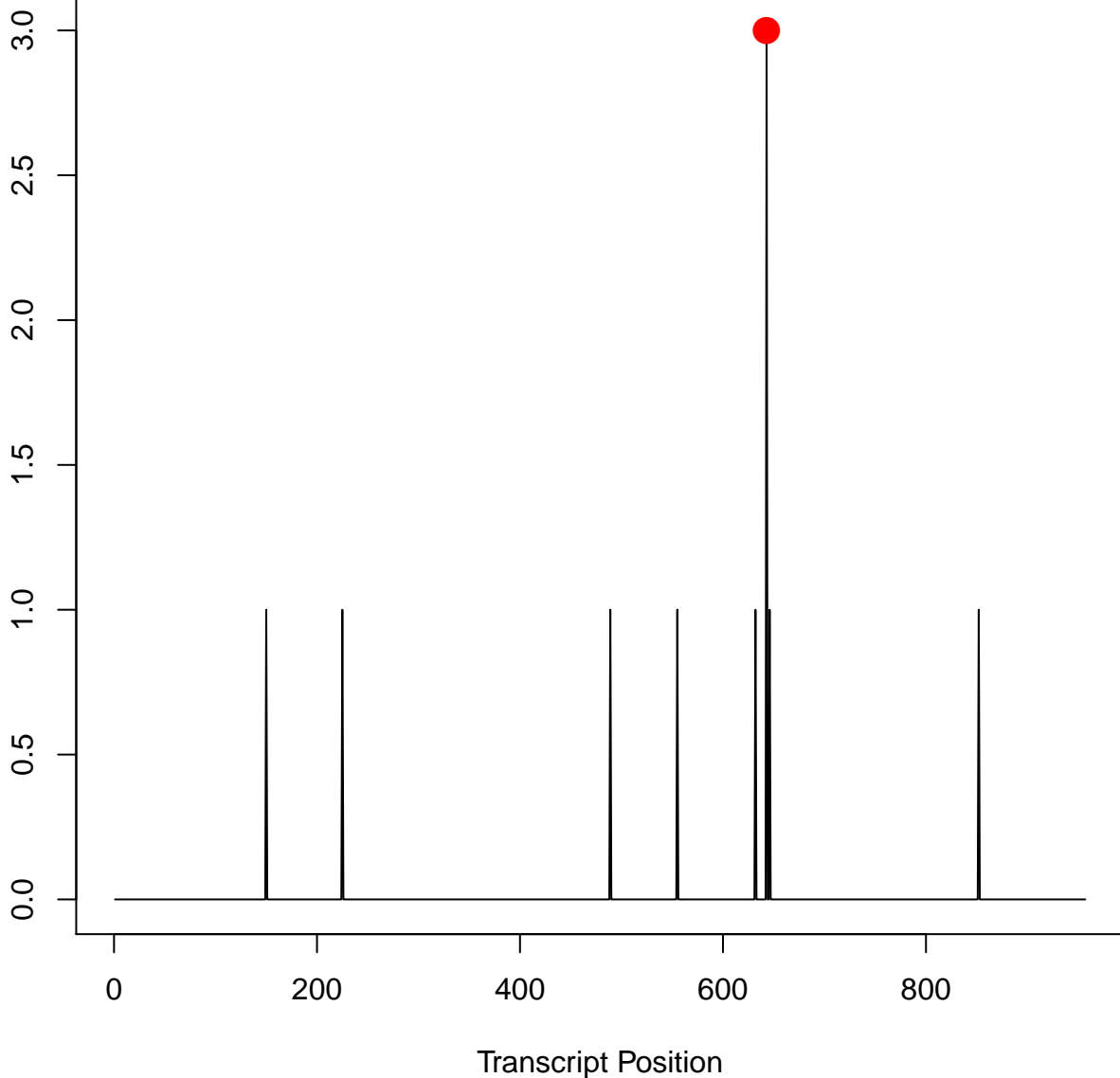

Supplement: Supplementary file 3 [file Data_Sheet_9.ZIP › GSM2230751.plot/Lsa-miR164c_Lsat_1_v5_gn_0_45640.1_643_TPlot.pdf]

**T=Lsat\_1\_v5\_gn\_4\_169300.1\_Q=Lsa-miR164c\_S=643**

category=0\_p=0.0022152168131917

Degradome 5' end Frequency

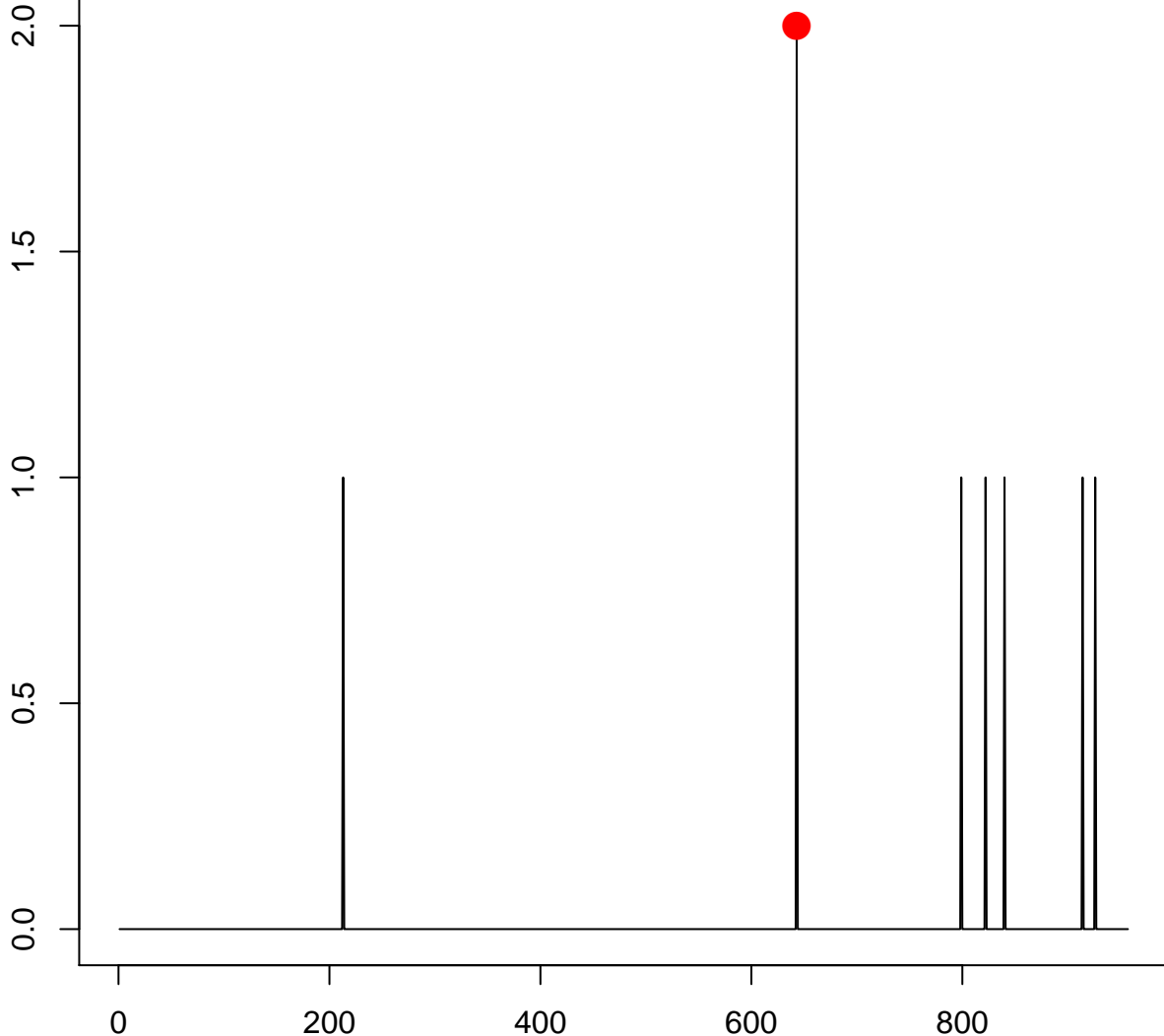

Transcript Position

Supplement: Supplementary file 3 [file Data_Sheet_9.ZIP › GSM2230751.plot/Lsa-miR164c_Lsat_1_v5_gn_4_169300.1_643_TPlot.pdf]

**T=Lsat\_1\_v5\_gn\_4\_62541.1\_Q=Lsa-miR164c\_S=396**

category=2\_p=0.551344504311096

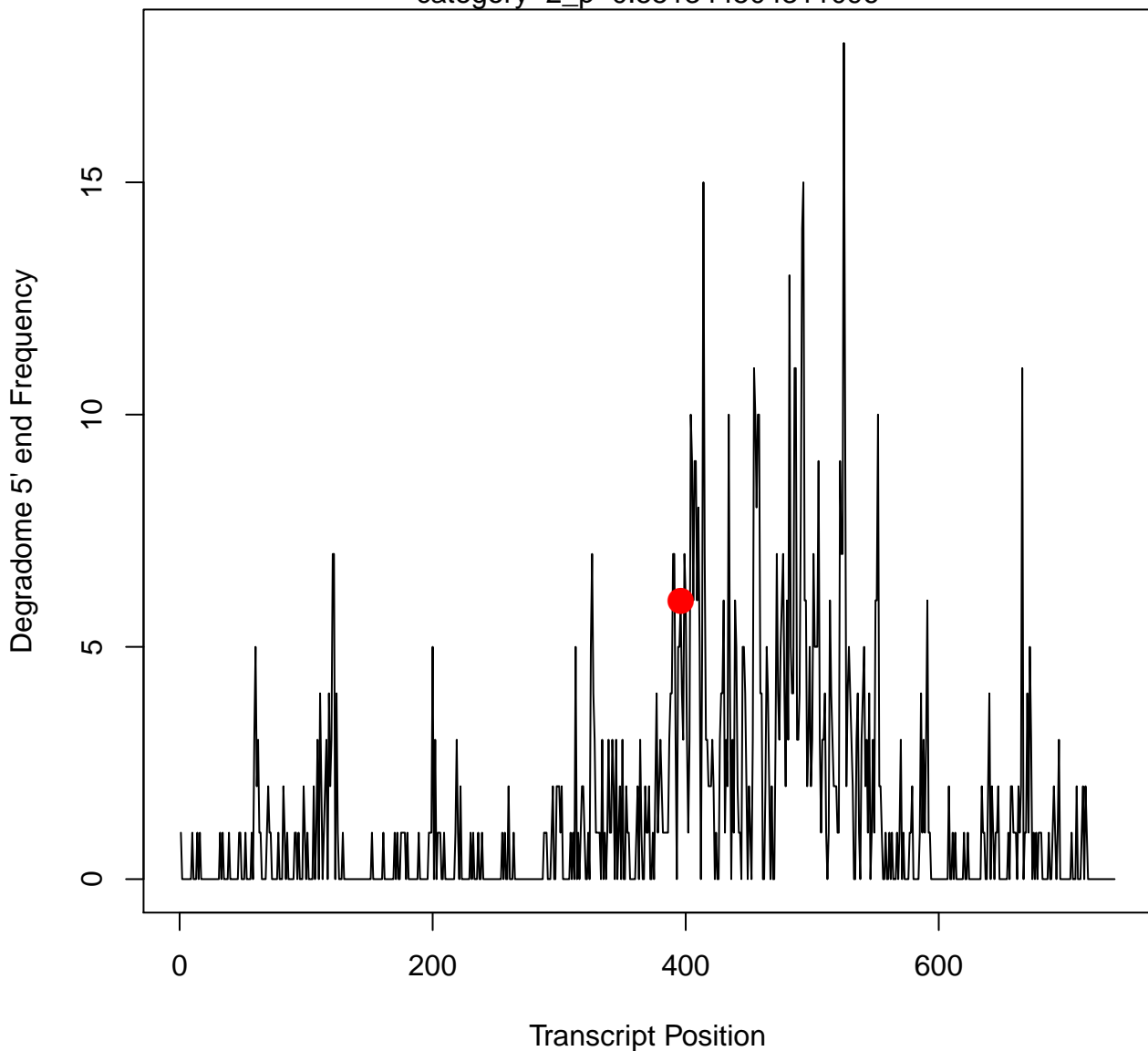

Supplement: Supplementary file 3 [file Data_Sheet_9.ZIP › GSM2230751.plot/Lsa-miR164c_Lsat_1_v5_gn_4_62541.1_396_TPlot.pdf]

**T=Lsat\_1\_v5\_gn\_7\_104460.1\_Q=Lsa-miR164c\_S=727**

category=0\_p=0.000369544041020964

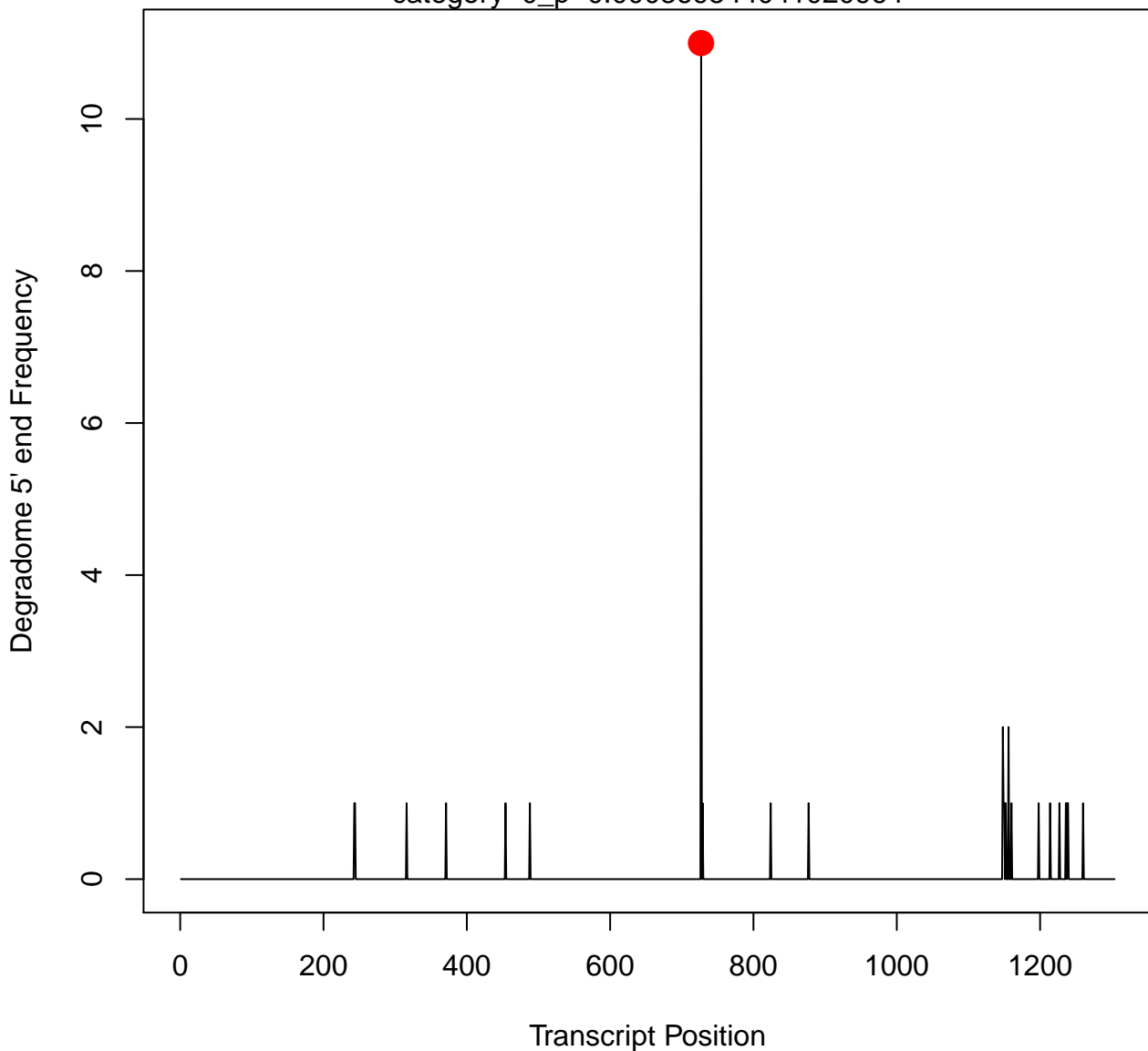

Supplement: Supplementary file 3 [file Data_Sheet_9.ZIP › GSM2230751.plot/Lsa-miR164c_Lsat_1_v5_gn_7_104460.1_727_TPlot.pdf]

**T=Lsat\_1\_v5\_gn\_9\_26661.1\_Q=Lsa-miR164c\_S=363**

category=2\_p=0.845902007472907

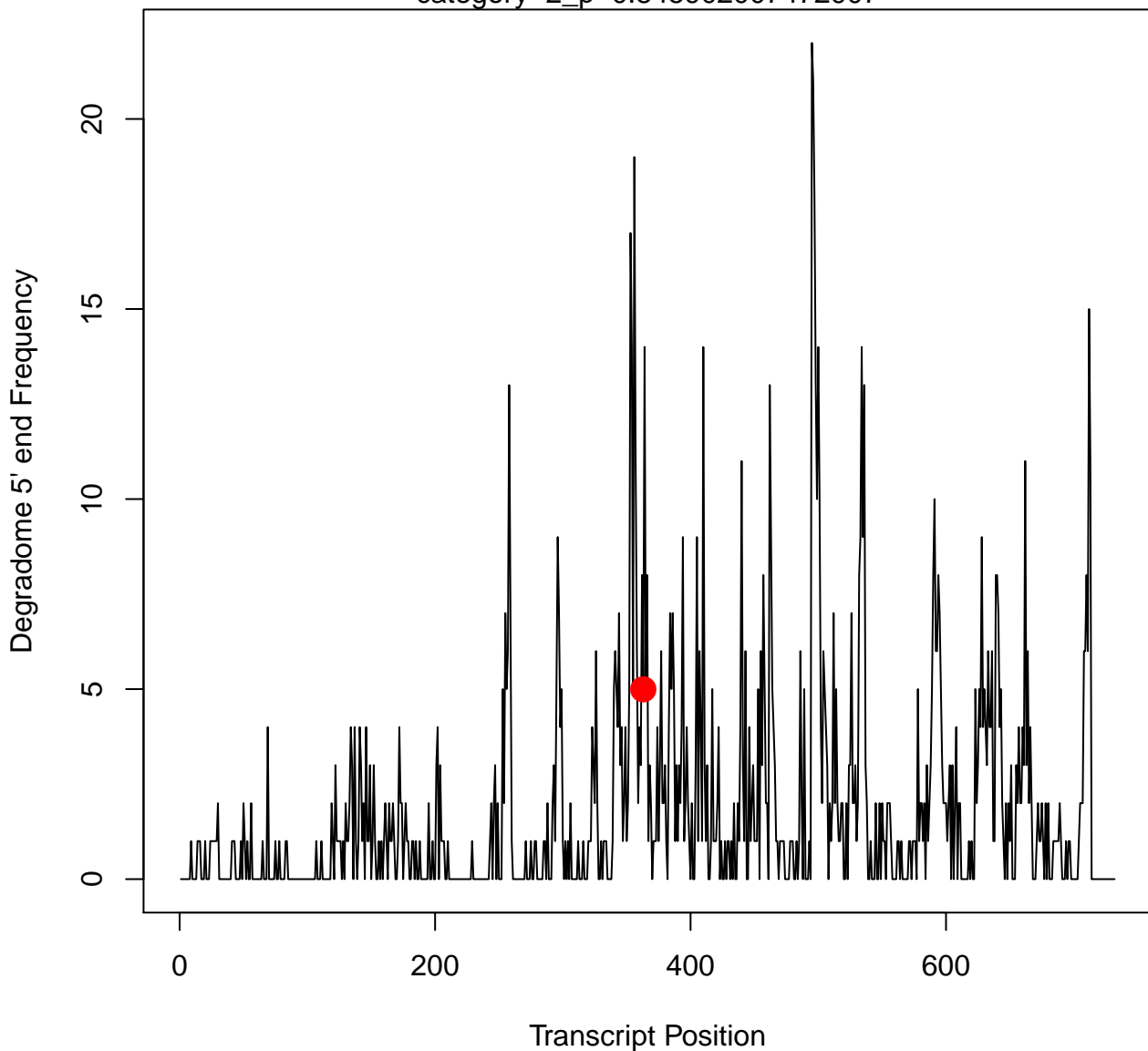

Supplement: Supplementary file 3 [file Data_Sheet_9.ZIP › GSM2230751.plot/Lsa-miR164c_Lsat_1_v5_gn_9_26661.1_363_TPlot.pdf]

**T=Lsat\_1\_v5\_gn\_1\_75020.1\_Q=Lsa-miR166a\_S=1310**

category=2\_p=0.551344504311096

Degradome 5' end Frequency

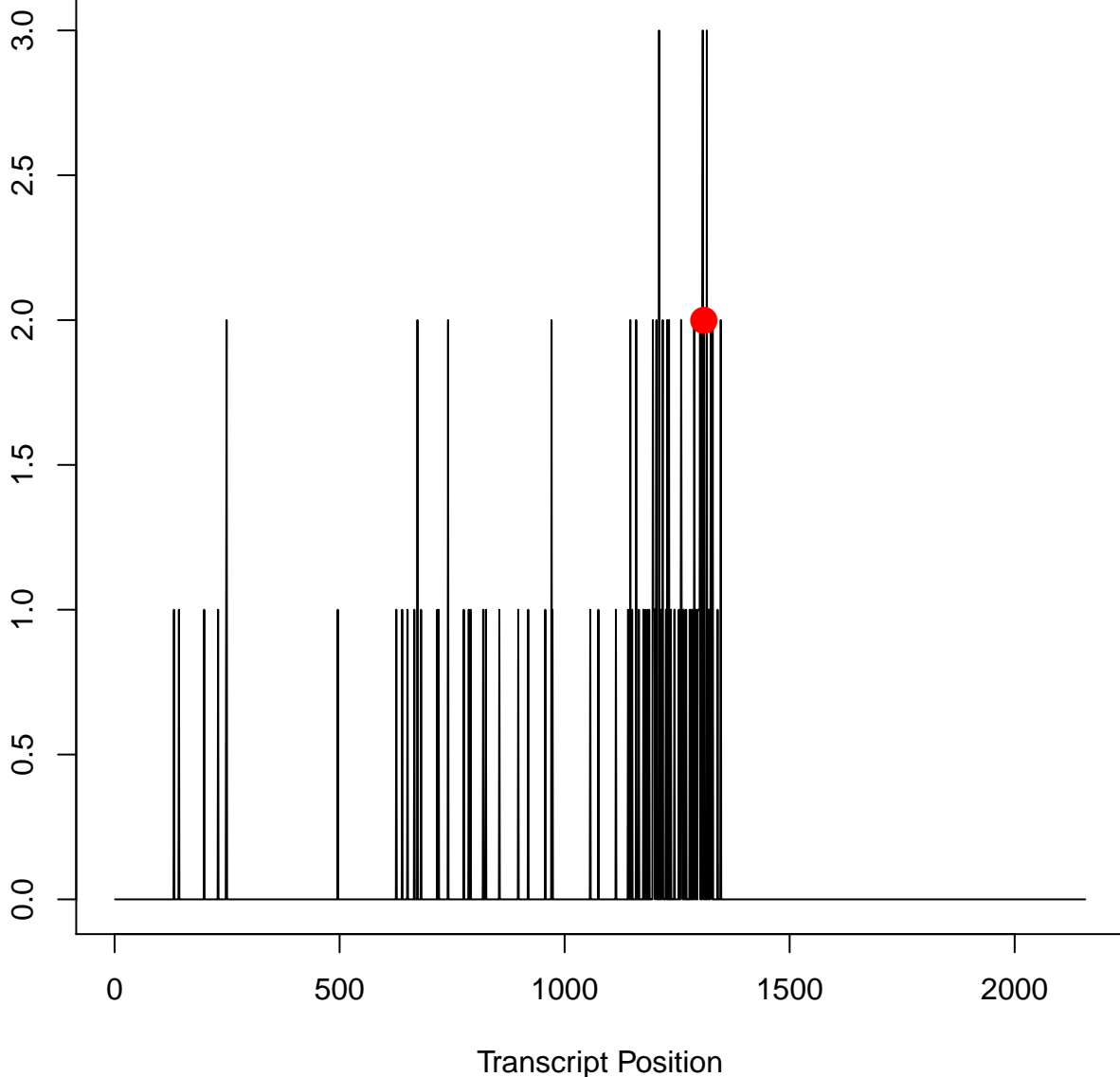

Supplement: Supplementary file 3 [file Data_Sheet_9.ZIP › GSM2230751.plot/Lsa-miR166a_Lsat_1_v5_gn_1_75020.1_1310_TPlot.pdf]

**T=Lsat\_1\_v5\_gn\_4\_113220.1\_Q=Lsa-miR166a\_S=830**

category=2\_p=0.882030978163789

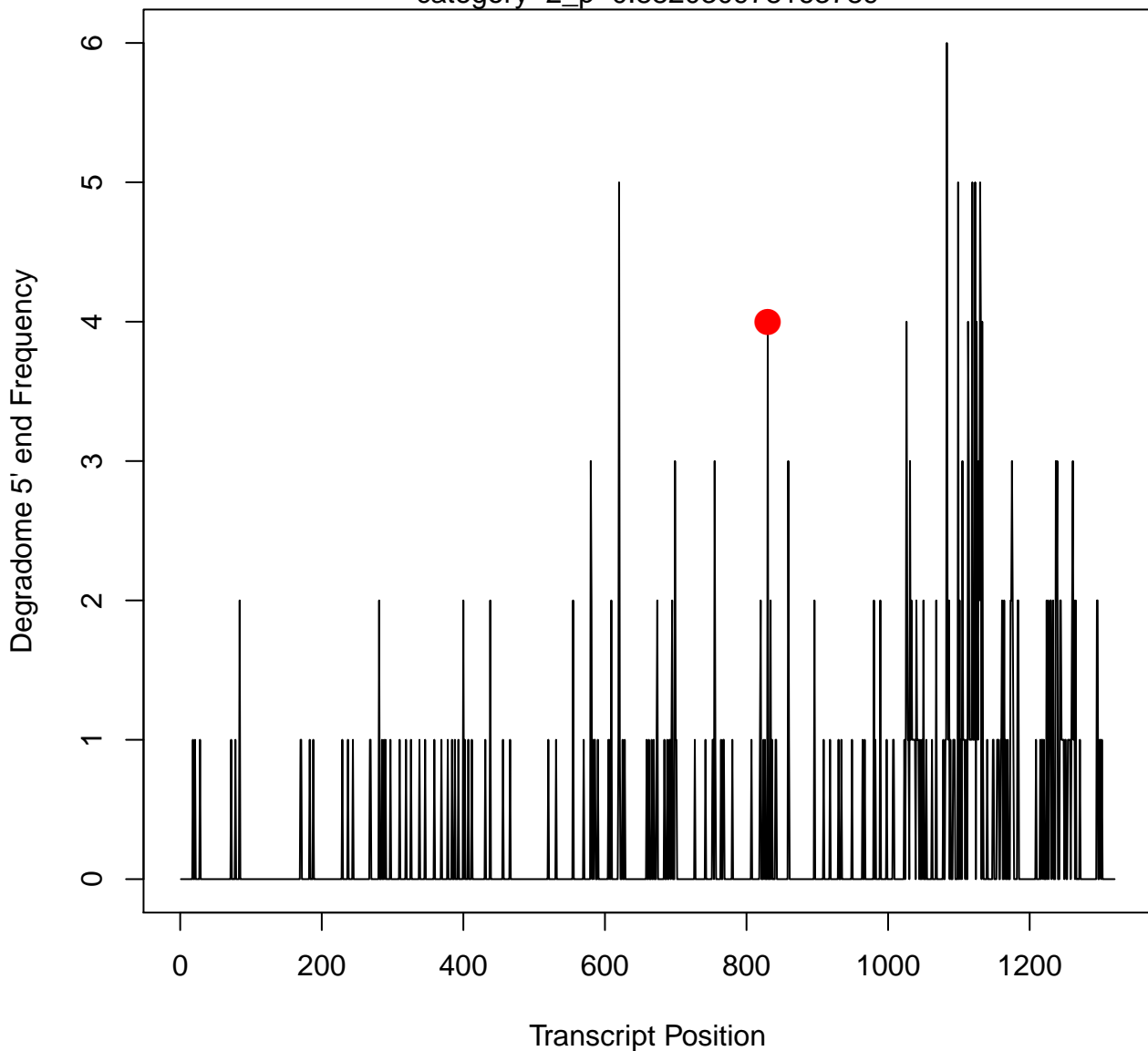

Supplement: Supplementary file 3 [file Data_Sheet_9.ZIP › GSM2230751.plot/Lsa-miR166a_Lsat_1_v5_gn_4_113220.1_830_TPlot.pdf]

**T=Lsat\_1\_v5\_gn\_6\_22720.1\_Q=Lsa-miR166a\_S=565**

category=0\_p=0.00147735698913964

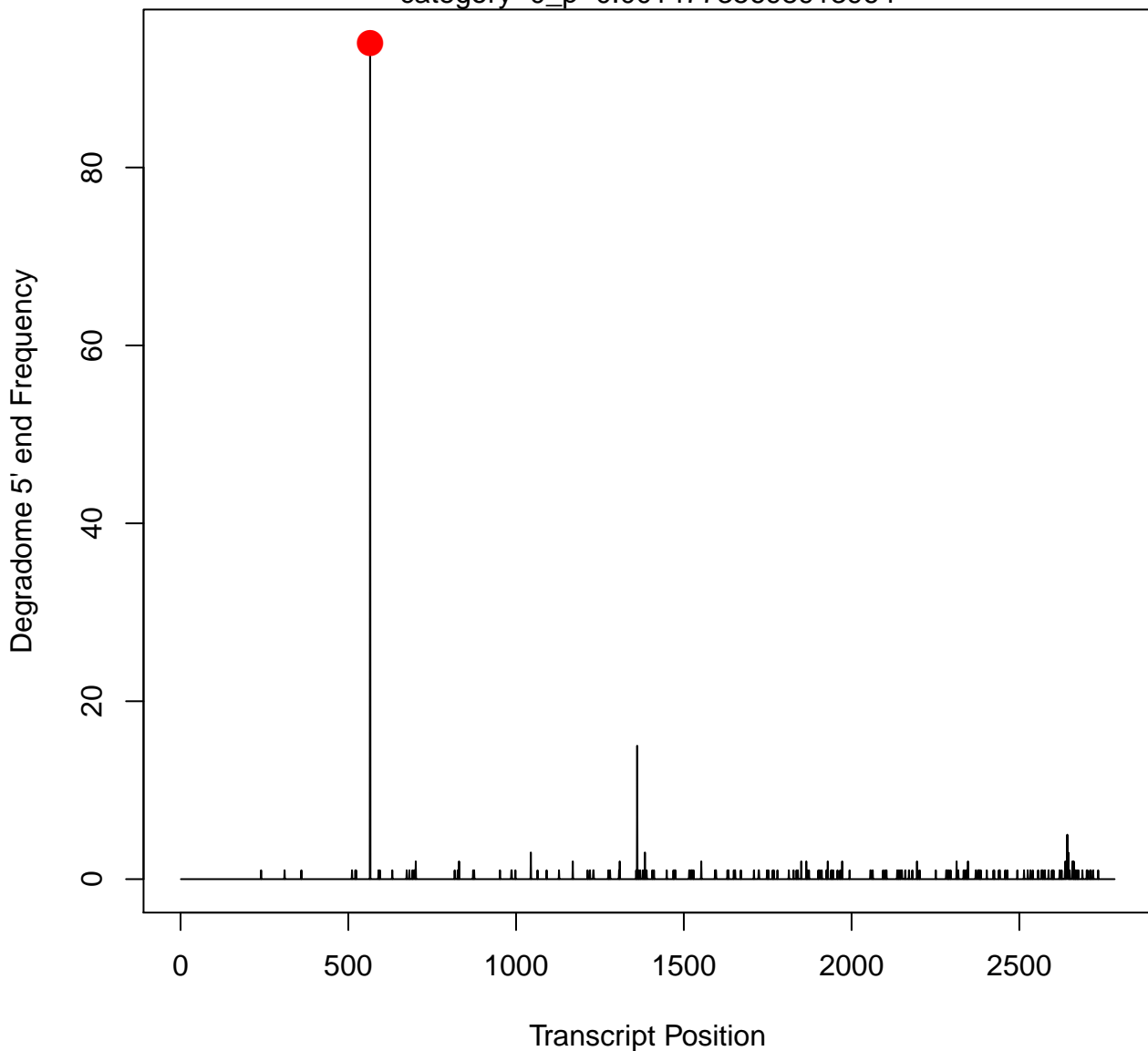

Supplement: Supplementary file 3 [file Data_Sheet_9.ZIP › GSM2230751.plot/Lsa-miR166a_Lsat_1_v5_gn_6_22720.1_565_TPlot.pdf]

**T=Lsat\_1\_v5\_gn\_2\_100140.1\_Q=Lsa-miR166c\_S=1400**

category=0\_p=0.00147735698913964

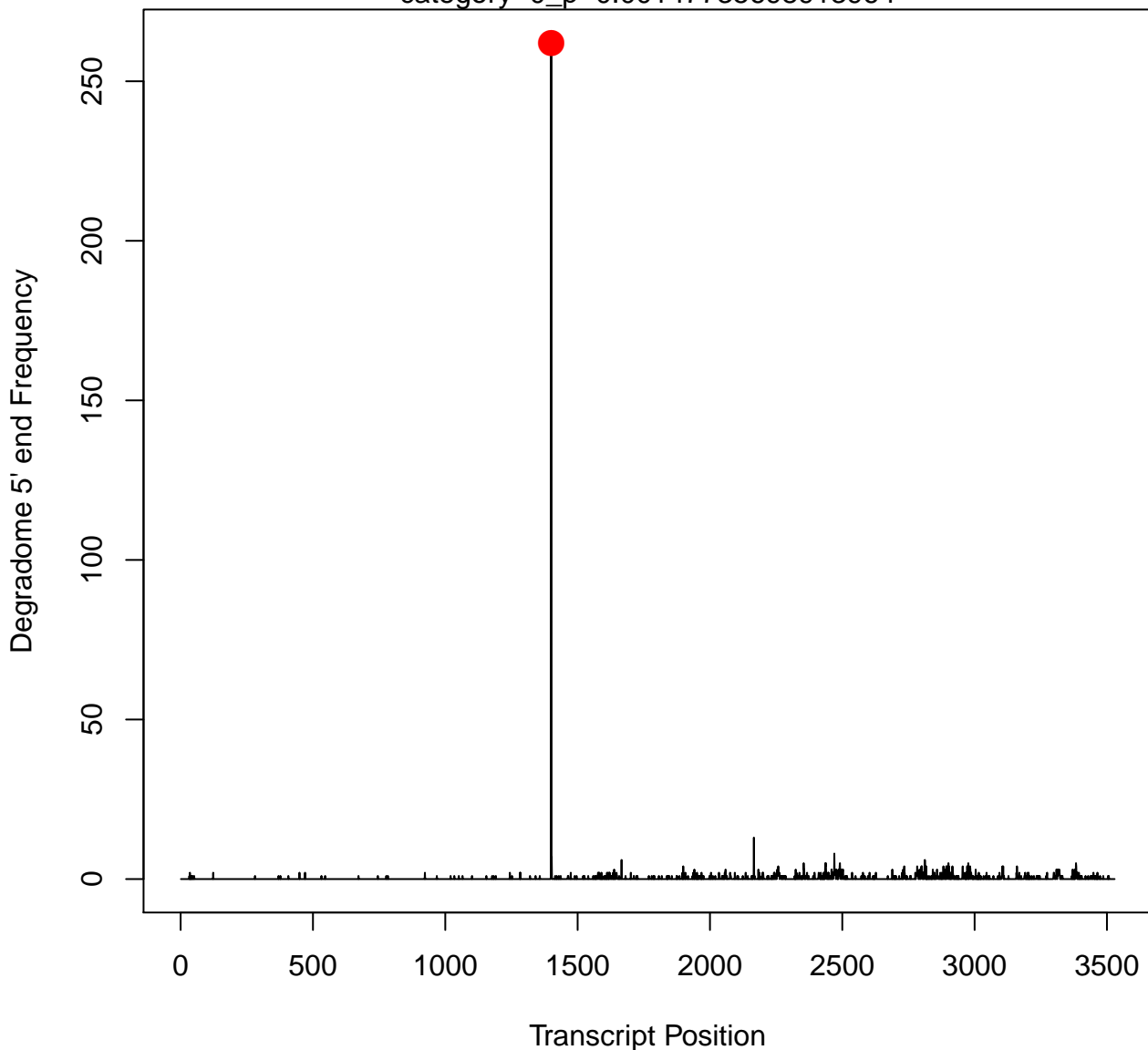

Supplement: Supplementary file 3 [file Data_Sheet_9.ZIP › GSM2230751.plot/Lsa-miR166c_Lsat_1_v5_gn_2_100140.1_1400_TPlot.pdf]

**T=Lsat\_1\_v5\_gn\_2\_103120.1\_Q=Lsa-miR166f\_S=484**

category=2\_p=0.854784572309762

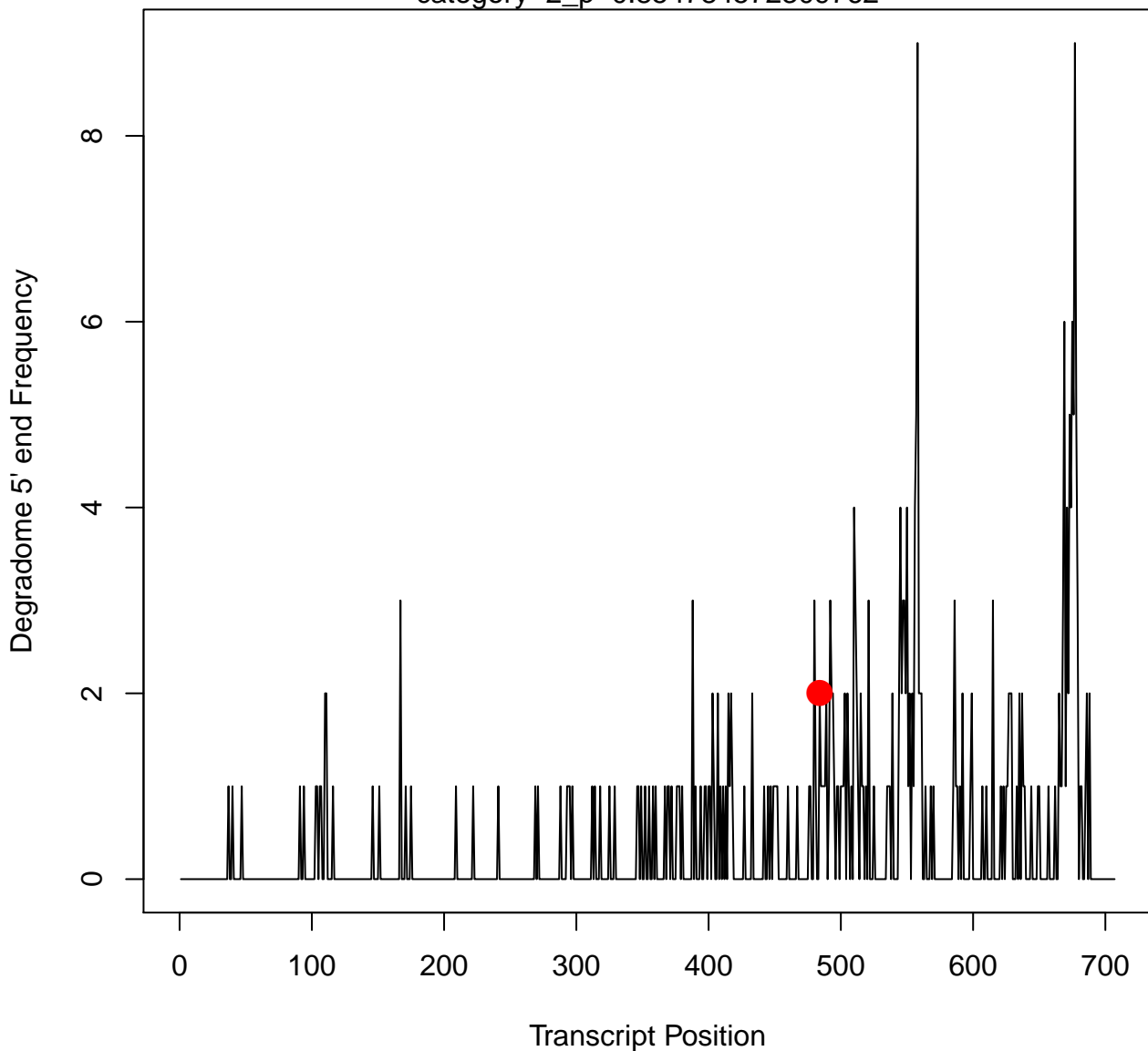

Supplement: Supplementary file 3 [file Data_Sheet_9.ZIP › GSM2230751.plot/Lsa-miR166f_Lsat_1_v5_gn_2_103120.1_484_TPlot.pdf]

**T=Lsat\_1\_v5\_gn\_3\_115940.1\_Q=Lsa-miR166f\_S=1058**

category=2\_p=0.211388494938405

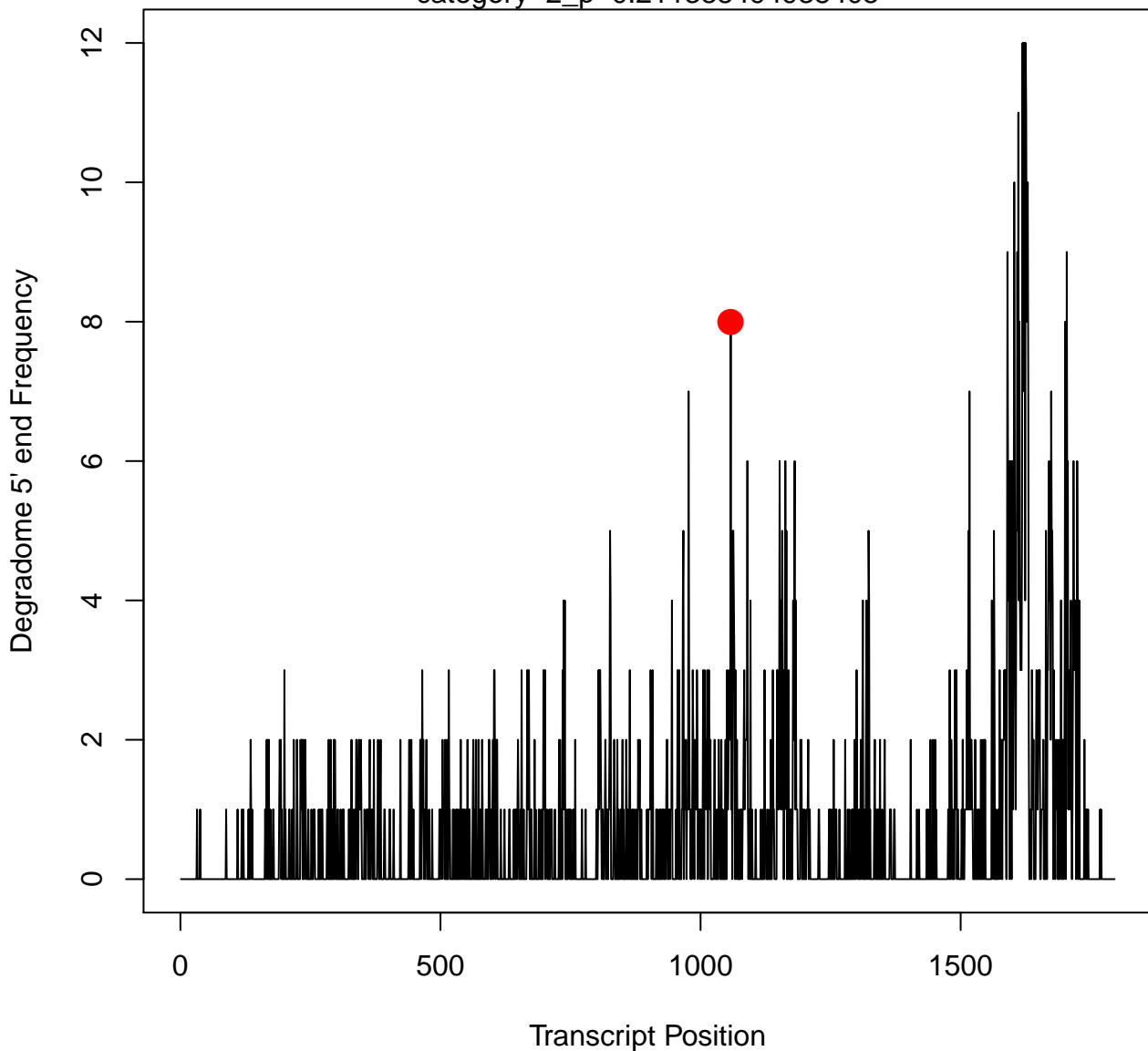

Supplement: Supplementary file 3 [file Data_Sheet_9.ZIP › GSM2230751.plot/Lsa-miR166f_Lsat_1_v5_gn_3_115940.1_1058_TPlot.pdf]

**T=Lsat\_1\_v5\_gn\_6\_45641.1\_Q=Lsa-miR166f\_S=1309**

category=0\_p=0.00147735698913964

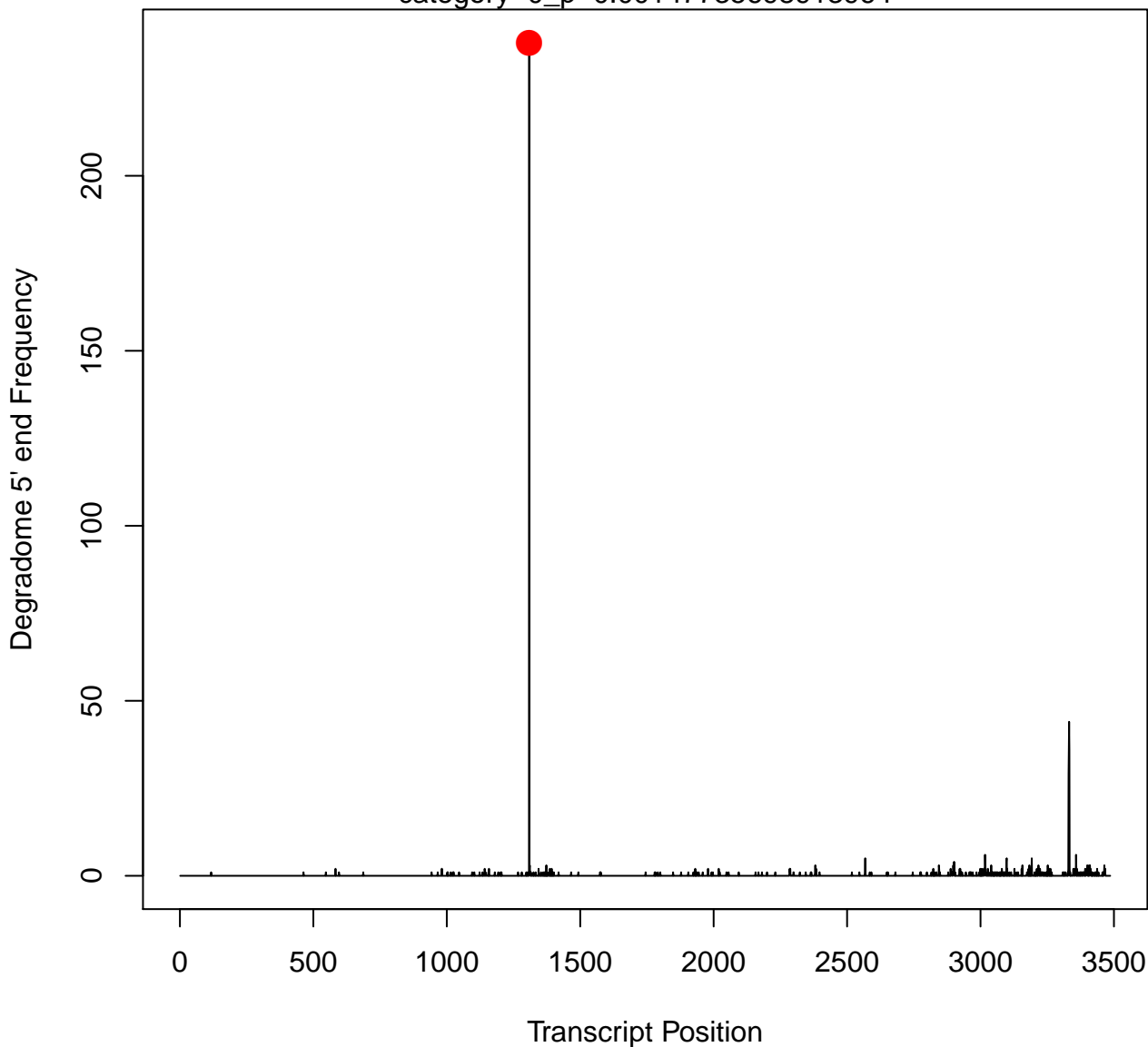

Supplement: Supplementary file 3 [file Data_Sheet_9.ZIP › GSM2230751.plot/Lsa-miR166f_Lsat_1_v5_gn_6_45641.1_1309_TPlot.pdf]

**T=Lsat\_1\_v5\_gn\_9\_29121.1\_Q=Lsa-miR166f\_S=2923**

category=2\_p=0.759464793296244

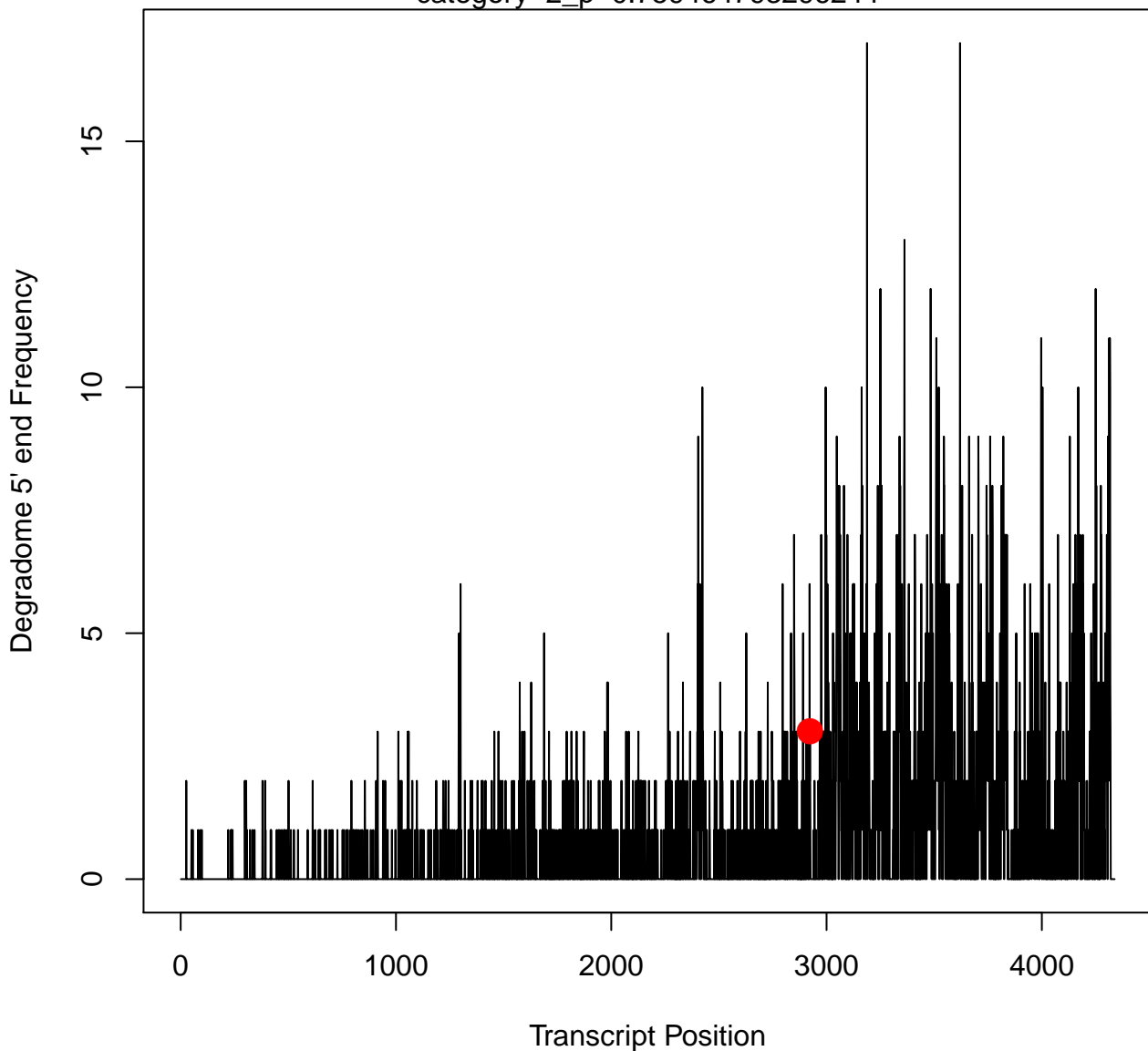

Supplement: Supplementary file 3 [file Data_Sheet_9.ZIP › GSM2230751.plot/Lsa-miR166f_Lsat_1_v5_gn_9_29121.1_2923_TPlot.pdf]

**T=Lsat\_1\_v5\_gn\_2\_103140.1\_Q=Lsa-miR166g\_S=617**

category=2\_p=0.983370138768379

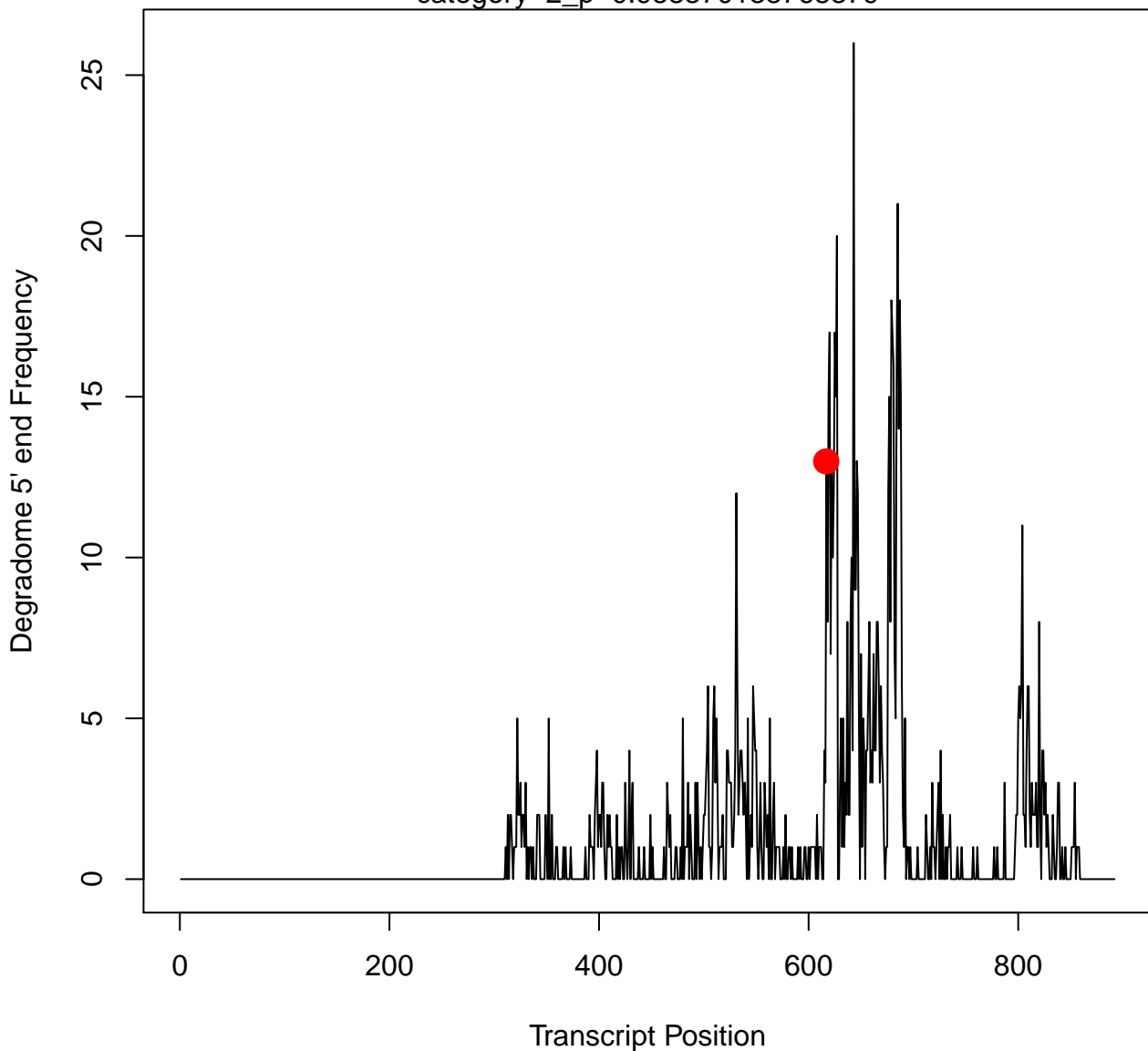

Supplement: Supplementary file 3 [file Data_Sheet_9.ZIP › GSM2230751.plot/Lsa-miR166g_Lsat_1_v5_gn_2_103140.1_617_TPlot.pdf]

**T=Lsat\_1\_v5\_gn\_5\_134441.1\_Q=Lsa-miR166g\_S=568**

category=0\_p=0.000738951519243747

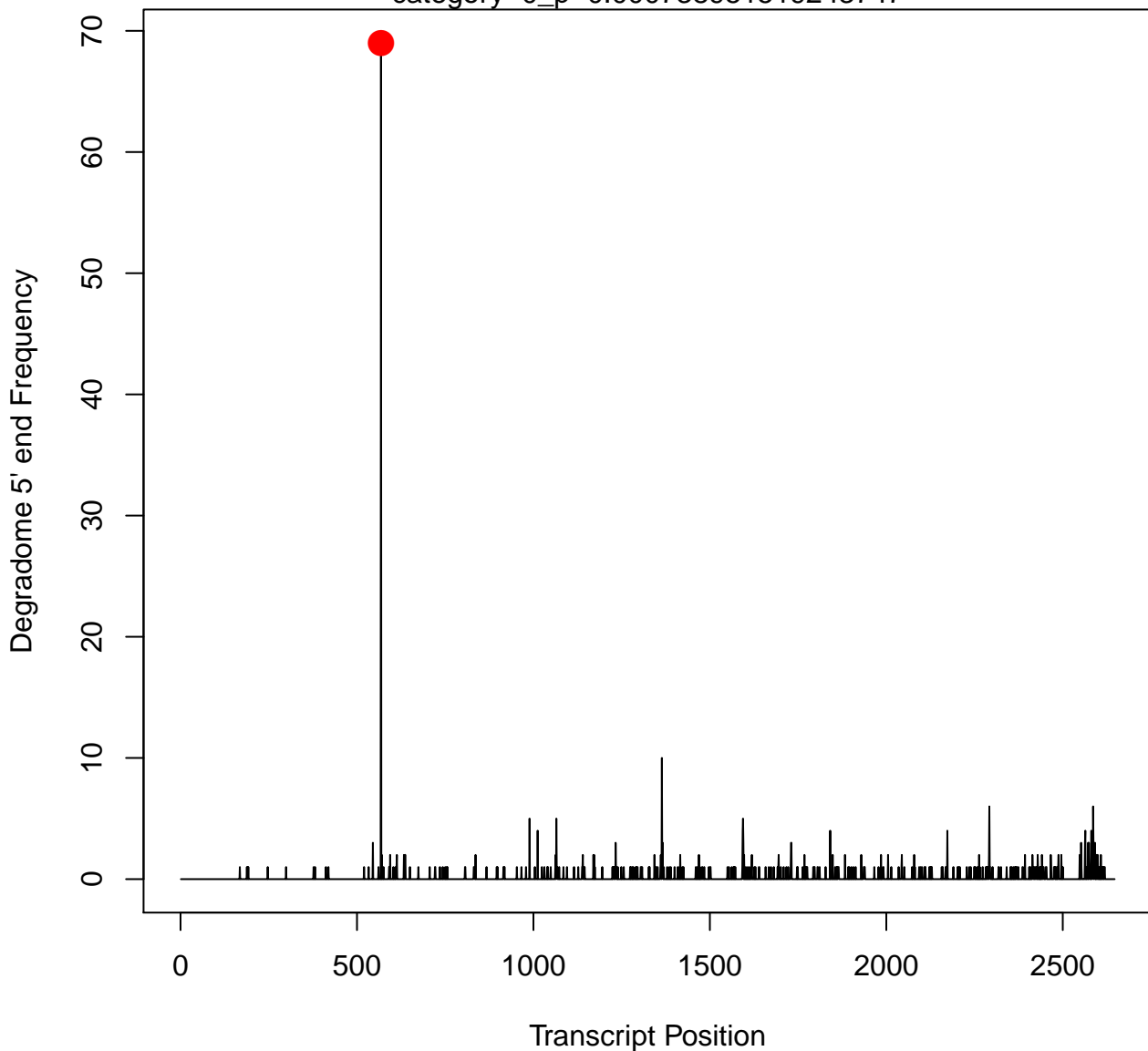

Supplement: Supplementary file 3 [file Data_Sheet_9.ZIP › GSM2230751.plot/Lsa-miR166g_Lsat_1_v5_gn_5_134441.1_568_TPlot.pdf]
