# Supplementary figures and images for: Comprehensive Annotation and Functional Exploration of MicroRNAs in Lettuce (part 6 of 6)
Source: Front Plant Sci. 2021 Dec 24;12:781836. doi: 10.3389/fpls.2021.781836 (PMC8739914; doi:10.3389/fpls.2021.781836)

**T=Lsat\_1\_v5\_gn\_5\_134441.1\_Q=Lsa-miR166i\_S=570**

category=2\_p=0.0564350766159708

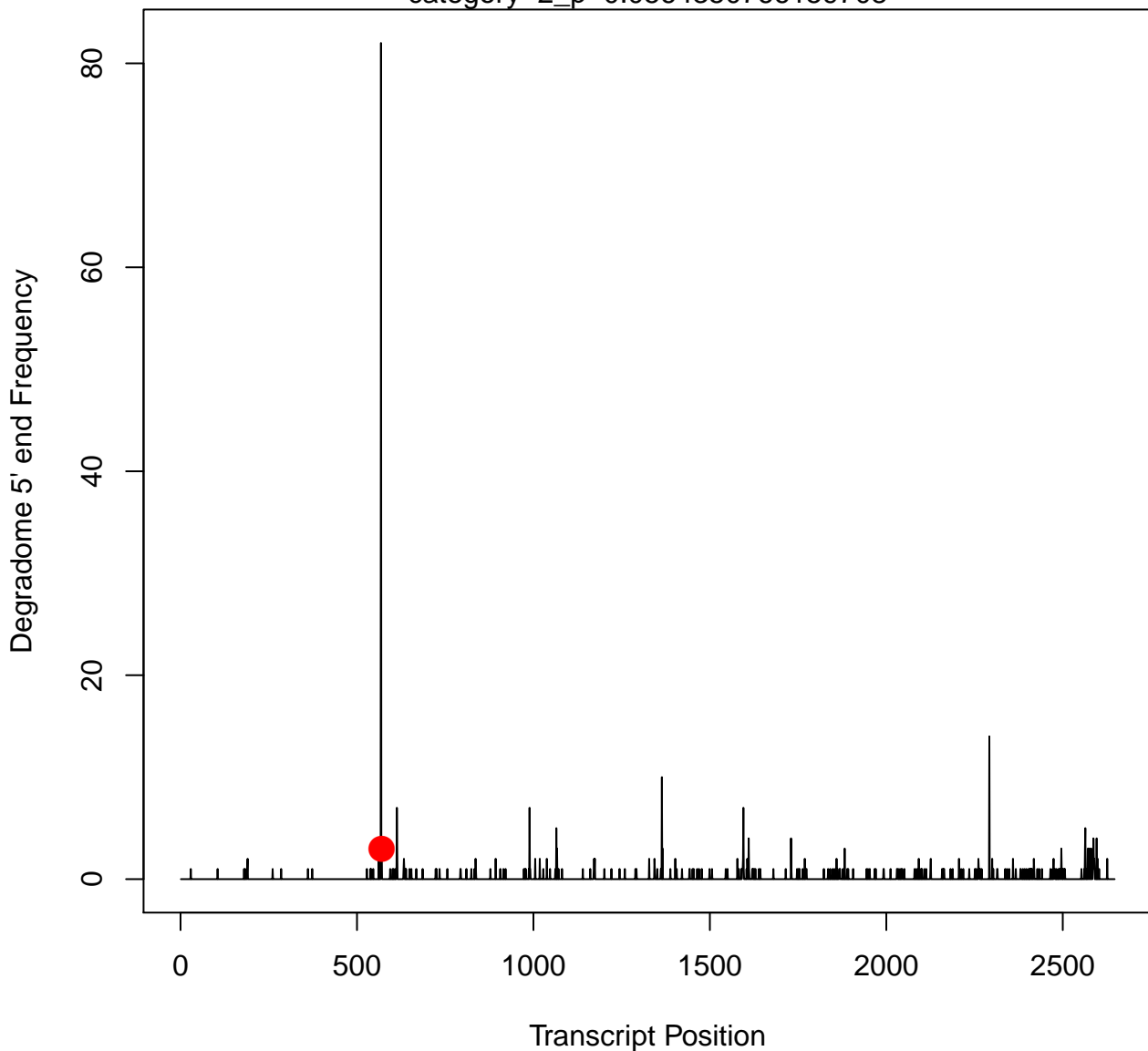

Supplement: Supplementary Data 1 — Results of categories 0–2 from PARE-Seq analysis (including three subfiles:1_1, 1_2, 1_3). [file Data_Sheet_10.ZIP › GSM2230754.plot/Lsa-miR166i_Lsat_1_v5_gn_5_134441.1_570_TPlot.pdf]

**T=Lsat\_1\_v5\_gn\_5\_184280.1\_Q=Lsa-miR166i\_S=914**

category=0\_p=0.00300231227468162

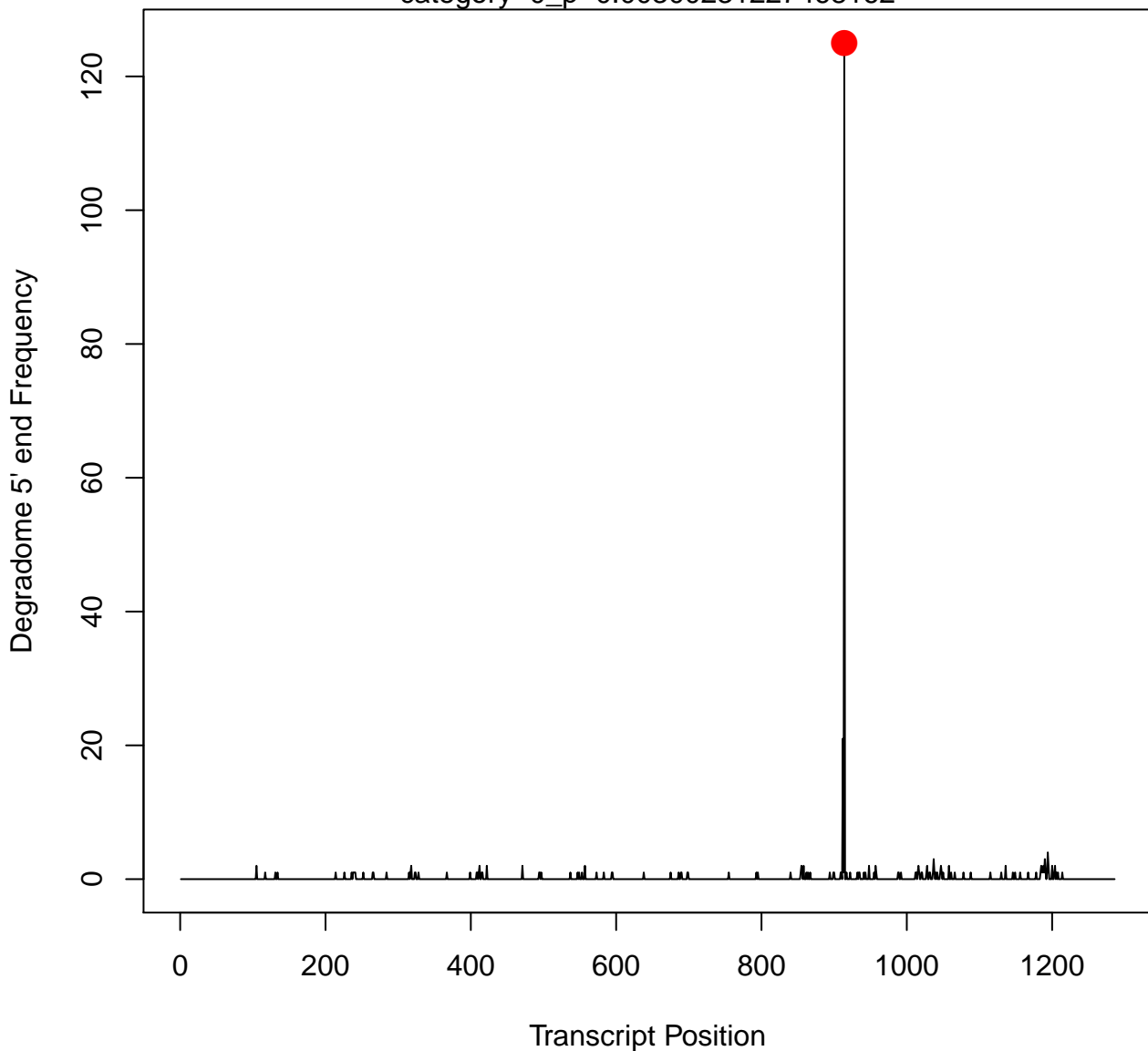

Supplement: Supplementary Data 1 — Results of categories 0–2 from PARE-Seq analysis (including three subfiles:1_1, 1_2, 1_3). [file Data_Sheet_10.ZIP › GSM2230754.plot/Lsa-miR166i_Lsat_1_v5_gn_5_184280.1_914_TPlot.pdf]

**T=Lsat\_1\_v5\_gn\_5\_32500.1\_Q=Lsa-miR166i\_S=567**

category=2\_p=0.183979146537541

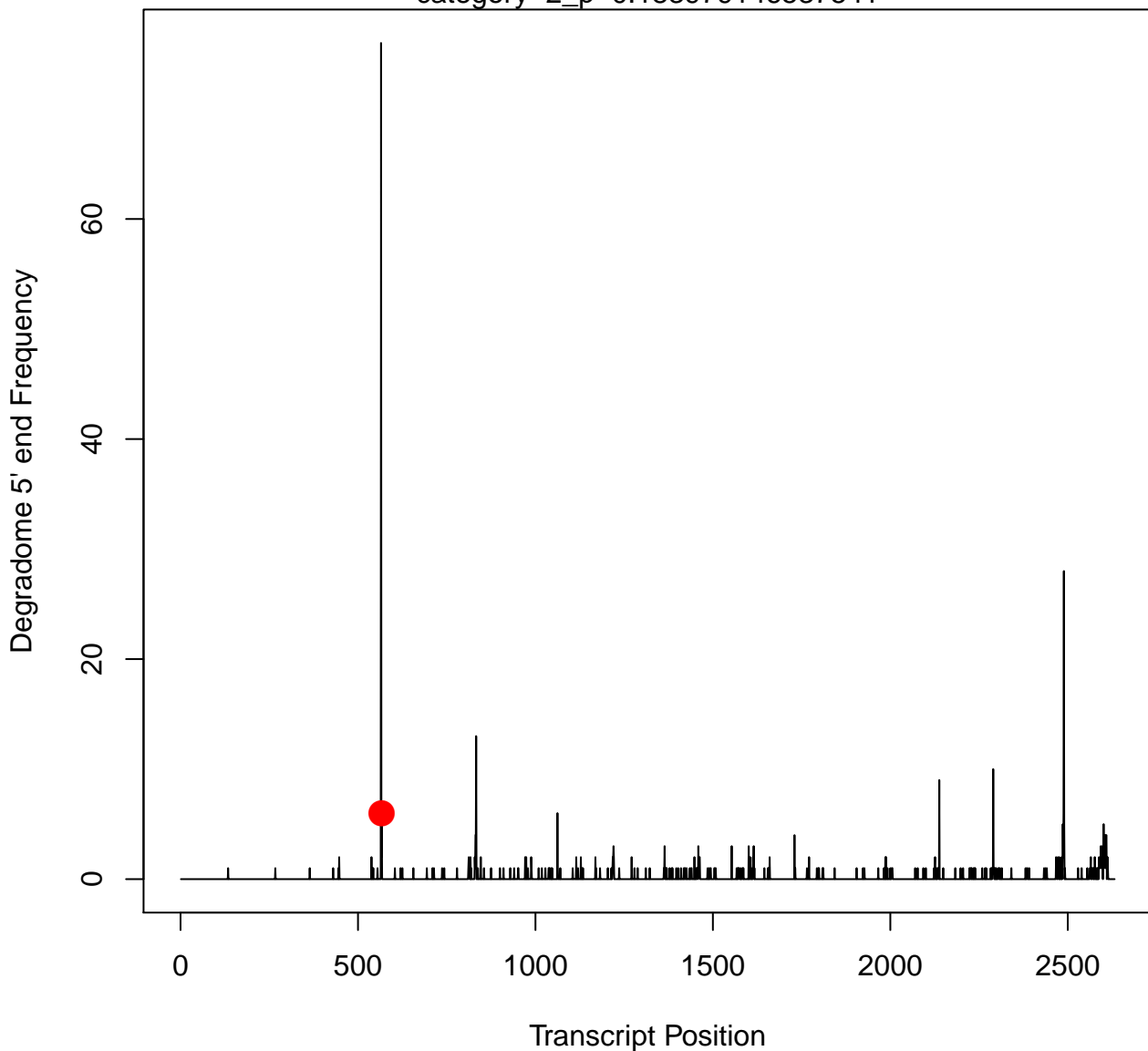

Supplement: Supplementary Data 1 — Results of categories 0–2 from PARE-Seq analysis (including three subfiles:1_1, 1_2, 1_3). [file Data_Sheet_10.ZIP › GSM2230754.plot/Lsa-miR166i_Lsat_1_v5_gn_5_32500.1_567_TPlot.pdf]

**T=Lsat\_1\_v5\_gn\_5\_84680.1\_Q=Lsa-miR166i\_S=2196**

category=2\_p=0.848614909898006

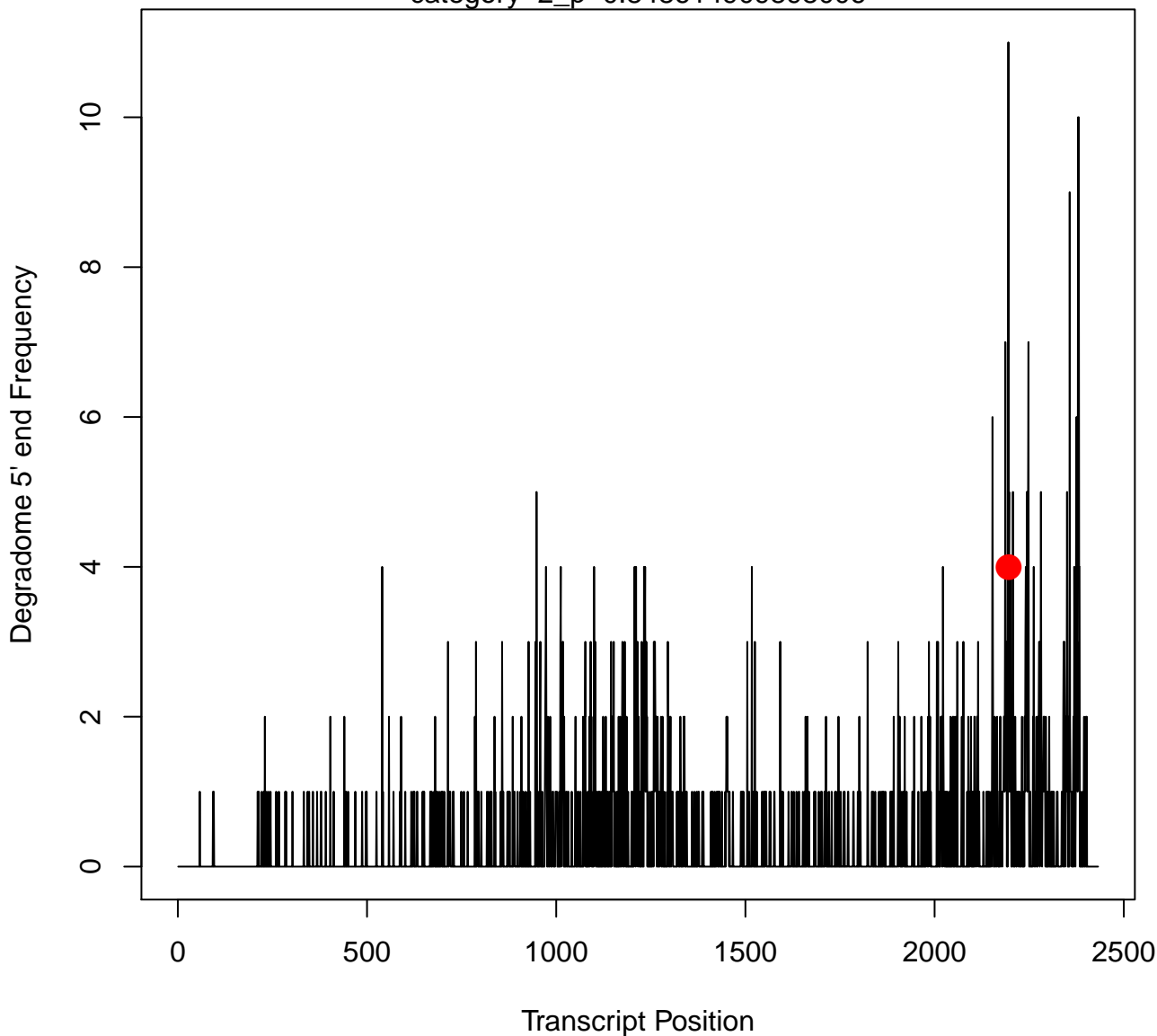

Supplement: Supplementary Data 1 — Results of categories 0–2 from PARE-Seq analysis (including three subfiles:1_1, 1_2, 1_3). [file Data_Sheet_10.ZIP › GSM2230754.plot/Lsa-miR166i_Lsat_1_v5_gn_5_84680.1_2196_TPlot.pdf]

**T=Lsat\_1\_v5\_gn\_6\_2981.1\_Q=Lsa-miR166i\_S=487**

category=2\_p=0.627505047091162

Degradome 5' end Frequency

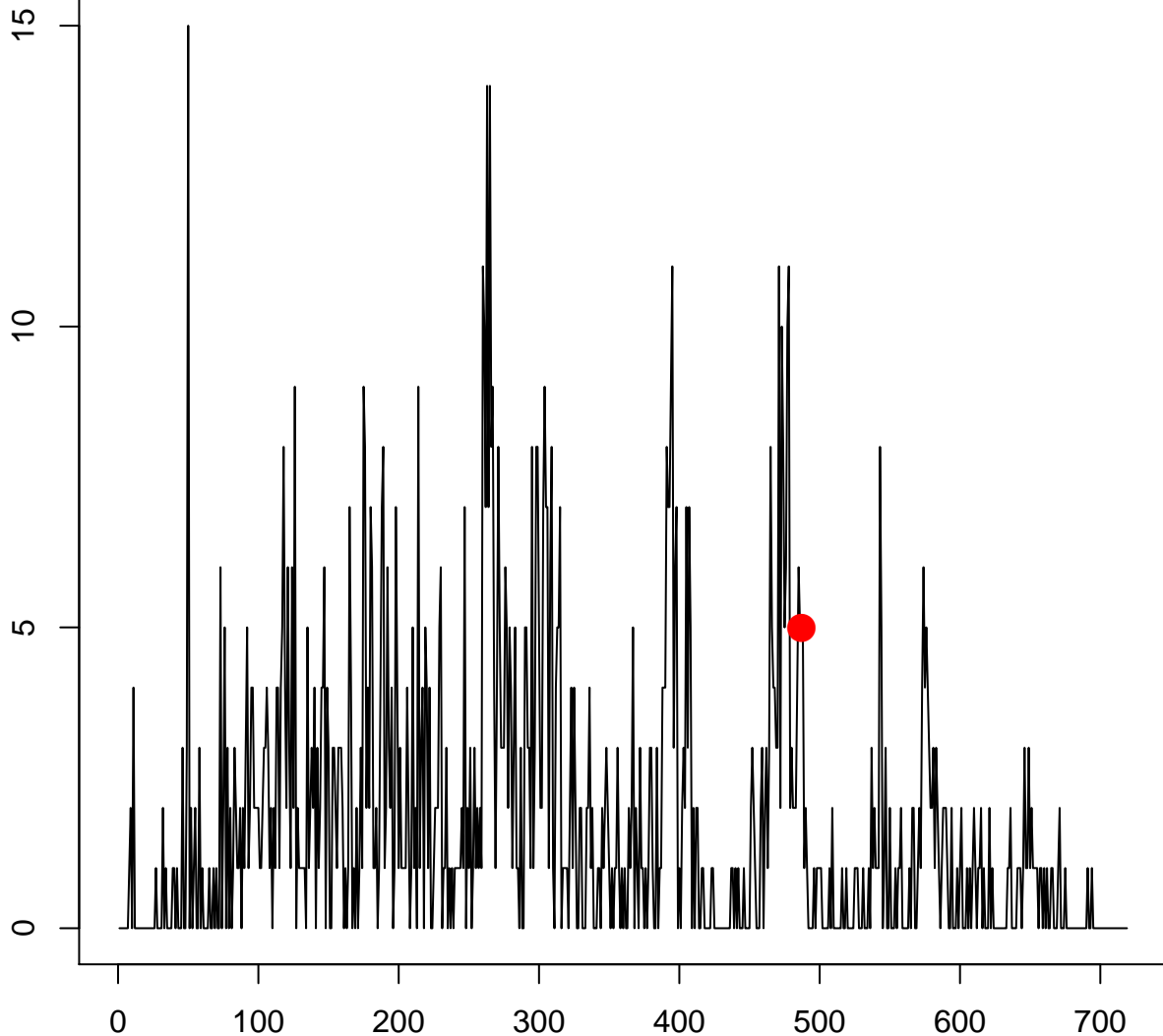

Transcript Position

Supplement: Supplementary Data 1 — Results of categories 0–2 from PARE-Seq analysis (including three subfiles:1_1, 1_2, 1_3). [file Data_Sheet_10.ZIP › GSM2230754.plot/Lsa-miR166i_Lsat_1_v5_gn_6_2981.1_487_TPlot.pdf]

**T=Lsat\_1\_v5\_gn\_6\_45641.1\_Q=Lsa-miR166i\_S=1311**

category=2\_p=0.159930217314119

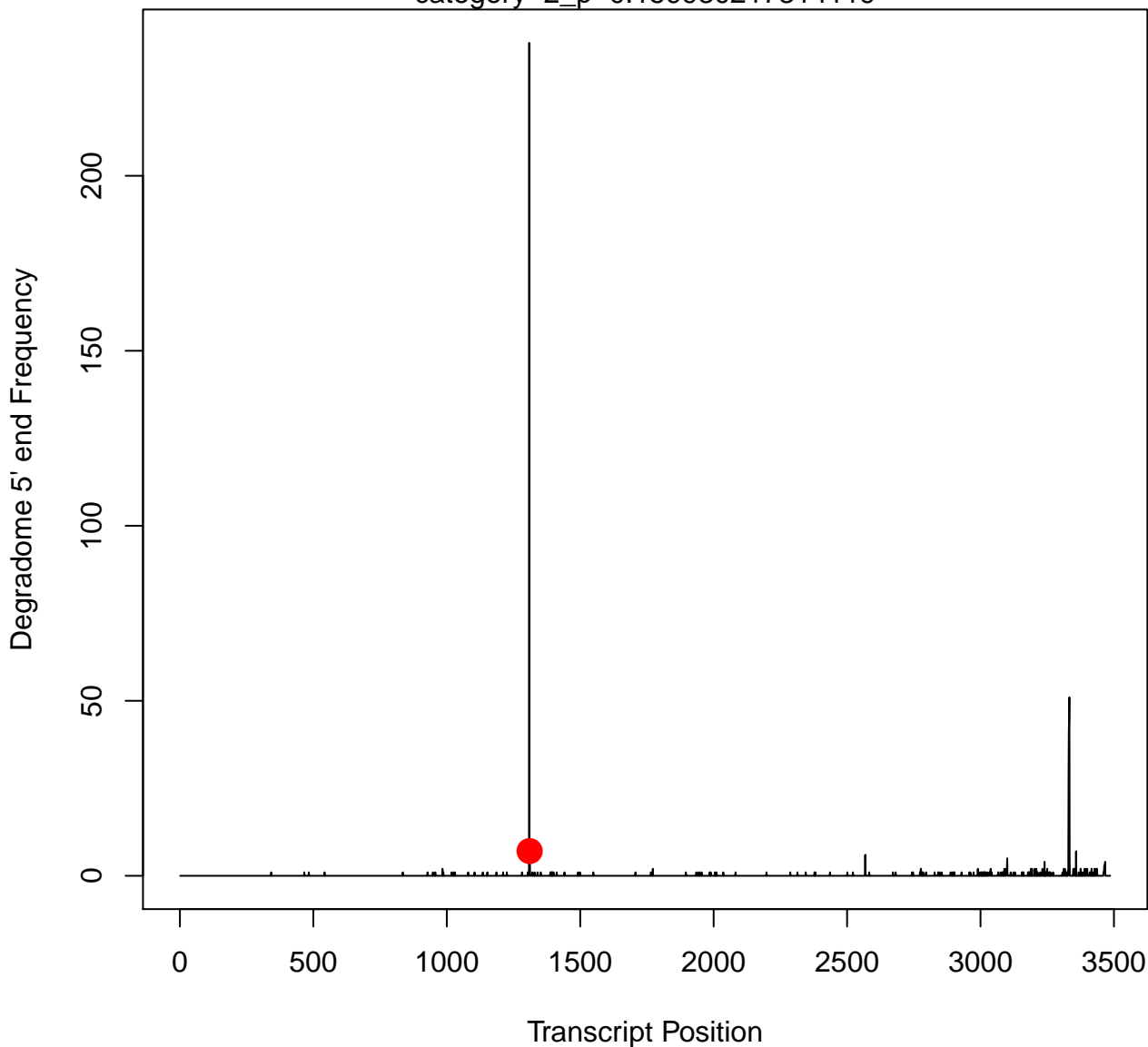

Supplement: Supplementary Data 1 — Results of categories 0–2 from PARE-Seq analysis (including three subfiles:1_1, 1_2, 1_3). [file Data_Sheet_10.ZIP › GSM2230754.plot/Lsa-miR166i_Lsat_1_v5_gn_6_45641.1_1311_TPlot.pdf]

**T=Lsat\_1\_v5\_gn\_6\_46140.1\_Q=Lsa-miR166i\_S=207**

category=2\_p=0.668362243671116

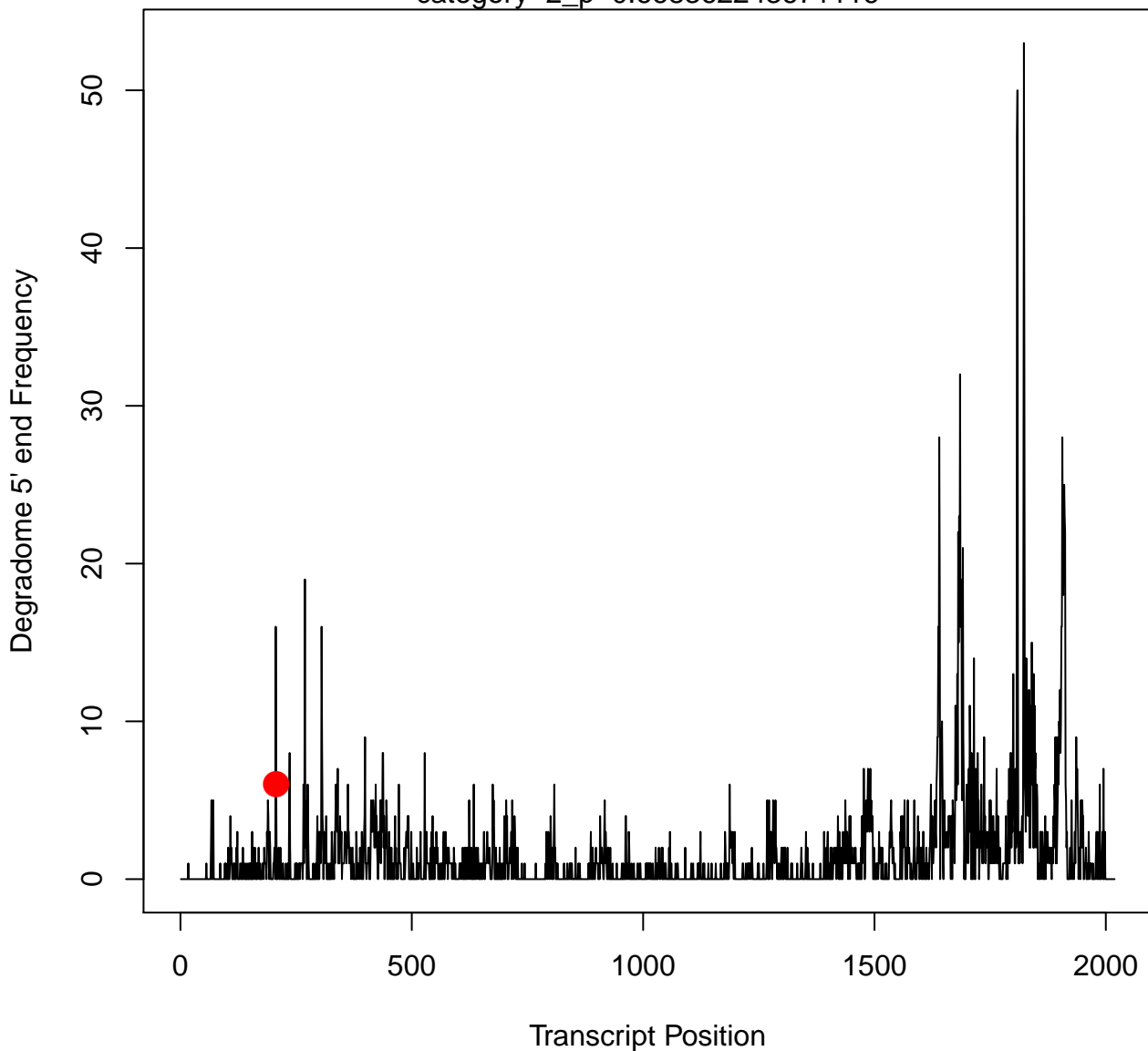

Supplement: Supplementary Data 1 — Results of categories 0–2 from PARE-Seq analysis (including three subfiles:1_1, 1_2, 1_3). [file Data_Sheet_10.ZIP › GSM2230754.plot/Lsa-miR166i_Lsat_1_v5_gn_6_46140.1_207_TPlot.pdf]

**T=Lsat\_1\_v5\_gn\_8\_60221.1\_Q=Lsa-miR166i\_S=2205**

category=2\_p=0.926763015987664

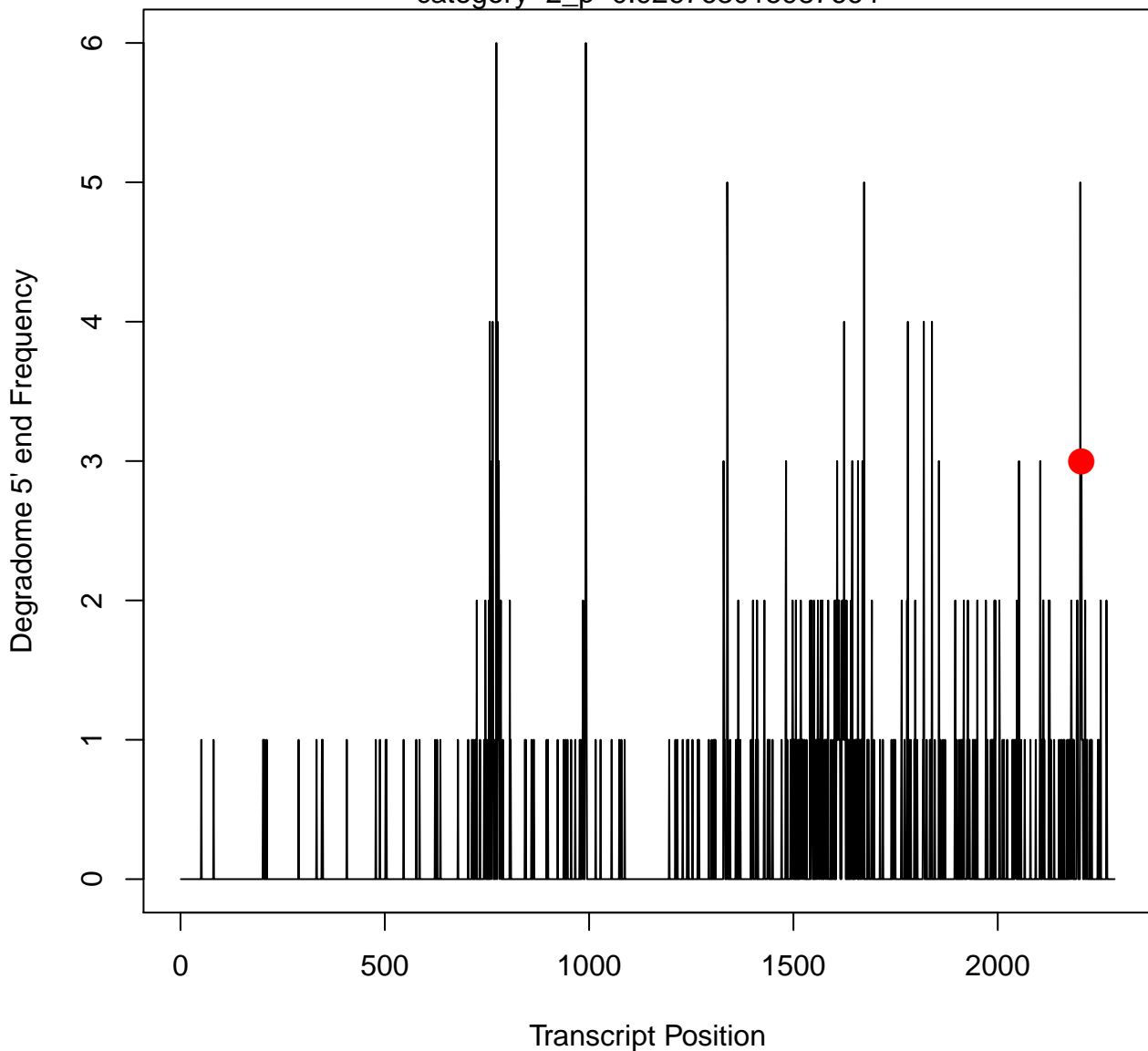

Supplement: Supplementary Data 1 — Results of categories 0–2 from PARE-Seq analysis (including three subfiles:1_1, 1_2, 1_3). [file Data_Sheet_10.ZIP › GSM2230754.plot/Lsa-miR166i_Lsat_1_v5_gn_8_60221.1_2205_TPlot.pdf]

**T=Lsat\_1\_v5\_gn\_8\_99701.1\_Q=Lsa-miR166i\_S=1628**

category=2\_p=0.951232222278289

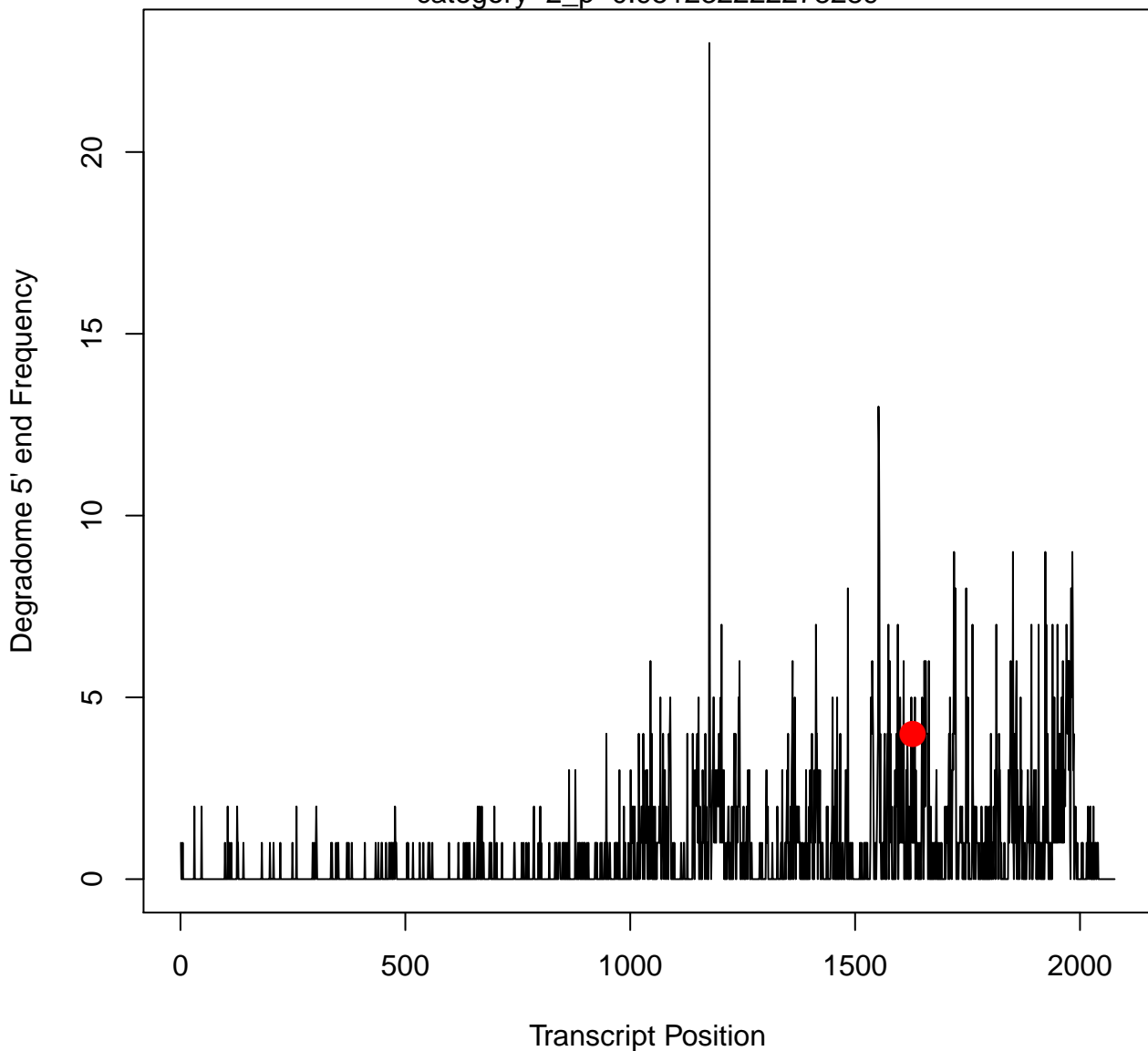

Supplement: Supplementary Data 1 — Results of categories 0–2 from PARE-Seq analysis (including three subfiles:1_1, 1_2, 1_3). [file Data_Sheet_10.ZIP › GSM2230754.plot/Lsa-miR166i_Lsat_1_v5_gn_8_99701.1_1628_TPlot.pdf]

**T=Lsat\_1\_v5\_gn\_3\_72820.1\_Q=Lsa-miR167a\_S=531**

category=2\_p=0.759060058132066

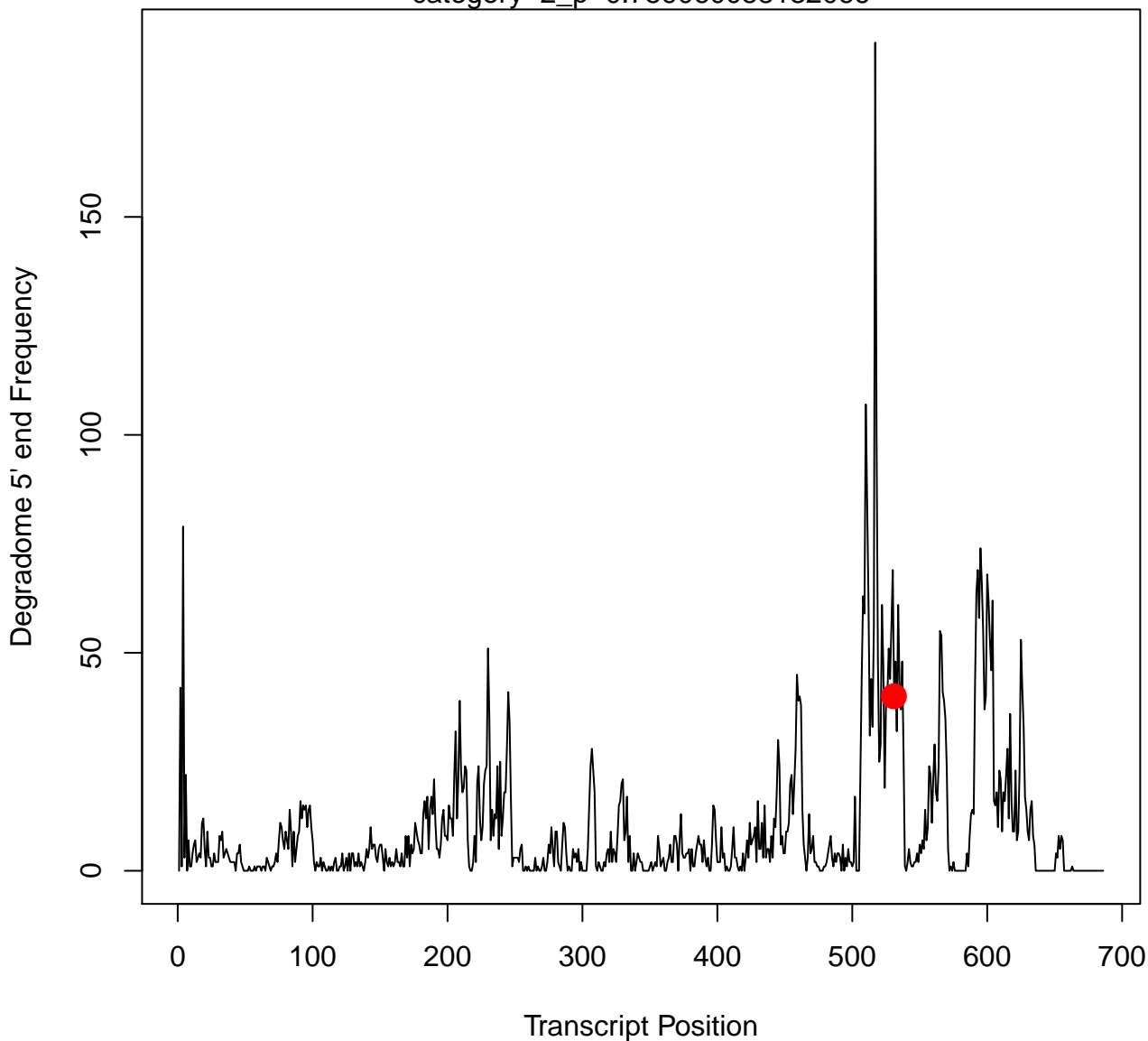

Supplement: Supplementary Data 1 — Results of categories 0–2 from PARE-Seq analysis (including three subfiles:1_1, 1_2, 1_3). [file Data_Sheet_10.ZIP › GSM2230754.plot/Lsa-miR167a_Lsat_1_v5_gn_3_72820.1_531_TPlot.pdf]

**T=Lsat\_1\_v5\_gn\_3\_68721.1\_Q=Lsa-miR167c\_S=2661**

category=2\_p=0.135172542726729

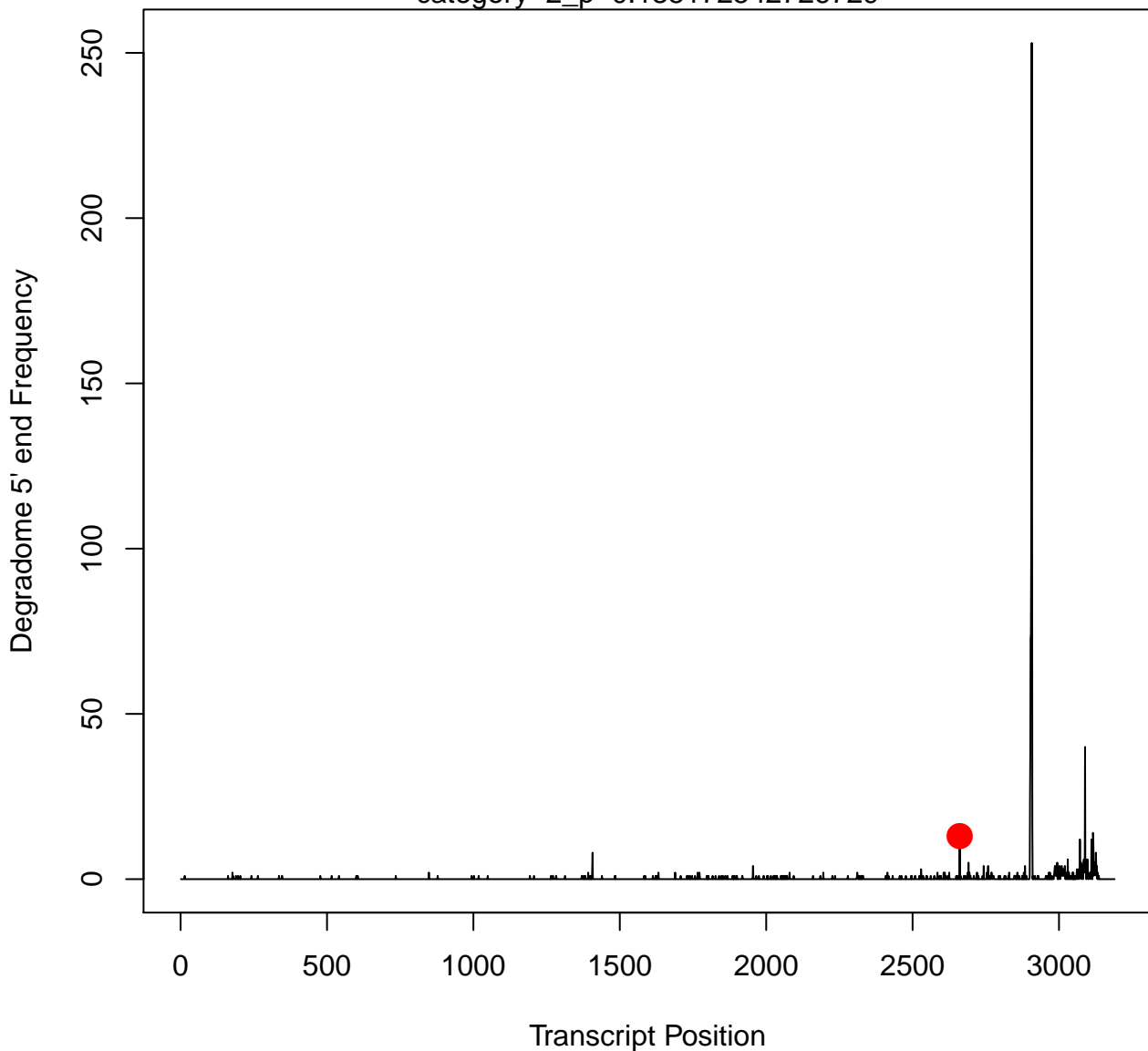

Supplement: Supplementary Data 1 — Results of categories 0–2 from PARE-Seq analysis (including three subfiles:1_1, 1_2, 1_3). [file Data_Sheet_10.ZIP › GSM2230754.plot/Lsa-miR167c_Lsat_1_v5_gn_3_68721.1_2661_TPlot.pdf]

**T=Lsat\_1\_v5\_gn\_7\_27300.1\_Q=Lsa-miR167c\_S=2921**

category=0\_p=0.00150228456680068

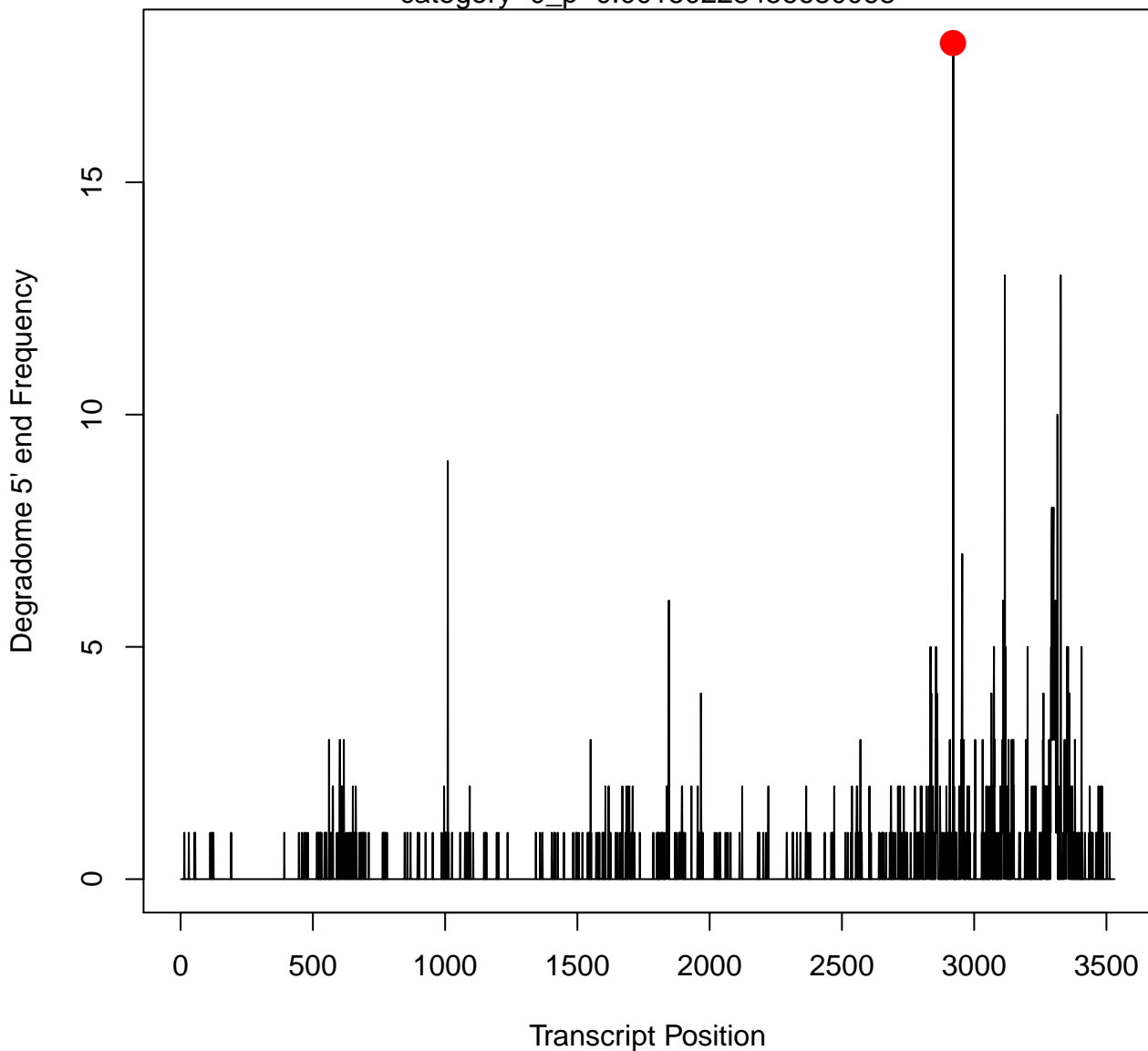

Supplement: Supplementary Data 1 — Results of categories 0–2 from PARE-Seq analysis (including three subfiles:1_1, 1_2, 1_3). [file Data_Sheet_10.ZIP › GSM2230754.plot/Lsa-miR167c_Lsat_1_v5_gn_7_27300.1_2921_TPlot.pdf]

**T=Lsat\_1\_v5\_gn\_4\_159141.1\_Q=Lsa-miR167d\_S=1744**

category=2\_p=0.993033929629704

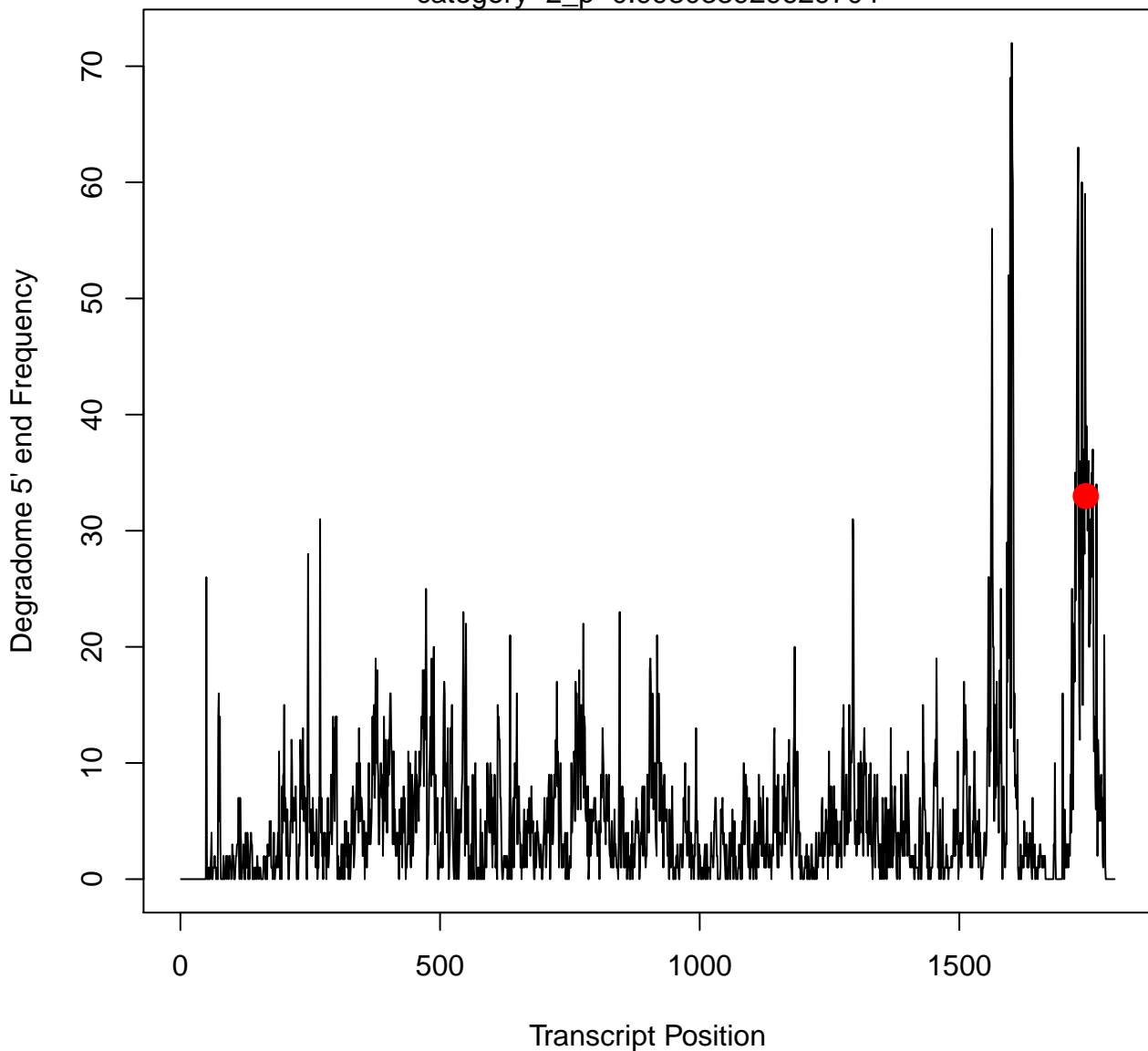

Supplement: Supplementary Data 1 — Results of categories 0–2 from PARE-Seq analysis (including three subfiles:1_1, 1_2, 1_3). [file Data_Sheet_10.ZIP › GSM2230754.plot/Lsa-miR167d_Lsat_1_v5_gn_4_159141.1_1744_TPlot.pdf]

**T=Lsat\_1\_v5\_gn\_2\_72540.1\_Q=Lsa-miR167e\_S=2777**

category=2\_p=0.135172542726729

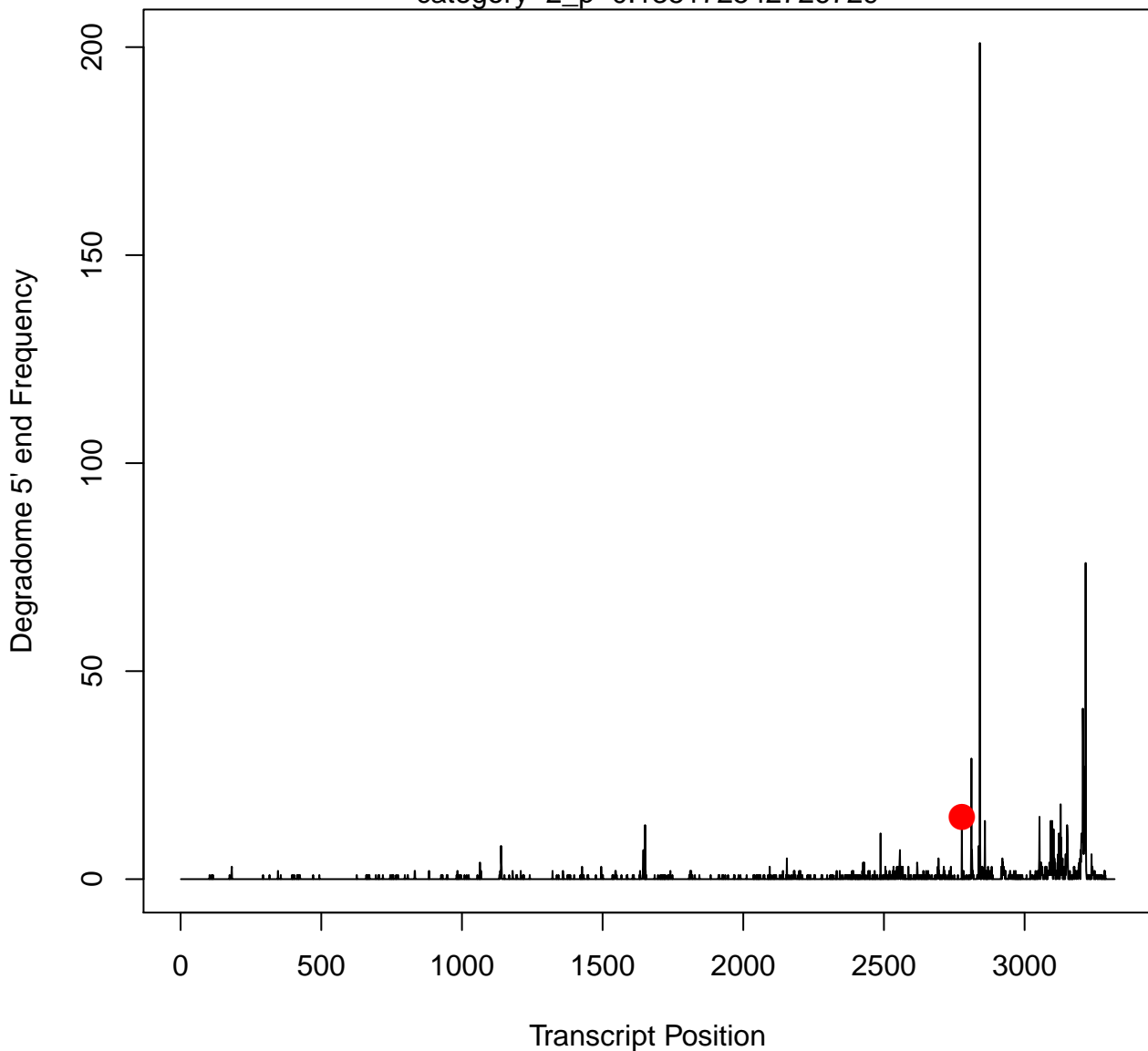

Supplement: Supplementary Data 1 — Results of categories 0–2 from PARE-Seq analysis (including three subfiles:1_1, 1_2, 1_3). [file Data_Sheet_10.ZIP › GSM2230754.plot/Lsa-miR167e_Lsat_1_v5_gn_2_72540.1_2777_TPlot.pdf]

**T=Lsat\_1\_v5\_gn\_7\_36161.1\_Q=Lsa-miR167e\_S=520**

category=2\_p=0.516220626727551

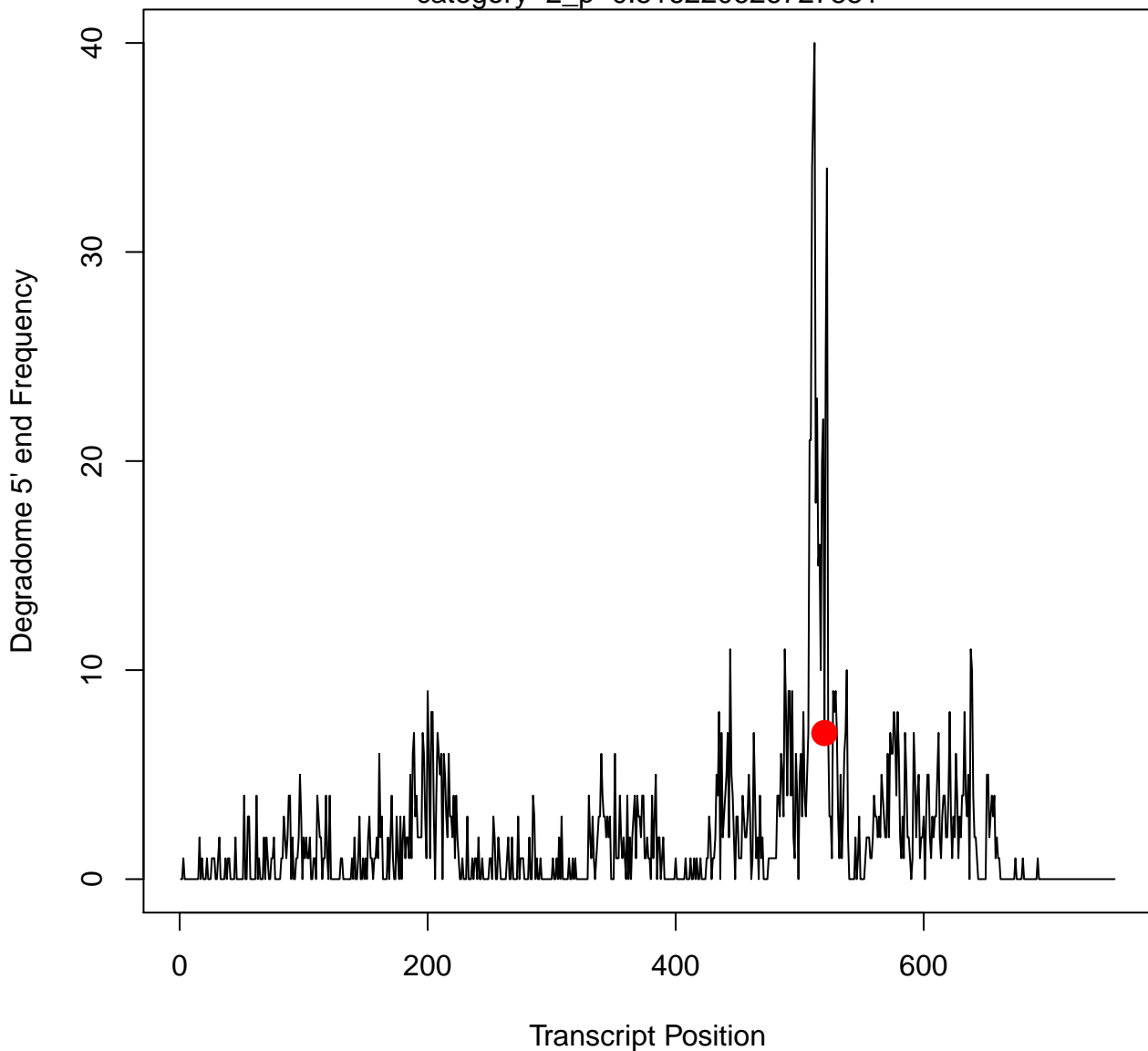

Supplement: Supplementary Data 1 — Results of categories 0–2 from PARE-Seq analysis (including three subfiles:1_1, 1_2, 1_3). [file Data_Sheet_10.ZIP › GSM2230754.plot/Lsa-miR167e_Lsat_1_v5_gn_7_36161.1_520_TPlot.pdf]

**T=Lsat\_1\_v5\_gn\_3\_35820.1\_Q=Lsa-miR168a\_S=1750**

category=2\_p=0.896223331549943

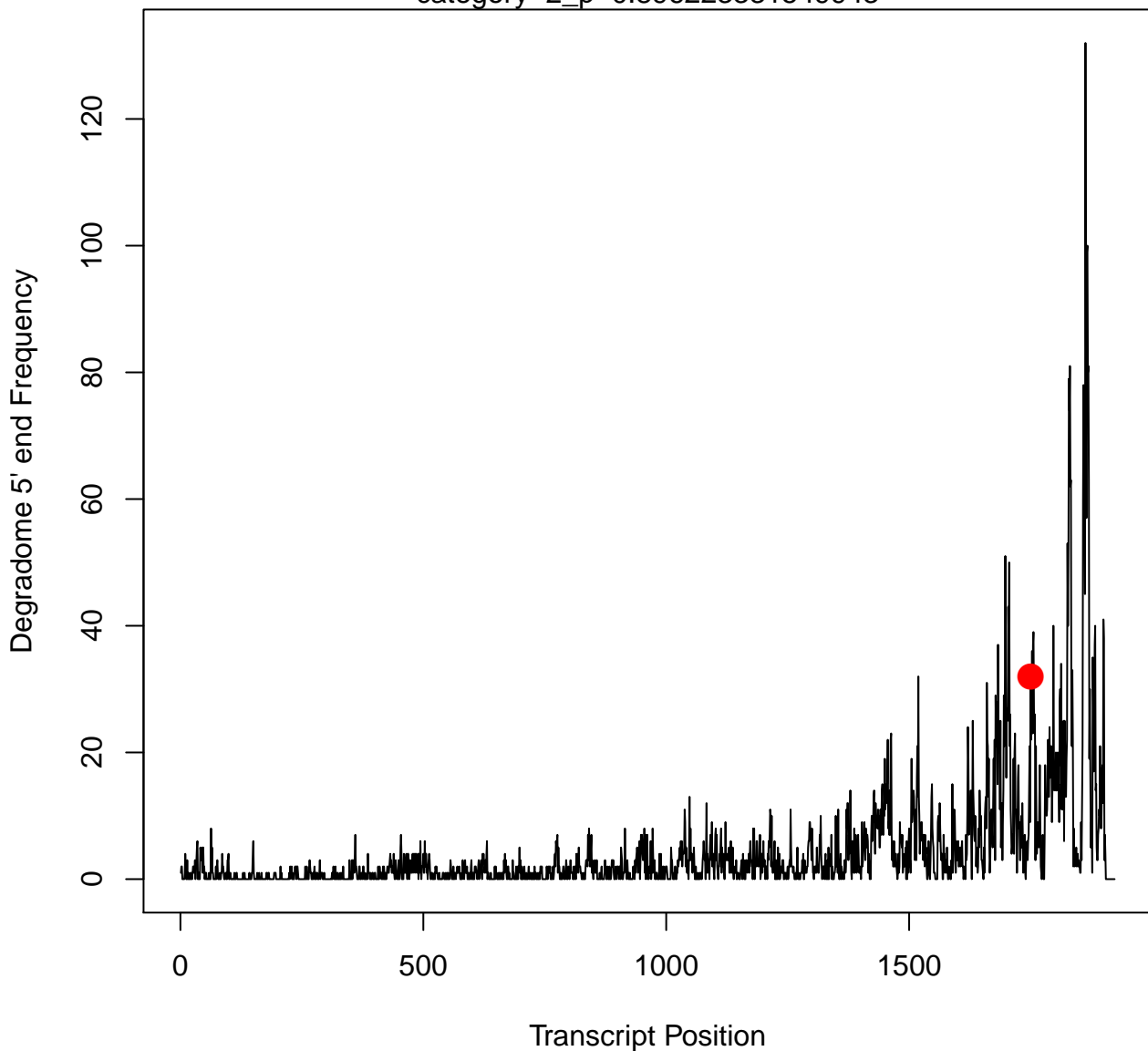

Supplement: Supplementary Data 1 — Results of categories 0–2 from PARE-Seq analysis (including three subfiles:1_1, 1_2, 1_3). [file Data_Sheet_10.ZIP › GSM2230754.plot/Lsa-miR168a_Lsat_1_v5_gn_3_35820.1_1750_TPlot.pdf]

**T=Lsat\_1\_v5\_gn\_5\_103680.1\_Q=Lsa-miR168a\_S=1484**

category=2\_p=0.834832185608514

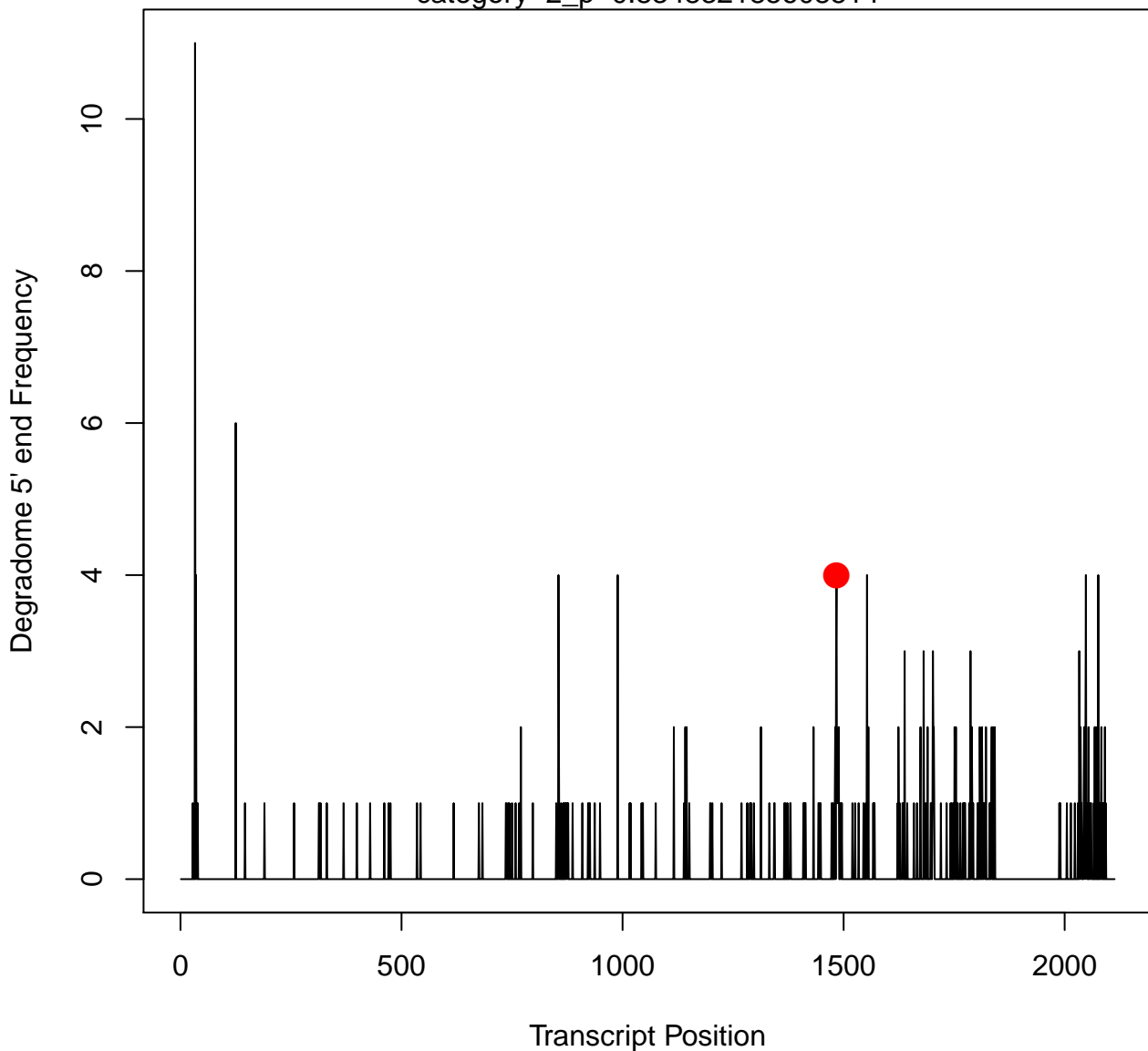

Supplement: Supplementary Data 1 — Results of categories 0–2 from PARE-Seq analysis (including three subfiles:1_1, 1_2, 1_3). [file Data_Sheet_10.ZIP › GSM2230754.plot/Lsa-miR168a_Lsat_1_v5_gn_5_103680.1_1484_TPlot.pdf]

**T=Lsat\_1\_v5\_gn\_8\_261.1\_Q=Lsa-miR168a\_S=1295**

category=0\_p=0.00037578290783058

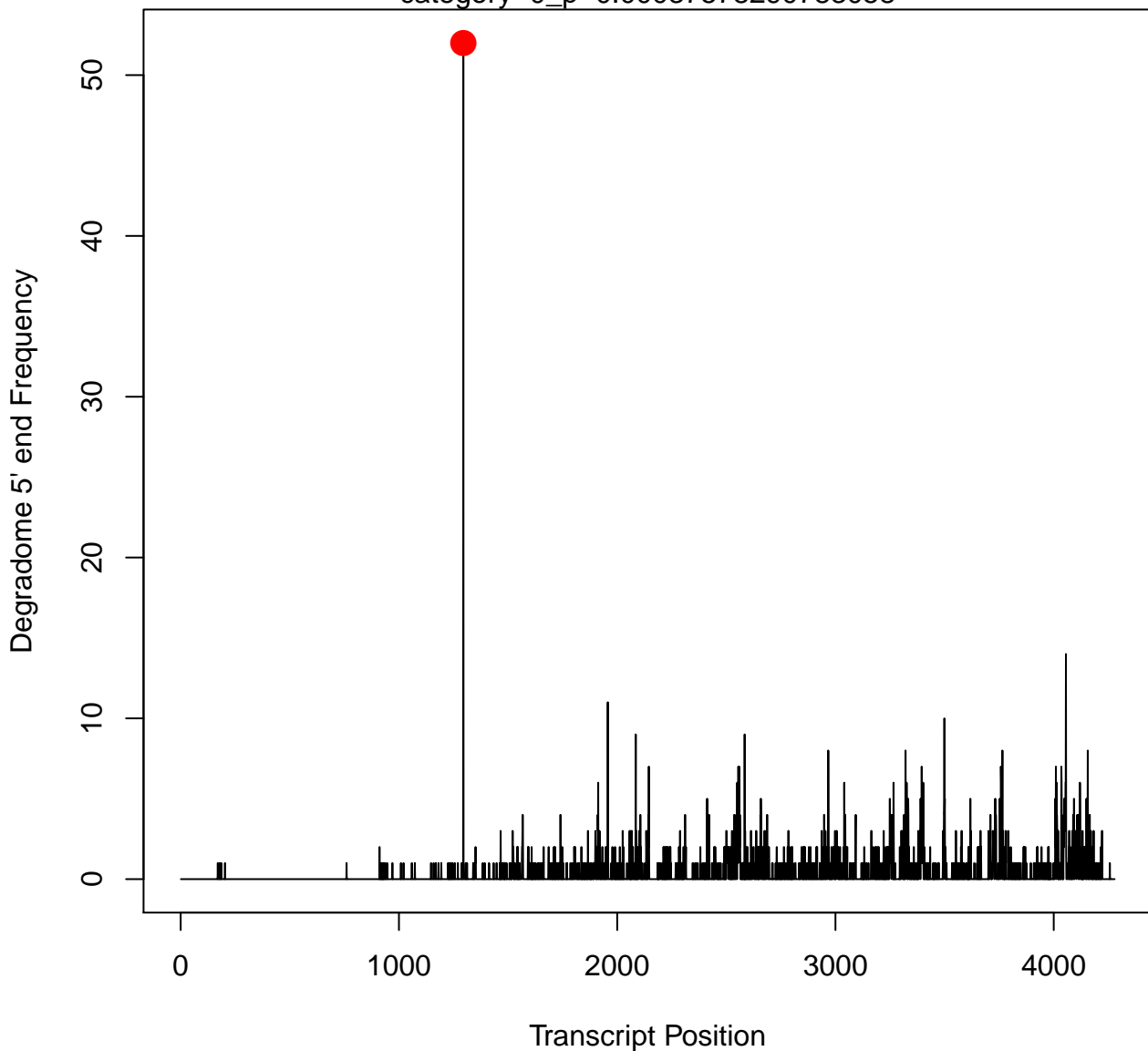

Supplement: Supplementary Data 1 — Results of categories 0–2 from PARE-Seq analysis (including three subfiles:1_1, 1_2, 1_3). [file Data_Sheet_10.ZIP › GSM2230754.plot/Lsa-miR168a_Lsat_1_v5_gn_8_261.1_1295_TPlot.pdf]

**T=Lsat\_1\_v5\_gn\_8\_85500.1\_Q=Lsa-miR168a\_S=307**

category=2\_p=0.501963177474613

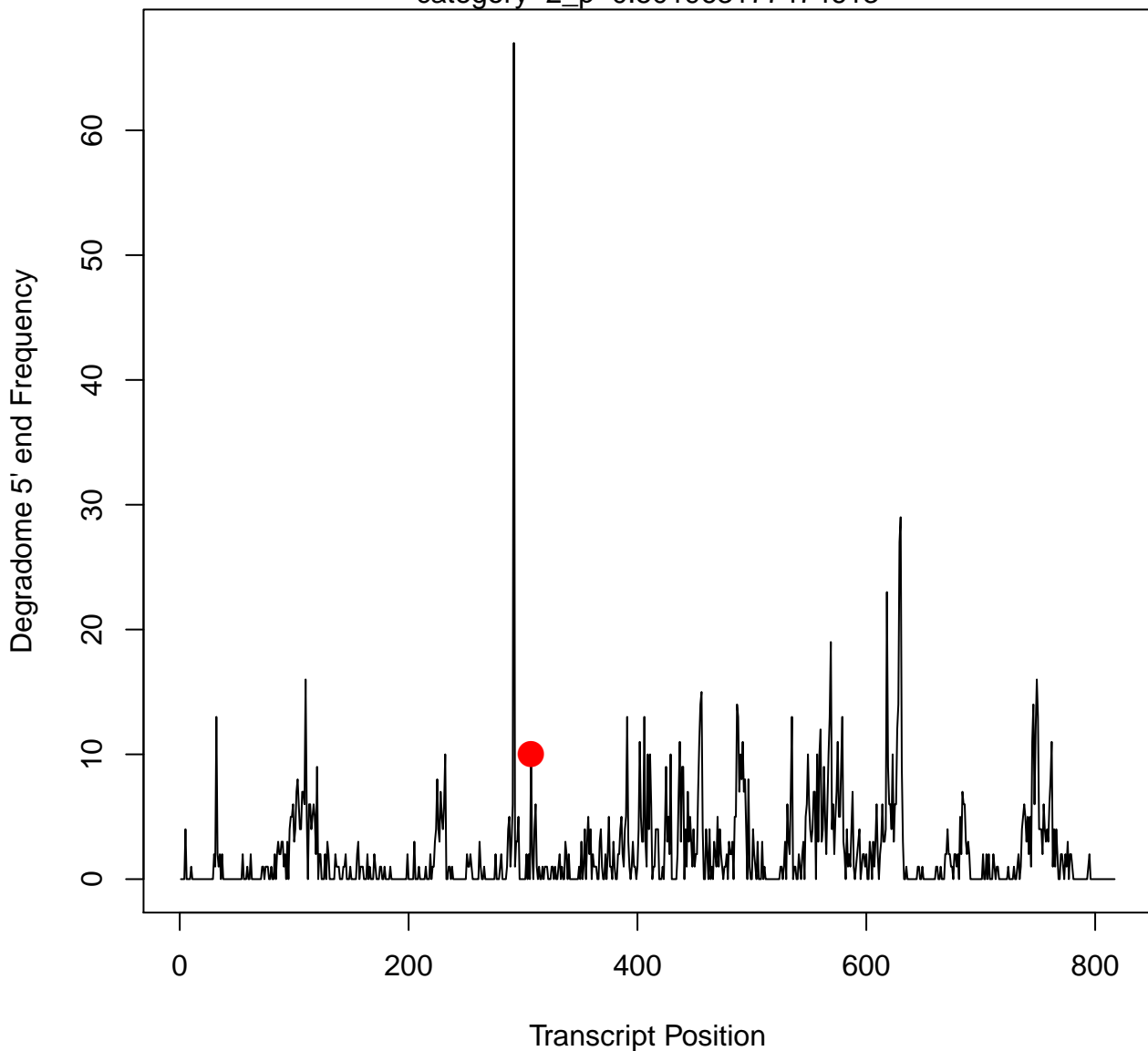

Supplement: Supplementary Data 1 — Results of categories 0–2 from PARE-Seq analysis (including three subfiles:1_1, 1_2, 1_3). [file Data_Sheet_10.ZIP › GSM2230754.plot/Lsa-miR168a_Lsat_1_v5_gn_8_85500.1_307_TPlot.pdf]

**T=Lsat\_1\_v5\_gn\_9\_103440.1\_Q=Lsa-miR168a\_S=1363**

category=2\_p=0.648526828297634

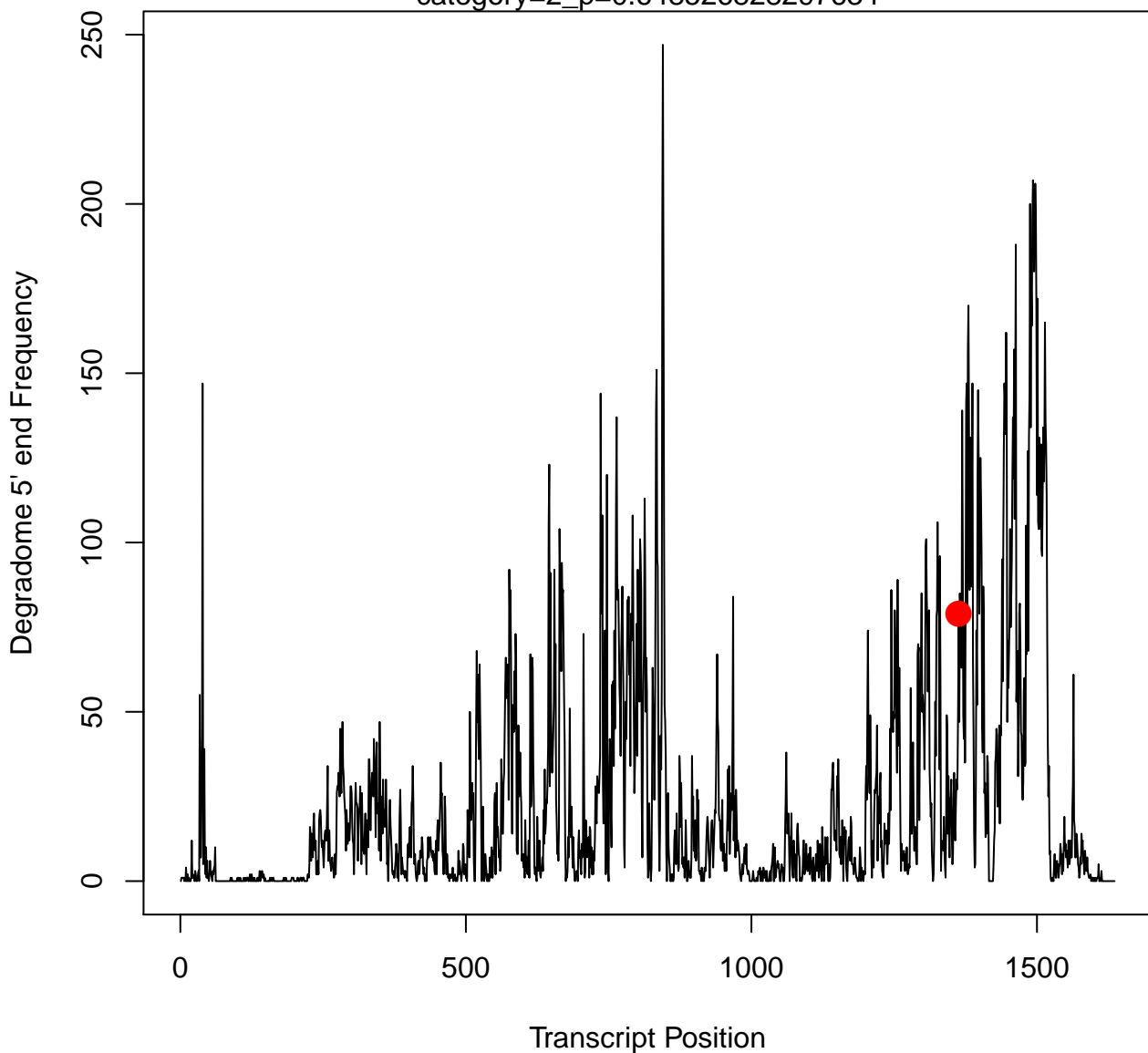

Supplement: Supplementary Data 1 — Results of categories 0–2 from PARE-Seq analysis (including three subfiles:1_1, 1_2, 1_3). [file Data_Sheet_10.ZIP › GSM2230754.plot/Lsa-miR168a_Lsat_1_v5_gn_9_103440.1_1363_TPlot.pdf]

**T=Lsat\_1\_v5\_gn\_2\_19760.1\_Q=Lsa-miR168b\_S=1492**

category=0\_p=0.00037578290783058

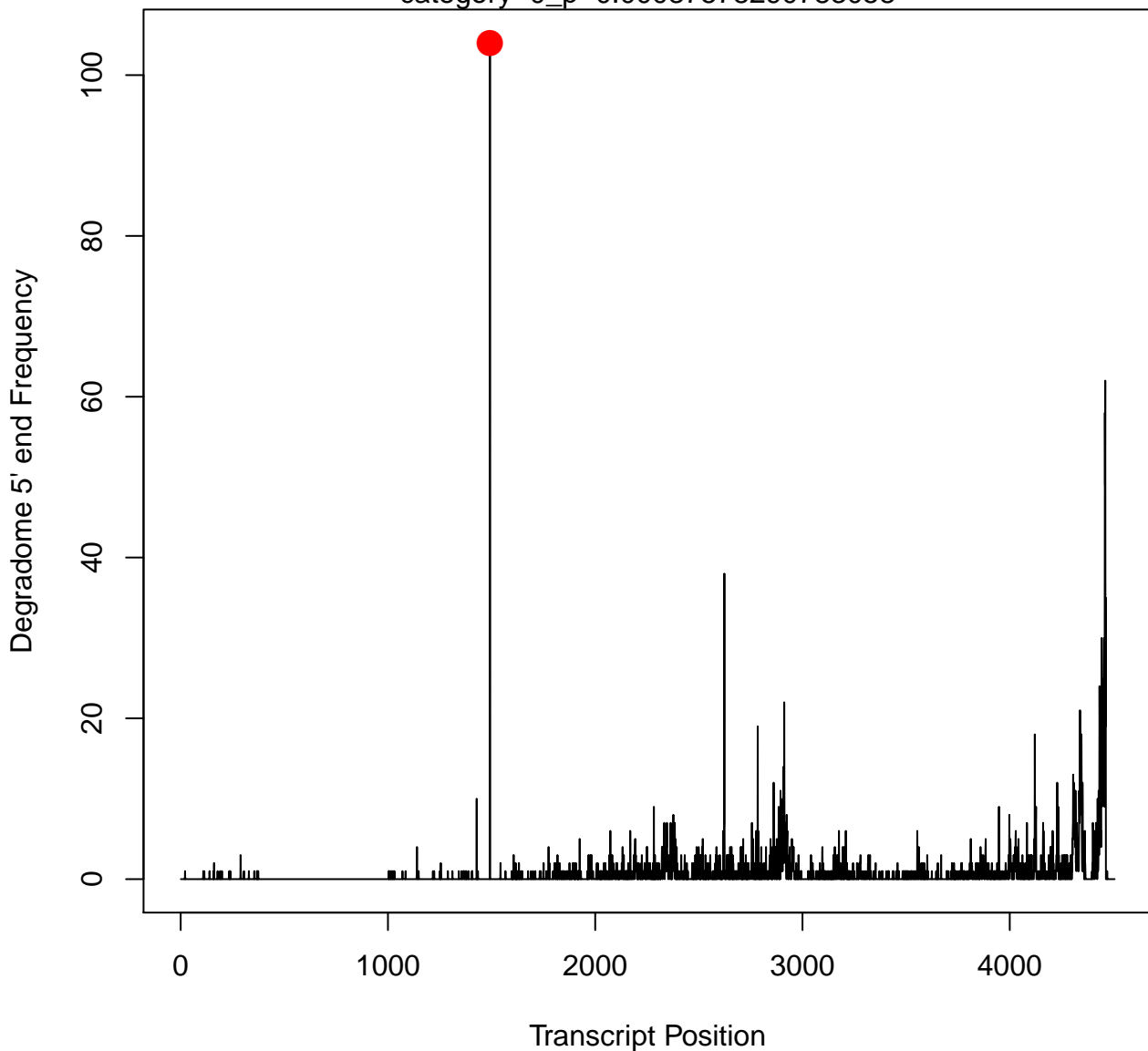

Supplement: Supplementary Data 1 — Results of categories 0–2 from PARE-Seq analysis (including three subfiles:1_1, 1_2, 1_3). [file Data_Sheet_10.ZIP › GSM2230754.plot/Lsa-miR168b_Lsat_1_v5_gn_2_19760.1_1492_TPlot.pdf]

**T=Lsat\_1\_v5\_gn\_8\_88620.1\_Q=Lsa-miR168b\_S=991**

category=2\_p=0.389676353965506

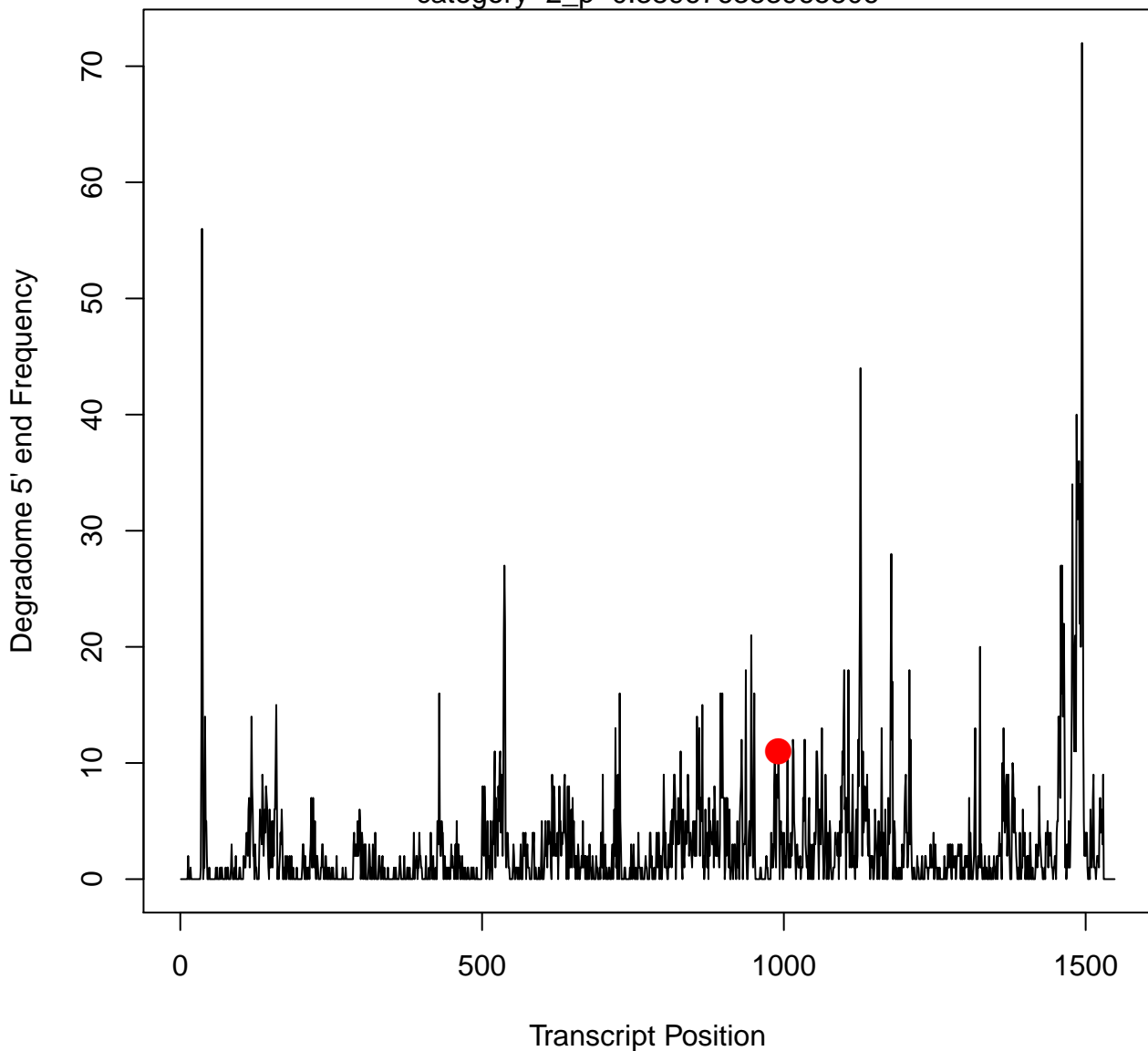

Supplement: Supplementary Data 1 — Results of categories 0–2 from PARE-Seq analysis (including three subfiles:1_1, 1_2, 1_3). [file Data_Sheet_10.ZIP › GSM2230754.plot/Lsa-miR168b_Lsat_1_v5_gn_8_88620.1_991_TPlot.pdf]

**T=Lsat\_1\_v5\_gn\_1\_37160.1\_Q=Lsa-miR169a\_S=2396**

category=2\_p=0.0286272994447346

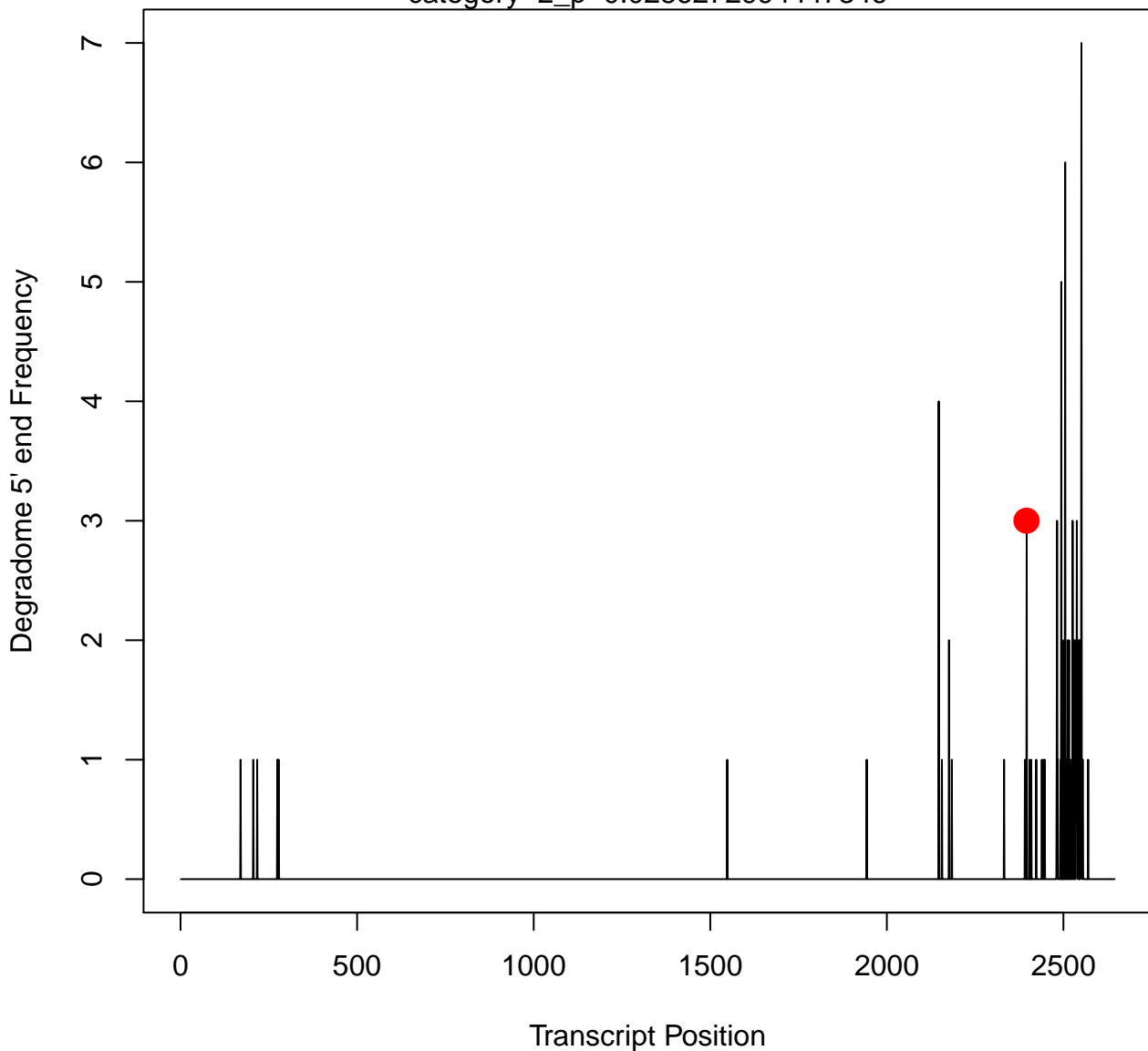

Supplement: Supplementary Data 1 — Results of categories 0–2 from PARE-Seq analysis (including three subfiles:1_1, 1_2, 1_3). [file Data_Sheet_10.ZIP › GSM2230754.plot/Lsa-miR169a_Lsat_1_v5_gn_1_37160.1_2396_TPlot.pdf]

**T=Lsat\_1\_v5\_gn\_3\_36300.1\_Q=Lsa-miR169a\_S=1467**

category=2\_p=0.721401142110361

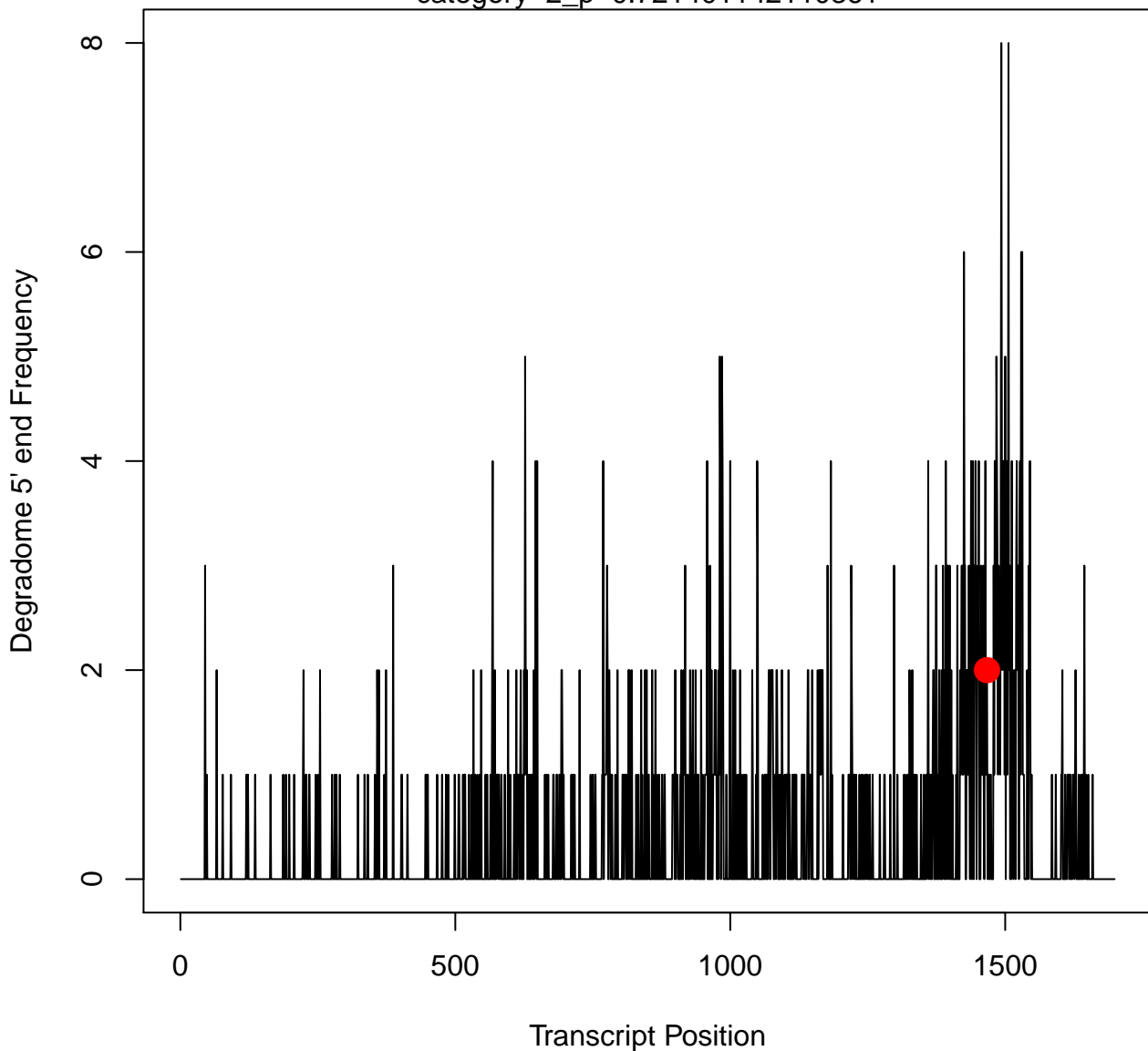

Supplement: Supplementary Data 1 — Results of categories 0–2 from PARE-Seq analysis (including three subfiles:1_1, 1_2, 1_3). [file Data_Sheet_10.ZIP › GSM2230754.plot/Lsa-miR169a_Lsat_1_v5_gn_3_36300.1_1467_TPlot.pdf]

**T=Lsat\_1\_v5\_gn\_7\_34841.1\_Q=Lsa-miR169a\_S=986**

category=0\_p=0.00112692513817547

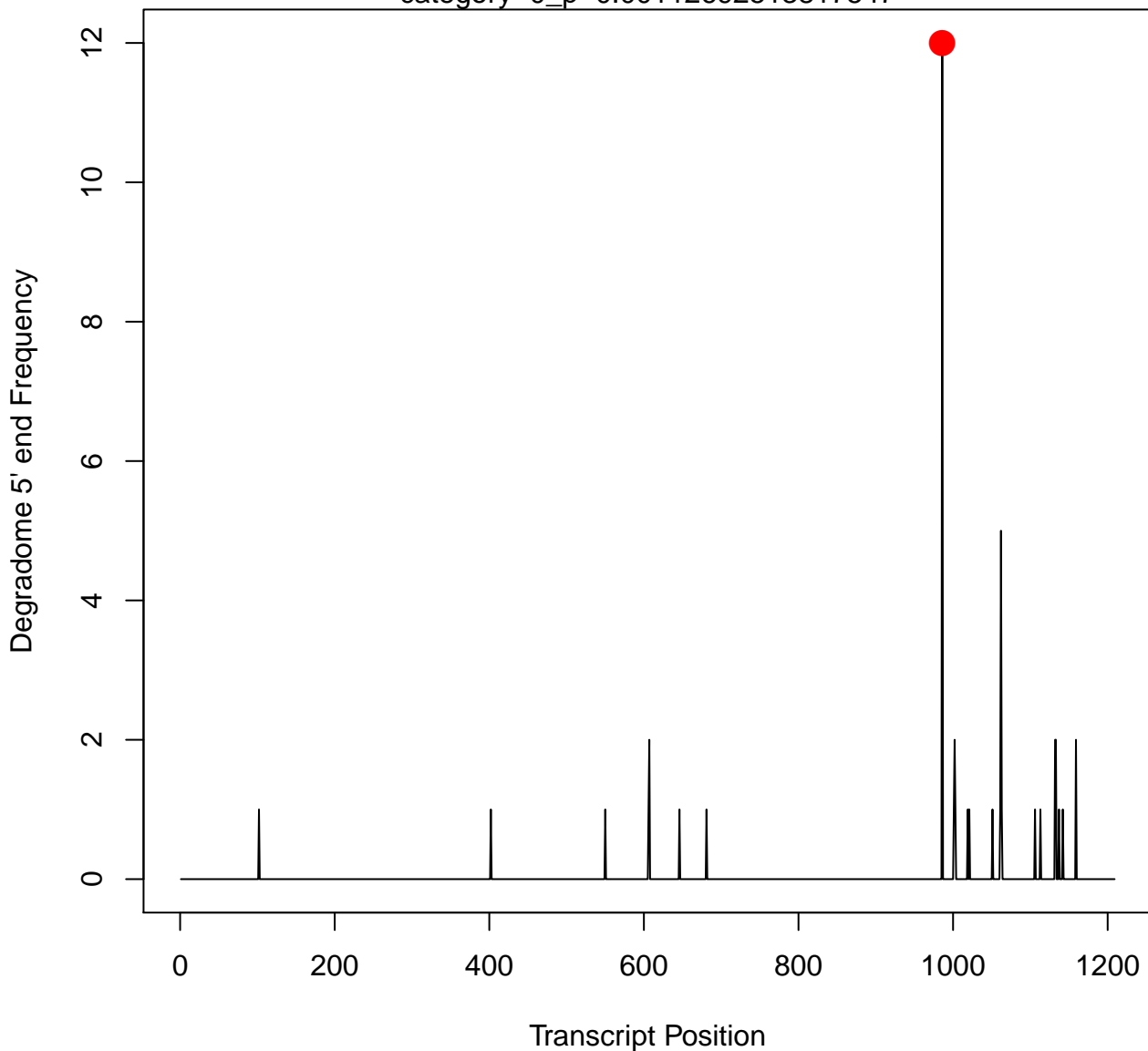

Supplement: Supplementary Data 1 — Results of categories 0–2 from PARE-Seq analysis (including three subfiles:1_1, 1_2, 1_3). [file Data_Sheet_10.ZIP › GSM2230754.plot/Lsa-miR169a_Lsat_1_v5_gn_7_34841.1_986_TPlot.pdf]

**T=Lsat\_1\_v5\_gn\_4\_134101.1\_Q=Lsa-miR169b\_S=2182**

category=2\_p=0.543522752723408

Degradome 5' end Frequency

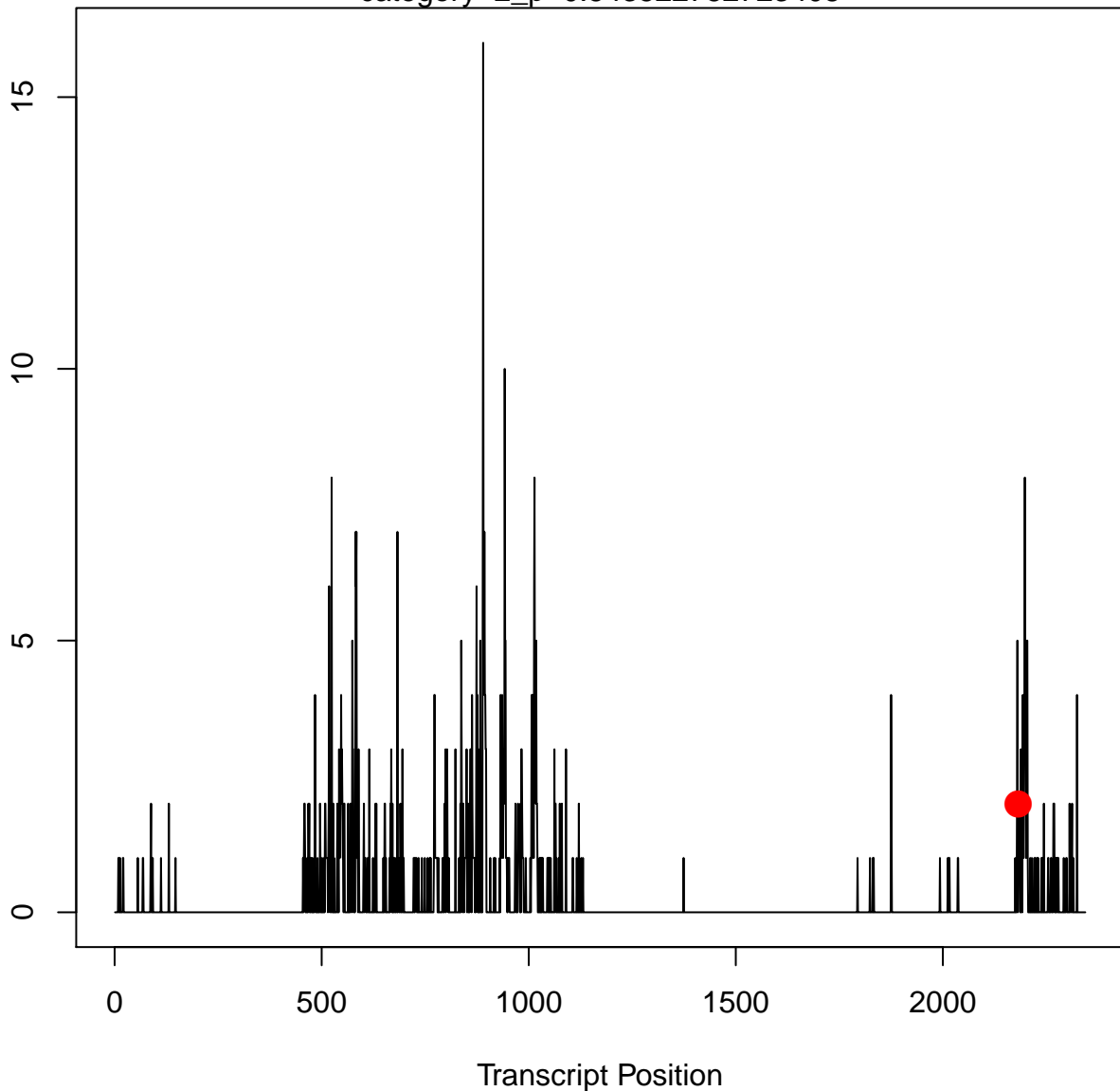

Supplement: Supplementary Data 1 — Results of categories 0–2 from PARE-Seq analysis (including three subfiles:1_1, 1_2, 1_3). [file Data_Sheet_10.ZIP › GSM2230754.plot/Lsa-miR169b_Lsat_1_v5_gn_4_134101.1_2182_TPlot.pdf]

**T=Lsat\_1\_v5\_gn\_4\_15920.1\_Q=Lsa-miR169b\_S=803**

category=2\_p=0.917740346537001

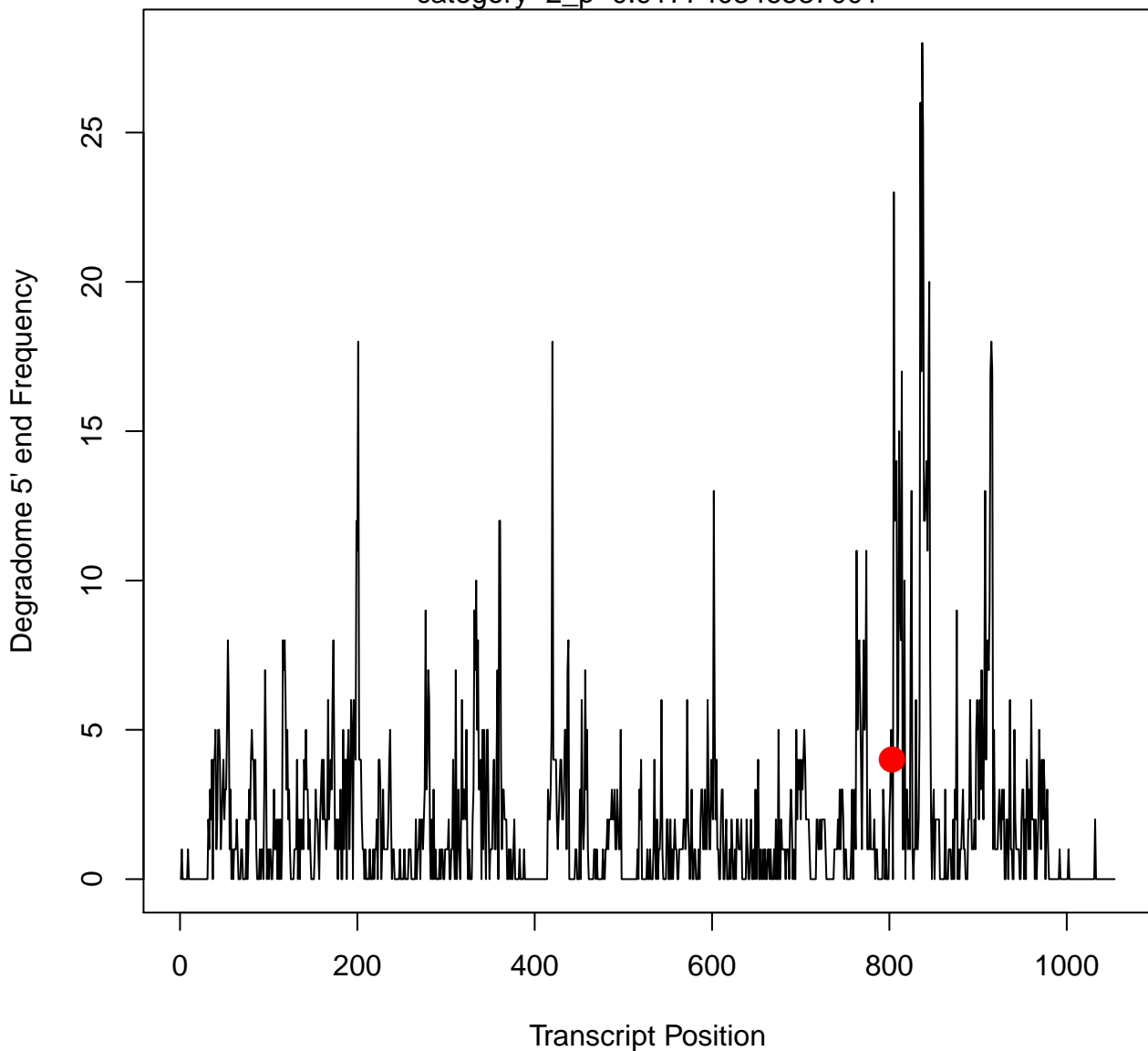

Supplement: Supplementary Data 1 — Results of categories 0–2 from PARE-Seq analysis (including three subfiles:1_1, 1_2, 1_3). [file Data_Sheet_10.ZIP › GSM2230754.plot/Lsa-miR169b_Lsat_1_v5_gn_4_15920.1_803_TPlot.pdf]

**T=Lsat\_1\_v5\_gn\_8\_111520.1\_Q=Lsa-miR169g\_S=266**

category=2\_p=0.89919417731304

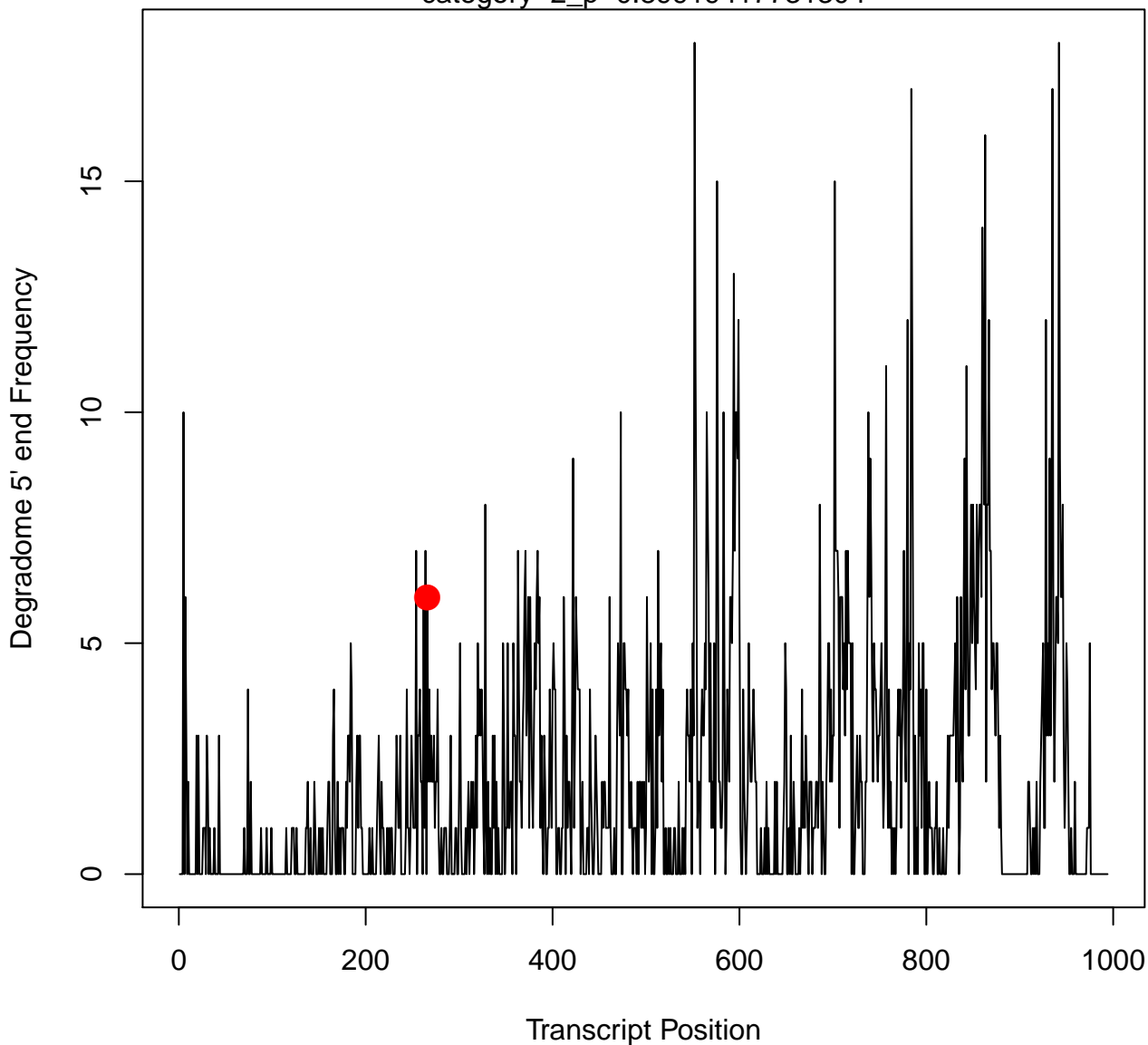

Supplement: Supplementary Data 1 — Results of categories 0–2 from PARE-Seq analysis (including three subfiles:1_1, 1_2, 1_3). [file Data_Sheet_10.ZIP › GSM2230754.plot/Lsa-miR169g_Lsat_1_v5_gn_8_111520.1_266_TPlot.pdf]

**T=Lsat\_1\_v5\_gn\_7\_116120.1\_Q=Lsa-miR169h\_S=323**

category=2\_p=0.987180116299196

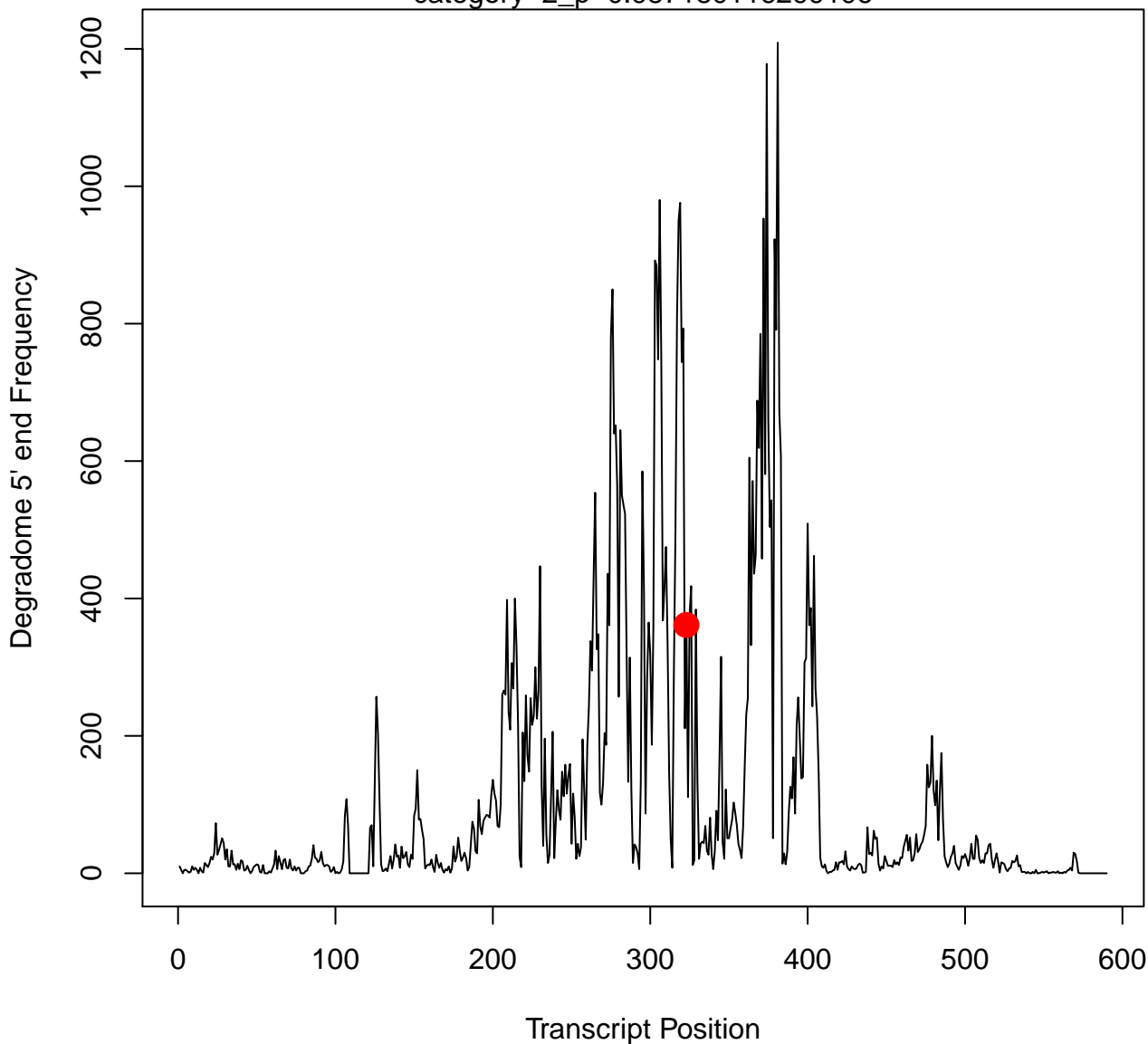

Supplement: Supplementary Data 1 — Results of categories 0–2 from PARE-Seq analysis (including three subfiles:1_1, 1_2, 1_3). [file Data_Sheet_10.ZIP › GSM2230754.plot/Lsa-miR169h_Lsat_1_v5_gn_7_116120.1_323_TPlot.pdf]

**T=Lsat\_1\_v5\_gn\_3\_90900.1\_Q=Lsa-miR169i\_S=642**

category=2\_p=0.785487612363305

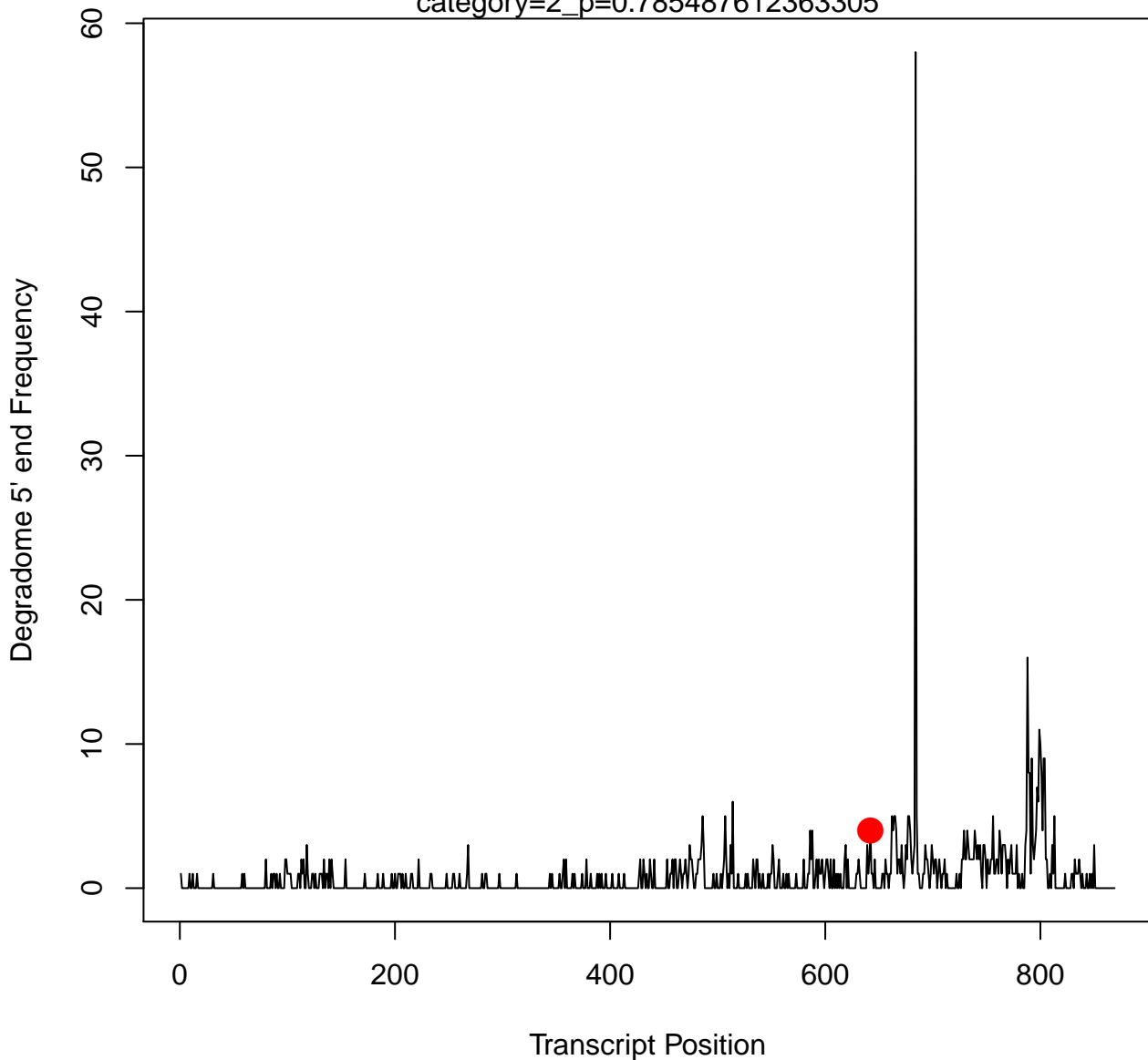

Supplement: Supplementary Data 1 — Results of categories 0–2 from PARE-Seq analysis (including three subfiles:1_1, 1_2, 1_3). [file Data_Sheet_10.ZIP › GSM2230754.plot/Lsa-miR169i_Lsat_1_v5_gn_3_90900.1_642_TPlot.pdf]

**T=Lsat\_1\_v5\_gn\_6\_47121.1\_Q=Lsa-miR169i\_S=1633**

category=0\_p=0.0045000865140874

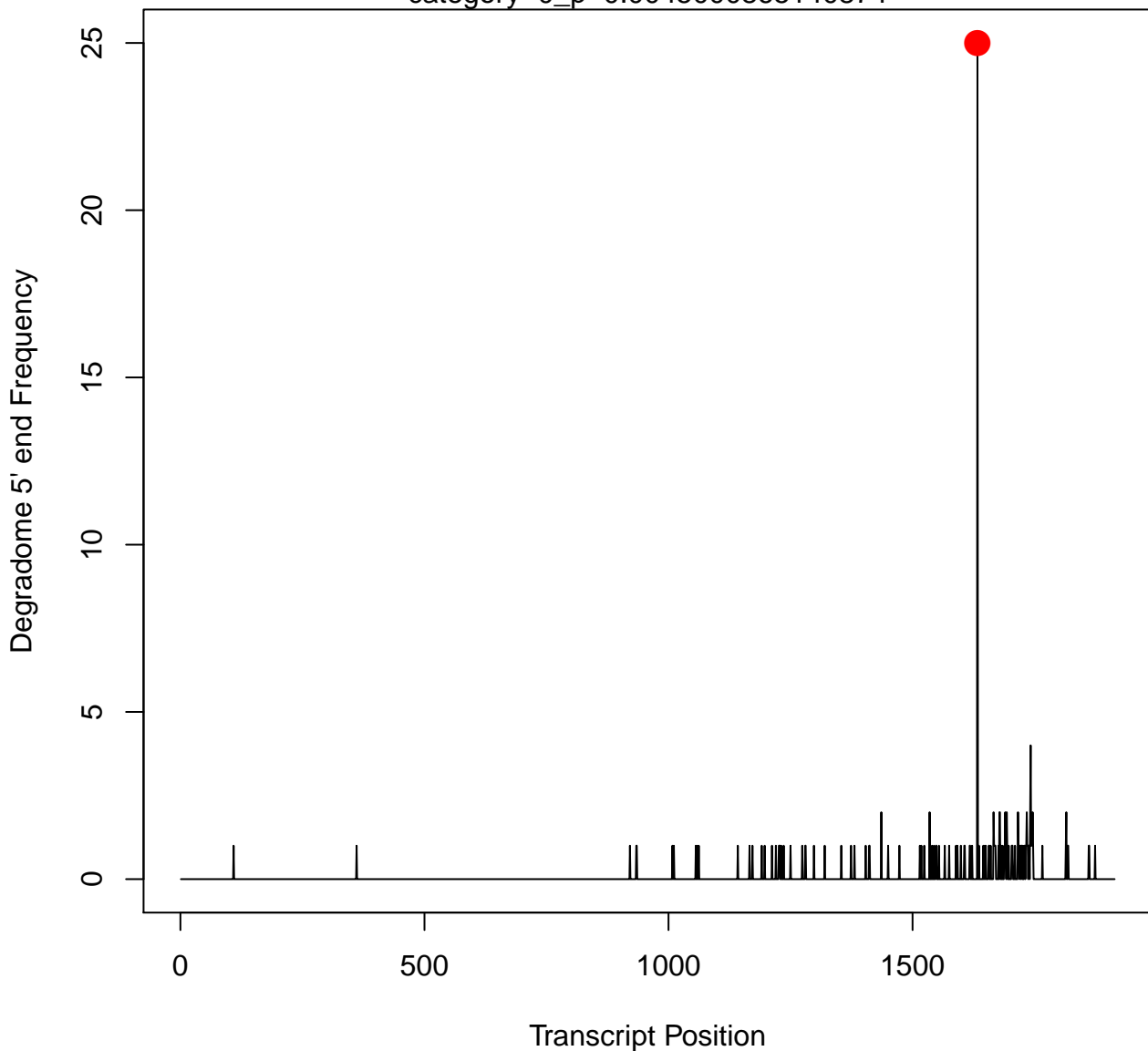

Supplement: Supplementary Data 1 — Results of categories 0–2 from PARE-Seq analysis (including three subfiles:1_1, 1_2, 1_3). [file Data_Sheet_10.ZIP › GSM2230754.plot/Lsa-miR169i_Lsat_1_v5_gn_6_47121.1_1633_TPlot.pdf]

**T=Lsat\_1\_v5\_gn\_1\_27961.1\_Q=Lsa-miR171a\_S=2558**

category=2\_p=0.964569457774148

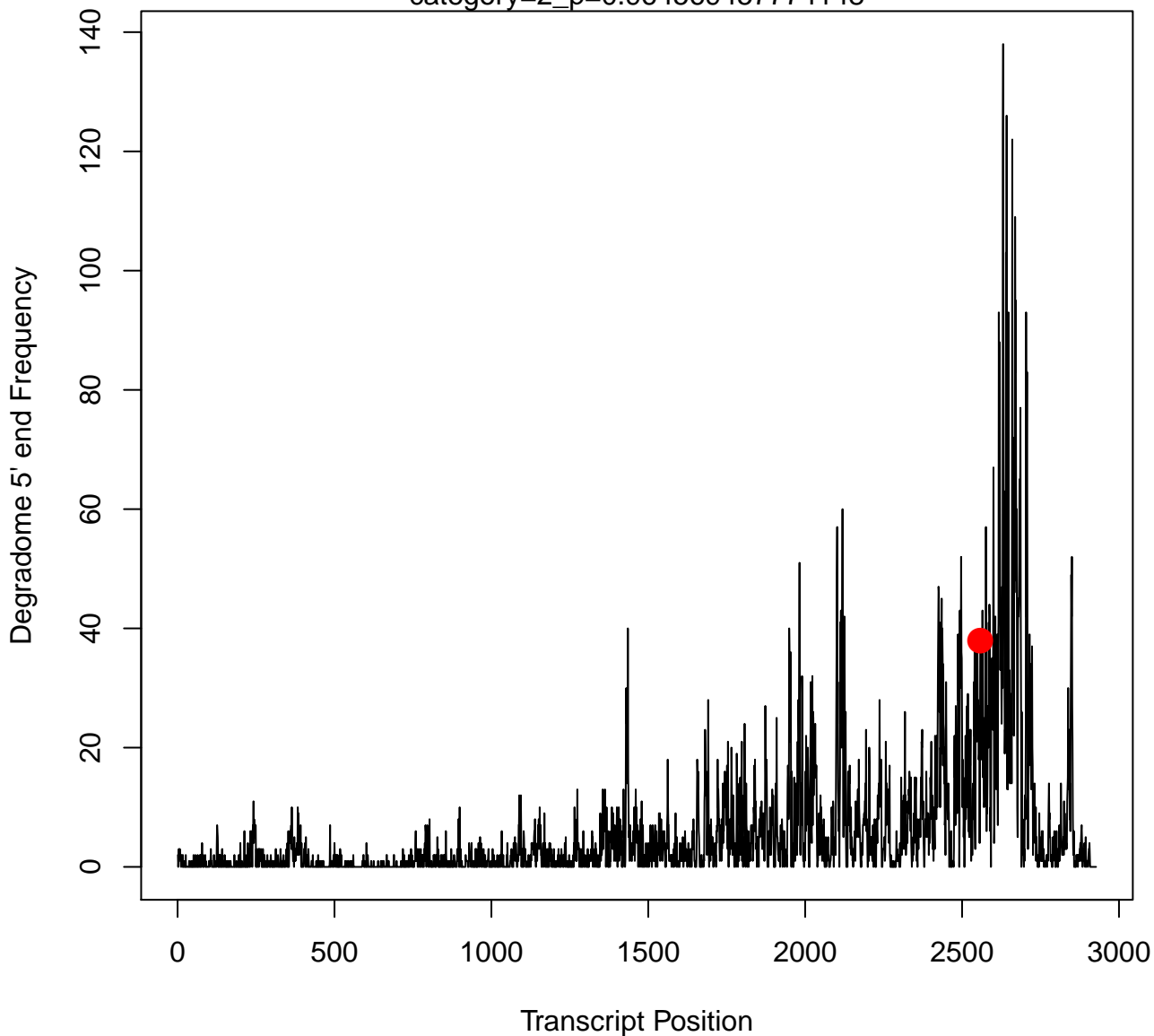

Supplement: Supplementary Data 1 — Results of categories 0–2 from PARE-Seq analysis (including three subfiles:1_1, 1_2, 1_3). [file Data_Sheet_10.ZIP › GSM2230754.plot/Lsa-miR171a_Lsat_1_v5_gn_1_27961.1_2558_TPlot.pdf]

**T=Lsat\_1\_v5\_gn\_3\_128721.1\_Q=Lsa-miR171a\_S=47**

category=0\_p=0.000751424602867257

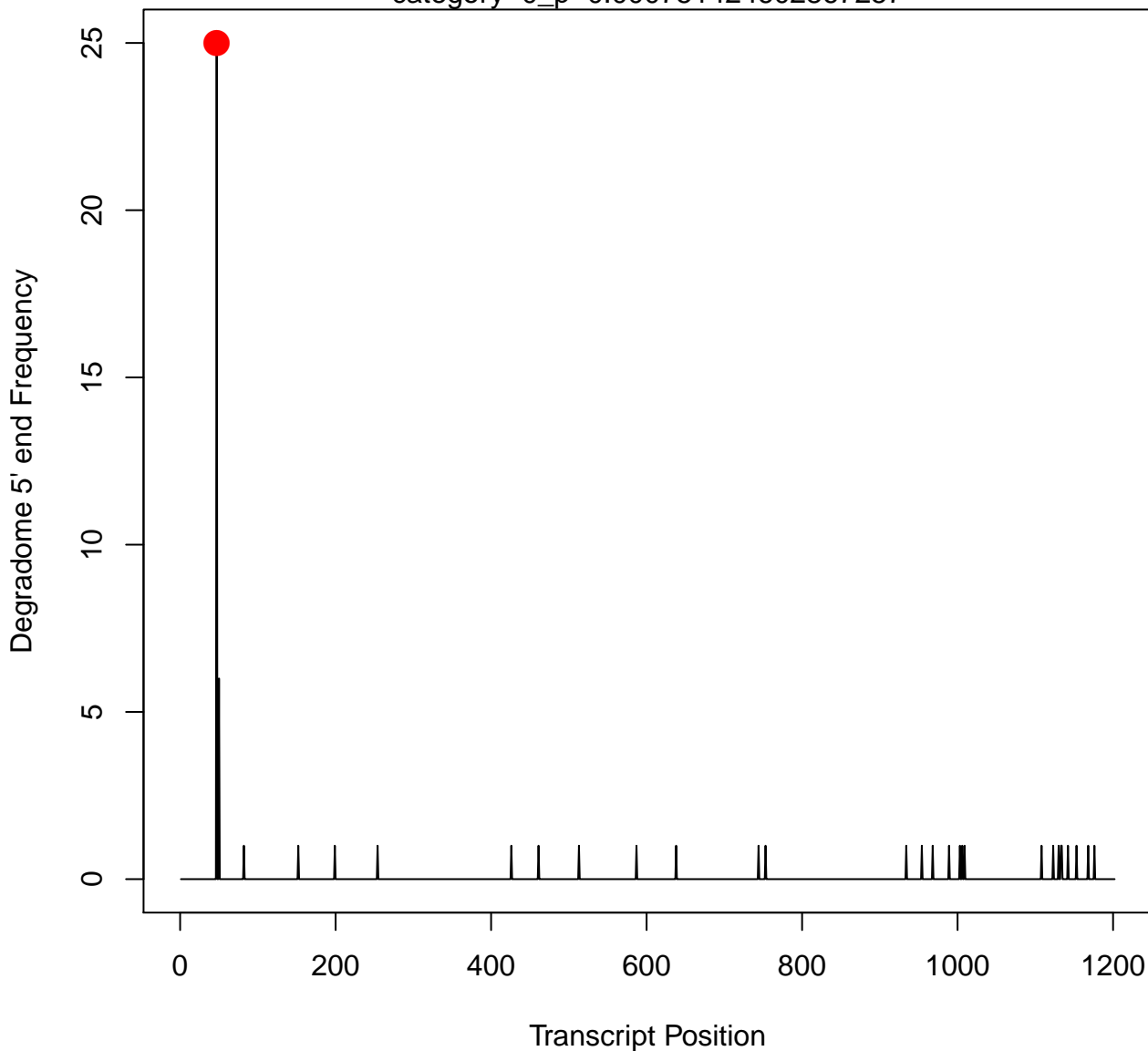

Supplement: Supplementary Data 1 — Results of categories 0–2 from PARE-Seq analysis (including three subfiles:1_1, 1_2, 1_3). [file Data_Sheet_10.ZIP › GSM2230754.plot/Lsa-miR171a_Lsat_1_v5_gn_3_128721.1_47_TPlot.pdf]

**T=Lsat\_1\_v5\_gn\_3\_681.1\_Q=Lsa-miR171a\_S=713**

category=0\_p=0.00112692513817547

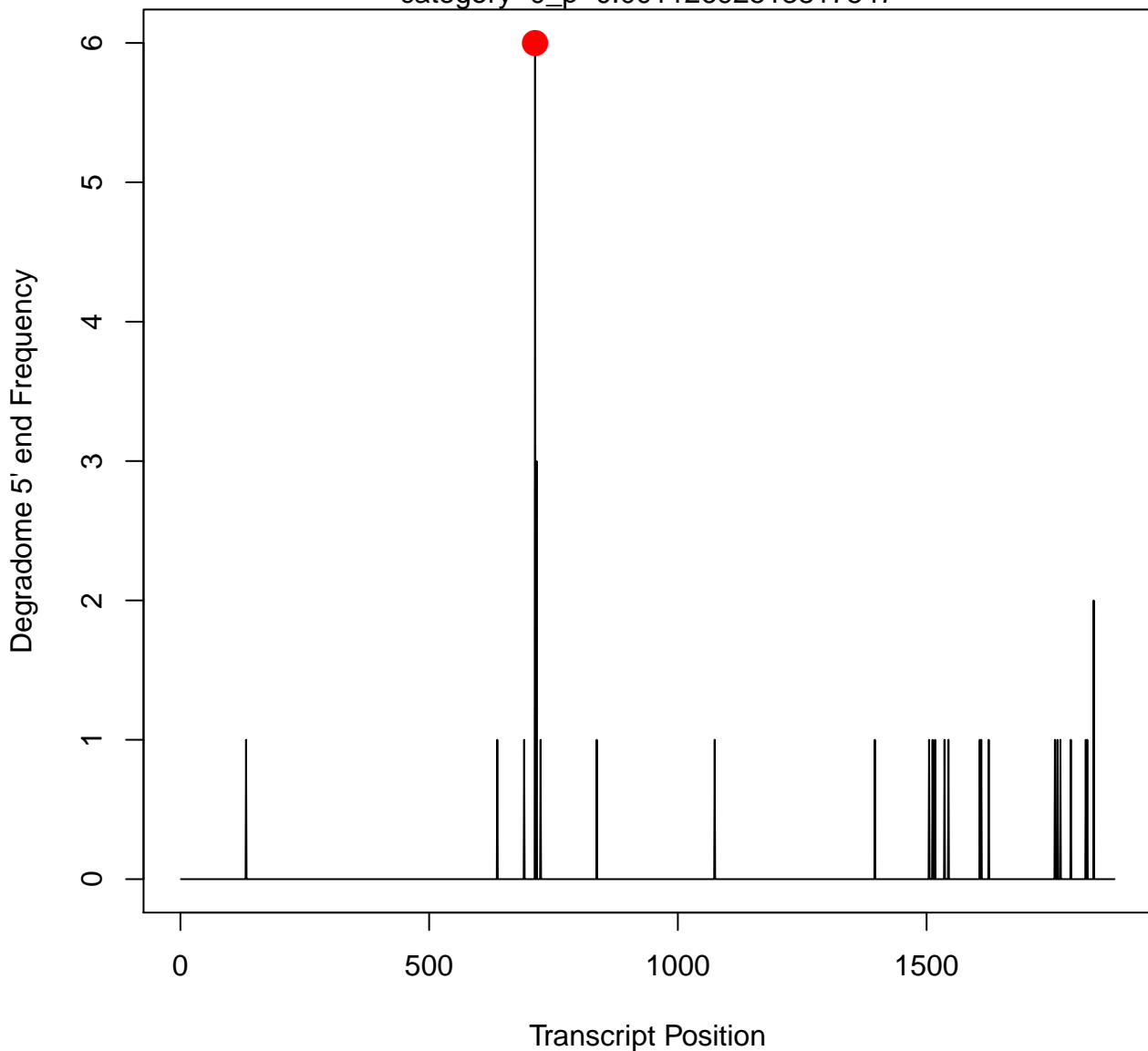

Supplement: Supplementary Data 1 — Results of categories 0–2 from PARE-Seq analysis (including three subfiles:1_1, 1_2, 1_3). [file Data_Sheet_10.ZIP › GSM2230754.plot/Lsa-miR171a_Lsat_1_v5_gn_3_681.1_713_TPlot.pdf]

**T=Lsat\_1\_v5\_gn\_4\_109141.1\_Q=Lsa-miR171a\_S=1369**

category=2\_p=0.696036350616871

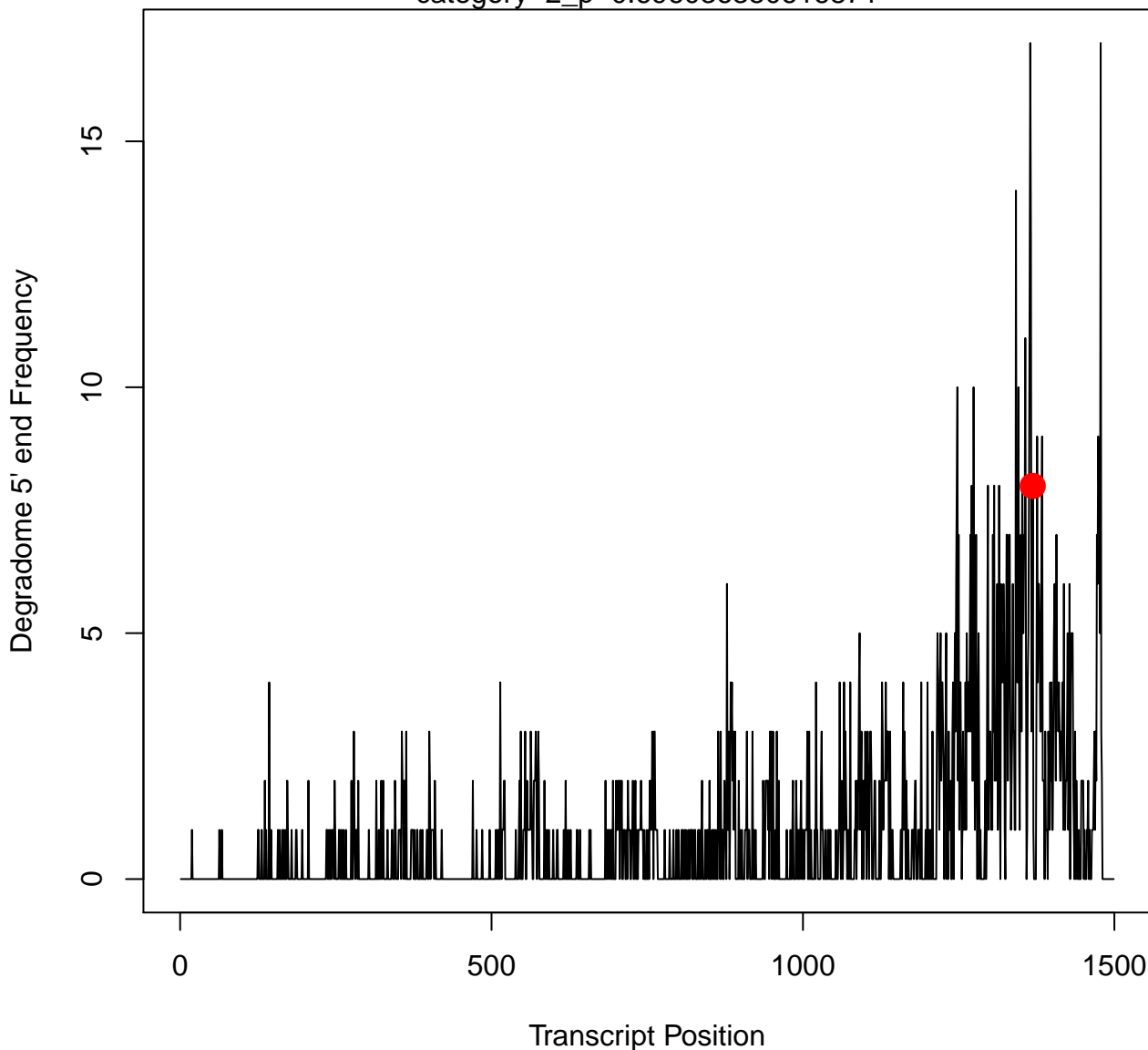

Supplement: Supplementary Data 1 — Results of categories 0–2 from PARE-Seq analysis (including three subfiles:1_1, 1_2, 1_3). [file Data_Sheet_10.ZIP › GSM2230754.plot/Lsa-miR171a_Lsat_1_v5_gn_4_109141.1_1369_TPlot.pdf]

**T=Lsat\_1\_v5\_gn\_5\_174381.1\_Q=Lsa-miR171a\_S=873**

category=2\_p=0.252073469146249

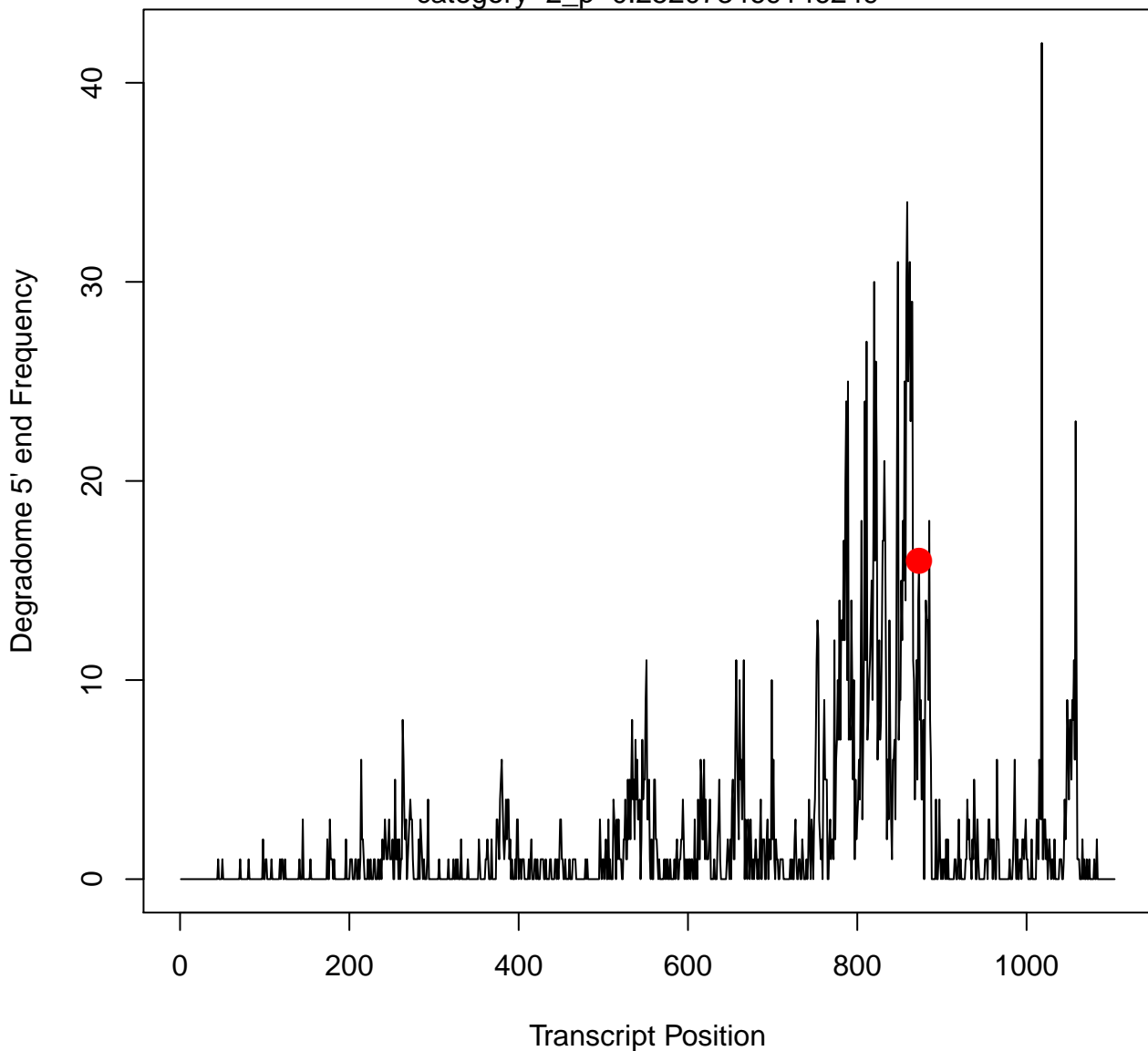

Supplement: Supplementary Data 1 — Results of categories 0–2 from PARE-Seq analysis (including three subfiles:1_1, 1_2, 1_3). [file Data_Sheet_10.ZIP › GSM2230754.plot/Lsa-miR171a_Lsat_1_v5_gn_5_174381.1_873_TPlot.pdf]

**T=Lsat\_1\_v5\_gn\_7\_9261.1\_Q=Lsa-miR171a\_S=1164**

category=2\_p=0.135172542726729

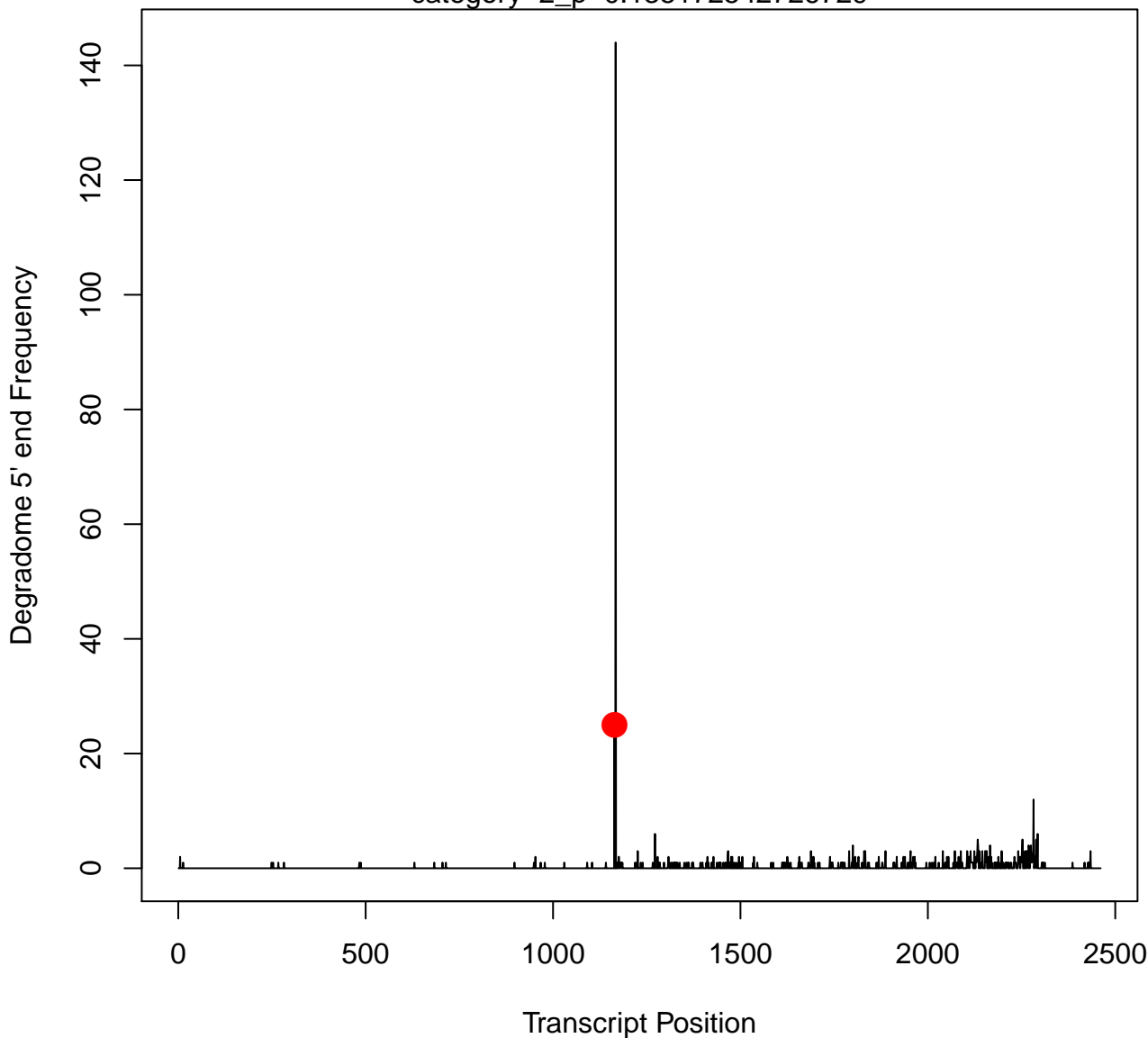

Supplement: Supplementary Data 1 — Results of categories 0–2 from PARE-Seq analysis (including three subfiles:1_1, 1_2, 1_3). [file Data_Sheet_10.ZIP › GSM2230754.plot/Lsa-miR171a_Lsat_1_v5_gn_7_9261.1_1164_TPlot.pdf]

**T=Lsat\_1\_v5\_gn\_1\_27961.1\_Q=Lsa-miR171b\_S=2561**

category=2\_p=0.581614314731531

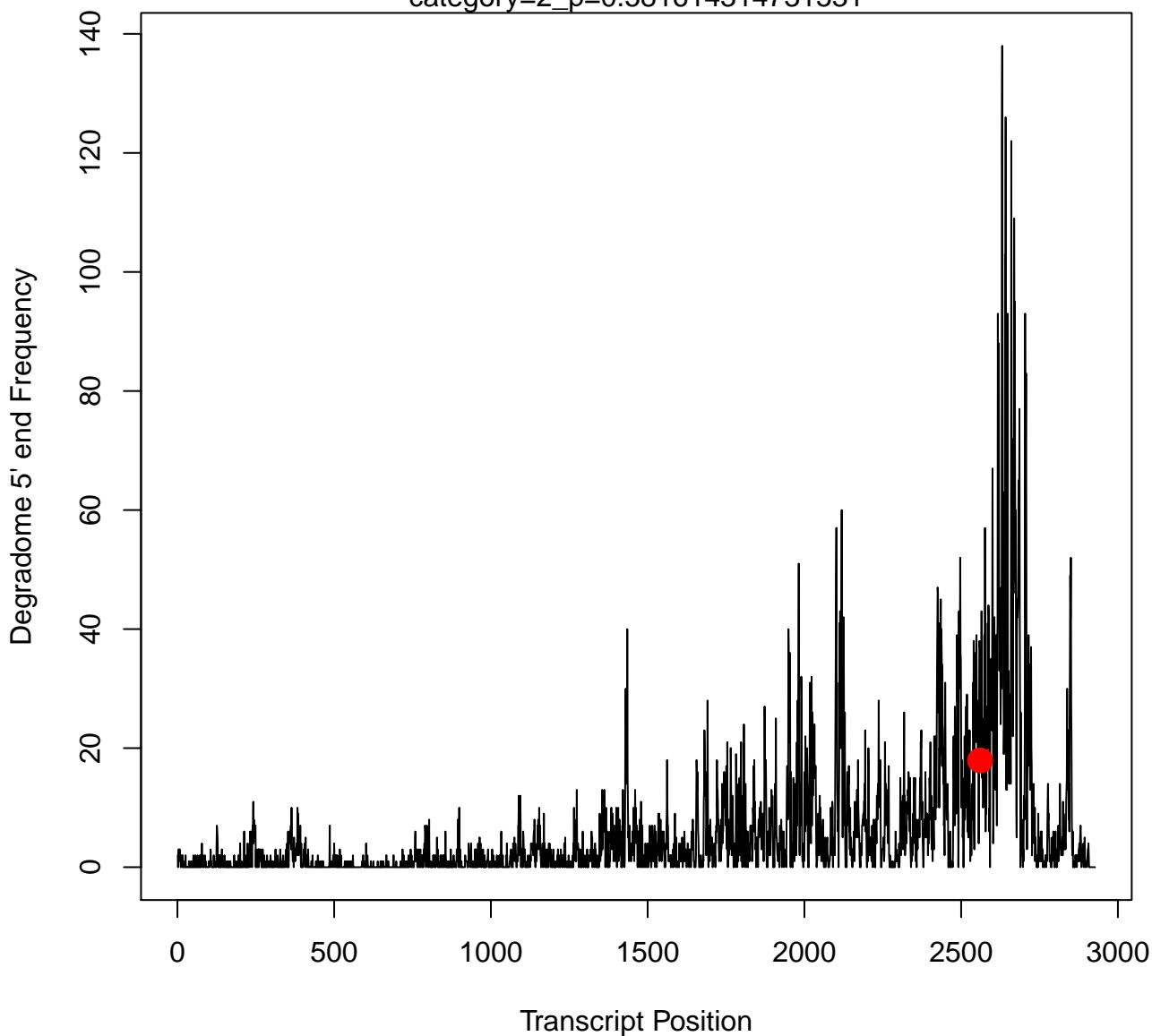

Supplement: Supplementary Data 1 — Results of categories 0–2 from PARE-Seq analysis (including three subfiles:1_1, 1_2, 1_3). [file Data_Sheet_10.ZIP › GSM2230754.plot/Lsa-miR171b_Lsat_1_v5_gn_1_27961.1_2561_TPlot.pdf]

**T=Lsat\_1\_v5\_gn\_3\_128721.1\_Q=Lsa-miR171b\_S=50**

category=2\_p=0.0834467922232335

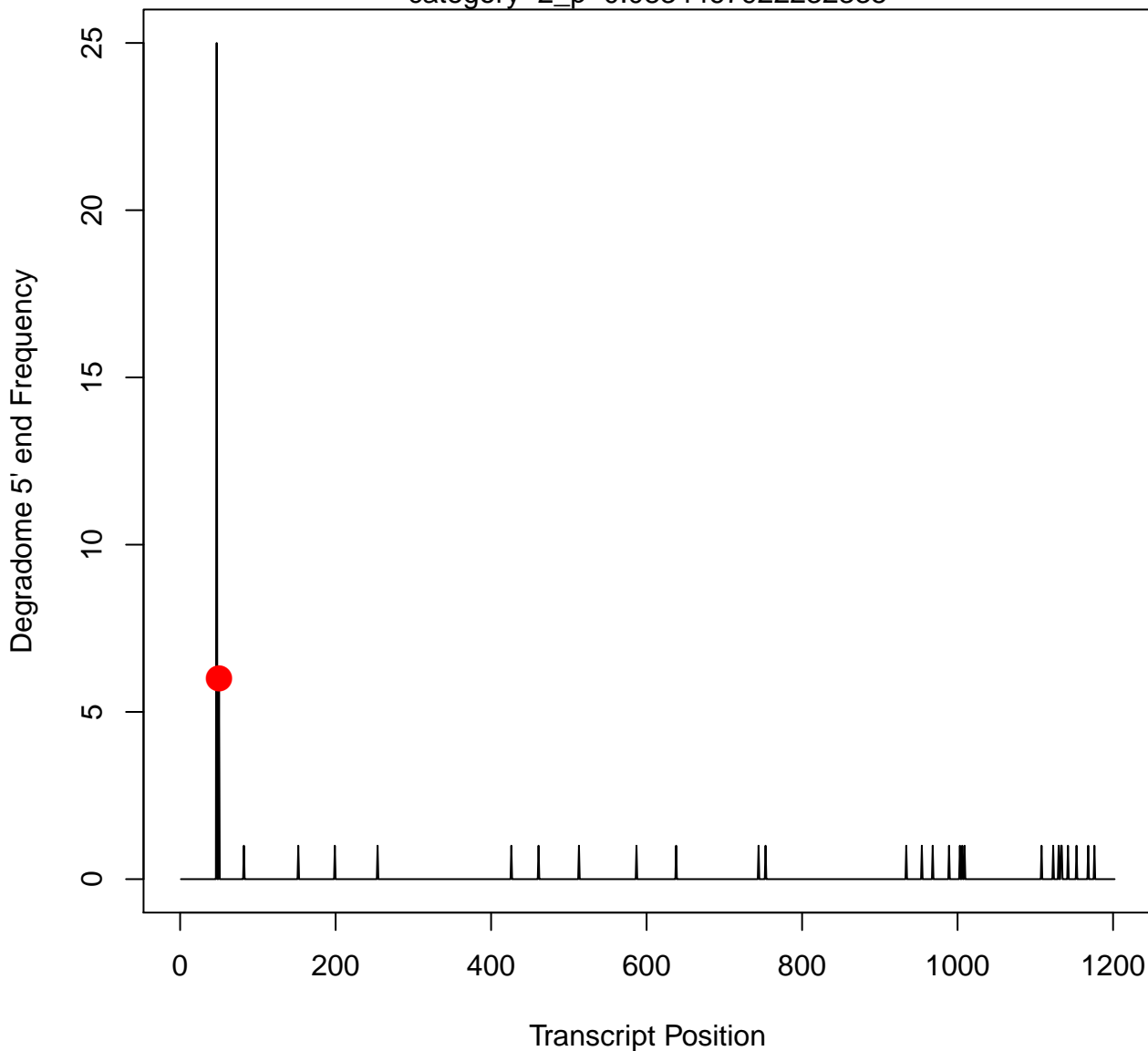

Supplement: Supplementary Data 1 — Results of categories 0–2 from PARE-Seq analysis (including three subfiles:1_1, 1_2, 1_3). [file Data_Sheet_10.ZIP › GSM2230754.plot/Lsa-miR171b_Lsat_1_v5_gn_3_128721.1_50_TPlot.pdf]

**T=Lsat\_1\_v5\_gn\_3\_681.1\_Q=Lsa-miR171b\_S=716**

category=2\_p=0.109685235359291

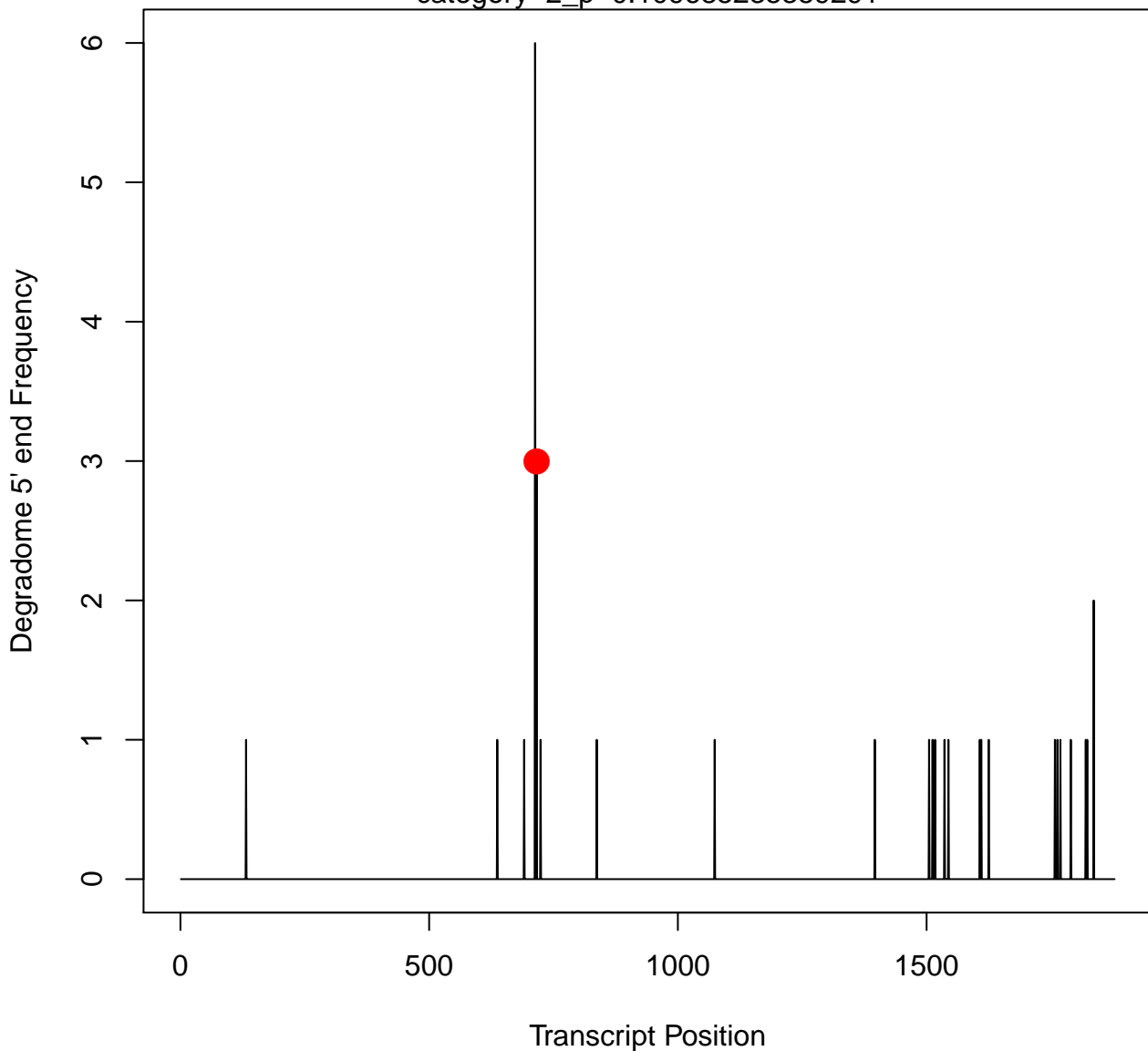

Supplement: Supplementary Data 1 — Results of categories 0–2 from PARE-Seq analysis (including three subfiles:1_1, 1_2, 1_3). [file Data_Sheet_10.ZIP › GSM2230754.plot/Lsa-miR171b_Lsat_1_v5_gn_3_681.1_716_TPlot.pdf]

**T=Lsat\_1\_v5\_gn\_3\_78621.1\_Q=Lsa-miR171b\_S=735**

category=2\_p=0.135172542726729

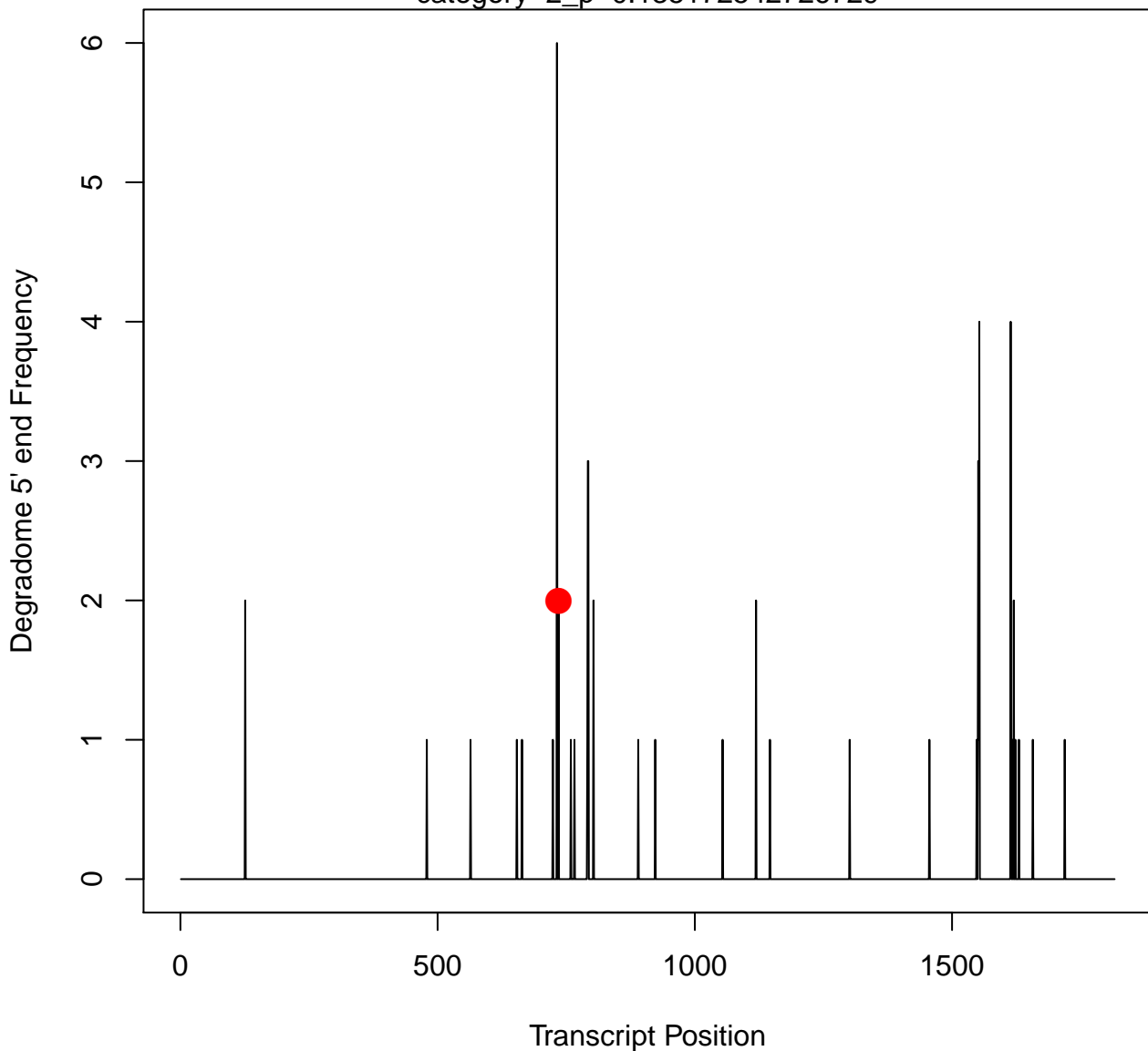

Supplement: Supplementary Data 1 — Results of categories 0–2 from PARE-Seq analysis (including three subfiles:1_1, 1_2, 1_3). [file Data_Sheet_10.ZIP › GSM2230754.plot/Lsa-miR171b_Lsat_1_v5_gn_3_78621.1_735_TPlot.pdf]

**T=Lsat\_1\_v5\_gn\_4\_153400.1\_Q=Lsa-miR171b\_S=648**

category=2\_p=0.159930217314119

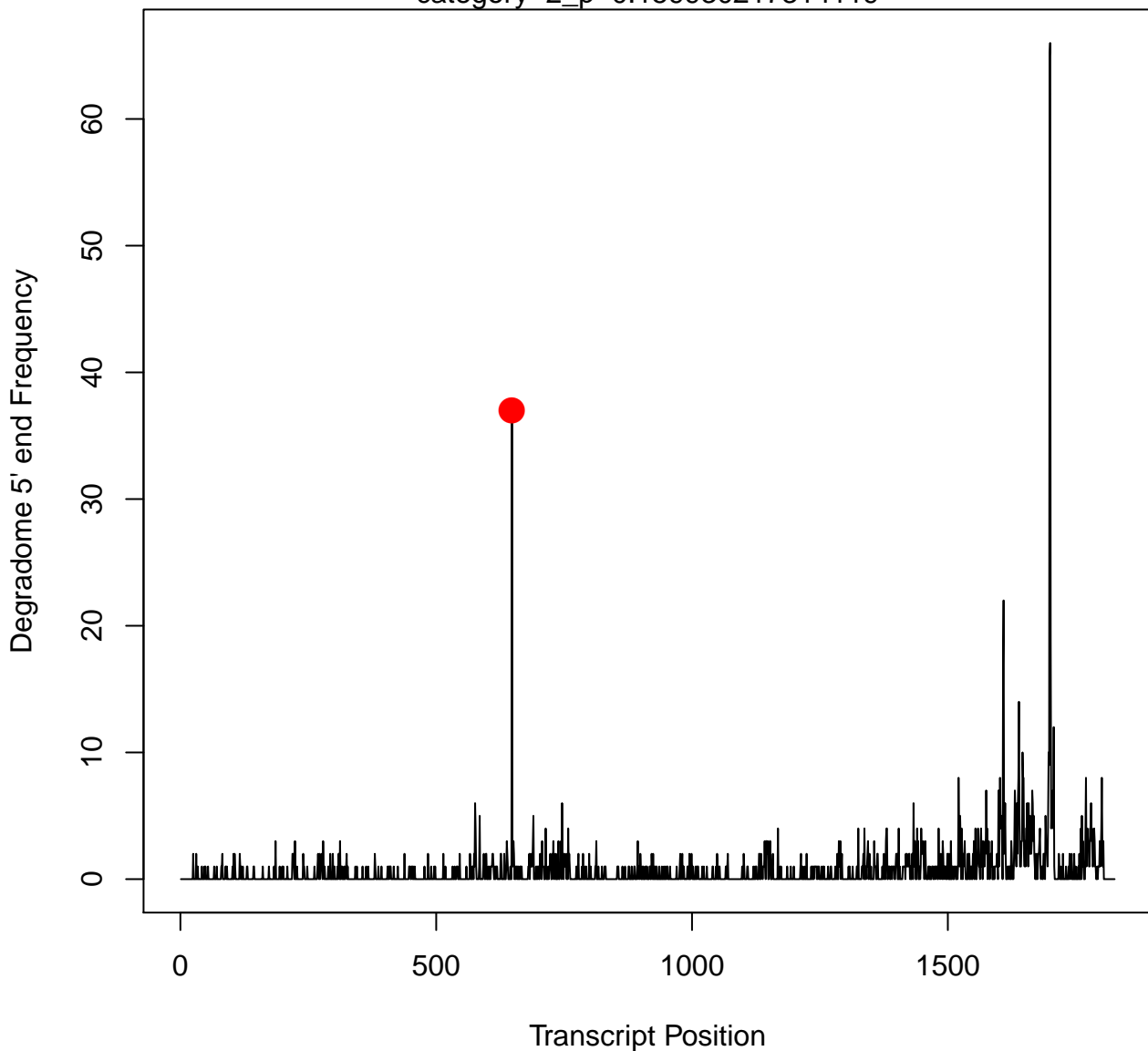

Supplement: Supplementary Data 1 — Results of categories 0–2 from PARE-Seq analysis (including three subfiles:1_1, 1_2, 1_3). [file Data_Sheet_10.ZIP › GSM2230754.plot/Lsa-miR171b_Lsat_1_v5_gn_4_153400.1_648_TPlot.pdf]

**T=Lsat\_1\_v5\_gn\_6\_3180.1\_Q=Lsa-miR171b\_S=1761**

category=0\_p=0.00037578290783058

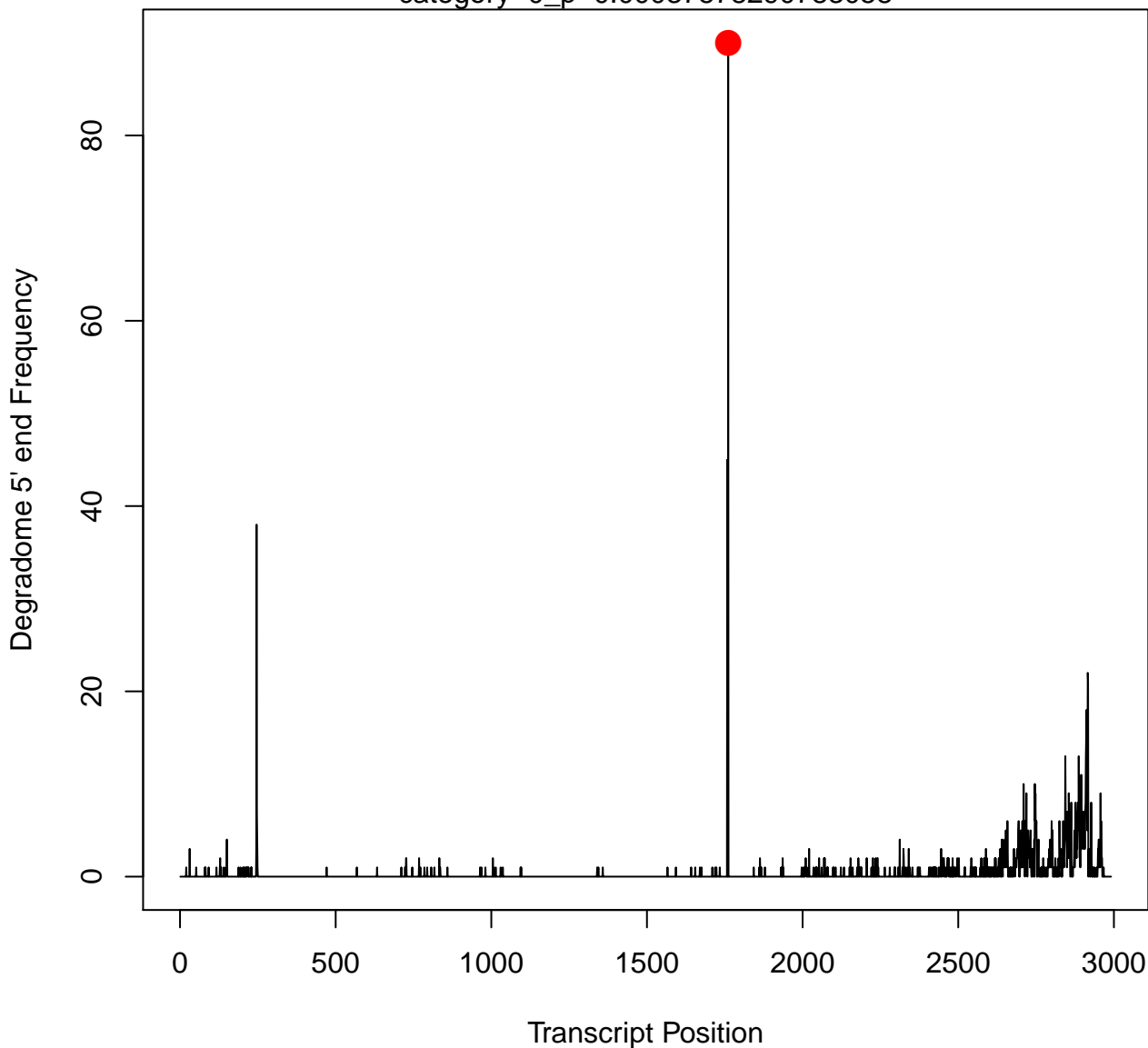

Supplement: Supplementary Data 1 — Results of categories 0–2 from PARE-Seq analysis (including three subfiles:1_1, 1_2, 1_3). [file Data_Sheet_10.ZIP › GSM2230754.plot/Lsa-miR171b_Lsat_1_v5_gn_6_3180.1_1761_TPlot.pdf]

**T=Lsat\_1\_v5\_gn\_7\_9261.1\_Q=Lsa-miR171b\_S=1167**

category=0\_p=0.000751424602867257

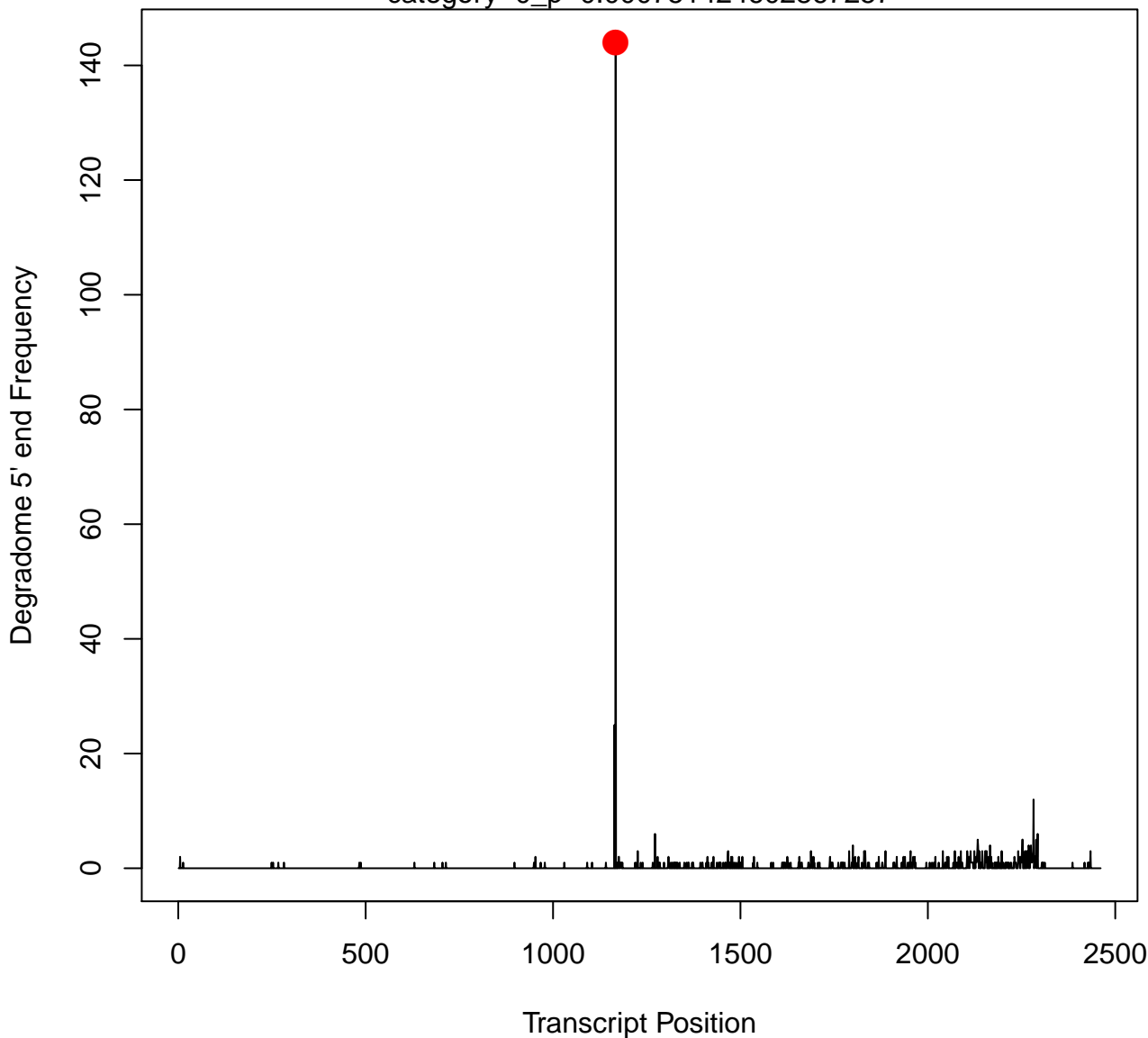

Supplement: Supplementary Data 1 — Results of categories 0–2 from PARE-Seq analysis (including three subfiles:1_1, 1_2, 1_3). [file Data_Sheet_10.ZIP › GSM2230754.plot/Lsa-miR171b_Lsat_1_v5_gn_7_9261.1_1167_TPlot.pdf]

**T=Lsat\_1\_v5\_gn\_8\_90501.1\_Q=Lsa-miR171b\_S=882**

category=2\_p=0.984739501449729

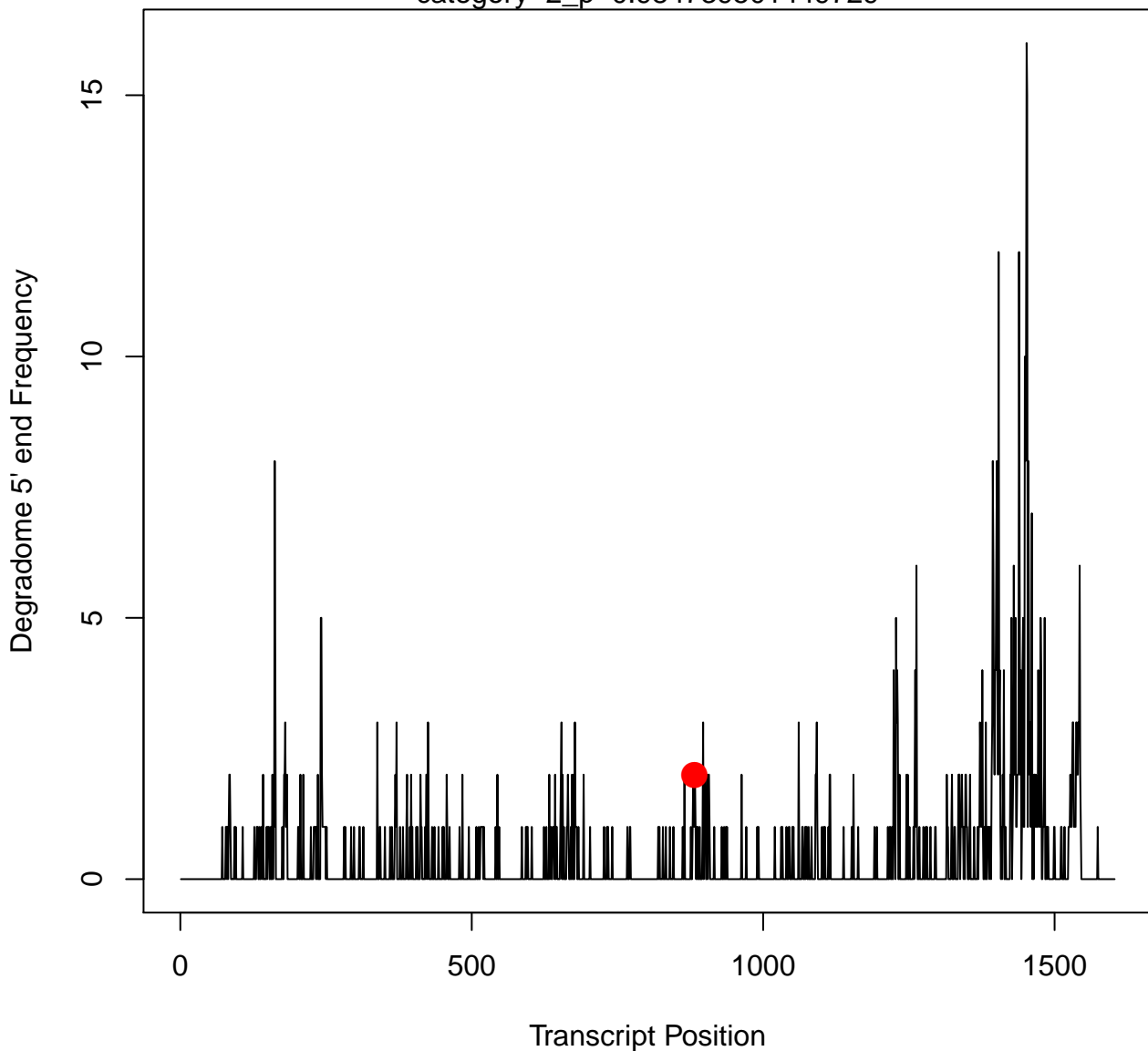

Supplement: Supplementary Data 1 — Results of categories 0–2 from PARE-Seq analysis (including three subfiles:1_1, 1_2, 1_3). [file Data_Sheet_10.ZIP › GSM2230754.plot/Lsa-miR171b_Lsat_1_v5_gn_8_90501.1_882_TPlot.pdf]

**T=Lsat\_1\_v5\_gn\_2\_136081.1\_Q=Lsa-miR171c\_S=993**

category=2\_p=0.638168571649735

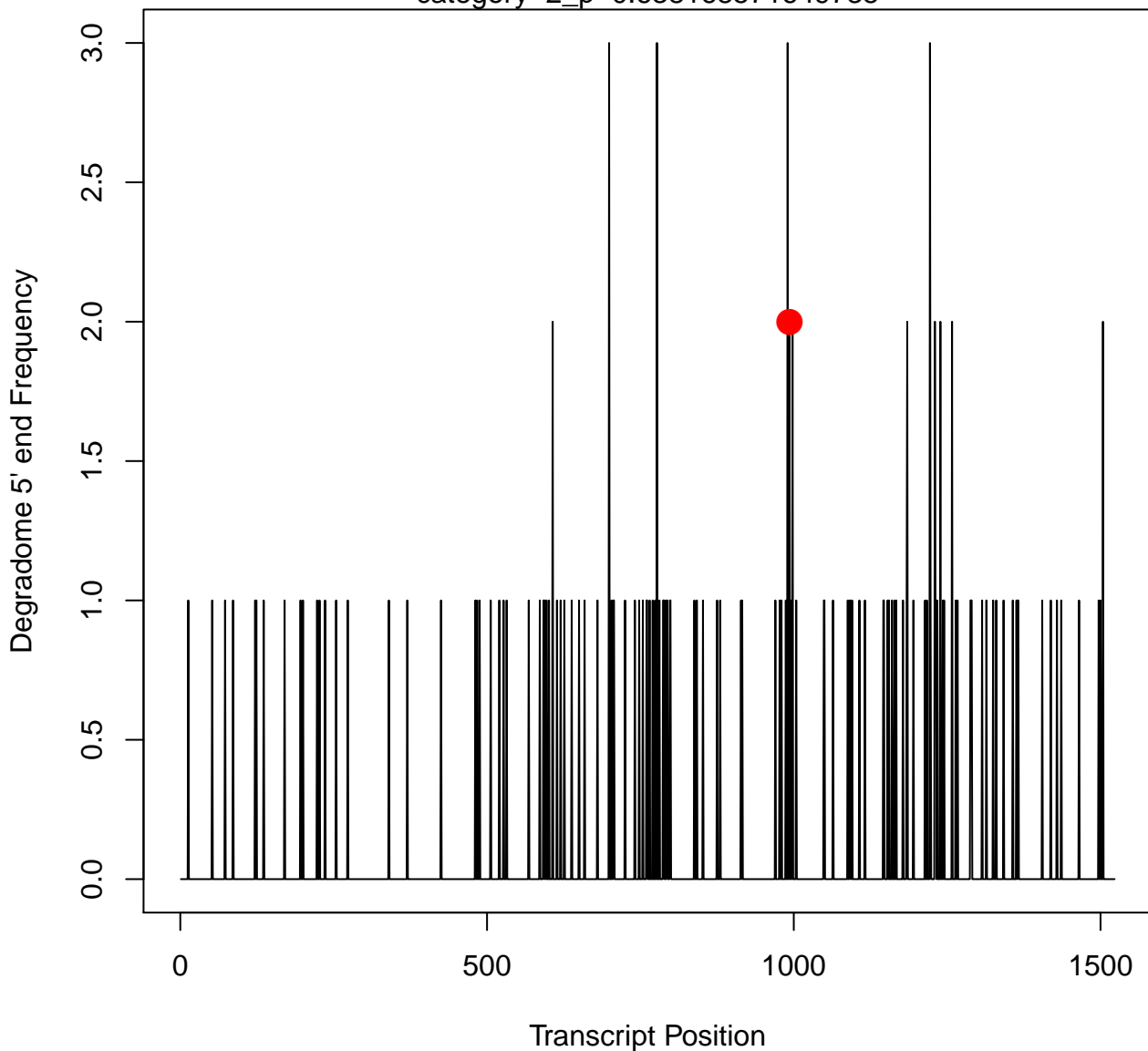

Supplement: Supplementary Data 1 — Results of categories 0–2 from PARE-Seq analysis (including three subfiles:1_1, 1_2, 1_3). [file Data_Sheet_10.ZIP › GSM2230754.plot/Lsa-miR171c_Lsat_1_v5_gn_2_136081.1_993_TPlot.pdf]

**T=Lsat\_1\_v5\_gn\_3\_63880.1\_Q=Lsa-miR171c\_S=577**

category=2\_p=0.98074774653474

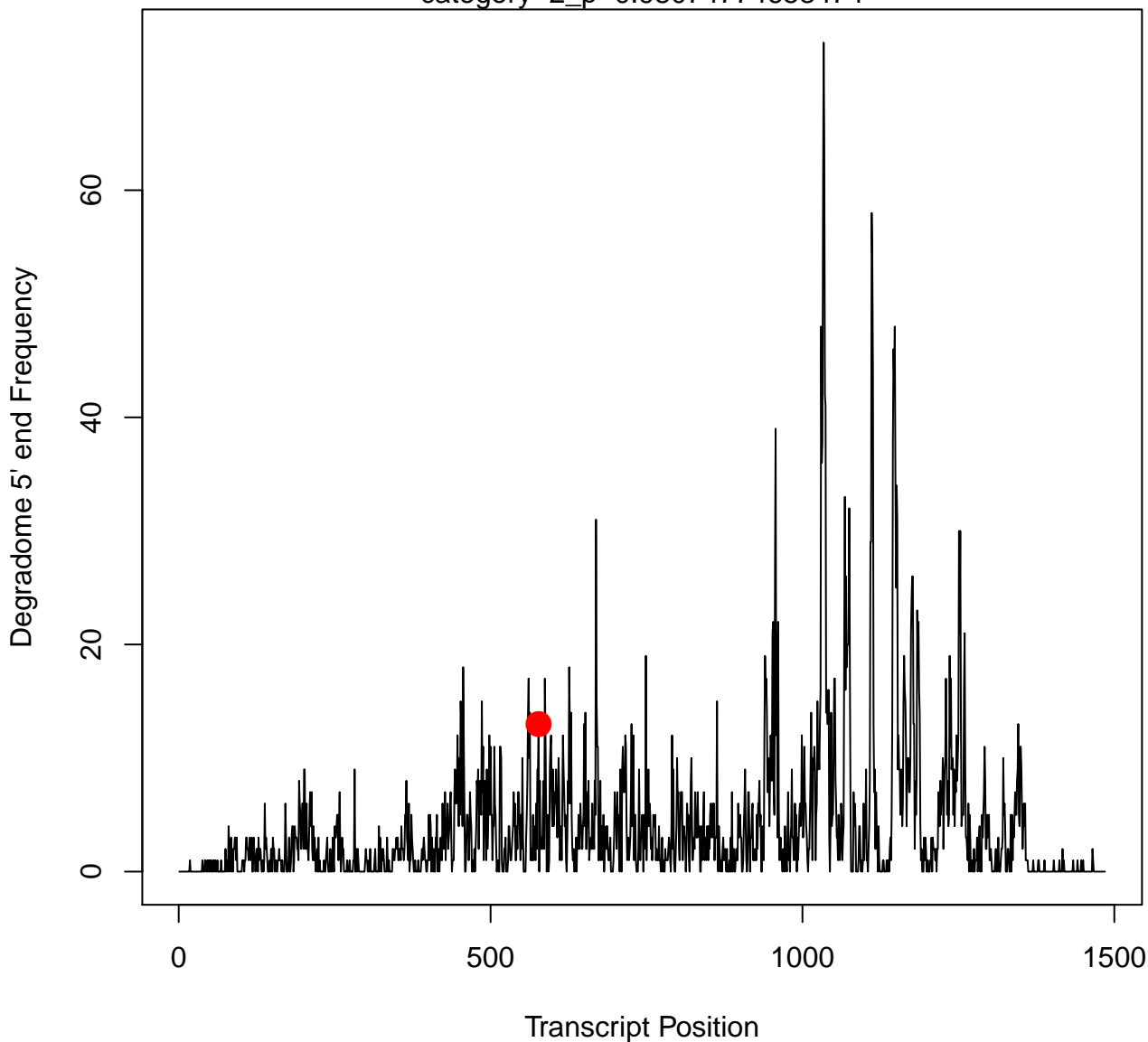

Supplement: Supplementary Data 1 — Results of categories 0–2 from PARE-Seq analysis (including three subfiles:1_1, 1_2, 1_3). [file Data_Sheet_10.ZIP › GSM2230754.plot/Lsa-miR171c_Lsat_1_v5_gn_3_63880.1_577_TPlot.pdf]

**T=Lsat\_1\_v5\_gn\_3\_65860.1\_Q=Lsa-miR171c\_S=2360**

category=2\_p=0.951232222278289

Degradsome 5' end Frequency

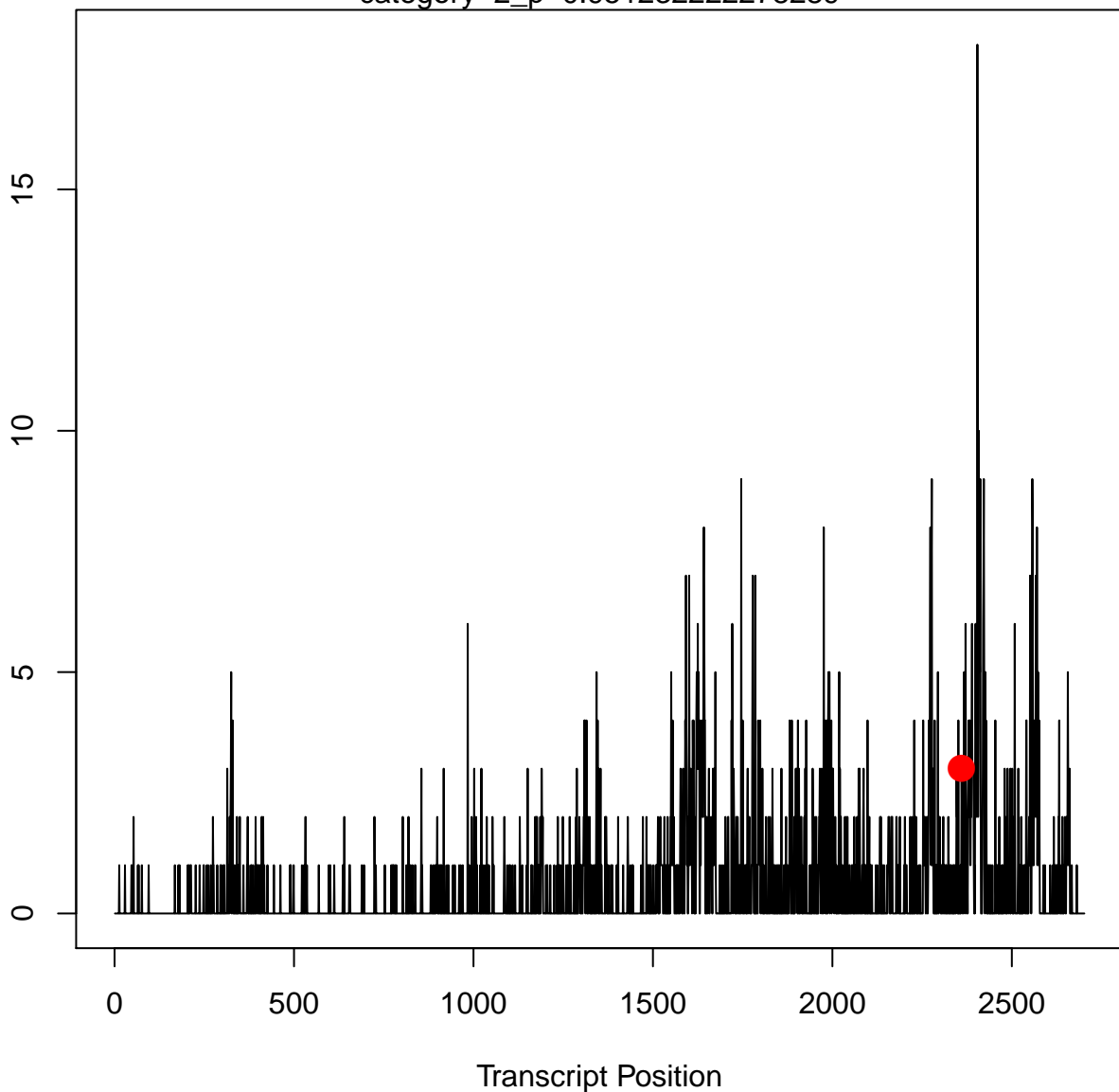

Supplement: Supplementary Data 1 — Results of categories 0–2 from PARE-Seq analysis (including three subfiles:1_1, 1_2, 1_3). [file Data_Sheet_10.ZIP › GSM2230754.plot/Lsa-miR171c_Lsat_1_v5_gn_3_65860.1_2360_TPlot.pdf]

**T=Lsat\_1\_v5\_gn\_6\_86061.1\_Q=Lsa-miR171c\_S=774**

category=2\_p=0.962450339825284

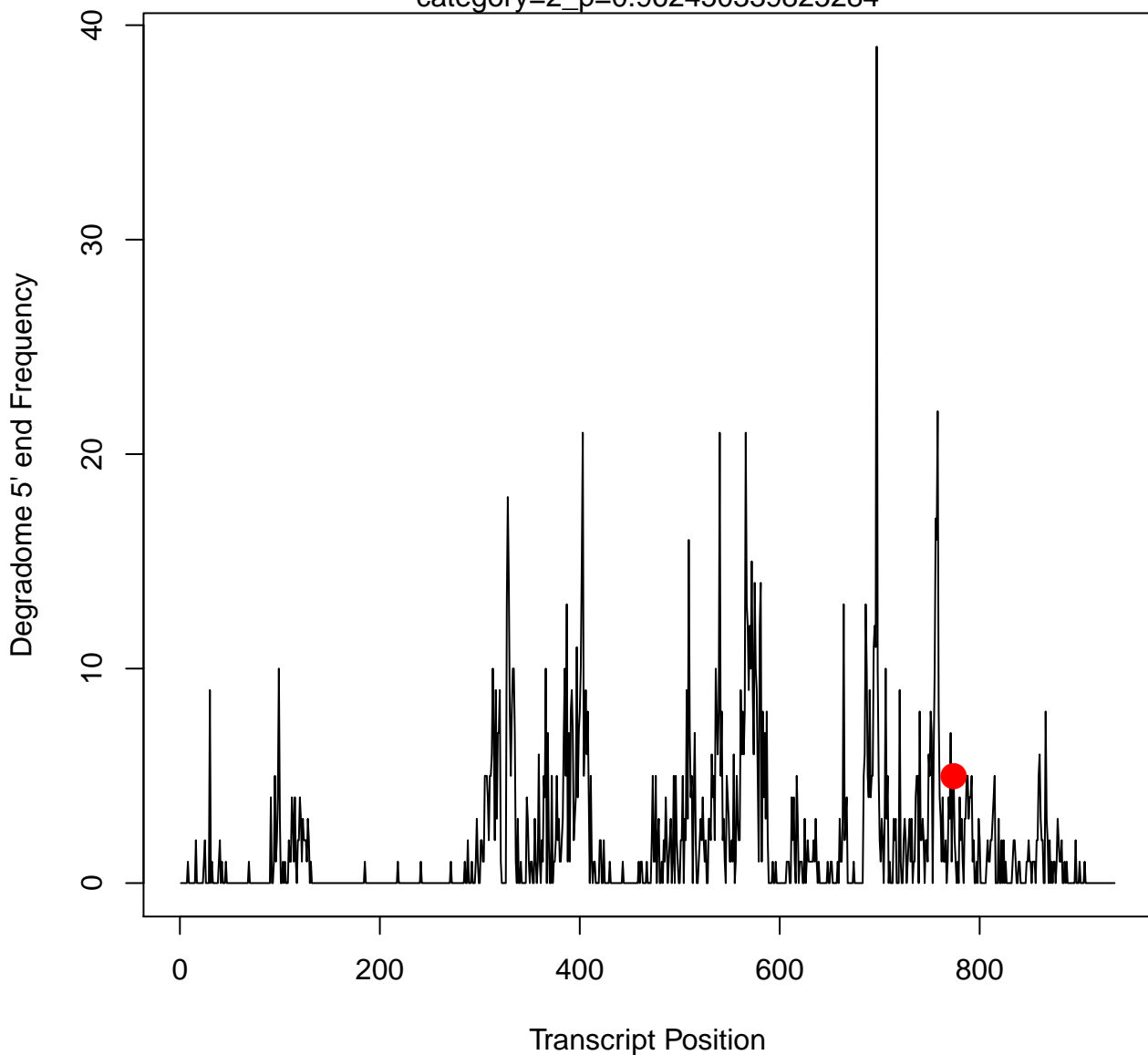

Supplement: Supplementary Data 1 — Results of categories 0–2 from PARE-Seq analysis (including three subfiles:1_1, 1_2, 1_3). [file Data_Sheet_10.ZIP › GSM2230754.plot/Lsa-miR171c_Lsat_1_v5_gn_6_86061.1_774_TPlot.pdf]

**T=Lsat\_1\_v5\_gn\_7\_105700.1\_Q=Lsa-miR171c\_S=500**

category=2\_p=0.992175721837987

Degradsome 5' end Frequency

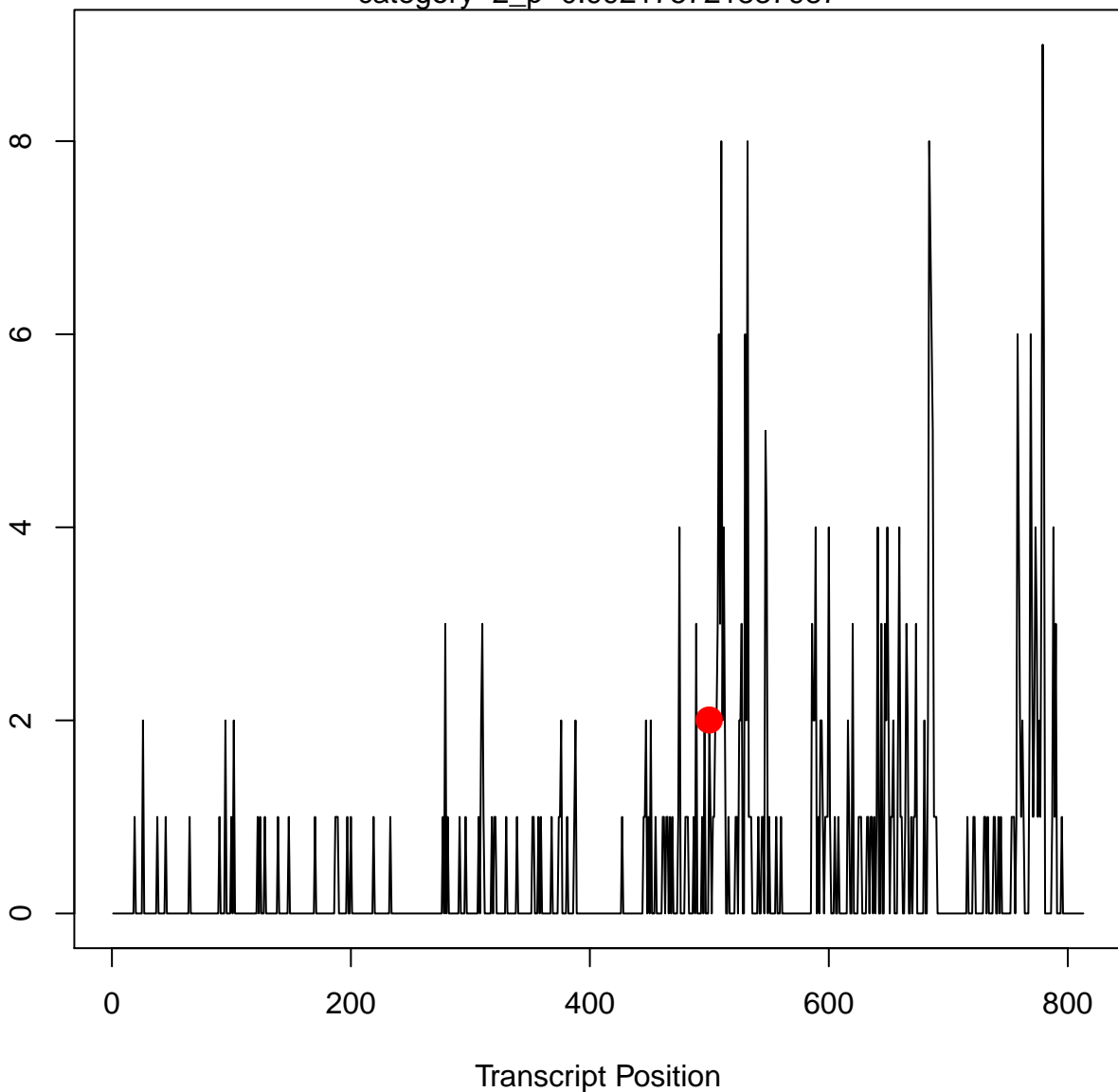

Supplement: Supplementary Data 1 — Results of categories 0–2 from PARE-Seq analysis (including three subfiles:1_1, 1_2, 1_3). [file Data_Sheet_10.ZIP › GSM2230754.plot/Lsa-miR171c_Lsat_1_v5_gn_7_105700.1_500_TPlot.pdf]

T=Lsat\_1\_v5\_gn\_7\_49541.1\_Q=Lsa-miR171c\_S=871

category=2\_p=0.371689521760685

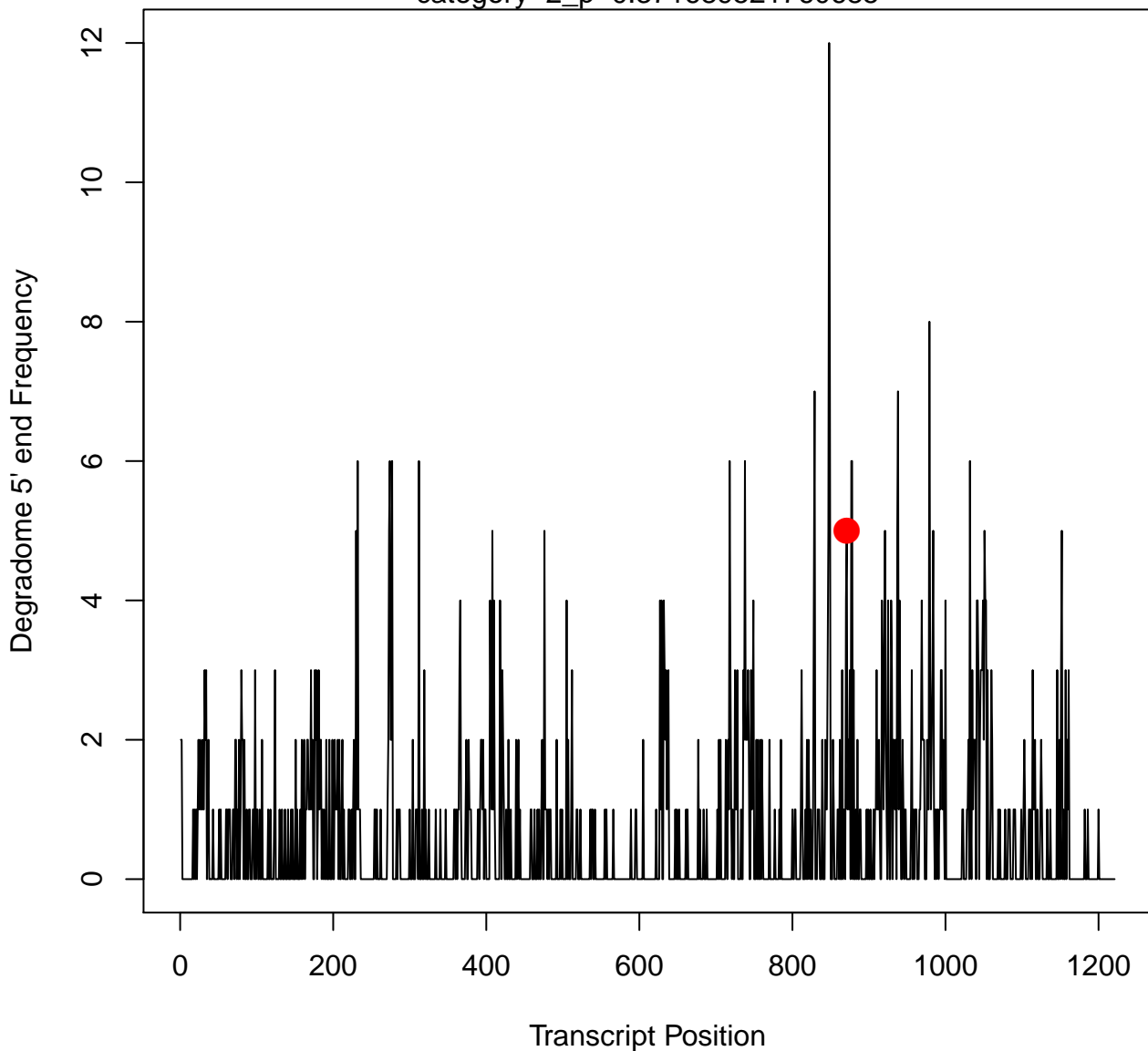

Supplement: Supplementary Data 1 — Results of categories 0–2 from PARE-Seq analysis (including three subfiles:1_1, 1_2, 1_3). [file Data_Sheet_10.ZIP › GSM2230754.plot/Lsa-miR171c_Lsat_1_v5_gn_7_49541.1_871_TPlot.pdf]

**T=Lsat\_1\_v5\_gn\_8\_164760.1\_Q=Lsa-miR171c\_S=1644**

category=2\_p=0.658588556030749

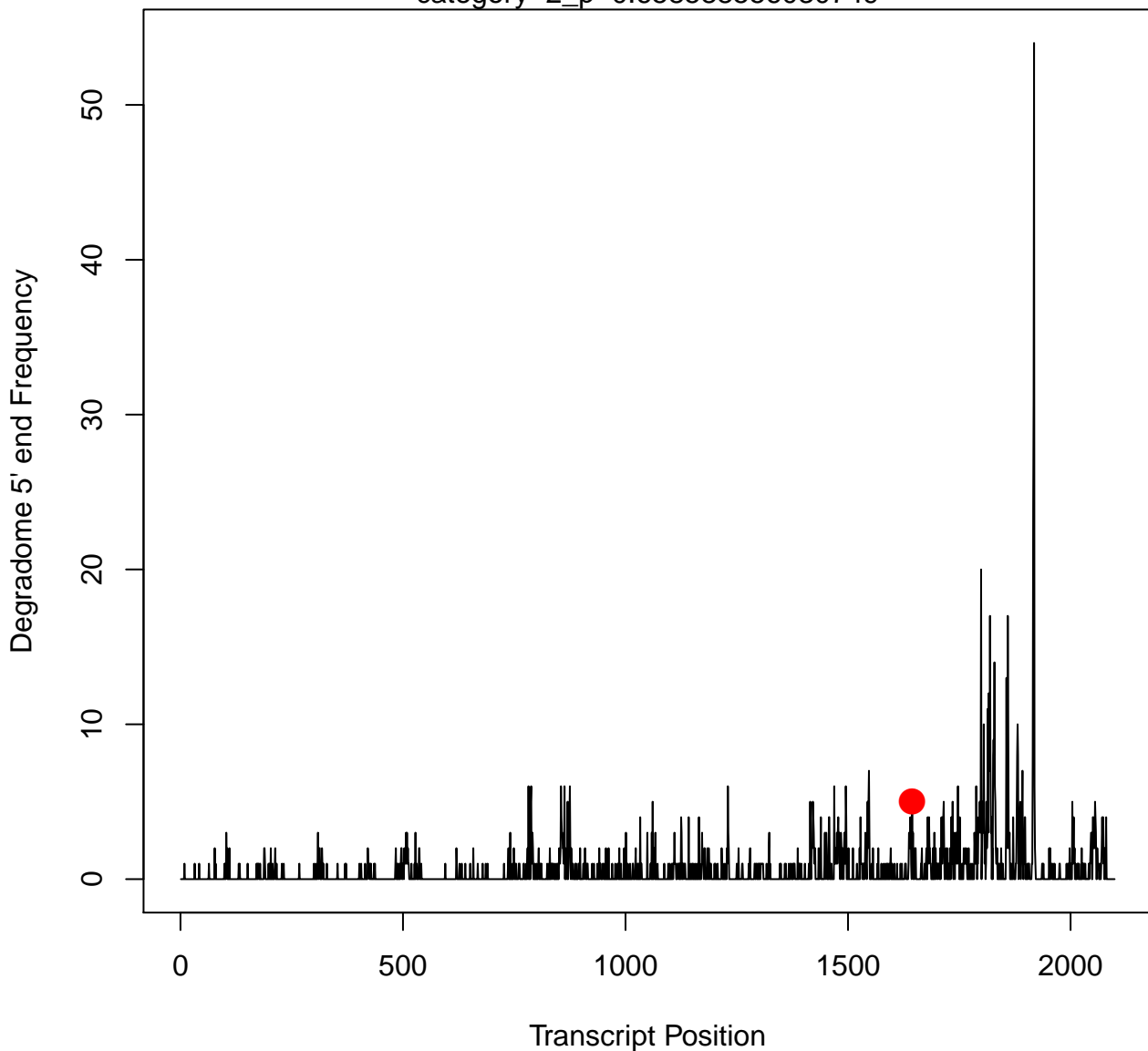

Supplement: Supplementary Data 1 — Results of categories 0–2 from PARE-Seq analysis (including three subfiles:1_1, 1_2, 1_3). [file Data_Sheet_10.ZIP › GSM2230754.plot/Lsa-miR171c_Lsat_1_v5_gn_8_164760.1_1644_TPlot.pdf]

**T=Lsat\_1\_v5\_gn\_8\_35100.1\_Q=Lsa-miR171c\_S=204**

category=2\_p=0.98953870756118

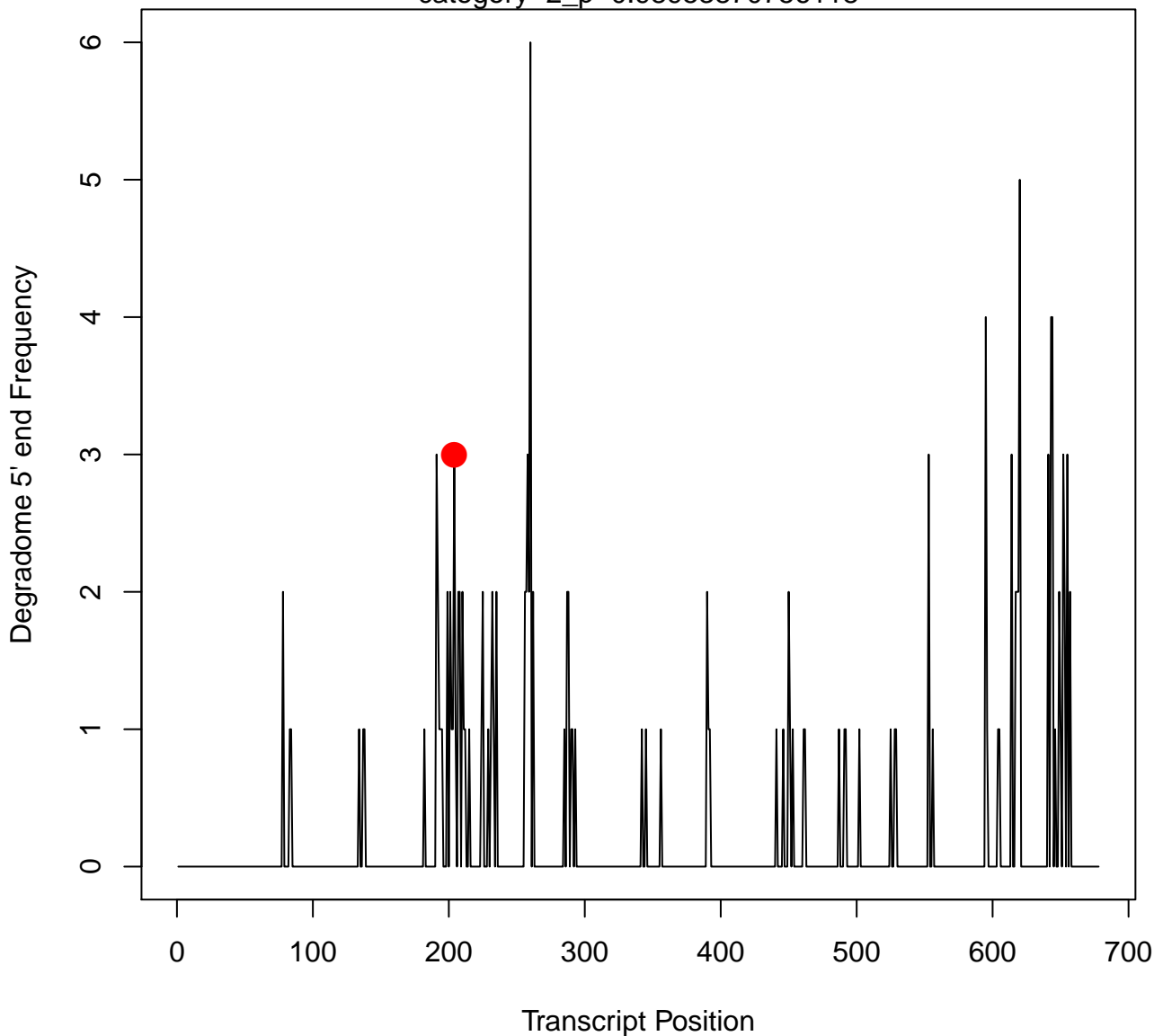

Supplement: Supplementary Data 1 — Results of categories 0–2 from PARE-Seq analysis (including three subfiles:1_1, 1_2, 1_3). [file Data_Sheet_10.ZIP › GSM2230754.plot/Lsa-miR171c_Lsat_1_v5_gn_8_35100.1_204_TPlot.pdf]

**T=Lsat\_1\_v5\_gn\_9\_35481.1\_Q=Lsa-miR171c\_S=210**

category=2\_p=0.987903607418058

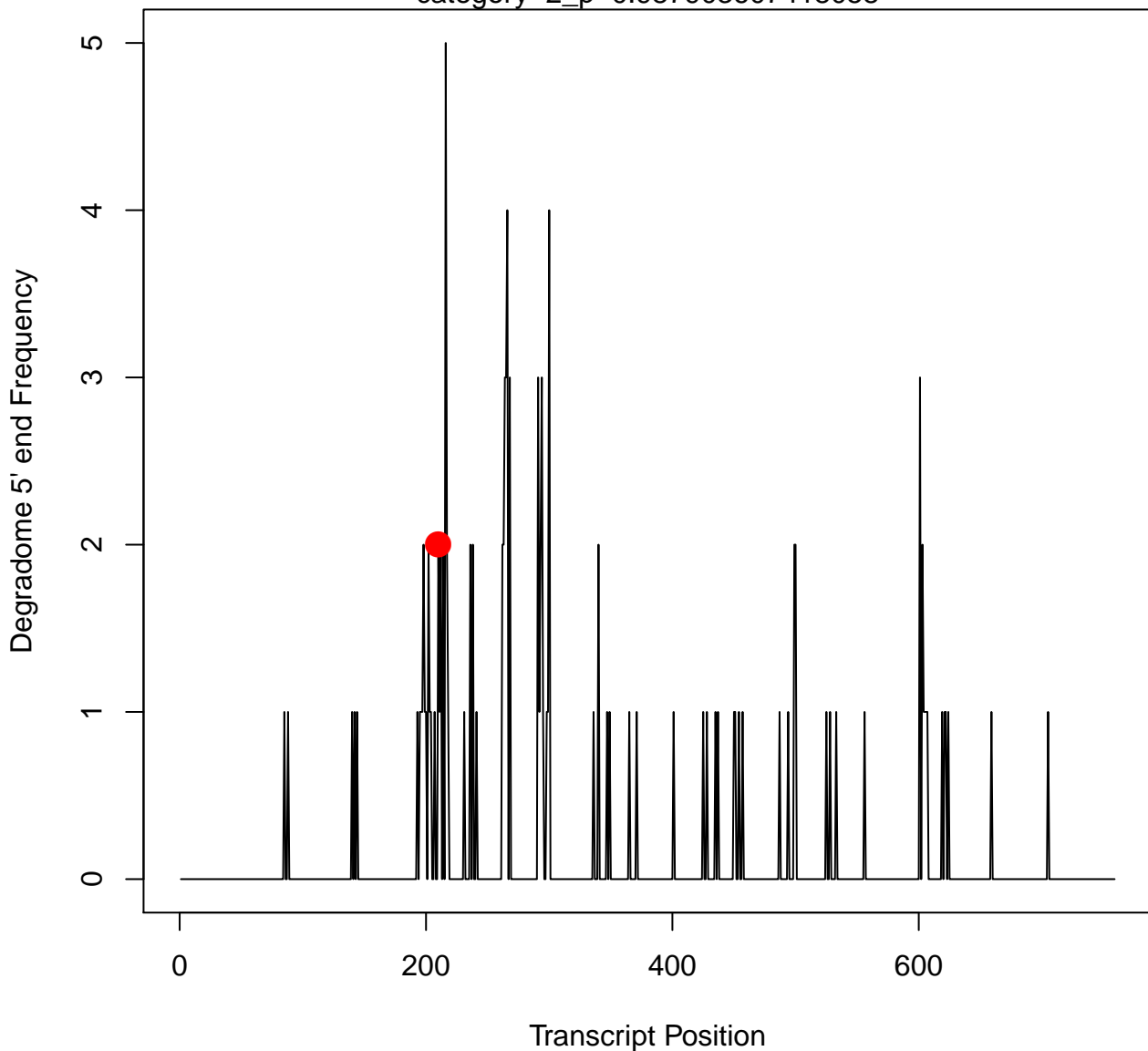

Supplement: Supplementary Data 1 — Results of categories 0–2 from PARE-Seq analysis (including three subfiles:1_1, 1_2, 1_3). [file Data_Sheet_10.ZIP › GSM2230754.plot/Lsa-miR171c_Lsat_1_v5_gn_9_35481.1_210_TPlot.pdf]

**T=Lsat\_1\_v5\_gn\_1\_130060.1\_Q=Lsa-miR171d\_S=1286**

category=2\_p=0.696036350616871

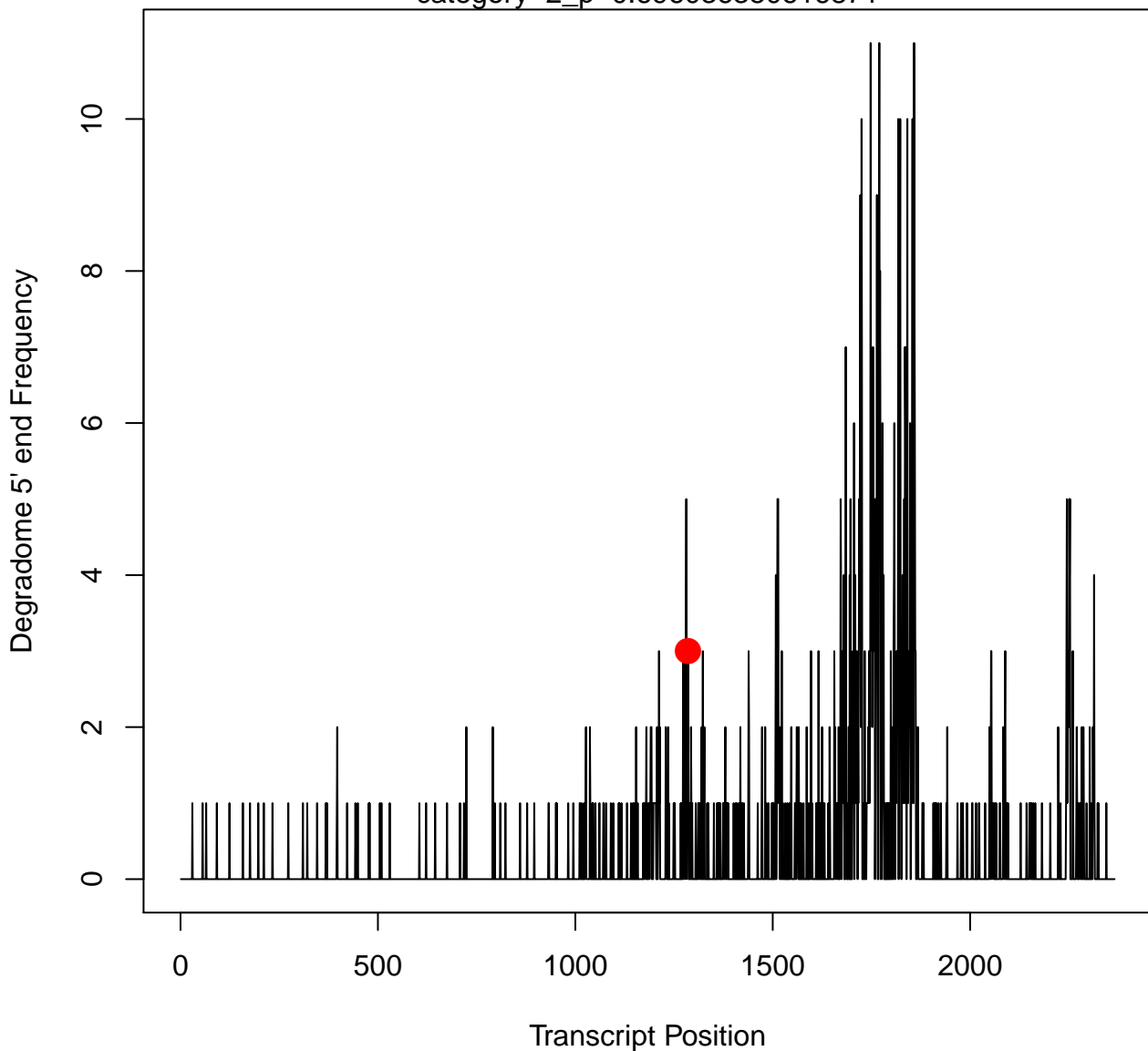

Supplement: Supplementary Data 1 — Results of categories 0–2 from PARE-Seq analysis (including three subfiles:1_1, 1_2, 1_3). [file Data_Sheet_10.ZIP › GSM2230754.plot/Lsa-miR171d_Lsat_1_v5_gn_1_130060.1_1286_TPlot.pdf]

**T=Lsat\_1\_v5\_gn\_6\_3180.1\_Q=Lsa-miR171d\_S=1758**

category=2\_p=0.0834467922232335

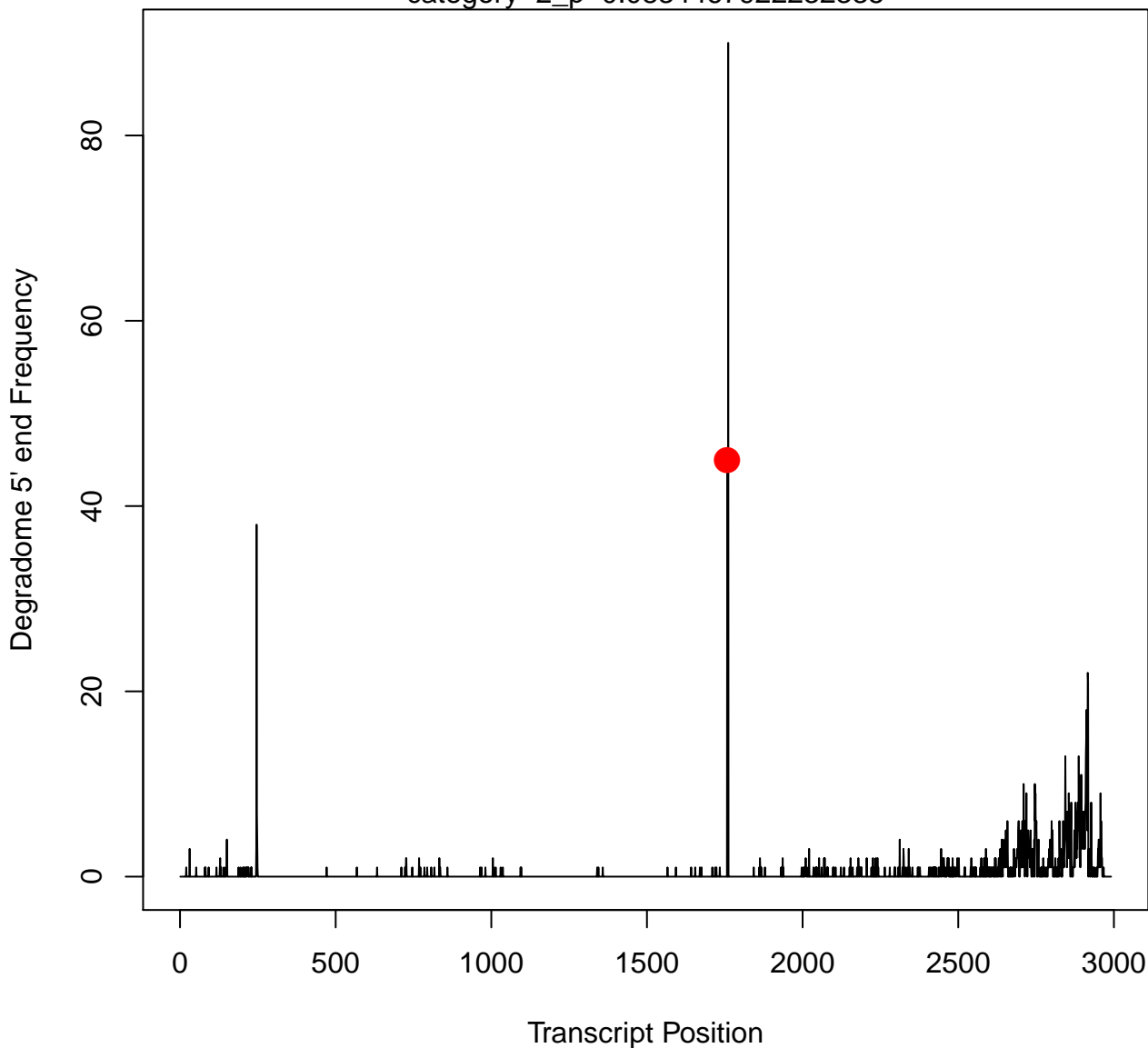

Supplement: Supplementary Data 1 — Results of categories 0–2 from PARE-Seq analysis (including three subfiles:1_1, 1_2, 1_3). [file Data_Sheet_10.ZIP › GSM2230754.plot/Lsa-miR171d_Lsat_1_v5_gn_6_3180.1_1758_TPlot.pdf]

**T=Lsat\_1\_v5\_gn\_8\_122761.1\_Q=Lsa-miR171d\_S=610**

category=0\_p=0.0296207286884169

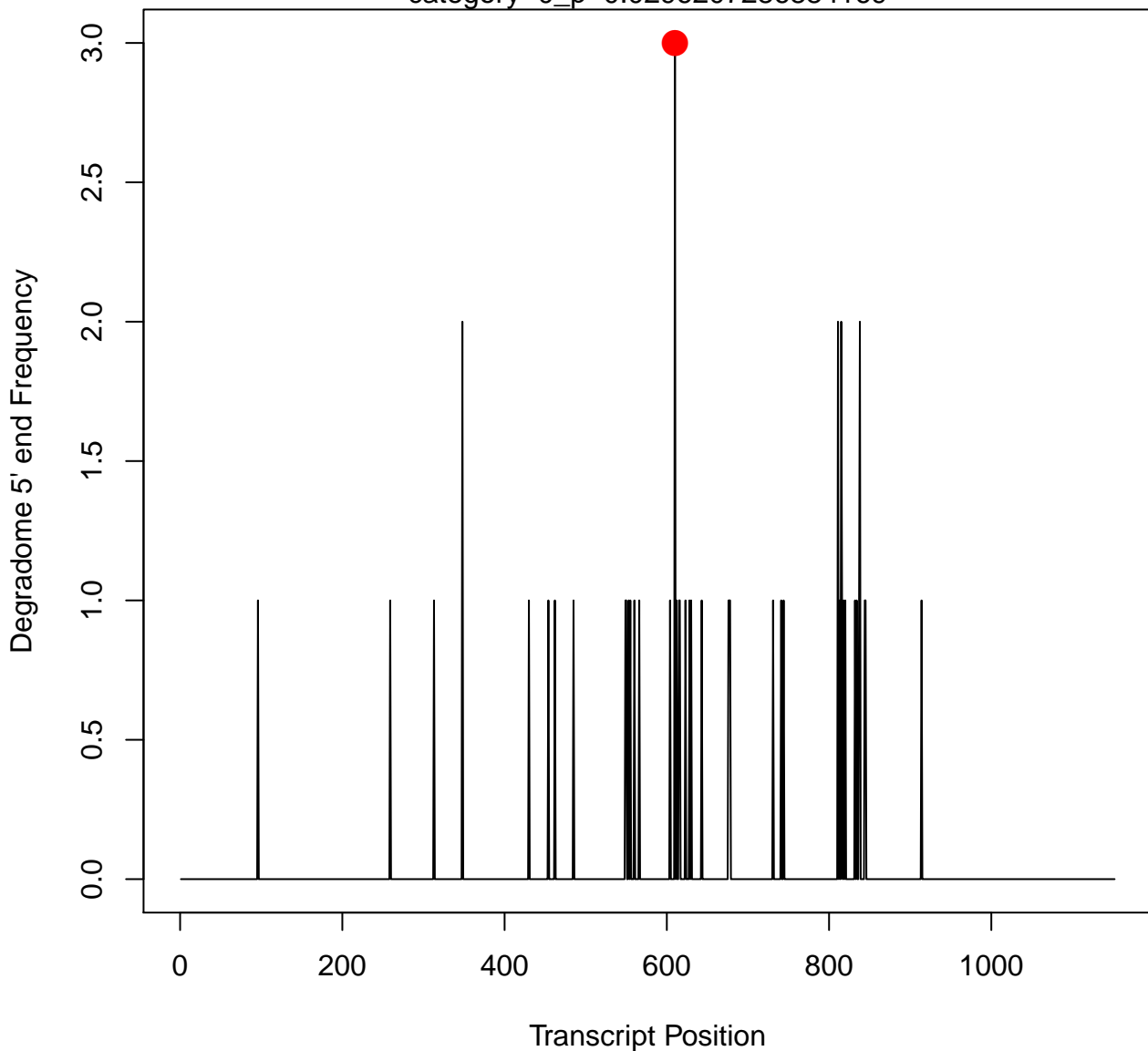

Supplement: Supplementary Data 1 — Results of categories 0–2 from PARE-Seq analysis (including three subfiles:1_1, 1_2, 1_3). [file Data_Sheet_10.ZIP › GSM2230754.plot/Lsa-miR171d_Lsat_1_v5_gn_8_122761.1_610_TPlot.pdf]

**T=Lsat\_1\_v5\_gn\_3\_55860.1\_Q=Lsa-miR171e\_S=341**

category=2\_p=0.135172542726729

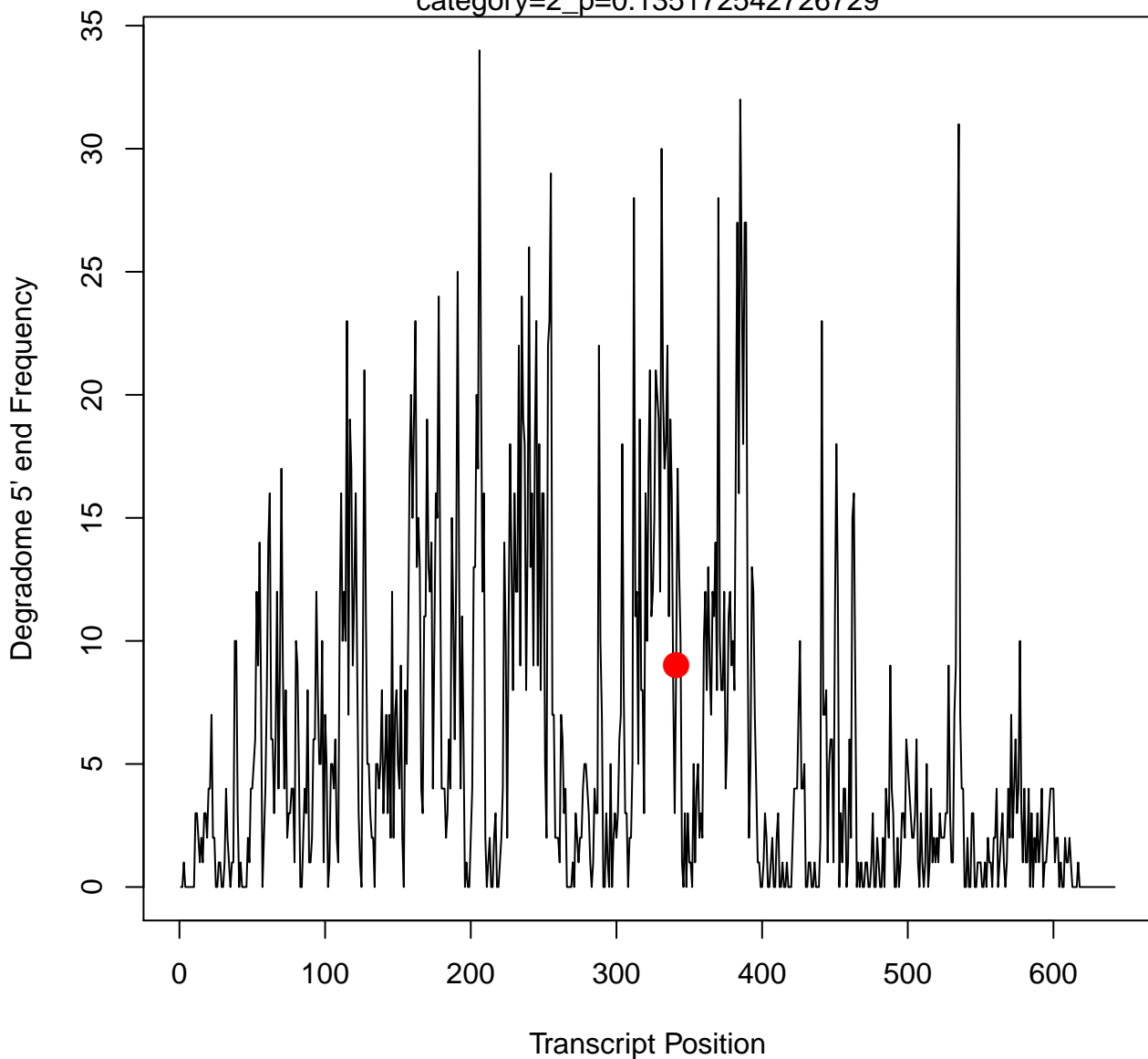

Supplement: Supplementary Data 1 — Results of categories 0–2 from PARE-Seq analysis (including three subfiles:1_1, 1_2, 1_3). [file Data_Sheet_10.ZIP › GSM2230754.plot/Lsa-miR171e_Lsat_1_v5_gn_3_55860.1_341_TPlot.pdf]

**T=Lsat\_1\_v5\_gn\_5\_45900.1\_Q=Lsa-miR171e\_S=1156**

category=2\_p=0.207339619862759

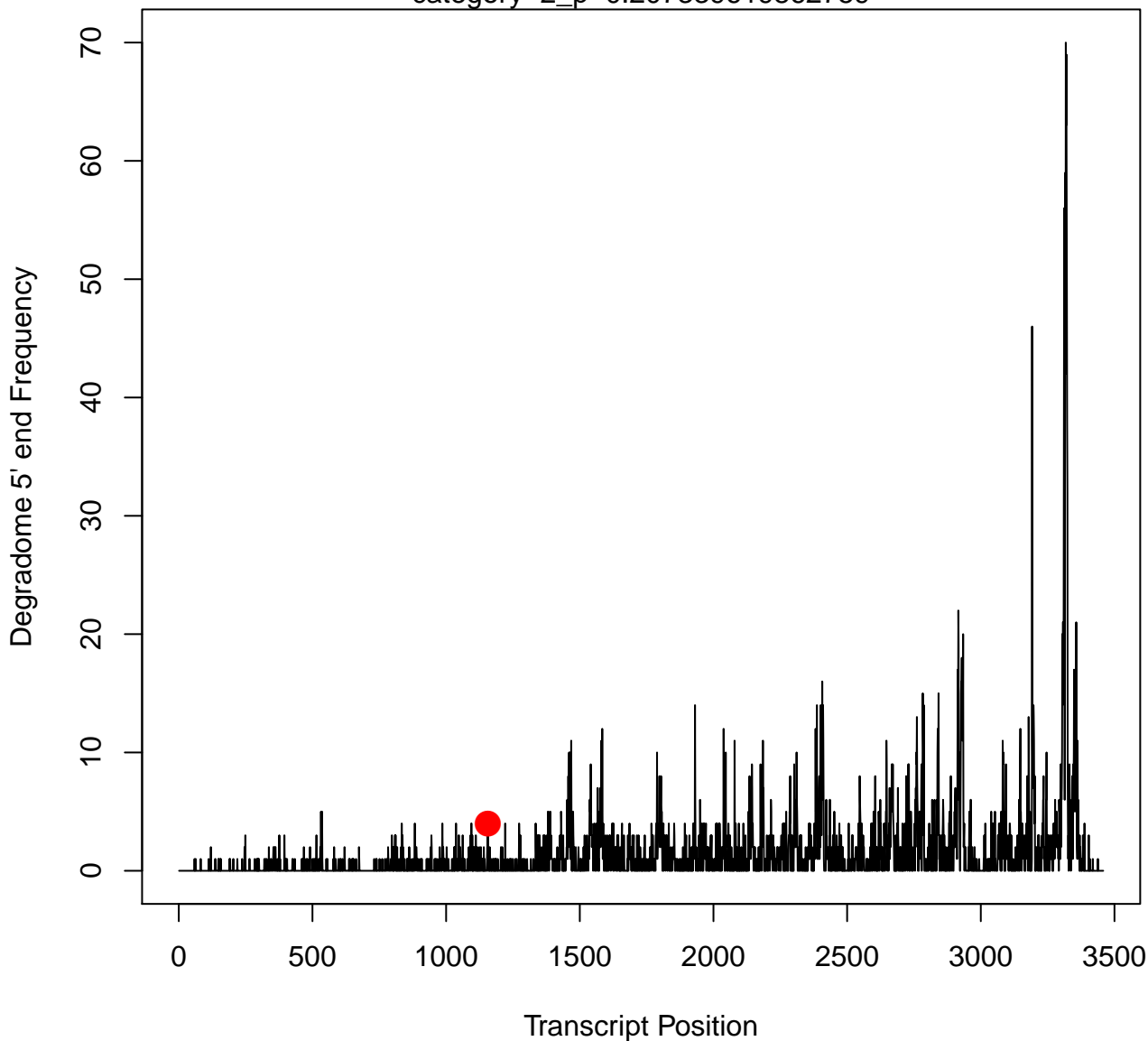

Supplement: Supplementary Data 1 — Results of categories 0–2 from PARE-Seq analysis (including three subfiles:1_1, 1_2, 1_3). [file Data_Sheet_10.ZIP › GSM2230754.plot/Lsa-miR171e_Lsat_1_v5_gn_5_45900.1_1156_TPlot.pdf]

**T=Lsat\_1\_v5\_gn\_8\_149261.1\_Q=Lsa-miR171e\_S=778**

category=2\_p=0.704738009028076

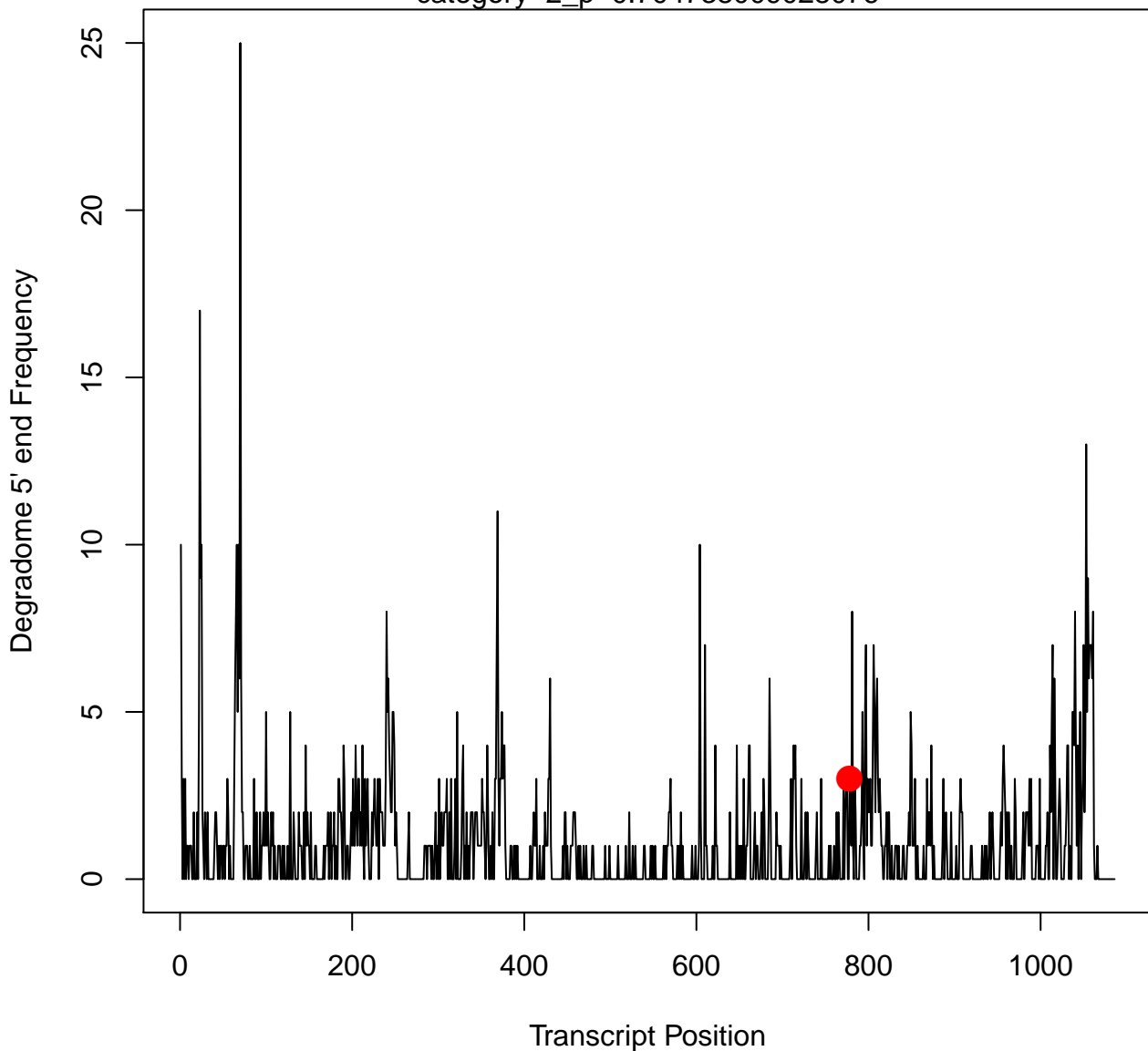

Supplement: Supplementary Data 1 — Results of categories 0–2 from PARE-Seq analysis (including three subfiles:1_1, 1_2, 1_3). [file Data_Sheet_10.ZIP › GSM2230754.plot/Lsa-miR171e_Lsat_1_v5_gn_8_149261.1_778_TPlot.pdf]

**T=Lsat\_1\_v5\_gn\_3\_36300.1\_Q=Lsa-miR171f\_S=1498**

category=2\_p=0.951232222278289

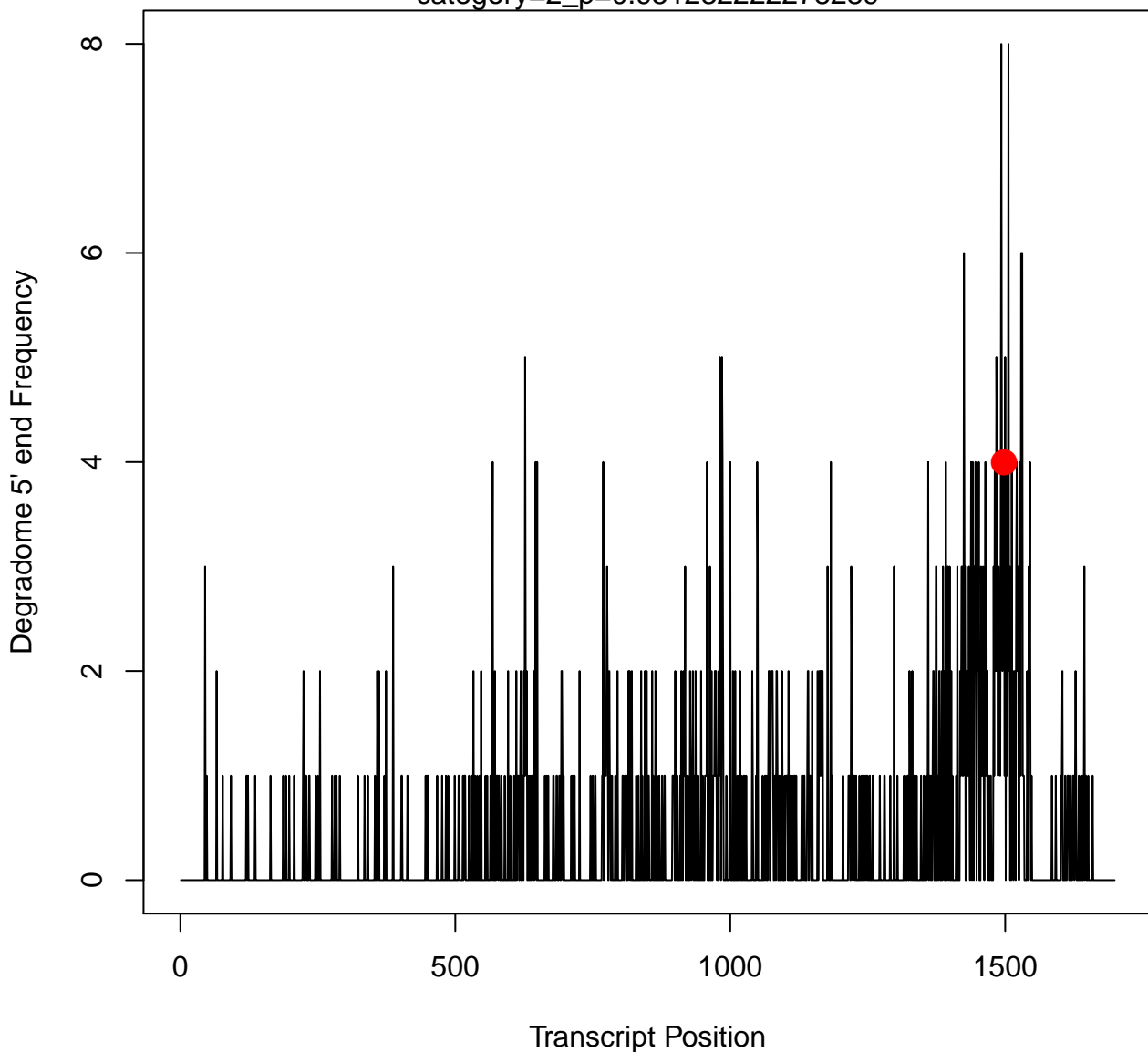

Supplement: Supplementary Data 1 — Results of categories 0–2 from PARE-Seq analysis (including three subfiles:1_1, 1_2, 1_3). [file Data_Sheet_10.ZIP › GSM2230754.plot/Lsa-miR171f_Lsat_1_v5_gn_3_36300.1_1498_TPlot.pdf]

T=Lsat\_1\_v5\_gn\_3\_78621.1\_Q=Lsa-miR171f\_S=732

category=0\_p=0.00037578290783058

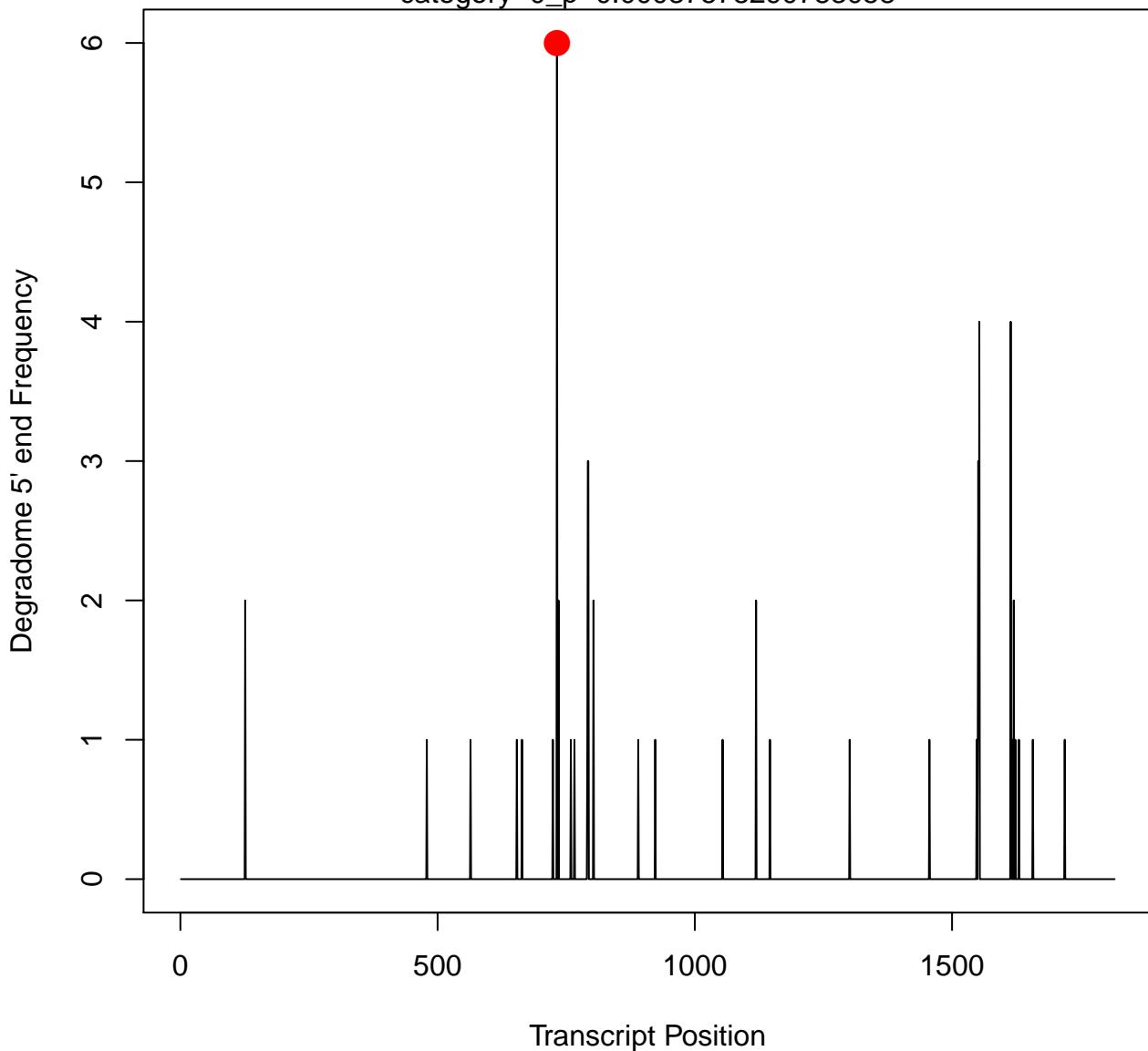

Supplement: Supplementary Data 1 — Results of categories 0–2 from PARE-Seq analysis (including three subfiles:1_1, 1_2, 1_3). [file Data_Sheet_10.ZIP › GSM2230754.plot/Lsa-miR171f_Lsat_1_v5_gn_3_78621.1_732_TPlot.pdf]

**T=Lsat\_1\_v5\_gn\_8\_110401.1\_Q=Lsa-miR171f\_S=1027**

category=2\_p=0.883438225926831

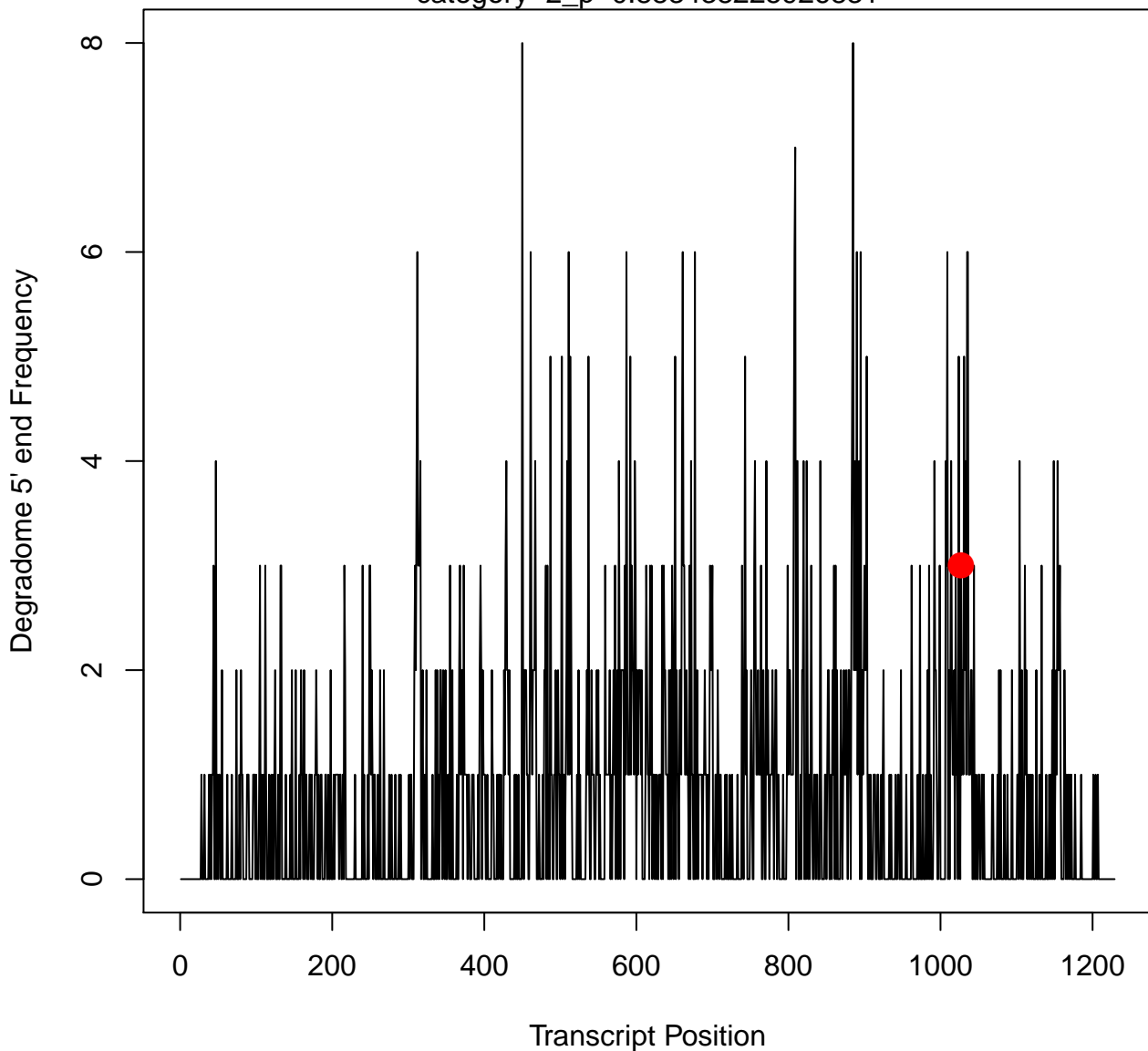

Supplement: Supplementary Data 1 — Results of categories 0–2 from PARE-Seq analysis (including three subfiles:1_1, 1_2, 1_3). [file Data_Sheet_10.ZIP › GSM2230754.plot/Lsa-miR171f_Lsat_1_v5_gn_8_110401.1_1027_TPlot.pdf]

**T=Lsat\_1\_v5\_gn\_8\_83841.1\_Q=Lsa-miR171f\_S=533**

category=2\_p=0.872825960256135

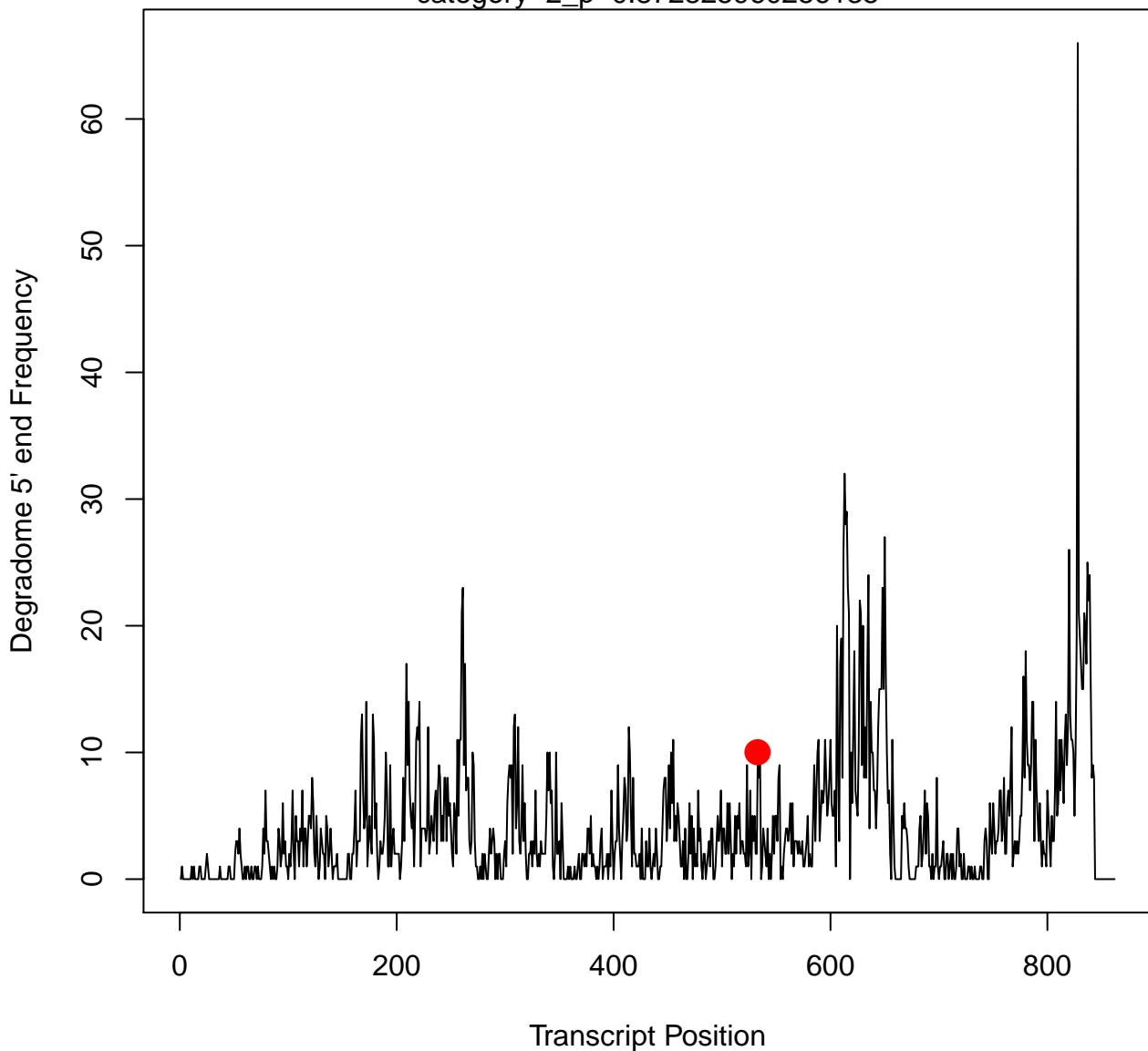

Supplement: Supplementary Data 1 — Results of categories 0–2 from PARE-Seq analysis (including three subfiles:1_1, 1_2, 1_3). [file Data_Sheet_10.ZIP › GSM2230754.plot/Lsa-miR171f_Lsat_1_v5_gn_8_83841.1_533_TPlot.pdf]

**T=Lsat\_1\_v5\_gn\_2\_109741.1\_Q=Lsa-miR172a\_S=1566**

category=0\_p=0.00112692513817547

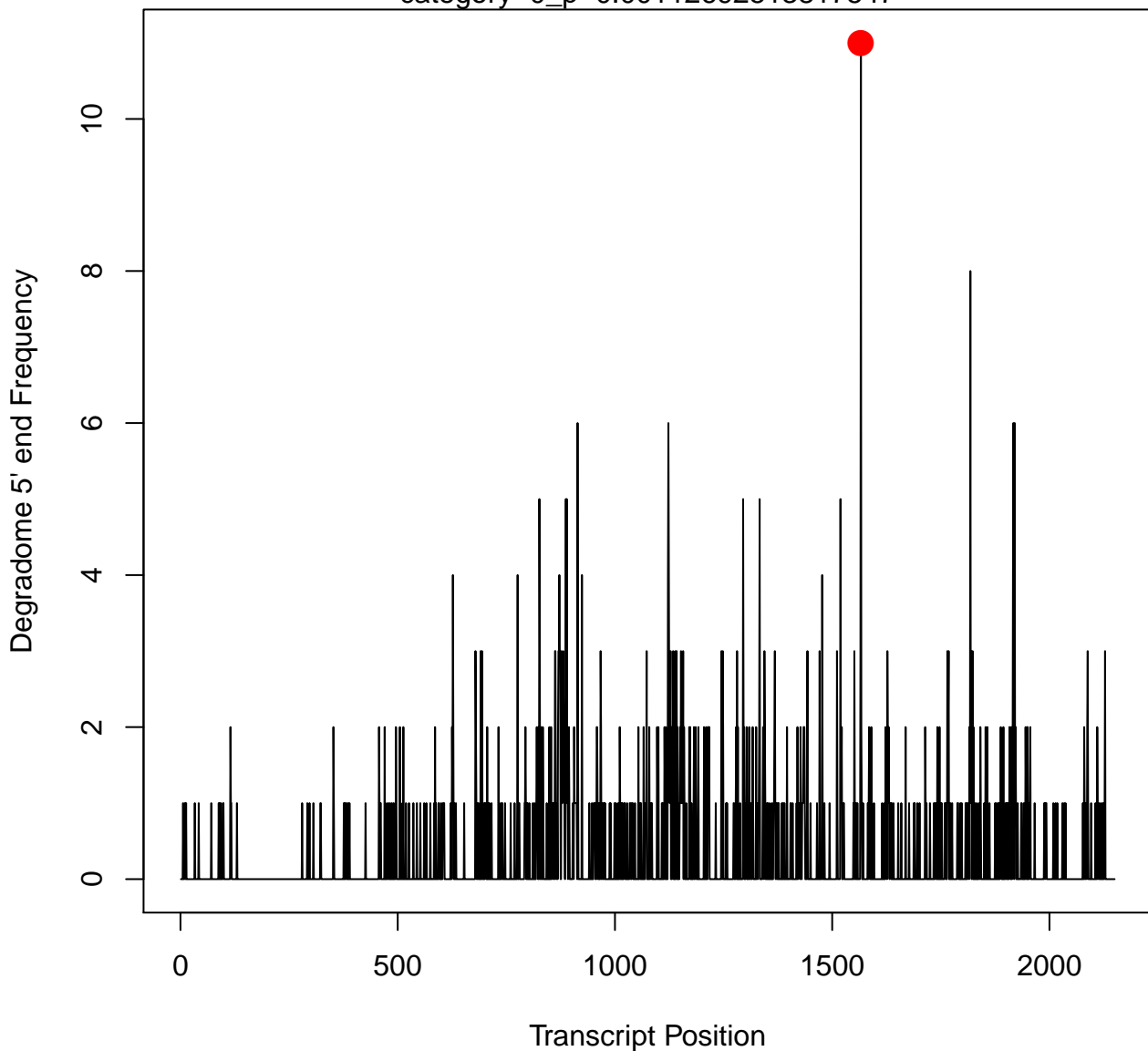

Supplement: Supplementary Data 1 — Results of categories 0–2 from PARE-Seq analysis (including three subfiles:1_1, 1_2, 1_3). [file Data_Sheet_10.ZIP › GSM2230754.plot/Lsa-miR172a_Lsat_1_v5_gn_2_109741.1_1566_TPlot.pdf]

**T=Lsat\_1\_v5\_gn\_2\_85920.1\_Q=Lsa-miR172a\_S=1664**

category=2\_p=0.159930217314119

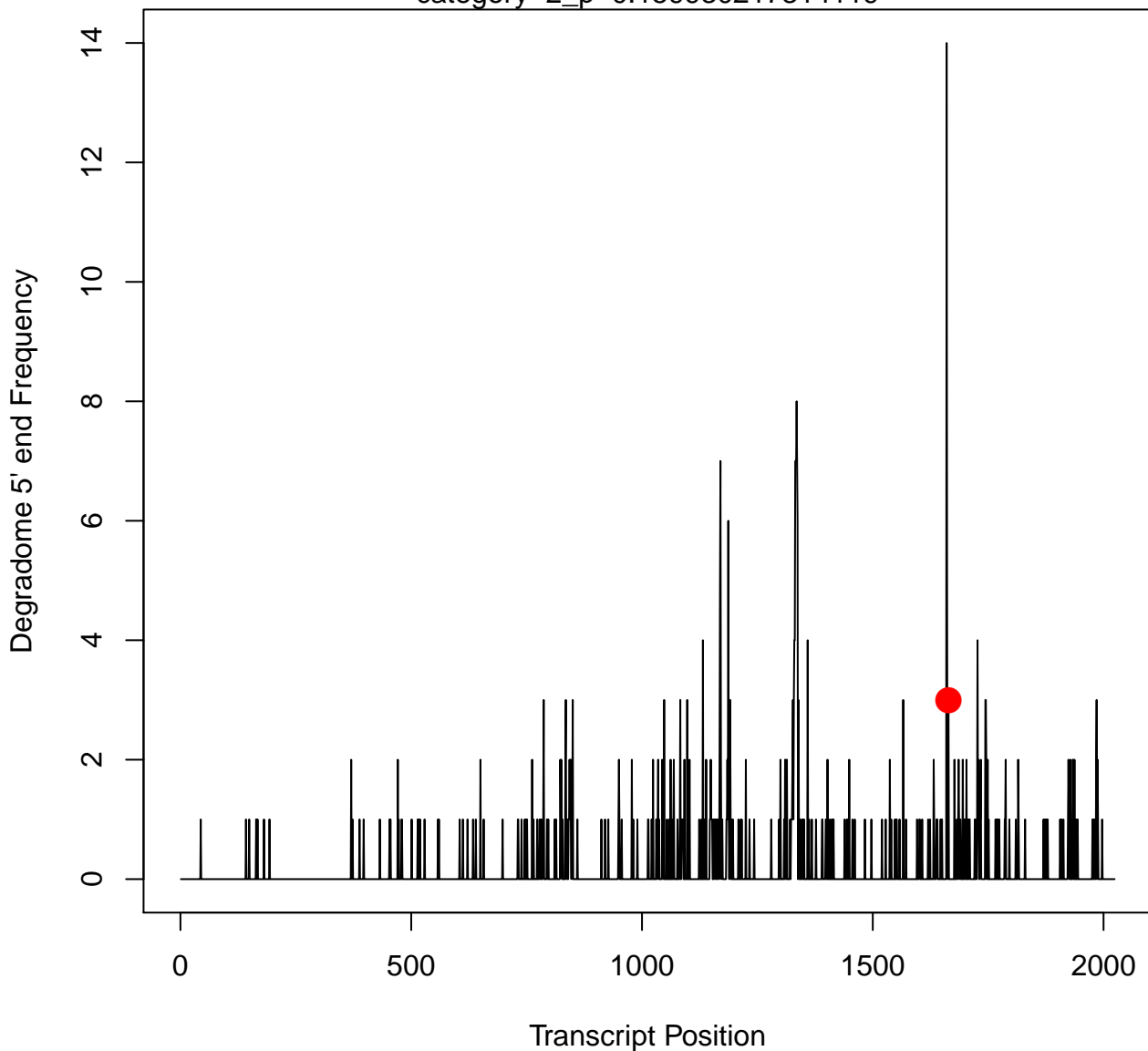

Supplement: Supplementary Data 1 — Results of categories 0–2 from PARE-Seq analysis (including three subfiles:1_1, 1_2, 1_3). [file Data_Sheet_10.ZIP › GSM2230754.plot/Lsa-miR172a_Lsat_1_v5_gn_2_85920.1_1664_TPlot.pdf]

**T=Lsat\_1\_v5\_gn\_4\_165341.1\_Q=Lsa-miR172a\_S=1632**

category=2\_p=0.183979146537541

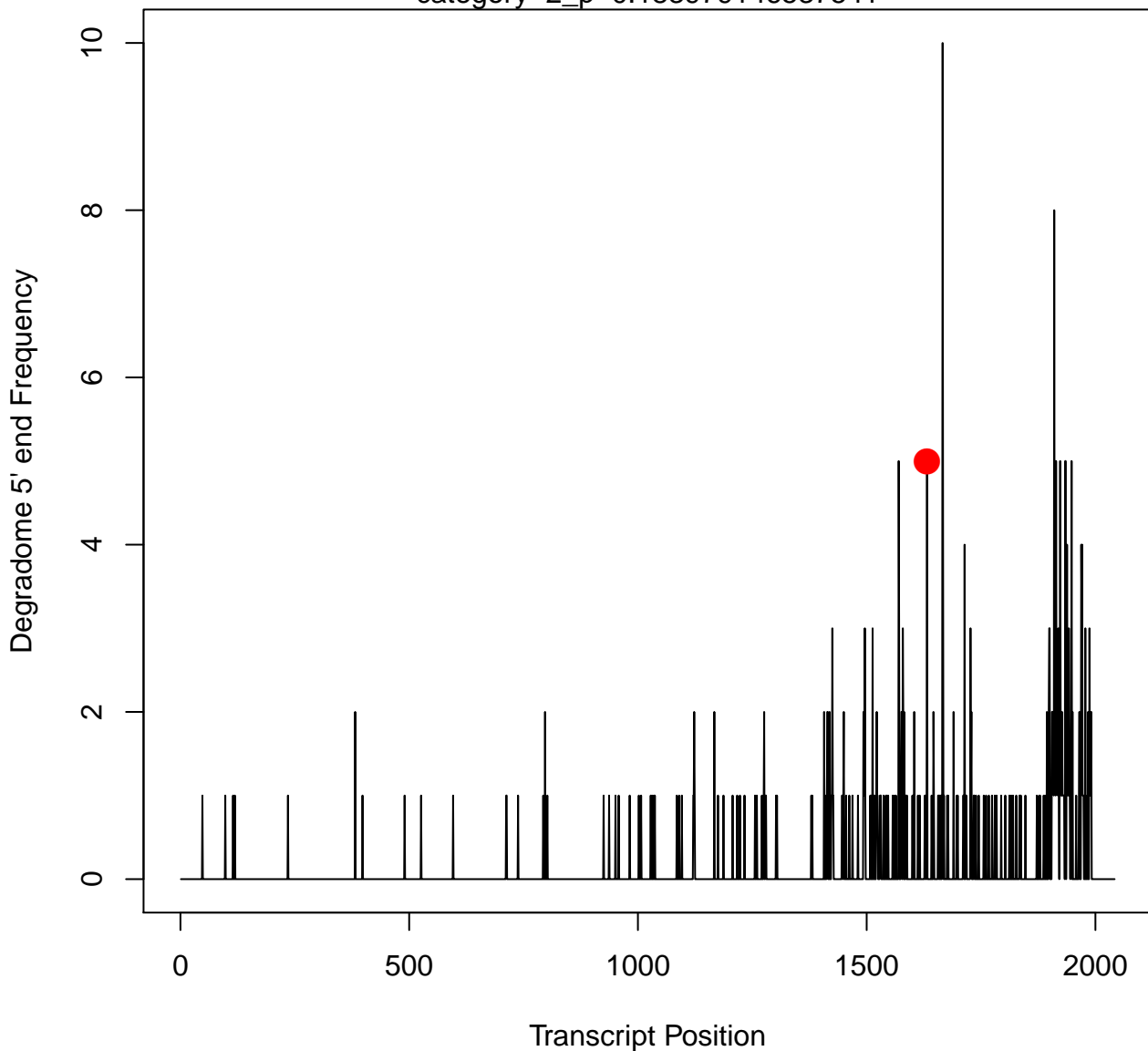

Supplement: Supplementary Data 1 — Results of categories 0–2 from PARE-Seq analysis (including three subfiles:1_1, 1_2, 1_3). [file Data_Sheet_10.ZIP › GSM2230754.plot/Lsa-miR172a_Lsat_1_v5_gn_4_165341.1_1632_TPlot.pdf]

T=Lsat\_1\_v5\_gn\_6\_29600.1\_Q=Lsa-miR172a\_S=964

category=2\_p=0.0564350766159708

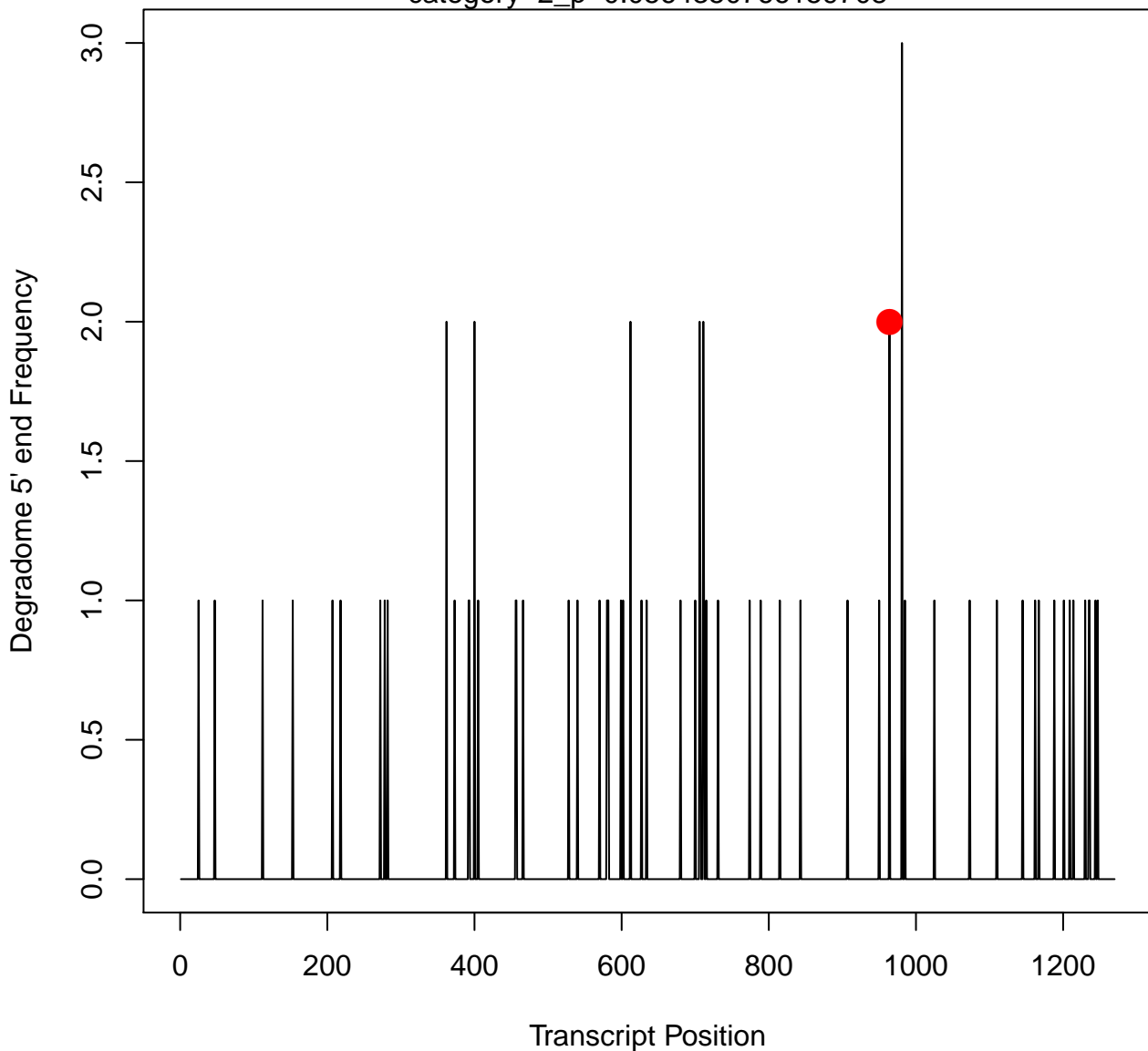

Supplement: Supplementary Data 1 — Results of categories 0–2 from PARE-Seq analysis (including three subfiles:1_1, 1_2, 1_3). [file Data_Sheet_10.ZIP › GSM2230754.plot/Lsa-miR172a_Lsat_1_v5_gn_6_29600.1_964_TPlot.pdf]

**T=Lsat\_1\_v5\_gn\_8\_31840.1\_Q=Lsa-miR172a\_S=661**

category=2\_p=0.999745914569905

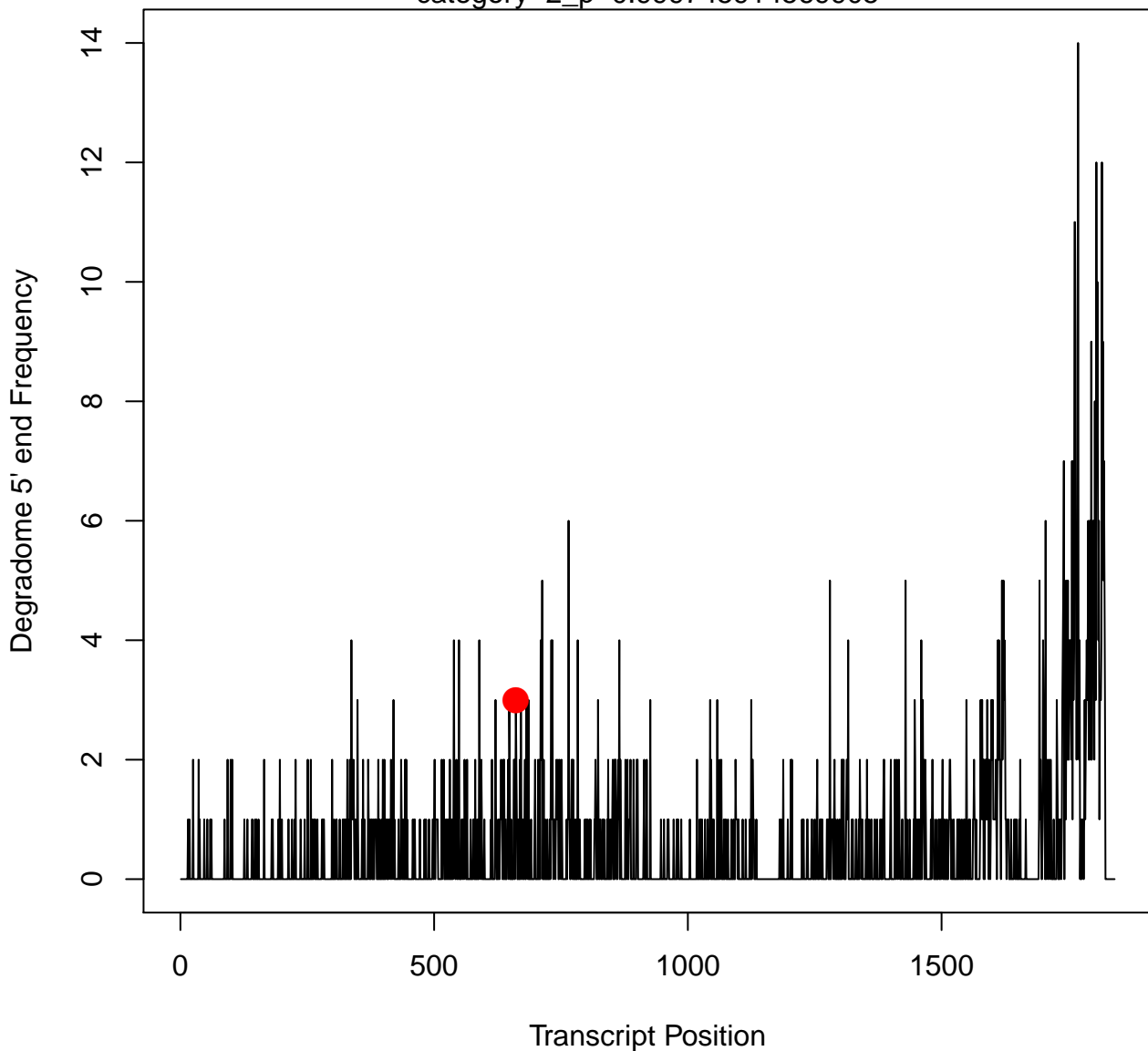

Supplement: Supplementary Data 1 — Results of categories 0–2 from PARE-Seq analysis (including three subfiles:1_1, 1_2, 1_3). [file Data_Sheet_10.ZIP › GSM2230754.plot/Lsa-miR172a_Lsat_1_v5_gn_8_31840.1_661_TPlot.pdf]

**T=Lsat\_1\_v5\_gn\_9\_20420.1\_Q=Lsa-miR172a\_S=1655**

category=2\_p=0.135172542726729

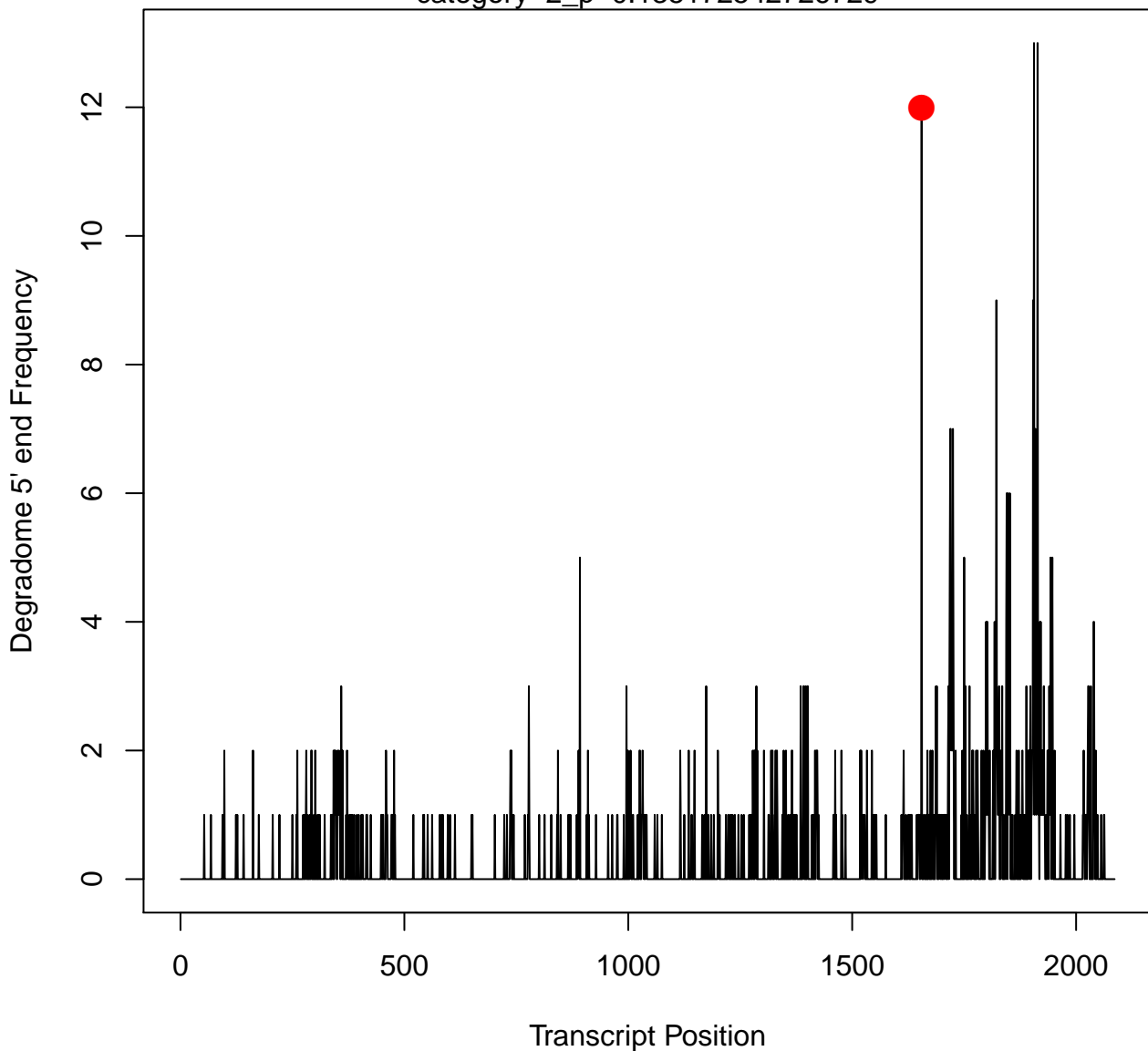

Supplement: Supplementary Data 1 — Results of categories 0–2 from PARE-Seq analysis (including three subfiles:1_1, 1_2, 1_3). [file Data_Sheet_10.ZIP › GSM2230754.plot/Lsa-miR172a_Lsat_1_v5_gn_9_20420.1_1655_TPlot.pdf]

**T=Lsat\_1\_v5\_gn\_9\_70241.1\_Q=Lsa-miR172a\_S=1426**

category=2\_p=0.999951473863596

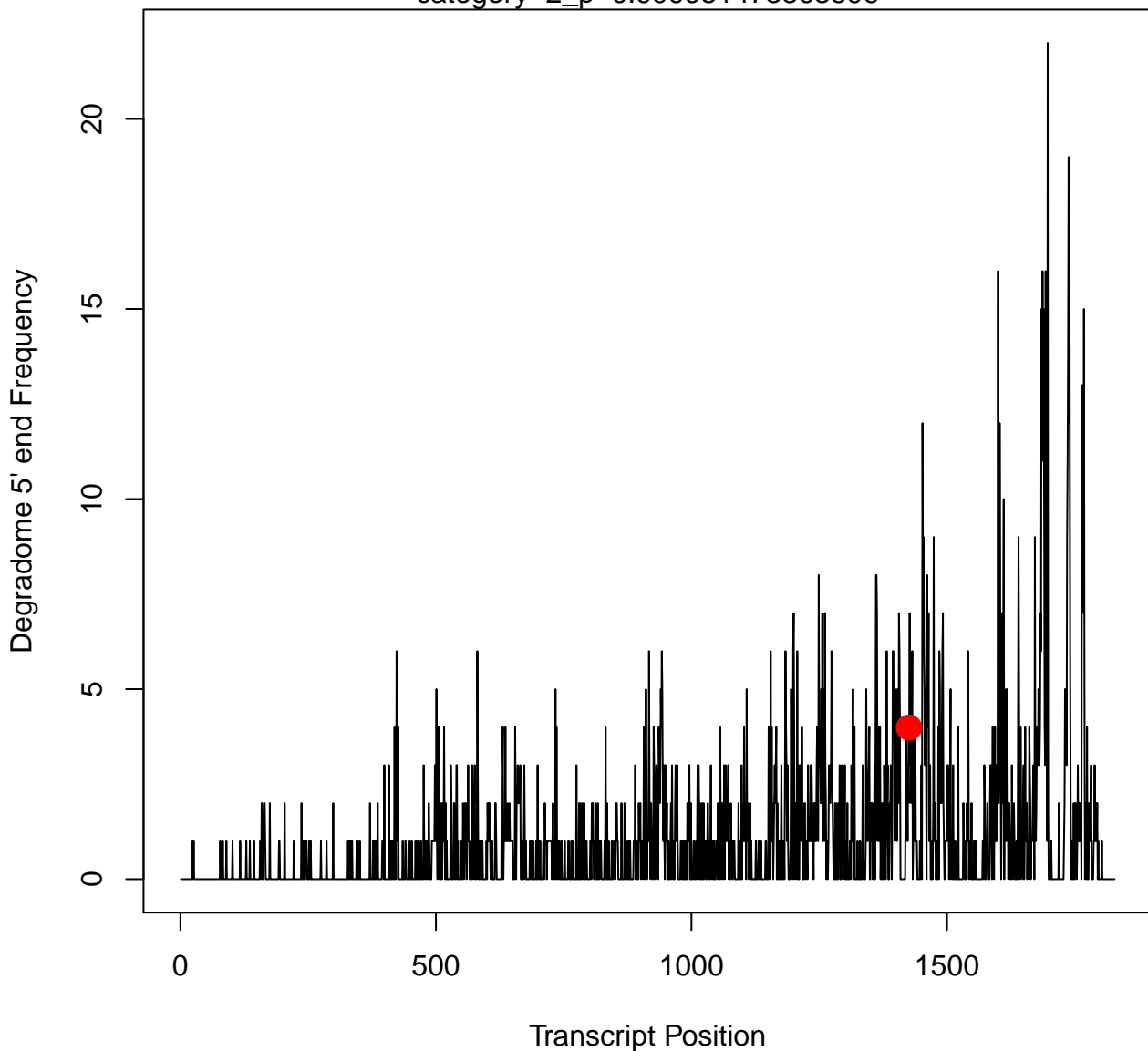

Supplement: Supplementary Data 1 — Results of categories 0–2 from PARE-Seq analysis (including three subfiles:1_1, 1_2, 1_3). [file Data_Sheet_10.ZIP › GSM2230754.plot/Lsa-miR172a_Lsat_1_v5_gn_9_70241.1_1426_TPlot.pdf]

**T=Lsat\_1\_v5\_gn\_3\_126700.1\_Q=Lsa-miR172b\_S=453**

category=2\_p=0.963525285191154

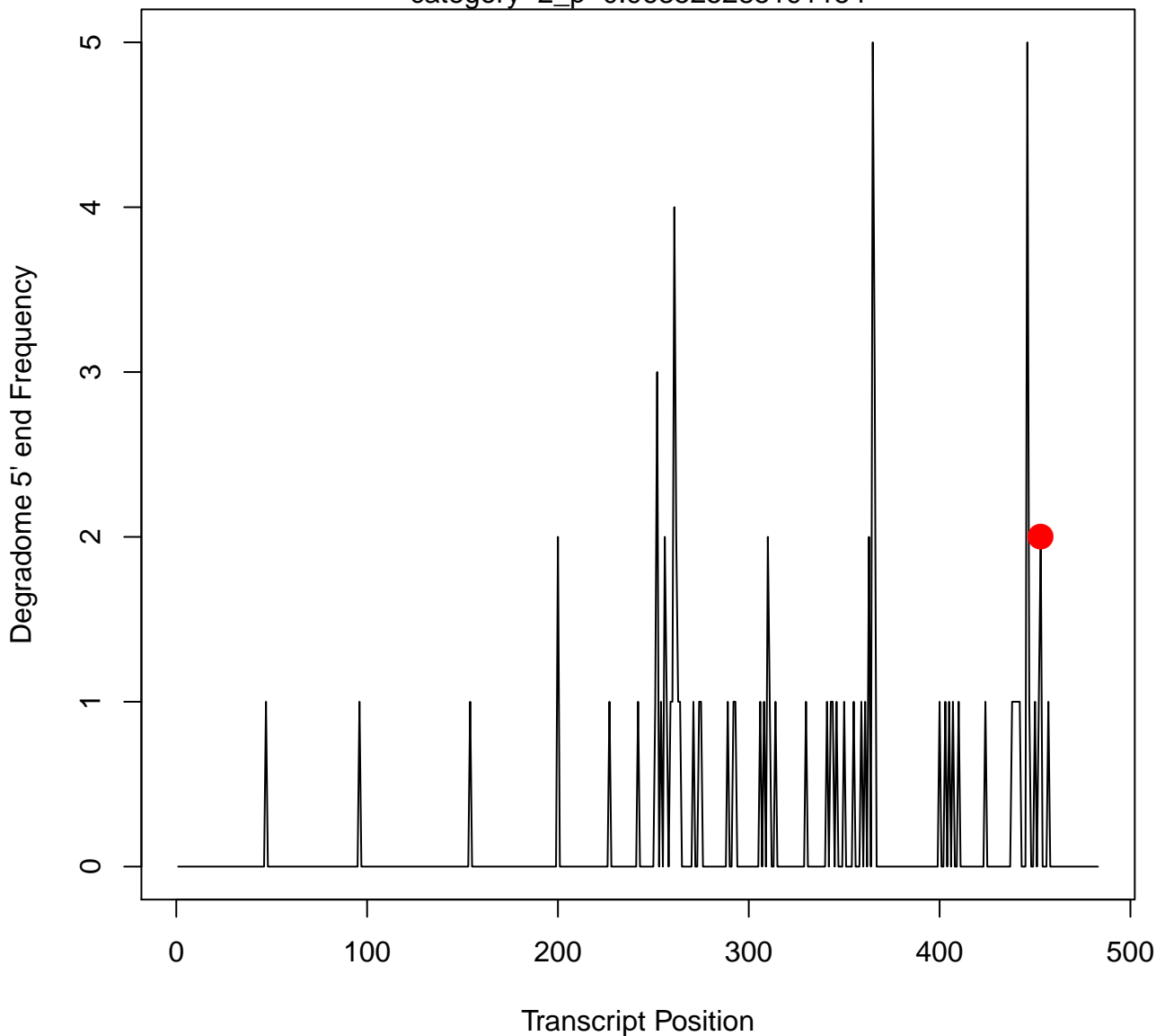

Supplement: Supplementary Data 1 — Results of categories 0–2 from PARE-Seq analysis (including three subfiles:1_1, 1_2, 1_3). [file Data_Sheet_10.ZIP › GSM2230754.plot/Lsa-miR172b_Lsat_1_v5_gn_3_126700.1_453_TPlot.pdf]

**T=Lsat\_1\_v5\_gn\_4\_105921.1\_Q=Lsa-miR172b\_S=770**

category=2\_p=0.992175721837987

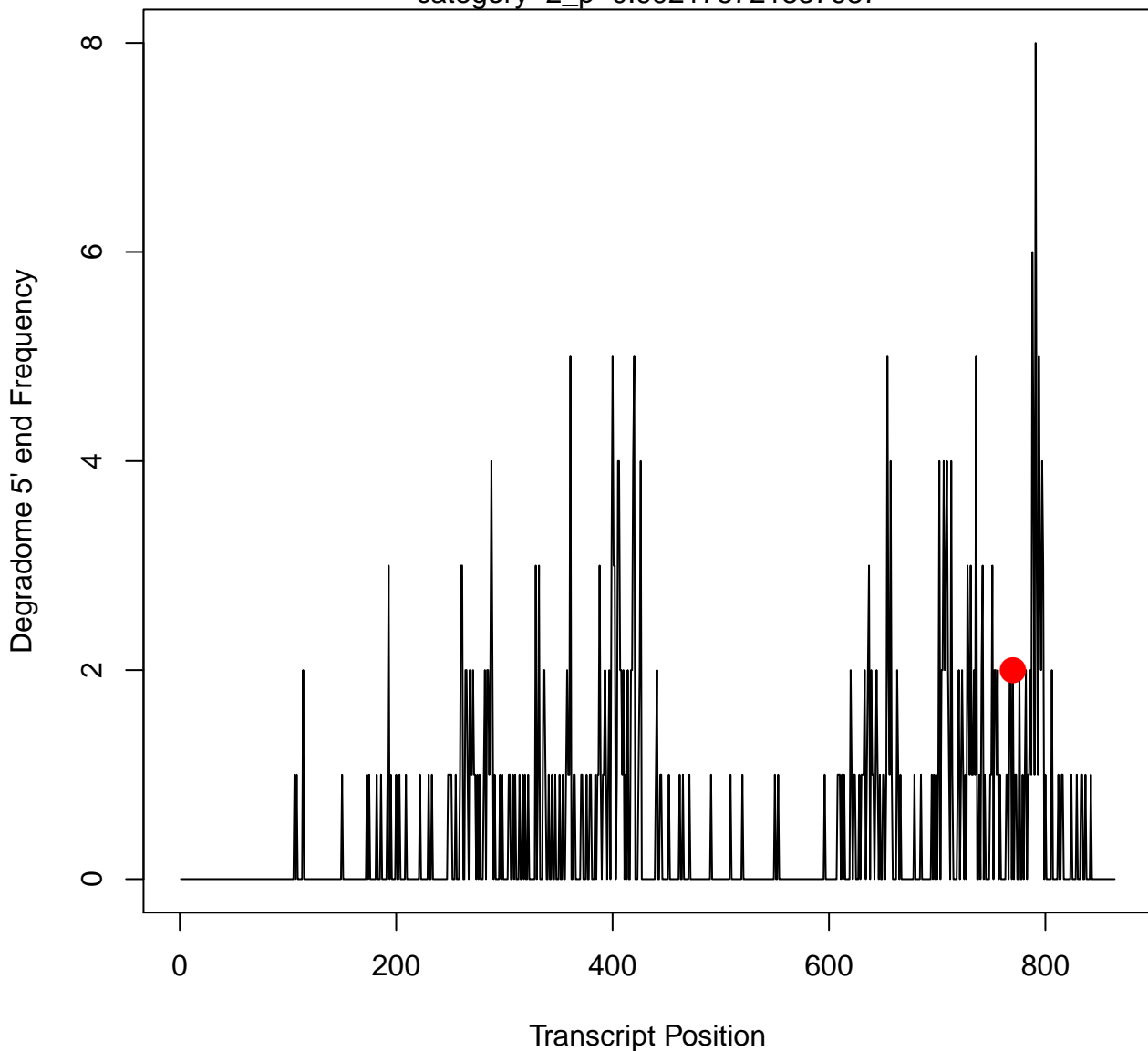

Supplement: Supplementary Data 1 — Results of categories 0–2 from PARE-Seq analysis (including three subfiles:1_1, 1_2, 1_3). [file Data_Sheet_10.ZIP › GSM2230754.plot/Lsa-miR172b_Lsat_1_v5_gn_4_105921.1_770_TPlot.pdf]

**T=Lsat\_1\_v5\_gn\_4\_124961.1\_Q=Lsa-miR172b\_S=407**

category=2\_p=0.999884015782297

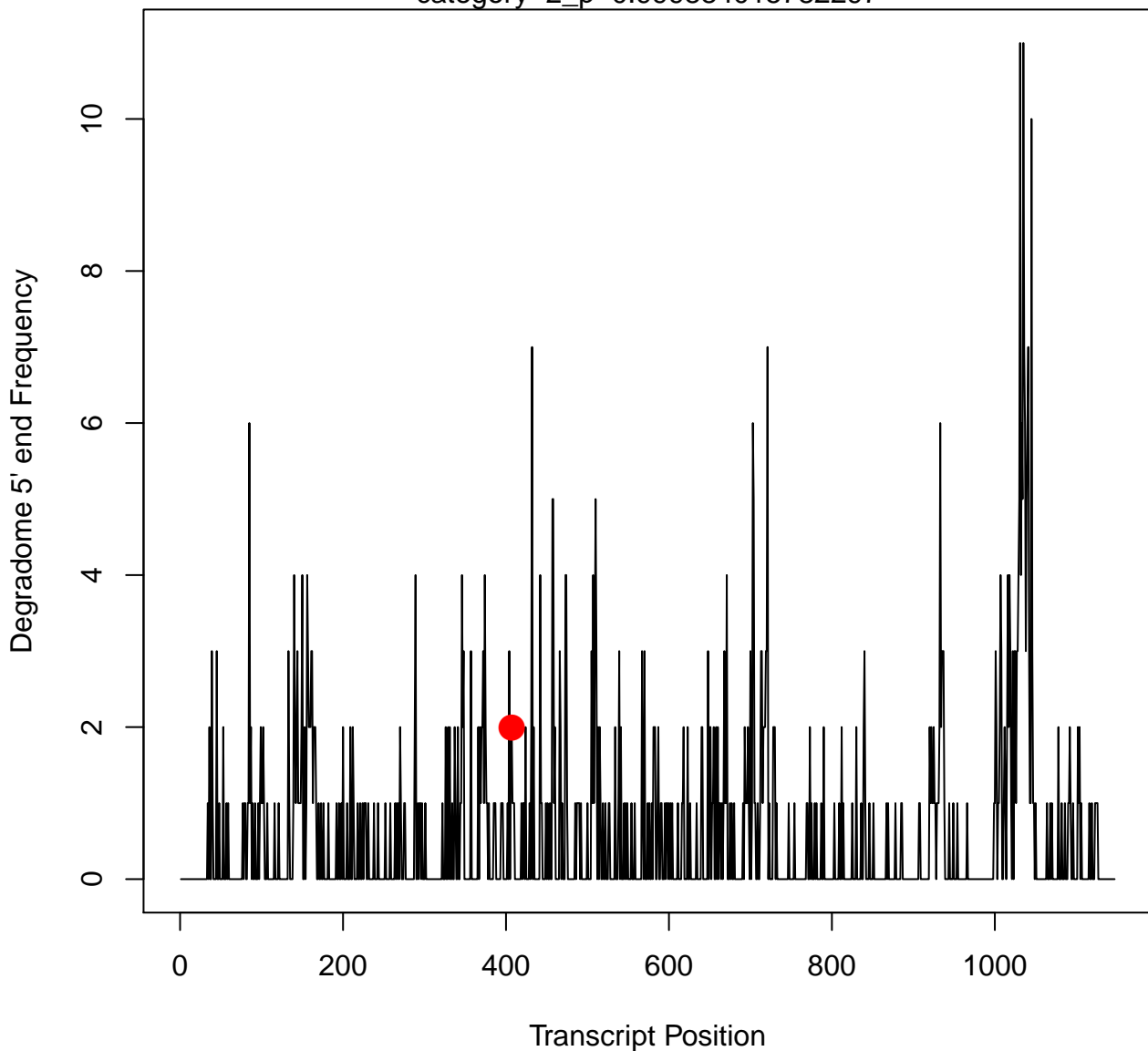

Supplement: Supplementary Data 1 — Results of categories 0–2 from PARE-Seq analysis (including three subfiles:1_1, 1_2, 1_3). [file Data_Sheet_10.ZIP › GSM2230754.plot/Lsa-miR172b_Lsat_1_v5_gn_4_124961.1_407_TPlot.pdf]

**T=Lsat\_1\_v5\_gn\_5\_2120.1\_Q=Lsa-miR172b\_S=1175**

category=2\_p=0.97271959308914

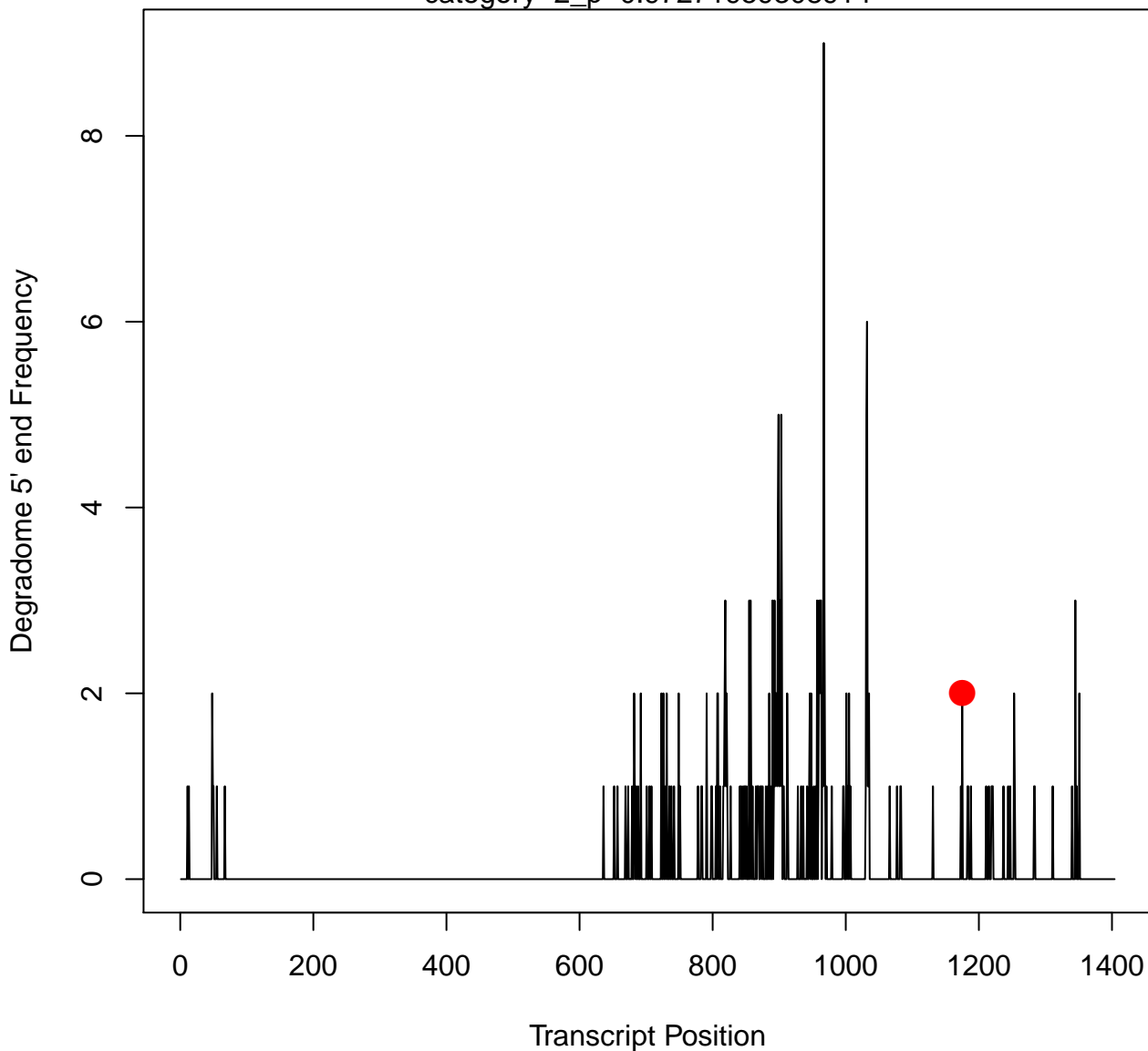

Supplement: Supplementary Data 1 — Results of categories 0–2 from PARE-Seq analysis (including three subfiles:1_1, 1_2, 1_3). [file Data_Sheet_10.ZIP › GSM2230754.plot/Lsa-miR172b_Lsat_1_v5_gn_5_2120.1_1175_TPlot.pdf]

**T=Lsat\_1\_v5\_gn\_4\_161761.1\_Q=Lsa-miR172c\_S=85**

category=1\_p=0.0511935326031917

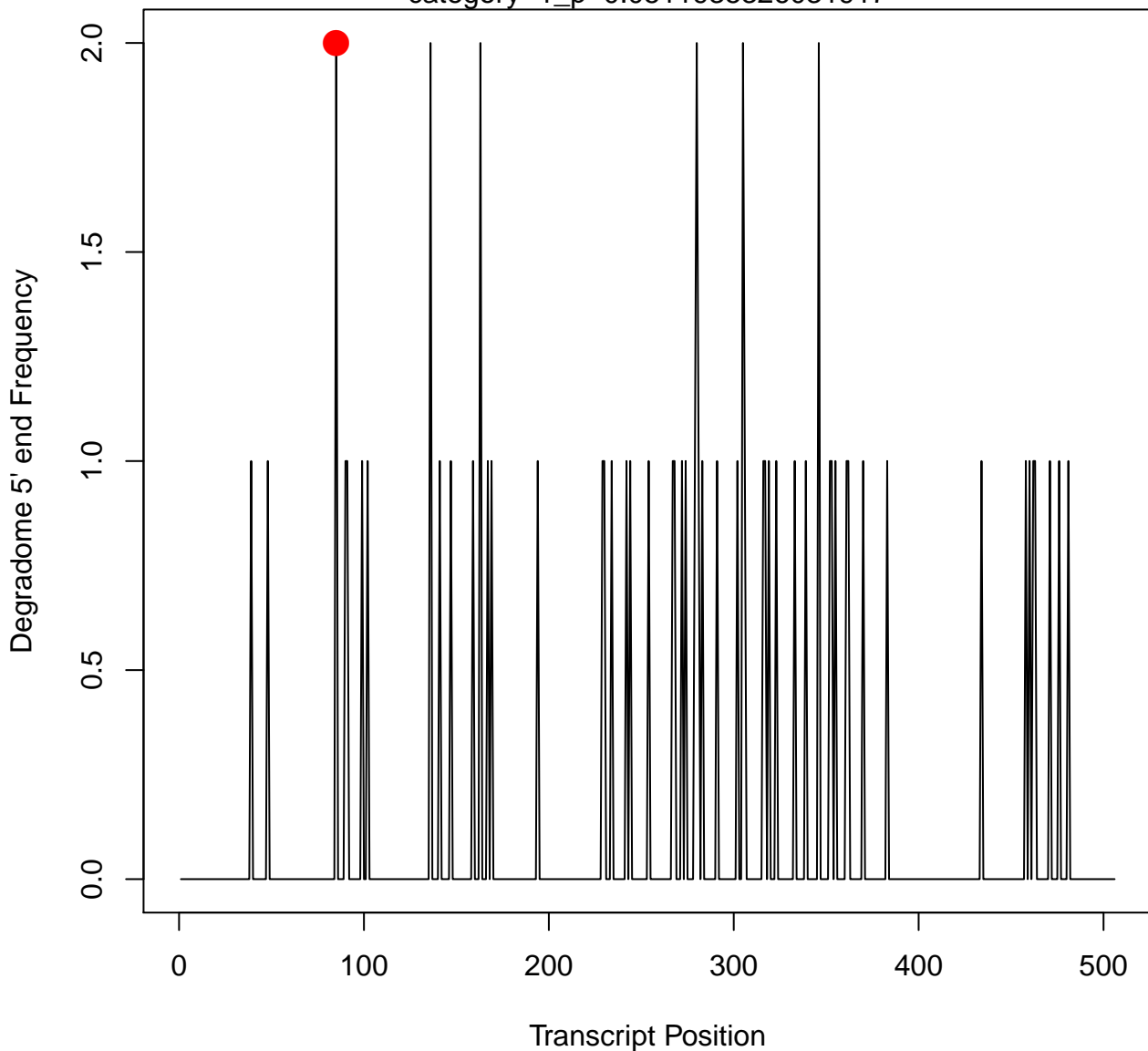

Supplement: Supplementary Data 1 — Results of categories 0–2 from PARE-Seq analysis (including three subfiles:1_1, 1_2, 1_3). [file Data_Sheet_10.ZIP › GSM2230754.plot/Lsa-miR172c_Lsat_1_v5_gn_4_161761.1_85_TPlot.pdf]

**T=Lsat\_1\_v5\_gn\_8\_18040.1\_Q=Lsa-miR172c\_S=1210**

category=0\_p=0.00037578290783058

Degradome 5' end Frequency

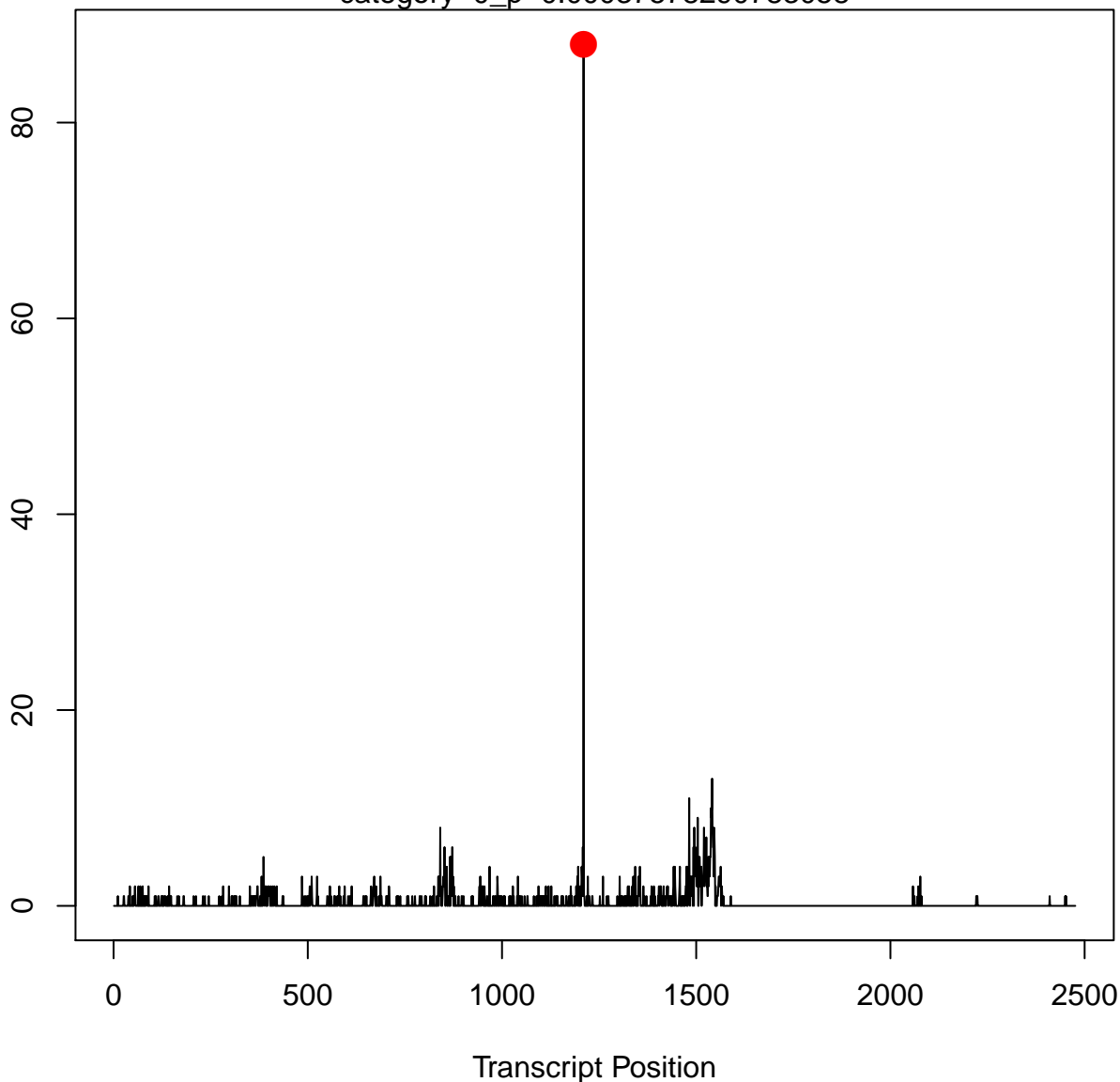

Supplement: Supplementary Data 1 — Results of categories 0–2 from PARE-Seq analysis (including three subfiles:1_1, 1_2, 1_3). [file Data_Sheet_10.ZIP › GSM2230754.plot/Lsa-miR172c_Lsat_1_v5_gn_8_18040.1_1210_TPlot.pdf]

**T=Lsat\_1\_v5\_gn\_9\_64940.1\_Q=Lsa-miR172c\_S=775**

category=2\_p=0.999954212439822

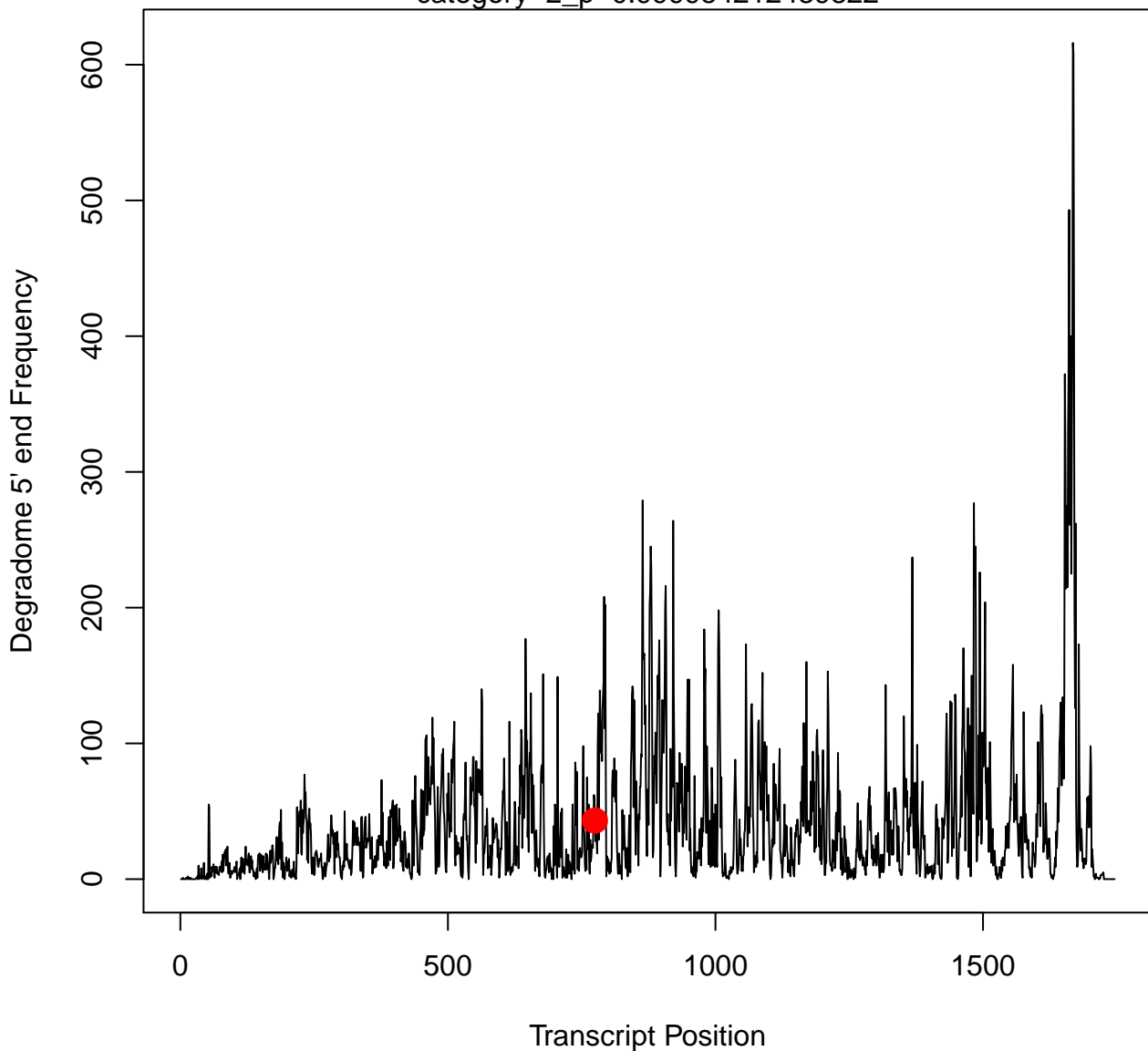

Supplement: Supplementary Data 1 — Results of categories 0–2 from PARE-Seq analysis (including three subfiles:1_1, 1_2, 1_3). [file Data_Sheet_10.ZIP › GSM2230754.plot/Lsa-miR172c_Lsat_1_v5_gn_9_64940.1_775_TPlot.pdf]

**T=Lsat\_1\_v5\_gn\_0\_12040.1\_Q=Lsa-miR1871\_S=2235**

category=2\_p=0.975711850941855

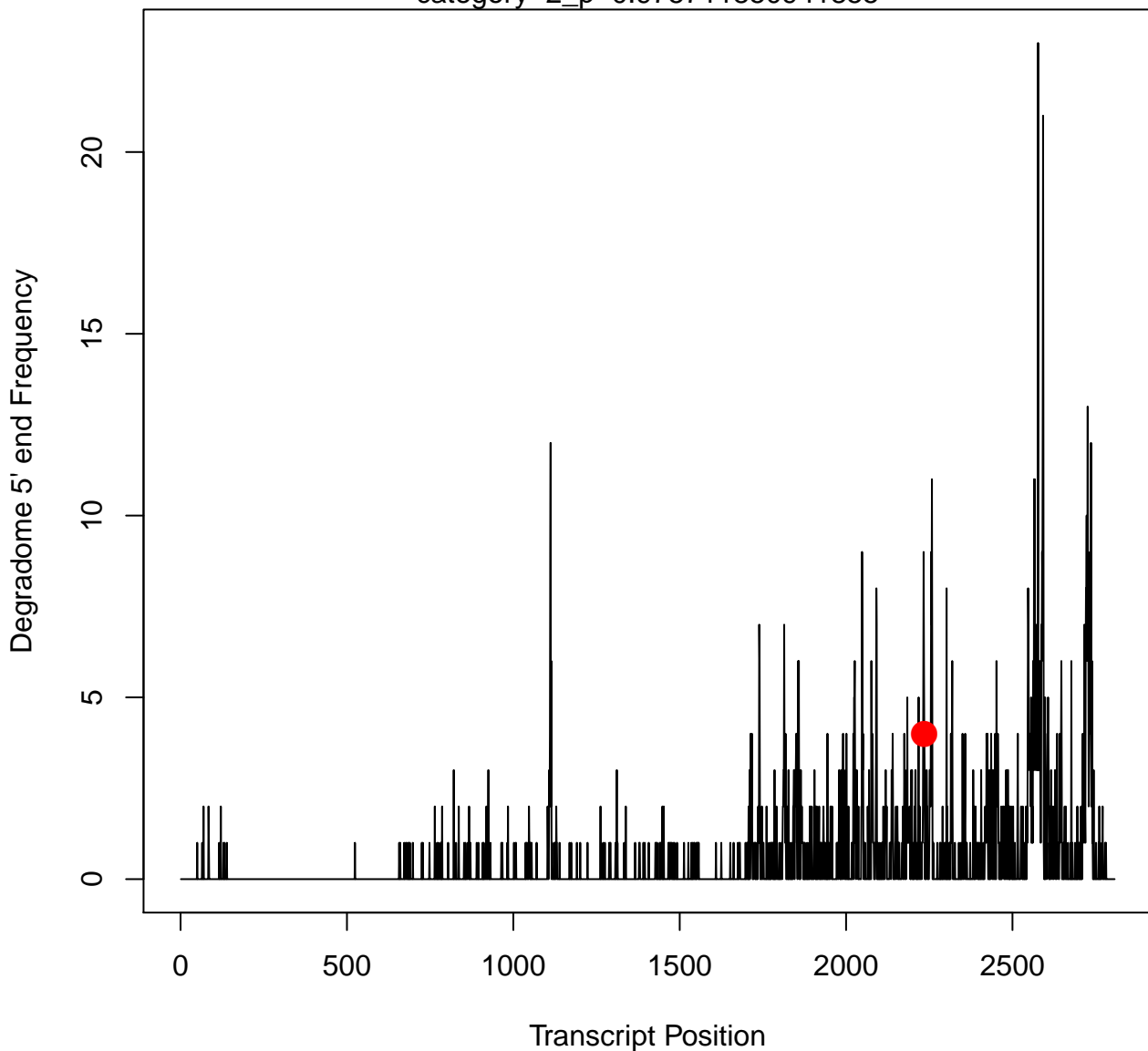

Supplement: Supplementary Data 1 — Results of categories 0–2 from PARE-Seq analysis (including three subfiles:1_1, 1_2, 1_3). [file Data_Sheet_10.ZIP › GSM2230754.plot/Lsa-miR1871_Lsat_1_v5_gn_0_12040.1_2235_TPlot.pdf]

**T=Lsat\_1\_v5\_gn\_5\_173401.1\_Q=Lsa-miR1871\_S=567**

category=2\_p=0.995224658024572

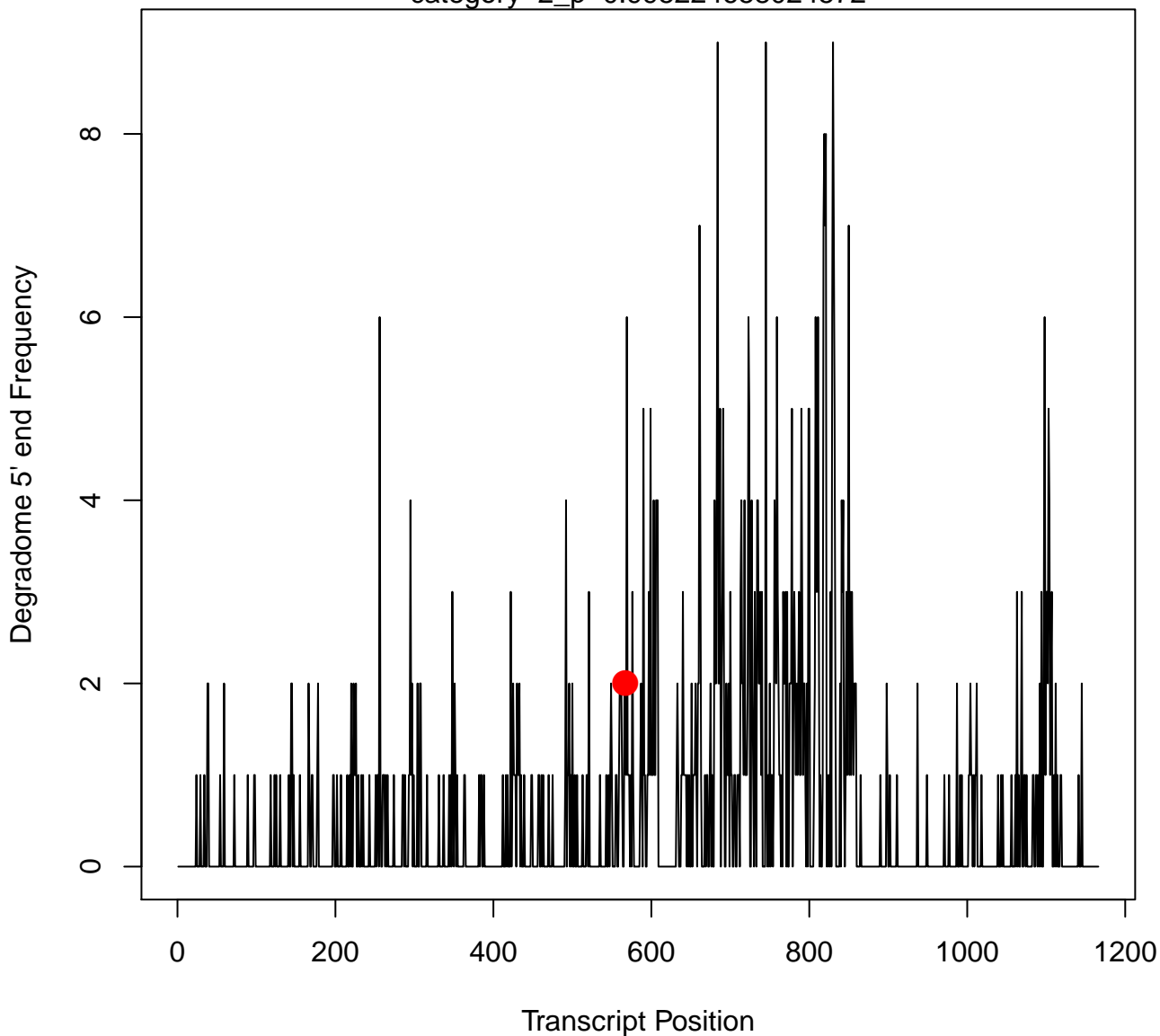

Supplement: Supplementary Data 1 — Results of categories 0–2 from PARE-Seq analysis (including three subfiles:1_1, 1_2, 1_3). [file Data_Sheet_10.ZIP › GSM2230754.plot/Lsa-miR1871_Lsat_1_v5_gn_5_173401.1_567_TPlot.pdf]

**T=Lsat\_1\_v5\_gn\_6\_14580.1\_Q=Lsa-miR1871\_S=1632**

category=2\_p=0.953984435550404

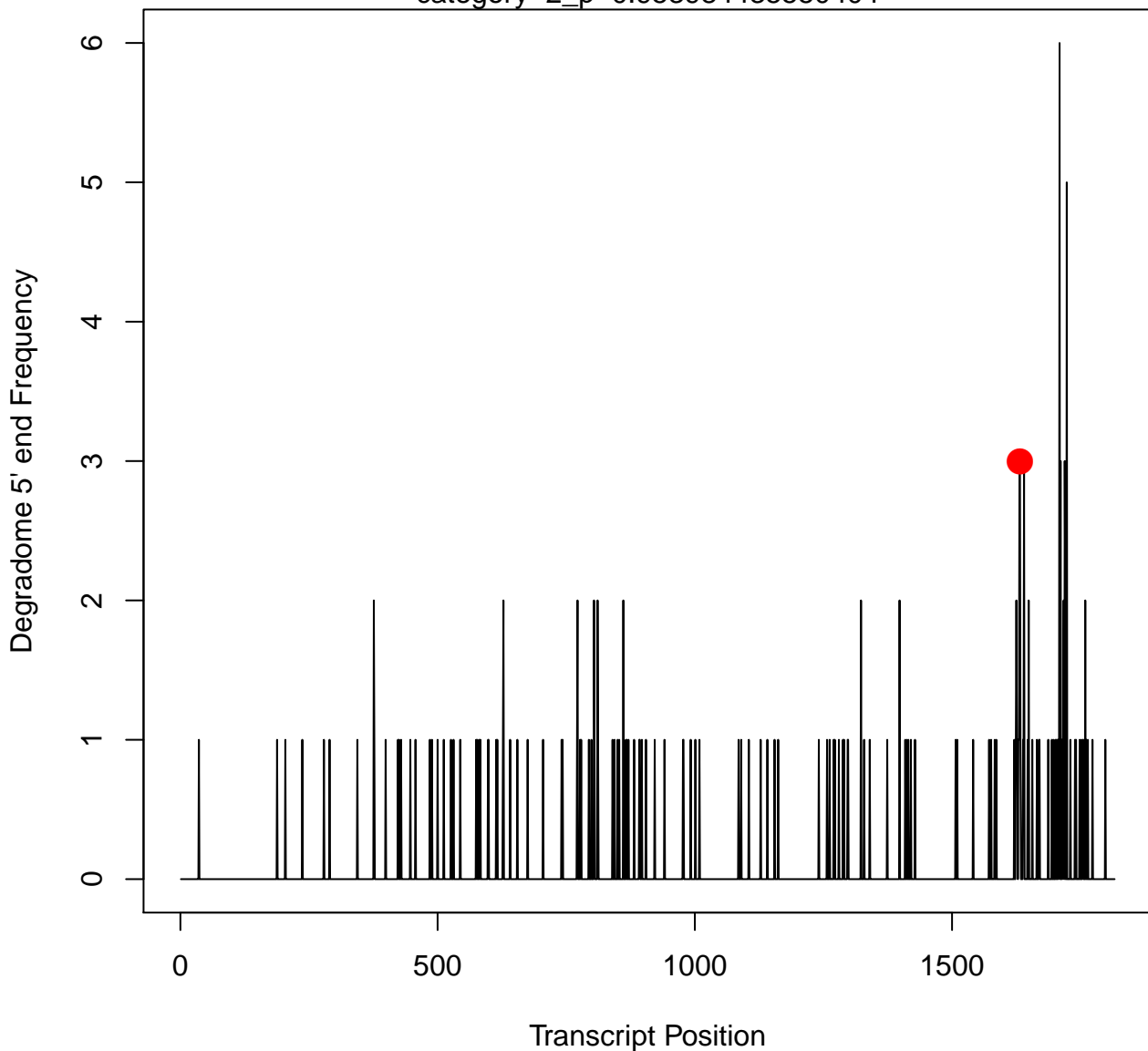

Supplement: Supplementary Data 1 — Results of categories 0–2 from PARE-Seq analysis (including three subfiles:1_1, 1_2, 1_3). [file Data_Sheet_10.ZIP › GSM2230754.plot/Lsa-miR1871_Lsat_1_v5_gn_6_14580.1_1632_TPlot.pdf]

**T=Lsat\_1\_v5\_gn\_8\_115261.1\_Q=Lsa-miR1871\_S=1024**

category=2\_p=0.995988379504452

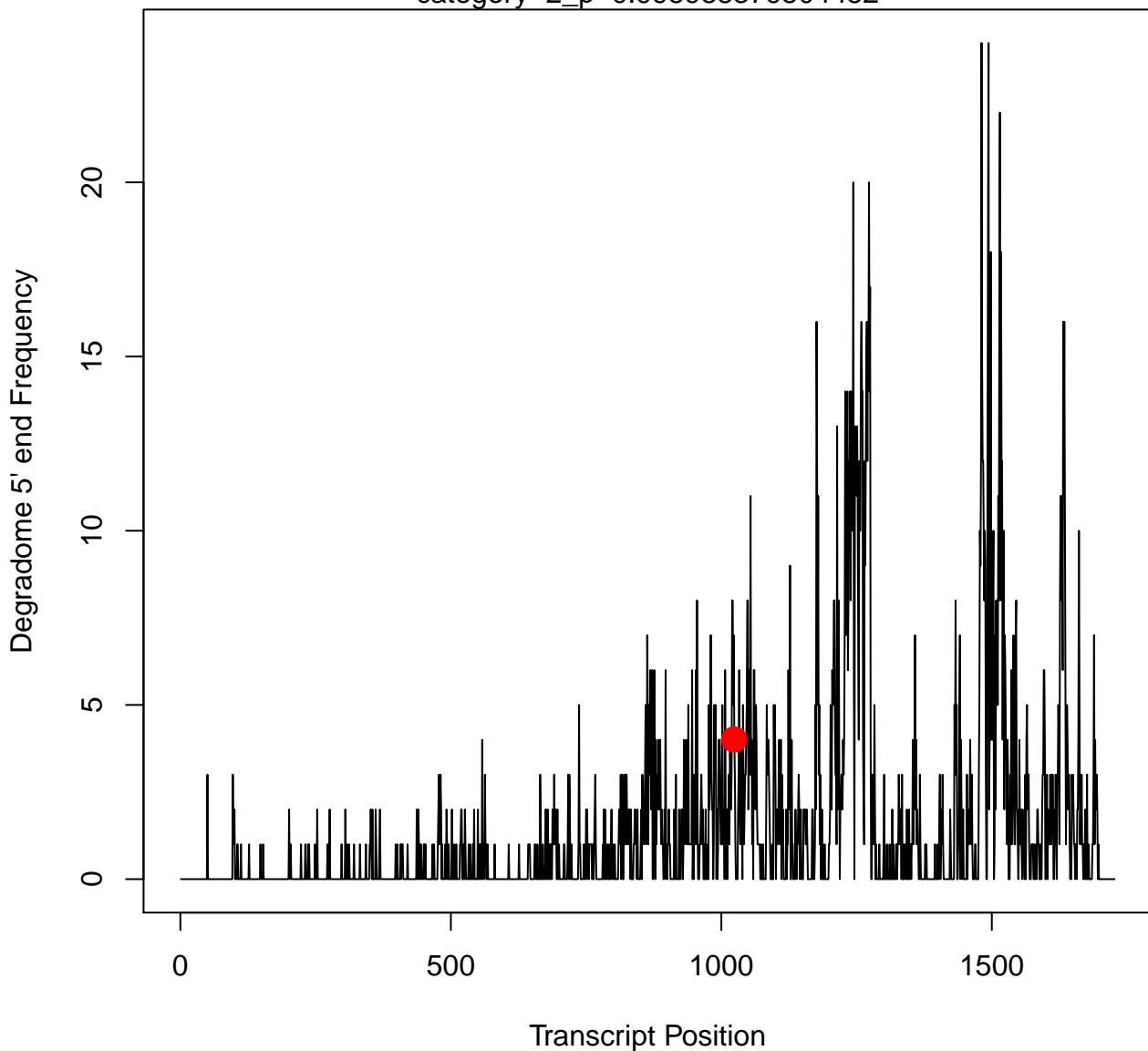

Supplement: Supplementary Data 1 — Results of categories 0–2 from PARE-Seq analysis (including three subfiles:1_1, 1_2, 1_3). [file Data_Sheet_10.ZIP › GSM2230754.plot/Lsa-miR1871_Lsat_1_v5_gn_8_115261.1_1024_TPlot.pdf]

**T=Lsat\_1\_v5\_gn\_8\_27820.1\_Q=Lsa-miR1871\_S=199**

category=2\_p=0.932874407375598

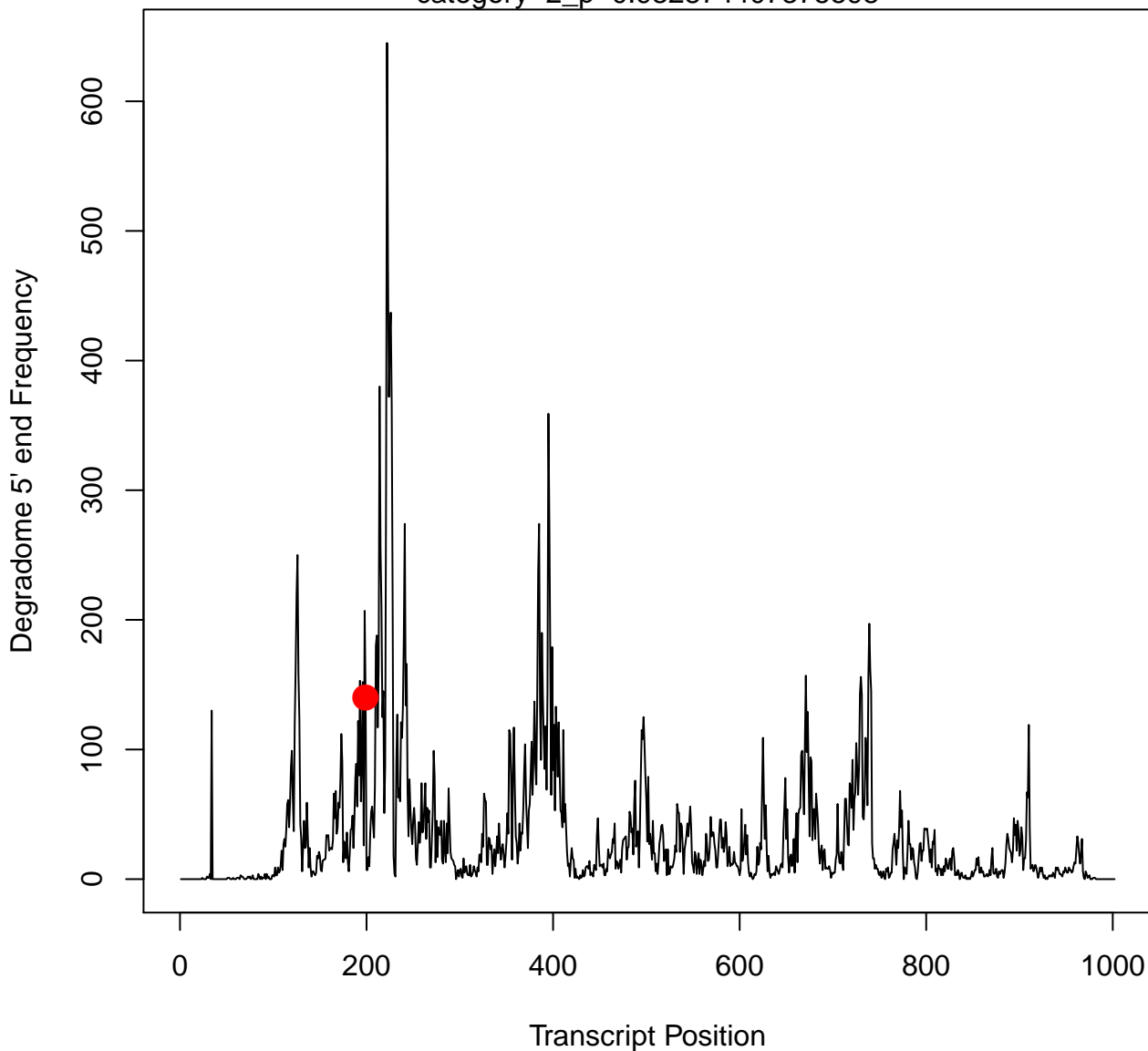

Supplement: Supplementary Data 1 — Results of categories 0–2 from PARE-Seq analysis (including three subfiles:1_1, 1_2, 1_3). [file Data_Sheet_10.ZIP › GSM2230754.plot/Lsa-miR1871_Lsat_1_v5_gn_8_27820.1_199_TPlot.pdf]

**T=Lsat\_1\_v5\_gn\_9\_63420.1\_Q=Lsa-miR1871\_S=950**

category=2\_p=0.978375902287739

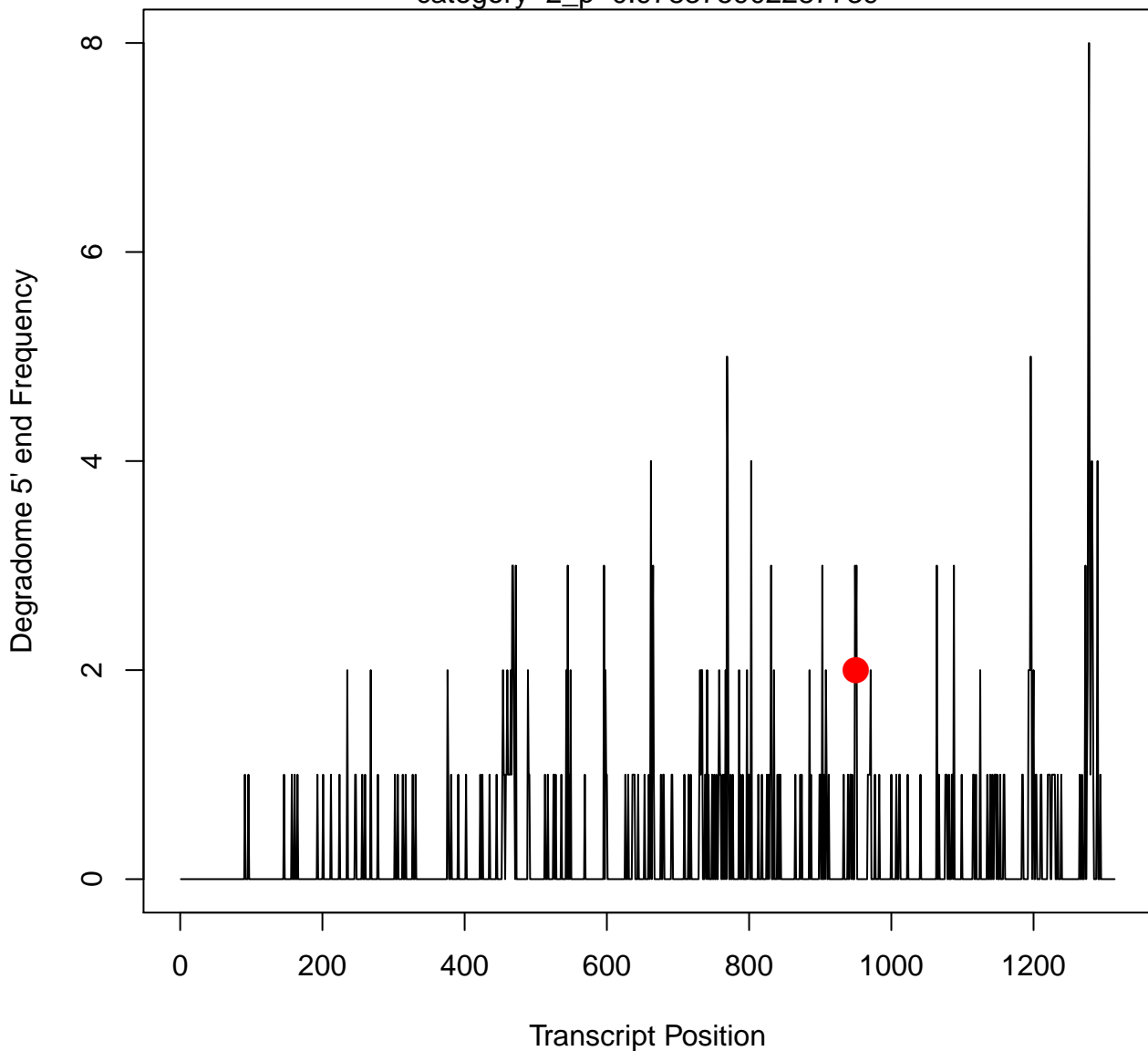

Supplement: Supplementary Data 1 — Results of categories 0–2 from PARE-Seq analysis (including three subfiles:1_1, 1_2, 1_3). [file Data_Sheet_10.ZIP › GSM2230754.plot/Lsa-miR1871_Lsat_1_v5_gn_9_63420.1_950_TPlot.pdf]

**T=Lsat\_1\_v5\_gn\_2\_105320.1\_Q=Lsa-miR2111\_S=1027**

category=2\_p=0.999087988256594

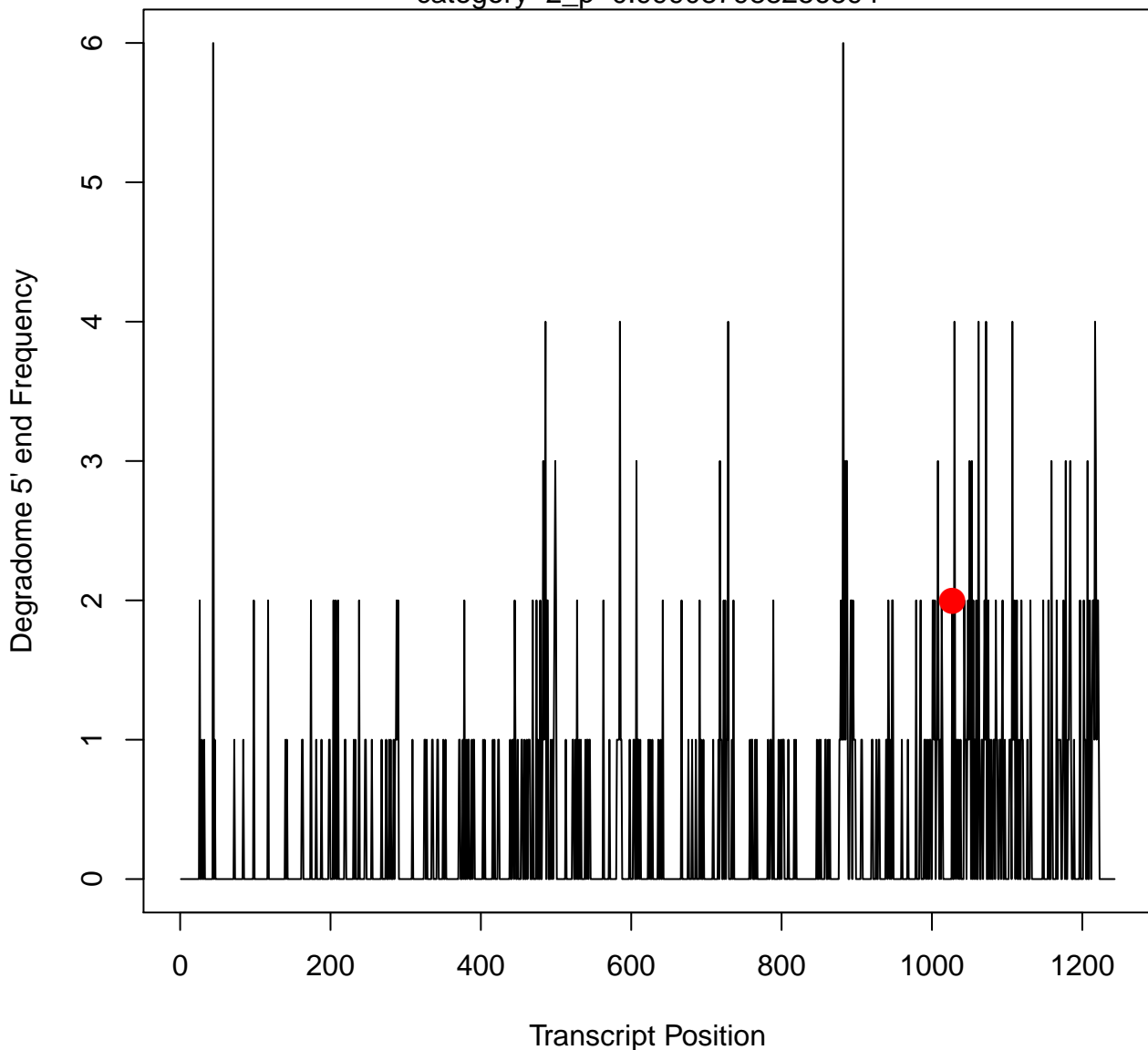

Supplement: Supplementary Data 1 — Results of categories 0–2 from PARE-Seq analysis (including three subfiles:1_1, 1_2, 1_3). [file Data_Sheet_10.ZIP › GSM2230754.plot/Lsa-miR2111_Lsat_1_v5_gn_2_105320.1_1027_TPlot.pdf]

**T=Lsat\_1\_v5\_gn\_4\_63601.1\_Q=Lsa-miR2111\_S=589**

category=2\_p=0.999297779545427

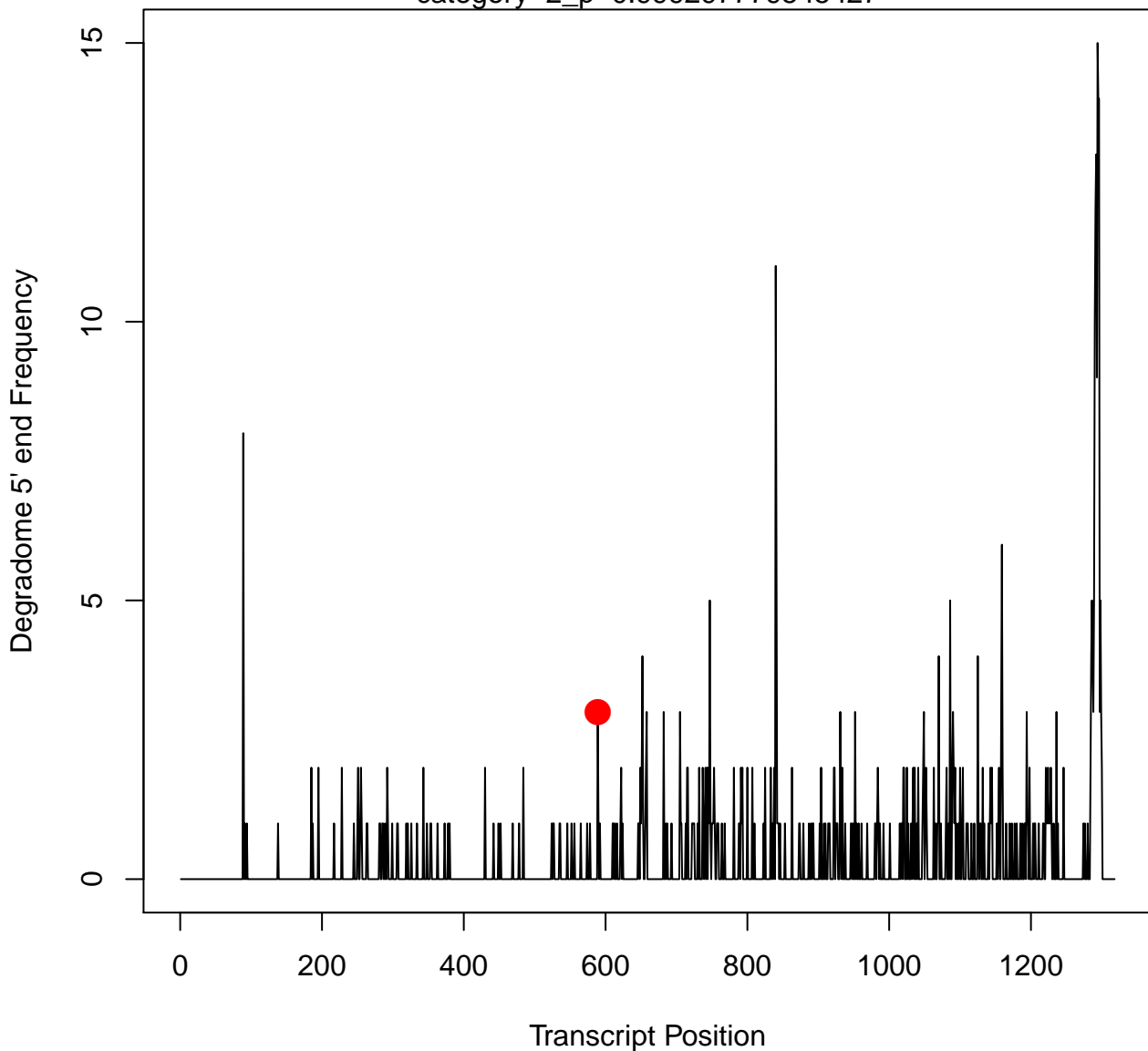

Supplement: Supplementary Data 1 — Results of categories 0–2 from PARE-Seq analysis (including three subfiles:1_1, 1_2, 1_3). [file Data_Sheet_10.ZIP › GSM2230754.plot/Lsa-miR2111_Lsat_1_v5_gn_4_63601.1_589_TPlot.pdf]

**T=Lsat\_1\_v5\_gn\_5\_136700.1\_Q=Lsa-miR2111\_S=2760**

category=2\_p=0.926763015987664

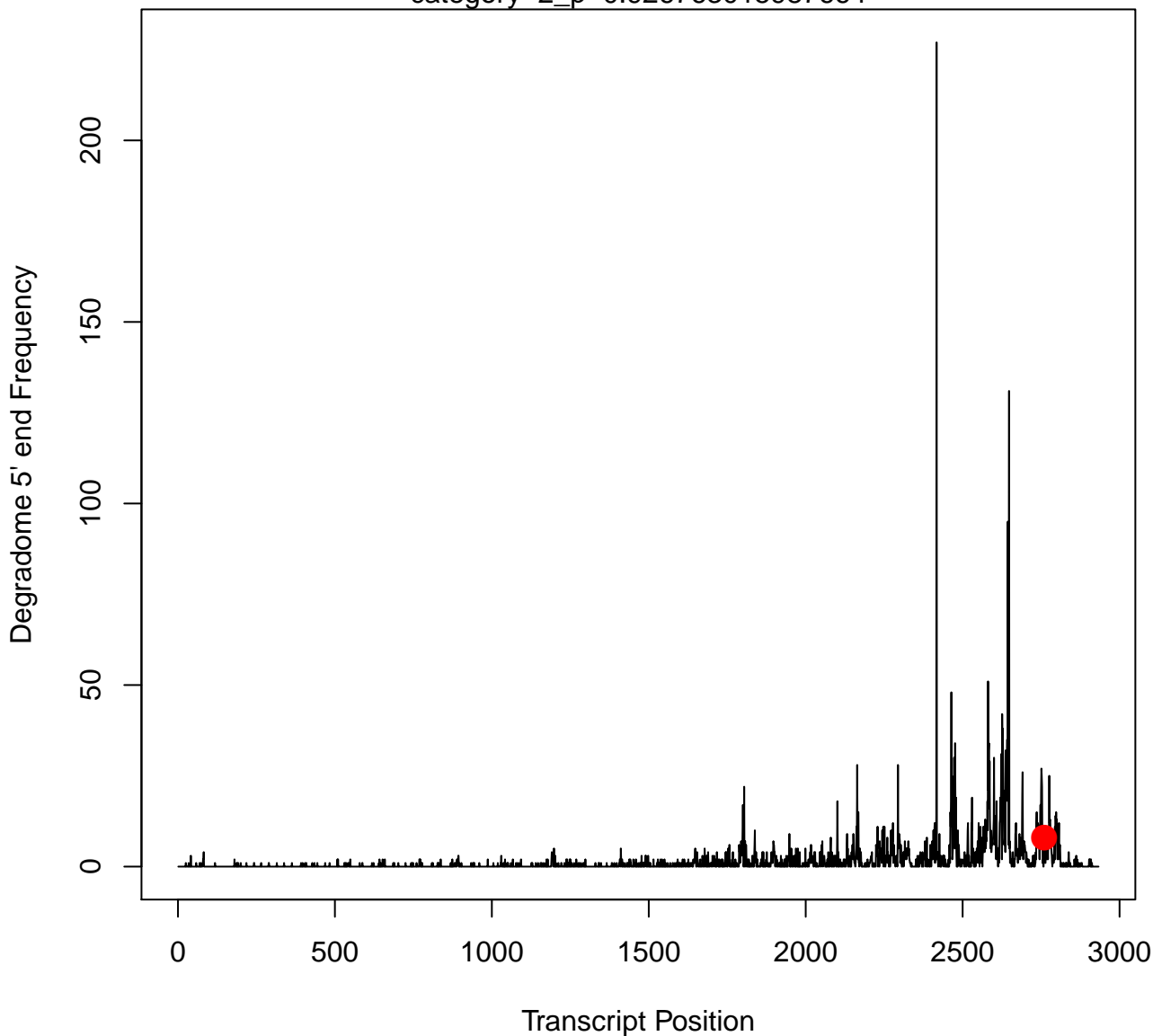

Supplement: Supplementary Data 1 — Results of categories 0–2 from PARE-Seq analysis (including three subfiles:1_1, 1_2, 1_3). [file Data_Sheet_10.ZIP › GSM2230754.plot/Lsa-miR2111_Lsat_1_v5_gn_5_136700.1_2760_TPlot.pdf]

**T=Lsat\_1\_v5\_gn\_5\_176920.1\_Q=Lsa-miR2111\_S=2265**

category=2\_p=0.593591567027102

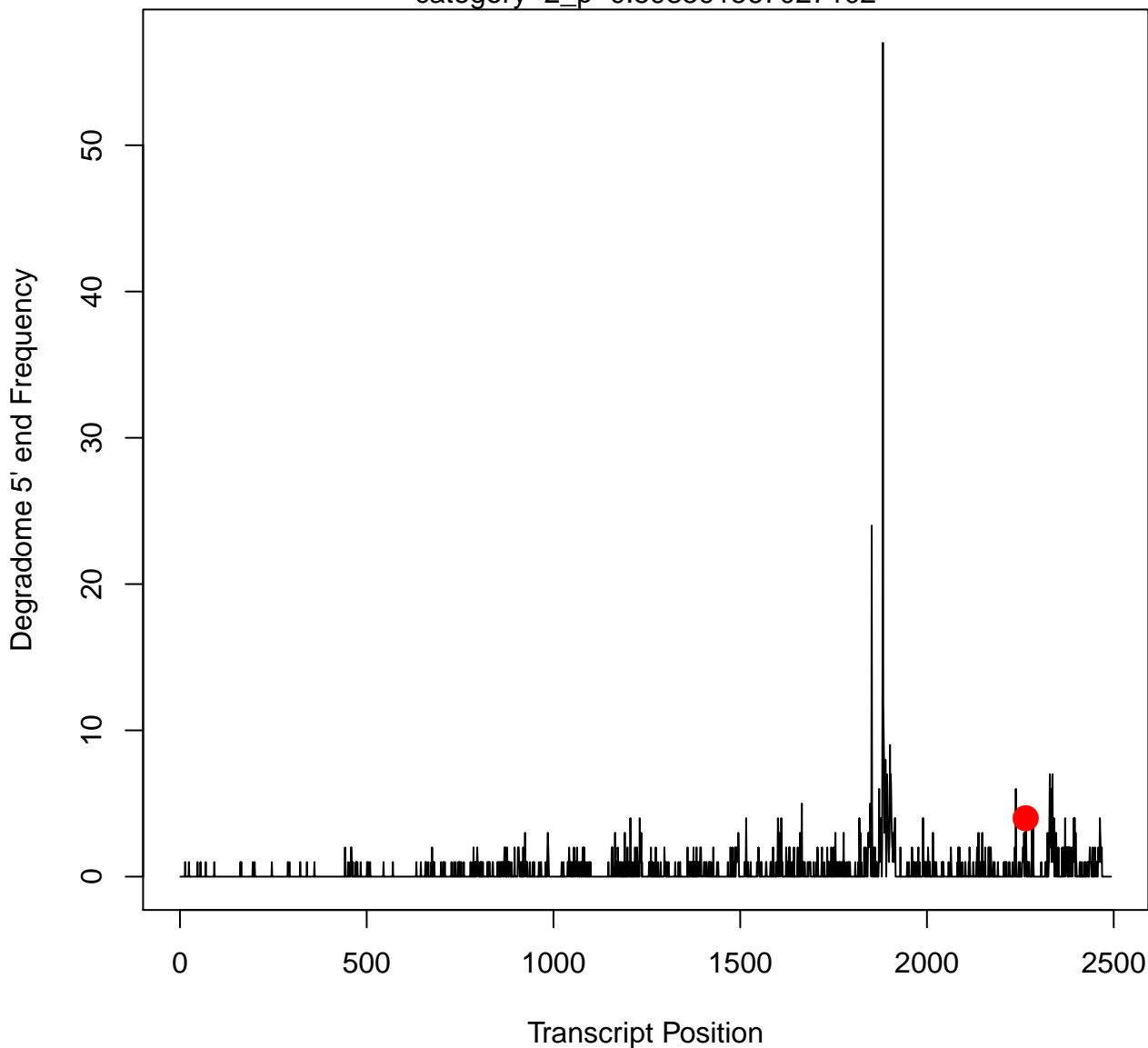

Supplement: Supplementary Data 1 — Results of categories 0–2 from PARE-Seq analysis (including three subfiles:1_1, 1_2, 1_3). [file Data_Sheet_10.ZIP › GSM2230754.plot/Lsa-miR2111_Lsat_1_v5_gn_5_176920.1_2265_TPlot.pdf]

**T=Lsat\_1\_v5\_gn\_5\_3140.1\_Q=Lsa-miR2111\_S=1834**

category=2\_p=0.987547114948745

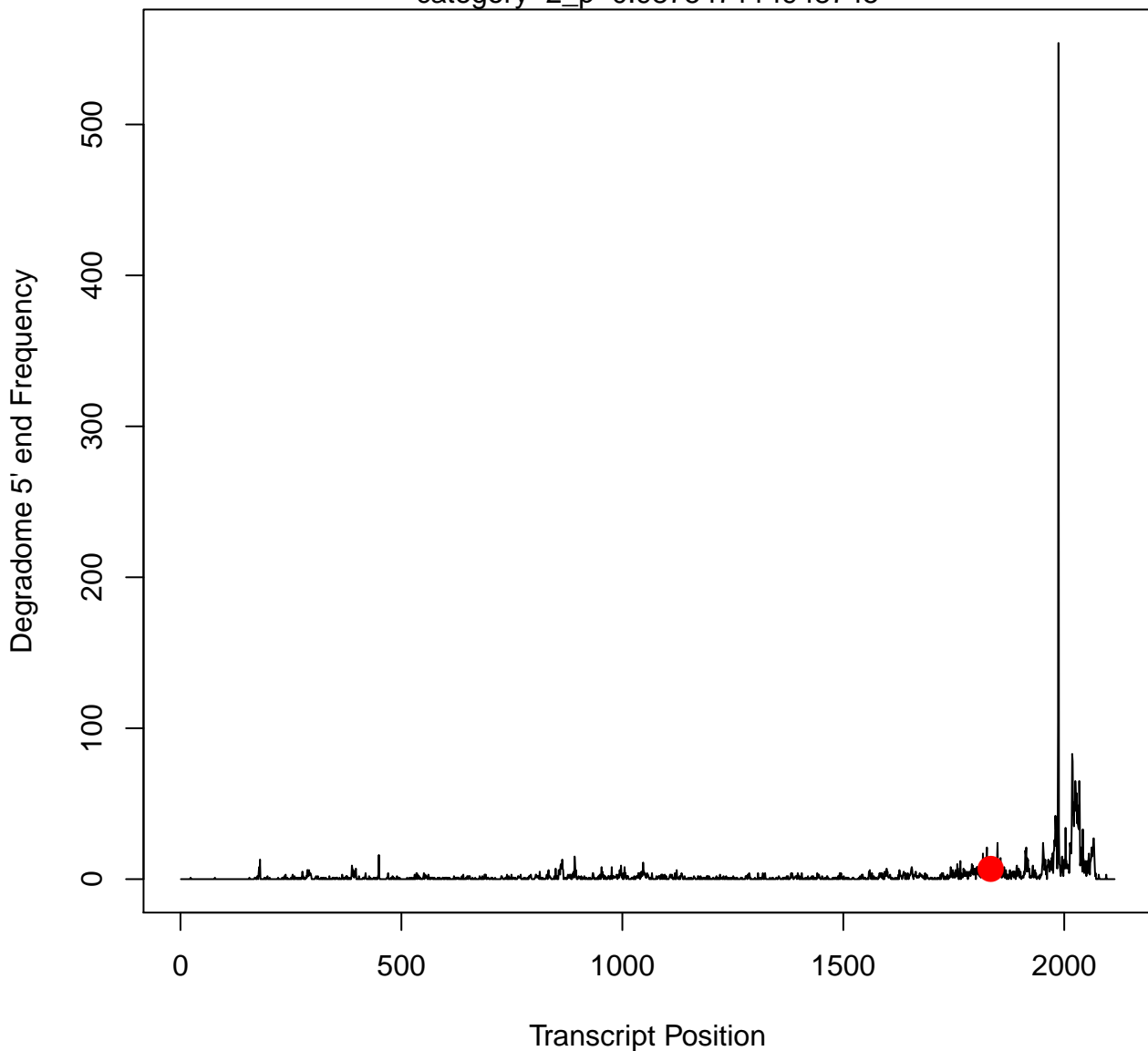

Supplement: Supplementary Data 1 — Results of categories 0–2 from PARE-Seq analysis (including three subfiles:1_1, 1_2, 1_3). [file Data_Sheet_10.ZIP › GSM2230754.plot/Lsa-miR2111_Lsat_1_v5_gn_5_3140.1_1834_TPlot.pdf]

**T=Lsat\_1\_v5\_gn\_6\_55380.1\_Q=Lsa-miR2111\_S=2578**

category=2\_p=0.999392700469826

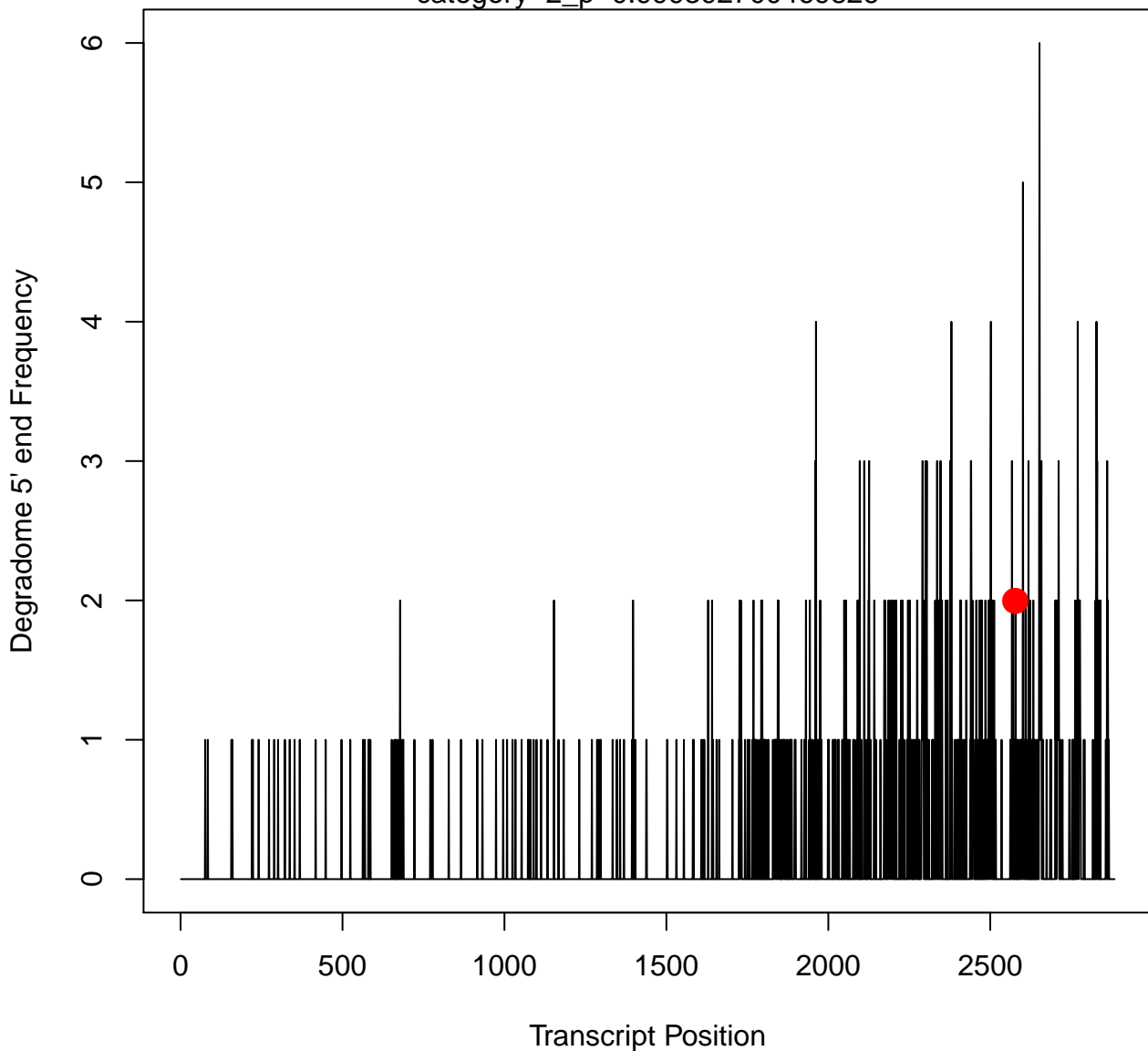

Supplement: Supplementary Data 1 — Results of categories 0–2 from PARE-Seq analysis (including three subfiles:1_1, 1_2, 1_3). [file Data_Sheet_10.ZIP › GSM2230754.plot/Lsa-miR2111_Lsat_1_v5_gn_6_55380.1_2578_TPlot.pdf]

**T=Lsat\_1\_v5\_gn\_6\_59920.1\_Q=Lsa-miR2111\_S=2012**

category=2\_p=0.999164092711051

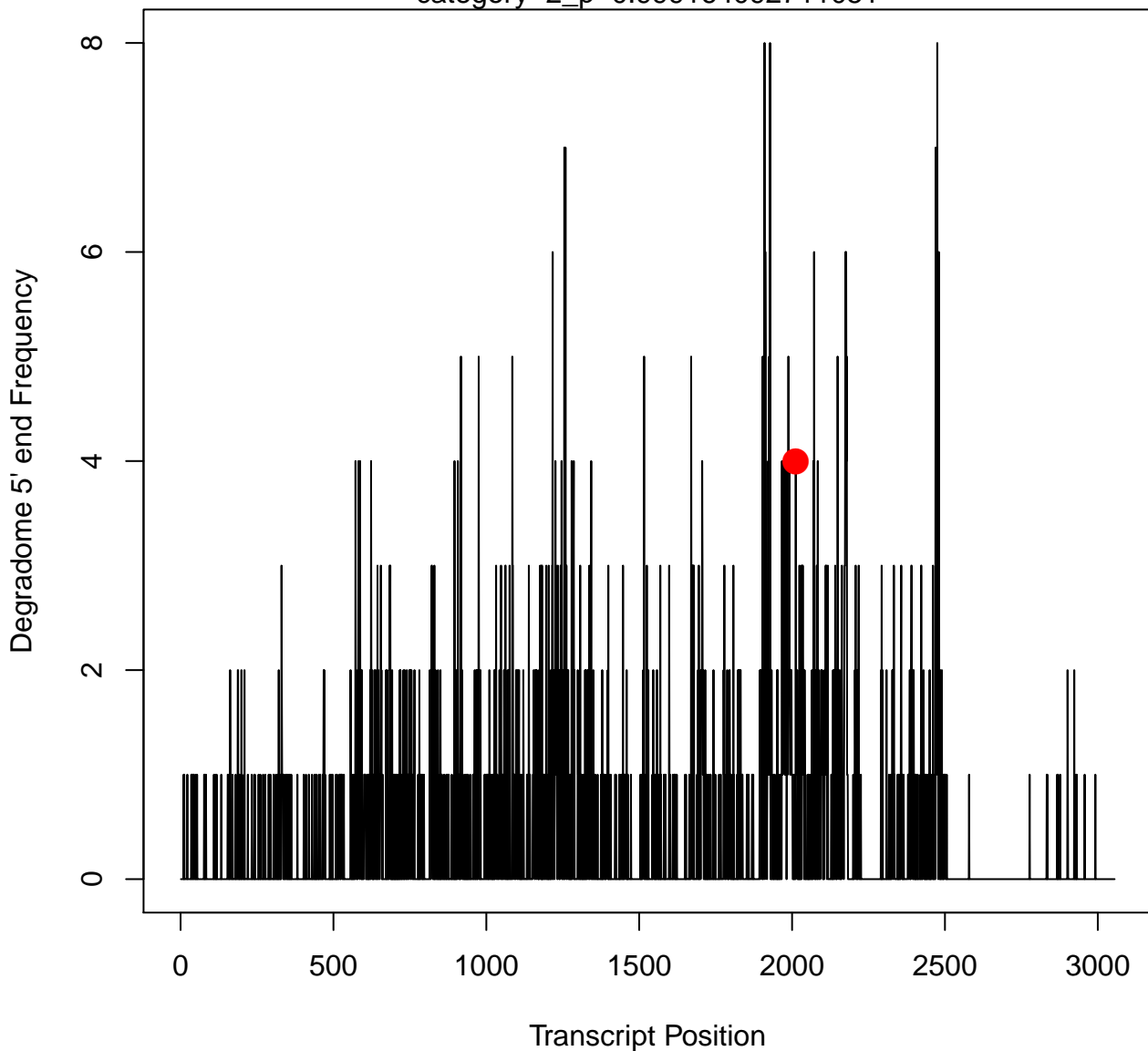

Supplement: Supplementary Data 1 — Results of categories 0–2 from PARE-Seq analysis (including three subfiles:1_1, 1_2, 1_3). [file Data_Sheet_10.ZIP › GSM2230754.plot/Lsa-miR2111_Lsat_1_v5_gn_6_59920.1_2012_TPlot.pdf]

**T=Lsat\_1\_v5\_gn\_7\_3500.1\_Q=Lsa-miR2111\_S=918**

category=2\_p=0.999773783990106

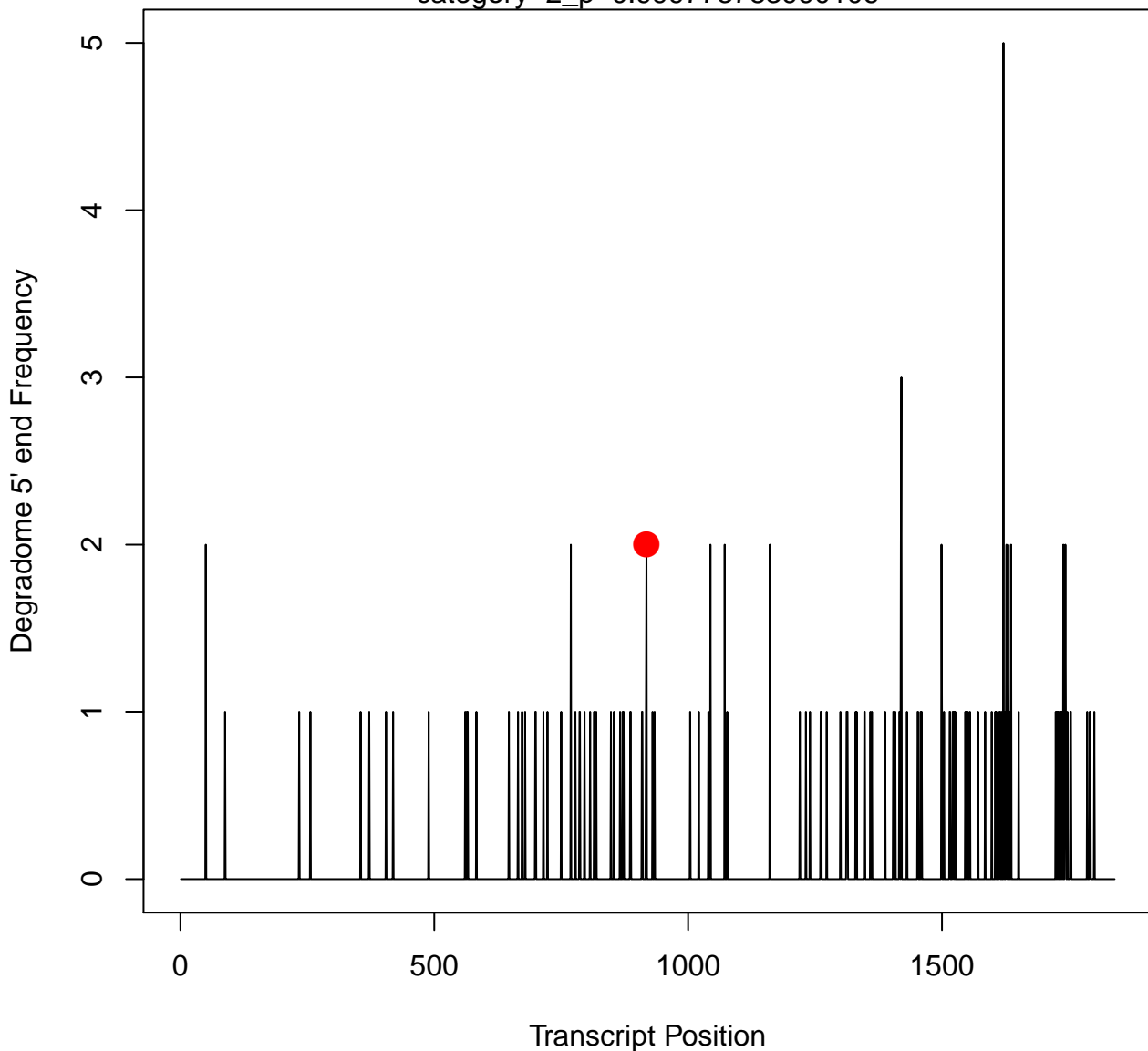

Supplement: Supplementary Data 1 — Results of categories 0–2 from PARE-Seq analysis (including three subfiles:1_1, 1_2, 1_3). [file Data_Sheet_10.ZIP › GSM2230754.plot/Lsa-miR2111_Lsat_1_v5_gn_7_3500.1_918_TPlot.pdf]

**T=Lsat\_1\_v5\_gn\_8\_340.1\_Q=Lsa-miR2111\_S=2200**

category=2\_p=0.994636344172777

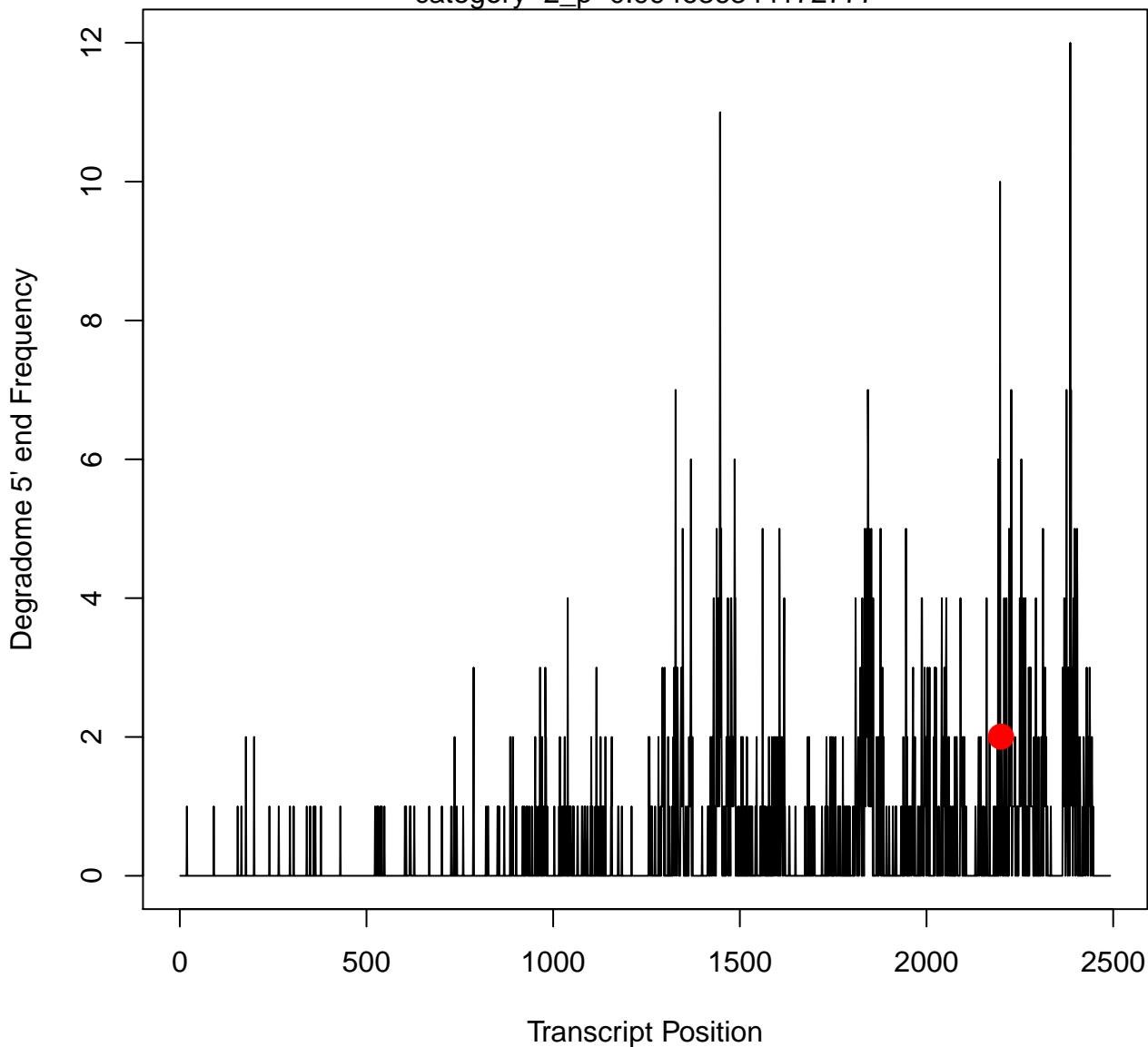

Supplement: Supplementary Data 1 — Results of categories 0–2 from PARE-Seq analysis (including three subfiles:1_1, 1_2, 1_3). [file Data_Sheet_10.ZIP › GSM2230754.plot/Lsa-miR2111_Lsat_1_v5_gn_8_340.1_2200_TPlot.pdf]

**T=Lsat\_1\_v5\_gn\_9\_75021.1\_Q=Lsa-miR2111\_S=578**

category=2\_p=0.999840355461903

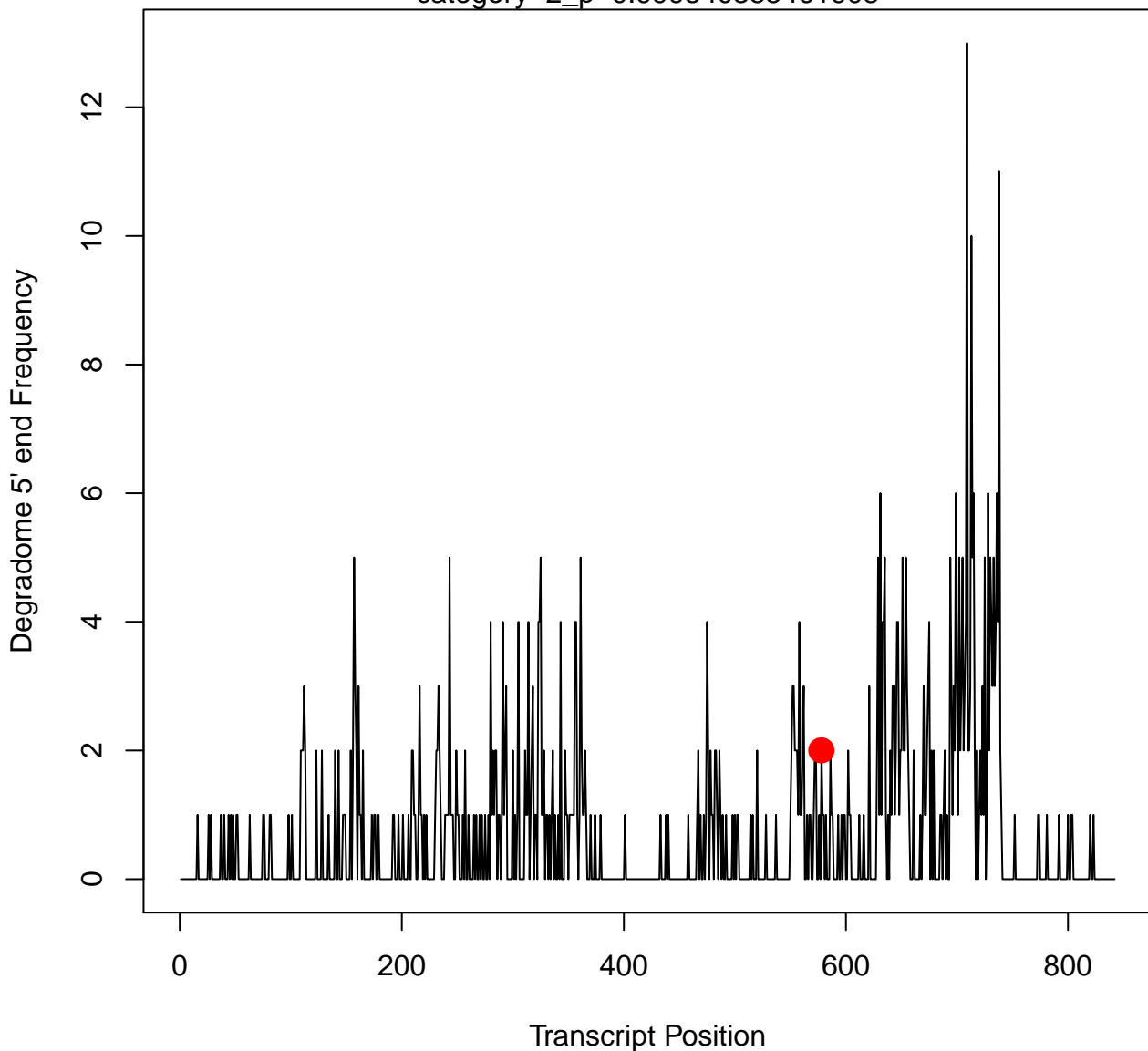

Supplement: Supplementary Data 1 — Results of categories 0–2 from PARE-Seq analysis (including three subfiles:1_1, 1_2, 1_3). [file Data_Sheet_10.ZIP › GSM2230754.plot/Lsa-miR2111_Lsat_1_v5_gn_9_75021.1_578_TPlot.pdf]

**T=Lsat\_1\_v5\_gn\_0\_27881.1\_Q=Lsa-miR2275\_S=1538**

category=2\_p=0.999869726727772

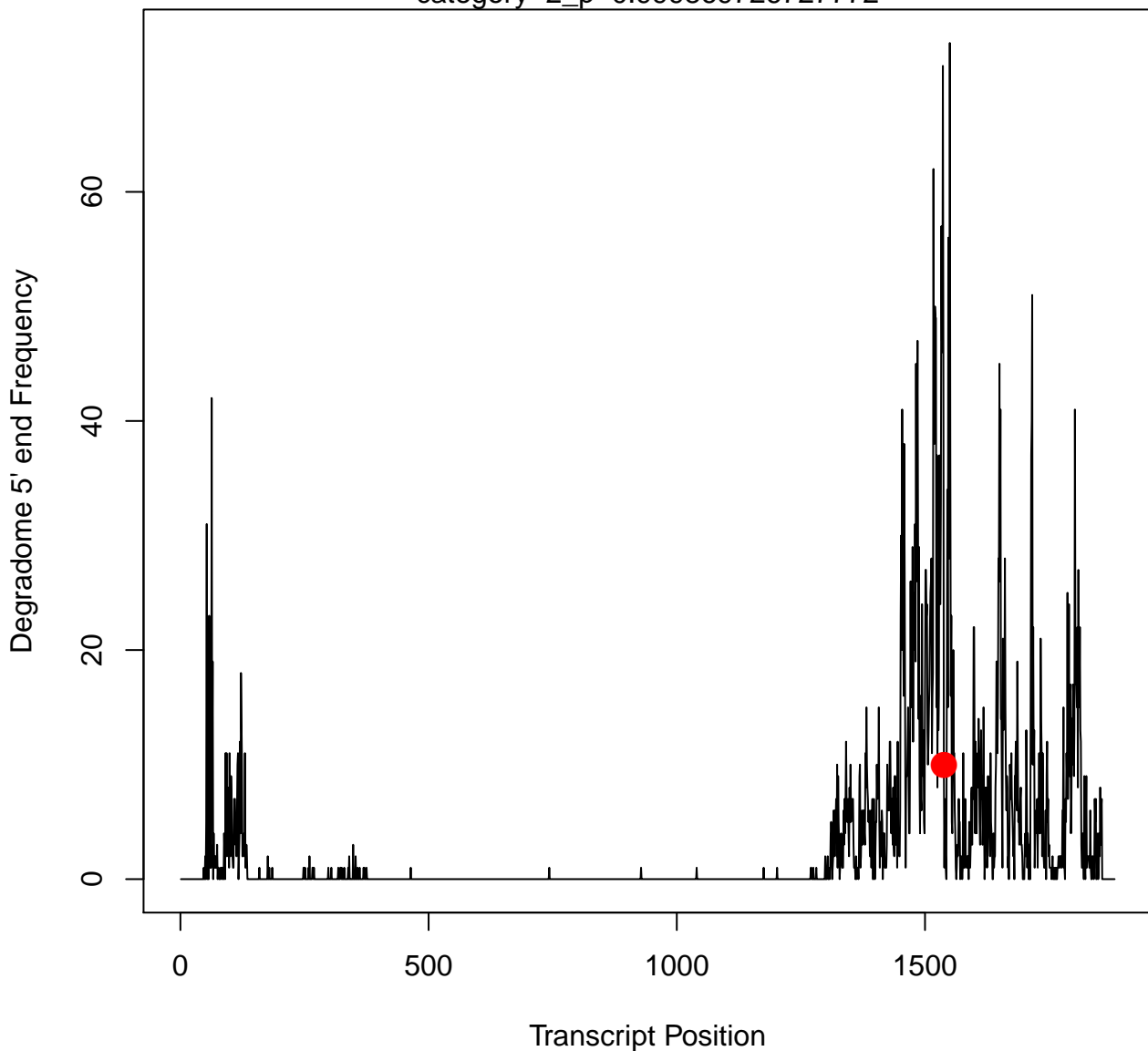

Supplement: Supplementary Data 1 — Results of categories 0–2 from PARE-Seq analysis (including three subfiles:1_1, 1_2, 1_3). [file Data_Sheet_10.ZIP › GSM2230754.plot/Lsa-miR2275_Lsat_1_v5_gn_0_27881.1_1538_TPlot.pdf]

**T=Lsat\_1\_v5\_gn\_1\_2520.1\_Q=Lsa-miR2275\_S=465**

category=2\_p=0.999999816317685

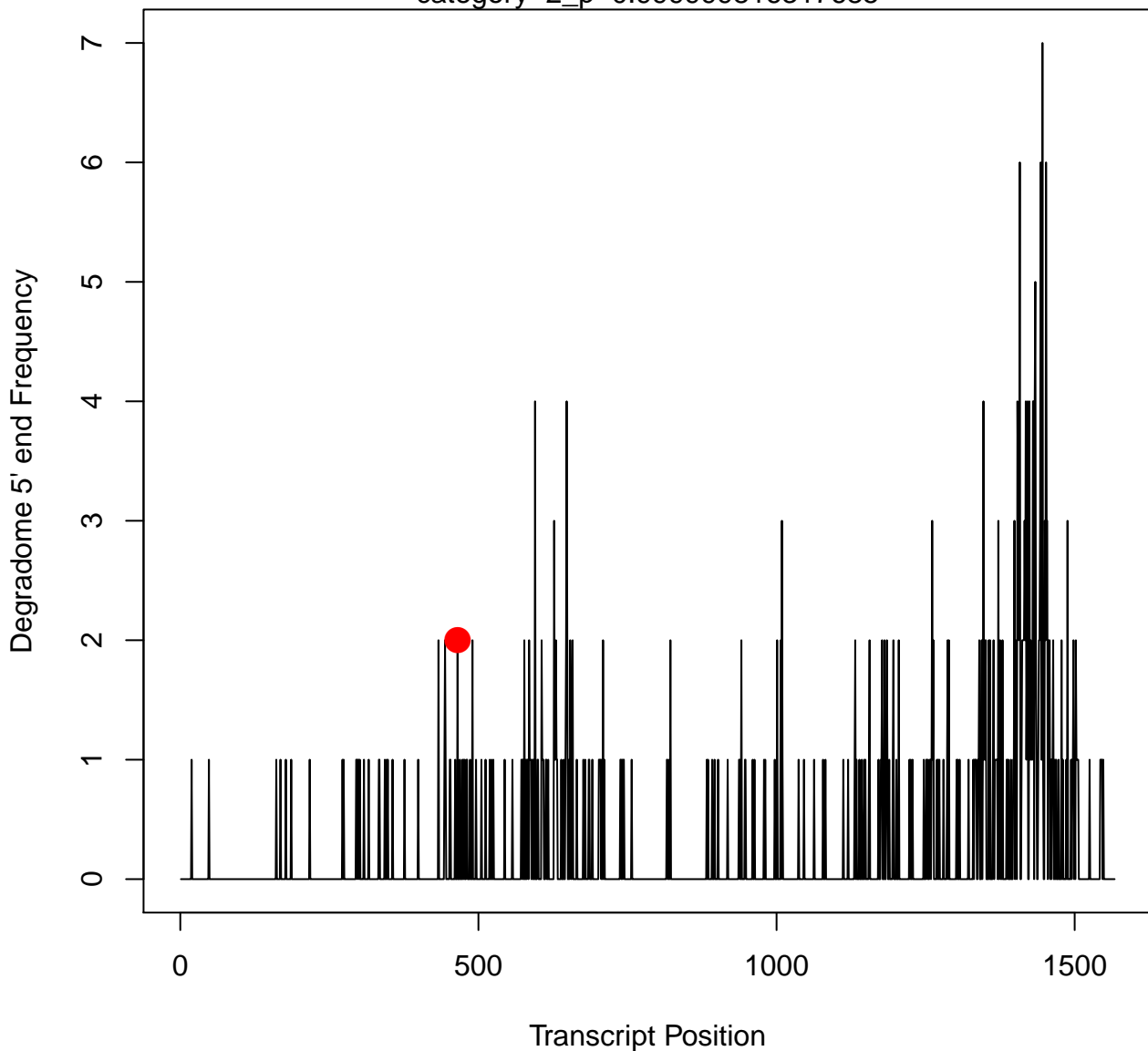

Supplement: Supplementary Data 1 — Results of categories 0–2 from PARE-Seq analysis (including three subfiles:1_1, 1_2, 1_3). [file Data_Sheet_10.ZIP › GSM2230754.plot/Lsa-miR2275_Lsat_1_v5_gn_1_2520.1_465_TPlot.pdf]

**T=Lsat\_1\_v5\_gn\_1\_62020.1\_Q=Lsa-miR2275\_S=1768**

category=2\_p=0.648526828297634

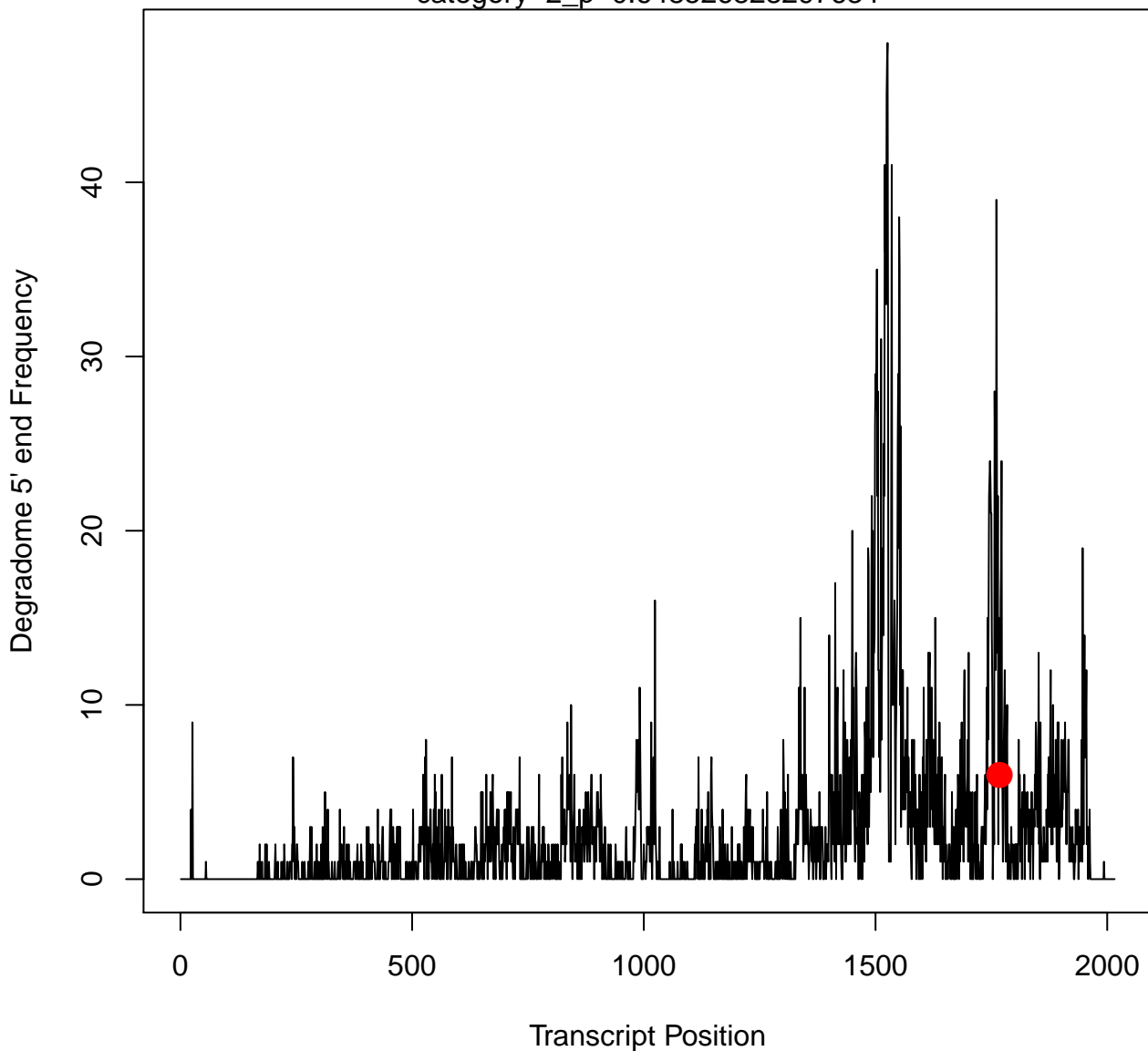

Supplement: Supplementary Data 1 — Results of categories 0–2 from PARE-Seq analysis (including three subfiles:1_1, 1_2, 1_3). [file Data_Sheet_10.ZIP › GSM2230754.plot/Lsa-miR2275_Lsat_1_v5_gn_1_62020.1_1768_TPlot.pdf]

**T=Lsat\_1\_v5\_gn\_1\_66101.1\_Q=Lsa-miR2275\_S=1242**

category=2\_p=0.999995516480999

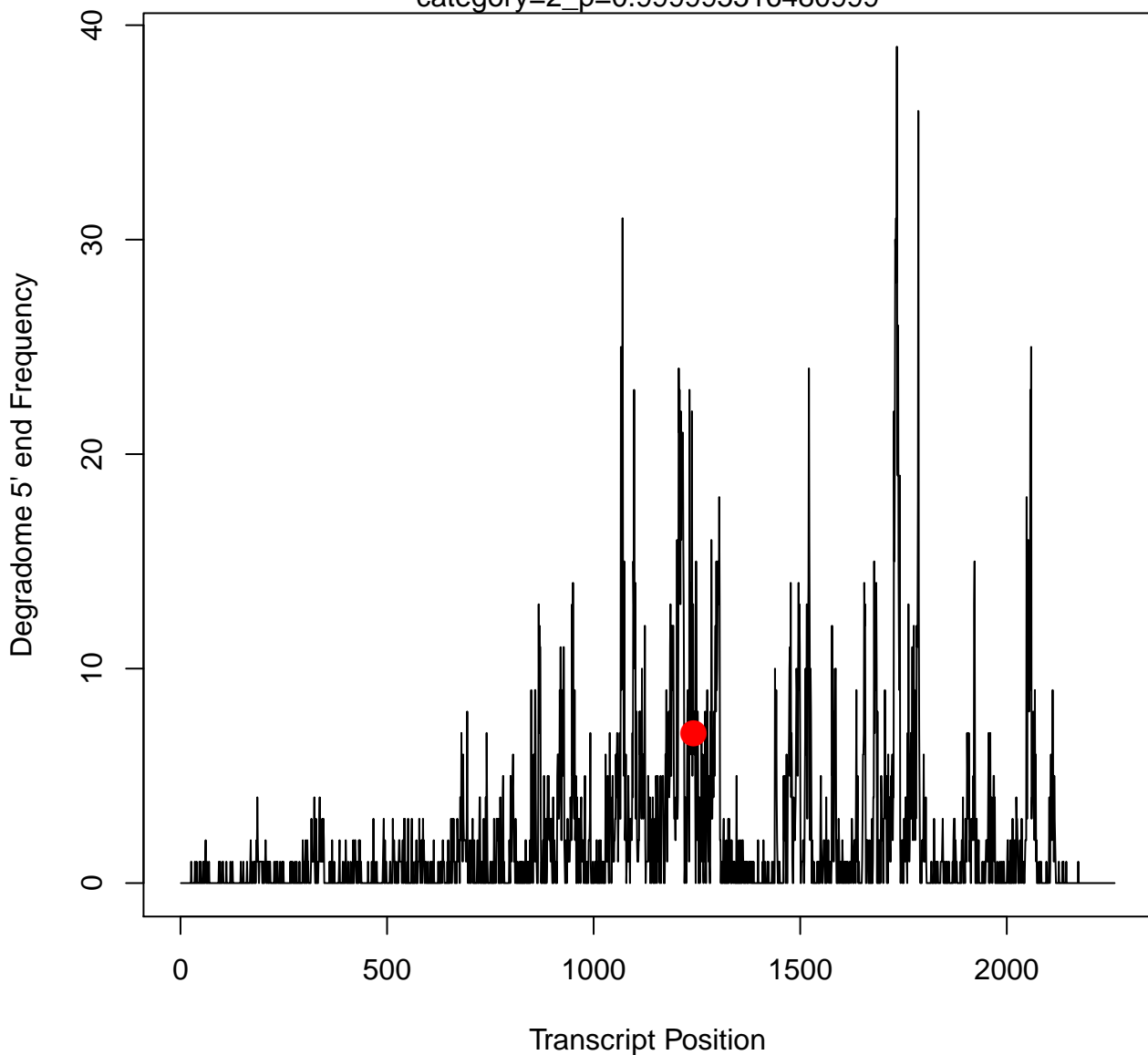

Supplement: Supplementary Data 1 — Results of categories 0–2 from PARE-Seq analysis (including three subfiles:1_1, 1_2, 1_3). [file Data_Sheet_10.ZIP › GSM2230754.plot/Lsa-miR2275_Lsat_1_v5_gn_1_66101.1_1242_TPlot.pdf]

**T=Lsat\_1\_v5\_gn\_2\_69001.1\_Q=Lsa-miR2275\_S=1369**

category=2\_p=0.999999092507218

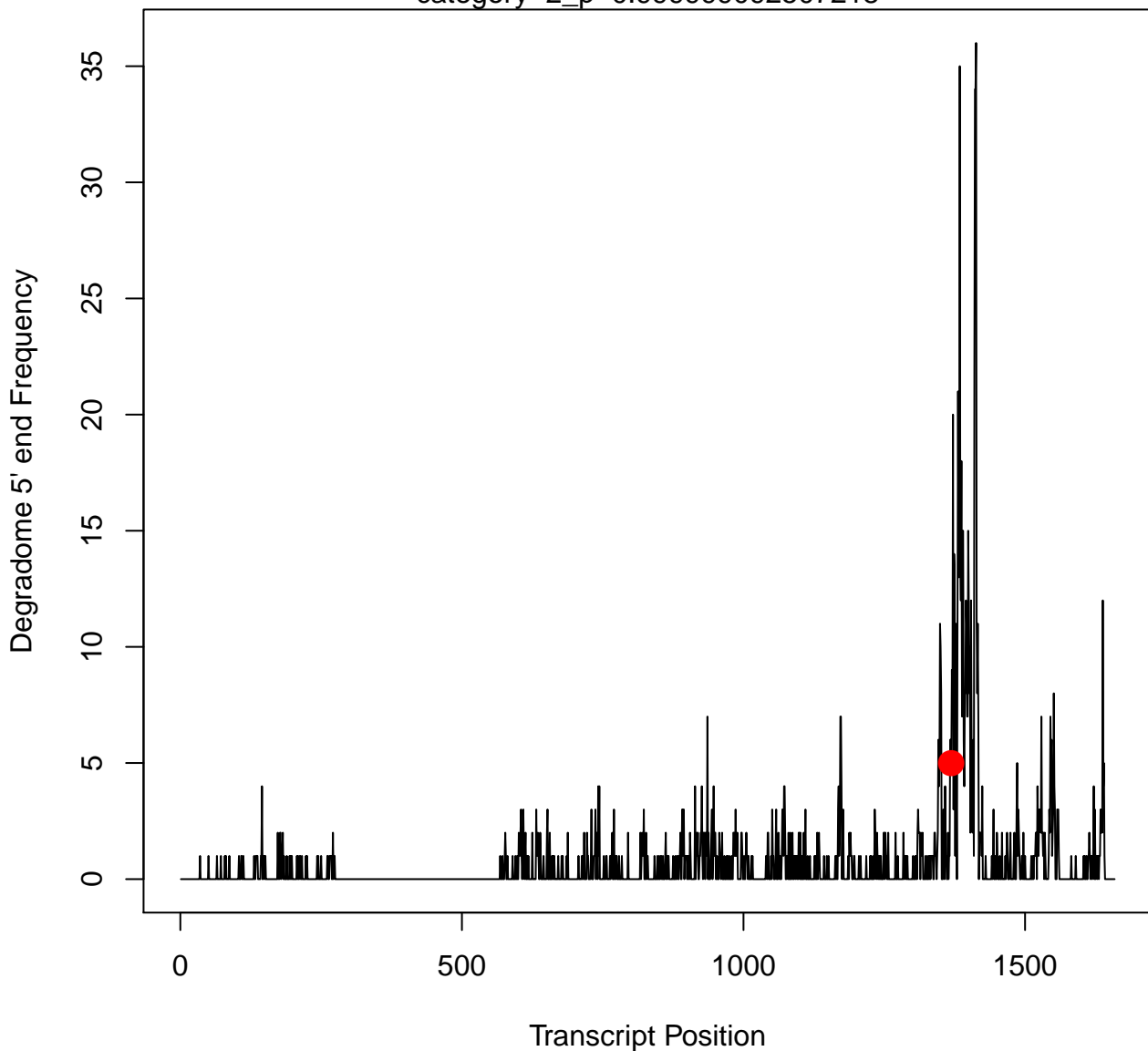

Supplement: Supplementary Data 1 — Results of categories 0–2 from PARE-Seq analysis (including three subfiles:1_1, 1_2, 1_3). [file Data_Sheet_10.ZIP › GSM2230754.plot/Lsa-miR2275_Lsat_1_v5_gn_2_69001.1_1369_TPlot.pdf]

**T=Lsat\_1\_v5\_gn\_4\_152040.1\_Q=Lsa-miR2275\_S=429**

category=2\_p=0.999545784569208

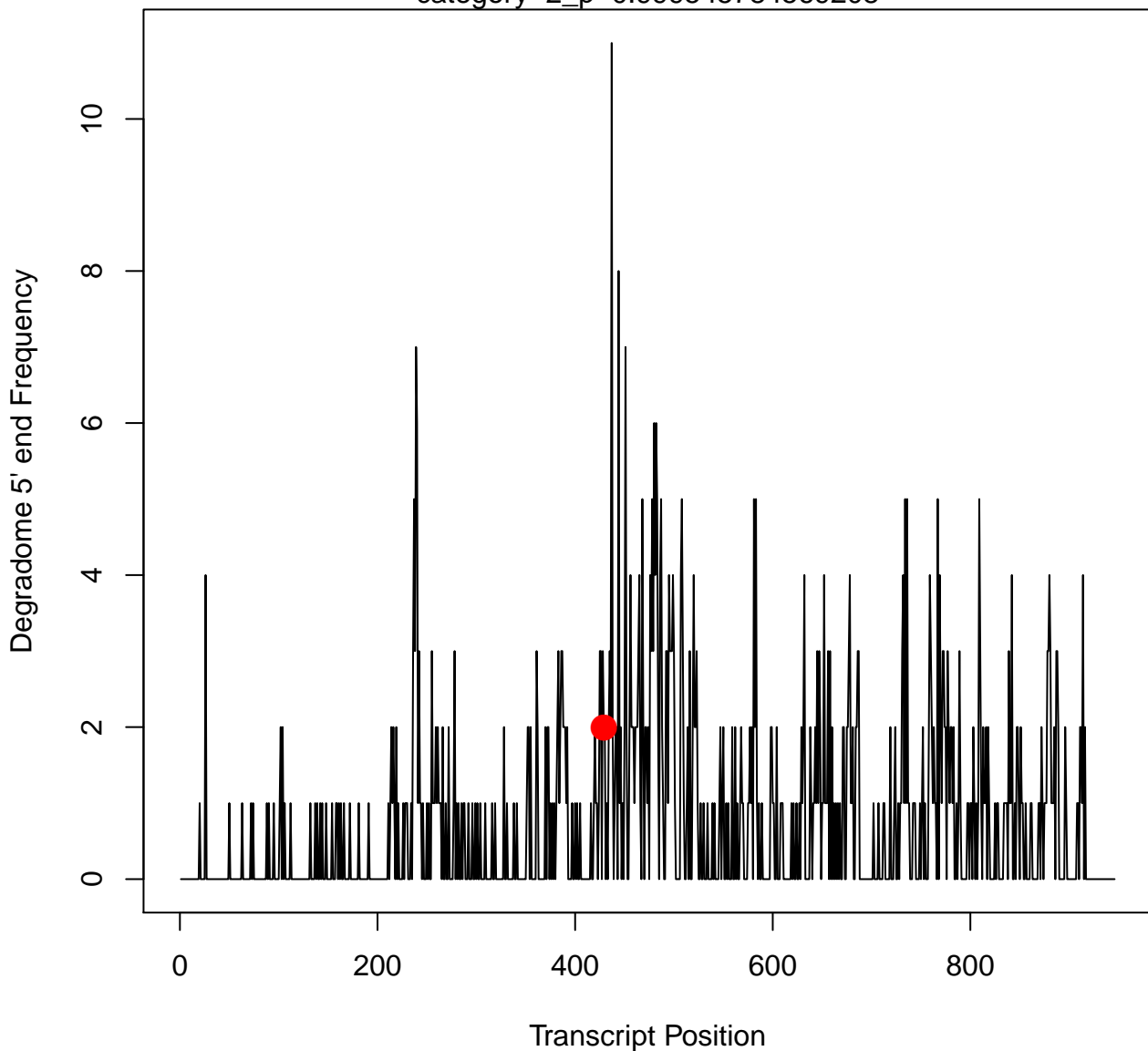

Supplement: Supplementary Data 1 — Results of categories 0–2 from PARE-Seq analysis (including three subfiles:1_1, 1_2, 1_3). [file Data_Sheet_10.ZIP › GSM2230754.plot/Lsa-miR2275_Lsat_1_v5_gn_4_152040.1_429_TPlot.pdf]
