# Supplementary figures and images for: Comprehensive Annotation and Functional Exploration of MicroRNAs in Lettuce (part 5 of 6)
Source: Front Plant Sci. 2021 Dec 24;12:781836. doi: 10.3389/fpls.2021.781836 (PMC8739914; doi:10.3389/fpls.2021.781836)

**T=Lsat\_1\_v5\_gn\_2\_133880.1\_Q=Lsa-miR1446\_S=1251**

category=2\_p=0.273484585907661

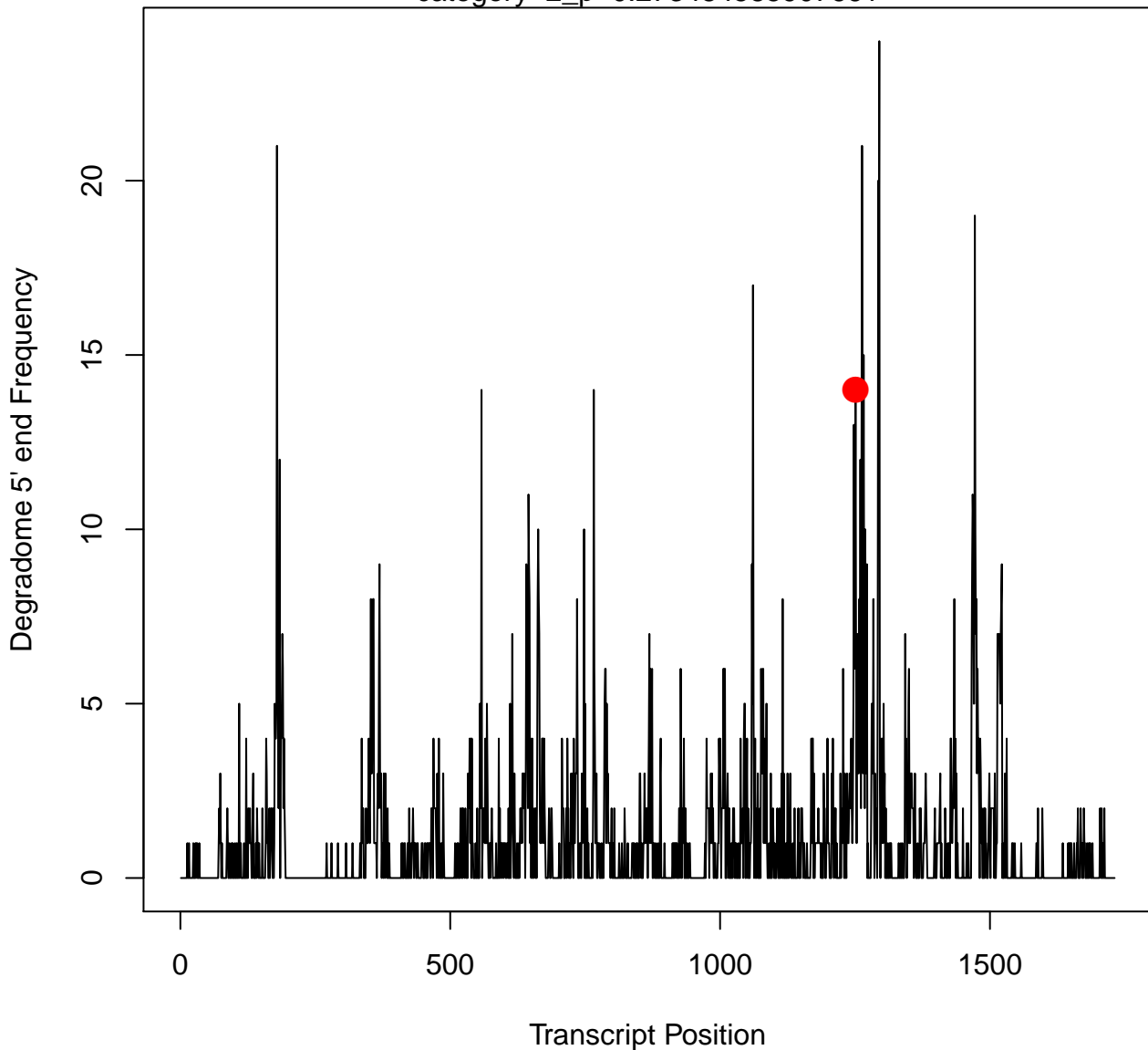

Supplement: Supplementary Data 1 — Results of categories 0–2 from PARE-Seq analysis (including three subfiles:1_1, 1_2, 1_3). [file Data_Sheet_10.ZIP › GSM2230754.plot/Lsa-miR1446_Lsat_1_v5_gn_2_133880.1_1251_TPlot.pdf]

**T=Lsat\_1\_v5\_gn\_3\_102000.1\_Q=Lsa-miR1446\_S=1448**

category=2\_p=0.713190562458278

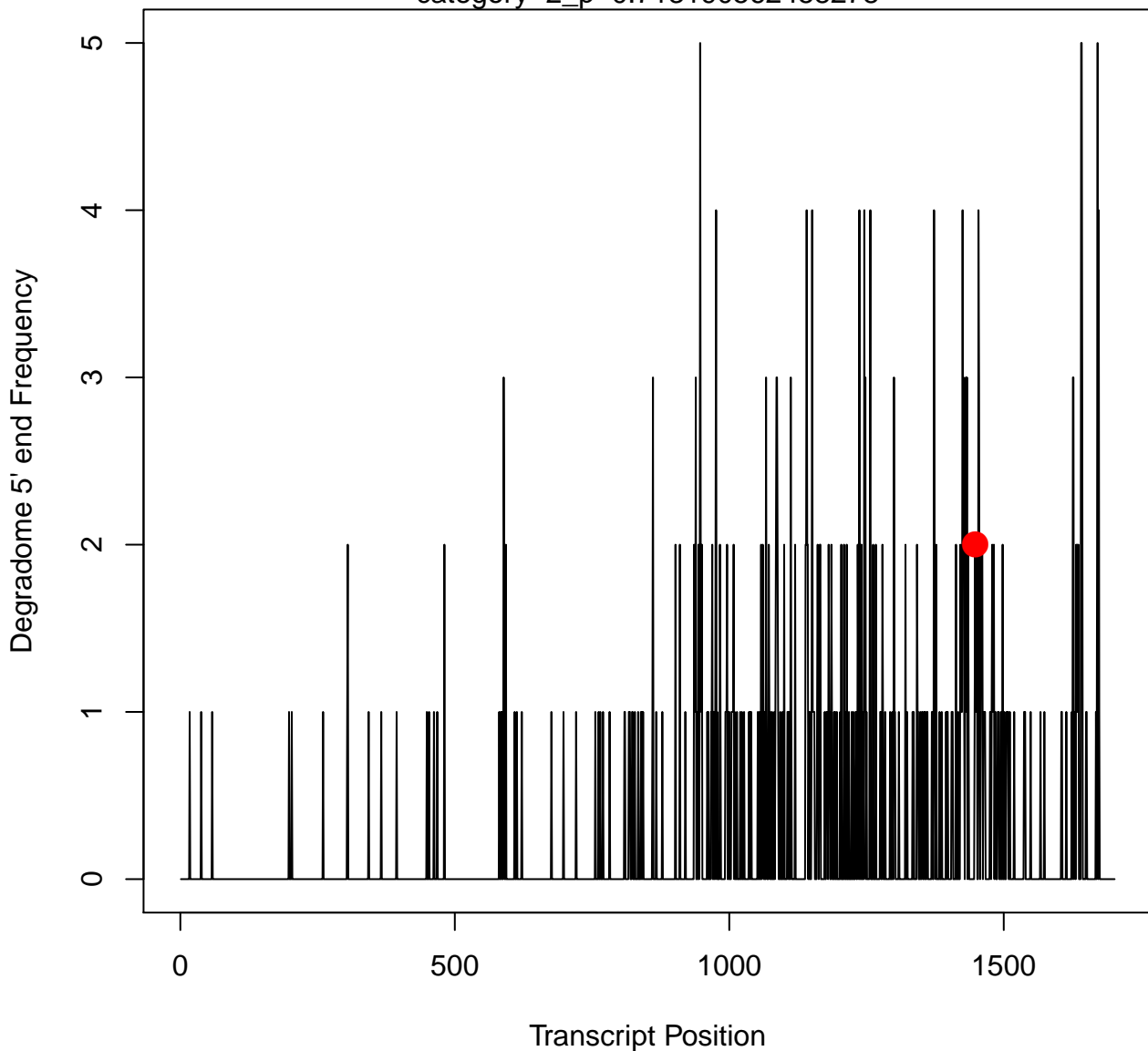

Supplement: Supplementary Data 1 — Results of categories 0–2 from PARE-Seq analysis (including three subfiles:1_1, 1_2, 1_3). [file Data_Sheet_10.ZIP › GSM2230754.plot/Lsa-miR1446_Lsat_1_v5_gn_3_102000.1_1448_TPlot.pdf]

**T=Lsat\_1\_v5\_gn\_3\_102160.1\_Q=Lsa-miR1446\_S=1169**

category=2\_p=0.407148271738738

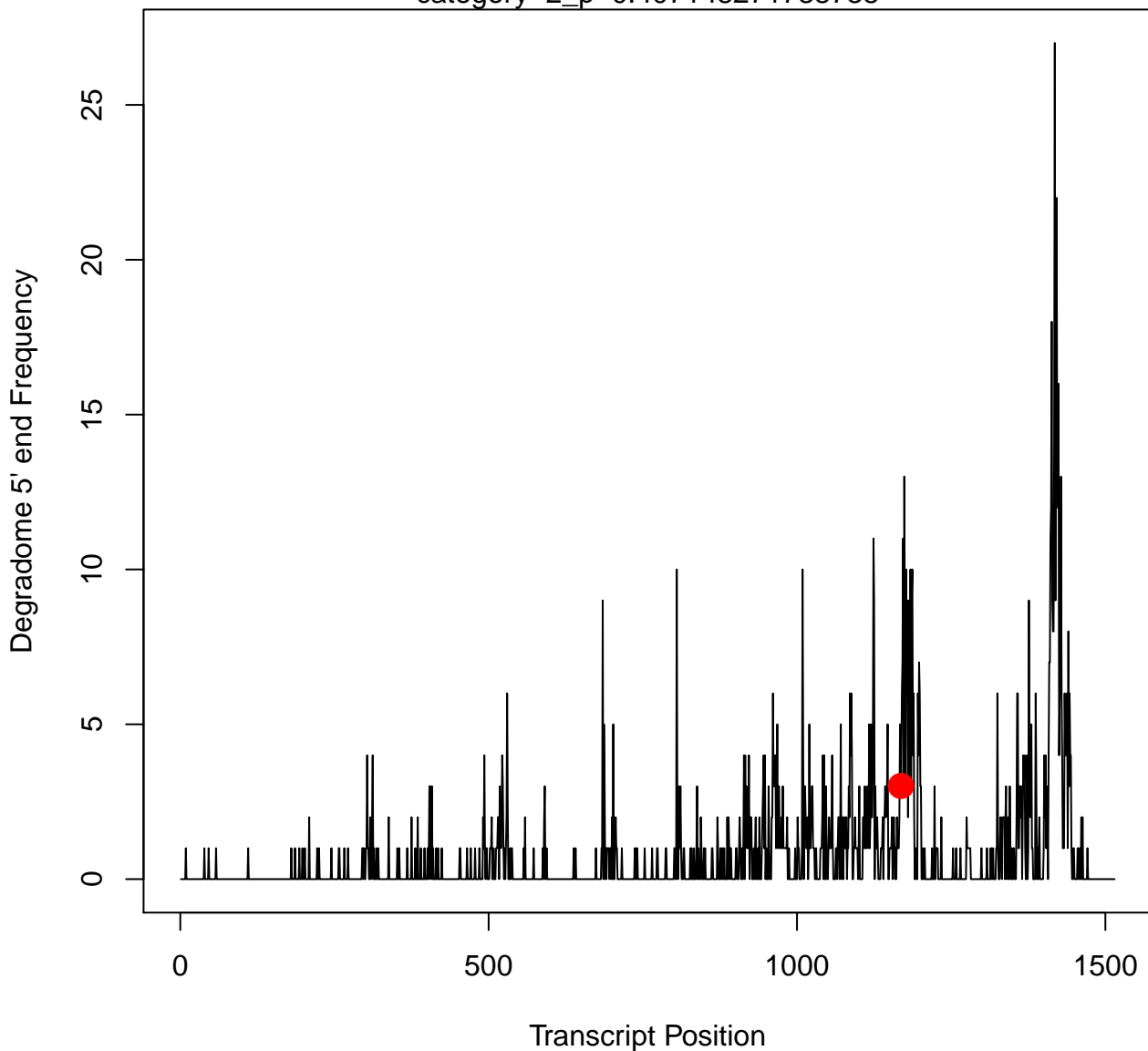

Supplement: Supplementary Data 1 — Results of categories 0–2 from PARE-Seq analysis (including three subfiles:1_1, 1_2, 1_3). [file Data_Sheet_10.ZIP › GSM2230754.plot/Lsa-miR1446_Lsat_1_v5_gn_3_102160.1_1169_TPlot.pdf]

**T=Lsat\_1\_v5\_gn\_3\_32820.1\_Q=Lsa-miR1446\_S=1167**

category=2\_p=0.593591567027102

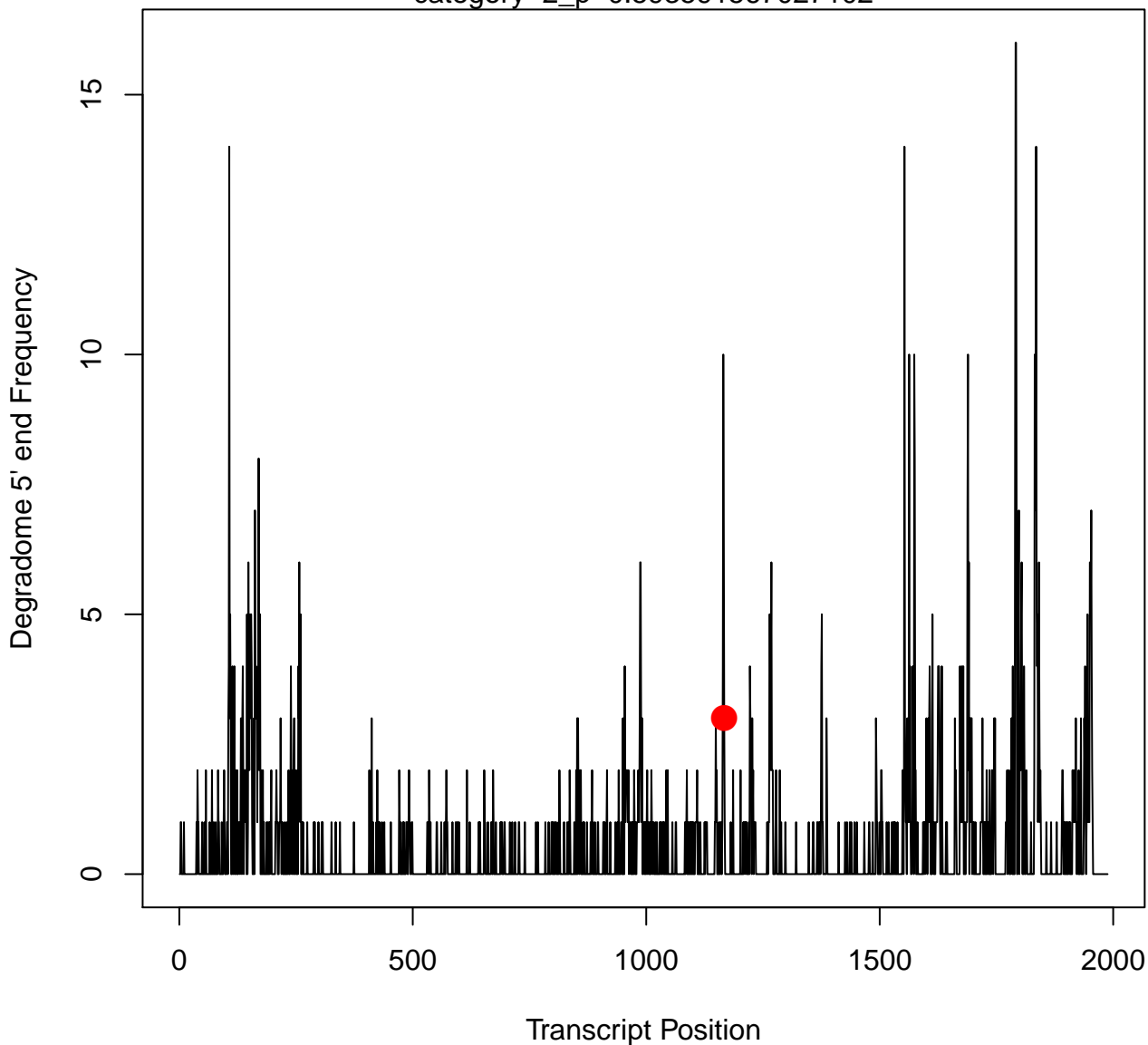

Supplement: Supplementary Data 1 — Results of categories 0–2 from PARE-Seq analysis (including three subfiles:1_1, 1_2, 1_3). [file Data_Sheet_10.ZIP › GSM2230754.plot/Lsa-miR1446_Lsat_1_v5_gn_3_32820.1_1167_TPlot.pdf]

**T=Lsat\_1\_v5\_gn\_3\_53980.1\_Q=Lsa-miR1446\_S=441**

category=2\_p=0.968455665137098

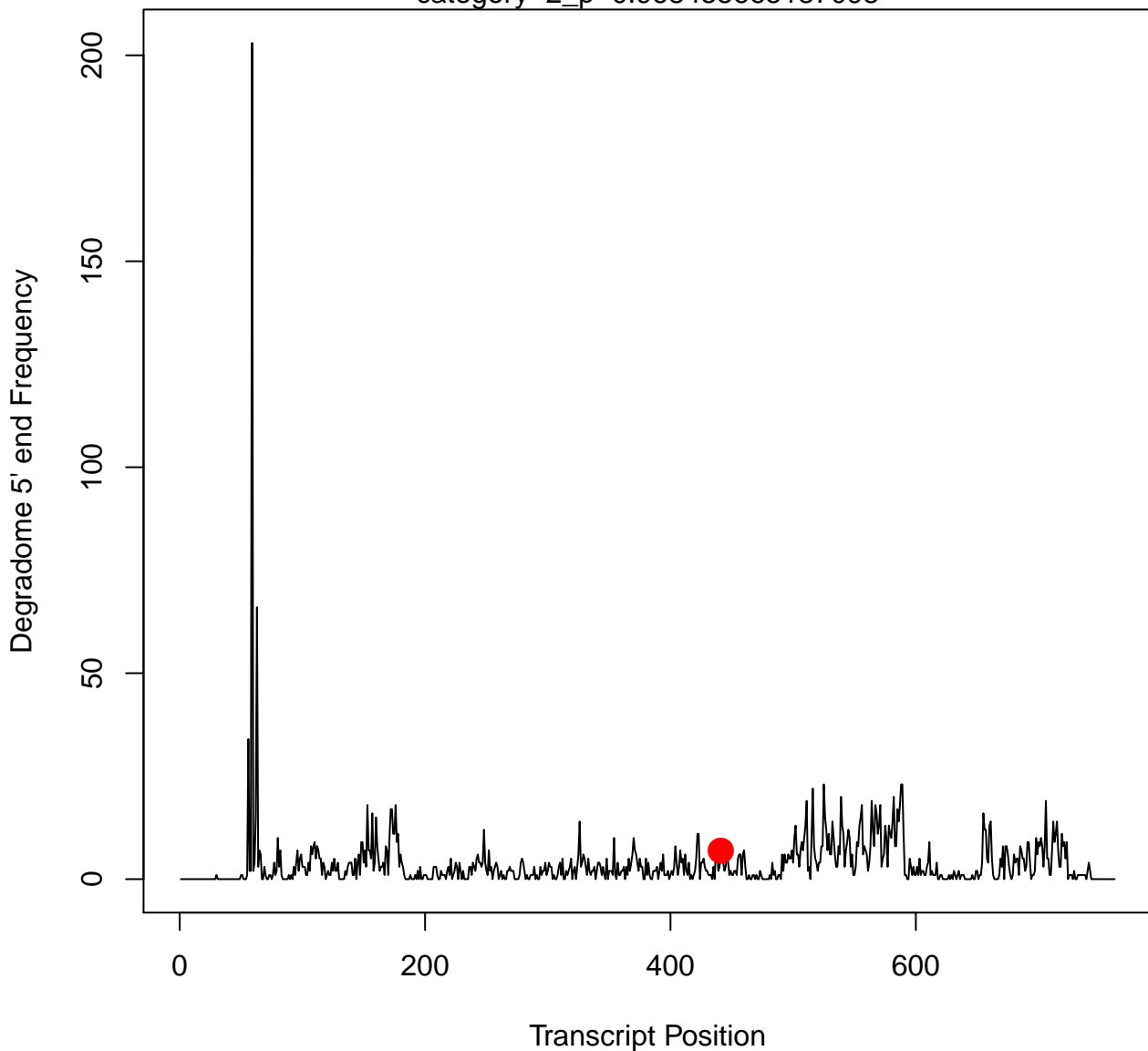

Supplement: Supplementary Data 1 — Results of categories 0–2 from PARE-Seq analysis (including three subfiles:1_1, 1_2, 1_3). [file Data_Sheet_10.ZIP › GSM2230754.plot/Lsa-miR1446_Lsat_1_v5_gn_3_53980.1_441_TPlot.pdf]

**T=Lsat\_1\_v5\_gn\_4\_76080.1\_Q=Lsa-miR1446\_S=648**

category=2\_p=0.839560494089731

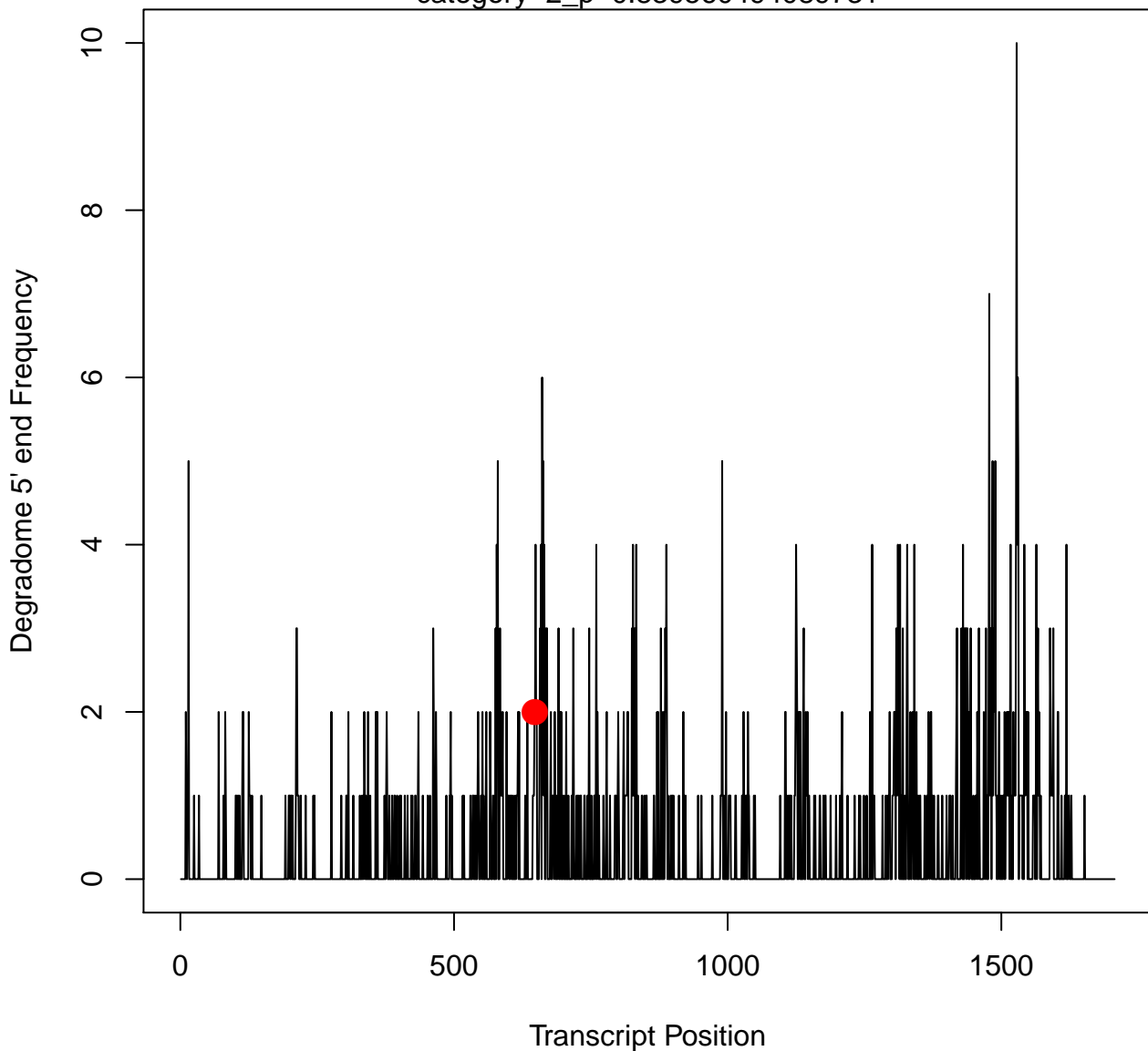

Supplement: Supplementary Data 1 — Results of categories 0–2 from PARE-Seq analysis (including three subfiles:1_1, 1_2, 1_3). [file Data_Sheet_10.ZIP › GSM2230754.plot/Lsa-miR1446_Lsat_1_v5_gn_4_76080.1_648_TPlot.pdf]

**T=Lsat\_1\_v5\_gn\_5\_170600.1\_Q=Lsa-miR1446\_S=1122**

category=2\_p=0.999607182023934

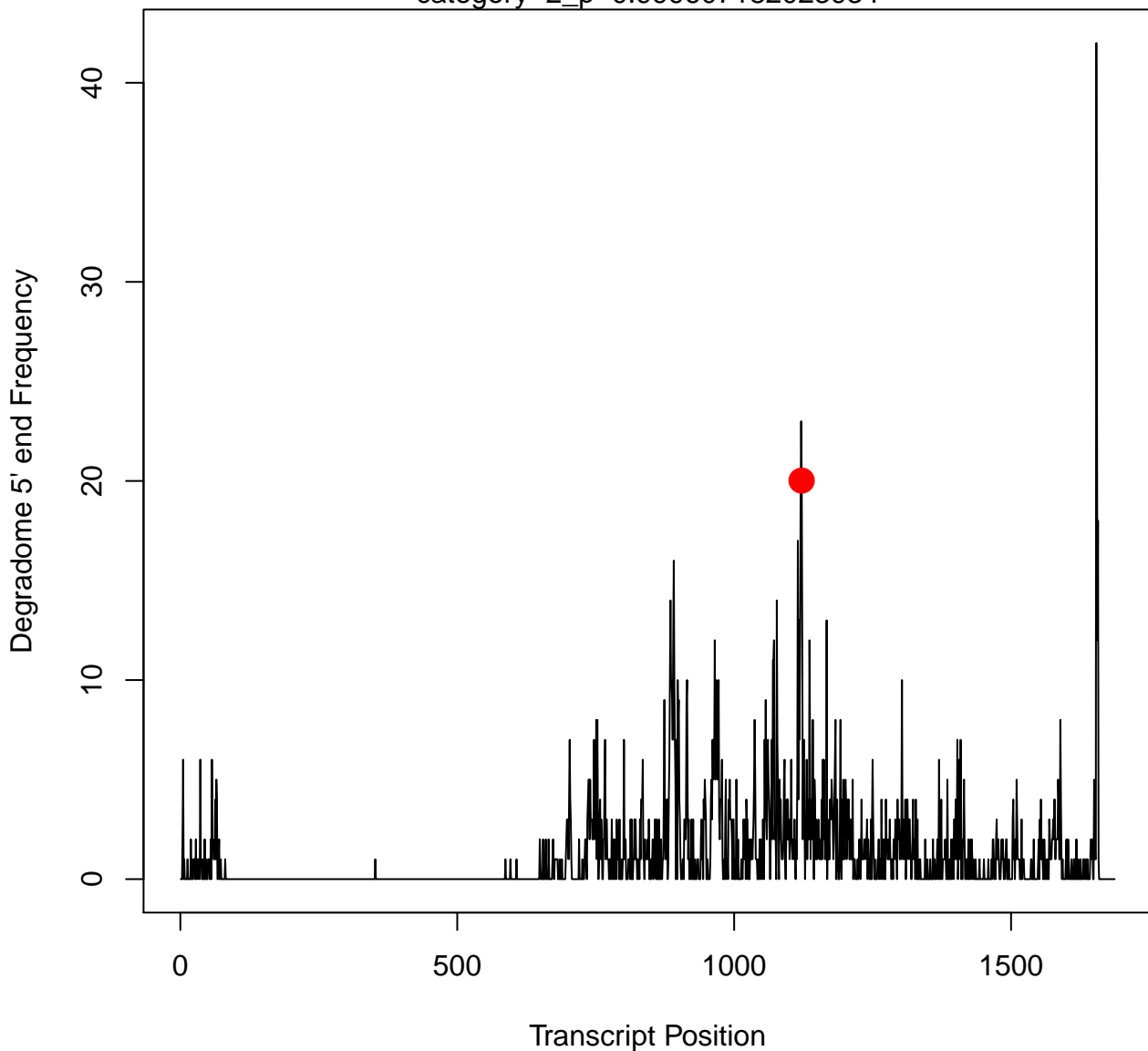

Supplement: Supplementary Data 1 — Results of categories 0–2 from PARE-Seq analysis (including three subfiles:1_1, 1_2, 1_3). [file Data_Sheet_10.ZIP › GSM2230754.plot/Lsa-miR1446_Lsat_1_v5_gn_5_170600.1_1122_TPlot.pdf]

**T=Lsat\_1\_v5\_gn\_5\_182181.1\_Q=Lsa-miR1446\_S=2079**

category=2\_p=0.999973631860563

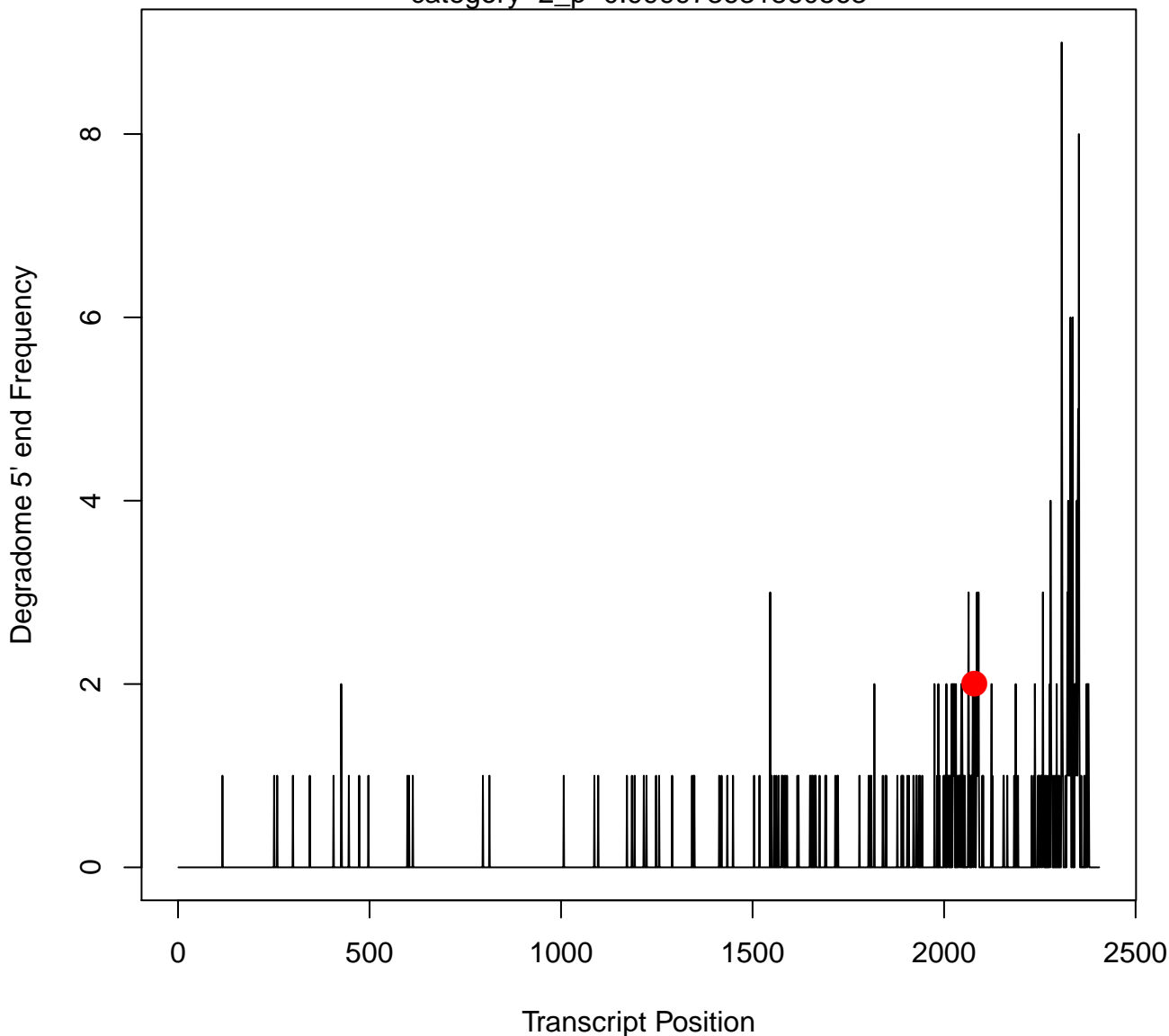

Supplement: Supplementary Data 1 — Results of categories 0–2 from PARE-Seq analysis (including three subfiles:1_1, 1_2, 1_3). [file Data_Sheet_10.ZIP › GSM2230754.plot/Lsa-miR1446_Lsat_1_v5_gn_5_182181.1_2079_TPlot.pdf]

**T=Lsat\_1\_v5\_gn\_6\_109860.1\_Q=Lsa-miR1446\_S=2856**

category=2\_p=0.658588556030749

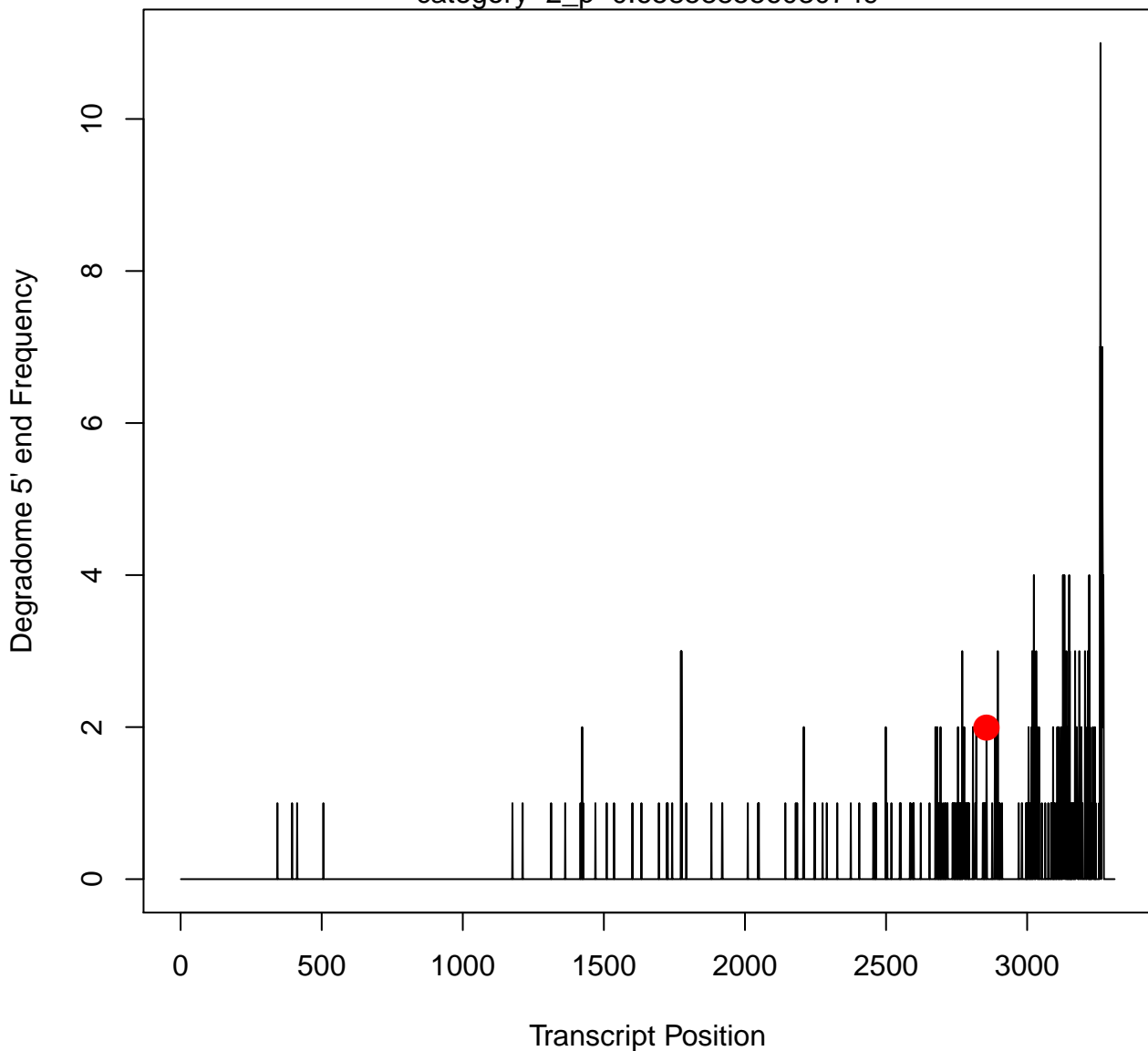

Supplement: Supplementary Data 1 — Results of categories 0–2 from PARE-Seq analysis (including three subfiles:1_1, 1_2, 1_3). [file Data_Sheet_10.ZIP › GSM2230754.plot/Lsa-miR1446_Lsat_1_v5_gn_6_109860.1_2856_TPlot.pdf]

**T=Lsat\_1\_v5\_gn\_6\_8501.1\_Q=Lsa-miR1446\_S=2875**

category=2\_p=0.803387983003733

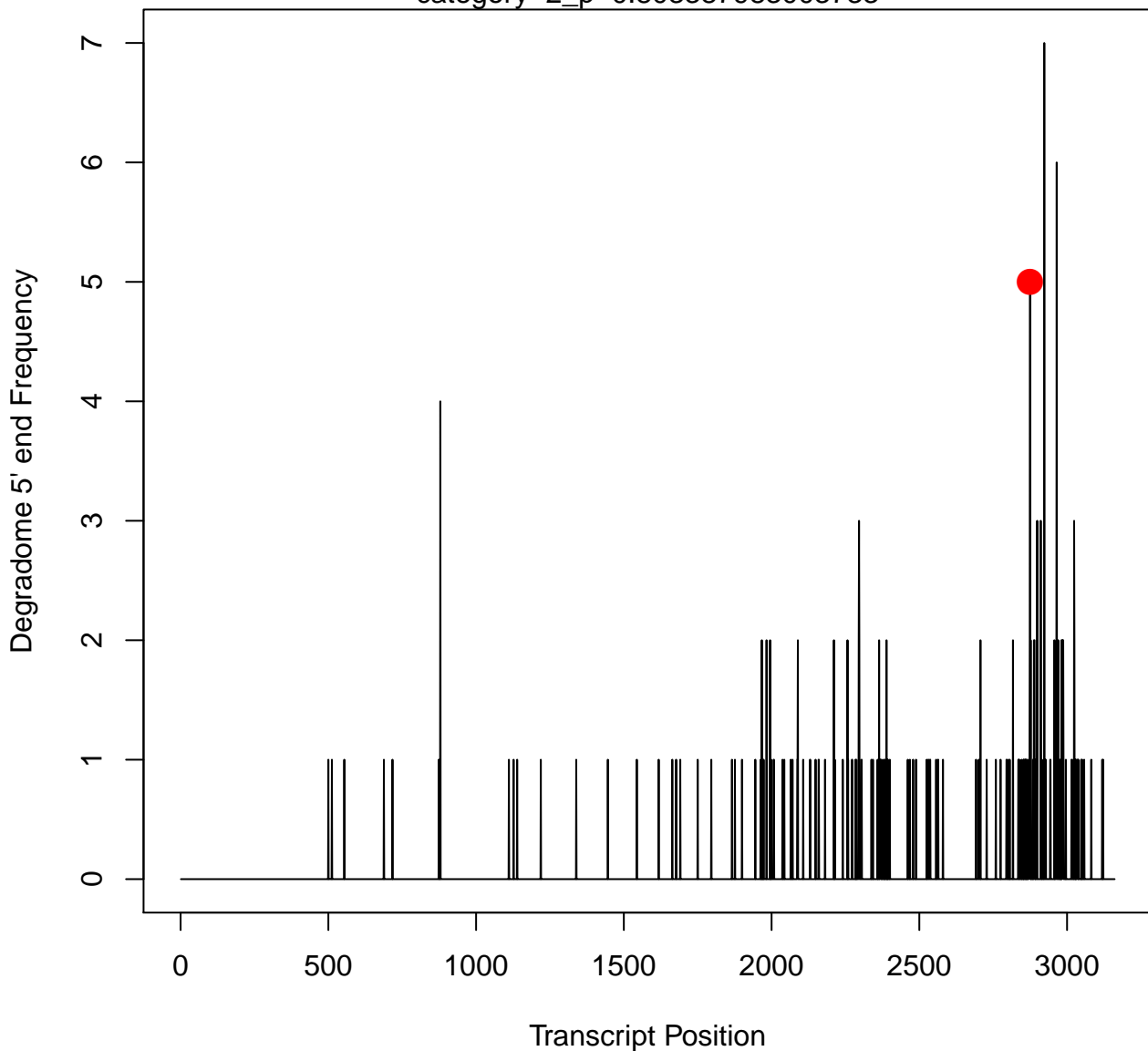

Supplement: Supplementary Data 1 — Results of categories 0–2 from PARE-Seq analysis (including three subfiles:1_1, 1_2, 1_3). [file Data_Sheet_10.ZIP › GSM2230754.plot/Lsa-miR1446_Lsat_1_v5_gn_6_8501.1_2875_TPlot.pdf]

**T=Lsat\_1\_v5\_gn\_8\_340.1\_Q=Lsa-miR1446\_S=2192**

category=2\_p=0.999639961423964

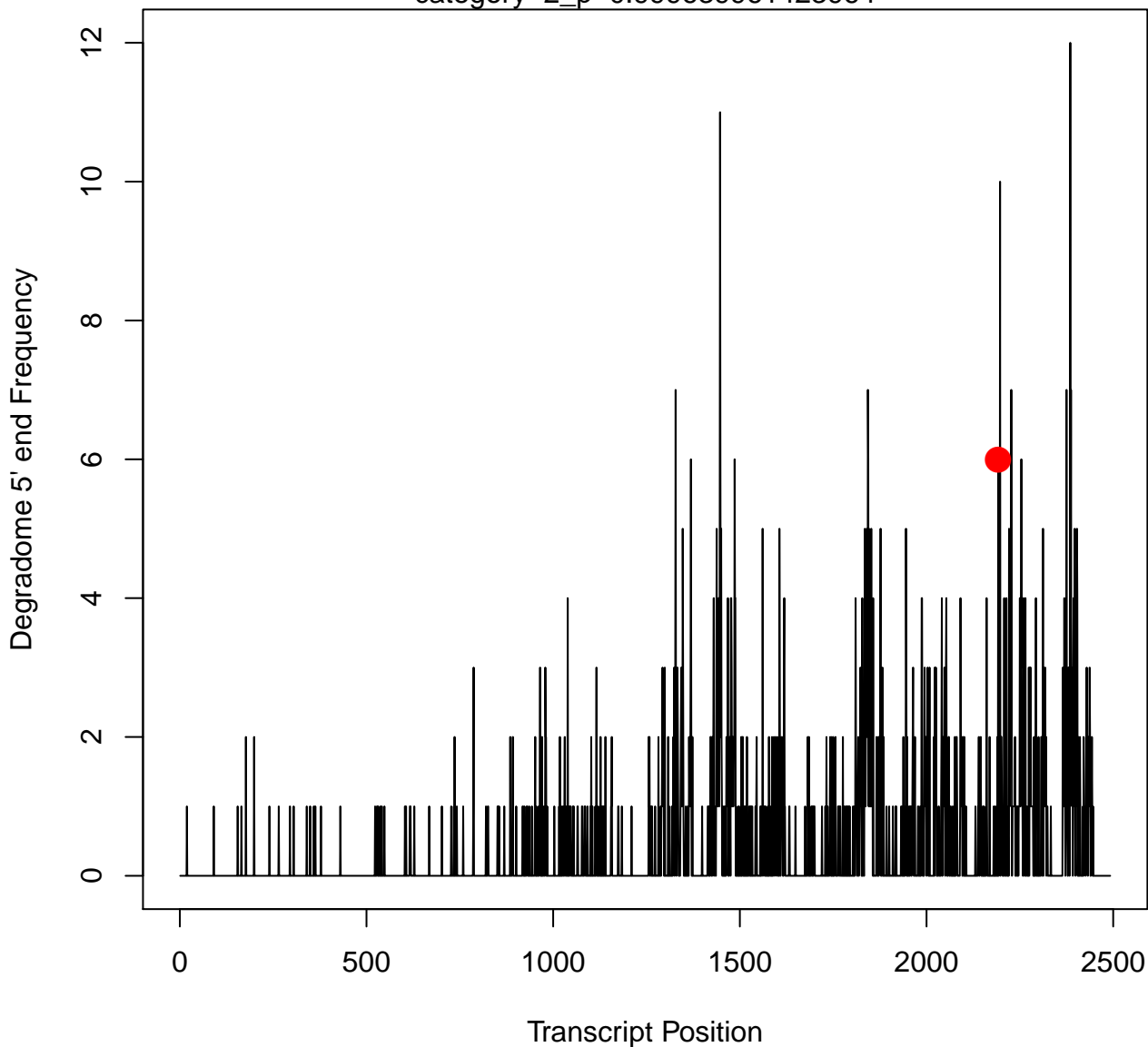

Supplement: Supplementary Data 1 — Results of categories 0–2 from PARE-Seq analysis (including three subfiles:1_1, 1_2, 1_3). [file Data_Sheet_10.ZIP › GSM2230754.plot/Lsa-miR1446_Lsat_1_v5_gn_8_340.1_2192_TPlot.pdf]

**T=Lsat\_1\_v5\_gn\_9\_23820.1\_Q=Lsa-miR1446\_S=1662**

category=2\_p=0.999738426424842

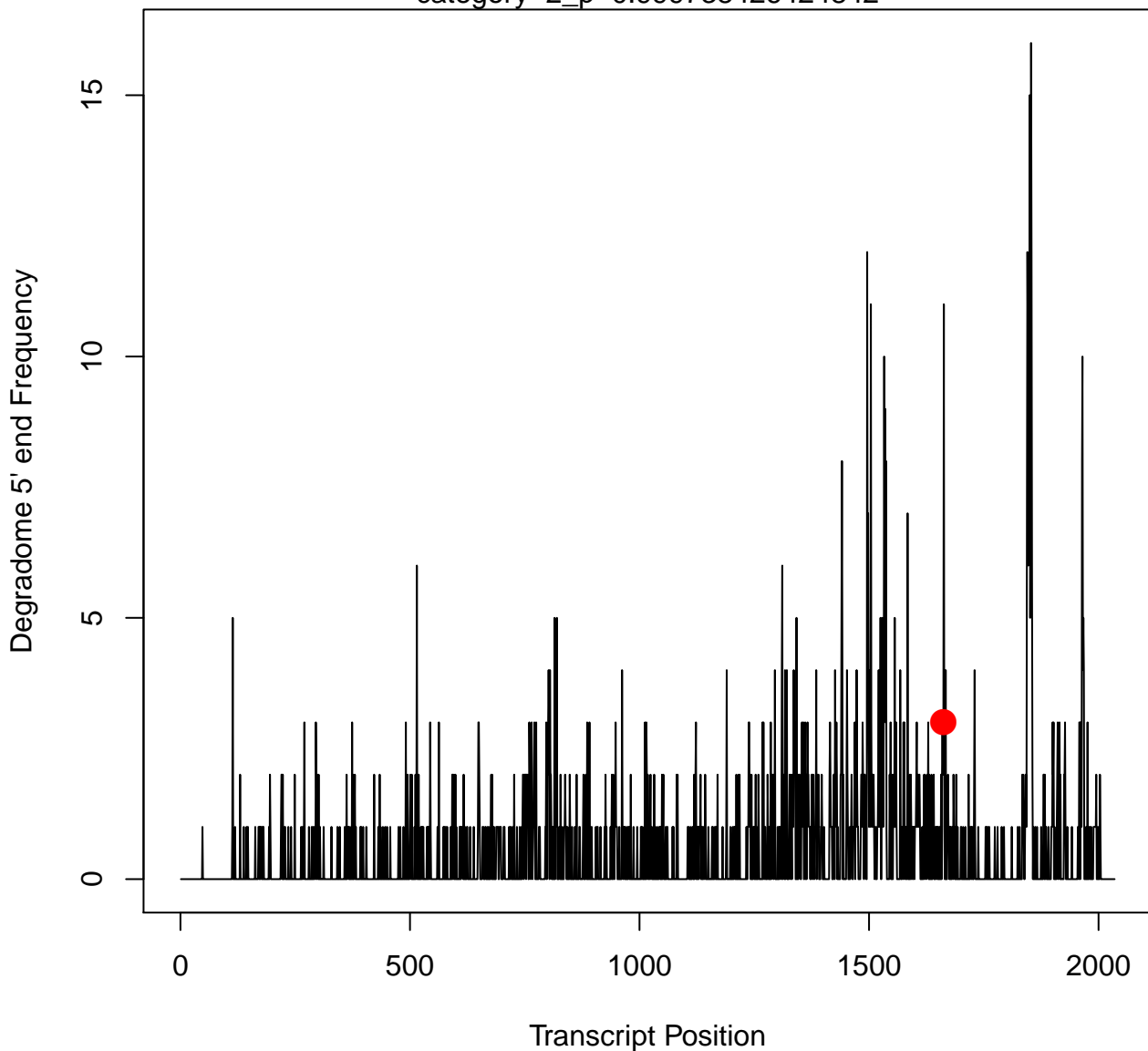

Supplement: Supplementary Data 1 — Results of categories 0–2 from PARE-Seq analysis (including three subfiles:1_1, 1_2, 1_3). [file Data_Sheet_10.ZIP › GSM2230754.plot/Lsa-miR1446_Lsat_1_v5_gn_9_23820.1_1662_TPlot.pdf]

**T=Lsat\_1\_v5\_gn\_7\_11040.1\_Q=Lsa-miR156a\_S=1087**

category=0\_p=0.00187750294176836

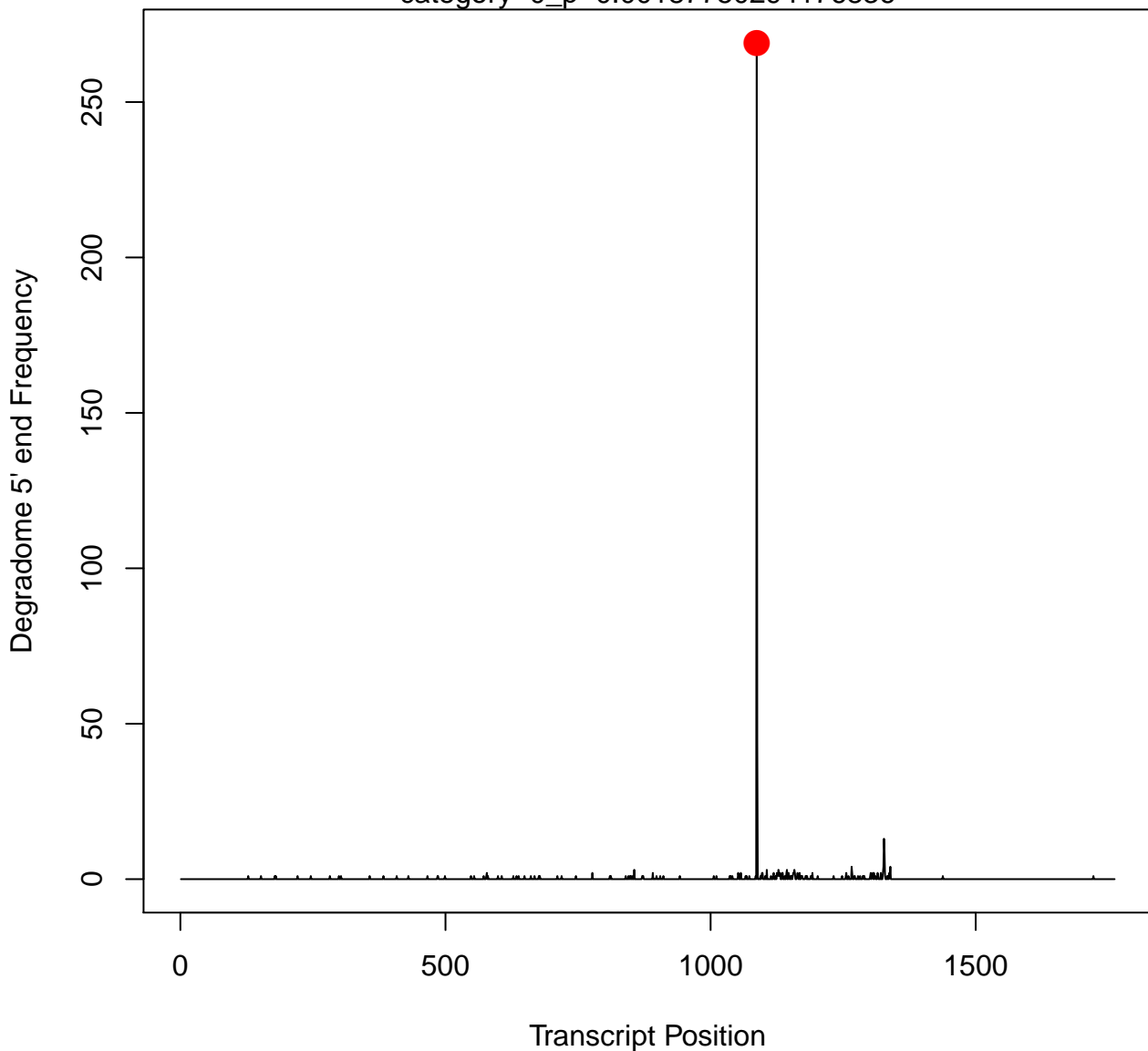

Supplement: Supplementary Data 1 — Results of categories 0–2 from PARE-Seq analysis (including three subfiles:1_1, 1_2, 1_3). [file Data_Sheet_10.ZIP › GSM2230754.plot/Lsa-miR156a_Lsat_1_v5_gn_7_11040.1_1087_TPlot.pdf]

**T=Lsat\_1\_v5\_gn\_3\_31000.1\_Q=Lsa-miR156b\_S=849**

category=2\_p=0.0564350766159708

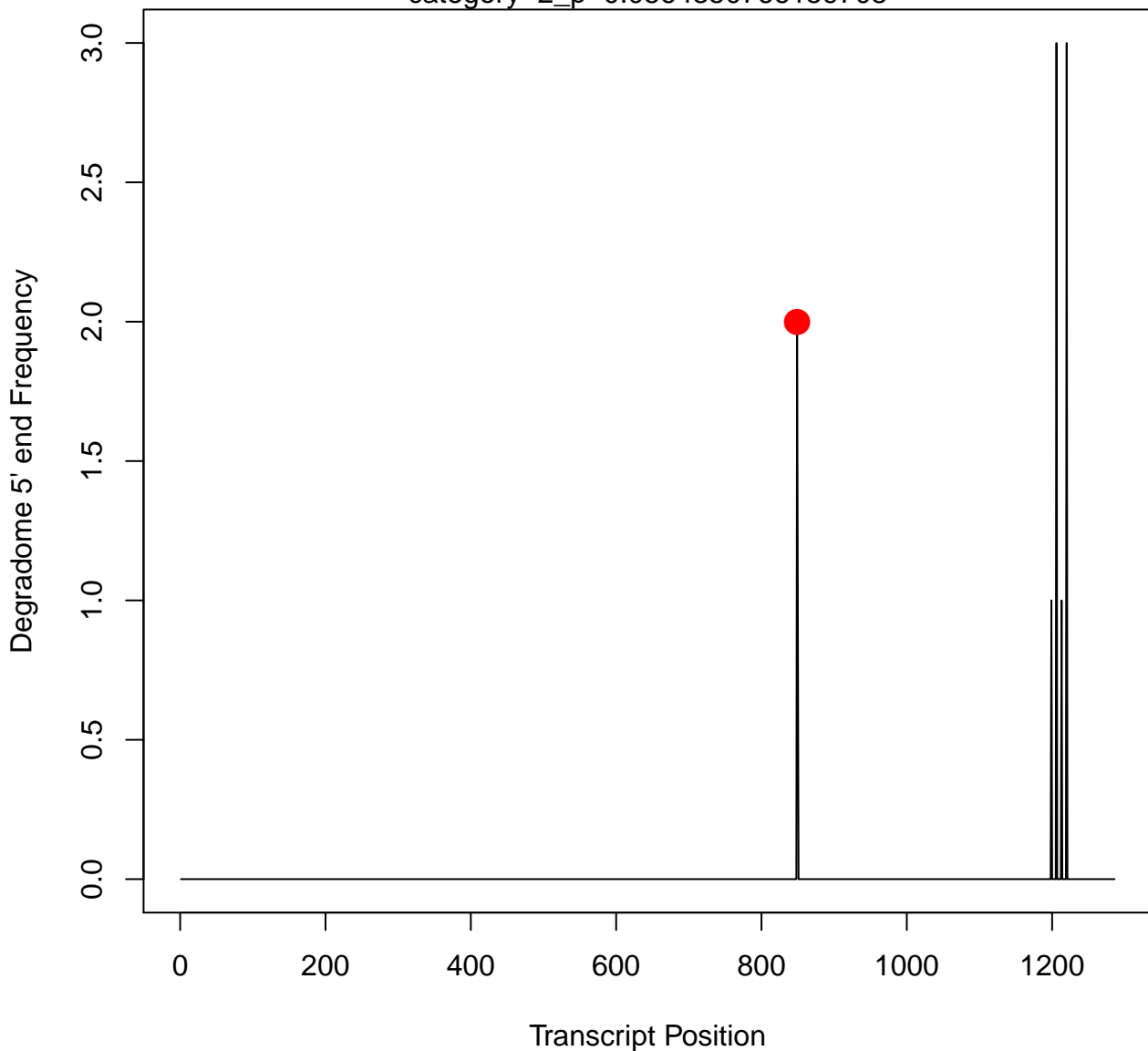

Supplement: Supplementary Data 1 — Results of categories 0–2 from PARE-Seq analysis (including three subfiles:1_1, 1_2, 1_3). [file Data_Sheet_10.ZIP › GSM2230754.plot/Lsa-miR156b_Lsat_1_v5_gn_3_31000.1_849_TPlot.pdf]

**T=Lsat\_1\_v5\_gn\_3\_4041.1\_Q=Lsa-miR156c\_S=207**

category=2\_p=0.936662645338248

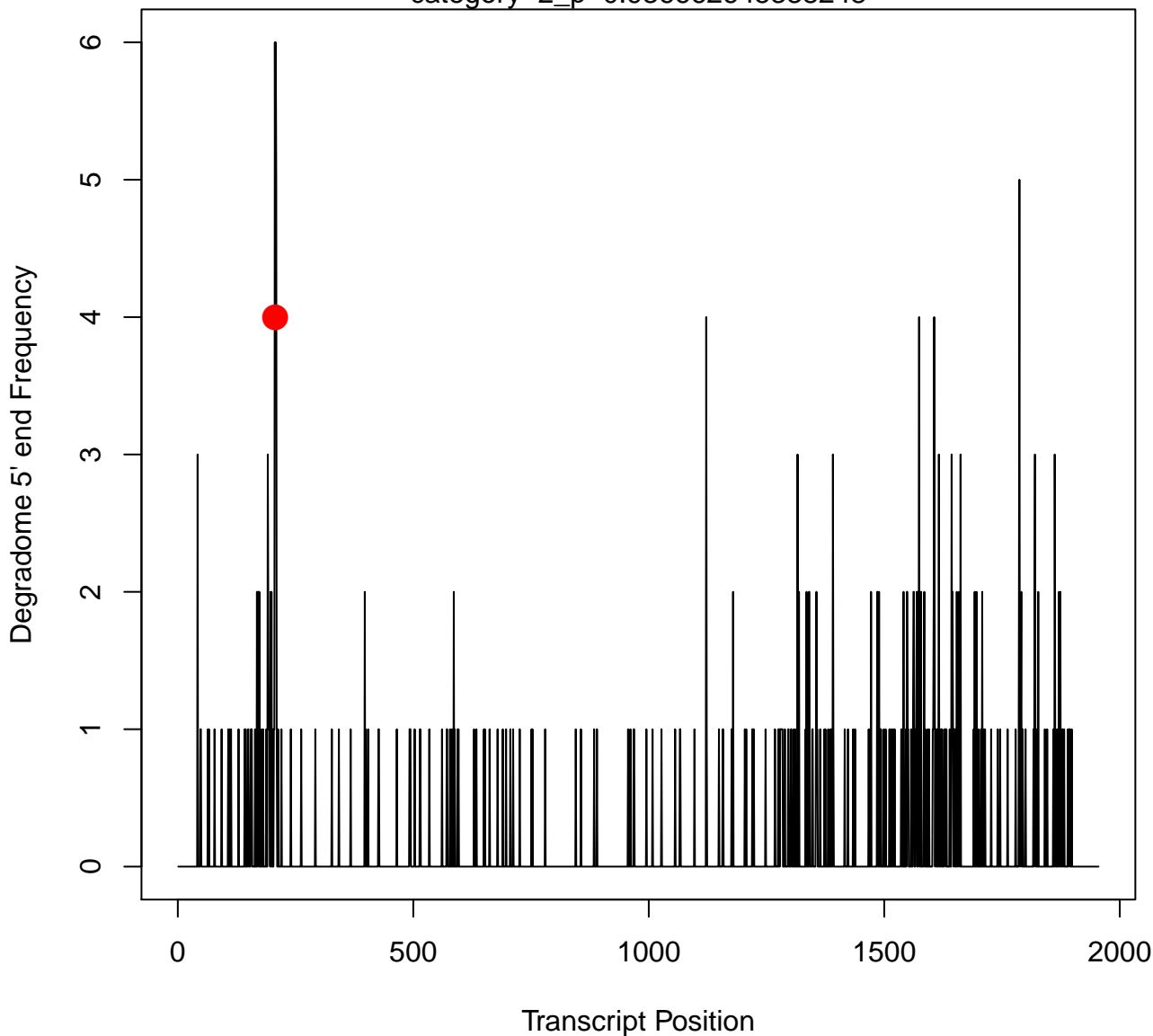

Supplement: Supplementary Data 1 — Results of categories 0–2 from PARE-Seq analysis (including three subfiles:1_1, 1_2, 1_3). [file Data_Sheet_10.ZIP › GSM2230754.plot/Lsa-miR156c_Lsat_1_v5_gn_3_4041.1_207_TPlot.pdf]

**T=Lsat\_1\_v5\_gn\_1\_50821.1\_Q=Lsa-miR156h\_S=1265**

category=2\_p=0.932874407375598

Degradome 5' end Frequency

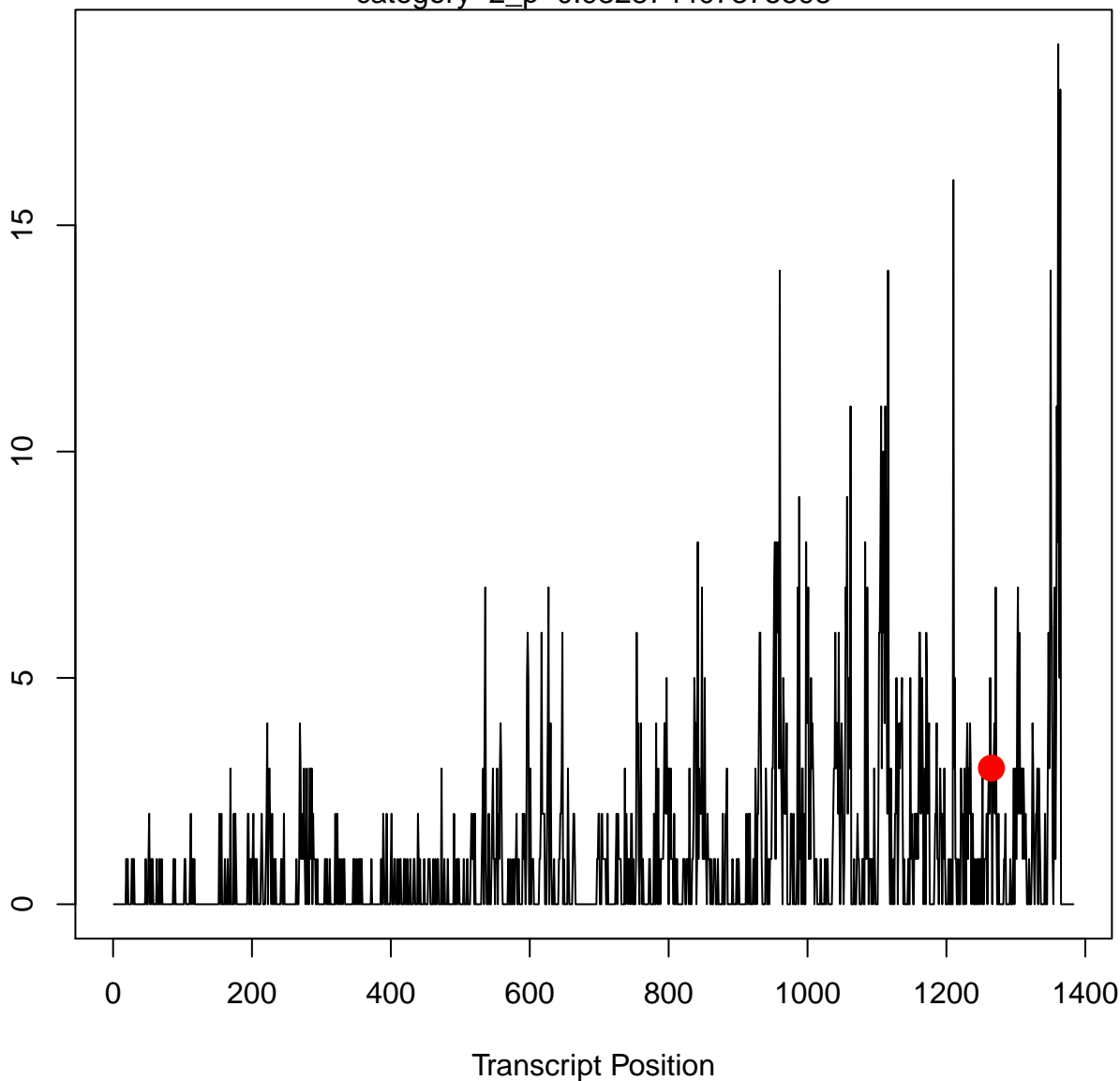

Supplement: Supplementary Data 1 — Results of categories 0–2 from PARE-Seq analysis (including three subfiles:1_1, 1_2, 1_3). [file Data_Sheet_10.ZIP › GSM2230754.plot/Lsa-miR156h_Lsat_1_v5_gn_1_50821.1_1265_TPlot.pdf]

T=Lsat\_1\_v5\_gn\_4\_81541.1\_Q=Lsa-miR156i\_S=850

category=0\_p=0.00375148086624022

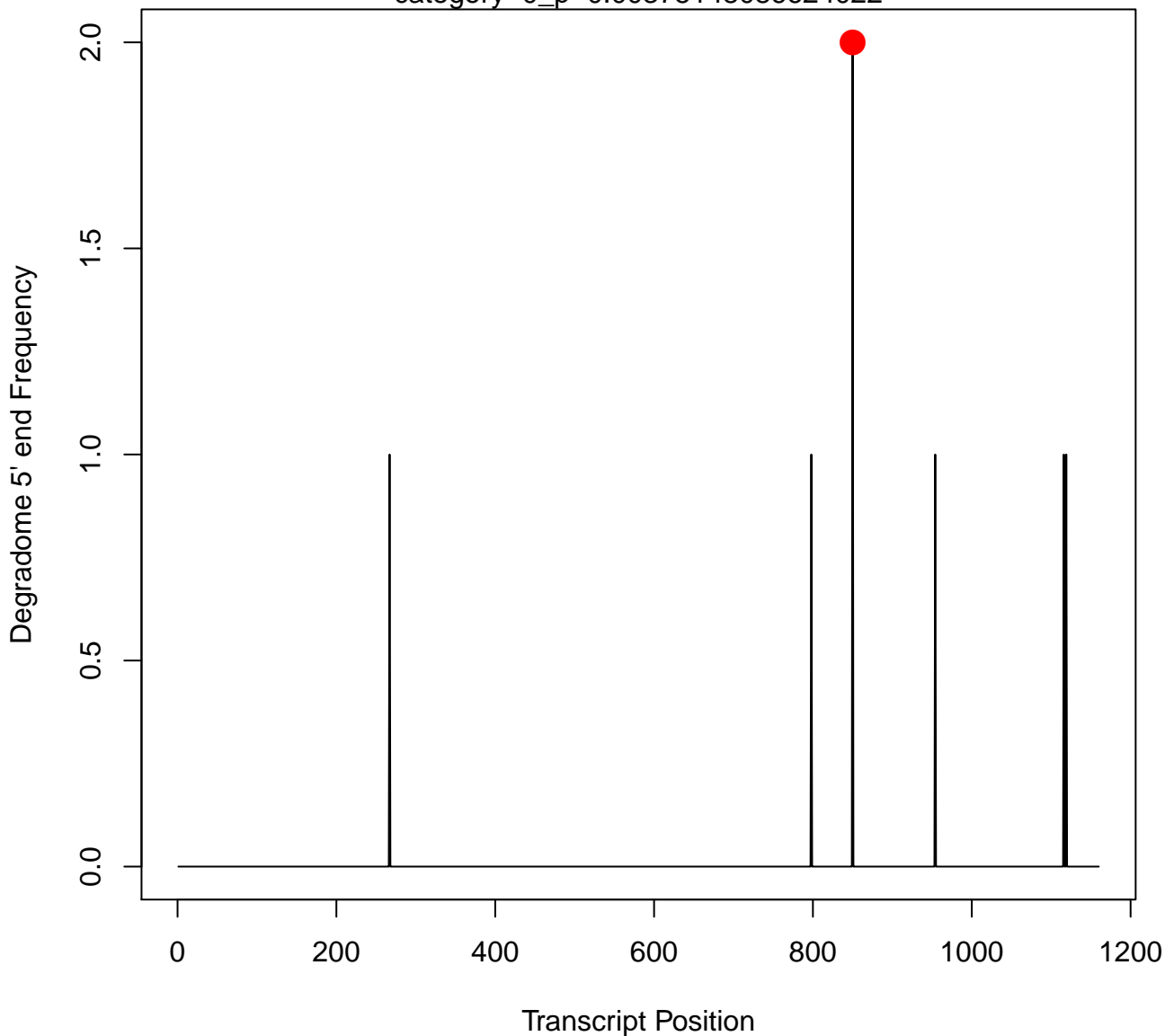

Supplement: Supplementary Data 1 — Results of categories 0–2 from PARE-Seq analysis (including three subfiles:1_1, 1_2, 1_3). [file Data_Sheet_10.ZIP › GSM2230754.plot/Lsa-miR156i_Lsat_1_v5_gn_4_81541.1_850_TPlot.pdf]

**T=Lsat\_1\_v5\_gn\_5\_12360.1\_Q=Lsa-miR156i\_S=1859**

category=0\_p=0.00112692513817547

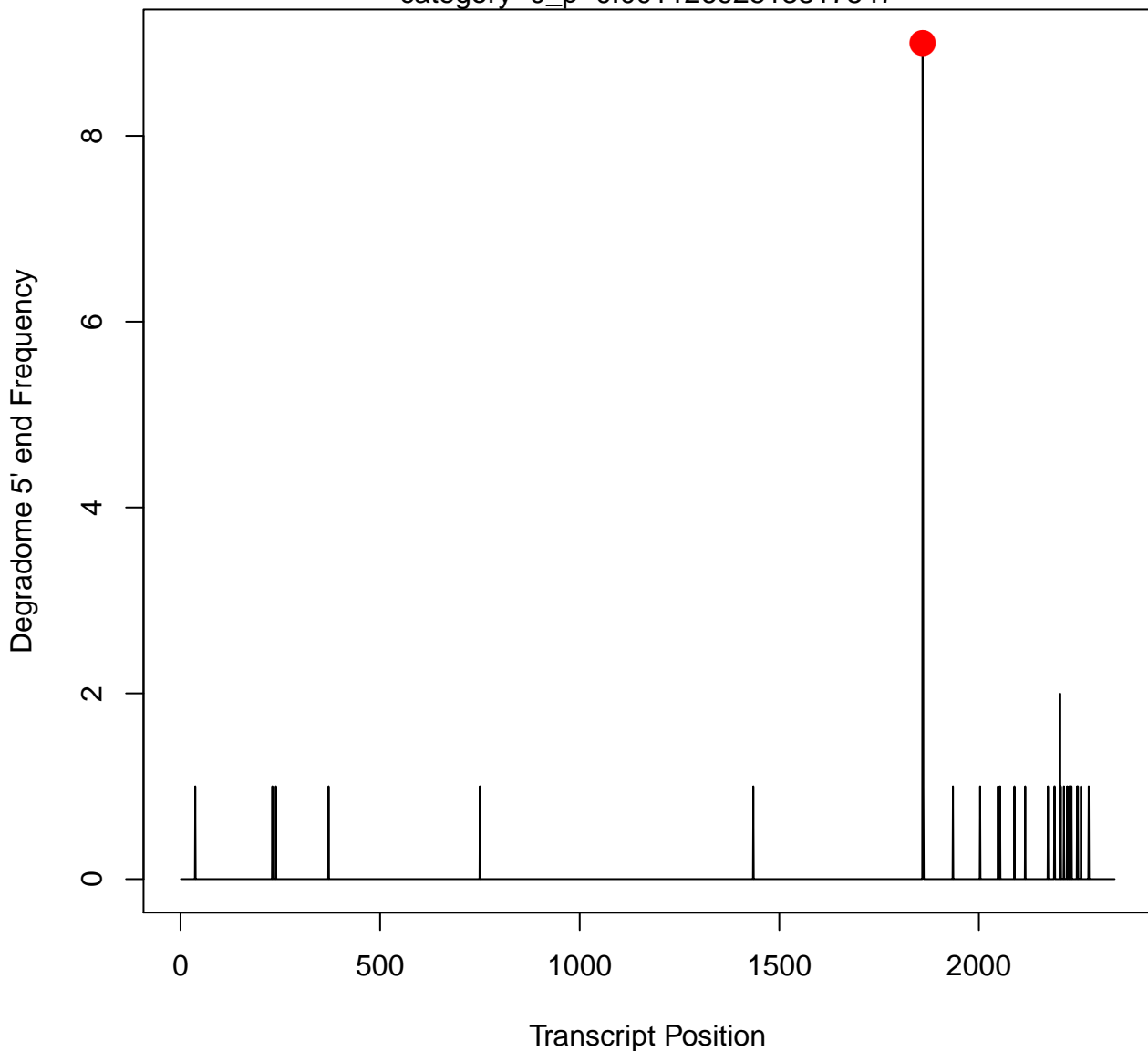

Supplement: Supplementary Data 1 — Results of categories 0–2 from PARE-Seq analysis (including three subfiles:1_1, 1_2, 1_3). [file Data_Sheet_10.ZIP › GSM2230754.plot/Lsa-miR156i_Lsat_1_v5_gn_5_12360.1_1859_TPlot.pdf]

**T=Lsat\_1\_v5\_gn\_8\_134640.1\_Q=Lsa-miR156i\_S=546**

category=2\_p=0.109685235359291

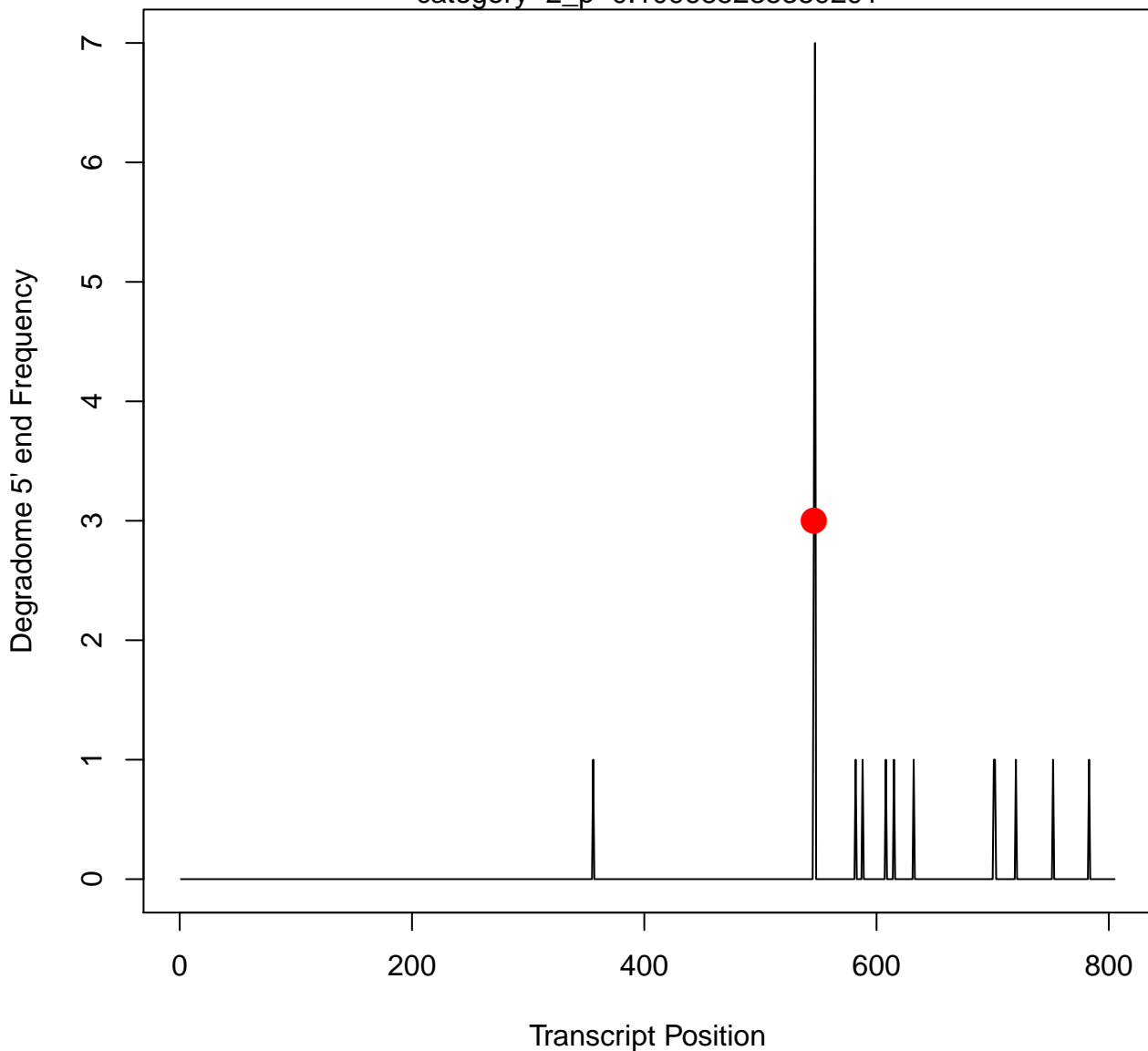

Supplement: Supplementary Data 1 — Results of categories 0–2 from PARE-Seq analysis (including three subfiles:1_1, 1_2, 1_3). [file Data_Sheet_10.ZIP › GSM2230754.plot/Lsa-miR156i_Lsat_1_v5_gn_8_134640.1_546_TPlot.pdf]

**T=Lsat\_1\_v5\_gn\_9\_28021.1\_Q=Lsa-miR156i\_S=1267**

category=2\_p=0.0564350766159708

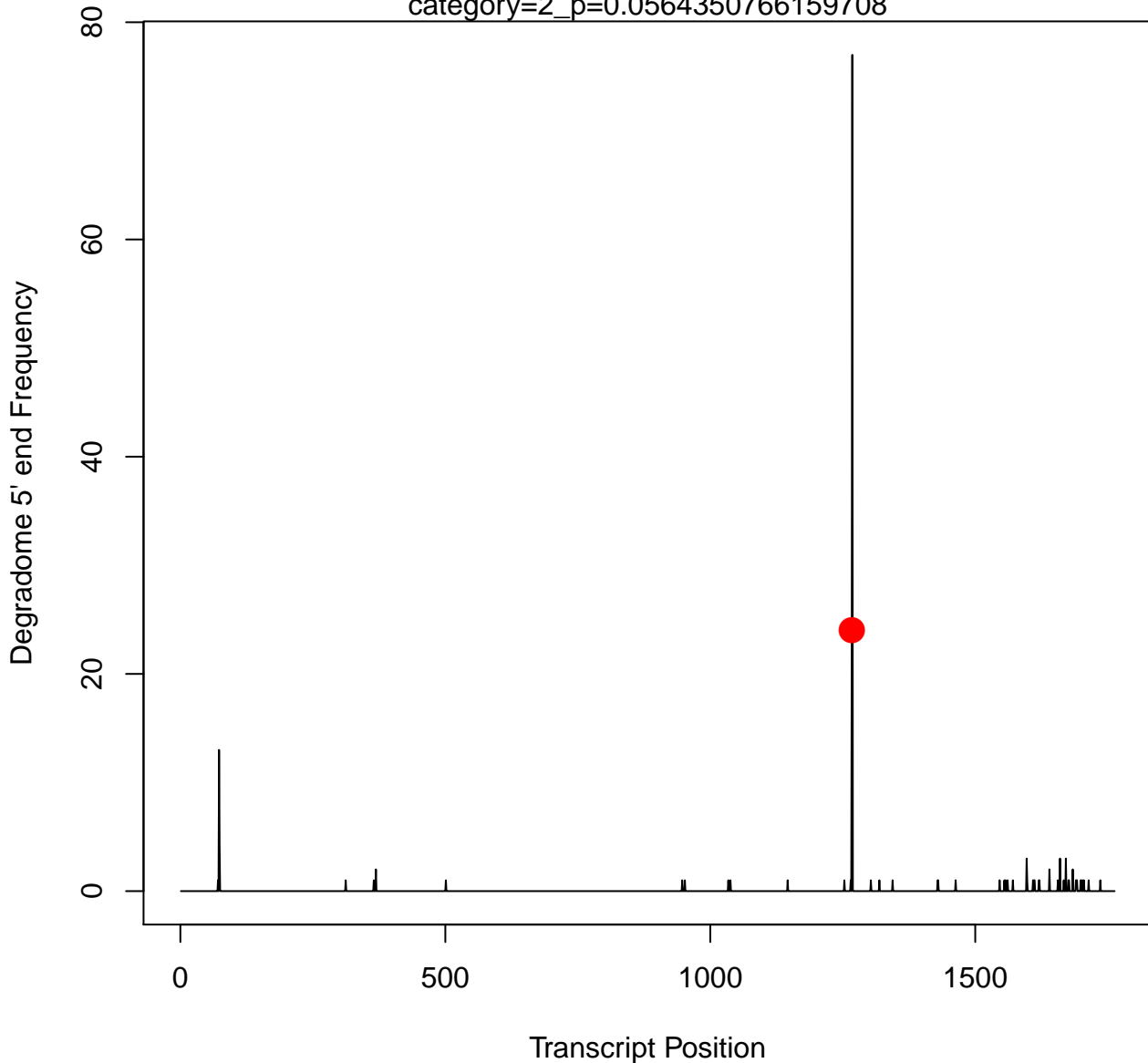

Supplement: Supplementary Data 1 — Results of categories 0–2 from PARE-Seq analysis (including three subfiles:1_1, 1_2, 1_3). [file Data_Sheet_10.ZIP › GSM2230754.plot/Lsa-miR156i_Lsat_1_v5_gn_9_28021.1_1267_TPlot.pdf]

**T=Lsat\_1\_v5\_gn\_9\_65321.1\_Q=Lsa-miR156i\_S=1998**

category=2\_p=0.45661984672614

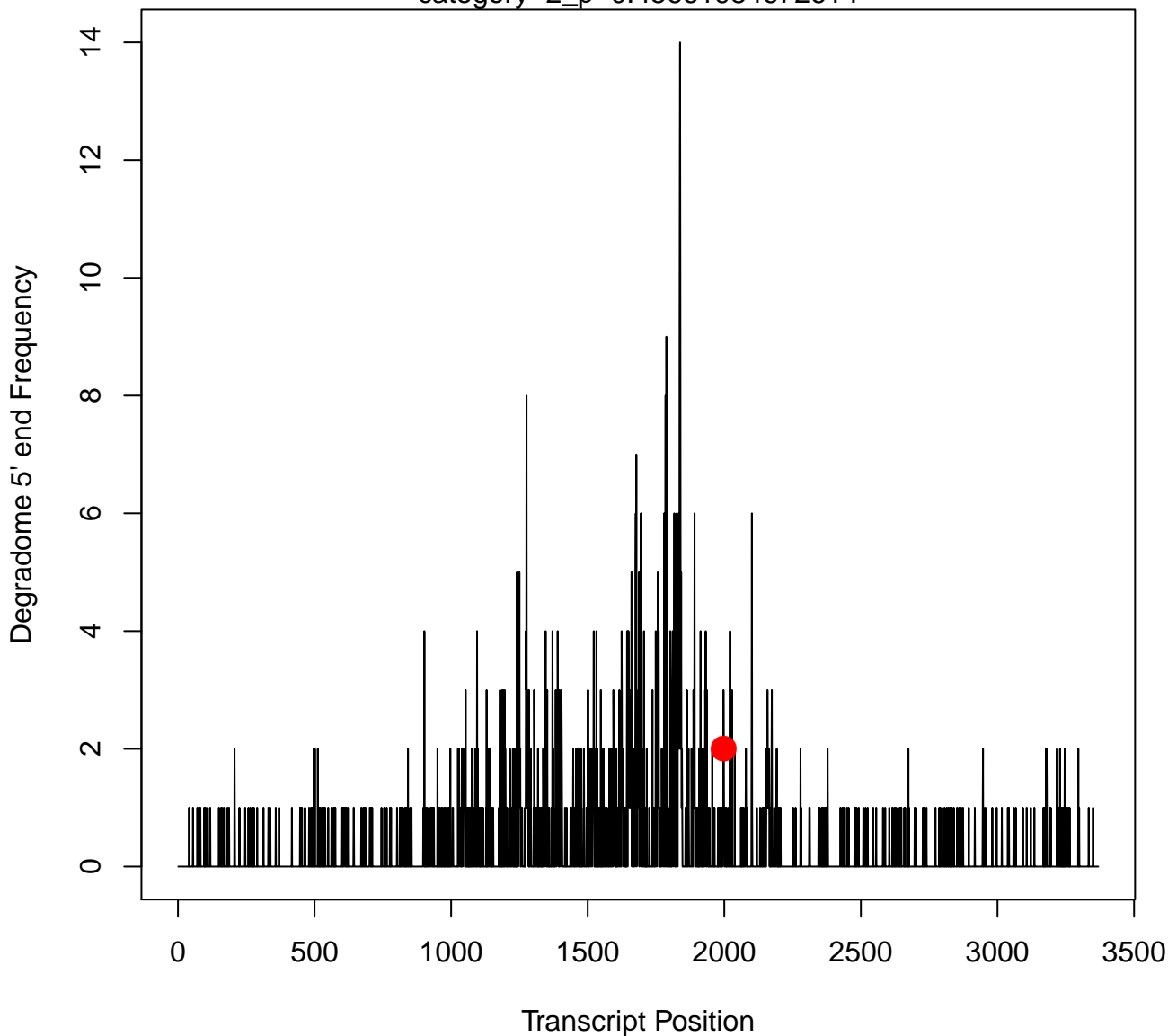

Supplement: Supplementary Data 1 — Results of categories 0–2 from PARE-Seq analysis (including three subfiles:1_1, 1_2, 1_3). [file Data_Sheet_10.ZIP › GSM2230754.plot/Lsa-miR156i_Lsat_1_v5_gn_9_65321.1_1998_TPlot.pdf]

T=Lsat\_1\_v5\_gn\_1\_126340.1\_Q=Lsa-miR156j\_S=1034

category=2\_p=0.924604650747883

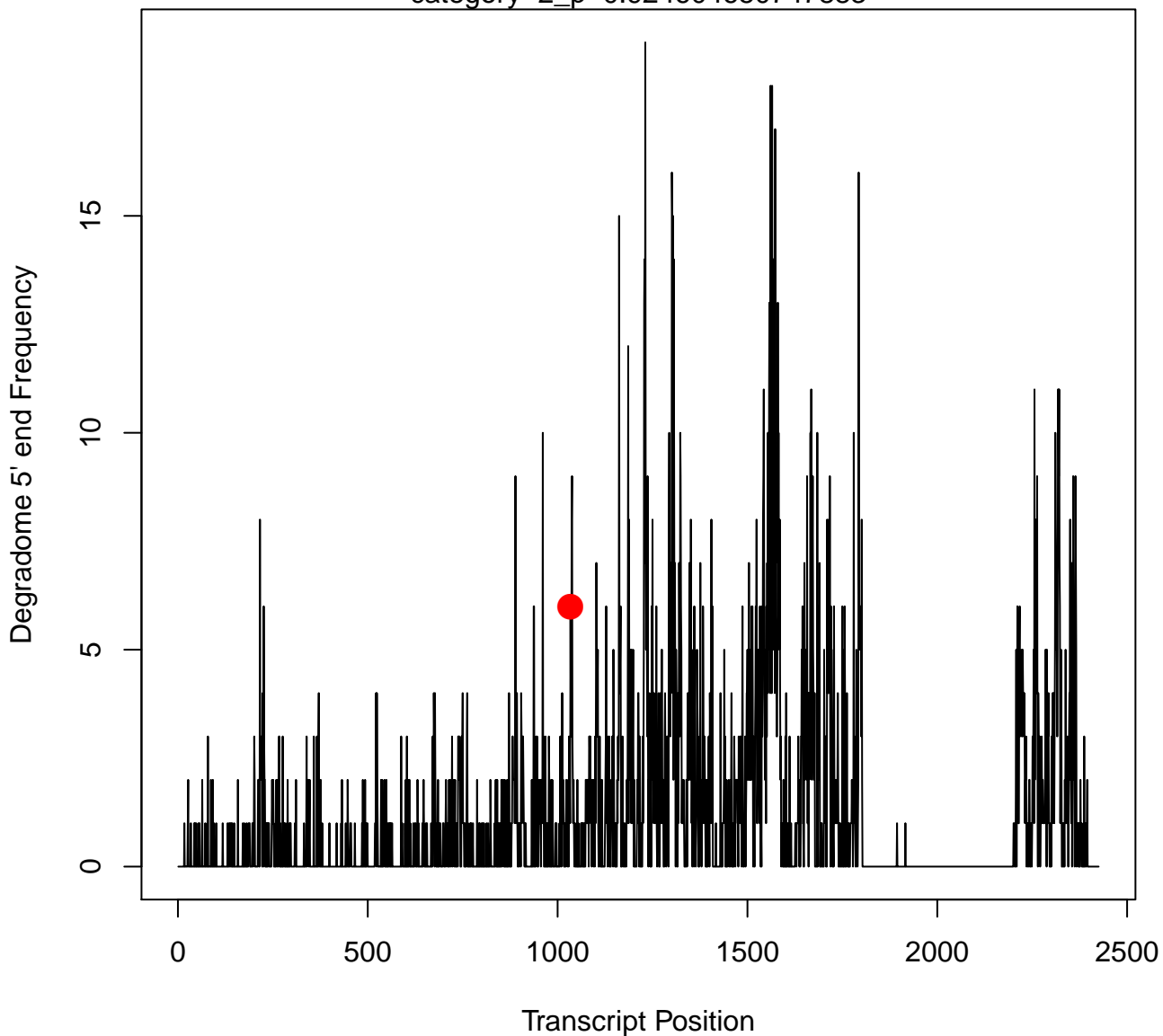

Supplement: Supplementary Data 1 — Results of categories 0–2 from PARE-Seq analysis (including three subfiles:1_1, 1_2, 1_3). [file Data_Sheet_10.ZIP › GSM2230754.plot/Lsa-miR156j_Lsat_1_v5_gn_1_126340.1_1034_TPlot.pdf]

T=Lsat\_1\_v5\_gn\_1\_14560.1\_Q=Lsa-miR156j\_S=192

category=0\_p=0.00037578290783058

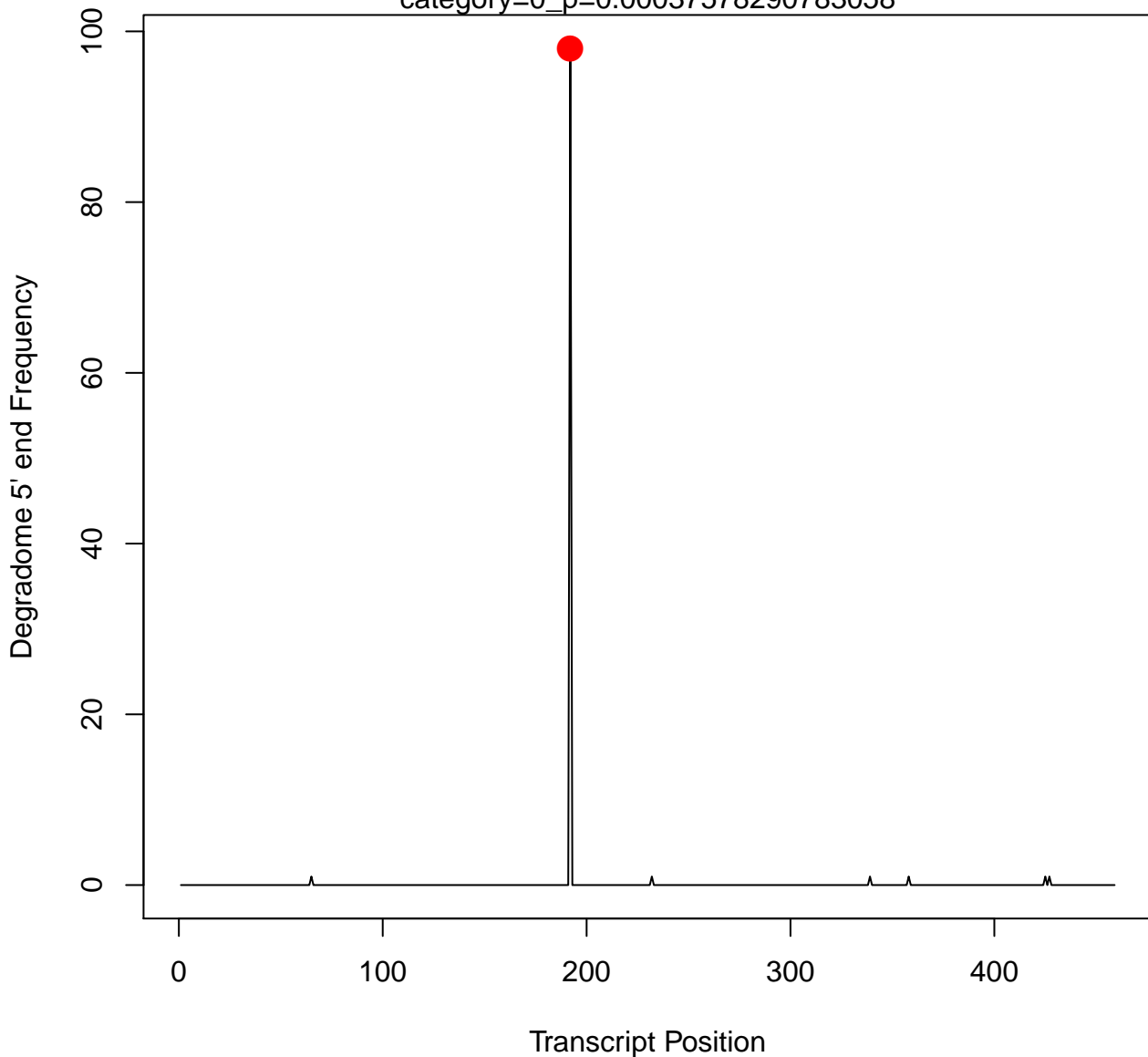

Supplement: Supplementary Data 1 — Results of categories 0–2 from PARE-Seq analysis (including three subfiles:1_1, 1_2, 1_3). [file Data_Sheet_10.ZIP › GSM2230754.plot/Lsa-miR156j_Lsat_1_v5_gn_1_14560.1_192_TPlot.pdf]

**T=Lsat\_1\_v5\_gn\_2\_136800.1\_Q=Lsa-miR156j\_S=2455**

category=2\_p=0.967526022869625

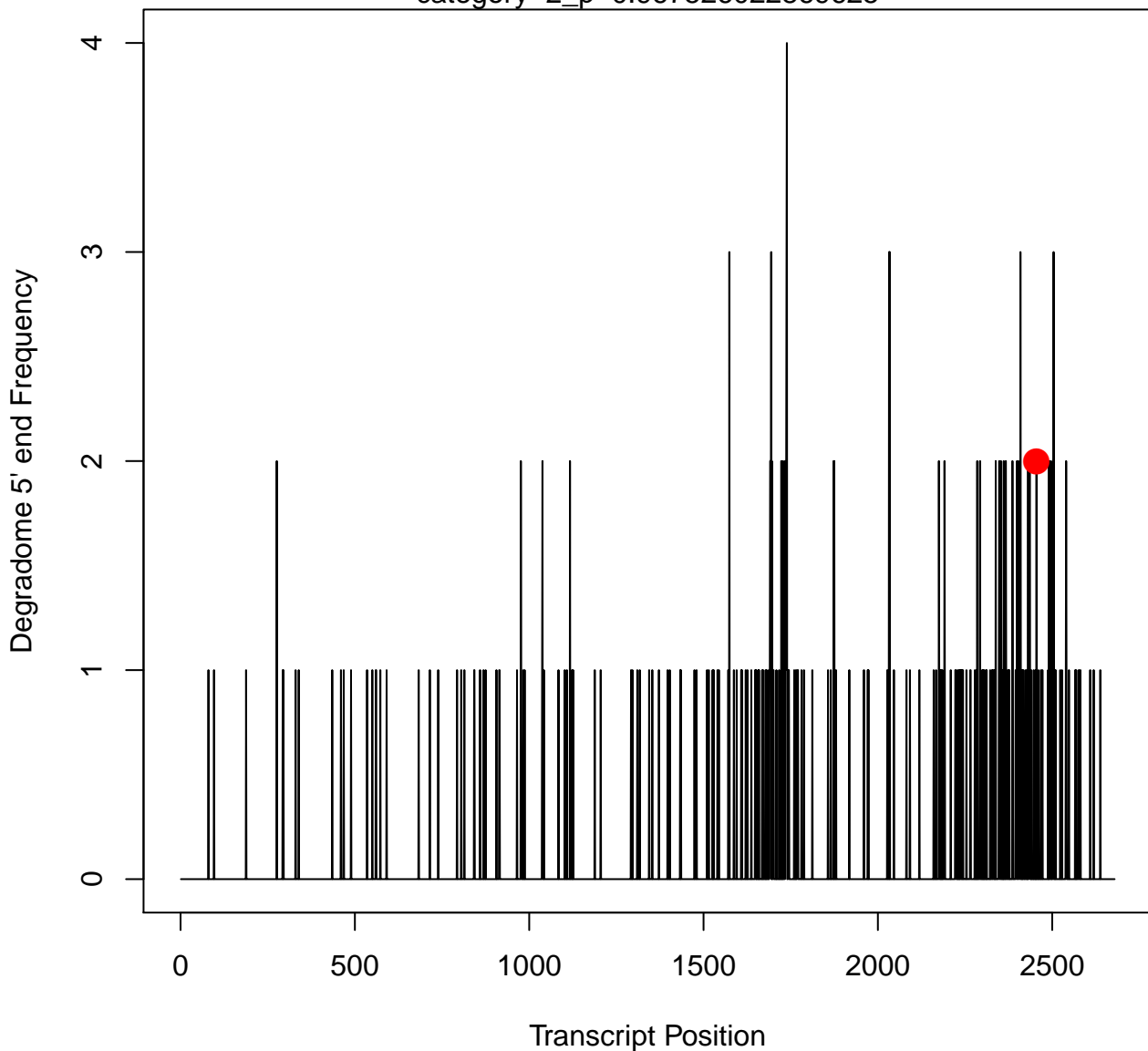

Supplement: Supplementary Data 1 — Results of categories 0–2 from PARE-Seq analysis (including three subfiles:1_1, 1_2, 1_3). [file Data_Sheet_10.ZIP › GSM2230754.plot/Lsa-miR156j_Lsat_1_v5_gn_2_136800.1_2455_TPlot.pdf]

**T=Lsat\_1\_v5\_gn\_4\_139200.1\_Q=Lsa-miR156j\_S=170**

category=0\_p=0.00860737384568244

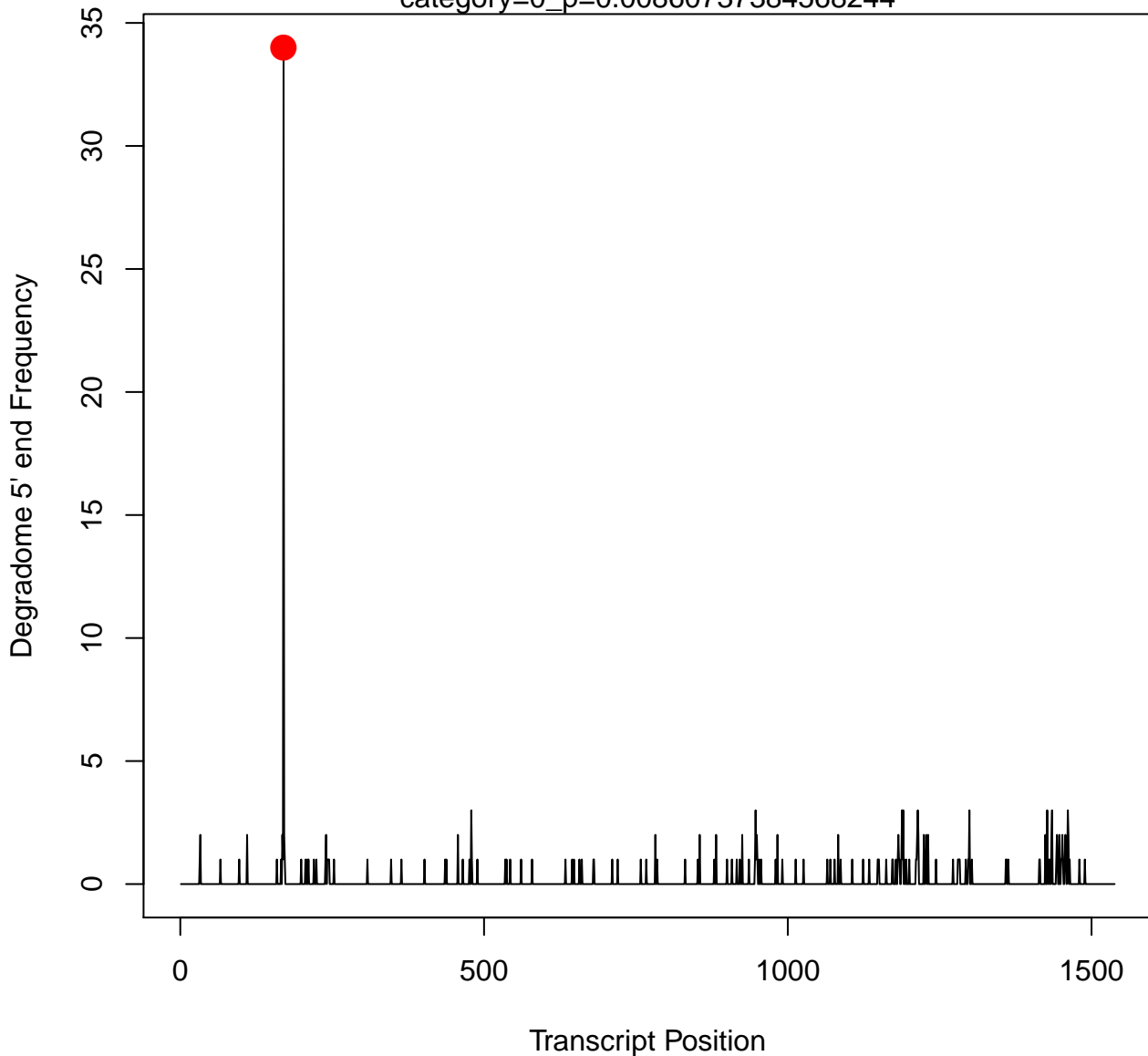

Supplement: Supplementary Data 1 — Results of categories 0–2 from PARE-Seq analysis (including three subfiles:1_1, 1_2, 1_3). [file Data_Sheet_10.ZIP › GSM2230754.plot/Lsa-miR156j_Lsat_1_v5_gn_4_139200.1_170_TPlot.pdf]

**T=Lsat\_1\_v5\_gn\_4\_64540.1\_Q=Lsa-miR156j\_S=748**

category=2\_p=0.910251087700046

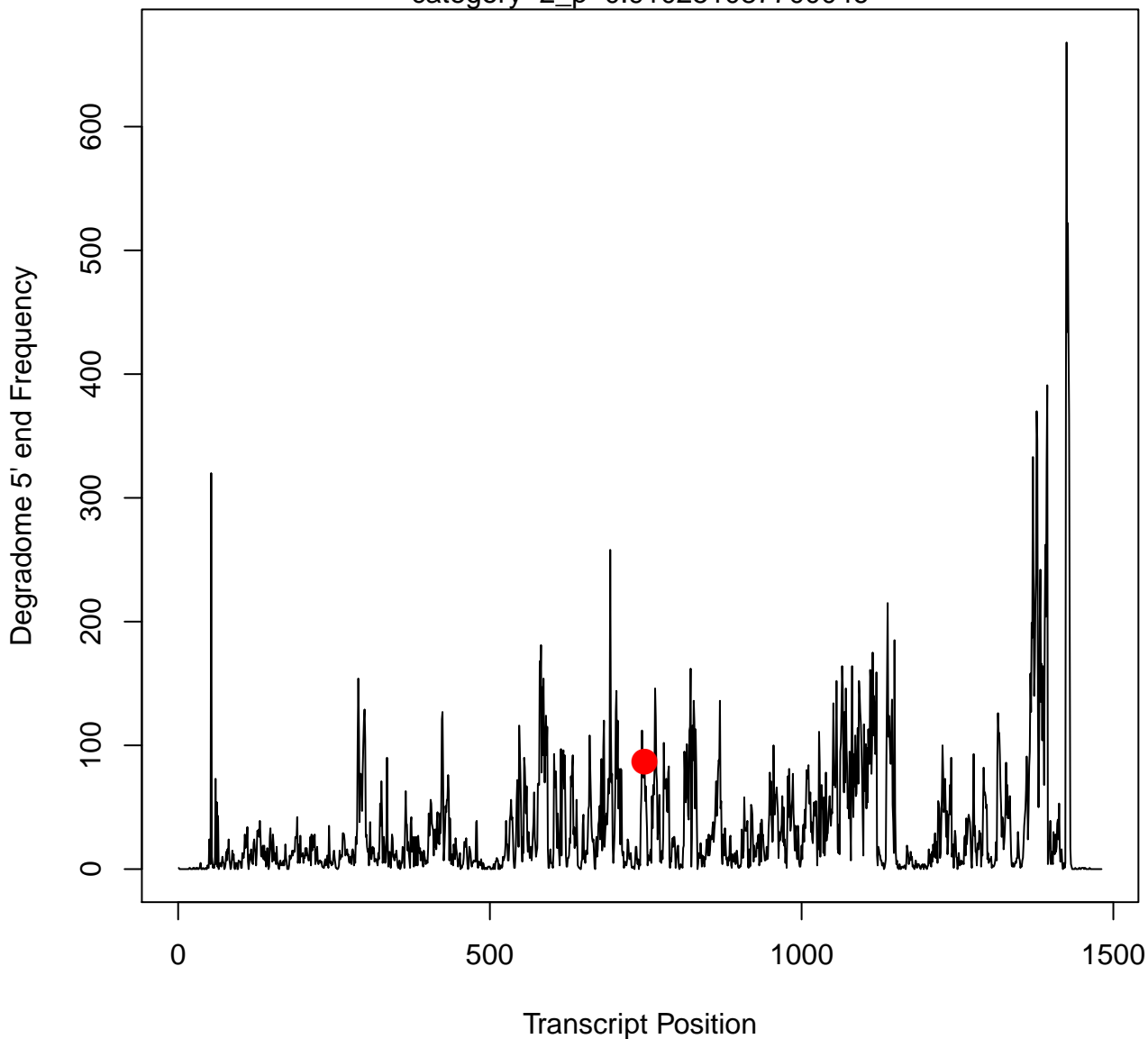

Supplement: Supplementary Data 1 — Results of categories 0–2 from PARE-Seq analysis (including three subfiles:1_1, 1_2, 1_3). [file Data_Sheet_10.ZIP › GSM2230754.plot/Lsa-miR156j_Lsat_1_v5_gn_4_64540.1_748_TPlot.pdf]

**T=Lsat\_1\_v5\_gn\_4\_7021.1\_Q=Lsa-miR156j\_S=627**

category=2\_p=0.819794625134612

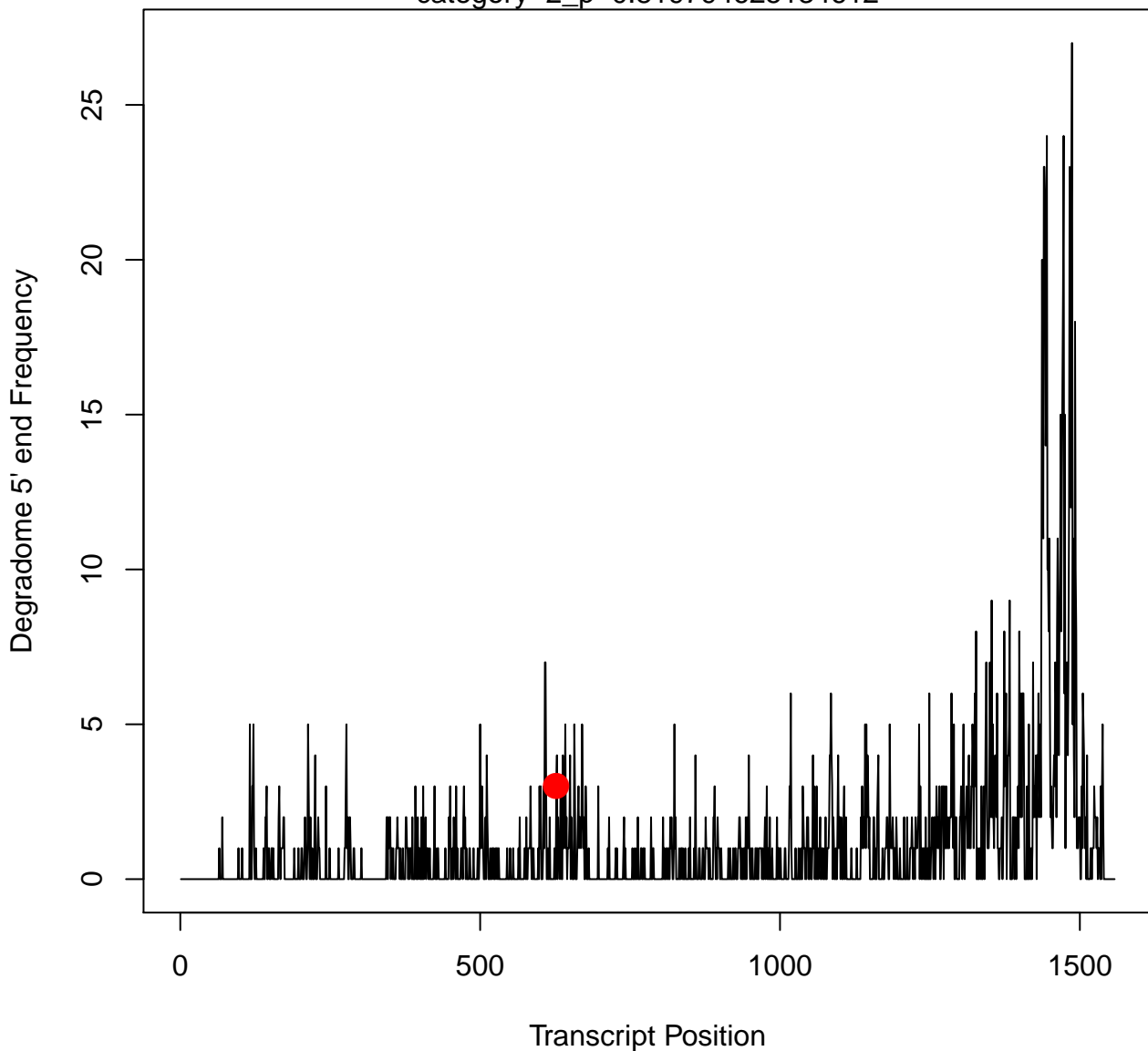

Supplement: Supplementary Data 1 — Results of categories 0–2 from PARE-Seq analysis (including three subfiles:1_1, 1_2, 1_3). [file Data_Sheet_10.ZIP › GSM2230754.plot/Lsa-miR156j_Lsat_1_v5_gn_4_7021.1_627_TPlot.pdf]

**T=Lsat\_1\_v5\_gn\_6\_84121.1\_Q=Lsa-miR156j\_S=687**

category=2\_p=0.996214775614472

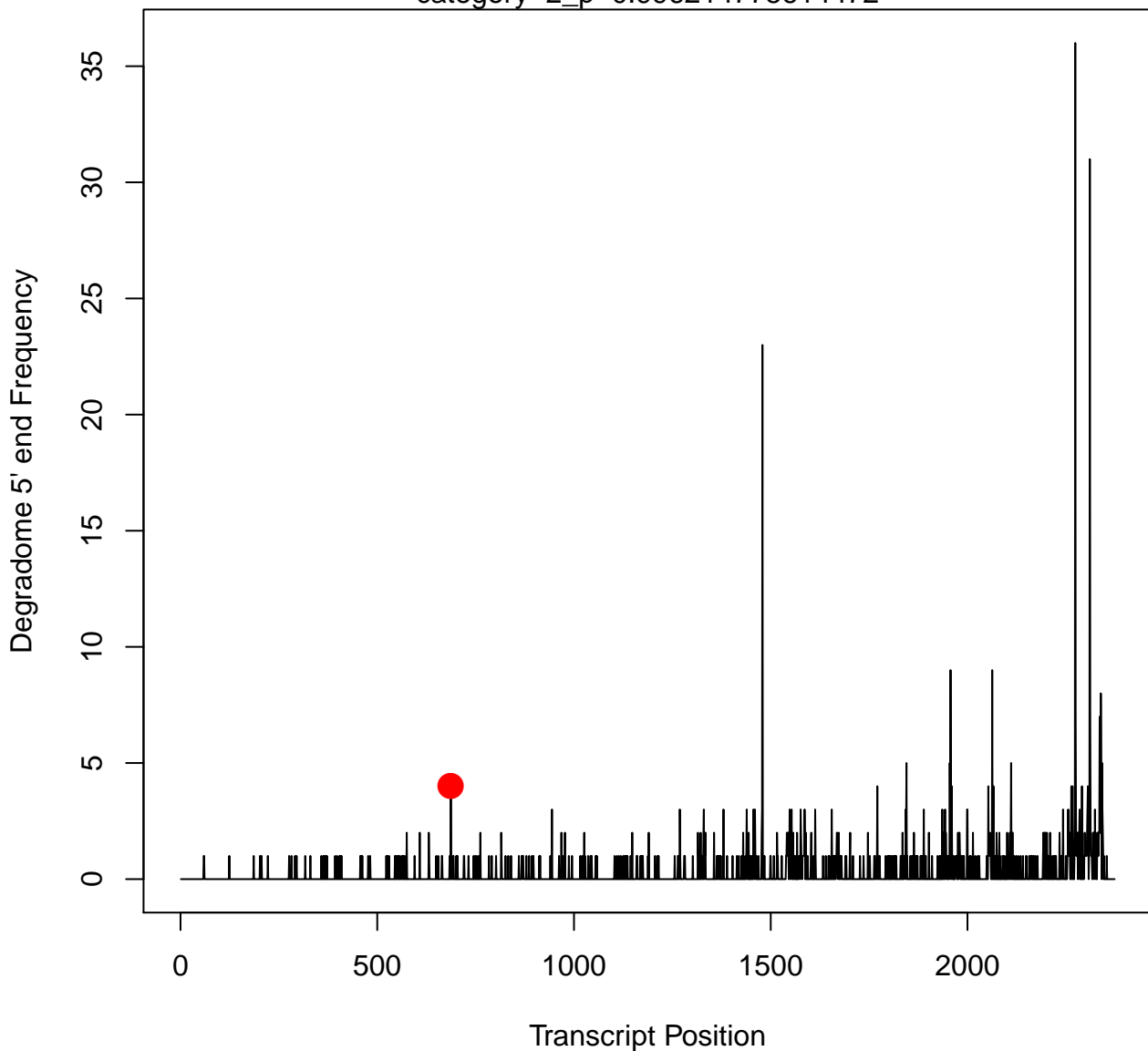

Supplement: Supplementary Data 1 — Results of categories 0–2 from PARE-Seq analysis (including three subfiles:1_1, 1_2, 1_3). [file Data_Sheet_10.ZIP › GSM2230754.plot/Lsa-miR156j_Lsat_1_v5_gn_6_84121.1_687_TPlot.pdf]

**T=Lsat\_1\_v5\_gn\_7\_49861.1\_Q=Lsa-miR156j\_S=1953**

category=2\_p=0.943609817991357

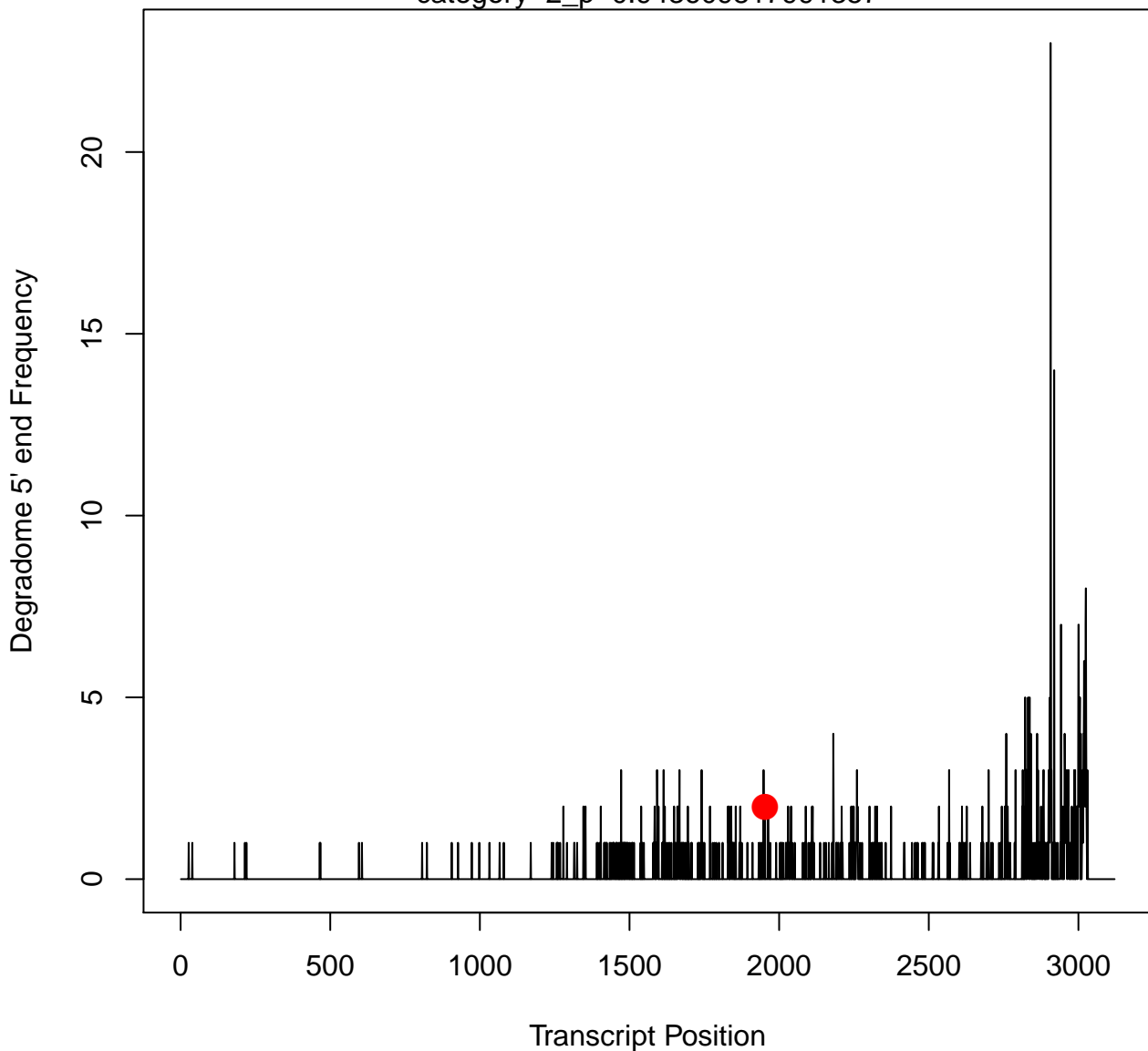

Supplement: Supplementary Data 1 — Results of categories 0–2 from PARE-Seq analysis (including three subfiles:1_1, 1_2, 1_3). [file Data_Sheet_10.ZIP › GSM2230754.plot/Lsa-miR156j_Lsat_1_v5_gn_7_49861.1_1953_TPlot.pdf]

**T=Lsat\_1\_v5\_gn\_1\_128400.1\_Q=Lsa-miR157a\_S=525**

category=0\_p=0.00337696696487544

Degradome 5' end Frequency

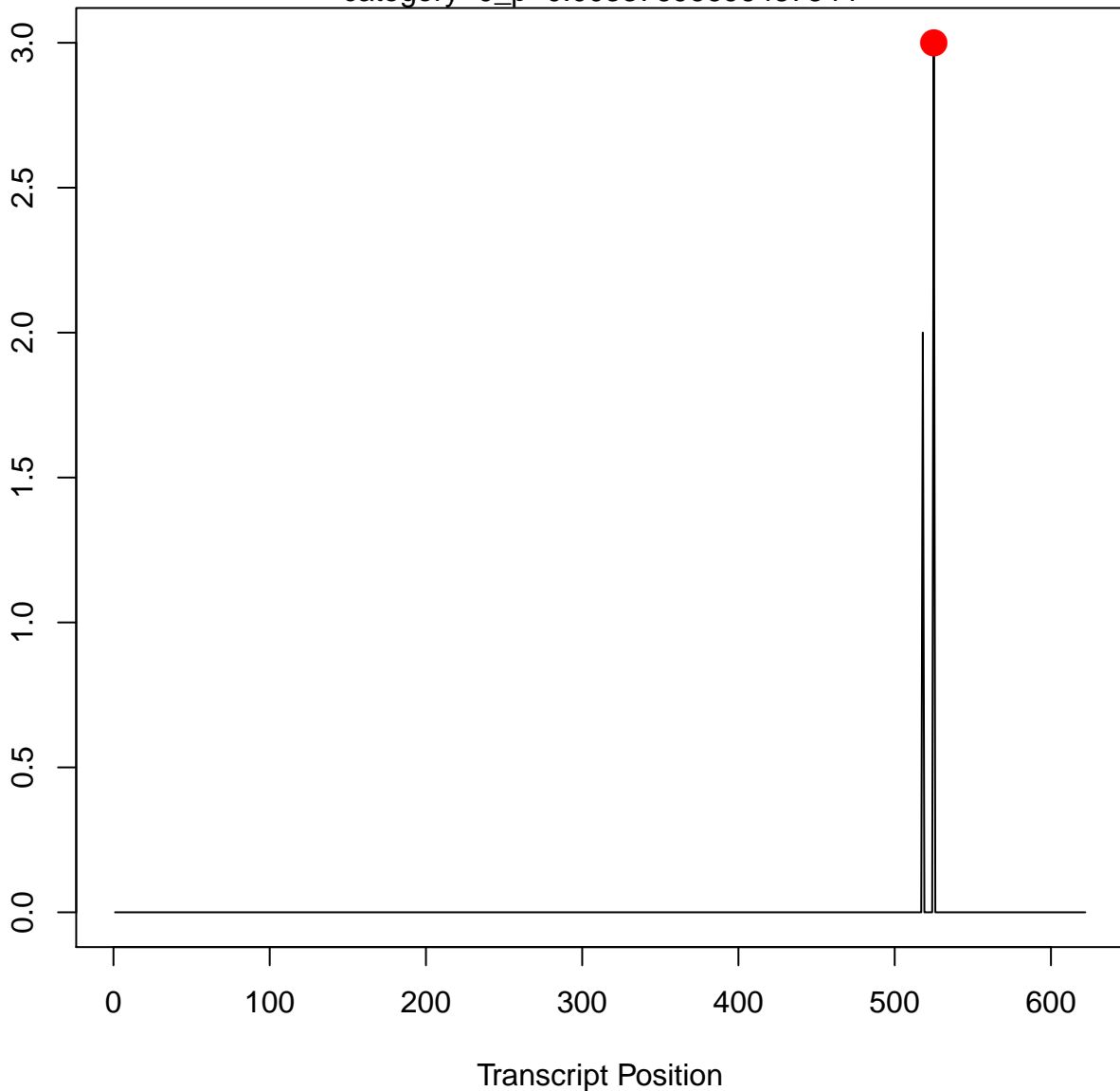

Supplement: Supplementary Data 1 — Results of categories 0–2 from PARE-Seq analysis (including three subfiles:1_1, 1_2, 1_3). [file Data_Sheet_10.ZIP › GSM2230754.plot/Lsa-miR157a_Lsat_1_v5_gn_1_128400.1_525_TPlot.pdf]

**T=Lsat\_1\_v5\_gn\_4\_1141.1\_Q=Lsa-miR157a\_S=1409**

category=0\_p=0.00150228456680068

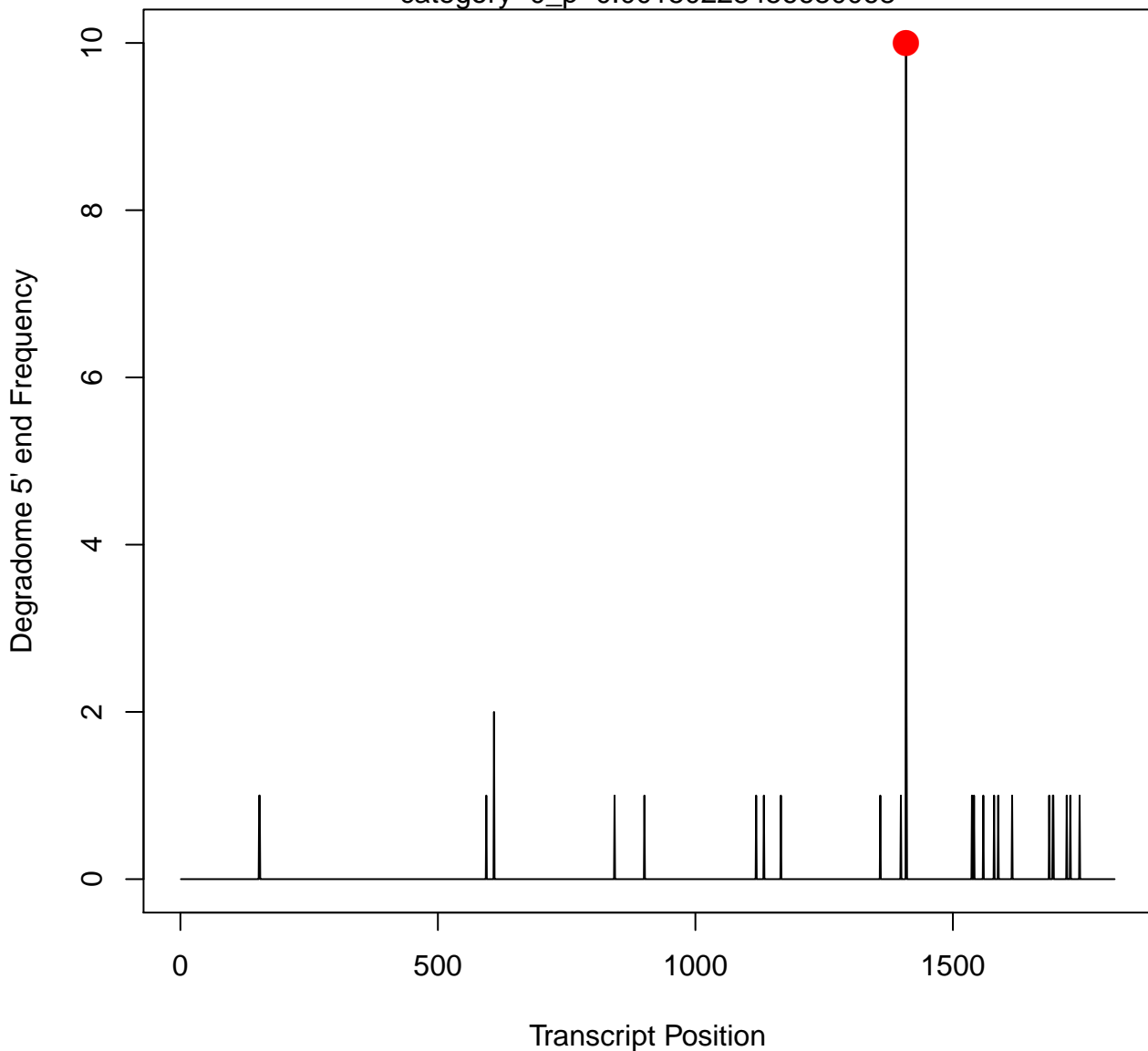

Supplement: Supplementary Data 1 — Results of categories 0–2 from PARE-Seq analysis (including three subfiles:1_1, 1_2, 1_3). [file Data_Sheet_10.ZIP › GSM2230754.plot/Lsa-miR157a_Lsat_1_v5_gn_4_1141.1_1409_TPlot.pdf]

**T=Lsat\_1\_v5\_gn\_4\_421.1\_Q=Lsa-miR157a\_S=2360**

category=0\_p=0.00037578290783058

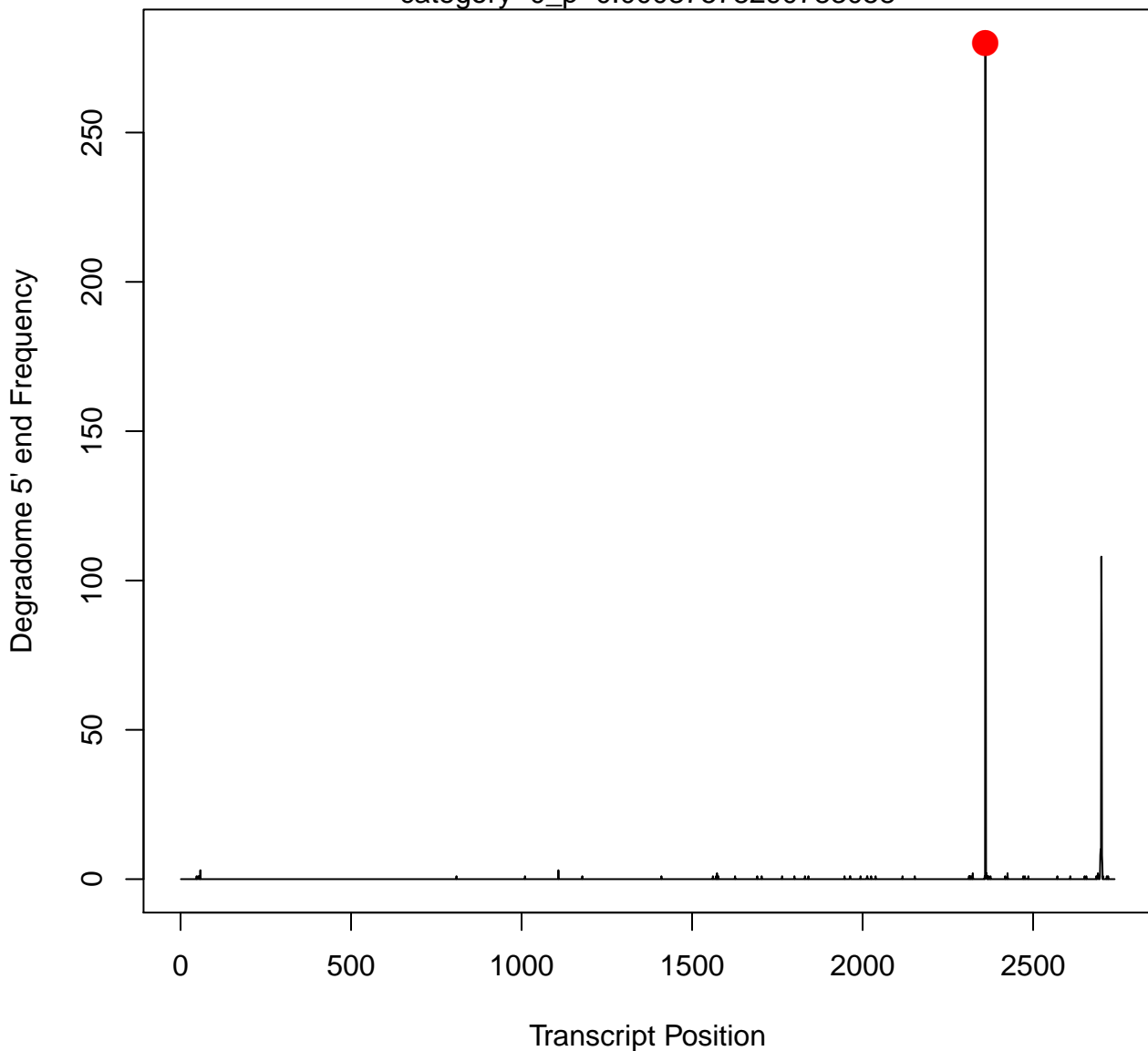

Supplement: Supplementary Data 1 — Results of categories 0–2 from PARE-Seq analysis (including three subfiles:1_1, 1_2, 1_3). [file Data_Sheet_10.ZIP › GSM2230754.plot/Lsa-miR157a_Lsat_1_v5_gn_4_421.1_2360_TPlot.pdf]

**T=Lsat\_1\_v5\_gn\_5\_142161.1\_Q=Lsa-miR157a\_S=3504**

category=2\_p=0.848614909898006

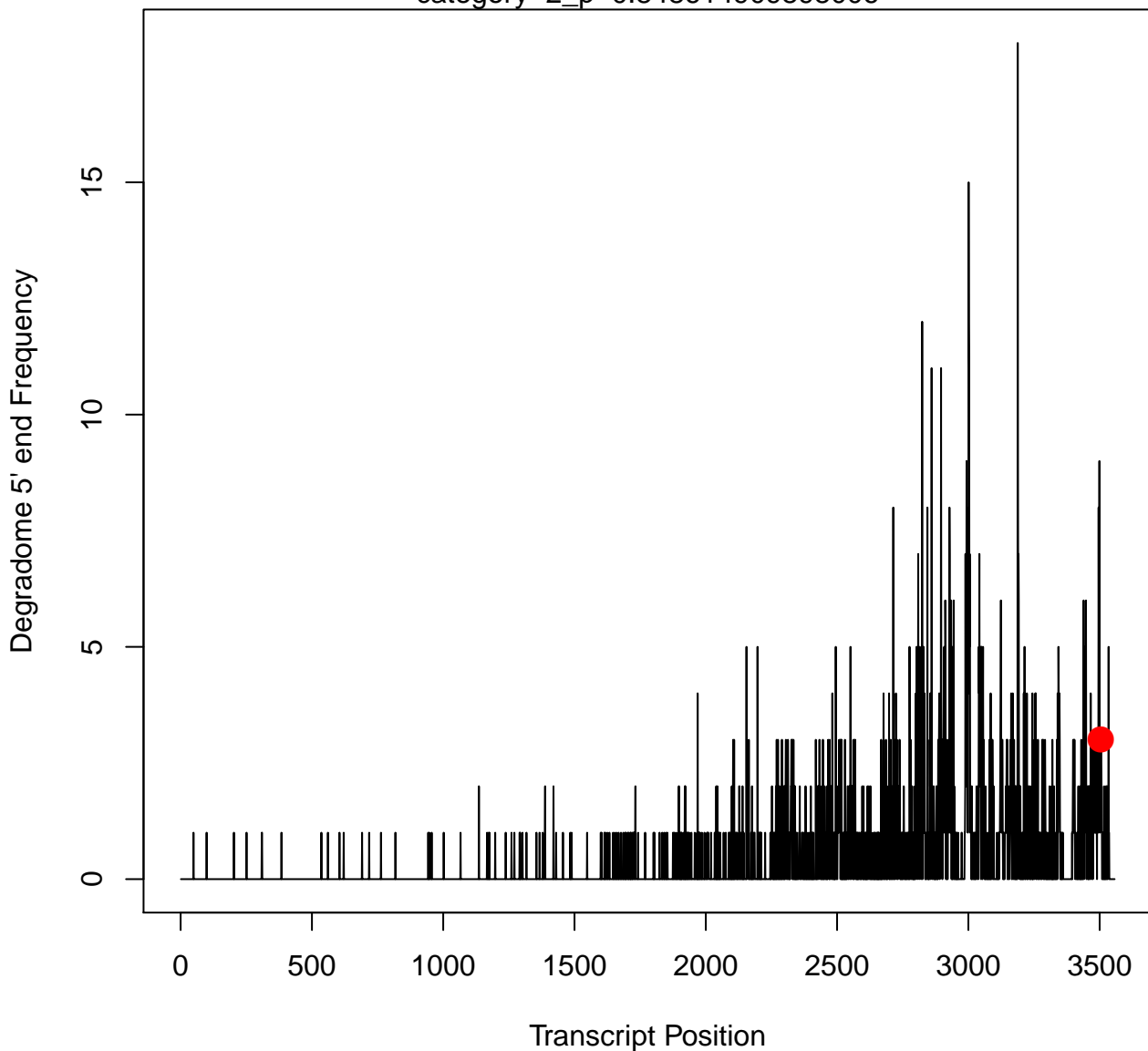

Supplement: Supplementary Data 1 — Results of categories 0–2 from PARE-Seq analysis (including three subfiles:1_1, 1_2, 1_3). [file Data_Sheet_10.ZIP › GSM2230754.plot/Lsa-miR157a_Lsat_1_v5_gn_5_142161.1_3504_TPlot.pdf]

**T=Lsat\_1\_v5\_gn\_9\_4540.1\_Q=Lsa-miR157a\_S=325**

category=2\_p=0.751959323408816

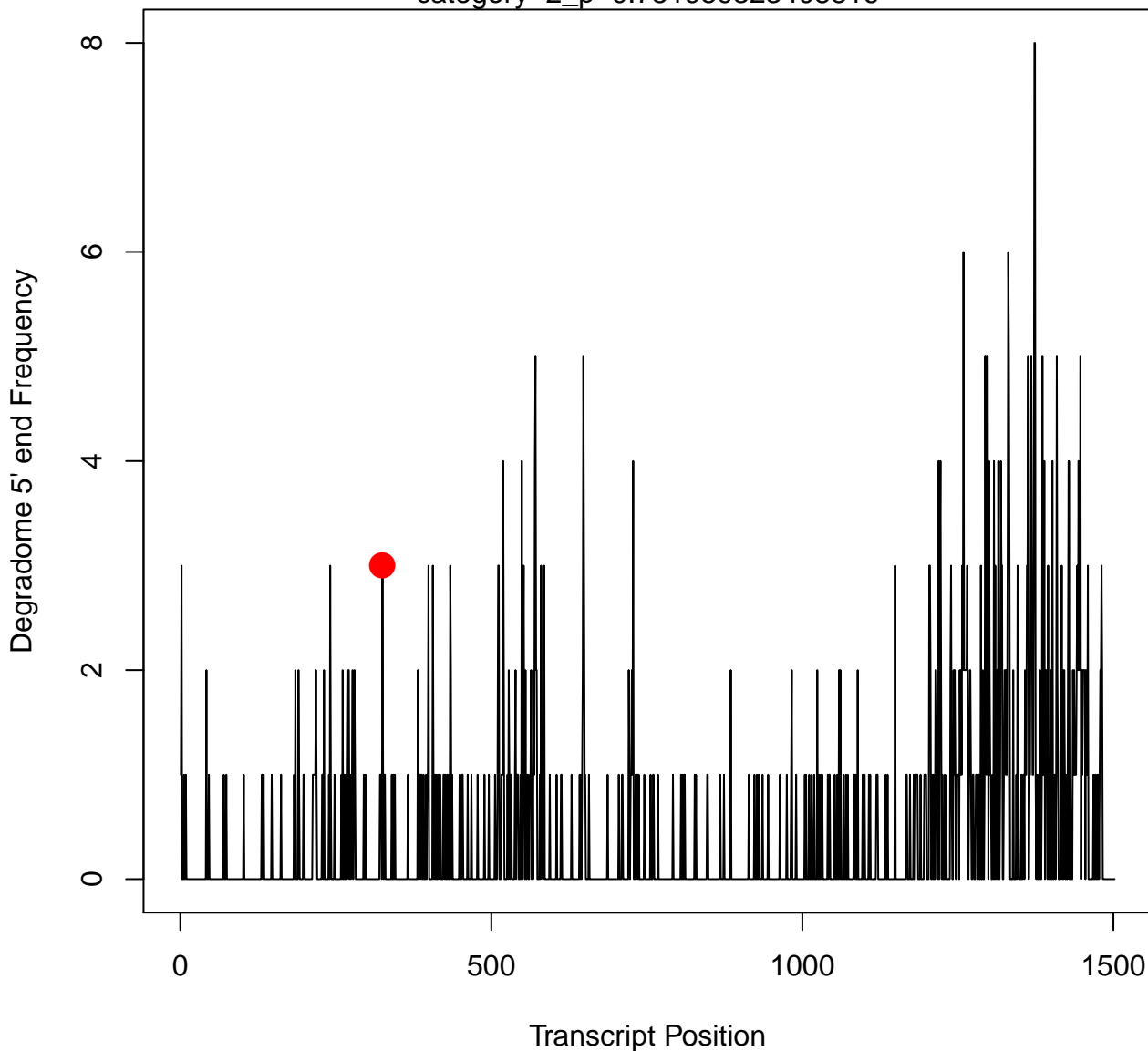

Supplement: Supplementary Data 1 — Results of categories 0–2 from PARE-Seq analysis (including three subfiles:1_1, 1_2, 1_3). [file Data_Sheet_10.ZIP › GSM2230754.plot/Lsa-miR157a_Lsat_1_v5_gn_9_4540.1_325_TPlot.pdf]

**T=Lsat\_1\_v5\_gn\_5\_12360.1\_Q=Lsa-miR157b\_S=1860**

category=2\_p=0.183979146537541

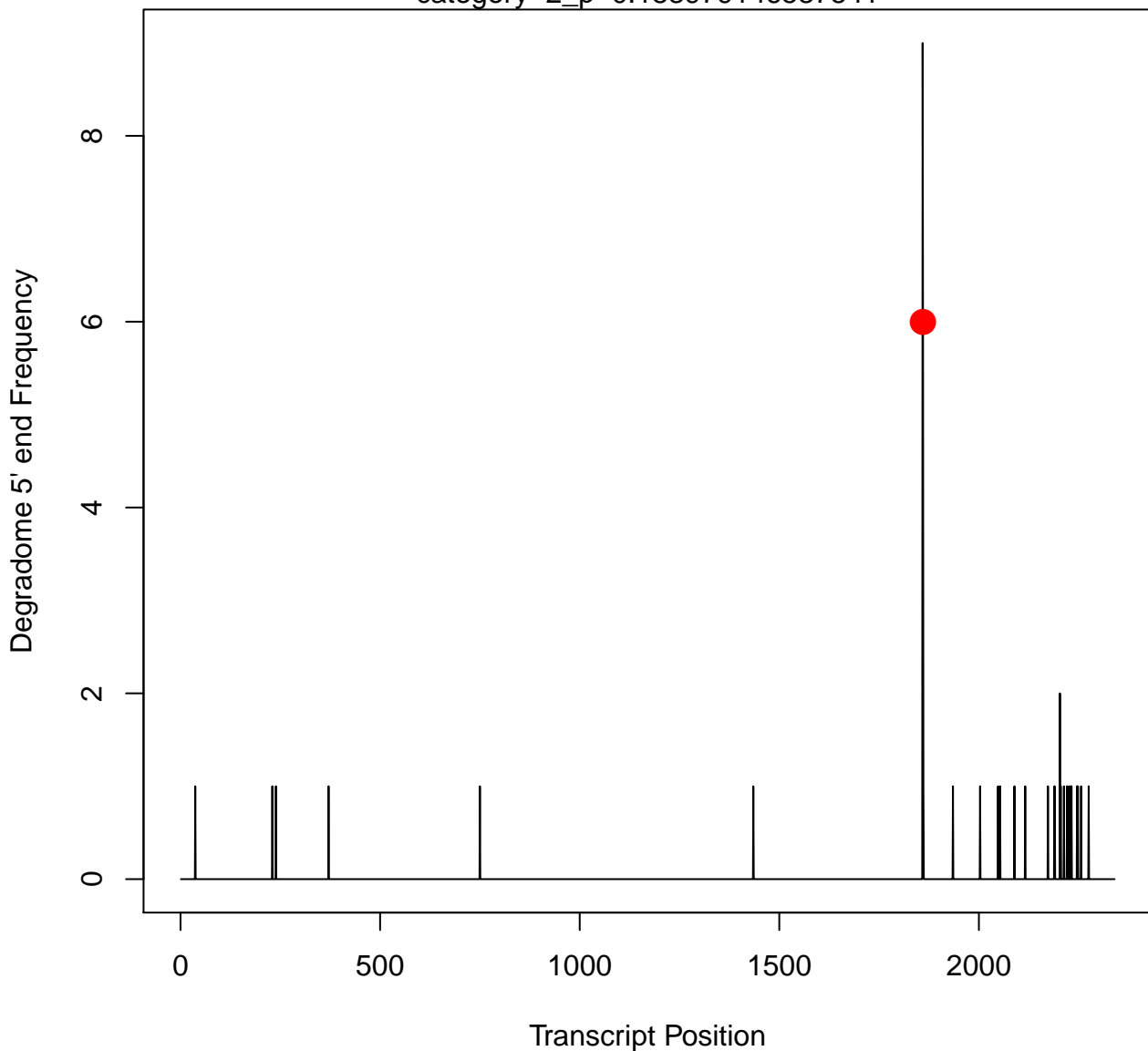

Supplement: Supplementary Data 1 — Results of categories 0–2 from PARE-Seq analysis (including three subfiles:1_1, 1_2, 1_3). [file Data_Sheet_10.ZIP › GSM2230754.plot/Lsa-miR157b_Lsat_1_v5_gn_5_12360.1_1860_TPlot.pdf]

**T=Lsat\_1\_v5\_gn\_5\_142161.1\_Q=Lsa-miR157b\_S=3505**

category=2\_p=0.880003036932643

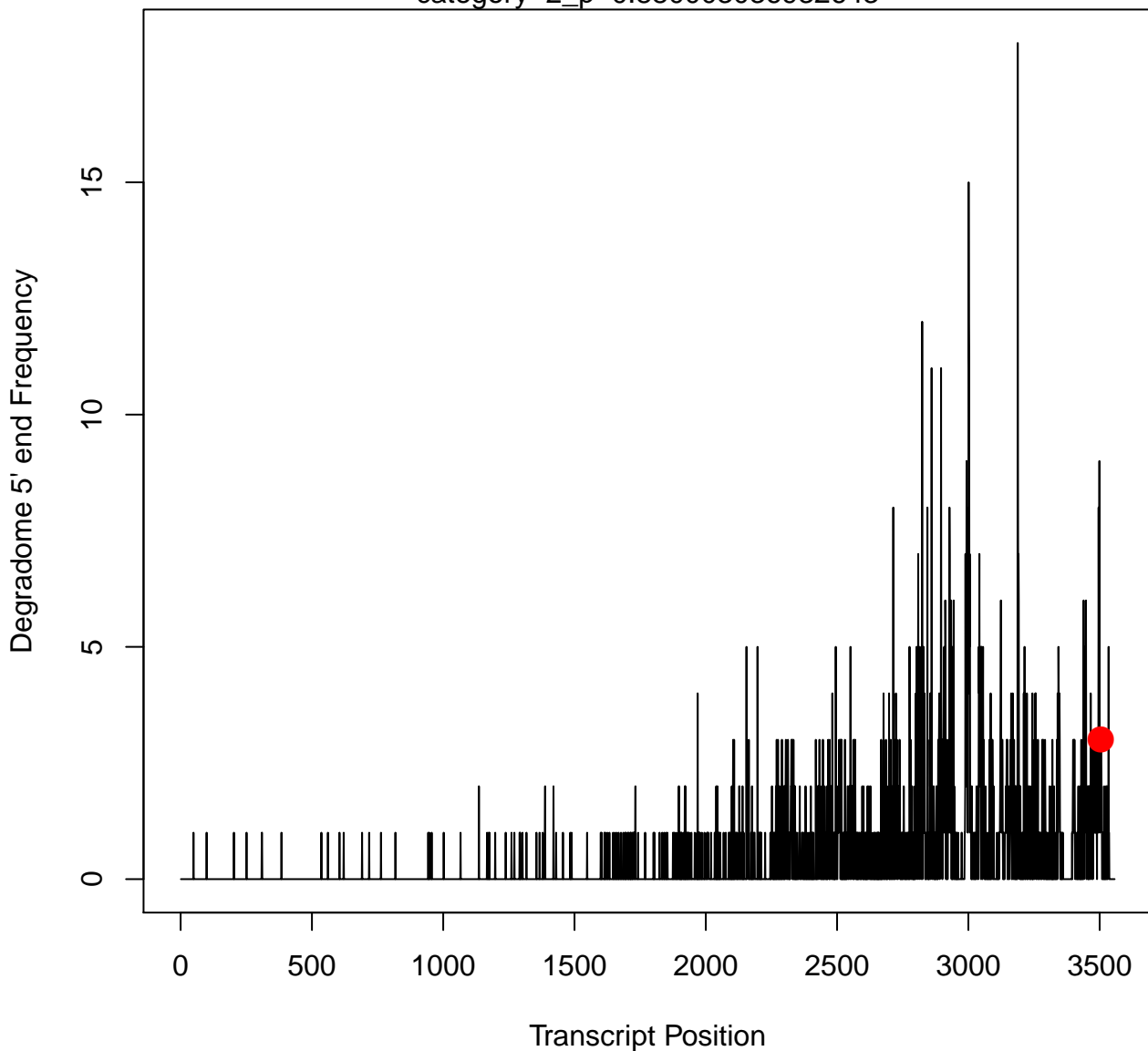

Supplement: Supplementary Data 1 — Results of categories 0–2 from PARE-Seq analysis (including three subfiles:1_1, 1_2, 1_3). [file Data_Sheet_10.ZIP › GSM2230754.plot/Lsa-miR157b_Lsat_1_v5_gn_5_142161.1_3505_TPlot.pdf]

**T=Lsat\_1\_v5\_gn\_7\_11040.1\_Q=Lsa-miR157b\_S=1088**

category=2\_p=0.135172542726729

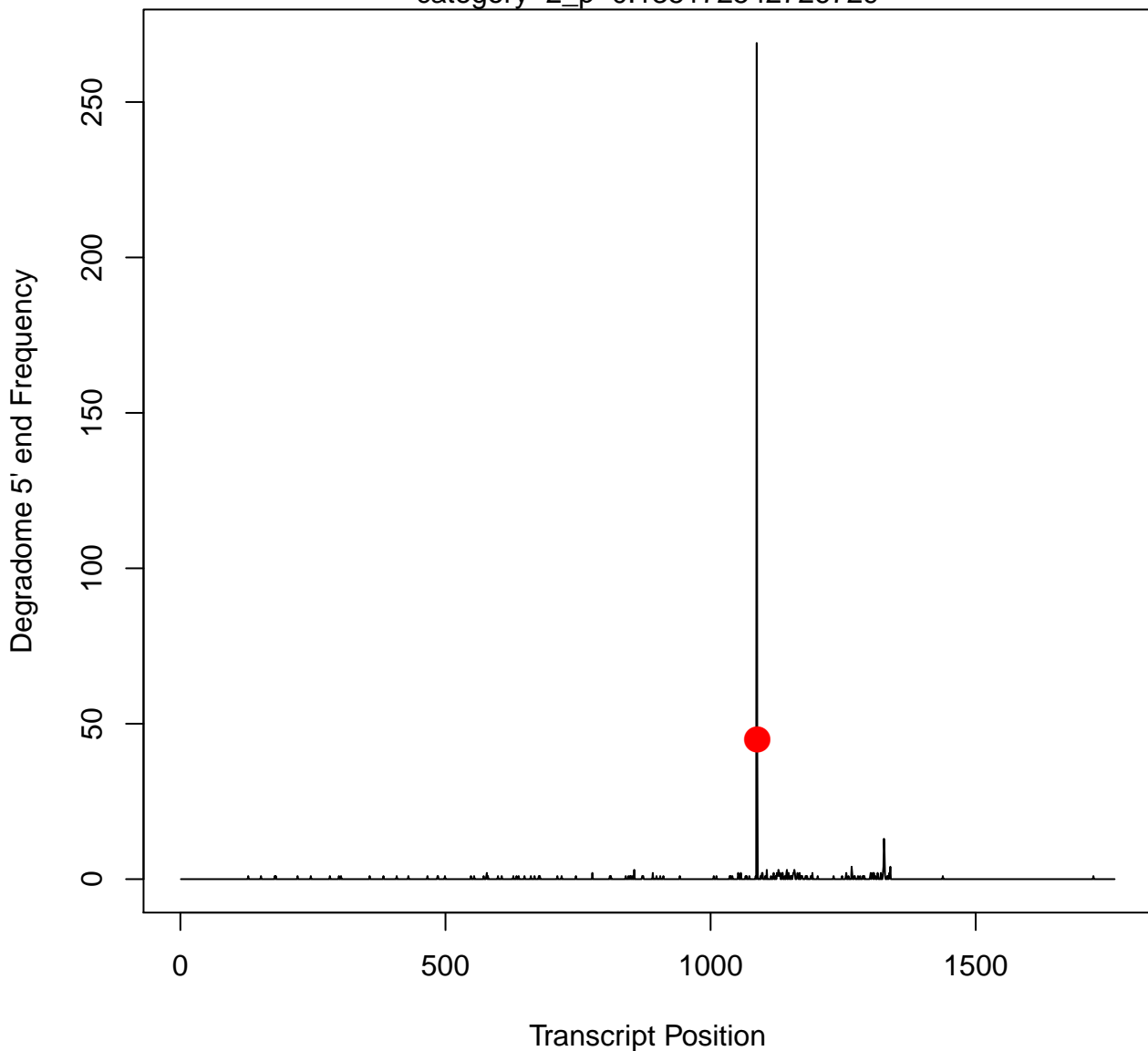

Supplement: Supplementary Data 1 — Results of categories 0–2 from PARE-Seq analysis (including three subfiles:1_1, 1_2, 1_3). [file Data_Sheet_10.ZIP › GSM2230754.plot/Lsa-miR157b_Lsat_1_v5_gn_7_11040.1_1088_TPlot.pdf]

**T=Lsat\_1\_v5\_gn\_9\_28021.1\_Q=Lsa-miR157b\_S=1268**

category=0\_p=0.00150228456680068

Degradome 5' end Frequency

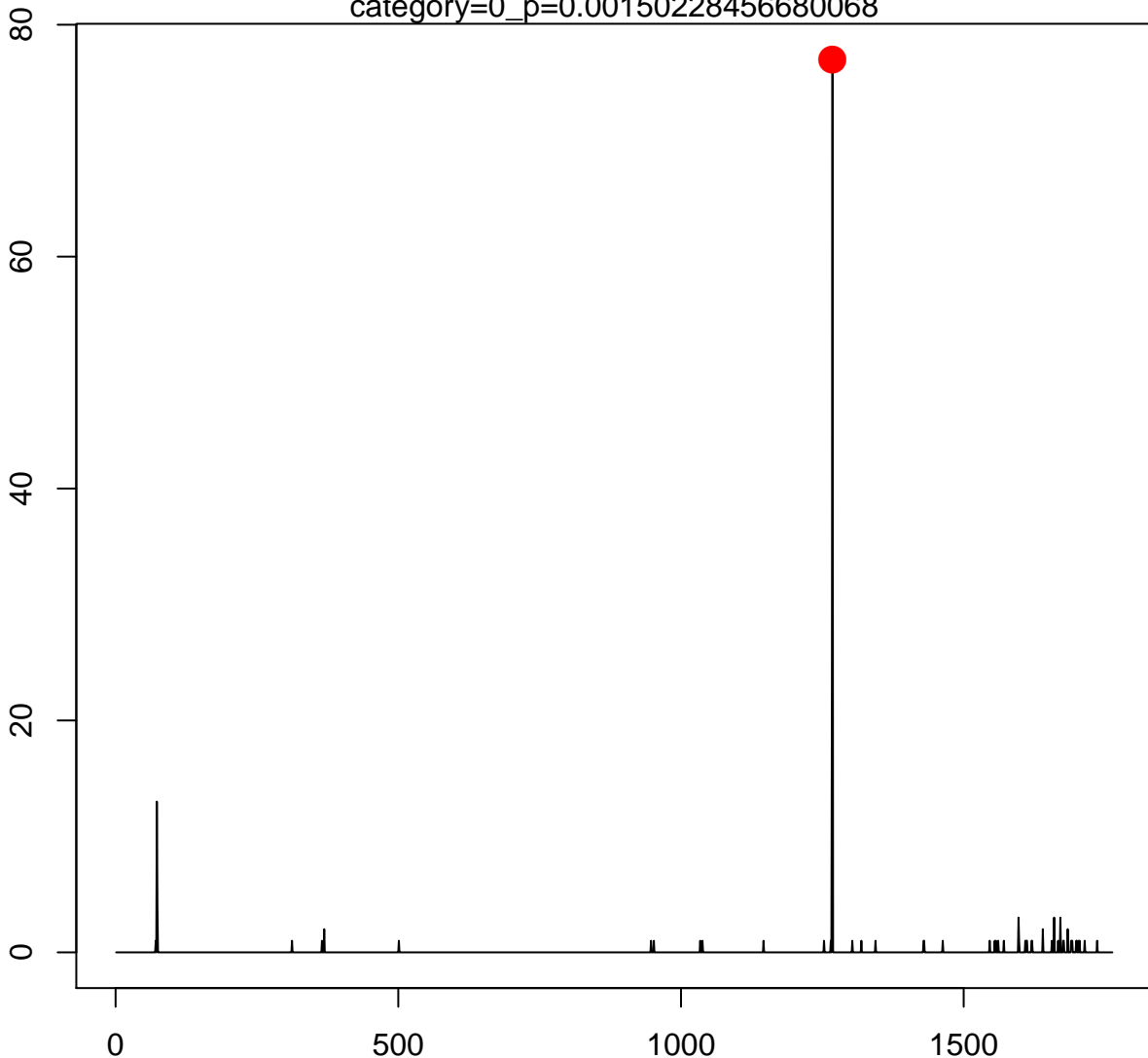

Transcript Position

Supplement: Supplementary Data 1 — Results of categories 0–2 from PARE-Seq analysis (including three subfiles:1_1, 1_2, 1_3). [file Data_Sheet_10.ZIP › GSM2230754.plot/Lsa-miR157b_Lsat_1_v5_gn_9_28021.1_1268_TPlot.pdf]

**T=Lsat\_1\_v5\_gn\_4\_1141.1\_Q=Lsa-miR157d\_S=1410**

category=2\_p=0.135172542726729

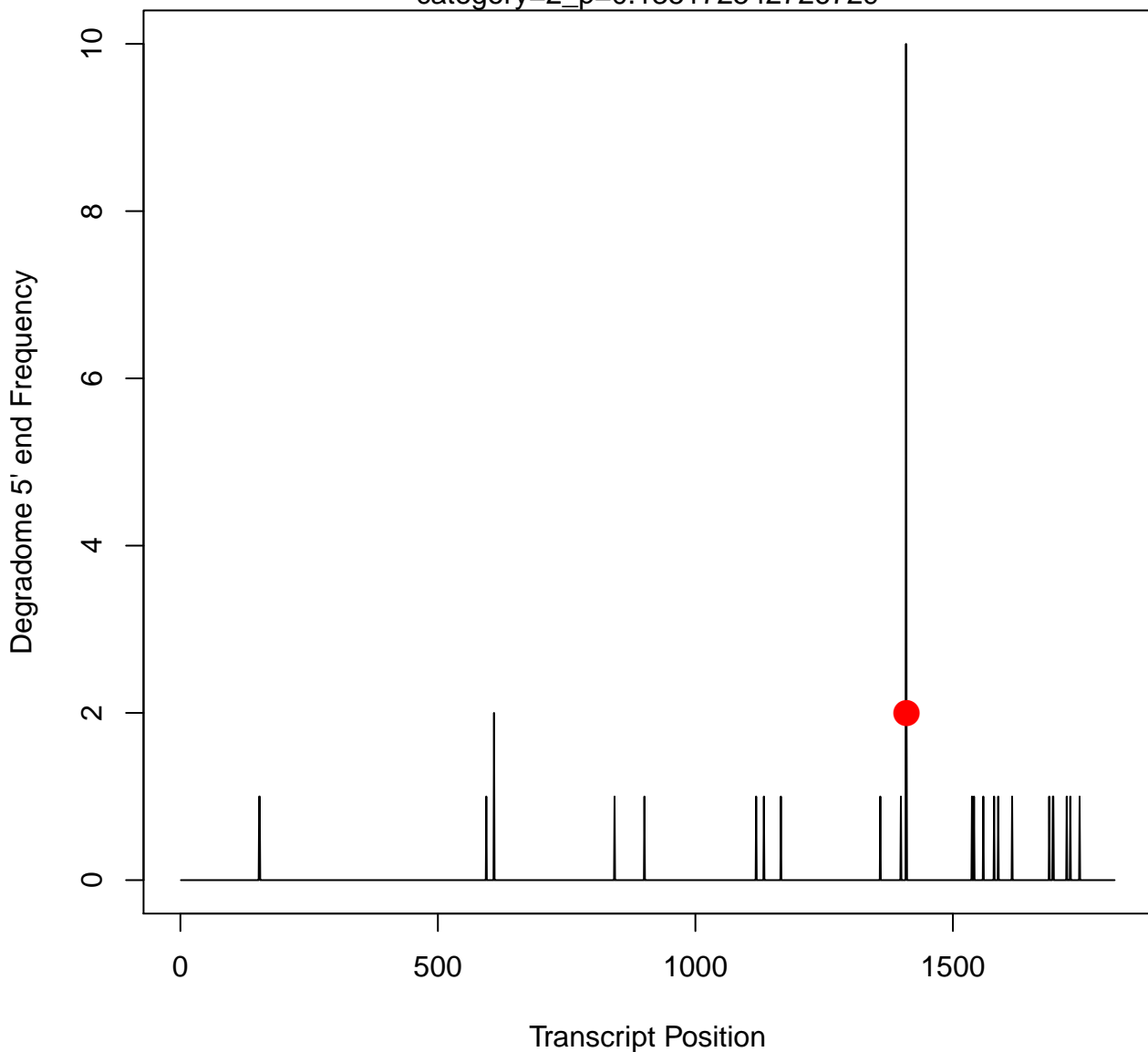

Supplement: Supplementary Data 1 — Results of categories 0–2 from PARE-Seq analysis (including three subfiles:1_1, 1_2, 1_3). [file Data_Sheet_10.ZIP › GSM2230754.plot/Lsa-miR157d_Lsat_1_v5_gn_4_1141.1_1410_TPlot.pdf]

**T=Lsat\_1\_v5\_gn\_4\_421.1\_Q=Lsa-miR157d\_S=2361**

category=2\_p=0.0286272994447346

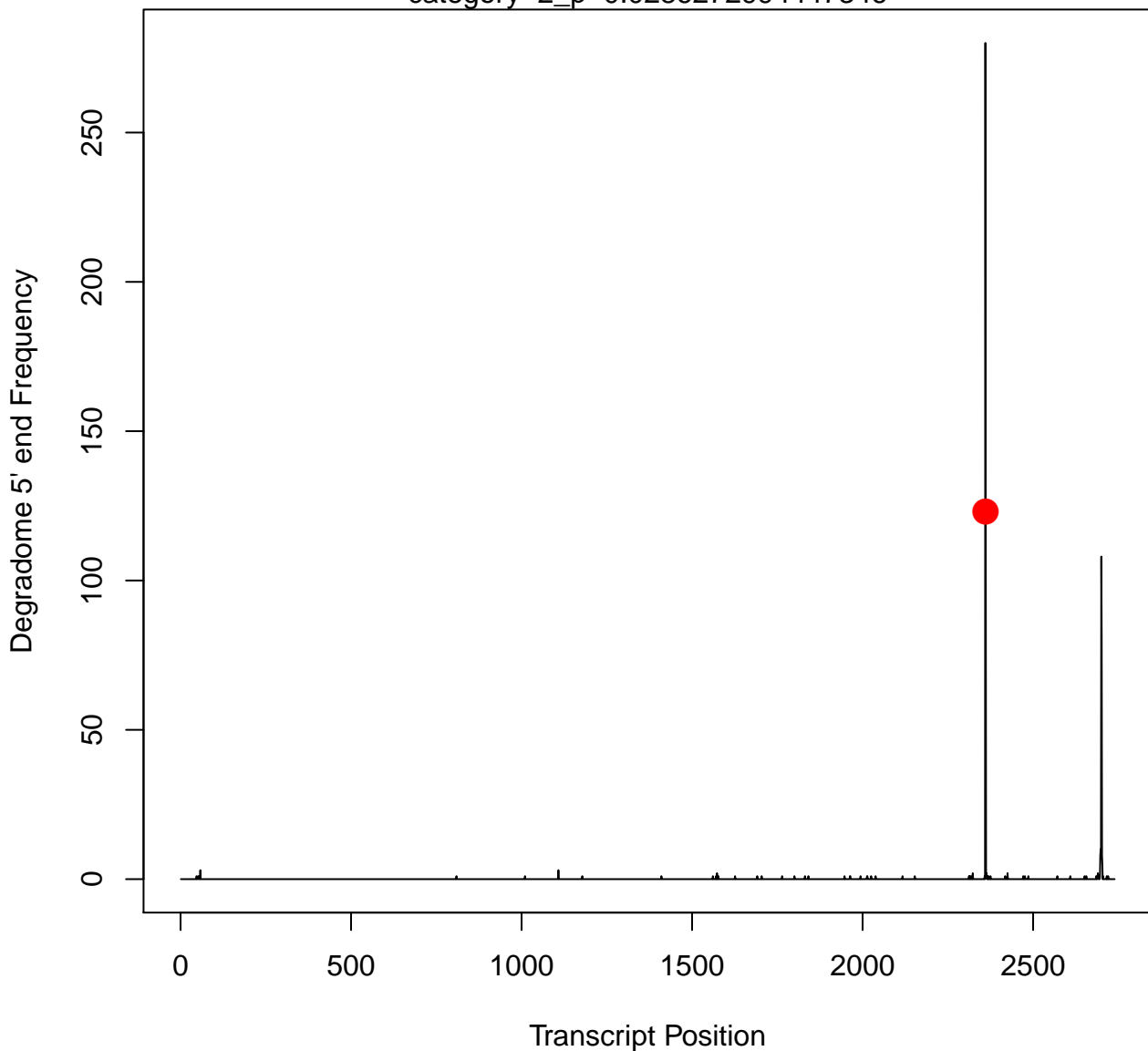

Supplement: Supplementary Data 1 — Results of categories 0–2 from PARE-Seq analysis (including three subfiles:1_1, 1_2, 1_3). [file Data_Sheet_10.ZIP › GSM2230754.plot/Lsa-miR157d_Lsat_1_v5_gn_4_421.1_2361_TPlot.pdf]

**T=Lsat\_1\_v5\_gn\_2\_134361.1\_Q=Lsa-miR157e\_S=223**

category=2\_p=0.605225942934683

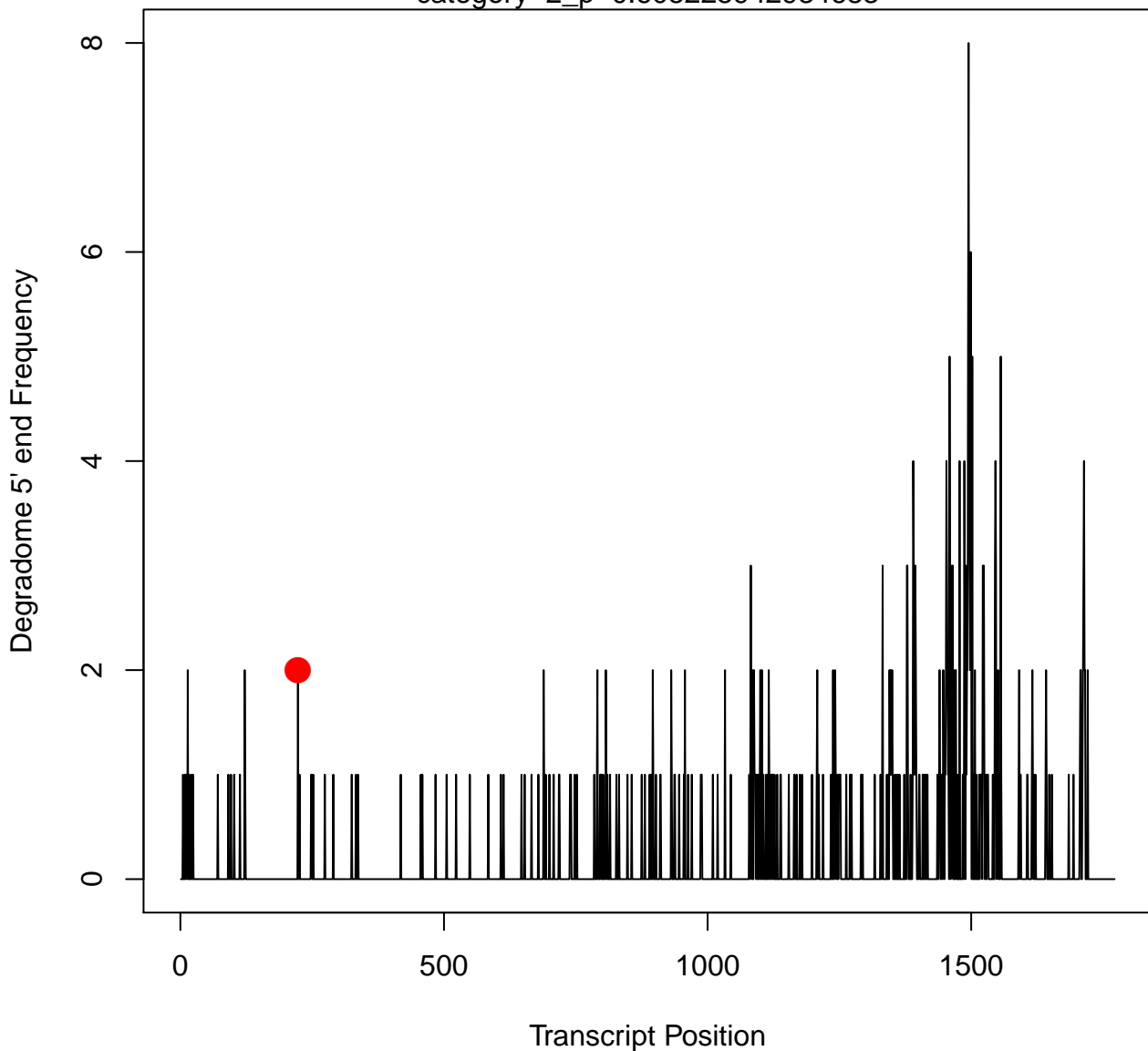

Supplement: Supplementary Data 1 — Results of categories 0–2 from PARE-Seq analysis (including three subfiles:1_1, 1_2, 1_3). [file Data_Sheet_10.ZIP › GSM2230754.plot/Lsa-miR157e_Lsat_1_v5_gn_2_134361.1_223_TPlot.pdf]

**T=Lsat\_1\_v5\_gn\_8\_134640.1\_Q=Lsa-miR157e\_S=547**

category=0\_p=0.00300231227468162

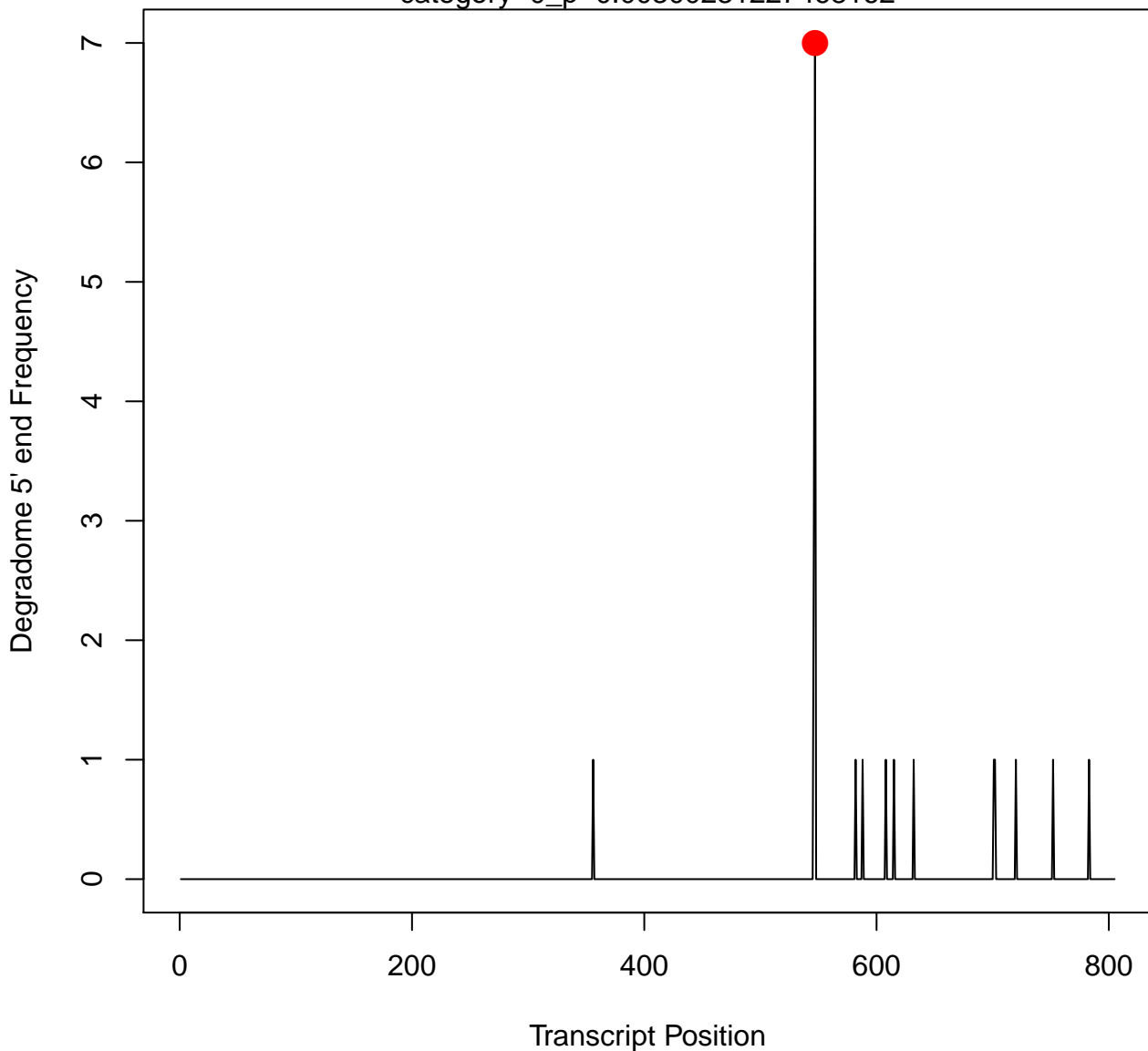

Supplement: Supplementary Data 1 — Results of categories 0–2 from PARE-Seq analysis (including three subfiles:1_1, 1_2, 1_3). [file Data_Sheet_10.ZIP › GSM2230754.plot/Lsa-miR157e_Lsat_1_v5_gn_8_134640.1_547_TPlot.pdf]

**T=Lsat\_1\_v5\_gn\_0\_39281.1\_Q=Lsa-miR159a\_S=2161**

category=2\_p=0.0286272994447346

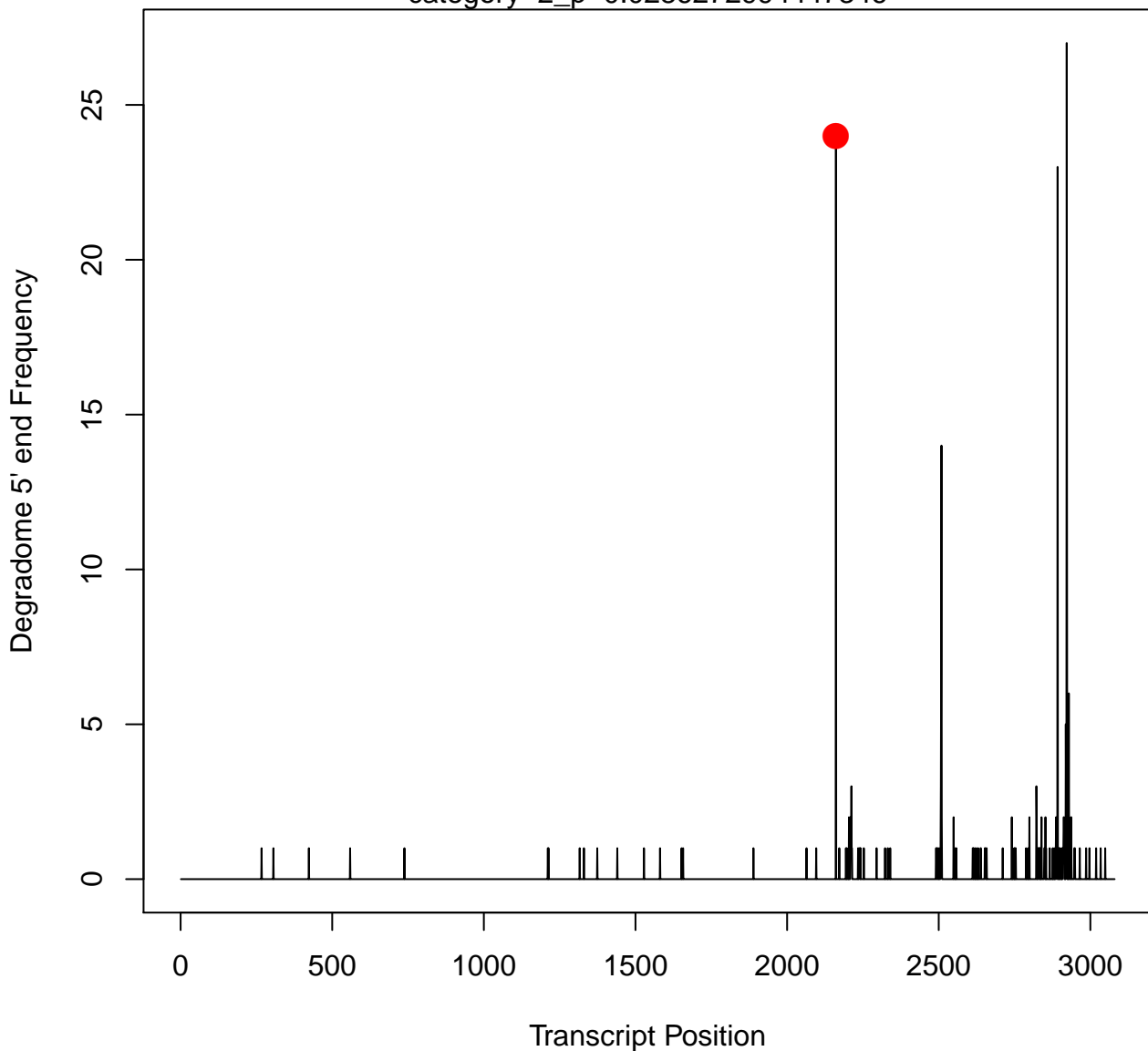

Supplement: Supplementary Data 1 — Results of categories 0–2 from PARE-Seq analysis (including three subfiles:1_1, 1_2, 1_3). [file Data_Sheet_10.ZIP › GSM2230754.plot/Lsa-miR159a_Lsat_1_v5_gn_0_39281.1_2161_TPlot.pdf]

T=Lsat\_1\_v5\_gn\_2\_81541.1\_Q=Lsa-miR159a\_S=727

category=2\_p=0.943609817991357

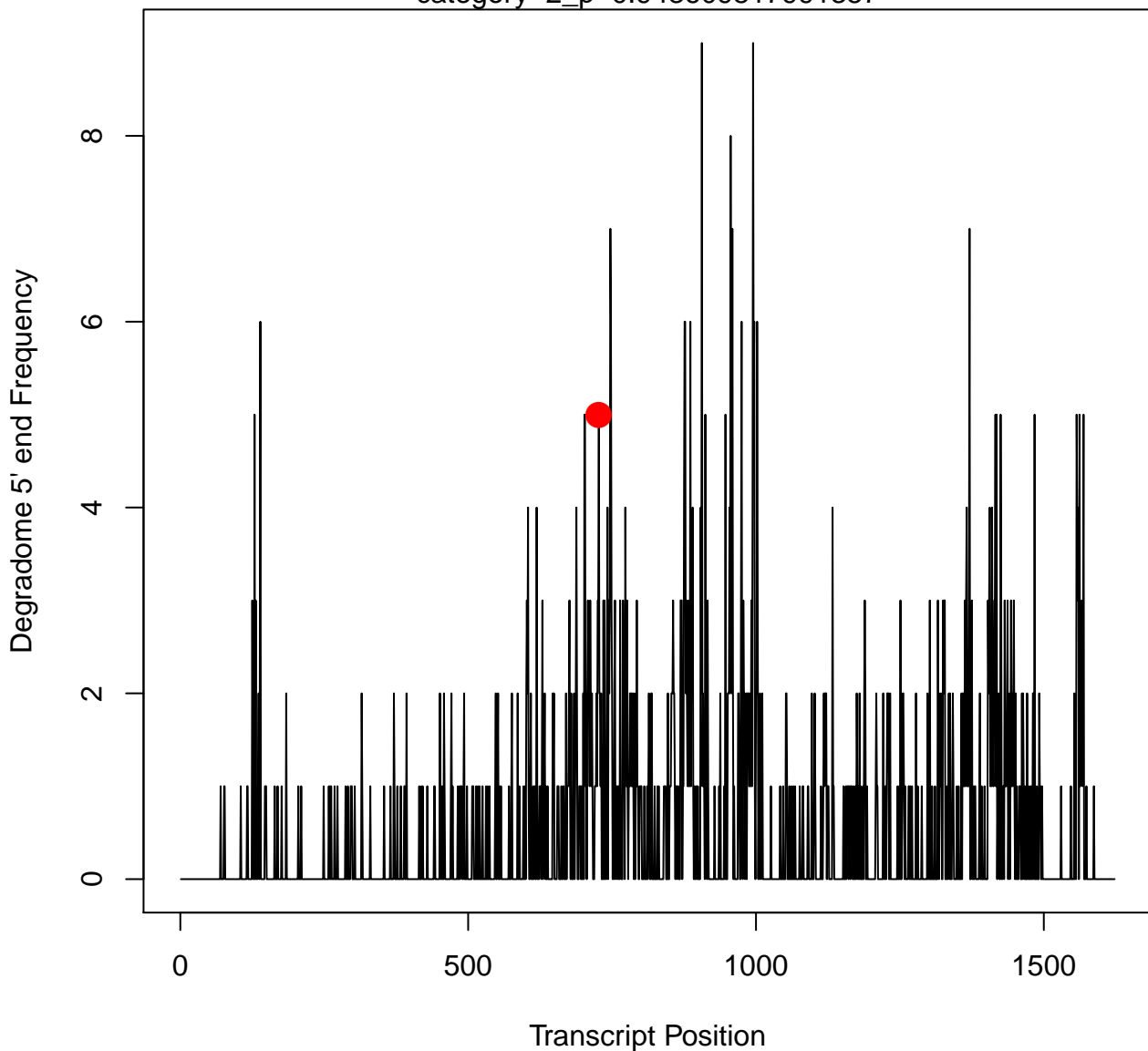

Supplement: Supplementary Data 1 — Results of categories 0–2 from PARE-Seq analysis (including three subfiles:1_1, 1_2, 1_3). [file Data_Sheet_10.ZIP › GSM2230754.plot/Lsa-miR159a_Lsat_1_v5_gn_2_81541.1_727_TPlot.pdf]

**T=Lsat\_1\_v5\_gn\_3\_18820.1\_Q=Lsa-miR159a\_S=3231**

category=2\_p=0.834832185608514

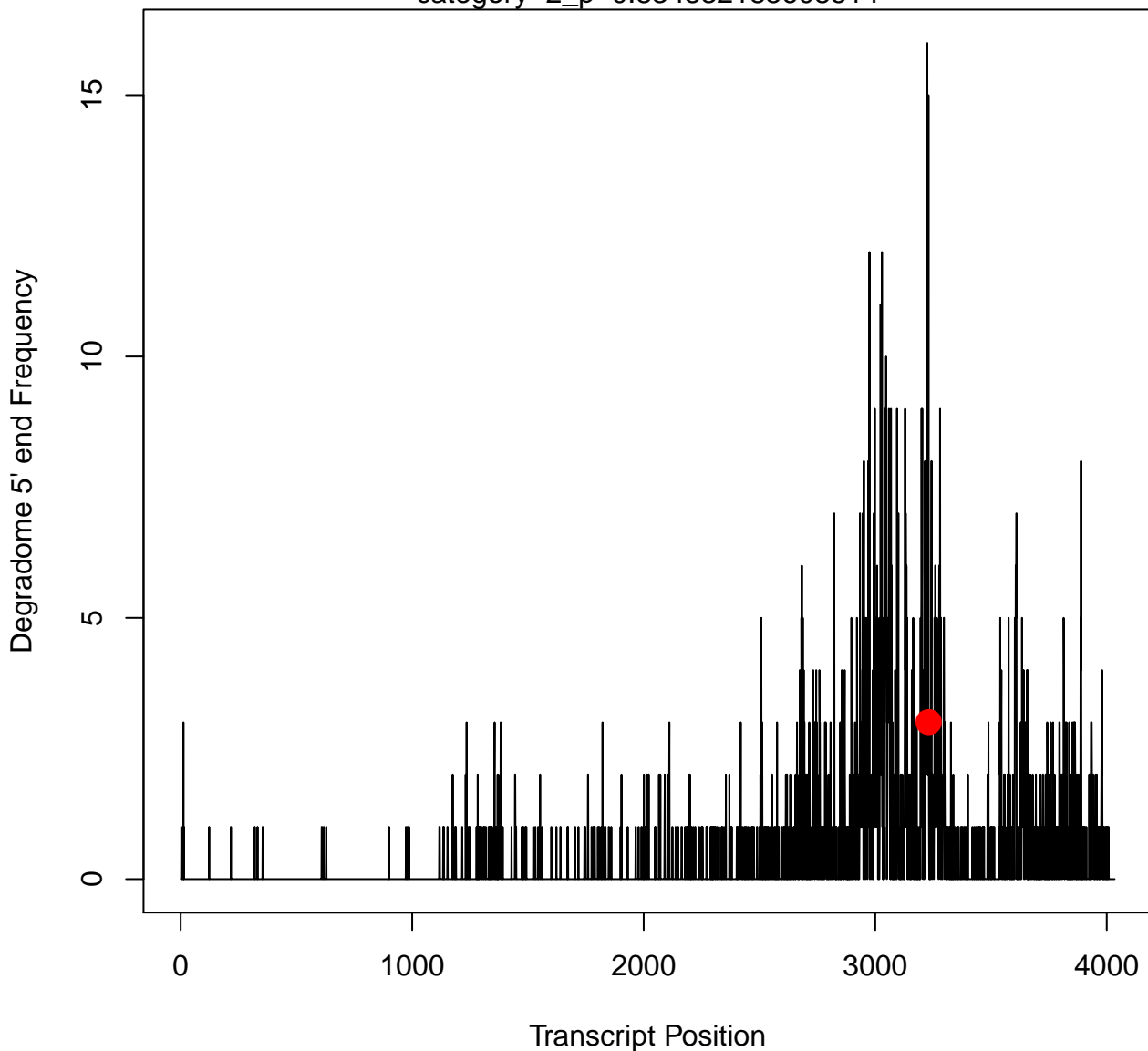

Supplement: Supplementary Data 1 — Results of categories 0–2 from PARE-Seq analysis (including three subfiles:1_1, 1_2, 1_3). [file Data_Sheet_10.ZIP › GSM2230754.plot/Lsa-miR159a_Lsat_1_v5_gn_3_18820.1_3231_TPlot.pdf]

**T=Lsat\_1\_v5\_gn\_5\_129201.1\_Q=Lsa-miR159a\_S=733**

category=2\_p=0.371689521760685

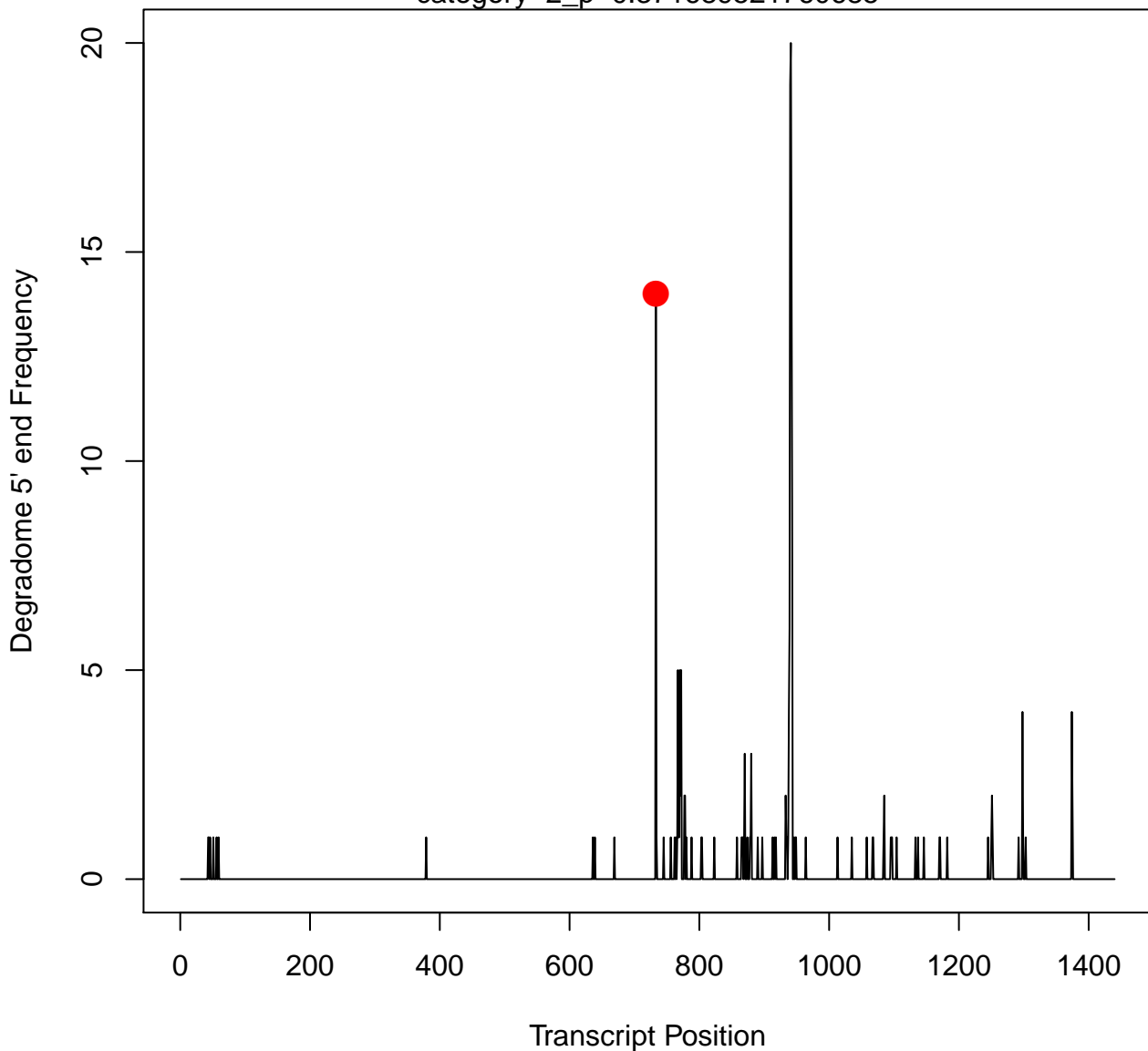

Supplement: Supplementary Data 1 — Results of categories 0–2 from PARE-Seq analysis (including three subfiles:1_1, 1_2, 1_3). [file Data_Sheet_10.ZIP › GSM2230754.plot/Lsa-miR159a_Lsat_1_v5_gn_5_129201.1_733_TPlot.pdf]

**T=Lsat\_1\_v5\_gn\_6\_87540.1\_Q=Lsa-miR159a\_S=1095**

category=2\_p=0.990952787060343

Degradsome 5' end Frequency

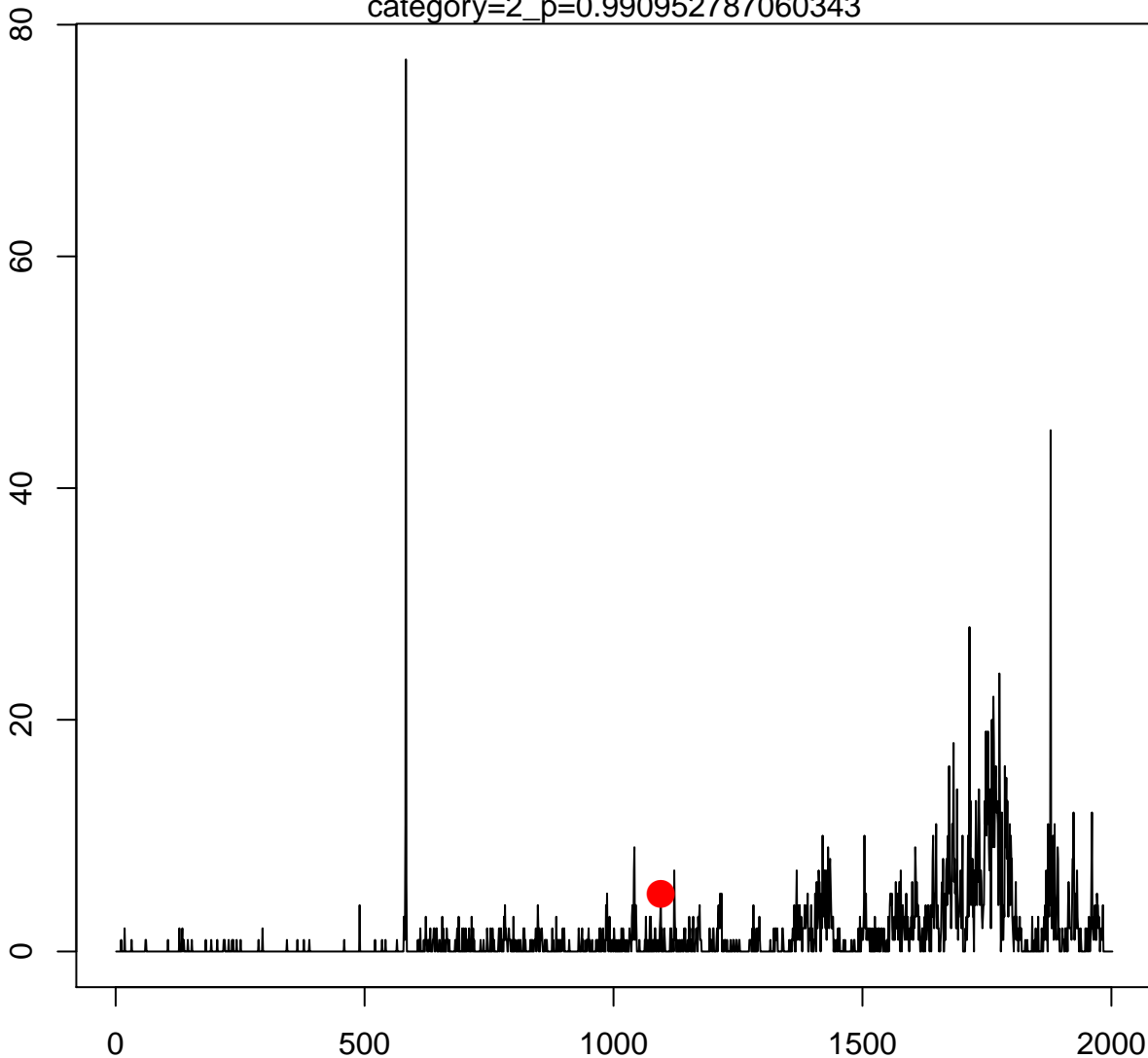

Transcript Position

Supplement: Supplementary Data 1 — Results of categories 0–2 from PARE-Seq analysis (including three subfiles:1_1, 1_2, 1_3). [file Data_Sheet_10.ZIP › GSM2230754.plot/Lsa-miR159a_Lsat_1_v5_gn_6_87540.1_1095_TPlot.pdf]

**T=Lsat\_1\_v5\_gn\_8\_123241.1\_Q=Lsa-miR159a\_S=401**

category=2\_p=0.993427060344764

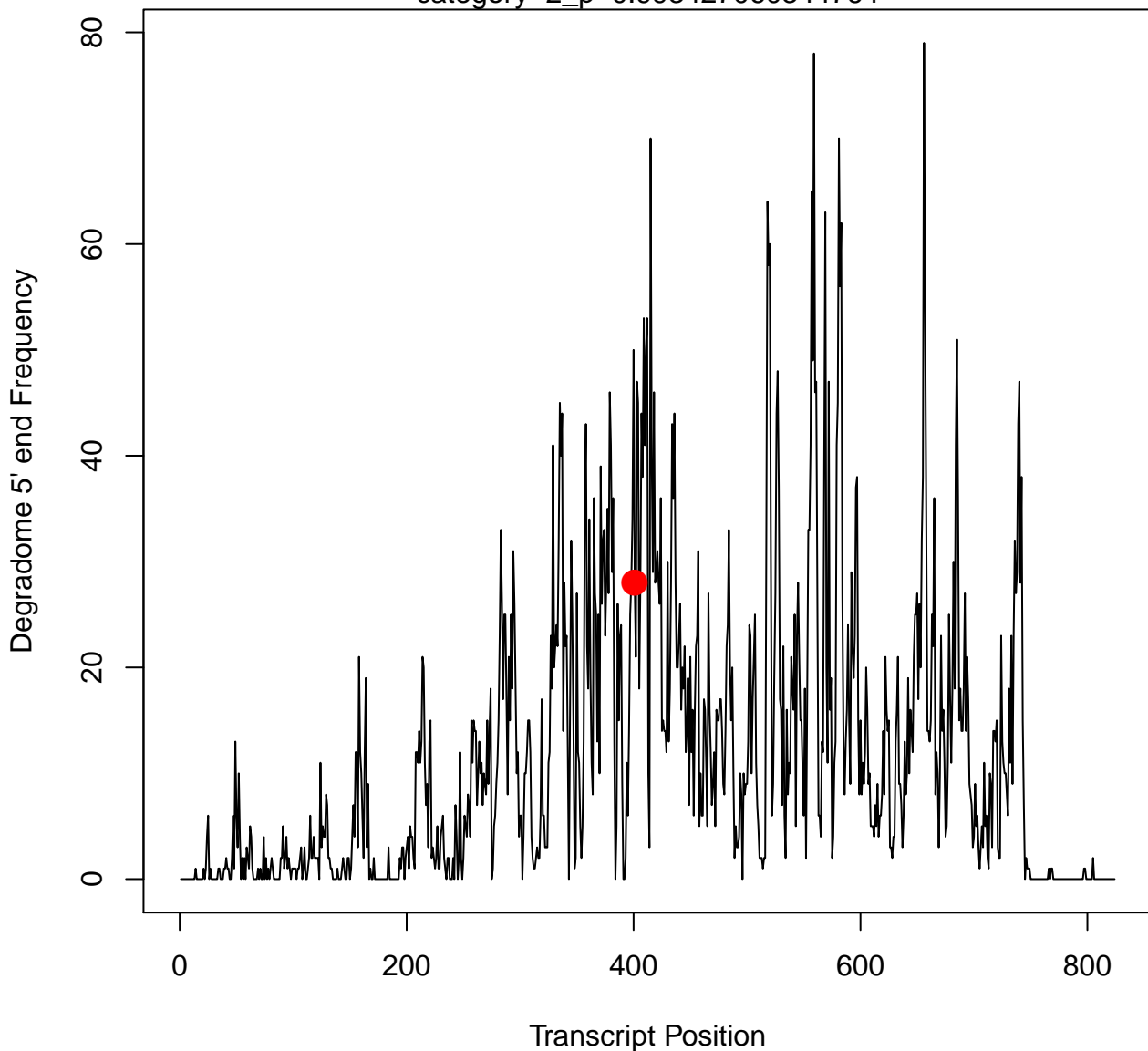

Supplement: Supplementary Data 1 — Results of categories 0–2 from PARE-Seq analysis (including three subfiles:1_1, 1_2, 1_3). [file Data_Sheet_10.ZIP › GSM2230754.plot/Lsa-miR159a_Lsat_1_v5_gn_8_123241.1_401_TPlot.pdf]

**T=Lsat\_1\_v5\_gn\_9\_65581.1\_Q=Lsa-miR159a\_S=1332**

category=2\_p=0.999971231196167

Degradome 5' end Frequency

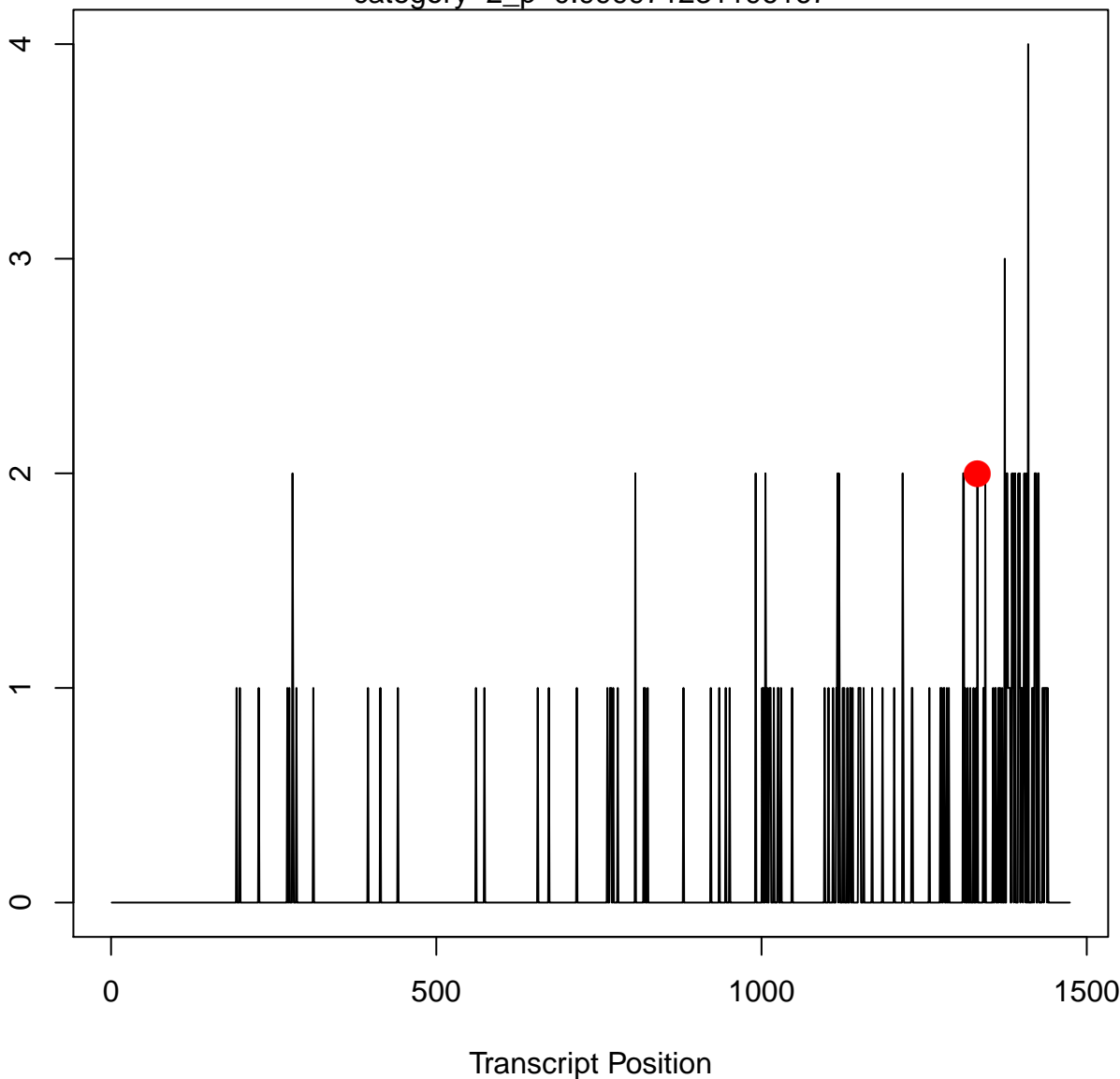

Supplement: Supplementary Data 1 — Results of categories 0–2 from PARE-Seq analysis (including three subfiles:1_1, 1_2, 1_3). [file Data_Sheet_10.ZIP › GSM2230754.plot/Lsa-miR159a_Lsat_1_v5_gn_9_65581.1_1332_TPlot.pdf]

**T=Lsat\_1\_v5\_gn\_9\_9761.1\_Q=Lsa-miR159a\_S=337**

category=2\_p=0.998168786519074

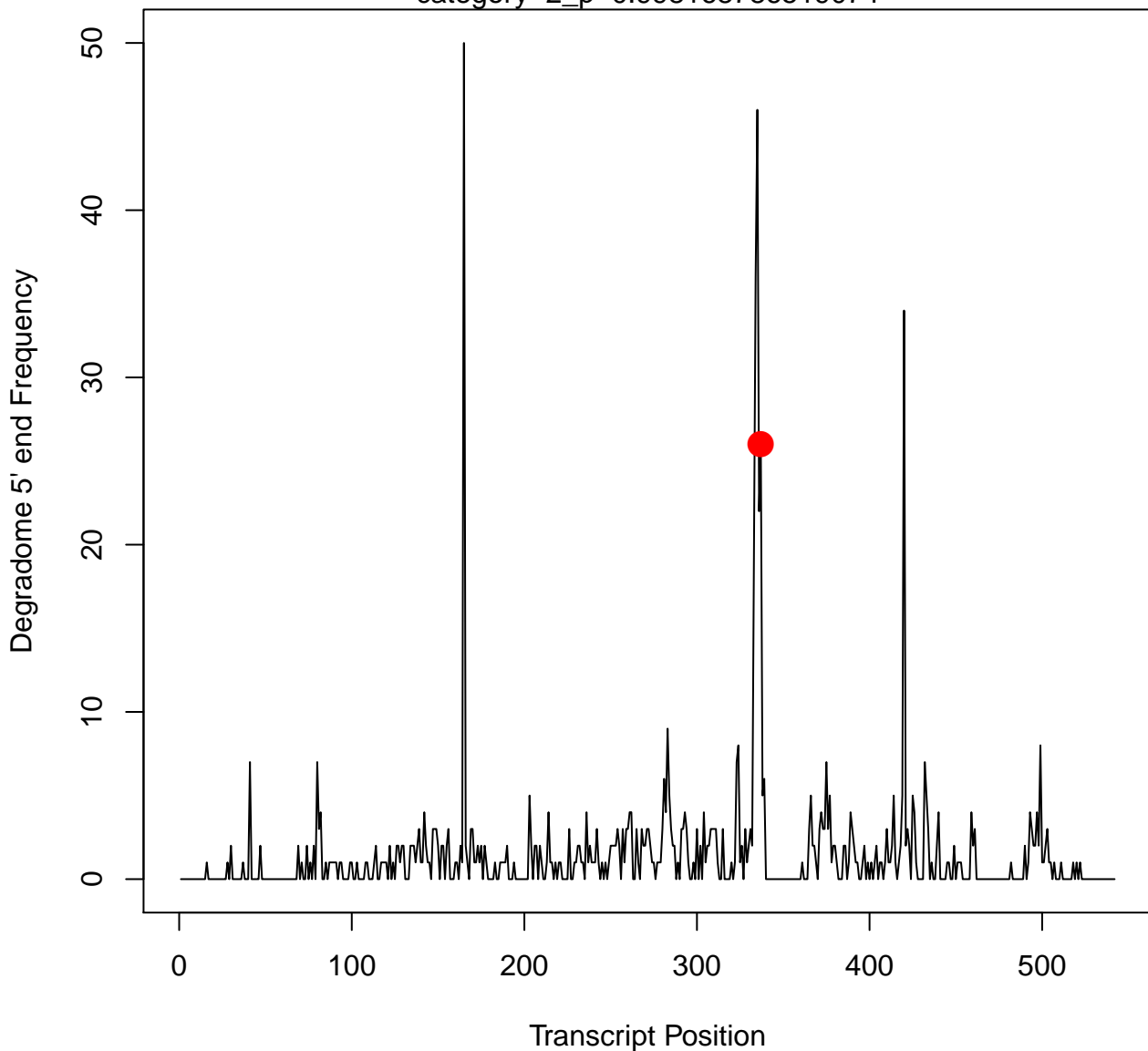

Supplement: Supplementary Data 1 — Results of categories 0–2 from PARE-Seq analysis (including three subfiles:1_1, 1_2, 1_3). [file Data_Sheet_10.ZIP › GSM2230754.plot/Lsa-miR159a_Lsat_1_v5_gn_9_9761.1_337_TPlot.pdf]

**T=Lsat\_1\_v5\_gn\_1\_64661.1\_Q=Lsa-miR159b\_S=652**

category=2\_p=0.993615225856506

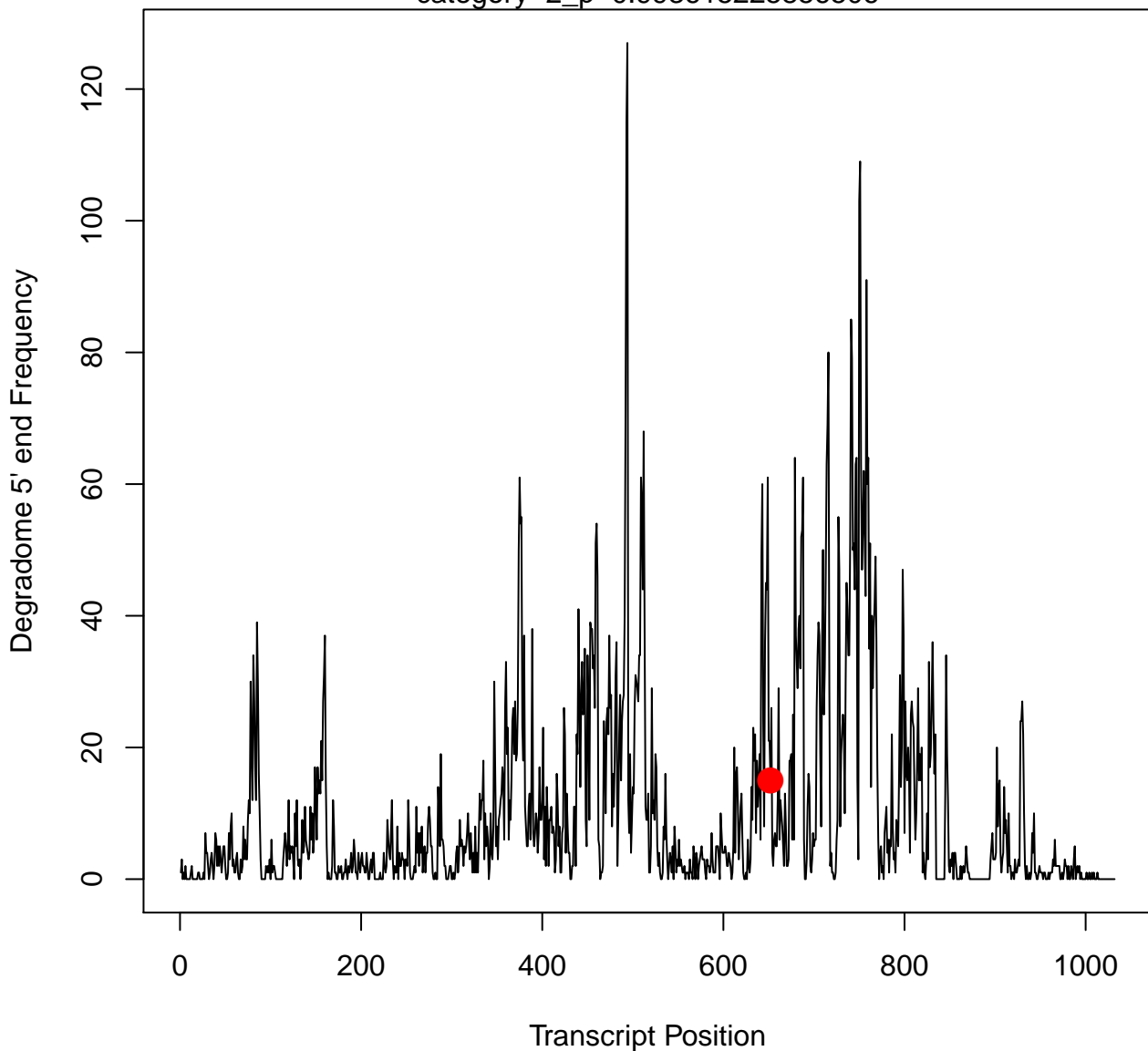

Supplement: Supplementary Data 1 — Results of categories 0–2 from PARE-Seq analysis (including three subfiles:1_1, 1_2, 1_3). [file Data_Sheet_10.ZIP › GSM2230754.plot/Lsa-miR159b_Lsat_1_v5_gn_1_64661.1_652_TPlot.pdf]

**T=Lsat\_1\_v5\_gn\_2\_78240.1\_Q=Lsa-miR159b\_S=2013**

category=2\_p=0.998744676677582

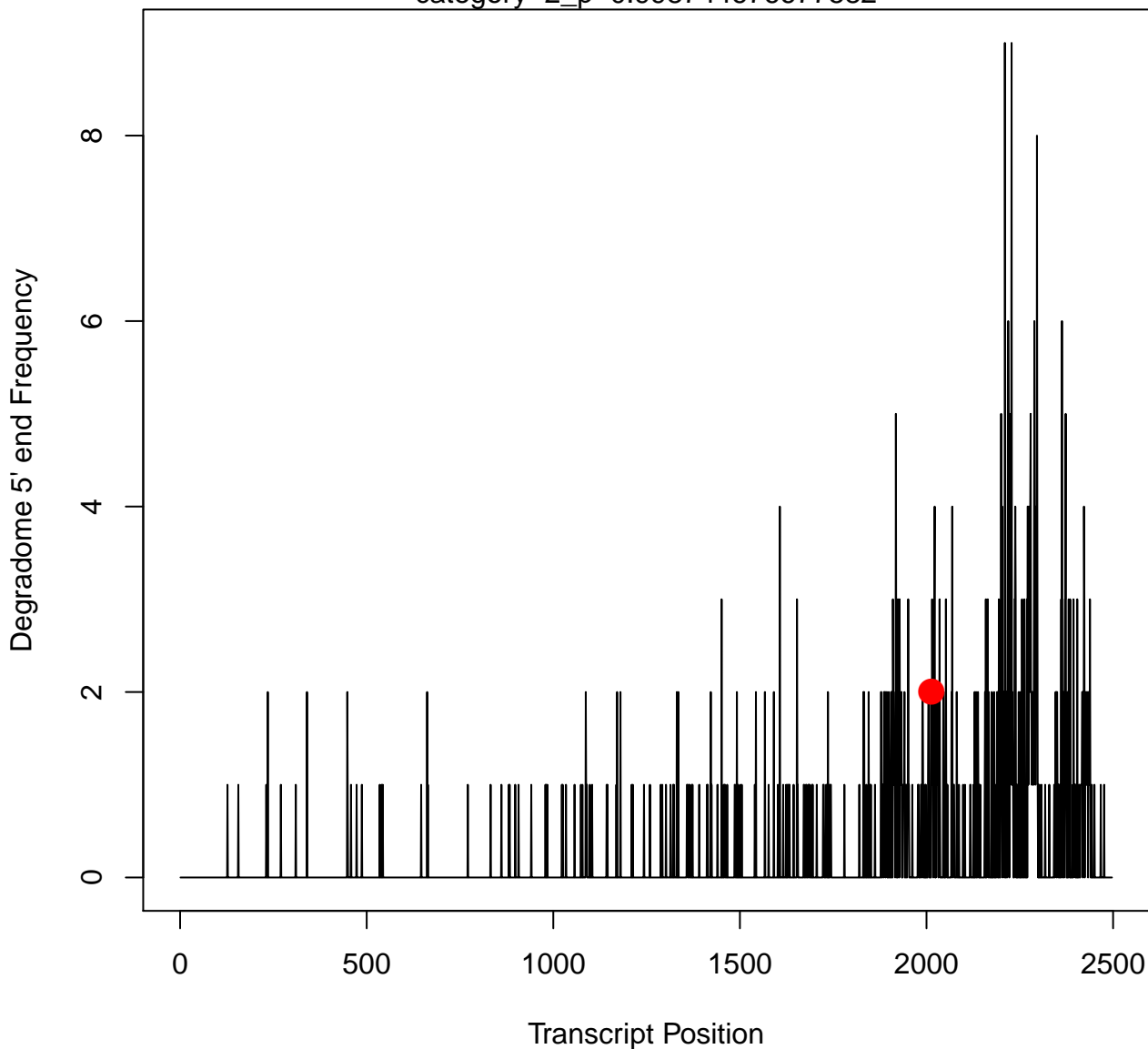

Supplement: Supplementary Data 1 — Results of categories 0–2 from PARE-Seq analysis (including three subfiles:1_1, 1_2, 1_3). [file Data_Sheet_10.ZIP › GSM2230754.plot/Lsa-miR159b_Lsat_1_v5_gn_2_78240.1_2013_TPlot.pdf]

**T=Lsat\_1\_v5\_gn\_3\_54301.1\_Q=Lsa-miR159b\_S=217**

category=2\_p=0.999865887447574

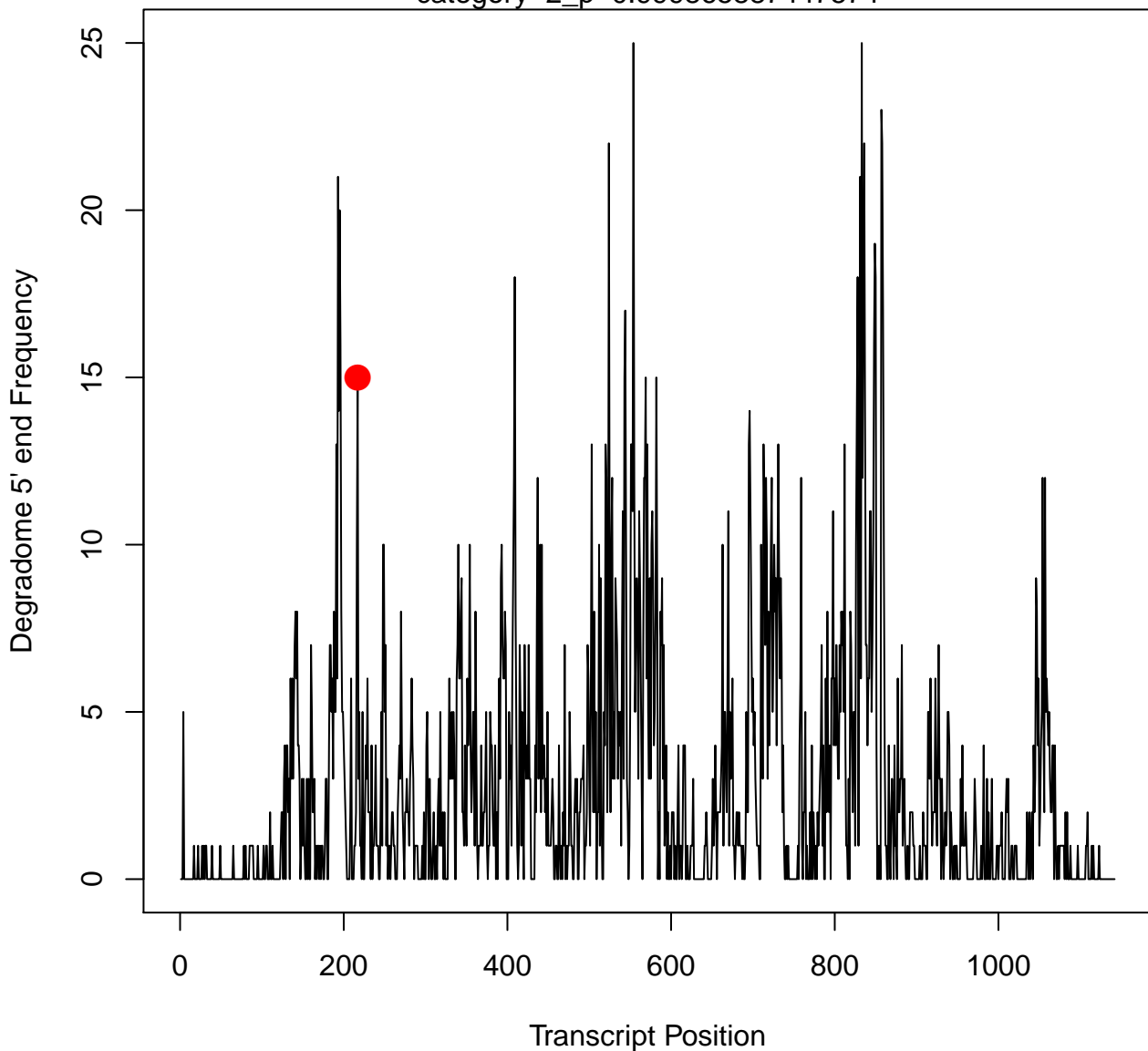

Supplement: Supplementary Data 1 — Results of categories 0–2 from PARE-Seq analysis (including three subfiles:1_1, 1_2, 1_3). [file Data_Sheet_10.ZIP › GSM2230754.plot/Lsa-miR159b_Lsat_1_v5_gn_3_54301.1_217_TPlot.pdf]

**T=Lsat\_1\_v5\_gn\_3\_94000.1\_Q=Lsa-miR159b\_S=137**

category=2\_p=0.389676353965506

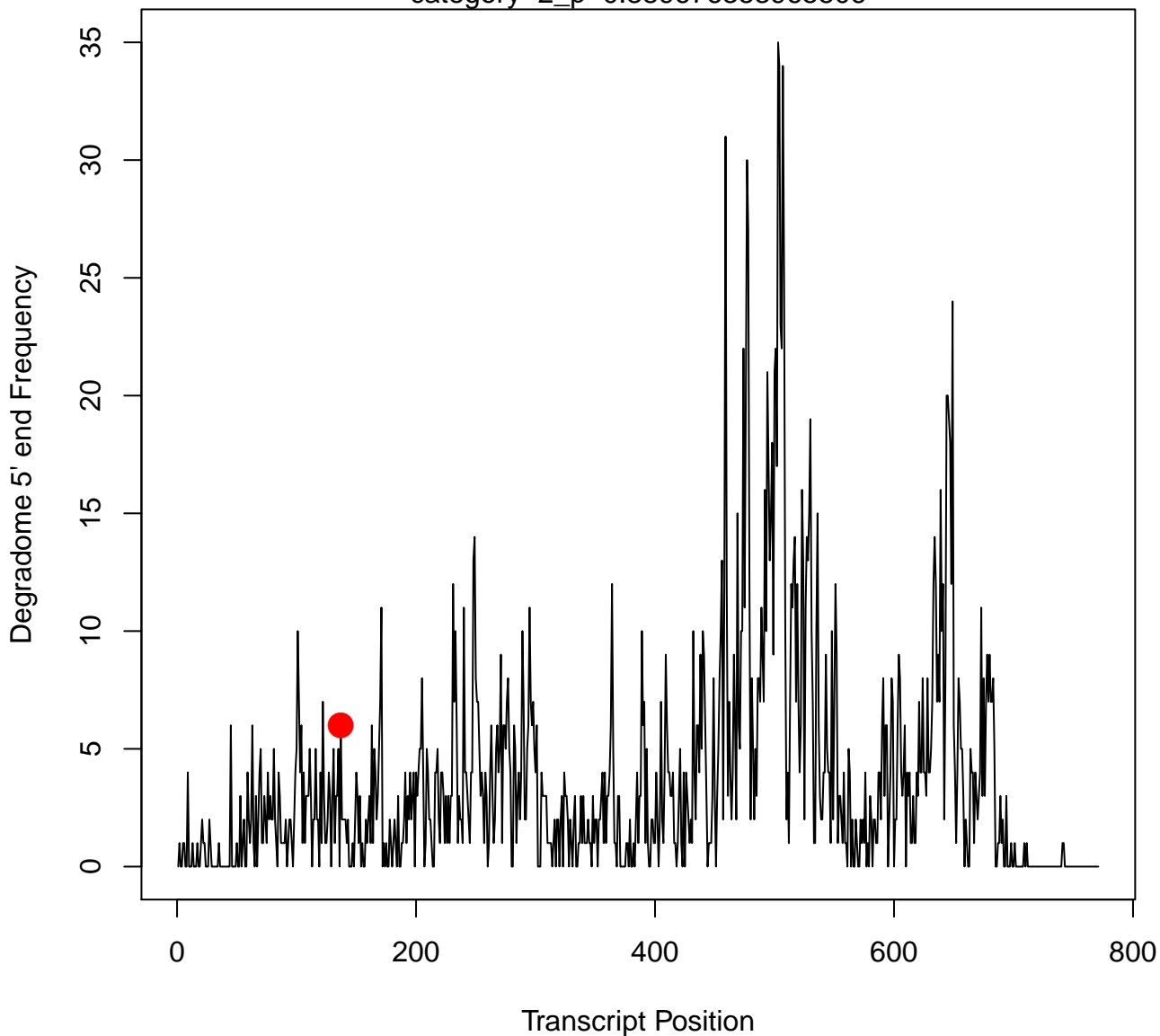

Supplement: Supplementary Data 1 — Results of categories 0–2 from PARE-Seq analysis (including three subfiles:1_1, 1_2, 1_3). [file Data_Sheet_10.ZIP › GSM2230754.plot/Lsa-miR159b_Lsat_1_v5_gn_3_94000.1_137_TPlot.pdf]

**T=Lsat\_1\_v5\_gn\_4\_51460.1\_Q=Lsa-miR159b\_S=379**

category=2\_p=0.252073469146249

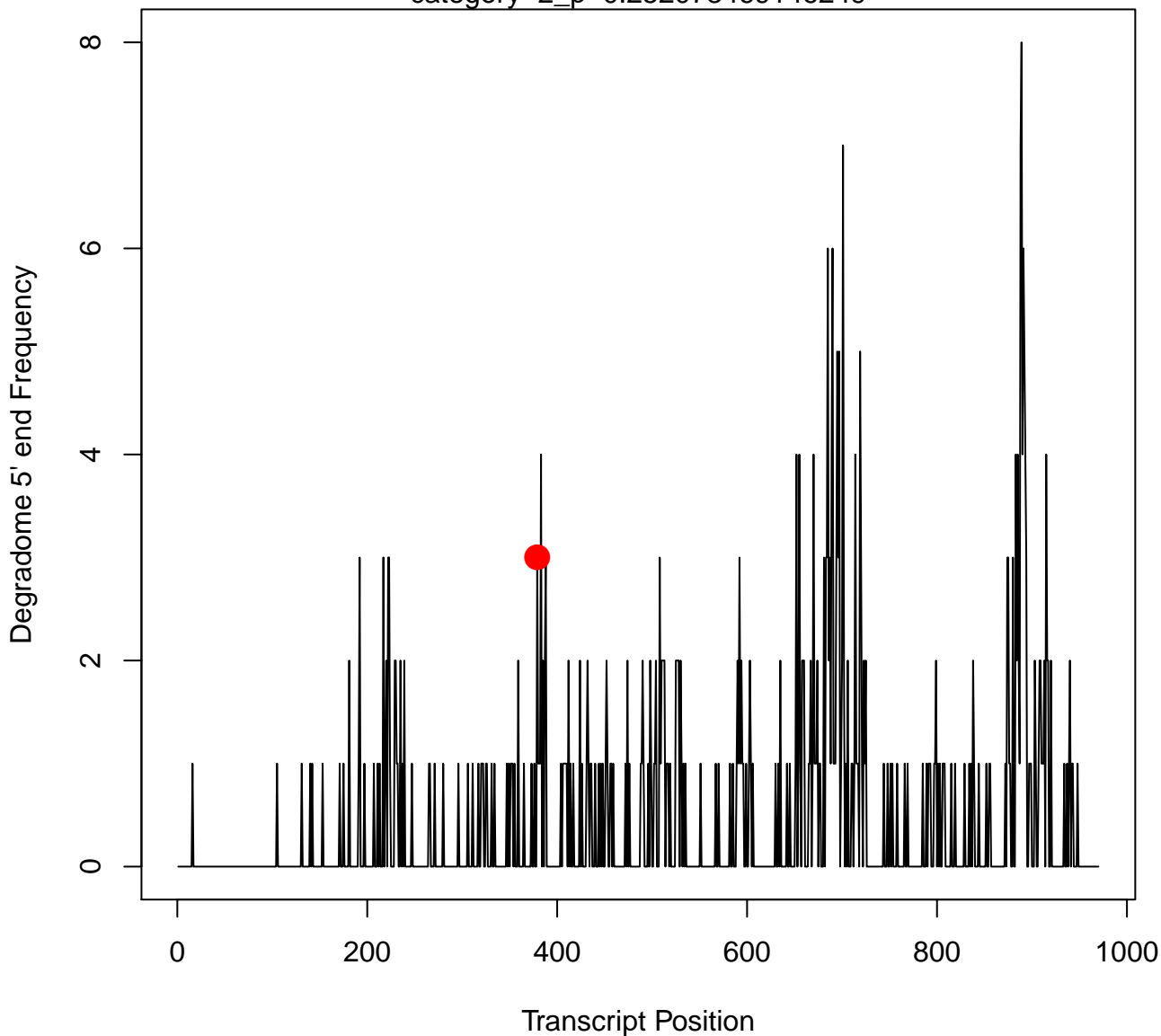

Supplement: Supplementary Data 1 — Results of categories 0–2 from PARE-Seq analysis (including three subfiles:1_1, 1_2, 1_3). [file Data_Sheet_10.ZIP › GSM2230754.plot/Lsa-miR159b_Lsat_1_v5_gn_4_51460.1_379_TPlot.pdf]

**T=Lsat\_1\_v5\_gn\_5\_45900.1\_Q=Lsa-miR159b\_S=3068**

category=2\_p=0.998849429382041

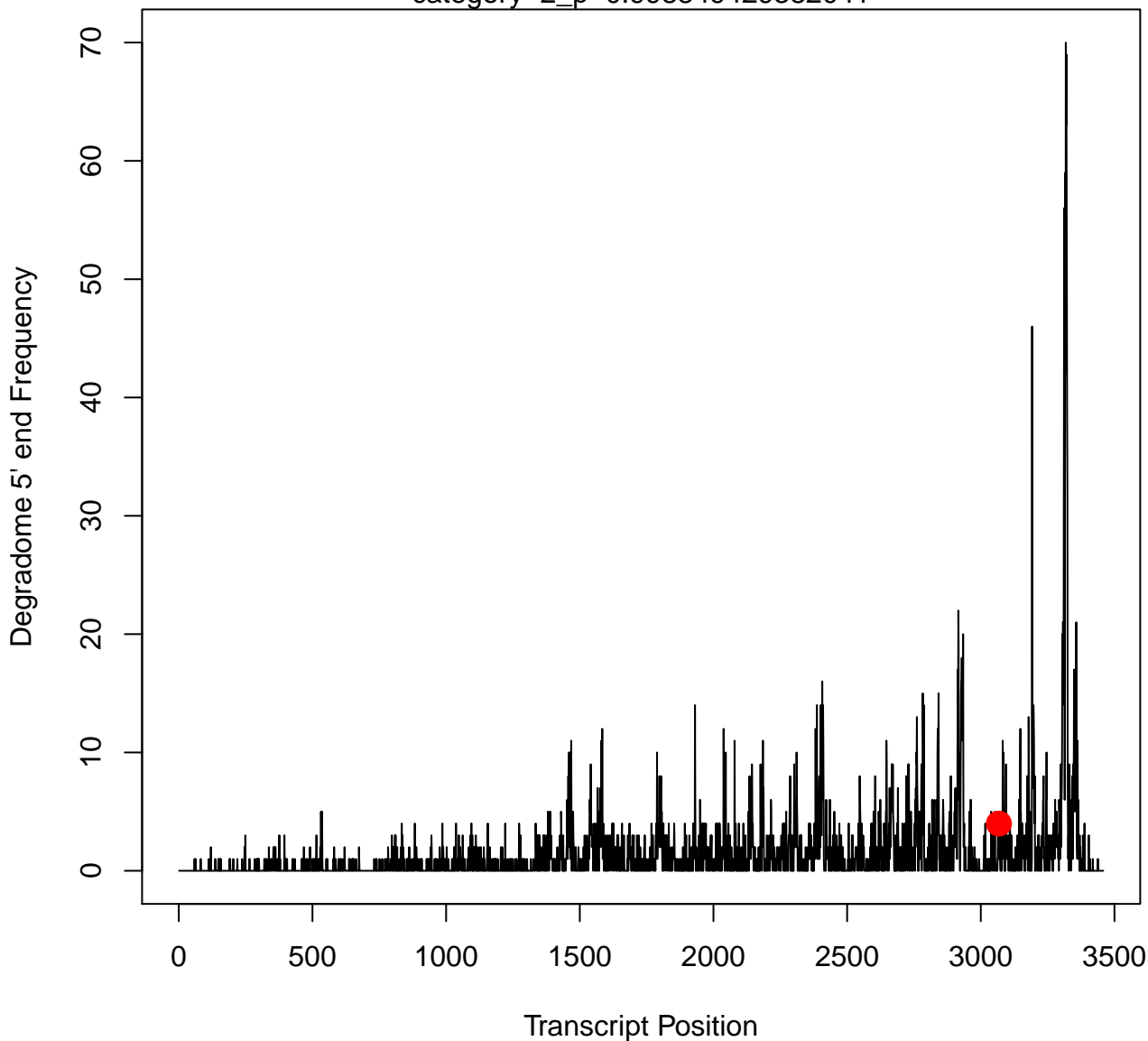

Supplement: Supplementary Data 1 — Results of categories 0–2 from PARE-Seq analysis (including three subfiles:1_1, 1_2, 1_3). [file Data_Sheet_10.ZIP › GSM2230754.plot/Lsa-miR159b_Lsat_1_v5_gn_5_45900.1_3068_TPlot.pdf]

**T=Lsat\_1\_v5\_gn\_5\_68180.1\_Q=Lsa-miR159b\_S=795**

category=2\_p=0.984289759696203

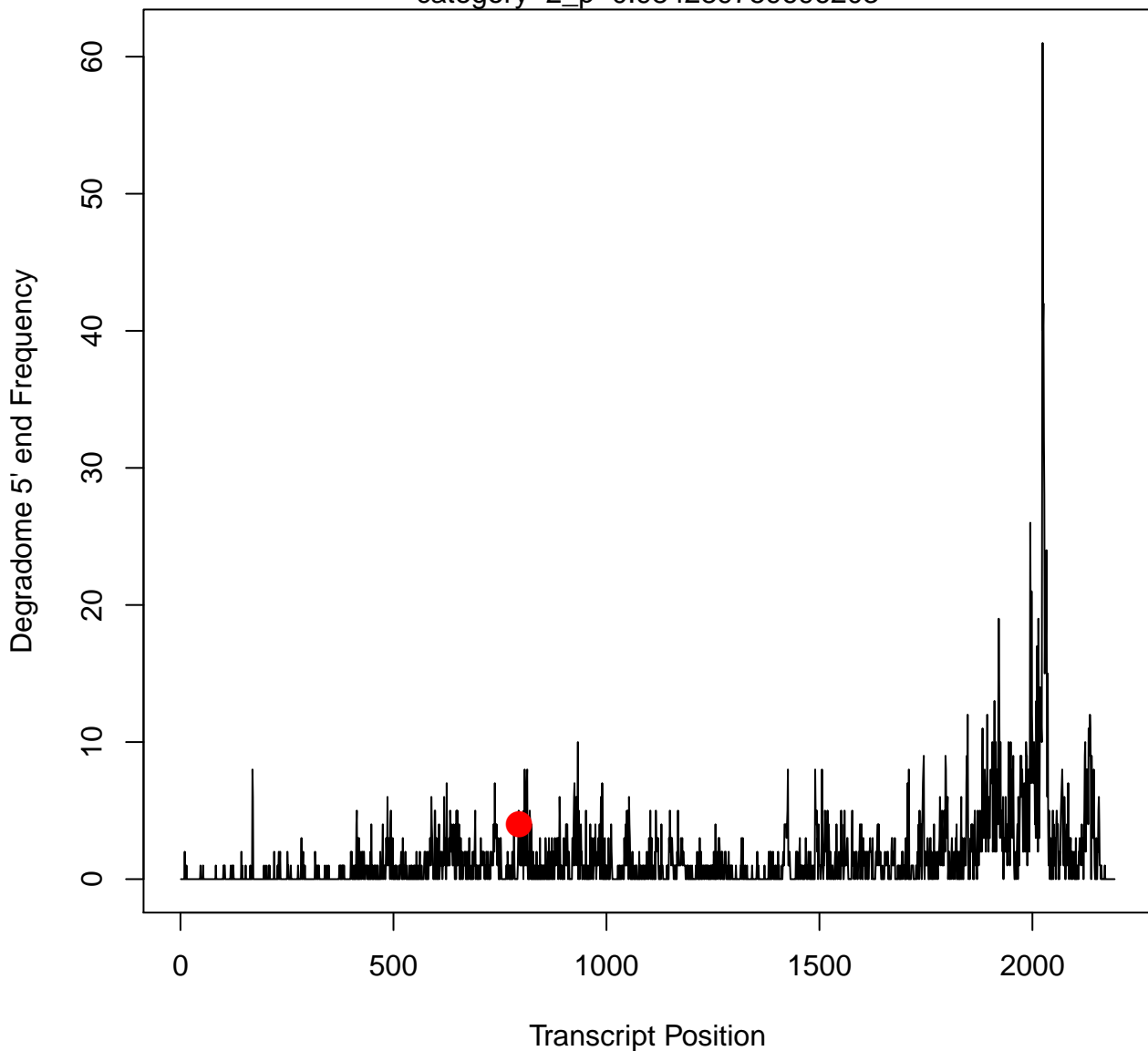

Supplement: Supplementary Data 1 — Results of categories 0–2 from PARE-Seq analysis (including three subfiles:1_1, 1_2, 1_3). [file Data_Sheet_10.ZIP › GSM2230754.plot/Lsa-miR159b_Lsat_1_v5_gn_5_68180.1_795_TPlot.pdf]

**T=Lsat\_1\_v5\_gn\_5\_75800.1\_Q=Lsa-miR159b\_S=1860**

category=2\_p=0.0564350766159708

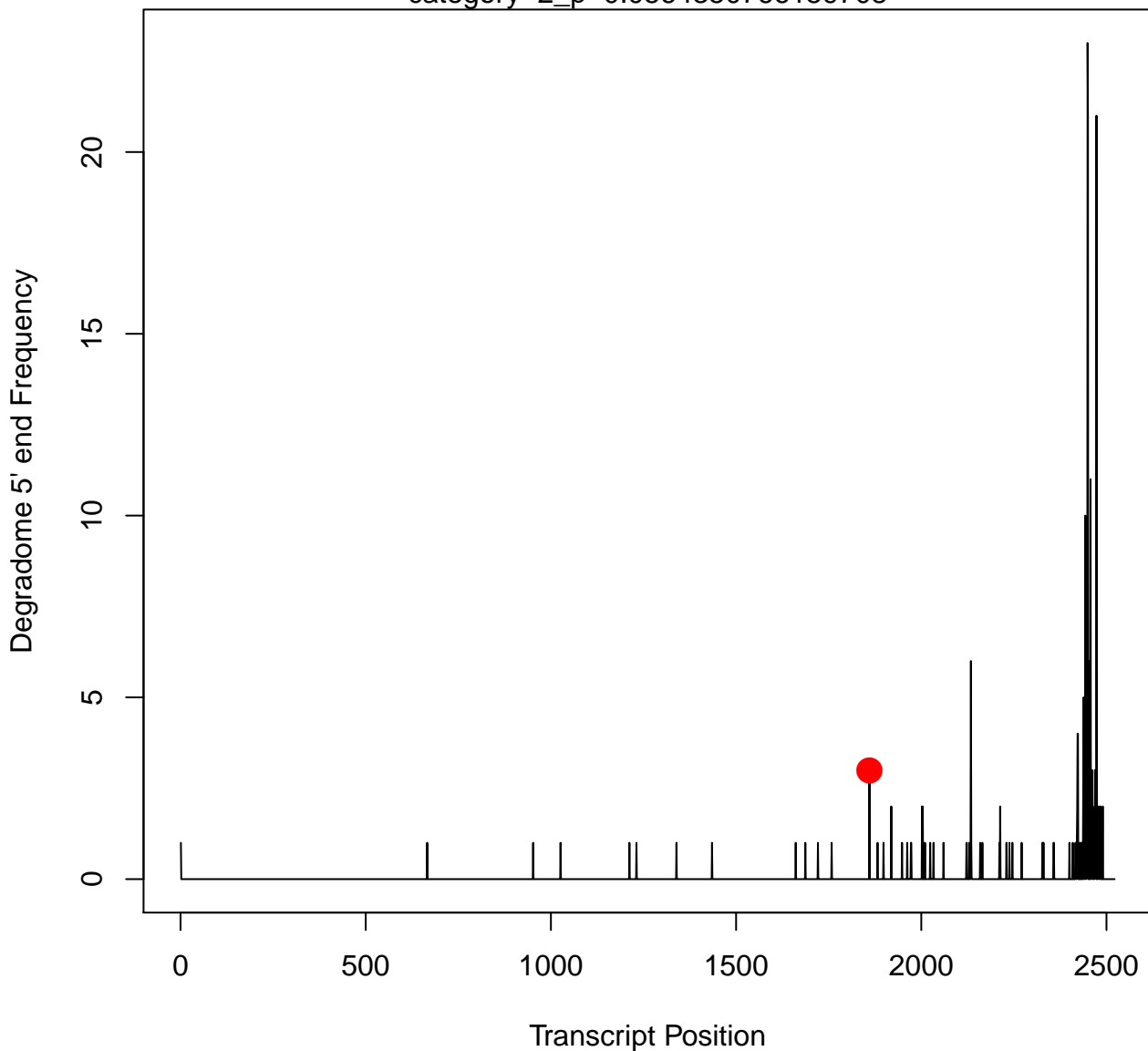

Supplement: Supplementary Data 1 — Results of categories 0–2 from PARE-Seq analysis (including three subfiles:1_1, 1_2, 1_3). [file Data_Sheet_10.ZIP › GSM2230754.plot/Lsa-miR159b_Lsat_1_v5_gn_5_75800.1_1860_TPlot.pdf]

**T=Lsat\_1\_v5\_gn\_6\_23160.1\_Q=Lsa-miR159b\_S=1005**

category=2\_p=0.729376675040129

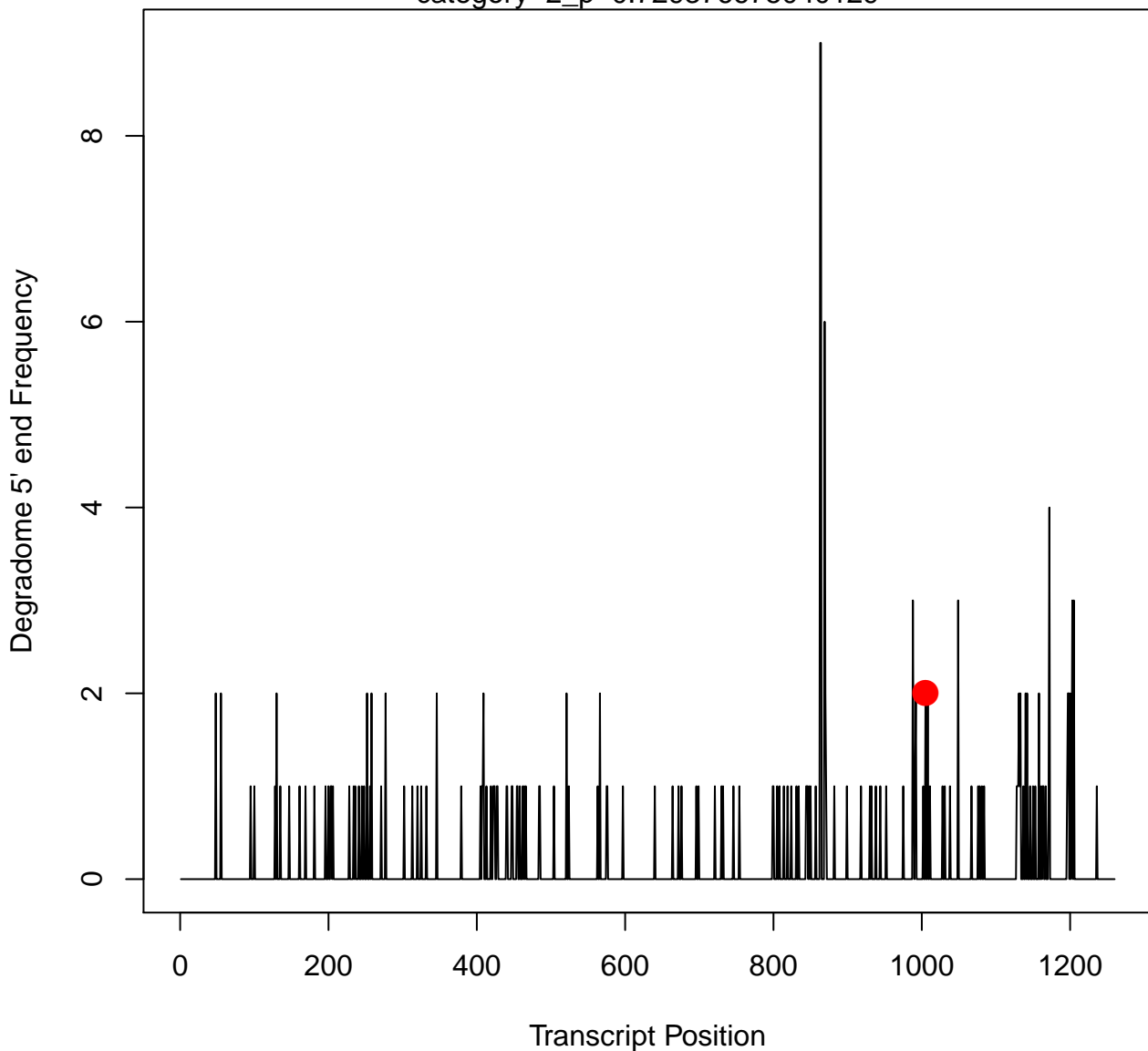

Supplement: Supplementary Data 1 — Results of categories 0–2 from PARE-Seq analysis (including three subfiles:1_1, 1_2, 1_3). [file Data_Sheet_10.ZIP › GSM2230754.plot/Lsa-miR159b_Lsat_1_v5_gn_6_23160.1_1005_TPlot.pdf]

**T=Lsat\_1\_v5\_gn\_7\_29160.1\_Q=Lsa-miR159b\_S=1916**

category=2\_p=0.999798596546394

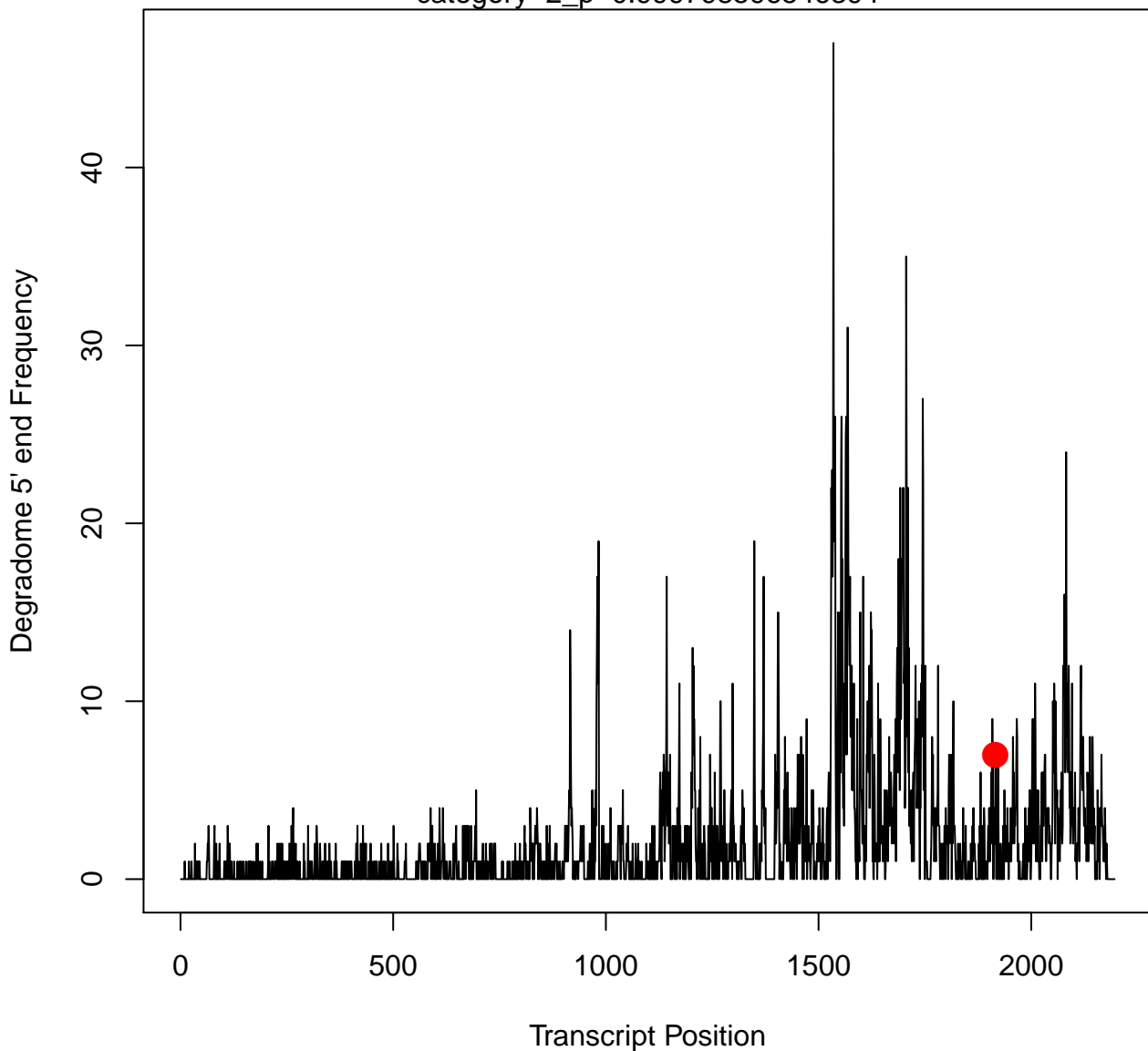

Supplement: Supplementary Data 1 — Results of categories 0–2 from PARE-Seq analysis (including three subfiles:1_1, 1_2, 1_3). [file Data_Sheet_10.ZIP › GSM2230754.plot/Lsa-miR159b_Lsat_1_v5_gn_7_29160.1_1916_TPlot.pdf]

**T=Lsat\_1\_v5\_gn\_9\_121780.1\_Q=Lsa-miR159b\_S=2972**

category=2\_p=0.999474790691517

Degradome 5' end Frequency

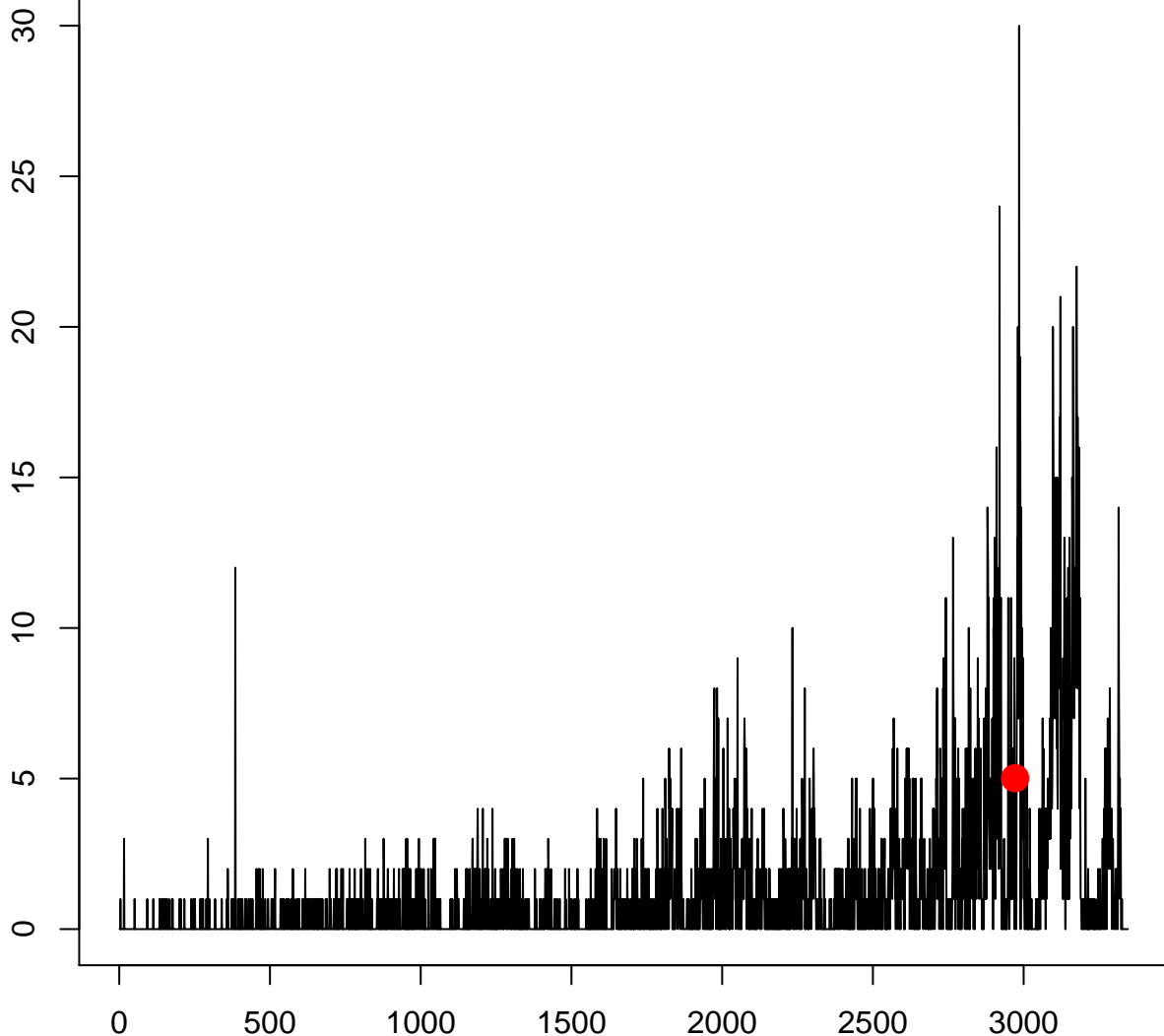

Transcript Position

Supplement: Supplementary Data 1 — Results of categories 0–2 from PARE-Seq analysis (including three subfiles:1_1, 1_2, 1_3). [file Data_Sheet_10.ZIP › GSM2230754.plot/Lsa-miR159b_Lsat_1_v5_gn_9_121780.1_2972_TPlot.pdf]

**T=Lsat\_1\_v5\_gn\_2\_112381.1\_Q=Lsa-miR160a\_S=1289**

category=0\_p=0.00112692513817547

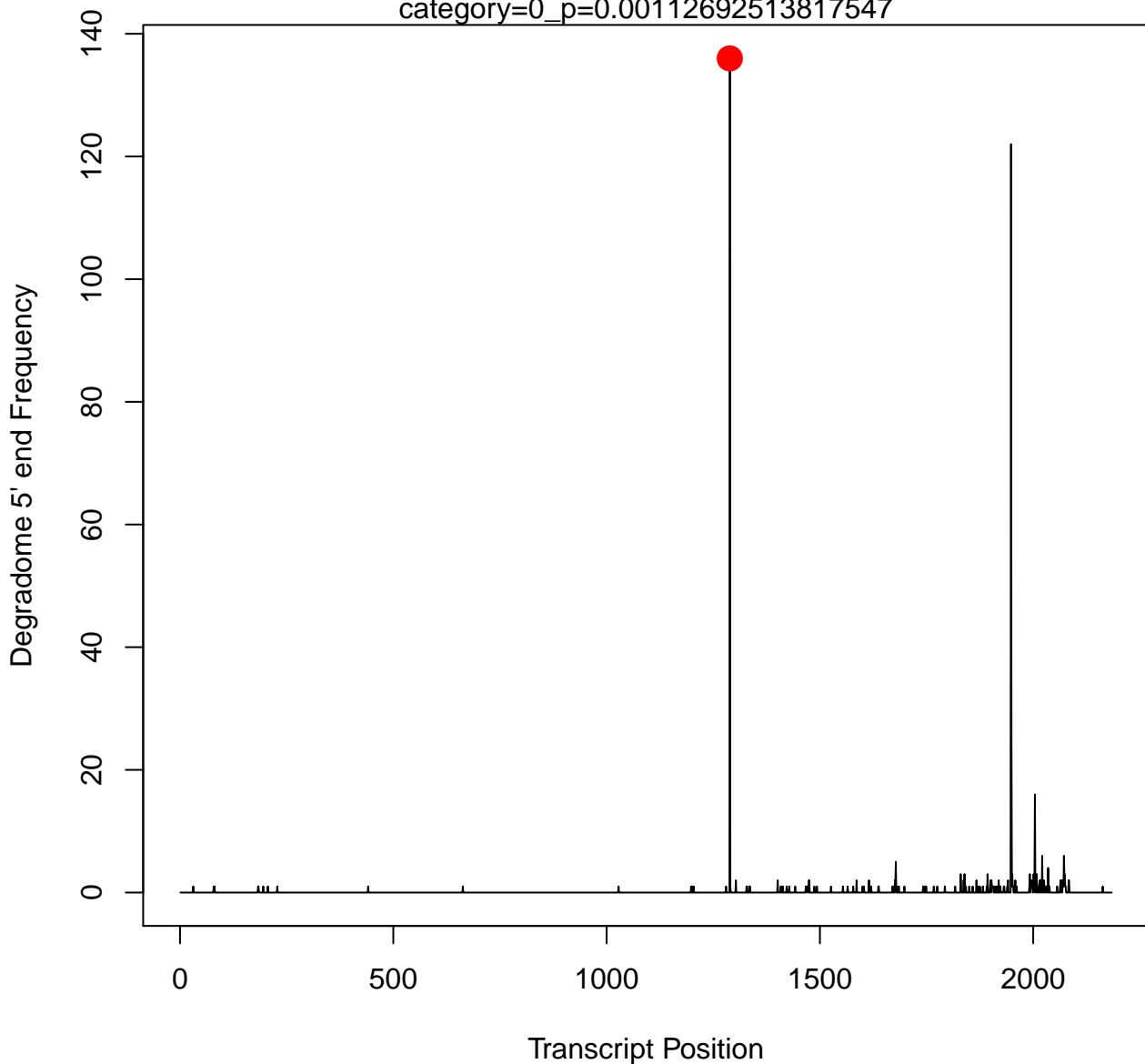

Supplement: Supplementary Data 1 — Results of categories 0–2 from PARE-Seq analysis (including three subfiles:1_1, 1_2, 1_3). [file Data_Sheet_10.ZIP › GSM2230754.plot/Lsa-miR160a_Lsat_1_v5_gn_2_112381.1_1289_TPlot.pdf]

**T=Lsat\_1\_v5\_gn\_2\_39021.1\_Q=Lsa-miR160a\_S=906**

category=2\_p=0.772657522211231

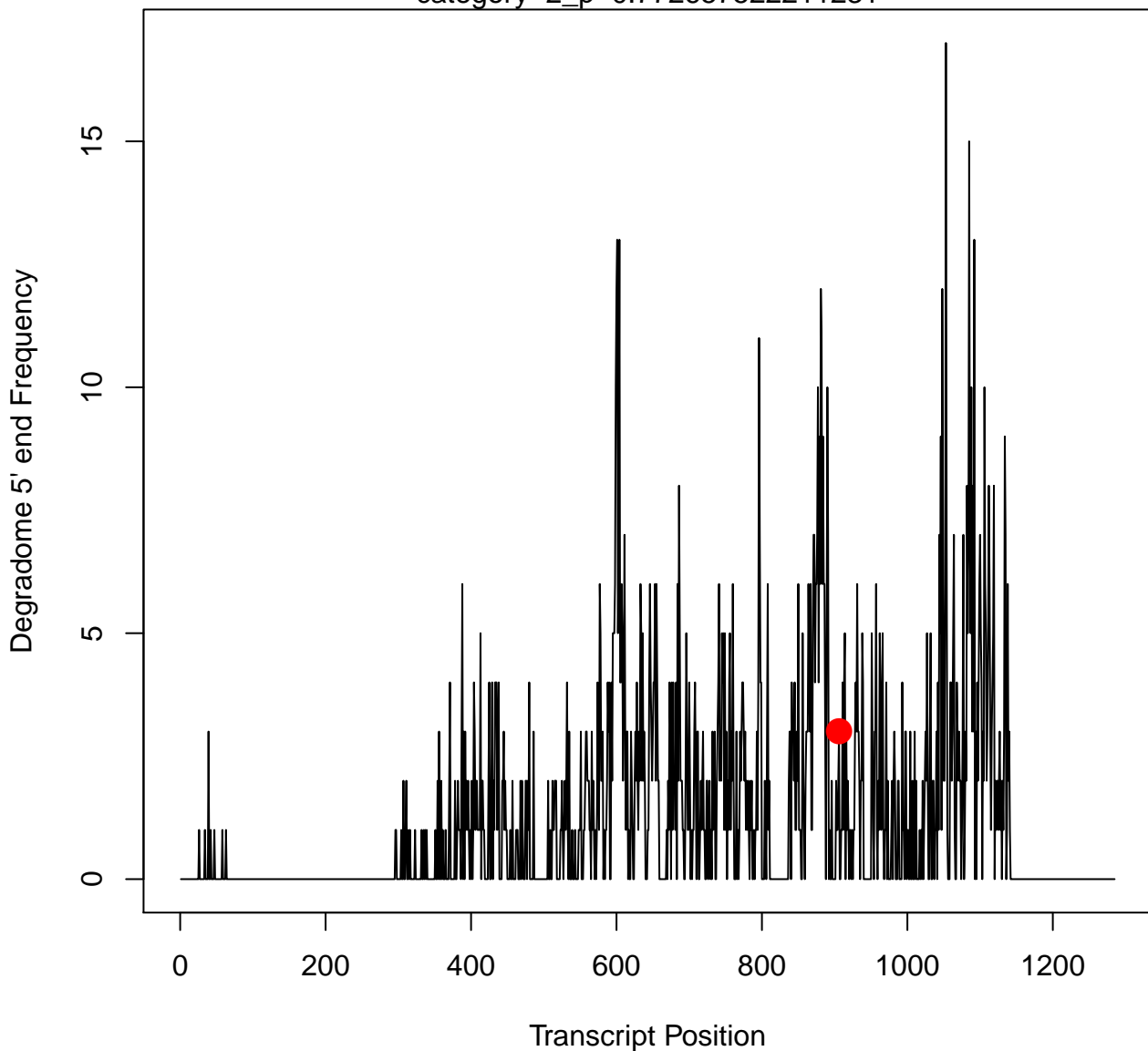

Supplement: Supplementary Data 1 — Results of categories 0–2 from PARE-Seq analysis (including three subfiles:1_1, 1_2, 1_3). [file Data_Sheet_10.ZIP › GSM2230754.plot/Lsa-miR160a_Lsat_1_v5_gn_2_39021.1_906_TPlot.pdf]

**T=Lsat\_1\_v5\_gn\_8\_20161.1\_Q=Lsa-miR160a\_S=1819**

category=0\_p=0.00150228456680068

Degradome 5' end Frequency

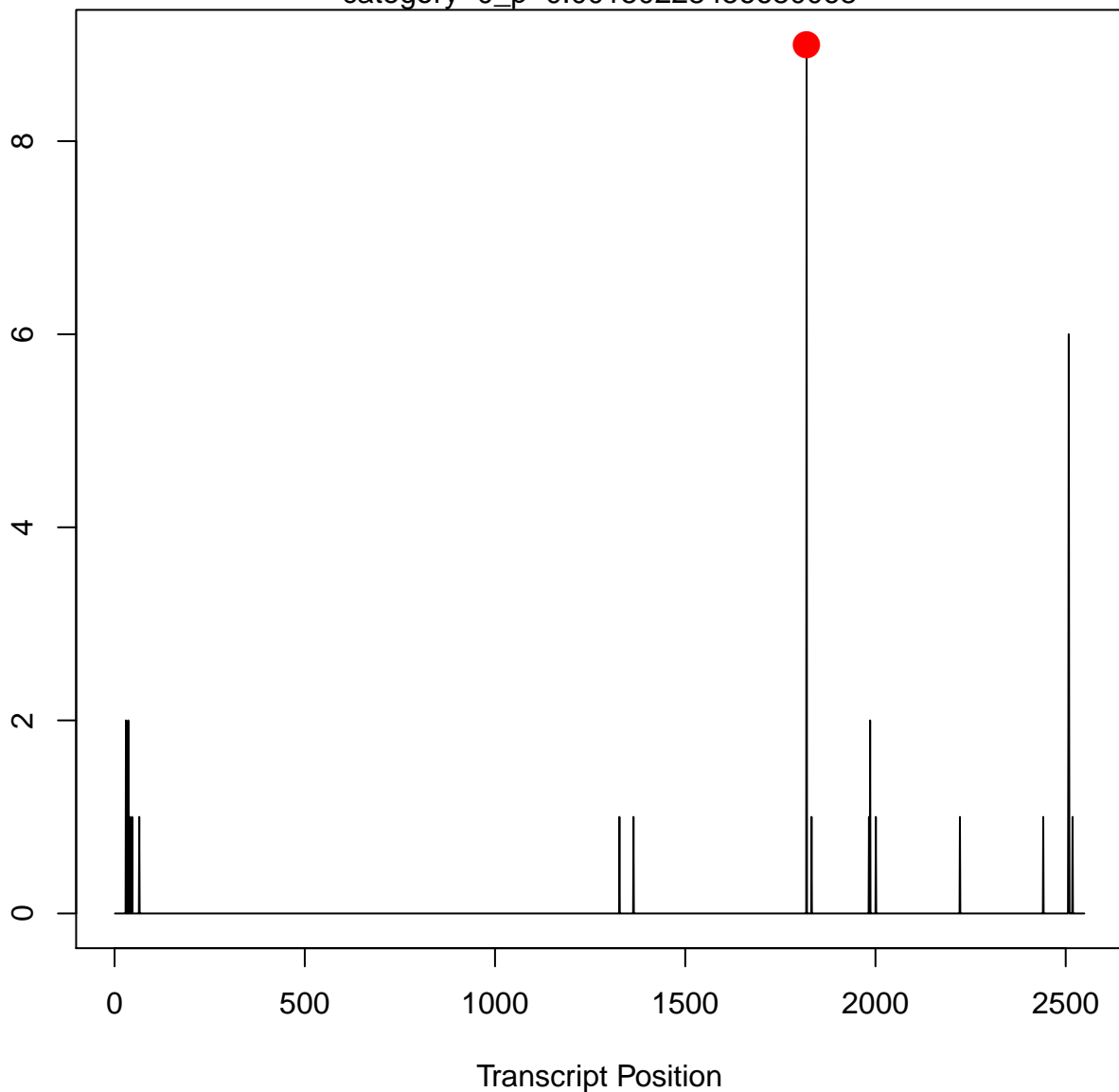

Supplement: Supplementary Data 1 — Results of categories 0–2 from PARE-Seq analysis (including three subfiles:1_1, 1_2, 1_3). [file Data_Sheet_10.ZIP › GSM2230754.plot/Lsa-miR160a_Lsat_1_v5_gn_8_20161.1_1819_TPlot.pdf]

**T=Lsat\_1\_v5\_gn\_2\_100181.1\_Q=Lsa-miR160b\_S=2076**

category=2\_p=0.0834467922232335

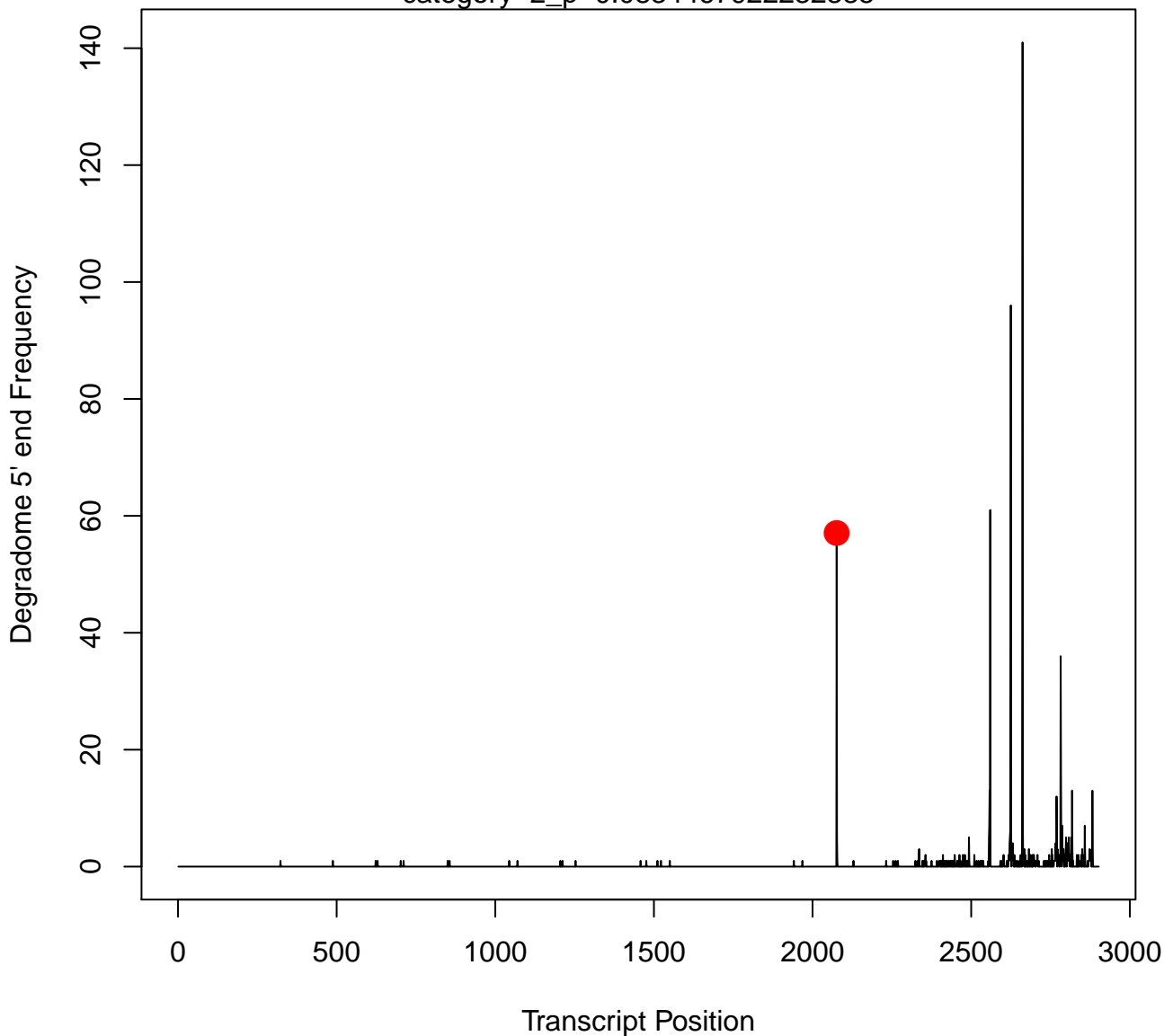

Supplement: Supplementary Data 1 — Results of categories 0–2 from PARE-Seq analysis (including three subfiles:1_1, 1_2, 1_3). [file Data_Sheet_10.ZIP › GSM2230754.plot/Lsa-miR160b_Lsat_1_v5_gn_2_100181.1_2076_TPlot.pdf]

**T=Lsat\_1\_v5\_gn\_6\_30741.1\_Q=Lsa-miR160b\_S=2067**

category=0\_p=0.00037578290783058

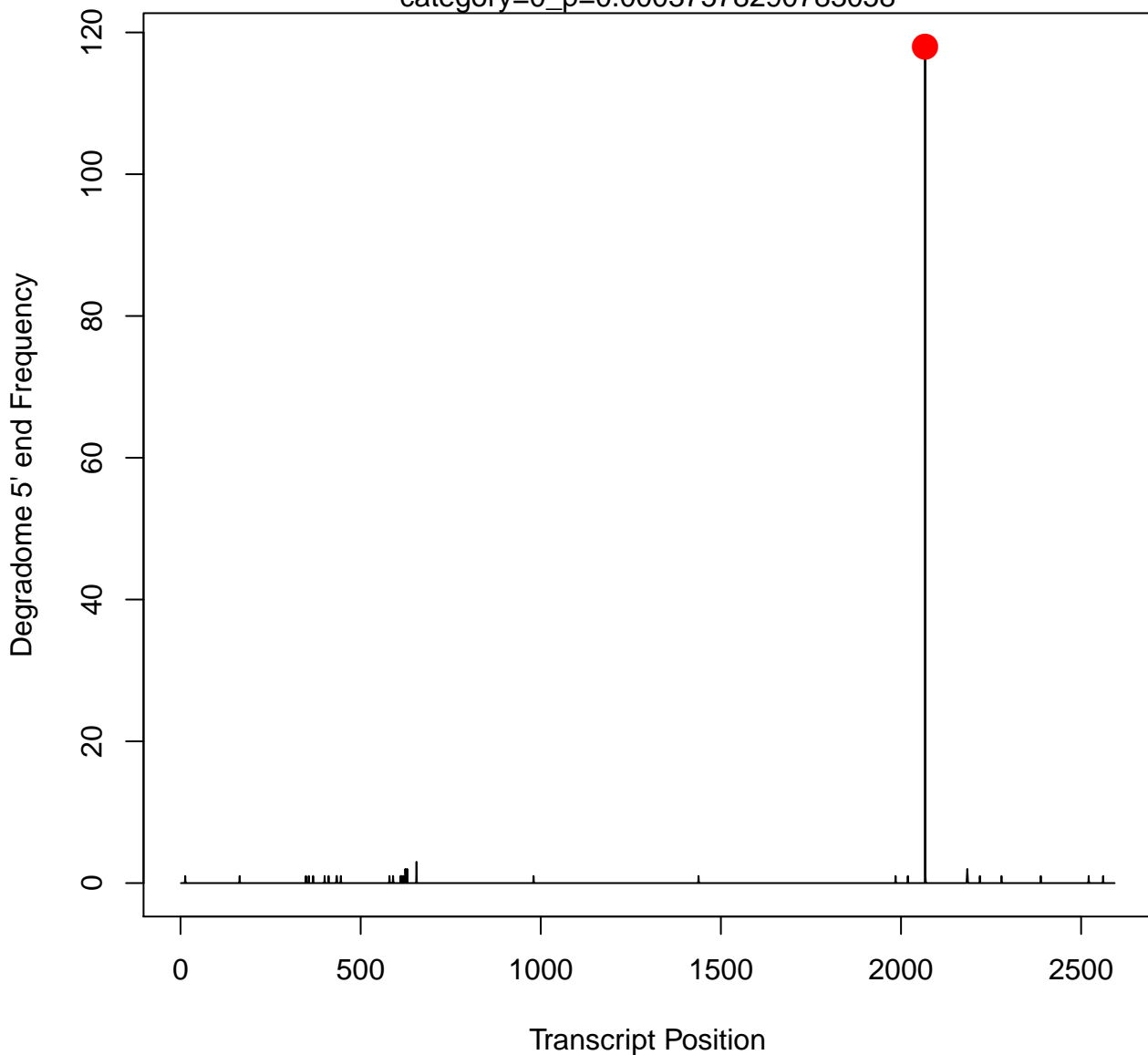

Supplement: Supplementary Data 1 — Results of categories 0–2 from PARE-Seq analysis (including three subfiles:1_1, 1_2, 1_3). [file Data_Sheet_10.ZIP › GSM2230754.plot/Lsa-miR160b_Lsat_1_v5_gn_6_30741.1_2067_TPlot.pdf]

**T=Lsat\_1\_v5\_gn\_7\_80140.1\_Q=Lsa-miR160b\_S=1719**

category=2\_p=0.834832185608514

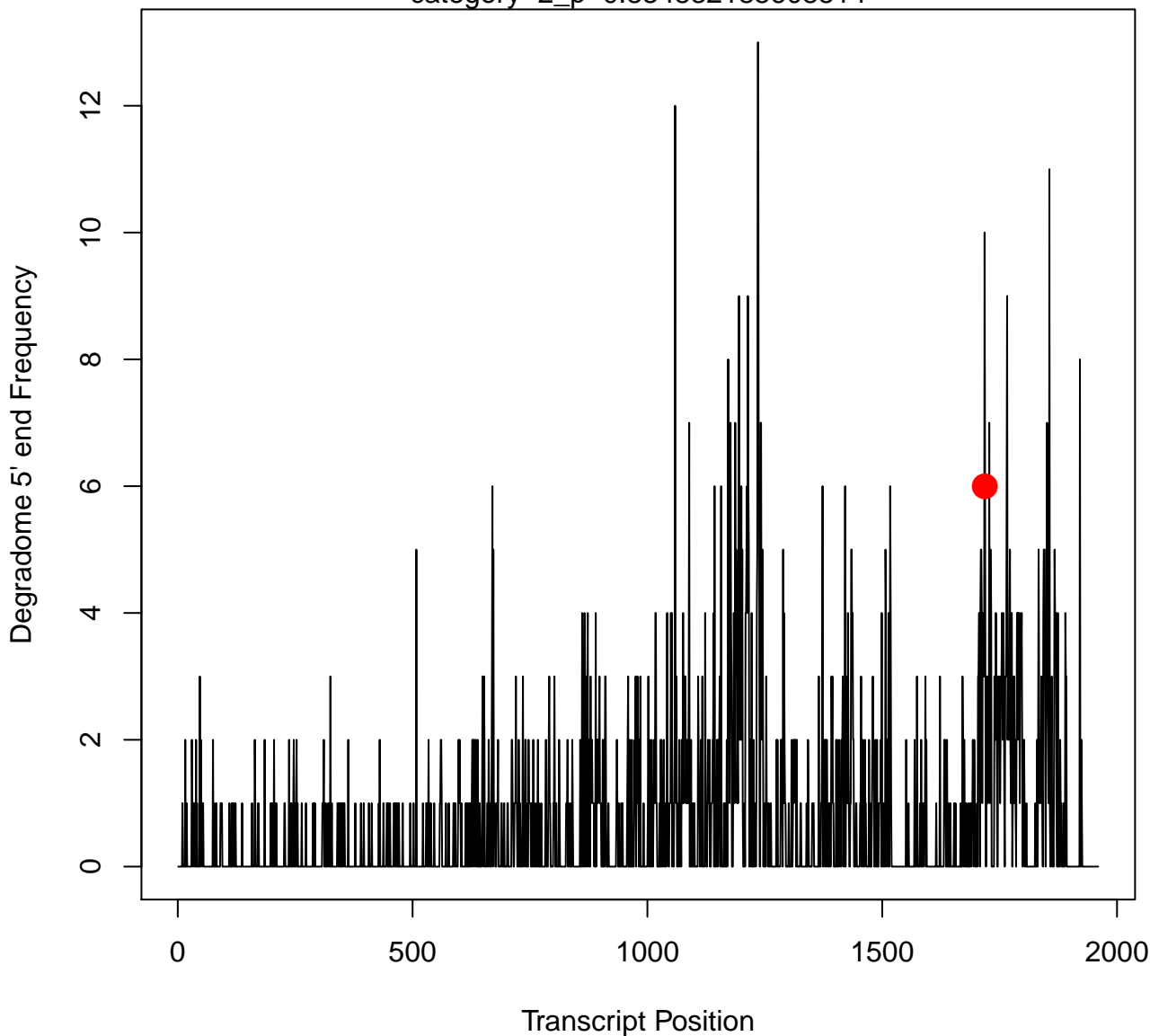

Supplement: Supplementary Data 1 — Results of categories 0–2 from PARE-Seq analysis (including three subfiles:1_1, 1_2, 1_3). [file Data_Sheet_10.ZIP › GSM2230754.plot/Lsa-miR160b_Lsat_1_v5_gn_7_80140.1_1719_TPlot.pdf]

**T=Lsat\_1\_v5\_gn\_9\_69060.1\_Q=Lsa-miR160b\_S=1181**

category=0\_p=0.000751424602867257

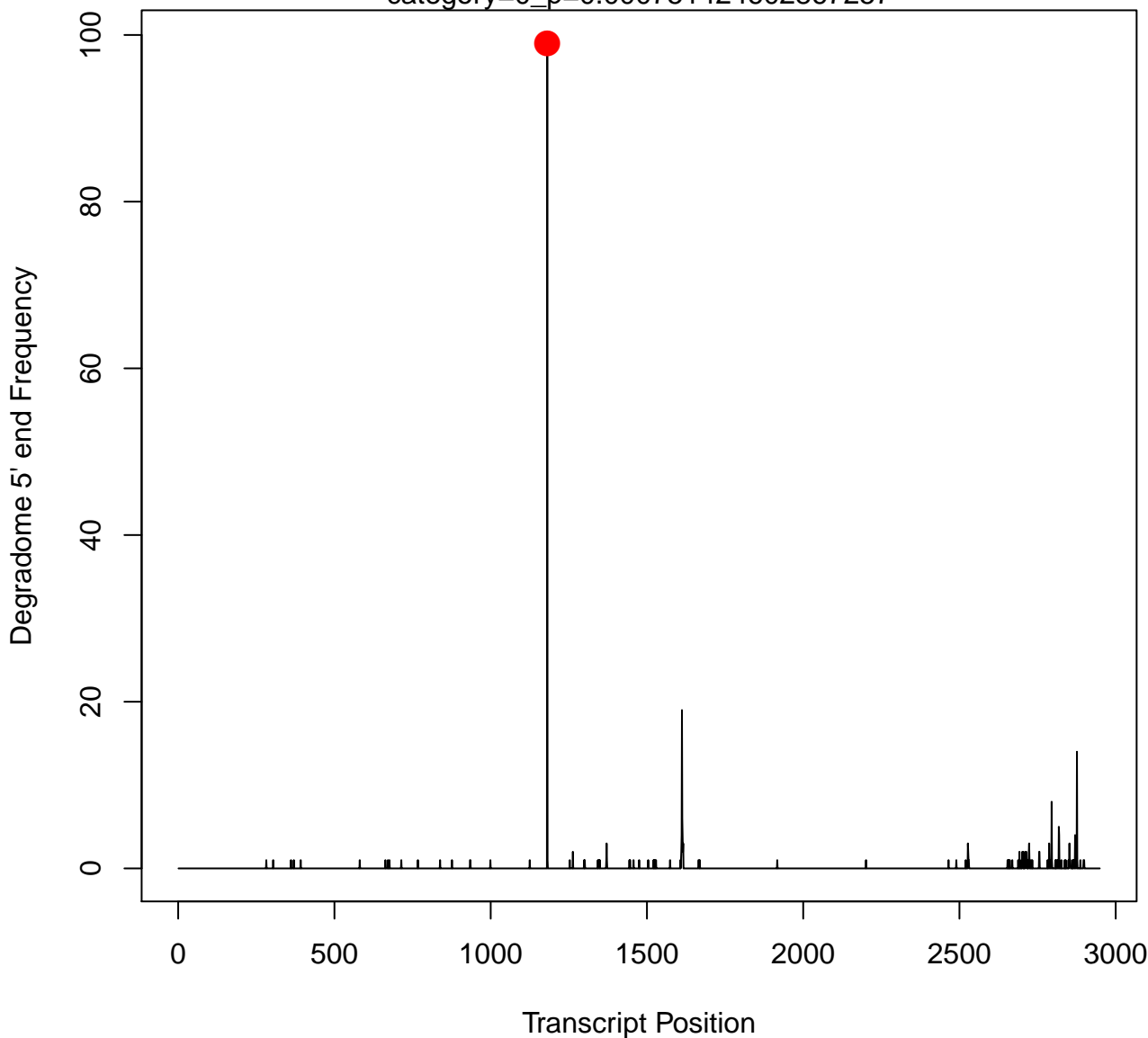

Supplement: Supplementary Data 1 — Results of categories 0–2 from PARE-Seq analysis (including three subfiles:1_1, 1_2, 1_3). [file Data_Sheet_10.ZIP › GSM2230754.plot/Lsa-miR160b_Lsat_1_v5_gn_9_69060.1_1181_TPlot.pdf]

**T=Lsat\_1\_v5\_gn\_4\_109600.1\_Q=Lsa-miR164a\_S=877**

category=0\_p=0.00112692513817547

Degradome 5' end Frequency

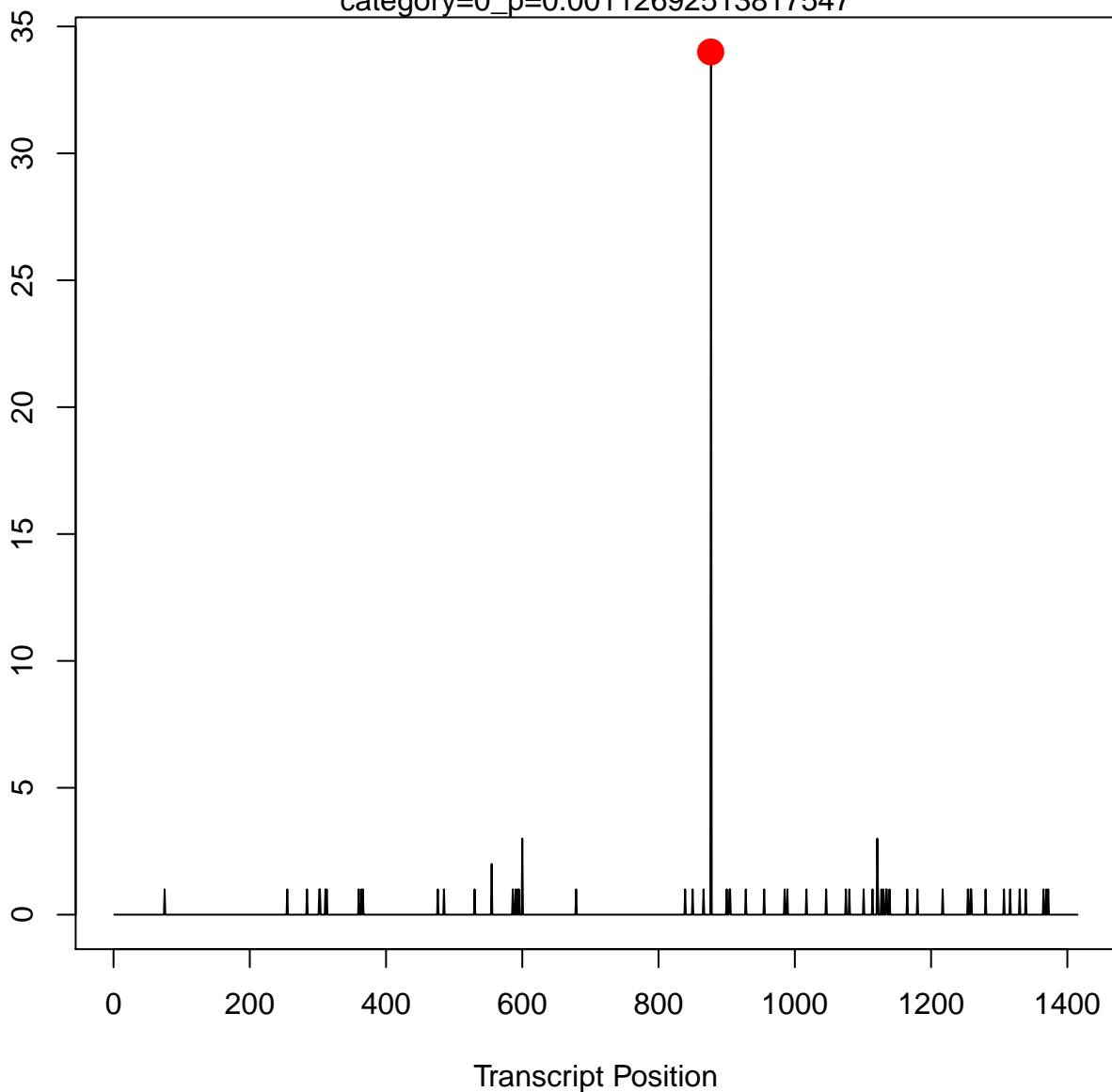

Supplement: Supplementary Data 1 — Results of categories 0–2 from PARE-Seq analysis (including three subfiles:1_1, 1_2, 1_3). [file Data_Sheet_10.ZIP › GSM2230754.plot/Lsa-miR164a_Lsat_1_v5_gn_4_109600.1_877_TPlot.pdf]

**T=Lsat\_1\_v5\_gn\_0\_45640.1\_Q=Lsa-miR164b\_S=643**

category=0\_p=0.0026275167427332

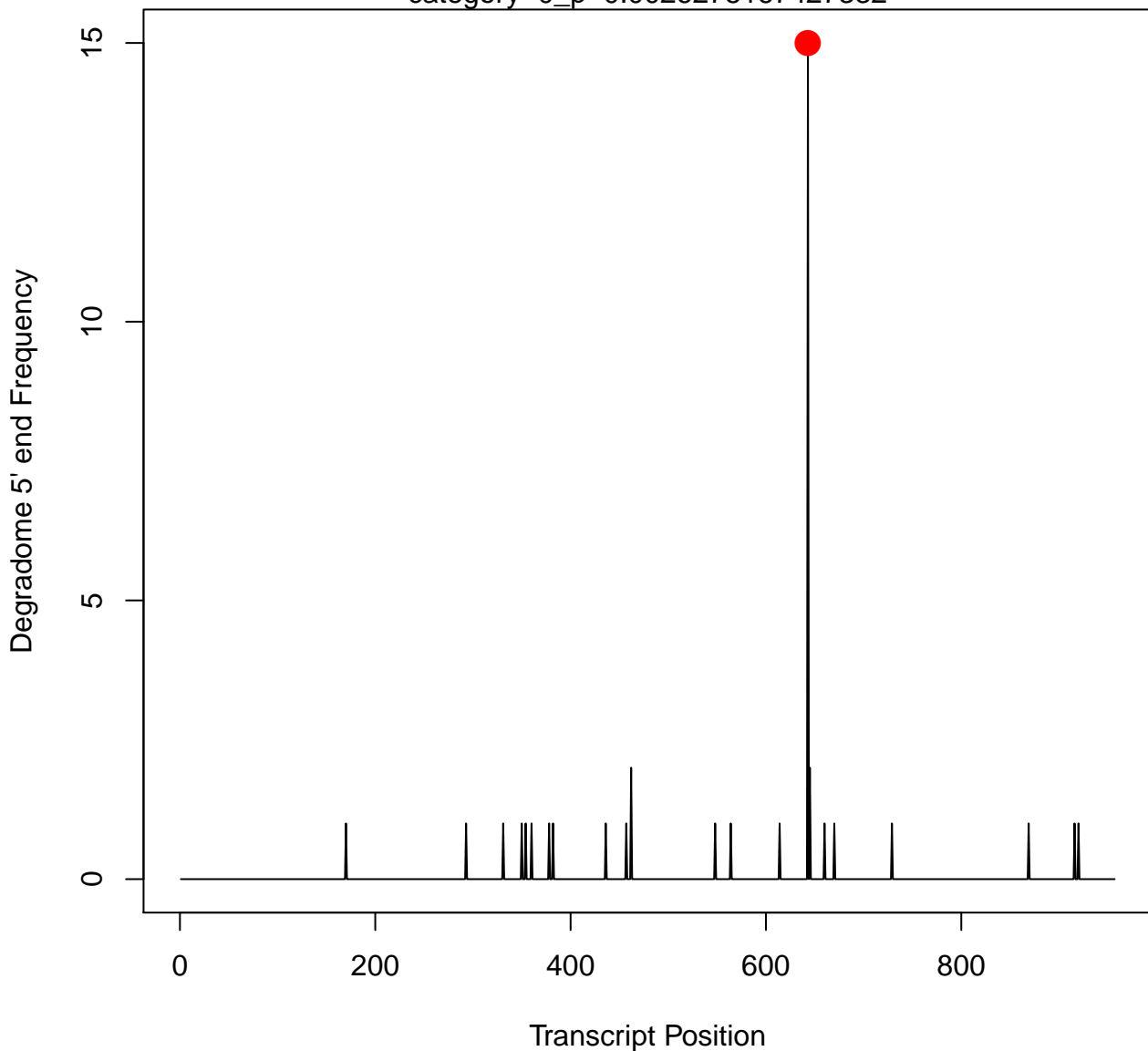

Supplement: Supplementary Data 1 — Results of categories 0–2 from PARE-Seq analysis (including three subfiles:1_1, 1_2, 1_3). [file Data_Sheet_10.ZIP › GSM2230754.plot/Lsa-miR164b_Lsat_1_v5_gn_0_45640.1_643_TPlot.pdf]

**T=Lsat\_1\_v5\_gn\_4\_73580.1\_Q=Lsa-miR164b\_S=963**

category=2\_p=0.803387983003733

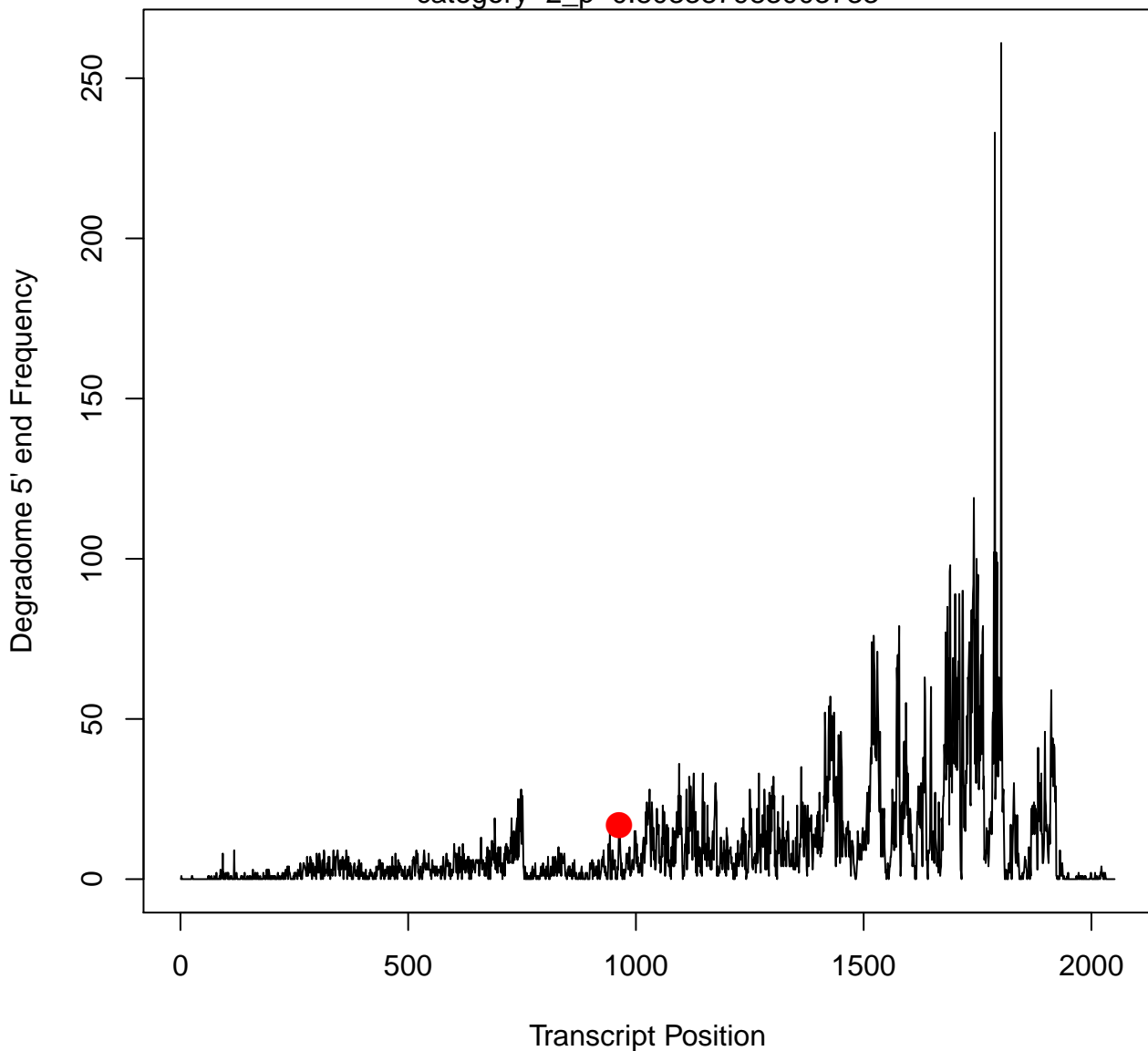

Supplement: Supplementary Data 1 — Results of categories 0–2 from PARE-Seq analysis (including three subfiles:1_1, 1_2, 1_3). [file Data_Sheet_10.ZIP › GSM2230754.plot/Lsa-miR164b_Lsat_1_v5_gn_4_73580.1_963_TPlot.pdf]

**T=Lsat\_1\_v5\_gn\_5\_981.1\_Q=Lsa-miR164b\_S=601**

category=0\_p=0.00112692513817547

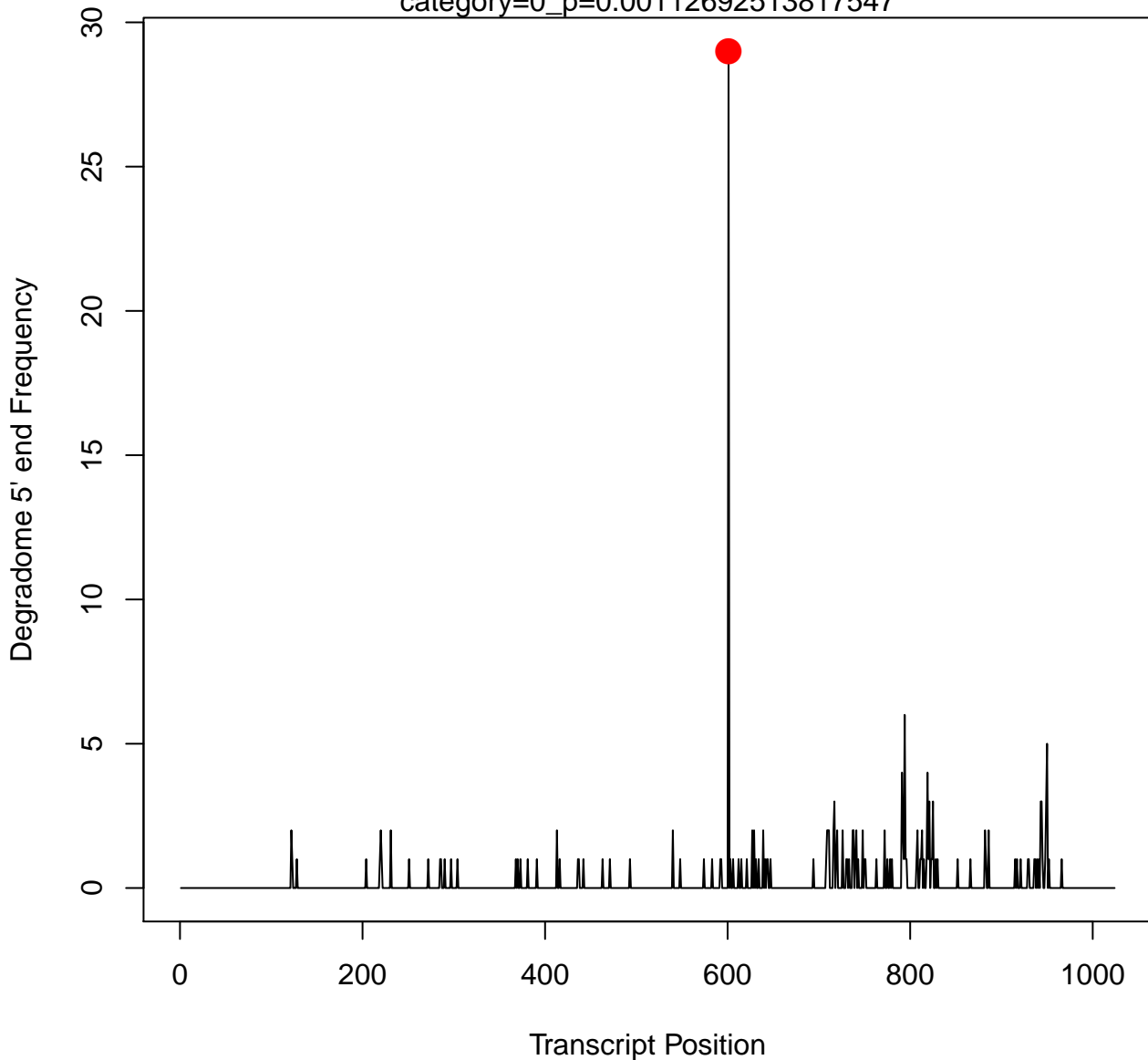

Supplement: Supplementary Data 1 — Results of categories 0–2 from PARE-Seq analysis (including three subfiles:1_1, 1_2, 1_3). [file Data_Sheet_10.ZIP › GSM2230754.plot/Lsa-miR164b_Lsat_1_v5_gn_5_981.1_601_TPlot.pdf]

**T=Lsat\_1\_v5\_gn\_8\_121820.1\_Q=Lsa-miR164b\_S=665**

category=2\_p=0.252073469146249

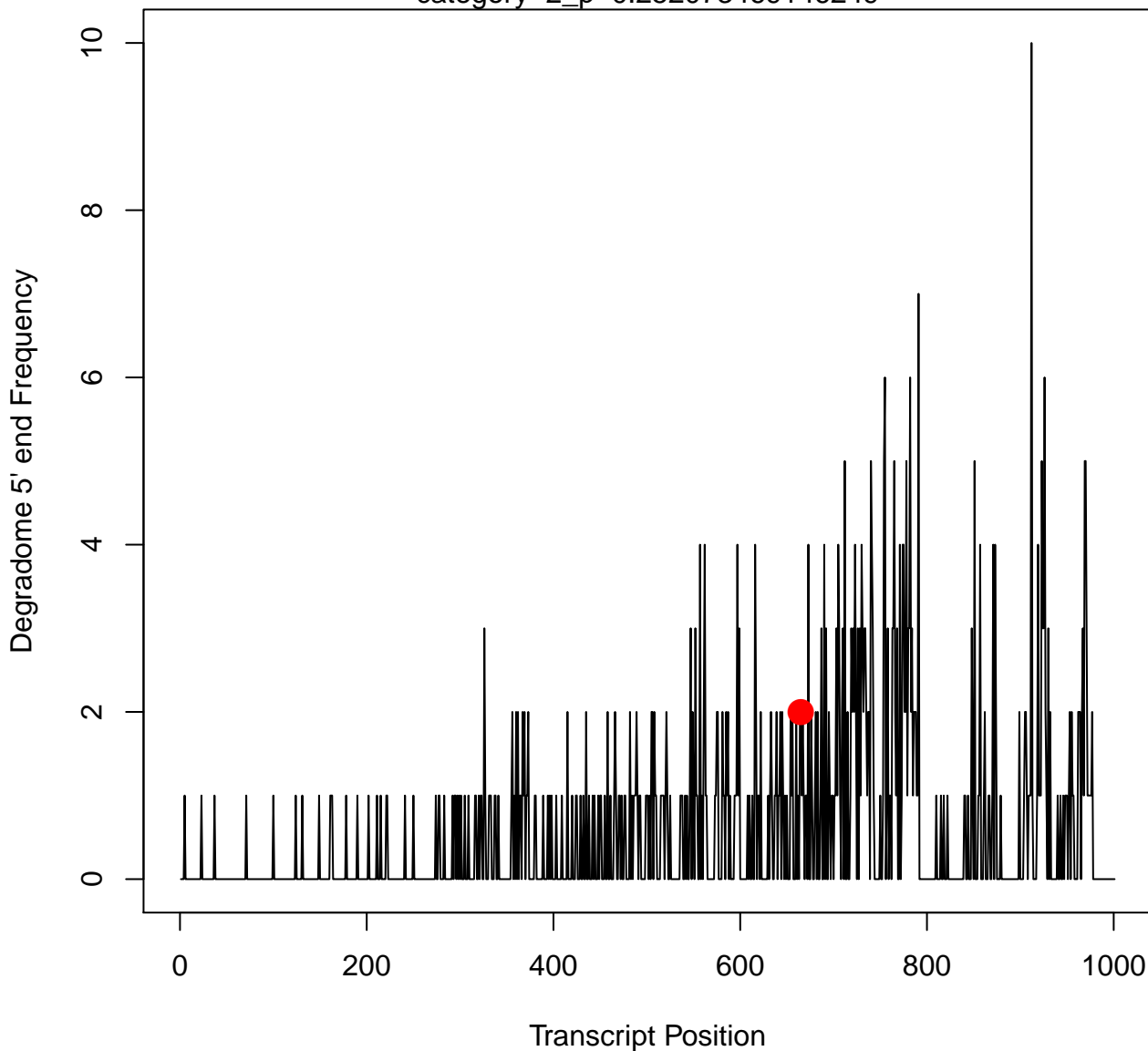

Supplement: Supplementary Data 1 — Results of categories 0–2 from PARE-Seq analysis (including three subfiles:1_1, 1_2, 1_3). [file Data_Sheet_10.ZIP › GSM2230754.plot/Lsa-miR164b_Lsat_1_v5_gn_8_121820.1_665_TPlot.pdf]

**T=Lsat\_1\_v5\_gn\_4\_169300.1\_Q=Lsa-miR164c\_S=643**

category=0\_p=0.00225258031608389

Degradome 5' end Frequency

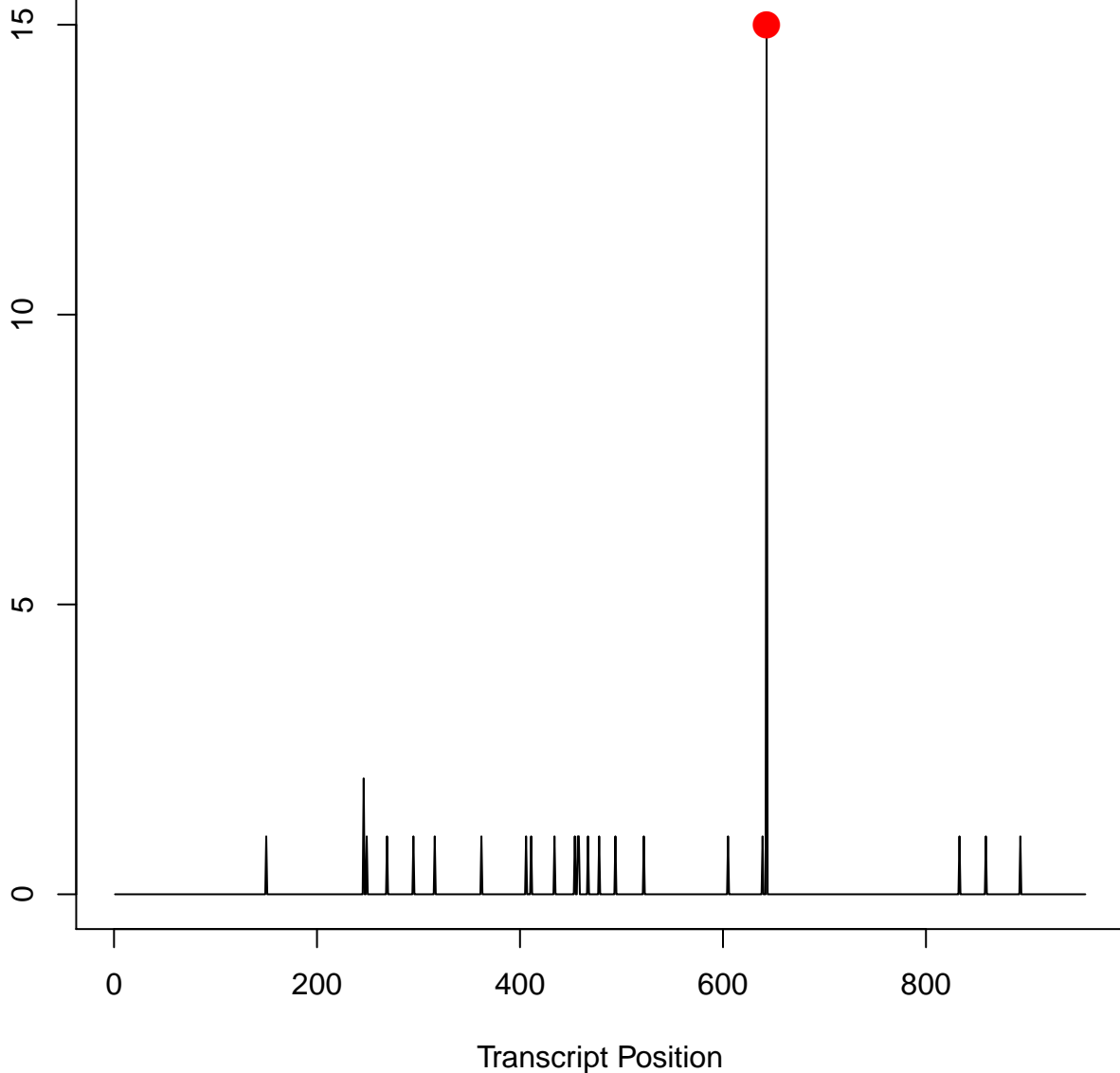

Supplement: Supplementary Data 1 — Results of categories 0–2 from PARE-Seq analysis (including three subfiles:1_1, 1_2, 1_3). [file Data_Sheet_10.ZIP › GSM2230754.plot/Lsa-miR164c_Lsat_1_v5_gn_4_169300.1_643_TPlot.pdf]

**T=Lsat\_1\_v5\_gn\_4\_62541.1\_Q=Lsa-miR164c\_S=396**

category=2\_p=0.543522752723408

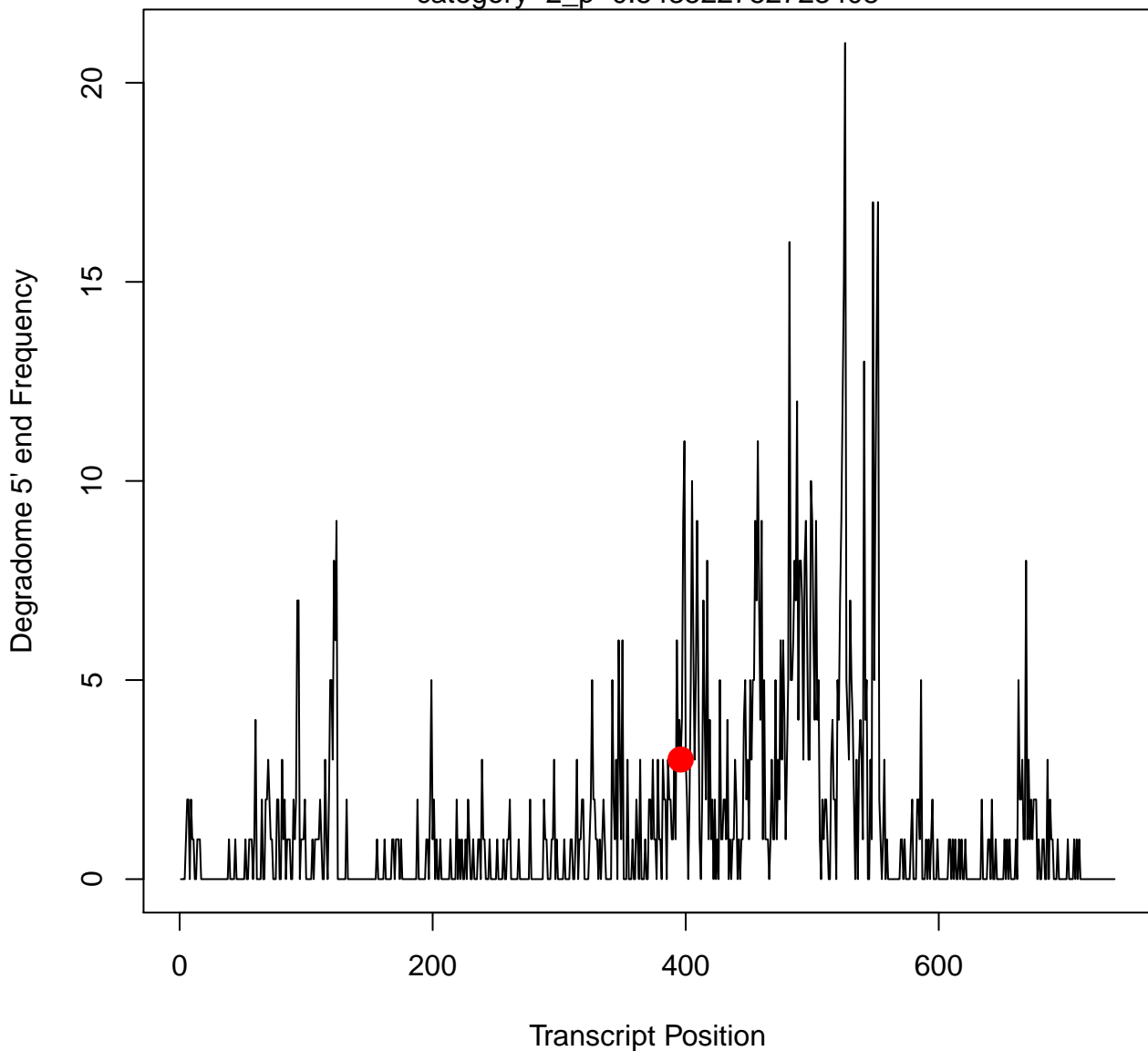

Supplement: Supplementary Data 1 — Results of categories 0–2 from PARE-Seq analysis (including three subfiles:1_1, 1_2, 1_3). [file Data_Sheet_10.ZIP › GSM2230754.plot/Lsa-miR164c_Lsat_1_v5_gn_4_62541.1_396_TPlot.pdf]

**T=Lsat\_1\_v5\_gn\_5\_188301.1\_Q=Lsa-miR164c\_S=1875**

category=2\_p=0.872825960256135

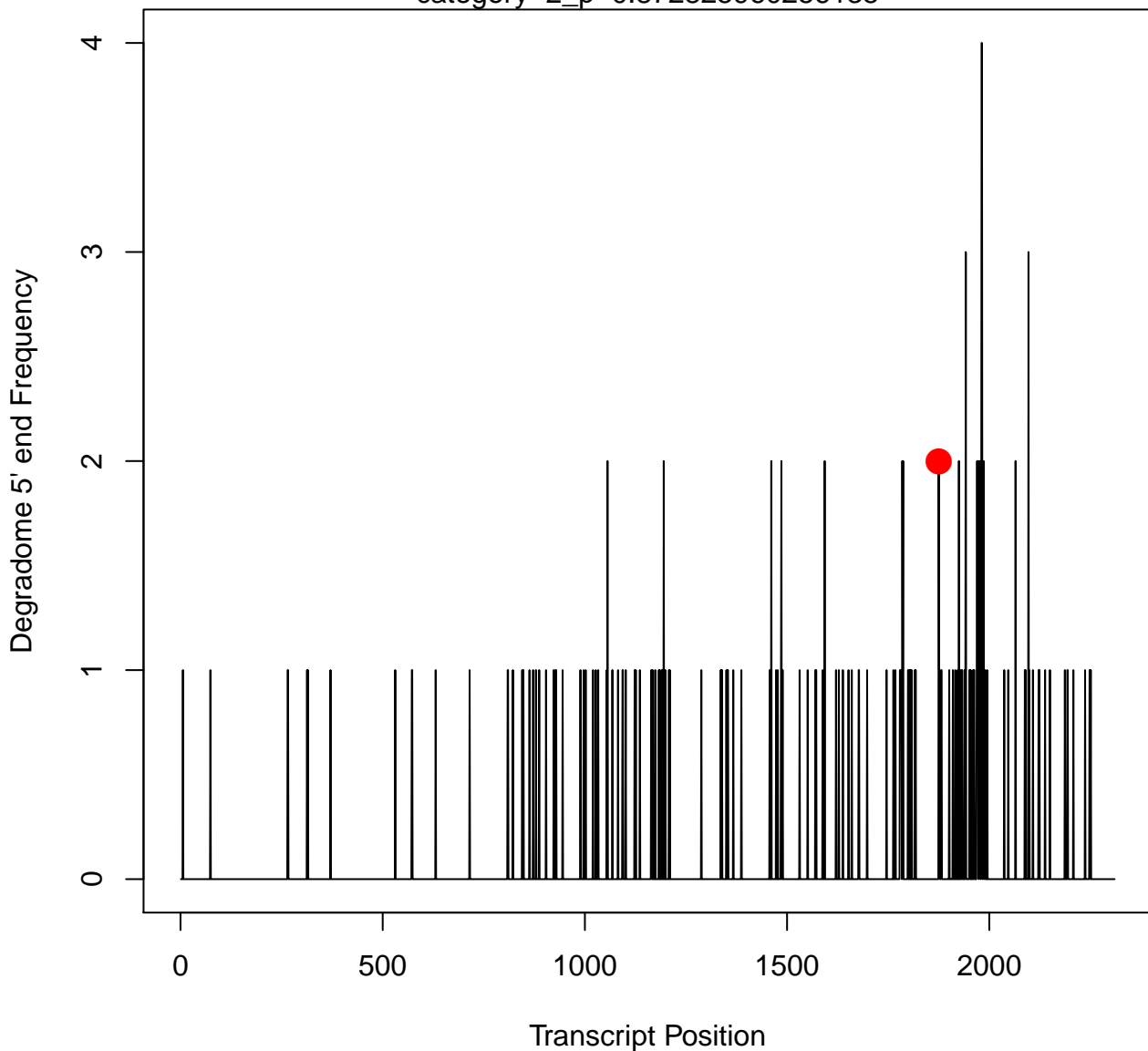

Supplement: Supplementary Data 1 — Results of categories 0–2 from PARE-Seq analysis (including three subfiles:1_1, 1_2, 1_3). [file Data_Sheet_10.ZIP › GSM2230754.plot/Lsa-miR164c_Lsat_1_v5_gn_5_188301.1_1875_TPlot.pdf]

**T=Lsat\_1\_v5\_gn\_7\_104460.1\_Q=Lsa-miR164c\_S=727**

category=0\_p=0.00037578290783058

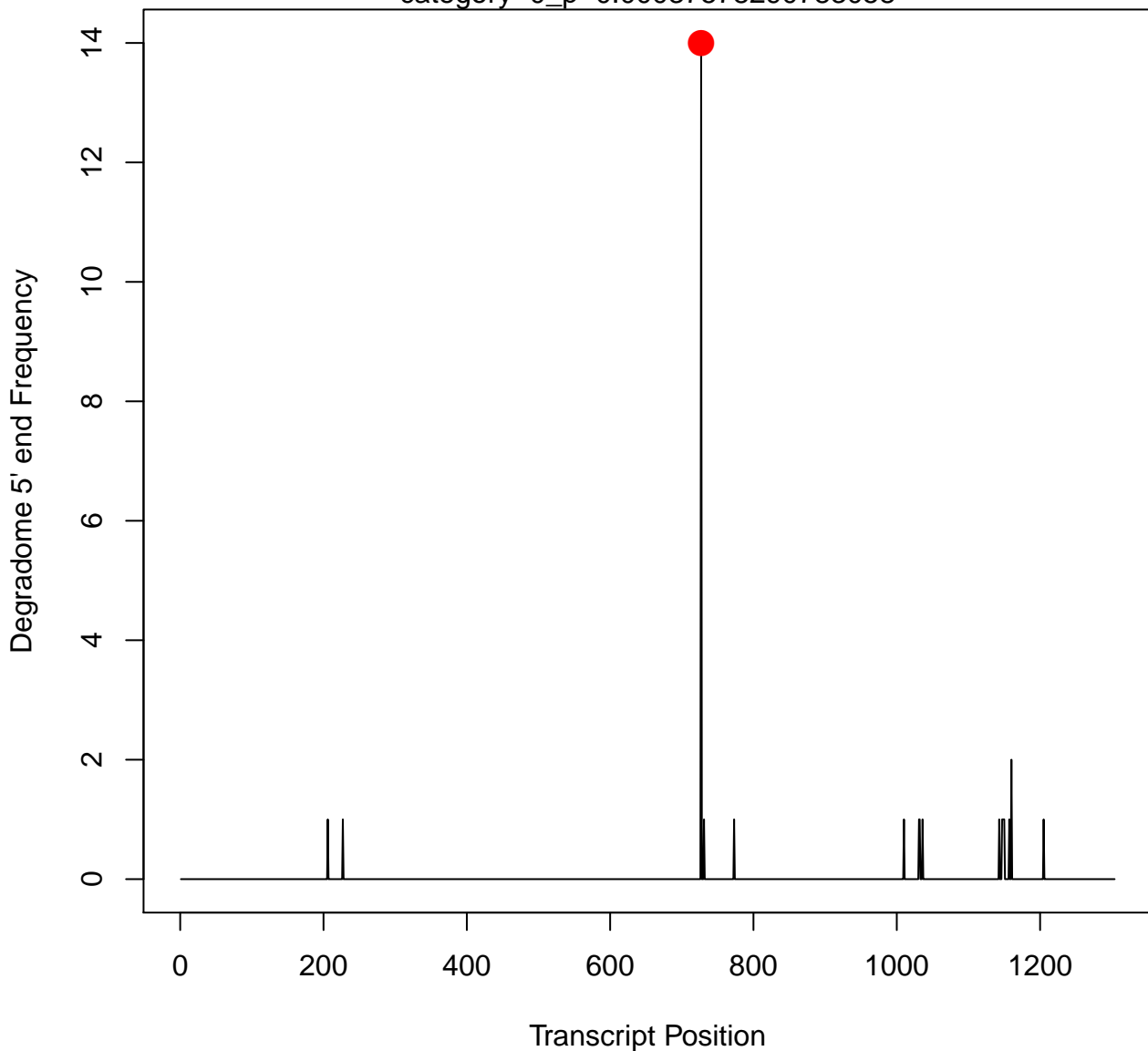

Supplement: Supplementary Data 1 — Results of categories 0–2 from PARE-Seq analysis (including three subfiles:1_1, 1_2, 1_3). [file Data_Sheet_10.ZIP › GSM2230754.plot/Lsa-miR164c_Lsat_1_v5_gn_7_104460.1_727_TPlot.pdf]

**T=Lsat\_1\_v5\_gn\_4\_177820.1\_Q=Lsa-miR166a\_S=1350**

category=2\_p=0.648526828297634

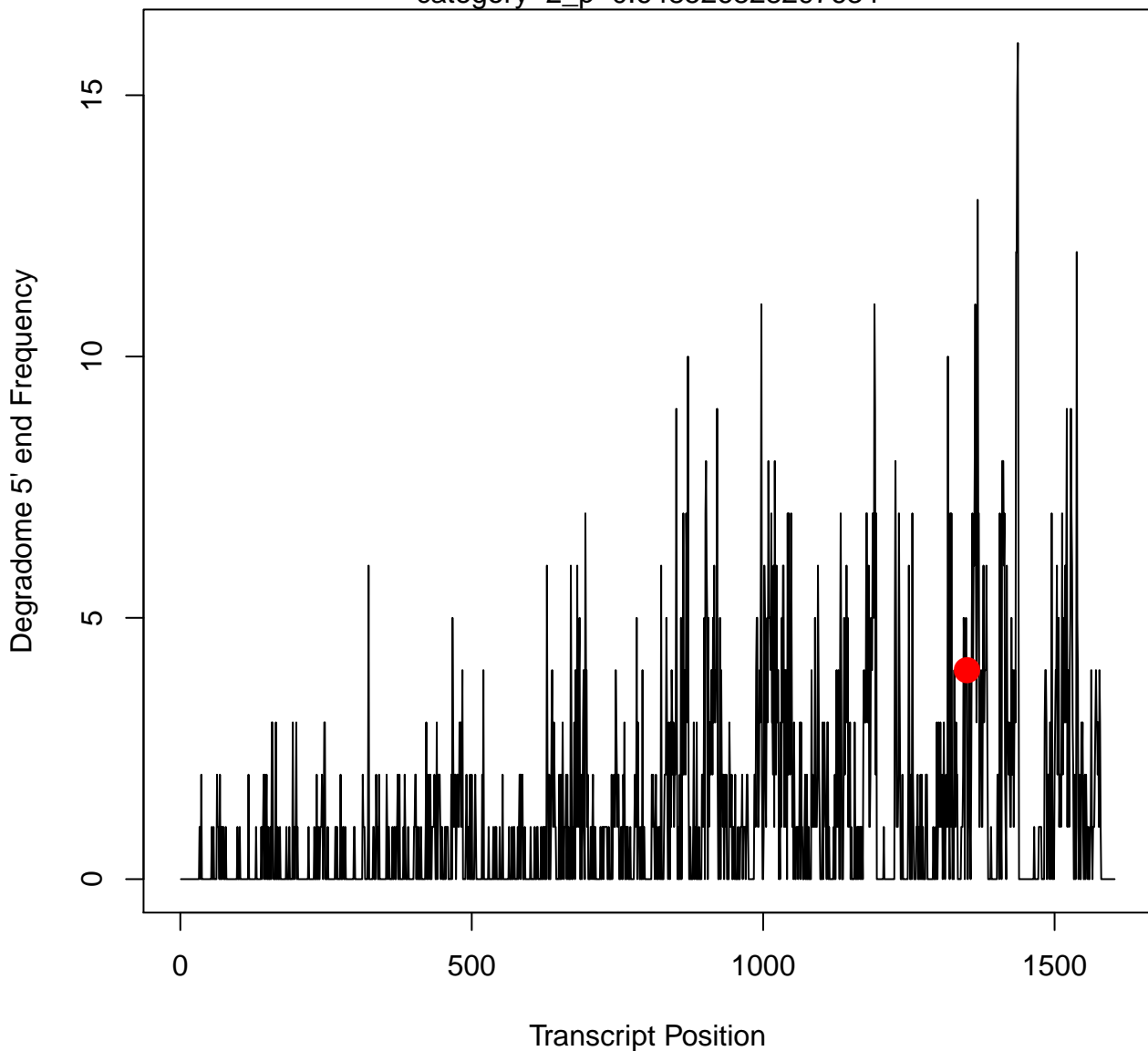

Supplement: Supplementary Data 1 — Results of categories 0–2 from PARE-Seq analysis (including three subfiles:1_1, 1_2, 1_3). [file Data_Sheet_10.ZIP › GSM2230754.plot/Lsa-miR166a_Lsat_1_v5_gn_4_177820.1_1350_TPlot.pdf]

**T=Lsat\_1\_v5\_gn\_5\_32500.1\_Q=Lsa-miR166a\_S=565**

category=0\_p=0.00150228456680068

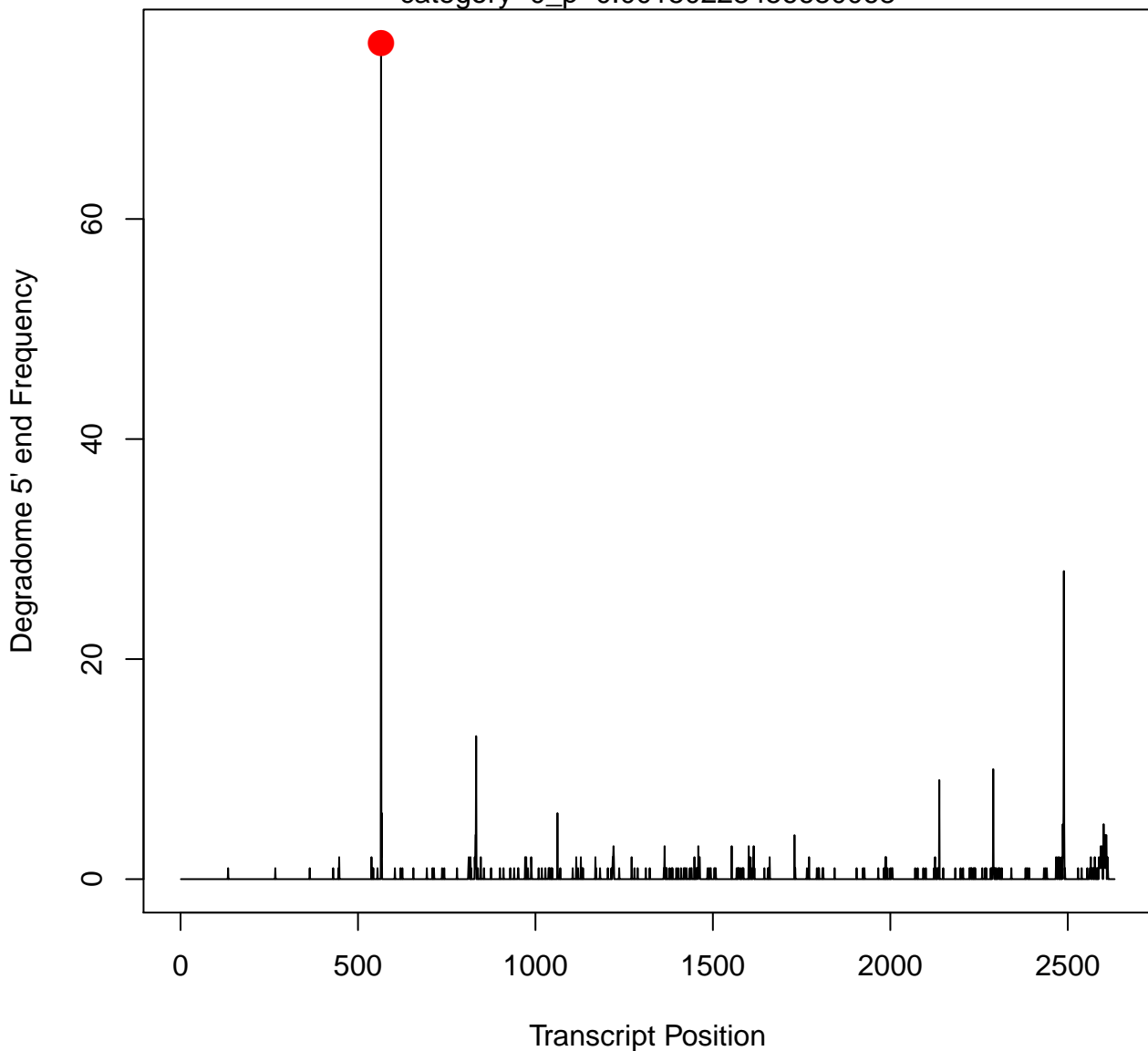

Supplement: Supplementary Data 1 — Results of categories 0–2 from PARE-Seq analysis (including three subfiles:1_1, 1_2, 1_3). [file Data_Sheet_10.ZIP › GSM2230754.plot/Lsa-miR166a_Lsat_1_v5_gn_5_32500.1_565_TPlot.pdf]

**T=Lsat\_1\_v5\_gn\_6\_45641.1\_Q=Lsa-miR166c\_S=1309**

category=0\_p=0.00150228456680068

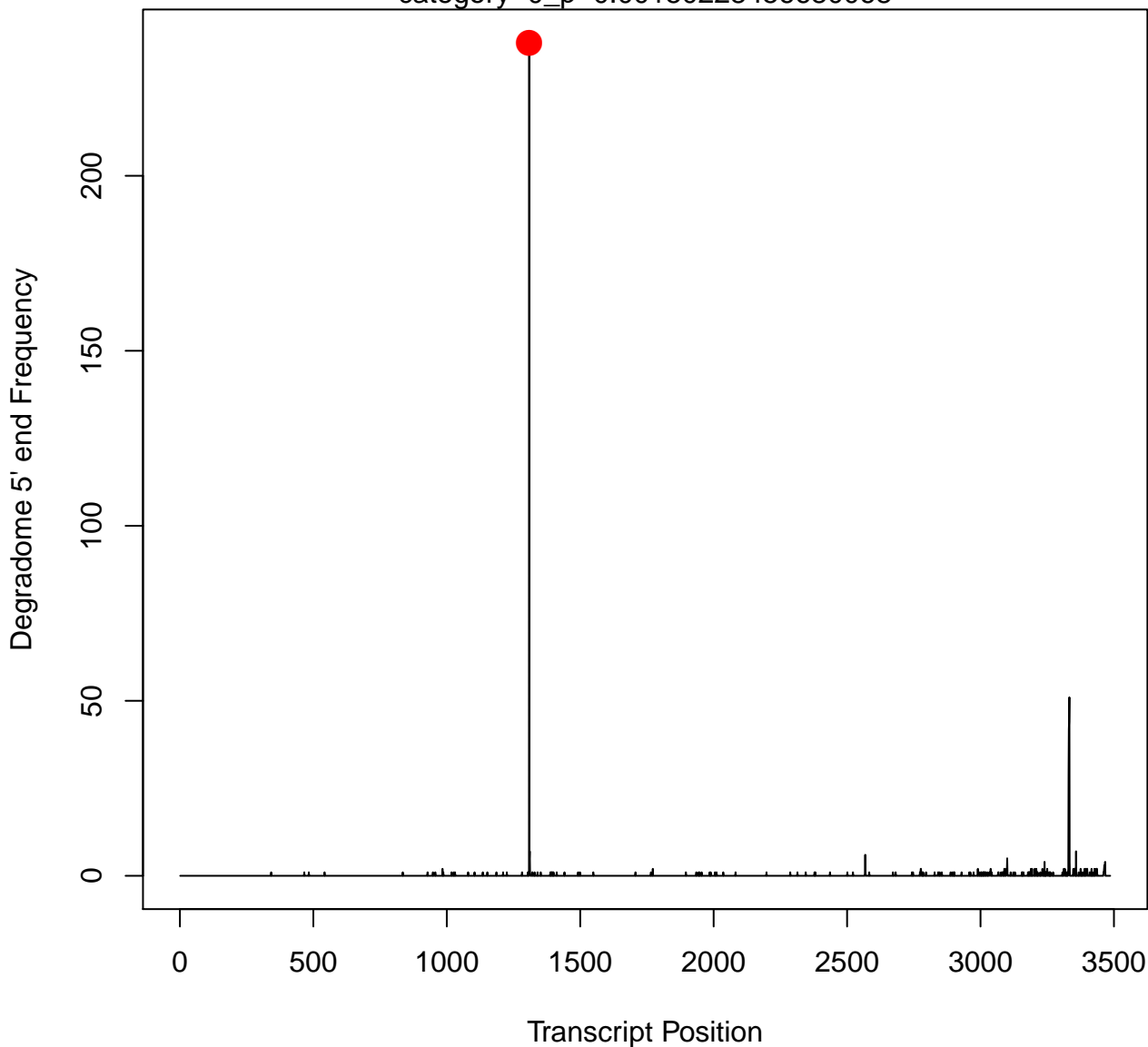

Supplement: Supplementary Data 1 — Results of categories 0–2 from PARE-Seq analysis (including three subfiles:1_1, 1_2, 1_3). [file Data_Sheet_10.ZIP › GSM2230754.plot/Lsa-miR166c_Lsat_1_v5_gn_6_45641.1_1309_TPlot.pdf]

**T=Lsat\_1\_v5\_gn\_2\_100140.1\_Q=Lsa-miR166d\_S=1400**

category=0\_p=0.00150228456680068

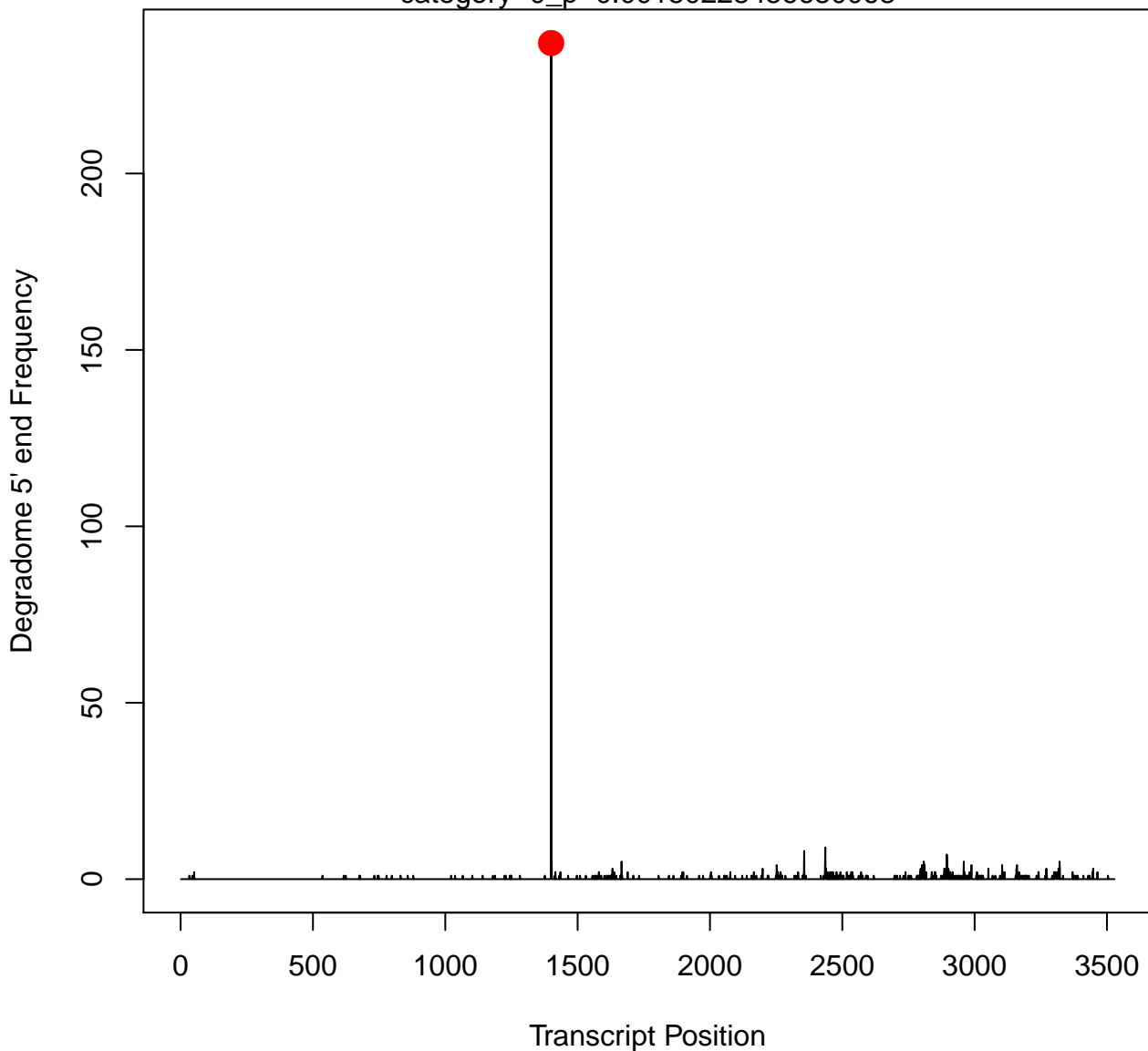

Supplement: Supplementary Data 1 — Results of categories 0–2 from PARE-Seq analysis (including three subfiles:1_1, 1_2, 1_3). [file Data_Sheet_10.ZIP › GSM2230754.plot/Lsa-miR166d_Lsat_1_v5_gn_2_100140.1_1400_TPlot.pdf]

**T=Lsat\_1\_v5\_gn\_6\_22720.1\_Q=Lsa-miR166e\_S=565**

category=0\_p=0.00150228456680068

Degradome 5' end Frequency

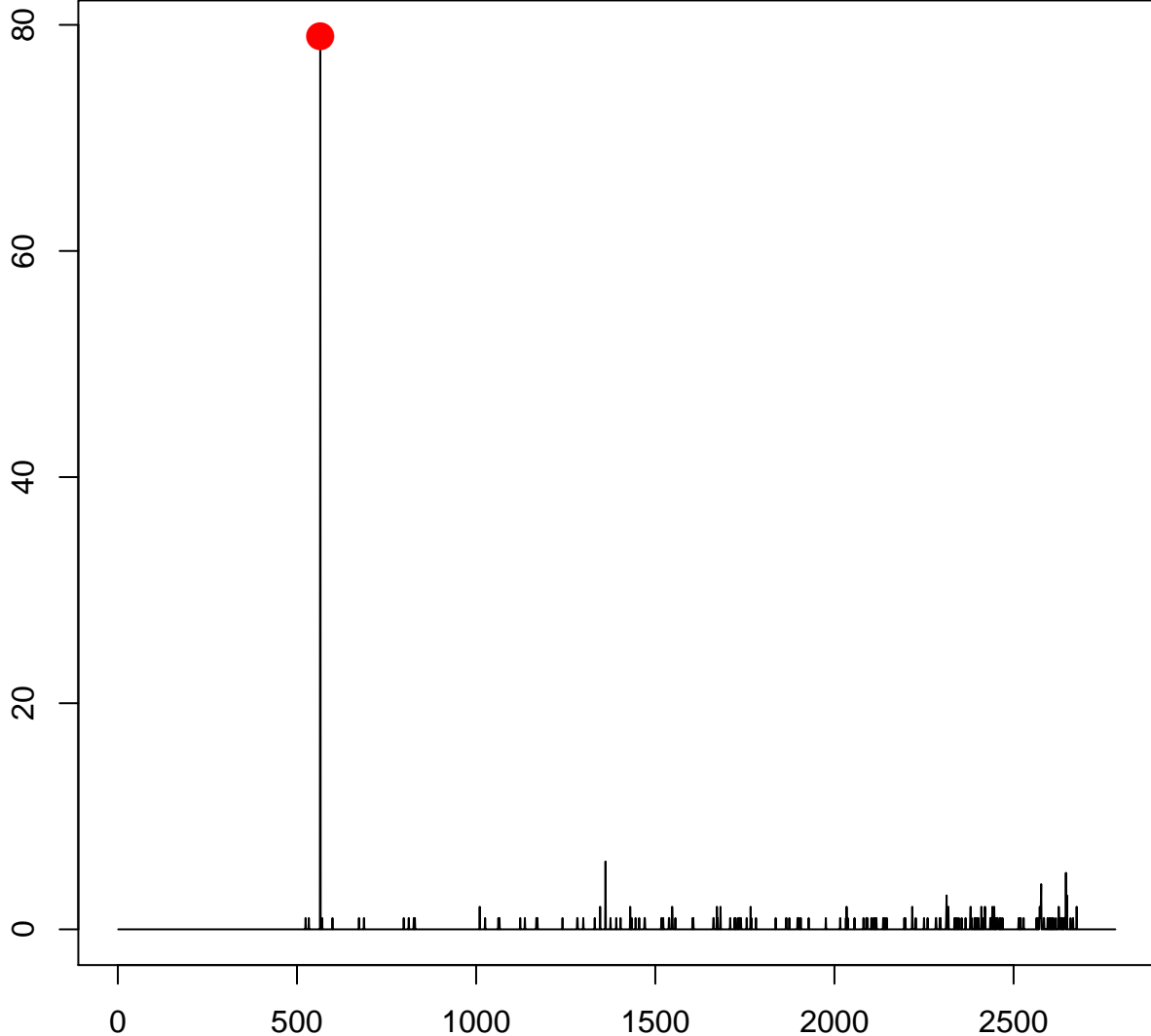

Transcript Position

Supplement: Supplementary Data 1 — Results of categories 0–2 from PARE-Seq analysis (including three subfiles:1_1, 1_2, 1_3). [file Data_Sheet_10.ZIP › GSM2230754.plot/Lsa-miR166e_Lsat_1_v5_gn_6_22720.1_565_TPlot.pdf]

**T=Lsat\_1\_v5\_gn\_9\_29020.1\_Q=Lsa-miR166f\_S=2923**

category=2\_p=0.721401142110361

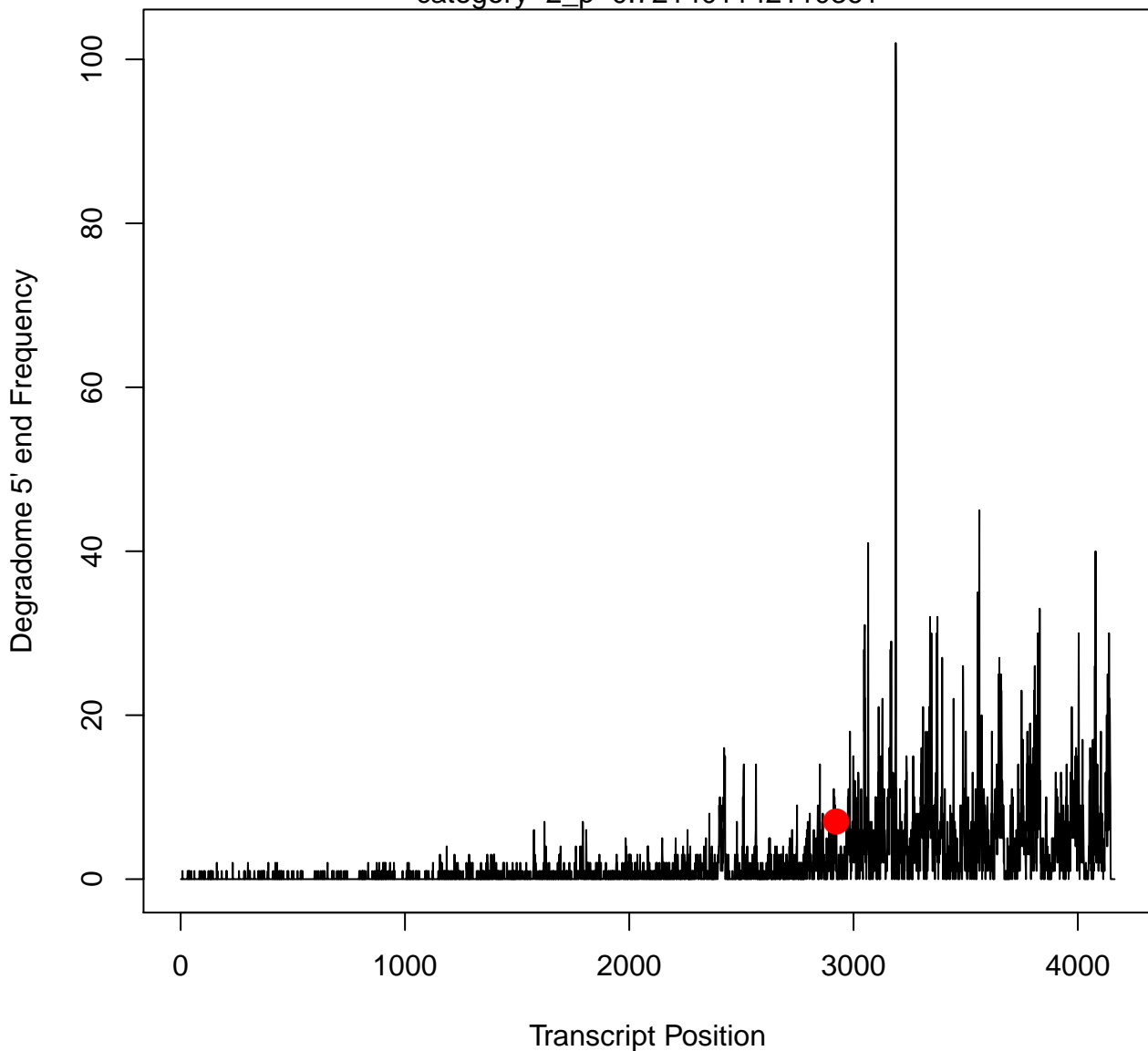

Supplement: Supplementary Data 1 — Results of categories 0–2 from PARE-Seq analysis (including three subfiles:1_1, 1_2, 1_3). [file Data_Sheet_10.ZIP › GSM2230754.plot/Lsa-miR166f_Lsat_1_v5_gn_9_29020.1_2923_TPlot.pdf]

**T=Lsat\_1\_v5\_gn\_9\_29121.1\_Q=Lsa-miR166f\_S=2923**

category=2\_p=0.729376675040129

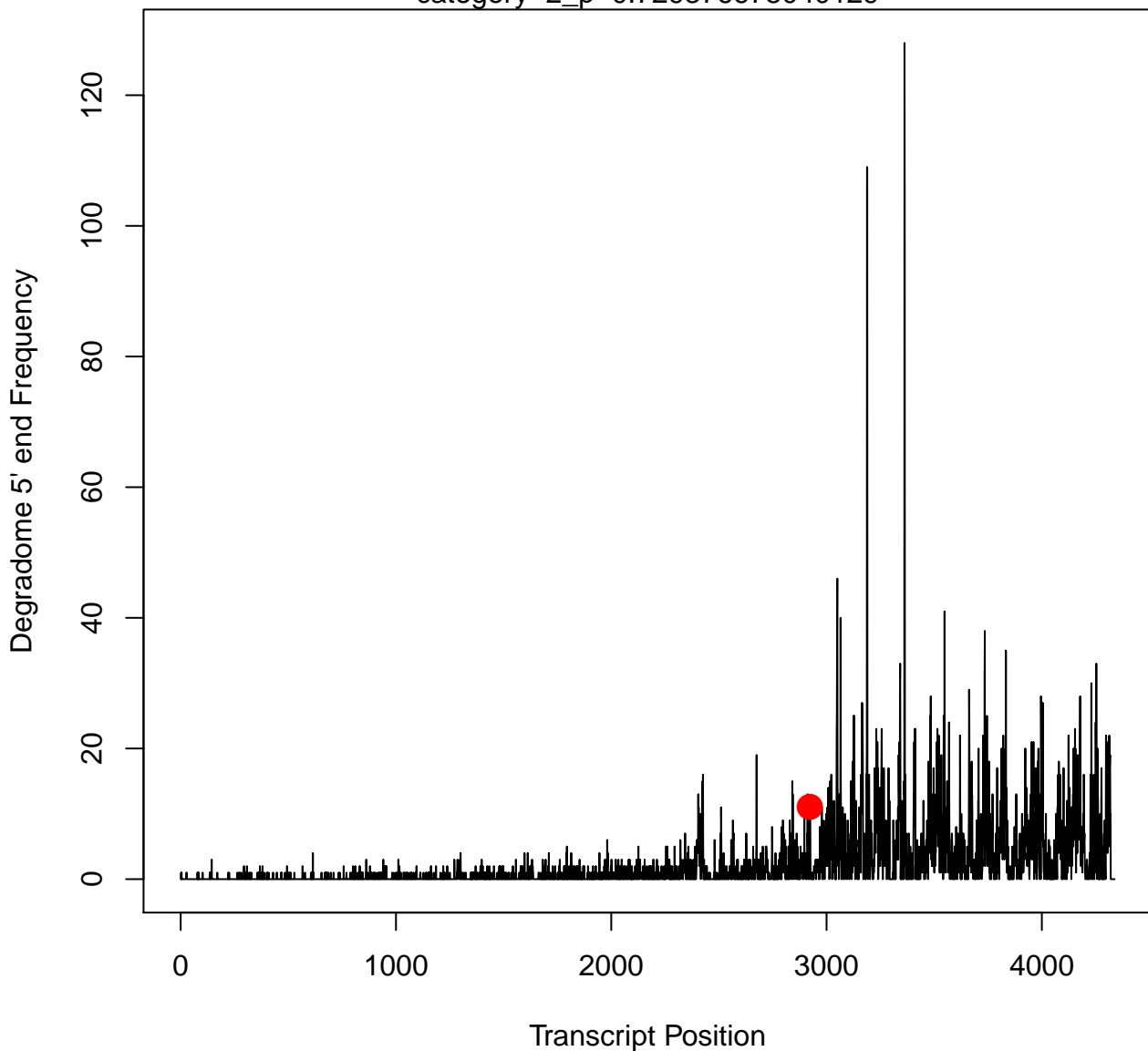

Supplement: Supplementary Data 1 — Results of categories 0–2 from PARE-Seq analysis (including three subfiles:1_1, 1_2, 1_3). [file Data_Sheet_10.ZIP › GSM2230754.plot/Lsa-miR166f_Lsat_1_v5_gn_9_29121.1_2923_TPlot.pdf]

**T=Lsat\_1\_v5\_gn\_3\_10861.1\_Q=Lsa-miR166g\_S=514**

category=2\_p=0.982859434487274

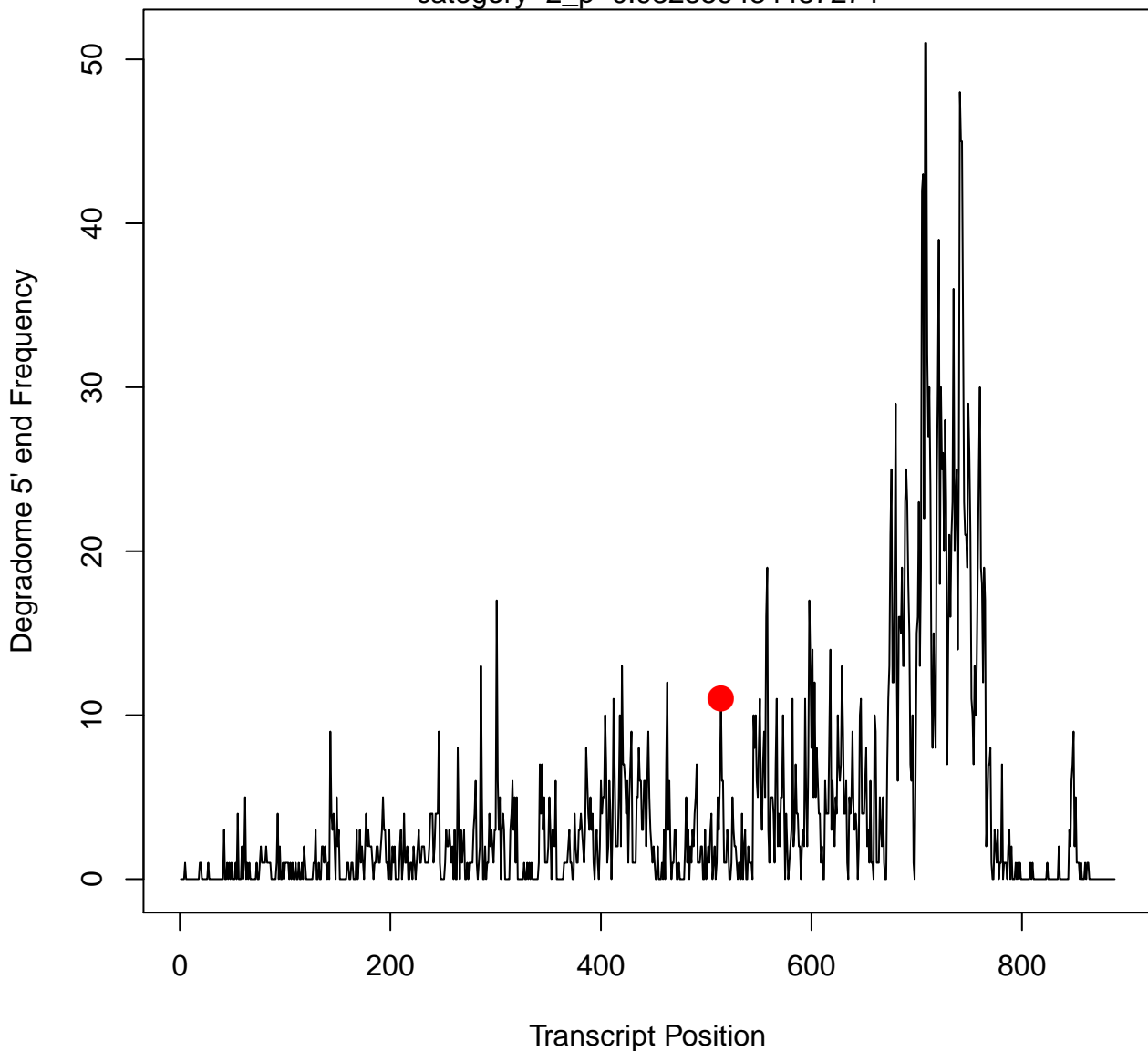

Supplement: Supplementary Data 1 — Results of categories 0–2 from PARE-Seq analysis (including three subfiles:1_1, 1_2, 1_3). [file Data_Sheet_10.ZIP › GSM2230754.plot/Lsa-miR166g_Lsat_1_v5_gn_3_10861.1_514_TPlot.pdf]

**T=Lsat\_1\_v5\_gn\_4\_100080.1\_Q=Lsa-miR166g\_S=1067**

category=0\_p=0.000751424602867257

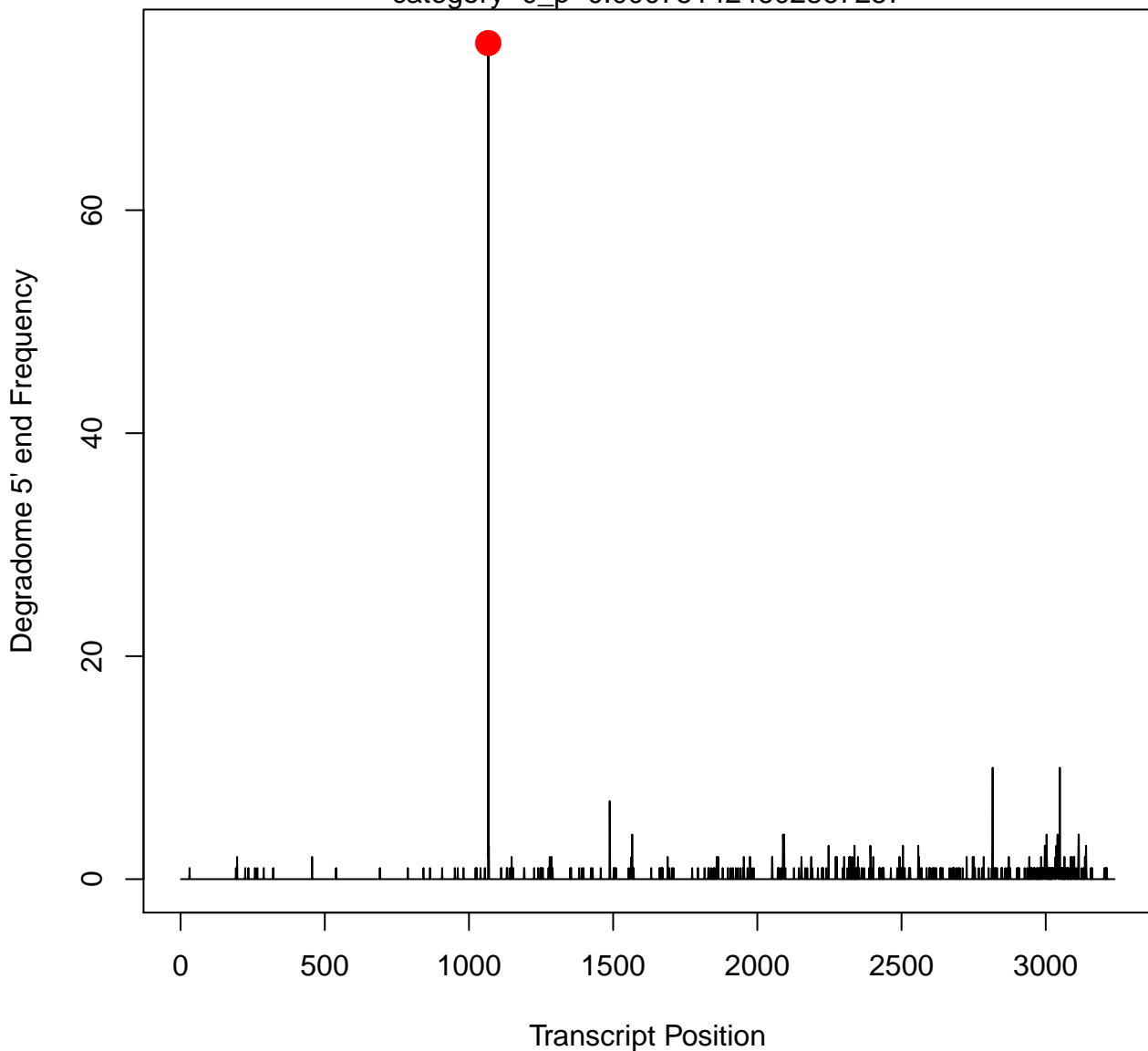

Supplement: Supplementary Data 1 — Results of categories 0–2 from PARE-Seq analysis (including three subfiles:1_1, 1_2, 1_3). [file Data_Sheet_10.ZIP › GSM2230754.plot/Lsa-miR166g_Lsat_1_v5_gn_4_100080.1_1067_TPlot.pdf]

**T=Lsat\_1\_v5\_gn\_5\_184280.1\_Q=Lsa-miR166g\_S=912**

category=2\_p=0.230031345922925

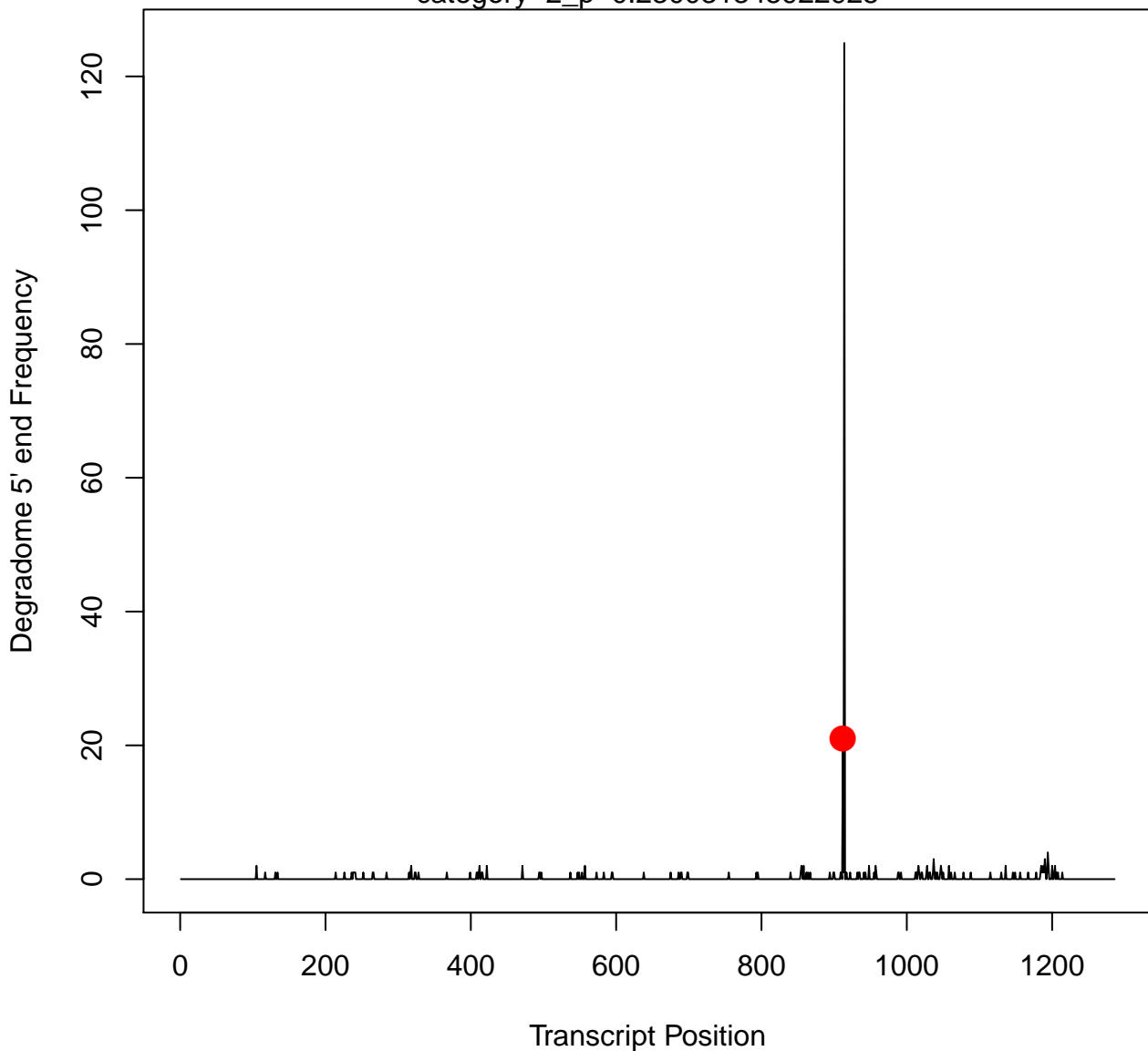

Supplement: Supplementary Data 1 — Results of categories 0–2 from PARE-Seq analysis (including three subfiles:1_1, 1_2, 1_3). [file Data_Sheet_10.ZIP › GSM2230754.plot/Lsa-miR166g_Lsat_1_v5_gn_5_184280.1_912_TPlot.pdf]

**T=Lsat\_1\_v5\_gn\_9\_89140.1\_Q=Lsa-miR166g\_S=1993**

category=2\_p=0.960204476402062

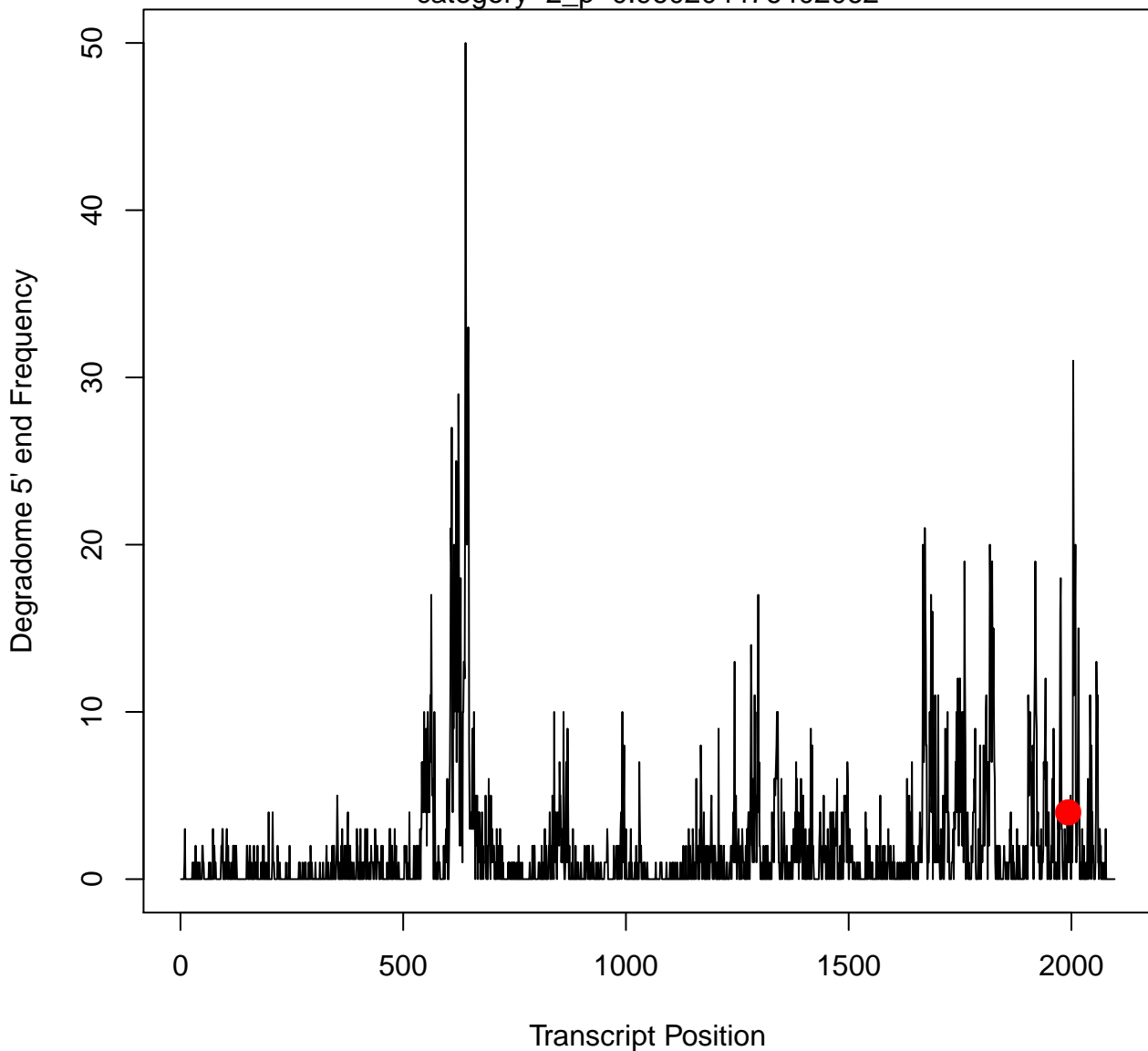

Supplement: Supplementary Data 1 — Results of categories 0–2 from PARE-Seq analysis (including three subfiles:1_1, 1_2, 1_3). [file Data_Sheet_10.ZIP › GSM2230754.plot/Lsa-miR166g_Lsat_1_v5_gn_9_89140.1_1993_TPlot.pdf]

**T=Lsat\_1\_v5\_gn\_1\_37081.1\_Q=Lsa-miR166h\_S=1030**

category=0\_p=0.00037578290783058

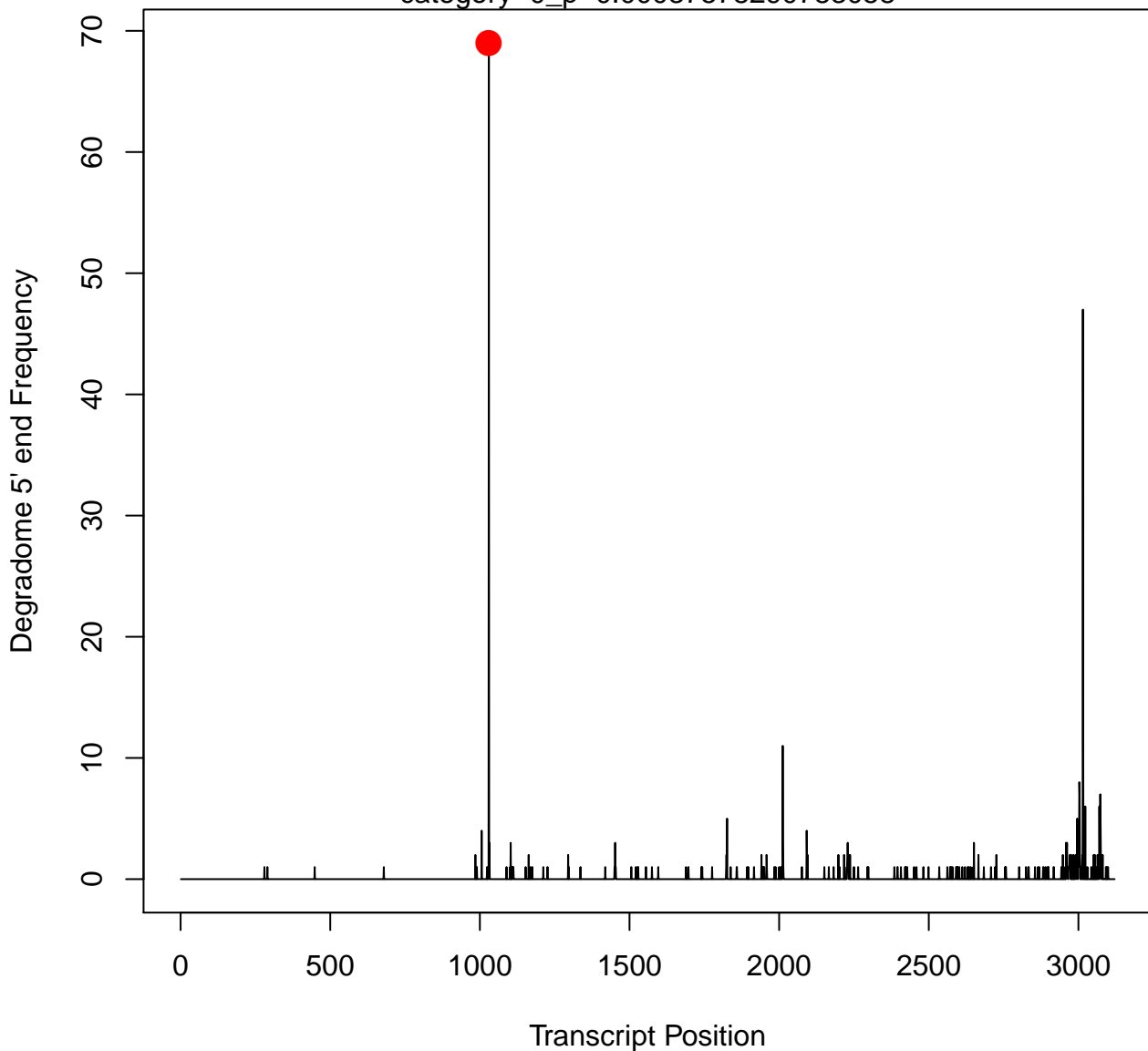

Supplement: Supplementary Data 1 — Results of categories 0–2 from PARE-Seq analysis (including three subfiles:1_1, 1_2, 1_3). [file Data_Sheet_10.ZIP › GSM2230754.plot/Lsa-miR166h_Lsat_1_v5_gn_1_37081.1_1030_TPlot.pdf]

**T=Lsat\_1\_v5\_gn\_2\_103140.1\_Q=Lsa-miR166h\_S=617**

category=2\_p=0.98074774653474

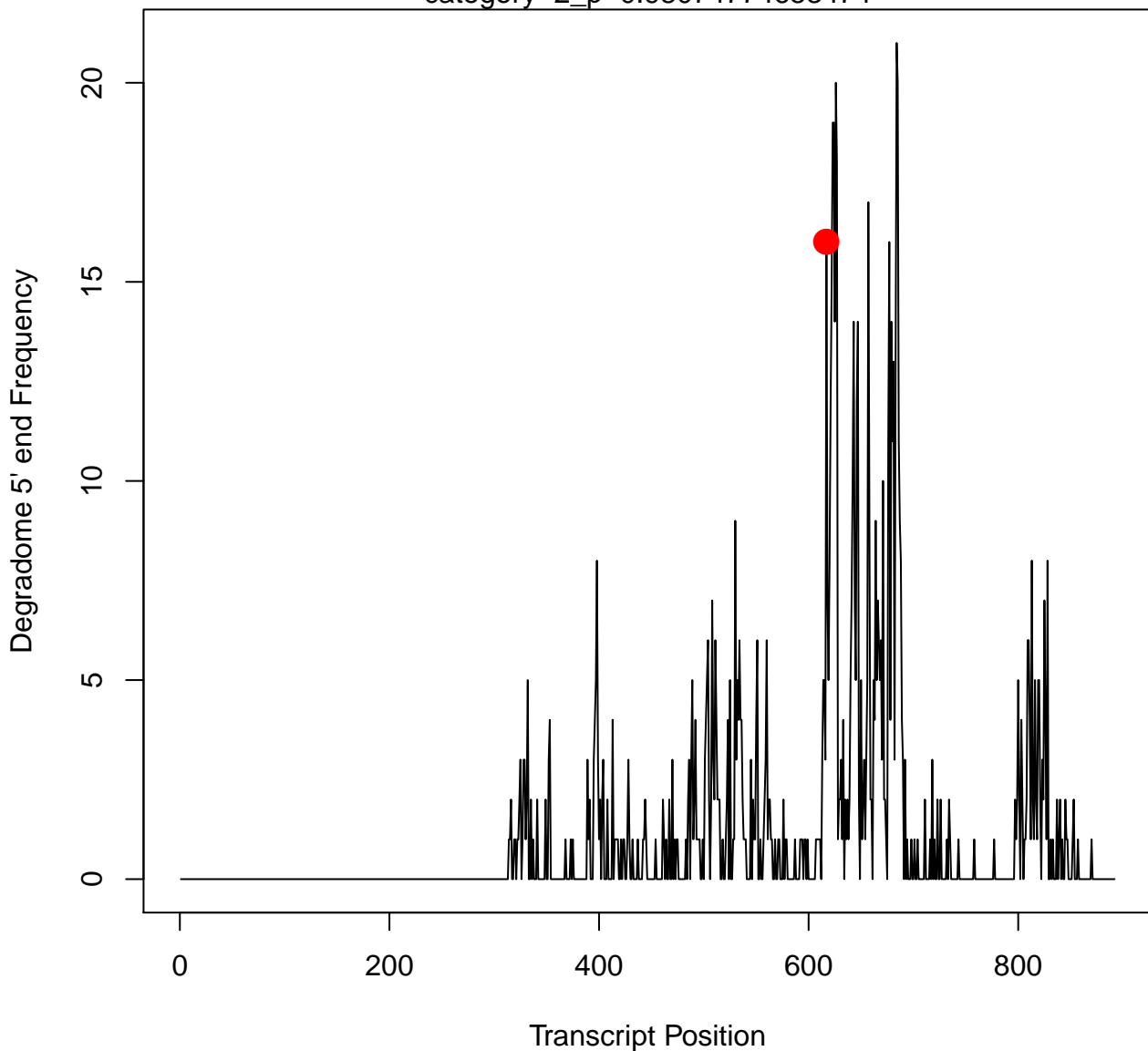

Supplement: Supplementary Data 1 — Results of categories 0–2 from PARE-Seq analysis (including three subfiles:1_1, 1_2, 1_3). [file Data_Sheet_10.ZIP › GSM2230754.plot/Lsa-miR166h_Lsat_1_v5_gn_2_103140.1_617_TPlot.pdf]

**T=Lsat\_1\_v5\_gn\_5\_105880.1\_Q=Lsa-miR166h\_S=1864**

category=2\_p=0.981298886559676

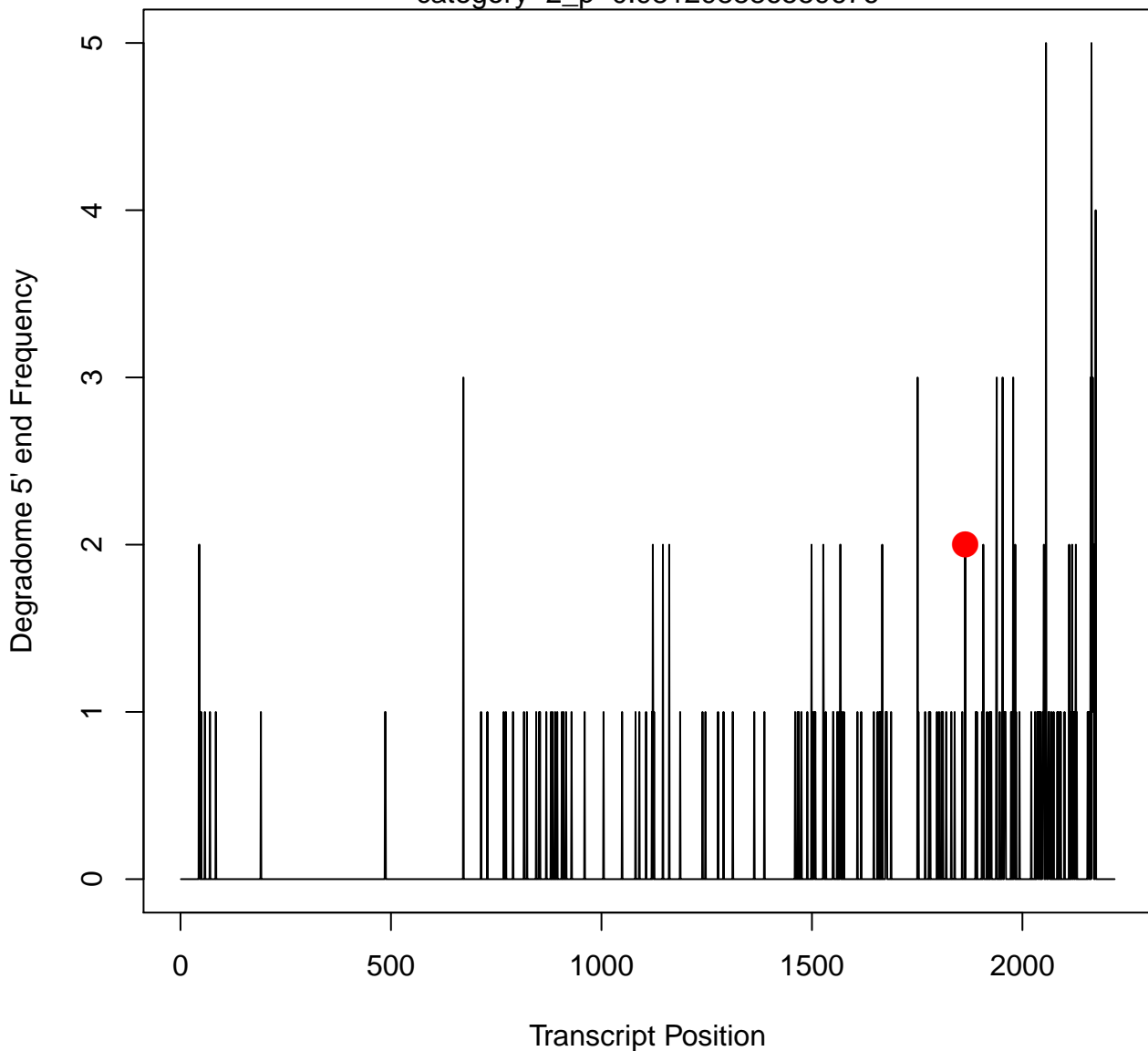

Supplement: Supplementary Data 1 — Results of categories 0–2 from PARE-Seq analysis (including three subfiles:1_1, 1_2, 1_3). [file Data_Sheet_10.ZIP › GSM2230754.plot/Lsa-miR166h_Lsat_1_v5_gn_5_105880.1_1864_TPlot.pdf]

**T=Lsat\_1\_v5\_gn\_5\_134441.1\_Q=Lsa-miR166h\_S=568**

category=0\_p=0.000751424602867257

Degradome 5' end Frequency

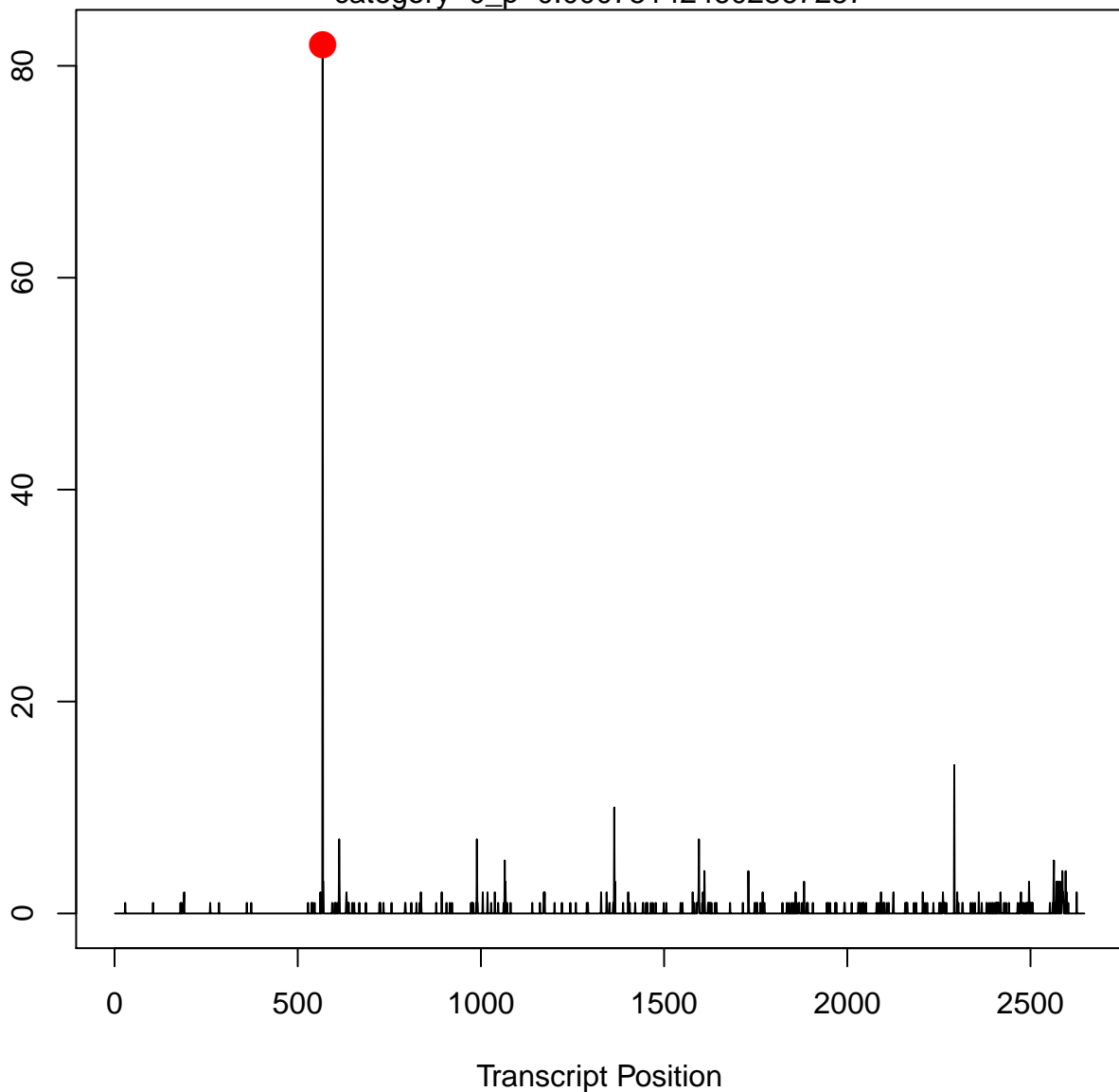

Supplement: Supplementary Data 1 — Results of categories 0–2 from PARE-Seq analysis (including three subfiles:1_1, 1_2, 1_3). [file Data_Sheet_10.ZIP › GSM2230754.plot/Lsa-miR166h_Lsat_1_v5_gn_5_134441.1_568_TPlot.pdf]

**T=Lsat\_1\_v5\_gn\_5\_44541.1\_Q=Lsa-miR166h\_S=515**

category=2\_p=0.934796031816062

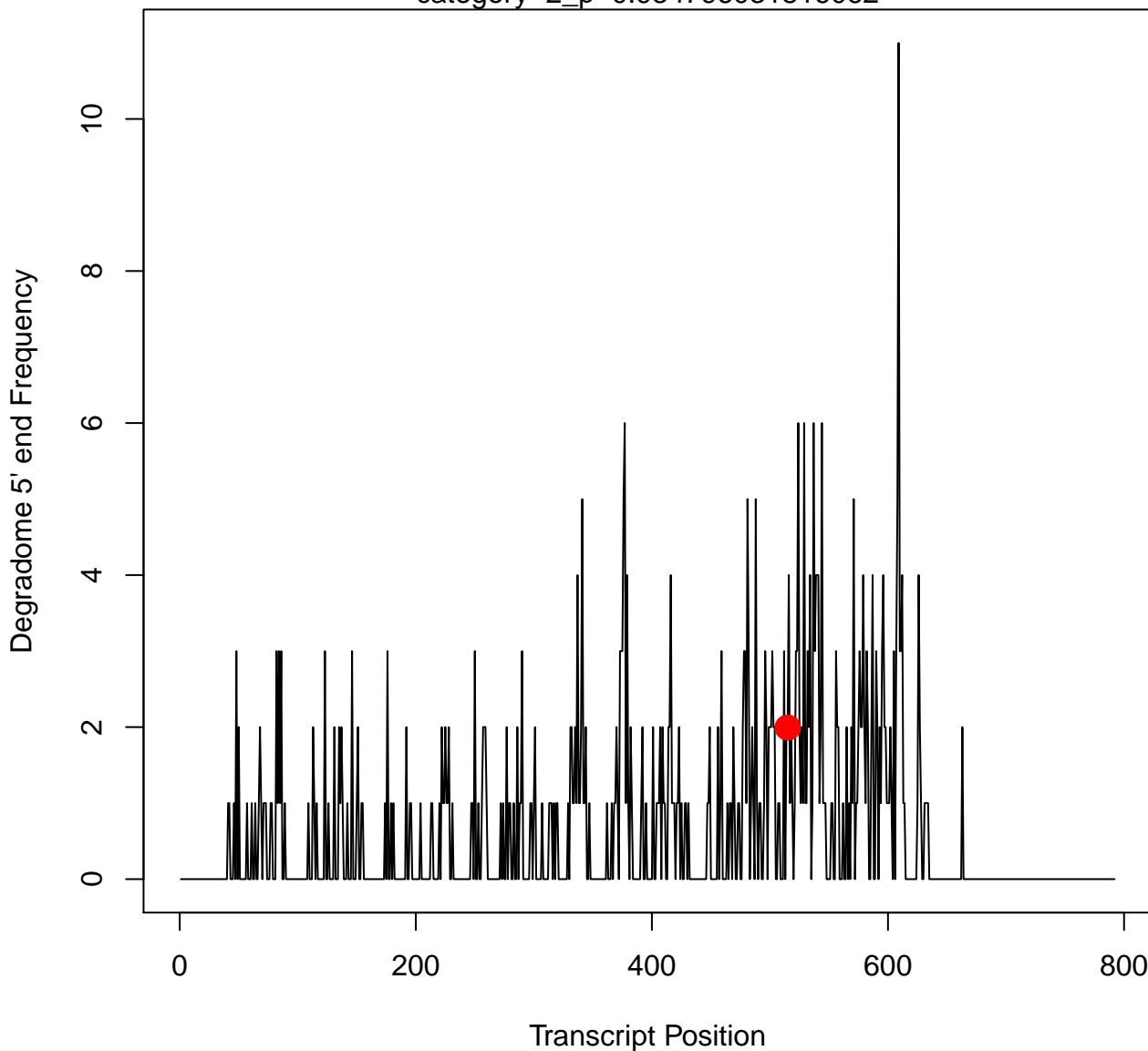

Supplement: Supplementary Data 1 — Results of categories 0–2 from PARE-Seq analysis (including three subfiles:1_1, 1_2, 1_3). [file Data_Sheet_10.ZIP › GSM2230754.plot/Lsa-miR166h_Lsat_1_v5_gn_5_44541.1_515_TPlot.pdf]

**T=Lsat\_1\_v5\_gn\_5\_84680.1\_Q=Lsa-miR166h\_S=2194**

category=2\_p=0.973500557466751

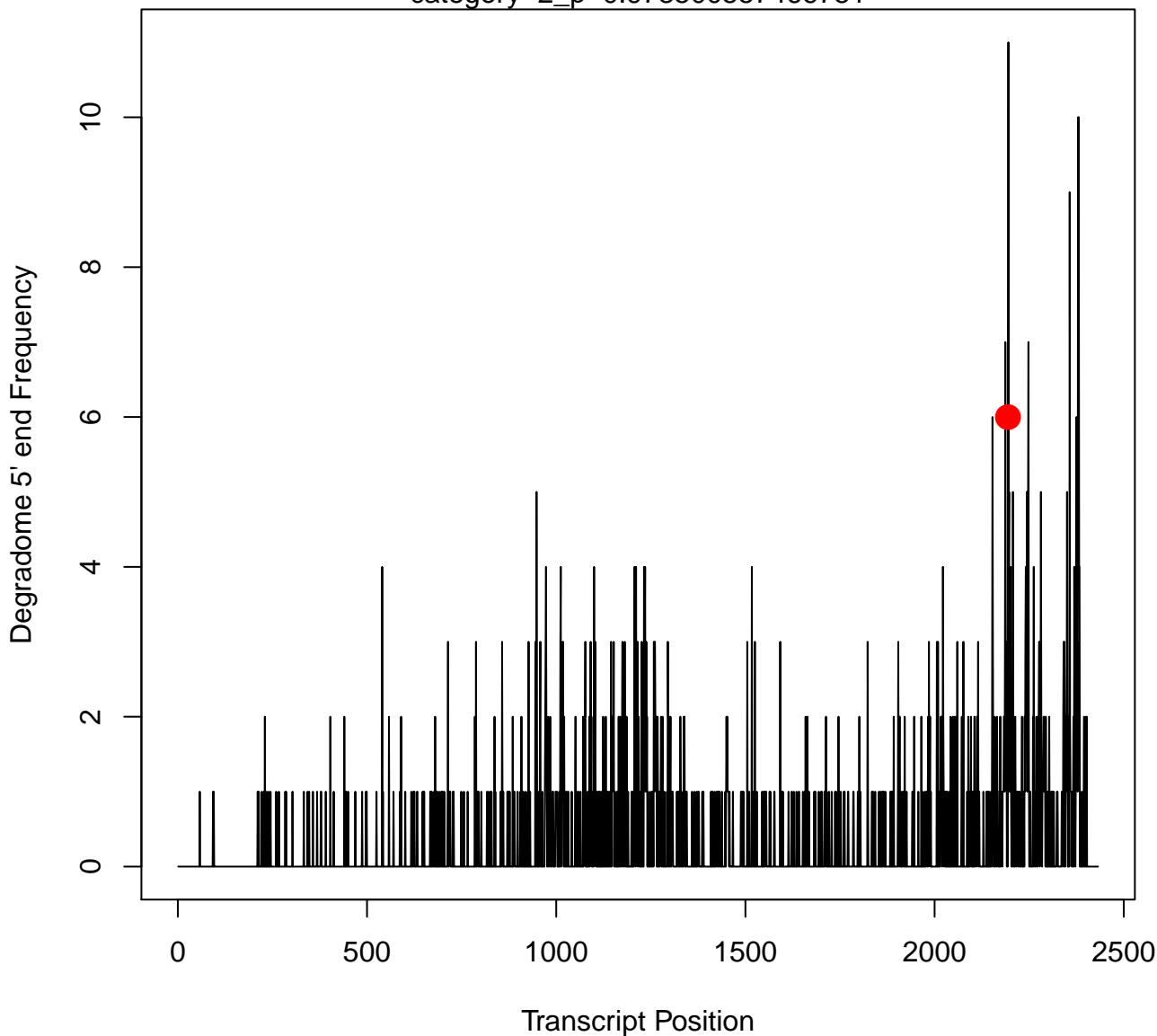

Supplement: Supplementary Data 1 — Results of categories 0–2 from PARE-Seq analysis (including three subfiles:1_1, 1_2, 1_3). [file Data_Sheet_10.ZIP › GSM2230754.plot/Lsa-miR166h_Lsat_1_v5_gn_5_84680.1_2194_TPlot.pdf]

**T=Lsat\_1\_v5\_gn\_8\_60221.1\_Q=Lsa-miR166h\_S=2203**

category=2\_p=0.955301736893021

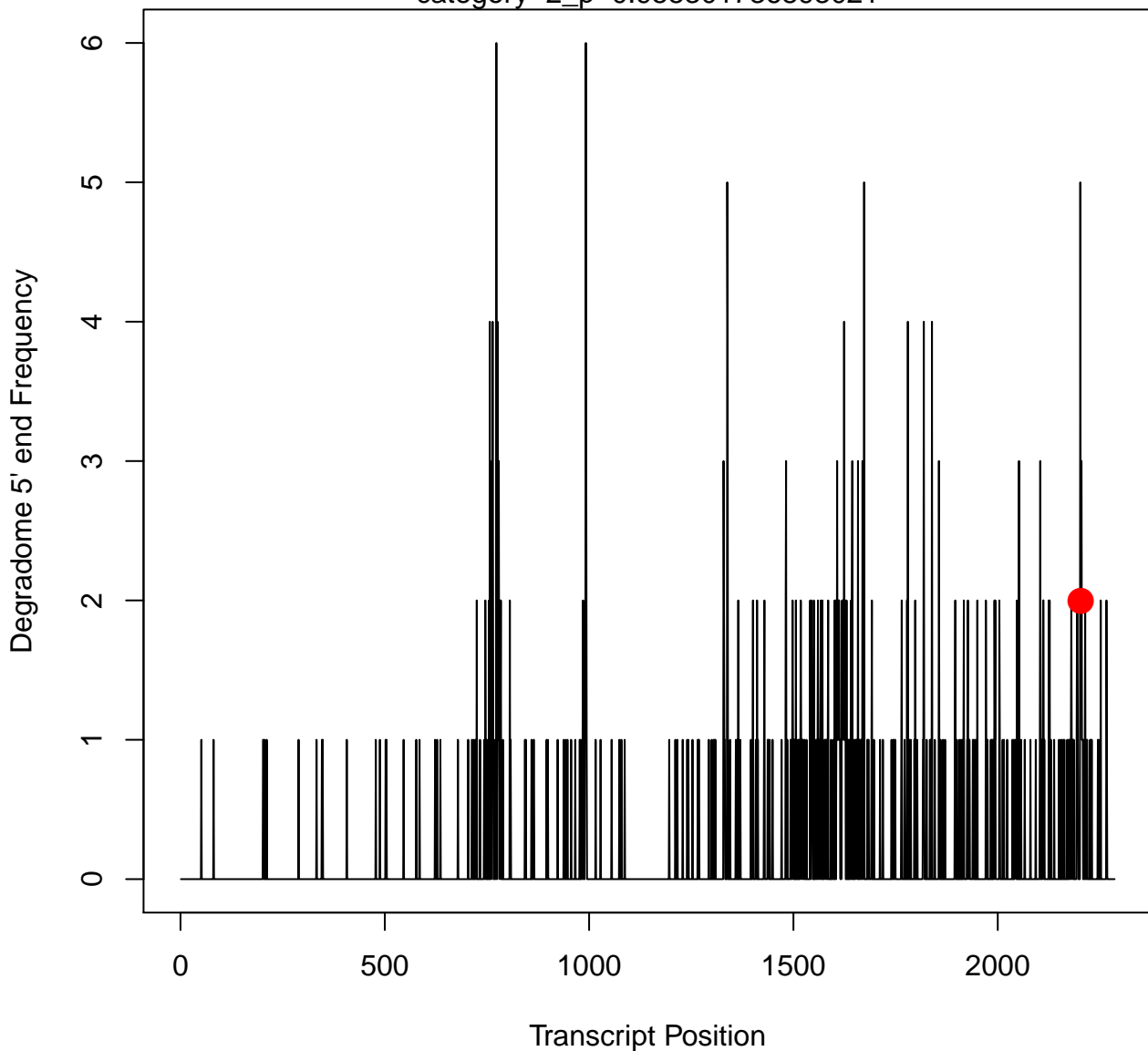

Supplement: Supplementary Data 1 — Results of categories 0–2 from PARE-Seq analysis (including three subfiles:1_1, 1_2, 1_3). [file Data_Sheet_10.ZIP › GSM2230754.plot/Lsa-miR166h_Lsat_1_v5_gn_8_60221.1_2203_TPlot.pdf]

**T=Lsat\_1\_v5\_gn\_8\_97261.1\_Q=Lsa-miR166h\_S=54**

category=2\_p=0.779165723399398

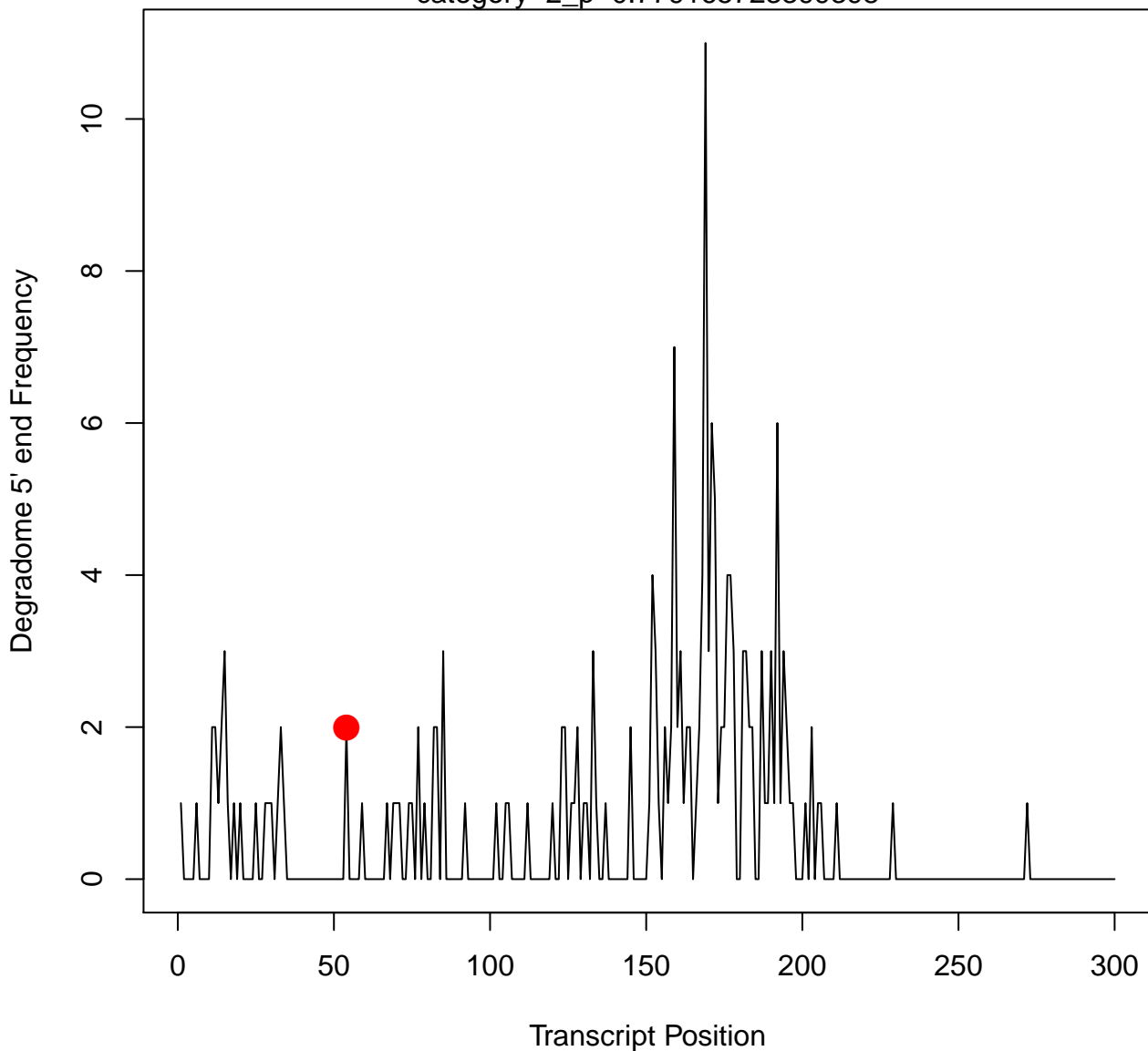

Supplement: Supplementary Data 1 — Results of categories 0–2 from PARE-Seq analysis (including three subfiles:1_1, 1_2, 1_3). [file Data_Sheet_10.ZIP › GSM2230754.plot/Lsa-miR166h_Lsat_1_v5_gn_8_97261.1_54_TPlot.pdf]

**T=Lsat\_1\_v5\_gn\_1\_37081.1\_Q=Lsa-miR166i\_S=1032**

category=2\_p=0.0286272994447346

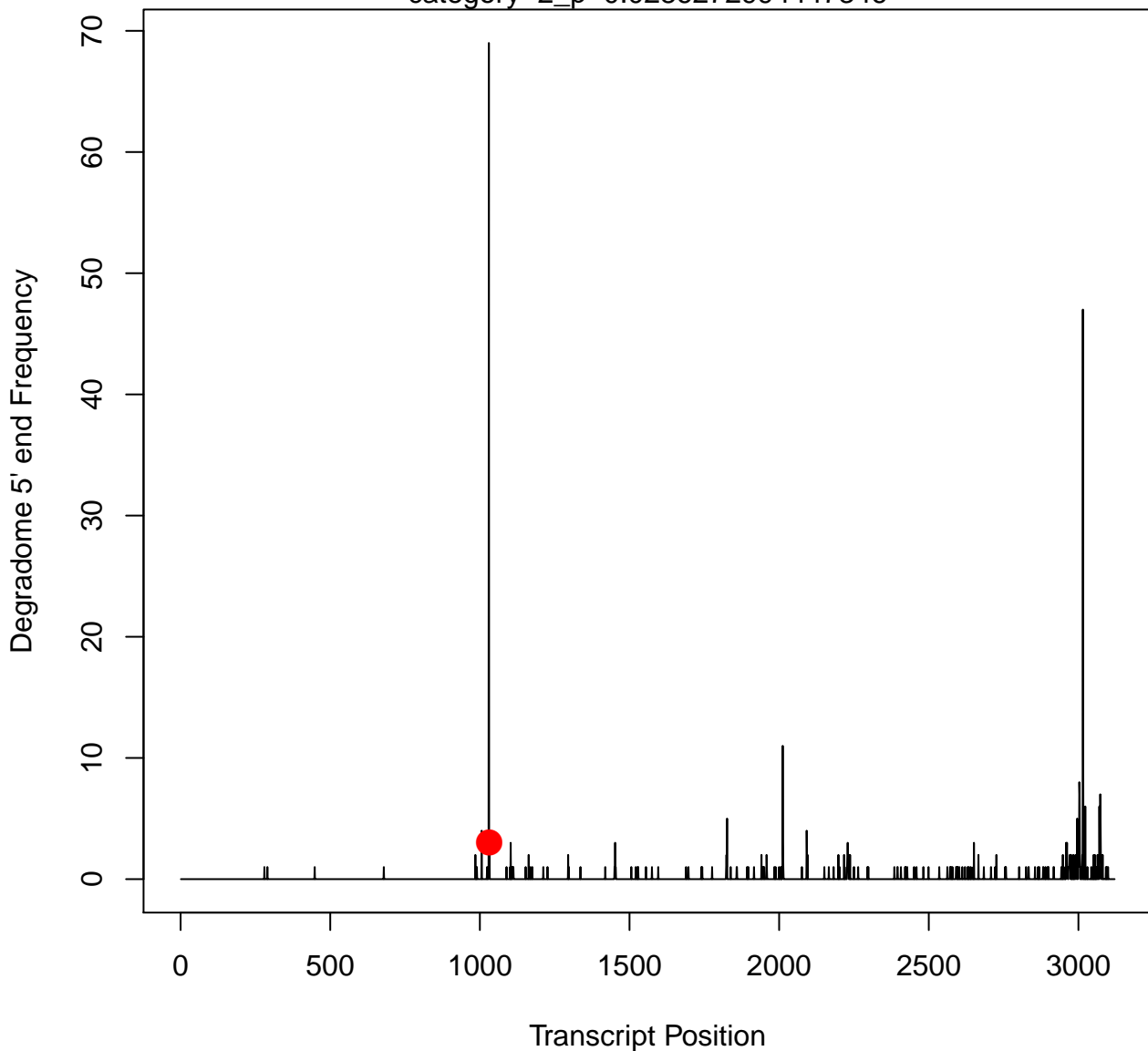

Supplement: Supplementary Data 1 — Results of categories 0–2 from PARE-Seq analysis (including three subfiles:1_1, 1_2, 1_3). [file Data_Sheet_10.ZIP › GSM2230754.plot/Lsa-miR166i_Lsat_1_v5_gn_1_37081.1_1032_TPlot.pdf]

**T=Lsat\_1\_v5\_gn\_2\_100140.1\_Q=Lsa-miR166i\_S=1402**

category=2\_p=0.135172542726729

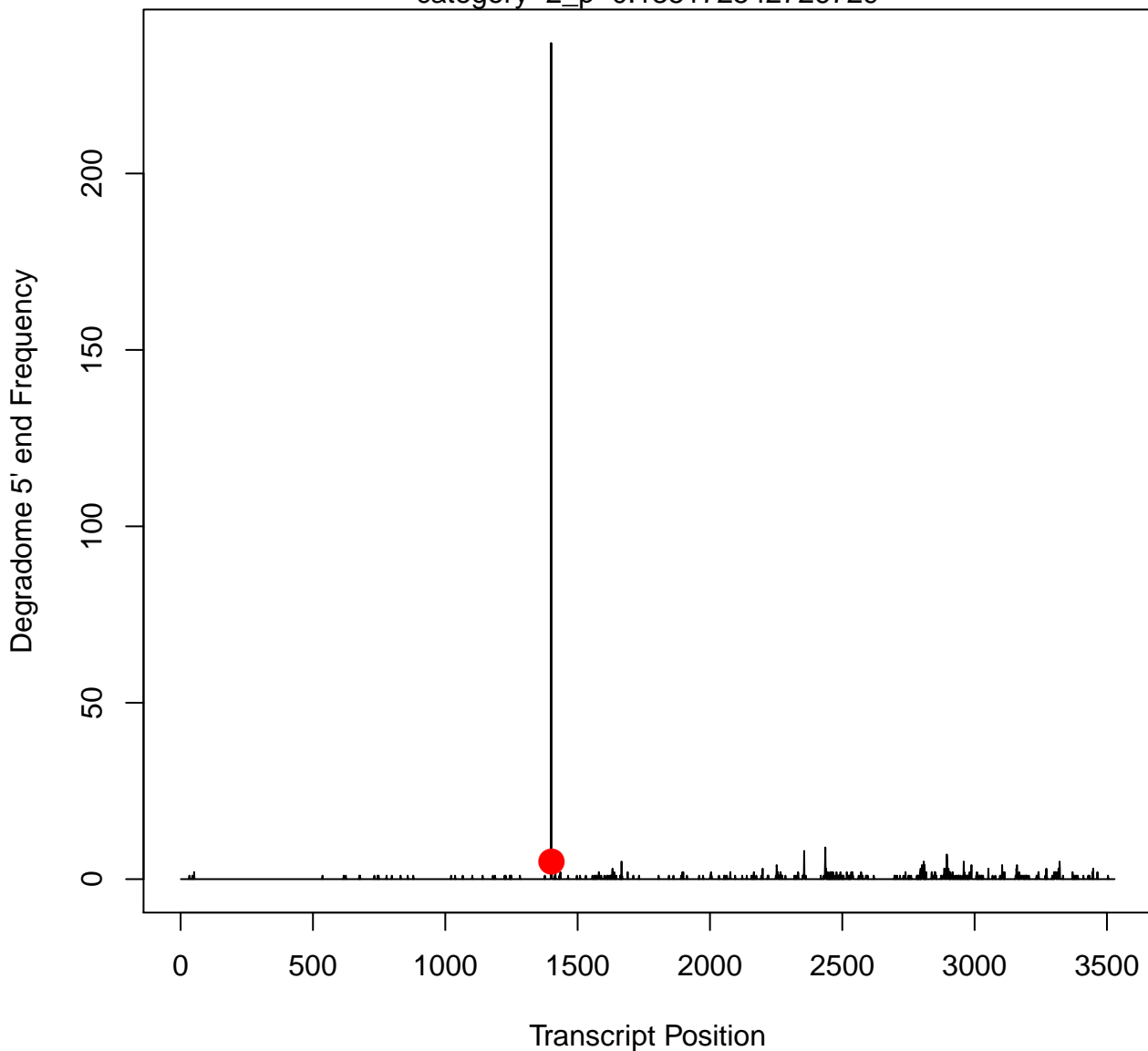

Supplement: Supplementary Data 1 — Results of categories 0–2 from PARE-Seq analysis (including three subfiles:1_1, 1_2, 1_3). [file Data_Sheet_10.ZIP › GSM2230754.plot/Lsa-miR166i_Lsat_1_v5_gn_2_100140.1_1402_TPlot.pdf]

T=Lsat\_1\_v5\_gn\_3\_6400.1\_Q=Lsa-miR166i\_S=1216

category=2\_p=0.886775074737033

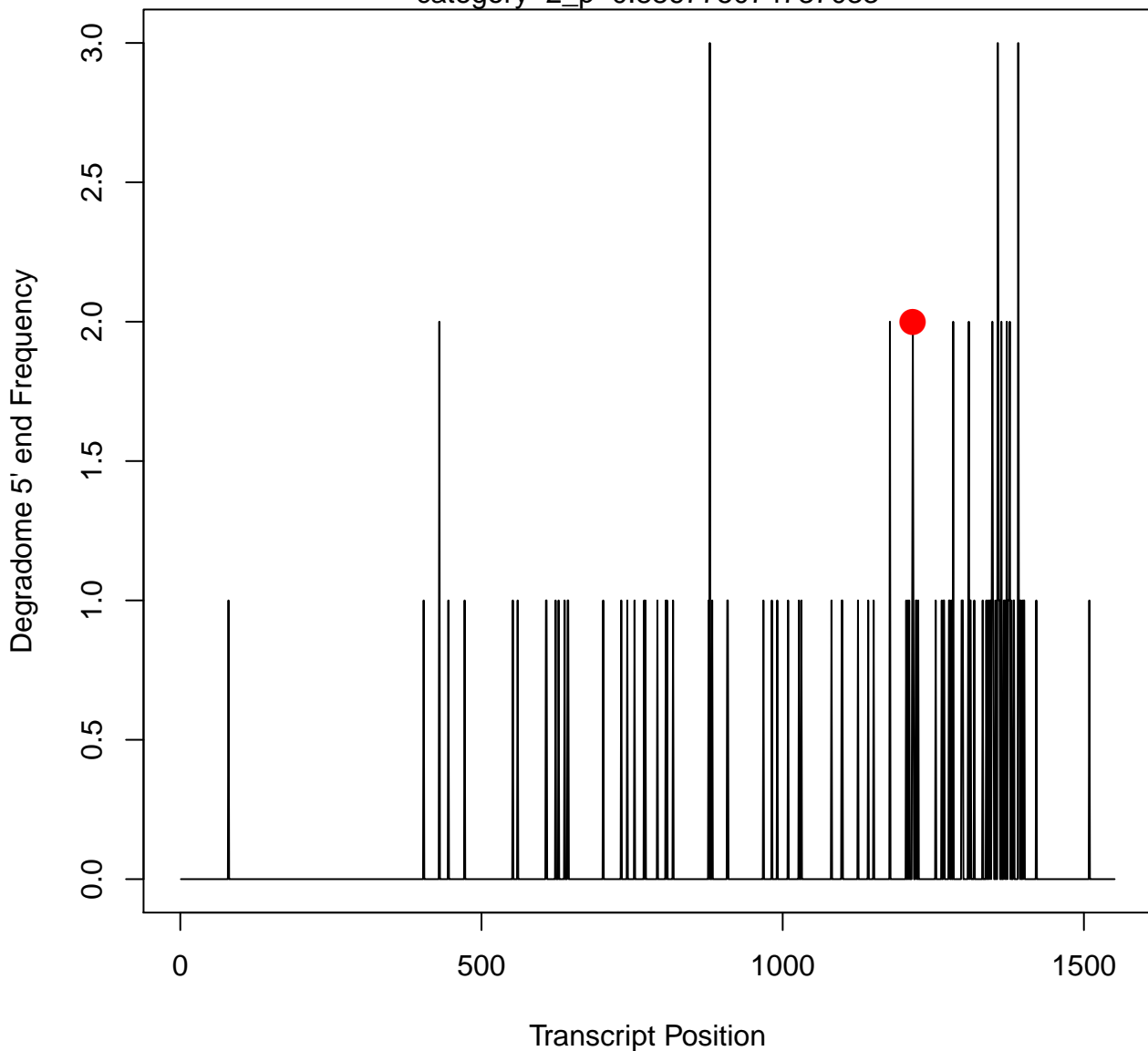

Supplement: Supplementary Data 1 — Results of categories 0–2 from PARE-Seq analysis (including three subfiles:1_1, 1_2, 1_3). [file Data_Sheet_10.ZIP › GSM2230754.plot/Lsa-miR166i_Lsat_1_v5_gn_3_6400.1_1216_TPlot.pdf]

**T=Lsat\_1\_v5\_gn\_4\_100080.1\_Q=Lsa-miR166i\_S=1069**

category=2\_p=0.0834467922232335

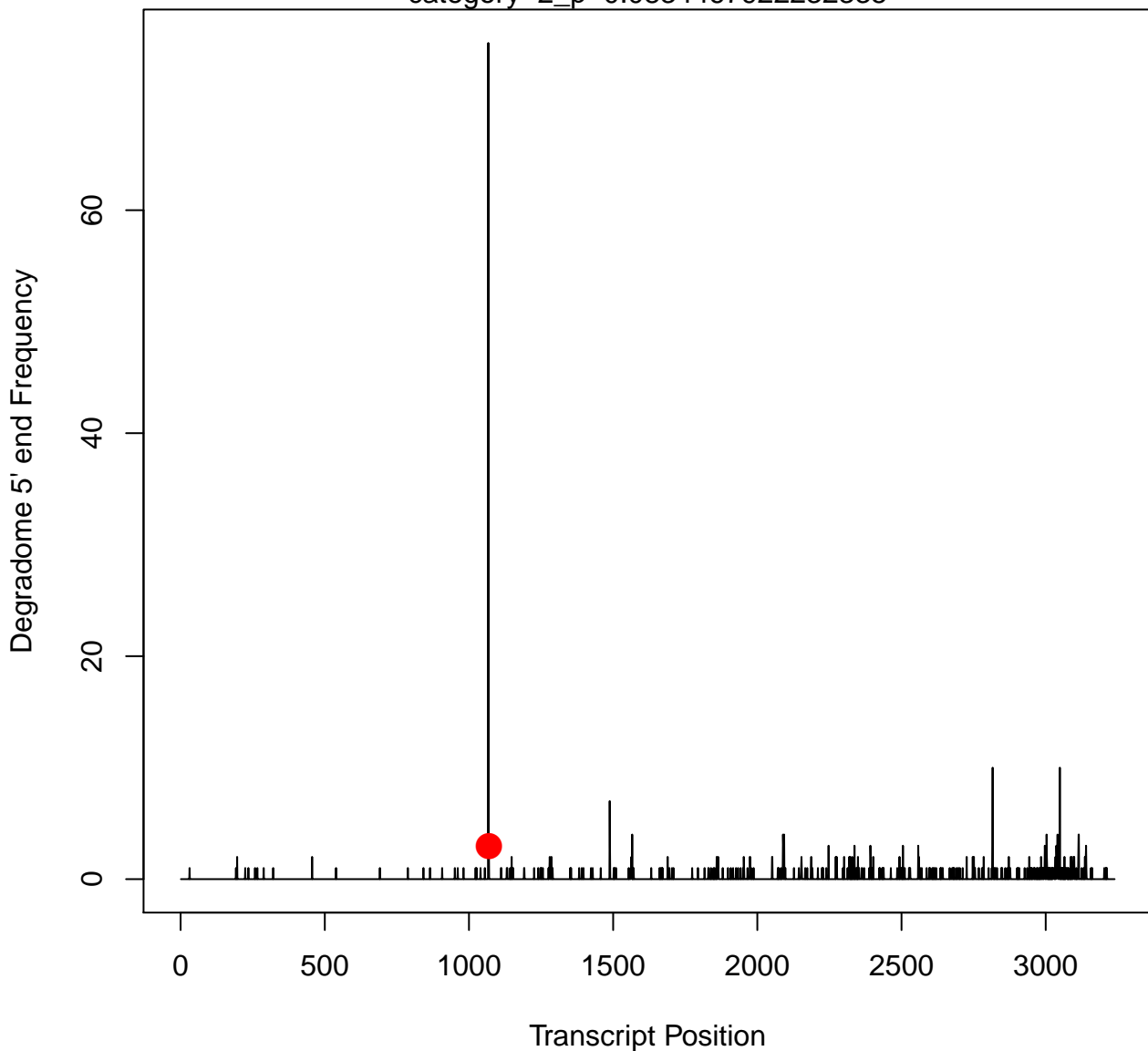

Supplement: Supplementary Data 1 — Results of categories 0–2 from PARE-Seq analysis (including three subfiles:1_1, 1_2, 1_3). [file Data_Sheet_10.ZIP › GSM2230754.plot/Lsa-miR166i_Lsat_1_v5_gn_4_100080.1_1069_TPlot.pdf]
