# Supplementary material for: Comprehensive Annotation and Functional Exploration of MicroRNAs in Lettuce
Source: Front Plant Sci. 2021 Dec 24;12:781836. doi: 10.3389/fpls.2021.781836 (PMC8739914; doi:10.3389/fpls.2021.781836)
Supplement: Supplementary file 5 [file Data_Sheet_1.PDF]

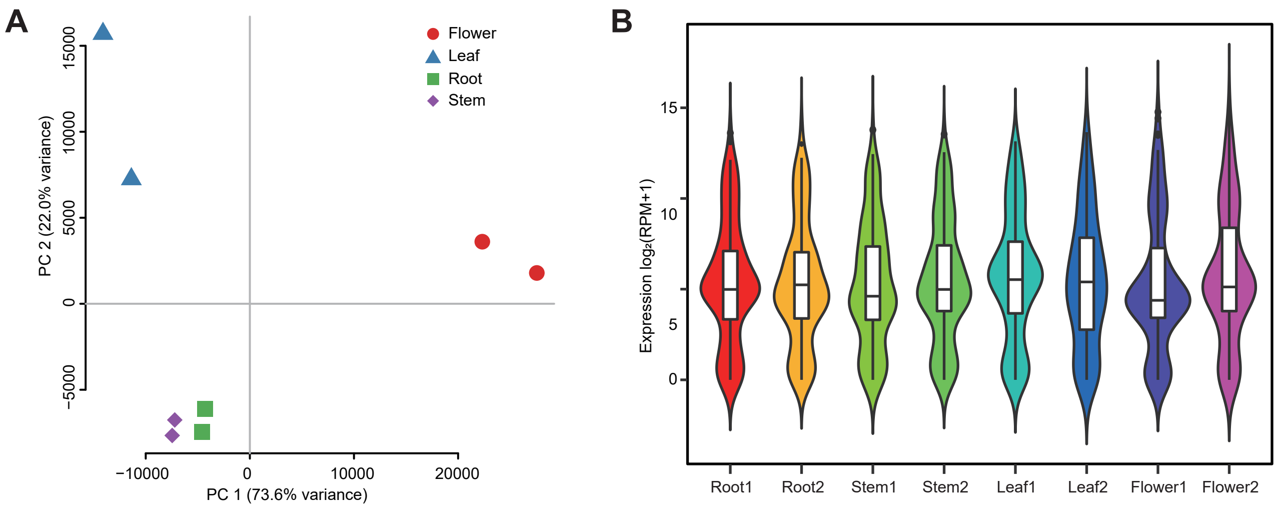

Supplementary Figure 1. Evaluation of sRNA libraries by miRNA expression.
(A) Principal component analysis (PCA) of sRNA samples based on miRNA expression values. **(B)** Expression value distribution of all miRNAs. For each sample, the overall distribution is displayed in violin plot while the median and quartiles are indicated in the inside box plot.
