# Supplementary material for: Comprehensive Annotation and Functional Exploration of MicroRNAs in Lettuce
Source: Front Plant Sci. 2021 Dec 24;12:781836. doi: 10.3389/fpls.2021.781836 (PMC8739914; doi:10.3389/fpls.2021.781836)
Supplement: Supplementary file 6 [file Data_Sheet_2.PDF]

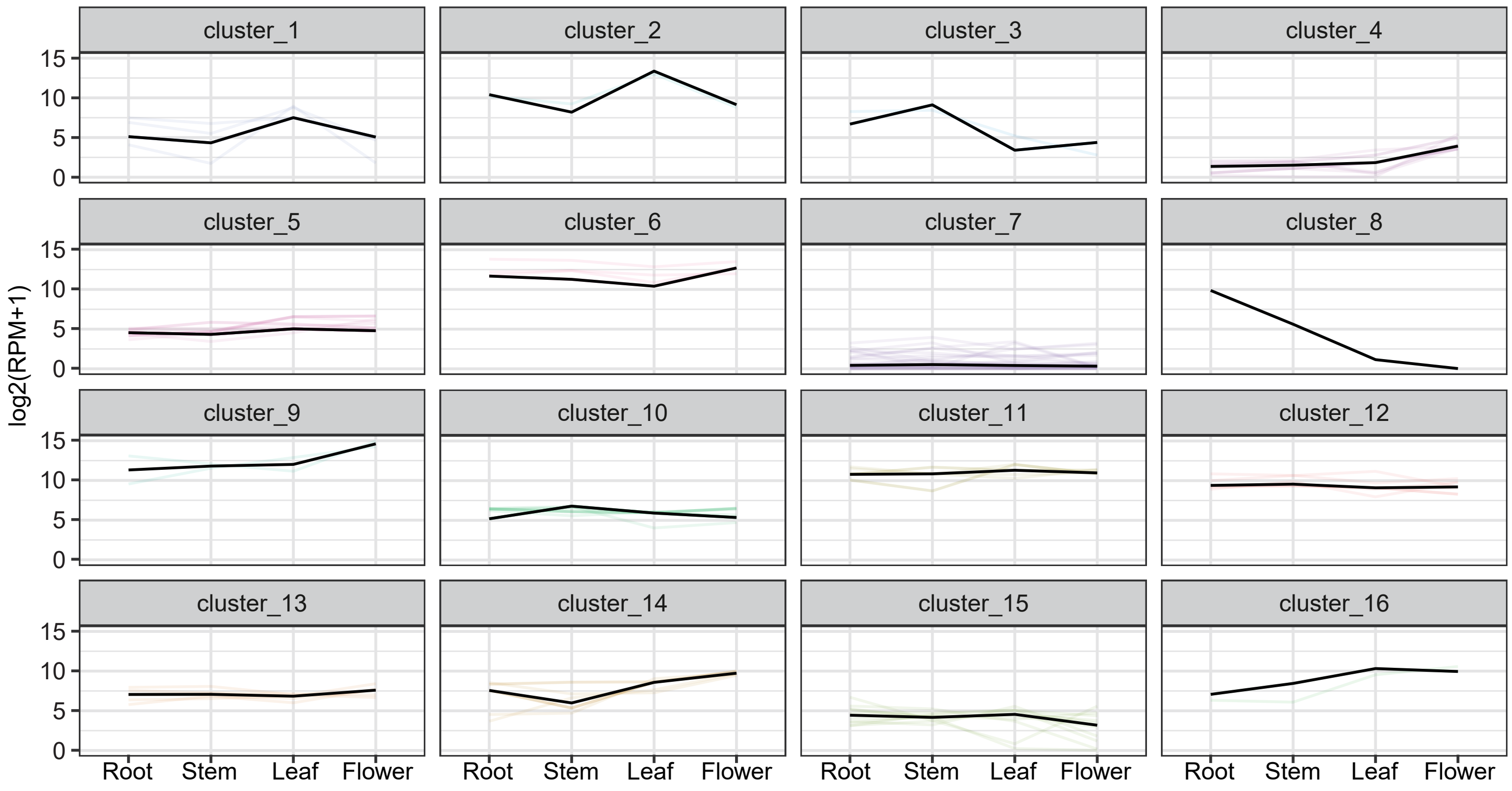

Supplementary Figure 2. K-means cluster analysis of miRNAs in lettuce.
The expression patterns of 157 miRNAs could be grouped into 16 categories.
