# Supplementary material for: Comprehensive Annotation and Functional Exploration of MicroRNAs in Lettuce
Source: Front Plant Sci. 2021 Dec 24;12:781836. doi: 10.3389/fpls.2021.781836 (PMC8739914; doi:10.3389/fpls.2021.781836)
Supplement: Supplementary file 7 [file Data_Sheet_3.PDF]

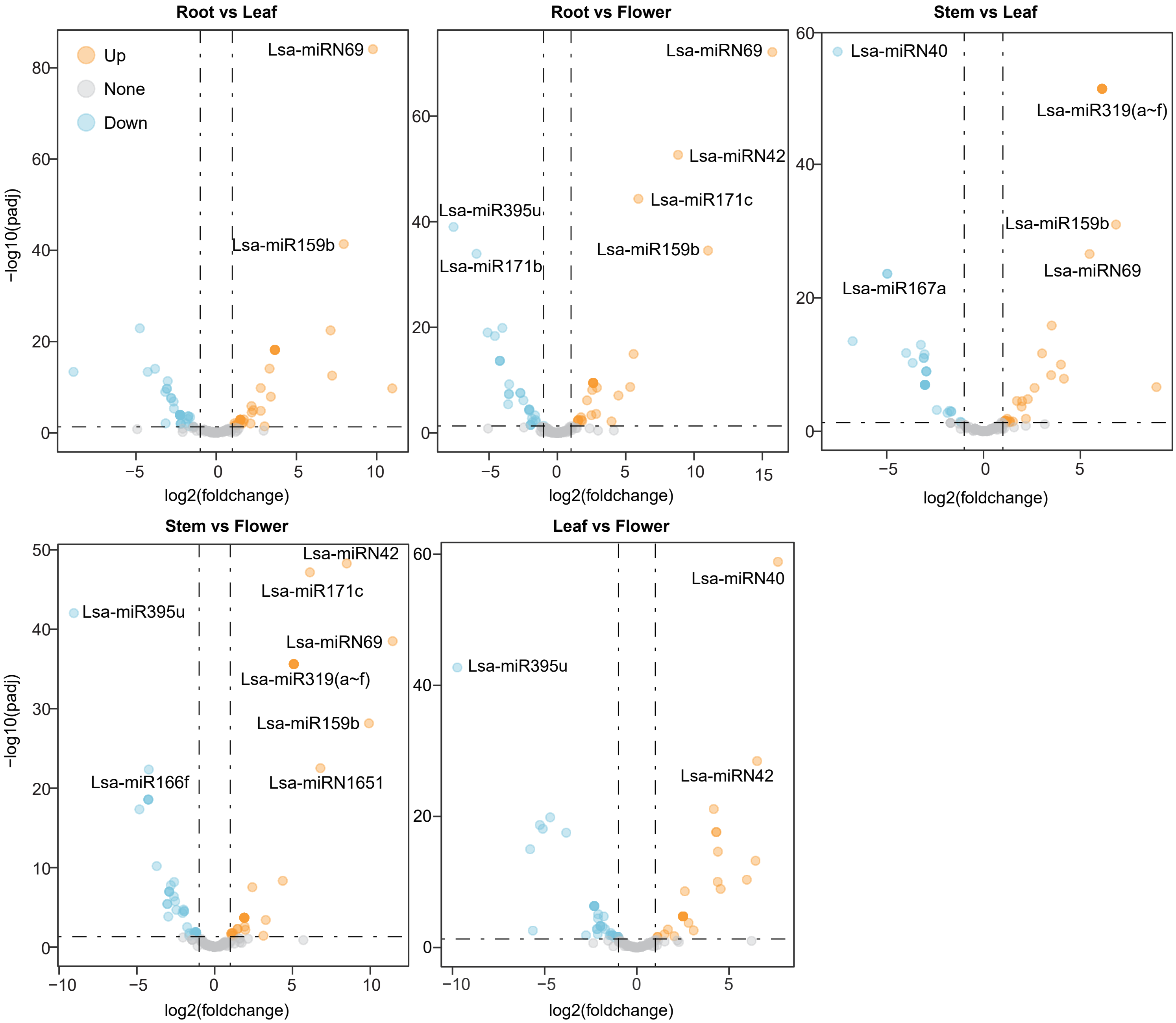

Supplementary Figure 3. Differentially expressed analysis of five paired-tissue comparisons.
The comparison of z-scored expression pattern between Ath-miR408 and Lsa-miR408 in root, stem, leaf and flower organs.
