# Supplementary material for: Comprehensive Annotation and Functional Exploration of MicroRNAs in Lettuce
Source: Front Plant Sci. 2021 Dec 24;12:781836. doi: 10.3389/fpls.2021.781836 (PMC8739914; doi:10.3389/fpls.2021.781836)
Supplement: Supplementary file 8 [file Data_Sheet_4.PDF]

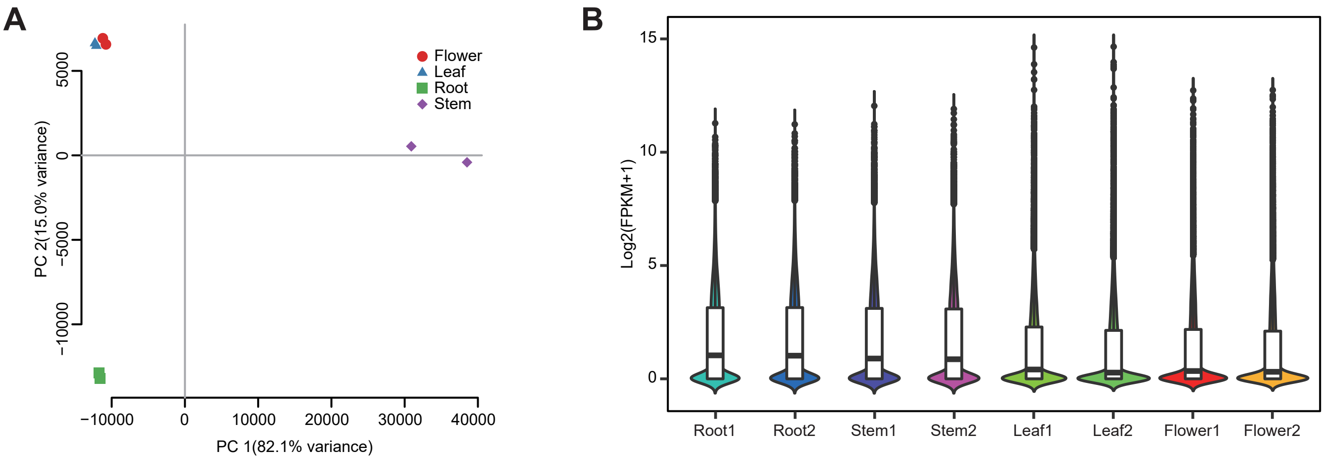

Supplementary Figure 4. Evaluation of mRNA libraries via expression.

(A) Principal component analysis (PCA) of RNA samples via mRNA transcript expression values. **(B)** Expression distribution of mRNA transcripts in each sample. For each sample, the overall distribution is displayed in violin plot while the median and quartiles are indicated in the inside box plot.
