# Supplementary material for: Comprehensive Annotation and Functional Exploration of MicroRNAs in Lettuce
Source: Front Plant Sci. 2021 Dec 24;12:781836. doi: 10.3389/fpls.2021.781836 (PMC8739914; doi:10.3389/fpls.2021.781836)
Supplement: Supplementary file 9 [file Data_Sheet_5.PDF]

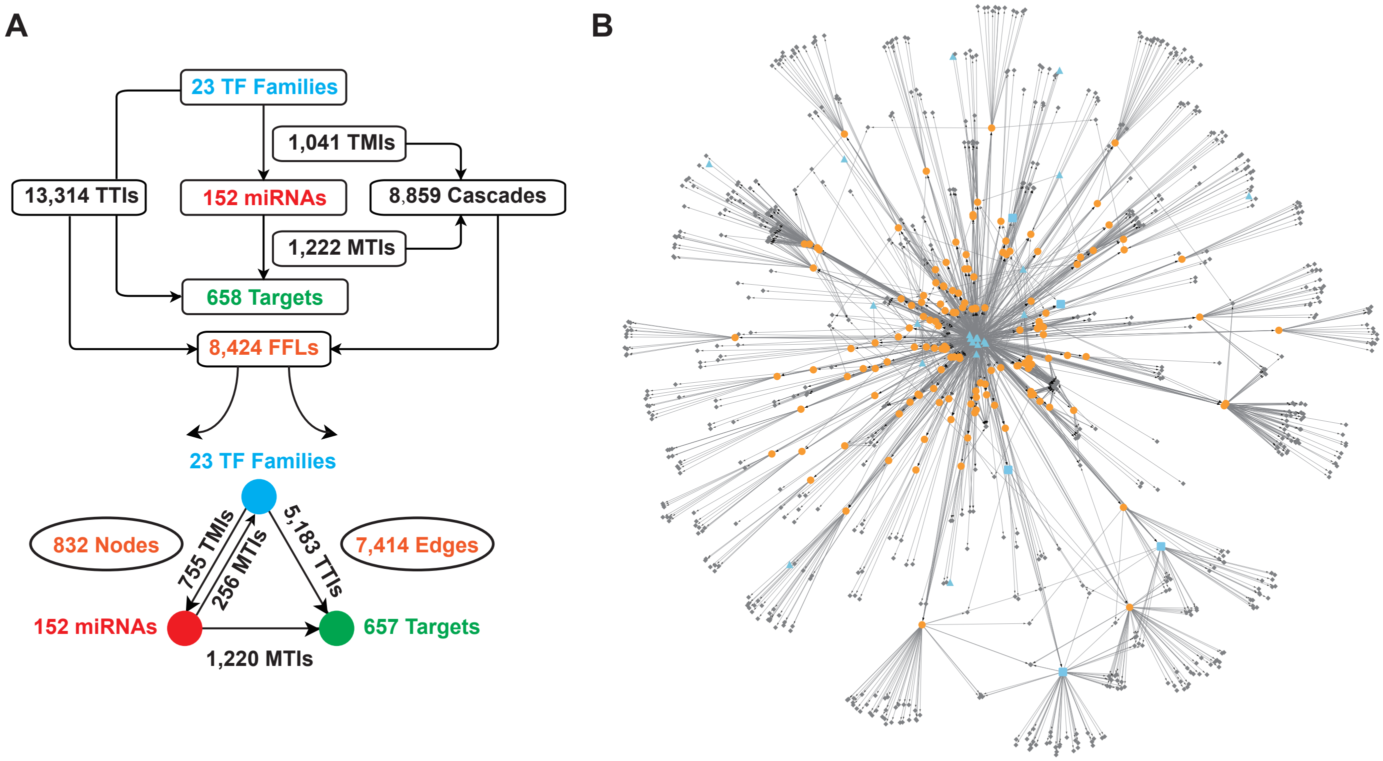

Supplementary Figure 5. FFLs (Feed-forward loops) network based on 'intersection' datasets.
(A) The analysis process and detailed information of the FFLs network. **(B)** The FFLs regulatory network where the blue, orange and gray nodes were transcription factors, miRNAs and targets, respectively.
