# Supplementary material for: Comprehensive Annotation and Functional Exploration of MicroRNAs in Lettuce
Source: Front Plant Sci. 2021 Dec 24;12:781836. doi: 10.3389/fpls.2021.781836 (PMC8739914; doi:10.3389/fpls.2021.781836)
Supplement: Supplementary file 10 [file Data_Sheet_6.PDF]

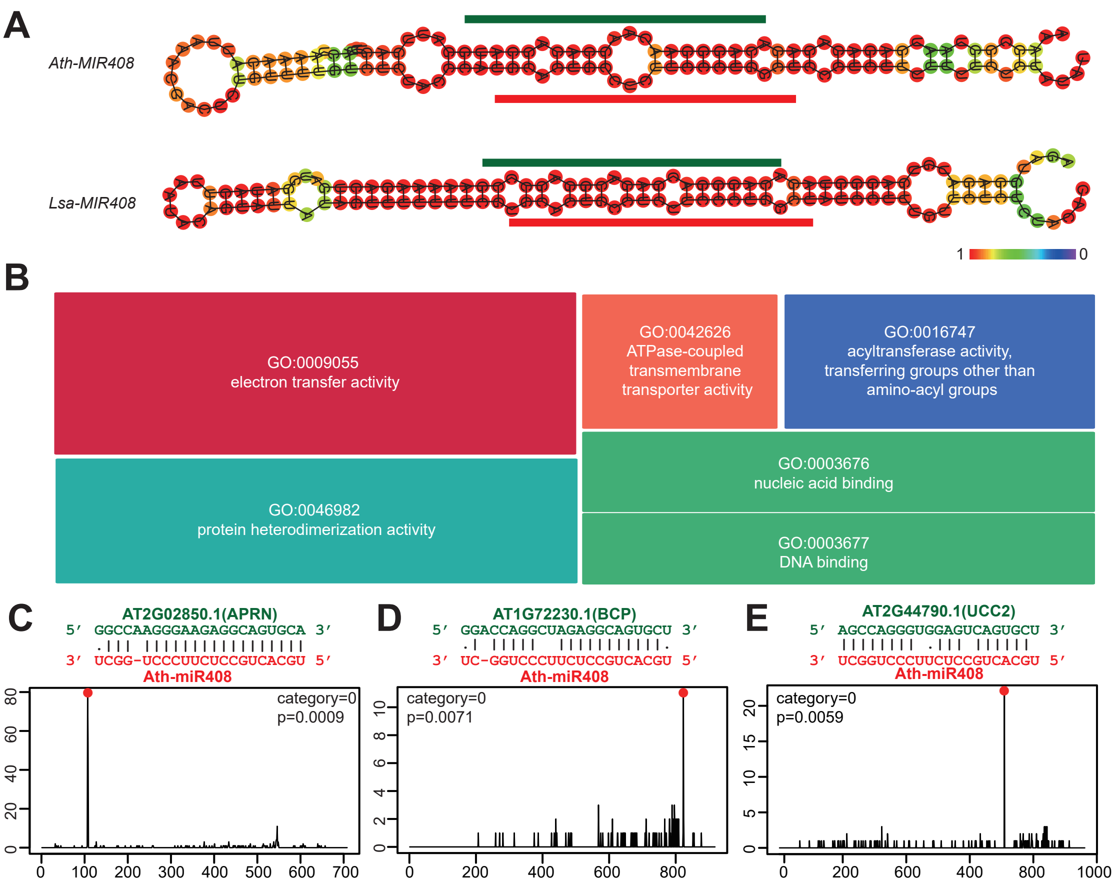

Supplementary Figure 6. MF (Molecular Function) enrichment for the targets of *Lsa-MIR408* and Comparison of *MIR408* in Arabidopsis and lettuce.
(A) Secondary structures of *Ath-MIR408* and *Lsa-MIR408*. Red and green lines indicate the positions of mature and star miRNAs, respectively. The colors covering letters show structure stability in terms of free energy. The secondary structure were predicted by RNAfold. **(B)** Tree map of Molecular Function enrichment result for the targets of *Lsa-MIR408*. **(CDE)** Top three targets of Ath-miR408. The data were retrieved from PmiREN database.
